# ﻿Catalog of the Hydroptilidae (Insecta, Trichoptera)

**DOI:** 10.3897/zookeys.1140.85712

**Published:** 2023-01-16

**Authors:** Robin E. Thomson

**Affiliations:** 1 Department of Entomology, University of Minnesota, 219 Hodson Hall, 1980 Folwell Avenue, St. Paul, Minnesota, 55108, USA University of Minnesota St. Paul United States of America

**Keywords:** Caddisflies, Trichoptera, microcaddisflies, Hydroptilidae, catalog, taxonomy, distribution, valid names, synonyms, bibliography

## Abstract

The microcaddisfly (Trichoptera: Hydroptilidae) fauna is catalogued from a review of more than 1,300 literature citations through the end of 2020 to include 2,665 currently recognized, valid species in six subfamilies and 76 genera. Fourteen subspecies are included in the total as well as 23 fossil species and three fossil genera. The family Ptilocolepidae (Trichoptera), also covered in this catalogue, comprises 19 valid species in two genera; two subspecies and two fossil species are included in the total. The monotypic genus *Eutonella*, currently considered incertae sedis within Trichoptera, was formerly placed in Hydroptilidae and is also included in this catalogue. Genus-group and species-group synonyms are listed. Information on the type locality, type depository, sex of type, distribution by country, and other relevant taxonomic or biological information is included for each nominal species. Summary information on taxonomy, phylogeny, distribution, immature stages, and biology are provided for each subfamily, tribe, and genus where known. An index to all nominal taxa is provided to facilitate catalog use.

## ﻿Introduction

Hydroptilidae is the largest of the approximately 50 families in the order Trichoptera containing more than 2,600 species found in all faunal regions of the world and distributed in six subfamilies and 76 genera (including three fossil genera) (Table 1). However, the family is also the smallest family in the order in terms of body size, with adults ranging from between 1.5 mm to usually no more than 5 mm in length (Holzenthal et al. 2007b).

**Table 1.** Number of extant and fossil species of Hydroptilidae and Ptilocolepidae, by genus.

Family	Subfamily	Tribe	Genus	Subgenus	No. Species	
					Extant	Fossil
**Hydroptilidae**						
	Hydroptilinae					
			*Acanthotrichia*		1	-
			*Acritoptila*		16	-
			*Aenigmatrichia*		1	-
			*Agraylea*			
				*Agraylea*	8	3
				*Nanoagraylea*	-	3
			*Allotrichia*		10	4
			*Austratrichia*		1	-
			*Cyclopsiella*		1	-
			*Dhatrichia*		14	-
			*Hellyethira*		44	-
			*Hydroptila*		495	1
			*Jabitrichia*		4	-
			*Kholaptila*		1	-
			*Maeyaptila*		1	-
			*Microptila*		20	-
			*Missitrichia*		3	-
			*Mulgravia*		2	-
			*Oxyethira*			
				*Argyrobothrus*	6	-
				*Dactylotrichia*	16	-
				*Dampfitrichia*	31	-
				*Holarctotrichia*	11	-
				*Loxotrichia*	16	-
				*Mesotrichia*	5	-
				*Oxyethira*	52	-
				*Oxytrichia*	19	-
				*Pacificotrichia*	15	-
				*Tanytrichia*	20	-
				*Trichoglene*	25	-
				unplaced	36	1
			*Paroxyethira*		25	-
			*Paucicalcaria*		1	-
			*Sutheptila*		1	-
			*Tangatrichia*		1	-
			*Tricholeiochiton*		10	-
			*Ugandatrichia*		31	-
			*Vietrichia*		1	-
			*Wlitrichia*		1	-
			*Xuthotrichia*		2	-
	Leucotrichiinae					
		Alisotrichiini				
			*Alisotrichia*		61	1
			*Byrsopteryx*		16	-
			*Celaenotrichia*		1	-
			*Cerasmatrichia*		10	-
			*Mejicanotrichia*		7	-
			*Scelobotrichia*		3	-
		Leucotrichiini				
			*Acostatrichia*		15	-
			*Anchitrichia*		8	-
			*Ascotrichia*		6	-
			*Betrichia*		10	-
			*Ceratotrichia*		5	-
			*Costatrichia*		20	-
			*Leucotrichia*		45	1
			*Peltopsyche*		6	-
			*Tupiniquintrichia*		2	-
			*Zumatrichia*		53	-
	Neotrichiinae					
			*Kumanskiella*		2	-
			*Mayatrichia*		7	-
			*Neotrichia*		205	-
			*Taraxitrichia*		1	-
	Ochrotrichiinae					
			*Angrisanoia*		5	-
			*Caledonotrichia*		11	-
			*Dibusa*		1	-
			*Maydenoptila*		8	-
			*Metrichia*		141	-
			*Nothotrichia*		6	-
			*Ochrotrichia*		221	5
			*Ragatrichia*		5	-
			*Rhyacopsyche*		30	-
	Orthotrichiinae					
			*Ithytrichia*		7	-
			*Orthotrichia*		271	1
			*Saranganotrichia*		4	-
	Stactobiinae					
			*Bredinia*		17	-
			*Catoxyethira*		68	-
			*Chrysotrichia*		70	-
			*Flintiella*		17	-
			*Maetalaiptila*		1	-
			*Niuginitrichia*		24	-
			*Pseudoxyethira*		64	-
			*Orinocotrichia*		3	-
			*Plethus*		27	-
			*Stactobia*		164	-
			*Stactobiella*		22	-
			*Tizatetrichia*		2	-
	*incertae sedis*					
			♰ *Burminoptila*		-	1
			*Dicaminus*		1	-
			♰ *Electrotrichia*		-	1
			*Macrostactobia*		2	-
			♰ *Novajerseya*		-	1
			*Orphninotrichia*		20	-
			**TOTAL HYDROPTILIDAE**		**2642**	**23**
**Ptilocolepidae**						
			*Palaeagapetus*		9	2
			*Ptilocolepus*		8	-
			**TOTAL PTILOCOLEPIDAE**		**17**	**2**
*incertae sedis*						
			*Eutonella*		1	-

Marshall (1979b) proposed that the family Hydroptilidae contains two subfamilies: Hydroptilinae and Ptilocolepinae, with several tribes included within Hydroptilinae. The subfamily Ptilocolepinae was later elevated to family status (Malicky 2001b, 2008b), restricting Hydroptilidae to a single subfamily, Hydroptilinae, as defined by Marshall (1979b). As a result, all of the tribes originally listed by Marshall were raised to subfamily status and remain so today. Thus, Hydroptilidae now consists of six subfamilies and six unplaced (incertae sedis) genera and Ptilocolepidae consists of two genera. One ‘hydroptilid’ genus, *Eutonella*, is unplaced within the order Trichoptera.

Of the hydroptilid subfamilies, three are largely endemic to the Neotropical faunal region (Leucotrichiinae, Neotrichiinae, and Ochrotrichiinae), though distributions of some of the included species extend well into North America. Hydroptilinae occurs in the Old World, but does include two large cosmopolitan genera (*Hydroptila* and *Oxyethira*) and several genera that are endemic to the Australasian or Afrotropical faunal regions. The subfamily Orthotrichiinae is small, but includes the cosmopolitan genus *Orthotrichia*, and the subfamily Stactobiinae comprises a varied collection of genera that are either endemic to a particular region or occur in a wider distribution throughout multiple regions. Ptilocolepidae is distributed throughout the Holarctic faunal region. No larval stages are described for any species in many genera.

### ﻿General morphology and biology

Hydroptilids, like all Trichoptera, are holometabolous with a terrestrial adult stage and aquatic larval and pupal stages. Members of the family are typically minute, with few exceeding 5.0 mm in body length, which has led to their common name, of microcaddisflies. The adults are attracted to ultraviolet lights and may congregate in huge numbers at collecting sites, giving them the potential to be one of the most commonly collected of all Trichoptera. An in-depth account of hydroptilid morphology will not be given here; however, features that have traditionally been considered of importance for hydroptilid taxonomy are briefly described.

#### Larva

Hydroptilids display various structural adaptations to the wide range of aquatic environments they occur in, making them one of the most diverse caddisfly families regarding the form of larvae and larval cases (Marshall 1979b). In Nielsen’s (1948) classic work on hydroptilid larvae, he gave a very detailed account of larval morphology and biology; unfortunately, it is a fairly limited view of the family as a whole, since he expounded on only five of the more specialized genera within Hydroptilidae (*Agraylea*, *Hydroptila*, *Ithytrichia*, *Orthotrichia*, and *Oxyethira*). However, Nielsen’s work laid an excellent foundation for future trichopterists and made Marshall’s (1979b) more general account of larval morphology possible.

One of the characteristic features of hydroptilid larvae is the simple form of hypermetamorphosis they undergo. Marshall (1979b) commented that it had not been confirmed if ptilocolepid larvae also undergo hypermetamorphosis, but it has since been confirmed that ptilocolepids do experience the same changes, although not as pronounced as those of the hydroptilids (Wells 2010b). Early instars I–IV of both families are relatively smaller (0.5–2.7 mm in length), of short duration, and caseless, i.e., “free-living”, while final instar V is larger (2.0–7.0 mm in length) and constructs a portable or secondarily fixed case (Marshall 1979b). The final instar also functions as the primary feeding and growing stage in the hydroptilid life cycle, during which the abdomen becomes greatly enlarged (Marshall 1979b) or physogastric. The early instars I–IV can be characterized by features associated by their absence of a case, including narrowly tapering abdomens, freely projecting anal prolegs; the long, fine setae on the body can offer resistance to sinking and help the larva to swim and disperse (Marshall 1979b). The duration of these free-living stages is usually much shorter than the final instar. In only a very few species is the final instar also free-living.

The case-building larvae of instar V are prognathous and campodeiform, similar to the “saddle-case” bearing glossosomatids, as opposed to the hypognathous and eruciform “tube-case” building families (Marshall 1979b). Instar V tends to be more easily identified and distinct among the genera and can be separated from all other Trichoptera larvae by a combination of features, including the enlarged abdomen, three pairs of well-developed thoracic tergites, the absence of segmentally arranged tracheal gills, fusion of the well-developed abdominal prolegs to the sides of abdominal segment X, and, while the number of abdominal tergites may vary, one is always present on abdominal segment IX (Marshall 1979b; Wiggins 1996). Hydroptilid larvae have dorsal sclerotized rings on abdominal segments II/III to VII/VIII which have not been recorded in ptilocolepid larvae; these rings may be regions of specialized chloride epithelial cells adapted for ion absorption and osmoregulation (Wichard 1976; Wiggins 1977). Features of the thoracic legs of instar V have proven useful at the generic level in hydroptilid taxonomy; for example, though basically ambulatory, they may be modified to be robust for clinging to substrate in swiftly moving waters, long and slender in vegetation dwellers, or may bear a specialized process that aids in the manipulation of algal filaments (Marshall 1979b). In instar V, the abdomen becomes hugely distended; the overall form and shape of the abdominal expansion is unique and typically characteristic for each genus (Marshall 1979b). Unique among trichopteran, members of the genus *Orthotrichia* have a pronounced triangular ‘tooth’ on the larval labrum.

The larval case constructed during the final instar is often referred to as a “purse-case”, a term coined by Ross (1967) to separate hydroptilid cases from glossosomatid “saddle-cases” and phryganidean (sensu Thomas et al. 2020) “tube-cases”. The typical “purse-case” consists of two closely apposed silken halves into which various organic or inorganic particles may be incorporated; the two halves are joined along the lateral margins, leaving narrow openings at the anterior and posterior ends, and may be either laterally or dorso-ventrally compressed (Stephens 1836; Marshall 1979b). As the larval abdomen increases in size during the final instar, the case is expanded by splitting the margins, adding new layers of silk, and then resealing them (Nielsen 1948; Wiggins 1996).

#### Pupa

As in most Trichoptera, hydroptilid pupae are exarate, dectitious, and do not offer many features useful for identification past the family level (Marshall 1979b). Aside from the absence of any structural features used to positively characterize other caddis families, hydroptilid pupae might be recognized by the absence of abdominal gills or lateral lines, the presegmental dorsal abdominal plates on segments III–VII, the postsegmental plates on segments III–V, and their relatively small size (1.5–6.0 mm in length) (Marshall 1979b). Overall, the pupal case is similar to the larval case, but firmly attached to the substrate with the anterior and posterior openings sealed; once the case is attached and sealed, the larva spins one final internal lining and adopts a characteristic resting posture in which the thorax becomes distended, the abdomen straightens, and the intersegmental grooves become less obvious (Barnard 1971). Ptilocolepid and hydroptilid pupae can be separated based on the presence or absence of medial teeth on the mandibles: in Hydroptilidae medial teeth are absent, while in Ptilocolepidae either one (*Palaeagapetus*) or two (*Ptilocolepus*) teeth are present (Marshall 1979b).

#### Adult

Features that comprise the generally accepted typical hydroptilid form include small size (1.2–6.0 mm forewing length); narrow, pointed wings with long setal fringes along the anal margin and reduced venation; a dense layer of setae on the wings and body parts creates a general appearance of pubescence (Stephens 1836; Marshall 1979b). While most genera bear setae that are white and either black or brown in hue, giving them a mottled appearance, some tropical genera are known to exhibit patches of distinct metallic hues. As the overall size of genera decreases, the wings also become reduced, leading to decreasingly distinct venation and increasingly longer setal fringes that compensate for the loss of wing membrane area (extreme examples or reduced wings and venation can be seen in the genera *Chrysotrichia* and *Neotrichia*) (Stephens 1836; Marshall 1979b). Because of this reduction in venation, venational features that are of taxonomic importance in other families of larger caddisflies are not constant in hydroptilid genera and are considered unreliable taxonomic characters.

On the head capsule, dorsal ocelli vary from none to two or three and, posteriorly, there is typically a pair of dorsal postoccipital lobes or warts which may be modified as eversible scent-dispersing organs (e.g., *Hydroptila*). In some genera (e.g., *Leucotrichia*), the head may bear modifications, such as patches of scales or setiferous protuberances. Antennal segments, typically the basal-most, in some genera may also be modified to appear elongated or enlarged. On the thorax, the angular warts and near-vertical posterior face of the mesoscutellum are distinct characters for the family. Additional taxonomically important features on the thorax at the subfamily and generic levels include the shape of the meso- and metathoracic nota and the presence or absence of transverse sutures. The posterior mesothoracic katepisternal suture is typically present in ptilocolepids and absent in hydroptilids, a feature first noted by Ross (1956) and later confirmed by Marshall (1979b). Wings of some genera may also bear modifications (e.g., *Peltopsyche*, *Costatrichia*), such as patches of scales or a costal “pouch” or “bulla”. The spur formula, which refers to the number of spurs present on the tibiae, is another important diagnostic feature used in hydroptilid taxonomy. The formula indicates the number of spurs on the fore-, mid-, and hind tibiae, respectively, with four being the maximum number of spines on any one leg (two preapical and two apical) (Marshall 1979b); for example, the formula for the genus *Leucotrichia* is 1, 3, 4.

The hydroptilid abdomen consists of the typical eleven basic segments, with segments X and XI generally being regarded as one, and the sclerites of the posterior segments modified to form the genitalic structures, which provide features of taxonomic importance at both the generic and specific levels; most hydroptilid species are known from male specimens only. Segment IX forms a distinct genital capsule with a membranous posterior concavity, out of which originates segment X, the phallus, and any ventral appendages (Marshall 1979b). Segment X is present as a tergite only, is usually completely membranous but may be weakly sclerotized, varies considerably in size and shape, and may fuse ventrally with structures beneath the phallus to form a structure known as the “phallic tube” or “phallocrypt” (Marshall 1979b). Inferior appendages, often referred to as “claspers”, are single-segmented, in contrast to the 2-segmented condition generally found in many other caddis families, and vary greatly in size, shape, and whether or not they bear additional processes or setae; these appendages can provide taxonomically important features at both the generic and specific levels (Marshall 1979b). The phallus (also sometimes referred to as the aedeagus, penis, or copulatory organ) is essentially a long, slender, sclerotized tube that varies structurally between groups. In the Hydroptilinae, Neotrichiinae, and Orthotrichiinae, the phallus bears a spiral “titillator” and is divided into a proximal half bearing an ejaculatory duct and a distal half bearing an intromittent organ. In Leucotrichiinae, the phallus bears a complicated median complex with “windows”, “loops”, and a membranous apex. In Ochrotrichiinae, the phallus may be very slender or heavily spined, whereas in Stactobiinae, the phallus is essentially a common median duct bearing a pair of lateral processes that may be fused (Marshall 1979b). Overall, not much is really known about the comparative morphology of hydroptilid male genitalia, and the terminology of different structures varies greatly between authors. For example, Marshall (1979b) uses the term “subgenital” to refer to any structures occurring ventral to the phallus; when separate and paired, she calls them “appendages” (which have been referred to variously as “intermediate appendages”, “lateral penis-sheaths”, or “parameres”) and when they are fused refers to the structure as a “plate” (“lower penis cover” or “ventral plate of X”). Oláh and Johanson (2008) argued for the use of appendicular terminology (“gonopods”, “paraprocts”, “cercus”, etc.) over the directional terms (appendages referred to as “inferior”, “intermediate”, “superior”, etc.). Their work, however, addressed Trichoptera as a whole and did not address the complex genitalia of Hydroptilidae in specific, as Marshall’s (1979b) monograph did. Further work regarding hydroptilid male genitalia is needed to infer the homology of these structures.

Female genitalia of hydroptilids are of the generalized trichopteran condition, a simple “telescopic ovipositor” or “oviscapt” which consists of modified abdominal segments VIII–X and a gonopore occurring ventrally between segments IX and X (Scudder 1971; Marshall 1979b). The posterior margin of the ringlike segment VIII provides features that can be of taxonomic importance, such as dorsal and ventral processes or rows or setae (Marshall 1979b).

#### Ptilocolepidae

While some of the above description can be extended to the ptilocolepids, there are some fundamental differences that can be used to differentiate between the two families. Although ptilocolepid larvae closely resemble those of hydroptilids, the adults look more similar to small rhyacophilids or glossosomatids. They are relatively larger than hydroptilids (4.0–6.0 mm in length) and their wings are relatively broad with rounded apices and short marginal setal fringes (Marshall 1979b). The wings also boast a much more complete venation that resembles that of primitive rhyacophilids, differing in the subcosta of the forewing and the fusion of various veins in the hindwing (Ross 1956). Ptilocolepid venation differs from hydroptilids by the presence of a distinct discoidal cell, separate M_3_ and M_4_, and a forked Cu_1_ in the forewing (Marshall 1979b). Additionally, ptilocolepids bear short, unmodified macrotrichia in a sparse, scattered distribution on their body, which gives them more of an overall granulose appearance, rather than pubescent.

### ﻿Taxonomic history

#### Hydroptilidae

The family Hydroptilidae was established in 1836 by Stephens for the genera *Hydroptila*, *Agraylea*, and *Narycia*. However, the only species of the genus *Narycia* that was figured, *Naryciaelegans*, subsequently proved to be a moth from the family Psychidae. The remaining genera, *Hydroptila* and *Agraylea*, could be distinguished from other trichopteran families by the “cleft-like” openings of the larval cases and by the filiform antennae and unfolded posterior wings of the adults (Marshall 1979b). The cases and larvae of *Hydroptilapulchricornis* and *Oxyethiraflavicornis* had actually been described previously by Pictet (1834) as a unique taxon under the general name “les Hydroptiles”. For this reason, McLachlan (1880) considered Pictet (1834), and not Stephens, to be the true founder of the family Hydroptilidae.

In 1948, Nielsen made the first attempt to divide Hydroptilidae, which was proving to be a large and heterogeneous group, into subfamilies. Based on morphological similarities of the larvae, he proposed the subfamilies Orthotrichiinae for the genera *Ithytrichia* and *Orthotrichia* and Hydroptilinae for the genera *Agraylea*, *Hydroptila*, and *Oxyethira*. While other genera, such as *Ptilocolepus* and *Stactobia*, had been established by this time, Nielsen commented on their relative position within the family but declined to formally place them in either of his proposed subfamilies. At this time, Nielsen also placed Hydroptilidae between the “saddle-case” building Glossosomatinae and the “tube-case” building Integripalpia, based on features of the larvae and pupae. In this work, Nielsen described in great detail the morphology, life histories, and feeding and case building behaviors of five microcaddisfly genera, which provided a very accurate, but fairly restricted, overview of microcaddisflies in general.

Botosaneanu (1956) established the subfamily Stactobiinae for the previously unplaced genus *Stactobia* and what he referred to as “its immediate relatives”, which most likely included the genera *Plethotrichia*, *Plethus*, *Lamonganotrichia*, *Stactobiella*, and probably *Catoxyethira* (Marshall 1979b).

In Ross’s (1956) new classification, the “purse-case” making hydroptilids, included in the “case-maker division”, were divided into the subfamilies Ptilocolepinae for the genera *Palaeagapetus* and *Ptilocolepus*, and Hydroptilinae for the remaining genera; Hydroptilinae was further divided into the tribes Hydroptilini and Neotrichiini. Flint (1970) later declined to follow Ross’s (1956) classification and proposed another new classification with the subfamily Leucotrichinae (subsequently corrected to Leucotrichiinae) for the genus *Leucotrichia* and its related genera and retained Hydroptilinae, Orthotrichiinae, Ptilocolepinae, and Stactobiinae as separate and distinct subfamilies.

Marshall (1979b) provided the first review of Hydroptilidae at the generic level, including all 42 genera described at the time. For each genus, she included information regarding nomenclature, distribution, morphology of adult and immature stages, biology, and possible species groupings. Marshall also provided keys to the subfamilies, at the time considered tribes, for the adult stage and to the genera for both the adult and immature stages. She discussed the phylogeny of the family and offered a new classification. Marshall’s proposed classification was based on that of Ross (1956) by recognizing only two subfamilies, Hydroptilinae and Ptilocolepinae. However, it also reflected the classification proposed by Flint (1970) by dividing Hydroptilinae into six tribes that corresponded to the subfamilies he had proposed: Hydroptilini, Leucotrichiini, Neotrichiini, Ochrotrichiini, and Stactobiini, and the newly proposed Ochrotrichiini. The morphology-based phylogeny proposed by Marshall was not backed by any statistical analyses and therefore offered no support values for any of the proposed relationships. Marshall’s phylogeny represents the last attempt to assess the relationships of the subfamilies and genera of Hydroptilidae as a whole.

In 2010, Wells wrote a review of hydroptilid studies published from the time of Marshall’s (1979b) review up through 2009; in this work, she reviewed hydroptilid taxonomy, included new discoveries regarding aspects of the biology of some species, and suggested that future work should place emphasis on life history studies.

In 2011, Oláh and Johanson produced a paper in which they described many new species from the Neotropical faunal region and referred to the subfamilies as tribes. In this work, Oláh and Johanson provided several tables containing either features or character states of species groups, subgenera, generic clusters, or genera in tribes. However, the tables did not cover all of the taxa present in the paper, no information on generic features or character states was provided for *Hydroptila* or any of the included stactobiine genera (*Bredinia*, *Flintiella*, and *Orinocotrichia*), nor was there any discussion of the information outlined in the tables. As interpreted from the tables, several genera were transferred between subfamilies or from incertae sedis status.

The most recent update to hydroptilid classification was based on a phylogenetic assessment of Leucotrichiinae using both molecular and morphological data (Santos et al. 2016a). The nomenclatural modifications proposed in the paper included the recognition of two tribes within Leucotrichiinae, Alisotrichiini, and Leucotrichiini, one generic synonymy, and one newly established genus. Santos et al. also discussed potential convergent evolution between Leucotrichiinae and Stactobiinae in this work.

#### Ptilocolepidae

The family Ptilocolepidae, which was once considered to be a group within Hydroptilidae and is considered in recent classifications as a distinct family, includes the genera *Ptilocolepus* and *Palaeagapetus*. The type species was originally described in *Rhyacophila* and therefore placed in the family Rhyacophilidae (Pictet 1834), but was subsequently shown to be a senior synonym of *Ptilocolepusturbidus*, making the type species *Ptilocolepusgranulatus* (Hagen 1855). Thienemann (1904a) noted similarities between the larvae of *P.granulatus* and both members of Hydroptilidae and Glossosomatidae, which led Ulmer (1907) to transfer the genus to Hydroptilidae. Subsequently, Martynov (1913b) established the subfamily Ptilocolepinae for *Ptilocolepus*, but it was retained in Rhyacophilidae. Ross (1956) transferred Ptilocolepinae to Hydroptilidae and redefined it to include *Palaeagapetus* (incorrectly referring to it as Palaeagapetinae). Kristensen (1997) suggested that *Ptilocolepus* and *Palaeagapetus* together may represent the sister group of all other Trichoptera.

In 2001, Ptilocolepidae was elevated to the family status, which is currently recognized, effectively also elevating each of the six tribes within Hydroptilinae to the rank of subfamily (Malicky 2001b). Malicky claimed that previous authors had placed differing levels of importance on morphological features of either the adults or the larvae, which led to the group being placed in different families at different times. He stated that, because all these characters should be considered equally, it was appropriate to raise the group to family rank of Ptilocolepidae. Subsequently, the deeper-level relationships within Trichoptera were explored using molecular data and both the monophyly of Ptilocolepidae and its status as a family were questioned (Holzenthal et al. 2007a). According to the work of Holzenthal et al. (2007a), Ptilocolepidae consistently groups with Hydroptilidae, indicating that the elevation to family status might be an unnecessary taxonomic change. Malicky (2008b) countered by referring to the differences in geographical distribution of the two families, stating that the high level of endemism shown by Hydroptilidae and the relictual distribution of Ptilocolepidae were additional evidence that the families are distinct from one another. In their work resolving a higher-level phylogeny of Trichoptera based on multiple genes, Thomas et al. (2020) concluded that Ptilocolepidae is a monophyletic unit, sister to Hydroptilidae.

### ﻿Other checklists, catalogs, and bibliographies

In preparing this catalog, several published (or electronic) resources concerning Trichoptera fauna were consulted. In all cases, the accuracy of the names, citations, or listings in these works were checked and corrected as necessary before inclusion in the present catalog. However, as these former works may be useful to the user of this catalog in further research on the microcaddisfly fauna, these works are listed and discussed below.

The world catalog, ‘Trichopterorum Catalogus’, Volumes I–XV + Index, 1960–1973, by F.C.J. Fischer is an indispensable and first source of taxonomic and associated literature pertaining to Trichoptera. The catalog and its supplements cover all literature from 1758 to the end of 1960. Volume II and Volume XII (Fischer 1961, 1971) cover literature addressing Hydroptilidae, making them important resources for this catalog. The world bibliography, ‘Bibliographia Trichopterorum’, by A.P. Nimmo (1996) is another important compilation of trichopteran literature references; Volume 1, the only published volume, covers literature from 1961 to 1970. The searchable World ‘Trichoptera Checklist’ is available over the World Wide Web [www.entweb.sites.clemson.edu/database/trichopt/]. Morse (1997b) discussed the format of this checklist. Another important source of information was the checklist of Neotropical Trichoptera fauna first organized by Flint et al. (1999a) and updated by Holzenthal and Calor (2017). This checklist was extremely helpful both for providing an excellent starting point for covering the Neotropical region, an extremely diverse region for microcaddisflies, but also for providing an organizational structure that could be reproduced in this catalog. ‘Zoological Record’ and other electronic abstracting services (e.g., ‘Web of Science’) are of paramount importance in accessing the taxonomic literature.

### ﻿Purpose of the catalog

A catalog is a list of nominal species and associated taxonomic and nomenclatural references arranged in a logical, easily accessible format. Catalogs are important tools to anyone requiring knowledge of currently accepted names, including synonyms and distributional data. Because the binomen is usually the starting point of the information storage and retrieval system afforded by the Linnaean hierarchy, an accurate list of currently accepted species names is essential for anyone needing information about a species, be it for basic or applied research. By accumulating and organizing all the previously published microcaddisfly (Hydroptilidae, Ptilocolepidae) taxonomic information into a single, easily accessed source, I hope to facilitate and stimulate further exploration and research on the fauna. Furthermore, I hope that this catalog benefits research beyond general Trichoptera systematics, such as ecology, behavior, conservation, and the application of Trichoptera as biological indicators of water quality.

### ﻿Format of the catalog

This catalog follows the format used in the Neotropical Trichoptera catalogs produced by Flint et al. (1999a) and Holzenthal and Calor (2017): organization is alphabetic by family, subfamily, tribe (when applicable), genus, and species. A single genus currently considered incertae sedis within Trichoptera, but previously included within Hydroptilidae, is listed at the end. Valid family names are presented in **boldface** type, centered on the page, and followed by the author. A family synonymy follows, which includes the currently recognized, valid family name, followed by its author, date and bibliographic citation of publication, and page number on which the name was formally established. Following this, in square brackets, the type genus with author and date is presented. Other citations containing other important nomenclatural acts, taxonomic revisions, or generic keys are next included with annotations added in square brackets. Following the family synonymy, introductory information, including literature citations, of a general nature is given concerning distribution, diversity, taxonomy, biology, habitat, and knowledge of larval states, if available. For simplicity, unless otherwise specified, the larvae referred to are final instar larvae.

In hierarchical order, valid subfamily and tribe names are next presented in **boldface** type, centered on the page, and followed by the author, each followed by the synonymy. The currently recognized, valid taxon name is followed by its author, date and bibliographic citation of publication, and page number on which the name was formally established. Following this, in square brackets, the type genus with author and date is presented. Other citations containing other important nomenclatural acts, taxonomic revisions, or generic keys are next included with annotations given in square brackets. Following the subfamily or tribe synonymy, introductory information similar to that presented for the family is given.

Valid generic names are next presented in **boldface** type, centered on the page, and followed by the author. A generic synonymy follows. The currently recognized, valid genus name is followed by its author, date and bibliographic citation of publication, and page number on which the name was formally established. Following this, in square brackets, the type species in its original combination with author and date is presented, the manner in which the type species was established (e.g., original designation, monotypy, subsequent selection, etc.), and the family in which it was originally described if different from the current family. Other citations containing other important nomenclatural acts, generic revisions, or larval descriptions are included next with annotations added in square brackets. Generic synonyms follow, in chronological order (oldest names first), and are presented in the same format and with the same information as presented for the valid genus name, with the addition of the citation where the generic synonymy was established. Subgeneric names are presented as generic synonyms and with the same information, but the subgeneric status is so indicated and the citation included. Following the generic synonymy, introductory information on the genus, similar to that presented for the family, is given.

All currently recognized, valid species and subspecies names (specific epithets only), in their current orthography, are then listed in alphabetical order and in ***boldface italic*** type. Fossil species (and genera) are preceded by the symbol ♰. In cases where subgenera are used, the subgenus name follows the specific epithet, in parentheses. Each species name is followed by author, date and bibliographic citation of publication, and page number on which the name was formally established. Following, in square brackets, the type locality is presented, annotated for clarity, but otherwise given as indicated in the original publication, except the country of origin is always listed first. The type depository is then given if known, and indicated thus if unknown, according to the institution codes presented below. Sex of the type is presented next, if known, and so indicated if not known. Sex of type is followed (separated by a semicolon) by the sex or stage of any other specimens illustrated and described with the type specimen (these also separated by semicolons). Finally, still in square brackets and separated by a semicolon, the genus of the original combination, or the original orthography of the specific epithet if different from present orthography, is presented. In addition, citations for any significant publications containing redescriptions, lectotype or neotype designations or other nomenclatural acts, systematic revisions, larval descriptions, or new distribution records follow their appropriate species’ entries. Synonyms are indicated in *italics*, preceded by an em dash (—), and listed in chronological order (if more than one) and in their present orthography under the valid species entry. All species-group synonyms are included in the catalog. Information presented for synonyms is the same as presented for the senior name (date and bibliographic citation of the synonymy, sex of type, type depository, genus of original combination or original orthography), but also includes the date and bibliographic reference where the synonymy was established. Lastly, for each species entry the distribution by country, based on published records, is presented.

In addition to original citations and important taxonomic or nomenclatural works, all recent and important literature published after 1960 is included in the catalog. However, the extensive bibliographies presented by Fischer (1960–1973) for the literature prior to 1961 are NOT repeated in this catalog if not of primary importance. The reader is referred to Fischer’s catalog, ‘Trichopterorum Catalogus’ for this additional literature.

All literature cited in the introduction and catalog itself is listed in the References section. The complete title of the journal, book, or other bibliographic source is given to assist the user in obtaining literature. In all cases, the original citation was consulted by the author in compiling the catalog to ensure accuracy of information or to check date of issue.

The catalog includes all literature known to me up to the end of 2020. The user is cautioned, however, that I make no claims to have included all the literature published in 2020, and certainly not later, but I have done my best to do so. Some literature is not abstracted in ‘Zoological Records or Web of Science’ until several years after its date of publication and thus may have been missed. Again, the user should check the appropriate bibliographic sources to ensure complete coverage and overlap by several years the bibliography in this catalog when searching the literature in the future.

The catalog ends with an Index that lists all names presented in the catalog and the primary page number where the name occurs. Format of names in the index generally follows that presented in the catalog: valid species and subspecies epithets are presented in bold italics, followed by the current genus in italics; synonyms of species or subspecies names are presented in italics, followed by the current genus in italics. The original orthography of species names, including synonyms, is also indexed, but referred to the species in its current combination and orthography. For subspecies names, the trinomen is also indexed, but referred to the name in combination with the nominotypical name. Homonyms are also indexed, but with the author of the name and date of publication included. Valid genus names are presented in bold, followed by the family in square brackets. Generic synonyms are presented in italics, except that currently recognized subgeneric names are presented in bold italics, both followed by the family in square brackets. Fossil species are followed by the symbol ♰.

### ﻿List of type depositories

Depository codes have been sourced from the GBIF Registry of Scientific Collections, The Insect and Spider Collections of the World Website, and original publications (Evenhuis 2021; GBIF 2022). If a code could not be found in these three sources, a code was assigned for use in this catalog.

**AMGS** Albany Museum, Grahamstown, Cape Province, South Africa

**AMNZ** Auckland Institute and Museum, Auckland, New Zealand

**ANIC** Australian National Insect Collection, CSIRO, Canberra City, Australian Capital Territory, Australia

**ANSP** Academy of Natural Sciences, Philadelphia, Pennsylvania, USA

**CAS** California Academy of Sciences, San Francisco, California, USA

**CBGP** Centre de Biologie pour la Gestion des Populations, Campus International de Baillarguet, Montferrier-sur-Lez, France

**CBM-ZI** Natural History Museum and Institute of Chiba, Chiba, Japan

**CLEV** Cleveland Museum of Natural History, Cleveland, Ohio, USA

**CMNH** Carnegie Museum of Natural History, Pittsburgh, Pennsylvania, USA

**CMNZ** Canterbury Museum, Christchurch, New Zealand

**CMOR** Moretti collection, University of Perugia, Perugia, Italy

**CNC** Canadian National Collection of Insects, Ottawa, Ontario, Canada

**CNIN** Colección Nacional de Insectos, Instituto de Biología, Universidad Nacional Autónoma de México, Mexico City, Mexico (formerly IBUNAM)

**Collection Banks** private collection, Nathan Banks, likely deposited in MCZ

**Collection Henderson** private collection, Ian M. Henderson, Massey University, Palmerston North, New Zealand

**Collection Malicky** private collection, Hans Malicky, Lunz am See, Austria

**Collection Mey** private collection, Wolfram Mey, Berlin, Germany

**Collection Moretti** private collection, G. Moretti, University of Perugia, Italy

**Collection Oláh** private collection, János Oláh, Debrecen, Hungary, presently under protection of HNHM

**Collection Tillyard** private collection, R. J. Tillyard, deposited in NHMUK

**Collection Wichard** private collection, Wilfried Wichard, Bonn, Germany

**COZEM** Colección Zoológica Dr. Eustorgio Méndez, Instituto Conmemorativo Gorgas de Estudio de la Salud, Panama City, Panama

**CUAC** Clemson University, Clemson, South Carolina, USA

**CZMA** Coleção Zoológica do Maranhão, Universidade Estadual do Maranhão, Caxias, Maranhão, Brazil

**DSIR** Department of Scientific and Industrial Research, Cawthron Institute, Nelson, New Zealand

**DZRJ** Coleção Entomológica Prof. José Alfredo Pinheiro Dutra, Departamento de Zoologia, Universidade Federal do Rio de Janeiro, Rio de Janeiro, Brazil

**DZUSC** M. A. González collection, Department of Zoology, University of Santiago de Compostela, Santiago de Compostela (La Coruña), Galicia, Spain

**EIHU** Entomological Institute of Hokkaido University, Sapporo, Hokkaido, Japan

**EMEC** Essig Museum of Entomology, University of California, Berkeley, California, USA

**ETSI** Departamento de Zoología y Entomología, Escuela Técnica Superior de Ingeniería de Montes, Madrid, Spain

**ESUW** University of Wyoming, Laramie, Wyoming, USA (includes D. G. Denning’s personal collection)

**FHCU** Facultad de Humanidades y Ciencias (Departamento de Artropodos), Universidad de la Republica, Montevideo, Uruguay

**FSCA** Florida State Collection of Arthropods, Gainesville, Florida, USA

**HAUZ** Henan Agricultural University, Zhengzhou, Henan, China

**HNHM** Hungarian Natural History Museum, Budapest, Hungary

**HUAT** Hacettepe University, Beytepe, Ankara, Turkey

**IBSS-RAS** Institute of Biology and Soil Sciences of Russian Academy of Sciences, Vladivostok, Russia

**ICN** Institute of Natural Sciences, Universidad Nacionale de Colombia, Bogotá, Colombia

**IFML** Instituto Fundación Miguel Lillo, Tucumán, Argentina

**INBIO** Instituto Nacional de Biodiversidad, Santo Domingo de Heredia, Costa Rica

**INHS** Illinois Natural History Survey, Champaign, Illinois, USA

**INPA** Nacional de Pesquisas da Amazônia, Manaus, Amazonas, Brazil

**IRSNB** Institut Royal des Sciences Naturelles de Belgique, Brussels, Belgium

**IZSK** I. I. Schmalhausen Institute of Zoology of the National Academy of Sciences of Ukraine, Kiev, Ukraine

**KMUL** Karl-Marx-University, Leipzig, Germany

**KUM** Kyoto University Museum, Kyoto, Japan

**LIPI** Indonesian Institute of Sciences, Jakarta (Bogor), West Java, Indonesia

**LNKD** Landessammlung für Naturkunde, Karlsruhe, Germany

**MACN** Museo Argentina de Ciencias Naturales “Bernardino Rivadavia”, Buenos Aires, Argentina

**MBBJ** Museum Zoologicum Bogoriense, Bogor, Indonesia

**MBCG** Museo di Scienze Naturali “Enrico Caffi”, Bergamo, Italy

**MCZ** Museum of Comparative Zoology, Harvard University, Cambridge, Massachusetts, USA

**MDLA** Museu do Dundo, Luanda, Angola

**MHNJP** Museo de Historia Natural “Javier Prado”, Universidad Nacional Mayor de San Marcos, Lima, Peru

**MIUP** Universidad de Panamá Museo de Invertebrados, Panama

**MLPA** Museo de la Plata, Universidad Nacional de La Plata, La Plata, Argentina

**MNHN** Muséum National d’Histoire Naturelle, Paris, France

**MNRJ** Museu Nacional, Universidade Federal do Rio de Janeiro, Rio de Janeiro, Brazil (including F. Müller material)

**MPEG** Museu Paraense Emílio Goeldi, Belém, Pará, Brazil

**MPMP** National Museum of the Philippines, Manila, Philippines

**MRAC** Musée Royal de l’Afrique Centrale, Tervuren, Belgium

**MZBS** Museo de Zoologia, Barcelona, Spain

**MZHF** Finnish Museum of Natural History, Helsinki, Finland

**MZLS** Musée Zoologique, Lausanne, Switzerland

**MZLU** Lund University, Lund, Sweden

**MZPW** Polish Academy of Science, Museum and Institute of Zoology, Warsaw, Poland

**MZUFBA** Museu de Zoologia da Universidade Federal da Bahia, Salvador, Brazil

**MZUSP** Museu de Zoologia, Universidade de São Paulo, São Paulo, Brazil

**MZVU** Museum of Zoology, Vilnius University, Vilnius, Lithuania

**NHMUK**Natural History Museum London, United Kingdom [formerly British Museum (Natural History), BMNH or NHM],

**NAUJ** Nanjing Agricultural University, Nanjing, Jiangsu, China

**NHMB** Naturhistorisches Museum, Basel, Switzerland

**NHMW** Naturhistorisches Museum Wien, Vienna, Austria

**NHRS** Naturhistoriska Riksmuseet, Stockholm, Sweden

**NIBR** National Institute of Biological Resources, Incheon, South Korea

**NMID** National Museum of Ireland, Dublin, Ireland

**NMNH** National Museum of Natural History, Washington, DC, USA [formerly United States National Museum, USNM]

**NMNS** National Museum of Natural Science, Taichung, Taiwan

**NMPC** National Museum (Natural History), Prague, Czech Republic

**NMPG** Museum der Natur-Gotha, Gotha, Germany

**NMV** Museums Victoria, Melbourne, Victoria, Australia [formerly National Museum of Victoria]

**NMZ** National Museum of Zimbabwe, Harare, Zimbabwe

**NTM** Museum and Art Gallery of the Northern Territory, Darwin, Northern Territory, Australia

**NZAC** New Zealand Arthropod Collection, Landcare Research, Auckland, New Zealand

**NZSI** National Zoological Collection, Zoological Survey of India, Calcutta, West Bengal, India

**PSUC** Frost Entomological Museum, Pennsylvania State University, University Park, Pennsylvania, USA

**QMB** Queensland Museum, South Brisbane, Queensland, Australia

**QMOR** Collection Entomologique Ouellet-Robert, University of Montreal, Montreal, Quebec, Canada

**RMNH** Naturalis Biodiversity Centre, Leiden, Netherlands [formerly Rijksmuseum van Natuurlijke Historie]

**ROM** Royal Ontario Museum, Toronto, Ontario, Canada

**SAMC** Iziko Museum of Capetown, Cape Town, South Africa [formerly South African Museum]

**SOFM** National Museum of Natural History, Sofia, Bulgaria

**TAU** Tel Aviv University, Tel Aviv, Israel

**UMQ** University of Montreal, Montreal, Quebec, Canada

**UMSP** University of Minnesota, St. Paul, Minnesota, USA

**UNHC** University of New Hampshire, Durham, New Hampshire, USA

**UOBF** University of Ouagadougou, Ouagadougou, Burkina Faso

**UPLB** Museum of Natural History, University of the Philippines Los Baños, Los Baños, Laguna, Philippines

**USCM** “Luis Iglesias” Museum of Natural History, University of Santiago de Compostela, Santiago de Compostela (La Coruña), Galicia, Spain

**VNMN** Vietnam National Museum of Nature, Hanoi, Vietnam

**WAM** Western Australia Museum, Perth, Western Australia, Australia

**ZIN** Academy of Sciences, Zoological Institute, St. Petersburg, Russia

**ZMHB** Museum für Naturkunde der Humboldt-Universität, Berlin, Germany

**ZMUA** Zoölogisch Museum, Instituut voor Taxonomische Zoologie, Universiteit van Amsterdam, Amsterdam, Netherlands

**ZMUB** Zoological Museum, University of Bergen, Bergen, Norway

**ZMUC** Zoological Museum, University of Copenhagen, Copenhagen, Denmark

**ZMUH**Zoologisches Institut und Zoologisches Museum, Universität von Hamburg, Hamburg, Germany [formerly Zoologische Staatsinstitut und Zoologisches Museum]

**ZRC** Zoological Reference Collection, Raffles Museum of Biodiversity Research, National University of Singapore, Singapore

**ZSM** Zoologische Staatssammlung München, Munich, Germany

## ﻿Catalog

### ﻿Family HYDROPTILIDAE Stephens, 1836

Hydroptilidae Stephens, 1836: 151 [type genus: *Hydroptila* Dalman, 1819]. —McLachlan 1880: 501 [revision synopsis]. —Betten 1934: 145 [key to genera]. —Barnard 1934: 390 [key to African genera]. —Milne 1936: 74 [key to North American species]. —Mosely 1939b: 252 [key to British genera]. —Ross 1944: 117 119 [generic key to larvae and adults]. —Kimmins 1951: 194 [key to genera of India]. —Leader 1970: 121 [setae]. —Neboiss 1977: 39 [key to Tasmanian genera]. —Marshall 1979b: 135 [family monograph; key to genera]. —Blickle 1979: 3 [key to genera America north of Mexico]. —Wells 1990b: 365 [key to genera of North Sulawesi]. —Wells and Dudgeon 1990: 162 [key to males of Hong Kong]. —Wells 1991: 498 [key to males and mature larvae of genera in New Guinea]. —Botosaneanu 1992: 39 [key to genera in the Levant]. —Wells and Andersen 1995: 145 [key to genera and species in Tanzania]. —Wiggins 1996: 1–457 [larvae of the North American genera]. —Solem and Gullefors 1996: 234 [key to genera for larvae in North Europe]. —Moulton and Stewart 1996: 89 [keys to the larvae and adults of genera and species in the Interior Highlands of North America]. —Waringer and Graf 1997: 72 [atlas of species in Australia central Europe and Palearctic region; key to larvae]. —Wells 1997: 1–28 [checklist key to Australian larvae]. —Kachalova in Medvedev 1998: 179 [key to the genera of the European part of the USSR]. —Flint et al. 1999a: 82 [catalog of Neotropical species]. —Wallace et al. 2003: 74 [generic key to larvae of United Kingdom and Ireland]. —Posada-García and Roldán-Pérez 2003: 183 [generic key to Colombian]. —Pescador et al. 2004: 48 [key to final instar larvae of Florida USA]. —Waringer and Graf 2011: 72 [key to European larvae]. —Wichard 2013: 37 [key to fossil Hydroptilidae in Baltic amber]. —Rinne and Wiberg-Larsen 2017: 38 [key to larvae of Finland]. —Armitage and Harris 2018b: 95 [review of diversity of Panama]. —Wells 2020: 24 [review of Australian cases].

The family Hydroptilidae exhibits a cosmopolitan distribution, with members occurring in all major faunal regions except for Antarctica. Currently recognized within the family are six distinct subfamilies, containing more than 2,600 species: Hydroptilinae, Leucotrichiinae, Neotrichiinae, Ochrotrichiinae, Orthotrichiinae, and Stactobiinae. Each subfamily can be characterized by fundamental morphological features of the adult, larval, and pupal stages (Marshall 1979b). Despite the heterogeneous nature of the family, these features unite the subfamilies and can be used to separate them from the genera of Ptilocolepidae. Both cool- and warm-adapted genera occur in a variety of habits, including swiftly flowing montane streams, splash zones of waterfalls, seeps, rivers of varying sizes, and even still waters. Some larvae are detritus feeders, while other groups specialize on the intracellular contents of filamentous green algae.

### ﻿Subfamily HYDROPTILINAE Stephens, 1836

Hydroptilidae Stephens, 1836: 151 [type genus: *Hydroptila* Dalman, 1819]. —Marshall 1979b: 161 [reviewed as tribe Hydroptilini]. —Wells 1987: 133 [biogeography of *Oxyethira* group].

Hydroptilinae consists of 26 genera occurring in all biogeographic regions of the world, excluding the polar regions. As Marshall (1979b) noted, the subfamily may seem to be very heterogenous and varied in morphological features of both the adults and larvae, but can be united by a number of basic similarities, including features of the adult thorax and male genitalia and the larval association with filamentous green algae. Marshall (1979b) further divided the subfamily into three subgroups based on characteristics of the male and female genitalia and general larval appearances and habitats. The *Agraylea* group, including the genera *Agraylea*, *Allotrichia*, *Dhatrichia*, *Microptila*, and *Ugandatrichia*, is based on the larger and more generalized appearance of the adults in comparison to other hydroptilids and the distinctive male genitalia. The *Hydroptila* group, essentially the genus *Hydroptila*, is also united by the distinct form of the male genitalia and can be separated from other groups and genera by the postoccipital scent caps of the males and the absence of ocelli. The *Oxyethira* group, including *Oxyethira*, *Paroxyethira*, *Tricholeiochiton*, and *Xuthotrichia*, exhibits more variety than the others in features of the adults and the genitalic form, but is united by similarities in the larvae. Larval stages are unknown for the genera *Aenigmatrichia*, *Austratrichia*, *Cyclopsiella*, *Jabitrichia*, *Kholaptila*, *Maeyaptila*, *Missitrichia*, *Mulgravia*, *Paucicalcaria*, *Sutheptila*, *Tangatrichia*, *Vietrichia*, *Wlitrichia*, and *Xuthotrichia*.

#### Genus *Acanthotrichia* Wells, 1982

*Acanthotrichia* Wells, 1982: 267 [type species: *Acanthotrichiabilamina* Wells, 1982, original designation]. —Wells 1985b: 15 [larva; pupa; case].

*Acanthotrichia* is a monotypic genus occurring in Australia. Based on features of the male genitalia, it was placed in Hydroptilinae and may be most closely related to the genera *Tricholeiochiton* and *Paroxyethira* (Wells 1982). The larval stage was described by Wells (1985b).

***bilamina*** Wells, 1982: 269 [type locality: Victoria, Genoa River, near Wangarabell; NMV; ♂]. —Wells 1985b: 16 [larva, case]. —Neboiss 1986: 73 [atlas; ♂].

**Distribution.** —Australia.

#### Genus *Acritoptila* Wells, 1982

*Acritoptila* Wells, 1982: 262 [type species: *Acritoptilaglobosa* Wells, 1982, original designation]. Wells 1985b: 15 [larva; pupa; case]. —Wells 1997: 1 [checklists; larvae; species]. —Wells and Johanson 2014: 1 [generic review of New Caledonian species; key to New Caledonian species].

*Acritoptila* consists of 16 species occurring in Australia and New Caledonia. It can be distinguished from members of *Austratrichia* and *Mulgravia* by differences in the inferior appendages of the male genitalia (Wells 1982). Wells (1985b) described the larvae of *A.globosa* and *A.margaretae* and stated that the larvae of *Acritoptila* are indistinguishable from those of *Hellyethira*.

***>amphapsis*** Kelley, 1989: 191 [type locality: New Caledonia, Honailu River; BPBM; ♂]. —Wells 1995: 238 [case; distribution]. —Wells and Johanson 2014: 14 [♂; distribution]. —Johanson and Wells 2019: 92 [checklist].

**Distribution.** —New Caledonia.

***capistra*** Wells, 1990c: 117 [type locality: [Australia] NE Queensland, Yuccabine Creek; NMV; ♂].

**Distribution.** —Australia.

***chiasma*** Kelley, 1989: 192 [type locality: New Caledonia, mountain stream up Boulari River; BPBM; ♂]. —Wells and Johanson 2014: 9 [♂; ♀; distribution]. —Johanson and Wells 2019: 92 [checklist].

**Distribution.** —New Caledonia.

***crinita*** Kelley, 1989: 193 [type locality: New Caledonia, headwaters of Honailu River; BPBM; ♂]. —Wells 1995: 238 [distribution]. —Wells and Johanson 2014: 7 [♂; ♀; distribution]. —Johanson and Wells 2019: 92 [checklist]. —*karika* Oláh & Johanson, 2010a: 7 [type locality: New Caledonia, Province Nord, 50 m upstream bridge on Hienghene-Tnédo road, 3.9 km S summit of Mt Tneda, 2,2 km E Tnédo, 20°43.085'S, 164°49.928'E, 29 m; ♂]. —Wells and Johanson 2014: 7 [♂; ♀; distribution; to synonymy].

**Distribution.** —New Caledonia.

***csavar*** Oláh & Johanson, 2010a: 6 [type locality: New Caledonia, d’Amieau Fauna Reserve; MNHN; ♂]. —Wells and Johanson 2014: 11 [♂; distribution]. —Johanson and Wells 2019: 92 [checklist].

**Distribution.** —New Caledonia.

***disjuncta*** Kelley, 1989: 193 [type locality: New Caledonia, mountain stream up Boulari River; BPBM; ♂]. —Wells 1995: 235 [case; distribution]. —Oláh and Johanson 2010a: 7 [distribution]. —Wells and Johanson 2014: 4 [♂; ♀; distribution]. —Johanson and Wells 2019: 92 [checklist].

**Distribution.** —New Caledonia.

***forficata*** Wells & Johanson, 2014: 13 [type locality: New Caledonia, Province Sud, Monts des Koghis, ca 800 m S Koghi Restaurant, 22.18406°S, 166.50383°E, 420 m; MNHN; ♂]. —Johanson and Wells 2019: 92 [checklist].

**Distribution.** —New Caledonia.

***globosa*** Wells, 1982: 265 [type locality: Western Australia, Harvey River, near Harvey Falls, 15 km E. of Harvey; NMV; ♂; ♀]. —Wells 1985b: 15 [larva, case]. —Neboiss 1986: 69 [atlas; ♂; ♀].

**Distribution.** —Australia.

***glossocercus*** Kelley, 1989: 193 [type locality: New Caledonia, mountain stream up Boulari River; BPBM; ♂]. —Wells and Johanson 2014: 11 [♂; distribution]. —Johanson and Wells 2019: 92 [checklist].

**Distribution.** —New Caledonia.

***hamatus*** Wells, 1982: 265 [type locality: Queensland, Mothar Mountain, 12 km SE. of Gympie; NMV; ♂]. —Neboiss 1986: 69 [atlas; ♂].

**Distribution.** —Australia.

***macrospina*** Wells & Johanson, 2014: 19 [type locality: New Caledonia, Province Nord, Wemwâdiu stream, 850 m E summit Kögi Mtn, 5 m upstream road, about 200 m S Tiwaka River, 20°49.020'S, 165°14.165'E, 24 m; MNHN; ♂]. —Johanson and Wells 2019: 92 [checklist].

**Distribution.** —New Caledonia.

***margaretae*** Wells, 1982: 265 [type locality: Western Australia, Harvey River below Harvey Falls; NMV; ♂; ♀]. —Wells 1985b: 15 [case]. —Neboiss 1986: 69 [atlas; ♂; ♀].

**Distribution.** —Australia.

***ouenghica*** Wells, 1995: 235 [type locality: [New Caledonia], Ouenghi River, nr Boulouparis; ANIC; ♂]. —Wells and Johanson 2014: 17 [♂; distribution]. —Johanson and Wells 2019: 92 [checklist].

**Distribution.** —New Caledonia.

***parallela*** Wells & Johanson, 2014: 13 [type locality: New Caledonia, Province Nord, Mt Panié, stream at camp, 20.58139°S, 164.76444°E, 1310 m; MNHN; ♂]. —Johanson and Wells 2019: 92 [checklist].

**Distribution.** —New Caledonia.

***pearsoni*** Wells, 1990c: 115 [type locality: [Australia] NE Queensland, Yuccabine Creek; NMV; ♂].

**Distribution.** —Australia.

***planichela*** Kelley, 1989: 194 [type locality: New Caledonia, mountain stream up Boulari River; BPBM; ♂]. —Wells and Johanson 2014: 15 [♂; distribution]. —Johanson and Wells 2019: 92 [checklist].

**Distribution.** —New Caledonia.

#### Genus *Aenigmatrichia* Wells & de Moor, 2020

*Aenigmatrichia* Wells & de Moor, 2020: 503 [type species: *Aenigmatrichiaasymmetrica* Wells & de Moor, 2020, original designation].

*Aenigmatrichia* is a monotypic genus occurring in Angola. Based on a combination of features shared with the Tanzanian genus *Tangatrichia*, Wells and de Moor placed *Aenigmatrichia* in the subfamily Hydroptilinae, and also noted similarities with the genera *Oxyethira*, *Pseudoxyethira*, and *Catoxyethira*.

***asymmetrica*** Wells & de Moor, 2020: 505 [type locality: Angola, Moxico Province, Collecting event 3 — Lungue Bungo River, along marshy river banks with swift flowing river containing trailing and marginal aquatic vegetation, light trap downstream of road-bridge, -12.58391, 18.66511; AGMS; ♂; ♀].

**Distribution.** —Angola.

#### Genus *Agraylea* Curtis, 1834

*Agraylea* Curtis, 1834: 217 [type species: *Agrayleasexmaculata* Curtis, 1834, subsequent designation by Westwood 1840]. —Hagen 1864b: 115 [comments on larvae and case]. —McLachlan 1880: 505 [revision]. —Mosely 1939b: 253 [key to the British species]. —Solem 1972: 79 [key to full-grown larvae]. —Marshall 1979b: 193 [generic review]. —Blickle 1979: 6 [key to species of America north of Mexico]. —Kachalova in Medvedev 1998: 182 [key to the species of the European part of the USSR].

*Agraules* Agassiz, 1846: 32 [Unjustified emendation of *Agraylea* according to Fischer 1961].

*Hydrorchestria* Kolenati, 1848: 103 [type species: *Agrayleasexmaculata* Curtis, 1834, subsequent designation by Kimmins 1950]. —Kimmins 1950: 58 [to synonymy].

♰ *Nanoagraylea* Botosaneanu, 1995b: 2 [fossil subgenus of *Agraylea*].

The genus Agraylea currently contains two subgenera. The subgenus Agraylea consists of eleven species, including three fossil species known from Baltic amber, and has a Holarctic distribution. The subgenus Nanoagraylea consists of three fossil species (Botosaneanu 1995b). According to Marshall (1979b), the extant members of the genus are most similar morphologically to *Allotrichia*, from which they differ in hindwing venation, but features of the male genitalia are similar to those of the genera *Dhatrichia* and *Ugandatrichia*. Larval descriptions of *A.cognatella*, *A.multipunctata*, and *A.sexmaculata* were given by Solem (1972), Nielsen (1948), and Barnard (1971), respectively.

***costello*** (*Agraylea*) Ross, 1941b: 15 [type locality: [Canada], Costello Lake, Algonquin Park, Ontario, Ontario Fisheries Research Laboratory, Cage number 4; INHS; ♂]. —Ross 1944: 122 [♂]. —Roy and Harper 1975: 1083 [distribution]. —Roy and Harper 1979: 150 [checklist]. —Blickle 1979: 47, 57 [checklist; ♂]. —Parker and Voshell 1981: 4 [distribution]. —Etnier 2010: 485 [distribution]. —Myers et al. 2011: 105 [distribution].

**Distribution.** —Canada, U.S.A.

♰ ***cretaria*** (*Nanoagraylea*) Botosaneanu, 1995b: 2 [type locality: [United States], Sayreville, Middlesex Co., New Jersey; AMNH; ♀; in amber; ♂]. —Wichard and Lüer 2003: 132 [checklist]. —Eskov et al. 2008: 78 [checklist]. —Wichard 2013: 39 [species review]. —Ivanov and Melnitsky 2017: 131 [checklist].

**Distribution.** —New Jersey amber.

♰ ***cumsacculo*** (*Agraylea*) Wichard, 2013: 40 [type locality: [Baltic region]; ZMHB; ♂; in amber].

**Distribution.** —Baltic amber.

***dactylina*** (*Agraylea*) Zhou, Yang, & Morse, 2016: 204 [type locality: China, Si-chuan Province, Kang-ding County, unnamed waterfall, tributary of Da-du River, 100 m upstream of G13 18 at 2824.9 km stone marker, N30.0665°, E102.1178°, 1675 m; NAUJ; ♂].

**Distribution.** —China.

***drosima*** (*Agraylea*) Navás, 1917a: 67 [type locality: [Spain], Zaragoza; depository not designated; ♂; as *Agraylia*]. —Malicky 2005b: 546 [checklist].

**Distribution.** —Spain.

♰ ***glaesaria*** (*Agraylea*) Wichard, 2013: 38 [type locality: [Baltic region]; ZMHB; ♂; in amber].

**Distribution.** —Baltic amber.

***insularis*** (*Agraylea*) (Hagen, 1865a): 219 [type locality: locality not given; depository not designated; ♂; in *Hydrorchestria*]. —Eaton 1873: 148 [to *Agraylea*]. —McLachlan 1880: 508 [revision]. —Nybom 1948: 4 [distribution]. —Malicky 2005b: 546 [checklist].

**Distribution.** —Portugal.

♰ ***lentiginosa*** (*Nanoagraylea*) Botosaneanu, Johnson, & Dillon, 1998: 222 [type locality: United States, New Jersey; ANSP; ♂; in amber]. —Wichard and Lüer 2003: 132 [checklist]. —Eskov et al. 2008: 78 [checklist]. —Ivanov and Melnitsky 2017: 131 [checklist].

**Distribution.** —New Jersey amber.

***multipunctata*** (*Agraylea*) Curtis, 1834: 217 [type locality: “Britain”; type not designated]. —McLachla 1865: 92 [♂]. —Eaton 1873: 147 [♂]. —McLachlan 1880: 506 [revision; ♂]. —Morton 1886: 269 [notes on larva and case]. —Morton 1899b: 281 [distribution]. —Morton 1904: 323 [distribution]. —Morton 1905: 74 [♂; distribution]. —Banks 1907a: 49 [catalogue]. —Martynov 1924: 40 [♂]. —Sibley 1926: 204 [biology]. —Betten 1934: 147 [larva; ♂; distribution]. —Martynov 1934: 114 [♂]. —Mosely 1939b: 253 [♂]. —Tjeder 1940: 10 [distribution]. —Kimmins 1943: 154 [distribution]. —Ross 1944: 122 [♂; ♀; distribution]. —Denning 1947b: 170 [distribution]. —Nielsen 1948: 41 [larva]. —Berg 1948: table 14, between pages 124–125 [distribution]. —Ross and Spencer 1952: 46 [distribution]. —Morse and Blickle 1953: 72 [checklist]. —Nybom 1960: 17 [checklist]. —Fischer 1961: 87 [cited as senior synonym]. —Spuris 1962: 57, 61, 70 [distribution]. —Neboiss 1963: 613 [distinct from *A.sexmaculata*]. —Spuris 1964: 12 [distribution]. —Etnier 1965: 146 [checklist]. —Botosaneanu 1967: 294 [distribution]. —Spuris 1972: 19, 21, 23, 27, 30 [checklist]. —Obr 1975: 128 [distribution]. —Watts 1976: 15 [pupa]. —Botosaneanu and Malicky 1978: 341 [checklist]. —Etnier and Schuster 1979: 17 [distribution]. —Roy and Harper 1979: 150 [checklist]. —Blickle 1979: 47, 57 [checklist; ♂]. —Swegman et al. 1981: 132 [distribution]. —Waltz and McCafferty 1983a: 9 [distribution]. —Malicky 1983b: 53, 57 [atlas; ♂; ♀]. —Huryn and Foote 1983: 790 [distribution]. —Steven and Hilsenhoff 1984: 163 [distribution]. —Lake 1984: 219 [distribution]. —Kumanski 1985: 114 [♂]. —Wiberg-Larsen 1985: 40 [checklist]. —Andersen and Wiberg-Larsen 1987: 168 [checklist]. —Botosaneanu and Levanidova 1988: 173 [distribution; comparison with *A.cognatella*]. —Harper 1989: 541 [distribution]. —Spuris 1989: 15[checklist]. —Stroot 1989: 157 [larval coloration patterns]. —Wrubleski and Ross 1989: 163 [ecology]. —Stroot 1989: 157 [larval coloration]. —Masteller and Flint 1992: 69 [checklist]. —Ross and Murkin 1993: 27 [ecology]. —Masteller 1993: 134 [distribution]. —Andersen et al. 1993b: 3 [distribution]. —Nógrádi and Uherkovich 1994: 31 [distribution]. —Czachorowski and Prishchepchik 1998: 11 [distribution]. —Uherkovich and Nógrádi 1999: 421 [distribution]. —Wiberg-Larsen and Karsholt 1999: 126 [distribution]. —Huryn and Harris 2000: 193 [distribution]. —Houghton et al. 2001: 504 [distribution]. —Morse et al. 2001: 102 [distribution]. —Gullefors 2002: 138 [checklist]. —Ujvárosi 2002: 384 [distribution]. —Nógrádi and Uherkovich 2002: 130 [distribution]. —Cibaitė 2003a: 10 [checklist]. —Gullefors 2003: 195 [distribution]. —Mey 2003a: 40 [head]. —Malicky 2004a: 66, 73 [atlas]. —Graf and Hutter 2004: 147 [distribution]. —Gullefors 2005a: 118, 119 [distribution]. —Gullefors 2005b: 138 [distribution]. —Hohmann 2005: 106 [checklist]. —Berlin 2005: 128 [distribution]. —Mey 2005b: 119 [distribution]. —Graf et al. 2005: 55 [distribution]. —Lubini-Ferlin and Vicentini 2005: 67 [checklist]. —Zack et al. 2006: 134 [phenology; distribution]. —Morse et al. 2006: 320 [distribution]. —Gullefors 2006: 136, 137 [distribution]. —Chvojka and Komzák 2006: 358 [distribution]. —Robert 2007: 82 [checklist]. —Berlin and Thiele 2007: 49 [checklist]. —Ivanov and Melnitsky 2007: 32 [distribution]. —Gullefors and Johanson 2007: 64 [distribution]. —Višinskienė 2009: 27 [checklist]. —Szczęsny and Godunko 2008: 14 [checklist]. —Chvojka and Komzák 2008: 13 [distribution]. —Ujvárosi et al. 2008: 113 [checklist]. —Schrankel et al. 2008: 90 [checklist]. —Vieira et al. 2009: 257 [distribution]. —Houghton and Holzenthal 2010: 486 [distribution]. —Corallini and Cianficconi 2011: 628 [checklist]. —Djaernes 2011: 19 [sternum V glands]. —Djaernes and Sperling 2011: 86 [sternum V glands]. —Myers et al. 2011: 105 [distribution]. —Ivanov 2011: 194 [checklist]. —Waringer and Graf 2011: 282 [larval synopsis]. —Houghton et al. 2011b: 5 [phenology; habitat]. —Armitage et al. 2011: 13 [checklist]. —Viidalepp et al. 2011: 196 [distribution]. —Zuyderduyn and Tempelman 2013: 25 [distribution]. —O’Connor 2013: 63 [distribution]. —Tempelman and Sanabria 2013a: 20 [distribution]. —Blinn and Ruiter 2013: 280, 290 [biology; distribution]. —Wright et al. 2013: 466 [biology; distribution]. —Tempelman and Sanabria 2013b: 144 [distribution]. —O’Connor and O’Connor 2014: 272 [distribution]. —Chalkley 2014: 13 [distribution]. —Hohmann et al. 2014: 85 [distribution]. —O’Connor 2015: 28, 71 [distribution]. —O’Connor and O’Connor 2015: 203 [distribution]. —Stojanović et al. 2015: 55 [distribution]. —DeWalt et al. 2016: 51 [distribution]. —Vshivkova et al. 2016: 79 [distribution]. —O’Connor and O’Connor 2016: 165 [distribution]. —Chuluunbat et al. 2016: 101 [distribution]. —Potikha and Vshivkova 2016: 363 [distribution]. —Pan’kov and Krasheninnikov 2016: 333 [distribution]. —Smirnova et al. 2016: 401 [distribution]. —Buczyńska et al. 2016: 161 [distribution]. —Houghton 2016: 45 [biology]. —Gullefors 2016: 155 [checklist]. —Melnitsky and Ivanov 2017: 19 [distribution]. —Houghton et al. 2017: 62 [checklist]. —O’Connor and O’Connor 2018: 80 [distribution]. —O’Connor et al. 2018: 23 [distribution]. —Gullefors 2018: 108 [biology; distribution]. —Lock and van Butsel 2018: 3 [distribution]. —Mendez et al. 2019: 128 [checklist]. —Edmonds-Brown 2020: 91 [checklist]. —Houghton and Lardner 2020: 42 [distribution]. —O’Connor 2020: 140 [distribution].

—*argyricola* (Kolenati, 1848): 104 [type locality: [Sweden], Suecia meridionali ad Holmiam, in Dalecarlia; probably NHMW; probably ♂; in *Hydrorchrestia*]. —Eaton 1873: 147 [treated as possibly distinct species; to *Agraylea*]. —McLachlan 1880: 508 [revision]. —Malicky 2005b: 546 [as synonym].

—*cognatella* McLachlan, 1880: 507 [type locality: Finland; depository not designated; ♂]. —Siltala 1908: 14 [distribution]. —Martynov 1924: 40 [♂]. —Martynov 1934: 121 [♂]. —Nybom 1960: 17 [checklist]. —Botosaneanu 1967: 294 [distribution]. —Solem 1970a: 2 [distribution]. —Solem 1972: 77 [larva]. —Botosaneanu and Malicky 1978: 341 [checklist]. —Lillehammer 1978: 256 [distribution]. —Andersen and Wiberg-Larsen 1987: 168 [checklist]. —Botosaneanu and Levanidova 1988: 173 [distribution; comparison with *A.multipunctata*]. —Spuris 1989: 15 [checklist]. —Andersen et al. 1993a: 51 [distribution]. —Andersen et al. 1993b: 2 [distribution]. —Wiggins and Parker 1997: 794 [distribution]. —Zasypkina and Ryabukhin 2001: 45 [checklist]. —Gullefors 2002: 138 [checklist]. —Malicky 2005b: 546 [to synonymy]. —Ivanov 2011: 194 [checklist]. —Andersen and Hagenlund 2012: 135 [distribution]. —Kendrick and Huryn 2014: 280 [distribution]. —Zasypkina 2016: 486 [distribution]. —Gullefors 2016: 155 [checklist].

—*flavida* (Banks, 1907b): 164 [type locality: [United States], Ft. Collins, Colorado; MCZ; ♂; in *Allotrichia*]. —Ross 1938b: 8 [lectotype designated]. —Ross 1944: 295 [to synonymy].

—*fraterna* Banks, 1907b: 164 [type locality: [United States], Falls Church, Va.; MCZ; ♂]. —Milne 1936: 77 [as synonym]. —Ross 1939b: 8 [lectotype designated; to synonymy].

—*multiguttata* Uljanin, 1869: 37, 100 [type locality: Russia; no depository designated, no type specimen designated]. —Fischer 1961: 92 [treated as a synonym].

—*signata* (Banks, 1904a): 215 [type locality: [United States], Virginia, Falls Church; Collection Banks; ♂; in *Allotrichia*]. —Banks 1907a: 50 [catalogue]. —Betten 1934: 149 [checklist]. —Milne 1936: 77 [to synonymy].

**Distribution.** —Austria, Belarus, Canada, Czech Republic, Denmark, England, Estonia, Finland, Germany, Hungary, Iran, Ireland, Italy, Kazakhstan, Latvia, Luxembourg, Mongolia, Netherlands, Norway, Poland, Serbia, Romania, Russia, Scotland, Sweden, Ukraine, U.S.A.

♰ ***parva*** (*Nanoagraylea*) Wichard & Bölling, 2000: 346 [type locality: [United States], New Jersey, Middlesex Co., Sayreville, White Oaks Pit; AMNH; ♂; in amber]. —Wichard and Lüer 2003: 132 [checklist]. —Eskov et al. 2008: 78 [checklist]. —Ivanov and Melnitsky 2017: 131 [checklist].

**Distribution.** —New Jersey amber.

***saltesea*** (*Agraylea*) Ross, 1938a: 114 [type locality: [United States], Montana, Saltese; INHS; ♂]. —Blickle 1979: 47, 57 [checklist; ♂]. —Vineyard 1982: 73 [distribution]. —Ruiter 1999: 165 [distribution]. —Blinn and Ruiter 2013: 290 [biology; distribution]. —Mendez et al. 2019: 118 [checklist].

**Distribution.** —U.S.A.

***sexmaculata*** (*Agraylea*) Curtis, 1834: 217 [type locality: “Britain”, Sept. Lisson Grove (according to Neboiss 1963: 619, “most likely a street near Marylebone Station, London and the northern end of it crosses the Grand Union Canal”); NMV; ♂]. —Kolenati 1848: [revision; distribution; as *Hydrochorestia*]. —Eaton 1873: 147 [treated as synonym of *A.multipunctata*]. —Neboiss 1963: 619 [designation of lectoholotype [sic]; treated as species distinct from *A.multipunctata*]. —Botosaneanu 1967: 294 [distribution]. —Barnard 1971: 253 [larva]. —Botosaneanu and Malicky 1978: 341 [checklist]. —Kumanski 1979: 15 [♂; distribution]. —Moretti et al. 1981: 350, 354 [biology; distribution]. —Moretti and Cianficconi 1981: 201 [checklist]. —Moretti et al. 1981: 239 [ecology; distribution]. —Bagge 1982: 78 [distribution]. —Andrikovics and Ujhelyi 1983: 6 [distribution]. —Malicky 1983b: 53, 57 [atlas; ♂; ♀]. —González and Otero 1983: 118 [distribution]. —Wiberg-Larsen 1985: 40 [checklist]. —Kumanski 1985: 113 [♂]. —Andersen and Wiberg-Larsen 1987: 168 [checklist]. —Sipahiler and Malicky 1987: 106, 107 [distribution]. —O’Connor and O’Hanrahan 1988: 478 [distribution]. —Spuris 1989: 15 [checklist]. —Usseglio-Polatera and Bournau 1989: 254 [distribution]. —Andersen et al. 1990: 52 [distribution]. —González et al. 1990: 212 [checklist]. —Andersen et al. 1993b: 3 [distribution]. —Nógrádi and Uherkovich 1994: 31 [distribution]. —Uherkovich and Nógrádi 1997: 461 [distribution]. —Uherkovich and Nógrádi 1998: 52 [distribution]. —Nógrádi and Uherkovich 1998: 339 [distribution]. —Czachorowski and Prishchepchik 1998: 11 [distribution]. —Cianficconi et al. 1999a: 57 [distribution]. —Wiberg-Larsen and Karsholt 1999: 126 [distribution]. —Malicky 1999c: 96 [distribution]. —Uherkovich and Nógrádi 1999: 421 [distribution]. —Uherkovich and Nógrádi 2001: 95 [distribution]. —Nógrádi and Uherkovich 2001: 297 [checklist]. —Valle 2001: 67 [distribution]. —Mirmoayedi and Malicky 2002: 164 [checklist]. —Ujvárosi 2002: 384 [distribution]. —Nógrádi and Uherkovich 2002: 130 [distribution]. —Gullefors 2002: 138 [checklist]. —Cibaitė 2003a: 10 [checklist]. —Sipahiler 2003b: 33 [distribution]. —Gullefors 2003: 194 [distribution]. —Urbanič 2004: 51 [distribution]. —Malicky 2004a: 66, 73 [atlas]. —Berlin 2005: 128 [distribution]. —Mey 2005b: 119 [distribution]. —Malicky 2005b: 546 [checklist]. —Malicky 2005a: 57 [distribution]. —Sipahiler 2005: 396 [distribution]. —Gullefors 2005a: 118 [distribution]. —Gullefors 2005b: 138 [distribution]. —Hohmann 2005: 106 [checklist]. —Graf et al. 2005: 55 [distribution]. —Lubini-Ferlin and Vicentini 2005: 67 [checklist]. —Sweeney 2006: 300 [distribution]. —Chvojka and Komzák 2006: 358 [distribution]. —Kiss et al. 2006: 139 [biology]. —Ruiz-García et al. 2006: 77 [distribution]. —Waringer and Graf 2006: 356 [distribution]. —Schiess-Bühler and Rezbanyai-Reser 2006: 73 [distribution]. —Robert 2007: 82 [checklist]. —Berlin and Thiele 2007: 49 [checklist]. —Cianficconi et al. 2007b: 576 [distribution]. —Schrankel et al. 2008: 90 [checklist]. —Waringer and Graf 2008: 142 [distribution]. —Ujvárosi et al. 2008: 113 [checklist]. —Szczęsny and Godunko 2008: 14 [checklist]. —González and Menéndez 2008: 188 [distribution]. —Chvojka and Komzák 2008: 13 [distribution]. —Višinskienė 2009: 27 [checklist]. —Oláh 2010: 91 [distribution]. —González and Menénde 2011: 119 [distribution]. —Ivanov 2011: 195 [checklist]. —Waringer and Graf 2011: 282 [larval synopsis]. —Cianficconi et al. 2011: 47 [distribution]. —Valladolid et al. 2011: 501 [distribution]. —Gombeer et al. 2011a: 362 [distribution]. —Gombeer et al. 2011b: 112 [distribution]. —Nowinszky et al. 2011: 231 [biology]. —Viidalepp et al. 2011: 196 [distribution]. —Kiss 2012: 28 [distribution]. —Komzák and Chvojka 2012: 718 [distribution]. —Zuyderduyn and Tempelman 2013: 25 [distribution]. —Tempelman and Sanabria 2013a: 20 [distribution]. —Tempelman et al. 2013: 288 [distribution]. —Mey 2014: 187 [distribution]. —Chalkley 2014: 13 [distribution]. —Hohmann et al. 2014: 85 [distribution]. —Chalkley 2015: 44 [distribution]. —O’Connor 2015: 28, 74 [distribution]. —Stanić-Kroštroman et al. 2015: 85 [distribution]. —Pan’kov and Krasheninnikov 2016: 333 [distribution]. —O’Connor and O’Connor 2016: 165 [distribution]. —Oláh and Beshkov 2016: 100 [distribution]. —Smirnova et al. 2016: 401 [distribution]. —Buczyńska et al. 2016: 161 [distribution]. —Martín et al. 2016: 262 [distribution]. —Gullefors 2016: 155 [checklist]. —Ruiz-García et al. 2016: 4 [distribution]. —Sipahiler 2017b: 12 [distribution]. —Melnitsky and Ivanov 2017: 19 [distribution]. —O’Connor and O’Connor 2017a: 243 [distribution]. —O’Connor and O’Connor 2017b: 52 [distribution]. —O’Connor and O’Connor 2018: 81 [distribution]. —O’Connor et al. 2018: 23 [distribution]. —Edmonds-Brown 2020: 91 [checklist].

—*flabellifera* (Bremi) in Hagen 1864b: 116 [type locality not given; type not designated; in *Hydroptila*]. —Lauterborn 1934: 220 [specimens re-identified as *A.pallidula* and *Tricholeiochitonfagesii*].

—*pallidula* McLachlan, 1875: 46 [type locality: [Russia]; type not designated]. —McLachlan 1880: 507 [♂; distribution]. —McLachlan 1884: 70 [distribution]. —Morton 1904: 324 [distribution]. —Martynov 1924: 39 [♂]. —Martynov 1927: 176 [distribution]. —Martynov 1934: 118 [♂]. —Mosely 1939b: 255 [♂]. —Tjeder 1940: 10 [distribution]. —Kimmins 1943: 154 [distribution]. —Berg 1948: table 14, between pages 124–125 [distribution]. —Jacquemart 1958: 1 [larva]. —Schmid 1959b: 686 [distribution]. —Nybom 1960: 17 [checklist]. —Spuris 1962: 61 [distribution]. —Neboiss 1963: 619 [to synonymy]. —Botosaneanu 1967: 294 [as synonym]. —Spuris 1972: 27, 28 [checklist].

**Distribution.** —Austria, Belarus, Belgium, Bosnia-Herzegovina, Bulgaria, Czech Republic, Denmark, England, Estonia, Finland, France, Germany, Greece, Hungary, Iran, Ireland, Italy, Kazakhstan, Latvia, Luxembourg, Macedonia, Netherlands, Norway, Poland, Portugal, Slovenia, Romania, Russia, Spain, Sweden, Switzerland, Turkey, Ukraine.

♰ ***spathifera*** (*Agraylea*) Ulmer, 1912a: 39 [type locality: [Baltic region]; holotype missing, originally deposited in “Museum Königsberg” (no. 11883); ♂; in amber]. —Eskov et al. 2008: 78 [checklist]. —Wichard 2013: 37 [species review].

**Distribution.** —Baltic amber.

***taymyrensis*** (*Agraylea*) Mey, 2003a: 39 [type locality: Russia, Northern Siberia, Norilsk, 40 km östlich, Pyany Insel; ZMHB; ♂]. —Ivanov 2011: 195 [checklist].

**Distribution.** —Russia.

#### Genus *Allotrichia* McLachlan, 1880

*Allotrichia* McLachlan, 1880: 508 [type species: *Agrayleapallicornis* Eaton, 1873, monotypic]. —Marshall 1979: 196 [generic review]. —Botosaneanu 1992: 51 [key to species in the Levant; as subgenus of *Agraylea*]. —Kachalova in Medvedev 1998: 182 [key to the species of the European part of the USSR]. —Malicky 2005b: 545 [treated as genus]. —Ivanov 2011: 183 [referred to as distinct genus].

*Allotrichia* consists of 14 species, including four fossil species known from Baltic amber. The genus has a Palaearctic distribution. Marshall (1979b) noted that the genus is morphologically very similar to *Agraylea*, and that *Agraylea* may be a junior subjective synonym of *Allotrichia*. The larvae of *A.pallicornis* were described by Giudicelli and Vaillant (1967).

♰ ***ampullata*** Ulmer, 1912a: 40 [type locality: [Baltic region]; holotype missing, originally deposited in “Museum Königsberg” (no. 14038); ♂; in amber]. —Eskov et al.2008: 78 [checklist]. —Wichard 2013: 45 [species review].

**Distribution.** —Baltic amber.

♰ ***clara*** Wichard, 2013: 46 [type locality: [Baltic region]; ZMHB; ♂; in amber].

**Distribution.** —Baltic amber.

***galaica*** González & Malicky, 1980: 214 [type locality: Spain, Provinz Lugo, 500 m, Fluß Moreira; USCM; ♂]. —Malicky 1983b: 56 [atlas; ♂]. —González et al. 1986: 113 [distribution]. —Malick 2004a: 67 [atlas]. —Malicky 2005b: 545 [checklist]. —Coppa and González 2007: 95 [distribution]. —González and Menéndez 2011: 119 [distribution]. —Martín et al. 2016: 262 [distribution].

**Distribution.** —France, Portugal, Spain.

***heterocera*** Navás, 1917b: 17 [type locality: [Spain], Seo de Urgel (Lérida), a orillas del Segre; depository not designated; ♀]. —Malicky 2005b: 545 [checklist; may be *nomen dubium*].

**Distribution.** —Spain.

***laerma*** Malicky, 1976: 92 [type locality: Greece, Insel Rhodos, Laerma; Collection Malicky; ♂]. —Botosaneanu and Malicky 1978: 341 [checklist]. —Malicky 1983b: 56 [atlas; ♂]. —Malick 2004a: 67, 73 [atlas]. —Malicky 2005b: 545 [checklist]. —Malicky 2005a: 58 [distribution].

**Distribution.** —Greece.

***marinkovicae*** Malicky, 1977: 65 [type locality: Herzegovina, Mostar; NHMW; ♂]. —Malicky 2004a: 67, 73 [atlas]. —Malicky 1983b: 56 [atlas; ♂]. —Malicky 2005b: 545 [checklist]. —Malicky 2005a: 58 [distribution]. —Karaouzas and Malicky 2015: 4 [distribution]. —Stanić-Koštroman et al. 2015: 85 [distribution]. —Oláh 2017: 136 [distribution].

**Distribution.** —Bosnia-Herzegovina, Greece, Serbia.

***militsa*** Malicky, 1992b: 40 [type locality: Greece, Peloponnes, Methoni, 6 km westlich von Militsa; Collection Malicky; ♂]. —Malicky 2004a: 67 [atlas]. —Malicky 2005b: 545 [checklist]. —Malicky 2005a: 58 [distribution].

**Distribution.** —Greece.

***pallicornis*** (Eaton, 1873): 148 [type locality: [Italy], Turin (Chiliani); NHMUK; ♂; in *Agraylea*]. —McLachlan 1880: 509 [revision; ♂; ♀; to *Allotrichia*]. —McLachlan 1884: 70 [distribution]. —Morton 1896: 102 [distribution]. —Morton 1904: 324 [distribution]. —Mosely 1930a: 183 [checklist]. —Racięcka 1936: 98 [distribution]. —Mosely 1939b: 256 [♂]. —Kimmins 1957a: 107 [lectotype designation]. —Schmid 1959b: 685 [distribution]. —Jacquemart 1960: 1 [♂; distribution]. —Giudicelli and Vaillant 1967: 29 [larva]. —Botosaneanu 1967: 294 [distribution]. —Malicky 1974: 122 [checklist]. —Kumanski 1979: 17 [♂; distribution]. —Moretti et al. 1981: 350, 354 [biology; distribution]. —Moretti and Cianficconi 1981: 201 [checklist]. —Moretti et al. 1981: 239 [ecology; distribution]. —Malicky 1983b: 53, 56 [atlas; ♂; ♀]. —Kumanski and Malicky 1984: 199 [distribution]. —Kumanski 1985: 115 [♂]. —González et al. 1986: 113 [distribution]. —Sipahiler and Malicky 1987: 112, 129, 143 [distribution]. —Malicky and Lounaci 1987: 15 [checklist]. —Spuris 1989: 15 [checklist]. —Usseglio-Polatera and Bournaud 1989: 254 [distribution]. —Krušnik 1991: 13 [distribution]. —Duke 1994: 7 [distribution]. —Uherkovich and Nógrádi 1999: 421 [distribution]. —Valle 2001: 68 [distribution]. —Mirmoayedi and Malicky 2002: 164 [checklist]. —Nógrádi and Uherkovich 2002: 130 [distribution]. —Ujvárosi 2002: 384 [distribution]. —Cibaitė 2003a: 10 [checklist]. —Bonada et al. 2004: 52 [distribution]. —Cianficconi et al. 2004b: 330 [distribution]. —Malicky 2004a: 67, 73 [atlas]. —Sipahiler 2005: 396 [distribution]. —Sipahiler 2005: 396 [distribution]. —Bonada et al. 2005: 787 [distribution]. —Malicky 2005b: 545 [checklist]. —Malicky 2005a: 58 [distribution]. —Coppa and Tachet 2005: 132 [distribution]. —Graf et al. 2005: 55 [distribution]. —Lubini-Ferlin and Vicentini 2005: 67 [checklist]. —Schiess-Bühler and Rezbanyai-Reser 2006: 73 [distribution]. —Robert 2007: 82 [checklist]. —Sipahiler 2007: 38 [distribution]. —Cianficconi et al. 2007b: 569 576 [distribution]. —Dohet et al. 2008: 46 [distribution; ecology]. —Szczęsny and Godunko 2008: 14 [checklist]. —Ujvárosi et al. 2008: 113 [checklist]. —Schrankel et al. 2008: 90 [checklist]. —Chvojka and Komzák 2008: 13 [distribution]. —Višinskienė 2009: 27 [checklist]. —Hohmann 2010: 40 [distribution]. —Corallini and Cianficconi 2011: 628 [checklist]. —González and Menéndez 2011: 119 [distribution]. —Waringer and Graf 2011: 282 [larval synopsis]. —Ivanov 2011: 195 [checklist]. —Viidalepp et al. 2011: 196 [distribution]. —Komzák and Chvojka 2012: 718 [distribution]. —Wolf et al. 2012: 75 [distribution]. —Corallini et al. 2013b: 26 [distribution]. —Martín et al. 2014: 72 [distribution]. —Malicky 2014b: 8 [teratological structures]. —Karaouzas and Malicky 2015: 14 [distribution]. —Martínez et al. 2015: 40 [distribution]. —Martín et al. 2015: 75 [distribution]. —Stanić-Koštroman et al. 2015: 85 [distribution]. —O’Connor 2015: 28 75 [distribution]. —Karaouzas and Malicky 2016: 18 [distribution]. —Martínez et al. 2016: 52 [distribution]. —Sekhi et al. 2016: 58 [distribution]. —Ruiz-García et al. 2016: 4 [distribution]. —Valle and Lodovici 2018: 146 [distribution]. —Mabrouki et al. 2020: 11 [distribution].

—*tauri* Jacquemart, 1965: 5 [type locality: [Turkey] 69 km avant Gülek, St. 37; IRSNB; ♂]. —Botosaneanu and Malicky 1978: 341 [to synonymy].

**Distribution.** —Algeria, Austria, Belgium, Bulgaria, Czech Republic, England, Estonia, France, Germany, Greece, Hungary, Iran, Ireland, Italy, Lithuania, Luxembourg, Morocco, Portugal, Romania, Russia, Serbia, Scotland, Slovenia, Spain, Switzerland, Turkey, Ukraine.

***rhynchophyllum*** Zhou, Yang, & Morse, 2016: 206 [type locality: China, Hei-long-jiang Province, Yi-chun City, Wu-yi-ling, Wu-yun River in the Town of Yong-sheng, N47.54°, E128.53°, 160 m; NAUJ; ♂]. —Ito and Shimura 2019: 32 [♂; distribution].

**Distribution.** —China, Japan.

♰ ***succinica*** Ulmer, 1912a: 41 [type locality: [Baltic region]; holotype missing, originally deposited in the “Klebs collection” (no. 14038); ♂; in amber]. —Eskov et al. 2008: 78 [checklist]. —Wichard 2013: 44 [species review; as *succinea*].

**Distribution.** —Baltic amber.

♰ ***superba*** Wichard, 2013: 48 [type locality: [Baltic region]; ZMHB; ♂; in amber].

**Distribution.** —Baltic amber.

***teldanica*** Botosaneanu, 1974: 164 [type locality: [Israel], Tel el Kadi (Tel Dan); TAU; ♂]. —Botosaneanu and Malicky 1978: 341 [checklist]. —Malicky 1983b: 56 [atlas; ♂]. —Botosaneanu 1992: 87 [♂; ♀]. —Sipahiler 2003b: 33 [distribution]. —Malicky 2004a: 67, 73 [atlas]. —Sipahiler 2005: 396 [distribution]. —Malicky 2005b: 545 [checklist]. —Malicky 2005a: 58 [distribution]. —Dia 2015: 51 [distribution]. —Karaouzas and Malicky 2016: 18 [distribution]. —Sipahiler 2018: 41 [distribution].

**Distribution.** —Greece, Israel, Lebanon, Turkey.

***vilnensis orientalis*** Botosaneanu, 1992: 54 [type locality: [Lebanon], Nabaa Joun spring, basin of the Nahr el Aouali (one of the small coastal basins of Lebanon), 50 m]; ZMUA; ♀; ♂]. —Malicky 2005b: 545 [note on subspecies differences].

**Distribution.** —Iran, Lebanon.

***vilnensis vilnensis*** Racięcka, 1937: 477 [type locality: [Lithuania], Wilno; MZVU; ♂; ♀]. —Racięcka 1936: 98 [distribution; not treated as new species]. —Schmid 1959b: 685 [distribution]. —Botosaneanu 1967: 294 [distribution]. —Botosaneanu and Malicky 1978: 341 [checklist]. —Çakin 1983: 246 [distribution]. —Malicky 1983b: 56 [atlas; ♂]. —Sipahiler and Malicky 1987: 122 [distribution]. —Spuris 1989: 15 [checklist]. —Mirmoayedi and Malicky 2002: 164 [checklist]. —Cibaitė 2003a: 10 [checklist]. —Malicky 2004a: 67, 73 [atlas]. —Sipahiler 2005: 396 [distribution]. —Malicky 2005b: 545 [checklist]. —Malicky 2005a: 59 [distribution]. —Višinskienė 2009: 27 [checklist]. —Ivanov 2011: 195 [checklist]. —Karaouzas and Malicky 2015: 19 [distribution]. —Dia 2015: 51 [distribution]. —Pan’kov and Krasheninnikov 2016: 333 [distribution]. —Sipahiler 2016: 12 [distribution].

**Distribution.** —Greece, Iran, Lebanon, Lithuania, Russia, Turkey.

#### Genus *Austratrichia* Wells, 1982

*Austratrichia* Wells, 1982: 259 [type species: *Austratrichianeboissi* Wells, 1982, original designation].

The monotypic genus *Austratrichia* is endemic to Australia. It is most similar to the genus *Hellyethira*. According to Wells (1982), the genus can be distinguished using characters of the male genitalia (Wells 1982). The larval stage is unknown.

***neboissi*** Wells, 1982: 260 [type locality: Victoria, Mitta Mitta River-Snowy Creek junction; NMV; ♂; ♀]. —Neboiss 1986: 68 [atlas; ♂; ♀].

**Distribution.** —Australia.

#### Genus *Cyclopsiella* Kjærandsen, 1997

*Cyclopsiella* Kjærandsen, 1997: 234 [type species: *Cyclopsiellaanderseni* Kjærandsen, 1997, original designation].

The monotypic genus *Cyclopsiella*, recorded only from Ghana, can be distinguished from all other hydroptilid genera by having only a single medial ocellus and lacking postoccipital lobes (Kjærandsen 1997). Kjærandsen (1997) noted that the male genitalia of *Cyclopsiella* share some similarities with the genera *Hydroptila* and *Hellyethira*, yet, in the accompanying parsimony analysis, *Cyclopsiella* grouped with the genera *Jabitrichia*, *Oxyethira*, and *Tangatrichia*. The larval stage is unknown.

***anderseni*** Kjærandsen, 1997: 235 [type locality: Ghana, Western Region, Ankasa Game Production Preserve, station 8; ZMUB; ♂].

**Distribution.** —Ghana.

#### Genus *Dhatrichia* Mosely, 1948

*Dhatrichia* Mosely, 1948b: 78 [type species: *Dhatrichiainasa* Mosely, 1948b, original designation]. —Marshall 1979b: 199 [generic review]. —Kjærandsen 2004: 131 [revision; phylogenetic analysis; species group designation; keys to males, females, fifth instar larvae, and pupae].

The genus *Dhatrichia* consists of 14 species recorded from Burkina Faso, Ghana, Madagascar, Tanzania, Yemen, and Zaire. Marshall (1979b) stated that *Dhatrichia* shares similarities in the male genitalia with *Agraylea* and in the thorax with *Microptila*, Kjærandsen (2004), however, postulated that the genus is actually sister to either *Kumanskiella* or *Microptila*, although *Kumanskiella* is currently placed in the Neotrichiinae. The larvae of *D.ankasaensis*, *D.hunukani*, *D.lerabae*, *D.minuta*, and *D.wliensis* were described by Kjærandsen (2004).

***anderseni*** Kjærandsen, 2004: 168 [type locality: Tanzania, Tanga Region, West Usambara Mountains, Mazumbai, Kaputu stream, loc. 10, 1420 m asl; ZMUB; ♂; ♀].

**Distribution.** —Tanzania.

***ankasaensis*** Kjærandsen, 2004: 148 [type locality: Ghana, Western Region, Ankasa Game Production Reserve, site 4; ZMUB; ♂; ♀, larva, pupa].

**Distribution.** —Ghana.

***bipunctata*** Statzner, 1977: 394 [type locality: Zaire, Kivu Region, Kalengo stream 10 km west of Lake Kivu; ZMHB; ♂; ♀]. —Kjærandsen 2004: 164 [♂, ♀; distribution].

**Distribution.** —Congo, Zaire.

***botiensis*** Kjærandsen, 2004: 168 [type locality: Ghana, Eastern Region, Boti Waterfalls; ZMUB; ♂; ♀].

**Distribution.** —Ghana.

***cinyra*** Wells & Andersen, 1995: 157 [type locality: Tanzania, Tanga region, West Usambara Mts, Mazumbai, Kaputu Stream, loc. 4, 1680 m a.s.l.; ZMUB; ♂]. —Kjærandsen 2004: 158 [♂, ♀; distribution].

**Distribution.** —Tanzania.

***divergenta*** Wells & Andersen, 1995: 156 [type locality: Tanzania, Tanga region, West Usambara Mts, Mazumbai, Kaputu Stream, loc. 7, 1535 m a.s.l.; ZMUB; ♂]. —Kjærandsen 2004: 173 [♂, ♀].

**Distribution.** —Tanzania.

***giboni*** Kjærandsen, 2004: 161 [type locality: Madagascar, Rianila river basin, Analamazaotra Nature Reserve, small brook near Andasibe, 18°54'37"S, 48°25'14"E, 890 m asl; ZMUB; ♂].

**Distribution.** —Madagascar.

***hunukani*** Kjærandsen, 2004: 150 [type locality: Ghana, Volta Region, Wli, Agumatsa Waterfalls, site 5; ZMUB; ♂; ♀, larva, pupa].

**Distribution.** —Ghana.

***inasa*** Mosely, 1948b: 78 [type locality: Yemen, Wadi Dhahr, north-west of San’a, c. 7900 ft; NHMUK; ♂]. —Botosaneanu 1973: 66 [taxonomic note]. —Malicky 1983b: 57 [atlas; ♂]. —Kjærandsen 2004: 165 [♂]. —Kjærandsen 2004: 165 [♂]. —Malicky 2004a: 66 [atlas]. —Malicky 2005b: 547 [checklist].

**Distribution.** —Yemen.

***lerabae*** (Gibon, Guenda, & Coulibaly, 1994): 109 [type locality: sur la haute Léraba (bassin de la Comoé, région de Banfora, Burkina Faso); MNHN; ♂; in *Ugandatrichia*]. —Kjærandsen and Andersen 1997: 244 [distribution]. —Kjærandsen 2004: 145 [♂, ♀, larva, pupa; distribution, to *Dhatrichia*].

**Distribution.** —Burkina Faso, Ghana.

***madagascarensis*** Kjærandsen, 2004: 159 [type locality: Madagascar, Efaho River basin, River Ambahibe near Ezoambo Village, 24°49'10"S, 46°51'59"E, 25 m asl; ZMUB; ♂; ♀].

**Distribution.** —Madagascar.

***minuta*** Kjærandsen, 2004: 167 [type locality: Ghana, Western Region, Ankasa Game Production Reserve, site 4; ZMUB; ♂; ♀, larva, pupa].

**Distribution.** —Ghana.

***paraminuta*** Kjærandsen, 2004: 165 [type locality: Ghana, Volta Region, Wli, Agumatsa Waterfalls, site 1; ZMUB; ♂; ♀].

**Distribution.** —Ghana.

***wliensis*** Kjærandsen, 2004: 152 [type locality: Ghana, Volta Region, Wli, Agumatsa Waterfalls, site 5(C); ZMUB; ♂; ♀, larva, pupa].

**Distribution.** —Ghana.

#### Genus *Hellyethira* Neboiss, 1977

*Hellyethira* Neboiss, 1977: 42 [type species: *Hellyethiravallecula* Neboiss, 1977, original designation]. —Wells 1979b: 312 [revision, key to males]. —Wells 1985b: 10 [key to cased larvae]. —Wells 1991: 494 [key to males of New Guinea]. —Wells 1997: 1 [checklist; key to larvae of Australian species].

The genus *Hellyethira* consists of 44 species, 30 occurring in Australia (one of which is also found in New Caledonia), others in New Guinea and Southeast Asia, and a single species described from Ethiopia. It can be distinguished from the genera *Paroxyethira* and *Orthotrichia* by differences in wing venation (Neboiss 1977). Wells (1985b) stated that the larvae of the genus are indistinguishable from those of *Acritoptila*; she described the larval stages of *H.simplex* and final instar larvae of many others in the genus.

***Hellyethiraagosana*** Mey, 2003b: 433 [type locality: Philippines, Luzon, Quezon province, east of Infanta, Magsaysay; ZMHB, to be transferred to either MPMP or UPLB; ♂]. —Malicky and Chantaramongkol 2007: 1028 [distribution]. —Malicky 2009b: 10 [distribution].

**Distribution.** —Philippines.

***Hellyethiraallynensis*** Wells, 1979b: 316 [type locality: [Australia] New South Wales, Upper Allyn River; ANIC; ♂]. —Wells 1985b: 11 [case]. —Neboiss 1986: 78 [atlas; ♂].

**Distribution.** —Australia.

***Hellyethiraamutiel*** Malicky, Melnitsky, & Ivanov, 2014a: 833 [type locality: [Indonesia] Papua, Insel Biak, Warsa, Wafsarak Wasserfall, 0°47'39"S, 135°55'31"E, 50 m; ZIN; ♂].

**Distribution.** —Indonesia.

***Hellyethirababuyana*** Wells & Mey, 2002: 131 [type locality: [Philippines] Palawan, Cayasan, Babuyan River, LF; ZMHB; ♂]. —Mey and Freitag 2020: 57 [distribution].

**Distribution.** —Philippines.

***Hellyethirabasilobata*** Wells, 1979b: 316 [type locality: [Australia] Victoria, Yarra River, below Upper Yarra Dam; NMV; ♂]. —Wells 1985b: 10 [larva, case]. —Neboiss 1986: 76 [atlas; ♂]. —Neboiss 2002: 53 [checklist]. —Oláh and Johanson 2010a: 8 [distribution].

**Distribution.** —Australia.

***Hellyethirabulat*** Wells & Huisman, 1992: 110 [type locality: East Malaysia, Sabah, Long Pa Sia, Sg. Ritan-Rurun, 1040 m; RMNH; ♂; ♀]. —Malicky and Chantaramongkol 2007: 1028 [distribution]. —Malicky 2007a: 177 [checklist]. —Oláh and Johanson 2010b: 26 [distribution]. —Malicky 2010a: 40 [atlas; ♂]. —Malicky et al. 2018: 1322, 1323 [distribution]. —Melnitsky et al. 2019: 539 [distribution].

**Distribution.** —Brunei, Indonesia, Malaysia, Thailand.

***Hellyethiracornuta*** Wells, 1979b: 325 [type locality: [Australia] Queensland, Little Mulgrave River; ANIC; ♂]. —Wells 1985b: 13 [larva, case]. —Neboiss 1986: 78 [atlas; ♂; ♀]. —Oláh and Johanson 2010a: 10 [distribution].

**Distribution.** —Australia.

***Hellyethiracubitans*** Wells, 1979b: 317 [type locality: [Australia] Queensland, Palmer River; ANIC; ♂; ♀]. —Wells 1985b: 11 [case]. —Neboiss 1986: 76 [atlas; ♂]. —Wells et al. 2019: 33 [detection frequency].

**Distribution.** —Australia.

***Hellyethiradavidi*** Wells, 2005: 388 [type locality: Australia, N Queensland, 11°42.9'S, 142°20.0'E, Gunshot Creek, Telegraph Crossing; QM; ♂].

**Distribution.** —Australia.

***dentata*** Wells, 1979a: 319 [type locality: [Australia] Western Australia, Mitchell Plateau, Camp Creek; WAM; ♂]. —Neboiss 1986: 78 [atlas; ♂].

**Distribution.** —Australia.

***digitata*** Wells, 2005: 387 [type locality: Australia, N Queensland, 11°42.9'S, 142°20.0'E, Gunshot Creek, Telegraph Crossing; QM; ♂].

**Distribution.** —Australia.

***eskensis*** (Mosely, 1934a): 141 [type locality: [Australia] Esk, Queensland; Collection Tillyard (transferred to NHMUK); ♂; in *Xuthotrichia*]. —Mosely and Kimmins 1953: 526 [♂]. —Wells 1979b: 321 [♂, ♀, to Hellyethira]. —Wells 1985b: 12 [larva, case]. —Neboiss 1986: 79 [atlas; ♂; ♀]. —Wells 1991: 495 [distribution].

**Distribution.** —Australia, Papua New Guinea.

***exserta*** Wells, 1979a: 319 [type locality: [Australia] New South Wales, Boonoo Boonoo River; NMV; ♂; ♀]. —Wells 1985b: 11 [larva, case, biology]. —Neboiss 1986: 77 [atlas; ♂; ♀]. —Neboiss 2002: 53 [checklist]. —Oláh and Johanson 2010a: 10 [distribution].

**Distribution.** —Australia.

***fimbriata*** (Mosely, 1934a): 142 [type locality: [Australia] Heathcote, New South Wales; Collection Tillyard (transferred to NHMUK according to Mosely and Kimmins 1953: 525); ♂; in *Xuthotrichia*]. —Mosely and Kimmins 1953: 523 [♂]. —Wells 1979b: 320 [to *Hellyethira*]. —Neboiss 1986: 78 [atlas; ♂].

**Distribution.** —Australia.

***forficata*** Wells, 1990c: 111 [type locality: [Australia] Northern Territory, Kakadu National Park, Radon Springs, 12°45'S 132°55'E; NTM; ♂].

**Distribution.** —Australia.

***haitimlain*** Wells, 1991: 497 [type locality: Papua New Guinea, Central Province, Laloki River at Rouna Falls, 9°25'S 147°27'E; ANIC; ♂; case].

**Distribution.** —Papua New Guinea.

***imparalobata*** Wells, 1990c: 113 [type locality: [Australia] NE Queensland, Yuccabine Creek; NMV; ♂].

**Distribution.** —Australia.

***khukri*** Wells & Dostine, 2016: 596 [type locality: [Australia], Northern Territory, Petherick’s Rainforest Reserve; ANIC; ♂].

**Distribution.** —Australia.

***kukensis*** Wells, 1991: 495 [type locality: Papua New Guinea, East Highlands Province, Ukarumpa, Ba’i River, 6°17'S 145°50'E; ANIC; ♂; ♀; case].

**Distribution.** —Papua New Guinea.

***lacustris*** Mey, 2006b: 203 [type locality: Indonesia, Sulawesi Selatan, Soroako, Lake Matano; LIPI; ♂; ♀]. —Malicky 2013: 42 [possible junior synonym to *H.lititia*].

**Distribution.** —Indonesia.

***litita*** Wells, 1990b: 393 [type locality: [Indonesia] Sulawesi Utara, Dumoga-Bone N.P., Toraut and Tumpah R. junction; NMV; ♂; ♀; case]. —Wells and Huisman 2001: 210 [distribution]. —Malicky et al. 2010: 163 [distribution]. —Malicky 2013: 42 [possible senior synonym to *H.lacustris*].

**Distribution.** —Indonesia.

***litua*** Wells, 1979b: 328 [type locality: [Australia] Western Australia, Jandakota; ANIC; ♂]. —Wells 1985b: 13 [larva, case]. —Neboiss 1986: 77 [atlas; ♂].

**Distribution.** —Australia.

***loripes*** Wells, 1979b: 322 [type locality: [Australia] Western Australia, Mitchell Plateau, Camp Creek; WAM; ♂]. —Neboiss 1986: 76 [atlas; ♂].

**Distribution.** —Australia.

***maai*** Wells, 1991: 498 [type locality: [Indonesia], Irian Jaya (West New Guinea), Waris, 3°30'S 140°55'E; BPBM; ♂].

**Distribution.** —Indonesia.

***marioch*** Malicky & Graf, 2015: 31 [type locality: Ethiopia, Kleiner Waldbach N von Addis Abeba, 9°05'N, 38°43'E, 2800 m; Collection Malicky; ♂].

**Distribution.** —Ethiopia.

***malleoforma*** Wells, 1979b: 325 [type locality: [Australia] South Australia, Uraidla, farm dam; ANIC; ♂; ♀]. —Wells 1985b: 13 [larva, case, biology]. —Neboiss 1986: 77 [atlas; ♂; ♀]. —Wells 1995: 232 [distribution]. —Neboiss 2002: 54 [checklist]. —Oláh and Johanson 2010a: 11 [distribution]. —Wells and Johanson 2015: 85 [distribution]. —Johanson and Wells 2019: 93 [checklist].

**Distribution.** —Australia, New Caledonia.

***multilobata*** Wells, 1979b: 326 [type locality: [Australia] Victoria, Lake Purrumbete; ANIC; ♂; ♀]. —Neboiss 1986: 77 [atlas; ♂; ♀]. —Neboiss 2002: 53 [checklist].

**Distribution.** —Australia.

***narakain*** Wells, 1991: 497 [type locality: Papua New Guinea, Central Province, Iomari Creek on Bereina-Port Morseby road, 9°04'S 147°06'E; ANIC; ♂; ♀; case].

**Distribution.** —Papua New Guinea.

***naumanni*** Wells, 1990c: 113 [type locality: [Australia] Western Australia, Charnley River, 2 km SW Roly Hill, CALM Site 25/2; NMV; ♂].

**Distribution.** —Australia.

***piala*** Wells & Huisman, 1992: 109 [type locality: Brunei, 45 km on Labir road, Sg. Madoram, 50 m; RMNH; ♂]. —Malicky and Chantaramongkol 2007: 1028 [distribution]. —Malicky 2010a: 40 [atlas; ♂].

**Distribution.** —Brunei, Malaysia.

***pulvina*** Wells, 1979b: 324 [type locality: [Australia] Western Australia, Mitchell Plateau, Camp Creek; WAM; ♂]. —Neboiss 1986: 76 [atlas; ♂]. —Wells et al. 2019: 33 [detection frequency].

**Distribution.** —Australia.

***quadrata*** Wells, 1990c: 115 [type locality: [Australia] NE Queensland, Yuccabine Creek; NMV; ♂].

**Distribution.** —Australia.

***radonensis*** Wells, 1990c: 113 [type locality: [Australia] Northern Territory, Kakadu National Park, Radon Springs, 12°45'S 132°55'E; NTM; ♂].

**Distribution.** —Australia.

***ramosa*** Wells, 1983: 632 [type locality: Australia, Northern Territory, Goanna Lagoon, 1 km W. of Jabiru off Arnhem Highway; NMV; ♂]. —Wells 1985b: 12 [larva; case]. —Neboiss 1986: 78 [atlas; ♂; ♀]. —Wells et al. 2019: 33 [detection frequency].

**Distribution.** —Australia.

***rovid*** Oláh & Johanson, 2010a: 11 [type locality: Malaysia, Sabah, Tawau, Maliau Basin, Nepenthes Camp, crossing stream, 4°43'58.9"N 116°52'40.7"E, 994 m; NHRS; ♂].

**Distribution.** —Malaysia.

***sarina*** Oláh, 2012: 48 [type locality: Indonesia, Papua, Raja Empat Archipelago, Batanta Island, Sarinam River, 0°50'04.24"S 130°47'59.22"E; Collection Oláh; ♂]. —Oláh 2016: 109 [distribution]. —Oláh and Kovács 2018: 178 [distribution].

**Distribution.** —Indonesia.

***selaput*** Wells & Huisman, 1992: 109 [type locality: Brunei, Sg. Temburong, 140 m; RMNH; ♂]. —Malicky 2010a: 40 [atlas; ♂].

**Distribution.** —Brunei.

***sentisa*** Wells, 1979b: 322 [type locality: [Australia] Western Australia, Millstream H.S., 21°35'S 117°04'E; ANIC; ♂]. —Neboiss 1986: 79 [atlas; ♂].

**Distribution.** —Australia.

***sheldoni*** Wells, 2005: 388 [type locality: Australia, N Queensland, 18°57'S 146°10'E, Mt Spec State Forest, Camp Creek tributary. 760 m; NMV; ♂].

**Distribution.** —Australia.

***simplex*** (Mosely, 1934a): 145 [type locality: [Australia] Warwick, Queensland; Collection Tillyard (since transferred to NHMUK according to Neboiss 2002: 53); ♂; in *Xuthotrichia*]. —Mosely and Kimmins 1953: 521 [♂]. —Wells 1979b: 315 [♂, ♀, to *Hellyethira*]. —Wells 1985b: 10 [larva, case]. —Neboiss 1986: 76 [atlas; ♂; ♀]. —Neboiss 2002: 53 [checklist]. —Oláh and Johanson 2010a: 13 [distribution].

—*vallecula* Neboiss, 1977: 42 [type locality: [Australia] Hellyer River Gorge, Tasmania; NMV; ♂; ♀]. —Wells 1979b: 315 [to synonymy].

—*hiana* Oláh & Johanson, 2010a: 10 [type locality: Australia, Queensland, Brisbane Forest Park, Northbrook Creek, downstream 3^rd^ bridge on Northbrook Parkway from Cedar Flats, 27°18.203'S, 152°41.380'E, 174 m; ANIC; ♂]. —Wells 2012: 66 [to synonymy].

**Distribution.** —Australia.

***spinosa*** Wells, 1990c: 115 [type locality: [Australia] NE Queensland, Yuccabine Creek; NMV; ♂].

**Distribution.** —Australia.

***tros*** Malicky & Chantaramongkol, 2007: 1027 [type locality: Thailand, Kao Soi Dao NP, 13°06'N 102°12'E, 300 m; Collection Malicky; ♂]. —Melnitsky and Malicky 2008: 25 [distribution]. —Malicky 2010a: 40 [atlas; ♂].

**Distribution.** —Thailand.

***vernoni*** Wells, 1983: 632 [type locality: Australia, Queensland, Crystal Creek, nr turnoff to Mt Spec; NMV; ♂]. —Wells 1985b: 12 [larva; case]. —Neboiss 1986: 79 [atlas; ♂; ♀]. —Wells et al. 2019: 33 [detection frequency].

**Distribution.** —Australia.

***veruta*** Wells, 1985a: 97 [type locality: Australia, Northern Territory, Magela Creek, S. of Georgetown Billabong; NTM; ♂; ♀]. —Neboiss 1986: 79 [atlas; ♂; ♀]. —Wells et al. 2019: 33 [detection frequency].

**Distribution.** —Australia.

#### Genus *Hydroptila* Dalman, 1819

*Hydroptila* Dalman, 1819: 125 [type species: *Hydroptilatineoides* Dalman, 1819, monotypic]. —Kolenati 1848: 104 [revision]. —McLachlan 1880: 5010 [revision]. —Mosely 1939b: 257 [key to the British species]. —Ross 1944: 141, 142 [diagnosis of larvae; species key for adults]. —Bueno-Soria 1984: 83 [revision of Mexican and Central American species]. —Wells 1978: 746 [key to adults of Australian species]. —Kumanski 1979: 7 [key to species of Bulgaria]. —Marshall 1979b: 200 [generic review]. —Blickle 1979: 11 [key to species of America north of Mexico]. —Lewis and Fairchild 1983: 134 [phoretic association observed]. —Wells 1984: 263 [key to males from New Guinea and New Britain]. —Wells 1985b: 3 [larva; pupa; case; key to cased Australian larvae]. —Wells 1990b: 379 [key to adults of North Sulawesi species]. —Flint 1991b: 46 [key to Antioquian species]. —Wells 1991: 491 [key to males of New Guinea]. —Botosaneanu 1992: 59 [key to species in the Levant]. —Moulton and Stewart 1996: 92 [key to species of the Interior Highlands of North America]. —Wells 1997: 1 [checklist; key to species of larvae]. —Kachalova in Medvedev 1998: 185 [key to the species of the European part of the USSR]. —Harris and Holzenthal 1999: 16 [key to Central American species]. —Zhou et al. 2009a: 909 [key to Chinese species].

*Phrixocoma* Eaton, 1873: 132 [type species: *Hydroptilasparsa* Curtis, 1834, original designation]. —McLachlan 1880: 511 [to synonymy].

*Hydropneuma* Enderlein, 1929: 232 [type species: *Hydropneumajuba* Enderlein, 1929, original designation]. —Kimmins 1957a: 107 [transferred to *Hydroptila*].

*Hydroptilina* Martynov, 1934: 117 [type species: *Hydroptilinaangustipennis* Martynov, 1934, monotypic]. —Fischer 1971: 289 [to synonymy, following Lepneva, 1953: 406]. —Marshall 1979b: 200 [considered as a synonym of *Hydroptila*].

*Oxydroptila* Martynov, 1935: 114 [type species: *Oxydroptilafurcata* Martynov, 1935, original designation]. —Marshall 1979b: 200 [to synonymy].

*Oeceotrichia* Ulmer, 1951: 85 [type species: *Oeceotrichiaelongata* Ulmer, 1951, original designation]. —Marshall 1979b: 200 [to synonymy].

*Pasirotrichia* Ulmer, 1951: 90 [type species: *Pasirotrichiacrenata* Ulmer, 1951, original designation]. —Marshall 1979b: 200 [to synonymy].

*Sumatranotrichia* Ulmer, 1951: 87 [type species: *Sumatranotrichiatrullata* Ulmer, 1951, original designation]. —Marshall 1979b: 200 [to synonymy].

*Hydroptila* is a large, cosmopolitan genus occurring in all regions excluding polar regions. It is the most species-rich genus in the family, consisting of 495 extant species and one fossil species. Marshall (1979b) divided *Hydroptila* into thirteen species groups (*capensis*, *consimilis*, *dikirilagoda*, *forcipata*, *losida*, *occulta*, *pulchricornis*, *sparsa*, *tigurina*, *tineoides*, *uncinata*, *vectis*, *waubesiana*) which she thought might one day be recognized as subgenera, based on distribution and form of the male and female genitalia. Despite the large number of species and the proposed species groups, she also listed several characters that unite the genus, including basic structure of the genitalia, thorax, absence of ocelli, presence of dorsal postoccipital scent-organs in male adults, and the general appearance of the immature stage. The larvae of *H.delineata* were described by Sibley (1926), and larvae of many other species have been described since (Lepneva 1932, 1964; Ross 1944; Nielsen 1948; Hanna 1961; Jacquemart and Coineau 1962; Botosaneanu and Sykora 1963; Flint 1964; Jacquemart 1965; Hicken 1967; Fahy 1971; Ito and Kawamura 1980; Botosaneanu and Giudicelli 1981; Wells 1985b, 1997; Keiper and Foote 1999).

***abantica*** Sipahiler, 1996: 30 [type locality: [Turkey, Bolu, Abant, 1400 m. (from the spring); ZSM; ♂; ♀]. —Malicky 2004a: 55 [atlas]. —Malicky 2005b: 543 [checklist]. —Sipahiler 2005: 396 [distribution]. —Sipahiler 2007: 38 [distribution]. —Sipahiler 2008: 104 [checklist].

**Distribution.** —Turkey.

***abbotti*** Moulton & Harris, 1997: 494 [type locality: United States, Texas, Anderson Co., Skeet Branch, Engeling Wildlife Management Area, 3.2 km W Blackfoot; NMNH; ♂]. —Abbott et al. 1997: 44 [distribution].

**Distribution.** —U.S.A.

***acadia*** Ross, 1941a: 63 [type locality: [Canada], Nova Scotia, Hubbard; INHS; ♂; ♀]. —Blickle 1979: 47, 69 [checklist; ♂]. —Harris et al. 2012: 5 [♂; checklist]. —Denson et al. 2016: 5 [distribution].

**Distribution.** —Canada, U.S.A.

***acantha*** Wells & Mey, 2002: 128 [type locality: [Philippines] Panay, San Reminigio, Aningalan; ZMHB; ♂].

**Distribution.** —Philippines.

***acinacis*** Wells, 1978: 755 [type locality: [Australia] Victoria, Koornalla, Traralgon Creek, La Trobe River Environmental Survey, Site 24a; NMV; ♂; ♀]. —Wells 1985b: 6 [larva]. —Neboiss 1986: 63 [atlas; ♂; ♀]. —Neboiss 2002: 52 [checklist].

**Distribution.** —Australia.

***acuminata*** Bueno-Soria, 1984: 88 [type locality: Mexico, Tamaulipas, 40 km S Ciudad Victoria, Río Purificación; CNIN; ♂]. —Moulton and Stewart 1997: 350 [checklist]. —Bowles et al. 2007: 21 [distribution; biology].

**Distribution.** —Mexico, U.S.A.

***acuta*** Mosely, 1930a: 177 [type locality: [France], Corsica, Corte; NHMUK; ♂; ♀]. —Jacquemart and Coineau 1962: 50 [♂; larva]. —Botosaneanu 1967: 294 [distribution]. —Botosaneanu and Malicky 1978: 340 [checklist]. —Moretti and Cianficconi 1981: 201 [checklist]. —Malicky 1983b: 46 [atlas; ♂]. —González et al. 1990: 214 [distribution]. —Malicky 1997: 139 [distribution; ♂]. —Valle 2001: 65 [distribution]. —Malicky 2002: 4 [distribution]. —Coppa and Tachet 2004: 124 [♀]. —Malicky 2004a: 57 [atlas]. —Malicky 2005b: 543 [checklist]. —González and Menéndez 2011: 118 [distribution]. —Corallini et al. 2013a: 38 [checklist]. —Cianficconi et al. 2016: 136 [distribution]. —Ruiz-García et al. 2016: 3 [distribution].

**Distribution.** —France, Italy, Spain.

***acutangulata*** Yang & Wang in Yang, Wang, and Leng 1997: 285 [type locality: [China], Longyuwan forest farm, 1000 m, Luanchuan County, Henan Prov.; NAUJ; ♂].

**Distribution.** —China.

***adana*** Mosely, 1948b: 81 [type locality: [Yemen], Western Aden Protectorate, Jebel Harir, c. 5000 ft; NHMUK; ♂]. —Botosaneanu and Gasith 1971: 99 [distribution]. —Botosaneanu, 1973: 66 [♂]. —Botosaneanu 1982b: 11 [habitat threat]. —Malicky 1983b: 50, 52 [atlas; ♂; ♀]. —Botosaneanu 1992: 63 [wings; head; ♂; ♀]. —Malicky 1999a: 345 [distribution]. —Mirmoayedi and Malicky 2002: 164 [checklist]. —Malicky 2004a: 62 [atlas]. —Malicky 2005b: 543 [checklist]. —Chvojka 2006: 253 [distribution]. —Malicky 2014b: 17 [teratological structures].

**Distribution.** —Iran, Israel, Yemen.

***aegyptia*** Ulmer, 1963: 267 [type locality: [Egypt], Maadi, Nilufer; ZMUH; ♂]. —Botosaneanu and Malicky 1978: 340 [checklist]. —Moretti et al. 1978: 28 [larva; ecology]. —Moretti and Bicchierai 1979: 173 [androconial structure]. —Moretti and Cianficconi 1981: 201 [checklist]. —Moretti et al. 1981: 350, 354 [biology; distribution]. —Malicky 1983b: 43 [atlas; ♂]. —Kumanski and Malicky 1984: 199 [distribution]. —Kumanski 1985: 135 [♂]. —Moubayed and Botosaneanu 1985: 63 [distribution]. —Spuris 1989: 15 [checklist]. —Malicky and Lounaci 1987: 15, 17 [checklist]. —Botosaneanu 1992: 81 [♂; ♀]. —Bicchierai and Moretti 1994: 108, 111 [palps]. —Dallai and Afzelius 1995: 166 [sperm structure]. —Cianficconi et al. 1999a: 57 [distribution]. —Valle 2001: 65 [distribution]. —Cianficconi et al. 2002: 146 [distribution]. —Spinelli and Corallini 2002: 32 [leg morphology]. —Sipahiler 2003b: 33 [distribution]. —Malicky 2004a: 52 [atlas]. —Cianficconi et al. 2004a: 256, 257, 258 [distribution; case; biology]. —Malicky 2005b: 543 [checklist]. —Malicky 2005a: 59 [distribution]. —Sipahiler 2005: 396 [distribution]. —Chvojka 2006: 253 [distribution]. —Cianficconi et al. 2007b: 569, 575 [distribution]. —Corallini 2007: 76 [absence of goblet cells]. —Szczęsny and Godunko 2008: 14 [checklist]. —Ujvárosi et al. 2008: 112 [checklist]. —Oláh 2010: 91 [distribution]. —Cianficconi et al. 2011: 47 [distribution]. —Corallini and Cianficconi 2011: 628 [checklist]. —Corallini et al. 2013a: 38 [checklist]. —Karaouzas and Malicky 2015: 14 [distribution]. —Dia 2015: 51 [distribution]. —Cianficconi et al. 2016: 136 [distribution]. —Corallini and Bicchierai 2016: 151 [biology]. —Sipahiler 2018: 41 [distribution].

—*kurnas* Malicky, 1974: 109 [type locality: [Greece], Kreta, Kournas-See]. —Botosaneanu and Malicky 1978: 340 [to synonymy].

**Distribution.** —Bulgaria, Egypt, Greece, Iran, Italy, Lebanon, Tunisia, Romania, Russia, Turkey, Ukraine.

***africana*** Kimmins, 1958a: 364 [type locality: [Zimbabwe], S. Rhodesia, Victoria Falls; NHMUK; ♂].

**Distribution.** —Zimbabwe.

***agosensis*** Mey, 2003b: 431 [type locality: Philippines, Luzon, Quezon province, east of Infanta, Magsaysay; ZMHB, to be transferred to either MPMP or UPLB; ♂].

**Distribution.** —Philippines.

***ajax*** Ross, 1938a: 127 [type locality: United States, Illinois, Oakwood, along Salt Fork River; INHS; ♂]. —Ross 1944: 153 [♂; ♀; distribution]. —Denning 1947b: 174 [distribution]. —Etnier 1965: 146 [checklist]. —Blickle 1979: 47, 71 [checklist; ♂]. —Parker and Voshell 1981: 4 [checklist]. —Huryn and Foote 1983: 790 [distribution]. —Waltz and McCafferty 1983a: 9 [distribution]. —Hamilton et al. 1983: 18 [distribution]. —Bueno-Soria 1984: 109 [♂; distribution]. —Tarter 1990: 239 [checklist]. —Masteller and Flint 1992: 69 [checklist]. —Mathis and Bowles 1992: 24 [distribution]. —Bowles and Mathis 1992: 32 [distribution]. —Moulton and Stewart 1996: 95 [♂; distribution]. —Moulton and Stewart 1997: 350 [checklist]. —Abbott et al. 1997: 44 [distribution]. —Ruiter 1999: 165 [distribution]. —Houghton et al. 2001: 504 [distribution]. —Blinn and Ruiter 2005: 68 [distribution; biology]. —Blinn and Ruiter 2006: 332 [biology; distribution]. —Zeullig et al. 2006: 42 [distribution]. —Zack et al. 2006: 134 [phenology; distribution]. —Bowles et al. 2007: 21 [distribution; biology]. —Chamorro-Lacayo et al. 2007: 42 [checklist]. —Blinn and Ruiter 2009a: 304 [biology] —Blinn and Ruiter 2009b: 186 [phenology; distribution]. —Houghton et al. 2011a: 388 [distribution; biology]. —Armitage et al. 2011: 13 [checklist]. —Blinn and Ruiter 2013: 291 [biology; distribution]. —Harris et al. 2012: 5 [♂; distribution]. —de Walt et al. 2016: 51 [distribution]. —Houghton et al. 2017: 62 [checklist]. —Mendez et al. 2019: 118 [checklist].

**Distribution.** —Mexico, Nicaragua, U.S.A.

***alabama*** Harris & Kelley, 1984a: 572 [type locality: [United States], Alabama, Escambia County, Little Escambia Creek at Hwy. 31; NMNH; ♂]. —Harris et al. 1984: 108 [distribution]. —Harris et al. 1991: 166 [distribution]. —Frazer et al. 1991: 19 [distribution]. —Masteller and Flint 1992: 69 [checklist]. —Abbott et al. 1997: 44 [distribution]. —Moulton and Stewart 1997: 350 [checklist]. —Huryn and Harris 2000: 193 [distribution]. —DeWalt and Heinold 2005: 41 [phenology; distribution]. —Myers et al. 2011: 105 [distribution]. —Harris et al. 2012: 5 [distribution]. —Denson et al. 2016: 5 [distribution].

**Distribution.** —U.S.A.

***alai*** Johanson, Wells, Malm, & Espeland, 2011: 291 [type locality: [Vanuatu] Espiritu Santo, Central Santo, stream in small canyon crossing path to village, 5.5 km NW Nambel, 208 m, loc#21, 15°27.459'S, 167°04.022'E; NHRS; ♂].

**Distribution.** —Vanuatu.

***alara*** Sipahiler, 1994: 12 [type locality: Turkey, Antalya, Gündogmus, Güneycik Köyü, Alara çayi, Alibey köprüsü, 31°48'E, 36°46'N, 180 m; depository not designated; ♂]. —Malicky 2004a: 63 [atlas]. —Malicky 2005b: 543 [checklist] —Sipahiler 2005: 396 [distribution].

**Distribution.** —Turkey.

***albicornis*** Hagen, 1861: 275 [type locality: Canada (Osten Sacken), St. Lawrence River; MCZ; ♂]. —Eaton 1873: 138 [distribution; as *Phrixocoma*]. —Banks 1907a: 50 [catalogue]. —Betten 1934: 157 [♂; distribution]. —Ross 1938b: 9 [lectotype designated; ♂]. —Ross 1944: 151 [♂; ♀; larva; distribution]. —Denning 1947b: 174 [distribution]. —Etnier 1965: 146 [checklist]. —Unzicker et al. 1970: 172 [distribution]. —Etnier and Schuster 1979: 17 [distribution]. —Roy and Harper 1979: 150 [checklist]. —Blickle 1979: 47, 69 [checklist; ♂]. —Huryn and Foote 1983: 790 [distribution]. —Waltz and McCafferty 1983a: 9 [distribution]. —Bowles and Mathis 1989: 238 [distribution]. —Bowles and Mathis 1992: 32 [distribution]. —Mathis and Bowles 1992: 24 [distribution]. —Moulton and Stewart 1996: 95 [♂; distribution]. —Houghton et al. 2001: 504 [distribution]. —Myers et al. 2011: 105 [checklist]. —Armitage et al. 2011: 14 [checklist]. —DeWalt et al. 2016: 5 [distribution]. —Houghton et al. 2017: 62 [checklist]. —Bowles et al. 2020: 7 [distribution].

**Distribution.** —Canada, U.S.A.

***aldricki*** Bueno-Soria, 1984: 108 [type locality: Mexico, Guerrero, Cocula; NHMUK; ♂].

**Distribution.** —Mexico.

***amoena*** Ross, 1938a: 124 [type locality: [United States], Illinois, Herod; INHS; ♂]. —Ross 1944: 150 [♂; ♀; distribution]. —Denning 1947b: 173 [distribution]. —Morse and Blickle 1953: 72 [distribution]. —Etnier 1965: 146 [distribution]. —Unzicker et al. 1970: 172 [distribution]. —Roy and Harper 1975: 1082 [distribution]. —Etnier and Schuster 1979: 17 [distribution]. —Roy and Harper 1979: 150 [distribution]. —Etnier and Schuster 1979: 17 [checklist]. —Blickle 1979: 47 63 [checklist; ♂]. —Parker and Voshell 1981: 4 [checklist]. —Huryn and Foote 1983: 790 [distribution]. —Harris et al. 1984: 108 [distribution]. —Bowles and Mathis 1989: 238 [distribution]. —Harris et al. 1991: 167 [distribution]. —Masteller and Flint 1992: 69 [distribution]. —Bowles and Mathis 1992: 32 [distribution]. —Floyd and Morse 1993: 176 [distribution]. —Moulton and Stewart 1996: 96 [♂; distribution]. —Houghton et al. 2001: 504 [distribution]. —Etnier 2010: 485 [distribution]. —Armitage et al. 2011: 14, 32 [checklist; ♂]. —Houghton et al. 2011b: 5 [phenology; habitat; distribution]. —Houghton 2016: 46 [biology]. —Houghton et al. 2017: 62 [checklist].

**Distribution.** —Canada, U.S.A.

***ampoda*** Ross, 1941b: 16 [type locality: [Canada], Moser River, Nova Scotia, Gold Mine Brook; INHS; ♂; ♀]. —Etnier 1968: 191 [distribution]. —Roy and Harper 1975: 1082 [distribution]. —Roy and Harper 1979: 150 [checklist]. —Blickle 1979: 47, 63 [checklist; ♂]. —Roy and Harper 1981: 105 [distribution]. —Waltz and McCafferty 1983a: 9 [distribution]. —Masteller and Flint 1992: 69 [checklist]. —Houghton et al. 2001: 504 [distribution]. —Flint 2014: 90 [distribution]. —Myers et al. 2011: 105 [distribution]. —Houghton et al. 2017: 62 [checklist].

**Distribution.** —Canada, U.S.A.

***ancistrion*** Flint, 1968b: 48 [type locality: Jamaica, Portland, Rio Grande, at Fellowship; NMNH; ♂; ♀]. —Flint 1968a: 82 [checklist]. —Botosaneanu and Hyslop 1998: 15 [distribution]. —Botosaneanu 2002b: 83 [checklist].

**Distribution.** —Jamaica.

***andalusiaca*** González & Cobo, 1994: 253 [type locality: Spain, Cadiz, Puente de la Terrona, river Guadalete, 360 m; DZUSC; ♂]. —Malicky 1997: 140 [distribution; ♂]. —Malicky 2004a: 58 [atlas]. —Malicky 2005b: 543 [checklist]. —González and Menéndez 2011: 118 [distribution]. —Martín et al. 2015: 74 [distribution].

**Distribution.** —Spain.

***angulata*** Mosely, 1922: 179 [type locality: [England], “Britain”; NHMUK; ♂]. —Mosely 1923: 292 [scent organ]. —Martynov 1934: 130 [♂]. —Mosely 1939b: 262 [♂]. —Schmid 1952: 650 [distribution]. —Nybom 1960: 18 [checklist]. —Schmid 1960: 98 [distribution]. —Botosaneanu 1967: 294 [distribution]. —Malicky 1974: 122 [checklist]. —Botosaneanu and Malicky 1978: 340 [checklist]. —Moretti and Cianficconi 1981: 201 [checklist]. —Malicky 1983b: 47, 52 [atlas; ♂; ♀]. —Kumanski 1985: 123 [♂]. —Andersen and Wiberg-Larsen 1987: 168 [checklist]. —Malicky and Lounaci 1987: 15, 17 [checklist]. —Rojas-Camousseight and Tachet 1988: 313–314 [♀]. —Usseglio-Polatera and Bournaud 1989: 25 [distribution]. —Spuris 1989: 15 [checklist]. —González et al. 1990: 214 [distribution]. —Oláh 1994: 282 [distribution]. —Malicky 1997: 140 [distribution; ♂]. —Malicky 1999c: 96 [distribution]. —Cianficconi et al. 1999b: 278 [distribution]. —Morse et al. 2001: 102 [distribution]. —Mirmoayedi and Malicky 2002: 164 [checklist]. —Gullefors 2002: 138 [checklist]. —Gullefors 2003: 194, 195 [distribution]. —Cibaitė 2003a: 10 [checklist]. — Cibaitė 2003b: 8 [distribution]. —Urbanič 2004: 51 [distribution]. —Malicky 2004a: 57 [atlas]. —Malicky 2005b: 543 [checklist]. —Coppa and Tachet 2005: 132 [distribution]. —Yang et al. 2005: 458 [checklist]. —Graf et al. 2005: 55 [distribution]. —Gullefors 2005a: 118 [distribution]. —Gullefors 2005b: 138 [distribution]. —Komzák and Chvojka 2005: 65 [distribution]. —Malicky 2005a: 59 [distribution]. —Hohmann 2005: 106 [checklist]. —Lubini-Ferlin and Vicentini 2005: 67 [checklist]. —Gullefors 2006: 136 [distribution]. —Morse et al. 2006: 309, 321 [♂; ♀; distribution]. —Wiggers et al. 2006: 54 [checklist]. —Robert 2007: 82 [checklist]. —Ivanov and Melnitsky 2007: 32 [distribution]. —Cianficconi et al. 2007b: 569, 575 [distribution]. —Chvojka and Komzák 2008: 13 [distribution]. —González and Menéndez 2008: 188 [distribution]. —Schrankel et al. 2008: 90 [checklist]. —Višinskienė 2009: 27 [checklist]. —Neu 2010: 149 [♀]. —Ivanov 2011: 195 [checklist]. —Corallini and Cianficconi 2011: 628 [checklist]. —Cianficconi et al. 2011: 47 [distribution]. —González and Menéndez 2011: 118 [distribution]. —Lukáš and Chvojka 2011: 116 [distribution]. —Viidalepp et al. 2011: 194, 196 [distribution]. —Lock and Goethals 2012: 28 [checklist]. —Komzák and Chvojka 2012: 719 [distribution]. —Corallini et al. 2013a: 38 [checklist]. —O’Connor 2013: 63 [distribution]. —O’Connor and O’Connor 2014: 273 [distribution]. —O’Connor and Bond 2014: 24 [distribution]. —O’Connor 2015: 28, 76 [distribution]. —Stanić-Koštroman et al. 2015: 85 [distribution]. —Stanić-Kroštroman et al. 2015: 85 [distribution]. —Cianficconi et al. 2016: 137 [distribution]. —Chuluunbat et al. 2016: 102[distribution]. —Pan’kov and Krasheninnikov 2016: 333 [distribution]. —Yang et al. 2016: 476 [checklist]. —O’Connor and O’Connor 2016: 165 [distribution]. —Ruiz-García et al. 2016: 4 [distribution]. —Gullefors 2016: 155 [checklist]. —Corallini and Bicchierai 2016: 151 [biology]. —Küttner et al. 2016: 178 [distribution]. —Wallace 2016: 15, 18, 24 [conservation status]. —O’Connor and O’Connor 2017b: 52 [distribution]. —Lock and van Butsel 2017: 33 [distribution; ♂; ♀]. —O’Connor and O’Connor 2018: 81 [distribution]. —Valle and Lodovici 2018: 146 [distribution]. —O’Connor et al. 2018: 23 [distribution]. —Kroča and Komzák 2020: 146 [distribution]. —Park and Kong 2020: 297 [checklist]. —Navara et al. 2020: 46 [distribution]. —Smirnova et al. 2020: 68 [distribution].

—*emarginata* Martynov, 1927: 175 [type locality: [Uzbekistan], Tashkent; depository not designated; ♂]. —Martynov 1934: 133 [♂]. —Schmid 1959b: 692 [distribution]. —Botosaneanu 1967: 294 [distribution]. —Botosaneanu 1970: 289 [distribution; ♀]. —Botosaneanu and Malicky 1978: 341 [checklist]. —Mey 1981: 56 [distribution]. —Spuris 1989: 15 [checklist]. —Kumanski 1990: 46 [distribution]. —Yang et al. 1997b: 93 [checklist]. —Malicky 1997: 140 [to synonymy]. —Malicky 2005b: 543 [to synonymy]. —Huang et al. 2005: 469 [distribution]. —Park and Kong 2020: 297 [checklist].

—*bajgirana* Botosaneanu, 1983: 139 [type locality: Iran Bajgiran Ostan 9 5500 ft au col du Karaul Dagh petite source dans un ravin sec; CNC; ♂]. —Kumanski 1990: 46 [as synonym of *H.emarginata*]. —Xue and Yang 1991: 21 [distribution]. —Xue et al. 1992: 353–356 [distribution]. —Malicky 1997: 140 [to synonymy]. —Malicky 2005b: 543 [to synonymy]. —Lonsdale 2020: 32 [holotype depository].

**Distribution.** —Albania, Austria, Belgium, Bosnia-Herzegovina, China, Czech Republic, England, Estonia, Finland, France, Germany, Greece, Iran, Ireland, Italy, Kazakhstan, Korea, Lithuania, Luxembourg, Mongolia, Netherlands, Pakistan, Portugal, Serbia, Slovakia, Slovenia, Russia, Slovakia, Spain, Sweden, Uzbekistan.

***angulifera*** Kumanski, 1974: 71 [type locality: [Bulgaria], Le Rhodope, la rivière Trigradskä, juste avant sa confluence avec la rivière Tchairska, 800 m; SOFM; ♂; ♀]. —Botosaneanu and Malicky 1978: 340 [checklist]. —Kumanski 1979: 14 [♂; distribution]. —Malicky and Moretti 1987: 194 [♂]. —Kumanski 1993: 39 [distribution]. —Malicky 2004a: 53 [atlas]. —Malicky 2005b: 543 [checklist]. —Oláh 2017: 136 [distribution].

**Distribution.** —Bulgaria.

***angusta*** Ross, 1938a: 130 [type locality: United States, Illinois, Muncie, along Stony Creek; INHS; ♂]. —Ross 1944: 152 [♂; ♀]. —Edwards 1973: 506 [distribution]. —Hamilton et al. 1975: 1003 [biology]. —Bueno-Soria and Flint 1978: 201 [distribution]. —Resh et al. 1978: 383 [distribution]. —Blickle 1979: 47 73 [checklist; ♂]. —Huryn and Foote 1983: 790 [distribution]. —Waltz and McCafferty 1983a: 9 [distribution]. —Hamilton et al. 1983: 18 [distribution]. —Harris et al. 1984: 108 [distribution]. —Bueno-Soria 1984: 122 [♂; distribution]. —Ruiter 1990: 91 [distribution]. —Harris et al. 1991: 168 [distribution]. —Masteller and Flint 1992: 69 [checklist]. —Mathis and Bowles 1992: 24 [distribution]. —Bowles and Mathis 1992: 32 [distribution]. —Moulton et al. 1993: 21 [distribution]. —Moulton and Stewart 1996: 96 [♂; distribution]. —Abbott et al. 1997: 44 [distribution]. —Moulton and Stewart 1997: 350 [checklist]. —Houghton and Stewart 1998: 105 [biology; distribution]. —Ruiter 1999: 165 [distribution]. —Houghton et al. 2001: 504 [distribution]. —Baumgardner and Bowles 2005: 11 [distribution]. —Zeullig et al. 2006: 42 [distribution]. —Bowles et al. 2007: 21 [distribution; biology]. —Armitage et al. 2011: 14 [checklist]. —de Walt et al. 2016: 51 [distribution]. —Houghton et al. 2017: 62 [checklist]. —Mendez et al. 2019: 128 [checklist].

**Distribution.** —Mexico, U.S.A.

***angustata*** Mosely, 1939c: 46 [type locality: Egypt; NHMUK; ♂]. —Ulmer 1963: 267 [distribution]. —Dia and Botosaneanu 1982: 140 [description of gynandromorphous specimen]. —Malicky 1983b: 47 [atlas; ♂]. —Kumanski and Malicky 1984: 199 [distribution]. —Moubayed and Botosaneanu 1985: 63 [distribution]. —Kumanski 1985: 124 [♂]. —Sipahiler and Malicky 1987: 106, 122 [distribution]. —Rojas-Camousseight and Tachet 1988: 315 [♀]. —Spuris 1989: 15 [checklist]. —González et al. 1990: 214 [distribution]. —Botosaneanu 1992: 63 [♂; ♀]. —Nógrádi and Uherkovich 1994: 31 [distribution]. —Nógrádi 1994: 277 [♂ ♀]. —Malicky 1997: 141 [distribution; ♂]. —Uherkovich and Nógrádi 1997: 461 [distribution]. —Malicky 1999c: 96 [distribution]. —Uherkovich and Nógrádi 1999: 420 [distribution]. —Malicky 1999f: 31 [distribution]. —Uherkovich and Nógrádi 2001: 95 [distribution]. —Nógrádi and Uherkovich 2001: 297 [checklist]. —Valle 2001: 66 [distribution]. —Mirmoayedi and Malicky 2002: 164 [distribution]. —Ujvárosi 2002: 384 [distribution]. —Sipahiler 2003b: 33 [distribution]. —Malicky 2004a: 57 [atlas]. —Huang et al. 2005: 469, 471 [distribution]. —Malicky 2005a: 59 [distribution]. —Sipahiler 2005: 396 [distribution]. —Chvojka 2006: 253 [distribution]. —Sipahiler 2007: 38 [distribution]. —Szczęsny and Godunko 2008: 14 [checklist]. —Chvojka and Komzák 2008: 13 [distribution]. —Ujvárosi et al. 2008: 112 [checklist]. —Oláh 2010: 91 [distribution]. —Ivanov 2011: 195 [checklist]. —González and Menéndez 2011: 118 [distribution]. —Komzák and Kroča 2011: 189 [distribution]. —Sipahiler 2012a: 7 [distribution]. —Kiss 2012: 28 [distribution]. —Komzák and Chvojka 2012: 719 [distribution]. —Corallini et al. 2013a: 38 [checklist]. —Malicky 2014b: 17 [teratological structures]. —Martín et al. 2015: 75 [distribution]. —Dia 2015: 51 [distribution]. —Sipahiler 2016: 12 [distribution]. —Cianficconi et al. 2016: 139 [distribution]. —Sipahiler 2016: 12 [distribution]. —Yang et al. 2016: 476 [checklist]. —Melnitsky et al. 2017: 6 [distribution]. —Sipahiler 2017b: 12 [distribution]. —Sipahiler 2018: 41 [distribution].

—*neglecta* Kumanski, 1983: 15 [type locality: [Bulgaria], from the outflow of limestone spring “Topliza” near Goze Delchev; SOFM; ♂; ♀]. —Kumanski 1985: 124 [to synonymy]. —Sipahiler and Malicky 1987: 84 [treated as likely synonym]. —Malicky 2005b: 543 [checklist].

**Distribution.** —Austria, Bulgaria, China, Cyprus, Czech Republic, Egypt, Greece, Hungary, Iran, Italy, Kazakhstan, Lebanon, Romania, Russia, Spain, Syria, Turkey, Ukraine, Uzbekistan.

***angustipennis*** (Martynov, 1934): 144 [type locality: [Russia]; depository not designated; ♂; in *Hydroptilina*]. —Spuris 1989: 16 [checklist]. —Ivanov 2011: 195 [checklist].

**Distribution.** —Russia.

***annulicornis*** Maatsumura, 1931: 1136 [type locality: [Japan]; holotype not designated; as *Hydroptilia*].

**Distribution.** —Japan.

***anongraksa*** Malicky & Chantaramongkol, 2007: 1016 [type locality: Thailand, Sai Yok NP, 14°26'N 98°51'E, 100 m; Collection Malicky; ♂]. —Malicky 2010a: 27 [atlas; ♂].

**Distribution.** —Thailand.

***antennopedia*** Sykora & Harris, 1994: 68 [type locality: [United States], Pennsylvania, Fayette Co., Youghiogheny River Lake outflow near Confluence; CMNH; ♂]. —Houghton et al. 2001: 504 [distribution]. —Myers et al. 2011: 106 [distribution]. —Houghton et al. 2017: 62 [checklist].

**Distribution.** —U.S.A.

***antilliarum*** Flint, 1968a: 58 [type locality: Dominica, Pont Casse, 1.6 mi W; NMNH; ♂; ♀]. —Malicky 1983c: 264 [distribution]. —Botosaneanu 1989: 100 [♂; scent organ; distribution]. —Flint and Sykora 1993: 50 [checklist]. —Botosaneanu 1994a: 40 [distribution]. —Sheath et al. 1995: 890 [red algal association]. —Botosaneanu 2000: 256 [distribution]. —Botosaneanu 2002b: 83 [checklist]. —Botosaneanu and Thomas 2005: 40 [distribution].

**Distribution.** —Dominica, Guadeloupe, Martinique, St. Lucia.

***apalachicola*** Harris, Pescador, & Rasmussen, 1998: 221 [type locality: [United States], Florida, Liberty County, Nature Conservancy Apalachicola Bluffs and Ravines Preserve, Little Sweetwater Creek; NMNH; ♂]. —Pescador et al. 2004: 132 [checklist]. —Harris et al. 2012: 5 [checklist].

**Distribution.** —U.S.A.

***arctia*** Ross, 1938a: 129 [type locality: United States, Idaho, Bear River Narrows; INHS; ♂]. —Ross and Spencer 1952: 47 [distribution]. —Denning 1947b: 175 [distribution]. —Flint and Herrmann 1976: 898 [distribution]. —Resh and Sorg 1978: 396 [distribution; as *arcita*]. —Blickle 1979: 47, 67 [checklist; ♂]. —Bueno-Soria 1984: 97 [♂; distribution]. —Moulton et al. 1994: 169 [distribution]. —Moulton and Stewart 1997: 350 [checklist]. —Ruiter 1999: 165 [distribution]. —Houghton 2001: 90 [distribution]. —Flint et al. 2003: 31 [distribution; does not occur in Hawaii misidentification of *H.potosina*]. —Baumgardner and Bowles 2005: 11 [distribution]. —Blinn and Ruiter 2005: 68 [distribution; biology]. Blinn and Ruiter 2006: 332 [biology; distribution]. —Zack et al. 2006: 134 [phenology; distribution]. —Bueno-Soria et al. 2007: 33 [distribution]. —Blinn and Ruiter 2009a: 303 [biology]. —Blinn and Ruiter 2009b: 186 [phenology; distribution]. —Vieira et al. 2009: 257 [distribution]. —Blinn and Ruiter 2013: 280, 291 [biology; distribution]. —Mendez et al. 2019: 118 [checklist]. —Razo-González et al. 2020: 5 [distribution].

—*acoma* Denning, 1947b: 175 [type locality: United States, California, Morgan Hill; ♂; UMSP]. —Blickle1979: 47 [to synonymy].

**Distribution.** —Canada, Mexico, U.S.A.

***arethusa*** Malicky, 1997: 148 [type locality: Portugal, Mantelaes; Collection Malicky; ♂]. —Malicky 2004a: 58 [atlas]. —Malicky 2005b: 543 [checklist]. —González and Menéndez 2011: 118 [distribution]. —Martínez et al. 2016: 51 [distribution].

**Distribution.** —Portugal, Spain.

***argentinica*** Flint, 1983: 43 [type locality: Argentina, Pcia. Tucumán, S Concepción; NMNH; ♂; ♀]. —Angrisano 1995a: 509 [distribution]. —Angrisano 1999: 32 [checklist]. —Blahnik et al. 2004: 5 [distribution]. —Paprocki et al. 2004: 11 [checklist]. —Angrisano and Sganga 2007: 32 [♂; ♀; distribution]. —Dumas et al. 2009: 366 [distribution]. —Calor 2011: 321 [checklist]. —Rueda Martín 2011: 6 —Dumas and Nessimian 2012: 15 [checklist]. —Paprocki and França 2014: 45 [checklist]. —Isa Miranda and Rueda Martín 2014: 199 [distribution].

**Distribution.** —Argentina, Bolivia, Brazil, Uruguay.

***argosa*** Ross, 1938a: 131 [type locality: [United States], Wyoming, Parco, along North Platte River; INHS; ♂]. —Denning 1947a: 149 [distribution]. —Resh and Sorg 1978: 396 [distribution]. —Blickle 1979: 47, 73 [checklist; ♂]. —Newell et al. 2001: 192 [distribution; phenology]. —Zack et al. 2006: 134 [phenology; distribution]. —Blinn and Ruiter 2013: 291 [biology; distribution]. —Mendez et al. 2019: 118 [checklist].

**Distribution.** —U.S.A.

***armata*** Ross, 1938a: 123 [type locality: [United States], Indiana, Winamac, drainage ditch west of town; INHS; ♂; ♀]. —Ross 1944: 147 [♂; ♀; larva; case; distribution]. —Denning 1947b: 173 [distribution]. —Etnier 1965: 146 [distribution] —Unzicker et al. 1970: 172 [distribution]. —Edwards 1973: 506 [distribution]. —Etnier and Schuster 1979: 17 [distribution]. —Roy and harper 1979: 150 [distribution]. —Etnier and Schuster 1979: 17 [checklist]. —Blickle 1979: 47, 65 [checklist; ♂]. —Parker and Voshell 1981: 4 [distribution]. —Harris et al. 1982a: 510 [distribution]. —Waltz and McCafferty 1983a: 9 [distribution]. —Huryn and Foote 1983: 790 [distribution]. —Hamilton et al. 1983: 18 [distribution]. —Harris et al. 1984: 108 [distribution]. —Bowles and Mathis 1989: 238 [distribution]. —Tarter 1990: 239 [distribution]. —Floyd and Schuster 1990: 130, 132 [distribution]. —Frazer et al. 1991: 19 [distribution]. —Harris et al. 1991: 169 [distribution]. —Masteller and Flint 1992: 69 [distribution]. —Mathis and Bowles 1992: 24 [distribution]. —Bowles and Mathis 1992: 32 [distribution]. —Masteller 1993: 134 [distribution]. —Floyd et al. 1993: 90 [phenology; distribution]. —Moulton and Stewart 1996: 97 [♂; distribution]. —Keiper and Foote 1999: 515 [biology; larva]. —Houghton et al. 2001: 504 [distribution]. —Pescador et al. 2004: 133 [distribution]. —DeWalt and Heinold 2005: 41 [phenology; distribution]. —Zeullig et al. 2006: 42 [distribution]. —Armitage et al. 2011: 14 [checklist]. —Houghton et al. 2011b: 5 [phenology; habitat; distribution]. —Flint 2011: 104 [distribution]. —Harris et al. 2012: 5 [checklist]. —DeWalt et al. 2016: 51 [distribution]. —Denson et al. 2016: 5 [distribution]. —Houghton et al. 2017: 62 [checklist]. —Bowles et al. 2020: 7 [distribution].

**Distribution.** —Canada, U.S.A.

***armathai*** Schmid, 1959b: 688 [type locality: Iran, Garna; CNC; ♂]. —Malicky 1983b: 45 [atlas; ♂]. —Sipahiler and Malicky 1987: 143 [distribution]. —Mirmoayedi and Malicky 2002: 164 [checklist]. —Malicky 2004a: 54 [atlas]. —Sipahiler 2005: 397 [distribution]. —Malicky 2005b: 543 [checklist]. —Sipahiler 2007: 38 [distribution]. —Ivanov 2011: 195 [checklist]. —Sipahiler 2018: 41 [distribution]. —Lonsdale 2020: 32 [holotype depository]. —Oláh et al. 2020: 45 [distribution].

**Distribution.** —Azerbaijan, Iran, Turkey.

***artemis*** Malicky, 1997: 148 [type locality: [Armenia], Asat bei Chuts; ZMHB; ♂]. —Malicky 2004a: 59 [atlas]. —Malicky 2005b: 543 [checklist].

**Distribution.** —Armenia.

***artesa*** Mathis & Bowles, 1990: 87 [type locality: [United States], Missouri, Shannon County, Alley Spring, Ozark National Scenic Riverways (O.N.S.R.), 5 mi W Eminence, Hwy 106; NMNH; ♂; ♀]. —Mathis and Bowles 1992: 24 [distribution]. —Moulton and Stewart 1996: 97 [♂; distribution]. —Armitage et al. 2011: 33 [♂].

**Distribution.** —U.S.A.

***asteria*** Malicky, 1997: 148 [type locality: [Turkey], Siirt, Botan Cayi-Tal; Collection Malicky; ♂]. —Malicky 2004a: 59 [atlas]. —Malicky 2005b: 543 [checklist]. —Sipahiler 2005: 397 [distribution]. —Mirmoayedi and Malicky 2002: 164 [distribution].

**Distribution.** —Turkey.

***astraia*** Malicky, 1997: 148 [type locality: Iran, 65 km W Schiras; Collection Malicky; ♂]. —Malicky 2004a: 59 [atlas]. —Malicky 2005b: 543 [checklist]. —Mirmoayedi and Malicky 2002: 164 [distribution].

**Distribution.** —Iran.

***asymmetrica*** Kumanski, 1990: 50 [type locality: Korea, Province Kangvon, stream and small torrents of the plain near Casan vill., 1–3 km from the sea (ca. 25 km E of Vonsan); SOFM; ♂; ♀]. —Arefina et al. 2002: 97 [distribution]. —Nozaki and Tanida 2007: 245 [distribution]. —Ito et al. 2011: 15 [♂, ♀; distribution]. —Ivanov 2011: 195 [checklist]. —Ito and Nagasaka 2014: 9 [distribution]. —Ito 2015: 15 [distribution]. —Tanida and Kuranishi 2016: 70 [checklist]. —Potikha and Vshivkova 2016: 364 [distribution]. —Park and Kong 2020: 297 [checklist].

**Distribution.** —Japan, Korea, Russia.

***atalante*** Malicky, 1997: 147 [type locality: [Bulgaria], Strandscha-Gebirge, 1 km S Kruschewez, 100 m; Collection Malicky; ♂]. —Malicky 2004a: 58 [atlas]. —Malicky 2005b: 543 [checklist]. —Oláh 2017: 137 [distribution].

**Distribution.** —Bulgaria.

***atargatis*** Malicky, 1997: 147 [type locality: [Lebanon], Jabboulé; Collection Malicky; ♂]. —Malicky 2004a: 59 [atlas]. —Malicky 2005b: 543 [checklist]. —Sipahiler 2005: 397 [distribution]. —Sipahiler 2007: 38 [distribution]. —Sipahiler 2012a: 7 [distribution]. —Dia 2015: 51 [distribution]. —Sipahiler 2016: 12 [distribution].

**Distribution.** —Lebanon, Turkey.

***ate*** Malicky, 1997: 146 [type locality: Pakistan, Penjab, Hassan Abdal; CNC; ♂]. —Malicky 2004a: 59 [atlas]. —Malicky 2005b: 543 [checklist].

**Distribution.** —India, Pakistan.

***auge*** Malicky, 1997: 146 [type locality: [Greece], Insel Lesbos, Agiassos, 300 m; Collection Malicky; ♂]. —Malicky 2004a: 59 [atlas]. —Malicky 2005b: 543 [checklist]. —Malicky 2005a: 59 [distribution]. —Sipahiler 2005: 397 [distribution]. —Karaouzas and Malicky 2016: 18 [distribution].

**Distribution.** —Greece, Turkey.

***auriscuspa*** Harris, Rasmussen, & Denson, 2012: 3 [type locality: [United States], Florida, Okaloosa Co., Blackwater River at Florida A&M University Biological Station, 4.5 mi NW Holt; NMNH; ♂].

**Distribution.** —U.S.A.

***aurora*** Malicky, 1997: 146 [type locality: [Tunisia], Oued Maden, 3 km S Nefza, 9°06'E, 36°55'N, 50 m; Collection Malicky; ♂]. —Malicky 2004a: 58 [atlas]. —Malicky 2005b: 543 [checklist]. —González and Menéndez 2011: 118 [distribution].

**Distribution.** —Morocco, Spain, Tunisia.

***autonoe*** Malicky, 1997: 145 [type locality: [Morocco], El Ksiba, 1100 m; Collection Malicky; ♂]. —Malicky 2004a: 58 [atlas]. —Malicky 2005b: 543 [checklist]. —González and Menéndez 2011: 118 [distribution].

**Distribution.** —Morocco, Portugal, Spain.

***banmaekap*** Malicky & Chantaramongkol, 2007: 1022 [type locality: Thailand, Ban Mae Kap, Nam Mae To, 18°51'N 98°37'E, 600 m; Collection Malicky; ♂]. —Malicky 2010a: 31 [atlas; ♂].

**Distribution.** —Thailand.

***batang*** Wells & Huisman, 1992: 98 [type locality: Brunei, Sg. Temburong, 140 m; RMNH; ♂]. —Malicky 2010a: 24 [atlas; ♂].

**Distribution.** —Brunei.

***batanta*** Oláh in Oláh and Kovács 2018: 178 [type locality: Indonesia, West Papua, Batanta Island, Northern cost, Warmon stream, above second waterfall, S00°50'29.47", E130°42'29.16"; Collection Oláh; ♂].

**Distribution.** —Indonesia.

***baukis*** Malicky, 1998a: 798 [type locality: [Indonesia, Central Java], Jawa Tengah, Gunung Selamat, Awu; Collection Malicky; ♂]. —Malicky 2010a: 25 [atlas; ♂]. —Malicky et al. 2014a: 5 [distribution].

**Distribution.** —Indonesia.

***begap*** Wells & Huisman, 1992: 102 [type locality: East Malaysia, Sabah, Tenom; NTM; ♂; ♀]. —Malicky 2010a: 34 [atlas; ♂].

**Distribution.** —East Malaysia.

***bellona*** Malicky, 1998a: 798 [type locality: [Indonesia], Sumatra, Fort de Kock; Collection Malicky; ♂]. —Malicky and Chantaramongkol 2007: 1023 [distribution]. —Malicky 2007a: 177 [checklist]. —Malicky 2010a: 29 [atlas; ♂]. —Melnitsky et al. 2019: 539 [distribution].

**Distribution.** —Indonesia, Malaysia, Thailand.

***bengkoka*** Wells, 1990b: 390 [type locality: [Indonesia] Sulawesi Utara, Dumoga-Bone N.P., Tumpah R. a1 km above Toraut R. junction; NMV; ♂; ♀; case]. —Malicky et al. 2010: 163 [distribution]. —Malicky et al. 2014b: 832 [distribution].

**Distribution.** —Indonesia.

***berkait*** Wells & Huisman, 1992: 101 [type locality: East Malaysia, Sabah, 8.5 km S Long Pa Sia, Sg. Malabit, 04°21'N 115°41'E, 1180 m; RMNH; ♂]. —Malicky 2010a: 34 [atlas; ♂].

**Distribution.** —Malaysia.

***berneri*** Ross, 1941a: 67 [type locality: [United States], Florida, Alachua County, Santa Fe River; INHS; ♂; ♀]. —Etnier 1965: 146 [distribution]. —Blickle 1979: 47, 69 [checklist; ♂]. —Roy and Harper 1975: 1082 [distribution]. —Roy and Harper 1979: 151 [checklist]. —Roy and Harper 1981: 105 [distribution]. —Harris et al. 1982a: 510 [distribution]. —Bowles and Mathis 1989: 238 [distribution]. —Harris et al. 1991: 170 [distribution]. —Moulton and Stewart 1996: 97 [♂; distribution]. —Abbott et al. 1997: 44 [distribution]. —Moulton and Stewart 1997: 350 [checklist]. —Pescador et al. 2004: 133 [checklist]. —Harris et al. 2012: 5 [checklist]. —Denson et al. 2016: 5 [distribution]. —Houghton et al. 2017: 62 [checklist].

**Distribution.** —Canada, U.S.A.

***biankii*** Ivanov, 1992: 234[type locality: [Kyrgyzstan], West Tianshan, Kyzart-Ouzy on the river Kara-Su under Chom-Tash mountain chain; ZIN; ♂].

**Distribution.** —Kyrgyzstan.

***bibir*** Wells & Huisman, 1992: 102 [type locality: East Malaysia, Sabah, Kinabalu National Park, Liwagu River; NTM; ♂; ♀]. —Malicky 2010a: 34 [atlas; ♂].

**Distribution.** —East Malaysia.

***bichromata*** Mey, 1998a: 557 [type locality: [Philippines, Mindanao], northern slope of Mt. Atuuganon range, 1050 m; ZMHB; ♂]. —Wells and Mey 2002: 134 [checklist].

**Distribution.** —Philippines.

***bidens*** Flint, 1983: 45 [type locality: Argentina, Ocia. Jujuy, Aguas Calientes; NMNH; ♂; ♀]. —Angrisano 1999: 32 [checklist]. —Rueda Martín 2011: 7 [♂; distribution]. —Isa Miranda and Rueda Martín 2014: 199 [distribution].

**Distribution.** —Argentina, Bolivia.

***bispina*** Kimmins, 1962: 106 [type locality: [Indonesia], Papua, Kokoda, 1200 ft; NHMUK; ♂; ♀]. —Wells 1984: 269 [distribution]. —Neboiss 1986: 61 [atlas; ♂]. —Wells 1991: 501, 526 [distribution; checklist].

**Distribution.** —Indonesia.

***bispinatella*** Mey, 2003b: 426 [replacement name for *H.bispina* Wells & Mey, 2002: 126, preoccupied by *H.bispina* Kimmins, 1962: 106] [type locality: [Philippines] Luzon, Camarines Sur, Mt Isarog, Pili, 600–800 m; BPBM; ♂]. —Malicky and Chantaramongkol 2007: 1023 [distribution]. —Malicky 2009b: 10 [distribution].

**Distribution.** —Philippines.

***biuncialis*** Zhou & Yang in Zhou, Sun, and Yang 2009a: 905, 910[type locality: [China], Jiangxi Province, Wuyishan National Nature Preserve, Litoujian Stream, 100 m upstream of protected area, 27.99°N, 117.86°E, 342 m; NAUJ; ♂]. —Yang et al. 2016: 476 [checklist].

**Distribution.** —China.

***blicklei*** Sykora & Harris, 1994: 72 [type locality: [United States], Maine, Dennistown; UNHC; ♂]. —Myers et al. 2011: 106 [distribution].

**Distribution.** —U.S.A.

***botosaneanui*** Kumanski, 1990: 48 [type locality: Korea, Province Kangvon, Kumgang Mts., the foothills near the hotel Go-song and Ondžong vill.; SOFM; ♂; ♀]. —Arefina et al. 2002: 97 [distribution]. —Ito et al. 2011: 20 [♂, ♀; distribution]. —Ivanov 2011: 195 [checklist]. —Ito 2015: 8 [checklist]. —Tanida and Kuranishi 2016: 70 [checklist]. —Potikha and Vshivkova 2016: 364 [distribution]. —Nozaki et al. 2019: 167 [distribution]. —Park and Kong 2020: 297 [checklist].

**Distribution.** —Japan, Korea, Russia.

***bozontos*** Oláh, 2012: 48 [type locality: Indonesia, Papua, Raja Empat Archipelago, Batanta Island, Warmon Creek, 2. waterfall, 0°50'23.25"S 130°42'35.18"E; Collection Oláh; ♂].

**Distribution.** —Indonesia.

***brailovskyi*** Bueno-Soria, 1984: 122 [type locality: Mexico, Veracruz, Chicontepec; CNIN; ♂]. —Harris and Holzenthal 1999: 34 [♂; distribution].

**Distribution.** —Costa Rica, Mexico.

***bribriae*** Harris, 2002: 50 [type locality: [United States], Florida, Santa Rosa County, Indigo Creek, at Base Rd. 213, Eglin Air Force Base; NMNH; ♂]. —Pescador et al. 2004: 133 [checklist]. —Harris et al. 2012: 5 [checklist].

**Distribution.** —U.S.A.

***brigittae*** Gibon, 1987a: 128 [type locality: sur le Niandan à Bambaya; MNHN; ♂]. —Wells and de Moor 2020: 500 [♂; distribution].

**Distribution.** —Angola, Guinea.

***brincki*** Jacquemart, 1963a: 409 [type locality: [South Africa], National Park, Tugela Valley, 5000 ft, at stony river (Loc. N° 258); IRSNB; ♂].

**Distribution.** —South Africa.

***brissaga*** Malicky, 1996a: 101 [type locality: [Switzerland], Tessin, Gordevio im Maggiatal; depository not designated; ♂]. —Cianficconi et al. 1999b: 278 [distribution]. —Malicky 2002: 4 [distribution]. —Urbanič 2004: 51 [distribution]. —Malicky 2004a: 60 [atlas]. —Malicky 2005a: 60 [distribution]. —Malicky 2005b: 543 [checklist]. —Lubini-Ferlin and Vicentini 2005: 67 [checklist]. —Szczęsny and Godunko 2008: 14 [checklist]. —Corallini and Cianficconi 2011: 628 [checklist]. —Corallini et al. 2013a: 38 [checklist]. —Karaouzas and Malicky 2015: 14 [distribution]. —Malicky 2016b: 22 [morphological comparison with *H.tacheti*]. —Cianficconi et al. 2016: 139 [distribution].

**Distribution.** —Greece, Italy, Slovenia, Switzerland, Ukraine.

***broweri*** Blickle, 1963: 18 [type locality: [United States], Maine, Allagash; INHS; ♂]. —Blickle 1979: 47, 71 [checklist; ♂]. —Mathis and Bowles 1992: 24 [distribution]. —Moulton and Stewart 1996: 98 [♂; distribution]. —Etnier 2010: 485 [distribution]. —Bowles et al. 2020: 7 [distribution].

**Distribution.** —U.S.A.

***bugata*** Wells, 1984: 267 [type locality: [Papua] New Guinea, NE., Bugu River, E. of Lae, 100 m; BPBM; ♂]. —Neboiss 1986: 62 [atlas; ♂]. —Wells 1991: 526 [checklist].

**Distribution.** —Papua New Guinea.

***bumbulensis*** Wells & Andersen, 1995: 161 [type locality: Tanzania, Tanga region, West Usambara Mts, Dule, Bumbuli River, 1220 m a.s.l.; ZMUB; ♂].

**Distribution.** —Tanzania.

***bureschi*** Kumanski, 1972: 1261 [type locality: [Bulgaria], Balkangebirge, kleiner Bach, Nebenfluß des Iskār beim Dorf Bov; SOFM; ♂]. —Botosaneanu and Malicky 1978: 341 [possible synonym of *H.vichtaspa*].

**Distribution.** —Bulgaria.

***caesariata*** Zhou & Yang in Zhou et al. 2009b: 355 [type locality: China, Guangxi Zhuang Autonomous Region, Shangsi City, Nalin He, tributary of Mingjiang He, 2.0 km NW of main entrance to Shiwandashan National Forest Park, 21°54'N 107°53'E, 281 m; NAUJ; ♂]. —Yang et al. 2016: 476 [checklist].

**Distribution.** —China.

***calcara*** Wells, 1978: 753 [type locality: [Australia], New South Wales, Maclaughlin River, near Ando; NMV; ♂; ♀]. —Wells 1985b: 6 [case]. —Neboiss 1986: 63 [atlas; ♂; ♀].

**Distribution.** —Australia.

***callia*** Denning, 1947a: 149 [type locality: [United States], North Carolina, Raleigh; ESUW; ♂]. —Morse and Blickle 1953: 72 [checklist]. —Etnier 1968: 191 [distribution]. —Roy and Harper 1975: 1082 [distribution]. —Roy and Harper 1979: 151 [checklist]. —Blickle 1979: 47, 63 [checklist; ♂]. —Parker and Voshell 1981: 4 [checklist]. —Huryn and Foote 1983: 790 [distribution]. —Harris et al. 1984: 108 [distribution]. —Morse et al. 1989: 22 [distribution]. —Harris et al. 1991: 171 [distribution]. —Frazer et al. 1991: 19 [distribution]. —Masteller and Flint 1992: 69 [checklist]. —Houghton et al. 2001: 504 [distribution]. —DeWalt and Heinold 2005: 41 [phenology; distribution]. —Armitage et al. 2011: 14 [checklist]. —Houghton et al. 2017: 62 [checklist; as *calia*].

**Distribution.** —Canada, U.S.A.

***calundoensis*** Marlier, 1965: 68 [type locality: [Angola] Moxico, Zambèze, Rives du Lac Calundo, Loc. 4647-17; MDLA; ♂]. —Wells and de Moor 2020: 512 [checklist].

**Distribution.** —Angola.

***caminopa*** Mey, 1998a: 555 [type locality: [Philippines, Mindanao], northern slope of Mt. Atuuganon range, 1050 m; ZMHB; ♂]. —Wells and Mey 2002: 134 [checklist].

**Distribution.** —Philippines.

***campanulata*** Morton, 1896: 103 [type locality: [Algeria]; NHMUK; ♂]. —Morton 1904: 324 [distribution]. —Schmid 1952: 650 [distribution; ♂]. —Kimmins 1957a: 107 [lectotype designation]. —Botosaneanu 1967: 294 [distribution]. —Botosaneanu and Malicky 1978: 341 [checklist]. —Malicky 1983b: 46 [atlas; ♂]. —Malicky and Lounaci 1987: 15, 17 [checklist]. —González et al. 1990: 214 [distribution]. —Malicky 1997: 142 [distribution; ♂]. —Malicky 2004a: 58 [atlas]. —Malicky 2005b: 543 [checklist]. —González and Menéndez 2011: 118 [distribution]. —Martín et al. 2015: 75 [distribution]. —Ruiz-García et al. 2016: 4 [distribution].

**Distribution.** —Algeria, Morocco, Spain, Tunisia.

***caperata*** Wells, 1984: 264 [type locality: [Papua] New Guinea, SE., Kokoda, 400 m; BPBM; ♂]. —Neboiss 1986: 64 [atlas; ♂]. —Wells 1991: 526 [checklist].

**Distribution.** —Papua New Guinea.

***carara*** Mey, 1998a: 557 [type locality: Costa Rica, San José, Reserva Biológica Carara, Quebrada Bonita, 9.775°N, 84.605°W; NMNH; ♂].

**Distribution.** —Costa Rica.

***carolae*** Holzenthal & Kelley, 1983: 466 [type locality: [United States], South Carolina, Aiken Co., Savannah River Plant, Upper Three Runs Creek at SRP road 8-1; NMNH; ♂].

**Distribution.** —U.S.A.

***catamarcensis*** Flint, 1983: 45 [type locality: Argentina, Pcia. Catamarca, Arroyo El Pintado, near La Viña; NMNH; ♂; ♀]. —Angrisano 1999: 32 [checklist].

**Distribution.** —Argentina.

***chattanooga*** Frazer & Harris, 1991b: 6 [type locality: [United States], Alabama, DeKalb County, West Fork of the Little River at Union covered bridge, near Cloudmont Resort (Sec. 9, T6S, R 10 E); NMNH; ♂]. —Frazer et al. 1991: 19 [distribution]. —Masteller and Flint 1992: 70 [distribution]. —Huryn and Harris 2000: 193 [distribution]. —DeWalt and Heinold 2005: 41 [phenology; distribution]. —Armitage et al. 2011: 14 [checklist].

**Distribution.** —U.S.A.

***cheaha*** Harris, 1991: 14 [type locality: [United States], Alabama, Talladega County, Dry Creek at Co. Hwy. 234, Talladega National Forest, 4.8 km SW Waldo (Sec. 23, T 19 S, R 5 E); NMNH; ♂]. —Harris et al. 1991: 172 [distribution].

**Distribution.** —U.S.A.

***chelops*** Harris, 1985a: 249 [type locality: [United States], Alabama, Choctaw County, unnamed spring along Hwy. 17, 4 miles SW Butler, T12N, R3W, S10; NMNH; ♂]. —Harris et al. 1991: 173 [distribution].

**Distribution.** —U.S.A.

***chinensis*** Xue & Yang, 1990: 126 [type locality: [China] Wudalianchi, Heilongjiang; NAUJ; ♂]. —Yang et al. 1997b: 93 [checklist]. —Morse et al. 2001: 201 [distribution]. —Arefina et al. 2002: 97 [♂, ♀; distribution]. —Yang et al. 2005: 458 [checklist]. —Ito et al. 2011: 5 [♂; ♀; distribution]. —Ivanov 2011: 195 [checklist]. —Ito and Nagasaka 2014: 9 [distribution]. —Ito 2015: 8 [checklist]. —Tanida and Kuranishi 2016: 70 [checklist]. —Yang et al. 2016: 476 [checklist]. —Potikha and Vshivkova 2016: 364 [distribution].

**Distribution.** —China, Japan, Russia.

***cintrana*** Morton, 1904: 324 [type locality: Portugal, Cintra; depository not designated; ♂]. —Botosaneanu 1967: 294 [distribution]. —Botosaneanu and Malicky 1978: 341 [checklist]. —Malicky 1983b: 46 [atlas; ♂]. —González et al. 1990: 214 [distribution]. —Malicky 1997: 142 [distribution; ♂]. —Malicky 2004a: 58 [atlas]. —Malicky 2005b: 543 [checklist]. —González and Menéndez 2011: 118 [distribution]. —Ruiz-García et al. 2016: 4 [distribution]. —Mabrouki et al. 2020: 12 [distribution].

**Distribution.** —Morocco, Portugal, Spain.

***circangula*** Harris, 1985b: 606 [type locality: [United States], Alabama, Baldwin County, Pine Log Creek at Hwy. 59; NMNH; ♂]. —Harris et al. 1991: 175 [distribution]. —Pescador et al. 2004: 133 [distribution]. —Harris et al. 2012: 5 [checklist]. —Denson et al. 2016: 5 [distribution].

**Distribution.** —U.S.A.

***cochlearis*** Xue & Yang, 1990: 127 [type locality: [China] Linxian Qihe, Henan; NAUJ; ♂]. —Xue et al. 1992: 353–356 [distribution]. —Yang et al. 1997b: 93 [checklist]. —Yang et al. 2005: 458 [checklist]. —Yang et al. 2016: 476 [checklist].

**Distribution.** —China.

***cognata*** Mosely, 1930b: 245 [type locality: France, Pyrénées-Orientales, Quillan; NHMUK; ♂]. —Botosaneanu 1967: 294 [distribution]. —Botosaneanu and Malicky 1978: 341 [checklist]. —Moretti and Cianficconi 1981: 201 [checklist]. —Malicky 1983b: 49 [atlas; ♂]. —Malicky 1998b: 396 [♂; distribution]. —Cianficconi et al. 1999a: 57 [distribution]. —Valle 2001: 66 [distribution]. —Coppa and Tachet 2004: 124 [♀]. —Malicky 2002: 4 [distribution]. —Urbanič 2004: 51 [distribution]. —Malicky 2004a: 61 [atlas]. —Malicky 2005b: 543 [checklist]. —Coppa and Tachet 2005: 130 [♀]. —Cianficconi and Corallini 2010: 87 [distribution]. —González and Menéndez 2011: 119 [distribution]. —Šemnički et al. 2011: 149 [distribution]. —Corallini et al. 2013a: 38 [checklist]. —Martín et al. 2015: 75 [distribution]. —Cianficconi et al. 2016: 139 [distribution].

**Distribution.** —Croatia, France, Italy, Slovenia, Spain.

***consimilis*** Morton, 1905: 65 [type locality: [United States] “Ithaca New York and Belfrage Texas”; depository not designated; ♂]. —Banks 1907a: 50 [catalogue]. —Mosely 1923: 293 [scent-organ]. —Betten 1934: 158 [♂; distribution]. —Ross 1944: 153 [♂; ♀; larva; distribution]. —Denning 1947b: 174 [distribution]. —Ross and Spencer 1952: 47 [distribution]. —Etnier 1965: 147 [checklist]. —Unzicker et al. 1970: 172 [distribution]. —Edwards 1973: 506 [distribution]. —Roy and Harper 1979: 151 [checklist]. —Etnier and Schuster 1979: 17 [checklist]. —Blickle 1979: 47, 67 [checklist; ♂]. —Parker and Voshell 1981: 4 [checklist]. —Swegman et al. 1981: 132 [distribution]. —Huryn and Foote 1983: 790 [distribution]. —Waltz and McCafferty 1983a: 10 [distribution]. —Light and Adler 1983: 77 [distribution; biology]. —Hamilton et al. 1983: 18 [distribution]. —Lake 1984: 219 [distribution]. —Steven and Hilsenhoff 1984: 163 [distribution]. —Bowles and Mathis 1989: 238 [distribution]. —Floyd and Schuster 1990: 130, 132 [distribution]. —Harris et al. 1991: 176 [distribution]. —Masteller and Flint 1992: 70 [checklist]. —Mathis and Bowles 1992: 24 [distribution]. —Bowles and Mathis 1992: 32 [distribution]. —Masteller 1993: 134 [distribution]. —Moulton et al. 1993: 21 [distribution]. —Moulton and Stewart 1996: 98 [♂; distribution]. —Moulton and Stewart 1997: 350 [checklist]. —Ruiter 1999: 165 [distribution]. —Keiper and Foote 2000: 226 [distribution; biology]. —Houghton 2001: 89 [checklist]. —Houghton et al. 2001: 504 [distribution]. —Blinn and Ruiter 2005: 68 [distribution; biology]. —Zeullig et al. 2006: 42 [distribution]. —Bowles et al. 2007: 21 [distribution; biology]. —Houghton and Holzenthal 2010: 486 [distribution]. —Biondi 2010: 60 [distribution]. —Flint 2011: 104 [distribution]. —Houghton et al. 2011a: 388 [distribution; biology]. —Houghton et al. 2011b: 5 [distribution; biology]. —Houghton et al. 2011a: 388 [distribution; biology]. —Armitage et al. 2011: 14 [checklist]. —Houghton et al. 2013: 37 [distribution; biology]. —Blinn and Ruiter 2013: 291 [biology; distribution]. —Ruiter et al. 2013: 3 [distribution; DNA barcoding; larval-adult association]. —DeWalt et al. 2016: 51 [distribution]. —Houghton 2016: 46 [biology]. —Houghton et al. 2017: 62 [checklist]. —Mendez et al. 2019: 128 [checklist]. —Bowles et al. 2020: 7 [distribution].

**Distribution.** —Canada, U.S.A.

***constricta*** Bueno-Soria, 1984: 99 [type locality: Mexico, Chiapas, La Prusia; NHMUK; ♂]. —Flint 1991b: 47 [♂; ♀; distribution]. —Flint and Reyes 1991: 484 [distribution]. —Harris and Holzenthal 1999: 34 [♂; distribution]. —Muñoz-Quesada 2000: 277 [checklist]. —Mey and Ospina-Torres 2018: 28 [♂; distribution].

**Distribution.** —Belize, Colombia, Costa Rica, Honduras, Mexico, Peru.

***coreana*** Kumanski, 1990: 52 [type locality: Korea, Province Phyongan pukdo (Northern Phyongan), Myohyang Mts., the foothills, the hotel; SOFM; ♂]. —Arefina et al. 2002: 98 [distribution]. —Mey and Nozaki 2006: 24 [distribution]. —Ito et al. 2011: 18 [♂; ♀; distribution]. —Ivanov 2011: 195 [checklist]. —Ito 2015: 8 [checklist]. —Tanida and Kuranishi 2016: 70 [checklist]. —Kobayashi et al. 2017: 17 [distribution]. —Park and Kong 2020: 297 [checklist].

**Distribution.** —Japan, Korea, Russia.

***cornea*** Yang & Xue, 1994: 10 [type locality: [China], Sichuan, Ping-wu county, 19 km E of Ping-wu, tributary of Fu-jiang River, 1090 m; NAUJ; ♂]. —Yang et al. 1997b: 93 [checklist]. —Yang et al. 2005: 458 [checklist]. —Yang et al. 2016: 476 [checklist].

**Distribution.** —China.

***cornuta*** Mosely, 1922: 179 [type locality: [England], “Britain”; NHMUK; ♂]. —Mosely 1923: 292 [scent-organ]. —Martynov 1934: 129 [♂]. —Racięcka 1936: 98 [distribution]. —Mosely 1939b: 262 [♂]. —Tjeder 1941: 10 [♀; distribution]. —Berg 1948: table 14 [distribution]. —Nybom 1960: 17 [checklist]. —Botosaneanu 1960: 148 [distribution]. —Kimmins 1961: 32 [comparison with *H.lotensis*; ♂; ♀]. —Botosaneanu 1967: 294 [distribution]. —Spuris 1972: 20 28 [checklist]. —Botosaneanu and Malicky 1978: 341 [checklist]. —Moretti and Cianficconi 1981: 201 [checklist]. —Malicky 1983b: 46 52 [atlas; ♂; ♀]. —Kumanski and Malicky 1984: 199 [distribution]. —Wiberg-Larsen 1985: 40 [checklist]. —Kumanski 1985: 122 [♂]. —González et al. 1986: 113 [distribution]. —Andersen and Wiberg-Larsen 1987: 168 [checklist]. —Sipahiler and Malicky 1987: 129 [distribution]. —Rojas-Camousseight and Tachet 1988: 315 [♀]. —Spuris 1989: 15 [checklist]. —González et al. 1990: 212 [checklist]. —Andersen et al. 1993b: 3 [distribution]. —Bagge 1995: 94, 95 [distribution; biology]. —Maier et al. 1995: 147 [distribution]. —Malicky 1997: 142 [♂]. —Gullefors 2002: 138 [checklist]. —Gullefors 2003: 194 [distribution]. —Malicky 2004a: 57 [atlas]. —Malicky 2005b: 543 [checklist]. —Sipahiler 2005: 397 [distribution]. —Gullefors 2006: 137 [distribution]. —Sipahiler 2007: 38 [distribution]. —Robert 2007: 82 [checklist]. —Szczęsny and Godunko 2008: 14 [checklist]. —Neu 2010: 149 150 [♀]. —Ivanov 2011: 195 [checklist]. —Viidalepp et al. 2011: 196 [distribution]. —Kiss 2012: 28 [distribution]. —Corallini et al. 2013a: 38 [checklist]. —O’Connor 2015: 28, 79 [distribution]. —O’Connor and O’Connor 2015: 203 [distribution]. —Cianficconi et al. 2016: 139 [distribution]. —Pan’kov and Krasheninnikov 2016: 333 [distribution]. —Gullefors 2016: 155 [checklist]. —Wallace 2016: 15, 20, 22, 23, 26, 48 [conservation status]. —O’Connor and O’Connor 2017b: 52 [distribution]. —Küçükbasmaci and Kiyak 2017: 488 [distribution]. —O’Connor and O’Connor 2018: 81 [distribution]. —Gullefors 2018: 108 [biology; distribution]. —O’Connor et al. 2018: 23 [distribution]. —Sipahiler 2018: 41 [distribution]. —O’Connor 2020: 140 [distribution].

**Distribution.** —Bulgaria, Denmark, England, Estonia, Finland, Germany, Hungary, Ireland, Italy, Lithuania, Norway, Poland, Russia, Spain, Sweden, Turkey, Ukraine.

***cortensis*** Mosely, 1937a: 121 [type locality: [France], Corsica; NHMUK; ♂]. —Botosaneanu 1967: 294 [distribution]. —Botosaneanu and Malicky 1978: 341 [checklist]. —Moretti and Cianficconi 1981: 201 [checklist]. —Malicky 1983b: 42 [atlas; ♂]. —Malicky 2004a: 51 [atlas]. —Malicky 2005b: 536, 544 [checklist]. —Cianficconi et al. 2007a: 67 [proposed as Italian endemic]. —Corallini et al. 2013a: 38 [checklist]. —Cianficconi et al. 2016: 139 [distribution].

**Distribution.** —France, Italy.

***coscaroni*** Flint, 1983: 46 [type locality: Argentina, Pcia. Salta, 5 km S Oran; NMNH; ♂]. —Angrisano 1999: 32 [checklist].

**Distribution.** —Argentina.

***cottaquilla*** Harris, 1994: 284 [type locality: [United States], Alabama, Calhoun County, South Branch to Cane Creek on Fort McClellan Military Reservation, Area 15C, 3 miles northeast Anniston; NMNH; ♂].

—*setigera* Harris, 1986a: 610 [type locality: [United States], Alabama, Calhoun County, South Branch to Cane Creek on Fort McClellan Military Reservation, Area 15C, 3 miles northeast Anniston; NMNH; ♂; preoccupied by Wells, 1984: 270]. —Harris et al. 1991: 205 [distribution]. —Harris 1994: 284 [replaced].

**Distribution.** —U.S.A.

***coweetensis*** Huryn, 1985: 444 [type locality: [United States], North Carolina, Macon County, Coweeta Hydrologic Laboratory, Experimental Watershed 27; NMNH; ♂; ♀; larva]. —Harris et al. 1991: 177 [distribution].

**Distribution.** —U.S.A.

***crenata*** (Ulmer, 1951): 91 [type locality: [Indonesia], Java, Sarangan, quelliger Zufluß am See Pasir; ZMUH; ♂; in *Pasirotrichia*]. —Malicky 1998a: 797 [♂; distribution]. —Malicky 2007a: 177 [checklist]. —Malicky 2010a: 29 [atlas; ♂]. —Malicky et al. 2014a: 5 [distribution].

**Distribution.** —Indonesia.

***cressae*** Thomson & Holzenthal, 2012: 23 [type locality: Venezuela, Bolívar, Gran Sabana, E. Pauji, “Río Curvita”, 04°31.237'N, 61°31.591'W, 869 m; UMSP; ♂].

**Distribution.** —Venezuela.

***cretosa*** Harris, 1985b: 611 [type locality: [United States], Alabama, Greene County, Trussels Creek at Co. Hwy. 23; NMNH; ♂]. —Harris et al. 1991: 178 [distribution].

**Distribution.** —U.S.A.

***criokera*** Harris, Rasmussen, & Denson, 2012: 2 [type locality: [United States], Florida, Liberty County, Gregory Mill Creek at CR-379, Apalachicola National Forest, N30°10'26", W85°00'48"; NMNH; ♂].

**Distribution.** —U.S.A.

***cruciata*** Ulmer, 1912b: 83 [type locality: [Tanzania], Deutsch-Ostafrika, Langenburg; ZMHB; ♂]. —Mosely 1934a: 149 [illustration of scent-organ]. —Johanson 1992: 118 [checklist]. —Wells and Andersen 1995: 160 [distribution]. —Kjærandsen and Andersen 1997: 244 [distribution]. —Malicky 1999a: 344 [distribution]. —Malicky 1999b: 492 [distribution]. —de Moor et al. 2000: 112 [distribution]. —Botosaneanu 2002a: 323 [discussion of male genitalia; ♂; distribution]. —Malicky 2004a: 62 [atlas]. —Malicky 2005b: 544 [checklist]. —de Moor 2007: 216 [distribution]. —Johanson and Mary 2009: 7 [distribution]. —Mey 2011: 343, 345 [distribution; checklist]. —de Moor 2011: 354 [distribution]. —Malicky 2015: 43 [checklist]. —Mey 2016: 305, 307 [distribution]. —Englmaier et al. 2020: 10 [distribution]. —Mey and de Moor 2019: 137, 139 [checklist; distribution]. —de Moor and Bellingan 2019: 157 [distribution]. —Wells and de Moor 2020: 500 [♂; distribution].

—*capensis* Barnard, 1934: 391 [type locality: [South Africa]; holotype not designated; depository not designated; ♂; ♀; larva; pupa]. —Scott 1963: 475 [distribution]. —Jacquemart 1963a: 405 [distribution]. —Botosaneanu 2002a: 323 [to synonymy].

—*hirra* Mosely, 1948b: 81 [type locality: [Yemen], Western Aden Protectorate, Wadi Dareija, near Dhala, c. 4600 ft; NHMUK; ♂]. —Botosaneanu and Gasith 1971: 99 [distribution]. —Botosaneanu 1973: 66 [♂]. —Botosaneanu and Giudicelli 1981: 21 [larva; biology; distribution]. —Botosaneanu 1982b: 11 [habitat threat]. —Malicky 1983a: 106 [to synonymy; distribution]. —Malicky 1983b: 50, 52 [atlas; ♂; ♀]. —Gibon 1987a: 125 [distribution]. —Botosaneanu 1992: 78 [head; ♂; ♀]. —Malicky 1999a: 344 [distribution].

—*airensis* Jacquemart, 1980b: 2 [type locality: [Niger], Guelta de Timia; IRSNB; ♂]. —Malicky 1983a: 106 [to synonymy; distribution].

**Distribution.** —Angola, Benin, Cabo Verde, Côte d’Ivoire, Ethiopia, Ghana, Guinea, Israel, Madagascar, Namibia, Niger, South Africa, Tanzania, Togo, the Comoros, Yemen.

***cubana*** Kumanski, 1987: 30 [type locality: Cuba, Province Las Villas, the massive of Guamuaya, Rio Nabujina near El Piojillo village; SOFM; ♂; ♀]. —Flint 1996a: 16 [checklist]. —Botosaneanu 2002b: 83 [checklist]. —Naranjo López and González Lazo 2005: 149 [checklist].

—*pseudomeralda* Botosaneanu, 1979: 51 [*nomen nudum*, attributed to Sykora]. —Kumanski 1987: 30 [to synonymy].

**Distribution.** —Cuba.

***cuembica*** Wells & de Moor, 2020: 498 [type locality: Angola, Moxico Province, Cuando River, Site 6 — Cuando campsite bridge, -13.5265, 19.27921; AMGS; ♂].

**Distribution.** —Angola.

***cuneata*** Wells & Dudgeon, 1990: 169 [type locality: Hong Kong, Tai Po Kao Forest stream; NHMUK; ♂]. —Harris and Holzenthal 1999: 38 [♂; distribution]. —Yang et al. 2016: 476 [checklist].

**Distribution.** —Hong Kong.

***curvata*** Bueno-Soria, 1984: 123 [type locality: Honduras, El Zamorano; NMNH; ♂].

**Distribution.** —Costa Rica, Honduras.

***dampfi*** Ulmer, 1929: 264 [type locality: [Germany]; depository not designated; ♂]. —Botosaneanu 1967: 294 [distribution]. —Botosaneanu and Malicky 1978: 341 [checklist]. —Andrikovics and Ujhelyi 1983: 6 [distribution]. —Malicky 1983b: 43 [atlas; ♂]. —Nógrádi 1986: 137 [distribution; ♀]. —Spuris 1989: 15 [checklist]. —Xue and Yang 1991: 21 [distribution]. —Xue et al. 1992: 353–356 [distribution]. —Nógrádi and Uherkovich 1994: 31 [distribution]. —Czachorowski 1995: 279 [distribution]. —Yang et al. 1997b: 93 [checklist]. —Uherkovich and Nógrádi 1997: 461 [distribution]. —Graf et al. 1998: 206 [distribution]. —Malicky 1999f: 31 [distribution]. —Uherkovich and Nógrádi 1999: 420 [distribution]. —Turunen 1999: 2 [distribution]. —Uherkovich and Nógrádi 2001: 95 [distribution]. —Nógrádi and Uherkovich 2001: 297 [checklist]. —Malicky 2002: 4 [distribution]. —Malicky 2004a: 52 [atlas]. —Malicky 2005a: 60 [distribution]. —Yang et al. 2005: 458 [checklist]. —Lubini-Ferlin and Vicentini 2005: 67 [checklist]. —Robert 2007: 82 [checklist]. —Bochert 2007: 119 [distribution; biology; ♂]. —Ito et al. 2011: 11 [♂ ♀; distribution]. —Ivanov 2011: 195 [checklist]. —Ito 2015: 8 [checklist]. —Vshivkova et al. 2016: 78, 79 [distribution]. —Yang et al. 2016: 476 [checklist]. —Tanida and Kuranishi 2016: 70 [checklist]. —Graf and Leitner 2016: 37 [distribution]. —Graf et al. 2017: 48 [distribution]. —Kobayashi et al. 2017: 17 [distribution]. —Park et al. 2018: 102 [♂; ♀; distribution]. —Park and Kong 2020: 297 [checklist].

—*itoi*Kobayashi 1977: 5 [type locality: [Japan] Utonai Pond Utonai Tomakomai-shi Hokkaido; depository not designated; ♂; ♀]. —Ito and Kawamura 1980: 113 [larva; pupa; case; biology]. —Ito et al. 1993: 142 [checklist]. —Morse et al. 2001: 102 [distribution]. —Arefina 2002: 8 [distribution]. —Tanida et al. 2005: 442 [larva; ♂]. —Ivanov 2011: 195 [checklist]. —Ito et al. 2011: 11 [to synonymy]. —Potikha and Vshivkova 2016: 364 [distribution].

—*ezoensis* (Kobayashi, 1977): 5 [type locality: [Japan], Utonai Pond, Utonai, Tomakomai-shi, Hokkaido; depository not designated; ♂; in *Oxyethira*]. —Ito and Kawamura 1984: 315 [as synonym of *H.itoi*]. —Tanida and Kuranishi 2016: 70 [as synonym of *H.dampfi*]. —Ito and Oláh 2017: 24 [to synonymy].

—*volgensis* Kachalova & Muhametšina, 1979: 82 [type locality: [Russia, Volga delta]; type depository not given; ♂; larva]. —Spuris 1989: 16 [checklist]. —Malicky 1999f: 32 [to synonymy]. —Malicky 2005b: 544 [checklist].

**Distribution.** —Austria, China, Finland, France, Germany, Greece, Hungary, Japan, Poland, South Korea, Russia, Switzerland.

***dandik*** Oláh & Johanson, 2010a: 14 [type locality: Malaysia, Sabah, Tawau, Maliau Basin, Nepenthes Camp, Camel Trophy Hut, 4°43'59.3"N 116°52'39.7"E, 999 m; NHRS; ♂].

**Distribution.** —Malaysia.

***danieli*** Harris & Armitage in Armitage et al. 2011: 30 [type locality: [United States], Ohio, Erie County, Margaretta Twp., Resthaven Wildlife Area, N41.4067, W82.81813; NMNH; ♂].

**Distribution.** —U.S.A.

***darda*** Oláh, 2016: 110 [type locality: Philippines, Negros Patag NR, 750 m; Collection Oláh; ♂].

**Distribution.** —Philippines.

***daun*** Wells & Huisman, 1992: 103 [type locality: East Malaysia, Sabah, Bundu Tuhan, Sg. Laidan, 05°58'N 116°31'E, 950 m; RMNH; ♂]. —Malicky 2010a: 31 [atlas; ♂].

**Distribution.** —Malaysia.

***dayung*** Wells & Huisman, 1992: 105 [type locality: East Malaysia, Sabah, 60 km W Lahad Datu, DVFC, confluence Sg. Segama - Sg. Palum Tambun, 04°58'N 117°48'E, 150 m; RMNH; ♂]. —Malicky and Chantaramongkol 2007: 1024 [♂; distribution]. —Malicky 2010a: 33 [atlas; ♂]. —Malicky 2014a: 1622 [checklist]. —Malicky et al. 2016: 92 [distribution]. —Yang et al. 2016: 476 [checklist].

**Distribution.** —Indonesia, Malaysia, Taiwan, Thailand, Vietnam.

***decia*** Etnier & Way, 1973: 425 [type locality: [United States], Ten-mile creek at bridge 0.5 air miles south of Kingston Pike (U.S. Highway 11 and 70), near Ebeneezer Road, Knox Co., Tenn.; NMNH; ♂]. —Blickle 1979: 48, 69 [checklist; ♂]. —Etnier and Schuster 1979: 17 [checklist].

—*choccolocco* Harris, 1985b: 609 [type locality: [United States], Alabama, Calhoun County, Choccolocco Creek, unmarked county road, 1.5 miles east Jenkins; NMNH; ♂]. —Harris et al. 1991: 174 [distribution]. —Harris and Etnier 1994: 262 [to synonymy].

**Distribution.** —U.S.A.

***dejaloni*** Botosaneanu, 1980: 166 [type locality: central Spain, Rio Jarama; ZMUA; ♂]. —Malicky 1983b: 43 [atlas; ♂]. —Malicky and Lounaci 1987: 15, 17 [checklist]. —Malicky 2004a: 52 [atlas]. —Malicky 2005b: 544 [checklist]. —González and Menéndez 2011: 119 [distribution].

**Distribution.** —Portugal, Spain.

***delineata*** Morton, 1905: 6 [type locality: [United States], Ithaca, New York; depository not designated; ♂; as *delineatus*]. —Banks 1907a: 50 [catalogue]. —Mosely 1923: 293 [scent-organ]. —Sibley 1926: 205 [biology]. —Betten 1934: 158 [♂; distribution]. —Etnier 1968: 191 [distribution]. —Roy and Harper 1979: 151 [checklist]. —Etnier and Schuster 1979: 17 [distribution]. —Blickle 1979: 48, 65 [checklist; ♂]. —Parker and Voshell 1981: 4 [checklist]. —Roy and Harper 1981: 105 [distribution]. —Waltz and McCafferty 1983a: 10 [distribution]. —Harris et al. 1984: 108 [distribution]. —Usis and Foote 1989: 84 [distribution]. —Bowles and Mathis 1989: 238 [distribution]. —Tarter 1990: 239 [checklist]. —Harris et al. 1991: 179 [distribution]. —Frazer et al. 1991: 19 [distribution]. —Masteller and Flint 1992: 70 [checklist]. —Floyd et al. 1993: 90 [phenology; distribution]. —Moulton and Stewart 1996: 99 [♂; distribution]. —Huryn and Harris 2000: 193 [distribution]. —Houghton et al. 2001: 504 [distribution]. —DeWalt and Heinold 2005: 41 [phenology; distribution]. —Biondi 2010: 61 [distribution]. —Flint 2011: 104 [distribution]. —Armitage et al. 2011: 14 [checklist]. —Houghton et al. 2017: 62 [checklist].

**Distribution.** —Canada, U.S.A.

***dentata*** Ross, 1938a: 126 [type locality: [United States], Virginia, Luray; INHS; ♂]. —Blickle 1979: 48, 67 [checklist; ♂]. —Parker and Voshell 1981: 4 [distribution]. —Masteller and Flint 1992: 70 [distribution]. —Myers et al. 2011: 106 [distribution].

**Distribution.** —U.S.A.

***dentina*** Mey, 1998a: 557 [type locality: [Philippines, Mindanao], northern slope of Mt. Atuuganon range, 1050 m; ZMHB; ♂]. —Wells and Mey 2002: 134 [checklist].

**Distribution.** —Philippines.

***denza*** Ross, 1948: 204 [type locality: Mexico, Tamaulipas, Hacienda Santa Engracia; INHS; ♂]. —Bueno-Soria and Flint 1978: 201 [distribution]. —Blickle 1979: 48, 71 [checklist; ♂]. —Waltz and McCafferty 1983b: 354 [distribution]. —Waltz and McCafferty 1983c: 414 [distribution]. —Bueno-Soria 1984: 114 [♂; distribution]. —Holzenthal 1988: 61 [distribution]. —Harris and Holzenthal 1999: 49 [head]. —Maes 1999: 1193 [checklist]. —Chamorro-Lacayo et al. 2007: 42 [checklist].

**Distribution.** —Costa Rica, Mexico, Nicaragua, U.S.A.

***desertorum*** Mey, 1993: 336 [type locality: China, Xinjiang, Kashi (=Kaschgar), Teichabfluß am Kaschgar-Fluß (39.29/75.59); ZMHB; ♂]. —Yang et al. 2005: 458 [checklist]. —Huang et al. 2005: 469 [distribution]. —Yang et al. 2016: 476 [checklist].

**Distribution.** —China.

***disgalera*** Holzenthal & Kelley, 1983: 466 [type locality: [United States], South Carolina, Aiken Co., Savannah River Plant, Upper Three Runs Creek at SRP road 8-1; NMNH; ♂]. —Harris et al. 1991: 180 [distribution]. —Pescador et al. 2004: 133 [checklist]. —Harris et al. 2012: 6 [checklist]. —Denson et al. 2016: 5 [distribution].

**Distribution.** —U.S.A.

***ditalea*** Flint, 1968b: 46 [type locality: Jamaica, St. Andrew, Fresh River, Ferry; NMNH; ♂; ♀]. —Flint 1968b: 82 [checklist]. —Bueno-Soria 1984: 119 [♂; distribution]. —Flint and Reyes 1991: 484 [distribution]. —Botosaneanu 1995a: 27 [distribution]. —Botosaneanu and Hyslop 1998: 16 [distribution]. —Flint and Pérez-Gelabert 1999: 39 [checklist]. —Botosaneanu 2002b: 83 [checklist]. —Flint and Sykora 2004: 31 [distribution]. —Pérez-Gelabert 2008: 300 [checklist]. —Ríos-Touma et al. 2017: 9 [checklist].

**Distribution.** —Dominican Republic, Ecuador, Jamaica, Mexico, Peru.

***dominicana*** Botosaneanu, 1995a: 27 [type locality: Dominican Republic, La Descubierta, north shore Lago Enriquillo, south from Sierra de Neiba; ZMUA; ♂; ♀]. —Botosaneanu 2002b: 83 [checklist]. —Flint and Pérez-Gelabert 1999: 39 [checklist]. —Flint and Sykora 2004: 31 [distribution]. —Pérez-Gelabert 2008: 300 [checklist].

**Distribution.** —Cuba, Dominican Republic.

***dorcas*** Mey, 1998a: 557 [type locality: [Philippines, Mindanao], northern slope of Mt. Atuuganon range, 1050 m; ZMHB; ♂]. —Wells and Mey 2002: 134 [checklist].

**Distribution.** —Philippines.

***dorsoprocessuata*** Botosaneanu, 1993a: 186 [type locality: [Russia], South Siberia, Tchitinskaia Oblasti (east from Tchita), at Ukurei - a village situated on Kuenga, a tributary of Shilka River (basin of Argun River); ZIN; ♂]. —Botosaneanu 1993c: 247 [addenda]. —Arefina 2004: 211 [distribution]. —Ivanov 2011: 195 [checklist]. —Potikha and Vshivkova 2016: 364 [distribution]. —Ito and Shimura 2019: 27 [♂; ♀; distribution].

**Distribution.** —Japan, Russia.

***dumoga*** Oláh, 2016: 112 [type locality: [Indonesia}, Dumoga-Bone N. P. Sulawesi; “specimens were not available; species description is based on the published drawings”; ♂].

**Distribution.** —Indonesia.

***ebroensis*** Harris, Rasmussen, & Denson, 2012: 4 [type locality: [United States], Florida, Bay Co., Little Crooked Creek at SR-79, 1.5 mi S Ebro, Pine Log State Forest, N30°24'48", W85°52'04"; NMNH; ♂].

**Distribution.** —U.S.A.

***eglinensis*** Harris, 2002: 49 [type locality: [United States], Florida, Okaloosa County, Rogue Creek, 0.6 km S Base Rd. 232, Eglin Air Force Base, 30°33'19"N, 86°34'51"W; NMNH; ♂]. —Pescador et al. 2004: 133 [checklist]. —Harris et al. 2012: 6 [checklist].

**Distribution.** —U.S.A.

***eileithyia*** Malicky, 1999a: 345 [type locality: [Yemen], Provinz Al-Mahwit, 30 km NE Bajil, 5 km NNE Khamis Bani Sa’d, 750 m, 15°11'N, 43°32'E; Collection Malicky; ♂]. —Malicky 2004a: 55 [atlas]. —Malicky 2005b: 544 [checklist].

**Distribution.** —Yemen.

***elongata*** (Ulmer, 1951): 86 [type locality: [Indonesia], Java, Kali Tjiwalen bei Tjibodas, ca. 1370 m; ZMUH; ♂; in *Oeceotrichia*]. —Malicky 1998a: 797 [♂; distribution]. —Malicky 2010a: 29 [atlas; ♂]. —Malicky et al. 2014a: 5 [distribution].

**Distribution.** —Indonesia.

***englishi*** Hamilton in Morse et. al 1989: 26 [type locality: [United States], South Carolina, Oconee County, Thompson River at North Carolina border, about 1,440 ft [439 m], Duke Power Company locality #583.2; NMNH; ♂].

**Distribution.** —U.S.A.

***engywuck*** Malicky & Lounaci, 1987: 6 [type locality: [Tunisia], Oued Titria (5 km E Ain Sobah); depository not designated; ♂]. —Malicky 1997: 143 [distribution; ♂]. —Malicky 2004a: 58 [atlas]. —Malicky 2005b: 544 [checklist]. —González and Menéndez 2011: 119 [distribution].

**Distribution.** —Spain, Tunisia.

***eramosa*** Harper, 1973: 393 [type locality: [Canada], Eramosa River at Cedar Valley, Wellington County, Ontario; QMOR; ♂]. —Blickle 1979: 48, 67 [checklist; ♂]. —Parker and Voshell 1981: 4 [checklist].

**Distribution.** —Canada, U.S.A.

***erawan*** Malicky & Chantaramongkol, 2007: 1018 [type locality: Thailand, Prov. Kanchanaburi, Erawan NP, 14°22'N 99°08'E, 200 m; Collection Malicky; ♂]. —Malicky 2010a: 25 [atlas; ♂].

**Distribution.** —Thailand.

***erkakanae*** Sipahiler, 1997: 15 [type locality: Turkey, Ankara, Beypazari, Urus, Kirmir Çayi; depository not designated; ♂]. —Malicky 2004a: 56 [atlas]. —Malicky 2005b: 544 [checklist]. —Sipahiler 2005: 397 [distribution].

**Distribution.** —Turkey.

***ernstreichli*** Malicky, 1998b: 395 [type locality: [Uzbekistan], Turapsaj, 1500–1700 m, 38°32'N, 67°31'E; Collection Malicky; ♂]. —Malicky 2004a: 62 [atlas]. —Malicky 2005b: 544 [checklist].

**Distribution.** —Uzbekistan.

***explicata*** Wells, 1984: 264 [type locality: [Papua] New Guinea, SE., Kokoda, 400 m; BPBM; ♂]. —Wells 1990b: 382 [as junior synonym of *H.obscura*]. —Neboiss 1986: 61 [atlas; ♂]. —Oláh 2016: 111 [diagnosed as distinct species; ♂]. —Oláh and Kovács 2018: 179 [distribution].

**Distribution.** —Indonesia, Papua New Guinea.

***extrema*** Kumanski, 1990: 50 [type locality: Korea, Province Phyongan pukdo (Northern Phyongan) Myohyang Mts., the foothills, the hotel; SOFM; ♂; ♀]. —Yang et al. 2005: 458 [checklist]. —Yang et al. 2016: 476 [checklist]. —Park and Kong 2020: 297 [checklist].

**Distribution.** —Korea.

***felfela*** Oláh & Johanson, 2011: 119 [type locality: Mexico, State of Veracruz, Los Manantioles, Tlilapan, 18°47.944'N 097°06.270'W, 1171 m; NHRS; ♂].

**Distribution.** —Mexico.

***fiorii*** Malicky & Moretti, 1987: 193 [type locality: [Italy], Sardinien, Domusdemaria; depository not designated; ♂]. —Malicky 2004a: 53 [atlas]. —Malicky 2005b: 544 [checklist]. —Cianficconi et al. 2007a: 67 [proposed as Italian endemic]. —Corallini et al. 2013a: 38 [checklist]. —Cianficconi et al. 2016: 139 [distribution].

**Distribution.** —Italy.

***fiskei*** Blickle, 1963: 19 [type locality: [United States], Maine, Dennistown; INHS; ♂]. —Blickle 1979: 48, 63 [checklist; ♂]. —Parker and Voshell 1981: 4 [checklist]. —Morse et al. 1989: 22 [distribution]. —Masteller and Flint 1992: 70 [checklist]. —Huryn and Harris 2000: 193 [distribution]. —DeWalt and Heinold 2005: 41 [phenology; distribution]. —Myers et al. 2011: 106 [distribution]. —Houghton 2020: 2 [distribution].

**Distribution.** —U.S.A.

***flinti*** Bueno-Soria, 1984: 107 [type locality: Costa Rica, Turrialba; NMNH; ♂]. —Holzenthal 1988: 61 [distribution]. —Harris and Holzenthal 1999: 38 [♂; distribution]. —Armitage et al. 2015b: 5 [distribution]. —Armitage et al. 2015a: 6 [checklist]. —Armitage and Harris 2018b: 97 [checklist]. —Armitage and Harris 2018c: 283 [distribution]. —Harris and Armitage 2019: 4 [distribution].

**Distribution.** —Costa Rica, Panama.

***florestani*** de Souza, Santos, & Takiya, 2014b: 640 [type locality: Brazil, Piauí, Parque Nacional de Sete Cidades, Riacho Piedade, 04°06'34"S 41°43'39"W, 169 m; CZMA; ♂].

**Distribution.** —Brazil.

***fonsorontina*** Botosaneanu & Moubayedin Moubayed and Botosaneanu 1985: 64 [type locality: [Lebanon], Liban, Hermel, l’Oronte en aval de la source Zarka - une source principale de cette rivière, 650 m; ZMUA; ♂; ♀]. —Botosaneanu 1992: 73 [♂; ♀]. —Malicky 2004a: 61 [atlas; as *fonsorentina*]. —Malicky 2005b: 544 [checklist; as *fonsorentina*]. —Dia 2015: 51 [distribution].

**Distribution.** —Lebanon.

***forcipata*** (Eaton, 1873): 135 [type locality: [England], Oakamoor, Staffordshire, and the River Dove, near Norbury and Ashbourne, Derbyshire; NHMUK; ♂; in *Phrixocoma*]. —McLachlan 1880: 513 [revision; ♂; ♀]. —Morton 1899a: 54 [distribution]. —Morton 1899b: 281 [distribution]. —Klapálek 1900b: 3 [distribution]. —Martynov 1913b: 11 [♂]. —Mosely 1919a: 395 [scent-organ]. —Martynov 1924: 44 [♂]. —Tjeder 1930b: 201 [distribution]. —Martynov 1934: 137 [♂]. —Mosely 1939b: 270 [♂]. —Kimmins 1943: 154 [distribution]. —Kimmins 1957a: 109 [lectotype designation]. —Nybom 1960: 18 [checklist]. —Botosaneanu 1967: 294 [distribution]. —Spuris 1972: 21, 27, 28, 30 [checklist]. —Fahy 1972: 202 [distribution]. —Malicky 1974: 122 [checklist]. —Szczęsny 1975: 41 [distribution]. —Botosaneanu and Malicky 1978: 341 [checklist]. —Kumanski 1979: 12 [♂; distribution]. —Moretti and Cianficconi 1981: 201 [checklist]. —Malicky 1983b: 45, 52 [atlas; ♂; ♀]. —Kumanski and Malicky 1984: 199 [distribution]. —Kumanski 1985: 137 [♂]. —Wiberg-Larsen 1985: 40 [checklist]. —Nógrádi 1986: 137 [distribution; ♀]. —Sipahiler and Malicky 1987: 84, 136 [♂; distribution]. —Andersen and Wiberg-Larsen 1987: 168 [checklist]. —Cooter 1987: 148 [distribution]. —Usseglio-Polatera and Bournaud 1989: 253 [distribution]. —Spuris 1989: 16 [checklist]. —Krušnik 1991: 13 [distribution]. —Andersen et al. 1993b: 3 [distribution]. —Andersen et al. 1993a: 51 [distribution]. —Bagge 1995: 93 [distribution; biology]. —Uherkovich and Nógrádi 1997: 461 [distribution]. —Uherkovich and Nógrádi 1998: 52 [distribution]. —Nógrádi and Uherkovich 1998: 338 [distribution]. —Peissner and Kappus 1998: 162 [distribution]. —Malicky 1999c: 96 [distribution]. —Uherkovich and Nógrádi 1999: 420 [distribution]. —Cianficconi et al. 1999a: 57 [distribution]. —Nógrádi and Uherkovich 2001: 297 [checklist]. —Nógrádi and Uherkovich 2002: 130 [distribution]. —Cibaitė 2003a: 10 [checklist]. —Graf and Hutter 2004: 147 [distribution]. —Graf et al. 2005: 55 [distribution]. —Gullefors 2005a: 119 [distribution]. —Sipahiler 2005: 397 [distribution]. —Uherkovich and Nógrádi 2001: 95 [distribution]. —Gullefors 2002: 138 [checklist]. —Ujvárosi 2002: 384 [distribution]. —Malicky 2004a: 54, 64 [atlas]. —Malicky 2005a: 60 [distribution]. —Malicky 2005b: 544 [checklist]. —Lubini-Ferlin and Vicentini 2005: 67 [checklist]. —Wiggers et al. 2006: 54 [distribution]. —Mey 2006a: 159 [distribution]. —Chvojka and Komzák 2006: 358 [distribution]. —Schiess-Bühler and Rezbanyai-Reser 2006: 72 [distribution]. —Ivanov and Melnitsky 2007: 32 [distribution]. —Robert 2007: 82 [checklist]. —Cianficconi et al. 2007b: 569, 575 [distribution]. —Sipahiler 2007: 38 [distribution]. —Chvojka and Komzák 2008: 13 [distribution]. —Ujvárosi et al. 2008: 112 [checklist]. —Schrankel et al. 2008: 90 [checklist]. —Szczęsny and Godunko 2008: 15 [checklist]. —Flint and Thomas 2008: 40 [distribution]. —Višinskienė 2009: 27 [checklist]. —Hohmann 2010: 40 [distribution]. —Ivanov 2011: 195 [checklist]. —González and Menéndez 2011: 119 [distribution]. —Viidalepp et al. 2011: 196 [distribution]. —Komzák and Chvojka 2012: 719 [distribution]. —Wolf et al. 2012: 75 [distribution]. —Andersen and Hagenlund 2012: 135 [distribution]. —Ibrahimi et al. 2012: 76 [distribution]. —Corallini et al. 2013a: 38 [checklist]. —Corallini et al. 2013b: 26 [distribution]. —O’Connor 2013: 64 [distribution]. —O’Connor and O’Connor 2014: 273 [distribution]. —Hohmann et al. 2014: 85 [distribution]. —O’Connor and O’Connor 2015: 203 [distribution]. —Stanić-Kroštroman et al. 2015: 85 [distribution]. —Stanić-Koštroman et al. 2015: 85 [distribution]. —O’Connor 2015: 28, 80 [distribution]. —Karaouzas and Malicky 2015: 14 [distribution]. —Cianficconi et al. 2016: 139 [distribution]. —Pan’kov and Krasheninnikov 2016: 333 [distribution]. —Küttner et al. 2016: 179 [distribution]. —Sipahiler 2016: 15 [checklist]. —Gullefors 2016: 155 [checklist]. —O’Connor and O’Connor 2017b: 53 [distribution]. —O’Connor and O’Connor 2018: 81 [distribution]. —Lock and van Butsel 2018: 3 [distribution]. —Kučinić et al. 2019: 450 [distribution]. —Cerjanec et al. 2020: 13 [distribution]. —Kroča and Komzák 2020: 147 [distribution]. —O’Connor 2020: 140 [distribution]. —Navara et al. 2020: 46 [distribution].

**Distribution.** —Austria, Bosnia-Herzegovina, Bulgaria, Croatia, Czech Republic, Denmark, England, Estonia, Finland, France, Germany, Greece, Hungary, Ireland, Italy, Luxembourg, Netherlands, Norway, Poland, Republic of Kosovo, Serbia, Romania, Russia, Scotland, Slovakia, Slovenia, Spain, Sweden, Switzerland, Turkey, Ukraine.

***fortunata*** Morton, 1893: 76 [type locality: [Spain, Canary Islands]; NHMUK; ♂]. —Nybom 1948: 5 [distribution; transferred to *Hydropneuma*; *H.juba* considered junior synonym]. —Nybom 1954: 2 [distribution; in *Hydropneuma*]. —Kimmins 1957a: 107 [lectotype designation]. —Nybom 1963: 114 [distribution]. —Nybom 1965: 89 [distribution]. —Botosaneanu 1967: 294 [distribution]. —Botosaneanu and Malicky 1978: 341 [checklist]. —Malicky 1981a: 183 [♂]. —Malicky 1983b: 44 [atlas; ♂]. —Botosaneanu and Dumont 1987: 115 [♂]. —Malicky 1988a: 23 [considered species distinct from *H.juba*] —Botosaneanu 1993b: 160 [distribution]. —Botosaneanu 2003: 107 [considered distinct from *H.juba*; *H.espada* considered junior synonym]. —Malicky 2004a: 53 [atlas]. —Malicky 2005b: 544 [checklist]. —Hughes 2006: 29 [biology].

**Distribution.** —Portugal, Spain.

***fowlesi*** Harris & Sykora, 1996: 19 [type locality: [United States], West Virginia, Lewis County, Right Fork of the West Fork River, Walkersville; CMNH; ♂]. —Armitage et al. 2011: 14 [checklist].

**Distribution.** —U.S.A.

***friedeli*** Malicky, 1972: 31 [type locality: [Turkey], Asia minor, 20 km westlich von Kizilcahamam, 1400 m; Collection Malicky; ♂]. —Malicky 1983b: 47 [atlas; ♂]. —Sipahiler and Malicky 1987: 122 [distribution]. —Malicky 1997: 143 [♂]. —Malicky 2004a: 59 [atlas]. —Malicky 2005b: 544 [checklist]. —Sipahiler 2005: 397 [distribution; as *friedli*]. —Sipahiler 2007: 38 [distribution].

**Distribution.** —Turkey

***fuentaldeala*** Schmid, 1952: 650 [type locality: Spain; CNC; ♂]. —Botosaneanu 1967: 294 [distribution]. —Botosaneanu and Malicky 1978: 341 [checklist]. —Malicky 1983b: 42 [atlas; ♂]. —González et al. 1986: 113[distribution]. —González et al. 1990: 212 [checklist]. —Malicky 2004a: 51 [atlas]. —Malicky 2005b: 544 [checklist]. —González and Menéndez 2011: 119 [distribution]. —Martín et al. 2014: 72 [distribution]. —Ruiz-García et al. 2016: 4 [distribution]. —Lonsdale 2020: 34 [holotype depository].

**Distribution.** —Portugal, Spain.

***fuentelarbola*** Schmid, 1952: 651 [type locality: Spain; CNC; ♂]. —Botosaneanu 1967: 294 [distribution]. —Botosaneanu and Malicky 1978: 341 [checklist]. —Malicky 1983b: 48 [atlas; ♂]. —Malicky 2004a: 60 [atlas]. —Malicky 2005b: 544 [checklist]. —González and Menéndez 2011: 119 [distribution]. —Lonsdale 2020: 35 [holotype depository].

**Distribution.** —Spain.

***furcata*** (Martynov, 1935): 114 [type locality: [India], above Kapildhara Fall, Rewah State, C. I.; NZSI; ♂; in *Oxydroptila*]. —Schmid 1958b: 66 [♂; distribution].

**Distribution.** —India, Sri Lanka.

***furcilla*** Yang & Xue, 1994: 10 [type locality: [China], Anhui, Jin-xian, Song-cun, Ding-xi River, 33 km E of Jin-xian, 120 m; NAUJ; ♂]. —Yang et al. 1997b: 93 [checklist]. —Yang et al. 2005: 458 [checklist]. —Yang et al. 2016: 476 [checklist].

**Distribution.** —China.

***furcula*** Wells, 1984: 269 [type locality: [Papua] New Guinea, SE., Kokoda, 400 m; BPBM; ♂]. —Wells 1991: 501 [distribution].

**Distribution.** —Papua New Guinea.

***furtiva*** Bueno-Soria, 1984: 104 [type locality: Mexico, Oaxaca, Puerta de Uxpanapa; CNIN; ♂]. —Barba-Álvarez et al. 2019: 85 [distribution].

**Distribution.** —Mexico.

***fuscina*** Harris, 1985b: 611 [type locality: [United States], Alabama, Tuscaloosa County, Turkey Creek at Hwy. 69; NMNH; ♂]. —Harris et al. 1991: 181 [distribution].

**Distribution.** —U.S.A.

***gandhara*** Schmid, 1960: 94 [type locality: [Pakistan] Himalaya, Naran; CNC; ♂]. —Schmid 1958c: 220 [as new species, *nomen nudum*]. —Malicky 1983b: 48 [atlas; ♂]. —Malicky 2004a: 62 [atlas]. —Malicky 2005b: 544 [checklist]. —Lonsdale 2020: 35 [holotype depository].

**Distribution.** —Pakistan.

***gapdoi*** Oláh, 1989: 283 [type locality: Vietnam, Tamdao, 200 m a.s.l.; HNHM; ♂]. —Wells and Malicky 1997: 183 [distribution]. —Armitage et al. 2005: 27 [checklist]. —Malicky and Chantaramongkol 2007: 1023 [distribution]. —Malicky 2007a: 177 [checklist]. —Oláh and Johanson 2010a: 14 [distribution]. —Malicky 2010a: 30 [atlas; ♂]. —Yang et al. 2016: 476 [checklist].

—*acrodonta* Xue & Yang, 1990: 126 [type locality: [China], Bawangling (320 m), Hainan; NAUJ; ♂]. —Yang et al. 1997b: 93 [checklist]. —Wells and Malicky 1997: 183 [to synonymy]. —Yang et al. 2005: 458 [checklist]. —Malicky and Chantaramongkol 2007: 1023 [as synonym]. —Malicky 2013: 42 [as synonym].

**Distribution.** —China, India, Indonesia, Thailand, Vietnam.

***gaya*** Oláh, 1989: 278 [type locality: Vietnam, Tamdao, 200 m a.s.l.; HNHM; ♂]. —Armitage et al. 2005: 27 [checklist]. —Malicky and Chantaramongkol 2007: 1023 [♂; distribution]. —Malicky 2010a: 32 [atlas; ♂].

**Distribution.** —Thailand, Vietnam.

***geniel*** Malicky, 2014a: 1610 [type locality: Taiwan, Prov. Pingtung, Huang-Lion, Forest recr. area; Collection Malicky; ♂].

**Distribution.** —Taiwan.

***giama*** Oláh, 1989: 285 [type locality: Vietnam, Tamdao, 1300 m a.s.l.; HNHM; ♂]. —Armitage et al. 2005: 27 [checklist]. —Mey 2005a: 280 [distribution]. —Yang et al. 2005: 458 [checklist]. —Malicky and Chantaramongkol 2007: 1023 [distribution]. —Oláh and Johanson 2010a: 15 [distribution]. —Malicky 2010a: 33 [atlas; ♂]. —Yang et al. 2016: 476 [checklist]. —Park and Kong 2020: 297 [checklist].

—*hubenovi* Kumanski, 1990: 54 [type locality: Korea, Province Kangvon, Kumgang Mts., the foothills near the hotel Go-sung and Ondžong vill.; SOFM; ♂; ♀]. —Malicky 2013: 42 [to synonymy].

**Distribution.** —China, Korea, Laos, Vietnam.

***gingoog*** Wells & Mey, 2002: 128 [type locality: [Philippines] Mindanao, Misamis Or., Dinawihan, Gingoog, 26 km E of Gingoog City, 100–300 m; BPBM; ♂].

**Distribution.** —Philippines.

***giudicellorum*** Botosaneanu, 1980: 167 [type locality: France, Provence, le complexe de sources et de ruisselets de 1’ “Etang du Comte”, à ca. 8 km N de St. Martin de Crau; ZMUA; ♂]. —Botosaneanu 1982b: 31 [habitat threat]. —González and Otero 1983: 118 [distribution]. —Malicky 1983b: 43 [atlas; ♂]. —Malicky and Lounaci 1987: 15, 17 [checklist]. —Cianficconi et al. 1999b: 278 [distribution]. —Valle 2001: 66 [distribution]. —Botosaneanu and Giudicelli 2004: 15 [♀; distribution]. —Malicky 2004a: 52 [atlas]. —Malicky 2005b: 544 [checklist]. —González and Menéndez 2011: 119 [distribution]. —Corallini and Cianficconi 2011: 628 [checklist]. —Corallini et al. 2013a: 38 [checklist]. —Cianficconi et al. 2016: 140 [distribution].

**Distribution.** —France, Italy, Portugal, Spain.

***grandiosa*** Ross, 1938a: 126 [type locality: [United States], Illinois, Oakwood, along Salt Fork River; INHS; ♂]. —Ross 1944: 151 [♂; ♀; larva; distribution]. —Denning 1947b: 174 [distribution]. —Etnier 1965: 147 [distribution]. —Unzicker et al. 1970: 172 [distribution]. —Edwards 1973: 506 [distribution]. —Blickle 1979: 48, 67 [checklist; ♂]. —Parker and Voshell 1981: 4 [distribution]. —Harris et al. 1982a: 510 [distribution]. —Huryn and Foote 1983: 790 [distribution]. —Waltz and McCafferty 1983a: 10 [distribution]. —Hamilton et al. 1983: 18 [distribution]. —Steven and Hilsenhoff 1984: 163 [distribution]. —Bowles and Mathis 1989: 239 [distribution]. —Tarter 1990: 239 [distribution]. —Harris et al. 1991: 182 [distribution]. —Floyd 1992: 50 [distribution]. —Masteller and Flint 1992: 70 [distribution]. —Mathis and Bowles 1992: 24 [distribution]. —Bowles and Mathis 1992: 32 [distribution]. —Moulton and Stewart 1996: 99 [♂; distribution]. —Abbott et al. 1997: 44 [distribution]. —Moulton and Stewart 1997: 350 [checklist]. —Houghton et al. 2001: 504 [distribution]. —DeWalt and Heinold 2005: 41 [phenology; distribution]. —Biondi 2010: 61 [distribution]. —Armitage et al. 2011: 14 [checklist]. —Houghton et al. 2011b: 5 [phenology; habitat; distribution]. —Wright et al. 2013: 466 [biology; distribution]. —DeWalt et al. 2016: 51 [distribution]. —Houghton 2016: 46 [biology]. —Houghton et al. 2017: 62 [checklist]. —Bowles et al. 2020: 7 [distribution].

**Distribution.** —U.S.A.

***grenadensis*** Flint, 1968a: 58 [type locality: Grenada, 2 mi W Grand Etang; NMNH; ♂; ♀]. —Flint and Reyes 1991: 484 [distribution]. —Flint and Sykora 1993: 57 [distribution]. —Flint 1996b: 97 [distribution]. —Harris and Holzenthal 1999: 27 [♂]. —Maes 1999: 1193 [checklist]. —Muñoz-Quesada 2000: 277 [checklist]. —Botosaneanu 2002b: 83 [checklist]. —Chamorro-Lacayo et al. 2007: 43 [checklist]. —Oláh and Johanson 2011: 120 [distribution]. —Armitage et al. 2015a: 6 [checklist]. —Ríos-Touma et al. 2017: 10 [checklist]. —Armitage and Harris 2018b: 97 [checklist]. —Armitage and Harris 2018c: 283 [distribution].

—*acutissima* Botosaneanu in Botosaneanu and Alkins-Koo 1993: 24 [type locality: Trinidad, upper course of River Guanapo (3^rd^ order and upper part of 4^th^ order stream); ZUA; ♂; ♀]. —Botosaneanu and Sakal 1992: 202 [distribution; ecology]. —Flint 1996b: 97 [to synonymy].

**Distribution.** —Colombia, Ecuador, Grenada, Nicaragua, Panama, Peru, Tobago, Trinidad, Venezuela.

***grucheti*** Marlier & Marlier, 1982: 12 [type locality: La Réunion, Station 53, Rivière Langevin, à la lumière; IRSNB; ♂; ♀; larva]. —Botosaneanu 2002a: 326 [♂].

**Distribution.** —Réunion.

***gunda*** Milne, 1936: 76 [type locality: [United States], Virginia, Falls Church; MCZ; ♂]. —Morse and Blickle 1953: 72 [checklist]. —Roy and Harper 1979: 151 [checklist]. —Blickle 1979: 48, 67 [checklist; ♂]. —Parker and Voshell 1981: 4 [checklist]. —Harris et al. 1982a: 510 [distribution]. —Waltz and McCafferty 1983a: 10 [distribution]. —Harris et al. 1984: 108 [distribution]. —Morse et al. 1989: 22 [distribution]. —Usis and Foote 1989: 84 [distribution]. —Floyd and Schuster 1990: 130, 132 [distribution]. —Harris et al. 1991: 183 [distribution]. —Frazer et al. 1991: 19 [distribution]. —Masteller and Flint 1992: 70 [checklist]. —Floyd and Morse 1993: 177 [distribution]. —Floyd et al. 1997: 136 [distribution]. —Huryn and Harris 2000: 193 [distribution]. —Flint 2011: 104 [distribution]. —Armitage et al. 2011: 14 [checklist]. —Harris et al. 2012: 6 [distribution]. —Denson et al. 2016: 5 [distribution].

—*dodgei* Denning, 1947a: 19 [type locality: [United States], Georgia, Macon; ESUW; ♂]. —Blickle 1979: 48 [to synonymy].

**Distribution.** —Canada, U.S.A.

***gurdi*** Wells, 1990b: 379 [type locality: [Indonesia] Sulawesi Utara, Dumoga-Bone N.P., Tumpah R. and tributary junction; NMV; ♂; ♀; case]. —Malicky et al. 2010: 163 [distribution].

**Distribution.** —Indonesia.

***halus*** Wells & Huisman, 1992: 104 [type locality: East Malaysia, Sabah, Bundu Tuhan, Sg. Laidan, 05°58'N 116°31'E, 950 m; RMNH; ♂]. —Malicky 2010a: 34 [atlas; ♂].

**Distribution.** —East Malaysia.

***hamata*** Morton, 1905: 67 [type locality: [United States], New York, Ithaca; depository not designated; ♂]. —Banks 1907a: 50 [catalogue]. —Mosely 1923: 293 [scent-organ]. —Betten 1934: 159 [distribution]. —Ross 1944: 149 [♂; larva; distribution]. —Denning 1947b: 173 [distribution]. —Morse and Blickle 1953: 72 [checklist]. —Etnier 1965: 147 [checklist]. —Edwards 1973: 506 [distribution]. —Bueno-Soria and Flint 1978: 201 [distribution]. —Resh et al. 1978: 383 [distribution]. —Roy and Harper 1979: 151 [checklist]. —Etnier and Schuster 1979: 17 [distribution]. —Blickle 1979: 48, 63 [checklist; ♂]. —Parker and Voshell 1981: 4 [checklist]. —Roy and Harper 1981: 105 [distribution]. —Harris et al. 1982a: 510 [distribution]. —Harris et al. 1982b: 81 [distribution]. —Huryn and Foote 1983: 790 [distribution]. —Waltz and McCafferty 1983a: 10 [distribution]. —Harris et al. 1984: 108 [distribution]. —Bueno-Soria 1984: 89 [distribution]. —Steven and Hilsenhoff 1984: 163 [distribution]. —Bowles and Mathis 1989: 239 [distribution]. —Morse et al. 1989: 22 [distribution]. —Floyd and Schuster 1990: 130, 132 [distribution]. —Tarter 1990: 239 [checklist]. —Harris et al. 1991: 184 [distribution]. —Frazer et al. 1991: 19 [distribution]. —Masteller and Flint 1992: 70 [checklist]. —Mathis and Bowles 1992: 24 [distribution]. —Bowles and Mathis 1992: 32 [distribution]. —Moulton and Stewart 1996: 99 [♂; distribution]. —Abbott et al. 1997: 44 [distribution]. —Moulton and Stewart 1997: 350 [checklist]. —Floyd et al. 1997: 136 [distribution]. USA —Huryn and Harris 2000: 193 [distribution]. —Houghton 2001: 90 [distribution]. —Houghton et al. 2001: 504 [distribution]. —Pescador et al. 2004: 133 [checklist]. —Blinn and Ruiter 2005: 68 [biology; distribution]. —DeWalt and Heinold 2005: 42 [phenology; distribution]. —Blinn and Ruiter 2006: 332 [biology; distribution]. —Zeullig et al. 2006: 43 [distribution]. —Bowles et al. 2007: 21 [distribution; biology]. —Bueno-Soria et al. 2007: 33 [distribution]. —Blinn and Ruiter 2009a: 303 [biology]. —Blinn and Ruiter 2009b: 186 [phenology; distribution]. —Houghton et al. 2011b: 5 [biology; distribution]. —Myers et al. 2011: 106 [distribution]. —Armitage et al. 2011: 14 [checklist]. —Harris et al. 2012: 6 [checklist]. —Blinn and Ruiter 2013: 280, 291 [biology; distribution]. —Denson et al. 2016: 5 [distribution]. —Houghton et al. 2017: 62 [checklist]. —Mendez et al. 2019: 118 [checklist]. —Bowles et al. 2020: 7 [distribution].

**Distribution.** —Canada, Mexico, U.S.A.

***hamiltoni*** Harris, 2002: 54 [type locality: [United States], Florida, Okaloosa County, Rogue Creek, 0.6 km S Base Rd. 232, Eglin Air Force Base, 30°33'19"N, 86°34'52"W; NMNH; ♂]. —Pescador et al. 2004: 133 [checklist]. —Harris et al. 2012: 6 [checklist].

**Distribution.** —U.S.A.

***hamistyla*** Xue & Wang, 1995: 208 [type locality: [China], Baotianman, Henan Province; HAUZ; ♂]. —Yang et al. 1997b: 93 [checklist]. —Yang et al. 2005: 458 [checklist]. —Yang et al. 2016: 476 [checklist].

**Distribution.** —China.

***harpagula*** Mey, 1998a: 555 [type locality: [Philippines, Mindanao], northern slope of Mt. Atuuganon range, 1050 m; ZMHB; ♂]. —Wells and Mey 2002: 134 [checklist].

**Distribution.** —Philippines.

***harpeodes*** Yang & Xue, 1994: 9 [type locality: [China], Fujian, Cong-an City, 29 km N of Cong-an, 408 km marker; NAUJ; ♂]. —Yang et al. 1997b: 93 [checklist]. —Yang et al. 2005: 458 [checklist]. —Yang et al. 2016: 476 [checklist].

**Distribution.** —China.

***helicina*** Flint, 1991b: 49 [type locality: Colombia, Depto. Antioquia, Quebrada Espadera, 7 km E Medellín, road to Sta. Elena; NMNH; ♂; ♀]. —Muñoz-Quesada 2000: 277 [checklist].

**Distribution.** —Colombia.

***helmali*** Chantaramongkol & Malicky, 1986: 515 [type locality: [Sri Lanka], Sabaraganuwa Province, Kitulgala, 21 mi N von Ratnapura, 60–150 m; MZLU; ♂].

**Distribution.** —Sri Lanka.

***hirsuta*** Wells & Mey, 2002: 126 [type locality: [Philippines] Misamis Or., Dinawihan, Gingoog, 26 km E of Gingoog City, 100–300 m; BPBM; ♂].

**Distribution.** —Philippines.

***hochyangha*** Schmid, 1959b: 692 [type locality: Iran, Firouzkuh (Ost. 2); CNC; ♂]. —Malicky 1983b: 47 [atlas; ♂]. —Malicky 1997: 143 [distribution; ♂]. —Mirmoayedi and Malicky 2002: 164 [checklist]. —Malicky 2004a: 59 [atlas]. —Malicky 2005b: 544 [checklist]. —Lonsdale 2020: 35 [holotype depository].

**Distribution.** —Iran.

***hodkovae*** Chvojka, 2006: 246 [type locality: Iran, Khuzestan prov., 10 km SW Izeh, 31°45'N 49°48'E, 880 m a.s.l.; NMPC; ♂]. —Malicky 2007b: 51 [checklist].

**Distribution.** —Iran.

***hoffmannae*** Bueno-Soria & Santiago-Fragoso, 1996: 345 [type locality: Mexico, Veracruz, Los Tuxtlas, Arroyo Tebanca, 15 k SE La Estación de Biología Los Tuxtlas; CNIN; ♂].

**Distribution.** —Mexico.

***holzenthali*** Sykora & Harris, 1994: 73 [type locality: [United States], Mississippi, Stone Co., Flint Creek, Hwy 26, 7.9 km E Wiggins; CUAC; ♂].

**Distribution.** —U.S.A.

***homochitta*** Harris & Sykora, 1996: 21 [type locality: [United States], Mississippi, Franklin County, Porter Creek (T5N, R4E, S8NW); CMNH; ♂].

**Distribution.** —U.S.A.

***hossa*** Oláh & Johanson, 2011: 121 [type locality: Peru, San Martin Prov., stream crossing Juan Guerra-Chazuta rd., 10 km (rd.) W Chazuta, 6°37.157'S 76°10.905'W; NHRS; ♂].

**Distribution.** —Peru.

***howelli*** Houp, Houp, & Harris, 1998: 99 [type locality: [United States], Kentucky, LaRue-Marion County line, Salt Lick Creek on Salt Lick Road; NMNH; ♂].

**Distribution.** —U.S.A.

***huaivat*** Malicky, Suwannarat, & Laudee, 2018: 1319 [type locality: Thailand, Huai Vat (Nebenbach des Klong Kay) bei Ban Pak Lang, nahe der Grenze zum Kao Nan Nationalpark, 8°47'N, 99°35'E, 140 m; Collection Malicky; ♂].

**Distribution.** —Thailand.

***hyllos*** Malicky, 2004b: 292 [type locality: [Nepal, Bardia National Park], am Rande der nordindischen Ebene im Südwesten von Nepal im Bereich des ersten Hügelkammes des Himalaya (Siwalik Range), unweit des Wehrs des Babai Flusses, über das die Brücke der Ost-West-Haupstraße Nepals (Mahindra Highway), 28°25'N, 81°23'E, 190 m; Collection Malicky; ♂]. —Malicky 2006: 252 [checklist]. —Mattern 2015: 500 [distribution].

**Distribution.** —Nepal.

***icona*** Mosely, 1937b: 161 [type locality: Mexico, Chiapas, Dolores; NHMUK; ♂]. —Edwards 1973: 506 [distribution]. —Cloud and Stewart 1974: 806 [biology; distribution]. —Bueno-Soria and Flint 1978: 201 [distribution]. —Resh et al. 1978: 383 [distribution]. —Blickle 1979: 48, 71 [checklist; ♂]. —Unzicker et al. 1982: 9, 13 [checklist]. —Bueno-Soria 1984: 110 [♂; distribution]. —Bowles and Mathis 1992: 32 [distribution]. —Moulton et al. 1993: 21 [distribution]. —Moulton et al. 1994: 169 [distribution]. —Moulton and Stewart 1996: 100 [♂; distribution]. —Moulton and Stewart 1997: 350 [checklist]. —Houghton and Stewart 1998: 106 [biology; distribution]. —Harris and Holzenthal 1999: 38 [♂; distribution]. —Maes 1999: 1193 [checklist]. —Flint et al. 2003: 33 [♀; distribution; introduced to Hawaii]. —Baumgardner and Bowles 2005: 11 [distribution]. —Blinn and Ruiter 2005: 68 [biology; distribution]. —Blinn and Ruiter 2006: 332 [biology; distribution]. —Bowles et al. 2007: 21 [distribution; biology]. —Bueno-Soria et al. 2007: 33 [distribution]. —Chamorro-Lacayo et al. 2007: 43 [checklist]. —Blinn and Ruiter 2009a: 305 [biology]. —Blinn and Ruiter 2009b: 186 [phenology; distribution]. —Harris et al. 2012: 6 [♂; distribution]. —Barba-Álvarez et al. 2019: 85 [distribution]. —Mendez et al. 2019: 118 [checklist].

**Distribution.** —Costa Rica, Honduras, Mexico, Nicaragua, U.S.A.

***idefix*** Malicky, 1979: 6 [type locality: Portugal, Foz do Alva; Collection Malicky; ♂]. —Malicky 1983b: 42 [atlas; ♂]. —González et al. 1986: 113 [distribution]. —Malicky 2004a: 51 [atlas]. —Malicky 2005b: 544 [checklist]. —González and Menéndez 2011: 119 [distribution]. —Martín et al. 2016: 261 [distribution].

**Distribution.** —Portugal, Spain.

***incertula*** Mosely, 1934a: 145 [type locality: [Australia, Queensland] Brisbane; Collection Tillyard (transferred to NHMUK according to Wells, 1978: 761); ♂]. —Mosely and Kimmins 1953: 507 [♂]. —Wells 1978: 761 [♂]. —Wells 1978: 761 [♂]. —Wells 1984: 269 [distribution]. —Wells 1985b: 7 [case]. —Neboiss 1986: 61 [atlas; ♂]. —Wells 1990b: 385 [♂; ♀; case; distribution]. —Wells 1991: 501 [distribution]. —Malicky et al. 2010: 163 [distribution]. —Malicky 2010a: 27 [atlas; ♂]. —Malicky et al. 2014b: 832 [distribution]. —Wells et al. 2019: 33 [detection frequency].

**Distribution.** —Australia, Indonesia, Papua New Guinea, Philippines, Vanuatu.

***inornata*** Flint, 1991b: 47 [type locality: Colombia, Dpto. Antioquia, Quebrada Espadera, 7 km E Medellín, road to Sta. Elena; NMNH; ♂; ♀]. —Muñoz-Quesada 2000: 277 [checklist].

**Distribution.** —Colombia.

***insubrica*** Ris, 1903: 16 [type locality: [Switzerland], Kantons Tessin, Mendrisio; depository not designated; ♂]. —Botosaneanu 1967: 294 [distribution]. —Botosaneanu and Malicky 1978: 341 [checklist]. —Moretti and Cianficconi 1981: 201 [checklist]. —Moretti et al. 1981: 350, 354 [biology; distribution]. —Malicky 1983b: 45 [atlas; ♂]. —Cianficconi et al. 1999a: 57 [distribution]. —Valle 2001: 66 [distribution]. —Malicky 2004a: 54 [atlas]. —Malicky 2005b: 544 [checklist]. —Lubini-Ferlin and Vicentini 2005: 67 [checklist]. —Cianficconi et al. 2007b: 569, 575 [distribution]. —Robert 2007: 82 [checklist]. —González and Menéndez 2011: 119 [distribution]. —Corallini et al. 2013a: 38 [checklist]. —Cianficconi et al. 2016: 140 [distribution].

**Distribution.** —Germany, Italy, Spain, Switzerland.

***introspinata*** Zhou & Sun in Zhou et al. 2009a: 906, 910 [type locality: [China], Heilongjiang Province, Yichun City, 21°43'N, 128°53'E, Wuyiling, Ximiganhe, 310 m; NAUJ; ♂]. —Malicky 2014a: 1610 [possible junior synonym to *H.spinosa*]. —Yang et al. 2016: 476 [checklist]. —Park et al. 2018: 103 [♂; distribution]. —Ito and Shimura 2019: 30 [♂; ♀; distribution]. —Park and Kong 2020: 297 [checklist].

**Distribution.** —China, Japan, Korea.

***ion*** Malicky, 2004b: 292 [type locality: [Nepal, Bardia National Park], am Rande der nordindischen Ebene im Südwesten von Nepal im Bereich des ersten Hügelkammes des Himalaya (Siwalik Range), bei dem Dorf Babai Basar in der Nähe der Straße von Nepalganj nach Birendranagar, ungefähr 30 km flussaufwärts vom Lager 1 (28°21'N, 81°42'E), lag das Ufer des Babai Nadi in wenigen Metern Entfemung, Kyuban Khola, 460 m; Collection Malicky; ♂]. —Malicky 2006: 252 [checklist]. —Mattern 2015: 500 [distribution].

**Distribution.** —Nepal.

***isabellae*** Gibon, 1987a: 129 [type locality: sur le Niouniourou à Zakpabéri (bassin du Niouniourou (Côte d’Ivoire); MNHN; ♂]. —Kjærandsen and Andersen 1997: 244 [distribution].

**Distribution.** —Côte d’Ivoire, Ghana.

***ivisa*** Malicky, 1972: 30 [type locality: Austria inf., Lunz, Biologische Station; Collection Malicky; ♂]. —Botosaneanu and Malicky 1978: 341 [checklist]. —Kumanski 1979: 12 [♂; distribution]. —Malicky 1983b: 45 [atlas; ♂]. —Kumanski 1985: 139 [♂]. —Cianficconi and Moretti 1987: 670 [distribution]. —Weinzierl and Dorn 1995: 43 [distribution]. —Cianficconi et al. 1999a: 57 [distribution]. —Urbanič 2004: 51 [distribution]. —Malicky 2004a: 54, 64 [atlas]. —Lubini-Ferlin and Vicentini 2005: 67 [checklist]. —Graf et al. 2005: 55 [distribution]. —Malicky 2005b: 544 [checklist]. —Robert 2007: 82 [checklist]. —Szczęsny and Godunko 2008: 15 [checklist]. —Coppa 2010: 23 [distribution]. —Komzák and Kroča 2011: 190 [distribution]. —Corallini et al. 2013a: 38 [checklist]. —Malicky 2014b: 15 [teratological structures]. —Cianficconi et al. 2016: 140 [distribution].

**Distribution.** —Austria, Bulgaria, Czech Republic, France, Germany, Italy, Slovenia, Ukraine.

***jackmanni*** Blickle, 1963: 17 [type locality: [United States], Maine, Dennistown; INHS; ♂]. —Etnier 1965: 147 [distribution]. —Roy and Harper 1979: 151 [checklist]. —Blickle 1979: 48, 67 [checklist; ♂]. —Parker and Voshell 1981: 4 [checklist]. —Swegman et al. 1981: 132 [distribution]. —Huryn and Foote 1983: 790 [distribution]. —Huryn 1983: 93 [♀]. —Waltz and McCafferty 1983a: 10 [distribution]. —Steven and Hilsenhoff 1984: 164 [distribution]. —Masteller and Flint 1992: 70 [checklist]. —Harris et al. 1996: 240 [distribution]. —Houghton et al. 2001: 504 [distribution]. —Houghton et al. 2011b: 5 [distribution; biology]. —Houghton et al. 2011a: 388 [distribution; biology]. —Armitage et al. 2011: 14 [checklist]. —Houghton 2016: 46 [biology]. —Houghton et al. 2017: 62 [checklist].

**Distribution.** —U.S.A.

***jamin*** Malicky, O’Connor, Ashe, & Dowling, 2010: 157 [type locality: Indonesia, Sulawesi, Bogani Nani Wartabone National Park, second waterfall on the Sungai Elok (waterfall stream), 0°36'N 123°54'E; NMID; ♂].

**Distribution.** —Indonesia.

***jaruma*** Wells, 1990b: 388 [type locality: [Indonesia] Sulawesi Utara, Dumoga-Bone N.P., Site 6; NHMUK; ♂].

**Distribution.** —Indonesia.

***jeannae*** Gibon, 1987a: 128 [type locality: [Côte d’Ivoire], sur le Cavally à Taï; MNHN; ♂]. —Kjærandsen and Andersen 1997: 244 [distribution].

**Distribution.** —Côte d’Ivoire, Ghana.

***juba*** Enderlein, 1929: 232 [type locality: [Spain], Tenerife SW, in vorderen Teile des Baranco del Infierno, oberhalb von Adeje, am Rande eines in Felsen eingehauenen Wasserlaufes, dermit niederen Pflanzen bestanden; depository not designated; ♂]. —Nybom 1948: 5 [considered synonym of *H.fortunata*]. —Botosaneanu 1967: 294 [as synonym of *H.fortunata*]. —Malicky 1987: 30 [considered distinct from *H.fortunata*]. —Malicky 1988a: 23 [morphological comparison with *H.fortunata*]. —Botosaneanu 2003: 107 [considered distinct from *H.fortunata*]. —Malicky 2004a: 53 [atlas]. —Malicky 2005b: 544 [checklist]. —Hughes 2006: 29 [biology]. —González and Menéndez 2008: 188 [distribution]. —González and Menéndez 2011: 119 [distribution]. —Ruiz-García et al. 2016: 4 [distribution].

—*espada* Malicky, 1981a: 182 [type locality: Portugal, Porto Espada; Collection Malicky; ♂]. —Malicky 1983b: 44 [atlas; ♂]. —Malicky 1987: 30 [to synonymy]. —Malicky 1988a: 23 [as synonym of *H.juba*]. —Botosaneanu 2003: 107 [as synonym of *H.fortunata*].

**Distribution.** —Portugal, Spain.

***judithae*** Gibon, 1987a: 128 [type locality: [Guinea], sur le Niger en amont de Kissidougou; MNHN; ♂].

**Distribution.** —Guinea.

***juram*** Malicky & Chantaramongkol, 2007: 1012 [type locality: Malaysia, Pahang: Merapoh, Taman Negara, Kuala Juram, 4°38'N 102°07'E, 150 m; Collection Malicky; ♂]. —Malicky 2010a: 27 [atlas; ♂].

**Distribution.** —Malaysia.

***kairos*** Malicky, 2004b: 293 [type locality: [Nepal, Bardia National Park], am Rande der nordindischen Ebene im Südwesten von Nepal im Bereich des ersten Hügelkammes des Himalaya (Siwalik Range), bei dem Dorf Babai Basar in der Nähe der Straße von Nepalganj nach Birendranagar, ungefähr 30 km flussaufwärts vom Lager 1 (28°21'N, 81°42'E), lag das Ufer des Babai Nadi in wenigen Metern Entfemung, vom “östlicher” Bach, 320 m; Collection Malicky; ♂]. —Malicky 2006: 252 [checklist]. —Mattern 2015: 500 [distribution].

**Distribution.** —Nepal.

***kakidaensis*** Nozaki & Tanida, 2007: 246 [type locality: Japan, Kakida, Shimizu-ho, Shizuoka, 35°06'N 138°54'E; CBM-ZI; ♂; ♀]. —Ito et al. 2011: 19 [♂, ♀; distribution]. —Ito 2015: 8 [checklist]. —Tanida and Kuranishi 2016: 70 [checklist].

**Distribution.** —Japan.

***kalchas*** Malicky, 2004b: 293 [type locality: [Nepal, Bardia National Park], am Rande der nordindischen Ebene im Südwesten von Nepal im Bereich des ersten Hügelkammes des Himalaya (Siwalik Range), bei dem Dorf Babai Basar in der Nähe der Straße von Nepalganj nach Birendranagar, ungefähr 30 km flussaufwärts vom Lager 1 (28°21'N, 81°42'E), lag das Ufer des Babai Nadi in wenigen Metern Entfemung, vom “östlicher” Bach, 320 m; Collection Malicky; ♂]. —Malicky 2006: 252 [checklist]. —Malicky and Chantaramongkol 2007: 1023 [checklist]. —Malicky 2010a: 23 [atlas; ♂]. —Mattern 2015: 500 [distribution].

**Distribution.** —Nepal, Thailand.

***kalonichtis*** Malicky, 1972: 30 [type locality: [Greece], Kreta, Kalonichtis; Collection Malicky; ♂]. —Malicky 1974: 122 [checklist]. —Botosaneanu and Malicky 1978: 341 [checklist]. —Kumanski 1979: 14 [♂; distribution]. —Malicky 1981a: 183 [♂]. —Malicky 1983b: 44 [atlas; ♂]. —Kumanski 1985: 127 [♂]. —Sipahiler and Malicky 1987: 129 [distribution]. —Malicky 2004a: 53 [atlas]. —Malicky 2005a: 60 [distribution]. —Malicky 2005b: 544 [checklist; distinct from *H.vichtaspa*]. —Sipahiler 2005: 397 [distribution]. —Karaouzas and Malicky 2015: 14 [distribution]. —Sipahiler 2017b: 12 [distribution]. —Oláh 2017: 137 [distribution].

**Distribution.** —Bulgaria, Greece, Turkey.

***karikatla*** Oláh & Johanson, 2011: 123 [type locality: Peru, San Martin Prov., creek crossing rd. Tarapoto-Yurimaguas, ca. 30 km (rd.) NE Tarapoto, 6°24.904'S 76°18.756'W; NHRS; ♂].

**Distribution.** —Peru.

***karima*** Oláh & Johanson, 2011: 123 [type locality: Peru, Amazonas Prov., river crossing Olmos-Tarapoto rd., 371 km (rd.) E Olmos Desv. Jaén, 5°41.178'S 77°46.421'W; NHRS; ♂].

**Distribution.** —Peru.

***kaschgari*** Mey, 1993: 335 [type locality: China, Xinjiang, Kashi (=Kaschgar), Abzugsgräben der Reisfelder im Süden; ZMHB; ♂]. —Yang et al. 2005: 458 [checklist]. —Huang et al. 2005: 469 [distribution]. —Yang et al. 2016: 476 [checklist].

**Distribution.** —China.

***kebawah*** Wells & Huisman, 1992: 104 [type locality: East Malaysia, Sabah, 20 km NE Ranau, Kg Nalumad, Sg. Mokodou, 06°06'N 116°43'E, 400 m; RMNH; ♂]. —Malicky 2010a: 34 [atlas; ♂].

**Distribution.** —Malaysia.

***keres*** Malicky, 2004b: 293 [type locality: [Nepal, Bardia National Park], am Rande der nordindischen Ebene im Südwesten von Nepal im Bereich des ersten Hügelkammes des Himalaya (Siwalik Range), unweit des Wehrs des Babai Flusses, über das die Brücke der Ost-West-Haupstraße Nepals (Mahindra Highway), 28°25'N, 81°23'E, 190 m, Budhi Khola; Collection Malicky; ♂]. —Malicky 2006: 252 [checklist]. —Malicky and Chantaramongkol 2007: 1023 [checklist]. —Oláh and Johanson 2010a: 15 [♂; distribution]. —Malicky 2010a: 24 [atlas; ♂]. —Mattern 2015: 500 [distribution]. —Bunlue et al. 2012: 15 [distribution]. —Malicky 2018: 49 [checklist]. —Malicky et al. 2018: 1323 [distribution].

**Distribution.** —Laos, Nepal, Thailand.

***khonga*** Oláh & Johanson, 2010a: 16 [type locality: Vietnam, Lamdong Province, Baoloc, Baco stream; Collection Oláh; ♂]. —Malicky 2013: 43 [possible junior synonym to *Hydroptilatrullata*].

**Distribution.** —Vietnam.

***kieneri*** Marlier & Marlier, 1982: 20 [type locality: La Réunion, Station 58, sud de Piton Sainte-Rose, Anse des Cascades, 20–30 m, dans Cascade d’eau très claire et ruisselet; IRSNB; ♂; larva]. —Botosaneanu 2002a: 328 [♂].

**Distribution.** —Réunion.

***kirilawela*** (Schmid, 1958b): 66 [type locality: [Sri Lanka], Ceylan, Kitulgala (Sab., 750 ft) 2-III, Kelani Ganga, belle rivière coulant dans une vallée étroite et boisée, à la sortie des montagnes; depository not designated; ♂; in *Oxydroptila*].

**Distribution.** —Sri Lanka.

***klapperichi*** Malicky, 1996b: 203 [type locality: Jordan, Amman; LNKD; ♂]. —Malicky 2004a: 64 [atlas]. —Malicky 2005b: 544 [checklist].

**Distribution.** —Jordan.

***koropa*** Wells, 1984: 266 [type locality: [Papua] New Guinea, NE., Korop, Upper Jimmi Valley, 1300 m; BPBM; ♂]. —Neboiss 1986: 64 [atlas; ♂]. —Wells 1991: 503 [distribution].

**Distribution.** —Papua New Guinea.

***koryaki*** Harris & Sykora, 1996: 17 [type locality: [United States], West Virginia, Lewis County, Right Fork of the West Fork River, Walkersville; CMNH; ♂]. —Armitage et al. 2011: 14 [checklist]].

**Distribution.** —U.S.A.

***kreusa*** Malicky, 2004b: 294 [type locality: [Nepal, Bardia National Park], am Rande der nordindischen Ebene im Südwesten von Nepal im Bereich des ersten Hügelkammes des Himalaya (Siwalik Range), bei dem Dorf Babai Basar in der Nähe der Straße von Nepalganj nach Birendranagar, ungefähr 30 km flussaufwärts vom Lager 1 (28°21'N, 81°42'E), lag das Ufer des Babai Nadi in wenigen Metern Entfemung, Kyuban Khola, 460 m; Collection Malicky; ♂]. —Malicky 2006: 252 [checklist]. —Mattern 2015: 500 [distribution]. —Malicky 2018: 49 [checklist].

**Distribution.** —Nepal.

***kuehnei*** Houp, Houp, & Harris, 1998: 100 [type locality: [United States], Kentucky, LaRue-Marion County line, Salt Lick Creek on Salt Lick Road; NMNH; ♂].

**Distribution.** —U.S.A.

***kurukepitiya*** Schmid, 1958b: 62 [type locality: [Sri Lanka], Ceylan, Nuwara Eliya (C. P.) 26-II, cours supérieur de la Nanu Oya, petite rivière rapide, sur lit caillouteux; depository not designated; ♂].

**Distribution.** —Sri Lanka.

***lacandona*** Bueno-Soria, 1984: 118 [type locality: Mexico, Chiapas, 10 km from Bonampak; CNIN; ♂]. —Barba-Álvarez et al. 2019: 85 [distribution].

**Distribution.** —Mexico.

***lagoi*** Harris, 1985a: 248 [type locality: [United States], Alabama, Tuscaloosa County, Big Sandy Creek at spring, 4 miles S Coaling, T22N, R7E, S25; NMNH; ♂]. —Harris et al. 1991: 185 [distribution].

**Distribution.** —U.S.A.

***laloka*** Wells, 1991: 503 [type locality: Papua New Guinea, Central Province, Laloki River at Rouna Falls, 9°25'S 147°27'E; ANIC; ♂].

**Distribution.** —Papua New Guinea.

***latifilis*** Zhou & Yang in Zhou et al. 2009b: 355 [type locality: China, Guangxi Zhuang Autonomous Region, Shangsi City, Nalin He, tributary of Mingjiang He, 2.0 km NW of main entrance to Shiwandashan National Forest Park, 21°51'N 107°53'E, 281 m; NAUJ; ♂]. —Yang et al. 2016: 476 [checklist].

**Distribution.** —China.

***latosa*** Ross, 1947: 148 [type locality: [United States], Georgia, Tharpes’ Pond; INHS; ♂]. —Blickle, 1979: 48, 73 [checklist; ♂]. —Harris et al. 1982b: 81 [distribution]. —Harris et al. 1991: 186 [distribution]. —Pescador et al. 2004: 133 [checklist]. —Biondi 2010: 60 [distribution]. —Harris et al. 2012: 6 [♂; checklist]. —Denson et al. 2016: 5 [distribution].

**Distribution.** —U.S.A.

***lennoxi*** Blickle, 1969: 79 [type locality: [United States], Jefferson, New Hampshire; INHS; ♂]. —Blickle 1979: 48, 65[checklist; ♂]. —Harris et al. 1991: 187 [distribution]. —Armitage et al. 2011: 14 [distribution].

**Distribution.** —U.S.A.

***lenora*** Blickle & Denning, 1977: 295 [type locality: [United States], Oregon, Malheur County, Three Forks, 40 miles south of Jordan Valley, on the main branch of the Owyhee River, one mile upriver of the confluence of the 3 branches of the river; FSCA; ♂]. —Blickle 1979: 48, 69 [checklist; ♂].

**Distribution.** —U.S.A.

***leptocera*** Zhou & Yang in Zhou et al. 2009a: 905, 910[type locality: [China], Guangxi Zhuang Autonomous Region, Shangsi City, Nalinhe, Trib of Mingjiang He, 2.0 km NW of main entrance to Shiwandashan National Forest Park, 21°54'N, 107°53'E, 281 m; NAUJ; ♂]. —Yang et al. 2016: 476 [checklist].

**Distribution.** —China.

***libanica*** Botosaneanu & Dia in Dia and Botosaneanu 1983: 130 [type locality: [Lebanon], Station 9, Le Nahr ed Damour à 500 m en aval de son confluent avec Nahr el Hammam, il s’agit des 4 derniers km du cours d’eau, avant qu’il ne se jette à la mer, de 40 à 0 m; ZMUA; ♂]. —Botosaneanu 1992: 71 [♂, ♀]. —Malicky 2004a: 61 [atlas]. —Malicky 2005b: 544 [checklist]. —Dia 2015: 51 [distribution].

**Distribution.** —Lebanon.

***licina*** Frazer & Harris, 1991b: 6 [type locality: [United States], Alabama, DeKalb County, West Fork of the Little River at DeSoto State Park, 50 m downstream mouth of Laurel Creek (Sec. 20, T 6 S, R 10 E); NMNH; ♂]. —Frazer et al. 1991: 19 [distribution].

**Distribution.** —U.S.A.

***lidah*** Wells & Huisman, 1992: 101 [type locality: East Malaysia, Sabah, 12 km NNE Ranau, Poring Hot Springs, Sg. Langanan, 06°03'N 116°43'E, 450 m; RMNH; ♂]. —Malicky 2010a: 34 [atlas; ♂].

**Distribution.** —East Malaysia.

***lingigi*** Mey, 1998b: 4 [type locality: [Philippines], Mindanao, Surigao del Sur, Lingig; ZMHB; ♂].

**Distribution.** —Philippines.

***lloganae*** Blickle, 1961: 131 [type locality: [United States], Chattahoochee, Florida; INHS; ♂]. —Blickle 1979: 48, 69 [checklist; ♂]. —Etnier and Baxter 1999: 147 [♂]. —Pescador et al. 2004: 133 [checklist]. —Harris et al. 2012: 6 [♂; checklist].

—*morsei* Sykora & Harris, 1994: 71 [type locality: [United States], South Carolina, Dorchester Co., Four Holes Swamp, Goodsons Lake; CUAC; ♂]. —Abbott et al. 1997: 44 [distribution]. —Moulton and Stewart 1997: 350 [checklist]. —Etnier and Baxter 1999: 147 [to synonymy].

**Distribution.** —U.S.A.

***lonchera*** Blickle & Morse, 1954: 122 [type locality: [United States], Lee, N. H.; INHS; ♂]. —Blickle 1979: 48, 67 [checklist; ♂]. —Usis and Foote 1989: 84 [distribution]. —Harris et al. 1991: 188 [distribution]. —Armitage et al. 2011: 14 [checklist]. —Myers et al. 2011: 106 [distribution]. —Flint 2014: 90 [distribution].

**Distribution.** —U.S.A.

***longidorsalis*** Zhou & Yang in Zhou et al. 2009a: 908, 911 [type locality: [China], Guangxi Zhuang Autonomous Region, Shangsi City, Shiwandashan National Forest Park, Shitouhe at Second Trib, 3.4 km SW of main entrance to park, 21°53'N, 107°54'E, 392 m; NAUJ; ♂]. —Yang et al. 2016: 476 [checklist; as *longitabularis*].

**Distribution.** —China.

***longifilis*** Yang & Xue, 1994: 10 [type locality: [China], Sichuan, Pingwu county, 19 km E of Pingwu, tributary of Fujiang River, 1090 m; NAUJ; ♂]. —Yang et al. 1997b: 93 [checklist]. —Yang et al. 2005: 458 [checklist]. —Yang et al. 2016: 476 [checklist].

**Distribution.** —China.

***longissima*** Bueno-Soria, 1984: 97 [type locality: Mexico, Guerrero, Acahuizotla; CNIN; ♂; as *longissimus*].

**Distribution.** —Honduras, Mexico.

***losida*** Mosely in Mosely and Kimmins 1953: 505 [type locality: [Australia] Queensland, Eidswold; ANIC; ♂]. —Wells 1978: 757 [♂; ♀; distribution]. Wells 1985b: 6 [case; larva]. —Neboiss 1986: 60 [atlas; ♂; ♀]. —Wells 1995: 231 [distribution]. —Oláh and Johanson 2010a: 18 [distribution]. —Wells and Johanson 2015: 82 [distribution]. —Johanson and Wells 2019: 93 [checklist].

**Distribution.** —Australia, New Caledonia.

***lotensis*** Mosely, 1930b: 243 [type locality: France, Lot, Cahors; NHMUK; ♂]. —Martynov 1934: 133 [♂]. —Racięcka 1936: 98 [distribution]. —Schmid 1959b: 691 [distribution]. —Nybom 1960: 17 [checklist]. —Kimmins 1961: 32 [distribution; ♂; ♀]. —Botosaneanu 1967: 294 [distribution]. —Szczęsny 1975: 41 [distribution]. —Botosaneanu and Malicky 1978: 341 [checklist]. —Kumanski 1979: 9 [♂; distribution]. —Çakin 1983: 246 [distribution]. —Malicky 1983b: 47, 52 [atlas; ♂; ♀]. —Kumanski and Malicky 1984: 199 [distribution]. —Nógrádi 1985: 131 [distribution; ♂]. —Kumanski 1985: 123 [♂]. —Nógrádi 1986: 139 [distribution]. —Andersen and Wiberg-Larsen 1987: 168 [checklist]. —Sipahiler and Malicky 1987: 122 [distribution]. —Cooter 1987: 148 [distribution]. —Rojas-Camousseight and Tachet 1988: 313–314 [♀]. —Usseglio-Polatera and Bournaud 1989: 253 [distribution]. —Spuris 1989: 16 [checklist]. —Nógrádi and Uherkovich 1994: 31 [distribution]. —Nógrádi 1994: 277 [♂ ♀]. —Uherkovich and Nógrádi 1997: 461 [distribution]. —Malicky 1997: 143 [distribution; ♂]. —Uherkovich and Nógrádi 1998: 52 [distribution]. —Graf et al. 1998: 206 [distribution]. —Peissner et al. 1998: 169 [distribution]. —Peissner and Kappus 1998: 162, 163 [distribution]. —Malicky 1999f: 31 [distribution]. —Uherkovich and Nógrádi 1999: 420 [distribution]. —Urbanič et al. 2000: 45 [distribution]. —Uherkovich and Nógrádi 2001: 95 [distribution]. —Nógrádi and Uherkovich 2001: 297 [checklist]. —Coppa 2001: 94 [distribution]. —Mirmoayedi and Malicky 2002: 164 [checklist]. —Ujvárosi 2002: 384 [distribution]. —Nógrádi and Uherkovich 2002: 130 [distribution]. —Cibaitė 2003a: 10 [checklist]. —Malicky 2004a: 57 [atlas]. —Malicky 2005b: 544 [checklist]. —Komzák and Chvojka 2005: 65 [distribution]. —Malicky 2005a: 61 [distribution]. —Sipahiler 2005: 397 [distribution]. —Gullefors 2006: 137 [distribution]. —Mey 2006a: 159 [distribution]. —Robert 2007: 82 [checklist]. —Szczęsny and Godunko 2008: 15 [checklist]. —Chvojka and Komzák 2008: 13 [distribution]. —Ujvárosi et al. 2008: 112 [checklist]. —Gullefors 2008: 64 [checklist]. —Višinskienė 2009: 27 [checklist]. —Neu 2010: 150 [♀]. —González and Menéndez 2011: 119 [distribution]. —Ivanov 2011: 195 [checklist]. —Komzák and Chvojka 2012: 719 [distribution]. —Lock and Goethals 2012: 28 [checklist]. —Kiss 2012: 28 [distribution]. —Stanić-Koštroman et al. 2015: 85 [distribution]. —Pan’kov and Krasheninnikov 2016: 333 [distribution]. —Sipahiler 2016: 12 [distribution]. —Gullefors 2016: 155 [checklist]. —Wallace 2016: 15, 20, 22, 23, 26, 50 [conservation status]. —Melnitsky et al. 2017: 6 [distribution]. —Ibrahimi et al. 2017: 189 [distribution]. —Lock and van Butsel 2018: 3 [distribution; ♀]. —O’Connor and O’Connor 2019: 229 [distribution]. —Cerjanec et al. 2020: 13 [distribution]. —Oláh et al. 2020: 45 [distribution]. —Navara et al. 2020: 46 [distribution].

**Distribution.** —Austria, Azerbaijan, Belgium, Bosnia-Herzegovina, Bulgaria, Croatia, Czech Republic, England, Finland, France, Germany, Greece, Hungary, Iran, Ireland, Lithuania, Luxembourg, Poland, Romania, Russia, Serbia, Slovakia, Slovenia, Spain, Sweden, Turkey, Ukraine.

***luzonensis*** Mey, 2003b: 433 [type locality: Philippines, Luzon, Quezon province, east of Infanta, Magsaysay; ZMHB, to be transferred to either MPMP or UPLB; ♂].

**Distribution.** —Philippines.

***lyaios*** Malicky, 2004b: 294 [type locality: [Nepal, Bardia National Park], am Rande der nordindischen Ebene im Südwesten von Nepal im Bereich des ersten Hügelkammes des Himalaya (Siwalik Range), bei dem Dorf Babai Basar in der Nähe der Straße von Nepalganj nach Birendranagar, ungefähr 30 km flussaufwärts vom Lager 1 (28°21'N, 81°42'E), lag das Ufer des Babai Nadi in wenigen Metern Entfemung, Ratomate Khola, 350 m; Collection Malicky; ♂]. —Malicky 2006: 252 [checklist]. —Mattern 2015: 501 [distribution].

**Distribution.** —Nepal.

***maculata*** (Banks, 1904b): 116 [type locality: [United States], Virginia, Falls Church; MCZ; ♂; in *Allotrichia*]. —Banks 1904a: 215 [distribution]. —Banks 1907a: 50 [catalogue]. —Betten 1934: 148 [checklist]. —Milne 1936: 76 [to *Hydroptila*]. —Ross 1938b: 9 [lectotype designated; ♂]. —Ross 1944: 296 [checklist]. —Blickle 1979: 48, 65 [checklist; ♂]. —Pescador et al. 2004: 133 [checklist]. —Harris et al. 2012: 6 [♂; checklist].

—*transversa* Banks, 1907b: 163 [type locality: [United States], Washington, D. C.; MCZ; ♂]. —Banks 1907a: 50 [catalogue]. —Milne 1936: 77 [as synonym of *albicornis*]. —Ross 1938b: 9 [lectotype designated; to synonymy].

**Distribution.** —U.S.A.

***maetalai*** Malicky & Chantaramongkol, 2007: 1014 [type locality: Thailand, Mae Talai (Süd), 19°16'N 98°37'E, 400 m; Collection Malicky; ♂]. —Malicky 2010a: 29 [atlas; ♂].

**Distribution.** —Thailand.

***makaplag*** Wells & Mey, 2002: 126 [type locality: [Philippines] Leyte, Makaplag; BPBM; ♂].

**Distribution.** —Philippines.

***malacitana*** González & Ruiz in González et al. 2013: 397 [type locality: [Spain], Júzcar (36°37'10"N, 005°09'13.9"W), Vado del Genal, Río Genal, Serranía de Ronda, 521 m, Málaga; DZUSC; ♂]. —Ruiz-García et al. 2016: 4 [distribution].

**Distribution.** —Spain.

***manavgatensis*** Malicky & Çakin in Çakin and Malicky 1983: 270 [type locality: [Turkey], Antalya, Manavgat, Besonak; depository not designated; ♂]. —Sipahiler and Malicky 1987: 129 [distribution]. —Malicky 2004a: 53 [atlas]. —Malicky 2005b: 544 [checklist]. —Sipahiler 2005: 397 [distribution]. —Sipahiler 2017b: 12 [distribution]. —Sipahiler 2018: 41 [distribution].

**Distribution.** —Turkey.

***maoae*** Gibon, Guenda, & Coulibaly, 1994: 110 [type locality: sur la haute Léraba (bassin de la Comoé, région de Banfora, Burkina Faso); MNHN; ♂]. —Wells and de Moor 2020: 497 [distribution].

**Distribution.** —Angola, Burkina Faso.

***mariatheresae*** Gibon, 1987a: 127 [type locality: sur le Bakoye à Kita (bassin du Sénégal, Mali); MNHN; ♂]. —Kjærandsen and Andersen 1997: 244 [distribution].

**Distribution.** —Ghana, Mali.

***marighellai*** de Souza, Santos, & Takiya, 2014b: 640 [type locality: Brazil, Ceará, Parque Nacional de Ubajara, Rio das Minas próximo ao teleférico, 03°48'58"S 40°53'53"W, 420 m; CZMA; ♂]. —Moreno et al. 2020: 265 [distribution].

**Distribution.** —Brazil.

***maritza*** Harris & Holzenthal, 1999: 21 [type locality: Costa Rica, Guanacaste, Parque Nacional Guanacaste, Maritza, Río Tempisquito, 10.958°N, 85.497°W; NMNH; ♂].

**Distribution.** —Costa Rica.

***martini*** Marshall, 1977: 116 [type locality: [England], R. Lambourne, Berkshire; NHMUK; ♂; ♀; *H.occulta* sensu aucttorum nec (Eaton, 1873)]. —Mosely 1939: 265 [[♀; as *H.occulta*]. —Schmid 1947: 529 [♀; as *H.occulta*]. —O’Connor and O’Connor 1980: 167 [distribution]. —Moretti et al. 1981a: 350, 354 [biology; distribution]. —Moretti and Cianficconi 1981: 201 [checklist]. —Malicky 1983b: 48, 52 [atlas; ♂; ♀]. —Maier et al. 1995: 147 [distribution]. —Hohmann 1998: 73 [distribution]. —Hohmann 1999: 34, 35 [distribution; checklist]. —Wiberg-Larsen and Holm 1999: 118 [distribution]. —Cianficconi et al. 1999b: 278 [distribution]. —Nógrádi and Uherkovich 2001: 297 [checklist]. —Cianficconi et al. 2002: 146 [distribution]. —Urbanič 2004: 51 [distribution]. —Malicky 2004a: 60 [atlas]. —Malicky 2005b: 544 [checklist]. —Coppa and Tachet 2005: 130 [♀]. —Cianficconi et al. 2005: 96 [habitat; distribution]. —Graf et al. 2005: 55 [distribution]. —Lubini-Ferlin and Vicentini 2005: 67 [checklist]. —Chvojka and Komzák 2006: 358 [distribution]. —Gullefors and Johanson 2007: 62 [distribution; ♂; ♀]. —Robert 2007: 82 [checklist]. —Cianficconi et al. 2007b: 569, 575 [distribution]. —Chvojka and Komzák 2008: 13 [distribution]. —Ujvárosi et al. 2008: 112 [checklist]. —Cianficconi and Corallini 2010: 87 [distribution]. —Corallini and Cianficconi 2011: 628 [checklist]. —Cianficconi et al. 2011: 47 [distribution]. —González and Menéndez 2011: 119 [distribution]. —Corallini et al. 2013a: 38 [checklist]. —O’Connor 2015: 28, 82 [distribution]. —Cianficconi et al. 2016: 140 [distribution]. —Gullefors 2016: 155 [checklist]. —Wallace 2016: 21, 24 [conservation status]. —O’Connor and O’Connor 2017b: 53 [distribution]. —Valle and Lodovici 2018: 146 [distribution]. —Komzák and Kroča 2018: 166 [distribution]. —O’Connor and O’Connor 2018: 82 [distribution]. —Edmonds-Brown 2020: 91 [checklist].

**Distribution.** —Austria, Czech Republic, Denmark, England, Germany, Hungary, Ireland, Italy, Slovenia, Romania, Spain, Sweden.

***martorelli*** Flint, 1964: 52 [type locality: Puerto Rico, Maricao, at fish hatchery; NMNH; ♂; ♀; larva; case]. —Flint 1968a: 82 [checklist]. —Malicky 1983c: 264 [distribution]. —Flint and Sykora 1993: 50 [checklist]. —Botosaneanu 1994a: 41 [distribution]. —Botosaneanu 2000: 256 [distribution]. —Botosaneanu 2002b: 83 [checklist]. —Botosaneanu and Thomas 2005: 55 [checklist].

**Distribution.** —Guadeloupe, Puerto Rico.

***maza*** Harris & Holzenthal, 1999: 29 [type locality: Costa Rica, San José, Reserva Biológica Carara, Río de Sur, 1.5 km (rd) S Carara, 9.769°N, 84.531°W; NMNH; ♂]. —Armitage et al. 2016: 7 [distribution]. —Armitage and Harris 2018b: 97 [checklist]. —Harris and Armitage 2019: 4 [distribution].

**Distribution.** —Costa Rica, Panama.

***mazumbaiensis*** Wells & Andersen, 1995: 160 [type locality: Tanzania, Tanga region, West Usambara Mts, Mazumbai, Kaputu Stream, loc. 7, 1535 m a.s.l.; ZMUB; ♂].

**Distribution.** —Tanzania.

***medinai*** Flint, 1964: 54 [type locality: Puerto Rico, Maricao, at fish hatchery; NMNH; ♂; ♀]. —Flint 1968a: 82 [checklist]. —Botosaneanu 1977: 271 [♂; variation; distribution]. —Botosaneanu 1979: 51 [distribution]. —Kumanski 1987: 30 [distribution]. —Botosaneanu 1991: 130 [distribution]. —Flint 1996a: 16 [checklist]. —Flint and Pérez-Gelabert 1999: 39 [checklist]. —Botosaneanu 2002b: 83 [checklist]. —Flint and Sykora 2004: 31 [distribution]. —Naranjo López and González Lazo 2005: 149 [checklist]. —Pérez-Gelabert 2008: 300 [checklist].

**Distribution.** —Cuba, Dominican Republic, Haiti, Puerto Rico.

***melia*** Ross, 1938a: 128 [type locality: [United States], Oklahoma, Turner Falls State Park, along Honey Creek; INHS; ♂]. —Blickle 1979: 48, 69 [checklist; ♂]. —Bowles and Mathis 1992: 32 [distribution]. —Moulton and Stewart 1996: 100 [♂; distribution]. —Moulton and Stewart 1997: 350 [checklist]. —Bowles et al. 2007: 21 [distribution; biology].

**Distribution.** —U.S.A.

***mendli mendli*** Malicky, 1980a: 7 [type locality: [Morocco], Gorges du Todra, 1400 m; depository not designated; ♂]. —Malicky 1983b: 49 [atlas; ♂]. —Botosaneanu 1984: 136 [♂]. —Malicky and Lounaci 1987: 15, 17 [checklist]. —Malicky 2004a: 61 [atlas]. —Malicky 2005b: 544 [checklist]. —Sipahiler 2005: 397 [distribution].

**Distribution.** —Morocco, Turkey.

***mendli levanti*** Botosaneanu, 1984: 137 [type locality: [Lebanon], sur le Nahr ed Damour en aval de son confluent avec le Nahr el Hammam, Liban Central, il s’agit des 4 derniers kilomètres d’une petite rivière prenant ses sources dans le versant occidental de la montagne du Barouk et se jetant à la Méditerranée entre Saïda et Beyrouth; ZMUA; ♂]. —Botosaneanu 1992: 67 [♂]. —Dia 2015: 51 [distribution].

**Distribution.** —Lebanon.

***meralda*** Mosely, 1937b: 162 [type locality: Mexico, Chiapas, Esmeralda; NHMUK; ♂]. —Bueno-Soria and Flint 1978: 201 [distribution]. —Bueno-Soria 1984: 109 [♂;]. —Harris and Holzenthal 1999: 42 [♂; distribution]. —Chamorro-Lacayo et al. 2007: 43 [checklist]. —Barba-Álvarez et al. 2019: 85 [distribution].

**Distribution.** —Costa Rica, Mexico, Nicaragua.

***metoeca*** Blickle & Morse, 1954: 127 [type locality: [United States], Lee, N. H.; INHS; ♂]. —Etnier 1968: 191 [distribution]. —Blickle 1979: 48, 65 [checklist; ♂]. —Parker and Voshell 1981: 4 [checklist]. —Marshall and Larson 1982: 30 [distribution]. —Lake 1984: 219 [checklist]. —Usis and Foote 1989: 84 [distribution]. —Masteller and Flint 1992: 70 [checklist]. —Huryn and Harris 2000: 193 [distribution]. —Houghton et al. 2001: 504 [distribution]. —Houghton and Holzenthal 2003: 37 [not found in MN; conservation status]. —Myers et al. 2011: 106 [distribution]. —Armitage et al. 2011: 14 [checklist]. —Houghton et al. 2011b: 5 [phenology; habitat]. —Wright et al. 2013: 466 [biology; distribution]. —Houghton 2016: 46 [biology]. —Houghton et al. 2017: 62 [checklist].

**Distribution.** —Canada, U.S.A.

***metteei*** Harris, 1991: 12 [type locality: [United States], Alabama, Houston County, Cowarts Creek at unnumbered Co. Hwy., 8.8 km ENE Cottonwood (Sec. 10, T 1 N., R 28 E); NMNH; ♂]. —Harris et al. 1991: 189 [distribution]. —Harris et al. 2012: 6 [distribution]. —Denson et al. 2016: 5 [distribution].

**Distribution.** —U.S.A.

***mexicana*** Mosely, 1937b: 160 [type locality: Mexico, Chiapas, Dolores; NHMUK; ♂]. —Bueno-Soria and Flint 1978: 201 [distribution]. —Bueno-Soria 1984: 109 [♂; distribution]. —Harris and Holzenthal 1999: 42 [♂; distribution]. —Maes 1999: 1193 [checklist]. —Bueno-Soria et al. 2005: 75 [distribution]. —Chamorro-Lacayo et al. 2007: 43 [checklist]. —Armitage et al. 2016: 7 [distribution]. —Armitage and Harris 2018b: 97 [checklist]. —Harris and Armitage 2019: 4 [distribution]. —Razo-González et al. 2020: 5 [distribution].

**Distribution.** —Costa Rica, Honduras, Mexico, Nicaragua, Panama.

***micropotamis*** Harris, 1989: 312 [type locality: [United States], Alabama, De Kalb County, Little River at Canyon Park, 4 miles E Dog Town, T8S, R9E, S10; NMNH; ♂]. —Harris et al. 1991: 190 [distribution]. —Frazer et al. 1991: 19 [distribution].

**Distribution.** —U.S.A.

***mindamontana*** Mey, 1998a: 553 [type locality: [Philippines, Mindanao], northern slope of Mt. Atuuganon range, 1050 m; ZMHB; ♂]. —Wells and Mey 2002: 134 [checklist]. —Malicky 2013: 43 [possible junior synonym to *H.pedemontana*].

**Distribution.** —Philippines.

***misolha*** Bueno-Soria, 1984: 127 [type locality: Mexico, Chiapas, Cascada de Misolha; CNIN; ♂]. —Maes and Flint 1988: 4 [distribution]. —Harris and Holzenthal 1999: 45 [♂; distribution]. —Maes 1999: 1193 [checklist]. —Bueno-Soria et al. 2005: 75 [distribution]. —Chamorro-Lacayo et al. 2007: 43 [checklist].

**Distribution.** —Belize, Costa Rica, Honduras, Mexico, Nicaragua.

***mitirigalla*** Schmid, 1958b: 64 [type locality: [Sri Lanka], Ceylan, Lauderdale (Sab., 3500 ft) 5-II, torrent très raide, avec chutes; depository not designated; ♂].

**Distribution.** —Sri Lanka.

***modica*** Mosely, 1937b: 163 [type locality: Mexico, Chiapas, Dolores; NHMUK; ♂]. —Bueno-Soria and Flint 1978: 202 [distribution]. —Blickle 1979: 48, 63 [checklist; ♂]. —Bueno-Soria 1984: 90 [♂; distribution]. —Moulton and Stewart 1997: 350 [distribution]. —Newell et al. 2001: 192 [distribution; phenology]. —Blinn and Ruiter 2005: 68 [distribution; biology]. —Bowles et al. 2007: 21 [distribution; biology]. —Bueno-Soria et al. 2007: 33 [distribution]. —Vieira et al. 2009: 257 [distribution]. —Blinn and Ruiter 2013: 291 [biology; distribution]. —Mendez et al. 2019: 128 [checklist].

**Distribution.** —Mexico, U.S.A.

***mokowu*** Wells & Huisman, 2001: 208 [type locality: Sulawesi Tenggara, N slope of Gunung Watuwila, 250 m, Sungai Mokowu; RMNH; ♂].

**Distribution.** —Indonesia.

***molsonae*** Blickle, 1961: 132 [type locality: [United States], Florida, Highlands Hammock State Park, Highlands Co.; INHS; ♂]. —Blickle 1979: 48, 67 [checklist; ♂]. —Harris et al. 1982a: 510 [distribution]. —Harris et al. 1991: 191 [distribution]. —Pescador et al. 2004: 133 [checklist]. —Harris et al. 2012: 6 [♂; checklist]. —Denson et al. 2016: 5 [distribution].

**Distribution.** —U.S.A.

***montatan*** Malicky & Chantaramongkol, 2007: 1019 [type locality: Thailand, Doi Suthep NP, Montatan WF, 18°49'N 98°55'E, 550 m; Collection Malicky; ♂]. —Malicky 2010a: 25 [atlas; ♂].

**Distribution.** —Thailand.

***morogorensis*** Wells & Andersen, 1995: 158 [type locality: Tanzania, Morogoro region, Morogoro, Sokoine University of Agriculture, 550 m a.s.l.; ZMUB; ♂].

**Distribution.** —Tanzania.

***morpheus*** Malicky, 2004b: 294 [type locality: [Nepal, Bardia National Park], am Rande der nordindischen Ebene im Südwesten von Nepal im Bereich des ersten Hügelkammes des Himalaya (Siwalik Range), unweit des Wehrs des Babai Flusses, über das die Brücke der Ost-West-Haupstraße Nepals (Mahindra Highway), 28°25'N, 81°23'E, 190 m, Budhi Khola; Collection Malicky; ♂]. —Malicky 2006: 252 [checklist]. —Mattern 2015: 501 [distribution].

**Distribution.** —Nepal.

***moselyi*** Ulmer, 1932: 42 [type locality: [China], Peiping; ZMUH; ♂]. —Kumanski 1990: 48 [distribution]. —Xue et al. 1992: 353–356 [distribution]. —Yang et al. 1997b: 93 [checklist]. —Yang et al. 2005: 458 [checklist]. —Yang et al. 2016: 476 [checklist]. —Park and Kong 2020: 297 [checklist].

**Distribution.** —China, Korea.

***moxica*** Wells & de Moor, 2020: 498 [type locality: Angola, Moxico Province, Cuando River, Site 8 — Cuando campsite bridge, -13.607, 19.53235; AMGS; ♂].

**Distribution.** —Angola.

***mugla*** Sipahiler, 1989: 131 [type locality: Turkey, Mugla, Fethiye, 30 km to Köycegiz, 29°02'N, 36°45'E; depository not designated; ♂]. —Malicky 2004a: 63 [atlas]. —Malicky 2005b: 544 [checklist]. —Sipahiler 2005: 397 [distribution]. —Melnitsky et al. 2017: 6 [distribution].

**Distribution.** —Turkey.

***murtlei*** Harris, Rasmussen, & Denson, 2012: 2 [type locality: [United States], Florida, Bay Co., Little Crooked Creek at SR-79, Pine Log State Forest, 2.4 km S Ebro, N30°24'48", W85°52'04"; NMNH; ♂]. —Denson et al. 2016: 5 [distribution].

**Distribution.** —U.S.A.

***nago*** Ito, in Ito and Shimura 2019: 27 [type locality: Japan, Ryukyu, Okinawa-jima, Nago-shi, Genka, Genka-gawa, Hogen-hashi (26.6292 N, 128.0847 E, 90 m above sea level); CBM-ZI; ♂].

**Distribution.** —Japan.

***nambelensis*** Johanson, Wells, Malm, & Espeland, 2011: 290 [type locality: [Vanuatu] Espiritu Santo, Central Santo, stream in small canyon crossing path to village, 5.5 km NW Nambel, 208 m, loc#21, 15°27.459'S, 167°04.022'E; NHRS; ♂].

**Distribution.** —Vanuatu.

***namcattien*** Malicky & Chantaramongkol, 2007: 1020 [type locality: Vietnam, Nam Cat Tien, 11°26'N 107°26'E, 200 m; Collection Malicky; ♂]. —Malicky 2010a: 32 [atlas; ♂].

—*motminh* Oláh & Johanson, 2010a: 18 [type locality: Laos, Luang Namtha Prov., Nam Ha NBCA, Nam Gnang stream, 300 m upstr. Namgnen Village, 558 m; NHRS; ♂]. —Malicky 2013: 43 [to synonymy].

**Distribution.** —Laos, Vietnam.

***nanseiensis*** Ito, in Ito et al. 2011: 15 [type locality: Japan, Okinawa, Yaeyima Islands, Ishigaki-jima, Omoto-dake, 24°25'N 124°11'E, 80 m; CBM-ZI; ♂]. —Ito 2015: 8, 15 [distribution]. —Tanida and Kuranishi 2016: 70 [checklist].

**Distribution.** —Japan.

***narifer*** Flint, 1991b: 47 [type locality: Colombia, Dpto. Antioquia, Quebrada La Jiménez, Sopetrán; NMNH; ♂; ♀]. —Muñoz-Quesada 2000: 278 [checklist].

**Distribution.** —Colombia.

***nasuli*** Wells & Mey, 2002: 130 [type locality: [Philippines] Mindanao, Nasuli nr Malaybalay, Bukidnon; BPBM; ♂]. Malicky and Chantaramongkol 2007: 1024 [distribution]. —Malicky 2009b: 10 [distribution].

**Distribution.** —Philippines.

***neciel*** Malicky, Melnitsky, & Ivanov, 2020: 538 [type locality: [Indonesia], Papua, 3 k S Wamena, Helaluwa river, 1679 m, 4°08'S, 138°56'E; ZIN; ♂].

**Distribution.** —Indonesia.

***nemtompa*** Oláh, 2012: 48 [type locality: Indonesia, Papua, Raja Empat Archipelago, Batanta Island, Ron Creek, 0°49'16.37"S 130°49'23.72"E; Collection Oláh; ♂]. —Oláh 2016: 113 [distribution]. —Oláh and Kovács 2018: 179 [distribution].

**Distribution.** —Indonesia.

***neoleonensis*** Bueno-Soria, 1984: 113 [type locality: Mexico, Nuevo Leon, Linares; NMNH; ♂].

**Distribution.** —Mexico.

***ngaythibaya*** Oláh, 1989: 283 [type locality: Vietnam, Ngoclac; HNHM; ♂]. —Armitage et al. 2005: 27 [checklist]. —Malicky 2010a: 26 [atlas; ♂].

**Distribution.** —Vietnam.

***nicoli*** Ross, 1941a: 69 [type locality: [Canda], Nova Scotia, Moser River, Gold Mine Brok; INHS; ♂; ♀]. —Blickle 1979: 48, 65 [checklist; ♂]. —Myers et al. 2011: 106 [distribution]. —Flint 2014: 90 [distribution]. —Houghton et al. 2017: 62 [checklist].

**Distribution.** —Canada, U.S.A.

***nigrovalvata*** Mey, 2003b: 433 [type locality: Philippines, Luzon, Laguna, Pangil; ZMHB, to be transferred to either MPMP or UPLB; ♂].

**Distribution.** —Philippines.

***novicola*** Blickle & Morse, 1954: 124 [type locality: [United States], Durham, N. H.; INHS; ♂]. —Etnier 1968: 191 [distribution]. —Blickle 1979: 48, 73 [checklist; ♂]. —Roy and Harper 1979: 151 [checklist]. —Roy and Harper 1981: 105 [distribution]. —Harris et al. 1982a: 510 [distribution]. —Harris et al. 1984: 108 [distribution]. —Harris et al. 1991: 192 [distribution]. —Frazer et al. 1991: 19 [distribution]. —Moulton and Stewart 1997: 350 [checklist]. —Abbott et al. 1997: 44 [distribution]. —Huryn and Harris 2000: 193 [distribution]. —Houghton et al. 2001: 504 [distribution]. —Houghton and Holzenthal 2003: 39 [distribution]. —Pescador et al. 2004: 133 [checklist]. —Myers et al. 2011: 107 [distribution]. —Harris et al. 2012: 6 [♂; checklist]. —Houghton 2016: 46 [biology]. —Houghton et al. 2017: 63 [checklist].

**Distribution.** —Canada, U.S.A.

***nusagandia*** Harris & Holzenthal, 1999: 29 [type locality: Panama, San Blas, Quebrada Pingad, 9 km N Nusagandi; NMNH; ♂]. —Armitage et al. 2015a: 6 [checklist]. —Armitage and Harris 2018b: 97 [checklist].

**Distribution.** —Panama.

***oakmulgeensis*** Harris, 1985b: 612 [type locality: [United States], Alabama, Choctaw County, Tallawampa Creek at Co. Hwy. 23; NMNH; ♂]. —Harris et al. 1991: 193 [distribution].

**Distribution.** —U.S.A.

***obscura*** Wells, 1978: 758 [type locality: [Australia], Queensland, Palmer River; ANIC; ♂; ♀]. —Wells 1985b: 7 [pupa case]. —Neboiss 1986: 62 [atlas; ♂; ♀]. —Wells 1990b: 382 [♂; ♀; case; distribution]. —Wells 1991: 504 [distribution]. —Wells and Huisman 1992: 97 [distribution]. —Wells and Mey 2002: 128 [distribution]. —Malicky and Chantaramongkol 2007: 1023 [distribution]. —Malicky 2009b: 10 [distribution]. —Oláh and Johanson 2010a: 19 [distribution]. —Malicky 2010a: 23 [atlas; ♂]. —Malicky et al. 2010: 163 [distribution]. —Oláh 2012: 49 [distribution]. —Oláh 2016: 111 [distribution; ♂].

**Distribution.** —Australia, Borneo, Indonesia, Malaysia, Papua New Guinea, Philippines.

***occulta*** (Eaton, 1873): 135 [type locality: [England], Mappleton, near Ashbourne, Derbyshire, between the bridge and the weir; NHMUK; ♂; in *Phrixocoma*]. —McLachlan 1880: 512 [revision; ♂]. —Morton 1899b: 281 [distribution]. —Mosely 1919a: 396 [scent-organ]. —Ulmer 1929: 263 [morphological notes]. —Racięcka 1936: 98 [distribution].—Nielsen 1951: 122 [distribution; ♂; ♀]. —Kimmins 1957a: 109 [lectotype designation]. —Botosaneanu 1967: 294 [distribution]. —Spuris 1972: 28, 30 [checklist]. —Marshall 1977: 119 [revision; ♂; ♀]. —Botosaneanu and Malicky 1978: 341 [checklist]. —Kumanski 1979: 12 [♂; distribution]. —O’Connor and O’Connor 1980: 167 [distribution]. —Mey 1981: 56 [distribution]. —Moretti and Cianficconi 1981: 201 [checklist]. —Çakin 1983: 246 [distribution]. —Malicky 1983b: 48, 52 [atlas; ♂; ♀]. —Kumanski and Malicky 1984: 199 [distribution]. —Kumanski 1985: 130 [♂]. —Andersen and Tysse 1985: 84 [distribution]. —Wiberg-Larsen 1985: 40 [checklist]. —Glapska 1986: 30 [distribution]. —Sipahiler and Malicky 1987: 112, 129, 143 [distribution]. —Andersen and Wiberg-Larsen 1987: 168 [checklist]. —Usseglio-Polatera and Bournaud 1989: 254 [distribution]. —Spuris 1989: 16 [checklist]. —Andersen et al. 1990: 52 [distribution]. —Uherkovich and Nógrádi 1998: 52 [distribution]. —Uherkovich and Nógrádi 1999: 420 [distribution]. —Nógrádi and Uherkovich 2001: 297 [checklist]. —Gullefors 2002: 138 [checklist]. —Mirmoayedi and Malicky 2002: 164 [checklist]. —Nógrádi and Uherkovich 2002: 130 [distribution]. —Ujvárosi 2002: 384 [distribution]. —Sipahiler 2003b: 33 [distribution]. —Cibaitė 2003a: 10 [checklist]. —Malicky 2004a: 60, 64 [atlas]. —Malicky 2005b: 544 [checklist]. —Coppa and Tachet 2005: 127, 130 [♂; ♀]. —Weinzierl et al. 2005: 46 [distribution]. —Malicky 2005a: 61 [distribution]. —Sipahiler 2005: 397 [distribution]. —Lubini-Ferlin and Vicentini 2005: 67 [checklist]. —Hohmann et al. 2006: 111 [distribution]. —Cianficconi et al. 2007b: 569, 575 [distribution]. —Sipahiler 2007: 38 [distribution]. —Gullefors and Johanson 2007: 64 [distribution]. —Robert 2007: 82 [checklist]. —Szczęsny and Godunko 2008: 15 [checklist]. —Gullefors 2008: 64 [checklist]. —Chvojka and Komzák 2008: 13 [distribution]. —Ujvárosi et al. 2008: 112 [checklist]. —Chvojka et al. 2009: 82 [distribution]. —Višinskienė 2009: 27 [checklist]. —González and Menéndez 2011: 119 [distribution]. —Crofts 2011: 72 [distribution]. —Ivanov 2011: 195 [checklist]. —Šemnički et al. 2011: 149 [distribution]. —Viidalepp et al. 2011: 196 [distribution]. —Komzák and Chvojka 2012: 720 [distribution]. —Lock and Goethals 2012: 28 [checklist]. —Wolf et al. 2012: 75 [distribution]. —Corallini et al. 2013a: 38 [checklist]. —Karaouzas and Malicky 2015: 14 [distribution]. —O’Connor 2015: 28, 84 [distribution]. —Dia 2015: 51 [distribution]. —Sipahiler 2016: 12 [distribution]. —Smirnova et al. 2016: 401 [distribution]. —Cianficconi et al. 2016: 141 [distribution]. —Pan’kov and Krasheninnikov 2016: 333 [distribution]. —Sipahiler 2016: 12 [distribution]. —Gullefors 2016: 155 [checklist]. —Wallace 2016: 21, 24 [conservation status]. —Sipahiler 2017b: 12 [distribution]. —Melnitsky et al. 2017: 6 [distribution]. —Komzák and Kroča 2018: 166 [distribution]. —Sipahiler 2018: 41 [distribution].

—*insignis* Martynov, 1927: 176 [type locality: [Kazakhstan?], Turkestan, River Boroldai, near the vill. Alexeievka, district Katchkar-ata, East Kara-tau; depository not designated; ♂]. —Martynov 1934: 143 [♂]. —Botosaneanu 1967: 294 [suggested synonym]. —Botosaneanu and Malicky 1978: 341 [to synonymy]. —Spuris 1989: 16 [checklist].

—*kimminsi* Mosely, 1930b: 245 [type locality: France, Lozère, Mende; NHMUK; ♂]. —Schmid 1947: 531 [distribution]. —Nybom 1960: 18 [checklist]. —Botosaneanu 1967: 294 [distribution]. —Marshall 1977: 119 [to synonymy]. —Spuris 1989: 16 [checklist].

—*parthava* Schmid, 1959b: 686 [type locality: [Iran] Durb Adam (Ost. 9); CNC; ♂]. —Botosaneanu 1967: 294 [probable synonym]. —Botosaneanu and Malicky 1978: 341 [to synonymy]. —Lonsdale 2020: 38 [holotype depository].

**Distribution.** —Belgium, Bulgaria, Croatia, Czech Republic, Denmark, England, Estonia, Finland, France, Germany, Greece, Hungary, Iran, Ireland, Italy, Kazakhstan, Lebanon, Lithuania, Norway, Poland, Romania, Russia, Scotland, Spain, Sweden, Switzerland, Turkey, Ukraine, Uzbekistan.

***oemerueneli*** Sipahiler, 2003a: 20 [type locality: Turkey, Kastamonu, Pinarbasi, Varla Mahallesi, Devrekani Kanyonu, Devrekani Deresi, 41°36'N, 33°54'E; depository not designated; ♂]. —Malicky 2004a: 63 [atlas]. —Malicky 2005b: 544 [checklist]. —Sipahiler 2005: 397 [distribution]. —Sipahiler 2007: 38 [distribution]. —Sipahiler 2008: 104 [checklist]. —Küçükbasmaci and Kiyak 2017: 488 [distribution].

**Distribution.** —Turkey.

***ogasawaraensis*** Ito in Ito et al. 2011: 7 [type locality: Japan, Ogasawara Islands, Chichi-jima, Ogasawara-mura, a headwater of Yasse-gawa, 27°03'55"N 142°13'08"E; CBM-ZI, 210 m; ♂]. —Ito 2015: 8 [checklist]. —Tanida and Kuranishi 2016: 70 [checklist].

**Distribution.** —Japan.

***oguranis*** Kobayashi, 1974: 68 [type locality: [Japan], Mt. Ogura, Shiroyama-Machi, Tsukuigun, Kanagawa Prefecture; depository not designated; ♂]. —Ito et al. 1993: 142 [checklist]. —Morse et al. 2001: 102 [distribution]. —Tanida et al. 2005: 441 [♂]. —Nozaki and Tanida 2007: 245 [distribution]. —Ito et al. 2011: 8 [♂, ♀; distribution]. —Ito 2015: 8, 15 [checklist]. —Tanida and Kuranishi 2016: 70 [checklist].

**Distribution.** —Japan, Russia.

***okaloosa*** Harris, 2002: 53 [type locality: [United States], Florida, Okaloosa County, Rogue Creek, 0.6 km S Base Rd. 232, Eglin Air Force Base, 30°33'19"N, 86°34'52"W; NMNH; ♂]. —Harris et al. 2012: 7 [checklist].

**Distribution.** —U.S.A.

***oknos*** Malicky, 2004b: 294 [type locality: [Nepal, Bardia National Park], am Rande der nordindischen Ebene im Südwesten von Nepal im Bereich des ersten Hügelkammes des Himalaya (Siwalik Range), bei dem Dorf Babai Basar in der Nähe der Straße von Nepalganj nach Birendranagar, ungefähr 30 km flussaufwärts vom Lager 1 (28°21'N, 81°42'E), lag das Ufer des Babai Nadi in wenigen Metern Entfemung, vom “östlicher” Bach, 320 m; Collection Malicky; ♂]. —Malicky 2006: 252 [checklist]. —Mattern 2015: 501 [distribution].

**Distribution.** —Nepal.

***oneili*** Harris, 1985b: 618 [type locality: [United States], Alabama, Bibb County, spring at Schutlz Creek Church, 2.5 miles southwest of West Blocton; NMNH; ♂]. —Harris et al. 1991: 194 [distribution]. —Frazer et al. 1991: 20 [distribution]. —Moulton and Stewart 1996: 101 [♂; distribution]. —Etnier 2010: 485 [distribution].

**Distribution.** —U.S.A.

***orion*** Malicky & Chantaramongkol, 2007: 1018 [type locality: Thailand, Tung Salaeng NP, 16°49'N 100°57'E, 600 m; Collection Malicky; ♂]. —Oláh and Johanson 2010a: 19 [distribution]. —Malicky 2010a: 26 [atlas; ♂].

**Distribution.** —Thailand, Vietnam.

***ornithocephala*** Yang & Xue, 1992: 27 [type locality: [China] Wudalianchi, Heilongjiang; NAUJ; ♂]. —Yang et al. 1997b: 93 [checklist]. —Morse et al. 2001: 102 [distribution]. —Yang et al. 2005: 458 [checklist]. —Yang et al. 2016: 476 [checklist]. —Potikha and Vshivkova 2016: 364 [distribution].

**Distribution.** —China, Russia.

***ortaca*** Sipahiler, 1989: 129 [type locality: Turkey, Mugla, Fethiye, 10 km to Köycegiz, 29°02'N, 36°45'E; depository not designated; ♂]. —Malicky 2004a: 53 [atlas]. —Malicky 2005b: 544 [checklist]. —Sipahiler 2005: 397 [distribution].

**Distribution.** —Turkey.

***osa*** Harris & Holzenthal, 1999: 21 [type locality: Costa Rica, Puntarenas, Quebrada Pita, ca 3 km (air) W Golfito, 8.642°N, 83.193°W; NMNH; ♂].

**Distribution.** —Costa Rica.

***ouachita*** Holzenthal & Kelley, 1983: 468 [type locality: [United States], Louisiana, Jackson Parish, Schoolhouse Spring, T17N, R1W, Sec. 12; NMNH; ♂]. —Moulton and Stewart 1997: 350 [checklist].

**Distribution.** —U.S.A.

***ovacikensis*** Sipahiler in Sipahiler and Malicky 1987: 86 [type locality: Turkey, Tunceli, 15 km NE Ovacik, Mercan Vadisi, Mollaaliler Köyü; type depository not given; ♂]. —Malicky 2004a: 64 [atlas]. —Malicky 2005b: 544 [checklist]. —Sipahiler 2005: 397 [distribution].

**Distribution.** —Turkey.

***palaestinae*** Botosaneanu & Gasith, 1971: 99 [type locality: [Israel], Beit She’an; TAU; ♂; ♀]. —Botosaneanu and Malicky 1978: 341 [checklist]. —Malicky 1983b: 49, 52 [atlas; ♂; ♀]. —Botosaneanu 1992: 69 [♂; ♀]. —Malicky 2004a: 61 [atlas]. —Malicky 2005b: 544 [checklist]. —Sipahiler 2005: 397 [distribution]. —Malicky 2005a: 61 [distribution]. —Karaouzas and Malicky 2015: 14 [distribution].

**Distribution.** —Greece, Israel, Turkey.

***panchaoi*** Schmid, 1960: 93 [type locality: [Pakistan] Himalaya, Kel; CNC; ♂]. —Schmid 1958c: 220 [as new species, *nomen nudum*; distribution]. —Lonsdale 2020: 38 [holotype depository].

**Distribution.** —Pakistan.

***parachelops*** Sykora & Harris, 1994: 69 [type locality: [United States], Pennsylvania, Fayette Co., Youghiogheny River Lake outflow near Confluence; CMNH; ♂].

**Distribution.** —U.S.A.

***paradenza*** Harris & Holzenthal, 1999: 25 [type locality: Costa Rica, Limón, E.A.R.T.H., Río Destierra, Poza Azul, 10.208°N, 83.574°W; NMNH; ♂]. —Chamorro-Lacayo et al. 2007: 43 [checklist]. —Armitage et al. 2016: 7 [distribution]. —Armitage and Harris 2018b: 97 [checklist]. —Armitage and Harris 2018c: 283 [distribution]. —Harris and Armitage 2019: 4 [distribution].

**Distribution.** —Costa Rica, Mexico, Nicaragua, Panama.

***parakampsis*** Malicky, 2004b: 294 [type locality: [Nepal, Bardia National Park], am Rande der nordindischen Ebene im Südwesten von Nepal im Bereich des ersten Hügelkammes des Himalaya (Siwalik Range), bei dem Dorf Babai Basar in der Nähe der Straße von Nepalganj nach Birendranagar, ungefähr 30 km flussaufwärts vom Lager 1 (28°21'N, 81°42'E), lag das Ufer des Babai Nadi in wenigen Metern Entfemung, vom “östlicher” Bach, 320 m; Collection Malicky; ♂]. —Malicky 2006: 252 [checklist]. —Mattern 2015: 501 [distribution]. —Malicky 2018: 49 [checklist].

**Distribution.** —Nepal.

***paralatosa*** Harris, 1985b: 608 [type locality: [United States], Alabama, Tuscaloosa County, Hurricane Creek at Old Mill Trace, 1 mile south Cottondale; NMNH; ♂]. —Harris et al. 1991: 195 [distribution]. —Harris et al. 2012: 7 [distribution]. —Denson et al. 2016: 5 [distribution].

**Distribution.** —U.S.A.

***paramoena*** Harris, 1985b: 616 [type locality: [United States], Alabama, Bibb County, Six Mile Creek at Hwy. 25; NMNH; ♂]. —Harris et al. 1991: 196 [distribution]. —Frazer et al. 1991: 20 [distribution].

**Distribution.** —U.S.A.

***parapiculata*** Yang & Xue, 1994: 9 [type locality: [China], Sichuan, E-mei Mountain, Yu-jia River, Jie-tuo bridge; NAUJ; ♂]. —Yang et al. 1997b: 93 [checklist]. —Yang et al. 2005: 458 [checklist]. —Yang et al. 2016: 476 [checklist]. —Kobayashi et al. 2017: 17 [distribution]. —Ito and Shimura 2019: 32 [♂; distribution].

**Distribution.** —China, Japan.

***parastrepha*** Kelley & Harris, 1983: 182 [type locality: [United States], Alabama, Mobile County, Puppy Creek at Co. Rd. 217, 7 miles southwest of Citronelle; NMNH; ♂]. —Harris et al. 1991: 197 [distribution]. —Pescador et al. 2004: 133 [checklist]. —Harris et al. 2012: 7 [checklist].

**Distribution.** —U.S.A.

***paraxella*** Harris & Armitage in Armitage et al. 2011: 34 [type locality: [United States], Ohio, Miami County, Charleston Falls, Charleston Falls Preserve, Miami County Park District, N39.91853, W84.1461; NMNH; ♂; ♀].

**Distribution.** —U.S.A.

***parhuzam*** Oláh & Johanson, 2011: 125 [type locality: Peru, Pasco Reg., Yanachaga-Chemillen NP., side river to Rio Huancabamba, N end of park, along Oxabamba-Pozuzo rd., 10°11.133'S 75°34.106'W; NHRS; ♂].

**Distribution.** —Peru.

***paschia*** Mosely, 1937b: 164 [type locality: Mexico, Chiapas, Dolores; NHMUK; ♂]. —Bueno-Soria and Flint 1978: 202 [distribution]. —Bueno-Soria 1984: 102 [♂; distribution]. —Holzenthal 1988: 61 [distribution]. —Harris and Holzenthal 1999: 45 [♂; distribution]. —Maes 1999: 1193 [checklist]. —Bueno-Soria et al. 2007: 33 [distribution]. —Chamorro-Lacayo et al. 2007: 43 [checklist]. —Ríos-Touma et al. 2017: 10 [distribution]. —Armitage and Harris 2018a: 9 [distribution]. —Armitage and Harris 2018b: 97 [checklist]. —Armitage and Harris 2018c: 283 [distribution].

**Distribution.** —Costa Rica, Ecuador, Mexico, Nicaragua, Panama.

***patriciae*** Harris, 1985a: 250 [type locality: [United States], Alabama, Bibb County, Little Schultz Creek at spring, 2.5 miles S West Blocton, T24N, R10E, S30; NMNH; ♂]. —Harris et al. 1991: 198 [distribution].

**Distribution.** —U.S.A.

***pecos*** Ross, 1941a: 64 [type locality: [United States], New Mexico, Carlsbad, along bank of Pecos River; INHS; ♂]. —Denning 1947a: 151 [distribution]. —Blickle 1979: 49, 71 [checklist; ♂]. —Hamilton et al. 1983: 18 [distribution]. —Moulton and Stewart 1997: 350 [checklist]. —Ruiter 1999: 165 [distribution]. —Blinn and Ruiter 2005: 68 [distribution; biology].

**Distribution.** —U.S.A.

***pectinifera*** Schmid, 1970: 118 [type locality: [Mongolia], 8 km N von Somon Burenchaan, am Fluß Delger mörön, 1450 m; CNC; ♂]. —Morse et al. 2006: 321 [distribution]. —Chuluunbat et al. 2016: 102 [distribution].

**Distribution.** —Mongolia.

***pedemontana*** Mey, 1995: 195 [type locality: [Philippines], Mindoro, Mamburao; Collection Mey; ♂]. —Wells and Mey 2002: 128 [distribution]. —Malicky and Chantaramongkol 2007: 1023 [distribution]. —Malicky 2009b: 10 [distribution]. —Malicky 2013: 43 [possible senior synonym to *H.mindamontana*]. —Malicky 2014a: 1623 [checklist]. —Yang et al. 2016: 476 [checklist].

**Distribution.** —Philippines, Taiwan.

***penthesileia*** Malicky & Chantaramongkol, 2007: 1019 [type locality: Thailand, Chiang Dao WRS, 19°22'N 98°55'E, 500 m; Collection Malicky; ♂]. —Malicky 2010a: 26 [atlas; ♂].

**Distribution.** —Thailand.

***perdita*** Morton, 1905: 67 [type locality: [United States], Ithaca, New York; depository not designated; ♂]. —Banks 1907a: 50 [catalogue]. —Mosely 1923: 293 [scent-organ]. —Betten 1934: 160 [distribution]. —Ross 1944: 153 [♂; ♀; distribution]. —Denning 1947b: 174 [distribution]. —Etnier 1965: 147 [checklist]. —Unzicker et al. 1970: 172 [distribution]. —Roy and Harper 1979: 151 [checklist]. —Blickle 1979: 49 [checklist]. —Huryn and Foote 1983: 790 [distribution]. —Waltz and McCafferty 1983a: 10 [distribution]. —Hamilton et al. 1983: 18 [distribution]. —Lake 1984: 220 [distribution]. —Bowles and Mathis 1989: 239 [distribution]. —Tarter 1990: 239 [checklist]. —Harris et al. 1991: 199 [distribution]. —Floyd 1992: 50 [distribution]. —Masteller and Flint 1992: 70 [checklist]. —Mathis and Bowles 1992: 24 [distribution]. —Bowles and Mathis 1992: 32 [distribution]. —Moulton and Stewart 1996: 101 [♂; distribution]. —Keiper and Foote 1999: 517 [biology; larva]. —Houghton et al. 2001: 504 [distribution]. —Zeullig et al. 2006: 43 [distribution]. —Flint 2011: 104 [distribution]. —Armitage et al. 2011: 14 [checklist]. —DeWalt et al. 2016: 51 [distribution]. —Houghton et al. 2017: 63 [checklist]. —Bowles et al. 2020: 8 [distribution].

**Distribution.** —Canada, U.S.A.

***perimele*** Malicky, 2004b: 295 [type locality: [Nepal, Bardia National Park], am Rande der nordindischen Ebene im Südwesten von Nepal im Bereich des ersten Hügelkammes des Himalaya (Siwalik Range), bei dem Dorf Babai Basar in der Nähe der Straße von Nepalganj nach Birendranagar, ungefähr 30 km flussaufwärts vom Lager 1 (28°21'N, 81°42'E), lag das Ufer des Babai Nadi in wenigen Metern Entfemung, vom “westlicher” Bach, 320 m; Collection Malicky; ♂]. —Malicky 2006: 252 [checklist]. —Mattern 2015: 501 [distribution].

**Distribution.** —Nepal.

***perplexa*** Mosely, 1923: 293 [type locality: [England] “Britain”; depository not designated; scent-organ]. —Milne 1936: 77 [as subspecies of *H.hamata*]. —Ross 1944: 296 [nomen dubium].

**Distribution.** —England.

***phaon*** Malicky, 1976: 93 [type locality: Greece, Epirus, Ambelos, 600 m; Collection Malicky; ♂]. —Botosaneanu and Malicky 1978: 341 [checklist]. —Malicky 1983b: 49 [atlas; ♂]. —Valle 2001: 66 [distribution]. —Malicky 2002: 4 [distribution]. —Malicky 2004a: 61 [atlas]. —Malicky 2005b: 544 [checklist]. —Coppa and Tachet 2005: 125 [♂; ♀]. —Malicky 2005a: 61 [distribution]. —Malicky 2007b: 51 [checklist]. —Cianficconi et al. 2007b: 575 [distribution]. —Botosaneanu and Giudicelli 2004: 15 [♀; distribution]. —Oláh 2010: 92 [distribution]. —Corallini and Cianficconi 2011: 628 [checklist]. —González and Menéndez 2011: 119 [distribution]. —Corallini et al. 2013a: 38 [checklist]. —Karaouzas and Malicky 2015: 14 [distribution]. —Cianficconi et al. 2016: 141 [distribution]. —Valle and Lodovici 2018: 146 [distribution]. —Kučinić et al. 2020: 80 [distribution].

**Distribution.** —Croatia, France, Greece, Italy, Spain.

***phenianica*** Botosaneanu, 1970: 290 [type locality: [North Korea], Station 18, Phjongjang, le fleuve Tedong-gang; MZPW; ♂; ♀]. —Kumanski 1990: 48 [distribution]. —Arefina et al. 2002: 9 [distribution]. —Nozaki and Tanida 2007: 246 [distribution]. —Nozaki 2010: 22 [distribution]. —Oláh and Johanson 2010a: 21 [distribution]. —Ivanov 2011: 195 [checklist]. —Ito et al. 2011: 2 [♂; ♀; distribution]. —Hirabayashi et al. 2011: 145 [distribution]. —Ito and Nagasaka 2014: 9 [distribution]. —Ito 2015: 8, 13 [checklist]. —Potikha and Vshivkova 2016: 364 [distribution]. —Tanida and Kuranishi 2016: 71 [checklist]. —Kimura et al. 2016: 246 [distribution]. —Nozaki et al. 2016: 324 [distribution]. —Kobayashi et al. 2017: 17 [distribution]. —Nozaki et al. 2019: 168 [distribution]. —Park and Kong 2020: 297 [checklist].

—*matsuii* Kobayashi, 1974: 67 [type locality: [Japan], Riv. Chikuma, Ueda City, Nagano Prefecture; depository not designated; ♂]. —Ito et al. 1993: 142 [checklist]. —Morse et al. 2001: 102 [distribution]. —Tanida et al. 2005: 441 [♂]. —Nozaki and Tanida 2007: 246 [to synonymy]. —Kimura et al. 2008: 264 [biology; distribution].

**Distribution.** —Japan, Korea, Russia.

♰ ***phileos*** Cockerell, 1920: 239 [type locality: [United States], Eocene (Green River) shales, “Cathedral Bluffs south of Little Tommies Draw at point where samples were taken”, Colorado; NMNH; ♂; in amber]. —Eskov et al. 2008: 78 [checklist].

**Distribution.** —Eocene amber.

***phoeniciae*** Botosaneanu & Dia in Dia and Botosaneanu 1983: 128 [type locality: [Lebanon], Station 5, Nabaa Bâter ech Chouf, source et ruisselet dans le bassin du Nahr el Aouali et dans le massif de Niha, en aval du village Niha, 820 m; ZMUA; ♂; ♀]. —Botosaneanu 1992: 65 [♂, ♀]. —Malicky 1997: 144 [♂]. —Malicky 2004a: 59 [atlas]. —Malicky 2005b: 544 [checklist]. —Dia 2015: 51 [distribution].

**Distribution.** —Lebanon.

***pintal*** Wells & Huisman, 1992: 99 [type locality: East Malaysia, Sabah, Tenom; NTM; ♂; ♀]. —Wells and Malicky 1997: 183 [distribution]. —Malicky and Chantaramongkol 2007: 1023 [distribution]. —Malicky 2007a: 177 [checklist]. —Oláh and Johanson 2010a: 21 [distribution]. —Malicky 2010a: 28 [atlas; ♂]. —Malicky et al. 2014a: 6 [distribution]. —Melnitsky et al. 2019: 539 [distribution].

**Distribution.** —Borneo, Indonesia, Malaysia.

***poirrieri*** Holzenthal & Kelley, 1983: 469 [type locality: [United States], Mississippi, Clarke Co., Chunky Creek at dirt road, 7.1 km. BNW of Hwy. 11 in Enterprise; NMNH; ♂]. —Etnier 2010: 485 [distribution].

**Distribution.** —U.S.A.

***portunus*** Malicky & Chantaramongkol, 2007: 1013 [type locality: Laos, Prov., Viangchan, Phou Khao Khouay NP, Tad Leuk, 90 km E Vientiane, 200 m; Collection Malicky; ♂]. —Oláh and Johanson 2010a: 22 [distribution]. —Malicky 2010a: 30 [atlas; ♂]. —Laudee and Prommi 2011: 283 [distribution]. —Malicky et al. 2018: 1321–1323 [distribution].

**Distribution.** —Laos, Thailand, Vietnam.

***poseidon*** Malicky & Chantaramongkol, 2007: 1014 [type locality: Thailand, Pu Pan NP, Kaengmoddaeng, 16°54'N 103°52'E, 400 m; Collection Malicky; ♂]. —Malicky 2010a: 26 [atlas; ♂].

**Distribution.** —Thailand.

***potosina*** Bueno-Soria, 1984: 95 [type locality: Mexico, San Luis Potosi, Palitla; NMNH; ♂]. —Moulton and Stewart 1997: 350 [distribution]. —Denning and Blickle 1971: 164 [distribution; mis-identified as *H.arctia*]. —Beardsley 1971: 15 [distribution; as *H.arctia*]. —Moulton and Stewart 1997: 350 [checklist]. —Flint et al. 2003: 31 [♂; ♀; distribution; introduced to Hawaii; correction of previous misidentification of Hawaiian specimens]. —Bowles et al. 2007: 21 [distribution; biology]. —McIntosh et al. 2002: 569 [biology]. —McIntosh et al. 2003: 298 [biology].

**Distribution.** —Mexico, U.S.A.

***priamos*** Malicky & Chantaramongkol, 2007: 1011 [type locality: Thailand, Kao Soi Dao NP, 13°06'N 102°12'E, 300 m; Collection Malicky; ♂]. —Oláh and Johanson 2010a: 22 [distribution]. —Malicky 2010a: 26 [atlas; ♂].

**Distribution.** —Laos, Thailand.

***producta*** Mosely, 1939a: 236 [type locality: Brazil, Edo. Santa Catarina, Nova Teutonia; NHMUK; ♂]. —Angrisano 1995a: 509 [distribution]. —Angrisano 1999: 32 [checklist]. —Paprocki et al. 2004: 11 [checklist]. —Dumas et al. 2009: 366 [distribution]. —Dumas and Nessimian 2012: 15 [checklist]. —Paprocki and França 2014: 45 [checklist].

**Distribution.** —Brazil, Uruguay.

***prokris*** Malicky & Chantaramongkol, 2007: 1021 [type locality: Taiwan, Taitung co., Chulai, 23°08'N 121°07'E, 370 m; Collection Malicky; ♂]. —Malicky 2014a: 1623 [checklist]. —Yang et al. 2016: 476 [checklist].

**Distribution.** —Taiwan.

***protera*** Ross, 1938a: 131 [type locality: [United States], Oklahoma, Turner Falls State Park, along Honey Creek; INHS; ♂]. —Blickle 1979: 49, 69 [checklist; ♂]. —Bowles and Mathis 1992: 32 [distribution]. —Moulton et al. 1993: 21 [distribution]. —Moulton and Stewart 1996: 101 [♂; distribution]. —Moulton and Stewart 1997: 350 [checklist].

**Distribution.** —U.S.A.

***pseudseirene*** Ito, 2015: 9 [type locality: Japan, Ryukyu Islands, Ishigaki-jima, Shiraizu, Nagura-gawa, unnamed tributary, 24°24'44"N, 124°11'11"E, 95 m; CBMI-ZI; ♂; ♀]. —Tanida and Kuranishi 2016: 71 [checklist].

**Distribution.** —Japan.

***psyche*** Malicky & Chantaramongkol, 2007: 1022 [type locality: Thailand, Doi Inthanon NP, Mae Klang bei 960 m, 18°32'N 98°34'E; Collection Malicky; ♂]. —Malicky 2010a: 32 [atlas; ♂]. —Bunlue et al. 2012: 15 [distribution].

**Distribution.** —Thailand.

***pulchricornis*** Pictet, 1834: 224 [type locality: [Switzerland]; no holotype designated]. —Kolenati 1848: 10 [revision; distribution]. —Hagen 1864b: 234 [comments on larvae and case]. —Eaton 1873: 134 [♂; distribution; as *Phrixocoma*]. —McLachlan 1880: 513 [revision; ♂]. —Morton 1899b: 281 [distribution]. —Mosely 1919a: 396 [scent-organ]. —Mosely 1923: 292 [scent-organ]. —Martynov 1924: 43 [♂]. —Martynov 1934: 137 [♂]. —Mosely 1939b: 268 [♂]. —Kimmins 1943: 154 [distribution]. —Nybom 1960: 18 [checklist]. —Spuris 1962: 62, 68 [distribution]. —Moretti and Cianficconi 1963: 199 [androconial formation]. —Spuris 1964: 13 [distribution]. —Moretti et al. 1966: 88 [distribution; note on attraction to light]. —Botosaneanu 1967: 294 [distribution]. —Solem 1970b: 93 [distribution]. —Botosaneanu and Gasith 1971: 99 [distribution]. —Spuris 1972: 19, 22, 23, 26, 28, 29 [checklist]. —Botosaneanu and Malicky 1978: 341 [checklist]. —Malicky 1983b: 43, 52 [atlas; ♂; ♀]. —Wiberg-Larsen 1985: 40 [checklist]. —Kumanski 1985: 136, 138 [♂]. —Andersen and Wiberg-Larsen 1987: 168 [checklist]. —Spuris 1989: 16 [checklist]. —Usseglio-Polatera and Bournaud 1989: 253 [distribution]. —Waringer 1989: 390 [distribution; ecology]. —Andersen et al. 1990: 26 [distribution]. —Andersen et al. 1990: 52 [distribution]. —Botosaneanu 1992: 63 [as synonym of *H.aegyptia*]. —Andersen et al. 1993b: 3 [distribution]. —Chvojka 1996: 131 [distribution]. —Nógrádi and Uherkovich 1998: 338 [distribution]. —Uherkovich and Nógrádi 1999: 420 [distribution]. —Malicky 1999c: 96 [distribution]. —Nógrádi and Uherkovich 2001: 297 [checklist]. —Nógrádi 2001: 83 [distribution; ♂]. —Gullefors 2002: 138 [checklist]. —Ujvárosi 2002: 384 [distribution]. —Cibaitė 2003a: 10 [checklist]. —Gullefors 2003: 194 [distribution]. —Malicky 2004a: 52, 64 [atlas]. —Malicky 2005: 544 [checklist]. —Gullefors 2005a: 119 [distribution]. —Gullefors 2005b: 138 [distribution]. —Lubini-Ferlin and Vicentini 2005: 67 [checklist]. —Chvojka and Komzák 2006: 358 [distribution]. —Schiess-Bühler and Rezbanyai-Reser 2006: 72 [distribution]. —Robert 2007: 82 [checklist]. —Chvojka and Komzák 2008: 13 [distribution]. —Ujvárosi et al. 2008: 112 [checklist]. —Višinskienė 2009: 27 [checklist]. —Ivanov 2011: 195 [checklist]. —Viidalepp et al. 2011: 196 [distribution]. —O’Connor 2013: 64 [distribution]. —Zuyderduyn and Tempelman 2013: 29 [distribution]. —Tempelman and Sanabria 2013b: 144 [distribution]. —O’Connor and O’Connor 2014: 273 [distribution]. —O’Connor 2015: 28, 84 [distribution]. —Pan’kov and Krasheninnikov 2016: 333 [distribution]. —Gullefors 2016: 155 [checklist]. —Morris 2016: 246 [distribution]. —Wallace 2016: 21, 23, 51 [conservation status]. —Graf et al. 2017: 48 [distribution]. —O’Connor and O’Connor 2018: 82 [distribution]. —Edmonds-Brown 2020: 91 [checklist].

**Distribution.** —Austria, Belarus, Czech Republic, Denmark, England, Estonia, Finland, France, Germany, Hungary, Ireland, Israel, Latvia, Netherlands, Norway, Romania, Russia, Scotland, Sweden, Switzerland.

***pulestoni*** Flint, 1980b: 138 [type locality: Argentina, Pcia. Buenos Aires, Estancia Delta, near Balneario Monte Hermosa; NMNH; ♂; ♀]. —Flint 1982b: 35 [distribution]. —Angrisano 1995a: 509 [distribution]. —Mangeaud 1996: 154 [distribution]. —Angrisano 1999: 32 [checklist]. —Muzón et al. 2005: 57 [distribution]. —Angrisano and Sganga 2007: 32 [♂; distribution]. —Oláh and Johanson 2011: 126 [distribution]. —Isa Miranda and Rueda Martín 2014: 199 [distribution].

**Distribution.** —Argentina, Chile, Uruguay.

***pullata*** Denning, 1947a: 150 [type locality: [United States], Wyoming, Bluegrass River, near Wheatland; ESUW; ♂; ♀; as *pullatus*]. —Blickle 1979: 49, 73 [checklist; ♂].

**Distribution.** —U.S.A.

***pyreneus*** Malicky & Chantaramongkol, 2007: 1012 [type locality: Thailand, Loei prov., Phu Luang WS, 700–900 m; Collection Malicky; ♂]. —Malicky 2010a: 28 [atlas; ♂].

**Distribution.** —Thailand.

***pythia*** Malicky & Chantaramongkol, 2007: 1015 [type locality: [Indonesia] Sumatra, Tinggi Raha, 3°09'N 98°48'E, 300 m; Collection Malicky; ♂]. —Malicky 2007a: 177 [checklist]. —Malicky 2010a: 27 [atlas; ♂].

**Distribution.** —Indonesia.

***quadrifida*** Wells, 1984: 266 [type locality: [Papua] New Guinea, SE., Kokoda, 400 m; BPBM; ♂]. —Neboiss 1986: 61 [atlas; ♂]. —Wells 1991: 526 [checklist].

**Distribution.** —Papua New Guinea.

***quinaria*** Wells & Dudgeon, 1990: 167 [type locality: Hong Kong, Tai Po Kao Forest stream; NHMUK; ♂; ♀]. —Yang et al. 2016: 476 [checklist].

**Distribution.** —Hong Kong.

***quinola*** Ross, 1947: 147 [type locality: [Canada], Ontario, Costello Lake, Station 4, Algonquin Park; INHS; ♂]. —Etnier 1968: 191 [distribution]. —Roy and Harper 1975: 1082 [distribution]. —Roy and Harper 1979: 151 [checklist]. —Blickle 1979: 49, 73 [checklist; ♂]. —Parker and Voshell 1981: 4 [checklist]. —Harris et al. 1982a: 510 [distribution]. —Harris et al. 1982b: 81 [distribution]. —Harris et al. 1984: 108 [distribution]. —Bowles and Mathis 1989: 239 [distribution]. —Morse et al. 1989: 22 [distribution]. —Harris et al. 1991: 200 [distribution]. —Frazer et al. 1991: 20 [distribution]. —Masteller and Flint 1992: 70 [checklist]. —Floyd and Morse 1993: 177 [distribution]. —Floyd et al. 1993: 90 [phenology; distribution]. —Moulton and Stewart 1996: 102 [♂; distribution]. —Abbott et al. 1997: 44 [distribution]. —Moulton and Stewart 1997: 350 [checklist]. —Floyd et al. 1997: 136 [distribution]. USA —Huyrn and Harris 2000: 193 [distribution]. —Houghton et al. 2001: 504 [distribution]. —Pescador et al. 2004: 133 [checklist]. —Flint et al. 2009:7 [distribution]. —Etnier 2010: 485 [distribution]. —Harris et al. 2012: 7 [distribution]. —Denson et al. 2016: 5 [distribution]. —Houghton 2016: 46 [biology]. —Houghton et al. 2017: 63 [checklist].

**Distribution.** —Canada, U.S.A.

***rahel*** Malicky, Ivanov, & Melnitsky, 2011: 1492 [type locality: [Indonesia], Lombok, Kembangkuning, 4 km N Kotaraja, 490 m, 8°33'33"S, 116°25'23"E; ZIN; ♂]. —Malicky et al. 2014a: 6 [distribution]. —Malicky et al. 2016: 92 [distribution].

**Distribution.** —Indonesia.

***rastrilla*** Harris & Holzenthal, 1999: 32 [type locality: Costa Rica, Limón, Reserva Biológica Barbilla, Río Dantas, 15 km (rd) S Pacuarito, 9.994°N, 83.443°W; NMNH; ♂]. —Armitage et al. 2015a: 6 [checklist]. —Armitage and Harris 2018b: 97 [checklist].

**Distribution.** —Costa Rica, Panama.

***recurvata*** Harris & Kelley, 1984: 572 [type locality: [United States], Alabama, Tuscaloosa County, Wallace Branch, 5 miles southeast of Berry; NMNH; ♂]. —Harris et al. 1991: 201 [distribution].

**Distribution.** —U.S.A.

***reducta*** Yang & Xue, 1994: 11 [type locality: [China], Sichuan, Pingwu county, 19 km E of Pingwu, tributary of Fujiang River, 1090 m; NAUJ; ♂]. —Yang et al. 1997b: 93 [checklist]. —Yang et al. 2005: 458 [checklist]. —Yang et al. 2016: 476 [checklist].

**Distribution.** —China.

***remita*** Blickle & Morse, 1954: 124 [type locality: [United States], Durham, N. H.; INHS; ♂]. —Blickle 1979: 49, 65 [checklist; ♂]. —Etnier and Schuster 1979: 17 [distribution]. —Harris et al. 1982a: 511 [distribution]. —Harris et al. 1982b: 81 [distribution]. —Harris et al. 1991: 202 [distribution]. —Masteller and Flint 1992: 70 [checklist]. —Floyd et al. 1993: 91 [phenology; distribution]. —Moulton and Stewart 1996: 102 [♂; distribution]. —Moulton and Stewart 1997: 350 [checklist]. —Abbott et al. 1997: 44 [distribution]. —Huryn and Harris 2000: 193 [distribution]. —Pescador et al. 2004: 133 [checklist]. —Etnier 2010: 485 [distribution]. —Harris et al. 2012: 7 [checklist]. —Denson et al. 2016: 5 [distribution].

**Distribution.** —U.S.A.

***rheni*** Ris, 1895: 241 [type locality: [Switzerland], am Rheinufer zwischen Rheinau und Ellikon (9 Exemplare); depository not designated; ♂]. —Ris 1897: 431 [distribution]. —Morton 1904: 325 [distribution]. —Botosaneanu 1967: 294 [distribution]. —Botosaneanu and Malicky 1978: 341 [checklist]. —Malicky 1983b: 51 [atlas; ♂]. —Urbanič 2004: 51 [distribution]. —Malicky 2004a: 56 [atlas]. —Malicky 2005b: 544 [checklist]. —Lubini-Ferlin and Vicentini 2005: 67 [checklist]. —Previšić et al. 2013: 8 [distribution].

**Distribution.** —Croatia, Slovenia, Switzerland.

***rhodica*** Jacquemart, 1973: 11 [type locality: [Greece], Loutani; IRSNB; ♂; larva]. —Malicky 1983b: 49 [atlas; ♂]. —Malicky 2004a: 61 [atlas]. —Malicky 2005b: 544 [checklist]. —Sipahiler 2005: 397 [distribution]. —Coppa and Tachet 2005: 128 [♂]. —Malicky 2005a: 62 [distribution]. —Karaouzas and Malicky 2016: 18 [distribution].

—*kumanskii* Malicky, 1974: 107 [type locality: [Greece], Kreta, Mithi; Collection Malicky; ♂]. —Botosaneanu and Malicky 1978: 341 [checklist; to synonymy].

**Distribution.** —Greece, Turkey.

***roberta*** Hamilton & Holzenthal, 1986: 165 [type locality: [United States], Georgia, Crawford County, Spring Creek below pond at Camp Eunice, approx. 5 miles SSE of Roberta (ca. 32°40'N, 83°59'W); NMNH; ♂].

**Distribution.** —U.S.A.

***robusta*** Wells, 1978: 747 [type locality: [Australia] Victoria, Millgrove, Yarra River; NMV; ♂; ♀]. —Wells 1985b: 5 [larva; case]. —Neboiss 1986: 60 [atlas; ♂; ♀].

**Distribution.** —Australia.

***roma*** Malicky & Chantaramongkol, 2007: 1013 [type locality: Thailand, Tung Salaeng NP, 16°49'N 100°57'E, 600 m; Collection Malicky; ♂]. —Malicky 2010a: 28 [atlas; ♂].

**Distribution.** —Thailand.

***rono*** Ross, 1941a: 66 [type locality: United States, Utah, Huntsville; INHS; ♂; ♀]. —Ross and Spencer 1952: 47 [distribution]. —Roy and Harper 1979: 151 [checklist]. —Blickle 1979: 49, 67 [checklist; ♂]. —Unzicker et al. 1982: 9 [checklist]. —Waltz and McCafferty 1983b: 354 [distribution]. —Waltz and McCafferty 1983c: 414 [distribution]. —Light and Adler 1983: 77 [distribution; biology]. —Hamilton et al. 1983: 18 [distribution]. —Bueno-Soria 1984: 93 [♂; distribution]. —Harper 1989: 541 [distribution]. —Masteller and Flint 1992: 70 [checklist]. —Moulton et al. 1994: 169 [distribution]. —Wiggins and Parker 1997: 794 [distribution]. —Moulton and Stewart 1997: 350 [checklist]. —Houghton 2001: 90 [distribution]. —Houghton et al. 2001: 504 [distribution]. —Blinn and Ruiter 2005: 68 [distribution; biology]. —Blinn and Ruiter 2006: 332 [biology; distribution]. —Blinn and Ruiter 2009b: 186 [phenology; distribution]. —Vieira et al. 2009: 257 [distribution]. —Blinn and Ruiter 2013: 279, 291 [distribution; biology]. —Givens 2014: 158 [distribution]. —Mendez et al. 2019: 118 [checklist].

**Distribution.** —Canada, Mexico, U.S.A.

***roperi*** Wells & Dostine, 2016: 592 [type locality: [Australia] Northern Territory, Roper River, McMinn Station; NTM; ♂].

**Distribution.** —Australia.

***ruben*** Malicky, Ivanov, & Melnitsky, 2011: 1493 [type locality: [Indonesia], Bali, Munduk, Bali Cottages, 860 m, 8°15'48"S, 115°08'42"E; ZIN; ♂]. —Malicky et al. 2014a: 6 [distribution].

**Distribution.** —Indonesia.

***ruffoi*** Moretti, 1981: 171 [type locality: [Italy], Abruzzi, Monti della Laga, Rio Castellana, 1070 m, Teramo; Collection Moretti; ♂]. —Moretti and Cianficconi 1981: 201 [checklist]. —Malicky 1983b: 42 [atlas; ♂]. —Valle 2001: 67, 83 [distribution; ♀]. —Malicky 2004a: 51 [atlas]. —Cianficconi et al. 2004b: 330 [distribution]. —Cianficconi et al. 2005: 96 [habitat; distribution]. —Malicky 2005b: 544 [checklist]. —Cianficconi et al. 2007a: 67 [proposed as Italian endemic]. —Cianficconi et al. 2007b: 569, 575 [distribution]. —Corallini et al. 2013a: 38 [checklist]. —Cianficconi et al. 2016: 141 [distribution]. —Valle and Lodovici 2018: 146 [distribution]. —Le Guellec et al. 2020: 139 [distribution].

**Distribution.** —France, Italy.

***rumpun*** Wells & Huisman, 1992: 100 [type locality: West Malaysia, Genting Highlands, tributary Sg. Gombak; NTM; ♂]. —Wells and Malicky 1997: 184 [♂; distribution]. —Malicky and Chantaramongkol 2007: 1023 [distribution]. —Malicky 2007a: 177 [checklist]. —Malicky 2010a: 24 [atlas; ♂]. —Malicky et al. 2014a: 6 [distribution]. —Malicky et al. 2016: 92 [distribution]. —Malicky et al. 2018: 1322, 1323 [distribution]. —Melnitsky et al. 2019: 539 [distribution]. —Malicky et al. 2019: 429 [distribution].

**Distribution.** —Indonesia, Malaysia, Thailand.

***sabit*** Wells & Huisman, 1992: 106 [type locality: West Malaysia, Genting Highlands, Gombak, tributary Sg. Gombak; NTM; ♂]. —Wells and Malicky 1997: 184 [♂; distribution]. —Malicky and Chantaramongkol 2007: 1023 [distribution]. —Malicky 2007a: 177 [checklist]. —Malicky 2010a: 25 [atlas; ♂]. —Malicky et al. 2018: 1322 [distribution].

—*phanla* Oláh & Johanson, 2010a: 20 [type locality: Laos, Luang Namcha Prov., Tong Om Village, UTM: 47Q 0750111 2321825, 552 m; NHRS; ♂]. —Malicky 2013: 43 [to synonymy].

**Distribution.** —Indonesia, Laos, Malaysia, Thailand.

***saimbeyli*** Sipahiler, 2018: 38 [type locality: Turkey, Adana, 11 km south of Saimbeyli, Feke direction, Göksu River, 1000 m, 37°51'N, 35°59'E; HUAT; ♂].

**Distribution.** —Turkey.

***salmo*** Ross, 1941a: 66 [type locality: [United States], Wisconsin, Trout Lake; INHS; ♂]. —Etnier 1965: 147 [distribution]. —Blickle 1979: 49, 69 [checklist; ♂]. —Roy and Harper 1979: 151 [distribution]. —Roy and Harper 1981: 105 [distribution]. —Ruiter 1999: 165 [distribution]. —Houghton et al. 2001: 504 [distribution]. —Armitage et al. 2011: 14 [checklist]. —Houghton et al. 2017: 63 [checklist]. —Mendez et al. 2019: 128 [checklist].

**Distribution.** —Canda, U.S.A.

***sandersoni*** Mathis & Bowles, 1990: 88 [type locality: [United States], Arkansas, Stone County, Sylamore Creek, Gunner Pool Recreation Area; NMNH; ♂]. —Harris et al. 1991: 203 [distribution]. —Floyd 1992: 50 [distribution]. —Bowles and Mathis 1992: 32 [distribution]. —Moulton and Stewart 1996: 102 [♂; distribution]. —Etnier 2010: 485 [distribution]. —Armitage et al. 2011: 14 [checklist]. —Bowles et al. 2020: 8 [distribution].

**Distribution.** —U.S.A.

***sanghala*** Schmid, 1960: 95 [type locality: [Pakistan], Hindou-Kouch, Shogor; CNC; ♂]. —Schmid 1958c: 220 [as new species *nomen nudum*]. —Schmid 1959b: 686 [distribution]. —Malicky 1983b: 50 [atlas; ♂]. —Mirmoayedi and Malicky 2002: 164 [checklist]. —Malicky 2004a: 62 [atlas]. —Malicky 2005b: 544 [checklist]. —Huang et al. 2005: 469 [distribution]. —Malicky 2006: 252 [checklist]. —Malicky and Chantaramongkol 2007: 1023 [distribution]. —Malicky 2010a: 25 [atlas; ♂]. —Mattern 2015: 501 [distribution]. —Yang et al. 2016: 476 [checklist]. —Malicky 2018: 49 [checklist]. —Lonsdale 2020: 39 [holotype depository].

**Distribution.** —China, Iran, Nepal, Pakistan, Thailand.

***santarosa*** Harris, Rasmussen, & Denson, 2012: 3 [type locality: [United States], Florida, Santa Rosa Co., McCostill Mill Creek at Ebenezeer Church Road, N30°55'06", W87°14'50"; NMNH; ♂].

**Distribution.** —U.S.A.

***sarahae*** Harris, 2002: 52 [type locality: [United States], Florida, Okaloosa County, Rogue Creek, 0.6 km S Base Rd. 232, Eglin Air Force Base, 30°33'19"N, 86°34'51"W; NMNH; ♂]. —Pescador et al. 2004: 133 [checklist]. —Harris et al. 2012: 7 [checklist].

**Distribution.** —U.S.A.

***sarkos*** Oláh & Johanson, 2011: 126 [type locality: Peru, San Martin Prov., creek crossing rd. Tarapoto-Yurimaguas, ca. 30 km (rd.) NE Tarapoto, 6°24.904'S 76°18.756'W; NHRS; ♂].

**Distribution.** —Peru.

***sauca*** Flint, 1980b: 141 [type locality: Argentina, Pcia. Buenos Aires, Rio Sauce Grande, Sierra de la Ventana; NMNH; ♂; ♀]. —Flint 1982b: 35 [distribution]. —Angrisano 1995a: 509 [distribution]. —Angrisano 1999: 32 [checklist]. —Angrisano and Sganga 2007: 32 [♂; distribution].

**Distribution.** —Argentina, Uruguay.

***scamandra*** Neboiss, 1977: 41 [type locality: [Australia] Tasmania, Scamander River, Upper Scamander; NMV; ♂]. —Wells 1978: 751 [♂; ♀; distribution]. —Wells 1985b: 5 [larva; pupa; case]. —Neboiss 1986: 63 [atlas; ♂; ♀]. —Neboiss 2002: 52 [checklist]. —Oláh and Johanson 2010a: 22 [distribution].

**Distribution.** —Australia.

***scheiringi*** Harris, 1986a: 609 [type locality: [United States], Alabama, Baldwin County, Pine Log Creek at Hwy. 59; NMNH; ♂]. —Harris et al. 1991: 204 [distribution]. —Harris et al. 2012: 7 [distribution].

**Distribution.** —U.S.A.

***scolops*** Ross, 1938a: 128 [type locality: [United States], Illinois, Shawneetown; INHS; ♂]. —Ross 1944: 152 [♂; distribution]. —Etnier 1965: 147 [distribution]. —Blickle 1979: 49, 71 [checklist; ♂]. —Hamilton et al. 1983: 18 [distribution]. —Moulton and Stewart 1996: 103 [♂; distribution]. —Abbott et al. 1997: 44 [distribution]. —Moulton and Stewart 1997: 350 [checklist]. —Houghton et al. 2001: 504 [distribution]. —Houghton et al. 2017: 63 [checklist].

**Distribution.** —U.S.A.

***sederhana*** Wells & Huisman, 1992: 99 [type locality: East Malaysia, Sabah, 60 km W Lahad Datu, Sg. Segama, 04°58'N 117°48'E, 150 m; RMNH; ♂]. —Malicky 2010a: 33 [atlas; ♂].

**Distribution.** —East Malaysia.

***segitiga*** Wells & Huisman, 1992: 98 [type locality: West Malaysia, Genting Highlands, tributary Sg. Gombak; NTM; ♂]. —Malicky 2010a: 27 [atlas; ♂].

**Distribution.** —West Malaysia.

***seirene*** Malicky & Chantaramongkol, 2007: 1022 [type locality: Taiwan, Nantou co., W Tatung, 24°01'N 121°05'E, 880 m; Collection Malicky; ♂]. —Malicky 2014a: 1623 [checklist]. —Ito 2015: 8 [♂; distribution]. —Yang et al. 2016: 476 [checklist].

**Distribution.** —Taiwan.

***selene*** Malicky, 2008a: 837 [type locality: [Indonesia, Borneo, Kalimantan], im Einzugsbereich der Flüsse Seturan und Rian in einem engen Bereich von ungefähr 8 × 8 km ca. 70 km südlich der Stadt Malinau, 116°29'48"–116°33'29"E, 2°59'29"–3°04'04"N, 100–200 m; MZLS; ♂]. —Malicky 2010a: 29 [atlas; ♂].

**Distribution.** —Indonesia.

***selvatica*** Botosaneanu, 1977: 269 [type locality: Cuba, Oriente, Baire, petit tuisseau, affluent de Rio Brazo Seco, a Matias; NMNH; ♂]. —Botosaneanu 1979: 51 [distribution]. —Flint 1996a: 16 [checklist]. —Botosaneanu 2002b: 83 [checklist]. —Naranjo López and González Lazo 2005: 149 [checklist].

**Distribution.** —Cuba.

***sengavi*** Schmid, 1960: 93 [type locality: [Pakistan] Karakoram, Gilgit; CNC; ♂]. —Schmid 1958c: 220 [as new species, *nomen nudum*; distribution]. —Lonsdale 2020: 39 [holotype depository].

**Distribution.** —Pakistan.

***serrata*** Morton, 1898: 108 [type locality: [Algeria]; NHMUK; ♂]. —Morton 1904: 325 [distribution]. —Kimmins 1957a: 108 [lectotype designation]. —Malicky 1981a: 183 [♂]. —Moretti and Cianficconi 1981: 201 [checklist]. —Malicky 1983b: 44 [atlas; ♂]. —Botosaneanu and Dumont 1987: 119 [♂; ♀]. —Malicky and Lounaci 1987: 15 [checklist]. —Malicky 2004a: 55 [atlas]. —Malicky 2005b: 544 [checklist]. —Cianficconi et al. 2007a: 67 [proposed as Italian endemic]. —Corallini et al. 2013a: 38 [checklist]. —Cianficconi et al. 2016: 141 [distribution].

—*bifurcata* Mosely, 1930a: 178 [type locality: [France], Corsica, Corte; NHMUK; ♂; ♀]. —Botosaneanu 1967: 294 [distribution]. —Botosaneanu and Malicky 1978: 340 [checklist]. —Malicky 1981a: 183 [suggested synonymy]. —Botosaneanu 1982a: 178 [as distinct species]. —Malicky 1988a: 21 [to synonymy]. —Cianficconi et al. 2002: 146 [distribution].

**Distribution.** —Algeria, Italy, France.

***setigera*** Wells, 1984: 270 [type locality: [Papua] New Guinea, NE., Banz, Waghi Valley, 1500 m; BPBM; ♂]. —Neboiss 1986: 62 [atlas; ♂]. —Wells 1991: 526 [checklist].

**Distribution.** —Papua New Guinea.

***sidong*** Oláh, 1989: 285 [type locality: Vietnam, Tamdao, 200 m a.s.l.; HNHM; ♂]. —Armitage et al. 2005: 27 [checklist]. —Malicky and Chantaramongkol 2007: 1023 [distribution]. —Zhou et al. 2009b: 356 [♂; distribution]. —Malicky 2010a: 23 [atlas; ♂]. —Yang et al. 2016: 476 [checklist].

—*tiani* Yang & Xue, 1992: 28 [type locality: [China] Yellow Mountain, Anhui; NAUJ; ♂]. —Yang et al. 1997b: 93 [checklist]. —Yang et al. 2005: 458 [checklist]. —Malicky 2013: 43 [to synonymy].

**Distribution.** —China, Vietnam.

***sikanda*** González & Malicky, 1988: 66 [type locality: Espagne, Prov. Càdiz, Benamahoma, Arroyo del Descansadero, 400 m; depository not designated; ♂]. —González et al. 1990: 213 [checklist]. —Malicky 2004a: 55 [atlas]. —Malicky 2005b: 544 [checklist]. —González and Menéndez 2011: 119 [distribution].

**Distribution.** —Spain.

***silicula*** Flint & Reyes, 1991: 484 [type locality: Peru, Dept. Lambayeque, Río Saña, Saña near ruins of Corbacho; NMNH; ♂].

**Distribution.** —Peru.

***simplex*** Nielsen in Berg 1948: 125 [type locality: [Denmark], Holløse; likely deposited at ZMUC; ♂]. —Botosaneanu and Malicky 1978: 341 [checklist]. —Malicky 2005b: 544 [checklist]. —Malicky 2014b: 6 [teratological structures; possible junior synonym of *H.sparsa*].

**Distribution.** —Denmark.

***simulans*** Mosely, 1919b: 391 [type locality: [England], river Test, Hampshire; no depository designated; ♂]. —Mosely 1919a: 395 [scent-organ]. —Tjeder 1930b: 201 [distribution]. —Racięcka 1936: 98 [distribution]. —Mosely 1939b: 260 [♂]. —Schmid 1959b: 691 [distribution (erroneously, according to Mirmoayedi and Malicky 2002: 164)]. —Nybom 1960: 17 [checklist]. —Botosaneanu 1967: 294 [distribution]. —Botosaneanu and Gasith 1971: 98 [distribution]. —Spuris 1972: 28, 30 [checklist]. —Malicky 1974: 122 [checklist]. —Botosaneanu and Malicky 1978: 341 [checklist]. —Kumanski 1979: 10 [♂; distribution]. —Moretti and Cianficconi 1981: 201 [checklist]. —Malicky 1983b: 46, 52 [atlas; ♂; ♀]. —Kumanski and Malicky 1984: 199 [distribution]. —Nógrádi 1985: 131 [distribution; ♂]. —Wiberg-Larsen 1985: 40 [checklist]. —Andersen and Tysse 1985: 84 [distribution]. —Kumanski 1985: 126 [♂]. —Nógrádi 1986: 139 [distribution]. —Sipahiler and Malicky 1987: 122, 129 [distribution]. —Andersen and Wiberg-Larsen 1987: 168 [checklist]. —Rojas-Camousseight and Tachet 1988: 310–314 [♀]. —Usseglio-Polatera and Bournaud 1989: 253 [distribution]. —Spuris 1989: 16 [checklist]. —Krušnik 1991: 13 [distribution]. —Botosaneanu 1992: 63 [as synonym of *H.angustata*]. —Andersen et al. 1993b: 3 [distribution]. —Andersen et al. 1993a: 51 [distribution]. —Nógrádi and Uherkovich 1994: 31 [distribution]. —Nógrádi 1994: 277 [♂; ♀]. —Maier et al. 1995: 147 [distribution]. —Brettfeld 1996: 127 [distribution]. —Malicky 1997: 144 [distribution; ♂]. —Peissner and Kappus 1998: 162, 164 [distribution]. —Nógrádi and Uherkovich 2001: 297 [checklist]. —Valle 2001: 67 [distribution]. —Gullefors 2002: 138 [checklist]. —Ujvárosi 2002: 384 [distribution]. —Mirmoayedi and Malicky 2002: 164 [distribution correction]. —Cibaitė 2003a: 10 [checklist]. —Gullefors 2003: 194 [distribution]. —Malicky 2004a: 57 [atlas]. —Malicky 2005b: 544 [checklist]. —Gullefors 2005b: 138 [distribution]. —Sipahiler 2005: 397 [distribution]. —Malicky 2005a: 62 [distribution]. —Coppa and Tachet 2005: 132 [distribution]. —Lubini-Ferlin and Vicentini 2005: 67 [checklist]. —Hohmann 2005: 106 [checklist]. —Gullefors 2006: 137 [distribution]. —Chvojka and Komzák 2006: 358 [distribution]. —Schiess-Bühler and Rezbanyai-Reser 2006: 72 [distribution]. —Robert 2007: 82 [checklist]. —Cianficconi et al. 2007b: 569, 575 [distribution]. —Chvojka and Komzák 2008: 13 [distribution]. —Ujvárosi et al. 2008: 112 [checklist]. —Schrankel et al. 2008: 90 [checklist]. —Gullefors 2008: 64 [checklist]. —Višinskienė 2009: 27 [checklist]. —Neu 2010: 151 [♀]. —Cianficconi and Corallini 2010: 87 [distribution]. —Corallini and Cianficconi 2011: 628 [checklist]. —Ivanov 2011: 195 [checklist]. —Cianficconi et al. 2011: 47 [distribution]. —Viidalepp et al. 2011: 196 [distribution]. —Lock and Goethals 2012: 28 [checklist]. —O’Connor 2013: 64 [distribution]. —Corallini et al. 2013a: 38 [checklist]. —Corallini et al. 2013b: 26 [distribution]. —Hohmann et al. 2014: 85 [distribution]. —Malicky 2014b: 6 [teratological structures]. —Karaouzas and Malicky 2015: 14 [distribution]. —O’Connor 2015: 28, 86 [distribution]. —Sipahiler 2016: 13 [distribution]. —Cianficconi et al. 2016: 141 [distribution]. —Pan’kov and Krasheninnikov 2016: 333 [distribution]. —Chvojka et al. 2016: 44 [distribution]. —Küttner et al. 2016: 179 [distribution]. —Sipahiler 2016: 13 [distribution]. —Gullefors 2016: 155 [checklist]. —Wallace 2016: 21, 24 [conservation status]. —Sipahiler 2017b: 13 [distribution]. —O’Connor and O’Connor 2017b: 53 [distribution]. —Lock and van Butsel 2017: 33 [distribution; ♂; ♀]. —O’Connor and O’Connor 2018: 82 [distribution]. —O’Connor and Bond 2018: 193 [distribution]. —Sipahiler 2018: 41 [distribution]. —Kučinić et. al. 2019: 450 [distribution]. —O’Connor 2020: 140 [distribution].

**Distribution.** —Austria, Belgium, Croatia, Czech Republic, Bulgaria, Denmark, England, Estonia, Finland, France, Germany, Greece, Hungary, Ireland, Israel, Italy, Lithuania, Luxembourg, Norway, Romania, Russia, Slovenia, Sweden, Switzerland, Turkey.

***simulauica*** Mey, 1998a: 555 [type locality: [Philippines, Mindanao], northern slope of Mt. Atuuganon range, 1050 m; ZMHB; ♂]. —Wells and Mey 2002: 134 [checklist].

**Distribution.** —Philippines.

***singri*** Harris & Holzenthal, 1999: 34 [type locality: Costa Ria, Puntarenas, Río Singrí, ca 2 km (air) S Finca Helechales, 9.057°N, 83.082°W; NMNH; ♂]. —Armitage et al. 2016: 7 [distribution]. —Armitage and Harris 2018b: 97 [checklist]. —Armitage and Harris 2018c: 283 [distribution]. —Harris and Armitage 2019: 4 [distribution].

**Distribution.** —Costa Rica, Panama.

***sinuosa*** Wells, 1978: 759 [type locality: [Australia] Queensland, Little Mulgrave River; ANIC; ♂]. —Neboiss 1986: 62 [atlas; ♂].

**Distribution.** —Australia.

***sitahoan*** Malicky & Chantaramongkol, 2007: 1017 [type locality: [Indonesia] Sumatra, Tinggi Raja, 3°09'N 98°47'E, 1500 m; Collection Malicky; ♂]. —Malicky 2007a: 177 [checklist]. —Malicky 2010a: 23 [atlas; ♂].

**Distribution.** —Indonesia.

***skylla*** Malicky & Chantaramongkol, 2007: 1020 [type locality: Thailand, Tung Yaw, 19°08'N 98°39'E, 1200 m; Collection Malicky; ♂]. —Malicky 2010a: 25 [atlas; ♂].

**Distribution.** —Thailand.

***spada*** Flint, 1991b: 47 [type locality: Colombia, Dpto. Antioquia, Quebrada Espadera, 7 km E Medellín, road to Sta. Elena; NMNH; ♂]. —Muñoz-Quesada 2000: 278 [checklist]. —Ríos-Touma et al. 2017: 10 [distribution].

**Distribution.** —Colombia, Ecuador.

***spangleri*** Bueno-Soria, 1984: 113 [type locality: Guatemala, Matias de Galvez; NMNH; ♂].

**Distribution.** —Guatemala.

***sparsa*** Curtis, 1834: 217 [type locality: [England], “Britain”; NMV; ♀]. —Stephens 1836: 152 [distribution]. —Eaton 1873: 133 [♂; ♀; distribution; as *Phrixocoma*]. —McLachlan 1880: 511 [revision; ♂; ♀]. —McLachlan 1884: 70 [distribution]. —Morton 1896: 102 [distribution]. —Ris 1897: 431 [distribution]. —Klapálek 1897: 1 [larva]. —Klapálek 1900b: 3 [distribution]. —Ris 1903: 16 [distribution]. —Morton 1904: 324 [distribution]. —Mosely 1919a: plate XVII [♂]. —Mosely 1919b: 395 [scent-organ]. —Martynov 1924: 42 [♂]. —Martynov 1934: 126 [♂]. —Racięcka 1936: 98 [♀; ♂]. —Mosely 1939b: 258 [♂]. —Berg 1948: table 14 [distribution]. —Schmid 1959b: 690 [distribution]. —Nybom 1960: 17 [checklist]. —Hanna 1961: 69 [larva; distribution]. —Neboiss 1963: 620 [lectoholotype designated]. —Botosaneanu 1967: 294 [distribution]. —Botosaneanu and Gasith 1971: 98 [distribution]. —Spuris 1972: 27, 30 [checklist]. —Szczęsny 1975: 41 [distribution]. —Botosaneanu and Malicky 1978: 341 [checklist]. —Mey 1978a: 122 [distribution]. —Moretti and Corallini-Sorcetti 1978: 36 [ecology]. —Kumanski 1979: 12 [♂; distribution]. —Moretti and Cianficconi 1981: 201 [checklist]. —Moretti et al. 1981: 239 [ecology; distribution]. —Malicky 1983b: 46, 47, 52 [atlas; ♂; ♀]. —Moubayed and Botosaneanu 1985: 63 [distribution]. —Wiberg-Larsen 1985: 40 [checklist]. —Kumanski 1985: 120 [♂]. —Glapska 1986: 30 [distribution]. —Cooter 1987: 148 [distribution]. —Andersen and Wiberg-Larsen 1987: 168 [checklist]. —Sipahiler and Malicky 1987: 129 [distribution]. —Rojas-Camousseight and Tachet 1988: 311–314 [♀]. —Spuris 1989: 16 [checklist]. —Waringer 1989: 390 [distribution; ecology]. —Usseglio-Polatera and Bournaud 1989: 253 [distribution]. —Krušnik 1991: 13 [distribution]. —Botosaneanu 1992: 61 [♂; ♀; wings]. —Nógrádi and Uherkovich 1994: 31 [distribution]. —Nógrádi 1994: 277 [♂ ♀]. —Haase 1994: 206 [distribution]. —Uherkovich and Nógrádi 1997: 461 [distribution]. —Malicky 1997: 1345 [distribution; ♂]. —Uherkovich and Nógrádi 1998: 52 [distribution]. —Nógrádi and Uherkovich 1998: 338 [distribution]. —Peissner and Kappus 1998: 162 [distribution]. —Uherkovich and Nógrádi 1999: 420 [distribution]. —Cianficconi et al. 1999a: 57 [distribution]. —Wiberg-Larsen and Karsholt 1999: 126 [distribution]. —Hohmann 1999: 35 [checklist]. —Malicky 1999c: 96 [distribution]. —Cianficconi et al. 1999b: 279 [distribution]. —Uherkovich and Nógrádi 2001: 95 [distribution]. —Nógrádi and Uherkovich 2001: 297 [checklist]. —Mirmoayedi and Malicky 2002: 164 [checklist]. —Cianficconi et al. 2002: 146 [distribution]. —Gullefors 2002: 138 [checklist]. —Nógrádi and Uherkovich 2002: 130 [distribution]. —Cibaitė 2003a: 10 [checklist]. —Malicky 2004a: 57, 64 [atlas]. —Cianficconi et al. 2004a: 256, 258 [distribution; biology]. —Graf and Hutter 2004: 147 [distribution]. —Malicky 2005a: 62 [distribution]. —Berlin 2005: 129 [distribution]. —Malicky 2005b: 545 [checklist]. —Coppa and Tachet 2005: 132 [distribution]. —Graf et al. 2005: 55 [distribution]. —Sipahiler 2005: 397 [distribution]. —Lubini-Ferlin and Vicentini 2005: 67 [checklist]. —Hohmann 2005: 106 [checklist]. —Wiggers et al. 2006: 54 [distribution]. —Waringer and Graf 2006: 356 [distribution]. —Chvojka and Komzák 2006: 358 [distribution]. —Mey 2006a: 159 [distribution]. —Schiess-Bühler and Rezbanyai-Reser 2006: 72 [distribution]. —Robert 2007: 82 [checklist]. —Cianficconi et al. 2007b: 569, 576 [distribution]. —Berlin and Thiele 2007: 49 [checklist]. —Previšić et al. 2007: 184 [distribution]. —Szczęsny and Godunko 2008: 15 [checklist]. —González and Menéndez 2008: 188 [distribution]. —Waringer and Graf 2008: 142 [distribution]. —Chvojka and Komzák 2008: 13 [distribution]. —Ujvárosi et al. 2008: 112 [checklist]. —Schrankel et al. 2008: 90 [checklist]. —Višinskienė 2009: 27 [checklist]. —O’Connor and Bond 2009: 131 [distribution]. —Neu 2010: 151 [♀]. —Hohmann 2010: 40 [distribution]. —Ivanov 2011: 195 [checklist]. —Kučinić et al. 2011: 260, 263 [distribution; biology]. —Corallini and Cianficconi 2011: 628 [checklist]. —Valladolid et al. 2011: 501 [distribution]. —Cianficconi et al. 2011: 47 [distribution]. —González and Menéndez 2011: 119 [distribution]. —Viidalepp et al. 2011: 196 [distribution]. —Komzák and Chvojka 2012: 720 [distribution]. —Kiss 2012: 28 [distribution]. —O’Connor 2013: 64 [distribution]. —Corallini et al. 2013a: 38 [checklist]. —Tempelman et al. 2013: 288 [distribution]. —Zuyderduyn and Tempelman 2013: 25 [distribution]. —Tempelman and Sanabria 2013a: 20 [distribution]. —Tempelman and Sanabria 2013b: 144 [distribution]. —Hohmann et al. 2014: 85 [distribution]. —Mey 2014: 187 [distribution]. —O’Connor and O’Connor 2014: 273 [distribution]. —Malicky 2014b: 10 [teratological structures]. —Wolf and Angersbach 2014: 32 [distribution]. —Karaouzas and Malicky 2015: 14, 18 [distribution]. —O’Connor 2015: 28, 87 [distribution]. —Stanić-Koštroman et al. 2015: 85 [distribution]. —Stojanović et al. 2015: 55 [distribution]. —O’Connor and O’Connor 2015: 203 [distribution]. —Dia 2015: 51 [distribution]. —Corallini and Bicchierai 2016: 151 [biology]. —Cianficconi et al. 2016: 142 [distribution]. —Pan’kov and Krasheninnikov 2016: 333 [distribution]. —O’Connor and O’Connor 2016: 166 [distribution]. —Gullefors 2016: 155 [checklist]. —Valle and Lodovici 2018: 146 [distribution]. —O’Connor and O’Connor 2018: 82 [distribution]. —Sipahiler 2018: 41 [distribution]. —Kučinić et. al. 2019: 450 [distribution]. —Edmonds-Brown 2020: 90, 91 [life history; checklist]. —Kroča and Komzák 2020: 147 [distribution]. —O’Connor 2020: 140 [distribution]. —Navara et al. 2020: 46 [distribution].

—*brunneicornis* Stephens, 1836: 152 [type locality: [England], near London; no type designated]. —Kolenati 1848: 106 [revision; distribution]. —Fischer 1961: 163 [to synonymy].

—*recurva* Dalman [unpublished manuscript name mentioned in Kolenati 1848: 106; no further information known]. —Kolenati 1848: 106 [to synonymy with *brunneicornis*].

**Distribution.** —Algeria, Austria, Belarus, Bosnia-Herzegovina, Bulgaria, Croatia, Czech Republic, Denmark, England, Estonia, Finland, France, Germany, Greece, Hungary, Iran, Ireland, Israel, Italy, Lebanon, Luxembourg, Netherlands, Poland, Portugal, Republic of Croatia, Serbia, Romania, Russia, Serbia, Slovakia, Slovenia, Spain, Sweden, Switzerland, Turkey, Ukraine.

***spatulata*** Morton, 1905: 66 [type locality: [United States], Ithaca, New York; depository not designated; ♂]. —Banks 1907a: 50 [catalogue]. —Betten 1934: 160 [♂; distribution]. —Ross 1944: 148 [♂; larva; distribution]. —Denning 1947b: 173 [distribution]. —Etnier 1965: 147 [checklist]. —Roy and Harper 1975: 1083 [distribution]. —Roy and Harper 1979: 151 [checklist]. —Etnier and Schuster 1979: 18 [distribution]. —Blickle 1979: 49, 65 [checklist; ♂]. —Parker and Voshell 1981: 4 [checklist]. —Swegman et al. 1981: 132 [distribution]. —Roy and Harper 1981: 105 [distribution]. —Huryn and Foote 1983: 790 [distribution]. —Waltz and McCafferty 1983a: 10 [distribution]. —Harris et al. 1984: 108 [distribution]. —Lake 1984: 220 [distribution]. —Bowles and Mathis 1989: 239 [distribution]. —Harris et al. 1991: 206 [distribution]. —Masteller and Flint 1992: 70 [checklist]. —Mathis and Bowles 1992: 24 [distribution]. —Bowles and Mathis 1992: 32 [distribution]. —Masteller 1993: 134 [distribution]. —Moulton and Stewart 1996: 103 [♂; distribution]. —Houghton et al. 2001: 504 [distribution]. —Etnier 2010: 485 [distribution]. —Houghton et al. 2011b: 5 [phenology; habitat; distribution]. —Flint 2011: 104 [distribution]. —Armitage et al. 2011: 14 [checklist]. —Ruiter Boyule and Zhou 2013: 3 [distribution; DNA barcoding; larval-adult association]. —DeWalt et al. 2016: 51 [distribution]. —Houghton et al. 2017: 63 [checklist].

**Distribution.** —Canada, U.S.A.

***sphinx*** Malicky & Chantaramongkol, 2007: 1017 [type locality: [Indonesia] Sumatra, Tinggi Raja, 3°09'N 98°47'E, 350 m; Collection Malicky; ♂]. —Malicky 2007a: 177 [checklist]. —Malicky 2010a: 23 [atlas; ♂]. —Malicky et al. 2014a: 6 [distribution]. —Malicky et al. 2016: 92 [distribution].

**Distribution.** —Indonesia.

***spinata*** Blickle & Morse, 1954: 123 [type locality: [United States], Lee, N. H.; INHS; ♂]. —Williams and Williams 1979: 2406 [distribution]. —Blickle 1979: 49, 67 [checklist; ♂]. —Parker and Voshell 1981: 4 [checklist]. —Swegman et al. 1981: 132 [distribution]. —Roy and Harper 1981: 105 [distribution]. —Harris et al. 1991: 207 [distribution]. —Masteller and Flint 1992: 70 [checklist]. —Myers et al. 2011: 107 [distribution].

**Distribution.** —Canada, U.S.A.

***spinosa*** Arefina & Armitage, 2003: 15 [type locality: [Russia] Central Sakhalin, middle part of Tym’ River, 20 km SW from Yasnoye Village; IBSS-RAS; ♂; ♀]. —Ito et al. 2011: 12 [♂, ♀; distribution]. —Ivanov 2011: 195 [checklist]. —Malicky 2014a: 1610 [possible senior synonym to *Hydroptilaintrospinata*]. —Ito and Nagasaka 2014: 9 [distribution]. —Ito 2015: 8 [checklist]. —Vshivkova et al. 2016: 78, 79 [distribution]. —Tanida and Kuranishi 2016: 71 [checklist]. —Potikha and Vshivkova 2016: 364 [distribution].

**Distribution.** —Japan, Russia.

***spiralis*** Ito, 2015: 11 [type locality: Japan, Ryukyu Islands, Okinawa-jima, Kunigami-son, Yona, Yona-gawa, Heigi-hashi, 26°45'41"N, 128°13'09"E, 33 m; CBMI-ZI; ♂; ♀]. —Tanida and Kuranishi 2016: 71 [checklist].

**Distribution.** —Japan.

***spirula*** Bueno-Soria, 1984: 121 [type locality: Mexico, Michoacán, Carácuaro; NHMUK; ♂; senior homonym of *H.spirula* Wells & Mey, 2002: 126, replaced by *H.spirulatella* Wells & Mey, 2003: 427].

**Distribution.** —Mexico.

***spirulatella*** Mey, 2003b: 427 [replacement name for *H.spirula* Wells & Mey, 2002: 126, preoccupied by *H.spirula* Bueno-Soria, 1984: 121] [type locality: [Philippines] Palawan, Cayasan, Babuyan Rivber, LF; ZMHB; ♂]. —Mey and Freitag 2020: 57 [distribution].

**Distribution.** —Philippines.

***spurcaria*** Mey, 1998a: 553 [type locality: [Philippines, Mindanao], northern slope of Mt. Atuuganon range, 1050 m; ZMHB; ♂]. —Wells and Mey 2002: 134 [checklist].

**Distribution.** —Philippines.

***srisungwan*** Malicky & Chantaramongkol, 2007: 1017 [type locality: Thailand, Mae Ping bei Ban Sop O Nok, 8 km S von Chiang Dao, 19°16'N 98°58'E, 370 m; Collection Malicky; ♂]. —Malicky 2010a: 31 [atlas; ♂].

**Distribution.** —Thailand.

***starmuehlneri*** Marlier & Marlier, 1982: 17 [type locality: La Réunion, Station 80, même station que 29–30 [Route St. Benoît-Takamaka, ravine Sèche en amont du pont, 550 m, ruisseau torrentueux, sur rochers], chasse à la lumière; IRSNB; ♂; larva]. —Botosaneanu 2002a: 327 [♂].

**Distribution.** —Réunion.

***stellifera*** Morton, 1893: 75 [type locality: Italy, Apennino Pistojese; NHMUK; ♂]. —Kimmins 1957a: 108 [lectotype designation]. —Botosaneanu 1967: 294 [distribution]. —Botosaneanu and Malicky 1978: 341 [checklist]. —Moretti and Cianficconi 1981: 201 [checklist]. —Malicky 1981a: 183 [♂]. —Malicky 1983b: 44 [atlas; ♂]. —Malicky 2002: 4 [distribution]. —Malicky 2004a: 55 [atlas]. —Malicky 2005b: 545 [checklist]. —Cianficconi et al. 2007b: 576 [distribution]. —Cianficconi et al. 2007a: 67 [proposed as Italian endemic]. —Corallini et al. 2013a: 38 [checklist]. —Cianficconi et al. 2016: 142 [distribution].

**Distribution.** —Italy.

***strepha*** Ross, 1941a: 68 [type locality: [United States], Pennsylvania, Athens, Susquehanna River; INHS; ♂]. —Etnier 1965: 147 [distribution]. —Blickle 1979: 49, 67 [checklist; ♂]. —Harris et al. 1982a: 511 [distribution]. —Huryn and Foote 1983: 790 [distribution]. —Masteller and Flint 1992: 70 [checklist]. —Moulton and Stewart 1996: 104 [♂; checklist]. —Harris and Huryn 2000: 80 [♂]. —Biondi 2010: 61 [distribution]. —Armitage et al. 2011: 14 [checklist].

**Distribution.** —U.S.A.

***suanhom*** Malicky & Chantaramongkol, 2007: 1014 [type locality: Thailand, Prov. Loei, Ban Phangam, Suanhom WF, 17°03'N 101°46'E, 700 m; Collection Malicky; ♂]. —Malicky 2010a: 31 [atlas; ♂].

**Distribution.** —Thailand.

***sudip*** Wells & Huisman, 1992: 97 [type locality: East Malaysia, Sarawak, Bako National Park, Sungai Delima; NTM; ♂]. —Malicky 2010a: 27 [atlas; ♂].

**Distribution.** —Malaysia.

***sumanmalie*** Chantaramongkol & Malicky, 1986: 514 [type locality: [Sri Lanka], Provimz Sabaragamuwa, Maratenna, 7 mi N von Balangoda; MZLU; ♂].

**Distribution.** —Sri Lanka.

***surinamensis*** Flint, 1974b: 64 [type locality: Suriname, Blanche Marie, falls behind camp; RMNH; ♂].

**Distribution.** —Suriname.

***sykorai*** Harris, 2002: 56 [type locality: [United States], Florida, Gadsden County, headwaters of Quincy Creek, 7 km N Quincy at Florida A&M Research and Extension Center, 30°39'27"N, 84°36'50"W; NMNH; ♂]. —Pescador et al. 2004: 133 [checklist]. —Harris et al. 2012: 7 [checklist].

**Distribution.** —U.S.A.

***sylvestris*** Morton, 1898: 107 [type locality: [Scotland], near Aviemore, the shores of Loch Morlich, Glen More, Inverness-Shire, 1046 feet; depository not designated; ♂]. —Morton 1904: 325 [distribution]. —Mosely 1939b: 264 [♂]. —Kimmins 1943: 154 [distribution]. —Botosaneanu 1967: 294 [distribution]. —Botosaneanu and Malicky 1978: 341 [checklist]. —Malicky 1983b: 51, 52 [atlas; ♂; ♀]. —González et al. 1990: 214 [distribution]. —Malicky 2002: 4 [distribution]. —Malicky 2004a: 56, 64 [atlas]. —Malicky 2005b: 545 [checklist]. —González and Menéndez 2011: 119 [distribution]. —Wallace 2016: 21, 23 [conservation status].

**Distribution.** —England, France, Scotland, Spain.

***tacheti*** Coppa & Malicky, 2005: 19 [type locality: [Italy], Friuli, Cornino 180 m, fiume Tagliamento; Collection Malicky; ♂]. —Coppa and Tachet 2005: 132 [distribution]. —Wolf et al. 2012: 75 [distribution]. —Corallini et al. 2013a: 38 [checklist]. —Cianficconi et al. 2016: 142 [distribution]. —Malicky 2016b: 22 [morphological comparison with *H.brissaga*].

**Distribution.** —France, Italy.

***tagus*** de Jalón & González, 1985: 73 [type locality: [Spain], River Tajo in Peralejos de las Truchas (Guadalajara, España), 1.100 m; ETSI; ♂]. —Malicky 2004a: 56 [atlas]. —Malicky 2005b: 545 [checklist]. —González and Menéndez 2011: 119 [distribution].

**Distribution.** —Spain.

***takamaka*** Marlier & Marlier, 1982: 20 [type locality: La Réunion, Station 46, Premier village, plaine des Palmistes, Cascade Biberon, sur Grand Bras Patience, 950 m, au pied de la grande cascade, cuvette d’eau claire agitée, rochers ruisselants; IRSNB; ♂; larva]. —Botosaneanu 2002a: 328 [♂].

**Distribution.** —[Réunion].

***talladega*** Harris, 1985b: 615 [type locality: [United States], Alabama, Cleburne County, unnamed tributary to Coleman lake, 3/4 mile northeast of Choccolocco Ranger Station (R10E, T14S, S27); NMNH; ♂]. —Morse et al. 1989: 22 [distribution]. —Harris et al. 1991: 208 [distribution]. —Frazer et al. 1991: 20 [distribution]. —Masteller and Flint 1992: 70 [distribution]. —Floyd et al. 1997: 136 [distribution]. —Houp 1999: 2 [distribution]. —DeWalt and Heinold 2005: 42 [phenology; distribution]. —Armitage et al. 2011: 14 [distribution; threatened].

**Distribution.** —U.S.A.

***tanduka*** Wells, 1990b: 388 [type locality: [Indonesia] Sulawesi Utara, Dumoga-Bone N.P., Edwards Camp near Tumpah R.; NMV; ♂; ♀]. —Malicky et al. 2010: 163 [distribution].

**Distribution.** —Indonesia.

***tannerorum*** Wells & Andersen, 1995: 160 [type locality: Tanzania, Tanga region, West Usambara Mts, Mazumbai, Kaputu Stream, loc. 7, 1535 m a.s.l.; ZMUB; ♂].

**Distribution.** —Tanzania.

***tasmanica*** Mosely, 1934a: 147 [type locality: [Australia] Tasmania, Wilmot; Collection Tillyard (transferred to NHMUK according to Mosely and Kimmins 1953: 509); ♂]. —Mosely and Kimmins 1953: 509 [♂]. —Neboiss 1977: 40 [♂]. —Wells 1978: 749 [♂; ♀; distribution]. —Neboiss 1986: 6 [atlas; ♂; ♀]. —Neboiss 2002: 52 [checklist].

**Distribution.** —Australia.

***taurica*** Martynov, 1934: 138 [type locality: [Ukraine]; depository not designated; ♂]. —Schmid 1959b: 687 [distribution]. —Botosaneanu and Sykora 1963: 126 [larva]. —Botosaneanu 1967: 294 [distribution]. —Malicky 1974: 122 [checklist]. —Botosaneanu and Malicky 1978: 341 [checklist]. —Kumanski 1979: 12 [♂; distribution]. —Malicky 1983b: 48 [atlas; ♂]. —Kumanski and Malicky 1984: 199 [distribution]. —Kumanski 1985: 132 [♂]. —Sipahiler and Malicky 1987: 112, 135 [distribution]. —Spuris 1989: 16 [checklist]. —Chvojka 1996: 131 [distribution]. —Malicky 1999f: 32 [note on distribution]. —Mirmoayedi and Malicky 2002: 164 [checklist]. —Malicky 2004a: 60 [atlas]. —Urbanič 2004: 51 [distribution]. —Sipahiler 2005: 397 [distribution]. —Malicky 2005a: 63 [distribution]. —Malicky 2005b: 545 [checklist]. —Coppa and Tachet 2005: 130 [♀]. —Chvojka 2006: 253 [distribution]. —Sipahiler 2007: 38 [distribution]. —Chvojka and Komzák 2008: 13 [distribution]. —Szczęsny and Godunko 2008: 15 [checklist]. —Ciubuc 2009: 103 [distribution]. —Ivanov 2011: 195 [checklist]. —Karaouzas and Malicky 2015: 14 [distribution]. —Sipahiler 2016: 15 [checklist]. —Melnitsky et al. 2017: 6 [distribution]. —Sipahiler 2018: 41 [distribution].

**Distribution.** —Bulgaria, Czech Republic, Greece, Iran, Slovenia, Romania, Russia, Turkey, Ukraine.

***terbela*** Wells, 1990b: 384 [type locality: [Indonesia] Sulawesi Utara, Dumoga-Bone N.P., Toraut R., 200 m above Tumpah R. junction; NMV; ♂; ♀]. —Wells and Huisman 2001: 210 [distribution]. —Malicky et al. 2010: 163 [distribution].

**Distribution.** —Indonesia.

***tethys*** Malicky & Chantaramongkol, 2007: 1021 [type locality: Thailand, Than Than Lod NP, 99°20'E 14°46'N, 500 m; Collection Malicky; ♂]. —Malicky 2010a: 33 [atlas; ♂].

**Distribution.** Thailand.

***thaphena*** Oláh, 1989: 280 [type locality: Vietnam, Tamdao, 200 m a.s.l.; HNHM; ♂]. —Armitage et al. 2005: 27 [checklist]. —Malicky 2010a: 32 [atlas; ♂]. —Yang et al. 2016: 476 [checklist].

—*triangula* Xue & Yang, 1990: 128 [type locality: Linxian Qihe, Henan; NAUJ; ♂]. —Xue et al. 1992: 353–356 [distribution]. —Yang et al. 1997b: 93 [checklist]. —Yang et al. 2005: 458 [checklist]. —Oláh and Johanson 2010a: 22 [to synonymy].

**Distribution.** —China, Vietnam.

***tharsis*** Malicky, 2008a: 837 [type locality: [Indonesia, Borneo, Kalimantan], im Einzugsbereich der Flüsse Seturan und Rian in einem engen Bereich von ungefähr 8 × 8 km ca. 70 km südlich der Stadt Malinau, 116°29'48"–116°33'29"E, 2°59'29"–3°04'04"N, 100–200 m; MZLS; ♂]. —Malicky 2010a: 33 [atlas; ♂].

**Distribution.** —Indonesia.

***theano*** Malicky & Chantaramongkol, 2007: 1011 [type locality: Thailand, Huai Huat NP, 16°55'N 104°11'E, 400 m; Collection Malicky; ♂]. —Oláh and Johanson 2010a: 22 [distribution]. —Malicky 2010a: 28 [atlas; ♂].

**Distribution.** —Thailand, Vietnam.

***theiodamas*** Malicky & Chantaramongkol, 2007: 1018 [type locality: [Indonesia] Sumatra, Sitahoan, 2°39'N 99°00'E, 1500 m; Collection Malicky; ♂]. —Malicky 2007a: 177 [checklist]. —Oláh and Johanson 2010a: 23 [distribution]. —Malicky 2010a: 31 [atlas; ♂].

**Distribution.** —Indonesia.

***thersandros*** Malicky & Chantaramongkol, 2007: 1015 [type locality: Thailand, Putoei NP, Ban Huai Hindam, 14°57'N 99°25'E, 400 m; Collection Malicky; ♂]. —Malicky 2010a: 30 [atlas; ♂].

**Distribution.** —Thailand.

***thiba*** Oláh, 1989: 277 [type locality: Vietnam, Tamdao; HNHM; ♂]. —Armitage et al. 2005: 27 [checklist]. —Oláh and Johanson 2010a: 23 [distribution]. —Malicky 2010a: 34 [atlas; ♂]. —Yang et al. 2016: 476 [checklist].

**Distribution.** —Hong Kong, Vietnam.

***thisa*** Oláh, 1989: 279 [type locality: Vietnam, Hoabinh, 8 km from the city in the direction of Dabac; HNHM; ♂]. —Armitage et al. 2005: 27 [checklist]. —Oláh and Johanson 2010a: 23 [distribution]. —Malicky 2010a: 32 [atlas; ♂].

**Distribution.** —Laos, Vietnam.

***thisbe*** Malicky, 2008a: 837 [type locality: [Indonesia, Borneo, Kalimantan], im Einzugsbereich der Flüsse Seturan und Rian in einem engen Bereich von ungefähr 8 × 8 km ca. 70 km südlich der Stadt Malinau, 116°29'48"–116°33'29"E, 2°59'29"–3°04'04"N, 100–200 m; MZLS; ♂]. —Malicky 2010a: 33 [atlas; ♂].

**Distribution.** —Indonesia.

***thuna*** Oláh, 1989: 281 [type locality: Vietnam, Hoabinh, 8 km from Hoabinh in the direction of Dabac; HNHM; ♂]. —Xue et al. 1992: 353–356 [distribution]. —Wells and Malicky 1997: 183 [♂; distribution]. —Armitage et al. 2005: 27 [checklist]. —Malicky and Chantaramongkol 2007: 1023 [distribution]. —Malicky 2007a: 177 [checklist]. —Oláh and Johanson 2010a: 23 [distribution]. —Malicky 2010a: 24 [atlas; ♂]. —Ito et al. 2011: 5 [♂ ♀; distribution]. —Ivanov 2011: 195 [checklist; distribution]. —Laudee and Prommi 2011: 283 [distribution]. —Bunlue et al. 2012: 15 [distribution]. —Malicky et al. 2014c: 33 [distribution]. —Malicky 2014a: 1623 [checklist]. —Mattern 2015: 501 [distribution]. —Ito 2015: 8, 13 [distribution]. —Tanida and Kuranishi 2016: 71 [checklist]. —Yang et al. 2016: 476 [checklist]. —Potikha and Vshivkova 2016: 364 [distribution]. —Malicky et al. 2019: 429 [distribution]. —Promwong and Thapanya 2019: 75 [distribution].

—*triangularis* Wells & Dudgeon, 1990: 168 [type locality: Hong Kong, Tau Po Kau Forest Stream; NHMUK; ♂]. —Xue et al. 1992: 353–356 [to synonymy]. —Wells and Malicky 1997: 183 [also to synonymy]. —Malicky 2013: 42 [also to synonymy].

—*apiculata* Yang & Xue, 1992: 26 [type locality: [China] Yangdian, Yixing, Jiangsu; NAUJ; ♂]. —Xue et al. 1992: 353–356 [to synonymy]. —Yang et al. 1997b: 93 [checklist]. —Arefina 2004: 210 [♂; ♀; distribution]. —Yang et al. 2005: 458 [checklist]. —Malicky and Chantaramongkol 2007: 1023 [to synonymy].

—*molione* Malicky, 2004b: 294 [type locality: [Nepal, Bardia National Park], am Rande der nordindischen Ebene im Südwesten von Nepal im Bereich des ersten Hügelkammes des Himalaya (Siwalik Range), unweit des Wehrs des Babai Flusses, über das die Brücke der Ost-West-Haupstraße Nepals (Mahindra Highway), 28°25'N, 81°23'E, 190 m; Collection Malicky; ♂]. —Malicky and Chantaramongkol 2007: 1023 [to synonymy]. —Oláh and Johanson 2010a: 23 [also to synonymy].

**Distribution.** —Cambodia, China, Hong Kong, Indonesia, Japan, Laos, Nepal, Russia, Taiwan, Thailand, Vietnam.

***tifica*** Sipahiler, 2012b: 1051 [type locality: Turkey, Ordu, Niksar, Ünye direction, Gökçebayir village, Tifi stream, 914 m; HUAT; ♂; ♀].

**Distribution.** —Turkey.

***tigurina*** Ris, 1894: 133 [type locality: [Switzerland], an den Pfeilern der Bahnhofbrücke in Zürich; depository not designated; ♂]. —Morton 1904: 325 [distribution]. —Mosely 1932: 176 [distribution]. —Mosely 1939b: 273 [♂]. —Kimmins 1943: 154 [distribution]. —Botosaneanu 1967: 294 [distribution]. —Botosaneanu and Malicky 1978: 341 [checklist]. —O’Connor 1978: 191 [distribution]. —Marshall 1979a: 213 [♀]. —Moretti and Cianficconi 1981: 201 [checklist]. —Malicky 1983b: 51, 52 [atlas; ♂; ♀]. —González et al. 1990: 215 [distribution]. —Schmidt-Brücken 1996: 85 [distribution]. —Valle 2001: 67 [distribution]. —Malicky 2004a: 56, 64 [atlas]. —Malicky 2005a: 63 [distribution]. —Malicky 2005b: 545 [checklist]. —Sipahiler 2005: 397 [distribution]. —Lubini-Ferlin and Vicentini 2005: 67 [checklist]. —Sipahiler 2007: 38 [distribution]. —Cianficconi and Corallini 2010: 88 [distribution]. —Corallini and Cianficconi 2011: 628 [checklist]. —González and Menéndez 2011: 119 [distribution]. —Corallini et al. 2013a: 38 [checklist]. —O’Connor 2015: 28, 89 [distribution]. —Martín et al. 2015: 75 [distribution]. —Karaouzas and Malicky 2015: 14 [distribution]. —Cianficconi et al. 2016: 142 [distribution]. —Wallace 2016: 21, 23, 52 [conservation status]. —Küçükbasmaci and Kiyak 2017: 488 [distribution]. —Cerjanec et al. 2020: 13 [distribution].

**Distribution.** —Croatia, England, France, Germany, Greece, Ireland, Italy, Spain, Switzerland, Turkey.

***tineoides*** Dalman, 1819: 126 [type locality: [Sweden], habitat in monte Kinnekulle ad littora lacus Wenneri; NHRS; ♂]. —Stephens 1836: 152 [distribution]. —Kolenati 1848: 105 [revision; distribution]. —McLachlan 1865: 94 [♂]. —Eaton 1873: 139 [revision]. —Forsslund 1955: 125 [topotype designation; distribution]. —Nybom 1960: 18 [checklist]. —Botosaneanu 1967: 294 [distribution]. —Solem 1970a: 2 [distribution]. —Andersen 1974: 26 [distribution]. —Botosaneanu and Malicky 1978: 341 [checklist]. —Kumanski 1979: 15 [♂; distribution]. —Malicky 1980a: 16 [checklist]. —Moretti et al. 1981: 350, 354 [biology; distribution]. —Moretti and Cianficconi 1981: 201 [checklist]. —Malicky 1983b: 43, 52 [atlas; ♂; ♀]. —Kumanski and Malicky 1984: 199 [distribution]. —Nógrádi 1985: 131 [distribution; ♂]. —Kumanski 1985: 134 [♂]. —Andersen and Tysse 1985: 84 [distribution]. —Wiberg-Larsen 1985: 40 [checklist]. —Andersen and Wiberg-Larsen 1987: 169 [checklist]. —Sipahiler and Malicky 1987: 114 [distribution]. —Usseglio-Polatera and Bournaud 1989: 253 [distribution]. —Spuris 1989: 16 [checklist]. —Andersen et al. 1990: 52 [distribution]. —Krušnik 1991: 13 [distribution]. —Andersen et al. 1993a: 51 [distribution]. —Andersen et al. 1993b: 3 [distribution]. —Cianficconi et al. 1999a: 57 [distribution]. —Uherkovich and Nógrádi 1999: 421 [distribution]. —Gullefors 2002: 138 [checklist]. —Ujvárosi 2002: 384 [distribution]. —Nógrádi and Uherkovich 2002: 130 [distribution]. —Cibaitė 2003a: 10 [checklist]. —Gullefors 2003: 195 [distribution]. —Malicky 2004a: 52, 64 [atlas]. —Malicky 2005b: 545 [checklist]. —Gullefors 2005a: 119 [distribution]. —Gullefors 2005b: 138 [distribution]. —Sipahiler 2005: 397 [distribution]. —Malicky 2005a: 63 [distribution]. —Graf et al. 2005: 55 [distribution]. —Lubini-Ferlin and Vicentini 2005: 67 [checklist]. —Schiess-Bühler and Rezbanyai-Reser 2006: 72 [distribution]. —Gullefors and Johanson 2007: 64 [distribution]. —Sipahiler 2007: 38 [distribution]. —Robert 2007: 82 [checklist]. —Cianficconi et al. 2007b: 569, 576 [distribution]. —Chvojka and Komzák 2008: 13 [distribution]. —Szczęsny and Godunko 2008: 15 [checklist]. —Ujvárosi et al. 2008: 112 [checklist]. —Baryshev 2008: 379 [ecology]. —Višinskienė 2009: 27 [checklist]. —Hohmann 2010: 40 [distribution]. —González and Menéndez 2011: 119 [distribution]. —Cianficconi et al. 2011: 47 [distribution]. —Ivanov 2011: 195 [checklist]. —Komzák and Kroča 2011: 190 [distribution]. —Viidalepp et al. 2011: 196 [distribution]. —Andersen and Hagenlund 2012: 136 [distribution]. —Lock et al. 2013: 22 [distribution]. —O’Connor 2013: 64 [distribution]. —Corallini et al. 2013b: 26 [distribution]. —Corallini et al. 2013a: 38 [checklist]. —Zuyderduyn and Tempelman 2013: 29 [distribution]. —Karaouzas and Malicky 2015: 14 [distribution]. —Stanić-Koštroman et al. 2015: 85 [distribution]. —O’Connor 2015: 28, 90 [distribution]. —Pan’kov and Krasheninnikov 2016: 333 [distribution]. —Smirnova et al. 2016: 401 [distribution]. —Cianficconi et al. 2016: 142 [distribution]. —O’Connor and O’Connor 2016: 166 [distribution]. —Chuluunbat et al. 2016: 102[distribution]. —Martín et al. 2016: 262 [distribution]. —Gullefors 2016: 155 [checklist]. —Graf and Leitner 2016: 37 [distribution]. —Sipahiler 2017b: 13 [distribution]. —Komzák and Kroča 2018: 167 [distribution]. —O’Connor and O’Connor 2018: 82 [distribution]. —Gullefors 2018: 108 [biology; distribution]. —Cerjanec et al. 2020: 13 [distribution]. —Edmonds-Brown 2020: 91 [checklist]. —Smirnova et al. 2020: 69 [distribution]. —Hansen and Gíslasen 2020: 132 [checklist].

—*Phrixocomafemoralis* (Eaton, 1873): 136 [type locality: [England], The Dove, at Mappleton, Derbyshire, between the bridge and the weir; NHMUK; ♂; ♀]. —McLachlan 1880: 512 [revision; ♂; ♀]. —Morton 1896: 104 [distribution]. —Ris 1903: 16 [distribution]. —Siltala 1908: 14 [distribution]. —Mosely 1919a: 396 [scent-organ]. —Martynov 1924: 44 [♂]. —Martynov 1934: 138 [♂]. —Henriksen 1937: 2 [distribution]. —Mosely 1939b: 267 [♂]. —Kimmins 1943: 154 [distribution]. —Nielsen 1948: 62 [larva]. —Kimmins 1957a: 109 [lectotype designation]. —Fischer 1961: 169 [to synonymy]. —Spuris 1962: 62 [distribution]. —Spuris 1972: 19, 27, 29, 30 [checklist].

—*longispina* McLachlan, 1884: 71 [type locality: England, Ambleside; NHMUK; ♂]. —King 1886: 290 [as var. of *femoralis*]. —Kimmins 1957a: 107 [lectotype designation]. —Fischer 1961: 169 [to synonymy].

**Distribution.** —Algeria, Austria, Belgium, Bosnia-Herzegovina, Bulgaria, Croatia, Czech Republic, Denmark, England, Estonia, Finland, France, Germany, Greece, Hungary, Ireland, Italy, Kazakhstan, Latvia, Mongolia, Netherlands, Norway, Portugal, Romania, Russia, Serbia, Slovenia, Spain, Sweden, Switzerland, Turkey, Ukraine.

***tobago*** Botosaneanu in Botosaneanu and Alkins-Koo 1993: 27 [type locality: Tobago, streamlet, cut by road Roxborough-Parlatuvier, near summit; ZMUA; ♂; ♀]. —Botosaneanu and Sakal 1992: 202 [distribution; ecology]. —Flint 1996b: 98 [distribution]. —Botosaneanu 2002b: 83 [checklist].

**Distribution.** —Tobago.

***tomah*** Harris & Huryn, 2000: 77 [type locality: [United States], Maine, Washington County, Tomah Stream @ floodplain, N45°28.28', W67°35.58'; NMNH; ♂].

**Distribution.** —U.S.A.

***tombolhitam*** Wells & Malicky, 1997: 185 [type locality: [Indonesia] N Sumatra, Huta Padang, 02°45'N 99°14'E; Collection Malicky; ♂]. —Malicky 2007a: 177 [checklist]. —Malicky 2010a: 33 [atlas; ♂].

**Distribution.** —Indonesia.

***tong*** Wells & Malicky, 1997: 182 [type locality: [Indonesia] N Sumatra, Sungai Aek Tarum, Labuan Julu near Aek Tarum, 2°42'18"N 99°22'31"E, 80 [m] asl; Collection Malicky; ♂]. —Malicky and Chantaramongkol 2007: 1023 [distribution]. —Malicky 2007a: 177 [checklist]. —Malicky 2010a: 34 [atlas; ♂]. —Malicky et al. 2014a: 6 [distribution].

**Distribution.** —Indonesia.

***tortosa*** Ross, 1938a: 125 [type locality: [United States], Virginia, Luray; INHS; ♂]. —Etnier 1968: 191 [distribution]. —Blickle 1979: 49, 63 [checklist; ♂]. —Morse et al. 1989: 22 [distribution]. —Houghton et al. 2001: 504 [distribution]. —Houghton and Holzenthal 2003: 37 [distribution; conservation status]. —Houghton et al. 2017: 63 [checklist].

**Distribution.** —U.S.A.

***touroumaya*** Schmid, 1960: 96 [type locality: [Pakistan] Penjab, Hassan Abdal; CNC; ♂]. —Schmid 1958c: 220 [as new species, *nomen nudum*; distribution]. —Lonsdale 2020: 41 [holotype depository].

**Distribution.** —Pakistan.

***traunica*** Wells, 1991: 504 [type locality: Papua New Guinea, West Highland Province, Baiyer River Sanctuary, Trauna River, 1160 m, 5°30'S 144°10'E; ANIC; ♂; ♀].

**Distribution.** —Papua New Guinea.

***tridentata*** Holzenthal & Kelley, 1983: 470 [type locality: [United States], South Carolina, Dorchester Co., Four Holes Swamp, Goodsons Lake; NMNH; ♂].

**Distribution.** —U.S.A.

***triloba*** Kimmins, 1957b: 300 [type locality: [Solomon Islands, Guadalcanal], Honiara; NHMUK; ♂]. —Wells 1984: 269 [distribution]. —Neboiss 1986: 62 [atlas; ♂; as *trilobata*]. —Wells 1991: 506 [distribution].

**Distribution.** —Papua New Guinea, Solomon Islands.

***trilobata*** Jacquemart, 1965: 13 [type locality: [Turkey] collection data not provided; IRSNB; type specimen not designated; larva]. —Malicky 2005b: 545 [checklist].

**Distribution.** —Turkey.

***trullata*** (Ulmer, 1951): 88 [type locality: [Indonesia], Sumatra, Tobagebiet, Bach südlich Balige; ZMUH; ♂; in *Sumatranotrichia*]. —Malicky 1998a: 797 [♂; distribution]. —Malicky and Chantaramongkol 2007: 1023 [distribution]. —Malicky 2007a: 177 [checklist]. —Malicky 2010a: 25 [atlas; ♂]. —Malicky 2013: 43 [possible senior synonym to *Hydroptilakhonga*]. —Malicky et al. 2014a: 6 [distribution]. —Melnitsky et al. 2019: 539 [distribution].

**Distribution.** —Indonesia, Malaysia, Thailand.

***tulipa*** Oláh & Johanson, 2011: 127 [type locality: Peru, Dep. Lima, Pacaran, Province Canete, River Chillon Obrajillo, 12°52'05"S 76°02'60"W, 877 m; NHRS; ♂].

**Distribution.** —Peru.

***tumpul*** Wells & Huisman, 1992: 105 [type locality: West Malaysia, Genting Highlands, Bukit Rengit; NTM; ♂]. —Malicky 2010a: 28 [atlas; ♂].

**Distribution.** —West Malaysia.

***tungsalaeng*** Malicky & Chantaramongkol, 2007: 1015 [type locality: Thailand, Prov. Kanchanaburi, Erawan NP, 14°22'N 99°08'E, 200 m; Collection Malicky; ♂]. —Malicky 2010a: 30 [atlas; ♂].

**Distribution.** —Thailand.

***tusculum*** Ross, 1947: 148 [type locality: [United States], Tennessee, Tusculum College, Green Co.; INHS; ♂; ♀]. —Etnier and Schuster 1979: 18 [distribution]. —Blickle 1979: 49, 71 [checklist; ♂]. —Harris et al. 1982a: 511 [distribution]. —Harris et al. 1991: 1209 [distribution]. —Mathis and Bowles 1992: 24 [distribution]. —Moulton and Stewart 1996: 104 [♂; distribution]. —Houghton et al. 2017: 63 [checklist].

**Distribution.** —U.S.A.

***uncinata*** Morton, 1893: 77 [type locality: Italy, Apennino Pistojese; NHMUK; ♂]. —Ris 1897: 416 [distribution]. —Mosely 1930a: 176 [distribution; ♂ scent-organ]. —Kimmins 1957a: 108 [lectotype designation]. —Botosaneanu 1967: 294 [distribution]. —Botosaneanu and Malicky 1978: 341 [checklist]. —Moretti and Cianficconi 1981: 201 [checklist]. —Malicky 1981a: 183 [♂]. —Malicky 1983b: 44 [atlas; ♂]. —Kumanski 1985: 129 [♂]. —Botosaneanu and Dumont 1987: 118 [♂]. —Malicky and Moretti 1987: 194 [♂]. —Malicky 2004a: 53 [atlas]. —Malicky 2005b: 545 [checklist]. —Cianficconi et al. 2007b: 569, 576 [distribution]. —Cianficconi et al. 2007a: 67 [proposed as Italian endemic]. —Corallini and Cianficconi 2011: 628 [checklist]. —Corallini et al. 2013a: 38 [checklist]. —Cianficconi et al. 2016: 143 [distribution]. —Valle and Lodovici 2018: 146 [distribution].

**Distribution.** —France, Italy, Switzerland.

***unicuspis*** Flint, 1991b: 49 [type locality: Colombia, Dpto. Antioquia, Quebrada La Cebolla, El Retiro; NMNH; ♂; ♀]. —Muñoz-Quesada 2000: 278 [checklist].

**Distribution.** —Colombia.

***upulmalie*** Chantaramongkol & Malicky, 1986: 514 [type locality: [Sri Lanka], Western Province, Yakkala, 18 mi NE von Colombo (Dambowa Estate), 30 m; MZLU; ♂].

**Distribution.** —Sri Lanka.

***usambarensis*** Wells & Andersen, 1995: 158 [type locality: Tanzania, Tanga region, West Usambara Mts, Dule, Bumbuli River, 1220 m a.s.l.; ZMUB; ♂].

**Distribution.** —Tanzania.

***vala*** Ross, 1938a: 123 [type locality: [United States], Illinois, Herod; INHS; ♂]. —Ross 1944: 148 [♂; ♀; distribution]. —Etnier and Schuster 1979: 18 [distribution]. —Blickle 1979: 49, 65 [checklist; ♂]. —Waltz and McCafferty 1983a: 10 [distribution]. —Huryn and Foote 1983: 790 [distribution]. —Bowles and Mathis 1989: 239 [distribution]. —Harris et al. 1991: 210 [distribution]. —Bowles and Mathis 1992: 32 [distribution]. —Moulton and Stewart 1996: 105 [♂; distribution]. —Etnier 2010: 485 [distribution]. —Armitage et al. 2011: 14 [checklist].

**Distribution.** —U.S.A.

***valesiaca*** Schmid, 1947: 530 [type locality: [Switzerland], Praz-de-Fort (Val Ferret, Valais); CNC; ♂]. —Botosaneanu 1967: 294 [distribution]. —Marshall 1977: 119 [revision; ♂; ♀]. —Botosaneanu and Malicky 1978: 341 [checklist]. —Malicky 1983b: 48, 52 [atlas; ♂; ♀]. —Nelson and Panter 1984: 39 [distribution]. —Maier et al. 1995: 145 [distribution]. —Kahnert 1995: 124 [distribution]. —Chvojka 1996: 131 [distribution]. —Urbanič 2004: 51 [distribution]. —Malicky 2004a: 60 [atlas]. —Malicky 2005b: 545 [checklist]. —Weinzierl et al. 2005: 47 [distribution]. —Coppa and Tachet 2005: 130 [♀]. —Lubini-Ferlin and Vicentini 2005: 67 [checklist]. —Chvojka and Komzák 2006: 358 [distribution]. —Robert 2007: 82 [checklist]. —Chvojka and Komzák 2008: 13 [distribution]. —O’Connor and O’Connor 2013: 189 [distribution]. —O’Connor 2015: 28 92 [distribution]. —Wallace 2016: 21, 23 [conservation status]. —Lonsdale 2020: 41 [holotype depository].

**Distribution.** —Czech Republic, Germany, Ireland, Slovenia, Scotland, Switzerland.

***valhalla*** Denning, 1947b: 175 [type locality: [United States], Minnesota, Taylors Falls; UMSP; ♂]. —Etnier 1965: 147 [checklist]. —Etnier 1968: 191 [distribution]. —Blickle 1979: 49, 71 [checklist; ♂]. —Steven and Hilsenhoff 1984: 164 [distribution]. —Harris et al. 1991: 211 [distribution]. —Huryn and Harris 2000: 193 [distribution]. —Houghton et al. 2001: 504 [distribution]. —Armitage et al. 2011: 14 [checklist]. —Houghton 2016: 46 [biology]. —Houghton et al. 2017: 63 [checklist].

**Distribution.** —U.S.A.

***vanuatensis*** Johanson, Wells, Malm, & Espeland, 2011: 289 [type locality: [Vanuatu] Espiritu Santo, East Santo, Sarekata River, 300 m E Fanafao, 166 m, loc#15, 15°24.345'S, 167°06.021'E; NHRS; ♂].

**Distribution.** —Vanuatu.

***varla*** Sipahiler, 1996: 29 [type locality: Turkey, Kastamonu, Pinarbasi, Varla Mahallesi, Devrekani deresi; ZSM; ♂]. —Malicky 2004a: 63 [atlas]. —Malicky 2005b: 545 [checklist]. —Sipahiler 2005: 397 [distribution]. —Sipahiler 2007: 38 [distribution]. —Sipahiler 2008: 104 [checklist].

**Distribution.** —Turkey.

***vazquezae*** Bueno-Soria, 1984: 105 [type locality: Mexico, Chiapas, Santa Elena, 50 km S Montebello; CNIN; ♂].

**Distribution.** —Mexico.

***vectis*** Curtis, 1834: 217 [type locality: “Britain”; NVM; ♂]. —Stephens 1836: 152 [distribution]. —Neboiss 1963: 626 [lectoholotype designated; note on type locality]. —Botosaneanu 1967: 294 [distribution]. —Botosaneanu and Gasith 1971: 98 [distribution]. —Malicky 1974: 122 [checklist]. —Botosaneanu and Malicky 1978: 341 [checklist]. —Kumanski 1979: 14 [♂; distribution]. —Malicky 1980a: 16 [checklist]. —Moretti and Cianficconi 1981: 201 [checklist]. —Mey 1981: 56 [distribution]. —González and Otero 1983: 118 [distribution]. —Malicky 1983b: 51, 52 [atlas; ♂; ♀]. —Kumanski and Malicky 1984: 199 [distribution]. —Moubayed and Botosaneanu 1985: 63 [distribution]. —Kumanski 1985: 140 [♂]. —Andersen and Wiberg-Larsen 1987: 169 [checklist]. —Sipahiler and Malicky 1987: 122 [distribution]. —Malicky and Lounaci 1987: 15, 17 [checklist]. —Usseglio-Polatera and Bournaud 1989: 254 [distribution]. —Spuris 1989: 16 [checklist]. —González et al. 1990: 213 [checklist]. —Krušnik 1991: 13 [distribution]. —Botosaneanu 1992: 84 [♂; ♀]. —Nógrádi and Uherkovich 1994: 31 [distribution]. —Uherkovich and Nógrádi 1997: 461 [distribution]. —Uherkovich and Nógrádi 1999: 421 [distribution]. —Cianficconi et al. 1999a: 57 [distribution]. —Malicky 1999c: 96 [distribution]. —Cianficconi et al. 1999b: 279 [distribution]. —Nógrádi and Uherkovich 2001: 297 [checklist]. —Gullefors 2002: 138 [checklist]. —Ujvárosi 2002: 384 [distribution]. —Cianficconi et al. 2002: 146 [distribution]. —Sipahiler 2003b: 33 [distribution]. —Malicky 2004a: 56, 64 [atlas]. —Bonada et al. 2004: 52 [distribution]. —Gullefors 2005b: 138 [distribution]. —Coppa and Tachet 2005: 132 [distribution]. —Sipahiler 2005: 397 [distribution]. —Lubini-Ferlin and Vicentini 2005: 67 [checklist]. —Malicky 2005b: 536, 543 [checklist; comparison with *H.corsicana*]. —Malicky 2005a: 64 [distribution]. —Graf et al. 2005: 55 [distribution]. —Hohmann 2005: 106 [checklist]. —Wiggers et al. 2006: 54 [distribution]. —Hughes 2006: 29 [biology]. —Voigt et al. 2006: 72 [distribution]. —Schiess-Bühler and Rezbanyai-Reser 2006: 72 [distribution]. —Gullefors and Johanson 2007: 64 [distribution]. —Robert 2007: 82 [checklist]. —Cianficconi et al. 2007b: 569, 576 [distribution]. —Ivanov and Melnitsky 2007: 32 [distribution]. —Previšić et al. 2007: 184 [distribution]. —Chvojka and Komzák 2008: 13 [distribution]. —Gullefors 2008: 64 [checklist]. —Ujvárosi et al. 2008: 112 [checklist]. —Schrankel et al. 2008: 90 [checklist]. —Dohet et al. 2008: 45, 48 [distribution; ecology]. —Szczęsny and Godunko 2008: 15 [checklist]. —González and Menéndez 2008: 188 [distribution]. —Višinskienė 2009: 27 [checklist]. —Oláh 2010: 92 [distribution]. —Hohmann 2010: 40 [distribution]. —Corallini and Cianficconi 2011: 628 [checklist]. —González and Menéndez 2011: 119 [distribution]. —Valladolid et al. 2011: 501 [distribution]. —Cianficconi et al. 2011: 47 [distribution]. —Ivanov 2011: 195 [checklist]. —Viidalepp et al. 2011: 196 [distribution]. —Wolf et al. 2012: 75 [distribution]. —Komzák and Chvojka 2012: 720 [distribution]. —Corallini et al. 2013a: 38 [checklist]. —Martín et al. 2014: 72 [distribution]. —Hohmann et al. 2014: 85 [distribution]. —Martínez et al. 2015: 40 [distribution]. —Martín et al. 2015: 75 [distribution]. —Stojanović et al. 2015: 55 [distribution]. —Karaouzas and Malicky 2015: 14 [distribution]. —Dia 2015: 51 [distribution]. —Pan’kov and Krasheninnikov 2016: 333 [distribution]. —Cianficconi et al. 2016: 143 [distribution]. —Küttner et al. 2016: 179 [distribution]. —Sekhi et al. 2016: 58 [distribution]. —Sipahiler 2016: 15 [checklist]. —Gullefors 2016: 155 [checklist]. —Ruiz-García et al. 2016: 4 [distribution]. —Sáinz-Bariáin et al. 2016: 676, 678 [ecology; distribution]. —Melnitsky et al. 2017: 6 [distribution]. —Valle and Lodovici 2018: 147 [distribution]. —O’Connor 2019a: 163 [distribution]. —O’Connor and O’Connor 2019: 232 [distribution]. —Dambri et al. 2020: 224 [distribution]. —Edmonds-Brown 2020: 91 [checklist]. —Mabrouki et al. 2020: 13 [distribution]. —Oláh et al. 2020: 46 [distribution]. —Navara et al. 2020: 46 [distribution]. —Smirnova et al. 2020: 68 [distribution].

—*machlachlani* Klapálek, 1891: 177 [type locality: [Czech Republic]; depository not designated; ♂; larva]. —Klapálek 1893: 136 [larva]. —Morton 1893: 78 [distribution; ♂]. —Ris 1894: 133 [distribution]. —Morton 1896: 104 [distribution]. —Morton 1899a: 54 [distribution]. —Morton 1904: 325 [distribution]. —Mosely 1919a: 395 [scent-organ]. —Martynov 1924: 45 [♂]. —Martynov 1934: 134 [♂; as *mclachlani*]. —Mosely 1939b: 271 [♂]. —Nybom 1948: 4 [distribution]. —Schmid 1960: 97 [distribution]. —Jacquemart and Coineau 1962: 61 [♂; larva]. —Nybom 1963: 114 [distribution]. —Neboiss 1963: 626 [to synonymy]. —Nybom 1965: 89 [distribution]. —Botosaneanu 1967: 294 [to synonymy].

—*machlachlani*var.corsicana Mosely, 1930a: 176 [type locality: [France], Corsica, Corte; NHMUK; ♂]. —Botosaneanu 1967: 294 [distribution; as *corsicanus*]. —Malicky 2005b: 536, 543 [checklist; comparison with *H.vectis*]. —Malicky 2016a: 39 [to synonymy]. —Kroča and Komzák 2020: 147 [distribution].

**Distribution.** —Algeria, Austria, Bulgaria, Croatia, Czech Republic, England, Estonia, Finland France, Georgia, Germany, Greece, Hungary, Ireland, Israel, Italy, Kazakhstan, Lebanon, Luxembourg, Morocco, Netherlands, Pakistan, Portugal, Romania, Serbia, Russia, Scotland, Slovakia, Slovenia, Spain, Sweden, Switzerland, Turkey, Ukraine, Uzbekistan.

***venezuelensis*** Flint, 1981: 29 [type locality: Venezuela, Aragua, Maracay, Río Limón, Estacion Piscicultura; NMNH; ♂; ♀]. —Botosaneanu 2002b: 83 [checklist]. —Oláh and Johanson 2011: 128 [distribution]. —Ríos-Touma et al. 2017: 10 [checklist].

**Distribution.** —Ecuador, Venezuela.

***venus*** Malicky & Chantaramongkol, 2007: 1016 [type locality: Malaysia, Perak, Belum exp. base camp, 5°30'N 101°26'E, 250 m; Collection Malicky; ♂]. —Malicky 2010a: 30 [atlas; ♂]. —Malicky et al. 2018: 1322 [distribution].

**Distribution.** —Malaysia, Thailand.

***veracruzensis*** Flint, 1967b: 13 [type locality: Mexico, Vera Cruz, Cuitlahuac; NMNH; ♂]. —Bueno-Soria 1984: 116 [♂; distribution]. —Bueno-Soria and Flint 1978: 202 [distribution]. —Botosaneanu and Alkins-Koo 1993: 24 [♂; ♀; distribution]. —Flint 1996b: 97 [distribution]. —Harris and Holzenthal 1999: 45 [♂; distribution]. —Maes 1999: 1193 [checklist]. —Botosaneanu and Viloria 2002: 106 [distribution]. —Bueno-Soria et al. 2005: 75 [checklist]. —Chamorro-Lacayo et al. 2007: 43 [checklist]. —Armitage et al. 2015b: 5 [distribution]. —Armitage et al. 2015a: 6 [checklist]. —Armitage and Harris 2018b: 97 [checklist]. —Armitage and Harris 2018c: 283 [distribution]. —Harris and Armitage 2019: 4 [distribution].

**Distribution.** —Costa Rica, Mexico, Nicaragua, Panama, Trinidad, Venezuela.

***verginia*** Malicky & Chantaramongkol, 2007: 1016 [type locality: Thailand, Erawan NP, 14°22'N 99°08'E, 200 m; Collection Malicky; ♂]. —Malicky 2010a: 23 [atlas; ♂].

**Distribution.** —Thailand.

***verticordia*** Malicky & Chantaramongkol, 2007: 1021 [type locality: Thailand, Jaeson NP, 18°46'N 99°28'E, 500 m; Collection Malicky; ♂]. —Zhou et al. 2009b: 357 [distribution]. —Malicky 2010a: 29 [atlas; ♂]. —Yang et al. 2016: 476 [checklist]. —Malicky et al. 2018: 1322, 1323 [distribution].

**Distribution.** —China, Thailand.

***vichtaspa*** Schmid, 1959b: 689 [type locality: Iran, Karasang (Ost. 2); CNC; ♂]. —Botosaneanu and Malicky 1978: 341 [checklist]. —Kumanski 1979: 14 [♂; distribution]. —Malicky 1981a: 183 [♂]. —Malicky 1983b: 44 [atlas; ♂]. —Kumanski and Malicky 1984: 199 [distribution]. —Kumanski 1985: 129 [♂]. —Botosaneanu and Dumont 1987: 116 [♂ ♀]. —Sipahiler and Malicky 1987: 107, 114, 129 [distribution]. —Mirmoayedi and Malicky 2002: 164 [checklist]. —Malicky 2004a: 53 [atlas]. —Sipahiler 2005: 397 [distribution]. —Malicky 2005b: 545 [checklist]. —Malicky 2005a: 65 [distribution]. —Sipahiler 2007: 38 [distribution]. —Cianficconi et al. 2007b: 569, 576 [distribution]. —Chvojka and Komzák 2008: 13 [distribution]. —González and Menéndez 2011: 119 [distribution]. —Komzák and Chvojka 2012: 720 [distribution]. —Corallini et al. 2013a: 38 [checklist]. —Cianficconi et al. 2016: 144 [distribution]. —Lonsdale 2020: 41 [holotype depository].

**Distribution.** —Bulgaria, Cyprus, Czech Republic, Greece, Iran, Italy, Portugal, Turkey.

***victoria*** Malicky & Chantaramongkol, 2007: 1020 [type locality: Taiwan, Pinglung co., E Shihtzu, 22°14'N 120°49'E, 370 m; Collection Malicky; ♂]. —Malicky 2014a: 1623 [checklist]. —Yang et al. 2016: 476 [checklist].

**Distribution.** —Taiwan.

***viganoi*** Botosaneanu, 1974: 160 [type locality: [Israel], Walladja, dans les Mts. de Judée, pas loin du village Amminadav; TAU; ♂]. —Malicky 1983b: 51 [atlas; ♂]. —Botosaneanu 1992: 87 [♂]. —Malicky 2004a: 56 [atlas]. —Malicky 2005b: 545 [checklist].

**Distribution.** —Israel.

***vilaverde*** Malicky & González, 1981: 151 [type locality: [Spain], Provinz la Coruna, Vilaverde, Rio Allones; depository not designated; ♂]. —Malicky 1983b: 45 [atlas; ♂]. —Malicky 2004a: 54 [atlas]. —Malicky 2005b: 545 [checklist]. —González and Menéndez 2011: 119 [distribution].

**Distribution.** —Portugal, Spain.

***virgata*** Ross, 1938a: 125 [type locality: [United States], Illinois, Herod; INHS; ♂]. —Ross 1944: 148 [♂; ♀; distribution]. —Etnier 1965: 147 [distribution]. —Unzicker et al. 1970: 172 [distribution]. —Etnier and Schuster 1979: 18 [distribution]. —Roy and Harper 1979: 151 [distribution]. —Blickle 1979: 49, 63 [checklist; ♂]. —Huryn and Foote 1983: 790 [distribution]. —Lake 1984: 220 [distribution]. —Steven and Hilsenhoff 1984: 164 [distribution]. —Bowles and Mathis 1989: 239 [distribution]. —Harris et al. 1991: 212 [distribution]. —Mathis and Bowles 1992: 24 [distribution]. —Bowles and Mathis 1992: 32 [distribution]. —Moulton and Stewart 1996: 105 [♂; distribution]. —Armitage et al. 2011: 14 [checklist]. —Bowles et al. 2020: 8 [distribution].

**Distribution.** —Canada, U.S.A.

***vitcona*** Oláh & Johanson, 2010a: 24 [type locality: Vietnam, Lamdong Province, Baoloc, loc. Chau stream; Collection Oláh; ♂]. —Melnitsky et al. 2019: 539 [distribution].

**Distribution.** —Malaysia, Vietnam.

***vittata*** Wells, 1984: 267 [type locality: [Papua] New Guinea, Bulolo River, 950 m; BPBM; ♂; ♀]. —Neboiss 1986: 64 [atlas; ♂; ♀]. —Wells 1991: 506 [distribution].

**Distribution.** —Papua New Guinea.

***voticia*** Malicky, 1992a: 146 [type locality: [Comoros], Anjouan, Oberlauf-Zufluß des Riv. Mutsamudu, 500 m; HM or IRSNB; ♂.

**Distribution.** —the Comoros.

***wakulla*** Denning, 1947a: 19 [type locality: [United States], Florida, Wakulla Springs; ESUW; ♂; ♀]. —Blickle 1979: 49, 69 [checklist; ♂]. —Pescador et al. 2004: 133 [checklist]. —Harris et al. 2012: 7 [♂; checklist].

**Distribution.** —U.S.A.

***warisa*** Wells, 1984: 266 [type locality: [Western New Guinea], Irian Jaya (New Guinea), Waris; BPBM; ♂]. —Neboiss 1986: 64 [atlas; ♂; ♀]. —Wells 1991: 507 [distribution].

**Distribution.** —Indonesia.

***waskesia*** Ross, 1944: 276 [type locality: [Canada], Saskatchewan, Lake Waskesieu, Prince Albert National Park; INHS; ♂]. —Etnier 1968: 191 [distribution]. —Roy and Harper 1979: 151 [checklist]. —Etnier and Schuster 1979: 18 [distribution]. —Blickle 1979: 49, 67 [checklist; ♂]. —Huryn and Foote 1983: 790 [distribution]. —Harris et al. 1984: 108 [distribution]. —Harris et al. 1991: 213 [distribution]. —Masteller and Flint 1992: 70 [checklist]. —Houp 1999: 2 [distribution]. —Houghton et al. 2001: 504 [distribution]. —Armitage et al. 2011: 14 [checklist].

**Distribution.** —Canada, U.S.A.

***waubesiana*** Betten, 1934: 160 [type locality: [United States]; depository not designated; ♂]. —Ross 1944: 150 [♂; ♀; larva; distribution]. —Denning 1947b: 173 [distribution]. —Denning 1947a: 19 [distribution]. —Etnier 1965: 147 [checklist]. —Unzicker et al. 1970: 172 [distribution]. —Resh et al. 1978: 383 [distribution]. —Roy and Harper 1979: 151 [checklist]. —Blickle 1979: 49, 65 [checklist; ♂]. —Swegman et al. 1981: 139 [distribution]. —Parker and Voshell 1981: 4 [checklist]. —Roy and Harper 1981: 105 [distribution]. —Harris et al. 1982a: 511 [distribution]. —Huryn and Foote 1983: 790 [distribution]. —Hamilton et al. 1983: 18 [distribution]. —Waltz and McCafferty 1983a: 10 [distribution]. —Harris et al. 1984: 108 [distribution]. —Steven and Hilsenhoff 1984: 164 [distribution]. —Lake 1984: 220 [distribution]. —Bowles and Mathis 1989: 239 [distribution]. —Floyd and Schuster 1990: 130, 132 [distribution]. —Frazer et al. 1991: 20 [distribution]. —Harris et al. 1991: 214 [distribution]. —Masteller and Flint 1992: 70 [checklist]. —Bowles and Mathis 1992: 32 [distribution; as *wausbesiana*]. —Mathis and Bowles 1992: 24 [distribution]. —Floyd et al. 1993: 91 [phenology; distribution]. —Moulton et al. 1993: 21 [distribution]. —Masteller 1993: 134 [distribution]. —Moulton and Stewart 1996: 106 [♂; distribution]. —Abbott et al. 1997: 44 [distribution]. —Moulton and Stewart 1997: 350 [checklist]. —Keiper et al. 1998b: 87 [biology]. —Stewart et al. 1998: 872 [distribution; biology]. —Houghton et al. 2001: 504 [distribution]. —Pescador et al. 2004: 133 [checklist]. —Zeullig et al. 2006: 43 [distribution]. —Bowles et al. 2007: 21 [distribution; biology]. —Flint 2011: 104 [distribution]. —Armitage et al. 2011: 14 [checklist]. —Houghton et al. 2011b: 5 [distribution; biology]. —Harris et al. 2012: 7 [checklist]. —Denson et al. 2016: 5 [distribution]. —DeWalt et al. 2016: 51 [distribution]. —Houghton 2016: 46 [biology]. —Houghton et al. 2017: 63 [checklist]. —Bowles et al. 2020: 8 [distribution].

**Distribution.** —Canada, U.S.A.

***wetumpka*** Harris, 1991: 13 [type locality: [United States], Alabama, Elmore County, Corn Creek at Hwy. 14, 4.8 km ENE Wetumpka (Sec. 9, T 18 N, R 19 E); NMNH; ♂]. —Harris et al. 1991: 215 [distribution]. —Harris et al. 2012: 8 [distribution].

**Distribution.** —U.S.A.

***wuchangensis*** Wang, 1963: 56 [type locality: [China], Lake Tunghu, Wuchang; depository not designated; ♂; ♀; larva]. —Yang et al. 1997b: 93 [checklist]. —Yang et al. 2005: 458 [checklist]. —Yang et al. 2016: 476 [checklist].

**Distribution.** —China.

***wyomia*** Denning, 1947a: 150 [type locality: [United States], Wyoming, Laramie, Laramie River; ESUW; ♂]. —Roy and Harper 1979: 151 [distribution]. —Blickle 1979: 49, 63 [checklist; ♂]. —Roy and Harper 1981: 105 [distribution]. —Steven and Hilsenhoff 1984: 164 [distribution]. —Harper 1989: 541 [distribution]. —Houghton et al. 2001: 504 [distribution]. —Houghton et al. 2011b: 5 [phenology; habitat; distribution]. —Houghton 2016: 46 [biology; as *wyomyia*]. —Houghton et al. 2017: 63 [checklist].

**Distribution.** —Canada, U.S.A.

***xella*** Ross, 1941a: 65 [type locality: [United States], Tennessee, Martin Springs; INHS; ♂]. —Etnier and Schuster 1979: 18 [distribution]. —Blickle 1979: 49, 63 [checklist; ♂]. —Harris et al. 1991: 216 [distribution]. —Armitage et al. 2011: 36 [♂; ♀].

**Distribution.** —U.S.A.

***xera*** Ross, 1938a: 132 [type locality: [United States], Wyoming, Parco, along North Platte River; INHS; ♂]. —Denning 1947a: 152 [♀; distribution]. —Ross and Spencer 1952: 47 [distribution]. —Roy and Harper 1979: 151 [distribution]. —Blickle 1979: 49, 69 [checklist; ♂]. —Harper 1989: 541 [distribution]. —Houghton et al. 2001: 504 [distribution]. —Zack et al. 2006: 134 [phenology; distribution]. —Vieira et al. 2009: 257 [distribution]. —Houghton et al. 2011b: 5 [phenology; habitat; distribution]. —Blinn and Ruiter 2013: 280, 291 [biology; distribution]. —Houghton 2016: 46 [biology]. —Houghton et al. 2017: 63 [checklist]. —Mendez et al. 2019: 118 [checklist]. —Houghton and Lardner 2020: 42 [distribution].

**Distribution.** —Canada, U.S.A.

***xoncla*** Ross, 1941b: 16 [type locality: [Canada], Moser River, Nova Scotia, Gold Mine River; INHS; ♂]. —Roy and Harper 1875: 1083 [distribution]. —Roy and Harper 1979: 151 [checklist]. —Blickle 1979: 49, 69 [checklist; ♂]. —Roy and Harper 1981: 105 [distribution]. —Lake 1984: 220 [distribution]. —Huryn and Harris 2000: 193 [distribution]. —Myers et al. 2011: 107 [distribution].

**Distribution.** —Canada, U.S.A.

***yaeyamensis*** Ito, 2015: 13 [type locality: Japan, Ryukyu Islands, Ishigaki-jima, Shiramizu, Nagura-gawa, unnamed tributary, 24°24'44"N, 124°11'11"E, 95 m; CBMI-ZI; ♂; ♀]. —Tanida and Kuranishi 2016: 71 [checklist].

**Distribution.** —Japan.

***zairiensis*** Statzner, 1977: 399 [type locality: Zaire, Kivu Region, Kalengo stream 10 km west of Lake Kivu; ZMHB; ♂; ♀].

**Distribution.** —Congo.

***zerbinae*** de Souza, Santos, & Takiya, 2014b: 641 [type locality: Brazil, Pernambuco, Vicência Cachoeira do Engenho Embú, 07°37'22"S 35°22'51"W, 186 m; DZRJ; ♂].

**Distribution.** —Brazil.

***zeus*** Malicky & Chantaramongkol, 2007: 1012 [type locality: Nepal, Chitwan NP, Temple Tiger Lodge, 27°32'N 84°04'E, 150 m; Collection Malicky; ♂]. —Mattern 2015: 501 [distribution].

**Distribution.** —Nepal.

***ziddensis*** Ivanov, 1992: 236 [type locality: [Tajikistan], Hissar Mountains, k. Ziddy, on the poplar and grass; ZIN; ♂].

**Distribution.** —Tajikisan.

#### Genus *Jabitrichia* Wells, 1990

*Jabitrichia* Wells, 1990c: 108 [type species: *Jabitrichiadostinei* Wells, 1990c, original designation]. —Kjærandsen and Andersen 2002: 134 [revision; key to males].

The genus *Jabitrichia* contains four species recorded from northern Australia, Malaysia, Thailand, Angola, and Ghana. *Jabitrichia* shares morphological similarities with both *Hydroptila* and *Oxyethira*, but differs enough that the definition of either genus would have required considerable modification to accommodate the addition (Wells 1990c). *Jabitrichia* and *Hydroptila* share a spur formula (0, 2, 4), a lack of ocelli, similar pattern of wing color, and form of thoracic scutella, while *Jabitrichia* and *Oxyethira* share a forewing without a jugal lobe, the general form of the female genitalia, and reductions of particular structures of the male genitalia (Wells 1990c). In their morphological analysis, Kjærandsen and Andersen (2002) placed the genus as sister to *Oxyethira*. The larval stage is unknown.

***dostinei*** Wells, 1990c: 109 [type locality: Northern Territory, Alligator Rivers region, Gulungul Creek at inlet to Gulungul Billabong, 12°38'S 132°53'E; NTM; ♂]. —Kjærandsen and Andersen 2002: 137 [♂; ♀; distribution].

**Distribution.** —Australia.

***flagellum*** (Marlier, 1965): 69 [type locality: [Angola] Moxico, Zambèze, Rives du Lac Calundo, Loc. 4510; MDLA; ♂; in *Orthotrichia*]. —Kjærandsen and Andersen 2002: 141 [to *Jabitrichia*]. —Wells and de Moor 2020: 512 [checklist].

**Distribution.** —Angola.

***voltensis*** Kjærandsen & Andersen, 2002: 138 [type locality: Ghana, Eastern region, Volta River, Kpong; ZMUB; ♂].

**Distribution.** —Ghana.

***wellsae*** O’Connor & Ashe, 1992: [type locality: Malaysia, Tasek Bera, 03°08'N 102°36'E; NMID; ♂]. —Kjærandsen and Andersen 2002: 138 [♂]. —Malicky and Chantaramongkol 2007: 1024 [distribution]. —Malicky 2010a: 39 [atlas; ♂].

**Distribution.** —Malaysia, Thailand.

#### Genus *Kholaptila* Malicky & Chantaramongkol, 2007

*Kholaptila* Malicky & Chantaramongkol, 2007: 1024 [type species: *Kholaptilaserrata* Malicky & Chantaramongkol, 2007, original designation].

The monotypic genus *Kholaptila* is recorded from Nepal. Malicky and Chantaramongkol (2007) placed the genus in Hydroptilinae based on the absence of the transverse suture of the mesoscutellum. There are some similarities between *Kholaptila* and *Microptila* in the male genitalia (Malicky and Chantaramongkol 2007). The larvae are unknown.

***serrata*** Malicky & Chantaramongkol, 2007: 1025 [type locality: Nepal, Dakhi Khola bei Kurin Ghat, 27°52'N 84°38'E, 300 m; Collection Malicky; ♂]. —Mattern 2015: 501 [distribution].

**Distribution.** —Nepal.

#### Genus *Maeyaptila* Malicky & Chantaramongkol, 2007

*Maeyaptila* Malicky & Chantaramongkol, 2007: 1025 [type species: *Maeyaptilaxuthos* Malicky & Chantaramongkol, 2007, original designation].

The monotypic genus *Maeyaptila* occurs in Thailand. Malicky and Chantaramongkol (2007) placed the genus in Hydroptilinae due to the absence of the transverse suture of the mesoscutellum, the spur formula (0, 2, 4), the lack of ocelli, and the general structure of the male genitalia. They also noted, however, that some of the genitalic structures are also similar to those of *Scelotrichia*, a member of Stactobiinae, making the placement somewhat tenuous (Malicky and Chantaramongkol 2007). The larval stage is unknown.

***xuthos*** Malicky & Chantaramongkol, 2007: 1025 [type locality: Thailand, Prov. Mae Hong Son, Oberlauf des Huai Mae Ya bei Doi Mae Ya, 19°14'N 98°35'E, 1200 m; Collection Malicky; ♂]. —Malicky 2010a: 22 [atlas; ♂].

**Distribution.** —Thailand.

#### Genus *Microptila* Ris, 1897

*Microptila* Ris, 1897: 416 [type species: *Microptilaminutissima* Ris, 1897, monotypic]. —Marshall, 1979b: 197 [generic review]. —Graf et al. 2004: 31 [larva of type species]. —Ito 2017a: 104 [generic review].

The genus *Microptila* consists of 20 species occurring in the West Palaearctic faunal region. Marshall (1979b) commented that adult *Microptila* bear similarities with those of the genus *Dhatrichia*. The larvae of *M.minutissima* were described by Graf et al. (2004).

***apsara*** Schmid, 1960: 87 [type locality: [Pakistan] Himalaya, Balakot; CNC; ♂]. —Schmid 1958c: 220 [as new species, *nomen nudum*; distribution]. —Kjærandsen and Ito 2009: 178 [checklist]. —Lonsdale 2020: 32 [holotype depository].

**Distribution.** —Pakistan.

***atlantis*** Malicky & Chantaramongkol, 2007: 1027 [type locality: Nepal, Mahdev Khola, 27°53'N, 85°39'E, 1300 m; Collection Malicky; ♂]. —Kjærandsen and Ito 2009: 178 [checklist]. —Mattern 2015: 501 [distribution].

**Distribution.** —Nepal.

***bejela*** Mosely, 1948b: 82 [type locality: [Yemen], Western Aden Protectorate, Jebel Jihaf, Wadi Leje, c. 6300 ft; NHMUK; ♂; ♀]. —Botosaneanu 1973: 69 [taxonomic note]. —Malicky 1983b: 57 [atlas; ♂; ♀]. —Malicky 2004a: 66 [atlas]. —Malicky 2005b: 547 [checklist]. —Kjærandsen and Ito 2009: 178 [checklist]. —Oláh and Kovács 2018: 180 [♂].

**Distribution.** —Yemen.

***chora*** Malicky & Chantaramongkol, 2007: 1027 [type locality: Nepal, Mahadev Khola, 27°53'N, 85°39'E, 1300 m; Collection Malicky; ♂]. —Kjærandsen and Ito 2009: 178 [checklist]. —Mattern 2015: 501 [distribution].

**Distribution.** —Nepal.

***dironga*** Oláh & Johanson, 2010: 26 [type locality: Vietnam, Lamdong Province, Baoloc, Baco stream; Collection Oláh; ♂].

**Distribution.** —Vietnam.

***feredougoubae*** (Gibon, 1987a): 123 [type locality: sur la Férédougouba (bassin du Sassandra; Côte d’Ivoire); MNHN; ♂; in *Dhatrichia*]. —Kjærandsen and Andersen 1997: 244 [distribution]. —Kjærandsen 2004: 9 [to *Microptila*]. —Kjærandsen and Ito 2009: 178 [checklist].

**Distribution.** —Côte d’Ivoire, Ghana.

***genka*** Ito, 2017a: 107 [type locality: Japan, Ryukyu Islands, Okinawa-jima, Nago-shi, Genka, hygropetric zone near Hogen-hashi, 26°36'16"N, 128°04'29"E, 65 m a.s.l.; CMB-ZI; ♂; ♀].

**Distribution.** —Japan.

***hamatilis*** Zhou, Yang, & Morse, 2016: 208 [type locality: China, Yun-nan Province, Da-li City, Zhong-he Village, N25.35°, E100.13°, alt. 2200 m; NAUJ; ♂].

**Distribution.** —China.

***hintama*** Oláh, 1989: 271 [type locality: Vietnam, Hoabinh, 20 km from the city in the direction of Tanlac, singled along a small stream under trees; Collection Oláh; ♂]. —Armitage et al. 2005: 27 [checklist]. —Oláh and Johanson 2010: 26 [distribution]. —Malicky and Chantaramongkol 2007: 1026 [♂; distribution]. —Kjærandsen and Ito 2009: 178 [checklist]. —Malicky 2010a: 37 [atlas; ♂]. —Bunlue et al. 2012: 15 [distribution].

—*xedapa* Oláh, 1989: 271 [type locality: Vietnam, Tamdao, 1300 m a.s.l.; Collection Oláh; ♂]. —Armitage et al. 2005: 27 [checklist]. —Malicky and Chantaramongkol 2007: 1026 [to synonymy].

**Distribution.** —Thailand, Vietnam.

***ikaros*** Malicky, 2004b: 295 [type locality: [Nepal, Bardia National Park], am Rande der nordindischen Ebene im Südwesten von Nepal im Bereich des ersten Hügelkammes des Himalaya (Siwalik Range), bei dem Dorf Babai Basar in der Nähe der Straße von Nepalganj nach Birendranagar, ungefähr 30 km flussaufwärts vom Lager 1 (28°21'N, 81°42'E), von hygropetrischen Stellen entlang der Straße bei Babai Bazar; Collection Malicky; ♂]. —Malicky 2006: 253 [checklist]. —Kjærandsen and Ito 2009: 178 [checklist]. —Mattern 2015: 501 [distribution]. —Oláh and Kovács 2018: 180 [♂].

**Distribution.** —Nepal.

***indra*** Schmid, 1960: 88 [type locality: [Pakistan] Karakoram, Shinghai Gan; CNC; ♂]. —Schmid 1958c: 220 [as new species, *nomen nudum*; distribution]. —Kjærandsen and Ito 2009: 178 [checklist]. —Oláh and Kovács 2018: 180 [♂]. —Lonsdale 2020: 36 [holotype depository].

**Distribution.** —Pakistan.

***innokentiyi*** Malicky, Ivanov, & Melnitsky, 2011: 1493 [type locality: [Indonesia], Lombok, Senaru WF, 455 m; ZIN; ♂]. —Malicky et al. 2014a: 6 [distribution]. —Malicky et al. 2016: 92 [distribution].

**Distribution.** —Indonesia.

***minutissima*** Ris, 1897: 417 [type locality: [Switzerland], Zürichberg, in grosser Menge an einer mit Schachtelhalmen bewachsenen kleinen Quelle im Trichtenhausertobel; depository not designated; ♂]. —Morton 1904: 325 [distribution]. —Botosaneanu 1967: 294 [distribution]. —Botosaneanu and Malicky 1978: 341 [checklist]. —Kumanski 1979: 17 [♂; distribution]. —Malicky 1983b: 57 [atlas; ♂; ♀]. —Kumanski 1985: 116 [♂]. —Cianficconi et al. 1993: 261 [distribution]. —Moretti et al. 1996: 297 [distribution]. —Chvojka 1997: 27–38 [distribution]. —Cianficconi et al.1999: 57, 59 [distribution; ♂]. —Malicky 1999f: 32 [distribution]. —Valle 2001: 68 [distribution]. —Graf et al. 2004: 31 [larva]. —Malicky 2004a: 66 [atlas]. —Malicky 2005b: 547 [checklist]. —Malicky 2005a: 66 [distribution]. —Lubini-Ferlin and Vicentini 2005: 68 [checklist]. —Kjærandsen and Ito 2009: 178 [checklist]. —Waringer and Graf 2011: 282 [larval synopsis]. —Oláh and Kovács 2018: 180 [♂].

**Distribution.** —Albania, Austria, Bulgaria, Greece, Italy, Switzerland.

***nakama*** Ito, 2017a: 107 [type locality: Japan, Ryukyu Islands, Iriomote-jima, Nakama-gawa river system, Nishi-funatsuki-gawa, Nishi-funatsuki-bashi, 24°18'10"N, 123°51'34"E, 10 m a.s.l.; CMB-ZI; ♂].

**Distribution.** —Japan.

***orienthula*** Kjærandsen & Ito, 2009: 177 [type locality: Japan, Hokkaido, Oshima, Shiriuchi-cho, hygropetric habitat beside Idesu River, 41°34'N, 140°20'E, 170–200 m a.s.l.; CMB-ZI; ♂]. —Tanida and Kuranishi 2016: 71 [checklist]. —Ito 2017a: 105 [♂; ♀; distribution].

**Distribution.** —Japan.

***pasak*** Wells, 1993: 352 [type locality: [Indonesia] Bali, Bali Barat, Sg. Bandangung, N of Medewi; NTM; ♂]. —Kjærandsen and Ito 2009: 178 [checklist]. —Malicky 2010a: 37 [atlas; ♂]. —Malicky et al. 2014a: 6 [distribution].

**Distribution.** —Indonesia.

***rinjani*** Malicky, Ivanov, & Melnitsky, 2011: 1493 [type locality: [Indonesia], Lombok, Senaru, irrigation, 8°18'29"S, 116°24'27"E, 508 m; ZIN; ♂]. —Malicky et al. 2014a: 6 [distribution]. —Malicky et al. 2016: 92 [distribution]. —Oláh and Kovács 2018: 180 [♂].

**Distribution.** —Indonesia.

***roudra*** Schmid, 1960: 87 [type locality: [Pakistan] Himalaya, Kawai; CNC; ♂]. —Schmid 1958c: 220 [as new species, *nomen nudum*]. —Malicky and Chantaramongko 2007: 1027 [distribution]. —Kjærandsen and Ito 2009: 178 [checklist]. —Mattern 2015: 501 [distribution]. —Lonsdale 2020: 39 [holotype depository].

**Distribution.** —Nepal, Pakistan.

***taji*** Wells, 1993: 352 [type locality: [Indonesia] Bali, Bali Barat, Sg. Bandangung, N of Medewi; NTM; ♂]. —Kjærandsen and Ito 2009: 178 [checklist]. —Malicky et al. 2014a: 6 [distribution].

**Distribution.** —Indonesia.

***tyndareos*** Malicky & Chantaramongkol, 2007: 1027 [type locality: Thailand, Boripat WF, 6°59'N, 100°09'E, 200 m; Collection Malicky; ♂]. —Kjærandsen and Ito 2009: 178 [checklist]. —Malicky 2010a: 37 [atlas; ♂].

**Distribution.** —Thailand.

#### Genus *Missitrichia* Wells, 1991

*Missitrichia* Wells, 1991: 508 [type species: *Missitrichianusam* Wells, 1991, original designation].

*Missitrichia* currently contains three species occurring in Papua New Guinea and Indonesia. There are several similarities between *Missitrichia* and *Hydroptila*, but the two can be distinguished by differences in wing venation, features of the adult head, and features of the male genitalia (Wells 1991). The larvae are unknown.

***kunkora*** Oláh, 2012: 49 [type locality: Indonesia, Papua, Raja Empat Archipelago, Batanta Island, Warmon Creek, 1. waterfall; Collection Oláh; ♂]. —Oláh and Kovács 2018: 179 [distribution].

**Distribution.** —Indonesia.

***nusam*** Wells, 1991: 510 [type locality: Papua New Guinea, Morobe Province, Mt Missam, 1300 m; BPBM; ♂].

**Distribution.** —Papua New Guinea.

***vagot*** Oláh, 2013: 67 [type locality: Indonesia, Batanta Island, northern coast, small stream with dry mouth, 1000–1500 m above Dry mouth; Collection Oláh; ♂]. —Oláh 2016: 113 [distribution].

**Distribution.** —Indonesia.

#### Genus *Mulgravia* Wells, 1982

*Mulgravia* Wells, 1982: 262 [type species: *Mulgraviacoronata* Wells, 1982, original designation]. —Wells 1997: 1 [checklist].

The genus *Mulgravia* comprises two species known from Australia. Adults of the genus share many similarities with *Hellyethira* but can be distinguished by several features of the male genitalia (Wells 1982). The larval stage is unknown.

***carteri*** Wells, 1983: 647 [type locality: Australia, New South Wales, Clarence R., at Yates Crossing; NMV; ♂; ♀]. —Neboiss 1986: 68 [atlas; ♂; ♀].

**Distribution.** —Australia.

***coronata*** Wells, 1982: 262 [type locality: Australia, Queensland, Little Mulgrave River; ANIC; ♂]. —Neboiss 1986: 68 [atlas; ♂].

**Distribution.** —Australia.

#### Genus *Oxyethira* Eaton, 1873

*Oxyethira* Eaton, 1873: 143 [type species: *Hydroptilacostalis* Curtis, 1834, type species original designation, is a species of *Orthotrichia* according to Neboiss (1963). *Oxyethiracostalis* Curtis sensu Eaton, 1873, is probably *Oxyethiraflavicornis* (Pictet, 1834)]. —McLachlan 1880: 520 [revision]. —Mosely 1939b: 281 [key to the British species]. —Ross 1944: 133 [species key for adults]. —Marshall 1979b: 203 [generic review]. —Blickle 1979: 36 [key to species of America north of Mexico]. —Wells 1981: 104 [key to males of Australian species]. —Kelley and Morse 1982: 260 [key to females from the southern United States]. —Kelley 1984a: 435 [generic revision; classification of subgenera and species groups]. —Kelley 1985: 230 [revision]. —Wells 1985b: 16 [larva; pupa; case]. —Flint 1991b: 51 [key to Antioquian species]. —Wells 1991: 491 [key to males of New Guinea]. —Botosaneanu 1992: 90 [key to species in the Levant]. —Moulton and Stewart 1996: 124 [key to the Interior Highlands of North America]. —Wells 1997: 1–28 [checklist; larvae of Australian species]. —Kachalova in Medvedev 1998: 188 [key to the species of the European part of the USSR]. —Oláh and Johanson 2011: 129 [subgeneric features]. —Wells and Johanson 2015: 87 [key to New Caledonian species].

*Lagenopsyche* Müller, 1879b: 39 [type species: *Lagenopsychespirogyrae* Müller, 1879b, subsequent designation by Fischer 1961: 112]. —Müller 1887: 338 [withdrawn in favor of *Oxyethira*]. —Kelley 1984a: 436 [to synonymy].

*Argyrobothrus* Barnard, 1934: 392 [type species: *Argyrobothrusvelocipes* Barnard, 1934, monotypic]. —Ross 1948: 202 [to synonymy]. —Kelley 1984a: 438 [as subgenus].

*Loxotrichia* Mosely, 1937b: 165 [type species: *Loxotrichiaazteca* Mosely, 1937b, original designation]. —Ross 1944: 133 [to synonymy]. —Kelley 1984a: 442 [as subgenus].

*Dampfitrichia* Mosely, 1937b: 169 [type species: *Dampfitrichiaulmeri* Mosely, 1937b, monotypic]. —Ross 1944: 133 [to synonymy]. —Kelley 1984a: 438 [as subgenus].

*Oxytrichia* Mosely, 1939b: 289 [type species: *Oxyethiramirabilis* Morton, 1904, original designation]. —Kimmins 1966: 114 [type species returned to *Oxyethira*, thus synonymizing genus]. —Kelley 1984a: 438 [as subgenus]. —Kachalova in Medvedev 1998: 191 [key to the species of the European part of the USSR, as genus].

*Stenoxyethira* Kimmins, 1951: 194, 207 [type species: *Stenoxyethiraminima* Kimmins, 1951, original designation]. —Marshall 1979b: 207 [generic review]. —Wells 1981: 114 [generic revision]. —Kelley 1984a: 438 [to synonymy with *Oxyethira*].

*Gnathotrichia* Ulmer, 1951 [type species: *Gnathotrichiaisabellina* Ulmer, 1951, original designation]. —Marshall 1979b: 207 [to synonymy with *Stenoxyethira*]. —Wells 1981: 112 [generic revision, as *Gnathotrichia*].

*Dactylotrichia* Kelley, 1984a: 459 [type species: *Oxyethirasantiagensis* Flint, 1982a, original designation, as subgenus].

*Trichoglene* Neboiss, 1977: 43 [type species: *Trichoglenecolumba* Neboiss, 1977, original designation]. —Wells 1981: 106 [considered a synonym of *Oxyethira*]. —Kelley 1984a: 436 [as subgenus].

*Holarctotrichia* Kelley, 1984a: 456 [type species: *Oxyethiradistinctella* McLachlan, 1880, original designation, as subgenus]. —Kelley 1986: 777 [revision].

*Mesotrichia* Kelley, 1984a: 458 [type species: *Oxyethirajamaicensis* Flint, 1968b, original designation, as subgenus]. —Özdikmen 2007: 444 [preoccupied in Apidae by Westwood, 1838: 112, replaced with *Kellyella*].

*Tanytrichia* Kelley, 1984a: 459 [type species: *Oxyethiralongissima* Flint, 1974b, original designation, as subgenus].

*Pacificotrichia* Kelley, 1989: 196 [type species: *Oxyethiraoropedion* Kelley, 1989, original designation, as subgenus].

*Kelleyella* Özdikmen, 2007: 444 [type species: *Oxyethirajamaicensis* Flint, 1968b, original designation, replacement name for *Mesotrichia*]. —Kelley 1984a: 458 [treated as subgenus Mesotrichia].

*Oxyethira* is a large genus of 253 species, including a single fossil species. The genus displays a near world-wide distribution, excluded only from the polar regions. The larvae are distinct and known for feeding on green filamentous algae (Marshall 1979b). The genus was divided into eleven species groups (*azteca*, *bidentata*, *distinctella*, *falcata*, *flavicornis*, *mirabilis*, *pallida*, *rivicola*, *simplex*, *ulmeri*, and *zeronia*) based on features of the male genitalia (Marshall 1979b). The genus was later divided into eleven subgenera, as listed above, which do not correspond to Marshall’s species groups (Kelley 1984a, 1985, 1986, 1989). The larvae of *Oxyethira* were first described, under the name *Lagenopsychespirogyrae*, by Müller (1879b), with many other species having been described since (Hudson 1886; Morton 1887; Barnard 1934; Ross 1944; Nielsen 1948; Macdonald 1950; Mosely and Kimmins 1953; Ulmer 1957; Jacquemart and Coineau 1962; Flint 1964; Lepneva 1964; Hickin 1967; Jacquemart 1973; Back 1983; Ito and Kawamura 1984; Wells 1985b; Keiper and Walton 1999).

***abacatia*** (*Oxytrichia*) Denning, 1947a: 12 [type locality: [United States], Georgia, Macon; ESUW; ♂]. —Blickle 1979: 53, 93 [checklist; ♂]. —Blickle 1980: 102 [♂]. —Kelley and Morse 1982: 257, 265 [checklist; ♀]. —Kelley 1984a: 440 [checklist]. —Harris et al. 1991: 242 [distribution]. —Floyd et al. 1993: 91 [phenology; distribution]. —Moulton and Stewart 1997: 350 [checklist]. —Moulton and Harris 1999: 546 [♂; distribution]. —Pescador et al. 2004: 133 [checklist]. —Harris et al. 2012: 10 [checklist]. —Flint 2014: 90 [distribution]. —Denson et al. 2016: 6 [distribution].

**Distribution.** —U.S.A.

***abbreviata*** (*Trichoglene*) Wells & Johanson, 2015: 46 [type locality: New Caledonia, Province Sud, Monts des Koghis, ca 800 m S Koghi Restaurant, 22.18447°S, 166.50315°E, 400 m; MNHN; ♂]. —Johanson and Wells 2019: 93 [checklist].

**Distribution.** —New Caledonia.

***absona*** (unplaced) Flint, 1991b: 51 [type locality: Colombia, Dpto. Antioquia, Quebrada La Cebolla, El Retiro; NMNH; ♂]. —Muñoz-Quesada 2000: 278 [checklist].

**Distribution.** —Colombia.

***acegua*** (*Dactylotrichia*) Angrisano, 1995a: 510 [type locality: Uruguay, Cerro Largo, Sa. da Acegua; FHCU; ♂]. —Angrisano 1999: 34 [checklist].

**Distribution.** —Uruguay.

***aculea*** (*Dampfitrichia*) Ross, 1941a: 53 [type locality: United States, Oklahoma, Honey Creek, Turner Falls State Park; INHS; ♂]. —Bueno-Soria and Flint 1978: 204 [distribution]. —Blickle 1979: 53, 91 [checklist; ♂]. —Kelley and Morse 1982: 257, 266 [checklist; ♀]. —Kelley 1984a: 439 [checklist]. —Bowles and Mathis 1992: 32 [distribution]. —Moulton et al. 1993: 21 [distribution]. —Moulton et al. 1994: 170 [distribution]. —Moulton and Stewart 1996: 125 [♂; distribution]. —Harris et al. 1996: 240 [distribution]. —Moulton and Stewart 1997: 350 [checklist]. —Baumgardner and Bowles 2005: 11 [distribution; biology]. —Blinn and Ruiter 2005: 69 [distribution; biology]. —Blinn and Ruiter 2006: 333 [biology; distribution]. —Bowles et al. 2007: 22 [distribution; biology]. —Bueno-Soria et al. 2007: 33 [distribution]. —Mendez et al. 2019: 128 [checklist].

**Distribution.** —Mexico, U.S.A.

***acuta*** (unplaced) Kobayashi, 1977: 6 [type locality: [Japan], Utonai Pond, Utonai, Tomakami-shi, Hokkaido; depository not designated; ♂; ♀]. —Ito and Kawamura 1984: 313 [larva; pupa; case; biology]. —Kelley 1984a: 442 [to *Hellyethira*]. —Ito et al. 1993: 142 [checklist; as *Oxyethira*]. —Tanida et al. 2005: 442 [larva; as *Oxyethira*]. —Oláh and Ito 2013: 32 [♂; distribution]. —Tanida and Kuranishi 2016: 72 [checklist]. —Ito and Oláh 2017: 3 [♂; ♀; distribution].

**Distribution.** —Japan.

***aeola*** (*Oxytrichia*) Ross, 1938a: 117 [type locality: [Canada], British Columbia, Vancouver, along Seymour Creek; INHS; ♂]. —Roy and Harper 1979: 151 [distribution]. —Blickle 1979: 53, 93 [checklist; ♂]. —Blickle 1980: 102 [♂]. —Kelley 1984a: 440 [checklist]. —Harper 1989: 541 [distribution]. —Harper 1990: 49 [distribution; biology]. —Masteller and Flint 1992: 70 [distribution]. —Monson and Holzenthal 1993: 442 [checklist]. —Moulton and Harris 1999: 547 [♂; distribution]. —Houghton et al. 2001: 505 [distribution]. —Houghton et al. 2011b: 6 [phenology; habitat; distribution]. —Houghton et al. 2017: 63 [checklist].

**Distribution.** —Canada, U.S.A.

***ahipara*** (*Trichoglene*) Wise, 1998: 21 [type locality: [New Zealand], Ahipara Plateau, Upper Hunahuna Stm. Vy.; AMNZ; ♂]. —Ward and Henderson 2004: 10 [checklist].

**Distribution.** —New Zealand.

***akibeel*** (*Dampfitrichia*) Malicky, 2012: 1266 [type locality: Indonesian, Kalimantan, PT Silva Rimba Lestari (area), Camp Limbang, 60 m, 0.07'N, 116°18'E; NMPC; ♂].

**Distribution.** —Indonesia.

***alaluz*** (*Dampfitrichia*) Botosaneanu, 1980: 112 [type locality: Cuba, Jardin Botanique de Soledad, Cienfuegos, Prov. Las Villas; ZMUA; ♂]. —Botosaneanu 1979: 50 [distribution]. —Kelley 1984a: 439 [checklist]. —Flint 1996a: 16 [checklist]. —Botosaneanu 2002b: 87 [checklist]. —Naranjo López and González Lazo 2005: 149 [checklist].

**Distribution.** —Cuba.

***albaeaquae*** (*Mesotrichia*) Botosaneanu, 1995a: 30 [type locality: Dominican Republic, Salto Aqua Blanca, Rio Grande, 3 km from Convento; ZMUA; ♂; ♀]. —Flint and Pérez-Gelabert 1999: 41 [checklist]. —Botosaneanu 2002b: 87 [checklist]. —Flint and Sykora 2004: 41 [distribution; to *Mesotrichia*]. —Pérez-Gelabert 2008: 301 [checklist].

**Distribution.** —Dominican Republic.

***albiceps*** (*Trichoglene*) (McLachlan, 1862): 304 [type locality: New Zealand; depository not designated; ♂; in *Hydroptila*]. —Eaton 1873: 130, 145 [revision; to *Oxyethira*]. —Mosely 1924: 673 [♂; distribution]. —Mosely and Kimmins 1953: 512 [♂]. —Wise 1964: 253 [distribution]. —Leader 1972: 196 [♂; ♀; pupal case]. —Wise 1972: 260 [larval-pupal cases; distribution]. —Cowley 1978: 672 [distribution; larva]. —Wise 1978: 113 [distribution]. —Towns 1981: 204 [distribution; life history]. —Kelley 1984a: 436 [checklist]. —Neboiss 1986: 84 [atlas; ♂; ♀]. —Bayly 1990: 52 [distribution; biology]. —Quinn et al. 1992: 265, 267 [distribution; biology]. —Winterbourn 1998: 68 [distribution; biology]. —Collier and Smith 1998: 57 [distribution; biology]. —Joy and Death 2000: 118 [distribution]. —Winterbourn and Crowe 2001: 1485 [biology]. —Ward and Henderson 2004: 10 [checklist]. —Quinn et al. 2004: 143 [distribution]. —James and Suren 2009: 2232 [biology]. —Oláh and Johanson 2010a: 27 [distribution]. —Larned and Kilroy 2014: 353 [biology]. —Wells and Kjer 2016: 51 [distribution].

**Distribution.** —New Zealand, Norfolk Island.

***allagashensis*** (*Oxyethira*) Blickle, 1963: 20 [type locality: [United States], Maine, Allagash; INHS; ♂]. —Blickle 1979: 53, 95 [checklist; ♂]. —Roy and Harper 1979: 151 [checklist]. —Kelley 1984a: 437 [checklist]. —Harper 1989: 541 [distribution]. —Harper 1990: 49 [distribution; biology].

**Distribution.** —Canada, U.S.A.

***amieu*** (*Trichoglene*) Wells & Johanson, 2015: 52 [type locality: New Caledonia, Chute, ~15 km N Col d’Amieu on La Foa-Canala Rd; MNHN; ♂]. —Johanson and Wells 2019: 93 [checklist].

**Distribution.** —New Caledonia.

***anabola*** (*Oxytrichia*) Blickle, 1966: 185 [type locality: [United States], New Hampshire, Durham; INHS; ♂]. —Roy and Harper 1975: 1082 [distribution]. —Blickle 1979: 53, 93 [checklist; ♂]. —Roy and Harper 1979: 151 [checklist]. —Blickle 1980: 102 [♂]. —Roy and Harper 1981: 105 [distribution]. —Steven and Hilsenhoff 1984: 164 [distribution]. —Kelley 1984a: 440 [checklist]. —Harper 1989: 541 [distribution]. —Harris et al. 1991: 243 [distribution]. —Monson and Holzenthal 1993: 442 [checklist]. —Moulton and Harris 1999: 547 [♂; distribution]. —Huryn and Harris 2000: 193 [distribution]. —Houghton et al. 2001: 505 [distribution]. —Flint et al. 1994: 4 [distribution]. —Myers et al. 2011: 108 [distribution]. —Houghton et al. 2017: 63 [checklist].

**Distribution.** —Canada, U.S.A.

***andina*** (*Oxytrichia*) Kelley, 1983: 52 [type locality: Argentina, Rio Negro Prov., Rio Guillelmo, Villa Mascardi; NMNH; ♂]. —Kelley 1984a: 440 [checklist]. —Angrisano 1999: 34 [checklist].

**Distribution.** —Argentina, Chile.

***angustella*** (unplaced) Martynov, 1933: 139 [type locality: [Japan], Matsumoto, Prov. of Shinano, a limnocrene; depository not designated; ♀]. —Kelley 1984a: 442 [checklist]. —Ito et al. 1993: 142 [checklist]. —Tanida and Kuranishi 2016: 72 [checklist]. —Ito and Oláh 2017: 6 [♂; ♀; distribution]. —Nozaki et al. 2019: 168, 173 [distribution; seasonality].

—*kakida* Oláh & Ito, 2013: 30 [type locality: Japan, Honshu, Shizuoka, Shimizu-cho, kakida-gawa, N35°06'11" E138°54'10", 13 m; CMB-ZI; ♂]. —Nozaki and Tanida 2007: 256 [misidentified as *O.josifovi*, according to Oláh and Ito 2013: 30]. —Malicky and Chantaramongkol 2007: 1030 [misidentified as *O.datra*, according to Oláh and Ito 2013: 30]. —Tanida and Kuranishi 2016: 72 [checklist]. —Ito and Oláh 2017: 6 [to synonymy].

**Distribution.** —Japan.

***apinolada*** (*Oxytrichia*) Holzenthal & Harris, 1992: 157 [type locality: Costa Rica, Guanacaste, Parque Nacional Rincón de la Vieja, Quebrada Agua Apinolada, 10.759°N, 85.292°W; NMNH; ♂]. —Ríos-Touma et al. 2017: 10 [distribution]. —Harris and Armitage 2019: 5, 21 [distribution].

**Distribution.** —Costa Rica, Ecuador, Panama.

***arantala*** (unplaced) Oláh & Johanson, 2011: 130 [type locality: Peru, San Martin Prov., creek crossing rd. Juan Guerra-Chazuta, 14 km (rd.) E Colombia Bridge, 6°35.594'S 76°13.172'W; NHRS; ♂].

**Distribution.** —Peru.

***araya*** (*Holarctotrichia*) Ross, 1941b: 15 [type locality: [Canada], Hampton, New Brunswick; INHS; ♂]. —Etnier 1965: 147 [distribution]. —Roy and Harper 1979: 151 [checklist]. —Blickle 1979: 54, 91 [checklist; ♂]. —Kelley 1984a: 438 [checklist]. —Harper 1989: 541 [distribution]. —Monson and Holzenthal 1993: 442 [checklist]. —Wiggins and Parker 1997: 794 [distribution]. —Houghton et al. 2001: 505 [distribution; as *arraya*]. —Houghton et al. 2017: 63 [checklist].

**Distribution.** —Canada, U.S.A.

***archaica*** (*Holarctotrichia*) Malicky, 1975: 83 [type locality: Portugal, Rio Beça, Vidoeiro; Collection Malicky; ♂]. —Botosaneanu and Malicky 1978: 340 [checklist]. —Malicky 1983b: 59 [atlas; ♂]. —Kelley 1984a: 438 [checklist]. —González et al. 1986: 113 [distribution]. —Malicky 2004a: 71 [atlas]. —Malicky 2005b: 547 [checklist; taxonomic note]. —González and Menéndez 2011: 119 [distribution].

**Distribution.** —Portugal, Spain.

***arctodactyla*** (*Dactylotrichia*) Kelley, 1983: 42 [type locality: Venezuela, Merida State, Mucujun Valley, 19 km NE Merida; NMNH; ♂]. —Kelley 1984a: 442 [checklist].

**Distribution.** —Venezuela.

***argentinensis*** (unplaced) Flint, 1982a: 45 [type locality: Argentina, Pcia. Buenos Aires, Arroyo Pescado, Rt. 11, 15 km E La Plata; NMNH; ♂]. —Flint 1982b: 42 [distribution]. —Kelley 1984a: 442 [checklist]. —Angrisano 1995a: 510 [distribution]. —Angrisano 1995b: 34 [larva]. —Mangeaud 1996: 154 [distribution]. —Angrisano 1999: 34 [checklist]. —Angrisano and Sganga 2007: 36 [♂; distribution; as *O.argentiniensis*].

**Distribution.** —Argentina, Uruguay.

***arizona*** (*Dampfitrichia*) Ross, 1948: 202 [type locality: United States, Arizona, Pinal County, Superior, in Boyce Thompson Arboretum; INHS; ♂]. —Bueno-Soria and Flint 1978: 204 [distribution]. —Blickle 1979: 54, 91 [checklist; ♂]. —Kelly and Morse 1982: 257, 263 [checklist; ♀]. —Kelley 1984a: 439 [checklist]. —Holzenthal 1988: 62 [distribution; as *arizonica*]. —Botosaneanu 1989: 101 [distribution]. —Holzenthal and Harris 1992: 172 [distribution]. —Flint and Sykora 1993: 49 [checklist as *arizonensis*]. —Keiper and Walton 1999: 214 [larva; biology]. —Maes 1999: 1194 [checklist]. —Botosaneanu 2002b: 87 [checklist]. —Botosaneanu and Thomas 2005: 44 [distribution]. —Naranjo López and González Lazo 2005: 149 [checklist]. —Blinn and Ruiter 2005: 69 [distribution; biology]. —Blinn and Ruiter 2006: 333 [biology; distribution]. —Blinn and Ruiter 2009a: 305 [biology]. —Chamorro-Lacayo et al. 2007: 43 [distribution]. —Harris et al. 2012: 10 [♂; distribution]. —Armitage et al. 2015b: 5 [distribution]. —Armitage et al. 2015a: 7 [checklist]. —Armitage and Harris 2018b: 98 [checklist]. —Armitage and Harris 2018c: 283 [distribution]. —Mendez et al. 2019: 119 [checklist].

**Distribution.** —Costa Rica, Cuba, Dominica, Jamaica, Martinique, Mexico, Nicaragua, Panama, Puerto Rico, U.S.A.

***arok*** (*Trichoglene*) Oláh & Johanson, 2010a: 27 [type locality: New Caledonia, Province Sud, Monts Ksa Ne Mwa, on road between Nouméa and Yaté, 2.0 km E Pic Mouirange, 22°12.356'S, 166°40.798'E, 220 m; MNHN; ♂]. —Wells and Johanson 2015: 51 [♂; distribution]. —Johanson and Wells 2019: 93 [checklist].

**Distribution.** —New Caledonia.

***artuvillosa*** (*Dampfitrichia*) (Wells, 1981): 114 [type locality: [Australia] Western Australia, Mitchell Plateau, Camp Creek at Crusher; WAM; ♂; ♀; in *Stenoxyethira*, as *artuvillosus*]. —Kelley 1984a: 438 [checklist]. —Neboiss 1986: 82[atlas; ♂; ♀].

**Distribution.** —Australia.

***azteca*** (*Loxotrichia*) (Mosely, 1937b): 165 [type locality: Mexico, Chiapas, Dolores; NHMUK; ♂; in *Loxotrichia*]. —Flint 1968a: 55 [♂; ♀; distribution]. —Flint 1974b: 66 [♂; distribution]. —Bueno-Soria and Flint 1978: 205 [distribution]. —Blickle 1979: 93 [♂]. —White and Fox 1979: 76 [phoretic association observed]. —Flint 1981: 30 [♂; distribution]. —Kelley and Morse 1982: 257, 264 [checklist; ♀]. —Lewis and Fairchild 1983:135 [biology]. —Kelley 1984a: 442 [checklist]. —Holzenthal 1988: 62 [distribution]. —Flint and Reyes 1991: 488 [♂; ♀; distribution]. —Holzenthal and Harris 1992: 172 [distribution]. —Aguila 1992: 539 [distribution]. —Botosaneanu and Sakal 1992: 202 [distribution]. —Botosaneanu and Alkins-Koo 1993: 27 [distribution]. —Flint and Sykora 1993: 57 [distribution]. —Moulton et al. 1993: 21 [distribution]. —Flint 1996b: 99 [distribution]. —Flint 1996c: 401 [distribution]. —Moulton and Stewart 1996: 125 [♂; distribution]. —Moulton and Stewart 1997: 350 [checklist]. —Muñoz-Quesada 2000: 278 [checklist]. —Botosaneanu 2002b: 88 [distribution; biology]. —Zeullig et al. 2006: 43 [distribution]. —Bowles et al. 2007: 22 [distribution]. —Bueno-Soria et al. 2007: 33 [distribution]. —Chamorro-Lacayo et al. 2007: 43 [distribution]. —Oláh and Johanson 2011: 131 [distribution]. —Armitage et al. 2015a: 7 [checklist]. —Ríos-Touma et al. 2017: 10 [checklist]. —Razo-González 2018: 32 [distribution]. —Armitage and Harris 2018b: 98 [checklist]. —Armitage and Harris 2018c: 283 [distribution]. —Barba-Álvarez et al. 2019: 86 [distribution].

**Distribution.** —Belize, Colombia, Costa Rica, Ecuador, French Guiana, Grenada, Guatemala, Mexico, Nicaragua, Panama, Peru, Suriname, Trinidad, Venezuela, U.S.A.

***bamaga*** (*Trichoglene*) Wells & Dostine, 2016: 594 [type locality: [Australia] North East Queensland, Bamaga; ANIC; ♂].

**Distribution.** —Australia.

***baotianensis*** (*Oxyethira*) Xue, Luo, & Guo, 1992: 353 [type locality: [China] Baotianman, Henan; HAUZ; ♂]. —Yang et al. 2016: 476 [checklist].

**Distribution.** —China.

***baritu*** (*Dactylotrichia*) Angrisano, 1995b: 30 [type locality: Argentina, Salta, Parque Nacional Baritú; MACN; ♂]. —Angrisano 1999: 34 [checklist].

**Distribution.** —Argentina.

***bettyae*** (*Tanytrichia*) Thomson & Holzenthal, 2012: 29 [type locality: Venezuela, Guárico, UCV San Nicolasito Field Station, 08°8.296'N, 66°24.459'W, 62 m; UMSP; ♂]. —de Souza et al. 2013: 586 [distribution]. —Paprocki and França 2014: 51 [checklist]. —Rocha et al. 2018: 153 [checklist]. —Moreno et al. 2020: 266 [distribution].

**Distribution.** —Brazil, Venezuela.

***bicornuta*** (*Tanytrichia*) Kelley, 1983: 45 [type locality: Brazil, Amazonas State, Igarape do Mendu, nr. Manaus; NMNH; ♂]. —Kelley 1984a: 440 [checklist]. —Angrisano 1999: 34 [checklist]. —Paprocki et al. 2004: 11 [checklist]. —Santos et al. 2009: 36 [checklist]. —Paprocki and França 2014: 51 [checklist]. —Rocha et al. 2018: 153 [checklist].

**Distribution.** —Brazil.

***bidentata*** (*Oxytrichia*) Mosely, 1934a: 155 [type locality: Argentina, Terr. Rio Negro; NHMUK; ♂]. —Mosely 1939b: 289 [to *Oxytrichia*]. —Flint 1974a: 88 [checklist; to *Oxyethira*]. —Kelley 1984a: 440 [checklist]. —Angrisano 1999: 34 [checklist]. —Muzón et al. 2005: 57 [distribution]. —Miserendino and Brand 2007: 312 [biology]. —Brand and Miserendino 2011a: 35 [biology]. —Oláh and Johanson 2011: 132 [distribution]. —Brand et al. 2012: 90 [biology]. —Brand and Miserendino 2014: 77 [community ecology].

**Distribution.** —Argentina, Chile.

***bifurcata*** (*Oxyethira*) Yang & Kelley in Yang et al. 1997: 99 [type locality: [China], Sichuan Province, Jiangjinxian, Simianshan, Feilonghe, 800 m; NAUJ; ♂]. —Yang et al. 2005: 458 [checklist]. —Yang et al. 2016: 476 [checklist].

**Distribution.** —China.

***bogambara*** (*Oxyethira*) Schmid, 1958b: 67 [type locality: [Sri Lanka] Ceylan, Kandapola (C. P., 6300 ft) 1-III, Bamuraella Oya, en amont de la précédente station, ruisseau clair et profond, formant des méandres accentués, dans un pâturage humide à Aracées blanches; depository not designated; ♂]. —Kelley 1984a: 437 [checklist]. —Oláh 1989: 287 [distribution]. —Wells and Dudgeon 1990: 170 [distribution]. Wells 1990c: 117 [distribution]. —Xue and Yang 1991: 19 [distribution]. —Wells 1991: 493 [distribution]. —Wells and Huisman 1992: 107 [distribution]. —Yang et al. 1997b: 93 [checklist]. —Wells and Malicky 1997: 186 [distribution]. —Wells and Mey 2002: 130 [distribution]. —Yang et al. 2005: 458 [checklist]. Mey 2006b: 203 [distribution]. —Malicky and Chantaramongkol 2007: 1029 [distribution]. —Malicky 2007a: 177 [checklist]. —Melnitsky and Malicky 2008: 25 [distribution]. —Malicky 2009b: 10 [distribution]. —Oláh and Johanson 2010a: 29 [distribution]. —Malicky 2010a: 41 [atlas; ♂]. —Malicky et al. 2014a: 6 [distribution]. —Mattern 2015: 501 [distribution]. —Yang et al. 2016: 476 [checklist]. —Melnitsky et al. 2019: 539 [distribution]. —Malicky et al. 2019: 429 [distribution]. —Laudee and Mesuk 2019: 110 [distribution].

—*hainanensis* Yang & Xue, 1992: 230 [type locality: Bawangling, Hainan; NAUJ; ♂]. —Yang et al. 1997b: 93 [checklist]. —Yang et al. 2005: 458 [checklist]. —Malicky and Chantaramongkol 2007: 1030 [to synonymy].

—*paramartha* Schmid, 1960: 98 [type locality: [Pakistan], Bélouchistan, Central Zarghun; CNC; ♂]. —Schmid 1958c: 220 [as new species, *nomen nudum*]. —Kelley 1984a: 437 [checklist]. —Malicky and Chantaramongkol 2007: 1030 [to synonymy]. —Lonsdale 2020: 38 [holotype depository].

**Distribution.** —Australia, Bali, Borneo, China, Hong Kong, Indonesia, Malaysia, Nepal, New Guinea, Philippines, Sri Lanka, Thailand, Vietnam.

***brasiliensis*** (unplaced) Kelley, 1983: 49 [type locality: Brazil, Para State, Rio Cururu, area of Missao Cururu; NMNH; ♂]. —Kelley 1984a: 442 [checklist]. —Angrisano 1999: 35 [checklist]. —Paprocki et al. 2004: 11 [checklist]. —Santos et al. 2009: 36 [checklist]. —Paprocki and França 2014: 51 [checklist]. —Rocha et al. 2018: 153 [checklist].

**Distribution.** —Brazil.

***brevis*** (*Trichoglene*) Wells, 1981: 110 [type locality: [Australia] Western Australia, Cape Leeuwin National Park Spring; NMV; ♂; ♀]. —Kelley 1984a: 436 [checklist]. —Neboiss 1986: 84 [atlas; ♂; ♀].

**Distribution.** —Australia.

***buenoi*** (unplaced) Harris & Armitage, 2019: 16 [type locality: Panama, Bocas del Toro Province, Quebrada Rambala, near Rambala Jungle Lodge, 3.74 km SSE Rambala, 8.91627°N and 82.15469°W, 120 m; COZEM; ♂].

**Distribution.** —Panama.

***burkina*** (unplaced) Gibon, Guenda, & Coulibaly, 1994: 110 [type locality: Burkina Faso, sur la haute Mouhoun à Orodara; MNHN; ♂].

**Distribution.** —Burkina Faso.

***caledoniensis*** (*Trichoglene*) Kelley, 1989: 196 [type locality: New Caledonia, Plum, 20–60 m; BPBM; ♂]. —Wells 1995: 233 [distribution]. —Wells and Johanson 2015: 49 [♂; distribution]. —Johanson and Wells 2019: 93 [checklist].

**Distribution.** —New Caledonia.

***calori*** (*Dampfitrichia*) de Souza & Santos, 2017: 485 [type locality: Brazil, Bahia, Barreiras, Cachoeira Acaba Vidas, 12°8'00"S, 44°59'00"W [approximate coordinates]; DZRJ; ♂]. —Rocha et al. 2018: 152 [checklist].

**Distribution.** —Brazil.

***campanula*** (*Oxyethira*) Botosaneanu, 1970: 291 [type locality: [North Korea], Station 17, Mts. Mjohjang-san, district Hjangsan, Hjangam-ri; MZPW; ♂]. —Kelley 1984a: 437 [checklist]. —Wells and Dudgeon 1990: 171 [distribution]. —Xue and Yang 1991: 19 [distribution]. —Wells and Huisman 1992: 107 [distribution]. —Yang et al. 1997b: 93 [checklist]. —Wells and Huisman 2001: 210 [distribution]. —Wells and Mey 2002: 130 [distribution]. —Yang et al. 2005: 458 [checklist]. —Malicky and Chantaramongkol 2007: 1030 [distribution]. —Malicky 2007a: 177 [checklist]. —Oláh and Johanson 2010a: 29 [distribution]. —Malicky 2010a: 41 [atlas; ♂]. —Malicky 2014a: 1623 [checklist]. —Mattern 2015: 502 [distribution]. —Yang et al. 2016: 476 [checklist]. —Park and Kong 2020: 297 [checklist].

—*aspera* Yang & Kelley in Yang et al. 1997: 97 [type locality: [China], Fujian Province, Wuyishan, Taoyuandong, 100 m from Jiouqu, 235 m; NAUJ; ♂]. —Yang et al. 2005: 458 [checklist]. —Malicky and Chantaramongkol 2007: 1030 [to synonymy]. —Yang et al. 2016: 476 [checklist].

—*lobophora* Mey, 1998a: 553 [type locality: [Philippines, Mindanao] northern slope of Mt. Atuuganon range, 1050 m; ZMHB; ♂]. —Malicky and Chantaramongkol 2007: 1030 [to synonymy].

—*paieon* Malicky, 2004b: 296 [type locality: [Nepal, Bardia National Park], am Rande der nordindischen Ebene im Südwesten von Nepal im Bereich des ersten Hügelkammes des Himalaya (Siwalik Range), unweit des Wehrs des Babai Flusses, über das die Brücke der Ost-West-Haupstraße Nepals (Mahindra Highway), 28°25'N, 81°23'E, 190 m, Budhi Khola; Collection Malicky; ♂]. —Malicky 2006: 253 [checklist]. —Malicky and Chantaramongkol 2007: 1030 [to synonymy].

**Distribution.** —China, Indonesia, Korea, Malaysia, Nepal, Philippines, Taiwan, Thailand.

***campesina*** (*Dampfitrichia*) Botosaneanu, 1977: 275 [type locality: Cuba, Oriente, Baire, Rio Mogote; NMNH; ♂]. —Kelley 1984a: 439 [checklist]. —Kumanski 1987: 26 [distribution]. —Botosaneanu 1979: 51 [distribution]. —Flint 1996a: 16 [checklist]. —Botosaneanu 2002b: 87 [checklist]. —Naranjo López and González Lazo 2005: 149 [checklist].

**Distribution.** —Cuba.

***carajas*** (*Loxotrichia*) Neto, Ribeiro, & Passos, 2019: 388 [type locality: Brazil, Pará, Parauapebas municipality, Serra dos Carajás, low order stream, 6°2'24.828"S, 50°17'38.184"W; MPEG; ♂].

**Distribution.** —Brazil.

***cascadanta*** (*Loxotrichia*) Rocha, Dumas, & de Souza, 2018: 148[type locality: Brazil, Minas Gerais, São Roque de Minas, Parque Nacional da Serra da Canastra, parte baixa da Cachoeira Casca D’anta, Rio São Francisco, 20°18.54'S, 46°31.37'W, ca 900 m elev; DZRJ; ♂].

**Distribution.** —Brazil.

***chitosea*** (*Oxyethira*) Oláh & Ito, 2013: 38 [type locality: Japan, Hokkaido, Ishikari, Chitose-shi, Bibi, Lake Chitose-ko, N42°46'24" E141°43'29", 15 m; CMB-ZI; ♂]. —Tanida and Kuranishi 2016: 72 [checklist]. —Ito and Oláh 2017: 3 [♂; ♀; distribution].

**Distribution.** —Japan.

***chrysocara*** (*Holarctotrichia*) Harris, 2002: 47 [type locality: [United States], Florida, Clay County, Gold head Branch near old mill crossing, 29°49'56"N, 81°56'45"W; NMNH; ♂]. —Pescador et al. 2004: 133 [checklist]. —Harris et al. 2012: 10 [checklist].

**Distribution.** —U.S.A.

***circaverna*** (*Dactylotrichia*) Kelley, 1983: 50 [type locality: Panama, Canal Zone, Madden Dam; NMNH; ♂]. —Kelley 1984a: 439 [checklist]. —Flint 1992: 174 [distribution]. —Aguila 1992: 539 [distribution]. —Angrisano 1995a: 510 [distribution]. —Angrisano 1995b: 30 [larva; case; distribution]. —Angrisano 1999: 34 [checklist]. —Botosaneanu 2002b: 87 [checklist]. —Angrisano and Sganga 2007: 34 [♂; distribution]. —Santos et al. 2009: 42 [checklist]. —Manzo et al. 2014: 166 [distribution]. —Paprocki and França 2014: 51 [checklist]. —Armitage et al. 2015a: 7 [checklist]. —de Souza and Santos 2017: 504 [distribution]. —Ríos-Touma et al. 2017: 10 [checklist]. —Rocha et al. 2018: 152 [checklist]. —Armitage and Harris 2018b: 98 [checklist]. —Moreno et al. 2020: 265 [distribution].

**Distribution.** —Argentina, Brazil, Curacao, Ecuador, Panama, Uruguay.

***cirrifera*** (*Dampfitrichia*) Flint, 1964: 57 [type locality: Puerto Rico, Maricao, at fish hatchery; NMNH; ♂; ♀]. —Flint 1968b: 42 [♂; ♀; distribution]. —Flint 1968a: 55 [♂; ♀; distribution]. —Botosaneanu 1979: 50 [distribution]. —Kelley and Morse 1982: 258 [synonymized with *O.arizona*]. —Kelley 1984a: 439 [checklist]. —Kumanski 1987: 26 [♀; distribution]. —Botosaneanu 1991: 130 [distribution]. —Flint 1996a: 16 [checklist]. —Botosaneanu and Hyslop 1998: 16 [resurrected]. —Flint and Pérez-Gelabert 1999: 41 [checklist]. —Flint and Sykora 2004: 43 [distribution]. —Naranjo López and González Lazo 2005: 149 [distribution; as synonym of *O.arizona*]. —Pérez-Gelabert 2008: 301 [checklist].

**Distribution.** —Cuba, Dominica, Dominican Republic, Haiti, Puerto Rico.

***coercens*** (*Oxyethira*) Morton, 1905: 70 [type locality: [United States], Ithaca, New York; depository not designated; ♂]. —Banks 1907a: 50 [catalogue]. —Betten 1934: 161 [♂; distribution]. —Ross 1944: 137 [♂; distribution]. —Etnier 1965: 147 [distribution]. —Edwards 1973: 506 [distribution]. —Roy and Harper 1975: 1082 [distribution]. —Roy and Harper 1979: 151 [checklist]. —Blickle 1979: 54, 93 [checklist;] . —Parker and Voshell 1981: 4 [checklist]. —Kelley and Morse 1982: 257 [checklist]. —Waltz and McCafferty 1983a: 11 [distribution]. —Harris et al. 1984: 109 [distribution]. —Kelley 1984a: 437 [checklist]. —Bowles and Mathis 1989: 240 [distribution]. —Harris et al. 1991: 244 [distribution]. —Mathis and Bowles 1992: 24 [distribution]. —Bowles and Mathis 1992: 32 [distribution]. —Monson and Holzenthal 1993: 442 [checklist]. —Moulton and Stewart 1996: 126 [♂; distribution]. —Moulton and Stewart 1997: 350 [checklist]. —Huryn and Harris 2000: 193 [distribution]. —Houghton et al. 2001: 505 [distribution]. —Etnier 2010: 486 [distribution]. —Ruiter et al. 2013: 3 [distribution; DNA barcoding; larval-adult association]. —Houghton 2016: 46 [biology; as *coerscens*]. —Houghton et al. 2017: 63 [checklist]. —Bowles et al. 2020: 8 [distribution].

**Distribution.** —Canada, U.S.A.

***colombiensis*** (*Tanytrichia*) Kelley, 1983: 44 [type locality: Colombia, Valle Dept., Rio Raposo; NMNH; ♂]. —Kelley 1984a: 440 [checklist]. —Muñoz-Quesada 2000: 278 [checklist]. —Ríos-Touma et al. 2017: 11 [checklist].

**Distribution.** —Colombia, Ecuador.

***columba*** (*Trichoglene*) (Neboiss, 1977): 43 [type locality: [Australia] Tasmania, Dove River, Cradle Mtn Nat. Park; NMV; ♂; ♀; in *Trichoglene*]. —Wells 1981: 106 [♂; ♀; distribution; to *Oxyethira*]. —Kelley 1984a: 436 [checklist]. —Wells 1985b: 17 [larva, case]. —Neboiss 1986: 83 [atlas; ♂; ♀]. —Neboiss 2002: 54 [checklist]. —Oláh and Johanson 2010a: 30 [distribution].

—*elora* Oláh & Johanson, 2010a: 33 [type locality: Australia, Tasmania, Swansea, Meredith River, under bridge on A3, 42°06.945'S, 148°03.507'E, 15 m; ANIC; ♂]. —Wells 2012: 67 [to synonymy].

**Distribution.** —Australia.

***complicata*** (unplaced) Wells, 1990c: 117 [type locality: [Australia] NE Queensland, Yuccabine Creek; NMV; ♂].

**Distribution.** —Australia.

***copina*** (*Loxotrichia*) Angrisano, 1995b: 32 [type locality: Argentina, Cordoba, Copina; MACN; ♂; larva; case]. —Angrisano 1999: 34 [checklist].

**Distribution.** —Argentina.

***cornutata*** (*Trichoglene*) Wells, 1990c: 119 [type locality: [Australia] Northern Territory, Kakadu National Park, Radon Springs, 12°45'S 132°55'E; NTM; ♂].

**Distribution.** —Australia.

***costaricensis*** (*Dactylotrichia*) Kelley, 1983: 44 [type locality: Costa Rica, Heredia Prov., Los Cartagos; NMNH; ♂]. —Kelley 1984a: 442 [checklist]. —Holzenthal 1988: 62 [distribution].

**Distribution.** —Costa Ria.

***cotula*** (*Oxyethira*) Wells & Dudgeon, 1990: 171 [type locality: Hong Kong, Tai Po Kao Forest stream; NHMUK; ♂]. —Yang et al. 2016: 477 [checklist].

**Distribution.** —Hong Kong.

***cuernuda*** (*Tanytrichia*) Holzenthal & Harris, 1992: 157 [type locality: Costa Rica, Alajuela, Río Pizote, 5 km (air) S Brasilia, 10.972°N, 85.345°W; NMNH; ♂].

**Distribution.** —Costa Rica.

***culebra*** (*Oxytrichia*) Holzenthal & Harris, 1992: 160 [type locality: Costa Rica, Alajuela, Río Pizote, 5 km (air) S Brasilia, 10.972°N, 85.345°W; NMNH; ♂]. —Armitage et al. 2016: 11 [distribution]. —Armitage and Harris 2018b: 98 [checklist]. —Harris and Armitage 2019: 5 [distribution].

**Distribution.** —Costa Rica, Panama.

***dactylonedys*** (*Dactylotrichia*) Kelley, 1983: 42 [type locality: Paraguay, Amambat Dept., Rio Aquidaban, Cerro Cora; NMNH; ♂]. —Kelley 1984a: 442 [checklist]. —Angrisano 1999: 34 [checklist].

**Distribution.** —Paraguay.

***dalmeria*** (*Loxotrichia*) (Mosely, 1937b): 166 [type locality: Mexico, Chiapas, Esmeralda; NHMUK; ♂; in *Loxotrichia*]. —Bueno-Soria and Flint 1978: 205 [distribution]. —Kelley 1984a: 442 [checklist].

**Distribution.** —Mexico.

***datra*** (*Oxyethira*) Oláh, 1989: 287 [type locality: Vietnam, Cucphuong, 400 m a.s.l.; HNHM; ♂]. —Armitage et al. 2005: 27 [checklist]. —Malicky and Chantaramongkol 2007: 1030 [distribution (reported in error, according to Oláh and Ito 2013: 30)]. —Malicky 2010a: 41 [atlas; ♂]. —Oláh and Ito 2013: 29 [♂]. —Tanida and Kuranishi 2016: 72 [as junior synonym of *O.kakida*].

—*josifovi* Kumanski, 1990: 57 [type locality: Korea, Province Kangvon, stream and small torrents of the plain near Casan vill., 1–3 km from the sea (ca. 25 km E of Vonsan); SOFM; ♂; ♀]. —Morse et al. 2001: 102 [distribution]. —Arefina et al. 2002: 102 [distribution]. —Nozaki and Tanida 2007: 246 [distribution (reported in error, according to Oláh and Ito 2013: 30)]. —Malicky and Chantaramongkol 2007: 1030 [to synonymy]. —Ivanov 2011: 195 [checklist]. —Oláh and Ito 2013: 29 [♂; claim holotype is lost]. —Vshivkova et al. 2016: 79 [distribution]. —Potikha and Vshivkova 2016: 364 [distribution]. —Tanida and Kuranishi 2016: 72 [as junior synonym of *O.kakida*]. —Park and Kong 2020: 297 [checklist].

**Distribution.** —Japan, Korea, Russia, Vietnam.

***delcourti*** (*Oxyethira*) Jacquemart, 1973: 7 [type locality: [Greece], Rhodes, Pétaloudès; IRSNB; ♂; larva]. —Botosaneanu and Malicky 1978: 340 [checklist]. —Malicky 1983b: 59 [atlas; ♂]. —Kelley 1984a: 437 [checklist]. —Sipahiler and Malicky 1987: 129 [distribution]. —Botosaneanu 1992: 91 [♂; ♀]. —Malicky 2004a: 71, 72 [atlas]. —Malicky 2005b: 547 [checklist]. —Sipahiler 2005: 397 [distribution]. —Malicky 2005a: 66 [distribution]. —Sipahiler 2007: 38 [distribution]. —Karaouzas and Malicky 2015: 14 [distribution]. —Dia 2015: 51 [distribution]. —Karaouzas and Malicky 2016: 18 [distribution].

**Distribution.** —Greece, Lebanon, Turkey.

***desadorna*** (*Oxytrichia*) Moulton & Harris, 1997: 499 [type locality: Mexico, Nuevo Leon, Municipio de Santiago, spring along road above Cola de Caballo; NMNH; ♂]. —Bueno-Soria et al. 2007: 33 [distribution].

**Distribution.** —Mexico.

***digitata*** (*Pacificotrichia*) Wells & Johanson, 2015: 73 [type locality: New Caledonia, Province Sud side stream to Rivière Blanche, 10.75 km SW Pont Pérignon, 22°10.073'S a66°39.903'E, 180 m; MNHN; ♂]. —Johanson and Wells 2019: 93 [checklist].

**Distribution.** —New Caledonia.

***diplospissa*** (unplaced) de Souza & Santos, 2017: 490 [type locality: Brazil, Alagoas, Quebrangulo, Reserva Biológica de Pedra Talhada, Rio Cafuringa abaixo da represa, 9°15'15"S, 36°25'07"W; DZRJ; ♂]. —Rocha et al. 2018: 153 [checklist].

**Distribution.** —Brazil.

***discaelata*** (*Dampfitrichia*) Kelley, 1983: 48 [type locality: Venezuela, Bolivar State, Morichal Tauca, 22 km E Rio Caura; NMNH; ♂]. —Kelley 1984a: 439 [checklist]. —Angrisano 1999: 34 [checklist]. —Paprocki et al. 2004: 11 [checklist]. —Santos et al. 2009: 36 [checklist]. —Paprocki and França 2014: 51 [checklist]. —Rocha et al. 2018: 152 [checklist].

**Distribution.** —Brazil, Venezuela.

***distinctella*** (*Holarctotrichia*) McLachlan, 1880: 521 [type locality: Finland; NHMUK; ♂]. —Morton 1893: 80 [♂]. —Morton 1904: 328 [distribution]. —Martynov 1934: 149 [♂]. —Mosely 1939b: 286 [♂]. —Kimmins 1958b: 12 [♀]. —Nybom 1960: 19 [checklist]. —Botosaneanu 1967: 293 [distribution]. —Botosaneanu and Malicky 1978: 340 [checklist]. —Malicky 1983b: 59, 60 [atlas; ♂; ♀]. —Kelley 1984a: 438 [checklist]. —Wiberg-Larsen 1985: 40 [checklist]. —Andersen and Tysse 1985: 84 [distribution]. —Andersen and Wiberg-Larsen 1987: 169 [checklist]. —Spuris 1989: 17 [checklist]. —Andersen et al. 1990: 26 [distribution]. —Andersen et al. 1990: 52 [distribution]. —Mey 1991: 270 [distribution]. —Andersen et al. 1993b: 3 [distribution; ♂; ♀]. —Czachorowski 1995: 279 [distribution]. —Gullefors 2002: 138 [checklist]. —Arefina and Armitage 2003: 17 [distribution]. —Malicky 2004a: 71, 72 [atlas]. —Malicky 2005b: 547 [checklist]. —Gullefors 2008: 64 [checklist]. —Ivanov 2011: 195 [checklist]. —Viidalepp et al. 2011: 196 [distribution]. —Salokannel et al. 2012: 202 [confirmed as distinct species]. —Barndt 2014: 106 [distribution]. —Jacquemin and Coppa. 2015: 107 [distribution]. —Potikha and Vshivkova 2016: 364 [distribution]. —Vshivkova et al. 2016: 78, 80 [distribution]. —Gullefors 2016: 155 [checklist]. —Wallace 2016: 21, 23, 67 [conservation status]. —Gullefors 2018: 108 [biology; distribution].

**Distribution.** —Denmark, Estonia, Finland, France, Germany, Norway, Poland, Russia, Sweden.

***dorsennus*** (*Pacificotrichia*) Kelley, 1989: 199 [type locality: New Caledonia, mountain stream up Boulari River; BPBM; ♂]. —Wells and Johanson 2015: 61 [♂; distribution]. —Johanson and Wells 2019: 93 [checklist].

**Distribution.** —New Caledonia.

***driesseni*** (*Trichoglene*) Wells, 2002b: 39 [type locality: [Australia], Tasmania, Lake St Clair, Site RCE young; ANIC; ♂]. —Neboiss 2002: 54 [checklist].

**Distribution.** —Australia.

***dualis*** (*Oxytrichia*) —Morton, 1905: 71 [type locality: [United States], Las Vegas, New Mexico; depository not designated; ♂]. —Banks 1907a: 50 [catalogue]. —Sibley 1926: 205 [biology]. —Betten 1934: 162 [checklist]. —Ross 1944: 139 [♀; distribution]. —Unzicker et al. 1970: 172 [distribution]. —Edwards 1973: 506 [distribution]. —Flint and Herrmann 1976: 898 [distribution]. —Etnier and Schuster 1979: 18 [distribution]. —Blickle 1979: 54, 95 [checklist; ♂]. —Kelley and Morse 1982: 257, 265 [checklist; ♀]. —Waltz and McCafferty 1983a: 11 [distribution]. —Hamilton et al. 1983: 19 [distribution]. —Kelley 1984a: 440 [checklist]. —Bowles and Mathis 1989: 240 [distribution]. —Harris et al. 1991: 245 [distribution]. —Masteller and Flint 1992: 70 [checklist]. —Mathis and Bowles 1992: 24 [distribution]. —Bowles and Mathis 1992: 32 [distribution]. —Moulton et al. 1994: 171 [distribution]. —Moulton and Stewart 1996: 126 [♂; distribution]. —Moulton and Stewart 1997: 351 [checklist]. —Harris and Huryn 2000: 80 [♂]. —Blinn and Ruiter 2005: 69 [distribution; biology]. —Blinn and Ruiter 2006: 333 [biology; distribution]. —Biondi 2010: 60 [distribution]. —Armitage et al. 2011: 14 [checklist]. —Blinn and Ruiter 2013: 291 [biology; distribution]. —Mendez et al. 2019: 119 [checklist]. —Bowles et al. 2020: 8 [distribution].

—*allosi* Blickle, 1980: 101 [type locality: [United States], California, Butte Co., Oroville, concrete fish ladders at fish hatchery; CAS; ♂; ♀]. —Kelley and Morse 1982: 258 [to synonymy].

**Distribution.** —U.S.A.

***dunbartonensis*** (*Holarctotrichia*) Kelley, 1981: 368 [type locality: [United States], South Carolina, Aiken Co., Savannah River Plant, Upper Three Runs Creek at SRP 2-1; NMNH; ♂; ♀]. —Kelley and Morse 1982: 257, 263 [checklist; ♀]. —Kelley 1984a: 438 [checklist]. —Floyd et al. 1993: 91 [phenology; distribution]. —Flint 2014: 90 [distribution].

**Distribution.** —U.S.A.

***ecornuta*** (*Oxyethira*) Morton, 1893: 79 [type locality: Finland, Teisko; MZHF; ♂]. —Morton 1904: 326 [distribution]. —Nybom 1960: 19 [checklist]. —Botosaneanu 1967: 293 [distribution]. —Tobias 1970: 227 [♂; distribution]. —Botosaneanu and Malicky 1978: 340 [checklist]. —Malicky 1983b: 60 [atlas; ♂]. —Kelley 1984a: 437 [checklist]. —Andersen and Wiberg-Larsen 1987: 169 [checklist]. —Botosaneanu and Levanidova 1988: 174 [distribution; ♂]. —Botosaneanu and Levanidova 1988: 174 [♂; distribution]. —Spuris 1989: 17 [checklist]. —Xue and Yang 1991: 20 [distribution]. —Xue et al. 1992: 353–356 [distribution]. —Monson and Holzenthal 1993: 442 [distribution]. —Yang et al. 1997b: 93 [checklist]. —Houghton et al. 2001: 505 [distribution]. —Morse et al. 2001: 102 [distribution]. —Zasypkina and Ryabukhin 2001: 45 [checklist]. —Arefina et al. 2002: 102 [distribution] —Gullefors 2002: 138 [checklist]. —Gullefors 2003: 195 [distribution]. —Houghton and Holzenthal 2003: 38 [distribution]. —Malicky 2004a: 72 [atlas]. —Yang et al. 2005: 458 [checklist]. —Chuluunbat and Morse 2007: 54 [distribution]. —Szczęsny and Godunko 2008: 15 [checklist]. —Gullefors 2008: 63 [checklist]. —Ivanov 2011: 195 [checklist]. —Salokannel et al. 2012: 202 [confirmed as distinct species]. —Oláh and Ito 2013: 33 [♂; distribution; lectotype designation]. —Yang et al. 2016: 477 [checklist]. —Zasypkina 2016: 486 [distribution]. —Chuluunbat et al. 2016: 102[distribution]. —Vshivkova et al. 2016: 79 [distribution]. —Gullefors 2016: 155 [checklist]. —Potikha and Vshivkova 2016: 364 [distribution]. —Houghton et al. 2017: 63 [checklist].

**Distribution.** —China, Finland, Mongolia, Russia, Sweden, Ukraine, U.S.A.

***efatensis*** (*Pacificotrichia*) Kelley, 1989: 201 [type locality: Vanuatu, Efate (NW), Maat, Ambryn Village, 3M.; BPBM; ♂]. —Johanson et al. 2011: 293 [♂; distribution].

**Distribution.** —Vanuatu.

***elerobi*** (unplaced) Blickle, 1961: 132 [type locality: [United States], Florida, Laurel Hill; INHS; ♂; in *Neotrichia*]. —Blickle 1979: 50, 77 [checklist; ♂]. —Kelley 1981: 370 [♂; ♀; to *Oxyethira*]. —Kelley and Morse 1982: 257, 261 [checklist; ♀]. —Harris et al. 1982b: 81 [distribution]. —Kelley 1984a: 438 [checklist]. —Kelley 1986: 777 [taxonomic placement]. —Harris et al. 1991: 246 [distribution]. —Abbott et al. 1997: 44 [distribution]. —Moulton and Stewart 1997: 351 [checklist]. —Pescador et al. 2004: 133 [checklist]. —Harris et al. 2012: 10 [checklist]. —Denson et al. 2016: 6 [distribution].

**Distribution.** —U.S.A.

***enigmatica*** (*Pacificotrichia*) Wells & Johanson, 2015: 66 [type locality: New Caledonia, Province Sud, W part of Plaine des lacs, 150 m downstream bridge at La Capture, 22°15.967'S, 166°49.493'E, 261 m; MNHN; ♂]. —Johanson and Wells 2019: 93 [checklist].

**Distribution.** —New Caledonia.

***espinada*** (*Tanytrichia*) Holzenthal & Harris, 1992: 160 [type locality: Costa Rica, Alajuela, Río Pizote, 5 km N Dos Ríos, 10.948°N, 85.291°W; NMNH; ♂]. —Blahnik et al. 2004: 5 [distribution]. —Paprocki et al. 2004: 12 [checklist]. —Santos et al. 2009: 36 [checklist]. —Paprocki and França 2014: 51 [checklist]. —de Souza and Santos 2017: 504 [distribution]. —Rocha et al. 2018: 153 [checklist].

**Distribution.** —Brazil, Costa Rica.

***espirita*** (unplaced) Johanson, Wells, Malm, & Espeland, 2011: 292 [type locality: [Vanuatu] Espiritu Santo, Central Santo, stream in small canyon crossing path to village, 5.5 km NW Nambel, 208 m, loc#21, 15°27.459'S, 167°04.022'E; NHRS; ♂].

**Distribution.** —Vanuatu.

***ezoensis*** (unplaced) Kobayashi, 1977: 5 [type locality: [Japan]. Utonai Pond, Utonai, Tomakomai-shi, Hokkaido; depository not designated; ♂; ♀]. —Kelley 1984a: 442 [checklist].

**Distribution.** —Japan.

***falcata*** (*Oxyethira*) Morton, 1893: 80 [type locality: type locality not given; depository not designated; ♂]. —Klapálek 1894: 6 [♂; distribution]. —Ris 1894: 131 [distribution]. —Morton 1896: 104 [distribution]. —Morton 1899a: 54 [distribution]. —Morton 1899b: 281 [distribution]. —Morton 1904: 327 [distribution]. —Ulmer 1929: 260 [morphological notes; comparison with *O.frici*]. —Martynov 1934: 154 [♂]. —Mosely 1939b: 285 [♂]. —Schmid 1952: 656 [distribution]. —Kimmins 1958b: 16 [♀; distribution]. —Schmid 1959b: 693 [distribution]. —Schmid 1960: 98 [distribution]. —Nybom 1960: 19 [checklist]. —Jacquemart and Coineau 1962: 16 [distribution; ♂; larva]. —Botosaneanu 1967: 293 [distribution]. —Botosaneanu and Gasith 1971: 98 [distribution]. —Malicky 1974: 122 [checklist]. —Botosaneanu and Malicky 1978: 340 [checklist]. —Kumanski 1979: 6 [♂; distribution]. —Malicky 1980a: 16 [checklist]. —Moretti and Cianficconi 1981: 201 [checklist]. —Malicky 1983b: 58, 60 [atlas; ♂; ♀]. —Kelley 1984a: 437 [checklist]. —Kelley 1984b: 186 [♂ ♀]. —Kumanski and Malicky 1984: 199 [distribution]. —Kumanski 1985: 144 [♂]. —Wiberg-Larsen 1985: 40 [checklist]. —Moubayed and Botosaneanu 1985: 63 [distribution]. —Andersen and Wiberg-Larsen 1987: 169 [checklist]. —Malicky and Lounaci 1987: 15, 17 [checklist]. —Sipahiler and Malicky 1987: 107, 122, 129 [distribution]. —Spuris 1989: 17 [checklist]. —Gullefors 1989: 119 [distribution]. —Krušnik 1991: 13 [distribution]. —Botosaneanu 1992: 93 [♂; ♀]. —Nógrádi and Uherkovich 1994: 31 [distribution]. —Kahnert 1995: 124 [distribution]. —Maier et al. 1995: 148 [distribution]. —Chvojka 1996: 131 [distribution]. —Weinzierl 1997: 80 [distribution]. —Uherkovich and Nógrádi 1997: 461 [distribution]. —Hohmann 1998: 73 [distribution]. —Uherkovich and Nógrádi 1998: 52 [distribution]. —Nógrádi and Uherkovich 1998: 338 [distribution]. —Graf et al. 1998: 206 [distribution]. —Hohmann 1999: 35, 36 [checklist; distribution]. —Malicky 1999f: 32 [distribution]. —Uherkovich and Nógrádi 1999: 420 [distribution]. —Cianficconi et al. 1999b: 278 [distribution]. —Uherkovich and Nógrádi 2001: 94 [distribution]. —Nógrádi and Uherkovich 2001: 297 [checklist]. —Valle 2001: 65 [distribution]. —Cianficconi et al. 2002: 146 [distribution]. —Nógrádi and Uherkovich 2002: 130 [distribution]. —Ujvárosi 2002: 384 [distribution]. —Mirmoayedi and Malicky 2002: 164 [distribution]. —Gullefors 2002: 138 [checklist]. —Sipahiler 2003b: 33 [distribution]. —Malicky 2004a: 70, 72 [atlas]. —Cianficconi et al. 2004a: 256, 258 [distribution; biology]. —Malicky 2005b: 547 [checklist]. —Sipahiler 2005: 397 [distribution]. —Huang et al. 2005: 469 [distribution]. —Armitage et al. 2005: 27 [checklist]. —Malicky 2005a: 67 [distribution]. —Mey 2005a: 280 [distribution]. —Lubini-Ferlin and Vicentini 2005: 68 [checklist]. —Chvojka 2006: 253 [distribution]. —Wiggers et al. 2006: 54 [distribution]. —Robert 2007: 82 [checklist]. —Cianficconi et al. 2007b: 569, 575 [distribution]. —Sipahiler 2007: 38 [distribution]. —Chvojka and Komzák 2008: 13 [distribution]. —Ujvárosi et al. 2008: 113 [checklist]. —Gullefors 2008: 64 [checklist]. —Szczęsny and Godunko 2008: 15 [checklist]. —Robinson 2009: 119 [distribution]. —Corallini and Cianficconi 2011: 628 [checklist]. —González and Menéndez 2011: 119 [distribution]. —Cianficconi et al. 2011: 47 [distribution]. —Ivanov 2011: 195 [checklist]. —Viidalepp et al. 2011: 195, 196 [distribution]. —Salokannel et al. 2012: 204 [confirmed as distinct species]. —Sipahiler 2012a: 7 [distribution]. —Andersen and Hagenlund 2012: 136 [distribution]. —Corallini et al. 2013b: 26 [distribution]. —Tempelman and Sanabria 2013a: 20 [distribution; larva; ♀]. —Malicky 2014b: 27 [teratological structures]. —Lock 2014: 199 [distribution]. —O’Connor 2015: 28, 98 [distribution]. —Martín et al. 2015: 74 [distribution]. —Karaouzas and Malicky 2015: 14 [distribution]. —Martínez et al. 2016: 52 [distribution]. —Smirnova et al. 2016: 401 [distribution]. —Dia 2015: 51 [distribution]. —Yang et al. 2016: 477 [checklist]. —Gullefors 2016: 155 [checklist]. —Sipahiler 2016: 15 [checklist]. —Martín et al. 2016: 262 [distribution]. —Ruiz-García et al. 2016: 4 [distribution]. —Melnitsky et al. 2017: 6 [distribution]. —Sipahiler 2017b: 13 [distribution]. —Graf et al. 2017: 48 [distribution]. —Valle and Lodovici 2018: 147 [distribution]. —Komzák and Kroča 2018: 169 [distribution]. —O’Connor and O’Connor 2018: 83 [distribution]. —Sipahiler 2018: 41 [distribution]. —Edmonds-Brown 2020: 91 [checklist]. —Smirnova et al. 2020: 68 [distribution].

—*assia* Botosaneanu & Moubayed, 1985 in Moubayed and Botosaneanu 1985: 64 [type locality: [Lebanon], Liban, Labwé, sources karstiques dans le bassin supérieur de l’Oronte, 1000 m; ZMUA; ♂; ♀]. —Botosaneanu 1992: 95 [♂; ♀]. —Malicky 2005b: 547 [to synonymy].

—*bidentata* Nybom, 1948: 9 [type locality: [Portugal], Azores; MZHF; ♂]. —Nybom 1954: 1 [replacement name *O.dentata*; preoccupied by *O.bidentata* Mosely, 1934a: 155].

—*boreella* Svensson & Tjeder, 1975: 131 [type locality: Sweden, Prov. Västerbotten, Finnmyran, 7 km W of Hällnäs, 64°19'N, 19°29'E; MZLU; ♂; ♀]. —Botosaneanu and Malicky 1978: 340 [checklist]. —Malicky 1983b: 58, 60 [atlas; ♂; ♀]. —Kelley 1984a: 437 [checklist]. —Kelley 1984b: 186 [♂, ♀]. —Andersen and Wiberg-Larsen 1987: 169 [checklist]. —Gullefors 2002: 138 [checklist]. —Malicky 2004a: 70, 72 [atlas]. —Malicky 2005b: 547 [checklist]. —Malicky 2007b: 51 [to synonymy]. —Gullefors 2008: 63 [checklist]. —Salokannel et al. 2012: 202 [confirmed as synonym].

—*dentata* Nybom, 1954: [type locality: [Portugal, Azores]; MZHF; ♂; replacement name for *O.bidentata*Nybom 1948: 9]. —Nybom 1965: 90 [distribution]. —Botosaneanu 1967: 293 [distribution]. —Botosaneanu and Malicky 1978: 340 [checklist]. —Kelley 1984b: 186 [to synonymy].

—*rhodani* Schmid, 1947: 531 [type locality: [Switzerland], Bois de Finges; NHMUK; ♂]. —Schmid 1960: 99 [to synonymy]. —Kelley 1984a: 437 [checklist].

**Distribution.** —Algeria, Austria, Belgium, Bulgaria, China, Czech Republic, England, Estonia, Denmark, Finland, France, Germany, Greece, Hungary, Iran, Ireland, Israel, Italy, Jordan, Kazakhstan, Lebanon, Morocco, Netherlands, Norway, Pakistan, Portugal, Romania, Russia, Scotland, Slovenia, Spain, Sweden, Switzerland, Turkey, Ukraine, Vietnam.

***fijiensis*** (*Pacificotrichia*) Kelley, 1989: 201 [type locality: Fiji, Levu, Nandarivatu; BPBM; ♂].

**Distribution.** —Fiji.

***flagellata*** (*Argyrobothrus*) Jacquemart, 1963c: 6 [type locality: [Mauritius], Ile de la Réunion, Hell-Bourg; IRSNB; ♂]. —Marlier and Marlier 1982: 24 [distribution; larva]. —Kelley 1984a: 438 [checklist].

**Distribution.** —Mauritius, Rèunion.

***flavicornis*** (*Oxyethira*) (Pictet, 1834): 225 [type locality: [Switzerland]; no holotype designated; in *Hydroptila*]. —Hagen 1864b: 234 [comments on larvae and case]. —Tobias 1970: 228 [♂; distribution]. —Solem 1970a: 2 [distribution]. —Andersen 1974: 26 [distribution]. —Botosaneanu and Malicky 1978: 340 [checklist]. —Andersen 1978: 149 [distribution]. —Moretti and Cianficconi 1981: 201 [checklist]. —Malicky 1983b: 58, 60 [atlas; ♂; ♀]. —Kelley 1984a: 437 [checklist]. —Kumanski 1985: 144 [♂]. —Nógrádi 1985: 131 [distribution; ♂]. —Andersen and Tysse 1985: 84 [distribution]. —Wiberg-Larsen 1985: 40 [checklist]. —Andersen and Wiberg-Larsen 1987: 169 [checklist]. —Spuris 1989: 17 [checklist]. —Waringer 1989: 390 [distribution; ecology]. —Usseglio-Polatera and Bournaud 1989: 254 [distribution]. —Andersen et al. 1990: 26 [distribution]. —Andersen et al. 1990: 52 [distribution]. —Andersen et al. 1993b: 3 [distribution]. —Andersen et al. 1993a: 51 [distribution]. —Nógrádi and Uherkovich 1994: 31 [distribution]. —Uherkovich and Nógrádi 1997: 461 [distribution]. —Uherkovich and Nógrádi 1998: 52 [distribution]. —Nógrádi and Uherkovich 1998: 338 [distribution]. —Uherkovich and Nógrádi 1999: 420 [distribution]. —Malicky 1999c: 96 [distribution]. —Wiberg-Larsen and Karsholt 1999: 126 [distribution]. —Cianficconi et al. 1999b: 278 [distribution]. —Morse et al. 2001: 102 [distribution]. —Uherkovich and Nógrádi 2001: 94 [distribution]. —Nógrádi and Uherkovich 2001: 297 [checklist]. —Gullefors 2002: 138 [checklist]. —Nógrádi and Uherkovich 2002: 130 [distribution]. —Cibaitė 2003a: 10 [checklist]. —Urbanič 2004: 51 [distribution]. —Malicky 2004a: 70, 72 [atlas]. —Graf and Hutter 2004: 147 [distribution]. —Berlin 2005: 130 [distribution]. —Gullefors 2005a: 118 [distribution]. —Gullefors 2005b: 138 [distribution]. —Mey 2005b: 119 [distribution]. —Hohmann 2005: 106 [checklist]. —Graf et al. 2005: 55 [distribution]. —Lubini-Ferlin and Vicentini 2005: 68 [checklist]. —Gullefors 2006: 136, 137 [distribution]. —Morse et al. 2006: 321 [distribution]. —Waringer and Graf 2006: 356 [distribution]. —Chvojka and Komzák 2006: 358 [distribution]. —Schiess-Bühler and Rezbanyai-Reser 2006: 73 [distribution]. —Berlin and Thiele 2007: 50 [checklist]. —Robert 2007: 82 [checklist]. —Gullefors and Johanson 2007: 64 [distribution]. —Cianficconi et al. 2007b: 575 [distribution]. —Ivanov and Melnitsky 2007: 32 [distribution]. —Szczęsny and Godunko 2008: 15 [checklist]. —Waringer and Graf 2008: 142 [distribution]. —Chvojka and Komzák 2008: 13 [distribution]. —Ujvárosi et al. 2008: 113 [checklist]. —Schrankel et al. 2008: 90 [checklist]. —Višinskienė 2009: 28 [checklist]. —Corallini and Cianficconi 2011: 628 [checklist]. —Ivanov 2011: 195 [checklist]. —Viidalepp et al. 2011: 196 [distribution]. —Andersen and Hagenlund 2012: 136 [distribution]. —Salokannel et al. 2012: 202 [confirmed as distinct species]. —Oláh and Ito 2013: 34 [♂]. —O’Connor 2013: 65 [distribution]. —Zuyderduyn and Tempelman 2013: 25 [distribution]. —Tempelman et al. 2013: 288 [distribution]. —Tempelman and Sanabria 2013b: 144 [distribution]. —O’Connor and O’Connor 2014: 273 [distribution]. —Mey 2014: 187 [distribution]. —O’Connor and Bond 2014: 24 [distribution]. —Chalkley 2014: 13 [distribution]. —Hohmann et al. 2014: 85 [distribution]. —O’Connor 2015: 28, 99 [distribution; states that *O.flavicornis* has been misidentified as *Orthotrichiacostalis* in earlier Irish literature]. —Smirnova et al. 2016: 401 [distribution]. —Küttner et al. 2016: 179 [distribution]. —Pan’kov and Krasheninnikov 2016: 333 [distribution]. —Buczyńska et al. 2016: 161 [distribution]. —Gullefors 2016: 155 [checklist]. —Chuluunbat et al. 2016: 102 [distribution]. —Melnitsky and Ivanov 2017: 19 [distribution]. —O’Connor and O’Connor 2018: 83 [distribution]. —O’Connor et al. 2018: 23 [distribution]. —Gullefors 2018: 108 [biology; distribution]. —Lock and van Butsel 2018: 1 [distribution]. —O’Connor 2020: 141 [distribution]. —Hansen and Gíslasen 2020: 132 [checklist].

—*costalis*Eaton 1873: 144 *nec* Curtis, 1834: 218 [type locality: [England], ponds in Woburn and Battlesden Parks, Bedfordshire; no holotype designated]. —Morton 1887: 201 [notes on case]. —Morton 1899a: 54 [distribution]. —Klapálek 1890: 204 [larva]. —Klapálek 1893: 138 [larva]. —Ris 1903: 17 [distribution]. —Siltala 1908: 16 [distribution]. —Spandl 1923: 357 [larva]. —Ulmer 1925: 432 [distribution]. —Martynov 1934: 149 [♂]. —Henriksen 1937: 3 [distribution]. —Mosely 1939b: 282 [♂]. Kimmins 1943: 155 [distribution]. —Nielsen 1948: 76 [larva]. —Nybom 1960: 19 [checklist]. —Spuris 1962: 57, 61 [distribution]. —Ulmer 1963: 266 [♂; distribution]. —Neboiss 1963: 594 [name to be replaced by *flavicornis*]. —Spuris 1964: 13 [distribution]. —Botosaneanu 1967: 293 [as synonym of *O.flavicornis*].

**Distribution.** —Austria, Belarus, Czech Republic, Denmark, Egypt, England, Estonia, Finland, France, Germany, Hungary, Ireland, Italy, Kazakhstan, Latvia, Luxembourg, Mongolia, Netherlands, Norway, Poland, Slovenia, Romania, Russia, Scotland, Sweden, Switzerland, Ukraine.

***florida*** (*Dampfitrichia*) Denning, 1947a: 12 [type locality: [United States], Florida, Miami; ESUW; ♂; ♀]. —Botosaneanu 1979: 40, 51 [♂; ♀; distribution]. —Blickle 1979: 54, 93 [checklist; ♂]. —Kelley and Morse 1982: 257, 266 [checklist; ♀]. —Flint 1996a: 16 [checklist]. —Botosaneanu 2002b: 87 [checklist]. —Kelley and Morse 1982: 266 [♀]. —Pescador et al. 2004: 134 [checklist]. —Naranjo López and González Laz 2005: 149 [checklist]. —Harris et al. 2012: 10 [checklist].

**Distribution.** —Cuba, U.S.A.

***forcipata*** (*Holarcotrichia*) Mosely, 1934a: 153 [type locality: U.S.A., Michigan; NHMUK; ♂]. —Morse and Blickle 1953: 72 [checklist]. —Etnier 1968: 191 [distribution]. —Roy and Harper 1975: 1082 [distribution]. —Roy and Harper 1979: 152 [checklist]. —Etnier and Schuster 1979: 18 [distribution]. —Blickle 1979: 54, 95 [checklist; ♂]. —Parker and Voshell 1981: 4 [checklist]. —Swegman et al. 1981: 139 [distribution]. —Roy and Harper 1981: 105 [distribution]. —Kelley and Morse 1982: 257, 267 [checklist; ♀]. —Huryn and Foote 1983: 791 [distribution]. —Harris et al. 1984: 109 [distribution]. —Kelley 1984a: 438 [checklist]. —Bowles and Mathis 1989: 240 [distribution]. —Floyd and Schuster 1990: 130, 132 [distribution]. —Harris et al. 1991: 247 [distribution]. —Frazer et al. 1991: 20 [distribution]. —Masteller and Flint 1992: 70 [checklist]. —Mathis and Bowles 1992: 24 [distribution]. —Monson and Holzenthal 1993: 442 [checklist]. —Masteller 1993: 134 [distribution]. —Moulton and Stewart 1996: 127 [♂; distribution]. —Huryn and Harris 2000: 193 [distribution]. —Houghton et al. 2001: 505 [distribution]. —Zuellig et al. 2006: 43 [distribution]. —Houghton et al. 2011b: 6 [phenology habitat]. —Armitage et al. 2011: 14 [checklist]. —Myers et al. 2011: 108 [distribution]. —DeWalt et al. 2016: 57 [distribution]. —Houghton 2016: 46 [biology]. —Hunt 2017: 108 [distribution]. —Houghton et al. 2017: 63 [checklist]. —Bowles et al. 2020: 8 [distribution].

**Distribution.** —Canada, U.S.A.

***frici*** (*Oxyethira*) Klapálek, 1891: 182 [type locality: [Czech Republic]; depository not designated; ♂]. —Morton 1893: 81 [♂]. —Ris 1894: 131 [distribution]. —Morton 1904: 327 [distribution]. —Martynov 1924: 57 [♂]. —Ulmer 1929: 260 [morphological notes; comparison with *O.falcata*]. —Martynov 1934: 154 [♂]. —Mosely 1939b: 285 [♂]. —Kimmins 1943: 155 [distribution]. —Schmid 1952: 656 [distribution]. —Kimmins 1958b: 15 [♀; distribution]. —Nybom 1960: 19 [checklist]. —Botosaneanu 1967: 293 [distribution]. —Solem 1970a: 2 [distribution]. —Tobias 1970: 226 [♂; distribution]. —Mogensen 1971: 13 [♂; distribution]. —Fahy 1972: 202 [distribution]. —Andersen 1974: 26 [distribution]. —Botosaneanu and Malicky 1978: 340 [checklist]. —Moretti and Cianficconi 1981: 201 [checklist]. —Malicky 1983b: 58, 60 [atlas; ♂; ♀]. —Kelley 1984a: 437 [checklist]. —Kumanski 1985: 144 [♂]. —Wiberg-Larsen 1985: 40 [checklist]. —Andersen and Tysse 1985: 84 [distribution]. —González et al. 1986: 113 [distribution]. —Andersen and Wiberg-Larsen 1987: 169 [checklist]. —Spuris 1989: 17 [checklist]. —Andersen et al. 1990: 26 [distribution]. —Andersen et al. 1990: 52 [distribution]. —Andersen et al. 1993b: 3 [distribution]. —Andersen et al. 1993a: 51 [distribution]. —Andersen and Klausen 1994: 14 [distribution]. —Bagge 1995: 94 [distribution; biology]. —Czachorowski and Prishchepchik 1998: 11 [distribution]. —Gullefors 2002: 138 [checklist]. —Gullefors 2003: 194 [distribution]. —Malicky 2004a: 70, 72 [atlas]. —Malicky 2005b: 547 [checklist]. —Gullefors 2005b: 138 [distribution]. —Komzák and Chvojka 2005: 66 [distribution]. —Robert 2007: 82 [checklist]. —Gullefors 2008: 64 [checklist]. —Chvojka and Komzák 2008: 13 [distribution]. —Hohmann 2010: 40 [distribution]. —González and Menéndez 2011: 119 [distribution]. —Ivanov 2011: 195 [checklist]. —Andersen and Hagenlund 2012: 136 [distribution]. —Salokannel et al. 2012: 202 [confirmed as distinct species]. —O’Connor 2013: 65 [distribution]. —Martínez et al. 2015: 40 [distribution]. —O’Connor 2015: 28, 102 [distribution]. —Gullefors 2016: 155 [checklist]. —Wallace 2016: 15, 19, 24 [conservation status].

**Distribution.** —Belarus, Czech Republic, Denmark, England, Finland, Germany, Ireland, Norway, Portugal, Russia, Scotland, Spain, Sweden.

***garifosa*** (*Dampfitrichia*) Moulton & Harris, 1997: 496 [type locality: Mexico, Tamaulipas, Municipio de Ciudad Victoria, Arroyo los Troncones, Ejido La Libertad, ca. 10 km NW Victoria; NMNH; ♂].

**Distribution.** —Mexico.

***geminata*** (*Mesotrichia*) Flint & Sykora, 2004: 41 [type locality: Dominica Republic, La Vega Province, 11.5 km S of Constanza (1 km N El Convento), 18°51.7'N, 70°41.0'W, 1410 m; NMNH; ♂; ♀]. —Pérez-Gelabert 2008: 301 [checklist].

**Distribution.** —Dominican Republic.

***glasa*** (*Argyrobothrus*) (Ross, 1941a): 70 [type locality: United States, Oklahoma, Honey Creek, Turner Falls State Park; INHS; ♂; in *Loxotrichia*]. —Denning 1947a: 14 [distribution]. —Botosaneanu 1979: 40, 50 [♂; distribution]. —Blickle 1979: 54, 91 [checklist; ♂]. —Kelley and Morse 1982: 257, 264 [checklist; ♀]. —Harris et al. 1982a: 511 [distribution]. —Harris et al. 1982b: 81 [distribution]. —Kelley 1984a: 438 [checklist]. —Holzenthal 1988: 63 [distribution]. —Harris et al. 1991: 248 [distribution]. —Frazer et al. 1991: 20 [distribution]. —Holzenthal and Harris 1992: 173 [distribution]. —Bowles and Mathis 1992: 32 [distribution]. —Floyd et al. 1993: 91 [phenology; distribution]. —Flint 1996a: 16 [checklist]. —Moulton and Stewart 1996: 127 [♂; distribution]. —Abbott et al. 1997: 44 [distribution]. —Moulton and Stewart 1997: 351 [checklist]. —Maes 1999: 1194 [checklist]. —Botosaneanu 2002b: 87 [checklist]. —Pescador et al. 2004: 134 [checklist]. —Naranjo López and González Lazo 2005: 149 [checklist]. —Chamorro-Lacayo et al. 2007: 43 [checklist]. —Flint et al. 1994: 4 [distribution]. —Etnier 2010: 486 [distribution]. —Harris et al. 2012: 10 [checklist]. —Armitage et al. 2015b: 5 [distribution]. —Armitage et al. 2015a: 7 [checklist]. —Denson et al. 2016: 6 [distribution]. —Armitage and Harris 2018b: 98 [checklist].

**Distribution.** —Costa Rica, Cuba, Nicaragua, Panama, U.S.A.

***gracilianoi*** (*Loxotrichia*) de Souza & Santos, 2017: 495 [type locality: Brazil, Alagoas, Quebrangulo, Reserva Biológica de Pedra Talhada, rio Caranguejo acima do alojamento, 9°15'26"S, 36°25'07"W; DZRJ; ♂]. —Rocha et al. 2018: 152 [checklist].

**Distribution.** —Brazil.

***grisea*** (*Oxyethira*) Betten, 1934: 162 [type locality: [United States], New York; depository not designated; ♂]. —Ross 1944: 138 [♂; distribution]. —Morse and Blickle 1953: 72 [checklist]. —Blickle 1979: 54, 97 [checklist; ♂]. —Roy and Harper 1979: 152 [checklist]. —Etnier and Schuster 1979: 18 [distribution]. —Roy and Harper 1981: 105 [distribution]. —Kelley and Morse 1982: 257, 261 [checklist; ♀]. —Waltz and McCafferty 1983a: 11 [distribution]. —Lake 1984: 220 [distribution]. —Kelley 1984a: 437 [checklist]. —Harper 1989: 541 [distribution]. —Morse et al. 1989: 23 [distribution]. —Usis and Foote 1989: 84 [distribution]. —Harris et al. 1991: 249 [distribution]. —Frazer et al. 1991: 20 [distribution]. —Masteller and Flint 1992: 70 [checklist]. —Floyd et al. 1993: 91 [phenology; distribution]. —Huryn and Harris 2000: 193 [distribution]. —Myers et al. 2011: 108 [distribution]. —Flint 2011: 104 [distribution]. —Armitage et al. 2011: 14 [checklist]. —Harris et al. 2012: 10 [checklist]. —Houghton et al. 2017: 63 [checklist].

**Distribution.** —Canada, U.S.A.

***guariba*** (*Dactylotrichia*) de Souza & Santos, 2017: 490 [type locality: Brazil, Paraíba, Mamanguape, Reserva Biológica Guaribas, rio Barro Branco, casa da cabeça de boi, 6°43'7"S, 35°10'55"W; DZRJ; ♂]. —Rocha et al. 2018: 152 [checklist].

**Distribution.** —Brazil.

***harpagella*** (*Oxyethira*) Kimmins, 1951: 205 [type locality: [India], Assam, Shillong; NHMUK; ♂]. —Kelley 1984a: 437 [checklist].

**Distribution.** —India.

***harpeodes*** (*Oxyethira*) Yang & Kelley in Yang et al. 1997: 101 [type locality: [China], Fujian Province, Jiouquxi, 230 m; NAUJ; ♂]. —Yang et al. 2005: 458 [checklist]. —Yang et al. 2016: 477 [checklist].

**Distribution.** —China.

***hartigi*** (*Oxyethira*) Moretti, 1981: 169 [type locality: [Italy], Sardegna, Sorgente Monte, 450 m, Sassari; Collection Moretti; ♂]. —Moretti and Cianficconi 1981: 201 [checklist]. —Malicky 1983b: 58 [atlas; ♂]. —Kelley 1984a: 437 [checklist]. —Malicky 2002: 4 [distribution]. —Malicky 2004a: 71 [atlas]. —Malicky 2005b: 547 [checklist]. —Cianficconi et al. 2007a: 67 [proposed as Italian endemic].

**Distribution.** —France, Italy.

***hena*** (*Oxyethira*) Oláh & Ito, 2013: 39 [type locality: China, Henan Province, Lin county, Qi river, N36.06° E113.81°; NAUJ; ♂]. —Xue and Yang 1991: 21 [♂; distribution; misidentified as *O.ecornuta*, according to Oláh and Ito 2013: 39].

**Distribution.** —China.

***hilosa*** (*Tanytrichia*) Holzenthal & Harris, 1992: 163 [type locality: Costa Rica, Alajuela, Río Pizote, 5 km (air) S Brasilia, 10.972°N, 85.345°W; NMNH; ♂]. —Bueno-Soria 1999: 117 [distribution]. —Chamorro-Lacayo et al. 2007: 43 [distribution]. —Armitage et al. 2015a: 7 [checklist]. —Armitage and Harris 2018b: 98 [checklist].

**Distribution.** —Costa Rica, Mexico, Nicaragua, Panama.

***hiroshima*** (*Oxyethira*) Oláh & Ito, 2013: 35 [type locality: Japan, Honshu, Hiroshima, Hatsukaichi-shi, Yoshiwa, Hosomidani, N34°33'01" E132°06'44", 820 m; CMB-ZI; ♂]. —Tanida and Kuranishi 2016: 72 [checklist]. —Ito and Oláh 2017: 10 [♂; ♀; distribution].

**Distribution.** —Japan.

***houailou*** (*Trichoglene*) Wells & Johanson, 2015: 52 [type locality: New Caledonia, Province Nord, small fall ~ 10 km SW Houaïlou, on Bourail road; MNHN; ♂]. —Johanson and Wells 2019: 93 [checklist].

**Distribution.** —New Caledonia.

***hozosa*** (unplaced) Harris & Davenport, 1999: 35 [type locality: Peru, Loreto, Rio Yanamono just below Explorama Lodge; NMNH; ♂].

**Distribution.** —Peru.

***hyalina*** (unplaced) Müller, 1879a: 143 [type locality: Brazil, Santa Catarina; no type nor type depository designated; larval case; in *Lagenopsyche*]. —Ulmer 1905: 74 [to *Oxyethira*]. —Ulmer 1957: 172 [bibliography]. —Kelley 1984a: 442 [checklist]. —Angrisano 1999: 35 [checklist]. —Paprocki et al. 2004: 12 [checklist]. —Santos et al. 2009: 36 [checklist]. —Paprocki and França 2014: 52 [checklist]. —Rocha et al. 2018: 152 [checklist].

**Distribution.** —Brazil.

***iannuzzae*** (*Dactylotrichia*) de Souza & Santos, 2017: 485 [type locality: Brazil, Alagoas, Quebrangulo, Reserva Biológica de Pedra Talhada, Rio Caranguejo, 2 km acima do alojamento, 9·15'03"S, 36°25'56"W, 814 m; DZRJ; ♂]. —Rocha et al. 2018: 152 [checklist].

**Distribution.** —Brazil.

***iglesiasi*** (*Oxyethira*) González & Terra, 1982: 299 [type locality: Portugal, Paúl, Ribeira do Paúl, 455 m; USCM; ♂]. —Kelley 1984a: 438 [checklist]. —Kelley 1986: 777 [taxonomic placement]. —Malicky 2004a: 70 [atlas]. —Malicky 2005b: 547 [checklist]. —González and Menéndez 2011: 119 [distribution].

**Distribution.** —Portugal.

***ikal*** (*Dampfitrichia*) Wells & Huisman, 1992: 107 [type locality: West Malaysia, Bukit Rengit; NTM; ♂]. —Malicky 2010a: 42 [atlas; ♂].

**Distribution.** —Malaysia.

***inaequispina*** (*Oxytrichia*) Flint, 1990: 118 [type locality: Chile, Prov. El Loa, brook of Toconao; IRSNB; ♂]. —Angrisano 1999: 34 [checklist].

**Distribution.** —Chile.

***incana*** (*Dampfitrichia*) Ulmer, 1906: 102 [type locality: [Indonesia]; ZMUH; ♂]. —Ulmer 1951: 70 [distribution; ♂]. —Kelley 1984a: 439 [checklist]. —Neboiss 1986: 82 [atlas; ♂]. —Wells 1990b: 392 [♂, ♀; distribution]. —Wells 1991: 491 [distribution]. —Wells and Malicky 1997: 186 [distribution]. —Malicky and Chantaramongkol 2007: 1029 [distribution]. —Malicky 2007a: 177 [checklist]. —Oláh and Johanso 2010a: 34 [distribution]. —Malicky, 2010a: 42 [atlas; ♂]. —Malicky et al. 20146 [distribution]. —Wells and Johanson 2015: 75 [♂; distribution]. —Wityi et al. 2015: 47 [checklist]. —Melnitsky et al. 2019: 539 [distribution]. —Johanson and Wells 2019: 93 [checklist]. —Wells et al. 2019: 33 [detection frequency].

—*australiensis* (Wells, 1981): 112 [type locality: [Australia] Queensland, Mt Spec, Little Crystal Creek; ANIC; ♂; in *Gnathotrichia*]. —Kelley 1984a: 439 [to synonymy].

—*excisa* (Kimmins, 1951): 209 [type locality: [Myanmar], Lower Burma, Thaton; NHMUK; ♂; in *Stenoxyethira*]. —Kelley 1984a: 439 [to synonymy].

—*galekoluma* Schmid, 1958b: 68 [type locality: [Sri Lanka], Ceylan, Maturata (C. P., 2400 ft) 1-III, Belihul Oya, assez grande rivière torrentueuse, lit très rocheux et encaissé, dans les rizières; depository not designated; ♂]. —Kelley 1984a: 439 [checklist]. —Malicky and Chantaramongkol 2007: 1029 [to synonymy].

—*isabellina* (Ulmer, 1951): 60 [type locality: [India], Zentral-Sumatra, Pangkalang; ZMUH; ♂; in *Gnathotrichia*]. —Marshall 1979b: 207 [as synonym of *Stenoxyethiraexcisa*]. —Kelley 1984a: 439 [to synonymy].

**Distribution.** —Australia, India, Indonesia, Malaysia, Myanmar, New Caledonia, Papua New Guinea, Sri Lanka, Thailand, Vietnam.

***incurvata*** (*Trichoglene*) Wells & Johanson, 2015: 47 [type locality: New Caledonia, Province Nord, Mt Panié, 20.57306°S, 164.77139°E, 902 m; MNHN; ♂]. —Johanson and Wells 2019: 93 [checklist].

**Distribution.** —New Caledonia.

***indorsennus*** (*Pacificotrichia*) Kelley, 1989: 199 [type locality: New Caledonia, mountain stream up Boulari River; BPBM; ♂]. —Wells and Johanson 2015: 61 [♂; distribution]. —Johanson and Wells 2019: 93 [checklist].

—*tompa* Oláh & Johanson, 2010a: 34 [type locality: New Caledonia, Province Sud, W slope Mt. Ningua, Kwé Néco Stream, 3.9 km W summit of Mt. Ningua, on Bouloparis-Thio Road, about 50 m upstream road, 21°44.359'S, 166°06.009'E, 117 m; MNHN; ♂]. —Wells and Johanson 2015: 61 [to synonymy].

**Distribution.** —New Caledonia.

***insularis*** (*Trichoglene*) Kelley, 1989: 196 [type locality: New Caledonia, mountain stream up Boulari River; BPBM; ♂]. —Wells and Johanson 2015: 54 [♂; distribution]. —Johanson and Wells 2019: 93 [checklist].

**Distribution.** —New Caledonia.

***itascae*** (*Holarctotrichia*) Monson & Holzenthal, 1993: 438 [type locality: [United States], Minnesota, Clearwater County, Itasca State Park, Nicollet Creek at Wilderness Drive, 47.194°N, 92.230°W, 1500 ft.; UMSP; ♂]. —Houghton et al. 2001: 505 [distribution]. —Houghton and Holzenthal 2003: 39 [distribution]. —Houghton et al. 2017: 63 [checklist].

**Distribution.** —U.S.A.

***jamaicensis*** (*Mesotrichia*) Flint, 1968b: 44 [type locality: Jamaica, St. Andrew, Hope River near Newcastle at mile post 16.5; NMNH; ♂; ♀]. —Flint 1968a: 82 [checklist]. —Kelley 1984a: 440 [checklist]. —Botosaneanu 2002b: 89 [checklist].

**Distribution.** —Jamaica.

***janella*** (*Loxotrichia*) Denning, 1948: 397 [type locality: United States, Florida, Winter Park; CAS; ♂]. —Flint 1968b: 42 [♂; ♀; distribution]. —Flint 1968a: 52 [♂; ♀; distribution]. —Bueno-Soria and Flint 1978: 205 [distribution]. —Botosaneanu 1979: 50 [distribution]. —Blickle 1979: 54, 93 [checklist; ♂]. —Kelley and Morse 1982: 257, 261 [checklist; ♀]. —Harris et al. 1982a: 511 [distribution]. —Harris et al. 1982b: 81 [distribution]. —Malicky 1983c: 264 [distribution]. —Harris et al. 1984: 109 [distribution]. —Kelley 1984a: 442 [checklist]. —Kumanski 1987: 27 [distribution]. —Holzenthal 1988: 63 [distribution]. —Botosaneanu 1989: 101 [distribution]. —Botosaneanu 1990a: 47 [distribution]. —Botosaneanu 1991: 132 [distribution]. —Harris et al. 1991: 250 [distribution]. —Holzenthal and Harris 1992: 173 [distribution]. —Aguila 1992: 539 [distribution]. —Flint and Sykora 1993: 57 [distribution]. —Floyd et al. 1993: 91 [phenology; distribution]. —Manuel and Bohart 1993: 139 [association between Trichoptera and Strepsiptera]. —Botosaneanu 1994a: 42 [distribution]. —Botosaneanu 1995a: 32 [distribution]. —Flint 1996b: 98 [distribution]. —Flint 1996a: 16 [checklist]. —Moulton and Stewart 1996: 127 [♂; distribution]. —Abbott et al. 1997: 44 [distribution]. —Moulton and Stewart 1997: 351 [checklist]. —Floyd et al. 1997: 136 [distribution]. USA —Botosaneanu and Hyslop 1998: 16 [distribution]. —Flint and Pérez-Gelabert 1999: 41 [checklist]. —Botosaneanu 2000: 256 [distribution]. —Botosaneanu 2002b: 88 [checklist]. —Pescador et al. 2004: 133 [checklist]. —Flint and Sykora 2004: 43 [distribution]. —Botosaneanu and Thomas 2005: 55 [checklist]. —Naranjo López and González Lazo 2005: 149 [checklist]. —Zeullig et al. 2006: 43 [distribution]. —Bowles et al. 2007: 22 [distribution biology]. —Pérez-Gelabert 2008: 301 [checklist]. —Harris et al. 2012: 10 [checklist]. —Denson et al. 2016: 6 [distribution]. —Armitage et al. 2016: 11 [distribution]. —Armitage and Harris 2018b: 98 [checklist]. —Armitage and Harris 2018c: 283 [distribution]. —Harris and Armitage 2019: 5 [distribution]. —Barba-Álvarez et al. 2019: 86 [distribution].

—*neglecta* (*Loxotrichia*) Flint, 1964: 57 [type locality: Puerto Rico, Maricao, fish hatchery; NMNH; ♂; ♀]. —Flint 1968b: 42 [to synonymy].

**Distribution.** —Barbados [?], Costa Rica, Cuba, Dominica, Dominican Republic, Grenada, Guadeloupe, Haiti, Jamaica, Martinique [?], Mexico, Panama, Puerto Rico, St Lucia, St. Vincent, U.S.A.

***kelleyi*** (*Holarctotrichia*) Harris in Harris and Armitage 1987: 106 [type locality: [United States], Florida, Okaloosa Co., Turkey Creek at Base Road 233, Eglin Air Force Base, 5.0 mile NW Niceville; NMNH; ♂]. —Pescador et al. 2004: 134 [checklist]. —Harris et al. 2012: 10 [checklist].

**Distribution.** —U.S.A.

***kerek*** (*Dactylotrichia*) Oláh & Johanson, 2011: 132 [type locality: Peru, San Martin Prov., Rio Negro, 37 km (rd.) W Moyobamba, near Olmos-Tarapoto rd., 6°00.278'S, 77°15.437'W; NHRS; ♂].

**Distribution.** —Peru.

***kingi*** (*Dactylotrichia*) Holzenthal & Kelley, 1983: 471 [type locality: [United States], Florida, Miami, Plant Inspection Station; NMNH; ♂]. —Pescador et al. 2004: 134 [checklist]. —Harris et al. 2012: 11 [checklist].

**Distribution.** —U.S.A.

***kirikiriroa*** (*Trichoglene*) Smith, 2008: [type locality: [New Zealand], WO, Kirikiriroa Stm, Mangaiti Reserve, Hamilton, E2710781, N6382111; NZAC; ♂].

**Distribution.** —New Zealand.

***klingstedti*** (*Oxyethira*) Nybom, 1983: 65 [type locality: Finland, Fennoscandia, Kuusamo, Posio; MZHF; ♂]. —Andersen and Wiberg-Larsen 1987: 169 [checklist]. —Gullefors 2001: 188 [distribution]. —Gullefors 2002: 138 [checklist]. —Malicky 2004a: 72 [atlas]. —Malicky 2005b: 547 [checklist]. —Gullefors 2008: 63 [checklist]. —Tobias et al. 2009: 25 [♀]. —Ivanov 2011: 195 [checklist]. —Salokannel et al. 2012: 202 [confirmed as distinct species]. —Gullefors 2016: 155 [checklist].

**Distribution.** —Finland, Sweden.

***lagunita*** (*Dampfitrichia*) Flint, 1980b: 142 [type locality: Argentina, Pcia. Entre Rios, Arroyo P. Verne, 4 km N Villa San José; NMNH; ♂]. —Flint 1982b: 42 [distribution]. —Kelley 1984a: 440 [checklist]. —Angrisano 1995a: 510 [distribution]. —Mangeaud 1996: 154 [distribution]. —Angrisano 1999: 34 [checklist]. —Paprocki et al. 2004: 12 [checklist]. —Angrisano and Sganga 2007: 36 [♂; distribution]. —Santos et al. 2009: 36 [checklist]. —Paprocki and França 2014: 52 [checklist]. —Rocha et al. 2018: 152 [checklist].

**Distribution.** —Argentina, Brazil, Uruguay.

***longipenis*** (*Oxytrichia*) Santos, Henriques-Oliveira, & Nessimian, 2009: 40 [type locality: Brazil, Amazonas, Manaus, tributary to Rio Cuieiras, 02°42'25.1"S, 60°22'28.2"W; INPA; ♂; compared with species in O. (Oxitrichia)]. —Paprocki and França 2014: 52 [checklist]. —Rocha et al. 2018: 153 [checklist].

**Distribution.** —Brazil.

***longispinosa*** (*Dampfitrichia*) Kumanski, 1987: 29 [type locality: Cuba, Province Pinar del Rio, Rio El Ballio near Isabel Rubio village, or Rio Esmeralda near Vinales; SOFM; ♂]. —Flint 1996a: 16 [checklist]. —Botosaneanu and Hyslop 1998: 16 [distribution; as *mirebalina* or *longispinosa*]. —Botosaneanu 2002b: 87 [checklist].

**Distribution.** —Cuba, Jamaica [?].

***longissima*** (*Tanytrichia*) Flint, 1974b: 66 [type locality: Suriname, Republiek; RMNH; ♂]. —Kelley 1984a: 440 [checklist]. —Santos et al. 2009: 42 [distribution]. —Paprocki and França 2014: 52 [checklist]. —Rocha et al. 2018: 153 [checklist]. —Moreno et al. 2020: 266 [distribution].

**Distribution.** —Brazil, Suriname.

***luanae*** (*Tanytrichia*) Santos, Henriques-Oliveira, & Nessimian, 2009: 37 [type locality: Brazil, Amazonas, Manaus, tributary to Igarapé da Cachoeira, basin of Rio Ciueiras, 02°41'46.0"S, 60°17'42.7"W; INPA; ♂]. —Paprocki and França 2014: 52 [checklist]. —Rocha et al. 2018: 153 [checklist].

**Distribution.** —Brazil.

***lumipollex*** (*Oxyethira*) Kelley & Harris, 1983: 184 [type locality: [United States], Alabama, Mobile County, Bennett Creek, 6 miles west of Citronelle; NMNH; ♂]. —Harris et al. 1984: 109 [distribution]. —Harris et al. 1991: 251 [distribution].

**Distribution.** —U.S.A.

***lumosa*** (*Oxyethira*) Ross, 1948: 204 [type locality: [United States], Florida, Daytona Beach; INHS; ♂]. —Blickle 1979: 54, 97 [checklist; ♂]. —Kelley and Morse 1982: 257, 267[checklist; ♀]. —Kelley 1984a: 437 [checklist]. —Harris et al. 1991: 252 [distribution]. —Floyd et al. 1993: 91 [phenology; distribution]. —Abbott et al. 1997: 44 [distribution]. —Moulton and Stewart 1997: 351 [checklist]. —Pescador et al. 2004: 134 [checklist]. —Harris et al. 2012: 11 [checklist]. —Denson et al. 2016: 6 [distribution].

**Distribution.** —U.S.A.

♰ ***lurida*** (unplaced) Melnitsky & Ivanov, 2016: 283 [type locality: [Ukraine], Rovno Amber, Bartonian, Eocene; IZSK; ♂; ♀].

**Distribution.** —Rovno amber.

***macropennis*** (unplaced) Wells & Johanson, 2015: 76 [type locality: New Caledonia, Province Sud, south of Plaine des Lacs, 4.0 km N Prony, 22°16.906'S, 166°49.402'E; MNHN; ♂]. —Johanson and Wells 2019: 93 [checklist].

**Distribution.** —New Caledonia.

***macrosterna*** (*Tanytrichia*) Flint, 1974b: 67 [type locality: Suriname, Nickerie River, Blanche Marie, falls in creek; RMNH; ♂]. —Kelley 1984a: 440 [checklist]. —Angrisano 1999: 34 [checklist]. —Santos et al. 2009: 42 [distribution]. —Oláh and Johanson 2011: 134 [distribution]. —Paprocki and França 2014: 52 [checklist]. —de Souza and Santos 2017: 505 [distribution]. —Rocha et al. 2018: 153 [checklist].

**Distribution.** —Brazil, French Guiana, Suriname.

***maranhensis*** (unplaced) de Souza & Santos, 2017: 495 [type locality: Brazil, Maranhão, Carolina, Parque Nacional da Chapada das Mesas, riacho Cancela atrás da Fazenda Cancela, 7°6'43"S, 47°17'46"W, 186 m; DZRJ; ♂]. —Rocha et al. 2018: 153 [checklist].

**Distribution.** —Brazil.

***maryae*** (*Oxytrichia*) Kelley, 1983: 53 [type locality: Colombia, Meta Dept, Refugio Macarena; NMNH; ♂]. —Kelley 1984a: 440 [checklist]. —Muñoz-Quesada 2000: 278 [checklist].

**Distribution.** —Colombia.

***matadero*** (*Dactylotrichia*) Harper & Turcotte, 1985: 138 [type locality: Ecuador, small stream, outlet of Laguna Verde Cocha, near junction with Rio Matadero, Chirimachay, Quinuas Valley; UMQ; ♂]. —Ríos-Touma et al. 2017: 11 [checklist].

**Distribution.** —Ecuador.

***maya*** (*Dampfitrichia*) Denning, 1947a: 16 [type locality: United States, Georgia, Macon; ESUW; ♂]. —Ross 1948: 257 [distribution]. —Zimmerman 1957: 173 [checklist; adult]. —Adachi 1958: 328 [distribution]. —Beardsley 1960: 181 [distribution]. —Beardsley 1971: 15 [distribution]. —Blickle 1979: 54, 95 [checklist; ♂]. —Kelley and Morse 1982: 257, 263 [checklist; ♀]. —Kelley 1984a: 440 [checklist]. —Harris et al. 1991: 253 [distribution]. —Moulton and Stewart 1997: 351 [checklist]. —Flint et al. 2003: 34 [♀; distribution; introduced to Hawaii]. —Pescador et al. 2004: 134 [checklist]. —Bowles et al. 2007: 22 [distribution; biology]. —Harris et al. 2012: 11 [checklist]. —Armitage et al. 2015b: 6 [distribution]. —Armitage et al. 2015a: 7 [checklist]. —Denson et al.Denson et al. 2016: 6 [distribution]. —Armitage and Harris 2018b: 98 [checklist]. —Evenhuis et al. 2020: 27 [distribution].

**Distribution.** —Mexico, Panama, U.S.A.

***mcgregori*** (unplaced) Harris & Huryn, 2000: 78 [type locality: [United States], Alabama, Lauderdale County, Cowpen Creek @ Co. Hwy. 8; NMNH; ♂].

**Distribution.** —U.S.A.

***mekunna*** (*Oxyethira*) Oláh & Ito, 2013: 36 [type locality: Japan, Hokkaido, Shiribeshi, Iwanai-cho, Mekkunai-shitsugen, marsh, N42°52'24" E140°30'17", 900 m; CMB-ZI; ♂]. —Tanida and Kuranishi 2016: 72 [checklist]. —Ito and Oláh 2017: 12 [♂; ♀; distribution].

**Distribution.** —Japan.

***melasma*** (*Pacificotrichia*) Kelley, 1989: 200 [type locality: New Caledonia, mountain stream up Boulari River; BPBM; ♂]. —Wells 1995: 233 [distribution]. —Wells and Johanson 2015: 68 [♂; distribution; note about mismatch between original description and type specimen]. —Johanson and Wells 2019: 93 [checklist].

**Distribution.** —New Caledonia.

***merga*** (*Tanytrichia*) Kelley, 1983: 45 [type locality: Venezuela, Bolivar State, Rio Cuyuni, El Dorado; NMNH; ♂]. —Kelley 1984a: 440 [checklist]. —Flint 1991a: 70 [distribution]. —Angrisano 1999: 34 [checklist]. —Paprocki et al. 2004: 12 [checklist]. —Santos et al. 2009: 36 [checklist]. —Paprocki and França 2014: 52 [checklist]. —de Souza and Santos 2017: 505 [distribution]. —Rocha et al. 2018: 153 [checklist]. —Moreno et al. 2020: 266 [distribution].

**Distribution.** —Brazil, Venezuela.

***michiganensis*** (*Holarctotrichia*) Mosely, 1934a: 153 [type locality: U.S.A., Michigan; NHMUK; ♂]. —Blickle and Morse 1954: 122 [♂; distribution]. —Etnier 1965: 147 [distribution]. —Roy and Harper 1979: 152 [checklist]. —Blickle 1979: 54, 91 [checklist; ♂]. —Parker and Voshell 1981: 4 [checklist]. —Roy and Harper 1981: 105 [distribution]. —Kelley and Morse 1982: 257, 265 [checklist; ♀]. —Huryn and Foote 1983: 791 [distribution]. —Kelley 1984a: 438 [checklist]. —Usis and Foote 1989: 84 [distribution]. ——Harper 1989: 541 [distribution]. —Morse et al. 1989: 23 [distribution]. —Harris et al. 1991: 254 [distribution]. —Masteller and Flint 1992: 70 [checklist]. —Monson and Holzenthal 1993: 442 [checklist]. —Floyd et al. 1997: 136 [distribution]. USA —Huryn and Harris 2000: 193 [distribution]. —Houghton et al. 2001: 505 [distribution]. —Houghton et al. 2011b: 6 [phenology habitat]. —Myers et al. 2011: 108 [distribution]. —Armitage et al. 2011: 14 [checklist]. —Houghton 2016: 46 [biology]. —Houghton et al. 2017: 63 [checklist].

—*sodalis* Ross & Spencer, 1952: 46 [type locality: [Canada] Soda Creek, British Columbia; INHS; ♂]. —Blickle 1979: 54 [distribution, to synonymy].

**Distribution.** —Canada, U.S.A.

***miea*** (*Oxyethira*) Oláh & Ito, 2013: 42 [type locality: Japan, Honshu, Mie, Taisei-cho, N36°19' E136°26'; CMB-ZI; ♂]. —Tanida and Kuranishi 2016: 72 [checklist]. —Ito and Oláh 2017: 14 [♂; ♀; distribution]. —Park et al. 2018: 107 [♂; ♀; distribution]. —Park and Kong 2020: 297 [checklist].

**Distribution.** —South Korea.

***mienica*** (*Trichoglene*) Wells, 1981: 108 [type locality: [Australia] Tasmania, Ouse River, 5 miles W. of Miena ANIC; ♂; ♀]. —Kelley 1984a: 436 [checklist]. —Neboiss 1986: 83 [atlas; ♂; ♀]. —Neboiss 2002: 54 [checklist].

**Distribution.** —Australia.

***minima*** (*Dampfitrichia*) (Kimmins, 1951): 208 [type locality: [Myanmar], S. Shan States, Inle Lak, S. end, 900 m; NHMUK; ♂; in *Stenoxyethira*]. —Kelley 1984a: 438 [checklist]. —Wityi et al. 2015: 47 [checklist].

**Distribution.** —Myanmar.

***mirabilis*** (*Oxytrichia*) Morton, 1904: 327 [type locality: [Scotland], Loch Eigheach, Rannoch, Perthshire; depository not designated; ♂]. —Siltala 1908: 16 [distribution]. —Mosely 1939b: 289 [♂]. —Nybom 1960: 19 [checklist]. —Botosaneanu 1967: 294 [distribution]. —Andersen 1974: 26 [distribution]. —Botosaneanu and Malicky 1978: 340 [checklist]. —Nybom 1983: 65 [distribution]. —Malicky 1983b: 59, 60 [atlas; ♂; ♀]. —Kelley 1984a: 440 [checklist]. —Andersen and Tysse 1985: 84 [distribution]. —Andersen and Wiberg-Larsen 1987: 169 [checklist]. —Harper 1989: 541 [distribution]. —Spuris 1989: 17 [checklist]. —Holmes et al. 1992: 202 [distribution]. —Moulton and Harris 1999: 550 [♂; distribution]. —Gullefors 2002: 138 [checklist]. —Malicky 2004a: 71, 72 [atlas]. —Malicky 2005b: 547 [checklist]. —Gullefors 2008: 63 [checklist]. —Ivanov 2011: 196 [checklist]. —Andersen and Hagenlund 2012: 136 [distribution]. —Salokannel et al. 2012: 202 [confirmed as distinct species]. —Pan’kov and Krasheninnikov 2016: 333 [distribution]. —Gullefors 2016: 155 [checklist]. —Wallace 2016: 15, 17, 18, 24 [conservation status]. —O’Connor 2019b: 231 [distribution].

—*barnstoni* Harper, 1976: 35 [type locality: [Canada], Québec, Radissonie, tourbière (Station SB-153) au nord du lac Nathalie (lac B-160) dans le bassin hydrographique de la Rivière du Castor, un tributaire de la Baie James, 53°25'20"N, 77°25'30"W; UMQ; ♀]. —Blickle 1979: 54, 95 [checklist; ♂]. —Roy and Harper 1979: 151 [checklist]. —Blickle 1980: 102 [♂]. —Kelley and Morse 1982: 258 [to synonymy].

**Distribution.** —Canada, Finland, Ireland, Norway, Russia, Scotland, Sweden, U.S.A, Wales.

***mirebalina*** (*Dampfitrichia*) Botosaneanu, 1991: 130 [type locality: Haiti, Département de l’Ouest, Grande rivière d l’Artibonite à Mirebalais; ZMUA; ♂; ♀]. —Botosaneanu 1995a: 29 [distribution]. —Botosaneanu and Hyslop 1998: 16 [distribution; as *mirebalina* or *longispinosa*]. —Flint and Pérez-Gelabert 1999: 41 [checklist]. —Botosaneanu 2002b: 87 [checklist]. —Flint and Sykora 2004: 41 [distribution; ♀ allotype is actually *Oxyethirasimulatrix*]. —Pérez-Gelabert 2008: 301 [checklist].

**Distribution.** —Dominican Republic, Haiti, Jamaica [?].

***misionensis*** (*Dactylotrichia*) Angrisano, 1995b: 30 [type locality: Argentina, Misiones, Posadas; MACN; ♂]. —Angrisano 1999: 34 [checklist].

**Distribution.** —Argentina.

***mithi*** (*Oxyethira*) Malicky, 1974: 109 [type locality: [Greece], Kreta, Mithi; Collection Malicky; ♂]. —Botosaneanu and Malicky 1978: 340 [checklist]. —Malicky 1983b: 59 [atlas; ♂]. —Kelley 1984a: 437 [checklist]. —Malicky 2004a: 71, 72 [atlas]. —Malicky 2005b: 547 [checklist] —Malicky 2005a: 68 [distribution].

**Distribution.** —Greece.

***mocoi*** (unplaced) Angrisano, 1995b: 34 [type locality: Argentina, Entre Rios, Parque Nacional El Palmar; MACN; ♂]. —Angrisano 1999: 35 [checklist]. —Angrisano and Sganga 2007: 38 [♂; distribution].

**Distribution.** —Argentina.

***mouirange*** (*Pacificotrichia*) Wells & Johanson, 2015: 65 [type locality: New Caledonia, Province Sud, Monts Ksa Ne Mwa, on road between Noumea and Yaté, 2.0 km E Pic Mouirange, 22°12.356'S, 166°40.798'E, 220 m; MNHN; ♂]. —Johanson and Wells 2019: 93 [checklist].

**Distribution.** —New Caledonia.

***nehoue*** (*Pacificotrichia*) Wells & Johanson, 2015: 69 [type locality: New Caledonia, Province Nord, Rivière Néhoué, camp Amenage de Néhoué, 20°25.037'S, 164°13.222'E, 12 m; MNHN; ♂]. —Johanson and Wells 2019: 93 [checklist].

**Distribution.** —New Caledonia.

***novasota*** (*Oxyethira*) Ross, 1944: 138 [type locality: [United States], Texas, Marquez, along Novasota River; INHS; ♂; ♀]. —Edwards 1973: 506 [distribution]. —Blickle 1979: 54, 97 [checklist; ♂]. —Kelley and Morse 1982: 257, 261 [checklist; ♀]. —Harris et al. 1982a: 512 [distribution]. —Harris et al. 1982b: 81 [distribution]. —Harris et al. 1984: 109 [distribution]. —Kelley 1984a: 437 [checklist]. —Bowles and Mathis 1989: 240 [distribution]. —Harris et al. 1991: 255 [distribution]. —Floyd et al. 1993: 91 [phenology; distribution]. —Moulton and Stewart 1996: 128 [♂; distribution]. —Abbott et al. 1997: 44 [distribution]. —Moulton and Stewart 1997: 351 [checklist]. —Pescador et al. 2004: 134 [checklist]. —DeWalt and Heinold 2005: 42 [phenology; distribution]. —Etnier 2010: 486 [distribution]. —Armitage et al. 2011: 14 [checklist]. —Harris et al. 2012: 11 [checklist]. —Denson et al. 2016: 6 [distribution]. —Houghton et al. 2017: 63 [checklist].

**Distribution.** —U.S.A.

***nyultka*** (*Tanytrichia*) Oláh & Johanson, 2011: 134 [type locality: French Guiana, Approuaguekaw, Kaw Mt 4°32.833'N, 52°11.452'W; NHRS; ♂].

**Distribution.** —French Guiana.

***obscura*** (*Oxytrichia*) Flint, 1974b: 69 [type locality: Suriname, Suriname River, Botopasie; RMNH; ♂]. —Kelley 1984a: 440 [checklist]. —Angrisano 1995a: 510 [distribution]. —Angrisano 1999: 34 [checklist].

**Distribution.** —Suriname, Uruguay.

***obtatus*** (*Holarctotrichia*) Denning, 1947b: 172 [type locality: [United States], Minnesota, St. Paul; UMSP; ♂]. —Etnier 1965: 147 [checklist]. —Roy and Harper 1979: 152 [checklist]. —Blickle 1979: 54, 93 [checklist; ♂]. —Marshall and Larson 1982: 31 [distribution]. —Kelley 1984a: 438 [checklist]. —Lake 1984: 220 [distribution]. —Harper 1989: 541 [distribution]. —Monson and Holzenthal 1993: 442 [checklist]. —Huryn and Harris 2000: 193 [distribution]. —Houghton et al. 2001: 505 [distribution]. —Houghton et al. 2011b: 6 [phenology; distribution; habitat]. —Myers et al. 2011: 108 [distribution]. —Wright et al. 2013: 466 [biology; distribution]. —Houghton 2016: 46 [biology]. —Houghton et al. 2017: 63 [checklist].

**Distribution.** —Canada, U.S.A.

***okinawa*** (*Oxyethira*) Oláh & Ito, 2013: 43 [type locality: Japan, Ryukyu Islands, Okinawa, Nago-shi, Genka-kawa, near Hogen-hashi, N26°36' E128°05'; CMB-ZI; ♂]. —Tanida and Kuranishi 2016: 72 [checklist]. —Ito and Oláh 2017: 16 [♂; ♀; distribution].

**Distribution.** —Japan.

***oropedion*** (*Pacificotrichia*) Kelley, 1989: 200 [type locality: New Caledonia, Plateau de Dogny; BPBM; ♂]. —Wells 1995: 233 [distribution]. —Wells and Johanson 2015: 56 [♂; distribution]. —Johanson and Wells 2019: 93 [checklist].

—*derek* Oláh & Johanson, 2010a: 31 [type locality: New Caledonia, Province Sud, Creek Froid, 10 m upstream bridge on La Foa-Koindé road, 200 m W crossroad to Quipouin, 21°38.581'S, 165°56.672'E, 180 m; MNHN; ♂]. —Wells and Johanson 2015: 56 [to synonymy].

**Distribution.** —New Caledonia.

***orellanai*** (*Tanytrichia*) Harris & Davenport, 1992: 465 [type locality: Peru, Loreto, Rio Sucusari just up stream from Explornapo Camp; NMNH; ♂].

**Distribution.** —Peru.

***ortizorum*** (*Mesotrichia*) Botosaneanu, 1995a: 29 [type locality: Dominican Republic, Arroyo el Dulce, sección Manavao-Los Dajaos of Jarabacoa; ZMUA; ♂]. —Botosaneanu 2002b: 88 [checklist]. —Flint and Pérez-Gelabert 1999: 41 [checklist]. —Flint and Sykora 2004: 41 [distribution]. —Pérez-Gelabert 2008: 301 [checklist].

**Distribution.** —Dominican Republic.

***ouenghi*** (*Pacificotrichia*) Wells & Johanson, 2015: 65 [type locality: New Caledonia, Province Nord, Bouérabate Stream, S Mont Ninndo, along road Barabache-Boulagoma, 20°17.409'S, 164°11.242'E, 60 m; MNHN; ♂]. —Johanson and Wells 2019: 93 [checklist].

**Distribution.** —New Caledonia.

***ozea*** (*Oxyethira*) Oláh & Ito, 2013: 41 [type locality: Japan, Honshu, Gumma, Oze, Yamanohama, N36°55' E139°13', 1400 m; CMB-ZI; ♂]. —Tanida and Kuranishi 2016: 73 [checklist]. —Ito and Oláh 2017: 18 [♂; distribution].

**Distribution.** —Japan.

***palisada*** (*Argyrobothrus*) Wells & de Moor, 2020: 503 [type locality: Angola, Moxico Province, Cuanavale River, Site 3 — Cuanavale source lake (at Mokoro), -13.0898, 18.89395; AGMS; ♂].

**Distribution.** —Angola.

***pallida*** (*Dampfitrichia*) (Banks, 1904a): 215 [type locality: [United States, Washington, D. C.], Potomac river near the Long Bridge; Collection Banks; ♂; in *Orthotrichia*]. —Banks 1907a: 50 [catalogue]. —Betten 1934: 152 [checklist]. —Ross 1938b: 10 [to *Oxyethira*]. —Ross 1944: 137 [♂; ♀; distribution]. —Ross 1948: 204 [distribution]. —Morse and Blickle 1953: 72 [checklist]. —Etnier 1965: 148 [distribution]. —Resh et al. 1978: 383 [distribution]. —Blickle 1979: 54; 95 [checklist; ♂]. —Parker and Voshell 1981: 4 [checklist]. —Kelley and Morse 1982: 257, 263 [checklist; ♀]. —Harris et al. 1982a: 512 [distribution]. —Waltz and McCafferty 1983a: 11 [distribution]. —Huryn and Foote 1983: 791 [distribution]. —Hamilton et al. 1983: 19 [distribution]. —Kelley 1984a: 440 [checklist]. —Lake 1984: 220 [distribution]. —Harris et al. 1984: 109 [distribution]. —Dewey 1986: 156 [biology]. —Bowles and Mathis 1989: 240 [distribution]. —Tarter 1990: 239 [checklist]. —Floyd and Schuster 1990: 130, 132 [distribution]. —Harris et al. 1991: 256 [distribution]. —Frazer et al. 1991: 20 [distribution]. —Masteller and Flint 1992: 70 [checklist]. —Mathis and Bowles 1992: 24 [distribution]. —Bowles and Mathis 1992: 32 [distribution]. —Floyd et al. 1993: 91 [phenology; distribution]. —Monson and Holzenthal 1993: 442 [checklist]. —Moulton et al. 1994: 171 [distribution]. —Moulton et al. 1994: 171 [distribution]. —Moulton and Stewart 1996: 128 [♂; distribution]. —Abbott et al. 1997: 44 [distribution]. —Moulton and Stewart 1997: 351 [checklist]. —Keiper et al. 1998b: 87 [biology]. —Ruiter 1999: 166 [distribution]. —Houghton et al. 2001: 505 [distribution]. —Pescador et al. 2004: 134 [checklist]. —DeWalt and Heinold 2005: 42 [phenology; distribution]. —Blinn and Ruiter 2005: 69 [distribution; biology]. —Blinn and Ruiter 2006: 333 [biology; distribution]. —Zeullig et al. 2006: 43 [distribution]. —Bowles et al. 2007: 22 [distribution; biology]. —Armitage et al. 2011: 14 [checklist]. —Flint 2011: 104 [distribution]. —Harris et al. 2012: 11 [checklist]. —Houghton et al. 2011b: 6 [phenology habitat]. —Denson et al. 2016: 6 [distribution]. —Houghton 2016: 46 [biology]. —DeWalt et al. 2016: 52 [distribution]. —Houghton et al. 2017: 63 [checklist]. —Mendez et al. 2019: 119 [checklist]. —Bowles et al. 2020: 8 [distribution].

—*cibola* Denning, 1947a: 12 [type locality: [United States], Georgia, Macon; ESUW; ♂]. —Denning 1947a: 148 [distribution]. —Ross 1948: 204 [to synonymy].

—*viminalis* Morton, 1905: 71 [type locality: [United States], Ithaca, New York; depository not designated; ♂]. —Banks 1907a: 50 [catalogue]. —Betten 1934: 163 [checklist]. —Ross 1938a: 10 [to synonymy].

**Distribution.** —U.S.A.

***paludicola*** (*Dampfitrichia*) Wells & Yule, 2008 [type locality: Peninsular Malaysia, Selangor, Peat Swamp Forest, 03°39'6.3"N 101°15'12.2"E, irrigation drain; ZRC; ♂]. —Malicky and Chantaramongkol 2007: 1029 [distribution]. —Malicky 2007a: 177 [checklist]. —Malicky 2010a: 42 [atlas; ♂].

**Distribution.** —Indonesia, Malaysia.

***parazteca*** (*Loxotrichia*) Kelley, 1983: 53 [type locality: Ecuador, Cotopaxi Prov., 133 km W Latacunga; NMNH; ♂]. —Kelley 1984a: 442 [checklist]. —Holzenthal 1988: 63 [distribution]. —Holzenthal and Harris 1992: 173 [distribution]. —Armitage et al. 2016: 11 [distribution]. —Ríos-Touma et al. 2017: 11 [checklist]. —Armitage and Harris 2018b: 98 [checklist]. —Armitage and Harris 2018c: 283 [distribution]. —Harris and Armitage 2019: 5 [distribution].

**Distribution.** —Costa Rica, Ecuador, Panama.

***parce*** (*Loxotrichia*) (Edwards & Arnold, 1961): 405 [type locality: United States, Texas, Caldwell Co., San Marcos River; type destroyed; ♂; in *Protoptila*]. —Etnier 1968: 191 [distribution]. —Flint 1981: 30 [as synonym of *O.azteca*]. —Flint 1991b: 51 [♂; distribution]. —Flint and Reyes 1991: 487 [♂; ♀; distribution]. —Holzenthal and Harris 1992: 173 [distribution]. —Flint 1996b: 99 [distribution]. —Mangeaud 1996: 154 [distribution]. —Harris et al. 1996: 240 [distribution]. —Moulton and Stewart 1997: 351 [checklist]. —Angrisano 1999: 34 [checkilist]. —Muñoz-Quesada 2000: 278 [checklist]. —Botosaneanu 2002b: 88 [checklist]. —Botosaneanu and Viloria 2002: 12 [checklist]. —Blahnik et al. 2004: 5 [distribution]. —Paprocki et al. 2004: 12 [checklist]. —Muzón et al. 2005: 57 [distribution]. —Rueda Martín 2011: 9 [♂; distribution]. Santos et al. 2009: 36 [checklist]. —Paprocki and França 2014: 52 [checklist]. —Isa Miranda and Rueda Martín 2014: 199 [distribution]. —Armitage et al. 2015a: 7 [checklist]. —Ríos-Touma et al. 2017: 11 [distribution]. —Rocha et al. 2018: 152 [checklist]. —Armitage and Harris 2018b: 98 [checklist]. —Armitage and Harris 2018c: 283 [distribution]. —Harris and Armitage 2019: 5 [distribution].

**Distribution.** —Argentina, Bolivia, Brazil, Colombia, Costa Rica, Ecuador, Guyana, Mexico, Panama, Peru, Trinidad, Venezuela, U.S.A.

***parinsularis*** (*Trichoglene*) Wells & Johanson, 2015: 55 [type locality: New Caledonia, Province Sud, Mt Dzumac, source stream of Ouinne River, near crosspoint to mountain track, 22°02.073'S, 166°28.460'E, 810 m; MNHN; ♂]. —Johanson and Wells 2019: 93 [checklist].

**Distribution.** —New Caledonia.

***paritentacula*** (*Tanytrichia*) Kelley, 1983: 45 [type locality: Belize, Cayo Dist., Rio Privassion, Blancaneaux Lodge; NMNH; ♂]. —Kelley 1984a: 440 [checklist].

**Distribution.** —Belize.

***pembertonensis*** (*Oxytrichia*) Harris & Flint, 2016: 5 [type locality: Canada, British Columbia, Pemberton, Pemberton Creek, N50 18.9', W122 48.2'; NMNH; ♂; ♀].

**Distribution.** —Canada.

***perignonica*** (*Trichoglene*) Wells & Johanson, 2015: 45 [type locality: New Caledonia, Province Sud, stream draining to Marais de la Rivière Blanche, 5 km SW Pont Pérignon, 22°09.513'S, 166°39.942'E, 180 m; MNHN; ♂]. —Johanson and Wells 2019: 93 [checklist].

**Distribution.** —New Caledonia.

***peruviana*** (unplaced) Harris & Davenport, 1999: 33 [type locality: Peru, Loreto, tributary to Rio Yanamono at Explorama Lodge; NMNH; ♂]. —Santos et al. 2009: 43 [distribution]. —Paprocki and França 2014: 53 [checklist]. —Rocha et al. 2018: 153 [checklist].

**Distribution.** —Brazil, Peru.

***pescadori*** (*Oxyethira*) Harris & Keth, 2002: 74 [type locality: [United States], Alabama, Henry County, East Fork of Choctawhatchee River at Co. Hwy. 40, 10.5 km WSW Abeville, T7N, R27E, S29; NMNH; ♂]. —Pescador et al. 2004: 134 [checklist]. —Etnier 2010: 486 [distribution]. —Harris et al. 2012: 11 [checklist]. —Denson et al. 2016: 6 [distribution].

**Distribution.** —U.S.A.

***petei*** (*Oxytrichia*) Angrisano, 1995b: 29 [type locality: Argentina, Entre Rios, Parque Nacional el Palmar; MACN; ♂]. —Angrisano 1999: 34 [checklist]. —Angrisano and Sganga 2007: 36 [♂; distribution].

**Distribution.** —Argentina.

***picita*** (*Tanytrichia*) Harris & Davenport, 1999: 35 [type locality: Peru, Loreto, edge of Rio Sucusari backwater, adjoining Explornapo Camp; NMNH; ♂]. —Santos et al. 2009: 43 [distribution]. —Thomson and Holzenthal 2012: 31 [distribution]. —Paprocki and França 2014: 53 [checklist]. —Rocha et al. 2018: 153 [checklist].

**Distribution.** —Brazil, Peru, Venezuela.

***ping*** (*Oxyethira*) Malicky & Chantaramongkol, 2007: 1028 [type locality: Thailand, Prov. Chiangmai, Mae Ping bei Elephant Camp, 16 km S von Chiang Dao; Collection Malicky; ♂]. —Malicky 2010a: 41 [atlas; ♂].

**Distribution.** —Thailand.

***pirisinui*** (*Oxyethira*) Moretti, 1981: 170 [type locality: [Italy], Isola Capraia, Vado del Porto, 10 m; Collection Moretti; ♂]. —Moretti and Cianficconi 1981: 201 [checklist]. —Malicky 1983b: 60 [atlas; ♂]. —Kelley 1984a: 438 [checklist]. —Malicky and Lounaci 1987: 15, 17 [checklist]. —Malicky 2004a: 72 [atlas]. —Malicky 2005b: 547 [checklist]. —Cianficconi et al. 2007: 67 [proposed as Italian endemic].

**Distribution.** —Italy.

***plumosa*** (*Dampfitrichia*) (Wells, 1981): 117 [type locality: [Australia] North Queensland, Mulgrave River; NMV; ♂; in *Stenoxyethira*]. —Kelley 1984a: 438 [checklist]. —Neboiss 1986: 82 [atlas; ♂]. —Wells 1991: 491 [distribution]. —Wells et al. 2019: 33 [detection frequency].

**Distribution.** —Australia, Papua New Guinea.

***poapi*** (unplaced) Angrisano & Sganga, 2009: 67 [type locality: Argentina, Misiones, Parque Provincial Salto Encantado MACN; ♂].

**Distribution.** —Argentina.

***presilla*** (unplaced) Harris & Davenport, 1999: 29 [type locality: Peru, Loreto, Yanamono Creek at jungle’s edge, near Explorama Lodge; NMNH; ♂]. —Santos et al. 2009: 43 [distribution]. —Paprocki and França 2014: 53 [checklist]. —Rocha et al. 2018: 153 [checklist].

**Distribution.** —Brazil, Peru.

***pseudofalcata*** (*Oxyethira*) Ivanov, 1992: 239 [type locality: [Tajikistan], Pamir, Vanch region, 24 km E Vanch, source Zak on the left bank of river Vanch; ZIN; ♂; ♀].

**Distribution.** —Tajikistan.

***puertoricensis*** (*Loxotrichia*) Flint, 1964: 55 [type locality: Puerto Rico, Maricao, at fish hatchery; NMNH; ♂; ♀; larva; case]. —Flint 1968b: 40 [distribution]. —Flint 1968a: 82 [checklist]. —Kelley 1984a: 442 [checklist]. —Botosaneanu 1991: 132 [distribution]. —Botosaneanu 1995a: 32 [distribution]. —Flint 1996a: 16 [checklist as *quelinda*]. —Botosaneanu and Hyslop 1998: 17 [distribution]. —Flint and Pérez-Gelabert 1999: 41 [checklist]. —Botosaneanu 2002b: 88 [checklist]. —Flint and Sykora 2004: 44 [distribution]. —Naranjo López and González Lazo 2005: 149 [checklist]. —Pérez-Gelabert 2008: 30 [checklist].

—*quelinda* (*Loxotrichia*) (Botosaneanu), 1977: 267 [type locality: Cuba, Oriente, Baracoa, Rio Sabanilla; NMNH; ♂; in *Loxotrichia*]. —Botosaneanu 1979: 50 [distribution]. —Kelley 1984a: 442 [checklist]. —Botosaneanu 1995a: 32 [to synonymy].

**Distribution.** —Cuba, Dominican Republic, Haiti, Jamaica, Puerto Rico.

***quadrata*** (*Pacificotrichia*) Wells & Johanson, 2015: 60 [type locality: New Caledonia, Province Sud, Mt Dzumac, source stream of Ouinne River, near crosspoint to mountain track, 22°02.073'S, 166°28.460'E, 810 m; MNHN; ♂]. —Johanson and Wells 2019: 93 [checklist].

**Distribution.** —New Caledonia.

***quadrilobata*** (*Loxotrichia*) Rocha, Dumas, & de Souza, 2018: 150[type locality: Brazil, Minas Gerais, São Roque de Minas, Parque Nacional da Serra da Canastra, parte baixa da Cachoeira Casca D’anta, Rio São Francisco, 20°18.54'S, 46°31.37'W, ca 900 m elev; DZRJ; ♂].

**Distribution.** —Brazil.

***quinquaginta*** (unplaced) Kelley, 1983: 54 [type locality: Ecuador, Pastaza Prov., Puyo; NMNH; ♂]. —Kelley 1984a: 442 [checklist]. —Ríos-Touma et al. 2017: 11 [checklist].

**Distribution.** —Ecuador.

***quiramae*** (*Dactylotrichia*) Thomson & Holzenthal, 2012: 31 [type locality: Venezuela, Guárico, UCV San Nicolasito Field Station, 08°8.296'N, 66°24.459'W, 62 m; UMSP; ♂].

**Distribution.** —Venezuela.

***rachanee*** (*Dampfitrichia*) Chantaramongkol & Malicky, 1986: 516 [type locality: [Sri Lanka], Central Province, Bacj 2 mi E von Madugoda, 18 mi E von Kandy, 800 m; MZLU; ♂].

**Distribution.** —Sri Lanka.

***rafaeli*** (unplaced) de Souza & Santos, 2017: 493 [type locality: Brazil, Piauí, Piracuruca, Parque Nacional de Sete Cidades, Riacho Piedade, 4°06'34"S, 41°43'39"W, 169 m; CZMA; ♂]. —Rocha et al. 2018: 153 [checklist]. —Moreno et al. 2020: 266 [distribution].

**Distribution.** —Brazil.

***ramosa*** (*Oxyethira*) Martynov, 1936: 306 [type locality: [India], Rewah State, C. I.; NZSI; ♀]. —Malicky and Chantaramongkol 2007: 1029 [distribution]. —Mattern 2015: 502 [distribution]. —Malicky 2018: 49 [checklist].

—*angustella* Martynov, 1935: 112 [type locality: [India], Rewah State, C. I.; NZSI; ♀]. —Martynov 1936: 306 [as replacement]. —Kelley 1984a: 437 [checklist]. —Malicky and Chantaramongkol 2007: 1029 [♂; distribution].

—*laodameia* Malicky, 2004b: 296 [type locality: [Nepal, Bardia National Park], am Rande der nordindischen Ebene im Südwesten von Nepal im Bereich des ersten Hügelkammes des Himalaya (Siwalik Range), unweit des Wehrs des Babai Flusses, über das die Brücke der Ost-West-Haupstraße Nepals (Mahindra Highway), 28°25'N, 81°23'E, 190 m; Collection Malicky; ♂]. —Malicky 2006: 253 [checklist].

**Distribution.** —India, Nepal.

***rareza*** (unplaced) Holzenthal & Harris, 1992: 33 [type locality: Costa Rica, Alajuela, Río Pizote, 5 km N Dos Ríos, 10.948°N, 85.291°W; NMNH; ♂]. —Armitage et al. 2016: 11 [distribution]. —Armitage and Harris 2018b: 98 [checklist].

**Distribution.** —Costa Rica, Panama.

***redunca*** (unplaced) Thomson & Holzenthal, 2012: 33 [type locality: Venezuela, Bolívar, Gran Sabana, E. Pauji, “Río Curvita”, 4°31.237'N, 61°31.591'W, 869 m; UMSP; ♂].

**Distribution.** —Venezuela.

***retracta*** (*Trichoglene*) Wells, 1981: 110 [type locality: [Australia] Western Australia, Serpentine River, Serpentine Falls; NMV; ♂; ♀]. —Kelley 1984a: 436 [checklist]. —Neboiss 1986: 84 [atlas; ♂; ♀].

**Distribution.** —Australia.

***retrosa*** (*Dactylotrichia*) de Souza & Santos, 2017: 486 [type locality: Brazil, Sergipe, Itabaiana, Parque Nacional de Serra de Itabaiana, Riacho dos Negros, 10°44'50"S, 37°20'24"W, 202 m; DZRJ; ♂]. —Rocha et al. 2018: 152 [checklist].

**Distribution.** —Brazil.

***ritae*** (*Loxotrichia*) Angrisano, 1995a: 510 [type locality: Uruguay, Paysandu, Sta. Rita; FHCU; ♂]. —Angrisano 1999: 34 [checklist].

**Distribution.** —Uruguay.

***rivicola*** (*Oxyethira*) Blickle & Morse, 1954: 121 [type locality: [United States], Lee, N. H.; INHS; ♂]. —Etnier 1965: 148 [distribution]. —Blickle 1979: 54, 93 [checklist]. —Roy and Harper 1979: 152 [checklist]. —Etnier and Schuster 1979: 18 [distribution]. —Parker and Voshell 1981: 4 [checklist]. —Roy and Harper 1981: 105 [distribution]. —Kelley and Morse 1982: 257, 264 [checklist; ♀]. —Kelley 1984a: 437 [checklist]. —Harper 1989: 541 [distribution]. —Bowles and Mathis 1989: 240 [distribution]. —Harper 1990: 49 [distribution; biology]. —Harris et al. 1991: 257 [distribution]. —Frazer et al. 1991: 20 [distribution]. —Masteller and Flint 1992: 70 [checklist]. —Monson and Holzenthal 1993: 442 [checklist]. —Moulton and Stewart 1996: 129 [♂; distribution]. —Floyd et al. 1997: 136 [distribution]. USA —Huryn and Harris 2000: 193 [distribution]. —Houghton et al. 2001: 505 [distribution]. —Etnier 2010: 486 [distribution]. —Myers et al. 2011: 109 [distribution]. —Houghton et al. 2011b: 6 [phenology; habitat]. —Houghton 2016: 46 [biology]. —Houghton et al. 2017: 63 [checklist].

**Distribution.** —Canada, U.S.A.

***roberti*** (*Argyrobothrus*) Roy & Harper, 1980: 117 [type locality: [Canada], les Basses laurentides, dans la partie supérieure du bassin hydrographique de la rivière l’Achigan, 46°00'N, 74°00'O, lac Tracy (Cromwell); UMQ; ♂]. —Roy and Harper 1981: 105 [distribution]. —Kelley 1984a: 438 [checklist]. —Harris et al. 1991: 258 [distribution]. —Abbott et al. 1997: 44 [distribution]. —Moulton and Stewart 1997: 351 [checklist]. —Pescador et al. 2004: 134 [checklist]. —Harris et al. 2012: 11 [checklist]. —Denson et al. 2016: 6 [distribution].

—*leonensis* Kelley, 1981: 374 [type locality: [United States], Florida, Leon Co., Tall Timbers Res. Sta.; NMNH; ♂]. —Kelley and Morse 1982: 257 [checklist]. —Kelley 1984a: 438 [to synonymy].

**Distribution.** —Canada, U.S.A.

***rossi*** (*Oxyethira*) Blickle & Morse, 1957: 48 [type locality: [United States], Bow, N.H. [New Hampshire]; INHS; ♂]. —Etnier and Schuster 1979: 18 [distribution]. —Blickle 1979: 54, 95 [checklist; ♂]. —Kelley and Morse 1982: 257, 264 [checklist; ♀. —Kelley 1984a: 437 [checklist]. —Monson and Holzenthal 1993: 442 [checklist]. —Huryn and Harris 2000: 193 [distribution]. —Houghton et al. 2001: 505 [distribution]. —Myers et al. 2011: 109 [distribution].

—*berneri* Etnier, 1965: 142 [type locality: [United States], Minnesota, Lake County, Finland; UMSP; ♂]. —Etnier 1965: 147 [checklist]. —Etnier 1968: 191 [to synonymy].

**Distribution.** —U.S.A.

***rougensis*** (*Pacificotrichia*) Wells & Johanson, 2015: 63 [type locality: New Caledonia, Province Nord, Plaine des Gaïacs, Rivière Rouge, 14.2 km NW summit of Mt Rouge, 50 m upstream road RT1 Noumea-Koné, 21°31.573'S, 164°46.690'E, 23 m; MNHN; ♂]. —Johanson and Wells 2019: 93 [checklist].

**Distribution.** —New Caledonia.

***sagittifera*** (*Holarctotrichia*) Ris, 1897: 421 [type locality: [Switzerland], Hausersee bei Ossingen, Ct. Zürich; depository not designated; ♂]. —Morton 1904: 328 [distribution]. —Mosely 1939b: 287 [♂]. —Kimmins 1943: 155 [distribution]. —Kimmins 1958b: 11 [♀; distribution]. —Nybom 1960: 19 [checklist]. —Spuris 1962: 68 [distribution]. —Spuris 1964: 13 [distribution]. —Botosaneanu 1967: 293 [distribution]. —Andersen 1974: 26 [distribution]. —Botosaneanu and Malicky 1978: 340 [checklist]. —Malicky 1983b: 59, 60 [atlas; ♂; ♀]. —Kelley 1984a: 438 [checklist]. —Wiberg-Larsen 1985: 40 [checklist]. —Andersen and Wiberg-Larsen 1987: 169 [checklist]. —Spuris 1989: 17 [checklist]. —Mey 1991: 270 [distribution]. —Gullefors 2002: 138 [checklist]. —Malicky 2004a: 71, 72 [atlas]. —Malicky 2005b: 547 [checklist]. —Gullefors 2005a: 118, 119 [distribution]. —Lubini-Ferlin and Vicentini 2005: 68 [checklist]. —Wiggers et al. 2006: 54 [distribution]. —Robert 2007: 83 [checklist]. —Ivanov 2011: 196 [checklist]. —Salokannel et al. 2012: 202 [confirmed as distinct species]. —Andersen and Hagenlund 2012: 136 [distribution]. —O’Connor 2013: 65 [distribution]. —Barndt 2014: 106 [distribution]. —O’Connor 2015: 28, 103 [distribution]. —Zuyderduyn 2016: 7 [distribution]. —Gullefors 2016: 156 [checklist]. —Wallace 2016: 21, 23, 68 [conservation status]. —O’Connor and O’Connor 2018: 84 [distribution]. —Gullefors 2018: 108 [biology; distribution].

**Distribution.** —Denmark, England, Finland, Germany, Ireland, Latvia, Netherlands, Norway, Russia, Scotland, Sweden, Switzerland.

***santiagensis*** (*Dactylotrichia*) Flint, 1982a: 46 [type locality: Argentina, Pcia. Buenos Aires, Rio Santiago, Palo Blanco, Berisso; NMNH; ♂]. —Flint 1982b: 43 [distribution]. —Kelley 1984a: 442 [checklist]. —Angrisano 1995a: 511 [distribution]. —Angrisano 1999: 34 [checklist]. —Paprocki et al. 2004: 12 [checklist]. —Santos et al. 2009: 37 [checklist]. —Paprocki and França 2014: 53 [checklist]. —Rocha et al. 2018: 152 [checklist].

**Distribution.** —Argentina, Brazil, Uruguay.

***savanniensis*** (*Oxyethira*) Kelley & Harris, 1983: 184 [type locality: [United States], South Carolina, Aiken County, Savannah River Plant, Upper Three Runs Creek at SRP 8-1; NMNH; ♂]. —Harris et al. 1991: 259 [distribution]. —Pescador et al. 2004: 134 [checklist]. —Harris et al. 2012: 11 [checklist]. —Denson et al. 2016: 6 [distribution].

**Distribution.** —U.S.A.

***scaeodactyla*** (*Dactylotrichia*) Kelley, 1983: 42 [type locality: Ecuador, Pastaza Prov., Puyo; NMNH; ♂]. —Kelley 1984a: 442 [checklist]. —Ríos-Touma et al. 2017: 11 [checklist].

**Distribution.** —Ecuador.

***scopulina*** (*Mesotrichia*) Flint & Sykora, 2004: 43 [type locality: Dominican Republic, Peravia Province, 3 km SW La Nuez, upper Rio Las Cuevas, 18°40'N, 70°36'W, 1850 m; CMNH; ♂; ♀]. —Pérez-Gelabert 2008: 301 [checklist].

**Distribution.** —Dominican Republic.

***scutica*** (*Pacificotrichia*) Kelley, 1989: 200 [type locality: New Caledonia, mountain stream up Boulari River; BPBM; ♂]. —Wells 1995: 233 [distribution]. —Wells and Johanson 2015: 70 [♂; distribution]. —Johanson and Wells 2019: 93 [checklist].

**Distribution.** —New Caledonia.

***sechellensis*** (unplaced) Malicky, 1993: 19 [type locality: [Seychelles], Mahé, Anse aux Pins; Collection Malicky; ♂]. —Malicky 2013: 214 [♂; checklist]. —Wells and de Moor 2020: 502 [♂; distribution].

**Distribution.** —Angola, Seychelles.

***sencilla*** (*Tanytrichia*) Holzenthal & Harris, 1992: 165 [type locality: Costa Rica, Alajuela, Río Pizote, 5 km N Dos Ríos, 10.948°N, 85.291°W; NMNH; ♂].

**Distribution.** —Costa Rica.

***septentrionalis*** (*Tanytrichia*) de Souza & Santos, 2017: 498 [type locality: Brazil, Piauí, Piracuruca, Parque Nacional de Sete Cidades, alojamento, 4°05'57"S, 41°42'34"W, 193 m; CZMA; ♂]. —Rocha et al. 2018: 153 [checklist]. —Moreno et al. 2020: 266 [distribution].

**Distribution.** —Brazil.

***serrata*** (*Holarctotrichia*) Ross, 1938a: 117 [type locality: [United States], Illinois, Fox Lake, at light in town; INHS; ♂; ♀]. —Ross 1944: 136 [♂; ♀; case; distribution]. —Denning 1947a: 148 [distribution]. —Denning 1947b: 171 [distribution]. —Ross and Spencer 1952: 46 [distribution]. —Morse and Blickle 1953: 72 [distribution]. —Etnier 1965: 148 [distribution]. —Roy and Harper 1975: 1082 [distribution]. —Etnier and Schuster 1979: 18 [distribution]. —Roy and Harper 1979: 152 [distribution]. —Blickle 1979: 54, 91 [checklist; ♂]. —Kelley and Morse 1982: 257, 267 [checklist; ♀]. —Waltz and McCafferty 1983a: 11 [distribution]. —Kelley 1984a: 438 [checklist]. —Harper 1989: 541 [distribution]. —Monson and Holzenthal 1993: 442 [checklist]. —Houghton et al. 2001: 505 [distribution]. —Houghton et al. 2011b: 6 [phenology; habitat; distribution]. —Blinn and Ruiter 2013: 291 [biology; distribution]. —Blinn and Ruiter 2013: 280 [biology; distribution]. —DeWalt et al. 2016: 52 [distribution]. —Houghton 2016: 46 [biology]. —Houghton et al. 2017: 63 [checklist].

**Distribution.** —Canada, U.S.A.

***setosa*** (*Oxyethira*) Denning, 1947a: 16 [type locality: [United States], Georgia, Macon; ESUW; ♂]. —Blickle 1979: 54, 91 [checklist; ♂]. —Kelley and Morse 1982: 257 [checklist]. —Harris et al. 1982b: 81 [distribution]. —Kelley 1984a: 438 [checklist]. —Kelley 1986: 777 [taxonomic position]. —Harris et al. 1991: 260 [distribution]. —Floyd et al. 1993: 91 [phenology; distribution]. —Pescador et al. 2004: 134 [checklist]. —Harris et al. 2012: 11 [checklist]. —Flint 2014: 90 [distribution]. —Denson et al. 2016: 6 [distribution].

**Distribution.** —U.S.A.

***shumari*** (*Oxyethira*) Ito & Oláh, 2017: 20 [type locality: [Japan], Hokkaido, Horokanai-cho, Shumarinai, Shumarinai-gawa River, a small tributary, 44°17'56"N, 142°09'31"E, 270 m; CBM-ZI; ♂; ♀].

**Distribution.** —Japan.

***sichuanensis*** (*Oxyethira*) Yang & Kelley in Yang et al. 1997: 92 [type locality: [China], Sichuan province, Nanpingxian, Jiouzhaigou, Shuzhengqunhai, 2250 m; NAUJ; ♂]. —Yang et al. 2005: 458 [checklist]. —Oláh and Ito 2013: 31 [♂]. —Yang et al. 2016: 477 [checklist].

**Distribution.** —China.

***sida*** (*Oxyethira*) Blickle & Morse, 1954: 122 [type locality: [United States], Lee, N. H.; INHS; ♂]. —Etnier 1965: 148 [distribution]. —Roy and Harper 1975: 1082 [distribution]. —Blickle 1979: 55, 97 [checklist; ♂]. —Roy and Harper 1979: 152 [checklist]. —Marshall and Larson 1982: 31 [distribution]. —Kelley 1984a: 437 [checklist]. —Masteller and Flint 1992: 70 [checklist]. —Monson and Holzenthal 1993: 442 [checklist]. —Huryn and Harris 2000: 193 [distribution]. —Houghton et al. 2001: 505 [distribution]. —Myers et al. 2011: 109 [distribution]. —Houghton et al. 2017: 63 [checklist].

**Distribution.** —Canada, U.S.A.

***sierruca*** (unplaced) Holzenthal & Harris, 1992: 165 [type locality: Costa Rica, Guanacaste, Quebrada Garcia, 10.6 km ENE Quebrada Grande, 10.862°N, 85.428°W; NMNH; ♂]. —Armitage et al. 2015b: 6 [distribution]. —Armitage et al. 2015a: 7 [checklist]. —Armitage and Harris 2018b: 98 [checklist]. —Armitage and Harris 2018c: 284 [distribution].

**Distribution.** —Costa Rica, Panama.

***simanka*** (unplaced) Oláh & Johanson, 2011: 135 [type locality: Ecuador, Wild Sumaco, near Pacto Sumaco; Collection Oláh; ♂]. —Ríos-Touma et al. 2017: 11 [checklist].

**Distribution.** —Ecuador.

***simplex*** (*Oxyethira*) Ris, 1897: 420 [type locality: [Switzerland]; ZMUA; ♂]. —Morton 1899a: 54 [distribution]. —Morton 1899b: 281 [distribution]. —Morton 1904: 328 [distribution]. —Mosely 1939b: 283 [♂]. —Macdonald 1950: 25 [larva]. —Kimmins 1958b: 13 [♀; distribution]. —Nybom 1960: 19 [checklist]. —Botosaneanu 1967: 294 [distribution]. —Solem 1970a: 2 [distribution]. —Botosaneanu and Malicky 1978: 340 [checklist]. —Moretti and Cianficconi 1981: 201 [checklist]. —Wallace et al. 1983: 168 [distribution]. —Malicky 1983b: 58, 60 [atlas; ♂; ♀]. —Kelley 1984a: 437 [checklist]. —Andersen and Tysse 1985: 84 [distribution]. —Andersen and Wiberg-Larsen 1987: 169 [checklist]. —Spuris 1989: 17 [checklist]. —Andersen et al. 1993b: 3 [distribution]. —Andersen et al. 1993a: 51 [distribution]. —Kahnert 1995: 124 [distribution]. —Maier et al. 1995: 145 [distribution]. —Cianficconi et al. 2002: 146 [distribution]. —Gullefors 2002: 138 [checklist]. —Malicky 2004a: 70, 72 [atlas]. —Malicky 2005b: 547 [checklist]. —Malicky 2005a: 68 [distribution]. —Botosaneanu 2005: 17 [distribution]. —Weinzierl et al. 2005: 48 [distribution]. —Lubini-Ferlin and Vicentini 2005: 68 [checklist]. —Chvojka and Komzák 2006: 358 [distribution]. —Robert 2007: 83 [checklist]. —Gullefors 2008: 64 [checklist]. —Chvojka and Komzák 2008: 13 [distribution]. —Ivanov 2011: 196 [checklist]. —Viidalepp et al. 2011: 195, 196 [distribution]. —Salokannel et al. 2012: 202 [confirmed as distinct species]. —Andersen and Hagenlund 2012: 136 [distribution]. —Lock et al. 2013: 22 [distribution]. —Chalkley 2014: 13 [distribution]. —O’Connor 2015: 28, 104 [distribution]. —Gullefors 2016: 156 [checklist]. —Wallace 2016: 18, 24 [conservation status]. —Graf et al. 2017: 48 [distribution]. —Valle and Lodovici 2018: 147 [distribution]. —Jacquemin et al. 2019: 99 [distribution; habitat description].

**Distribution.** —Austria, Belgium, Czech Republic, England, Estonia, Finland, France, Germany, Greece, Ireland, Italy, Netherlands, Norway, Russia, Scotland, Sweden, Switzerland.

***simulatrix*** (*Dampfitrichia*) Flint, 1968b: 43 [type locality: Jamaica, St. Andrew, Fresh River, Ferry; NMNH; ♂; ♀]. —Flint 1968a: 82 [checklist]. —Kelley 1984a: 439 [checklist]. —Holzenthal 1988: 63 [distribution]. —Holzenthal and Harris 1992: 174 [distribution]. —Flint 1996a: 16 [checklist]. —Botosaneanu and Hyslop 1998: 16 [distribution]. —Maes 1999: 1194 [checklist]. —Botosaneanu 2002b: 88 [checklist]. —Flint and Sykora 2004: 44 [distribution taxonomic remarks]. —Chamorro-Lacayo et al. 2007: 44 [checklist]. —Harris et al. 2012: 11 [♂; distribution]. —Armitage et al. 2015b: 6 [distribution]. —Armitage et al. 2015a: 7 [checklist]. —Armitage and Harris 2018b: 98 [checklist]. —Harris and Armitage 2019: 5 [distribution].

—*simulatrix cubana* Kumanski, 1987: 27 [type locality: Cuba, Provine Pinar del Rio, Rio El Ballio, near Isabel Rubio village, or Rio Esmeralda in the vicinity of Viñales; SOFM; ♂; ♀; as *Orthotrichia* sp.]. —Botosaneanu 1991: 130 [distribution; ♀; attributed is undoubtedly *tega*]. —Botosaneanu and Hyslop 1998: 16 [distribution]. —Flint and Pérez-Gelabert 1999: 41 [checklist]. —Botoseananu 2002: 88 [checklist]. —Flint and Sykora 2004: 44 [to synonymy]. —Botosaneanu and Thomas 2005: 55 [checklist]. —Naranjo López and González Lazo 2005: 149 [checklist]. —Pérez-Gelabert 2008: 301 [checklist].

—*mirebalina* Botosaneanu, 1991: 130, part [type locality: Haiti; ♀ allotype, misidentification of *O.simulatrix* sensu Flint and Sykora 2004].

**Distribution.** —Costa Rica, Cuba, Dominican Republic, Guadeloupe, Haiti, Jamaica, Mexico, Nicaragua, Panama, U.S.A.

***singularis*** (*Tanytrichia*) de Souza & Santos, 2017: 500 [type locality: Brazil, Bahia, Barreiras, cachoeira Acaba Vidas, 12°08'00"S, 44°59'00"W [approximate coordinates]; MZUFBA; ♂]. —Rocha et al. 2018: 153 [checklist].

**Distribution.** —Brazil.

***sininsigne*** (*Oxytrichia*) Kelley, 1981: 372 [type locality: [United States], Florida, Clay Co., Keystone Heights; NMNH; ♂; ♀]. —Kelley and Morse 1982: 257, 265 [checklist; ♀]. —Harris et al. 1982a: 512 [distribution]. —Kelley 1984a: 440 [checklist]. —Harris et al. 1991: 261 [distribution]. —Pescador et al. 2004: 134 [checklist]. —Harris et al. 2012: 12 [checklist]. —Denson et al. 2016: 6 [distribution].

**Distribution.** —U.S.A.

***sinistra*** (unplaced) Santos, Henriques-Oliveira, & Nessimian, 2009: 40 [type locality: Brazil, Amazonas, Manaus, Igarapé Arumã, tributary to Rio Cuieiras, 02°30'55.2"S, 60°15'44.4"W; INPA; ♂]. —Paprocki and França 2014: 53 [checklist]. —Rocha et al. 2018: 153 [checklist].

**Distribution.** —Brazil.

***smolpela*** Wells, 1991: 493 [type locality: Papua New Guinea, Central Province, Iomari Creek on Bereina-Port Morseby road, 9°04'S 147°06'E; ANIC; ♂; ♀].

**Distribution.** —Papua New Guinea.

***spicula*** (*Pacificotrichia*) Wells & Johanson, 2015: 72 [type locality: New Caledonia, Province Sud, Rivière des Lacs, 1.1 km NW Lac en Huit, 4.9 km NW summit of Pic du Grand Kaori, 22°15.195'S, 166°52.178'E; MNHN; ♂]. —Johanson and Wells 2019: 93 [checklist].

**Distribution.** —New Caledonia.

***spinifera*** (*Trichoglene*) Wells & Johanson, 2015: 42 [type locality: New Caledonia, small fall ~10 km SW Houailou on Houailou-Bourail road; MNHN; ♂]. —Johanson and Wells 2019: 93 [checklist].

**Distribution.** —New Caledonia.

***spinosella*** (*Oxyethira*) McLachlan, 1884: 72 [type locality: [Portugal], Madeira, Ribiera Fria near Faial, “levada” on cliff below Sant’ Anna; NHMUK; ♂]. —Morton 1893: 80 [♂]. —Nybom 1948: 9 [distribution]. —Kimmins 1957a: 108 [lectotype designation]. —Nybom 1965: 90 [distribution]. —Botosaneanu 1967: 294 [distribution]. —Botosaneanu and Malicky 1978: 340 [checklist]. —Malicky 1983b: 59 [atlas; ♂]. —Kelley 1984b: 188 [♂, ♀]. —Kelley 1984a: 437 [checklist]. —Malicky and Lounaci 1987: 15, 17 [checklist]. —Botosaneanu 1993b: 160 [distribution; ♀]. —Malicky 2004a: 71 [atlas]. —Malicky 2005b: 547 [checklist]. —Hughes 2006: 29 [biology].

—*fischeri* Higler, 1974: 62 [type locality: Madeira, Rio de Faial, 2 km W of Porto da Cruz; RMNH; ♂]. —Botosaneanu and Malicky 1978: 340 [checklist]. —Botosaneanu 1981b: 186 [♂; distribution]. —Kelley 1984b: 188 [to synonymy]. —Kelley 1984a: 437 [to synonymy].

—*gomera* Kelley, 1984b: 187 [type locality: Canary Islands, Gomera, Chejelipes; ZMUA; ♂]. —Botosaneanu 2003: 107 [to synonymy].

**Distribution.** —Portugal, Spain.

***spirogyrae*** (unplaced) Müller, 1879b: 48 [type locality: Brazil, Santa Catarina; no type nor type depository designated; case; larva; in *Lagenopsyche*; ♂]. —Müller 1879b: 339 [to *Oxyethira*]. —Ulmer 1957: 172 [bibliography]. —Kelley 1984a: 442 [checklist]. —Angrisano 1999: 35 [checklist]. —Paprocki et al. 2004: 12 [checklist]. —Santos et al. 2009: 37 [checklist]. —Paprocki and França 2014: 53 [checklist]. —Rocha et al. 2018: 153 [checklist].

**Distribution.** —Brazil.

***spissa*** (unplaced) Kelley, 1983: 48 [type locality: brazil, Pará State, Rio Cururu, area of Missao Cururu; NMNH; ♂]. —Kelley 1984a: 442 [checklist]. —Angrisano 1999: 35 [checklist]. —Paprocki et al. 2004: 12 [checklist]. —Santos et al. 2009: 37 [checklist]. —Paprocki and França 2014: 53 [checklist]. —Rocha et al. 2018: 152 [checklist]. —Moreno et al. 2020: 266 [distribution].

**Distribution.** —Brazil.

***tamandua*** (unplaced) Angrisano & Sganga, 2009: 65 [type locality: Argentina, Misiones, Parque Provincial Salto Encantado; MACN; ♂].

**Distribution.** —Argentina.

***tamperensis*** (*Oxyethira*) Malicky, 1999d: 44 [type locality: Finland, Tampere; Collection Malicky; ♂]. —Malicky 2004a: 72 [atlas]. —Malicky 2005b: 547 [checklist]. —Tobias et al. 2009: 25 [♀]. —Salokannel et al. 2012: 202 [confirmed as distinct species].

**Distribution.** —Finland.

***tasmaniensis*** (*Trichoglene*) Wells, 1998: 83 [type locality: Australia, Tasmanian World Heritage Area, Southwest National Park, Melaleuca, 43°25'10"S, 146°08'46"E; ANIC; ♂]. —Neboiss 2002: 54 [checklist]. —Wells 2002b: 40 [distribution].

**Distribution.** —Australia.

***tega antillularum*** (*Dampfitrichia*) Botosaneanu, 1994a: 41 [type locality: Guadeloupe, rivière St-Louis dans les Hauts du Matouba; ZMUA; ♂]. —Flint and Sykora 1993: 57 [distribution, as *tega*]. —Botosaneanu 2002b: 88 [checklist]. —Botosaneanu and Thomas 2005: 55 [checklist].

**Distribution.** —Dominica, Guadeloupe.

***tega tega*** (*Dampfitrichia*) Flint, 1968b: 44 [type locality: Jamaica, Trelawny, Martha Brae, near Falmouth; NMNH; ♂; ♀]. —Flint 1968a: 56 [distribution]. —Botosaneanu 1977: 273 [variability; distribution]. —Botosaneanu 1979: 50 [distribution]. —Malicky 1983c: 264 [distribution]. —Kelley 1984a: 440 [checklist]. —Flint 1996a: 16 [checklist]. —Botosaneanu and Hyslop 1998: 16 [distribution]. —Botosaneanu 2002b: 88 [checklist]. —Flint and Sykora 2004: 45 [distribution; taxonomic remarks]. —Naranjo López and González Lazo 2005: 149 [checklist]. —Pérez-Gelabert 2008: 301 [checklist].

**Distribution.** —Cuba, Jamaica, Dominica, Haiti, Hispaniola.

***teixeirai*** (*Tanytrichia*) Harris & Davenport, 1992: 470 [type locality: Peru, Loreto, small tributary to the Rio Sucusari at Explornapo Camp; NMNH; ♂].

**Distribution.** —Peru.

***tenei*** (unplaced) Gibon, Guenda, & Coulibaly, 1994: 111 [type locality: Burkina Faso, sur le Téné (bassin du Sénégal, Fouta-Djalon, Guinée; MNHN; ♂].

**Distribution.** —Guinea.

***tenuella*** (*Oxyethira*) Martynov, 1924: 54 [type locality: [Russia]; depository not designated; ♂]. —Martynov 1934: 1450 [♂]. —Botosaneanu 1967: 294 [distribution]. —Botosaneanu and Malicky 1978: 340 [checklist]. —Malicky 1983b: 59 [atlas; ♂]. —Kelley 1984a: 437 [checklist]. —Spuris 1989: 17 [checklist]. —Malicky 2004a: 71 [atlas]. —Malicky 2005b: 547 [checklist]. —Ivanov 2011: 196 [checklist]. —Viidalepp et al. 2011: 196 [distribution]. —Pan’kov and Krasheninnikov 2016: 333 [distribution]. —Salokannel and Mattila 2018: 362 [♀].

**Distribution.** —Estonia, Russia.

***tica*** (*Loxotrichia*) Holzenthal & Harris, 1992: 168 [type locality: Costa Rica, Guanacaste, Parque Nacional Santa Rosa, Quebrada El Duende near La Casona, 10.838°N, 85.614°W; NMNH; ♂; ♀]. —Botosaneanu and Sakal 1992: 202 [distribution; ecology]. —Botosaneanu and Alkins-Koo 1993: 27 [♂; distribution]. —Botosaneanu 1994a: 43 [distribution]. —Flint 1996b: 98 [distribution]. —Botosaneanu 2000: 256 [distribution]. —Botosaneanu 2002b: 88 [checklist]. —Blahnik Paprocki and Holzenthal 2004: 5 [distribution]. —Paprocki et al. 2004: 12 [checklist]. —Botosaneanu and Thomas 2005: 44 [distribution]. —Chamorro-Lacayo et al. 2007: 44 [checklist]. —Dumas et al. 2009: 366 [♂; distribution]. —Santos et al. 2009: 43 [distribution]. Oláh and Johanson 2011: 137 [distribution]. —Dumas and Nessimian 2012: 15 [checklist]. —Paprocki and França 2014: 53 [checklist]. —Armitage et al. 2015a: 7 [checklist]. —de Souza and Santos 2017: 504 [distribution]. —Ríos-Touma et al. 2017: 11 [checklist]. —Rocha et al. 2018: 153 [checklist]. —Armitage and Harris 2018b: 98 [checklist]. —Harris and Armitage 2019: 5 [distribution]. —Moreno et al. 2020: 266 [distribution].

**Distribution.** —Brazil, Costa Rica, Dominica, Ecuador, French Guiana, Grenada, Guadeloupe, Honduras, Martinique, Mexico, Nicaragua, Panama, St. Lucia, St. Vincent, Trinidad, Venezuela.

***tiwaka*** (*Trichoglene*) Wells & Johanson, 2015: 44 [type locality: New Caledonia, Province Nord, Bouérabate Stream, S Mont Ninndo, along road Barabache-Boulagoma, 20°17.409'S, 164°11.242'E, 60 m; MNHN; ♂]. —Johanson and Wells 2019: 93 [checklist].

**Distribution.** —New Caledonia.

***tiunovae*** (*Oxyethira*) Arefina & Armitage, 2003: 16 [type locality: [Russia] Khabarovsk Territory, Ussuri River Basin, Kiya River at Ekaterinoslavka Village; IBSS-RAS; ♂; ♀]. —Ivanov 2011: 196 [checklist]. —Oláh and Ito 2013: 40 [♂; distribution].

**Distribution.** —Russia.

***torquata*** (*Trichoglene*) Wells, 2002b: 39 [type locality: Australia, Tasmania, McPartlan Pass, Site 8A; ANIC; ♂]. —Neboiss 2002: 54 [checklist].

**Distribution.** —Australia.

***torresiana*** (*Dampfitrichia*) Wells & Dostine, 2016: 594 [type locality: [Australia] North East Queensland, Sesia via Bamaga; ANIC; ♂].

**Distribution.** —Australia.

***torza*** (unplaced) Oláh & Johanson, 2011: 137 [type locality: French Guiana, Roura, Cacao, 4°33.639'N, 52°24.629'W, 66 m; NHRS; ♂].

**Distribution.** —French Guiana.

***touba*** (*Dampfitrichia*) Gibon, 1987a: 123 [type locality: sur le Nzo au niveau de la route Man/Danané (bassin du Sassandra, Côte d’Ivoire); MNHN; ♂].

**Distribution.** —Côte d’Ivoire.

***triangulata*** (*Trichoglene*) Wells, 1981: 108 [type locality: [Australia] Queensland, Crystal Creek, nr turnoff to Mt Spec; NMV; ♂; ♀]. —Kelley 1984a: 436 [checklist]. —Neboiss 1986: 83 [atlas; ♂; ♀]. —Wells and Dostine 2016: 597 [distribution].

**Distribution.** —Australia.

***tristella*** (*Oxyethira*) Klapálek, 1895: 168 [type locality: [Czech Republic], on the “Zlata Stoka” in Trēbon, Bohemia; no depository designated; ♂]. —Klapálek 1897: 11 [larva]. —Morton 1899b: 281 [distribution]. —Morton 1904: 327 [distribution]. —Martynov 1924: 53 [♂]. —Martynov 1934: 153 [♂]. —Mosely 1939b: 283 [♂]. —Kimmins 1958b: 9 [♀; distribution]. —Nybom 1960: 19 [checklist]. —Botosaneanu 1967: 294 [distribution]. —Solem 1970b: 93 [distribution]. —Spuris 1972: 20, 22, 26 [checklist]. —Botosaneanu and Malicky 1978: 340 [checklist]. —Malicky 1983b: 58, 60 [atlas; ♂; ♀]. —Kelley 1984a: 437 [checklist]. —Wiberg-Larsen 1985: 40 [checklist]. —Andersen and Tysse 1985: 84 [distribution; as *trictella*]. —Andersen and Wiberg-Larsen 1987: 169 [checklist]. —Spuris 1989: 17 [checklist]. —Andersen et al. 1993a: 51 [distribution]. —Dorn et al. 1993: 259 [distribution]. —Nógrádi 1994: 271 [distribution; ♂; ♀]. —Uherkovich and Nógrádi 1997: 461 [distribution]. —Uherkovich and Nógrádi 1998: 52 [distribution]. —Uherkovich and Nógrádi 2001: 95 [distribution]. —Nógrádi 2001: 85 [distribution]. —Wiberg-Larsen and Czachorowski 2002: 151 [distribution]. —Gullefors 2002: 138 [checklist]. —Cibaitė 2003a: 10 [checklist]. —Gullefors 2003: 194 [distribution]. —Malicky 2004a: 70, 72 [atlas]. —Graf and Hutter 2004: 147 [distribution]. —Hohmann 2005: 106 [checklist]. —Mey 2005b: 119 [distribution]. —Gullefors 2005a: 118 [distribution]. —Gullefors 2005b: 138 [distribution]. —Malicky 2005b: 547 [checklist]. —Graf et al. 2005: 55 [distribution]. —Gullefors 2006: 137 [distribution]. —Berlin and Thiele 2007: 48, 50 [distribution; checklist]. —Robert 2007: 83 [checklist]. —Chvojka and Komzák 2008: 13 [distribution]. —Višinskienė 2009: 28 [checklist]. —Ivanov 2011: 196 [checklist]. —Viidalepp et al. 2011: 196 [distribution]. —Salokannel et al. 2012: 202 [confirmed as distinct species]. —Hohmann et al. 2014: 85 [distribution]. —O’Connor 2015: 28, 105 [distribution]. —Gullefors 2016: 156 [checklist]. —Küttner et al. 2016: 179 [distribution]. —Wallace 2016: 21, 23, 69 [conservation status]. —Graf et al. 2017: 48 [distribution]. —Komzák and Kroča 2018: 168 [distribution]. —Cerjanec et al. 2020: 13 [distribution].

**Distribution.** —Austria, Croatia, Czech Republic, Denmark, Estonia, Finland, Germany, Hungary, Ireland, Norway, Poland, Russia, Scotland, Sweden.

***tropis*** (*Oxyethira*) Yang & Kelley in Yang et al. 1997: 95 [type locality: [China], Sichuan province, Jiangjinxian, Simianshan, Dam of Dahonghai, 1000 m; NAUJ; ♂]. —Yang et al. 2005: 458 [checklist]. —Yang et al. 2016: 477 [checklist].

**Distribution.** —China.

***tsuruga*** (*Oxyethira*) Ito & Oláh, 2017: 19 [type locality: [Japan], Honshu, Fukui, Tsuruga-shi, Ikenokôchi Marsh, 35°40'n, 136°08'E, 300 m; CBM-ZI; ♂; ♀].

**Distribution.** —Japan.

***tuveva*** (*Tanytrichia*) Oláh & Johanson, 2011: 138 [type locality: French Guiana, Approuaguekaw, Kaw Mt, 4°32.833'N, 52°11.452'W, 77 m; NHRS; ♂].

**Distribution.** —French Guiana.

***ulmeri*** (*Dampfitrichia*) (Mosely, 1937b): 169 [type locality: Mexico, Chiapas, Dolores; NHMUK; ♂; in *Dampfitrichia*]. —Kelley 1984a: 439 [checklist]. —Bueno-Soria and Flint 1978: 205 [distribution]. —Blickle 1979: 55, 91 [checklist; ♂]. —Kelley and Morse 1982: 257, 267 [checklist; ♀]. —Angrisano 1995a: 510 [distribution]. —Abbott et al. 1997: 44 [distribution]. —Moulton and Stewart 1997: 351 [checklist]. —Angrisano 1999: 34 [distribution]. —Bowles et al. 2007: 22 [distribution; biology]. —Bueno-Soria et al. 2007: 33 [distribution]. —Rueda Martín 2011: 9 [♂; distribution]. —Isa Miranda and Rueda Martín 2014: 199 [distribution].

**Distribution.** —Argentina, Mexico, Uruguay, U.S.A.

***una*** (*Tanytrichia*) de Souza & Santos, 2017: 500 [type locality: Brazil, Bahia, Una, Reserva Biológica de Una riacho de 1^a^ ordem após a fazenda Piedade, 15°09'36"S, 39°10'31"W; DZRJ; ♂]. —Rocha et al. 2018: 153 [checklist].

**Distribution.** —Brazil.

***unidentata*** (*Oxyethira*) McLachlan, 1884: 73 [type locality: Portugal, streamlet west of Silves, Alrgarve; NHMUK; ♂]. —Morton 1893: 80 [♂]. —Kimmins 1957a: 108 [lectotype designation]. —Botosaneanu 1967: 294 [distribution]. —Botosaneanu and Malicky 1978: 340 [checklist]. —Malicky 1980a: 16 [checklist]. —Moretti and Cianficconi 1981: 201 [checklist]. —Malicky 1983b: 58 [atlas; ♂]. —Kelley 1984a: 437 [checklist]. —Malicky and Lounaci 1987: 14 [checklist]. —Malicky and Lounaci 1987: 15, 17 [checklist]. —González et al. 1990: 212 [checklist]. —Cianficconi et al. 1999b: 278 [distribution]. —Malicky 2004a: 70 [atlas]. —Malicky 2005b: 547 [checklist]. —Ruiz-García et al. 2006: 77 [distribution]. —Corallini and Cianficconi 2011: 628 [checklist]. —González and Mendéndez 2011: 119 [distribution]. —Martín et al. 2015: 74 [distribution]. —Sekhi et al. 2016: 58 [distribution]. —Ruiz-García et al. 2016: 4 [distribution]. —Valle and Lodovici 2018: 147 [distribution]. —Mabrouki et al. 2020: 14 [distribution].

—*fuentejalona* Schmid, 1952: 656 [type locality: Spain; CNC; ♂]. —Botosaneanu 1967: 294 [as synonym]. —Marshall 1979b: 232 [to synonymy]. —Lonsdale 2020: 34 [holotype depository].

—*meridionalis* Jacquemart & Coineau, 1962: 16 [type locality: [France], les Pyrenees orientales, des Albères; depository not designated; ♂; larva]. —Botosaneanu 1967: 294 [as synonym]. —Marshall 1979b: 232 [to synonymy].

**Distribution.** —Algeria, France, Italy, Morocco, Portugal, Spain.

***unispina*** (*Oxytrichia*) Flint, 1974b: 67 [type locality: Suriname, Republiek; RMNH; ♂]. —Kelley 1984a: 440 [checklist].

**Distribution.** —Suriname.

***vaina*** (unplaced) Harris & Davenport, 1999: 33 [type locality: Peru, Loreto, edge of Rio Sucusari backwater, adjoining Explornapo Camp; NMNH; ♂].

**Distribution.** —Peru.

***vaza*** (*Dampfitrichia*) Oláh & Johanson, 2011: 140 [type locality: French Guiana, Roura, Cacao, 4°33.639'N, 52°24.629'W, 66 m; NHRS; ♂].

**Distribution.** —French Guiana.

***velocipes*** (*Argyrobothrus*) (Barnard, 1934): 393 [type locality: [South Africa]; holotype not designated; depository not designated; ♂; ♀; pupa; in *Argyrobothrus*]. —Scott 1963: 476 [distribution; larva; pupa]. —Jacquemart 1963a: 410 [distribution]. —Kelley 1984a: 438 [checklist]. —de Moor 2011: 354 [distribution]. —de Moor and Bellingan 2019: 157 [distribution].

**Distribution.** —South Africa.

***verna*** (*Dampfitrichia*) Ross, 1938a: 118 [type locality: [United States], Illinois, Spring Grove; INHS; ♂]. —Ross 1944: 139 [♂; distribution]. —Denning 1947a: 17 [distribution; ♀]. —Denning 1947b: 171 [distribution]. —Blickle 1979: 55, 95 [checklist; ♂]. —Kelley and Morse 1982: 257, 266 [checklist; ♀]. —Harris et al. 1982a: 512 [distribution]. —Kelley 1984a: 440 [checklist]. —Harris et al. 1991: 262 [distribution]. —Monson and Holzenthal 1993: 442 [distribution]. —Abbott et al. 1997: 44 [distribution]. —Moulton and Stewart 1997: 351 [checklist]. —Houghton et al. 2001: 505 [distribution]. —Pescador et al. 2004: 134 [distribution]. —Harris et al. 2012: 12 [distribution]. —Houghton et al. 2017: 63 [checklist].

**Distribution.** —U.S.A.

***vipera*** (*Oxytrichia*) Kelley, 1983: 50 [type locality: Chile, Valdavia Prov., S of Valdavia; NMNH; ♂]. —Kelley 1984a: 440 [checklist]. —Angrisano 1999: 34 [checklist]. —Oláh and Johanson 2011: 141 [distribution].

**Distribution.** —Chile.

***volsella*** (*Oxyethira*) Yang & Kelley in Yang et al. 1997: 97 [type locality: [China], SFujian Province, Conganshi, 29 km north of Congan, at 408 km marker; NAUJ; ♂]. —Yang et al. 2005: 458 [checklist]. —Yang et al. 2016: 477 [checklist].

**Distribution.** —China.

***waipoua*** (*Trichoglene*) Wise, 1998: 18 [type locality: [New Zealand], Waipoua Forest, Waipoua R. bridge, swept at edge of river, ca. 100 m, O06 624 164; AMNZ; ♂; ♀]. —Ward and Henderson 2004: 10 [checklist].

**Distribution.** —New Zealand.

***warramunga*** (*Dampfitrichia*) Wells, 1985a: 99 [type locality: Australia, Northern Territory, Georgetown Billabong, nr Jabiru; NTM; ♂; ♀]. —Neboiss 1986: 82 [atlas; ♂; ♀]. —Wells et al. 2019: 33 [detection frequency].

**Distribution.** —Australia.

***zeronia*** (*Argyrobothrus*) Ross, 1941b: 15 [type locality: [United States], Twin Lake, Houghton Co., Michigan; INHS; ♂]. —Ross 1944: 139 [♂; distribution]. —Morse and Blickle 1953: 72 [checklist]. —Etnier 1968: 191 [distribution]. —Etnier and Schuster 1979: 18 [distribution]. —Blickle 1979: 55, 93 [checklist; ♂]. —Roy and Harper 1979: 152 [checklist]. —Parker and Voshell 1981: 4 [checklist]. —Roy and Harper 1981: 105 [distribution]. —Kelley and Morse 1982: 257, 266 [checklist; ♀]. —Harris et al. 1982a: 512 [distribution]. —Harris et al. 1982b: 81 [distribution]. —Huryn and Foote 1983: 791 [distribution]. —Hamilton et al. 1983: 19 [distribution]. —Kelley 1984a: 438 [checklist]. —Harris et al. 1984: 109 [distribution]. —Harper 1989: 541 [distribution]. —Morse et al. 1989: 24 [distribution]. —Bowles and Mathis 1989: 240 [distribution]. —Floyd and Schuster 1990: 130, 132 [distribution]. —Frazer et al. 1991: 20 [distribution]. —Harris et al. 1991: 263 [distribution]. —Masteller and Flint 1992: 70 [checklist]. —Mathis and Bowles 1992: 24 [distribution]. —Bowles and Mathis 1992: 32 [distribution]. —Floyd et al. 1993: 91 [phenology; distribution]. —Monson and Holzenthal 1993: 442 [checklist]. —Moulton and Stewart 1996: 129 [♂; distribution]. —Abbott et al. 1997: 44 [distribution]. —Moulton and Stewart 1997: 351 [checklist]. —Houghton et al. 2001: 505 [distribution]. —Pescador et al. 2004: 134 [checklist]. —Zeullig et al. 2006: 43 [distribution]. —Etnier 2010: 486 [distribution]. —Biondi 2010: 61 [distribution]. —Armitage et al. 2011: 14 [checklist]. —Houghton et al. 2011b: 6 [phenology habitat]. —Myers et al. 2011: 109 [distribution]. —Harris et al. 2012: 12 [checklist]. —Blinn and Ruiter 2013: 291 [biology; distribution]. —Denson et al. 2016: 6 [distribution]. —Houghton 2016: 46 [biology]. —Hunt 2017: 108 [distribution]. —Houghton et al. 2017: 63 [checklist]. —Bowles et al. 2020: 8 [distribution].

—*walteri* Denning, 1947a: 17 [type locality: [United States], Florida, Miami; ESUW; ♂]. —Blickle 1979: 55 [to synonymy].

**Distribution.** —Canada, U.S.A.

***zilaba*** (*Loxotrichia*) (Mosely), 1939a: 238 [type locality: Brazil, Edo. Santa Catarina, Nova Teutonia; NHMUK; ♂in *Loxotrichia*]. —Kelley 1984a: 442 [checklist]. —Angrisano 1995a: 510 [distribution]. —Angrisano 1999: 34 [checklist]. —Blahnik et al. 2004: 5 [distribution]. —Paprocki et al. 2004: 12 [checklist]. —Angrisano and Sganga 2007: 36 [♂; distribution]. —Santos et al. 2009: 37 [checklist]. —Calor 2011: 321 [checklist]. —Paprocki and França 2014: 54 [checklist]. —Rocha et al. 2018: 153 [checklist].

**Distribution.** —Argentina, Brazil, Paraguay, Uruguay.

#### Genus *Paroxyethira* Mosely, 1924

*Paroxyethira* Mosely, 1924: 670 [type species: *Paroxyethirahendersoni* Mosely, 1924, subsequent designation by Mosely and Kimmins 1953: 515]. —Leader 1972: 195; 198 [comparison with *Oxyethiraalbiceps*; key to New Zealand species]. —Marshall 1979b: 208 [generic review]. —Ward and Henderson 2004: 12 [re-diagnosis of male adults]. —Wells and Johanson 2012: 331 [key to New Caledonian species].

*Paroxyethira* consists of 25 species recorded from New Zealand, New Caledonia, Vanuatu, and Fiji. Marshall (1979b) considered the genus to be closely related to *Xuthotrichia*, based on similarities of the adult head and thorax, the female genitalia, and larval morphology and habits. Generalized figures of *Paroxyethira* larvae were given by Leader (1968), with a more detailed description given later (Leader 1972).

***anomala*** Wells & Johanson, 2012: 340 [type locality: [New Caledonia], Province Sud, Monts Kwa Ne Mwa, on road between Noumea and Yaté, Rivière des Pirogues, 22°11.225'S, 166°43.338'E, 100 m; MNHN; ♂; ♀]. —Johanson and Wells 2019: 93 [checklist].

**Distribution.** —New Caledonia.

***asymmetrica*** Wells & Johanson, 2012: 334 [type locality: [New Caledonia], Province Sud, Xwé Premöu Stream, 300 m N bridge over Dathio River at Atè, 6.2 km WNW Thio, 21.58835°S, 166.15117°E, 13 m; MNHN; ♂; ♀]. —Johanson and Wells 2019: 93 [checklist].

**Distribution.** —New Caledonia.

***atypica*** Wells & Johanson, 2012: 335 [type locality: [New Caledonia], Province Sud, Mt. Dzumac, source stream of Ouinne River, downstream crosspoint to mountain track, 22°01.997'S, 166°28.486'E, 795 m, over about 30 m waterfall; MNHN; ♂; ♀]. —Johanson and Wells 2019: 93 [checklist].

**Distribution.** —New Caledonia.

***auldorum*** Ward & Henderson, 2004: 12 [type locality: [New Zealand], MB Wakamarina River tributary above tunnel, 25633, 58939, 40 m; CMNZ; ♂].

**Distribution.** —New Zealand.

***dumagnes*** Kelley, 1989: 201 [type locality: New Caledonia, Boulari River; BPBM; ♂]. —Wells 1995: 234 [distribution]. —Oláh and Johanson 2010a: 35 [distribution]. —Wells and Johanson 2012: 333 [♂; ♀]. —Johanson and Wells 2019: 93 [checklist].

**Distribution.** —New Caledonia.

***dunedensis*** Ward & Henderson, 2004: 14 [type locality: [New Zealand], CO Rock and Pillar Range, north end, 22877, 55384, 1190 m; CMNZ; ♂].

**Distribution.** —New Zealand.

***dzumac*** Wells & Johanson, 2012: 337 [type locality: [New Caledonia], Province Sud, Mt. Dzumac, source stream of Ouinne River, downstream crosspoint to mountain track, 22°01.997'S, 166°28.486'E, 795 m, over ca. 30 m waterfall; MNHN; ♂; ♀]. —Johanson and Wells 2019: 93 [checklist].

**Distribution.** —New Caledonia.

***eatoni*** Mosely, 1924: 673 [type locality: New Zealand, South Island, Mackenzie County, River Tekapo; NHMUK; ♂; ♀]. —Mosely and Kimmins 1953: 518 [♂]. —Leader 1972: 197 [♀]. —Neboiss 1986: 80 [atlas; ♂; ♀]. —Ward and Henderson 2004: 11 [checklist].

**Distribution.** —New Zealand.

***hamata*** Wells & Johanson, 2012: 338 [type locality: [New Caledonia], Province Sud, Mt. Dzumac, source stream of Ouinne River, downstream crosspoint to mountain track, 22°01.997'S, 166°28.486'E, 795 m, over about 30 m waterfall; MNHN; ♂]. —Johanson and Wells 2019: 94 [checklist].

**Distribution.** —New Caledonia.

***hendersoni*** Mosely, 1924: 673 [type locality: New Zealand, South Island; NHMUK; ♂; ♀]. —Mosely and Kimmins 1953: 515 [♂]. —Leader 1970: 122 [larva]. —Leader 1972: 197 [♀; pupal case; larval leg]. —Cowley 1978: 674 [distribution; larva]. —Neboiss 1986: 80 [atlas; ♂; ♀]. —Ward and Henderson 2004: 11 [checklist]. —Oláh and Johanson 2010a: 36 [distribution].

**Distribution.** —New Zealand.

***hintoni*** Leader, 1972: 194 [type locality: New Zealand, North Island, Taranaki, Mount Egmont, Te Popo Stream; DSIR, transferred to NZAC; ♂; ♀]. —Neboiss 1986: 80 [atlas; ♂; ♀]. —Ward and Henderson 2004: 11 [checklist].

**Distribution.** —New Zealand.

***hughwilsoni*** Ward & Henderson, 2004: 14 [type locality: [New Zealand], MC Narbey Stream above Otanerito, Hinewai Reserve, 25138, 57047, 40 m; CMNZ; ♂].

**Distribution.** —New Zealand.

***kimminsi*** Leader, 1972: 191 [type locality: New Zealand, Swanson, near Auckland, Cascades Stream; DSIR, transferred to NZAC; ♂; ♀]. —Towns 1981: 195 [distribution]. —Neboiss 1986: 81 [atlas; ♂; ♀]. —Ward and Henderson 2004: 11 [checklist].

**Distribution.** —New Zealand.

***koegi*** Wells & Johanson, 2012: 333 [type locality: [New Caledonia], Province Nord, Ponandou Tiôgé River at Kögi, 3.9 km SSW Touho, 20°49.043'S, 165°13.551'E, 25 m; MNHN; ♂]. —Johanson and Wells 2019: 94 [checklist].

**Distribution.** —New Caledonia.

***manapouri*** Ward & Henderson, 2004: 15 [type locality: [New Zealand], FD Wolfe Flat, Turret Range; NZAC; ♂].

**Distribution.** —New Zealand.

***nigrispina*** Kelley, 1989: 202 [type locality: New Caledonia, Boulari River; BPBM; ♂]. —Wells and Johanson 2012: 340 [♂; distribution]. —Johanson and Wells 2019: 94 [checklist].

**Distribution.** —New Caledonia.

***opposita*** Wells & Johanson, 2012: 335 [type locality: [New Caledonia], Province Nord, Plaine des Gaïacs, Rivière Rouge, 14.2 km NW summit of Mt. Rouge, 50 m upstream road RT1 Noumea-Koné, 20°31.573'S, 164°46.609'E, 23 m; MNHN; ♂]. —Johanson and Wells 2019: 94 [checklist].

**Distribution.** —New Caledonia.

***pounamu*** Ward & Henderson, 2004: 15 [type locality: [New Zealand], FD Borland Burn tributary near Borland Lodge, 20855, 54787, 170 m; CMNZ; ♂].

**Distribution.** —New Zealand.

***ramifera*** Ward & Henderson, 2004: 15 [type locality: [New Zealand], ND Waipoua, Fire Lookout Track, stream, 25614, 66162, 140 m; CMNZ; ♂].

**Distribution.** —New Zealand.

***sarae*** Ward & Henderson, 2004: 14 [type locality: [New Zealand], BR Sabine River, above gorge, 24853, 59202, 520 m; Collection Henderson; ♂].

**Distribution.** —New Zealand.

***serrata*** Wells & Johanson, 2012: 339 [type locality: [New Caledonia], Province Nord, Mt. Panié, stream at camp, 20.58167°S, 164.76472°E, 1311 m; MNHN; ♂; ♀]. —Johanson and Wells 2019: 94 [checklist].

**Distribution.** —New Caledonia.

***takitimu*** Ward & Henderson, 2004: 17 [type locality: [New Zealand], BR Slab Hut Creek access road, swampy areas, 24100, 58940, 190 m; CMNZ; ♂].

**Distribution.** —New Zealand.

***teika*** Ward & Henderson, 2004: 15 [type locality: [New Zealand], WN Catchpool Campground, 26712, 59823, 50 m; CMNZ; ♂].

**Distribution.** —New Zealand.

***tillyardi*** Mosely, 1924: 670 [type locality: New Zealand, North Island, Tarawera; DSIR, transferred to NZAC; ♂; ♀]. —Mosely and Kimmins 1953: 518 [♂]. —Leader 1970: 122 [larva]. —Leader 1972: 197 [♀]. —Neboiss 1986: 81 [atlas; ♂; ♀]. —Ward and Henderson 2004: 11 [checklist].

**Distribution.** —New Zealand.

***zoae*** Ward & Henderson, 2004: 15 [type locality: [New Zealand], TO mount Ruahepu near Tawhai Falls, 27269, 62220, 980 m; Collection Henderson; ♂].

**Distribution.** —New Zealand.

#### Genus *Paucicalcaria* Mathis & Bowles, 1989

*Paucicalcaria* Mathis & Bowles, 1989: 187 [type species: *Paucicalcariaozarkensis* Mathis & Bowles, 1989, original designation].

The monotypic genus *Paucicalcaria* has been recorded only from Magazine Mountain in Arkansas, USA. Based on similarities of the genitalia and thoracic nota and the lack of ocelli, Mathis and Bowles (1989) placed it as most closely related to *Hydroptila*. The genus can be distinguished from all other hydroptilids by its unique tarsal formula (0, 1, 2) (Mathis and Bowles 1989). The larval stage is unknown.

***ozarkensis*** Mathis & Bowles, 1989: 188 [type locality: Arkansas, Logan Co., Gutter Rock Creek at low-water bridge on road to Green Bench, 35°11'46"N 93°39'46"W, 396 m; NMNH; ♂]. —Moulton and Stewart 1996: 130 [♂; distribution]. —Etnier 2010: 486 [distribution].

**Distribution.** —U.S.A.

#### Genus *Sutheptila* Malicky & Chantaramongkol, 2007

*Sutheptila* Malicky & Chantaramongkol, 2007: 1024 [type species: *Sutheptilakjaerandseni* Malicky & Chantaramongkol, 2007, original designation.]

The monotypic genus *Sutheptila* is recorded from Thailand. Malicky and Chantaramongkol (2007) placed the genus in Hydroptilinae based on the absence of the transverse suture of the mesoscutellum. They also commented that the general form of the male genitalia is similar to that of *Microptila*, but that the phallus differs noticeably (Malicky and Chantaramongkol 2007). The larval stage is unknown.

***kjaerandseni*** Malicky & Chantaramongkol, 2007: 1024 [type locality: Thailand, Doi Suthep NP, bei Tempel, 18°48'N 98°55'E, 1200 m; Collection Malicky; ♂]. —Malicky 2010a: 37 [atlas; ♂].

**Distribution.** —Thailand.

#### Genus *Tangatrichia* Wells & Andersen, 1995

*Tangatrichia* Wells & Andersen, 1995: 161 [type species: *Tangatrichiagracilenta* Wells & Andersen, 1995, original designation].

The monotypic genus *Tangatrichia*, occurring in Tanzania, shares similarities in the wings and form of the male genitalia with members of Stactobiinae (Wells and Andersen 1995). However, it has been placed within Hydroptilinae based on the presence of ocelli and the basic structure of the male genitalia; it shares similarities with both *Hydroptila* and *Jabitrichia* (Wells and Andersen 1995). The larval stage is unknown.

***gracilenta*** Wells & Andersen, 1995: 162 [type locality: Tanzania, Tanga region, West Usambara Mts, Mazumbai, Kaputu Stream, loc. 5, 1650 m a.s.l.; ZMUB; ♂].

**Distribution.** —Tanzania.

#### Genus *Tricholeiochiton* Kloet & Hincks, 1944

*Tricholeiochiton* Kloet & Hincks, 1944: 97 [type species: *Leiochitonfagesii* Guinard, 1879: 19, monotypic; replacement name for *Leiochiton* Guinard, 1879, preoccupied by *Leiochiton* Curtis, 1831, in Coleoptera]. —Marshall 1979b: 210 [generic review]. —Wells 1982: 252 [revision; key to Australian species]. —Wells 1985b: 18 [larva; pupa; case]. —Wells 1997: 1–28 [checklist; larvae]. —Wells 1998: 81 [distribution]. —Kachalova in Medvedev 1998: 189 [key to the species of the European part of the USSR].

*Synagotrichia* Ulmer, 1951: 81 [type species: *Synagotrichiafortensis* Ulmer, 1951, original designation and monotypic]. —Marshall 1979b: 210 [to synonymy].

*Tricholeiochiton* includes ten species occurring in Europe, Southeast Asia, Australia, and South America. The genus is most likely closely related to *Oxyethira*, based on the general form of the larvae and features of the adult head and thorax (Marshall 1979b). The larvae of *T.fagesii* have been described by both Lepneva (1970) and Wells (1985b).

***bifurca*** Wells, 1982: 256 [type locality: Western Australia, Mitchell Plateau, Camp Creek; NMV; ♂; ♀]. —Neboiss 1986: 75 [atlas; ♂; ♀]. —Wells et al. 2019: 33 [detection frequency; as *bifurcata*].

**Distribution.** —Australia.

***edmondsi*** Wells, 1982: 259 [type locality: Western Australia, Stonewall Creek; NMV; ♂; ♀]. —Neboiss 1986: 74 [atlas; ♂; ♀].

**Distribution.** —Australia.

***fagesii*** (Guinard), 1879: 19 [type locality: [France], dans les bassins des Prés d’Arènes; depository not designated; ♂; larva; pupal case; in *Leiochiton*]. —McLachlan 1880: 523 [*Hydroptilaflabellifera* larvae considered as *Leiochitonfagesii*]. —Lauterborn 1934: 220 [larvae of *Hydroptilaflabellifera* re-identified as either *Agrayleapallidula* or *Leiochitonfagesii*]. —Martynov 1934: 153 [♂; in *Oxyethira*]. —Mosely 1939b: 291 [♂]. —Spuris 1962: 66 [distribution]. —Botosaneanu 1967: 294 [distribution]. —Botosaneanu and Malicky 1978: 341 [checklist]. —Wiberg-Larsen 1981: 28 [distribution; larva; larval case]. —Moretti and Cianficconi 1981: 201 [checklist; as *fagesi*]. —Hiilivirta 1982: 154 [distribution]. —Malicky 1983b: 58 [atlas; ♂; ♀; as *fagesi*]. —Wiberg-Larsen 1985: 40 [checklist]. —Brock 1987: 85 [distribution; biology]. —Andersen and Wiberg-Larsen 1987: 169 [checklist]. —O’Connor and O’Hanrahan 1988: 478 [distribution]. —Spuris 1989: 18 [distribution]. —Chvojka 1996: 131 [distribution]. —Graf et al. 1998: 206 [distribution]. —Varga et al. 1998: 147 [distribution; larva]. —Malicky 1999f: 32 [distribution]. —Gullefors 2002: 133, 138 [redlisted in Sweden; checklist]. —Gullefors 2003: 195 [distribution]. —Malicky 2004a: 70 [atlas]. —Malicky 2005b: 54 [checklist]. —Gullefors 2005b: 138 [distribution]. —Lubini-Ferlin and Vicentini 2005: 68 [checklist]. —Arnold et al. 2005: 141 [distribution]. —Sweeney 2006: 300 [distribution]. —Cianficconi et al. 2007: 569, 576 [distribution]. —Gullefors and Johanson 2007: 68 [checklist]. —Robert 2007: 83 [checklist]. —Coppa and Jolivet 2008: 91 [distribution; biology]. —Gullefors 2008: 64 [distribution]. —Ujvárosi et al. 2008: 113 [distribution]. —Schrankel et al. 2008: 90 [distribution]. —Chvojka and Komzák 2008: 13 [distribution]. —Ivanov 2011: 196 [checklist]. —Viidalepp et al. 2011: 196 [distribution; as *fagesi*]. —O’Connor 2015: 28, 106 [distribution]. —Šidagytė et al. 2016: 79 [distribution; biology]. —Pan’kov and Krasheninnikov 2016: 333 [distribution]. —Sanabria and Tempelman 2016: 14 [distribution; biology]. —Gullefors 2016: 156 [checklist]. —Wallace 2016: 15, 18, 25 [conservation status]. —Graf et al. 2017: 48 [distribution]. —Dzhurtubaev et al. 2017: 58 [distribution]. —O’Connor 2019a: 165 [distribution]. —Labat et al. 2019: 107 [distribution]. —Brophy and O’Connor 2020: 244 [distribution]. —O’Connor 2020: 141 [distribution].

—*felina* (Ris, 1897): 422 [type locality: [Switzerland], Katzensee, aus Material vom Torfstich im Zimmer gezogen; depository not designated; ♂; in *Oxyethira*]. —Mosely 1939b: 291–292 [to synonymy].

**Distribution.** —Austria, Czech Republic, Denmark, Estonia, Finland, France, Germany, Hungary, Ireland, Italy, Latvia, Lithuania, Luxembourg, Netherlands, Romania, Russia, Sweden, Switzerland, Ukraine.

***fidelis*** Wells, 1982: 253 [type locality: north Queensland, Alice River on Hervey Range Road; NMV; ♂; ♀]. —Wells 1985b: 19 [larva; case]. —Neboiss 1986: 74 [atlas; ♂; ♀].

**Distribution.** —Australia.

***fortensis*** (Ulmer, 1951): 82 [type locality: [Indonesia], Sumatra, Fort de Kock; ZMUH; ♂; in *Synagotrichia*]. —Wells and Huisman 1992: 108 [♂; distribution]. —Wells and Malicky 1997: 185 [distribution]. —Malicky and Chantaramongkol 2007: 1025 [distribution]. —Malicky 2007a: 177 [checklist]. —Malicky 2010a: 38 [atlas; ♂]. —Malicky et al. 2014a: 6 [distribution]. —Mattern 2015: 502 [distribution].

—*lacustris* Kimmins, 1951: 210 [type locality: [Myanmar], S. Shan States, Inle Lake, S. end, 900 m; NHRS; ♂]. —Malicky 2013: 43 [to synonymy]. —Wityi et al. 2015: 47 [checklist].

**Distribution.** —India, Indonesia, Malaysia, Myanmar, Nepal, Thailand, Vietnam.

***jabirella*** Wells, 1985a: 99 [type locality: Australia, Northern Territory, Corndorl Billabong, nr Jabiru; NTM; ♂; ♀]. —Neboiss 1986: 74 [atlas; ♂; ♀]. —Wells and Dostine 2016: 599 [distribution]. —Wells et al. 2019: 33 [detection frequency].

**Distribution.** —Australia.

***neotropicalis*** Flint, 1991a: 70 [type locality: Brazil, Estado Roraima, Ilha Maraca, Rio Urariocoera; INPA; ♂; ♀]. —Angrisano 1999: 35 [checklist]. —Paprocki et al. 2004: 12 [checklist]. —Ribeiro et al. 2009: 34 [list of types]. —Paprocki and França 2014: 55 [checklist].

**Distribution.** —Brazil.

***pennyae*** Wells, 1998: 82 [type locality: [Australia] Tasmanian World Heritage Area, Southwest National Park, Melaleuca, 43°31'00"S, 146°09'41"E; ANIC; ♂]. —Neboiss 2002: 53 [checklist]. —Wells 2002b: 41 [distribution].

**Distribution.** —Australia.

***suwannee*** Chantaramongkol & Malicky, 1986: 515 [type locality: [Sri Lanka], Western Province, Yakkala, 18 mi NE von Colombo, 30 m; MZLU; ♂].

**Distribution.** —Sri Lanka.

***tridens*** Wells, 1982: 256 [type locality: Western Australia, Mitchell Plateau; NMV; ♂; ♀]. —Neboiss 1986: 75 [atlas; ♂; ♀].

**Distribution.** —Australia.

#### Genus *Ugandatrichia* Mosely, 1939

*Ugandatrichia* Mosely, 1939d: 36 [type species: *Ugandatrichiaminor* Mosely, 1939d, original designation]. —Marshall 1979b: 198 [generic review].

*Moselyella* Kimmins, 1951: 195 [type species: *Ithytrichiaviolacea* Morton, 1902, original designation]. —Schmid 1960 [to synonymy].

The genus *Ugandatrichia* consists of 31 relatively large species occurring in Africa and south and Southeast Asia. The genus was synonymized with *Microptila* by Schmid (1960), but later reinstated by Marshall (1979b). Marshall (1979b) considered *Ugandatrichia* to be more closely related to *Agraylea* than *Microptila*, based on similarities of the form of the male genitalia and the thoracic nota. The larvae of *U.rhodesiensis* were described by Scott (1976), with several other species having since been provided (Vaillant 1984; Hsu and Chen 2002; Laudee 2008; Ito and Ohkawa 2012).

***acuta*** Mosely, 1939d: 39 [type locality: Kenya, Chania Falls, nr. Nairobi, 4000 ft.; NHMUK; ♀]. —Kimmins 1959: 56 [distribution]. —Johanson 1992: 119 [checklist]. —Wells and Andersen 1995: 145 [checklist].

**Distribution.** —Kenya, Uganda

***africana*** (Marlier & Vaillant, 1967): 25 [type locality: [Congo], dans la cascade Kiromera, dans l’Urundi, qui se déverse, vers 1,500 m, dans le Kitenge, affluent de la rivière Ruzizi, territ. Bubanza; depository not designated; ♂; in *Allotrichia*]. —Vaillant 1984: 409 [larva; biology; to *Ugandatrichia*].

**Distribution.** —Congo.

***atakpamensis*** Gibon, 1987a: 122 [type locality: sur l’Amoutchou au niveau de la route Atakpamé/Kpalimé (bassin du Mono, Togo); MNHN; ♂]. —Gibon et al. 1994: 110 [distribution]. —Kjærandsen and Andersen 1997: 244 [distribution].

**Distribution.** —Burkina Faso, Ghana, Togo.

***batanta*** Oláh in Oláh and Kovács 2018: 179 [type locality: Indonesia, West Papua, Batanta Island, Kalijakut River, S00°52'52.0", E130°38'38.0"; Collection Oláh; ♂].

**Distribution.** —Indonesia.

***cathyae*** Wells, 1991: 507 [type locality: Papua New Guinea, Bougainville Island, Panguna, Konaiano Creek; ANIC; ♂; ♀].

**Distribution.** —Papua New Guinea.

***cyanotrichia*** (Kimmins, 1951): 198 [type locality: [Myanmar], N.E. Burma, Kambaiti, 6–7000 ft.; NHRS; ♂; ♀; in *Moselyella*]. —Wityi et al. 2015: 47 [checklist].

**Distribution.** —Myanmar.

***dentata*** Wells & Andersen, 1995: 156 [type locality: Tanzania, Tanga region, West Usambara Mts, Mazumbai, Kaputu Stream, loc. 8, 1510 m a.s.l.; ZMUB; ♂].

**Distribution.** —Tanzania.

***frigoris*** Mey, 1998b: 7 [type locality: [Vietnam], Quellbach, et. ca. 1400 m; ZMHB; ♂]. —Mey 2005a: 281 [distribution]. —Armitage et al. 2005: 27 [checklist]. —Malicky 2010a: 36 [atlas; ♂].

**Distribution.** —Vietnam.

***hairanga*** Oláh, 1989: 274 [type locality: Vietnam, Tamdao, 1300 m a.s.l.; HNHM; ♂; ♀]. —Wells and Huisman 1992: 95 [♂; distribution]. —Armitage et al. 2005: 27 [checklist]. —Laudee 2008: 32 [larva]. —Malicky 2010a: 35 [atlas; ♂]. —Bunlue et al. 2012: 15 [distribution].

**Distribution.** —Malaysia, Thailand, Vietnam.

***honga*** Oláh, 1989: 275 [type locality: Vietnam, Tamdao, 200 m a.s.l.; HNHM; ♂]. —Armitage et al. 2005: 27 [checklist]. —Laudee 2008: 33 [larva]. —Oláh and Johanson 2010a: 36 [distribution]. —Malicky 2010a: 35 [atlas; ♂]. —Laudee and Prommi 2011: 283 [distribution]. —Bunlue et al. 2012: 15 [distribution]. —Yang et al. 2016: 477 [checklist]. —Malicky et al. 2018: 1323 [distribution]. —Oláh and Kovács 2018: 180 [♂]. —Murray-Stoker et al. 2020: 97 [♂; distribution].

—*navicularis* Xue & Yang, 1990: 125 [type locality: [China] Bawangling (320 m), Hainan; NAUJ; ♂]. —Yang et al. 1997b: 93 [checklist]. —Yang et al. 2005: 458 [checklist]. —Malicky 2013: 43 [to synonymy].

**Distribution.** —China, Laos, Thailand, Vietnam.

***kanikar*** Malicky & Chantaramongkol, 1991: 81 [type locality: [Indonesia], Sumatra, Huta Padang; Collection Malicky; ♂]. —Malicky 2007a: 177 [checklist]. —Malicky 2010a: 35 [atlas; ♂].

**Distribution.** —Indonesia.

***kebumen*** Wells & Malicky, 1997: 181 [type locality: [Indonesia] Central Java, Kebumen near Salatiga; Collection Malicky; ♂]. —Malicky 2010a: 35 [atlas; ♂]. —Malicky et al. 2014a: 6 [distribution]. —Oláh and Kovács 2018: 180 [♂].

**Distribution.** —Indonesia.

***kerdmuang*** Malicky & Chantaramongkol, 1991: 81 [type locality: Thailand, Chattrakan; Collection Malicky; ♂]. —Malicky 1999e: 203 [larva; biology]. —Laudee 2004: 21 [larva; biology]. —Laudee 2008: 30 [larva]. —Melnitsky and Malicky 2008: 25 [distribution]. —Malicky 2010a: 34 [atlas; ♂]. —Laudee and Prommi 2011: 283 [distribution]. —Bunlue et al. 2012: 15 [distribution].

**Distribution.** —Thailand.

***kuringhat*** Malicky & Chantaramongkol, 2007: 1028 [type locality: Nepal, Dakhi Khola bei Kuring Ghat, 27°52'N 84°38'E, 300 m; Collection Malicky; ♂]. —Mattern 2015: 502 [distribution].

**Distribution.** —Nepal.

***lampai*** Wells & Huisman, 1992: 95 [type locality: East Malaysia, Sarawak, Lambir National Park, E of Miri; NTM; ♂; ♀]. —Malicky 2010a: 36 [atlas; ♂]. —Oláh and Kovács 2018: 180 [♂].

**Distribution.** —Malaysia.

***maliwan*** Malicky & Chantaramongkol, 1991: 80 [type locality: Thailand, Doi Inthanon, Bang Khun Klang, 1200 m; Collection Malicky; ♂]. —Malicky 1999e: 199 [larva; biology]. —Thani and Chantaramongkol 1999: 411 [distribution; biology]. —Laudee 2008: 30 [larva]. —Oláh and Johanson 2010a: 37 [distribution]. —Malicky 2010a: 35 [atlas; ♂]. —Bunlue et al. 2012: 6 [distribution].

**Distribution.** —Laos, Thailand.

***manensis*** Gibon, 1987a: 122 [type locality: sur le Ko á Man (bassin du Sassandra, Côte d’Ivoire); MNHN; ♂].

**Distribution.** —Côte d’Ivoire.

***mindanaensis*** Mey, 1998a: 547 [type locality: [Philippines, Mindanao], northern slope of Mt. Atuuganon rang, 1050 m; ZMHB; ♂]. —Wells and Mey 2002: 134 [checklist].

**Distribution.** —Philippines.

***mindoroensis*** Mey, 1995: 193 [type locality: [Philippines], Mindoro, Paluan, Calawagan-Fluß; Collection Mey; ♂]. —Wells and Mey 2002: 125 [distribution]. —Oláh and Kovács 2018: 180 [♂; as mindoroensii].

**Distribution.** —Philippines.

***minor*** Mosely, 1939d: 36 [type locality: Kenya, Thomson’s Falls, N. of Nakuru, 7500 ft.; NHMUK; ♂]. —Kimmins 1959: 56 [distribution]. —Johanson 1992: 119 [checklist]. —Wells and Andersen 1995: 145 [checklist].

**Distribution.** —Kenya, Uganda.

***nakijinensis*** Ito in Ito and Ohkawa 2012: 49 [type locality: Japan, Okinawa-jima, Nakijin-son, Shigema-gawa, 26°41'N, 127°56'E, 75 m above sea level; CMI-ZI; ♂; ♀; pupa; larva]. —Tanida and Kuranishi 2016: 74 [checklist].

**Distribution.** —Japan.

***nigra*** Mosely, 1939d: 37 [type locality: [Uganda], Ruwenzori, Namwamba Valley, 6500 ft.; NHMUK; ♂; ♀]. —Kimmins 1959: 51 [distribution]. —Johanson 1992: 119 [checklist]. —Wells and Andersen 1995: 145 [checklist].

**Distribution.** —Uganda.

***nikataruwa*** (Schmid, 1958b): 43 [type locality: [Sri Lanka], Ceylan, Pooprasie (C. P., 2700 ft) 21-I, petit ruisseau à fond caillouteux, dans les plantations de thé; depository not designated; ♂; in *Moselyella*].

**Distribution.** —Sri Lanka.

***rhodesiensis*** Hsu & Chen, 2002: 75 [type locality: Rhodesia, Chimanimani National Park, Bundi River, main waterfall (c. 1,580 m); AMGS; ♂; ♀; larva; pupa]. —Oláh and Kovács 2018: 180 [♂].

**Distribution.** —Zimbabwe.

***sanana*** Oláh, 1989: 276 [type locality: Vietnam, Tamdao, 800 m a.s.l.; HNHM; ♂]. —Armitage et al. 2005: 27 [checklist]. —Malicky 2010a: 36 [atlas; ♂].

—*spinata* Wells & Dudgeon, 1990: 166 [type locality: Hong Kong, Tai Po Kao Forest stream; NHMUK; ♂; ♀]. —Malicky 2013: 43 [to synonymy]. —Yang et al. 2016: 477 [checklist].

**Distribution.** —Hong Kong, Vietnam.

***shinshiroensis*** Ito, Nishimoto, & Nishimoto, 2018: 492 [type locality: Japan, Honshu, Aichi, Shinshiro-shi, Toyooka, Ichinose, Ôtsutani-gawa River, near river mouth (34°59'26"N, 137°37'29"E, 129 m above sea level); CBM-ZI; ♂; ♀; pupa; larva].

**Distribution.** —Japan.

***sourya*** (Schmid, 1960): 86 [type locality: [Pakistan] Himalaya, Surgun; CNC; ♂; in *Microptila*]. —Schmid 1958c: 220 [as new species, *nomen nudum*]. —Marshall 1979b: 199 [to *Ugandatrichia*]. —Oláh and Kovács 2018: 180 [♂]. —Lonsdale 2020: 40 [holotype depository].

**Distribution.** —Pakistan.

***taiwanensis*** Hsu & Chen, 2002: 75 [type locality: Taiwan, Taichung Co., Shiwern Stream, 700 m; NMNS; ♂; ♀; larva, pupa, case]. —Ito and Ohkawa 2012: 54 [♂; ♀; distribution]. —Malicky 2014a: 1623 [checklist]. —Tanida and Kuranishi 2016: 74 [checklist]. —Yang et al. 2016: 477 [checklist].

**Distribution.** —Japan, Taiwan.

***tanzaniensis*** Wells & Andersen, 1995: 155 [type locality: Tanzania, Tanga region, West Usambara Mts, Mazumbai, Kaputu Stream, loc. 7, 1535 m a.s.l.; ZMUB; ♂].

**Distribution.** —Tanzania.

***violacea*** (Morton), 1902: 283 [type locality: [India], Khasias; no type depository designated; ♂; in *Ithytrichia*]. —Kimmins 1951: 196 [♂; in *Moselyella*].

**Distribution.** —India.

***yameogoi*** Gibon, 1987a: 122 [type locality: Konsankoro, sur un affluent du Haut-Nilo (bassin du Niger, Guinée); MNHN; ♂]. —Kjærandsen and Andersen 1997: 244 [distribution].

**Distribution.** — Côte d’Ivoire, Ghana, Guinea, Togo.

#### Genus *Vietrichia* Oláh, 1989

*Vietrichia* Oláh, 1989: 272 [type species: *Vietrichialinghia* Oláh, 1989, original designation].

The monotypic genus *Vietrichia* is known only from Vietnam. The genus can be separated from all other hydroptilids by the spur formula (0, 2, 4), the convex, pentagonal mesoscutellum, and the general structure of the male genitalia (Oláh 1989). Using Marshall’s (1979b) key to hydroptilids, Oláh (1989) placed the genus in Hydroptilinae, based on the symmetrical male genitalia, the phallus that was not thread-like in appearance, and the absence of a transverse suture on the mesoscutellum. The larval stage is unknown.

***linghia*** Oláh, 1989: 273 [type locality: Vietnam, Hoabinh, 20 km in the direction of Tanlac along the road at a small waterfall; HNHM; ♂; ♀]. —Armitage et al. 2005: 27 [checklist]. —Malicky 2010a: 38 [atlas; ♂].

**Distribution.** —Vietnam.

#### Genus *Wlitrichia* Kjærandsen, 1997

*Wlitrichia* Kjærandsen, 1997: 230 [type species: *Wlitrichiaintropertica* Kjærandsen, 1997, original designation].

The monotypic genus *Wlitrichia* has been recorded only from Ghana. Kjærandsen (1997) stated that, in general, *Wlitrichia* is very similar morphologically to *Hydroptila*, but the male genitalia very closely resemble those of the subgenus Loxotrichia (Oxyethira) and differ distinctly in the structure of the inferior appendages. In the parsimony analysis by Kjærandsen (1997) using morphological characters, *Wlitrichia* grouped with the genera *Hydroptila* and *Paucicalcaria*. The larval stage is unknown.

***intropertica***Kjærandsen 1997: 232 [type locality: Ghana, Volta Region, Agumatsa waterfalls, Wli, station 3A; ZMUB; ♂; ♀].

**Distribution.** —Ghana.

#### Genus *Xuthotrichia* Mosely, 1934

*Xuthotrichia* Mosely, 1934a: 139 [type species: *Xuthotrichiaochracea* Mosely, 1934a, original designation]. —Marshall 1979b: 209 [generic review]. —Wells 1979b: 312 [generic review]. —Ward and Henderson 2004: 17 [re-diagnosis of male adults].

*Xuthotrichia* consists of two species, one each in New Zealand and Australia. The characteristic male genitalia are asymmetrical and very complex. The genus may be closely related to *Paroxyethira*, based on features of the adult head and thorax; the two genera together may be allied to *Oxyethira* (Marshall 1979b). The larval stage is unknown.

***aotea*** Ward & Henderson, 2004: 15 [type locality: [New Zealand], BR Lewis Pass, 1.6 km south, MT in snow tussock at beech forest edge, 24602, 58686, 850 m; CMNZ; ♂].

**Distribution.** —New Zealand.

***ochracea*** Mosely, 1934a: 140 [type locality: [Australia, Queensland] Brisbane; Collection Tillyard (transferred to NHMUK according to Wells 1979b: 312); ♂]. —Mosely and Kimmins 1953: 520 [♂]. —Wells 1979b: 312 [♂; distribution]. —Neboiss 1986: 73 [atlas; ♂].

**Distribution.** —Australia.

### ﻿Subfamily LEUCOTRICHIINAE Flint, 1970

Leucotrichiinae Flint, 1970: 2 [type genus: *Leucotrichia* Mosely, 1934a]. —Marshall 1979b: 175 [reviewed as tribe Leucotrichiini]. —Santos et al. 2016a: 1 [revised classification; phylogeny].

Leucotrichiinae consists of 16 genera in two tribes occurring predominantly in Central America and northern South America, with a few species recorded from North America and a few as far south as Chile. Flint (1970) established the subfamily for the genus *Leucotrichia* and several closely related genera. In the original description, Flint stated that there was no single character that defined the group as separate from other hydroptilid adults. However, he did consider the following set of character states to be diagnostic when all present: modified head and antennae, ocelli reduced to two in males, and the presence of a basal costal “pouch” or “bulla” on the male forewing. The basic structure of the male genitalia also proved to be difficult to define clearly and Flint merely noted that the form displayed “something characteristic”. Marshall (1979b) gave a more detailed description, but also noted that, while it may seem to form a distinct unit, the subfamily was very difficult to define. Further, she also stated that the genera are difficult to distinguish. Features used to establish genera have been inconsistent, with some genera originally established and defined based on characteristics of the head, antennae, and wings, while others were based on characteristics of the genitalia (Marshall 1979b). Most recently, Santos et al. (2016a) conducted a phylogenetic analysis of Leucotrichiinae relationships based on both morphological and molecular evidence and proposed several taxonomic changes, including the establishment of the tribe Alisotrichiini.

Leucotrichiinae shares many morphological similarities with members of Stactobiinae, but this observation may indicate convergent evolution and not a shared common ancestry (Marshall 1979b). Bowles et al. (1999) assessed genera of Stactobiinae occurring in the New World and transferred several to Leucotrichiinae based on larval characters which they concluded are derived for Leucotrichiinae. Several genera have been transferred back and forth between Leucotrichiinae and Stactobiinae, indicating that the limits between the subfamilies are poorly defined and in need of further research. Larval descriptions have been published for most genera, excluding *Ascotrichia*, *Betrichia*, and *Costatrichia*.

#### Tribe ALISOTRICHIINI Santos, Nessimian, & Takiya

Alisotrichiini Santos, Nessimian, & Takiya, 2016: 471 [type genus: *Alisotrichia* Flint, 1964]. —Marshall 1979b: 175 [referred to as the *Alisotrichia* group]. —Oláh and Johanson 2011: 142 [referred to as the *Celaenotrichia* genus cluster].

The tribe Alisotrichiini contains six genera, with type genus *Alisotrichia*. When Flint (1970) first established the subfamily Leucotrichiinae, he considered *Alisotrichia* to be a distinct unit separate from the other included genera. As additional genera were described and considered part of the *Alisotrichia* group, they were at different times placed in either Leucotrichiinae (Bowles et al. 1999) or Stactobiinae (Harris and Holzenthal 1993). In a summary of generic character states, all six genera were placed in a *Celaenotrichia* genus cluster by Oláh and Johanson (2011), although no statistical analysis was performed or discussed. These genera were recently found to be united in a monophyletic clade within Leucotrichiinae, based on a combined analysis of morphological and molecular data (Santos et al. 2016a). Santos et al. (2016a) also stated that morphological synapomorphies included features found in the male genitalia.

##### Genus *Alisotrichia* Flint, 1964

*Alisotrichia* Flint, 1964: 46 [type species: *Alisotrichiahirudopsis* Flint, 1964, original designation]. —Flint 1970: 24 [revision; in Leucotrichiinae]. —Marshall 1979b: 183 [generic review]. —Flint 1991b: 44 [key to Antioquian species]. —Harris and Holzenthal 1993: 155 [phylogeny; placement in Hydroptilinae, Stactobiini]. —Bowles et al. 1999: 51 [immatures; placement in Hydroptilinae, Leucotrichiini]. —Oláh and Johanson 2011: 142 [placement in *Celaenotrichia* genus cluster]. —Santos et al. 2016a: 471 [type genus of tribe Alisotrichiini].

*Rioptila* Blickle & Denning, 1977: 299 [type species: *Rioptilaarizonica* Blickle & Denning, 1977, original designation]. —Harris and Holzenthal 1993: 155 [to synonymy].

The genus *Alisotrichia* contains 62 species, including one fossil species known from Dominican amber. The distribution of the genus extends from the southwestern United States, through Mexico and Central America into Venezuela, and also the Antilles. The genus was first placed in Leucotrichiinae by both Flint (1964) and Marshall (1979b), but later was transferred to Stactobiinae (Harris and Holzenthal 1993) and then returned to Leucotrichiinae (Bowles et al. 1999). Flint (1970) divided *Alisotrichia* into several species groups based on adult features, which Marshall (1979b) claimed were not well defined and declined to discuss further. Harris and Holzenthal (1993) later divided the genus into 8 species groups based on tibial spur formula, antennal structure, and features of the male genitalia. The three basal species groups have since been transferred to genera of their own (*blantoni*, *dominicensis*, and *quemada* to *Mejicanotrichia*, *Cerasmatrichia*, and *Scelobotrichia*, respectively). The mature larva of the type species, *A.hirudopsis*, was first described by Flint (1964) and several additional species have been described since. Larvae of *Alisotrichia* are distinct from all other larvae of Leucotrichiinae in that, instead of building a case during the fifth and final instar, they remain free-living until pupation (Marshall 1979b).

***aglae*** Botosaneanu, 1991: 118 [type locality: Haiti, Département de l’Oest, Ville Bonheur (Ville Saut d’Eau); ZMUA; ♂]. —Botosaneanu 2002b: 81 [checklist]. —Flint and Pérez-Gelabert 1999: 39 [checklist]. —Flint and Sykora 2004: 26 [distribution]. —Pérez-Gelabert 2008: 300 [checklist].

**Distribution.** —Dominican Republic, Haiti.

***alayoana*** Botosaneanu, 1977: 256 [type locality: Cuba, Oriente, Baire, Rio Mogote; NMNH; ♂].—Botosaneanu 1979: 48 [distribution]. —Botosaneanu 1994b: 455 [larva]. —Flint 1996a: 16 [checklist]. —Botosaneanu 2002b: 81 [checklist]. —López del Castillo et al. 2004: 229 [distribution]. —Naranjo López and González Lazo 2005: 149 [checklist].

**Distribution.** —Cuba.

***aquaecadentis*** Botosaneanu, 1991: 116 [type locality: Haiti, Département de Sud, Saut Mathurine, Rivière du Cavaillon; ZMUA; ♂; ♀]. —Botosaneanu 2002b: 81 [checklist]. —Flint and Pérez-Gelabert 1999: 39 [checklist]. —Flint and Sykora 2004: 27 [distribution]. —Pérez-Gelabert 2008: 300 [checklist].

**Distribution.** —Dominican Republic, Haiti.

***arcana*** Botosaneanu, 1991: 124 [type locality: Haiti, Département de Sud, près de Camp Perrin, Résurgence du Moreau; ZMUA; ♂]. —Flint and Pérez-Gelabert 1999: 39 [checklist]. —Flint and Sykora 2004: 27 [distribution]. —Pérez-Gelabert 2008: 300 [checklist].

**Distribution.** —Dominican Republic, Haiti.

***argentilinea*** Flint, 1968b: 34 [type locality: Jamaica, St. Andrew, Chestervale, Yallahs River; NMNH; ♂; ♀; larva; case]. —Flint 1968a: 81 [checklist]. —Botosaneanu 2002b: 81 [checklist].

**Distribution.** —Jamaica.

♰ ***arizela*** Wells & Wichard, 1989: 43 [type locality: Dominican Republic; Collection Wichard; ♂; in amber]. —Flint and Pérez-Gelabert 1999: 39 [checklist]. —Botosaneanu 2002b: 81 [checklist]. —Wichard 2007: 48 [checklist]. —Eskov et al. 2008: 78 [checklist]. —Pérez-Gelabert 2008: 300 [checklist].

**Distribution.** —Dominican amber.

***arizonica*** (Blickle & Denning, 1977): 300 [type locality: [U.S.A.] Oak Creek Canyon, Arizona; CAS; ♂; in *Rioptila*]. —Blickle 1979: 54, 61 [checklist; ♂; as *R.arizonensis*]. —Harris and Holzenthal 1993: 155 [♂, to *Alisotrichia*]. —Moulton et al. 1994: 169 [distribution]. —Moulton and Stewart 1997: 350 [checklist]. —Bowles et al. 1999: 44 [larva]. —Blinn and Ruiter 2005: 68 [distribution; biology].

**Distribution.** —U.S.A.

***asta*** Harris & Flint, 2002: 207 [type locality: Panama, Barro Colorado Island, Snyder-Molino trail, marker 3; NMNH; ♂]. —Armitage et al. 2015a: 6 [checklist]. —Armitage and Harris 2018b: 97 [checklist]. —Harris and Armitage 2019: 4 [distribution].

**Distribution.** —Panama.

***befoga*** Oláh & Flint, 2012: 159 [type locality: Peru, Huanuco Province, Tingo Maria, 672 m, premontane rain forest; NMNH; ♂].

**Distribution.** —Peru.

***benji*** Rueda-Martín, 2011: 2 [type locality: Argentina, Jujuy, A° Yuto, Parque Nacional Calilegua, S23°38'40.2", W64°35'53.7", 505 m; IML; ♂].

**Distribution.** —Argentina.

***bernali*** Harris & Armitage, 2019: 8 [type locality: Panama, Bocas del Toro Province, Quebrada Rambala, near Rambala Jungle Lodge, 3.74 km SSE Rambala, 8.91543°N and 82.15527°W, 120 m; COZEM; ♂].

**Distribution.** —Panama.

***bisetosa*** Flint & Sykora, 2004: 27 [type locality: Dominican Republic, Independencia Province, Río Guyabal, 4.5 km N Postrer Río, 18°34.7'N, 71°37.7'W, 150 m; NMNH; ♂]. —Pérez-Gelabert 2008: 300 [checklist].

**Distribution.** —Dominican Republic.

***cacaulandia*** Harris & Flint, 2002: 200 [type locality: Brazil, Rondonia, creek 8 km S Cacaulandia; NMNH; ♂]. —Paprocki et al. 2004: 10 [checklist]. Paprocki and França 2014: 40 [checklist].

**Distribution.** —Brazil.

***cainguas*** Angrisano & Sganga, 2009: 58 [type locality: [Argentina] Misiones, Parque Provincial Salto Encantado, tributary of Arroyo Cuñá-Pirú; MACN; ♂].

**Distribution.** —Argentina.

***chihuahua*** Bueno-Soria & Harris, 1993: 54 [type locality: Mexico, Chihuahua, Río Concheno, ruta 16 cerca de Basaseachic; NMNH; ♂; ♀]. —Bueno-Soria et al. 2007: 33 [distribution].

**Distribution.** —Mexico.

***chiquitica*** Botosaneanu, 1977: 258 [type locality: Cuba, Oriente, Baracoa, Rio Jojo; NMNH; ♂; ♀]. —Botosaneanu 1979: 48 [distribution]. —Flint 1996a: 16 [checklist]. —Botosaneanu 2002b: 81 [checklist]. —López del Castillo et al. 2004: 229 [distribution]. —Naranjo López and González Lazo 2005: 149 [checklist].

**Distribution.** —Cuba.

***chorra*** Flint, 1970: 27 [type locality: Mexico, Chiapas, El Chorreadero, 6.4 mi S. Chiapa de Corzo; NMNH; ♂]. —Bueno-Soria and Flint 1978: 200 [distribution].

**Distribution.** —Mexico.

***cimarrona*** Botosaneanu, 1977: 254 [type locality: Cuba, Pinar del Rio, Soroa, Rio Manantiales; NMNH; ♂; ♀]. —Botosaneanu 1979: 48 [distribution]. —Flint 1996a: 16 [checklist]. —Botosaneanu 2002b: 82 [checklist]. —Naranjo López and González Lazo 2005: 149 [checklist].

**Distribution.** —Cuba.

***circinata*** Flint, 1992a: 383 [type locality: Puerto Rico, El Verde Field Station, Quebrada Prieta; NMNH; ♂]. —Botosaneanu 2002b: 82 [checklist].

**Distribution.** —Puerto Rico.

***coclensis*** Armitage & Harris, 2020a: 3 [type locality: Panama, Coclé Province, Cuenca 105, Omar Torrijos Herrera National Park, Quebrada Corazones, PSPSCB-PNGDOTH-C103-2017-001, 8.6776°N, 80.6001°W, 728 m; COZEM; ♂].

**Distribution.** —Panama.

***cornicula*** Bueno-Soria & Harris, 1993: 52 [type locality: Mexico, Guerrero, Soyatapec; CNIN; ♂].

**Distribution.** —Mexico.

***cuernita*** Harris & Flint, 2002: 207 [type locality: Panama, Barro Colorado Island, Snyder-Molino trail, marker 3; NMNH; ♂]. —Armitage et al. 2015a: 6 [checklist]. —Armitage and Harris 2018b: 97 [checklist]. —Harris and Armitage 2019: 4 [distribution].

**Distribution.** —Panama.

***cyanolenus*** Flint, 1996b: 91 [type locality: Trinidad, Blue Basin Waterfall, 10°44'N, 61°32'W; NMNH; ♂]. —Botosaneanu 2002b: 82 [checklist].

**Distribution.** —Trinidad, Venezuela.

***euphrosyne*** Botosaneanu, 1991: 118 [type locality: Haiti, Département de l’Ouest, Ville Bonheur (Ville Saut d’Eau); ZMUA; ♂]. —Flint and Pérez-Gelabert 1999: 39 [checklist]. —Botosaneanu 2002b: 82 [checklist]. —Flint and Sykora 2004: 27 [distribution]. —Pérez-Gelabert 2008: 300 [checklist].

**Distribution.** —Dominican Republic, Haiti.

***flintiana*** Botosaneanu, 1977: 253 [type locality: Cuba, Oriente, Baire, Rio Mogote; NMNH; ♂]. —Botosaneanu 1979: 48 [distribution]. —Kumanski 1987: 15 [♀]. —Botosaneanu 1994b: 455 [larva]. —Flint 1996a: 16 [checklist]. —Botosaneanu 2002b: 82 [checklist]. —Naranjo López and González Lazo 2005: 149 [checklist].

**Distribution.** —Cuba.

***fundorai*** (Botosaneanu & Sykora, 1973): 397 [type locality: Cuba, Petit affluent du Rio Caburny, Sierra Escambray, près Topes de Collantes; NMNH; ♂; in *Oxyethira*]. —Botosaneanu 1979: 40 [♂, to *Alisotrichia*]. —Kumanski 1987: 15 [♀]. —Flint 1996a: 16 [checklist]. —Botosaneanu 2002b: 82 [checklist]. —Naranjo López and González Lazo 2005: 149 [checklist].

**Distribution.** —Cuba.

***gabriel*** Angrisano & Burgos, 2002: 108 [type locality: Argentina, Misiones, Bernardo de Irigoyen, Cuenca del arroyo Urugua-í, Establecimiento Intercontinental; MACN; ♂].

**Distribution.** —Argentina.

***giampaolina*** Botosaneanu in Botosaneanu and Hyslop 1998: 10 [type locality: Jamaica, St. Ann, Ocho Rios, Shaw Park Gardens; ZMUA; ♂; ♀]. —Botosaneanu 2002b: 82 [checklist].

**Distribution.** —Jamaica.

***hirudopsis aitija*** Botosaneanu, 1995a: 22 [type locality: Dominican Republic, Arroyo los Guineos, on road San Francisco de Macoris to Loma; ZMUA; ♂]. —Flint and Pérez-Gelabert 1999: 39 [checklist]. —Flint and Sykora 2004: 27 [distribution]. —Pérez-Gelabert 2008: 300 [checklist].

**Distribution.** —Dominican Republic.

***hirudopsishirudopsis*** Flint, 1964: 47 [type locality: Puerto Rico, El Yunque, stream crossing road 191 at km 6.4; NMNH; ♂; ♀, larva, pupa, case]. —Flint 1968a: 81 [checklist]. —Botosaneanu 2002b: 82 [checklist]. —Santos et al. 2016a: 466 [♂].

**Distribution.** —Puerto Rico.

***hispaniolina*** Botosaneanu, 1991: 116 [type locality: Haiti, Département de l’Ouest, Rivière Tombe à Mirebalais, ZMUA; ♂; ♀]. —Botosaneanu 1995a: 23 [distribution]. —Flint and Pérez-Gelabert 1999: 39 [checklist]. —Botosaneanu 2002b: 82 [checklist]. —Flint and Sykora 2004: 27 [distribution]. —Pérez-Gelabert 2008: 300 [checklist].

**Distribution.** —Dominican Republic, Haiti.

***holzenthali*** Santos, 2011: 60 [type locality: Brazil, Minas Gerais State, Santana do Riacho municipality, Cardeal Mota, Rio Cipó, Cachoeira Grande, 19°20'46.7"S, 43°38'09.7"W; DZRJ; ♂; ♀]. —Paprocki and França 2014: 40 [checklist].

**Distribution.** —Brazil.

***kantala*** Oláh & Johanson, 2011: 143 [type locality: Peru, San Martin Prov., La Catarata de Ahuashiyascu, 6°27.544'S, 76°18.192'W; NHRS; ♂].

**Distribution.** —Peru.

***kanukua*** Harris & Flint, 2002: 200 [type locality: Guyana, Kanuku Mountains, Moco River, 3°18.2'N, 59°38.9'W; NMNH; ♂; ♀]. —Oláh and Johanson 2011: 144 [distribution].

**Distribution.** —French Guiana, Guyana.

***kevera*** Oláh & Johanson, 2011: 144 [type locality: French Guiana, Approuaguekaw, Kaw Mt., 4°33.257'N, 52°11.920'W, 216 m; NHRS; ♂].

**Distribution.** —French Guiana.

***latipalpis*** Flint, 1991b: 44 [type locality: Colombia, Dpto. Antioquia, Quebrada La Jiménez, Sopretrán; NMNH; ♂]. —Muñoz-Quesada 2000: 277 [checklist].

**Distribution.** —Colombia.

***linterna*** Harris & Flint, 2002: 198 [type locality: Panama, Barro Colorado Island, Snyder-Molino trail, marker 3; NMNH; ♂]. —Armitage et al. 2015a: 6 [checklist]. —Armitage and Harris 2018b: 97 [checklist].

**Distribution.** —Panama.

***lobata*** Flint, 1968a: 43 [type locality: Dominica, Clarke Hall; NMNH; ♂; ♀]. —Flint 1968a: 81 [checklist]. —Flint and Sykora 1993: 49 [checklist]. —Botosaneanu 2002b: 82 [checklist]. —Botosaneanu and Thomas 2005: 55 [checklist].

**Distribution.** —Dominica.

***macae****Santos*, 2011: 65 [type locality: Brazil, Rio de Janeiro State, Macaé Municipality, Rio São Pedro, 22°13'47.6"S, 42°08'04.7"W, 470 m; DZRJ; ♂]. —Paprocki and França 2014: 40 [checklist].

**Distribution.** —Brazil.

***mathisi*** Harris & Flint, 2002: 202 [type locality: Jamaica, St. Andrew, Mavis Bank (1.7 km E), Yallahs River, 18°2.4'N, 77°39.5'W, 575 m; NMNH; ♂; ♀].

**Distribution.** —Jamaica.

***muellita*** Harris & Flint, 2002: 197 [type locality: Peru, Madre de Dios, Manu, Pakitza, 11°56'S, 71°18'W, 250 m; NMNH; ♂].

**Distribution.** —Peru.

***neblina*** Harris & Flint, 2002: 205 [type locality: Venezuela, Territorio Federal Amazonas, Cerro de la Neblina, basecamp, 0°50'N, 66°10'W, 140 m; NMNH; ♂; ♀].

**Distribution.** —Venezuela.

***nessimiani****S*antos, 2011: 66 [type locality: Brazil, Rio de Janeiro State, Nova Friburgo municipality, Cascata, tributary to Rio Macaé, 22°21'54.9"S, 42°15'20.5"W, 391 m; DZRJ; ♂; ♀]. —Paprocki and França 2014: 41 [checklist].

**Distribution.** —Brazil.

***orophila guadeloupea*** Botosaneanu, 1994a: 35 [type locality: [Guadeloupe] rivière du Grand Carbet, dans son cours supérieur, 3° chute du Carbet; ZMUA; ♂]. —Botosaneanu 2000: 256 [distribution]. —Botosaneanu 2002b: 82 [checklist]. —Botosaneanu and Thomas 2005: 55 [checklist].

**Distribution.** —Guadeloupe.

***orophila orophila*** Flint, 1968a: 41 [type locality: Dominica, D’leau Gommier; NMNH; ♂; larva; pupa; case]. —Flint 1968a: 81 [checklist]. —Botosaneanu 1989: 97 [distribution]. —Botosaneanu 1990b: 44 [distribution]. —Flint and Sykora 1993: 49 [checklist]. —Botosaneanu 2002b: 82 [checklist]. —Botosaneanu and Thomas 2005: 37 [distribution].

**Distribution.** —Dominica, Martinique.

***panamensis*** Harris & Flint, 2002: 195 [type locality: Panama, Barro Colorado Island, Canal Zone; NMNH; ♂]. —Armitage et al. 2015a: 6 [checklist]. —Armitage and Harris 2018b: 97 [checklist]. —Harris and Armitage 2019: 4 [distribution].

**Distribution.** —Panama.

***paxilla*** Harris & Flint, 2002: 204 [type locality: Jamaica, St. Elizabeth, Elim, 18°7.1'N, 77°40.5'W; NMNH; ♂].

**Distribution.** —Jamaica.

***rugoka*** Oláh & Johanson, 2011: 146 [type locality: French Guiana, Approuaguekaw, Kaw Mt., 4°33.035'N, 52°11.661'W, 104 m; NHRS; ♂].

**Distribution.** —French Guiana.

***schmidi*** Kumanski, 1987: 16 [type locality: Cuba, Province Las Villas, massive of Guamuaya, Rio Nabujina, near El Piojillo village; SOFM; ♂; ♀]. —Flint 1996a: 16 [checklist]. —Botosaneanu 2002b: 82 [checklist]. —Naranjo López and González Lazo 2005: 149 [checklist].

**Distribution.** —Cuba.

***setigera*** Flint, 1992a: 383 [type locality: Puerto Rico, El Verde Field Station, Quebrada Prieta; NMNH; ♂]. —Botosaneanu 2002b: 82 [checklist].

**Distribution.** —Puerto Rico.

***sonora*** Bueno-Soria & Harris, 1993: 51 [type locality: Mexico, Sonora, Maycoba River, west of Maycoba; NMNH; ♂]. —Bueno-Soria et al. 2007: 33 [distribution].

**Distribution.** —Mexico.

***tenuivirga*** Botosaneanu in Botosaneanu and Hyslop 1998: 10 [type locality: Jamaica, Buff Bay River in Green Hill at “Regele”, Blue Mountains, Portland; ZMUA; ♂]. —Botosaneanu 2002b: 82 [checklist].

**Distribution.** —Jamaica.

***tetraespinosa*** Bueno-Soria & Harris, 1993: 53 [type locality: Mexico, Guerrero, ruta 130, 80 km N. Zihuatanejo; CNIN; ♂].

**Distribution.** —Mexico.

***thalia*** Botosaneanu, 1991: 120 [type locality: Haiti, Département de l’Ouest, Ville Bonheur (Ville Saut d’Eau); ZMUA; ♂]. —Flint and Pérez-Gelabert 1999: 39 [checklist]. —Botosaneanu 2002b: 82 [checklist]. —Flint and Sykora 2004: 29 [distribution]. —Pérez-Gelabert 2008: 300 [checklist].

**Distribution.** —Dominican Republic, Haiti.

***timouchela*** Botosaneanu, 1989: 98 [type locality: Martinique, Rivière Coco (Morne-Vert); ZMUA; ♂; p. 96; ♀; as *Bredinia* sp.]. —Botosaneanu 1990b: 44 [larva; pupa; case; synonymy of *Bredinia* sp.; distribution]. —Flint and Sykora 19393: 49 [checklist]. —Botosaneanu, 2002b: 82 [checklist]. —Harris and Flint 2002: 210 [distribution].

**Distribution.** —Martinique, St. Vincent, Venezuela.

***tiza*** Harris & Holzenthal, 1993: 157 [type locality: Costa Rica, Guanacaste, Río Tizate, 7.2 km NE Cañas Dulces; NMNH; ♂].

**Distribution.** —Costa Rica.

***ubatuba****S*antos, 2011: 62 [type locality: Brazil, São Paulo State, Ubatuba municipality, Rio Canoas, 23°20'18.7"S, 44°50'16.8"W, 475 m; DZRJ; ♂; ♀]. —Paprocki and França 2014: 41 [checklist].

**Distribution.** —Brazil.

***ultima*** Flint & Sykora, 2004: 29 [type locality: Dominican Republic, Azua Province, Río Las Cuevas, 8 km NE Padre Las Casas, 18°46'N, 70°53'W, 580 m; CMNH; ♂]. —Pérez-Gelabert 2008: 300 [checklist].

**Distribution.** —Dominican Republic.

***ventricosa*** Flint, 1991b: 44 [type locality: Colombia, Dpto. Antioquia, Quebrada La Jiménez, Sopretrán; NMNH; ♂]. —Muñoz-Quesada 2000: 277 [checklist].

**Distribution.** —Colombia.

***viuda*** Harris & Flint, 2002: 205 [type locality: Venezuela, Sucre, Parque Nacional Peninsula de Paria, Uquire, Rio La Viuda, 10°42.83'N, 61°57.66'W, 15 m; NMNH; ♂].

**Distribution.** —Venezuela.

***woldai*** Harris & Flint, 2002: 198 [type locality: Panama, Barro Colorado Island, Snyder-Molino trail, marker 3; NMNH; ♂]. —Armitage et al. 2015a: 6 [checklist]. —Armitage and Harris 2018b: 97 [checklist].

**Distribution.** —Panama.

***woodruffi*** Flint & Sykora, 2004: 29 [type locality: Dominican Republic, Monseñor Nouel Province [not La Vega as labelled], 6 km [not mi. as labelled] NW Rt.1 on road to Constanza; FSCA; ♂]. —Pérez-Gelabert 2008: 300 [checklist].

**Distribution.** —Dominican Republic.

##### Genus *Byrsopteryx* Flint, 1981

*Byrsopteryx* Flint, 1981: 27 [type species: *Byrsopteryxmirifica* Flint, 1981, original designation]. —Harris and Holzenthal 1994: 154 [revision; transferred to Stactobiini]. —Bowles et al. 1999: 45 [returned to Leucotrichiini]. —Botosaneanu 2000: 252 [larva; case]. —Oláh and Johanson 2011: 142 [placement in *Celaenotrichia* genus cluster]. —Santos et al. 2016a: 471 [phylogenetic placement]. —Vázquez-Ramos et al. 2020: 487 [larva].

*Byrsopteryx* consists of 16 species occurring in southern North America, Central America, South America, and the Lesser Antilles. In the original description, Flint (1981) stated that the general appearance and behavior of living *Byrsopteryx* adults was very similar to adults of *Alisotrichia* and that the two genera were probably very closely related, despite the larval body form differing greatly between the two. Flint (1981) assigned *Byrsopteryx* to Leucotrichiinae based on the basic larval and adult morphology, despite the portable larval case. He stated that the genus did not fit well in the subfamily. *Byrsopteryx* was later transferred to Stactobiinae (Harris and Holzenthal 1994) and subsequently returned to Leucotrichiinae (Bowles et al. 1999). A description of the larva of *B.mirifica* was provided by Holzenthal and Harris (1992).

***abrelata*** Harris & Holzenthal, 1994: 157 [type locality: Brazil, Rio de Janeiro, Nova Friburgo, municipal water supply; MZUSP; ♂; ♀]. —Blahnik et al. 2004: 4 [distribution]. —Paprocki et al. 2004: 11 [checklist]. —Dumas et al. 2009: 366 [distribution]. —Santos and Nessimian 2010a: 52 [larva; pupa; case]. —Dumas and Nessimian 2012: 15 [checklist]. —Paprocki and França 2014: 42 [checklist]. —Santos et al. 2016a: 465 [adult photograph].

**Distribution.** —Brazil.

***bipartiterga*** Botosaneanu, 2000: 252 [type locality: Guadeloupe, La Deuxième Chute de Carbet, formée par la Rivière du Grand Carbet, sur le territoire du Parc National de la Guadeloupe, 580 m; ZMUA; ♀]. —Botosaneanu 2002b: 82 [checklist].

**Distribution.** —Guadeloupe.

***carioca*** Santos & Nessimian, 2010a: 45 [type locality: Brazil, Rio de Janeiro State, Rio de Janeiro, Floresta de Tijuca; Parque Nacional da Tijuca, Rio Humaitá, 22°57'30.1"S, 43°17'21.4"W, 475 m; DZRJ; ♂; ♀; larva; pupa; biology]. —Paprocki and França 2014: 42 [checklist]. —Santos et al. 2016a: 466 [♂].

**Distribution.** —Brazil.

***chaconi*** Harris & Holzenthal, 1994: 160 [type locality: Costa Rica, Puntarenas, roadside seep, route 2, just W km 234, 8.976°N, 83.299°W; NMNH; ♂; ♀].

**Distribution.** —Costa Rica.

***cuchilla*** Harris & Holzenthal, 1994: 164 [type locality: Costa Rica, Cartago, Chitaria; NMNH; ♂; ♀]. —Armitage et al. 2016: 6 [distribution]. —Armitage and Harris 2018b: 97 [checklist]. —Harris and Armitage 2019: 4 [distribution].

**Distribution.** —Costa Rica, Panama.

***esparta*** Harris & Holzenthal, 1994: 163 [type locality: Costa Rica, Puntarenas, 14.1 mi SE Esparta; NMNH; ♂]. —Armitage et al. 2020: 4 [distribution].

**Distribution.** —Costa Rica, Panama.

***espinhosa*** Harris & Holzenthal, 1994: 164 [type locality: Brazil, Rio de Janeiro, km 17, 18 km S Teresopolis; MZUSP; ♂]. —Paprocki et al. 2004: 11 [checklist]. —Dumas et al. 2009: 366 [distribution]. —Santos and Nessimian 2010a: 52 [♀; larva; pupa; case]. —Paprocki and França 2014: 43 [checklist]. —Santos et al. 2016a: 464 [larva photograph].

**Distribution.** —Brazil.

***gomezi*** Harris & Holzenthal, 1994: 164 [type locality: Costa Rica, Puntarenas, Río Bellavista, ca. 1.5 km NW las Alturas, 8.951°N, 82.846°W; NMNH; ♂; ♀].

**Distribution.** —Costa Rica.

***loja*** Harris & Holzenthal, 1994: 167 [type locality: Ecuador, Zamora-Chinchipe, 30 km E Loja; NMNH; ♂; ♀]. —Ríos-Touma et al. 2017: 9 [checklist].

**Distribution.** —Ecuador.

***mirifica*** Flint, 1981: 27 [type locality: Venezuela, Aragua, Maracay, Río Limón, Estación Piscicultura; NMNH; ♂; ♀; larva; case]. —Holzenthal and Harris 1991: 405 [♂; ♀; larva; case]. —Harris and Holzenthal 1994: 170 [♂; ♀].

**Distribution.** —Venezuela.

***rayada*** Harris & Holzenthal, 1994: 172 [type locality: Ecuador, Cañar, Río Chauchas, 3 km N Zhud; NMNH; ♂; ♀]. —Ríos-Touma et al. 2017: 9 [checklist].

**Distribution.** —Ecuador.

***septempunctata*** (Flint, 1968a): 46 [type locality: Dominica, Pont Casse, 2.2 mi E; NMNH; ♂; in *Alisotrichia*]. —Flint 1981: 27 [to *Byrsopteryx*]. —Flint and Sykora 1993: 49 [checklist]. —Harris and Holzenthal 1994: 172 [♂]. —Botosaneanu 2000: 254 [♂; ♀]. —Botosaneanu 2002b: 82 [checklist]. —Botosaneanu and Thomas 2005: 55 [checklist].

**Distribution.** —Dominica, Guadeloupe.

***solisi*** Harris & Holzenthal, 1994: 175 [type locality: Costa Rica, Puntarenas, Río Singrí, 2 km (air) S Finca Helechales, 9.057°N, 83.082°W; NMNH; ♂; ♀]. —Armitage et al. 2020: 4 [distribution].

**Distribution.** —Costa Rica, Panama.

***tabasquensis*** Bueno-Soria, Santiago-Fragoso, & Barba-Álvarez, 2001: 146 [type locality: Mexico, Tabasco, Municipio de Huimanguillo, Arroyo Las Flores, Villa de Guadalupe 2^a^ Sección Los Chimalapas, km 5 Ruta Malpasito-Carlos A. Madrazo, 17°22'05"N, 93°36'25"W; CNIN; ♂]. —Bueno-Soria et al. 2005: 75 [distribution].

**Distribution.** —Mexico.

***tapanti*** Harris & Holzenthal, 1994: 177 [type locality: Costa Rica, Cartago, Res. Tapantí, Quebrada Palmitos and falls, 9.72°N, 83.78°W; NMNH; ♂; ♀].

**Distribution.** —Costa Rica.

***tica*** Harris & Holzenthal, 1994: 179 [type locality: Costa Rica, Res. Tapantí, unnamed tributary, ca. 8 km (rd.) S headquarters, 9.72°N, 83.78°W; NMNH; ♂; ♀].

**Distribution.** —Costa Rica.

##### Genus *Celaenotrichia* Mosely, 1934

*Celaenotrichia* Mosely, 1934a: 158 [type species: *Celaenotrichiaedwardsi* Mosely, 1934a, original designation]. —Marshall 1979b: 183 [generic review]. —Harris and Flint 1993: 101 [re-description; larva; placement]. —Bowles et al. 1999: 45 [taxonomic position]. —Oláh and Johanson 2011: 142 [placement in *Celaenotrichia* genus cluster]. —Santos et al. 2016a: 471 [phylogenetic placement].

*Celaenotrichia* is a monotypic genus recorded from Chile and Argentina. The genus was first placed in Leucotrichiinae by Marshall (1979b), transferred to Stactobiinae by Harris and Flint (1993), and then returned to Leucotrichiinae by Bowles et al. (1999). Marshall (1979b) asserted that characteristic features of the genus include the distinct structure of the male genitalia and the unmodified antennae and forewings. Larvae were described by Harris and Flint (1993).

***edwardsi*** Mosely, 1934a: 158 [type locality: Chile, Chiloe Island, Castro; NHMUK; ♂]. —Flint 1974a: 87 [checklist]. —Harris and Flint 1993: 101 [♂; ♀; larva; case; distribution]. —Angrisano 1999: 32 [checklist].

**Distribution.** —Argentina, Chile.

##### Genus *Cerasmatrichia* Flint, Harris, & Botosaneanu, 1994

*Cerasmatrichia* Flint, Harris, & Botosaneanu, 1994: 360 [type species: *Cerasmatrichiatrinitatis* Flint, Harris, & Botosaneanu, 1994, original designation]. —Bowles et al. 1999: 46 [taxonomic position]. —Oláh and Johanson 2011: 142 [placement in *Celaenotrichia* genus cluster]. —Santos et al. 2016a: 464 [larva photograph].

*Cerasmatrichia* consists of ten species distributed in the Neotropical faunal region from Costa Rica south to Peru, east to Trinidad, and throughout the Lesser Antilles. The genus, once included in *Alisotrichia* as the *dominicensis* species group, was originally placed in Stactobiinae but has since been transferred to Leucotrichiinae (Bowles et al. 1999). Flint et al. (1994) mentioned in the original description that the tarsal formula of *Cerasmatrichia* (1, 3, 4) differs from the rest of *Alisotrichia* in that no members of the latter genus bear a fore-tibial spur. The larvae of *C.spinosa* were described by Flint et al. (1994).

***adunca*** (Flint, 1991b): 44 [type locality: Colombia, Dpto. Antioquia, 10 km E Medellín, road to Guarne; NMNH; ♂; in *Alisotrichia*, not *Rioptila* as indicated in Oláh and Johanson 2011: 248]. —Flint et al. 1994: 377 [♂; ♀; to *Cerasmatrichia*]. —Muñoz-Quesada 2000: 277 —Oláh and Johanson 2011: 148 [distribution].

**Distribution.** —Colombia, Peru.

***akanthos*** Armitage & Harris, 2020a: 3 [type locality: Panama, Coclé Province, Cuenca 134, Omar Torrijos Herrera National Park, Quebrada La Yayas, PSPSCB-PNGDOTH-C134-2017-004, 8.66168°N, 80.5952°W, 602 m; COZEM; ♂].

**Distribution.** —Panama.

***argylensis*** Flint, Harris, & Botosaneanu, 1994: 370 [type locality: Tobago, St. Paul Parish, Argyle River at Argyle Waterfall; ZMUA; ♂; ♀]. —Botosaneanu 2002b: 82 [checklist].

—Hydroptilid genus, sp. 2: Botosaneanu and Sakal 1992: 201. —Botosaneanu and Alkins-Koo 1993: 14 [distribution]. —Flint et al. 1994: 370 [to synonymy].

**Distribution.** —Tobago, Trinidad.

***blahniki*** Harris & Armitage, 2019: 8 [type locality: Panama, Bocas del Toro Province, Quebrada Rambala, near Rambala Jungle Lodge, 3.74 km SSE Rambala, 8.91672°N and 82.15469°W, 120 m; COZEM; ♂].

**Distribution.** —Panama.

***dominicensis*** (Flint, 1968a): 44 [type locality: Dominica, 2.2 mi E Pont Casse; NMNH; ♂; in *Alisotrichia*]. —Flint 1968a: 81 [checklist]. —Botosaneanu 1989: 97 [distribution]. —Flint and Sykora 1993: 49 [checklist]. —Flint et al. 1994: 369 [♂; ♀; to *Cerasmatrichia*]. —Botosaneanu 2000: 256 [distribution]. —Botosaneanu 2002b: 823[checklist]. —Botosaneanu and Thomas 2005: 38 [probable; distribution].

—Ochrotrichia (O.) species, Flint and Sykora 1993: 58 [misidentification]. —Flint et al. 1994: 369 [to synonymy].

**Distribution.** —Dominica, Guadeloupe, Martinique.

***fulika*** Oláh & Johanson, 2011: 148 [type locality: French Guiana, Approuaguekaw, Kaw Mt., 4°33.035'N, 52°11.661'W, 104 m; NHRS; ♂].

**Distribution.** —French Guiana.

***hidala*** Oláh & Johanson, 2011: 150 [type locality: Peru, San Martin Prov., creek crossing rd. Tarapoto-Yurimaguas, ca. 30 km (rd.) NE Tarapoto, 6°24.904'S, 76°18.756'W; NHRS; ♂].

**Distribution.** —Peru.

***spinosa*** Flint, Harris, & Botosaneanu, 1994: 368 [type locality: Venezuela, Edo. Aragua, Rio El Limón, fish hatchery, Maracay; NMNH; ♂; ♀; larva; case]. —Flint 1981: 26 [in part, misidentification of *Alisotrichiawirthi* material from Rio El Limón].

**Distribution.** —Venezuela.

***trinitatis*** Flint, Harris, & Botosaneanu, 1994: 374 [type locality: Trinidad, St. George County, Northern Range, Maracas Waterfall; ZMUA; ♂; ♀]. —Flint 1996b: 90 [distribution]. —Botosaneanu 2000: 83 [checklist].

—Hydroptilid genus, sp. 1: Botosaneanu and Sakal 1992: 201. —Botosaneanu and Alkins-Koo 1993: 14 [distribution]. —Flint et al. 1994: 374 [to synonymy].

**Distribution.** —Trinidad, Venezuela.

***wirthi*** (Flint, 1968a): 46 [type locality: Dominica, Fond Figues River; NMNH; ♂; in *Alisotrichia*]. —Flint et al. 1994: 374 [♂; ♀; to *Cerasmatrichia*]. —Botosaneanu 2000: 83 [checklist]. —Botosaneanu and Thomas 2005: 38 [probable; distribution]. —Armitage et al. 2016: 6 [distribution]. —Armitage and Harris 2018b: 97 [checklist]. —Harris and Armitage 2019: 4 [distribution].

**Distribution.** —Dominica, Guadeloupe, Martinique [?], Panama, Venezuela.

##### Genus *Mejicanotrichia* Harris & Holzenthal, 1997

*Mejicanotrichia* Harris & Holzenthal, 1997: 129 [type species: *Alisotrichiablantoni* Flint, 1970, original designation]. —Bueno-Soria and Barba-Álvarez 1999b: 122 [key to males]. —Bowles et al. 1999: 51 [taxonomic position]. —Oláh and Johanson 2011: 142 [placement in *Celaenotrichia* genus cluster]. —Santos et al. 2016a: 471 [phylogenetic placement].

*Mejicanotrichia* consists of seven species occurring in the Neotropical faunal region. The genus was once included in *Alisotrichia* as the *blantoni* species group (Harris and Holzenthal 1997). The modified male forewings and features of the male genitalia separate *Mejicanotrichia* from the rest of *Alisotrichia* (Harris and Holzenthal 1997). Wiggins (1977) described the larval stage based on individuals identified as ‘*Alisotrichia* species’, which were later synonymized with *M.estaquillosa* by Harris and Holzenthal (1993).

***blantoni*** (Flint, 1970): 28 [type locality: Mexico, San Luis Potosi, Rancho Quemado, 3.5 mi S Tamazunchale; NMNH; ♂; in *Alisotrichia*]. —Bueno-Soria and Flint 1978: 200 [distribution]. —Harris and Holzenthal 1997: 131 [♂; ♀; re-description; to M*ejicanotrichia*]. —Bowles et al. 1999: 46 [larva].

**Distribution.** —Mexico.

***estaquillosa*** Harris & Holzenthal, 1997: 135 [type locality: Mexico, Nuevo Leon, Mpio. de Santiago, Cola de Caballo below falls, 3 km SW Cienquilla; NMNH; ♂; ♀]. —Bowles et al. 1999: 47 [larva].

—*Alisotrichia* species, Wiggins 1996: 80 [larva]. —Harris and Holzenthal 1997: 47 [to synonymy].

**Distribution.** —Mexico.

***harrisi*** Bueno-Soria & Barba-Álvarez, 1999a: 118 [type locality: Mexico, Guerrero, Municipio de Taxco, Teusisapan, Río Temascalapa, 18°25.083'N, 99°41.490'W; CNIN; ♂].

**Distribution.** —Mexico.

***rara*** Bueno-Soria & Barba-Álvarez, 1999a: 118 [type locality: Mexico, Guerrero, Municipio de Taxco, Teusisapan, Río Temascalapa, 18°25.083'N, 99°41.490'W; CNIN; ♂].

**Distribution.** —Mexico.

***tamaza*** (Flint, 1970): 28 [type locality: Mexico, Oaxaca, Tamazulapan; NMNH; ♂; in *Alisotrichia*]. —Bueno-Soria and Flint 1978: 200 [distribution]. —Harris and Holzenthal 1997: 133 [♂; ♀; re-description; to *Mejicanotrichia*].

**Distribution.** —Mexico.

***tridentata*** (Bueno-Soria & Hamilton, 1986): 301 [type locality: Mexico, Chiapas, tributario del Río Teapa, 3 km N Ixhuatan; NMNH; ♂; in *Alisotrichia*]. —Harris and Holzenthal 1997: 134 [♂; ♀; re-description; to *Mejicanotrichia*].

**Distribution.** —Mexico.

***trifida*** (Flint, 1970): 29 [type locality: Guatemala, Izabal, Las Escobas near Matias de Galvez; NMNH; ♂; in *Alisotrichia*]. —Harris and Holzenthal 1997: 134 [♂; re-description; to *Mejicanotrichia*].

**Distribution.** —Guatemala.

##### Genus *Scelobotrichia* Harris & Bueno-Soria, 1993

*Scelobotrichia* Harris & Bueno-Soria, 1993: 75 [type species: *Scelobotrichiacontrerasi* Harris & Bueno-Soria, 1993, original designation]. —Bowles et al. 1999: 47 [larva; taxonomic remarks]. —Oláh and Johanson 2011: 142 [placement in *Celaenotrichia* genus cluster].

The genus *Scelobotrichia* contains three species occurring in Mexico. The genus was once included in *Alisotrichia* as the *quemada* species group (Harris and Bueno-Soria 1993). The enlarged basal antennal segment and the unique lobe on the fore-tibia separate *Scelobotrichia* from the rest of *Alisotrichia* (Harris and Bueno-Soria 1993). It was originally placed in Stactobiinae by Harris and Bueno-Soria (1993) and was then transferred to Leucotrichiinae by Bowles et al. (1999). Descriptions of the larvae of *S.contrerasi* and *S.profunda* were given by Bowles et al. (1999).

***contrerasi*** Harris & Bueno-Soria, 1993: 77 [type locality: Mexico, Nuevo Leon, Municipio de Santiago, roadside waterfall near Cola de Caballo, 3 km SW Cieneguilla; NMNH; ♂; ♀]. —Bowles et al. 1999: 47 [larva].

**Distribution.** —Mexico.

***profunda*** Harris & Bueno Soria, 1993: 78 [type locality: Mexico, Guerrero, Rio en Barranca, Ruta Taxco-Telolapan; CNIN; ♂; ♀]. —Bowles et al. 1999: 48 [larva].

**Distribution.** —Mexico.

***quemada*** (Flint, 1970): 28 [type locality: Mexico, San Luis Potosi, Rancho Quemado, Rt. 85, 6 km S Tamazunchale; NMNH; ♂; in *Alisotrichia*]. —Harris and Bueno 1993: 80 [♂, to *Scelobotrichia*].

**Distribution.** —Mexico.

#### Tribe LEUCOTRICHIINI Flint, 1970

Leucotrichiini Flint, 1970: 2 [type genus: *Leucotrichia* Mosely, 1934a]. —Marshall 1979b: 175 [referred to as the *Leucotrichia* group]. —Oláh and Johanson 2011: 152 [referred to as the *Leucotrichia* genus cluster]. —Santos et al. 2016a: 472 [resurrection sensu novo; phylogenetic analysis].

Originally established as Leucotrichiinae for five genera (Flint 1970), including the now-excluded Alisotrichia, Leucotrichiini sensu novo currently contains ten genera. These genera were placed together in the *Leucotrichia* genus cluster based on a character state assessment with no statistical analysis (Oláh and Johanson 2011), and were recovered with strong support as a monophyletic unit in a phylogenetic analysis performed with both morphological and molecular data (Santos et al. 2016a). Males of these genera all bear a median complex on the phallus that is unique to the group and has been considered a unifying feature. Santos et al. (2016a) also identified several additional synapomorphies, including features found on the larvae, pupae, and both male and female genitalia.

##### Genus *Acostatrichia* Mosely, 1939

*Acostatrichia* Mosely, 1939a: 228 [type species: *Acostatrichiaplaumanni* Mosely, 1939a, original designation]. —Marshall 1979b: 182 [generic review]. —Angrisano and Sganga 2010: 56 [immatures; biology]. —Oláh and Johanson 2011: 152 [placement in *Leucotrichia* genus cluster]. —Santos et al. 2016a: 458 [phylogeny]. —Santos 2020: 202 [generic review].

*Acostatrichia* consists of 15 species distributed through much of South America. The larva of *A.simulans* was described by Angrisano and Sganga (2010), however, the female is still unknown. Both Mosely (1939a) and Marshall (1979b) noted that *Acostatrichia* is most similar to the genus *Costatrichia*, differing only in the wing venation and the unmodified antennae.

***araca*** Santos & Pes in Santos 2020: 204 [type locality: Brazil, Amazonas, base Serra do Aracá, Igarapé da Cobra, 00°52'34"N, 63°27'04"W; INPA; ♂].

**Distribution.** —Brazil.

***brevipenis*** Flint, 1974b: 54 [type locality: Suriname, Lawa River, Anapaike; RMNH; ♂]. —Flint 1991a: 69 [distribution]. —Angrisano 1999: 31 [checklist]. —Oláh and Johanson 2011: 156 [distribution]. —Paprocki and França 2014: 40 [checklist]. —Santos 2020: 213 [♂; distribution].

**Distribution.** —Brazil, French Guiana, Suriname.

***buborektala*** Oláh & Johanson, 2011: 155 [type locality: Peru, San Martin Prov., Rio Huallaga, at Pumarihri Huallaga Lodge, between Juan Guerra and Chazuta, 14 km (rd.) W Chazuta, 6°36.643'S, 76°12.555'W; NHRS; ♂]. —Oláh and Flint 2012: 143 [distribution]. —Santos 2020: 213 [♂].

**Distribution.** —Brazil, Peru.

***cerna*** Oláh & Flint, 2012: 143 [type locality: Ecuador, Los Rios Province, Quevedo (56 km North), 250 m, Rio Palenque Biological Station; NMNH; ♂]. —Ríos-Touma et al. 2017: 9 [checklist]. —Santos 2020: 219 [♂].

**Distribution.** —Ecuador.

***darda*** Oláh & Flint, 2012: 145 [type locality: Peru, Cuscu Department, Pilcopata, 600 m, premontane moist forest; NMNH; ♂]. —Santos 2020: 221 [♂].

— *hosulaba* Oláh & Flint, 2012: 147 [type locality: Ecuador, Pastaza Province, Puyo (1.5 km South); NMNH; ♂]. —Ríos-Touma et al. 2017: 9 [checklist]. —Santos 2020: 221 [to synonymy].

— *pika* Oláh & Flint, 2012: 151 [type locality: Ecuador, Pichincha Province, Santo Domingo de los Colorados, 14 km East; NMNH; ♂]. —Ríos-Touma et al. 2017: 9 [checklist]. —Santos 2020: 221 [to synonymy].

**Distribution.** —Ecuador, Peru.

***digitata*** Thomson & Holzenthal, 2012: 21 [type locality: Venezuela, Bolívar, E Tumeremo, W Bochinche, Río Botonamo, 07°25.462'N, 61°14.318'W, 150 m; UMSP; ♂]. —Santos 2020: 216 [review].

**Distribution.** —Venezuela.

***elvesta*** Oláh & Flint, 2012: 16 [type locality: Brazil, Rondonia State, creek, 8 km South Cacaulandia; NMNH; ♂]. —Santos 2020: 205 [review].

**Distribution.** —Brazil.

***fimbriata*** Flint, 1974b: 53 [type locality: Suriname, Coppename River, Raleigh Falls; RMNH; ♂]. —Santos 2020: 216 [♂].

**Distribution.** —Suriname.

***fluminensis*** (Santos & Nessimian, 2010b): 840 [type locality: Brazil, Rio de Janeiro, Mangaratiba, Reserva Ecológica Rio das Pedras, 22°59'29.4"S 44°06'02.6"W; DZRJ; ♂; in *Costatrichia*]. —Paprocki and França 2014: 43 [checklist]. —Santos et al. 2016a: 472 [to *Acostatrichia*]. —Santos 2020: 205 [♂; distribution].

**Distribution.** —Brazil.

***kihara*** Oláh & Flint, 2012: 150 [type locality: Ecuador, Napo Province, Pano, at stream, 580 m; NMNH; ♂]. —Ríos-Touma et al. 2017: 9 [checklist]. —Santos 2020: 223 [♂].

**Distribution.** —Ecuador, Venezuela.

***plaumanni*** Mosely, 1939a: 228 [type locality: Brazil, Edo. Santa Catarina, Nova Teutonia; NHMUK; ♂]. —Angrisano 1995a: 505 [distribution]. —Angrisano 1999: 31 [checklist]. —Paprocki et al. 2004: 10 [checklist]. —Manzo et al. 2014: 66 [distribution]. —Paprocki and França 2014: 40 [checklist]. —Santos et al. 2016a: 466 [♂, head]. —Santos 2020: 208 [♂].

**Distribution.** —Argentina, Brazil, Uruguay.

***simulans*** Mosely, 1939a: 229 [type locality: Brazil, Edo. Santa Catarina, Nova Teutonia; NHMUK; ♂]. —Angrisano 1995a: 505 [distribution]. —Angrisano 1999: 31 [checklist]. —Paprocki et al. 2004: 10 [checklist]. —Angrisano and Sganga 2010: 56 [distribution; larva, case, pupa]. —Paprocki and França 2014: 40 [checklist]. —Santos 2020: 209 [♂; distribution].

**Distribution.** —Argentina, Brazil, Uruguay.

***spinifera*** Flint, 1974b: 53 [type locality: Suriname, Nickerie River, Lombok Falls; RMNH; ♂]. —Santos 2020: 210 [review].

**Distribution.** —Suriname.

***tuskera*** Oláh & Flint, 2012: 157 [type locality: Brazil, São Paulo State, Piracicaba; NMNH; ♂]. —Santos 2020: 217 [♂].

**Distribution.** —Brazil.

***ujasa*** Oláh & Flint, 2012: 158 [type locality: Ecuador, Pastaza Province, Puyo (27 km North), Estacion Fluviometrica; NMNH; ♂]. —Ríos-Touma et al. 2017: 9 [checklist]. —Santos 2020: 225 [♂; distribution].

**Distribution.** —Ecuador, Peru.

##### Genus *Anchitrichia* Flint, 1970

*Anchitrichia* Flint, 1970: 14 [type species: *Anchitrichiaspangleri* Flint, 1970, original designation]. —Marshall 1979b: 181 [generic review]. —Pes and Hamada 2004: 31 [new records]. —Oláh and Johanson 2011: 152 [placement in *Leucotrichia* genus cluster].

The eight species currently contained within the genus *Anchitrichia* occur throughout Central America and extend south to Argentina. Marshall (1979b) commented that, while *Anchitrichia* may prove to be synonymous with one or more of the other genera of Leucotrichiinae, the genus may be very closely related to *Zumatrichia*. Members can be distinguished by several adult morphological features, including the relatively larger body size, the unmodified antennae, and the general form of the male genitalia. The larvae of *A.spangleri* were described by Flint (1970) and those of *A.duplifurcata* by Guahyba (1991).

***agaboga*** Oláh & Flint, 2012: 161 [type locality: Ecuador, Cotopaxi Province, Latacunga, 133 km West, 1080 m; NMNH; ♂]. —Ríos-Touma et al. 2017: 9 [checklist].

**Distribution.** —Ecuador.

***carolae*** Oláh & Flint, 2012: 163 [type locality: Venezuela, Barinas State, Rio Santo Domingo, Barinas; NMNH; ♂].

**Distribution.** —Venezuela.

***duplifurcata*** Flint, 1983: 36 [type locality: Paraguay, Dpto. Amambay, 2 km S Cerro Cora; NMNH; ♂]. —Guahyba 1991: 121 [larva; pupa; case]. —Angrisano 1999: 31 [checklist]. —Blahnik et al. 2004: 4 [distribution]. —Paprocki et al. 2004: 10 [checklist]. —Dumas et al. 2009: 365 [distribution]. —Paprocki and França 2014: 41 [checklist]. —Santos et al. 2016a: 464 [larva; pupa].

**Distribution.** —Brazil, Paraguay.

***harrisi*** Oláh & Flint, 2012: 164 [type locality: Venezuela, Zulia State, El Tucuco, Sierra de Perija, montane forest; NMNH; ♂].

**Distribution.** —Colombia, Venezuela.

***holzenthali*** Oláh & Flint, 2012: 166 [type locality: Ecuador, Napo Province, Rio Jondachi, 30 km North Tena; NMNH; ♂]. —Ríos-Touma et al. 2017: 9 [checklist].

**Distribution.** —Ecuador.

***palmatiloba*** Flint, 1991b: 38 [type locality: Colombia, Dpto. Antioquia, Río Aurrá, km 50, E San Jerónimo; NMNH; ♂]. —Ríos-Touma et al. 2017: 9 [distribution].

**Distribution.** —Colombia, Ecuador, Venezuela.

***spangleri*** Flint, 1970: 14 [type locality: Mexico, Chiapas, Arriaga; NMNH; ♂; larva; case]. —Bueno-Soria and Flint 1978: 200 [distribution]. —Holzentha 1988: 60 [distribution]. —Aguila 1992: 537 [distribution]. —Chamorro-Lacayo et al. 2007: 42 [checklist; distribution]. —Armitage et al. 2015a: 6 [checklist]. —Armitage and Harris 2018b: 97 [checklist].

**Distribution.** —Costa Rica, Guatemala, Honduras, Mexico, Nicaragua, Panama.

***trifurcata*** Angrisano, 1984: 4 [type locality: Argentina, Salta, Parque Nacional Baritú; MACN; ♂]. —Angrisano 1999: 31 [checklist]. —Oláh and Johanson 2011: 156 [distribution]. —Oláh and Flint 2012: 167 [distribution].

**Distribution.** —Argentina, Peru.

##### Genus *Ascotrichia* Flint, 1983

*Ascotrichia* Flint, 1983: 35 [type species: *Ascotrichiafrontalis* Flint, 1983, original designation]. —Oláh and Johanson 2011: 152 [placement in *Leucotrichia* genus cluster]. —Thomson 2019: 7 [generic revision].

*Ascotrichia* is a small genus of six species occurring in eastern South America. When establishing the genus, Flint (1983) stated that it clearly belongs in Leucotrichiinae and that it is most closely related to *Peltopsyche*, but could be easily distinguished by differences in the head, antennae, and forewings. The larvae are unknown.

***adirecta*** Thomson, 2019: 9 [type locality: Brazil, Minas Gerais, confluence Rio Peixe & Rio Preto do Itambé, 19°17.525'S, 43°15.547'W, 500 m; MZUSP; ♂].

**Distribution.** —Brazil.

***frontalis*** Flint, 1983: 36 [type locality: Paraguay, Dpto. Alto Paraná, Salto del Monday, near Puerto Presidente Franco; NMNH; ♂]. —Angrisano 1995a: 505 [distribution]. —Angrisano 1999: 31 [checklist]. —Paprocki et al. 2004: 10 [checklist]. —Dumas et al. 2009: 366 [distribution]. —Oláh and Johanson 2011: 157 [distribution]. —Paprocki and França 2014: 41 [checklist]. —Santos et al. 2016a: 465 [adult photograph]. —Thomson 2019: 8 [♂; distribution].

**Distribution.** —Brazil, Paraguay, Uruguay.

***hystricosa*** Thomson, 2019: 11 [type locality: Brazil, Minas Gerais, Serra do Cipó, Rio Cipó in Cardeal Mota, 19°21.011'S, 43°38.171'W, 720 m; MZUSP; ♂].

**Distribution.** —Brazil.

***simoma*** Thomson, 2019: 13 [type locality: Brazil, São Paulo, Estação Biológica Boraceia, Rio Guaratuba, 23°40.039'S, 45°53.759'W, 775 m; MZUSP; ♂].

**Distribution.** —Brazil.

***spangleri*** Oláh & Flint, 2012: 167 [type locality: Venezuela, Amazonas Federal Territory, Puerto Ayacucho (40 km South), El Tobogan, Cano Coromoto; NMNH; ♂]. —Thomson 2019: 15 [♂].

**Distribution.** —Venezuela.

***surinamensis*** (Flint, 1974b): 57 [type locality: Suriname, Nickerie River, Blanche Marie; RMNH; ♂; in *Betrichia*]. —Flint 1983: 36 [to *Ascotrichia*]. —Oláh and Johanson 2011: 157 [distribution]. —Thomson 2019: 17 [♂].

**Distribution.** —French Guiana, Guyana, Suriname.

##### Genus *Betrichia* Mosely, 1939

*Betrichia* Mosely, 1939a: 230 [type species: *Betrichiazilbra* Mosely, 1939a, original designation]. —Marshall 1979b: 182 [generic review]. —Oláh and Johanson 2011: 152 [placement in *Leucotrichia* genus cluster]. —Santos et al. 2016a: 472 [phylogenetic position; larva photograph].

*Betrichia* is a genus consisting of ten species distributed through eastern South America. The larval stages are unknown. As species have been added to *Betrichia*, the characters states originally given to define the genus have proven instead to be specific; there are no precise diagnostic characteristics that can be used to clearly distinguish members of the genus from other genera of Leucotrichiinae (Marshall 1979b).

***argentinica*** Flint, 1972b: 232 [type locality: Argentina, Prov. Misiones, Capiovi; NMNH; ♂]. —Angrisano 1995a: 505 [distribution]. —Angrisano 1999: 32 [checklist]. —Thomson 2012: 2 [checklist].

**Distribution.** —Argentina, Uruguay.

***bispinosa*** Flint, 1974b: 59 [type locality: Suriname, Lawa River, Anapaike; RMNH; ♂]. —Thomson 2012: 2 [checklist]. —de Souza et al. 2016a: 295 [distribution].

**Distribution.** —Brazil, Suriname.

***kagyla*** Oláh & Flint, 2012: 170 [type locality: Brazil, Amazonas State, Igarape Tarumanzinho, near Manaus; MZUSP; ♂].

**Distribution.** —Brazil.

***longistyla*** Flint, 1983: 38 [type locality: Brazil, Edo. Santa Catarina, Nova Teutonia; NMNH; ♂]. —Angrisano 1999: 32 [checklist]. —Paprocki et al. 2004: 10 [checklist]. —Thomson 2012: 2 [checklist]. —Paprocki and França 2014: 42 [checklist].

**Distribution.** —Brazil.

***nhundiaquara*** de Souza, Santos, & Takiya, 2016a: 291 [type locality: Brazil, Paraná, Morretes, Rio Nhundiaquara, 25°25'25"S 48°54'0"W, 89 m; DZRJ; ♂]. —Moreno et al. 2020: 265 [distribution].

**Distribution.** —Brazil.

***occidentalis*** Flint, 1974b: 60 [type locality: Suriname, Blanche Marie, falls in creek; RMNH; ♂]. —Oláh and Johanson 2011: 159 [distribution]. —Thomson 2012: 2 [checklist].

**Distribution.** —French Guiana, Suriname.

***rovatka*** Oláh & Johanson, 2011: 159 [type locality: French Guiana, Roura, Cacao, 4°33.639'N, 52°24.629'W, 66 m; NHRS; ♂]. —Oláh and Flint 2012: 171 [distribution]. —Thomson 2012: 2 [checklist]. —Ríos-Touma et al. 2017: 9 [checklist].

**Distribution.** —Ecuador, French Guiana.

***uruguayensis*** Angrisano, 1995a: 505 [type locality: Uruguay, Paysandu, Sta. Rita, Puerto Pepeaji; FHCU; ♂]. —Angrisano 1999: 32 [checklist]. —Thomson 2012: 2 [checklist]. —Oláh and Flint 2012: 171 [distribution].

**Distribution.** —Brazil, Uruguay.

***varratlana*** Oláh & Flint, 2012: 171 [type locality: Brazil, Rondonia State, creek 8 km South Cacaulandia; NMNH; ♂].

**Distribution.** —Brazil, Guyana.

***zilbra*** Mosely, 1939a: 231 [type locality: Brazil, Edo. Santa Catarina, Nova Teutonia; NHMUK ♂]. —Angrisano 1995a: 505 [distribution]. —Angrisano 1999: 32 [checklist]. —Paprocki et al. 2004: 10 [checklist]. —Oláh and Flint 2012: 173 [distribution]. —Thomson 2012: 2 [checklist]. —Paprocki and França 2014: 42 [checklist]. —de Souza et al. 2016a: 293 [♂; distribution].

**Distribution.** —Argentina, Brazil, Guyana, Uruguay.

##### Genus *Ceratotrichia* Flint, 1992

*Ceratotrichia* Flint, 1992b: 527 [type species: *Ceratotrichiafairchildi* Flint, 1992b, original designation]. —Pes and Hamada 2004: 31 [larva; pupa; taxonomic remarks; distribution]. —Oláh and Johanson 2011: 152 [placement in *Leucotrichia* genus cluster].

The genus *Ceratotrichia* currently contains five species recorded from Panama, northern South America, Bolivia, and Brazil. In the original description, Flint (1992b) stated that *Ceratotrichia* is most closely related to *Zumatrichia*, in that the two genera shared the male reduction of ocelli, a basic wing venation, and the general structure of both male and female genitalia. He also stated that the male secondary sexual modifications are quite different between the two genera: the modifications to the antennae occur on different segments, *Ceratotrichia* lacks the deep indentation on the head present in *Zumatrichia*, and *Zumatrichia* lacks the specialized brushes and patches of hairs present on the forewings of *Ceratotrichia*. The larvae of an unidentified species of *Ceratotrichia* were described by Pes and Hamada (2004).

***balra*** Oláh & Johanson, 2011: 157 [type locality: Bolivia, Hung. Soil. Exp. II, S. Amer. No.B-B: No.493, Alcoche (La Paz), surroundings of Hotel, 600 m; HNHM; ♂].

**Distribution.** —Bolivia.

***fairchildi*** Flint, 1992b: 528 [type locality: Panama, Comarca of San Blas, Quebrada Pingandi, 9 km N Nusagandi; NMNH; ♂]. —Aguila 1992: 537 [distribution]. —Armitage et al. 2015a: 6 [checklist]. —Armitage and Harris 2018b: 97 [checklist].

**Distribution.** —Panama.

***felgorba*** Oláh & Flint, 2012: 173 [type locality: Ecuador, Napo Province, Pano, at stream, 580 m; NMNH; ♂]. —Ríos-Touma et al. 2017: 9 [checklist].

**Distribution.** —Ecuador.

***flavicoma*** Flint, 1992b: 529 [type locality: Venezuela, State of Barinas, Puente Parangula, 8 km S Barinitas; NMNH; ♂]. —Flint 1996c: 396 [distribution]. —Ríos-Touma et al. 2017: 9 [checklist].

**Distribution.** —Ecuador, Peru, Venezuela.

***jobbra*** Oláh & Flint, 2012: 174 [type locality: Ecuador, Manabi Province, 29 km West Santo Domingo, Rancho Ronald; NMNH; ♂]. —Ríos-Touma et al. 2017: 9 [checklist].

**Distribution.** —Ecuador.

##### Genus *Costatrichia* Mosely, 1937

*Costatrichia* Mosely, 1937b: 166 [type species: *Costatrichialodora* Mosely, 1937b, original designation]. —Flint 1970: 11 [revision]. —Marshall 1979b: 181 [generic review]. —Holzenthal and Harris 1999: 540 [revision; key to species]. —Oláh and Johanson 2011: 152 [placement in *Leucotrichia* genus cluster].

The genus *Costatrichia* consists of 20 species distributed from Mexico through Central America and south to southeastern South America. Flint (1970) separated *Costatrichia* from *Zumatrichia* based on the presence of three ocelli in males and the generally unmodified antennal segments of the former. Marshall (1979b) asserted that it was not possible to define this genus satisfactorily and that some members would key out with other genera. The genus was divided by Holzenthal and Harris (1999) into two species groups (*simplex* and *lodora*) based on adult features present on the head, wings, and male genitalia. The larval stage is unknown.

***bipartita*** Flint, 1970: 12 [type locality: Nicaragua, Chontales, Puente Quinama, near Villa Somoza; NMNH; ♂]. —Maes and Flint 1988: 4 [distribution]. —Holzenthal and Harris 1999: 564 [♂]. —Maes 1999: 1193 [checklist]. —Chamorro-Lacayo et al. 2007: 42 [checklist].

**Distribution.** —Nicaragua.

***carara*** Holzenthal & Harris, 1999: 552 [type locality: Costa Rica, San José, Reserva Biológica Carara, Río del Sur, 1.5 km (rd) S Carara, 9.769°N, 84.531°W; NMNH; ♂; ♀].

**Distribution.** —Costa Rica.

***cressae*** Holzenthal & Harris, 1999: 555 [type locality: Venezuela, Distrito Federal, Río Camuri Grande, 1 km S Camuri (nucleo U.S.B.), 10.616°N, 66.175°W; NMNH; ♂; ♀].

**Distribution.** —Venezuela.

***devestiva*** Thomson & Armitage, 2018: 2 [type locality: Panama, Chiriquí Province, Cuenca 102 (Río Chiriquí Viejo), Quebrada Norte, Mount Totumas Biological Reserve, 8.873613°N, 82.690512°W; COZEM; ♂].

**Distribution.** —Panama.

***dietrichi*** Thomson & Armitage, 2018: 3 [type locality: Panama, Chiriquí Province, Cuenca 102 (Río Chiriquí Viejo), Quebrada Norte, Mount Totumas Biological Reserve, 8.873613°N, 82.690512°W; COZEM; ♂]. —Harris and Armitage 2019: 4 [distribution].

**Distribution.** —Panama.

***flinti*** Holzenthal & Harris, 1999: 545 [type locality: Costa Rica, Puntarenas, Río Singrí, ca. 2 km (air) S Finca Helechales, 9.057°N, 83.082°W; NMNH; ♂].

**Distribution.** —Costa Rica.

***hamulifera*** (Flint, 1983): 38 [type locality: Argentina, Pcia. Entre Rios, Rio Uruguay, Salto Grande; NMNH; ♂; in *Betrichia*]. —Angrisano 1995a: 505 [distribution]. —Angrisano 1999: 32 [checklist]. —Angrisano and Sganga 2007: 30 [♂; distribution]. —Calor 2011: 321 [checklist]. —Oláh and Johanson 2011: 159 [distribution]. —Oláh and Flint 2012: 170 [distribution]. —Thomson 2012: 2 [checklist]. —de Souza et al. 2013: 585 [distribution]. —Paprocki and França 2014: 42 [checklist]. —Santos et al. 2016a: 472 [to *Costatrichia*].

**Distribution.** —Argentina, Brazil, French Guiana, Paraguay, Uruguay.

***inaequalis*** Gama Neto, Ribeiro, & Passos, 2019: 386 [type locality: Brazil, Pará, Parauapebas municipality, Serra dos Carajás, low order stream, 6°2'24.828"S, 50°17'38.184"W; MPEG; ♂].

**Distribution.** —Brazil.

***ipixuna*** Santos, Takiya, & Nessimian, 2013: 448 [type locality: Brazil, Amazonas, Ipixuna, Rio Liberdade, Comunidade São Vicente, 07°21'47"S 71°52'07"W, 175 m; INPA; ♂; ♀]. —Paprocki and França 2014: 43 [checklist]. —Santos et al. 2016a: 466 [♂ antennae; ♀].

**Distribution.** —Brazil.

***lodora*** Mosely, 1937b: 168 [type locality: Mexico, Chiapas, Dolores; NHMUK; ♂]. —Flint 1970: 12 [♂; distribution]. —Bueno-Soria and Flint 1978: 200 [distribution]. —Holzenthal 1988: 60 [distribution]. —Holzenthal and Harris 1999: 541 [♂; distribution]. —Bueno-Soria et al. 2005: 75 [checklist]. —Chamorro-Lacayo et al. 2007: 42 [checklist].

**Distribution.** —Costa Rica, Mexico, Nicaragua.

***nelsonferreirai*** Santos & Nessimian, 2010b: 838 [type locality: Brazil, Pará, Canaã dos Carajás, Floresta Nacional - FLONA - de Carajás, lagoa Redonda, 06°21'20.7"S 50°23'26.7"W, 705 m; DZRJ; ♂; ♀]. —Paprocki and França 2014: 43 [checklist].

— *ketvilla* (Oláh & Flint, 2012): 149 [type locality: Brazil, Pará State, Rio Xingu Camp, circa. 60 km South Altamira, 52°22'W, 3°39'S; MZUSP; ♂; in *Acostatrichia*]. —Santos 2020: 227 [to synonymy].

**Distribution.** —Brazil.

***noite*** Angrisano, 1995a: 507 [type locality: Uruguay, Tacuarembo, Ao. Laureles, Rincón de la Vasoura; FHCU; ♂]. —Angrisano 1999: 32 [checklist]. —Holzenthal and Harris 1999: 540, 564 [♂; distribution]. —Oláh and Flint 2012: 176 [distribution]. —Santos et al. 2013: 450 [distribution]. —Paprocki and França 2014: 43 [checklist]. —Ríos-Touma et al. 2017: 9 [checklist].

**Distribution.** —Brazil, Ecuador, Paraguay, Peru, Uruguay.

***panamensis*** Flint, 1967b: 11 [type locality: Panama, Canal Zone, Río Agua Salud; NMNH; ♂]. —Flint 1970: 12 [♂]. —Aguila 1992: 538 [distribution]. —Holzenthal and Harris 1999: 568 [♂]. —Armitage et al. 2015a: 6 [checklist]. —Armitage and Harris 2018b: 97 [checklist].

**Distribution.** —Panama.

***rovidka*** (Oláh & Flint, 2012): 153 [type locality: Guyana, Moco-Moco, 30 km East Lethem, 3°18.2'N, 59°39.0'W; NMNH; ♂; in *Acostatrichia*]. —Santos 2020: 228 [to *Costatrichia*].

**Distribution.** —Guyana.

***santosi*** Harris & Armitage, 2019: 9 [type locality: Panama, Bocas del Toro Province, Quebrada Rambala, near Rambala Jungle Lodge, 3.74 km SSE Rambala, 8.91627°N and 82.15469°W, 120 m; COZEM; ♂].

**Distribution.** —Panama.

***simplex*** Flint, 1970: 13 [type locality: El Salvador, San Salvador, Lake Ilopango, near Apulo; NMNH; ♂]. —Bueno-Soria and Flint 1978: 200 [distribution]. —Holzenthal 1988: 61 [distribution]. —Holzenthal and Harris 1999: 545 [♂; ♀; distribution]. —Chamorro-Lacayo et al. 2007: 42 [checklist].

**Distribution.** —Costa Rica, El Salvador, Honduras, Mexico, Nicaragua.

***spinifera*** Flint, 1970: 13 [type locality: Panama, Canal Zone, Río Agua Salud, Pipeline Road; NMNH; ♂]. —Aguila 1992: 538 [distribution]. —Holzenthal and Harris 1999: 558 [♂; distribution]. —Armitage et al. 2015a: 6 [checklist]. —Armitage and Harris 2018b: 97 [checklist].

**Distribution.** —Costa Rica, Panama.

***tapada*** (Oláh & Flint, 2012): 154 [type locality: Venezuela, Bolivar State, Rio Caroni at Paso Caruachi; NMNH; ♂; in *Acostatrichia*]. —Santos 2020: 229 [to *Costatrichia*].

**Distribution.** —Venezuela.

***tripartita*** Flint, 1970: 13 [type locality: Panama, Canal Zone, Río Agua Salud, Pipeline Road; NMNH; ♂]. —Aguila 1992: 538 [distribution]. —Holzenthal and Harris 1999: 549 [♂; ♀; distribution]. —Armitage et al. 2015a: 6 [checklist]. —Armitage and Harris 2018b: 97 [checklist].

**Distribution.** —Costa Rica, Panama.

***venezuelensis*** Flint, 1981: 25 [type locality: Venezuela, Aragua, Maracay Río Limón, Estación Piscicultura; NMNH; ♂; as subspecies of *tripartita*]. —Holzenthal and Harris 1999: 558 [new status; diagnosis; ♂; distribution]. —Oláh and Flint 2012: 176 [distribution]. —Armitage et al. 2016: 6 [distribution].

**Distribution.** —Costa Rica, Panama, Venezuela.

##### Genus *Leucotrichia* Mosely, 1934

*Leucotrichia* Mosely, 1934a: 157 [type species: *Leucotrichiamelleopicta* Mosely, 1934a, original designation]. —Ross 1944: 271 [key to males of Nearctic species]. —Flint 1970: 3 [key; revision]. —Marshall 1979b: 178 [revision]. —Blickle 1979: 7 [key to species of America north of Mexico]. —Flint 1991b: 39 [key to Antioquian species]. —Oláh and Johanson 2011: 152 [placement in *Leucotrichia* genus cluster]. —Thomson and Holzenthal 2015: 1 [generic revision; key to males]. —Santos et al. 2016a: 475 [assessment of monophyly].

The genus *Leucotrichia* consists of 46 species, including one fossil species known from Dominican amber. Its distribution includes most of the United States, Central and northern South America, the Greater Antilles, and the southernmost Lesser Antilles. Two main species groups were outlined by Flint (1970) based on adult features including ocelli number, the presence of head modifications, and the presence of a process or brush of setae on abdominal sternite VII. The larva of *L.pictipes* was first described as that of *Ithytrichiaconfusa*; the larvae and cases for many other species have since been described (Lloyd 1915; Wiggins 1996).

♰ ***adela*** Wells & Wichard, 1989: 42 [type locality: Dominican Republic; NMNH; ♂; in amber]. —Flint and Pérez-Gelabert 1999: 39 [checklist]. —Botosaneanu 2002b: 84 [checklist]. —Wichard 2007: 48 [checklist]. —Eskov et al. 2008: 78 [checklist]. —Pérez-Gelabert 2008: 300 [checklist]. —Thomson and Holzenthal 2015: 9 [♂].

**Distribution.** —Dominican amber.

***alibrachia*** (Thomson, 2012): 4 [type locality: Brazil, Rio de Janeiro, Resende, Ribeirás do Palmital, 22°25'26.2"S, 44°44'21.6"W, 969 m; DZRJ; ♂; in *Betrichia*]. —Paprocki and Franca 2014: 41 [checklist]. —Santos et al. 2016a: 472 [to *Leucotrichia*].

**Distribution.** —Brazil.

***alisensis*** Rueda Martín, 2011: 4 [type locality: Argentina, Tucamán, Parque Nacional Campo de Los Alisos, Río de las Pavas, S27°12'39", W65°55'39", 1655 m; IML; ♂; metamorphotype; larva, pupa]. —Thomson 2012: 2 [checklist]. —Thomson and Holzenthal 2015: 10 [♂]. —Isa Miranda and Rueda Martín 2014: 199 [distribution].

**Distribution.** —Argentina.

***angelinae*** Thomson & Holzenthal, 2015: 11 [type locality: Venezuela, Mérida, Cacuta, 10 km E Tabay; NMNH; ♂].

**Distribution.** —Venezuela.

***ayura*** Flint, 1991b: 41 [type locality: Colombia, Dpto. Antioquia, 12 km NW Medellín, road to San Pedro; NMNH; ♂]. —Muñoz-Quesada 2000: 278 [checklist]. —Thomson 2012: 2 [checklist]. —Thomson and Holzenthal 2015: 11 [♂].

**Distribution.** —Colombia.

***bicornuta*** Thomson, 2012: 4 [type locality: Brazil, Rio de Janeiro, Panedo, Rio das Pedras, Tres Bacias 22°24'32.2"S, 44°33'06.5"W, 735 m; DZRJ; ♂]. —Paprocki and França 2014: 45 [checklist]. —Thomson and Holzenthal 2015: 12 [♂].

**Distribution.** —Brazil.

***botosaneanui*** Flint, 1996b: 86 [type locality: Tobago, big waterfall 4 km S Charlotteville, 11°19'N, 60°33'W; NMNH; ♂]. —Botosaneanu and Sakal 1992: 201 [distribution; ecology; as *limpia*]. —Botosaneanu and Alkins-Koo 1993: 10 [larva; as *limpia*]. —Botosaneanu 2002b: 84 [checklist]. —Thomson 2012: 2 [checklist]. —Thomson and Holzenthal 2015: 13 [♂].

**Distribution.** —Tobago, Trinidad.

***brasiliana*** Sattler & Sykora, 1977: 239 [type locality: Brazil, Amazonas Staat, bereich des Rio Marauía, bei Tapuruquara, oberer Rio Negro; type depository unknown; ♂; larva; pupa; case]. —Paprocki et al. 2004: 11 [checklist]. —Thomson 2012: 2 [checklist]. —Paprocki and França 2014: 45 [checklist]. —Thomson and Holzenthal 2015: 14 [♂].

**Distribution.** —Brazil.

***brochophora*** Flint, 1991b: 41 [type locality: Colombia, Dpto. Antioquia, Quebrada Espadera, 7 km E Medellín, road to Sta. Elena; NMNH; ♂]. —Muñoz-Quesada 2000: 278 [checklist]. —Thomson 2012: 2 [checklist]. —Thomson and Holzenthal 2015: 15 [♂].

**Distribution.** —Colombia.

***chiriquiensis*** Flint, 1970: 6 [type locality: Panama, Chiriqui, Also Lino above Bouquet; NMNH; ♂; larva; case]. —Aguila 1992: 538 [distribution]. —Thomson 2012: 2 [checklist]. —Thomson and Holzenthal 2015: 16 [♂]. —Armitage et al. 2015a: 6 [checklist]. —Armitage and Harris 2018b: 97 [checklist].

**Distribution.** —Panama.

***denticulata*** Thomson & Holzenthal, 2015: 17 [type locality: Mexico, Nuevo Leon, Municipio de Santiago, Arroyo San Juan on road to Laguna de Sanchez, 3.5 km W La Cienegra, 25°24'N, 100°17'W, 1400 m; UMSP; ♂].

**Distribution.** —Mexico.

***dianeae*** Thomson & Holzenthal, 2015: 17 [type locality: Costa Rica, Cartago, Reserva Tapantí, waterfall, ca. 1 km (road) NW tunnel, 9.69°N, 83.76°W, 1600 m; UMSP; ♂].

**Distribution.** —Costa Rica.

***dinamica*** Bueno-Soria, 2010: 23 [type locality: Mexico, Distrito Federal, Delgación Magdalena-Contretas, Parque “Los Dinamos”, 3091 m; CNIN; ♂]. —Thomson 2012: 2 [checklist]. —Thomson and Holzenthal 2015: 18 [♂].

**Distribution.** —Mexico.

***extraordinaria*** Bueno-Soria, Santiago-Fragoso, & Barba Álvarez, 2001: 145 [type locality: Mexico, Tabasco, Municipio de Huimanguillo, Arroyo las Flores, Villa de Guadelupe 2^a^ sección Los Chimalapas, km 5 Ruta Malpasito-Carlos A. Madrazo, 17°22'05"N, 93°36'25"W; CNIN; ♂]. —Bueno-Soria et al. 2005: 75 [distribution]. —Thomson 2012: 2 [checklist] —Thomson and Holzenthal 2015: 19 [♂]. —Armitage et al. 2018: 5 [distribution]. —Harris and Armitage 2019: 4, 19 [distribution; ♂].

**Distribution.** —Panama, Mexico.

***fairchildi*** Flint, 1970: 10 [type locality: Panama, Cocle, El Valle; MCZ; ♂]. —Flint 1968b: 38 [♂; ♀; Grenada, but misidentified as *sarita*]. —Flint 1981: 25 [♂; distribution]. —Flint 1991b: 39 [♂; distribution]. —Aguila 1992: 538 [distribution]. —Botosaneanu and Sakal 1992: 201 [distribution; ecology]. —Flint and Sykora 1993: 54 [Grenada but misidentified as *sarita*]. —Botosaneanu and Alkins-Koo 1993: 7 [larva; case]. —Flint 1996b: 86 [distribution]. —Moulton and Stewart 1997: 350 [checklist]. —Muñoz-Quesada 2000: 278 [checklist]. —Botosaneanu 2002b: 84 [checklist]. —Botosaneanu and Viloria 2002: 106 [distribution]. —Thomson 2012: 2 [checklist]. —Thomson and Holzenthal 2015: 20 [♂]. —Armitage et al. 2015a: 6 [checklist]. —Ríos-Touma et al. 2017: 10 [checklist]. —Armitage and Harris 2018b: 97 [checklist]. —Harris and Armitage 2019: 4 [distribution].

—Leucotrichiini, case 2 Botosaneanu and Alkins-Koo, 1993: 14 [♀]. —Flint 1996: 86 [to synonymy].

**Distribution.** —Colombia, Costa Rica, Ecuador, El Salvador, Grenada, Panama, Tobago, Trinidad, Venezuela.

***falsa*** (Santos, Takiya, & Nessimian, 2013): 448 [type locality: Costa Rica, Puntarenas, La Gamba, Esquinas Lodge, river at waterfall trail, 08°41'05" 83°12'17"W, 70 m; INBIO; ♂; ♀; in *Costatrichia*]. —Santos et al. 2016a: 472 [to *Leucotrichia*]. —Armitage et al. 2020: 4 [distribution; as *Costatrichia*].

**Distribution.** —Costa Rica, Panama.

***forrota*** Oláh & Johanson, 2011: 160 [type locality: Peru, San Martín Province, Río Huallaga tributary, small river passing Chazuta, NHRS; ♂]. —Oláh and Flint 2012: 176 [distribution]. —Thomson 2012: 2 [checklist]. —Thomson and Holzenthal 2015: 22 [♂]. —Ríos-Touma et al. 2017: 10 [checklist].

**Distribution.** —Ecuador, Peru.

***fulminea*** Thomson & Holzenthal, 2015: 23 [type locality: Ecuador, Cañar, Río Chauchas, 3 km N Zhud; NMNH; ♂]. —Ríos-Touma et al. 2017: 10 [checklist].

**Distribution.** —Ecuador.

***gomezi*** Flint, 1970: 7 [type locality: Dominican Republic, La Palma, 12 km E El Rio; NMNH; ♂; larva, case]. —Flint and Pérez-Gelabert 1999: 39 [checklist]. —Botosaneanu 2002b: 84 [checklist]. —Flint and Sykora 2004: 32 [distribution]. —Pérez-Gelabert 2008: 300 [checklist]. —Thomson 2012: 2 [checklist]. —Thomson and Holzenthal 2015: 24 [♂].

**Distribution.** —Dominican Republic.

***hispida*** Thomson & Holzenthal, 2015: 25 [type locality: Costa Rica, San José, Río Savegre, 9°33.9'N, 83°48'W, 2270 m; NMNH; ♂].

**Distribution.** —Costa Rica.

***imitator*** Flint, 1970: 8 [type locality: Mexico, Vera Cruz, Plan del Rio Ver, Rt. 140, km 368; NMNH; ♂; larva; case]. —Bueno-Soria and Flint 1978: 200 [distribution]. —Holzenthal 1988: 61 [checklist]. —Bueno-Soria et al. 2007: 33 [distribution]. —Thomson 2012: 2 [checklist]. —Thomson and Holzenthal 2015: 26 [♂]. —Razo-González 2018: 32 [distribution].

**Distribution.** —Costa Rica, Guatemala, Mexico.

***inflaticornis*** Botosaneanu in Botosaneanu and Alkins-Koo 1993: 10 [type locality: Trinidad, 2^nd^. order stream at “La Laja”, catchment of Rio Guanapo; ZMUA; ♂; larva; case]. —Botosaneanu and Sakal 1992: 201 [distribution; ecology]. —Flint 1996b: 89 [distribution]. —Botosaneanu 2002b: 84 [checklist]. —Thomson 2012: 2 [checklist]. —Thomson and Holzenthal 2015: 27 [♂].

**Distribution.** —Trinidad.

***inops*** Flint, 1991b: 43 [type locality: Colombia, Dpto. Antioquia, 12 km E Medellín, road to Sta. Elenal NMNH; ♂]. —Muñoz-Quesada 2000: 278 [checklist]. —Thomson 2012: 2 [checklist]. —Thomson and Holzenthal 2015: 27 [♂; distribution].

**Distribution.** —Colombia, Ecuador.

***interrupta*** Flint, 1991b: 41 [type locality: Colombia, Dpto. Antioquia, Quebrada Espadera, 7 km E Medellín, on road to Sta. Elena; NMNH; ♂]. —Muñoz-Quesada 2000: 278 [checklist]. —Thomson 2012: 2 [checklist]. —Thomson and Holzenthal 2015: 28 [♂].

**Distribution.** —Colombia.

***kateae*** Thomson & Holzenthal, 2015: 29 [type locality: Venezuela, Aragua, 1 km E Estacíon Biológica Rancho Grande, 10.352°N, 67.680°W, 1100 m; UMSP; ♂].

**Distribution.** —Venezuela.

***laposka*** Oláh & Johanson, 2011: 162 [type locality: Peru, San Martín Province, creek crossing road Juan Guerra-Chazuta, 14 km (rd.) E Colombia Bridge; NHRS; ♂]. —Thomson 2012: 2 [checklist]. —Thomson and Holzenthal 2015: 30 [♂].

**Distribution.** —Peru.

***lerma*** Angrisano & Burgos, 2002: 106 [type locality: Argentina, Salta, Rí Lesser, 18 km NW Salta; IML; ♂]. —Thomson 2012: 2 [checklist]. —Isa Miranda and Rueda Martín 2014: 196 [larva; pupa; case; distribution]. —Thomson and Holzenthal 2015: 31 [♂].

**Distribution.** —Argentina.

***limpia*** Ross, 1944: 273 [type locality: United States, Texas, Fort Davis, Limpia Creek; INHS; ♂; ♀]. —Flint 1970: 6 [♂]. —Edwards 1973: 506 [distribution]. —Bueno-Soria and Flint 1978: 200 [distribution]. —Blickle 1979: 50, 57 [checklist; ♂]. —Holzenthal 1988: 61 [distribution]. —Moulton et al. 1994: 170 [distribution]. —Flint 1996b: 86 [correction of errors in 1970 paper]. —Baumgardner and Bowles 2005: 11 [distribution]. —Blinn and Ruiter 2005: 69 [distribution; biology]. —Bowles et al. 2007: 21 [distribution]. —Bueno-Soria et al. 2007: 33 [distribution]. —Blinn and Ruiter 2009b: 186 [phenology; distribution]. —Thomson 2012: 2 [checklist]. —Thomson and Holzenthal 2015: 31 [♂; distribution].

**Distribution.** —Costa Rica, Mexico, U.S.A.

***melleopicta*** Mosely, 1934a: 157 [type locality: Mexico, Tabasco, Teapa; NHMUK; ♂]. —Flint 1970: 5 [♂]. —Bueno-Soria and Flint 1978: 200 [distribution]. —Flint 1981: 25 [♂; distribution]. —Thomson 2012: 2 [checklist]. —Thomson and Holzenthal 2015: 9 [♂]. —Armitage et al. 2016: 8 [distribution]. —Armitage and Harris 2018b: 97 [checklist]. —Harris and Armitage 2019: 4 [distribution].

**Distribution.** —Mexico, Panama, Venezuela.

***mutica*** Flint, 1991b: 39 [type locality: Colombia, Dpto. Antioquia, Quebrada Honda, Marsella, 12 km SW Fredonia; NMNH; ♂]. —Muñoz-Quesada 2000: 278 [checklist]. —Thomson 2012: 2 [checklist]. —Thomson and Holzenthal 2015: 32 [♂]. —Armitage et al. 2018: 5 [distribution]. —Harris and Armitage 2019: 4, 20 [distribution; ♂].

**Distribution.** —Panama, Colombia.

***padera*** Flint, 1991b: 41 [type locality: Colombia, Dpto. Antioquia, Quebrada Espadera, 7 km E Medellín, road to Sta. Elena; NMNH; ♂]. —Muñoz-Quesada 2000: 278 [checklist]. —Thomson 2012: 2 [checklist]. —Thomson and Holzenthal 2015: 33 [♂].

**Distribution.** —Colombia.

***pectinata*** Thomson & Holzenthal, 2015: 34 [type locality: Ecuador, Tungurahua, 13 km E Baños, 1550 m; NMNH; ♂]. —Ríos-Touma et al. 2017: 10 [checklist].

**Distribution.** —Ecuador.

***pictipes*** (Banks, 1911): 359 [type locality: United States, New York, Johnstown, Hales Creek; MCZ; ♂; in *Orthotrichia*]. —Betten 1934: 152 [checklist]. —Ross 1938b: 10 [lectotype designated; as *Stactobiapictipes* (Banks); ♂]. —Ross 1944: 120 [to *Leucotrichia*; larva; case]. —Denning 1947b: 170 [distribution]. —Denning 1947a: 145 [♂; distribution]. —Nielsen 1948: 11 [misidentified as *Ithytrichiaconfusa* Morton]. —Etnier 1965: 147 [checklist]. —Flint 1970: 10 [♂; distribution]. —Etnier and Schuster 1979: 18 [distribution]. —Blickle 1979: 50, 57 [checklist; ♂]. —Parker and Voshell 1981: 4 [checklist]. —McAuliffe 1982: 1557 [biology; distribution]. —Hamilton et al. 1983: 18 [distribution]. —McAuliffe 1984: 894 [ecology; distribution]. —Lake 1984: 220 [distribution]. —Hart 1985b: 40 [ecology]. —Hart 1985a: 404 [ecology]. —Hart and Robinson 1990: 1496 [ecology]. —Tarter 1990: 239 [checklist]. —Hart et al. 1991: 330 [ecology]. —Harris et al. 1991: 217 [distribution]. —Masteller and Flint 1992: 70 [checklist]. —Hart 1992: 222 [ecology]. —Moulton and Stewart 1996: 108 [♂; larva; distribution]. —Houp 1999: 2 [distribution]. —Ruiter 1999: 165 [distribution]. —Newell et al. 2001: 192 [distribution; phenology]. —Houghton et al. 2001: 505 [distribution]. —Keiper and Bartolotta 2003: 255 [ecology; distribution]. —Blinn and Ruiter 2005: 69 [distribution; biology]. —Blinn and Ruiter 2006: 332 [biology; distribution]. —Bueno-Soria et al. 2007: 33 [distribution]. —Armitage et al. 2011: 14 [checklist]. —Myers et al. 2011: 107 [distribution]. —Thomson 2012: 2 [checklist]. —Thomson and Holzenthal 2015: 35[♂]. —Houghton et al. 2017: 63 [checklist]. —Mendez et al. 2019: 118 [checklist].

**Distribution.** —Mexico, U.S.A.

***repanda*** Thomson & Holzenthal, 2015: 37 [type locality: Venezuela, Sucre, Península de Paria, Santa Isabel, Río Sta. Isabel, 10°44.294'N, 62°38.954'W, 20 m; UMSP; ♂].

**Distribution.** —Venezuela.

***rhomba*** Thomson & Holzenthal, 2015: 38 [type locality: Costa Rica, Puntarenas, Río Jaba at rock quarry, 1.4 km (air) W Las Cruces, 8.79°N, 82.97°W, 1150 m; UMSP; ♂]. —Harris and Armitage 2019: 4, 20, 21 [distribution; ♂].

**Distribution.** —Costa Rica, Panama.

***riostoumae*** Thomson & Holzenthal, 2015: 39 [type locality: Ecuador, Imbabura, Reserva los Cedros, Río de la Plata, 00.32495°N, 78.78084°W, 1587 m; UMSP; ♂]. —Ríos-Touma et al. 2017: 10 [checklist].

**Distribution.** —Ecuador.

***sarita*** Ross, 1944: 274 [type locality: United States, Texas, Balmorhea, along stone irrigation flume; INHS; ♂]. —Flint 1968a: 38 [♂; ♀; larva; pupa; distribution]. —Flint 1970: 9 [♂; larva; case; distribution]. —Edwards 1973: 506 [distribution]. —Buneo-Soria and Flint 1978: 200 [distribution]. Blickle 1979: 50, 57 [checklist; ♂]. —Holzenthal 1988: 61 [distribution]. —Flint and Sykora 1993: 54 [distribution]. —Blinn and Ruiter 2005: 69 [distribution; biology]. —Bowles et al. 2007: 21 [distribution; biology]. —Bueno-Soria et al. 2007: 33 [distribution]. —Chamorro-Lacayo et al. 2007: 43 [checklist]. —Thomson 2012: 2 [checklist]. —Thomson and Holzenthal 2015: 40 [♂]. —Mendez et al. 2019: 118 [checklist].

**Distribution.** —Costa Rica, El Salvador, Grenada, Guatemala, Mexico, Nicaragua, U.S.A.

***sidneyi*** Thomson & Holzenthal, 2015: 42 [type locality: Venezuela, T. F. A., Camp IV, 0°58'N, 65°57'W, Cerro d. l. Neblina, 760 m; NMNH; ♂].

**Distribution.** —Venezuela.

***tapantia*** Thomson & Holzenthal, 2015: 42 [type locality: Costa Rica, Cartago, Reserva Tapantí, waterfall, ca. 1 km (road) NW tunnel, 9.69°N, 83.76°W, 1600 m; UMSP; ♂].

**Distribution.** —Costa Rica.

***termitiformis*** Botosaneanu in Botosaneanu and Alkins-Koo 1993: 13 [type locality: Trinidad, stream below Maracas waterfall; ZMUA; ♂; larva]. —Botosaneanu and Sakal 1992: 201 [distribution; ecology]. —Flint 1996b: 89 [distribution]. —Botosaneanu 2002b: 84 [checklist]. —Thomson 2012: 2 [checklist]. —Thomson and Holzenthal 2015: 43 [♂].

**Distribution.** —Trinidad.

***topora*** (Oláh & Flint, 2012): 156 [type locality: Panama, Barro Colorado Island, Snyder-Molino trail, marker 3; NMNH; ♂; in *Ascotrichia*]. —Armitage et al. 2015a: 6 [checklist]. —Armitage and Harris 2018b: 97 [checklist]. —Santos 2020: 229 [to *Leucotrichia*].

**Distribution.** —Panama.

***tritoven*** Flint, 1996b: 89 [type locality: Trinidad, streamlet, Lalaja Road, 10°43'N, 61°17'W; NMNH; ♂]. —Botosaneanu 2002b: 84 [checklist]. —Thomson 2012: 2 [checklist]. —Thomson and Holzenthal 2015: 44 [♂; distribution].

**Distribution.** —Guyana, Tobago, Trinidad, Venezuela.

***tubifex*** Flint, 1964: 44 [type locality: Puerto Rico, Maricao, at fish hatcheryl NMNH; ♂; ♀; larva; pupa; case]. —Flint 1968b: 33 [♂; ♀; larva; pupa; distribution]. —Flint 1968a: 81 [checklist]. —Flint 1970: 7 [♂; larva; case; distribution]. —Botosaneanu 1991: 116 [distribution]. —Botosaneanu 1995a: 22 [distribution]. —Botosaneanu and Bolland 1997: 71 [parasitized by mite, genus *Leptus*]. —Botosaneanu and Hyslop 1998: 7 [distribution]. —Flint and Pérez-Gelabert 1999: 39 [checklist]. —Botosaneanu 2002b: 84 [checklist]. —Flint and Sykora 2004: 32 [distribution]. —Pérez-Gelabert 2008: 300 [checklist]. —Thomson 2012: 2 [checklist]. —Thomson and Holzenthal 2015: 45 [♂].

**Distribution.** —Dominican Republic, Haiti, Jamaica, Puerto Rico.

***viridis*** Flint, 1967b: 10 [type locality: Guatemala, Izabal, Las Escobas near Matias de Galvez; NMNH; ♂]. —Flint 1970: 5 [♂; distribution]. —Bueno-Soria and Flint 1978: 201 [distribution]. —Aguila 1992: 538 [distribution]. —Thomson 2012: 2 [checklist]. —Thomson and Holzenthal 2015: 46 [♂]. —Armitage et al. 2015a: 6 [checklist]. —Armitage and Harris 2018b: 97 [checklist]. —Harris and Armitage 2019: 4 [distribution].

**Distribution.** —El Salvador, Guatemala, Mexico, Panama.

***yungarum*** Angrisano & Burgos, 2002: 105 [type locality: Argentina, Salta, Finca Jakúlica, 630 m; IML; ♂]. —Thomson 2012: 2 [checklist]. —Thomson and Holzenthal 2015: 47 [♂].

**Distribution.** —Argentina.

***zopilote*** (Holzenthal & Harris, 1999): 561 [type locality: Costa Rica, Guanacaste, Parque Nacional Rincón de la Vieja, Guebrada Zopilote, 10.765°N, 85.309°W; NMNH; ♂; ♀; in *Costatrichia*]. —Santos et al. 2016a: 472 [to *Leucotrichia*].

**Distribution.** —Costa Rica.

##### Genus *Peltopsyche* Müller, 1879

*Peltopsyche* Müller, 1879a: 144 [type species: *Peltopsychesieboldi* Müller, 1879a, subsequent selection of Fischer 1961]. —Ulmer 1957: 172 [bibliography; discussion]. —Marshall 1979b: 179 [generic review]. —Flint et al. 1999a: 118 [discussion].

*Abtrichia* Mosely, 1939a: 224 [type species *Abtrichiaantennata* Mosely, 1939a, original designation]. —Marshall 1979b: 183 [generic revision]. —Santos et al. 2016a: 472 [to synonymy].

The genus *Peltopsyche* was established for two species recorded from Brazil, and now currently contains a total of six species. In the original descriptions only a few larval features and the basal antennal segments of males are figured. Marshall (1979b) commented that, because the general larval morphology is very similar to that of *Zumatrichia* and the case is highly similar to that of *Leucotrichia*, *Peltopsyche* may one day prove to be a senior synonym of one or more of the other leucotrichiine genera. Santos et al. (2016a) proved this prediction true when they synonymized *Abtrichia*, a change supported by both morphological and molecular data.

***antennata*** (Mosely, 1939a): 227 [type locality: Brazil, Edo. Santa Catarina, Nova Teutonia; NHMUK; ♂; in *Abtrichia*]. —Flint 1972b: 233 [♂; larva; distribution; in *Abtrichia*]. —Angrisano 1995a: 505 [distribution]. —Angrisano 1999: 31 [checklist]. —Angrisano 2002: 406 [pupa]. —Blahnik et al. 2004: 4 [distribution]. —Paprocki et al. 2004: 10 [checklist]. —Angrisano and Sganga 2007: 29 [♂; larva; pupa; distribution]. —Dumas et al. 2010: 8 [distribution]. —Oláh and Flint 2012: 138 [distribution]. —de Souza et al. 2013: 585 [distribution]. —Paprocki and França 2014: 39 [checklist]. —Santos et al. 2016a: 472 [to *Peltopsyche*].

**Distribution.** —Argentina, Brazil, Uruguay.

***epara*** (Oláh & Flint, 2012): 139 [type locality: Argentina, Tucamá Province, South of Concepción; NMNH; ♂; in *Abtrichia*]. —Santos et al. 2016a: 472 [to *Peltopsyche*].

**Distribution.** —Argentina.

***sieboldi*** Müller, 1879a: 144 [type locality: Brazil, Santa Catarina, Garcia, Encano, and Warnow Rivers, tributaries of the Itajahy River; type depository unknown; case; ♂ antenna, larva]. —Müller 1880b: 133 [larval case]. —Müller 1880a: 83 [larval case]. —Müller 1921: 386 [♂ antenna]. —Ulmer 1957: 172 [bibliography; possibly *Abtrichiaantennata*]. —Angrisano 1999: 35 [checklist]. —Paprocki et al. 2004: 12 [checklist]. —Paprocki and França 2014: 54 [checklist].

**Distribution.** —Brazil.

***squamosa*** (Mosely, 1939a): 226 [type locality: Brazil, Edo. Santa Catarina, Nova Teutonia; NHMUK; ♂; in *Abtrichia*]. —Angrisano 1999: 31 [checklist]. —Blahnik et al. 2004: 4 [distribution]. —Paprocki et al. 2004: 2004”10 [checklist]. —Dumas et al. 2009: 365 [distribution]. —Dumas and Nessimian 2012: 14 [checklist]. —Oláh and Flint 2012: 140 [distribution]. —Paprocki and França 2014: 39 [checklist]. —Santos et al. 2016a: 472 [to *Peltopsyche*].

**Distribution.** —Argentina, Brazil.

***vegosa*** (Oláh & Flint, 2012): 140 [type locality: Paraguay, 2 km South, Cerra Cora; NMNH; ♂; in *Abtrichia*]. —Santos et al. 2016a: 472 [to *Peltopsyche*].

**Distribution.** —Brazil, Paraguay.

***veva*** (Oláh & Johanson, 2011): 154 [type locality: French Guiana, Maripasoula, Lawa River, Maripasoula, 83 mao 3°37.959'N, 54°1.426'W, 83 m; NHRS; ♂]. —Oláh and Flint 2012: 142 [distribution]. —Santos et al. 2016a: 472 [to *Peltopsyche*].

**Distribution.** —French Guiana, Guyana.

##### Genus *Tupiniquintrichia* Santos, Nessimian, a&nd Takiya, 2016

*Tupiniquintrichia* Santos, Nessimian, & Takiya, 2016: 475 [type species: *Peltopsychemaclachlani* Müller, 1879a, original designation].

*Tupiniquintrichia* contains two species, one originally described in the genus *Peltopsyche* and the other in *Leucotrichia*. The known distribution of the genus is limited to Brazil. The unique antennal organization can be used to define the genus, along with features of the male genitalia (Santos et al. 2016a). A larval description has been written for *P.maclachlani*.

***maclachlani*** (Müller, 1879a): 144 [type locality: Brazil, Santa Catarina, Warnow River, tributary of Itajahy River; MNRJ; case; ♂; antenna; larva; in *Peltopsyche*]. —Müller 1880b: 133 [larval case]. —Müller 1880a: 83 [larval case]. —Müller 1921: 386 [♂; antenna]. —Ulmer 1957: 172 [bibliography]. —Paprocki et al. 2004: 12 [checklist]. —Paprocki and França 2014: 54 [checklist]. —Santos et al. 2016a: 476 [♂, to *Tupiniquintrichia*; type status].

**Distribution.** —Brazil.

***procera*** (Thomson & Holzenthal, 2015): 37 [type locality: Brazil, Minas Geras, Córrego da Serra de Ouro, Fino, Vale do Tropeiro, 20°12.371'S, 43°38.581'W, 1000 m; MZUSP; ♂; in *Leucotrichia*]. —Santos et al. 2016a: 476 [to *Tupiniquintrichia*].

**Distribution.** —Brazil.

##### Genus *Zumatrichia* Mosely, 1937

*Zumatrichia* Mosely, 1937b: 187 [type species: *Zumatrichiafilosa* Mosely, 1937b, original designation]. —Flint 1970: 16 [revision]. —Marshall 1979b: 179 [generic review]. —Blickle 1979: 6 [key to species of America north of Mexico]. —Pes and Hamada 2004: 31 [new records]. —Oláh and Johanson 2011: 152 [placement in *Leucotrichia* genus cluster].

*Zumatrichia* contains 53 species occurring in Central America to northern South America, throughout the Lesser Antilles, and also in Mexico and the United States. Marshall (1979b) outlined four main species groups (*filosa*, *galtena*, *multisetosa*, and *palmara*) within the genus based on features of the male genitalia, a modification of the five originally outlined by Flint (1970). Larval descriptions have been published for *Z.antilliensis*, *Z.anomaloptera*, *Z.multisetosa*, and *Z.notosa* (Flint 1968a; Wiggins 1996).

***alarca*** Oláh & Johanson, 2011: 164 [type locality: Peru, San Martin Prov., Rio Huallaga tributary, small river passing Chazuta, 6°34.665'S, 76°08.209'W; NHRS; ♂].

**Distribution.** —Peru.

***angulata*** Flint, 1970: 21 [type locality: Panama, Chiriqui, Rovira, David; NMNH; ♂]. —Aguila 1992: 538 [distribution]. —Armitage et al. 2015a: 7 [checklist]. —Armitage and Harris 2018b: 99 [checklist].

**Distribution.** —Panama.

***anomaloptera*** Flint, 1968a: 37 [type locality: Grenada, Balthazar, NMNH; ♂; ♀]. —Malicky 1983c: 264 [distribution]. —Botosaneanu 1988: 221 [♂; ♀; distribution]. —Botosaneanu and Sakal 1992: 201 [distribution; ecology]. —Botosaneanu and Alkins-Koo 1993: 7 [distribution]. —Flint and Sykora 1993: 55 [distribution]. —Botosaneanu 1994a: 37 [distribution]. —Flint 1996b: 85 [distribution]. —Botosaneanu 2002b: 89 [checklist]. —Botosaneanu and Thomas 2005: 45 [distribution].

**Distribution.** —Dominica, Grenada, Guadeloupe, Martinique, St. Lucia, St. Vincent, Tobago, Trinidad.

***antilliensis*** Flint, 1968a: 34 [type locality: Dominica, Clarke Hall; NMNH; ♂; ♀; larva; pupa; case]. —Malicky 1983c: 264 [distribution]. —Botosaneanu 1988: 221 [♂; ♀; distribution]. —Flint and Sykora 1993: 54 [distribution]. —Botosaneanu 1994a: 37 [distribution]. —Flint 1996b: 85 [distribution]. —Botosaneanu 2000: 256 [distribution]. —Botosaneanu 2002b: 89 [checklist]. —Botosaneanu and Thomas 2005: 45 [distribution]. —Oláh and Flint 2012: 177 [distribution]. —Armitage et al. 2015a: 7 [checklist]. —Ríos-Touma et al. 2017: 11 [checklist]. —Armitage and Harris 2018b: 99 [checklist].

**Distribution.** —Colombia, Dominica, Ecuador, Grenada, Guadeloupe, Martinique, Panama, St. Lucia, St. Vincent, Venezuela.

***atmena*** Oláh & Flint, 2012: 177 [type locality: Venezuela, Aragua State, Cuyagua, Rio Grande; NMNH; ♂].

**Distribution.** —Venezuela.

***attenuata*** Flint, 1970: 22 [type locality: Costa Rica, Cartago, Quebrado Relleno, La Cruzada, E Turrialba; NMNH; ♂]. —Holzenthal 1988: 63 [distribution]. —Armitage et al. 2015b: 6 [distribution]. —Armitage et al. 2015a: 7 [distribution]. —Armitage and Harris 2018b: 99 [checklist]. —Armitage and Harris 2018c: 284 [distribution].

**Distribution.** —Costa Rica, Panama.

***befela*** Oláh & Flint, 2012: 179 [type locality: Venezuela, Barinas State, Rio Santo Domingo, Barinas; NMNH; ♂].

**Distribution.** —Venezuela.

***bevagota*** Oláh & Flint, 2012: 180 [type locality: Ecuador, Cotopaxi Province, Quevedo (36 km Northeast), 1100 m; NMNH; ♂]. —Ríos-Touma et al. 2017: 11 [checklist].

**Distribution.** —Ecuador.

***bifida*** Flint, 1970: 21 [type locality: Costa Rica, San Jose, Rio General, Pacuare; NMNH; ♂]. —Holzenthal 1988: 63 [distribution]. —Aguila 1992: 538 [distribution]. —Armitage et al. 2015a: 7 [checklist]. —Armitage and Harris 2018b: 99 [checklist].

**Distribution.** —Costa Rica, Panama.

***caudifera*** Flint, 1970: 23 [type locality: Panama, Chiriqui, Dolega; NMNH; ♂]. —Holzenthal 1988: 63 [distribution]. —Aguila 1992: 538 [distribution]. —Chamorro-Lacayo et al. 2007: 44 [checklist]. —Armitage et al. 2015a: 8 [checklist]. —Armitage and Harris 2018b: 99 [checklist].

**Distribution.** —Costa Rica, Nicaragua, Panama.

***chiriquiensis*** Flint, 1970: 20 [type locality: Panama, Chiriqui, Dolega; NMNH; ♂]. —Holzenthal 1988: 64 [distribution]. —Aguila 1992: 538 [distribution]. —Armitage et al. 2015a: 8 [checklist]. —Armitage and Harris 2018b: 99 [checklist].

**Distribution.** —Costa Rica, Panama.

***corosa*** Oláh & Flint, 2012: 181 [type locality: Ecuador, Cotopaxi Province, Quevedo (36 km Northeast), 1100 m; NMNH; ♂]. —Ríos-Touma et al. 2017: 11 [checklist].

**Distribution.** —Ecuador.

***dereka*** Oláh & Flint, 2012: 183 [type locality: Panama, San Blas Province, Rio Carti Grande, 2 km West Nusagandi; NMNH; ♂]. —Armitage et al. 2015a: 8 [checklist]. —Armitage and Harris 2018b: 99 [checklist].

**Distribution.** —Panama.

***diamphidia*** Flint, 1970: 12 [type locality: Costa Rica, Puntarenas, 2.8 miles E Golfito; NMNH; ♂]. —Holzenthal 1988: 64 [distribution].

**Distribution.** —Costa Rica.

***echinata*** Flint, 1967b: 11 [type locality: Guatemala, El Progreso, San Agustin Acasaguastlan; NMNH; ♂]. —Flint 1970: 18 [distribution]. —Chamorro-Lacayo et al. 2007: 44 [checklist,].

**Distribution.** —Guatemala, Honduras, Nicaragua.

***felfesa*** Oláh & Flint, 2012: 184 [type locality: Venezuela, Zulia State, Perijo El Tucuco, Mission el Tucuco, Rio El Tucuco, 0.5 km from Church; NMNH; ♂].

**Distribution.** —Venezuela.

***fesuka*** Oláh & Flint, 2012: 185 [type locality: Ecuador, Napo Province, Pano, at stream, 580 m; NMNH; ♂]. —Ríos-Touma et al. 2017: 11 [checklist].

**Distribution.** —Ecuador.

***filosa*** Mosely, 1937b: 187 [type locality: Mexico, Chiapas, Saltenango de la Paz; NHMUK; ♂]. —Flint 1970: 23 [♂; distribution]. —Bueno-Soria and Flint 1978: 201 [distribution]. —Maes and Flint 1988: 4 [distribution]. —Holzenthal 1988: 64 [distribution]. —Maes 1999: 1195 [checklist]. —Chamorro-Lacayo et al. 2007: 44 [checklist]. —Barba-Álvarez et al. 2019: 86 [distribution].

**Distribution.** —Costa Rica, Guatemala, Mexico, Nicaragua.

***flinti*** Harris & Armitage, 2019: 18 [type locality: Panama, Bocas del Toro Province, Quebrada Rambala, near Rambala Jungle Lodge, 3.74 km SSE Rambala, 8.91627°N and 82.15469°W, 120 m; COZEM; ♂].

**Distribution.** —Panama.

***galtena*** Mosely, 1937b: 188 [type locality: Mexico, Chiapas, Saltenango de la Paz; NHMUK; ♂]. —Flint 1970: 19 [♂; distribution]. —Bueno-Soria and Flint 1978: 201 [distribution]. —Holzenthal 1988: 64 [distribution]. —Chamorro-Lacayo et al. 2007: 44 [checklist]. —Armitage et al. 2015b: 6 [distribution]. —Armitage et al. 2015a: 8 [checklist]. —Armitage and Harris 2018b: 99 [checklist]. —Armitage and Harris 2018c: 284 [distribution]. —Harris and Armitage 2019: 6 [distribution].

**Distribution.** —Costa Rica, Honduras, Mexico, Nicaragua, Panama.

***gorba*** Oláh & Flint, 2012: 187 [type locality: Ecuador, Zamora Chinchipe Province, Rio Chicana, 9 km North Yanzatza, 880 m; NMNH; ♂]. —Ríos-Touma et al. 2017: 11 [checklist].

**Distribution.** —Ecuador.

***gula*** Oláh & Flint, 2012: 188 [type locality: Venezuela, Barinas State, Rio Santo Domingo, Barinas; NMNH; ♂].

**Distribution.** —Venezuela.

***haroma*** Oláh & Flint, 2012: 189 [type locality: Venezuela, Barinas State, Puente Parangula, 8 km South Barinitas; NMNH; ♂].

**Distribution.** —Venezuela.

***hazelae*** Harris & Armitage, 2019: 19 [type locality: Panama, Bocas del Toro Province, tributary of Quebrada Rambala, Rambala Jungle Lodge, 3.7 km SSE Rambala, 8.91627°N and 82.15469°W, 134 m; COZEM; ♂].

**Distribution.** —Panama.

***kerekeda*** Oláh & Flint, 2012: 191 [type locality: Colombia, Rio Raposo; NMNH; ♂]. —Ríos-Touma et al. 2017: 11 [distribution].

**Distribution.** —Colombia, Ecuador.

***kisgula*** Oláh & Flint, 2012: 192 [type locality: Ecuador, Napo Province, Lago Agrio (48 km West), Rio Aguarico; NMNH; ♂]. —Ríos-Touma et al. 2017: 11 [checklist].

**Distribution.** —Ecuador.

***kislaba*** Oláh & Flint, 2012: 194 [type locality: Ecuador, Pastaza Province, Puyo (3 km West); NMNH; ♂]. —Ríos-Touma et al. 2017: 11 [checklist].

**Distribution.** —Ecuador.

***koztesa*** Oláh & Flint, 2012: 195 [type locality: Venezuela, Aragua State, Parque Nacional Henri Pittier, Rio La Trilla, 22.5 km North of Rancho Grande on Road; NMNH; ♂].

**Distribution.** —Venezuela.

***lapa*** Oláh & Flint, 2012: 196 [type locality: Ecuador, Pastaza Province, Puyo (27 km North), Estacion Fluviometrica; NMNH; ♂]. —Ríos-Touma et al. 2017: 11 [checklist].

**Distribution.** —Ecuador.

***lezarda*** Malicky, 1980b: 220 [type locality: Guadeloupe, Mittellauf de Flusses Lezard bei Chemin de Diane; Collection Malicky; ♂]. —Malicky 1983c: 264 [checklist]. —Flint and Sykora 1993: 49 [checklist]. —Botosaneanu 2002b: 89 [checklist]. —Botosaneanu and Thomas 2005: 55 [checklist].

**Distribution.** —Guadeloupe.

***longispinga*** Bueno-Soria, 1983a: 454 [type locality: Mexico, Veracruz, Los Tuxtlas area, Rio La Palma; NMNH; ♂].

**Distribution.** —Mexico.

***marica*** Flint, 1981: 26 [type locality: Venezuela, Aragua, Maracay, Río Limón, Estacion Piscicultura; NMNH; ♂].

**Distribution.** —Venezuela.

***masa*** Oláh & Flint, 2012: 198 [type locality: Ecuador, Pastaza Province, Puyo; NMNH; ♂]. —Ríos-Touma et al. 2017: 11 [checklist].

**Distribution.** —Ecuador.

***maskara*** Oláh & Flint, 2012: 199 [type locality: Panama, San Blas Province, Rio Carti Grande, 2 km West Nusagandi; NMNH; ♂]. —Armitage et al. 2015a: 8 [checklist]. —Armitage and Harris 2018b: 99 [checklist].

**Distribution.** —Panama.

***maskoska*** Oláh & Flint, 2012: 201 [type locality: Venezuela, Zula State, Perijo El Tucuco, Mission El Tucuco, Rio El Tucuco, 0.5 km from Church; NMNH; ♂].

**Distribution.** —Venezuela.

***multisetosa*** Flint, 1970: 17 [type locality: Guatemala, Suchitepequez, Cuyotenango; NMNH; ♂; larva; case]. —Bueno-Soria and Flint 1978: 201 [distribution]. —Holzenthal 1988: 64 [distribution]. —Barba-Álvarez et al. 2019: 86 [distribution].

**Distribution.** —Costa Rica, Guatemala, Honduras, Mexico.

***nelkula*** Oláh & Flint, 2012: 202 [type locality: Panama, San Blas Province, Rio Carti Grande, 2 km West Nusagandi; NMNH; ♂]. —Armitage et al. 2015a: 8 [checklist]. —Armitage and Harris 2018b: 99 [checklist].

**Distribution.** —Panama.

***notosa*** (Ross), 1944: 271 [type locality: [U.S.A.] Missouri River, Toston, Montana; INHS; ♂; ♀; in *Leucotrichia*]. —Flint 1970: 20 [♂; to *Zumatrichia*]. —Blickle 1979: 55, 57 [checklist; ♂]. —Ruiter 1999: 166 [distribution]. —Houghton 2001: 90 [distribution]. —Blinn and Ruiter 2005: 69 [distribution; biology]. —Blinn and Ruiter 2006: 333 [distribution; biology].

**Distribution.** —U.S.A.

***palmara*** Flint, 1970: 22 [type locality: El Salvador, La Libertad, Rio El Palmar, 15 miles N La Libertad; NMNH; ♂]. —Holzenthal 1988: 64 [distribution]. —Flint and Reyes 1991: 484 [distribution]. —Chamorro-Lacayo et al. 2007: 44 [checklist]. —Armitage et al. 2015a: 8 [checklist]. —Ríos-Touma et al. 2017: 11 [checklist]. —Armitage and Harris 2018b: 99 [checklist].

**Distribution.** —Costa Rica, Ecuador, El Salvador, Nicaragua, Panama, Peru.

***picigula*** Oláh & Flint, 2012: 203 [type locality: Ecuador, Napo Province, Rio Jondachi, 30 km North Tena, 950 m; NMNH; ♂]. —Ríos-Touma et al. 2017: 11 [checklist].

**Distribution.** —Ecuador.

***rhamphoides*** Flint, 1970: 24 [type locality: Costa Rica, Puntarenas, Rio La Vieja, near Lagarto; NMNH; ♂]. —Holzenthal 1988: 64 [distribution]. —Aguila 1992: 538 [distribution]. —Armitage et al. 2015a: 8 [checklist]. —Armitage and Harris 2018b: 99 [checklist; as *rhampiodes*]. —Harris and Armitage 2019: 6 [distribution].

**Distribution.** —Costa Rica, Panama.

***saluda*** Flint, 1970: 19 [type locality: Panama, Canal Zone, pipeline road, Rio Agua Salud; NMNH; ♂]. —Aguila 1992: 538 [distribution]. —Armitage et al. 2015a: 8 [checklist]. —Armitage and Harris 2018b: 99 [checklist].

**Distribution.** —Panama.

***sima*** Oláh & Flint, 2012: 205 [type locality: Ecuador, Pichincha Province, Santo Domingo de los Colorados, 14 km East; NMNH; ♂]. —Ríos-Touma et al. 2017: 11 [checklist].

**Distribution.** —Ecuador.

***sortetla*** Oláh & Flint, 2012: 206 [type locality: Panama, Darien Province, Rio Tuira at Rio Pucuro; NMNH; ♂]. —Armitage et al. 2015a: 8 [checklist]. —Armitage and Harris 2018b: 99 [checklist].

**Distribution.** —Panama.

***strobilina*** Flint, 1970: 20 [type locality: Costa Rica, Cartago, 3 miles W Turrialba; NMNH; ♂]. —Holzenthal 1988: 64 [distribution].

**Distribution.** —Costa Rica.

***teapa*** Flint, 1970: 24 [type locality: Mexico, Tabasco, Rio Puyacatengo, E Teapa; NMNH; ♂]. —Bueno-Soria and Flint 1978: 201 [distribution].

**Distribution.** —Mexico.

***teribe*** Harris & Armitage, 2015: 2 [type locality: Panama, Chiriquí Province, Cuenca 108, Quebrada Grande, Boquete, Valle Escondido, below Sabor Restaurant, 8.77970°N, 82.44016°W, 1122 m; MIUP; ♂]. —Armitage et al. 2015a: 8 [checklist]. —Armitage and Harris 2018b: 99 [checklist]. —Armitage and Harris 2018c: 284 [distribution]. —Harris and Armitage 2019: 6 [distribution].

**Distribution.** —Panama.

***tompagula*** Oláh & Flint, 2012: 207 [type locality: Colombia, Meta Department, Quebrada Blanca, 3 km West Restrepo; NMNH; ♂].

**Distribution.** —Colombia.

***turuda*** Oláh & Flint, 2012: 208 [type locality: Panama, San Blas Province, Quebrada Pingadi, 9 km North Nusagandi; NMNH; ♂]. —Armitage et al. 2015a: 8 [checklist]. —Armitage and Harris 2018b: 99 [checklist].

**Distribution.** —Panama.

***tusa*** Oláh & Flint, 2012: 210 [type locality: Colombia, Choco department, Rio Atrato, Yuto; NMNH; ♂].

**Distribution.** —Colombia, Venezuela.

***varrata*** Oláh & Johanson, 2011: 165 [type locality: Peru, San Martin Prov., Rio Huallaga tributary, small river passing Chazuta, 6°34.665'S, 76°08.209'W; NHRS; ♂].

**Distribution.** —Peru.

***vieja*** Flint, 1970: 19 [type locality: Costa Rica, Puntarenas, Rio La Vieja, near Lagarto, E Palmar Norte; NMNH; ♂]. —Holzenthal 1988: 64 [distribution].

**Distribution.** —Costa Rica.

***zegla*** Harris & Armitage, 2015: 4 [type locality: Panama, Bocas del Toro Province, Cuenca 91, Río Teribe at Zegla; NMNH; ♂]. —Armitage et al. 2015a: 8 [checklist]. —Armitage and Harris 2018b: 99 [checklist].

**Distribution.** —Panama.

### ﻿Subfamily NEOTRICHIINAE Ross, 1956

Neotrichiini Ross, 1956: 18 [type genus: *Neotrichia* Morton, 1905]. —Marshall 1979b: 188 [reviewed as tribe Neotrichiini]. —Oláh and Johanson 2011: 167 [generic character states].

The subfamily Neotrichiinae contains four genera occurring in the Nearctic and Neotropical faunal regions. The name Neotrichiinae, as the tribe Neotrichiini, was first used by Ross (1956) in a phylogenetic diagram, although no diagnosis was outlined until Marshall (1979b). Notable features are their exceptionally small adult size (generally less than 2 mm), even for microcaddisflies; larvae construct cylindrical cases and have associated limnephiloid morphological features, as opposed to the more typical hydroptilid purse-case (Marshall 1979b). Larval descriptions have been provided for all genera.

#### Genus *Kumanskiella* Harris & Flint, 1992

*Kumanskiella* Harris & Flint, 1992: 581 [type species: *Kumanskiellakarenae* Harris & Flint, 1992, original designation]. —Oláh and Johanson 2011: 167 [in Neotrichiini].

The genus *Kumanskiella* includes two species recorded from Cuba and Puerto Rico. Although both the larvae and adults possess features that are intermediate between the genera *Mayatrichia* and *Neotrichia*, the species do not fit well within the limits of either previously established genus (Harris and Flint 1992). Harris and Flint (1992) gave a larval description for the type species, *K.karenae*.

***aliena*** (Kumanski, 1987): 24 [type locality: Cuba, Province Las Villas, Sierra de Trinidad, road Trinidad-Topes de Collantes; SOFM; ♂; in *Mayatrichia*]. —Harris and Flint 1992: 587 [♂; to *Kumanskiella*]. —Flint 1996a: 16 [checklist]. —Botosaneanu 2002b: 83 [checklist]. —Naranjo López and González Lazo 2005: 149 [checklist].

**Distribution.** —Cuba.

***karenae*** Harris & Flint, 1992: 582 [type locality: Puerto Rico, El Verde Field Station, Quebrada Prieta; NMNH; ♂; ♀; larva; case]. —Botosaneanu 2002b: 83 [checklist].

**Distribution.** —Puerto Rico.

#### Genus *Mayatrichia* Mosely, 1937

*Mayatrichia* Mosely, 1937b: 182 [type species: *Mayatrichiaayama* Mosely, 1937b, original designation]. —Ross 1944: 1278 [species key to males]. —Marshall 1979b: 191 [generic review]. —Blickle 1979: 7 [key to species of America north of Mexico]. —Harris and Holzenthal 1990: 453 [revision; key]. —Moulton and Stewart 1996: 109 [key to species of the Interior Highlands of North America].

*Mayatrichia* currently consists of seven species occurring widely through North and Central America. Diagnostic characteristics of the adults include features of the thorax and male genitalia (Harris and Holzenthal 1990). Descriptions of the larvae and cases have been published for *M.ayama* (Ross 1944) and *M.ponta* (Wiggins 1996).

***acuna*** Ross, 1944: 279 [type locality: [United States], Texas, Del Rio, San Felipe Springs; INHS; ♂]. —Edwards 1973: 506 [distribution]. —Blickle 1979: 50, 59 [checklist; ♂]. —Moulton and Stewart 1997: 350 [checklist]. —Blinn and Ruiter 2005: 69 [distribution; biology]. —Blinn and Ruiter 2006: 333 [biology; distribution]. —Bowles et al. 2007: 21 [distribution; biology].

**Distribution.** —U.S.A.

***ayama*** Mosely, 1937b: 182 [type locality: Mexico, Guerrero, Cocula; NHMUK; ♂]. —Ross 1944: 160 [♂; larva; case; distribution]. —Denning 1947b: 176 [distribution]. —Denning 1947a: 146 [distribution]. —Etnier 1965: 147 [checklist]. —Edwards 1973: 506 [distribution]. —Roy and Harper 1975: 1083 [distribution]. —Bueno-Soria and Flint 1978: 202 [distribution]. —Roy and Harper 1979: 152 [checklist]. —Etnier and Schuster 1979: 18 [distribution]. —Blickle 1979: 50, 57 [checklist; ♂]. —Parker and Voshell 1981: 4 [checklist]. —Roy and Harper 1981: 105 [distribution]. —Harris et al. 1982a: 511 [distribution]. —Harris et al. 1982b: 81 [distribution]. —Huryn and Foote 1983: 791 [distribution]. —Hamilton et al. 1983: 18 [distribution]. —Harris et al. 1984: 108 [distribution]. —Holzenthal 1988: 61 [distribution]. —Morse et al. 1989: 22 [distribution]. —Harris and Holzenthal 1990: 458 [♀; distribution]. —Harris et al. 1991: 218 [distribution]. —Frazer et al. 1991: 20 [distribution]. —Masteller and Flint 1992: 70 [checklist]. —Bowles and Mathis 1992: 32 [distribution]. —Moulton et al. 1993: 21 [distribution]. —Floyd et al. 1993: 91 [phenology; distribution]. —Moulton et al. 1994: 170 [distribution]. —Moulton and Stewart 1996: 109 [♂; distribution]. —Abbott et al. 1997: 44 [distribution]. —Moulton and Stewart 1997: 350 [checklist]. —Maes 1999: 1194 [checklist]. —Huryn and Harris 2000: 193 [distribution]. —Houghton et al. 2001: 505 [distribution]. —Pescador et al. 2004: 133 [checklist]. —Blinn and Ruiter 2005: 69 [distribution; biology]. —DeWalt and Heinold 2005: 42 [phenology; distribution]. USA —Baumgardner and Bowles 2005: 11 [distribution]. —Blinn and Ruiter 2006: 333 [biology; distribution]. —Blinn and Ruiter 2009a: 305 [biology]. —Bowles et al. 2007: 21 [distribution; biology]. —Chamorro-Lacayo et al. 2007: 43 [checklist]. —Etnier 2010: 485 [distribution]. —Armitage et al. 2011: 14 [checklist]. —Flint 2011: 104 [checklist]. —Myers et al. 2011: 107 [distribution]. —Harris et al. 2012: 8 [checklist]. —Denson et al. 2016: 5 [distribution]. —Houghton et al. 2017: 63 [checklist]. —Barba-Álvarez et al. 2019: 85 [distribution].

**Distribution.** —Canada, Costa Rica, Honduras, Mexico, Nicaragua, U.S.A.

***illobia*** Harris & Holzenthal, 1990: 456 [type locality: Costa Rica, Puntarenas, Río Guineal, ca. 1 km (air) E Finca Helechales, 9.076°N, 83.092°W; NMNH; ♂; ♀]. —Ríos-Touma et al. 2017: 10 [checklist]. —Armitage et al. 2020: 4 [distribution].

**Distribution.** —Costa Rica, Ecuador, Panama.

***moselyi*** Blickle & Denning, 1977: 297 [type locality: [United States], Utah, Sevier River, near Rock Canyon Mountain; ESUW; ♂; ♀]. —Blickle 1979: 50, 59 [checklist; ♂].

**Distribution.** —U.S.A.

***ponta*** Ross, 1944: 278 [type locality: [United States], Oklahoma, Turner Falls State Park, along Honey Creek; INHS; ♂]. —Blickle 1979: 50, 59 [checklist; ♂]. —Bowles and Mathis 1992: 32 [distribution]. —Moulton and Stewart 1996: 110 [♂; distribution]. —Moulton and Stewart 1997: 350 [checklist]. —Blinn and Ruiter 2005: 69 [distribution; biology]. —Wang and Kennedy 2004: 523 [distribution; life history]. —Bowles et al. 2007: 21 [distribution; biology].

**Distribution.** —U.S.A.

***rualda*** Mosely, 1937b: 183 [type locality: Mexico, Chiapas, Barranca Honda; NHMUK; ♂]. —Bueno-Soria and Flint 1978: 202 [distribution]. —Holzenthal 1988: 62 [distribution]. —Harris and Holzenthal 1990: 456, 459 [♀; distribution]. —Chamorro-Lacayo et al. 2007: 43 [checklist].

**Distribution.** —Costa Rica, Mexico, Nicaragua.

***tuscaloosa*** Harris & Sykora, 1996: 23 [type locality: [United States], Alabama, Tuscaloosa County, Big Sandy Creek at 4.5 mi south of Coaling on an unnumbered county road; CMNH; ♂]. —Harris et al. 1991: 235 [distribution]. —Frazer and Harris 1991a: 366–367[♂]. —Harris and Flint 2016: 7 [♂; distribution].

**Distribution.** —U.S.A.

#### Genus *Neotrichia* Morton, 1905

*Cyllene* Chambers, 1873: 124 [type species *Cylleneminutisimella* Chambers, 1873, monotypic. Preoccupied several times, *vide* Fischer, 1961].

*Neotrichia* Morton, 1905: 72 [type species: *Neotrichiacollata* Morton, 1905, monotypic]. —Ross 1944: 154 [species key for adults]. —Marshall 1979b: 189 [generic review]. —Blickle 1979: 21 [key to species of America north of Mexico]. —Flint 1991b: 53 [key to Antioquian species]. —Moulton and Stewart 1996: 111 [key to species of the Interior Highlands of North America]. —Harris and Rasmussen 2010: 25, 42 [key to males and females of the *Neotrichiacaxima* group in the southeastern United States].—Oláh and Johanson 2011: 168 [species groups reviewed]. —Keth et al. 2015: 19 [keys to species in North America Mexico and the Caribbean Islands].

*Microsiphon* Müller, 1921: 525 [type species: no species ever included. Preoccupied by del Guercio, 1907]. —Flint et al. 1999b: 77 [to synonymy].

*Exitrichia* Mosely, 1937b: 170 [type species: *Exitrichiaanahua* Mosely, 1937b, original designation]. —Ross 1944: 154 [to synonymy].

*Dolotrichia* Mosely, 1937b: 177 [type speies: *Dolotrichiacanixa* Mosely, 1937b, original designation]. —Ross 1944: 154 [to synonymy].

*Guerrotrichia* Mosely, 1937b: 179 [type species: *Guerrotrichiacaxima* Mosely, 1937b, original designation]. —Ross 1944: 154 [to synonymy].

*Lorotrichia* Mosely, 1937b: 181 [type species: *Lorotrichiahiaspa* Mosely, 1937b, original designation]. —Ross 1944: 154 [to synonymy].

*Neotrichia* consists of 205 species occurring in North, Central, and South America and the West Indies and is one of the most species-rich hydroptilid groups in the Neotropics (Flint et al. 1999a). Marshall (1979b) commented that *Neotrichia* could be divided into groups that correspond roughly to genera that were originally described by Mosely (1937b) and subsequently synonymized by Ross (1944). However, the addition of many new species to the genus has since weakened Marshall’s diagnosis of the species groups. *Neotrichia* can be separated from other genera in Neotrichiinae by the tibial spur formula (0, 2, 3) and features of the complicated male genitalia. Larvae of *N.minutisimella* were first described by Ross (1944) and many others have been described since (Flint 1964; Botosaneanu 1994b; Wiggins 1996).

***abbreviata*** Flint, 1983: 48 [type locality: Brazil, Edo. Santa Catarina, Nova Teutonia; NMNH; ♂]. —Angrisano 1995a: 511 [distribution]. —Angrisano 1999: 32 [checklist]. —Paprocki et al. 2004: 11 [checklist]. —Paprocki and França 2014: 46 [checklist].

**Distribution.** —Brazil, Uruguay.

***abbreviatoides*** Angrisano, 1995a: 513 [type locality: Uruguay, Artigas, Ao. da la Invernada; FHCU; ♂]. —Angrisano 1999: 32 [checklist].

**Distribution.** —Uruguay.

***aequispina*** Angrisano, 1995a: 515 [type locality: Uruguay, Tacuarembo, Tbo. Chico; FHCU; ♂]. —Angrisano 1999: 32 [checklist]. —Keth 2004: 172 [♂].

**Distribution.** —Uruguay.

***alabamensis*** Kelley & Harris, 1983: 182 [type locality: [United States], Alabama, Mobile County, Indian Grave Creek near junction with Cedar Creek, 4 miles east of Citronelle; NMNH; ♂]. —Harris et al. 1984: 108 [distribution]. —Harris et al. 1991: 219 [distribution]. —Pescador et al. 2004: 133 [checklist]. —Harris and Rasmussen 2010: 34 [♂; ♀; distribution]. —Harris et al. 2012: 8 [checklist]. —Keth et al. 2015: 40 [♂].

**Distribution.** —U.S.A.

***alata*** Flint, 1968b: 37 [type locality: Jamaica, Portland, Rio Grande at Fellowship; NMNH; ♂; ♀] —Flint 1968a: 81 [checklist]. —Botosaneanu 1979: 51 [distribution]. —Flint 1996a: 16 [checklist]. —Botosaneanu 2002b: 85 [checklist]. —Naranjo López and González Lazo 2005: 149 [checklist]. —Keth et al. 2015: 56 [♂].

**Distribution.** —Cuba, Jamaica.

***alsa*** Oláh & Johanson, 2011: 169 [type locality: Peru, San Martin Prov., Rio Negro, 37 km (rd.) W Moyobamba, near Olmos-Tarapoto rd., 6°00.278'S, 77°15.437'W; NHRS; ♂].

**Distribution.** —Peru.

***alyshae*** Keth in Keth et al. 2015: 57 [type locality: Mexico, Sonora, Rio Aros at Arroyo El Pavo; NMNH; ♂].

**Distribution.** —Mexico.

***amplector*** Keth, 2004: 164 [type locality: Mexico, Tabasco, Teapa, Grutas de Colona, Rio Puyacatengo; NMNH; ♂]. —Keth et al. 2015: 58 [♂].

**Distribution.** —Mexico.

***amplio*** Keth, 2004: 164 [type locality: Belize, Orange Walk District, New River Lagoon, dock area at Lamanai Ruins; NMNH; ♂].

**Distribution.** —Belize.

***anahua*** (Mosely, 1937b): 170 [type locality: Mexico, Chiapas, Dolores; NHMUK; ♂; in *Exitrichia*]. —Bueno-Soria and Flint 1978: 202 [distribution]. —Bueno-Soria et al. 2007: 33 [distribution]. —Keth et al. 2015: 59 [♂].

**Distribution.** —Mexico.

***anaua*** Gama Neto & Passos, 2020: 180 [type locality: Brazil, Roraima, Iracema municipality, Vicinal Campos Novos (Fazenda Rancho Fundo), small order stream, 2°21'26.22"N, 61°23'38.98"W, 209 m a.s.l.; MPEG; ♂].

**Distribution.** —Brazil.

***angulata*** Flint, 1983: 48 [type locality: Uruguay, Dpto. Artigas, Arroyo de la Invernada; NMNH; ♂]. —Angrisano 1995a: 511 [distribution]. —Angrisano 1999: 32 [checklist].

**Distribution.** —Uruguay.

***anzuelo*** Armitage & Harris, 2018a: 4 [type locality: Panama, Chiriquí Province, Cuenca 108, Quebrada Jaramillo, Jaramillo Centro road bridge, 8.75454°N, 82.41848°W, 1075 m; COZEM; ♂]. —Armitage and Harris 2018b: 98 [checklist]. —Armitage and Harris 2018c: 283 [distribution; as *anzuela*].

**Distribution.** —Panama.

***arista*** Harris, 1990: 251 [type locality: Venezuela, Territorio Federal Amazonas, Río Cataniapo, 10 km S Puerto Ayacucho; NMNH; ♂].

**Distribution.** —Venezuela.

***arkansasensis*** Mathis & Bowles, 1990: 90 [type locality: [United States], Arkansas, Madison County, Kings River, 5 mi S Kingston, NW 1/4, SW 1/4, Sect. 4, T 15 N, R 24 W; NMNH; ♂]. —Moulton and Stewart 1996: 112 [♂; distribution]. —Keth et al. 2015: 94 [♂].

**Distribution.** —U.S.A.

***armata*** Botosaneanu in Botosaneanu and Alkins-Koo 1993: 20 [type locality: Tobago, Argyll River below Argyll waterfall; ZMUA; ♂]. —Botosaneanu and Sakal 1992: 202 [distribution; ecology]. —Flint 1996b: 100 [distribution]. —Botosaneanu 2002b: 85 [checklist]. —Keth et al. 2015: 95 [♂].

—species B Botosaneanu and Alkins-Koo, 19923: 23 [♀]. —Flint 1996b: 100 [distribution; to synonymy].

**Distribution.** —Tobago, Trinidad.

***armitagei*** Harris, 1991: 15 [type locality: [United States], Florida, Okaloosa County, Turkey Gobble Creek at Base Rd. 211, Eglin Air Force Base, 11.2 km NW Niceville; NMNH; ♂]. —Harris and Keth 2002: 76 [♂]. —Pescador et al. 2004: 133 [checklist]. —Harris and Rasmussen 2010: 30 [♂; ♀; distribution]. —Harris et al. 2012: 8 [checklist]. —Keth et al. 2015: 41 [♂]. —Denson et al. 2016: 5 [distribution].

**Distribution.** —U.S.A.

***atopa*** Thomson & Armitage, 2018: 4 [type locality: Panama, Chiriquí Province, Cuenca 102 (Río Chiriquí Viejo), un. trib., Río Colorado, Mount Totumas Biological Reserve, 8.884717°N, 82.684077°W; COZEM; ♂].

**Distribution.** —Panama.

***baritu*** Angrisano, 1984: 4 [type locality: Argentina, Salta, Parque Nacional Baritú; MACN; ♂; ♀]. —Angrisano 1999: 32 [checklist]. —Isa Miranda and Rueda Martin 2014: 199 [distribution].

**Distribution.** —Argentina.

***bellini*** Santos & Nessimian, 2009a: 760 [type locality: Brazil, Amazonas, Rio Preto da Eva (tributary to Rio Preto da Eva, 02°36'45.5"S 59·43'59.1"W); INPA; ♂]. —Paprocki and França 2014: 199 [distribution].

**Distribution.** —Brazil.

***bifida*** Flint, 1974b: 77 [type locality: Suriname, Lawa River, Anapaike; RMNH; ♂]. —Oláh and Johanson 2011: 170 [distribution].

**Distribution.** —French Guiana, Suriname.

***bifurcata*** Harris in Flint and Sykora 2004: 34 [type locality: Dominican Republic, Pedernales Province, Rio Mulito, 13 k N Pedernales, 18°09'N 71°46'W, 230 m; CMNH; ♂]. —Pérez-Gelabert 2008: 300 [checklist]. —Keth et al. 2015: 104 [♂].

**Distribution.** —Dominican Republic.

***bika*** Oláh & Johanson, 2011: 170 [type locality: French Guiana, Approuaguekaw, Kaw Mt, 4°33.035'N, 52°11.661'W. 104 m; NHRS; ♂].

**Distribution.** —French Guiana.

***biuncifera*** Flint, 1974b: 72 [type locality: Suriname, Käyser Airstrip; RMNH; ♂]. —Ríos-Touma et al. 2017: 10 [distribution].

**Distribution.** —Ecuador, Suriname.

***blinni*** Ruiter, 2007: 276 [type locality: USA, Arizona, Cochise County, South Fork Cave Creek; NMNH; ♂]. —Keth et al. 2015: 76 [♂].

**Distribution.** —U.S.A.

***botka*** Oláh & Johanson, 2011: 172 [type locality: French Guiana, Approuaguekaw, Kaw Mt, 4°33.035'N, 52°11.551'W, 104 m; NHRS; ♂].

**Distribution.** —French Guiana.

***botonia*** Harris, 1990: 254 [type locality: Venezuela, Territorio Federal Amazonas, San Carlos de Río Negro; NMNH; ♂].

**Distribution.** —Venezuela.

***brevispina*** Flint, 1983: 51 [type locality: Argentina, Pcia. Misiones, Arroyo Coatí, 13 km E San José; NMNH; ♂]. —Angrisano 1995a: 511 [distribution]. —Angrisano 1999: 32 [checklist]. —Angrisano and Sganga 2007: 38 [♂; distribution].

**Distribution.** —Argentina, Uruguay.

***browni*** Harris, 1990: 248 [type locality: Venezuela, Territorio Federal Amazonas, San Carlos de Río Negro; NMNH; ♂]. —Santos and Nessimian 2009a: 766 [distribution]. —Paprocki and França 2014: 46 [checklist].

**Distribution.** —Brazil, Venezuela.

***buenoi*** Harris & Flint, 2016: 2 [type locality: Mexico, Veracruz, Las Tuxtlas, Rio Palma above La Palma; NMNH; ♂].

**Distribution.** —Mexico.

***bullata*** Flint, 1974b: 71 [type locality: Suriname, Käyser Airstrip; RMNH; ♂]. —Angrisano 1995a: 511 [distribution]. —Angrisano 1999: 32 [checklist]. —Oláh and Johanson 2011: 173 [distribution].

**Distribution.** —French Guiana, Suriname, Uruguay.

***caboca*** Gama Neto & Passos, 2019: 518 [type locality: Brazil, Roraima, Mucajai municipality, RR-325, Vicinal Apiaú, Igarapé Serrinha (Sítio Vaca), 02°33'7.78"N, 61°18'45.06"W, 74 m a.s.l.; MPEG; ♂].

**Distribution.** —Brazil.

***cameria*** (Mosely, 1937b): 180 [type locality: Mexico, Guerrero, Cocula; NHMUK; ♂; in *Guerrotrichia*]. —Bueno-Soria and Flint 1978: 202 [distribution]. —Keth et al. 2015: 42 [♂].

**Distribution.** —Mexico.

***canixa*** (Mosely, 1937b): 177 [type locality: Mexico, Chiapas, Dolores; NHMUK; ♂; in *Dolotrichia*]. —Bueno-Soria and Flint 1978: 202 [distribution]. —Armitage et al. 2015b: 5 [distribution]. —Armitage et al. 2015a: 7 [checklist]. —Keth et al. 2015: 26 [♂]. —Armitage and Harris 2018b: 98 [checklist]. —Armitage and Harris 2018c: 283 [distribution].

**Distribution.** —Mexico, Panama, U.S.A.

***capitiana*** Gama Neto & Passos, 2019: 519 [type locality: Brazil, Roraima, Mucajai municipality, RR-325, Vicinal 9, Apiaú (Sítio Sr. Nonato), small order stream, 2°31'00.78"N, 61°21'44.28"W, 104 m a.s.l.; MPEG; ♂].

**Distribution.** —Brazil.

***carlsoni*** Harris & Armitage, 2019: 12 [type locality: Panama, Bocas del Toro Province, Quebrada Rambala, near Rambala Jungle Lodge, 3.74 km SSE Rambala, 8.91627°N and 82.15469°W, 120 m; COZEM; ♂].

**Distribution.** —Panama.

***catrimani*** Gama Neto & Passos, 2020: 181 [type locality: Brazil, Roraima, Iracema municipality, Vicinal Campos Novos (Fazenda Rancho Fundo), small order stream, 2°21'26.22"N, 61°23'38.98"W, 209 m a.s.l.; MPEG; ♂].

**Distribution.** —Brazil.

***cauame*** Gama Neto & Passos, 2020: 182 [type locality: Brazil, Roraima, Iracema municipality, Vicinal Campos Novos (Fazenda Rancho Fundo), small order stream, 2°21'26.22"N, 61°23'38.98"W, 209 m a.s.l.; MPEG; ♂].

**Distribution.** —Brazil.

***caxima*** (Mosely, 1937b): 179 [type locality: Mexico, Guerrero, Cocula; NHMUK; ♂; in *Guerrotrichia*]. —Bueno-Soria and Flint 1978: 202 [distribution]. —Blickle 1979: 50, 75 [checklist; ♂]. —Keth et al. 2015: 43 [♂].

**Distribution.** —Mexico.

***cayada*** Harris in Harris and Davenport 1992: 455 [type locality: Venezuela, Territorio Federal Amazonas, Cerro de Neblina, basecamp near Rio Baria; NMNH; ♂].

**Distribution.** —Venezuela.

***chana*** Angrisano, 1995a: 513 [type locality: Uruguay, Tacuarembo, Tbo. Chico; FHCU; ♂]. —Angrisano 1999: 32 [checklist].

**Distribution.** —Uruguay.

***charrua*** Angrisano, 1984: 1 [type locality: Argentina, Entre Rios, Parque Nacional el Palmar; MACN; ♂; ♀]. —Angrisano 1999: 32 [checklist]. —Angrisano and Sganga 2007: 40 [♂; ♀; distribution].

**Distribution.** —Argentina.

***chihuahua*** Harris & Flint, 2016: 2 [type locality: Mexico, Chihuahua, Rio Concheno at Highway 16 near Basaseachic; NMNH; ♂].

**Distribution.** —Mexico.

***chilensis*** Flint, 1983: 53 [type locality: Chile, Pcia. Linares, Puente Malcho, Río Longavi; NMNH; ♂]. —Angrisano 1999: 33 [checklist].

**Distribution.** —Argentina, Chile.

***cliffordi*** Keth in Keth et al. 2015: 60 [type locality: U.S.A., Utah, Moab; CAS; ♂].

**Distribution.** —U.S.A (Utah).

***collata*** Morton, 1905: 72 [type locality: [United States], Ithaca, New York; type depository not desginated; ♂]. —Banks 1907a: 50 [catalogue]. —Betten 1934: 163 [wings; palpi; distribution]. —Ross 1944: 159 [♂; ♀; distribution]. —Blickle 1979: 50, 75 [checklist; ♂]. —Morse et al. 1989: 23 [distribution]. —Harris et al. 1991: 220 [distribution]. —Frazer et al. 1991: 20 [distribution]. —Floyd and Morse 1993: 177 [distribution]. —Moulton and Stewart 1996: 112 [♂; distribution]. —Etnier 2010: 486 [distribution]. —Myers et al. 2011: 107 [distribution]. —Flint 2014: 90 [distribution]. —Keth et al. 2015: 61 [♂].

**Distribution.** —U.S.A.

***collierorum*** Armitage & Harris, 2018a: 4 [type locality: Panama, Chiriquí Province, Cuenca 108, Quebrada Jaramillo, Alto Jaramillo Road, 8.76671°N, 82.41341°W, 1253 m; COZEM; ♂]. —Armitage and Harris 2018b: 98 [checklist]. —Armitage and Harris 2018c: 283 [distribution].

**Distribution.** —Panama.

***colmillosa*** Harris, 1990: 246 [type locality: Venezuela, Territorio Federal Amazonas, Cerro de Neblina basecamp; NMNH; ♂]. —Santos and Nessimian 2009a: 766 [distribution]. —Paprocki and França 2014: 46 [checklist].

**Distribution.** —Brazil, Venezuela.

***colombiensis*** Harris, 1990: 257 [type locality: Colombia, Antioquia Department, Quebrada la Jiménez, Sopetrán; NMNH; ♂]. —Flint 1991b: 53 [♂; distribution]. —Muñoz-Quesada 2000: 278 [checklist].

**Distribution.** —Colombia.

***connori*** Keth in Keth et al. 2015: 77 [type locality: Mexico, Nuevo Leon, Municipio de Sanchez, Arroyo San Juan on road to Laguna de Sanchez, 3.5 km west of La Cienegra, 25.4°N, 100.2833°W; PSUC; ♂].

**Distribution.** —Mexico.

***contrerasi*** Harris & Flint, 2016: 3 [type locality: Mexico, Nuevo Leon, Municipio de Sasntiago, Rio Ramos at Los Adjuntas, 4.5 km southeast Puerto Genoveno, N25°18', W100°08'; NMNH; ♂].

**Distribution.** —Mexico.

***corniculans*** Flint, 1968a: 50 [type locality: Dominica, D’leau Gommier; NMNH; ♂]. —Flint 1968a: 81 [checklist]. —Flint 1974b: 76 [♂; distribution]. —Flint and Sykora 1993: 49 [checklist]. —Harris and Tiemann 1993: 292 [♂]. —Botosaneanu 2002b: 85 [checklist]. —Keth et al. 2015: 27 [♂].

**Distribution.** —Dominica, Suriname.

***cruviana*** Gama Neto & Passos, 2019: 520 [type locality: Brazil, Roraima, Iracema municipality, Vicinal Campos Novos (Fazenda Rancho Fundo), small order stream, 2°21'26.22"N, 61°23'38.98"W, 209 m a.s.l.; MPEG; ♂].

**Distribution.** —Brazil.

***cuernuda*** Harris, 1990: 248 [type locality: Venezuela, Territorio Federal Amazonas, Agua Blanca, Cerro de la Neblina; NMNH; ♂].

**Distribution.** —Venezuela.

***damurida*** Gama Neto & Passos, 2019: 522 [type locality: Brazil, Roraima, Iracema municipality, Vicinal Campos Novos (Fazenda Rancho Fundo), small order stream, 2°21'26.22"N, 61°23'38.98"W, 209 m a.s.l.; MPEG; ♂].

**Distribution.** —Brazil.

***delgadeza*** Harris in Harris and Davenport 1992: 458 [type locality: Ecuador, Pastaza, Tzapino; NMNH; ♂]. —Ríos-Touma et al. 2017: 10 [checklist].

**Distribution.** —Ecuador.

***diabolica*** Bueno-Soria & Barba-Álvarez, 2018: 365 [type locality: Mexico, Chiapas, Mpio. Ocosingo, Reserva de la Biósfera Montes azules, Est. Biol. Tzendales (embarcadero) Río Tzendales, 16°17'49.09"N, 90°53'06.21"W, 144 m a.s.l.; CNIN; ♂].

**Distribution.** —Mexico.

***didii*** Santos & Nessimian, 2009a: 763 [type locality: Brazil, Amazonas, Rio Preto da Eva (Tributary to Rio Preto da Eva, 02°38'14.6"S 59°44'09.9"W; INPA; ♂]. —Paprocki and França 2014: 47 [checklist].

**Distribution.** —Brazil.

***dientera*** Harris, 1990: 251 [type locality: Venezuela, Territorio Federal Amazonas, San Carlos de Río Negro; NMNH; ♂].

**Distribution.** —Venezuela.

***digitata*** (Mosely, 1937b): 171 [type locality: Mexico, Guerrero, Cocula; NHMUK; ♂; in *Exitrichia*]. —Bueno-Soria and Flint 1978: 202 [distribution]. —Keth et al. 2015: 62 [♂].

**Distribution.** —Mexico.

***dikeros*** Flint, 1983: 48 [type locality: Argentina, Pcia. Entre Ríos, Arroyo P. Verne, 4 km N Villa San José; NMNH; ♂]. —Angrisano 1995a: 511 [distribution]. —Angrisano 1999: 33 [checklist].

**Distribution.** —Argentina, Uruguay.

***djalmasantosi*** Santos & Nessimian, 2009a: 759 [type locality: Brazil, Amazonas, Rio Preto da Eva (tributary to Rio Preto da Eva, 02°36'45.5"S 59°43'59.1"W); INPA; ♂]. —Paprocki and França 2014: 47 [checklist].

**Distribution.** —Brazil.

***doppelganger*** Keth in Keth et al. 2015: 63 [type locality: [United States], Alabama, Marion Co., North Fork Creek at Hwy. 17; PSUC; ♂].

**Distribution.** —U.S.A.

***downsi*** Ruiter, 1990: 88 [type locality: [United States], Colorado, Jackson County, Ginger Quill Ranch at the North Platte River, altitude 2,370 meters (7,700 feet) above mean sea level; INHS; ♂]. —Keth et al. 2015: 78 [♂].

**Distribution.** —U.S.A.

***dracanamalama*** Harris in Harris and Rasmussen 2010: 36 [type locality: [United States], Virginia, Middlesex County, Dragon Run Swamp, Rt. 603, 3 mi. S Warner, N37.380, W76.418; NMNH; ♂; ♀]. —Keth et al. 2015: 44 [♂].

**Distribution.** —U.S.A.

***dubitans*** (Mosely, 1939a): 235 [type locality: Brazil, Edo. Santa Catarina, Nova Teutonia; NHMUK; ♂; in *Dolotrichia*?]. —Angrisano 1999: 33 [checklist]. —Paprocki et al. 2004: 11 [checklist]. —Dumas et al. 2009: 366 [distribution]. —Dumas and Nessimian 2012: 15 [checklist]. —Paprocki and França 2014: 47 [checklist].

**Distribution.** —Brazil.

***durior*** Flint, 1983: 49 [type locality: Brazil, Edo. Santa Catarina, Nova Teutonia; NMNH; ♂]. —Angrisano 1995a: 511 [distribution]. —Angrisano 1999: 33 [checklist]. —Paprocki et al. 2004: 11 [checklist]. —Paprocki and França 2014: 47 [checklist].

**Distribution.** —Brazil, Uruguay.

***edalis*** Ross, 1941a: 62 [type locality: [United States], Oklahoma, along Pennington Creek, Reagan; INHS; ♂]. —Ross 1944: 138 [♂; ♀]. —Blickle 1979: 50, 77 [checklist;] . —Bowles and Mathis 1992: 32 [distribution]. —Moulton and Stewart 1996: 113 [♂; distribution]. —Moulton and Stewart 1997: 350 [checklist]. —Bowles et al. 2007: 21 [distribution; biology]. —Keth et al. 2015: 79 [♂]. —Bowles et al. 2020: 8 [distribution].

**Distribution.** —U.S.A.

***elongata*** Flint, 1983: 53 [type locality: Argentina, Pcia. Salta, Cañada la Gotera, Rt. 59, km 23.5; NMNH; ♂]. —Angrisano 1999: 33 [checklist]. —Muzón et al. 2005: 57 [distribution]. —Rueda Martín 2011: 7 [♂; distribution].

**Distribution.** —Argentina.

***eroga*** (Mosely, 1937b): [type locality: Mexico, Guerrero, Cocula; NHMUK; ♂; in *Exitrichia*]. —Bueno-Soria and Flint 1978: 202 [distribution]. —Keth et al. 2015: 80 [♂].

**Distribution.** —Mexico.

***ersitis*** Denning, 1947a: 152 [type locality: [Canada], Saskatchewan, Saskatoon; ESUW; ♂; ♀]. Blickle 1979: 50, 75 [checklist; ♂]. —Keth et al. 2015: 64 [♂].

**Distribution.** —Canada.

***esmalda*** (Mosely, 1937b): 173 [type locality: Mexico, Chiapas, Esmeralda; NHMUK; ♂; in *Exitrichia*]. —Bueno-Soria and Flint 1978: 202 [distribution]. —Holzenthal 1988: 62 [distribution]. —Maes 1999: 1194 [checklist]. —Bueno-Soria et al. 2005: 75 [distribution]. —Chamorro-Lacayo et al. 2007: 43 [checklist]. —Keth et al. 2015: 72 [♂]. —Armitage et al. 2016: 9 [distribution]. —Armitage and Harris 2018b: 98 [checklist].

**Distribution.** —Costa Rica, Mexico, Nicaragua, Panama.

***espinosa*** Armitage & Harris, 2020a: 5 [type locality: Panama, Coclé Province, Cuenca 134, Omar Torrijos Herrera National Park, Quebrada Las Yayas, PSPSCB-PNGDOTH-C134-2017-004, 8.66168°N, 80.5952°W, 728 m; COZEM; ♂; see Armitage and Harris 2020b errata]. —Armitage and Harris 2020b: 2 [correction to original illustration].

**Distribution.** —Panama.

***exicoma*** (Mosely, 1937b): 174 [type locality: Mexico, Chiapas, Dolores; NHMUK; ♂; in *Exitrichia*]. —Bueno-Soria and Flint 1978: 203 [distribution]. —Keth et al. 2015: 81 [♂].

**Distribution.** —Mexico.

***falca*** Ross, 1938a: 119 [type locality: [United States], Illinois, Muncie, along Stony Creek; INHS; ♂]. —Ross 1944: 159 [♂; ♀; distribution]. —Blickle 1979: 50, 77 [checklist; ♂]. —Huryn and Foote 1983: 791 [distribution]. —Hamilton et al. 1983: 18 [distribution]. —Floyd et al. 1993: 91 [phenology; distribution]. —Houghton et al. 2001: 505 [distribution]. —Harris and Rasmussen 2010: 28 [♂; ♀; distribution]. —Armitage et al. 2011: 14 [checklist]. —Keth et al. 2015: 45 [♂].

**Distribution.** —U.S.A.

***falcifera*** Flint, 1974b: 75 [type locality: Suriname, Nickerie River, Blanche Marie Falls; RMNH; ♂]. —Angrisano 1999: 33 [checklist].

**Distribution.** —Suriname.

***farkoska*** Oláh & Johanson, 2011: 173 [type locality: French Guiana, Approuaguekaw, Kaw Mt, 4°33.935'N, 52°11.661'W, 104 m; NHRS; ♂].

**Distribution.** —French Guiana.

***felkurta*** Oláh & Johanson, 2011: 175 [type locality: French Guiana, Sinnamary, Petit Saut, 05°03.853'N, 053°02.814'W, 9 m; NHRS; ♂].

**Distribution.** —French Guiana.

***feolai*** Santos & Nessimian, 2009a: 766 [type locality: Brazil, Amazonas, Rio Preto da Eva (tributary to Rio Preto da Eva, 02°38'14.6"S 59°44'09.9"W); INPA; ♂]. —Thomson and Holzenthal 2012: 25 [re-description; distribution]. —de Souza et al. 2013: 586 [distribution]. —Paprocki and França 2014: 47 [checklist].

**Distribution.** —Brazil, Venezuela.

***filifera*** Flint, 1983: 46 [type locality: Uruguay, Dpto. Lavalleja, Río Cebollati, Picada de Rodriguez; NMNH; ♂]. —Harris and Davenport 1992: 461 [re-description]. —Angrisano 1995a: 511 [distribution]. —Angrisano 1999: 33 [checklist]. —Blahnik et al. 2004: 5 [distribution]. —Paprocki et al. 2004: 11 [checklist]. —de Souza et al. 2013: 586 [distribution]. —Paprocki and França 2014: 47 [checklist].

**Distribution.** —Brazil, Uruguay.

***flowersi*** Harris, 1990: 257 [type locality: Panama, Bocas del Toro Province, Quebrada Canza at pipeline road; NMNH; ♂]. —Armitage et al. 2015a: 7 [checklist]. —Armitage and Harris 2018b: 98 [checklist].

**Distribution.** —Panama.

***fogaka*** Oláh & Johanson, 2011: 176 [type locality: French Guiana, Approuaguekaw, Kaw Mt, 4°33.235'N, 52°11.988'W, 225 m; NHRS; ♂].

**Distribution.** —French Guiana.

***garra*** Keth, 2004: 170 [type locality: Belize, Orange Walk District, New River Lagoon, dock area at Lamanai Ruins; NMNH; ♂].

**Distribution.** —Belize.

***garrinichai*** Santos & Nessimian, 2009a: 764 [type locality: Brazil, Amazonas, Manaus (Igarapé Arumã, tributary to Rio Cuieiras,02°30'55.2"S 60°15'44.4"W); INPA; ♂]. —Paprocki and França 2014: 47 [checklist].

**Distribution.** —Brazil.

***gilaensis*** Keth in Keth et al. 2015: 65 [type locality: U.S.A., Arizona, Gila Co., Tonto Fish Hatchery, 34.3400°N 111°.0598°W, 1628 m; NMNH; ♂].

**Distribution.** —U.S.A (Arizona).

***gilmari*** Santos & Nessimian, 2009a: 759 [type locality: Brazil, Amazonas, Rio Preto da Eva (tributary to Rio Urubu, 02°31'01.3"S 59°43'13.7"W; INPA; ♂]. —Paprocki and França 2014: 47 [checklist].

**Distribution.** —Brazil.

***gladia*** Keth in Keth et al. 2015: 82 [type locality: United States, Arizona, Coco Co., Grand Canyon National Park, mouth of Kansas Creek; INHS; ♂].

**Distribution.** —U.S.A.

***gotera*** Flint, 1983: 51 [type locality: Argentina, Pcia. Salta, Cañada la Gotera, Rt. 59, km 23.5; NMNH; ♂]. —Flint and Reyes 1991: 487 [♂; distribution]. —Angrisano 1999: 33 [checklist]. —Rueda Martín 2011: 8 [♂; distribution]. —Isa Miranda and Rueda Martí 2014: 199 [distribution].

**Distribution.** —Argentina, Bolivia, Peru.

***grehani*** Keth, 2015 in Keth, Harris, and Armitage 2015: 83 [type locality: U.S.A., Oregon, Clatsop Co., vicinity of Gronnel Rd. approx, 2 miles E Elsie; CAS; ♂].

**Distribution.** —U.S.A.

***hajla*** Oláh & Johanson, 2011: 177 [type locality: French Guiana, Approuaguekaw, Kaw Mt, 4°33.035'N, 52°11.661'W, 104 m; NHRS; ♂; ♀].

**Distribution.** —French Guiana.

***halia*** Denning, 1947a: 153 [type locality: [United States], Wyoming, Bluegrass River, near Wheatland; ESUW; ♂]. —Etnier 1965: 147 [distribution]. —Roy and Harper 1979: 152 [distribution]. —Blickle 1979: 50, 75 [checklist; ♂]. —Houghton et al. 2001: 505 [distribution]. —Blinn and Ruiter 2005: 69 [distribution; biology]. —Blinn and Ruiter 2006: 333 [biology; distribution]. —Keth et al. 2015: 66 [♂]. —Houghton et al. 2017: 63 [checklist]. —Mendez et al. 2019: 118 [checklist].

—*numii* Ross, 1948: 205 [type locality: [United States], Colorado, Lake George, in 11-mile canyon of the South Platte River; INHS; ♂]. —Blickle 1979: 50 [to synonymy].

**Distribution.** —Canada, U.S.A.

***harrisi*** Bueno-Soria & Barba-Álvarez, 2018: 365 [type locality: Mexico, Chiapas, Reserva de la Biósfera Montes azules, Est. Biol. Chajul, Arroyo José, 16°06'50.05"N, 90°56'02.83"W, 145 m asl; CNIN; ♂].

**Distribution.** —Mexico.

***heleios*** Flint, 1968b: 38 [type locality: Jamaica, St. Catherine, Bog Walk; MCZ; ♂; ♀]. —Flint 1968a: 81 [checklist]. —Botosaneanu 2002b: 85 [checklist]. —Keth et al. 2015: 96 [♂].

**Distribution.** —Jamaica.

***hiaspa*** (Mosely, 1937b): 181 [type locality: Mexico, Chiapas, Dolores; NHMUK; ♂; in *Lorotrichia*]. —Ross 1944: 154 [to synonymy]. —Bueno-Soria and Flint 1978: 203 [distribution]. —Chamorro-Lacayo et al. 2007: 43 [checklist]. —Oláh and Johanson 2011: 179 [checklist]. —Keth et al. 2015: 67 [♂]. —Armitage et al. 2016: 9 [distribution]. —Armitage and Harris 2018b: 98 [checklist].

**Distribution.** —Mexico, Nicaragua, Panama.

***horgoska*** Oláh & Johanson, 2011: 179 [type locality: French Guiana, Maripasoula, Lawa River, Gzaan Dayé, 4°01.130'N, 54°19.015'W, 74 m; NHRS; ♂].

**Distribution.** —French Guiana.

***interrupta*** Flint, 1974b: 77 [type locality: Suriname, Lucie River camp, Wilhelmina Mountains; RMNH; ♂].

**Distribution.** —Suriname.

***iridescens*** Flint, 1964: 51 [type locality: Puerto Rico, Maricao, at fish hatchery; NMNH; ♂; ♀; larva; case]. —Flint 1968b: 37 [♂; ♀; larva; distribution]. —Flint 1968a: 48 [♂; ♀; larva; distribution]. —Botosaneanu 1979: 51 [distribution]. —Malicky 1983c: 264 [distribution]. —Kumanski 1987: 23 [distribution]. —Botosaneanu 1989: 99 [distribution]. —Botosaneanu 1991: 128 [distribution]. —Flint and Sykora 1993: 49 [checklist]. —Botosaneanu 1994a: 43 [distribution]. —Botosaneanu 1995a: 32 [distribution]. —Flint 1996a: 16 [checklist]. —Botosaneanu and Hyslop 1998: 18 [distribution]. —Flint and Pérez-Gelabert 1999: 40 [checklist]. —Botosaneanu 2000: 256 [distribution]. —Botosaneanu 2002b: 85 [checklist]. —Flint and Sykora 2004: 34 [distribution]. —Botosaneanu and Thomas 2005: 42 [distribution]. —Naranjo López and González Lazo 2005: 149 [checklist]. —Pérez-Gelabert 2008: 300 [checklist]. —Keth et al. 2015: 97 [♂].

**Distribution.** —Cuba, Dominica, Dominican Republic, Guadeloupe, Haiti, Jamaica, Martinique, Puerto Rico, St. Lucia.

***ismetla*** Oláh & Johanson, 2011: 180 [type locality: French Guiana, Approuaguekaw, Kaw Mt, 4°33.257'N, 52°11.920'W; NHRS; ♂].

**Distribution.** —French Guiana.

***jarochita*** Bueno-Soria, 1999: 114 [type locality: Mexico, Veracruz, Estación de Biología Los Tuxtlas, UNAM, Arroyo del Zoológico; CNIN; ♂]. —Oláh and Johanson 2011: 181 [distribution]. —Keth et al. 2015: 28 [♂]. —Harris and Flint 2016: 14 [♂]. —Barba-Álvarez et al. 2019: 85 [distribution].

**Distribution.** —Mexico.

***juani*** Harris & Tiemann, 1993: 286 [type locality: United States, Texas, Comal County, Honey Creek at Honey Creek Nature Preserve; NMNH; ♂]. —Moulton and Stewart 1997: 350 [checklist]. —Bowles et al. 2007: 21 [distribution; biology]. —Keth et al. 2015: 29 [♂].

**Distribution.** —U.S.A.

***juntada*** Harris in Harris and Davenport 1992: 465 [type locality: Peru, Loreto, tributary to Rio Yanomono at Explorama Lodge; NMNH; ♂].

**Distribution.** —Peru, Venezuela.

***kampa*** Oláh & Johanson, 2011: 182 [type locality: Peru, San Martin Prov., Rio Mayo, 11 km (rd.) E Mayobamba, 6°04.989'S, 76°53.065'W; NHRS; ♂].

**Distribution.** —Peru.

***kampoka*** Oláh & Johanson, 2011: 183 [type locality: Peru, San Martin Prov., Rio Negro, 37 km (rd.) W Moyobamba, near Olmos-Tarapoto rd., 6°00.278'S, 77°15.437'W; NHRS; ♂].

**Distribution.** —Peru.

***kehelia*** Oláh & Johanson, 2011: 184 [type locality: Peru, San Martin Prov., La Catarata de Ahuashiyascu, 6°27.544'S, 76°18.192'W; NHRS; ♂].

**Distribution.** —Peru.

***ketaguka*** Oláh & Johanson, 2011: 186 [type locality: French Guiana, Approuaguekaw, Kaw Mt, 4°33.035'N, 52°11.661'W, 104 m; NHRS; ♂].

**Distribution.** —French Guiana.

***kimi*** Keth in Keth et al. 2015: 84 [type locality: U.S.A., California, Ventura Co., Matilija Hot Springs; CAS; ♂]. —Mendez et al. 2019: 118 [checklist].

**Distribution.** —U.S.A.

***kitae*** Ross, 1941a: 60 [type locality: [United States], Missouri, Hollister; INHS; ♂; ♀]. —Blickle and Denning 1977: 288 [checklist]. —Blickle 1979: 51, 75 [checklist; ♂]. —Mathis and Bowles 1992: 24 [distribution]. —Moulton and Stewart 1996: 113 [♂; distribution]. —Keth et al. 2015: 30 [♂].

**Distribution.** —U.S.A.

***kurta*** Oláh & Johanson, 2011: 187 [type locality: Peru, San Martin Prov., Rio Huallaga tributary, small river passing Chazuta, 6°34.665'S, 76°08.209'W; NHRS; ♂].

**Distribution.** —Peru.

***kurtika*** Oláh & Johanson, 2011: 188 [type locality: French Guiana, Maripasoula, Lawa River, Gzaan Dayé, 4°01.130'N, 54°19.015'W, 74 m; NHRS; ♂].

**Distribution.** —French Guiana.

***kurtitva*** Oláh & Johanson, 2011: 190 [type locality: Peru, San Martin Prov., Rio Huallaga, at Pumarihri Huallaga Lodge, between Juan Guerra and Chazuta, 14 km (rd.) W Chazuta, 6°36.643'S, 76°12.555'W; NHRS; ♂].

**Distribution.** —Peru.

***labios*** Keth in Keth et al. 2015: 98 [type locality: Mexico, Sonora, Rio Arros at Arroyo El Pavo; NMNH; ♂].

**Distribution.** —Mexico.

***lacertina*** Botosaneanu, 1994a: 43 [type locality: Guadeloupe, River Lézarde, Saut de la Lézarde; ZMUA; ♂]. —Botosaneanu 2000: 256, 259 [♂; ♀; distribution]. —Botosaneanu 2002b: 85 [checklist]. —Botosaneanu and Thomas 2005: 42 [distribution]. —Keth et al. 2015: 68 [♂].

**Distribution.** —Guadeloupe, Martinique.

***lefela*** Oláh & Johanson, 2011: 191 [type locality: French Guiana, Approuaguekaw, Kaw Mt, 4°33.035'N, 52°11.661'W, 104 m; NHRS; ♂].

**Distribution.** —French Guiana.

***leonensis*** Keth in Keth et al. 2015: 85 [type locality: Mexico, Nuevo Leon, Municipio de Santiago, Arroyo San Juan on road to Laguna de Sanchez, 3.5 km west of La Cienegra, 25°24'N 100°17'W; PSUC; ♂].

**Distribution.** —Mexico.

***lobata*** Flint, 1974b: 79 [type locality: Suriname, Lucie River camp, Wilhelmina Mountains; RMNH; ♂].

**Distribution.** —Suriname.

***longissima*** Flint, 1983: 49 [type locality: Brazil, Edo. Santa Catarina, Nova Teuronia; NMNH; ♂]. —Angrisano 1999: 33 [checklist]. —Paprocki et al. 2004: 11 [checklist]. —Paprocki and França 2014: 48 [checklist].

**Distribution.** —Brazil.

***lucrecia*** Angrisano, 1995a: 513 [type locality: Uruguay, Artigas, Potrero Sucio, Ao. Tres Cruces; FHCU; ♂]. —Angrisano 1999: 33 [checklist].

**Distribution.** —Uruguay.

***macuxi*** Gama Neto & Passos, 2019: 523 [type locality: Brazil, Roraima, Mucajai municipality, RR-325, Vicinal Apiaú, Igarapé Serrinha (Sítio Vaca), 02°33'7.78"N, 61°18'45.06"W, 74 m a.s.l.; MPEG; ♂].

**Distribution.** —Brazil.

***makunaima*** Gama Neto & Passos, 2019: 523 [type locality: Brazil, Roraima, Mucajai municipality, RR-325, Vicinal Apiaú, Igarapé Serrinha (Sítio Vaca), 02°33'7.78"N, 61°18'45.06"W, 74 m a.s.l.; MPEG; ♂].

**Distribution.** —Brazil.

***malickyi*** Harris in Harris and Tiemann 1993: 288 [type locality: Panama, Barro Colorado Island, Lutz; NMNH; ♂]. —Armitage et al. 2015a: 7 [checklist]. —Armitage and Harris 2018b: 98 [checklist].

**Distribution.** —Panama.

***manopla*** Keth in Keth et al. 2015: 46 [type locality: Mexico, Sonora, Cajon Bonito, Losojas Ranch, 31.27854°N, 109.00196°W; NMNH; ♂].

**Distribution.** —Mexico.

***margaritena*** Botosaneanu in Botosaneanu and Viloria 2002: 106 [type locality: Venezuela, Isla de Margarita, Rio San Juan at fuentidueno; ZMUA; ♂; ♀]. —Botosaneanu 2002b: 85 [checklist].

**Distribution.** —Venezuela.

***maria*** Bueno-Soria & Hamilton, 1986: 302 [type locality: Mexico, Oaxaca, 7 km NE Huautla de Jimenez; NMNH; ♂]. —Keth 2004: 177 [♂]. —Keth et al. 2015: 31 [♂].

**Distribution.** —Mexico.

***mathisi*** Keth, 2004: 170 [type locality: Belize, Orange Walk District, New River Lagoon, dock area at Lamanai Ruins; NMNH; ♂].

**Distribution.** —Belize.

***matula*** Gama Neto & Passos, 2019: 525 [type locality: Brazil, Roraima, Mucajai municipality, RR-325, Vicinal Apiaú, Igarapé Serrinha (Sítio Vaca), 02°33'7.78"N, 61°18'45.06"W, 74 m a.s.l.; MPEG; ♂].

**Distribution.** —Brazil.

***maya*** Harris & Flint, 2016: 3 [type locality: Belize, Stann Creek District, Cockscomb Wildlife Preserve, Cockscomb A, B4, Maya Mountains, 200 m, N16-80, W88-55, subtropic wet forest; NMNH; ♂].

**Distribution.** —Belize.

***mcpheroni*** Keth in Keth et al. 2015: 86 [type locality: [United States], Texas; PSUC; ♂].

**Distribution.** —U.S.A.

***mentonensis*** Frazer & Harris, 1991b: 7 [type locality: [United States], Georgia, Chattooga County, Gilreath Creek at Co. Hwy. 234 bridge (34°34'N, 85°27'W); NMNH; ♂]. —Frazer et al. 1991: 20 [distribution]. —Harris and Rasmussen 2010: 40 [♂; ♀; distribution]. —Keth et al. 2015: 47 [♂].

**Distribution.** —U.S.A.

***michaeli*** Armitage & Harris, 2020a: 5 [type locality: Panama, Coclé Province, Cuenca 134, Omar Torrijos Herrera National Park, Quebrada Las Yayas, PSPSCB-PNGDOTH-C134-2017-004, 8.66168°N, 80.5952°W, 728 m; COZEM; ♂; see Armitage and Harris 2020b errata]. —Armitage and Harris 2020b: 3 [correction to original illustration].

**Distribution.** —Panama.

***minutisimella*** (Chambers, 1873): 125 [type locality not given; type specimen not designated; ♂; in *Cyllene*, Lepidoptera]. —Milne 1934: 76, 77 [to *Orthotrichia*]. —Ross 1944: 157 [♂; ♀; larva; distribution; designated ♀ allotype; indicated type locality may be [United States], Kentucky, Covington; to *Neotrichia*]. —Denning 1047: 20 [distribution]. —Unzicker et al. 1970: 172 [distribution]. —Resh et al. 1978: 383 [distribution]. —Blickle 1979: 51, 75 [checklist; ♂]. —Harris et al. 1982a: 511 [distribution]. —Waltz and McCafferty 1983a: 10 [distribution]. —Hamilton et al. 1983: 18 [distribution]. —Harris et al. 1984: 108 [distribution]. —Bowles and Mathis 1989: 239 [distribution]. —Frazer et al. 1991: 20 [distribution]. —Harris et al. 1991: 221 [distribution]. —Mathis and Bowles 1992: 24 [distribution]. —Bowles and Mathis 1992: 32 [distribution]. —Floyd et al. 1993: 91 [phenology; distribution]. —Moulton et al. 1993: 21 [distribution]. —Moulton and Stewart 1996: 113 [♂; distribution]. —Moulton and Stewart 1997: 350 [checklist]. —Houghton et al. 2001: 505 [distribution]. —Pescador et al. 2004: 133 [distribution]. —Bowles et al. 2007: 21 [distribution; biology]. —Harris et al. 2012: 8 [checklist]. —Keth et al. 2015: 105 [♂]. —Denson et al. 2016: 5 [distribution]. —Houghton et al. 2017: 63 [checklist]. —Barba-Álvarez et al. 2019: 85 [distribution].

**Distribution.** —Mexico, U.S.A.

***mobilensis*** Harris, 1985a: 252 [type locality: [United States], Alabama, Mobile County, Mobile River at Mt. Vernon, T2N, R1W, S42; NMNH; ♂]. —Harris et al. 1991: 222 [distribution]. —Abbott et al. 1997: 44 [distribution]. —Moulton and Stewart 1997: 350 [checklist]. —Harris and Rasmussen 2010: 26 [♂; ♀; distribution]. —Keth et al. 2015: 48 [♂]. —Harris and Flint 2016: 7 [distribution].

**Distribution.** —Mexico, U.S.A.

***mojavensis*** Keth in Keth et al. 2015: 49 [type locality: United States, Arizona, Mojave Co., Colorado River at Lake Navasu marina; NMNH; ♂].

**Distribution.** —U.S.A.

***mucajai*** Gama Neto & Passos, 2020: 184 [type locality: Brazil, Roraima, Iracema municipality, Vicinal Campos Novos (Fazenda Rancho Fundo), small order stream, 2°21'26.22"N, 61°23'38.98"W, 209 m a.s.l.; MPEG; ♂].

**Distribution.** —Brazil.

***napoensis*** Harris in Harris and Davenport 1992: 461 [type locality: Ecuador, Napo, 7 km N Lago Agrio; NMNH; ♂]. —Ríos-Touma et al. 2017: 10 [checklist].

**Distribution.** —Ecuador.

***negroensis*** Harris, 1990: 254 [type locality: Venezuela, Territorio Federal Amazonas, San Carlos de Río Negro; NMNH; ♂].

**Distribution.** —Venezuela.

***nesiotes*** Flint & Sykora, 1993: 55 [type locality: Grenada, Parish St. Andrews, Balthazar Estate; FSCA; ♂]. —Botosaneanu and Alkins-Koo 1993: 20 [♂; ♀; distribution]. —Flint 1996b: 100 [distribution]. —Botosaneanu 2002b: 85 [checklist]. —Keth et al. 2015: 50 [♂].

—*intortigona* Botosaneanu & Sakal, 1992: 202 [*nomen nudum*; distribution; ecology] —Flint et al. 1999a: 104 [to synonymy; as *nesiotes*].

**Distribution.** —Grenada, Trinidad.

***niltonsantosi*** Santos & Nessimian, 2009a: 762 [type locality: Brazil, Amazonas, Manaus (tributary to Igarapé da Cachoeira, 02°41'45.4"S 60°17'42.7"W); INPA; ♂]. —Paprocki and França 2014: 48 [checklist].

**Distribution.** —Brazil.

***noteuna*** (Mosely, 1939a): 232 [type locality: Brazil, Edo. Santa Catarina, Nova Teutonia; NHMUK; ♂; in *Exitrichia*]. —Angrisano 1995a: 511 [distribution]. —Angrisano 1999: 33 [checklist]. —Paprocki et al. 2004: 11 [checklist]. —Paprocki and França 2014: 48 [checklist].

**Distribution.** —Brazil, Uruguay.

***novara*** (Mosely, 1939a): 232 [type locality: Brazil, Edo. Santa Catarina, Nova Teutonia; NHMUK; ♂; in *Exitrichia*]. —Angrisano 1995a: 511 [distribution]. —Angrisano 1999: 33 [checklist]. —Paprocki et al. 2004: 11 [checklist]. —Angrisano and Sganga 2007: 40 [♂; distribution]. —Manzo et al. 2014: 166 [distribution]. —Paprocki and França 2014: 48 [checklist].

**Distribution.** —Argentina, Brazil, Uruguay.

***okopa*** Ross, 1939: 629 [type locality: [United States], Pennsylvania, Athens, along Susquehanna R.; INHS; ♂; ♀]. —Ross 1944: 158 [♂; ♀; distribution]. —Etnier 1965: 147 [distribution]. —Edwards 1973: 506 [distribution]. —Roy and Harper 1975: 1083 [distribution]. —Roy and Harper 1979: 152 [distribution]. —Blickle 1979: 51, 75 [checklist; ♂]. —Roy and Harper 1981: 105 [distribution]. —Huryn and Foote 1983: 791 [distribution; as *okapa*]. —Hamilton et al. 1983: 18 [distribution]. —Frazer et al. 1991: 20 [distribution]. —Harris et al. 1991: 223 [distribution]. —Masteller and Flint 1992: 70 [distribution]. —Bowles and Mathis 1992: 32 [distribution]. —Moulton et al. 1993: 21 [distribution]. —Moulton and Stewart 1996: 114 [♂; distribution]. —Moulton and Stewart 1997: 350 [checklist]. —Houghton et al. 2001: 505 [distribution]. —Keth 2003: 169 [♂]. —Pescador et al. 2004: 133 [distribution]. —Bowles et al. 2007: 21 [distribution; biology]. —Blinn and Ruiter 2009b: 186 [phenology; distribution]. —Armitage et al. 2011: 14 [checklist]. —Keth et al. 2015: 87 [♂]. —Houghton et al. 2017: 63 [checklist]. —Mendez et al. 2019: 118 [checklist]. —Bowles et al. 2020: 8 [distribution].

**Distribution.** —Canada, U.S.A.

***oldalia*** Oláh & Johanson, 2011: 192 [type locality: Peru, San Martin Prov., Rio Huallaga, at Pumarihri Huallaga Lodge, between Juan Guerra and Chazuta, 14 km(rd.) W Chazuta, 6°36.643'S, 76°12.555'W; NHRS; ♂].

**Distribution.** —Peru.

***olorina*** (Mosely, 1937b): 175 [type locality: Mexico, Guerrero, Cocula; NHMUK; ♂; in *Exitrichia*]. —Bueno-Soria and Flint 1978: 203 [distribution]. —Blinn and Ruiter 2006: 333 [biology; distribution]. —Blinn and Ruiter 2009a: 305 [biology]. —Keth et al. 2015: 73 [♂].

**Distribution.** —Mexico, U.S.A.

***orejona*** Harris & Davenport, 1992: 26 [type locality: Peru, Loreto, edge of Rio Sucusari backwater, adjoining Explornapo Camp; NMNH; ♂].

**Distribution.** —Peru.

***orlandoi*** Santos & Nessimian, 2009a: 761 [type locality: Brazil, Amazonas, Manaus (tributary to Rio Branquinho, 02°31'24.6"S 60°20'05.3"W); INPA; ♂]. —Paprocki and França 2014: 48 [checklist].

**Distribution.** —Brazil.

***osmena*** Ross, 1944: 278 [type locality: [United States], Utah, Blacksmith Fork Canyon; INHS; ♂]. —Blickle 1979: 51, 75 [checklist; ♂]. —Moulton and Stewart 1997: 350 [checklist]. —Houghton 2001: 90 [distribution]. —Blinn and Ruiter 2005: 69 [distribution; biology]. —Bowles et al. 2007: 21 [distribution; biology]. —Keth et al. 2015: 88 [♂].

—*panneus* Denning, 1947a: 154 [type locality: [United States], Wyoming, Bluegrass River, near Wheatland; ESUW; ♂]. —Blickle 1979: 51 [to synonymy].

**Distribution.** —U.S.A.

***ovona*** (Mosely, 1939a): 233 [type locality: Brazil, Edo. Santa Catarina, Nova Teutonia; NHMUK; ♂; in *Exitrichia*]. —Angrisano 1999: 33 [checklist]. —Paprocki et al. 2004: 11 [checklist]. —Paprocki and França 2014: 48 [checklist].

**Distribution.** —Brazil.

***oxima*** (Mosely, 1937b): 176 [type locality: Mexico, Guerrero, Cocula; NHMUK; ♂; in *Exitrichia*]. —Bueno-Soria and Flint 1978: 203 [distribution]. —Keth et al. 2015: 89 [♂].

**Distribution.** —Mexico.

***palitla*** Harris & Flint, 2016: 4 [type locality: Mexico, San Luis Potosi, Palitla; NMNH; ♂].

**Distribution.** —Mexico.

***palma*** Flint, 1982a: 45 [type locality: Argentina, Pcia. Buenos Aires, Río Parana de las Palmas; NMNH; ♂; ♀]. —Flint 1982b: 38 [distribution]. —Angrisano 1995a: 511 [distribution]. —Angrisano 1999: 33 [checklist]. —Angrisano and Sganga 2007: 40 [♂; distribution]. —Moreno et al. 2020: 265 [distribution].

**Distribution.** —Argentina, Brazil, Paraguay, Uruguay.

***pamelae*** Harris & Armitage, 2015: 5 [type locality: Panama, Chiriquí Province, Cuenca 108, tributary of Quebrada Grande, at waterfall, Boquete, Valle Escondido, 8.78291°N, 82.44579°W, 1253 m; MIUP; ♂]. —Armitage et al. 2015a: 7 [checklist]. —Armitage and Harris 2018b: 98 [checklist]. —Armitage and Harris 2018c: 283 [distribution].

**Distribution.** —Panama.

***parabullata*** Harris & Armitage, 2015: 6 [type locality: Panama, Panama Canal Zone, Cuenca 115, Isla Abogada, 9.19903°N, 79.85980°W; NMNH; ♂]. —Armitage et al. 2015a: 7 [checklist]. —Armitage and Harris 2018b: 98 [checklist]. —Gama Neto and Passos 2019: 527 [distribution].

**Distribution.** —Brazil, Panama.

***parany*** Oláh & Johanson, 2011: 193 [type locality: Peru, Amazonas Prov., Rio Utcabamba, Bajra Grande, at Rio Hotel, 5°45.824'S, 78°25.414'W; NHRS; ♂].

**Distribution.** —Peru.

***paraokopa*** Keth in Keth et al. 2015: 90 [type locality: U.S.A., Missouri, Christian Co., Swan Creek at Missouri Rt. DD, approximately 0.8 kilometers; PSUC; ♂].

**Distribution.** —U.S.A (Missouri).

***pelei*** Santos & Nessimian, 2009a: 766 [type locality: Brazil, Amazonas, Rio Preta da Eva (tributary to Rio Preto da Eva, 02°32'09.4"S 59°49'59.3"W); INPA; ♂]. —Paprocki and França 2014: 48 [checklist].

**Distribution.** —Brazil.

***pequenita*** Botosaneanu, 1977: 277 [type locality: Cuba, Oriente, Baracoa, Rio Sabanilla; NMNH; ♂]. —Botosaneanu 1979: 51 [distribution]. —Kumanski 1987: 23 [distribution]. —Botosaneanu 1990b: 46 [♂; ♀; distribution]. —Botosaneanu 1991: 128 [distribution]. —Botosaneanu and Sakal 1992: 202 [distribution; ecology]. —Botosaneanu and Alkins-Koo 1993: 18 [distribution]. —Flint and Sykora 1993: 49 [checklist]. —Botosaneanu 1994b: 458 [larva]. —Flint 1996a: 16 [checklist]. —Flint 1996b: 101 [distribution]. —Botosaneanu and Hyslop 1998: 18 [distribution]. —Flint and Pérez-Gelabert 1999: 40 [checklist]. —Botosaneanu 2002b: 85 [checklist]. —Flint and Sykora 2004: 36 [distribution]. —Naranjo López and González Lazo 2005: 149 [checklist]. —Pérez-Gelabert 2008: 300 [checklist]. —Keth et al. 2015: 51 [♂].

—*Neotrichia* sp. 1: Kumanski 1987: 23 [♀]. —Flint and Sykora 2004: 36 [to synonymy].

**Distribution.** —Barbados, Dominican Republic, Cuba, Haiti, Jamaica, Trinidad.

***picada*** Flint, 1983: 53 [type locality: Uruguay, Dpto. Lavalleja, Rio Cebollati, Picada de Rodriguez; NMNH; ♂]. —Angrisano 1995a: 511 [distribution]. —Angrisano 1999: 33 [checklist].

**Distribution.** —Uruguay.

***pierpointorum*** Armitage & Harris, 2020a: 6 [type locality: Panama, Coclé Province, Cuenca 134, Omar Torrijos Herrera National Park, Quebrada Las Yayas, PSPSCB-PNGDOTH-C134-2017-004, 8.66168°N, 80.5952°W, 728 m; COZEM; ♂].

**Distribution.** —Panama.

***pinarenia*** Botosaneanu, 1980: 113 [type locality: Cuba, Prov. Pinar del Rio, Arroyo del Pinar de Viñales; ZMUA; ♂]. —Botosaneanu 1979: 51 [distribution]. —Flint 1996a: 16 [checklist]. —Botosaneanu 2002b: 86 [checklist]. —Naranjo López and González Lazo 2005: 149 [checklist]. —Keth et al. 2015: 32 [♂]

**Distribution.** —Cuba.

***pinnacles*** Harris & Flint, 2016: 4 [type locality: United States, California, San Benito County, Pinnacles National Monument, Chalone Creek, 1.4 km NW Bear Creek; NMNH; ♂]. —Mendez et al. 2019: 118 [checklist].

**Distribution.** —U.S.A.

***proboscidea*** Flint, 1974b: 73 [type locality: Suriname, Nickerie River, Lombok falls; RMNH; ♂].

**Distribution.** —Suriname.

***pulgara*** Keth, 2004: 174 [type locality: Belize, Orange Walk District, New River Lagoon, dock area at Lamanai Ruins; NMNH; ♂].

**Distribution.** —Belize.

***quitauau*** Gama Neto & Passos, 2020: 185 [type locality: Brazil, Roraima, Iracema municipality, Vicinal Campos Novos (Fazenda Rancho Fundo), small order stream, 2°21'26.22"N, 61°23'38.98"W, 209 m a.s.l.; MPEG; ♂].

**Distribution.** —Brazil.

***rambala*** Harris & Armitage, 2019: 13 [type locality: Panama, Bocas del Toro Province, Quebrada Rambala, near Rambala Jungle Lodge, 3.74 km SSE Rambala, 8.91543°N and 82.15527°W; COZEM; ♂].

**Distribution.** —Panama.

***rasmusseni*** Harris & Keth, 2002: 73 [type locality: [United States], Florida, Suwannee County, Santa Fe River at Hwy. 129; NMNH; ♂]. —Pescador et al. 2004: 133 [checklist]. —Harris and Rasmussen 2010: 32 [♂; ♀; distribution]. —Harris et al. 2012: 8 [checklist]. —Keth et al. 2015: 52 [♂].

**Distribution.** —U.S.A.

***riegeli*** Ross, 1941a: 61 [type locality: [United States], Illinois, Eddyville, Lusk Creek; INHS; ♂; ♀]. —Ross 1944: 159 [♂; ♀; distribution]. —Blickle 1979: 51, 77 [checklist; ♂]. —Moulton and Stewart 1996: 114 [♂; distribution]. —Etnier 2010: 486 [distribution]. —Harris and Rasmussen 2010: 29 [♂; ♀; distribution]. —Keth et al. 2015: 53 [♂].

**Distribution.** —U.S.A.

***riparia*** Flint & Reyes, 1991: 486 [type locality: Peru, Dept. La Libertad, Prov. Trujillo, Dist. Simbal, Río Lucumar, Simbal; NMNH; ♂].

**Distribution.** —Peru.

***rotundata*** Flint, 1974b: 76 [type locality: Suriname, Käyser Airstrip; RMNH; ♂]. —Flint 1991a: 69 [distribution; as near rotundata]. —Angrisano 1999: 33 [checklist]. —Paprocki et al. 2004: 11 [checklist]. —Paprocki and França 2014: 48 [checklist].

**Distribution.** —Brazil, Suriname.

***ruiteri*** Keth in Keth et al. 2015: 54 [type locality: Mexico, Sonora, Canon Alacran; NMNH; ♂].

**Distribution.** —Mexico.

***sala*** Angrisano, 1984: 1 [type locality: Argentina, SALTA, Parque Nacional el Rey, río La Sala; MACN; ♂]. —Angrisano 1999: 33 [checklist].

**Distribution.** —Argentina.

***salada*** Flint, 1982a: 43 [type locality: Argentina, Pcia. Buenos Aires, Rio Salado, Rt. 3, S San Miguel del Monte; NMNH; ♂; ♀]. —Flint 1982b: 39 [distribution]. —Angrisano 1995a: 513 [distribution]. —Angrisano 1999: 33 [checklist].

**Distribution.** —Argentina, Paraguay, Uruguay.

***sandersoni*** Harris & Flint, 2016: 5 [type locality: United States, Arizona, Coconino County, West Fork Oak Creek, A79-17; NMNH; ♂].

**Distribution.** —U.S.A.

***sandyae*** Ruiter, 2007: 275 [type locality: USA, Arizona, Cochise County, South Fork Cave Creek; NMNH; ♂]. —Keth et al. 2015: 33 [♂].

**Distribution.** —U.S.A.

***sepulga*** Harris, 1991: 15 [type locality: [United States], Alabama, Butler County, Duck Creek off Co. Hwy. 7, 3.2 km W Mt. Olive (Sec. 17, T 7 N, R 12 E); NMNH; ♂]. —Harris et al. 1991: 224 [distribution]. —Keth et al. 2015: 99 [♂].

**Distribution.** —U.S.A.

***serrata*** Harris & Armitage, 2019: 14 [type locality: Panama, Bocas del Toro Province, Quebrada Rambala, near Rambala Jungle Lodge, 3.74 km SSE Rambala, 8.91543°N and 82.15527°W, 120 m; COZEM; ♂].

**Distribution.** —Panama.

***sicilicula*** Flint, 1983: 51 [type locality: Brazil, Edo. Santa Catarina, Nova Teutonia; NMNH; ♂]. —Angrisano 1995a: 513 [distribution]. —Angrisano 1999: 33 [checklist]. —Paprocki et al. 2004: 11 [checklist]. —Paprocki and França 2014: 49 [checklist].

**Distribution.** —Brazil, Uruguay.

***sokaga*** Oláh & Johanson, 2011: 194 [type locality: Peru, San Martin Prov., creek crossing rd. Juan Guerra-Chazuta, 14 km (rd.) E Colombia Bridge, 6°35.594'S, 76°13.172'W; NHRS; ♂].

**Distribution.** —Peru.

***soleaferrea*** Botosaneanu in Botosaneanu and Hyslop 1998: 18 [type locality: Jamaica, St. Elizabeth, Black River in its upper course at Windsor; ZMUA; ♂; ♀]. —Botosaneanu 2002b: 86 [checklist]. —Keth et al. 2015: 100 [♂].

**Distribution.** —Jamaica.

***sonora*** Ross, 1944: 277 [type locality: [United States], Texas, Neville Spring at foot of Chisos Mountains; INHS; ♂]. —Edwards 1973: 506 [distribution]. —Blickle 1979: 51, 77 [checklist; ♂]. —Moulton and Stewart 1997: 350 [checklist]. —Blinn and Ruiter 2006: 333 [biology; distribution]. —Keth et al. 2015: 34 [♂].

**Distribution.** —U.S.A.

***starki*** Harris & Armitage, 2019: 14 [type locality: Panama, Bocas del Toro Province, Quebrada Rambala, near Rambala Jungle Lodge, 3.74 km SSE Rambala, 8.91543°N and 82.15527°W, 120 m; COZEM; ♂].

**Distribution.** —Panama.

***staufferi*** Keth in Keth et al. 2015: 91 [type locality: U.S.A., Illinois, White Pines State Park; INHS; ♂].

**Distribution.** —U.S.A (Illinois).

***sucusaria*** Harris & Davenport, 1992: 458 [type locality: Peru, Loreto, Rio Sucusari just up stream from Explornapo Camp; NMNH; ♂].

**Distribution.** —Peru.

***tatianae*** Armitage & Harris, 2018a: 7 [type locality: Panama, Chiriquí Province, Cuenca 108, tributary of Quebrada Grande at the waterfall, Valle Escondido, Boquete, 8.78291°N, 82.44579°W, 1253 m; COZEM; ♂]. —Armitage and Harris 2018b: 98 [checklist]. —Armitage and Harris 2018c: 283 [distribution]. —Harris and Armitage 2019: 5 [distribution].

**Distribution.** —Panama.

***tauricornis*** Malicky, 1980b: 220 [type locality: Guadeloupe, Bras de David beim Forsthaus, Zufluss des Flusses Goyaves; MPC; ♂]. —Malicky 1983c: 264 [checklist]. —Botosaneanu 1989: 99 [♀; distribution]. —Flint 1991b: 53 [♂; distribution]. —Botosaneanu and Sakal 1992: 202 [distribution; ecology]. —Flint and Sykora 1993: 56 [distribution]. —Harris and Tiemann 1993: 288 [♂; distribution]. —Botosaneanu and Alkins-Koo 1993: 21 [distribution]. —Botosaneanu 1994a: 43 [distribution]. —Flint 1996b: 101 [distribution]. —Muñoz-Quesada 2000: 278 [checklist]. —Botosaneanu 2000: 256 [distribution]. —Botosaneanu 2002b: 86 [checklist]. —Botosaneanu and Thomas 2005: 44 [distribution]. —Armitage et al. 2015a: 7 [checklist]. —Keth et al. 2015: 35 [♂]. —Armitage and Harris 2018b: 98 [checklist]. —Gama Neto and Passos 2019: 527 [distribution].

**Distribution.** —Brazil, Colombia, Grenada, Guadeloupe, Martinique, Panama, St. Lucia, Tobago, Trinidad.

***tertia*** (Mosely, 1939a): 235 [type locality: Brazil, Edo. Santa Catarina, Nova Teutonia; NHMUK; in *Exitrichia*; ♂]. —Angrisano 1995a: 513 [distribution]. —Angrisano 1999: 33 [checklist]. —Paprocki et al. 2004: 11 [checklist]. —Paprocki and França 2014: 49 [checklist].

**Distribution.** —Brazil, Uruguay.

***teutonia*** Flint, 1983: 49 [type locality: Brazil, Edo. Santa Catarina, Nova Teutonia; NMNH; ♂]. —Angrisano 1999: 33 [checklist]. —Paprocki et al. 2004: 11 [checklist]. —Paprocki and França 2014: 49 [checklist].

**Distribution.** —Brazil.

***tirabuzona*** Harris & Davenport, 1992: 27 [type locality: Peru, Loreto, edge of Rio Sucusari backwater, adjoining Explornapo Camp; NMNH; ♂].

**Distribution.** —Peru.

***tompa*** Oláh & Johanson, 2011: 197 [type locality: French Guiana, Approuaguekaw, Kaw Mt, 4°33.035'N, 52°11.661'W, 104 m; NHRS; ♂].

**Distribution.** —French Guiana.

***tubulifera*** Flint, 1980b: 141 [type locality: Argentina, Pcia. Entre Rios, Salto Grande, Rio Uruguay; NMNH; ♂]. —Flint 1982b: 40 [distribution]. —Angrisano 1995a: 513 [distribution]. —Angrisano 1999: 33 [checklist]. —Angrisano and Sganga 2007: 40 [♂; distribution].

**Distribution.** —Argentina, Uruguay.

***tuxtla*** Bueno-Soria, 1999: 113 [type locality: Mexico, Veracruz, Estación de Biología Los Tuxtlas, UNAM, Arroyo del Zoológico; CNIN; ♂]. —Keth et al. 2015: 69 [♂]. —Armitage et al. 2016: 9 [distribution]. —Razo-González 2018: 32 [distribution]. —Armitage and Harris 2018b: 98 [checklist]. —Armitage and Harris 2018c: 283 [distribution].

**Distribution.** —Mexico, Panama.

***unamas*** Botosaneanu in Botosaneanu and Alkins-Koo 1993: 22 [type locality: Tobago, Argyll River below Argyll waterfall; ZMUA; ♂]. —Botosaneanu and Sakal 1992: 202 [distribution; ecology]. —Flint 1996b: 101 [distribution]. —Botosaneanu 2002b: 86 [checklist]. —Keth et al. 2015: 36 [♂]. —Armitage et al. 2016: 9 [distribution]. —Armitage and Harris 2018b: 98 [checklist]. —Armitage and Harris 2018c: 283 [distribution]. —Harris and Armitage 2019: 5 [distribution].

—species A Botosaneanu and Alkins-Koo 1993: 23 [♀]. —Flint 1996b: 101 [distribution; to synonymy].

**Distribution.** —Panama, Tobago, Trinidad.

***unispina*** Flint, 1974b: 72 [type locality: Suriname, Lucie River camp, Wilhelmina Mountains; RMNH; ♂]. —Flint 1996c: 402 [distribution].

**Distribution.** —Peru, Suriname.

***vavai*** Santos & Nessimian, 2009a: 763 [type locality: Brazil, Amazonas, Manaus (tributary to Rio Branquinho, 02°31'24.6"S 60°20'05.3"W); INPA; ♂]. —Paprocki and França 2014: 49 [checklist].

**Distribution.** —Brazil.

***vekonyka*** Oláh & Johanson, 2011: 197 [type locality: French Guiana, Approuaguekaw, Kaw Mt, 4°33.035'N, 52°11.661'W, 104 m; NHRS; ♂].

**Distribution.** —French Guiana.

***vibrans*** Ross, 1938a: 119 [type locality: [United States], Illinois, Oakwood, along Middle Fork River; INHS; ♂]. —Morse and Blickle 1953: 73 [checklist]. —Etnier 1965: 147 [distribution]. —Unzicker et al. 1970: 172 [distribution; as *Noetrichiavibrans*]. —Bueno-Soria and Flint 1978: 203 [distribution]. —Etnier and Schuster 1979: 17 [distribution]. —Blickle 1979: 51, 77 [checklist; ♂]. —Parker and Voshell 1981: 4 [checklist]. —Harris et al. 1982a: 511 [distribution]. —Huryn and Foote 1983: 791 [distribution]. —Hamilton et al. 1983: 18 [distribution]. —Harris et al. 1984: 109 [distribution]. —Usis and Foote 1989: 84 [distribution]. —Bowles and Mathis 1989: 239 [distribution]. —Tarter 1990: 239 [checklist]. —Harris et al. 1991: 225 [distribution]. —Frazer et al. 1991: 20 [distribution]. —Mathis and Bowles 1992: 24 [distribution]. —Bowles and Mathis 1992: 32 [distribution]. —Moulton and Stewart 1996: 115 [♂; distribution]. —Abbott et al. 1997: 44 [distribution]. —Moulton and Stewart 1997: 350 [checklist]. —Houghton and Stewart 1998: 105 [distribution]. —Huryn and Harris 2000: 193 [distribution]. —Houghton et al. 2001: 505 [distribution]. —Pescador et al. 2004: 133 [checklist]. —Bowles et al. 2007: 21 [distribution; biology]. —Flint et al. 2009:7 [distribution]. —Etnier 2010: 486 [distribution]. —Houghton et al. 2011b: 5 [biology]. —Armitage et al. 2011: 14 [checklist]. —Myers et al. 2011: 108 [distribution]. —Harris et al. 2012: 8 [checklist]. —Wright et al. 2013: 466 [biology; distribution]. —Keth et al. 2015: 101 [♂]. —Denson et al. 2016: 5 [distribution]. —Houghton et al. 2017: 63 [checklist]. —Bowles et al. 2020: 8 [distribution].

—*ranea* Denning, 1947a: 19 [type locality: United States, Florida, Miami; ESUW; ♂]. —Ross 1948: 205 [to synonymy].

**Distribution.** —Mexico, U.S.A.

***villa*** Oláh & Johanson, 2011: 199 [type locality: French Guiana, Approuaguekaw, Kaw Mt, 4°33.035'N, 52°11.661'W, 104 m; NHRS; ♂].

**Distribution.** —French Guiana.

***vissa*** Oláh & Johanson, 2011: 200 [type locality: Peru, San Martin Prov., La Catarata de Ahuashiyascu, 6°27.544'S, 76°18.192'W; NHRS; ♂].

**Distribution.** —Peru.

***vonza*** Oláh & Johanson, 2011: 201 [type locality: French Guiana, Maripasoula, Maroni River, Damason campo, Village, 4°35.112'N, 54°24.799'W, 38 m; NHRS; ♂].

**Distribution.** —French Guiana.

***xereuini*** Gama Neto & Passos, 2020: 186 [type locality: Brazil, Roraima, Iracema municipality, Vicinal Campos Novos (Fazenda Rancho Fundo), small order stream, 2°21'26.22"N, 61°23'38.98"W, 209 m a.s.l.; MPEG; ♂].

**Distribution.** —Brazil.

***xicana*** (Mosely, 1937b): 178 [type locality: Mexico, Chiapas, Dolores; NHMUK; ♂; in *Dolotrichia*]. —Ross 1944: 154 [to synonymy]. —Bueno-Soria and Flint 1978: 203 [distribution]. —Maes 1999: 1194 [checklist]. —Bueno-Soria et al. 2005: 75 [distribution]. —Bueno-Soria et al. 2007: 33 [distribution]. —Chamorro-Lacayo et al. 2007: 43 [checklist]. —Oláh and Johanson 2011: 202 [distribution]. —Keth et al. 2015: 37 [♂]. —Armitage et al. 2016: 10 [distribution]. —Armitage and Harris 2018b: 98 [checklist]. —Armitage and Harris 2018c: 283 [distribution].

**Distribution.** —Mexico, Nicaragua, Panama.

***yagua*** Harris & Davenport, 1992: 463 [type locality: Peru, Loreto, Rio Sucusari just upstream from Explornapo Camp; NMNH; ♂].

**Distribution.** —Peru.

***yanomonoa*** Harris & Davenport, 1992: 454 [type locality: Peru, Loreto, small tributary to Rio Yanomono at Explorama Lodge; NMNH; ♂].

**Distribution.** —Peru.

***yavesia*** Bueno-Soria, 2010: 29 [type locality: Mexico, Oaxaca, Santa María de Yavesia, 17°14'04.76"N 96°25'35.06"W, 2058 m; CNIN; ♂]. —Keth et al. 2015: 102 [♂].

**Distribution.** —Mexico.

***yayas*** Armitage & Harris, 2020a: 7 [type locality: Panama, Coclé Province, Cuenca 134, Omar Torrijos Herrera National Park, Quebrada Las Yayas, PSPSCB-PNGDOTH-C134-2017-004, 8.66168°N, 80.5952°W, 728 m; COZEM; ♂].

**Distribution.** —Panama.

***zagalloi*** Santos & Nessimian, 2009a: 765 [type locality: Brazil, Amazonas, Rio Preta da Eva (Igarapé Jangada, tributary to Rio Urubu, 02°26'32.5"S 59°32'46.2"W); INPA; ♂]. —Paprocki and França 2014: 49 [checklist].

**Distribution.** —Brazil.

***zitoi*** Santos & Nessimian, 2009a: 762 [type locality: Brazil, Amazonas, Manaus (Igarapé Arumã, tributary to Rio Cuieiras, 02°30'55.2"S 60°15'44.4"W); INPA; ♂]. —Paprocki and França 2014: 49 [checklist].

**Distribution.** —Brazil.

#### Genus *Taraxitrichia* Flint & Harris, 1991

*Taraxitrichia* Flint & Harris, 1991: 441 [type species: *Taraxitrichiaamazonensis* Flint & Harris, 1991, original designation]. —Pes and Hamada 2003: 2 [larva; case; biology; distribution].

*Taraxitrichia* is a monotypic genus recorded from Brazil and the territory of Amazonas in Venezuela. When trying to identify the original specimens using Marshalls’ (1979b) hydroptilid key, Flint and Harris (1991) found that they ended at a couplet that resulted in either *Hydroptila* or *Orthotrichia*, but that the specimens did not fit into either option and instead warranted a new genus. Character states important in diagnosis of the genus include the absence of ocelli, the spur formula (0, 3, 4), and male genitalia sharing similarities with those of *Mayatrichia* (Flint and Harris 1991). The larvae, described by Pes and Hamada (2003), live in freshwater sponges.

***amazonensis*** Flint & Harris, 1991: 411 [type locality: Venezuela, Territorio Federal Amazonas, Rio Cataniapo, 10 km S Puerto Ayacucho; NMNH; ♂; ♀].

**Distribution.** —Brazil, Venezuela.

### ﻿Subfamily OCHROTRICHIINAE Marshall, 1979

Ochrotrichiini Marshall, 1979b: 184 [type genus: *Ochrotrichia* Mosely, 1934a]. —Oláh and Johanson 2011: 203 [generic character states].

Ochrotrichiinae is a subfamily containing nine genera with interestingly disparate distributions: seven genera (*Angrisanoia*, *Metrichia*, *Nothotrichia*, *Ochrotrichia*, *Ragatrichia*, and *Rhyacopsyche*) are New World genera found throughout North and Central America, while *Maydenoptila* is found only in Australia and *Caledonotrichia* is endemic to New Caledonia. Only recently the latter two genera were placed within Ochrotrichiinae, after historically being considered incertae sedis within Hydroptilidae, following a character state assessment presented with no statistical analysis (Oláh and Johanson 2011). Marshall (1979b) commented that the group may some day be considered a subgroup of Hydroptilinae, but outlined certain morphological and larval behavioral traits, such as the complex genitalia and the larval detritus-feeding habits, that she considered make it distinct. However, not all members of the subfamily are detritus feeders (Wells 1985b). Larval descriptions have been provided for several genera.

#### Genus *Angrisanoia* Ozdikmen, 2008

*Angrisanoia* Özdikmen, 2008: 615 [replacement name, *Paratrichia*, preoccupied, junior homonym of a Diptera genus within Scenopinidae (Kelsey 1969: 320)]. —Oláh and Johanson 2011: 235 [re-description].

*Paratrichia* Angrisano, 1995a: 507 [type species: Ochrotrichia (Paratrichia) cebollati, original description; monotypy; as subgenus of *Ochrotrichia*]. —Flint et al. 1999a: 106 [as subgenus of *Ochrotrichia*]. —Angrisano 2002: 405 [to genus status]. —Özdikmen 2008: 615 [preoccupied].

The genus *Agrisanoia* currently contains five species recorded from Argentina, French Guiana, Venezuela, and Uruguay. Angrisano (1995a) established the genus under the name *Paratrichia*, as a monotypic subgenus of *Ochrotrichia*, but neglected to explicitly state which morphological features were used to define it. Angrisano (2002) later elevated it to generic status, due to the elevation of the other *Ochrotrichia* subgenera, *Metrichia* and *Ochrotrichia*, by Flint and Bueno (1998). The larvae are unknown.

***acuti*** (Angrisano & Sganga, 2009): 62 [type locality: Argentina, Misiones, Parque Provincial Salto Encantado, Salto Acutí; MACN; ♂; in *Paratrichia*]. —Oláh and Johanson 2011: 236 [to *Angrisanoia*]. —Manzo et al. 2014: 166 [distribution].

**Distribution.** —Argentina.

***agazoka*** Oláh & Johanson, 2011: 236 [type locality: French Guiana, Approuaguekaw, Kaw Mt, 4°32.833'N, 52°11.452'W, 77 m; NHRS; ♂].

**Distribution.** —French Guiana.

***cebollati*** (Angrisano, 1995a): 509 [type locality: Uruguay, Lavalleja, río Cebollatí, Picada de Rodriguez; FHCU; ♂l in Ochrotrichia (Paratrichia)]. —Angrisano 1999: 34 [checklist]. —Angrisano 2002: 405 [to *Paratrichia*]. —Özdikmen 2008: 615 [to *Angrisanoia*]. —Oláh and Johanson 2011: 238 [checklist].

**Distribution.** —Uruguay.

***lemeza*** Oláh & Johanson, 2011: 238 [type locality: French Guiana, Approuaguekaw, Kaw Mt, 4°33.035'N, 52°11.661'W, 104 m; NHRS; ♂].

**Distribution.** —French Guiana.

***otarosa*** (Wasmund & Holzenthal, 2007): 17 [type locality: Venezuela, T. F. A. [Amazonas], Camp IV, Cerro d. l. Neblina, 0°58'N 65°57'W, 670 m; NMNH; ♂; in *Rhyacopsyche*]. —Oláh and Johanson 2011: 239 [to *Angrisanoia*].

**Distribution.** —Venezuela.

#### Genus *Caledonotrichia* Sykora, 1967

*Caledonotrichia* Sykora, 1967: 585 [type species: *Caledonotrichiailliesi* Sykora, 1967, original designation]. —Marshall 1979b: 221 [generic review; considered incertae sedis within Hydroptilidae]. —Wells 1995: 229 [larva]. —Harris and Armitage 1997: 127 [placement]. —Holzenthal et al. 2007b: 671 [placement]. —Oláh and Johanson 2011: 203 [diagnosis; placement]. —Wells et al. 2013: 59 [generic review; key to adult males].

*Caledonotrichia* consists of eleven species occurring in New Caledonia. The male genitalia of this genus are noted as both very distinctive within Hydroptilidae and very difficult to homologize with those of other hydroptilid genera (Sykora 1967; Marshall 1979b). Marshall (1979b) stated that neither the adult nor the larval stage offer any clues regarding placement of the group within Hydroptilidae and left the genus incertae sedis. Based on specimen comparison between genera, Harris and Armitage (1997) placed *Caledonotrichia* in Ochrotrichiinae, while also stating that uniting the group was difficult and that they were still determining synapomorphies. The genus was listed as incertae sedis by Holzenthal et al. (2007b), following a mistake in the Trichoptera World Checklist (Morse 2006). Oláh and Johanson (2011) indicated, in agreement with the conclusions of Harris & Armitage (1997) but offering no further explanation or discussion, that *Caledonotrichia* may belong in Ochrotrichiinae. Larval illustrations of *C.minor*, *C.illiesi*, and *C.* sp. are all available in Wells (1995).

***bifida*** Wells, Johanson, & Mary-Sasal, 2013: 71 [type locality: New Caledonia, Province Sud, Serraméa, forest stream, loc 10 21°37.883'S, 165°51.958'E; MNHN; ♂]. —Johanson and Wells 2019: 92 [checklist].

**Distribution.** —New Caledonia.

***capensis*** Wells, Johanson, & Mary-Sasal, 2013: 77 [type locality: New Caledonia, Rivière du Cap, Pont du Cap, ~8 km NW Naindai on Bourail-Poya road; MNHN; ♂]. —Johanson and Wells 2019: 92 [checklist].

**Distribution.** —New Caledonia.

***charadra*** Kelley, 1989: 194 [type locality: New Caledonia, mountain stream up Boulari River; BPBM; ♂]. —Wells et al. 2013: 78 [♂]. —Johanson and Wells 2019: 92 [checklist].

**Distribution.** —New Caledonia.

***extensa*** Kelley, 1989: 195 [type locality: New Caledonia, mountain stream up Boulari River; BPBM; ♂]. —Wells et al. 2013: 79 [♂]. —Johanson and Wells 2019: 92 [checklist].

**Distribution.** —New Caledonia.

***illiesi*** Sykora, 1967: 586 [type locality: New Caledonia, River near Col d’Amieu, 478 m; BPBM; ♂]. —Neboiss 1986: 70 [atlas; ♂]. —Wells 1995: 229 [larva; pupa; distribution]. —Oláh and Johanson 2010a: 37 [distribution]. —Wells et al. 2013: 64 [♂]. —Johanson and Wells 2019: 92 [checklist].

**Distribution.** —New Caledonia.

***minor*** Sykora, 1967: 587 [type locality: New Caledonia, River near Col d’Amieu, 478 m; BPBM; ♂]. —Neboiss 1986: 70 [atlas; ♂]. —Wells 1995: 230 [adult head and thorax; distribution]. —Wells et al. 2013: 74 [♂]. —Johanson and Wells 2019: 92 [checklist].

**Distribution.** —New Caledonia.

***minuta*** Wells, Johanson, & Mary-Sasal, 2013: 66 [type locality: New Caledonia, approx. 10 km SW of Houailou on Houailou-Bourail road, small fall; MNHN; ♂]. —Johanson and Wells 2019: 93 [checklist].

**Distribution.** —New Caledonia.

***nyurga*** Oláh & Johanson, 2010a: 37 [type locality: New Caledonia, Province Sud, W slope Mt. Ningua, Kwe Néco Stream, at Camp Jacob, 3.7 km WNW summit of Mt. Ningua, on Bouloparis-Thio Road, about 50 m upstream road, 21°43.613'S, 166°06.567'E, 150 m; MNHN; ♂]. —Wells et al. 2013: 81 [♂]. —Johanson and Wells 2019: 93 [checklist].

**Distribution.** —New Caledonia.

***ouinnica*** Wells, Johanson, & Mary-Sasal, 2013: 72 [type locality: New Caledonia, Province Sud, Mt. Dzumac, source stream of Ouinne River, near crosspoint to mountain track, 22°02.073'S, 166°28.460'E; MNHN; ♂]. —Johanson and Wells 2019: 93 [checklist].

**Distribution.** —New Caledonia.

***sykorai*** Wells, Johanson, & Mary-Sasal, 2013: 85 [type locality: New Caledonia, Province Sud, stream crossing way to sanatorium 2.3 km E St Laurent, ca. 150 m upstream bridge, 22°04.484'S, 166°19.910'E; MNHN; ♂]. —Johanson and Wells 2019: 93 [checklist].

**Distribution.** —New Caledonia.

***vexilla*** Wells, Johanson, & Mary-Sasal, 2013: 76 [type locality: New Caledonia, Parc de Rivière Bleu, approx 1 km W Kaori Giant; MNHN; ♂]. —Johanson and Wells 2019: 93 [checklist].

**Distribution.** —New Caledonia.

#### Genus *Dibusa* Ross, 1939

*Dibusa* Ross, 1939: 66 [type species: *Dibusaangata*Ross 1939, original designation]. —Wiggins 1977 [larva]. —Marshall 1979b: 218 [generic review; considered incertae sedis within Hydroptilidae]. —Resh and Houp 1986: 30 [life history]. —Oláh and Johanson 2011: 203 [diagnosis; placement].

The monotypic genus *Dibusa* occurs in the United States, recorded from Arkansas, Kentucky, North Carolina, Oklahoma, and Tennessee. Marshall (1979b) did not place the genus in any of the established hydroptilid subfamilies, but she did comment on the unique form of the male genitalia and made note of similarities between *Dibusa* and the genera *Agraylea* and *Nothotrichia*. Oláh and Johanson (2011) indicated that *Dibusa* may belong in the Ochrotrichiinae. The larva was first described by Wiggins (1977) and a detailed life history and description of the larval association with the red alga *Lemaneaaustralis* were given by Resh and Houp (1986).

***angata*** Ross, 1939: 67 [type locality: [United States], Dillsboro, North Carolina; INHS; ♂]. —Ross 1944: 121 [♂]. —Unzicker et al. 1970: 172 [distribution; as *Dibusiaangata*]. —Etnier and Schuster 1979: 17 [distribution]. —Etnier and Schuster 1979: 17 [checklist]. —Blickle 1979: 47, 57 [checklist; ♂]. —Parker and Voshell 1981: 4 [checklist]. —Waltz and McCafferty 1983a: 9 [distribution]. —Harris et al. 1984: 108 [distribution]. —Resh and Houp 1986: 28 [larva; biology]. —Bowles and Mathis 1989: 238 [distribution]. —Floyd and Schuster 1990: 130, 132 [distribution]. —Tarter 1990: 239 [checklist]. —Harris et al. 1991: 165 [distribution]. —Frazer et al. 1991: 19 [distribution]. —Masteller and Flint 1992: 239 [checklist]. —Bowles and Mathis 1992: 32 [distribution]. —Sheath et al. 1995: 890 [red algal association]. —Moulton and Stewart 1996: 91 [♂; larva; distribution]. —Etnier 2010: 485 [distribution]. —Biondi 2010: 61 [distribution]. —Armitage et al. 2011: 13 [distribution]. —Bowles et al. 2020: 7 [distribution].

**Distribution.** —U.S.A.

#### Genus *Maydenoptila* Neboiss, 1977

*Maydenoptila* Neboiss, 1977: 44 [type species: *Maydenoptilacuneola* Neboiss, 1977, original designation]. —Wells 1980: 635 [revision; key to males]. —Wells 1985b: 22 [larva; pupa; case; key to cased larvae]. —Wells 1997: 9 [checklist; key to larvae]. —Harris and Armitage 1997: 127 [placement]. —Holzenthal et al. 2007b: 671 [placement]. —Oláh and Johanson 2011: 203 [diagnosis; placement].

The genus *Maydenoptila* consists of eight species occurring in Australia and the island of Tasmania. Placed in the subfamily Ochrotrichiinae by Harris and Armitage (1997), it was later placed as incertae sedis by Holzenthal et al. (2007b). Oláh and Johanson (2011) indicated, without explanation, that *Maydenoptila* may belong in Ochrotrichiinae. Descriptions of early and mature larval stages of *M.rupina* and of mature larvae of *M.baynesi*, *M.cuneola*, and *M.pseudorupina* were given by Wells (1985b).

***antennifera*** Wells, 1983: 630 [type locality: Australia, New South Wales, Wiangaree State Forest, via Kyogle; NMV; ♂]. —Neboiss 1986: 71 [atlas; ♂].

**Distribution.** —Australia.

***baynesi*** Wells, 1983: 630 [type locality: Australia, Western Australia, Marrinup Brook on Pinjarra-Dwellingup Road at railway crossing; NMV; ♂]. —Wells 1985b: 24 [larva, case, pupa]. —Neboiss 1986: 72 [atlas; ♂].

**Distribution.** —Australia.

***commista*** Wells, 1980: 641 [type locality: [Australia] Victoria, Dee River, 2 km NW. of Millgrove; NMV; ♂]. —Neboiss 1986: 72 [atlas; ♂].

**Distribution.** —Australia.

***cuneola*** Neboiss, 1977: 44 [type locality: [Australia] Tasmania, Wedge River; NMV; ♂; ♀]. —Wells 1980: 637 [♂, ♀; distribution]. —Wells,1985b: 25 [larva, case, pupa]. —Neboiss 1986: 71 [atlas; ♂; ♀]. —Neboiss 2002: 53 [checklist].

**Distribution.** —Australia.

***explicata*** Wells, 1980: 639 [type locality: [Australia] Tasmania, Gordon River, 1 km above First Split; NMV; ♂] —Neboiss 1986: 72 [atlas; ♂]. —Neboiss 2002: 53 [checklist].

**Distribution.** —Australia.

***kurandica*** Wells, 1980: 641 [type locality: [Australia] Queensland, stream 3 miles E. of Kuranda; NMV; ♂]. —Neboiss 1986: 71 [atlas; ♂].

**Distribution.** —Australia.

***pseudorupina*** Wells, 1980: 643 [type locality: [Australia] Victoria, Brodribb River, Sardine Creek Track, 39 km N. of Orbost; NMV; ♂; ♀]. —Wells 1985b: 25 [larva, case, biology]. —Neboiss 1986: 73 [atlas; ♂; ♀].

**Distribution.** —Australia.

***rupina*** Neboiss, 1977: 45 [type locality: [Australia] Tasmania, Guide River Falls nr. Ridgley; NMV; ♂; ♀]. —Wells 1980: 643 [♂, ♀; distribution]. —Wells 1985b: 24 [larva, case, pupa, biology]. —Neboiss 1986: 72 [atlas; ♂; ♀]. —Neboiss 2002: 53 [checklist].

**Distribution.** —Australia.

#### Genus *Metrichia* Ross, 1938

*Metrichia* Ross, 1938b: 9 [type species: *Orthotrichianigritta* Banks, 1907b, original designation]. —Flint 1968b: 48 [to status of subgenus in *Ochrotrichia*]. —Bueno-Soria and Flint 1978: 204 [as subgenus of *Ochrotrichia*; catalog; distribution]. —Marshall 1979b: 186 [reviewed as subgenus of *Ochrotrichia*]. —Blickle 1979: 7 [key to species of America north of Mexico]. —Flint 1991b: 54 [key to Antioquian species]. —Wiggins 1996: 92 [returned to full generic status]. —Flint and Bueno 1998: 489 [checklist; bibliography]. —Bueo-Soria 2002: 224 [revision; Mexican species]. —Bueno-Soria and Holzenthal 2003: 174 [key to males of Central American species]. —Angrisano and Sganga 2005: 114 [key to males and larvae of Argentinian species]. —Oláh and Johanson 2011: 203 [re-description]. —Santos et al. 2016b: 1 [integrative taxonomy].

*Argentitrichia* Jacquemart, 1963b: 339 [type species: *Argentitrichiabulbosa* Jacquemart, 1963b, monotypic]. —Marshall 1979b: 186 [to synonymy].

The genus *Metrichia* consists of 141 species in a Neotropical distribution, recorded from the southwestern United States, throughout Central and South America, and the Greater and Lesser Antilles. *Metrichia* is most closely related to the genus *Ochrotrichia*, in which it was once considered to be a subgenus (Marshall 1979b). Males have characteristic dorsolateral setal brushes on abdominal segments V and VI and internal abdominal sacs (Marshall 1979b). Larvae have been associated and described for *M.nigritta* (Edwards and Arnold 1961; Wiggins 1996) and *M.juana* (Flint 1964).

***aberrans*** (Flint, 1972a): 14 [type locality: Mexico, Veracruz, Fortin de las Flores; NMNH; ♂; in Ochrotrichia (Metrichia)]. —Bueno-Soria and Flint 1978: 204 [distribution]. —Bueno-Soria 2002: 240 [checklist].

**Distribution.** —Mexico.

***acicula*** Bueno-Soria & Holzenthal, 2003: 175 [type locality: Costa Rica, Guanacaste, Río Mena, 4.2 km W Santa Cecilia, 11.059°N, 85.448°W, 260 m; UMSP; ♂].

**Distribution.** —Costa Rica.

***acuminata*** Santos, Takiya, & Nessimian, 2016: 8 [type locality: Brazil, Ceará, Ubajara, Parque Nacional de Ubajara, Cachoeira do Gameleira, 03°50'21"S 40°54'23"W, 880 m; CZMA; ♂].

**Distribution.** —Brazil.

***adamsae*** Flint & Bueno-Soria, 1998: 493 [type locality: Peru, Madre de Dios, Pakitza, 12°7'S 70°58'W; NMNH; ♂].

**Distribution.** —Peru.

***alajuela*** Bueno-Soria & Holzenthal, 2003: 177 [type locality: Costa Rixa, Alajuela, Reserva Forestal San Ramón, Río San Lorencito and tributaries, 10.216°N, 84.607°W, 980 m; UMSP; ♂].

**Distribution.** —Costa Rica.

***alhoma*** Oláh & Johanson, 2011: 204 [type locality: Peru, San Martin Prov., La Catarata de Ahuashiyascu, 6°27.544'S, 76°18.192'W; NHRS; ♂].

**Distribution.** —Peru.

***amplitudinis*** Bueno-Soria & Holzenthal, 2003: 177 [type locality: Costa Rica, Cartago, Reserva Tapantí, Río Grande de Orosí, 9.686°N, 83.756°W, 1650 m; UMSP; ♂]. —Armitage et al. 2020: 4 [distribution].

**Distribution.** —Costa Rica, Panama.

***ancora*** Bueno-Soria & Holzenthal, 2003: 177 [type locality: Costa Rica, Guanacaste, Río Góngora (Sulfur mine), 4 km (air) NE Quebrada Grande, 10.887°N, 85.470°W, 590 m; UMSP; ♂]. —Armitage and Harris 2018a: 9 [distribution]. —Armitage and Harris 2018b: 97 [checklist]. —Armitage and Harris 2018c: 283 [distribution].

**Distribution.** —Costa Rica, Panama.

***angulosa*** Bueno-Soria & Holzenthal, 2003: 179 [type locality: Costa Rica, Puntarenas, Río Cotón in Las Alturas, 8.938°N, 82.826°W, 1360 m; UMSP; ♂].

**Distribution.** —Costa Rica.

***anisoforficata*** Parker & Voshell, 1979: 43 [type locality: [United States], Virginia, Giles Co., Stony Creek between Olean and Interior, 650–670 m; NMNH; ♂]. —Parker and Voshell 1981: 4 [checklist].

**Distribution.** —U.S.A.

***anisoscola*** (Flint, 1991b): 58 [type locality: Colombia, Dpto. Antioquia, Quebrada La Ayura, Envigado (trap B); ♂; in Ochrotrichia (Metrichia)]. —Muñoz-Quesada 2000: 278 [checklist].

**Distribution.** —Colombia.

***anticheirion*** Thomson & Armitage, 2018: 5 [type locality: Panama, Chiriquí Province, Cuenca 102 (Río Chiriquí Viejo), Quebrada Norte, Mount Totumas Biological Reserve, 8.873613°N, 82.690512°W; COZEM; ♂].

**Distribution.** —Panama.

***araguensis*** (Flint, 1981): 29 [type locality: Venezuela, Aragua, Dos Riitos, 6 km N Rancho Grande; NMNH; ♂; in Ochrotrichia (Metrichia)].

**Distribution.** —Venezuela.

***arenifera*** (Flint, 1980a): 214 [type locality: Peru, Dept. del Cuzco, Rio Vilcanota above P’Isaq; NMNH; ♂; in Ochrotrichia (Metrichia)].

**Distribution.** —Peru.

***argentinica*** Schmid, 1958a: 195 [type locality: Argentina, Siambon, Tucuman; depository not designated; ♂]. —Flint 1974a: 87 [as Ochrotrichia (Metrichia)]. —Flint and Bueno-Soria 1998: 490 [as *Metrichia*]. —Angrisano 1999: 33 [checklist]. —Angrisano and Sganga 2005: 116 [larva; distribution]. —Isa Miranda and Rueda Martín 2014: 199 [distribution]. —Ríos-Touma et al. 2017: 10 [distribution].

**Distribution.** —Argentina, Chile, Ecuador, Peru.

***arizonensis*** (Flint, 1972a): 12 [type locality: U.S.A., Arizona, Santa Cruz Co., Sycamore Canyon, ATascosa Mts.; NMNH; ♂; in Ochrotrichia (Metrichia)]. —Blickle 1979: 50, 59 [checklist; ♂]. —Blinn and Ruiter 2005: 69 [distribution; biology]. —Bueno-Soria et al. 2007: 33 [distribution].

**Distribution.** —Mexico, U.S.A.

***avon*** (Bueno-Soria, 1983b): 82 [type locality: Mexico, Chiapas, Cascada de Misolha, 20 km SE Palenque; CNIN; ♂; in Ochrotrichia (Metrichia)]. —Bueno-Soria 2002: 240 [checklist]. —Bueno-Soria and Holzenthal 2003: 196 [distribution]. —Bueno-Soria et al. 2007: 75 [distribution]. —Armitage et al. 2016: 8 [distribution]. —Armitage and Harris 2018b: 97 [checklist]. —Harris and Armitage 2019: 5 [distribution].

—*extragna* Bueno-Soria and Barba-Álvarez, 1999b: 30 [type locality: Mexico, Guerrero, Taxco, Totoapan, río Temascalapa, 8 km NW Ahuehuepan, Rd. 51, 18°22.70'N: 99°39.77'W, 900 m; CNIN; ♂]. —Bueno-Soria 2002: 240 [to synonymy].

**Distribution.** —Costa Rica, Mexico, Panama.

***azul*** Santos, Takiya, & Nessimian, 2016: 13 [type locality: Brazil, Paraná, Céu Azul, Parque Nacional do Iguaçu, Rio Azul, 25°09'21"S 43°47'4"W, 510 m; DZRJ; ♂].

**Distribution.** —Brazil.

***bidentata*** (Flint, 1983): 41 [type locality: Argentina, Pcia. Neuquen, 13 km E Quila Quina; NMNH; ♂; in Ochrotrichia (Metrichia)]. —Angrisano 1999: 33 [checklist]. —Angrisano and Sganga 2005: 116 [larva; distribution].

**Distribution.** —Argentina, Chile.

***biungulata*** (Flint, 1972a): 13 [type locality: Panama, Cerro Campana; NMNH; ♂; in Ochrotrichia (Metrichia)]. —Aguila 1992: 539 [distribution]. —Bueno-Soria and Santiago-Fragoso 2002: 252 [checklist]. —Bueno-Soria and Holzenthal 2003: 196 [distribution]. —Armitage et al. 2015a: 7 [checklist]. —Armitage and Cornejo 2015: 195 [checklist]. —Armitage and Harris 2018b: 97 [checklist].

**Distribution.** —Costa Rica, Panama.

***bola*** (Flint, 1991b): 58 [type locality: Colombia, Dpto. Antioquia, Quebrada La Cebolla, El Retiro (trap A); NMNH; ♂; in Ochrotrichia (Metrichia)]. —Muñoz-Quesada 2000: 278 [checklist].

**Distribution.** —Colombia.

***bonita*** Santos, Takiya, & Nessimian, 2016: 15 [type locality: Brazil, Mato Grosso do Sul, Bonito, Rio Formosinho, 21°1'16"S 56°26'47"W, 275 m; DZRJ; ♂].

**Distribution.** —Brazil.

***bostrychion*** Thomson & Holzenthal, 2012: 23 [type locality: Venezuela, Monagas, Guachero Cave National Park at La Paila waterfall, 10°10.322'N, 63°33.315'W, 1110 m; UMSP; ♂].

**Distribution.** —Venezuela.

***bracui*** Santos, Takiya, & Nessimian, 2016: 19 [type locality: Brazil, Rio de Janeiro, Angra dos Reis, Rio Bracuí, 23°0'23"S 44°29'15"W, 75 m; DZRJ; ♂].

**Distribution.** —Brazil.

***brevitas*** Bueno-Soria & Santiago-Fragoso, 2002: 252 [type locality: Panama, Chiriqui, Guadalupe, Arriba, 8°52'26"N 82°33'13'W; NMNH; ♂]. —Armitage et al. 2015a: 7 [checklist]. —Armitage and Cornejo 2015: 195 [checklist]. —Armitage and Harris 2018b: 97 [checklist].

**Distribution.** —Panama.

***brocha*** Thomson & Armitage, 2018: 3 [type locality: Panama, Chiriquí Province, Cuenca 102 (Río Chiriquí Viejo), Quebrada Norte, Mount Totumas Biological Reserve, 8.873613°N, 82.690512°W; COZEM; ♂].

**Distribution.** —Panama.

***bulbosa*** (Jacquemart, 1963b): 339 [type locality: Argentina, [San Juan], Rio Sasso; IRSNB; ♂; in *Argentitrichia*]. —Mangeaud 1996: 154 [distribution]. —Angrisano 1999: 33 [checklist]. —Angrisano and Sganga 2005: 117 [♀; larva; distribution]. —Isa Miranda and Rueda Martín 2014: 199 [distribution].

**Distribution.** —Argentina.

***cafetalera*** Botosaneanu, 1980: 110 [type locality: Cuba, Prov., Las Villas, Cafetal Gavina, La Sierrita; ZMUA; ♂]. —Botosaneanu 1979: 49 [distribution]. —Botosaneanu 1995a: 26 [♂; ♀; distribution]. —Flint 1996a: 16 [checklist]. —Botosaneanu 2002b: 84 [checklist]. —Flint and Peréz-Gelabert 1999: 40 [checklist]. —Flint and Sykora 2004: 32 [distribution]. —Naranjo López and González Lazo 2005: 149 [checklist]. —Peréz-Gelabert 2008: 300 [checklist].

**Distribution.** —Cuba, Dominican Republic.

***calla*** Thomson & Armitage, 2018: 4 [type locality: Panama, Chiriquí Province, Cuenca 102 (Río Chiriquí Viejo), Quebrada Norte, Mount Totumas Biological Reserve, 8.873613°N, 82.690512°W; COZEM; ♂].

**Distribution.** —Panama.

***campana*** (Flint, 1968a): 62 [type locality: Dominica, D’leau Gommier; NMNH; ♂; in Ochrotrichia (Metrichia)]. —Flint and Sykora 1993: 50 [checklist]. —Botosaneanu 2002b: 84 [checklist]. —Botosaneanu and Thomas 2005: 55 [checklist].

**Distribution.** —Dominica, Guadeloupe.

***caraca*** Santos, Takiya, & Nessimian, 2016: 20 [type locality: Brazil, Minas Gerais, Catas Altas, RPPN Santuário do Caraça, Ribeirão, Caraça; DZRJ; ♂].

**Distribution.** —Brazil.

***carbetina*** (Botosaneanu, 1994a): 38 [type locality: Guadeloupe, Chute du Carbet, 580 m; ZMUA; ♂; in Ochrotrichia (Metrichia)]. —Botosaneanu 2002b: 84 [checklist]. —Botosaneanu and Thomas 2005: 40 [distribution].

**Distribution.** —Guadeloupe, Martinique.

***ceer*** (Flint, 1992a): 387 [type locality: Puerto Rico, El Verde Field Station, Quebrada Prieta; NMNH; ♂; in Ochrotrichia (Metrichia)]. —Botosaneanu 2002b: 84 [checklist].

**Distribution.** —Puerto Rico.

***circulatrix*** Bueno-Soria, 2002: 224 [type locality: Mexico, Tabasco, Municipio de Huimanguillo, Arroyo las Flores, Villa de Guadalupe, 2a sección Los Chimalapas, km 5+920, Ruta Malpasito-Carlos A. Madrazo, 17°22'05"N 93°36'25"W; CNIN; ♂].

**Distribution.** —Mexico.

***circuliforme*** Santos, Takiya, & Nessimian, 2016: 22 [type locality: Brazil, Rio de Janeiro, Itatiaia, Rio das Pedras, Cchoeira de Deus, 22°25'0"S 44°32'50"W, 689 m; DZRJ; ♂].

**Distribution.** —Brazil.

***continentalis*** (Flint, 1972a): 14 [type locality: Panama, Canal Zone, Barro Colorado Island; NMNH; ♂; in Ochrotrichia (Metrichia)]. —Aguila 1992: 539 [distribution]. —Bueno-Soria and Santiago-Fragoso 2002: 252 [checklist]. —Armitage et al. 2015a: 7 [checklist]. —Armitage and Cornejo 2015: 195 [checklist]. —Armitage and Harris 2018b: 97 [checklist]. —Harris and Armitage 2019: 5 [distribution].

**Distribution.** —Panama.

***corazones*** Armitage & Harris, 2020a: 4 [type locality: Panama, Coclé Province, Cuenca 105, Omar Torrijos Herrera National Park, Quebrada Corazones, PSPSCB-PNGDOTH-C103-2017-001, 8.6776°N, 80.6001°W, 728 m; COZEM; ♂].

**Distribution.** —Panama.

***crenula*** Bueno-Soria, 2002: 227 [type locality: Mexico, Morelos, Huautla Estación CEAMISH, 2.5 km N 4 km W, 18°27.871'N, 99°02.475'W, 940 m; CNIN; ♂].

**Distribution.** —Mexico.

***cuenca*** (Harper & Turcotte, 1985): 139 [type locality: Ecuador, small stream outlet of Laguna Verde Cocha, near junction with Rio Matadeto, Chirimachay, Quinuas Valley; UMQ; ♂ in Ochrotrichia (Metrichia)]. —Ríos-Touma et al. 2017: 10 [checklist].

**Distribution.** —Ecuador.

***cuniapiru*** Angrisano, 2005 in Angrisano and Sganga 2005: 114 [type locality: Argentina, Misiones, Cuñá Pirú Provincial Park, Cuñá Pirú Stream; MLPA; ♂; ♀].

**Distribution.** —Argentina.

***curta*** Santos, Takiya, & Nessimian, 2016: 24 [type locality: Brazil, Rio de Janeiro, Itatiaia, Rio das Pedras, 22°24'33"S 44°33'08"W; DZRJ; ♂].

**Distribution.** —Brazil.

***cuspidata*** (Flint, 1991b): 57 [type locality: Colombia, Dpto. Antioquia, 10 km E Medellin, road to Pas Palmas; NMNH; ♂; in Ochrotrichia (Metrichia)]. —Muñoz-Quesada 2000: 278 [checklist]. —Oláh and Johanson 2011: 205 [distribution].

**Distribution.** —Colombia, Mexico.

***decora*** Bueno-Soria & Holzenthal, 2003: 179 [type locality: Costa Rica, Heredia, Río Sarapiquí, 7 km W Puerto Vieho, 10.452°N, 84.067°W, 50 m; UMSP; ♂].

**Distribution.** —Costa Rica.

***difusa*** Bueno-Soria & Santiago-Fragoso, 2002: 246 [type locality: Panama, Barro Colorado, Island Snyder Molino Makers 3; NMNH; ♂]. —Armitage et al. 2015a: 7 [checklist]. —Armitage and Cornejo 2015: 195 [checklist]. —Armitage and Harris 2018b: 97 [checklist].

**Distribution.** —Panama.

***diosa*** Flint & Bueno-Soria, 1998: 491 [type locality: Peru, Madre de Dios, Pakitza, 12°7'S 70°58'W; NMNH; ♂].

**Distribution.** —Peru.

***disparilis*** (Flint, 1983): 41 [type locality: Argentina, Pcia. Tucuman, Rt. 307, 33.7 km W Acheral; NMNH; ♂; in Ochrotrichia (Metrichia)]. —Angrisano 1999: 33 [checklist]. —Angrisano and Sganga 2005: 119 [larva; distribution]. —Isa Miranda and Rueda Martín 2014: 199 [distribution].

**Distribution.** —Argentina.

***eltera*** Oláh & Johanson, 2011: 205 [type locality: French Guiana, Approuaguekaw, Kaw Mt, 4°33.035'N, 52°11.661'W, 104 m; NHRS; ♂].

**Distribution.** —French Guiana.

***enigmatica*** Bueno-Soria & Santiago-Fragoso, 2002: 249 [type locality: Panama, San Blas, Río Carti Grande, 2 km W Nusagandi; NMNH; ♂]. —Armitage et al. 2015a: 7 [checklist]. —Armitage and Cornejo 2015: 195 [checklist]. —Armitage and Harris 2018b: 97 [checklist].

**Distribution.** —Panama.

***espera*** Botosaneanu, 1977: 265 [type locality: Cuba, Pinar del Rio, Soroa, Rio Manantiales; NMNH; ♂]. —Botosaneanu 1979: 49 [distribution]. —Botosaneanu 1980: 111 [♀]. —Flint, 1996a: 16 [checklist]. —Botosaneanu 2002b: 84 [checklist]. Naranjo López and González Lazo 2005: 149 [checklist].

**Distribution.** —Cuba.

***excisa*** (Kumanski, 1987): 20 [type locality: Cuba, Province Las Villas, Sierra de Trinidad, small torrent on road Trinidad-Topes de Colantes; SOFM; ♂; ♀; in Ochrotrichia (Metrichia)]. —Flint 1996a: 16 [checklist]. —Botosaneanu 2002b: 84 [checklist]. —Naranjo López and González Lazo 2005: 149 [checklist].

**Distribution.** —Cuba.

***exclamationis*** (Flint, 1968a): 64 [type locality: Dominica, Clarke Hall, cocoa trail; NMNH; ♂; in Ochrotrichia (Metrichia)]. —Flint and Sykora 1993: 50 [checklist]. —Botosaneanu 1994a: 38 [distribution]. —Botosaneanu 2002b: 84 [checklist]. —Botosaneanu and Thomas 2005: 55 [checklist].

**Distribution.** —Dominica, Guadeloupe.

***extragma*** Bueno-Soria & Barba-Álvarez, 1999b: 30 [type locality: Mexico, Guerrero, Taxco, Totoapan, río Temascalapa, 8 km NW Ahuehuepan, Rd. 51, 18°22.70'N, 99°39.77'W, 900 m; CNIN; ♂]. —Bueno-Soria 2002: 240 [checklist].

**Distribution.** —Mexico.

***farofa*** Santos, Takiya, & Nessimian, 2016: 24 [type locality: Brazil, Minas Gerais, Jaboticatubas, Parque Nacional da Serra do Cipó, Cachoeira da Farofa, 19°22'47"S 43°34'36"W, 811 m; DZRJ; ♂].

**Distribution.** —Brazil.

***favus*** (Botosaneanu) in Botosaneanu and Alkins-Koo 1993: 18 [type locality: Trinidad, two 1^st^ order streams, La Laja catchment of Rio Guanapo; ZMUA; ♂; in Ochrotrichia (Metrichia)]. —Botosaneanu and Sakal 1992: 202 [distribution; ecology]. —Flint 1996b: 96 [distribution]. —Botosaneanu 2002b: 84 [checklist].

**Distribution.** —Trinidad.

***florecita*** Bueno-Soria, 2002: 228 [type locality: Mexico, Tabasco, Municipio de Huimanguillo Ejido, Villa de Guadalupe, 1a sección, Cascada Cerro de Las Flores, 17°21'39"N 93°37'29"W, Rta. Malpasito-Carlos A. Madrazo; CNIN; ♂].

**Distribution.** —Mexico.

***fontismoreaui*** (Botosaneanu, 1991): 125 [type locality: Haiti, Departement du Sud, pres de Camp Perrin, Resurgence du Moreau; ZMUA; ♂; in Ochrotrichia (Metrichia)]. —Botosaneanu 1995a: 27 [♀; distribution]. —Flint and Pérez-Gelabert 1999: 40 [checklist]. —Flint and Sykora 2004: 32 [distribution]. —Pérez-Gelabert 2008, 300 [checklist].

**Distribution.** —Dominican Republic, Haiti.

***forceps*** Santos, Takiya, & Nessimian, 2016: 27 [type locality: Brazil, Paraná, Céu Azul, Parque Nacional do Iguaçu, Rio Azul, 25°09'21"S 53°47'44"W, 510 m; DZRJ; ♂].

**Distribution.** —Brazil.

***formosinha*** Santos, Takiya, & Nessimian, 2016: 28 [type locality: Brazil, Mato Grosso do Sul, Bonito, Rio Formosinho, 21°10'16"S 56°26'46"W, 275 m; DZRJ; ♂].

**Distribution.** —Brazil.

***fugga*** Oláh & Johanson, 2011: 207 [type locality: Peru, San Martin Prov., La Catarata de Ahuashiyascu, 6°27.544'S, 76°18.192'W; NHRS; ♂].

**Distribution.** —Peru.

***geminata*** (Flint, 1996b): 96 [type locality: Trinidad, streamlet, Lalaja Rd., 520 m, 10°43'N 61°17'W; NMNH; ♂; in Ochrotrichia (Metrichia)]. —Botosaneanu 2002b: 85 [checklist].

**Distribution.** —Tobago, Trinidad.

***goiana*** Santos, Takiya, & Nessimian, 2016: 30 [type locality: Brazil, Goiás, Alto Paraíso de Goiás, Rio Bartolomeu tributary, 14°07'25"S 47°30'30"W, 1165 m; DZRJ; ♂].

**Distribution.** —Brazil.

***gomboska*** Oláh & Johanson, 2011: 208 [type locality: Peru, Huanuco, Tomayquichua Distr. River Tomayquichua, humid subtropical forest, 10°04'27"S 76°12'36"W, 2041 m; NHRS; ♂].

**Distribution.** —Peru.

***gordita*** Bueno-Soria & Holzenthal, 2003: 183 [type locality: Costa Rica, Puntarenas, Río Singri, ca. 2 km (air) S Finca Helechales, 9.057°N, 83.082°W, 720 m; UMSP; ♂].

**Distribution.** —Costa Rica.

***haranga*** Oláh & Johanson, 2011: 210 [type locality: Peru, San Martin Prov., La Catarata de Ahuashiyascu, 6°27.544'S, 76°18.192'W; NHRS; ♂].

**Distribution.** —Peru.

***helenae*** Flint & Bueno-Soria, 1998: 495 [type locality: Peru, Madre de Dios, Pakitza, 12°7'S 70°58'W; NMNH; ♂].

**Distribution.** —Peru.

***itabaiana*** Santos, Takiya, & Nessimian, 2016: 32 [type locality: Brazil, Sergipe, Areia Branca, Parque Nacional da Serra de Itabaiana, Rio dos Negros, 10°44'51"S 37°20'24"W, 208 m; DZRJ; ♂].

**Distribution.** —Brazil.

***jorobada*** Bueno-Soria, 2002: 228 [type locality: Mexico, Tabasco, Municipio de Huimanguillo, Arroyo las Flores, Vill ade Guadalupe, 2a sección Los Chimalapas, km 5+920, Ruta Malpasito-Carlos A. Madrazo, 17°22'05"N 93°36'25"W; CNIN; ♂].

**Distribution.** —Mexico.

***juana*** (Flint, 1964): 60 [type locality: Puerto Rico, Toro Negro Forest, Dona Juana Creek; NMNH; ♂; ♀; larva; in Ochrotrichia (Metrichia)]. —Flint 1968a: 82 [checklist]. —Botosaneanu 2002b: 85 [checklist].

**Distribution.** —Puerto Rico.

***kocka*** Oláh & Johanson, 2011: 212 [type locality: Peru, San Martin Prov., La Catarata de Ahuashiyascu, 6°27.544'S, 76°18.192'W; NHRS; ♂].

**Distribution.** —Peru.

***kumanskii jamaicae*** Botosaneanu in Botosaneanu and Hyslop 1998: 13 [type locality: Jamaica, Buff Bay River in Green Hill at “Regale”, Blue Mountains, Portland; ZMUA; ♂]. —Botosaneanu 2002b: 85 [checklist].

**Distribution.** —Jamaica.

***kumanskii kumanskii*** (Botosaneanu, 1991): 128 [type locality: Haiti, Departement de l’Quest, Ville Bonheur, Le Saut d’Eau; ZMUA; ♂; in Ochrotrichia (Metrichia)]. —Flint and Pérez-Gelabert 1999: 40 [checklist]. —Botosaneanu 2002b: 85 [checklist]. —Flint and Sykora 2004: 33 [distribution]. —Pérez-Gelabert 2008: 300 [checklist].

**Distribution.** —Dominican Republic, Haiti.

***lacuna*** (Bueno-Soria, 1983b): 79 [type locality: Mexico, Chiapas, Cascada de Misolha, 20 km SE Palenque; CNIN; ♂; in Ochrotrichia (Metrichia)]. —Bueno-Soria 2002: 240 [checklist]. —Bueno-Soria and Barba-Álvarez 2011: 357 [checklist].

**Distribution.** —Mexico.

***lemniscata*** (Flint, 1972a): 14 [type locality: Panama, Chiriqui, David, Rovira; NMNH; ♂; in Ochrotrichia (Metrichia)]. —Aguila 1992: 539 [distribution]. —Bueno-Soria and Santiago-Fragoso 2002: 252 [checklist]. —Bueno-Soria and Holzenthal 2003: 196 [distribution]. —Armitage et al. 2015a: 7 [checklist]. —Armitage and Cornejo 2016: 195 [checklist]. —Armitage and Harris 2018b: 97 [checklist]. —Armitage and Harris 2018c: 283 [distribution].

**Distribution.** —Costa Rica, Panama.

***lenophora*** (Flint, 1991b): 54 [type locality: Colombia, Dpto. Antioquia, 10 km E. Medellin, road to Guarne; NMNH; ♂; in Ochrotrichia (Metrichia)]. —Muñoz-Quesada 2000: 278 [checklist].

**Distribution.** —Colombia.

***longiphallata*** Mey & Ospina-Torres, 2018: 30 [type locality: Colombia, Bogotá, Chapinero, Quebrada La Vieja; ICN; ♂].

**Distribution.** —Colombia.

***longispina*** Flint & Sykora, 2004: 33 [type locality: Dominican Republic, [La Vega Province], Convento, 12 km S Constanza; NMNH; ♂; ♀]. —Pérez-Gelabert 2008: 300 [checklist].

**Distribution.** —Dominican Republic.

***longissima*** Santos, Takiya, & Nessimian, 2016: 34 [type locality: Brazil, Rio de Janeiro, Itatiaia, Rio Palmital, 22°25'34"S 44°32'52"W, 637 m; DZRJ; ♂].

**Distribution.** —Brazil.

***longitudinis*** Bueno-Soria, 2002: 231 [type locality: Mexico, Tabasco, Municipio de Huimanguillo Ejido Villa de Guadalupe, 1a sección, Cascada Cerro de Las Flores, 17°21'39"N 93°37'29"W, Rta. Malpasito-Carlos A. Madrazo; CNIN; ♂].

**Distribution.** —Mexico.

***luna*** Bueno-Soria & Holzenthal, 2003: 183 [type locality: Costa Rica, Alajuela, Reserva Forestal San Ramón, Río San Lorencito and tribs., 10.216°N, 84.607°W, 980 m; UMSP; ♂].

**Distribution.** —Costa Rica.

***macdonaldi*** Harris & Armitage, 2019: 10 [type locality: Panama, Bocas del Toro Province, Quebrada Rambala, near Rambala Jungle Lodge, 3.74 km SSE Rambala, 8.91627°N and 82.15469°W, 120 m; COZEM; ♂].

**Distribution.** —Panama.

***macrophallata*** (Flint, 1991b): 57 [type locality: Colombia, Dpto. AntioquiaQuebrada Honda, Marsella, 12 km SW Fredonia; NMNH; ♂; in Ochrotrichia (Metrichia)]. —Muñoz-Quesada 2000: 278 [checklist]. —Armitage and Harris 2020a: 8 [distribution].

**Distribution.** —Colombia, Panama.

***madicola*** (Botosaneanu, 1994a): 39 [type locality: Guadeloupe, Chute du Carpet, 580 m; ZMUA; ♂; in Ochrotrichia (Metrichia)]. —Botosaneanu 2002b: 85 [checklist]. —Botosaneanu and Thomas 2005: 40 [distribution].

**Distribution.** —Guadeloupe, Martinique.

***madre*** Flint & Bueno-Soria, 1998: 493 [type locality: Peru, Madre de Dios, Pakitza, 12°7'S 70°58'W; NMNH; ♂].

**Distribution.** —Peru.

***magna*** Bueno-Soria & Holzenthal, 2003: 185 [type locality: Costa Rica, Puntarenas, roadside seep, route 2, just W km 234, 8.976°N, 83.299°W, 100 m; UMSP; ♂].

**Distribution.** —Costa Rica.

***malada*** (Flint, 1991b): 55 [type locality: Colombia, Dpto. Antioquia, Quebrada Agua Mala, 34 km NW Medellin, road to San Jeronimo; NMNH; ♂; in Ochrotrichia (Metrichia)]. —Flint and Reyes 1991: 487 [distribution]. —Muñoz-Quesada 200: 278 [checklist].

**Distribution.** —Colombia, Peru.

***mastelleri*** Harris & Flint, 2016: 6 [type locality: United States, Arizona, Yavapai County, Fossil Creek, 7.5 km (air) NW Strawberry, N34°25.4', W111°34.4'; NMNH; ♂].

**Distribution.** —U.S.A.

***mechuda*** Bueno-Soria & Holzenthal, 2003: 185 [type locality: Costa Rica, San José, Río Savegra, ca. San Gerardo de Dota, 9.33°N, 83.48°W, 2200 m; CMNH; ♂].

**Distribution.** —Costa Rica.

***meta*** Bueno-Soria & Holzenthal, 2003: 185 [type locality: Costa Rica, Guanacaste, Parque Bacional Rincón de la Vieja, Quebrada Zopilote, 10.765°N, 85.309°W, 785 m; UMSP; ♂].

**Distribution.** —Costa Rica.

***minera*** Bueno-Soria, 2002: 231 [type locality: Mexico, Veracruz, Las Minas; CNIN; ♂].

**Distribution.** —Mexico.

***munieca*** Botosaneanu, 1977: 264 [type locality: Cuba, Oriente, Gran Piedra, Arroyos de la Idalia; NMNH; ♂]. —Botosaneanu 1979: 49 [distribution]. —Flint 1996a: 16 [checklist]. —Botosaneanu 2002b: 85 [checklist]. —Naranjo López and González Lazo 2005: 149 [checklist].

**Distribution.** —Cuba.

***necopina*** Botosaneanu & Thomas, 2005: 40 [type locality: Martinique, Riv Lezarde au départ de la route forestiere de Palourde, 250 m; ZMUA; ♂].

**Distribution.** —Martinique.

***neotropicalis*** Schmid, 1958a: 195 [type locality: Argentine, Siamba, Tucuman; NMNH; ♂]. —Flint 1967a: 56 [distribution; misidentified as *M.argentinica* according to Angrisano and Sganga 2005: 119]. —Flint 1974a: 88 [checklist]. —Flint 1980a: 216 [correction of switched captions in Schmid 1958a]. —Flint 1990: 118 [distribution]. —Mangeaud 1996: 154 [distribution]. —Vallania et al. 1998: 8 [distribution]. —Angrisano 1999: 33 [checklist]. —Angrisano and Sganga 2005: 117 [♀; larva; pupa; distribution]. —Muzón et al. 2005: 57 [distribution]. —Miserendino and Brand 2007: 312 [biology]. —Scheibler and Debandi 2008: 151 [distribution]. —Brand and Miserendino 2011a: 35 [biology]. —Brand and Miserendino 2011b: 143 [biology]. —Brand and Miserendino 2014: 6 [community ecology]. —Isa Miranda and Rueda Martín 2014: 199 [distribution].

**Distribution.** —Argentina, Chile, Peru.

***nigritta*** (Banks, 1907b): 163 [type locality: United States, Texas, Austin; MCZ; ♂; in *Orthotrichia*]. —Banks 1907a: 50 [catalogue]. —Ross 1938b: 9 [type species of *Metrichia*; lectotype designated; ♂]. —Ross 1944: 121 [♂]. —Edwards and Arnold 1961: 411 [larva]. —Flint 1968b: 48 [in Ochrotrichia (Metrichia)]. —Flint 1972a: 12 [♂; distribution]. —Edwards 1973: 506 [distribution]. —Bueno-Soria and Flint 1978: 204 [distribution]. —Blickle 1979: 50, 59 [checklist; ♂]. —Bowles and Mathis 1992: 32 [distribution; in *Ochrotrichia*]. —Moulton et al. 1994: 165 [distribution]. —Wiggins 1996: 92 [larva]. —Moulton and Stewart 1996: 110 [♂; distribution]. —Moulton and Stewart 1997: 350 [checklist]. —Bueno-Soria 2002: 240 [checklist]. —Bueno-Soria and Santiago-Fragoso 2002: 252 [distribution]. —Rojas-Ascencio et al. 2002: 377 [distribution]. —Blinn and Ruiter 2005: 69 [distribution; bioogy]. —Blinn and Ruiter 2006: 333 [biology; distribution]. —Bowles et al. 2007: 22 [distribution; biology]. —Blinn and Ruiter 2009b: 186 [phenology; distribution]. —Oláh and Johanson 2011: 212 [distribution]. —Armitage et al. 2015a: 7 [checklist]. —Armitage and Cornejo 2015: 195 [checklist]. —Armitage and Harris 2018b: 97 [checklist].

—*volada* Blickle & Denning, 1977: 295 [type locality: United States, Arizona, Page Springs Fish Hatchery; DPC; ♂; ♀]. —Blickle 1979: 50, 59 [checklist; ♂]. —Moulton et al. 1994: 165 [to synonymy].

**Distribution.** —El Salvador, Mexico, Panama, U.S.A.

***nowaczyki*** Harris & Armitage, 2015: 8 [type locality: Panama, Chiriquí Province, Cuenca 108, Quebrada Grande, Boquete, Valle Escondido, below Sabor Restaurant, 8.7790°N, 82.44016°W, 1122 m; MIUP; ♂]. —Armitage et al. 2015a: 7 [checklist]. —Armitage and Cornejo 2015: 195 [checklist]. —Armitage and Harris 2018b: 97 [checklist]. —Armitage and Harris 2018c: 283 [distribution].

**Distribution.** —Panama.

***pakitza*** Flint & Bueno-Soria, 1998: 491 [type locality: Peru, Madre de Dios, Pakitza, 12°7'S 70°58'W; NMNH; ♂].

**Distribution.** —Peru.

***palida*** Bueno-Soria & Santiago-Fragoso, 2002: 249 [type locality: Panama, Chiriqui, Guadalupe, Arriba, 8°52'26"N 82°33'13"W; NMNH; ♂]. —Armitage et al. 2015a: 7 [checklist]. —Armitage and Cornejo 2015: 195 [checklist]. —Armitage and Harris 2018b: 97 [checklist].

**Distribution.** —Panama.

***patagonica*** (Flint, 1983): 41 [type locality: Argentina, Pcia. Rio Negro, 5 km 5 Rio Villegas; NMNH; ♂; in Ochrotrichia (Metrichia)]. —Mangeaud 1996: 154 [distribution]. —Angrisano 1999: 33 [checklist]. —Angrisano and Sganga 2005: 121 [♀; larva; case; distribution]. —Brand and Miserendino 2011a: 35 [biology]. —Brand and Miserendino 2011b: 143 [biology]. —Oláh and Johanson 2011: 213 [distribution]. —Brand and Miserendino 2014: 6 [community ecology]. —Ríos-Touma et al. 2017: 10 [distribution].

**Distribution.** —Argentina, Chile, Ecuador, Peru.

***peluda*** Santos, Takiya, & Nessimian, 2016: 35 [type locality: Brazil, Rio de Janeiro, Itatiaia, 1^st^ order tributary of Rio Palmital, 22°25'40"S 44°32'46"W, 584 m; DZRJ; ♂].

**Distribution.** —Brazil.

***penicillata*** (Flint, 1972a): 13 [type locality: Guatemala, Escuintla, Grutas de San Pedro Martir; ♂; in Ochrotrichia (Metrichia)]. —Bueno-Soria 2002: 240 [checklist]. —Bueno-Soria and Santiago-Fragoso 2002: 253 [distribution]. —Bueno-Soria and Holzenthal 2003: 196 [distribution]. —Chamorro-Lacayo et al. 2007: 43 [checklist]. —Armitage et al. 2015a: 7 [checklist]. —Armitage and Cornejo 2015: 195 [checklist]. —Armitage and Harris 2018b: 97 [checklist]. —Harris and Armitage 2019: 5 [distribution].

**Distribution.** —Costa Rica, Guatemala, Nicaragua, Panama.

***pernambucana*** de Souza & Santos in de Souza et al. 2013: 584 [type locality: Brazil, Pernambuco State, Tamandaré, Reserva de Biológica de Saltinho, Riacho Mamucabas, 35°11;14.0: W, 08°43;21.6: S; DZRJ; ♂]. —Paprocki and França 2014: 46 [checklist].

**Distribution.** —Brazil.

***picuda*** Bueno-Soria & Holzenthal, 2003: 187 [type locality: Costa Rica, Alajuela, Reserva Forestal San Ramón, Río San Lorencito and tribs., 10.216°N, 84.607°W, 980 m; UMSP; ♂]. —Armitage et al. 2016: 8 [distribution]. —Armitage and Harris 2018b: 97 [checklist]. —Armitage and Harris 2018c: 283 [distribution]. —Harris and Armitage 2019: 5 [distribution].

**Distribution.** —Costa Rica, Panama.

***pitu*** Angrisano & Sganga, 2009: 60 [type locality: Argentina, Misiones, Departamento de Cainguás, Parque Provincial Salto Encantado; MACN; ♂].

**Distribution.** —Argentina.

***platigona*** (Botosaneanu) in Botosaneanu and Alkins-Koo 1993: 18 [type locality: Tobago, Argyll River below Argyll waterfall; ZMUA; ♂; in Ochrotrichia (Metrichia)]. —Botosaneanu and Sakal 1992: 202 [distribution; ecology]. —Flint 1996b: 95 [distribution]. —Botosaneanu 2002b: 85 [checklist].

**Distribution.** —Tobago, Trinidad, Venezuela.

***potosina*** Bueno-Soria, 2002: 232 [type locality: Mexico, San Luis Potosí, La Cascada del Tamasopo; NMNH; ♂].

**Distribution.** —Mexico.

***prolata*** Bueno-Soria & Holzenthal, 2003: 187 [type locality: Costa Rica, Alajuela, Reserva Forestal San Ramón, Río San Lorencito and tribs., 10.216°N, 84.607°W, 980 m; UMSP; ♂].

**Distribution.** —Costa Rica.

***prolixa*** Bueno-Soria, 2002: 233 [type locality: Mexico, Tabasco, Mpio. Huimanguillo, Villa de Guadalupe, 2^a^ sección los Chimalapas, km 5.92, Rta. Malpasito-Carlos A. Madraz, 17°22'05"N 93°36'25"W, 335 m; CNIN; ♂].

**Distribution.** —Mexico.

***protrudens*** (Flint, 1991b): 57 [type locality: Colombia, Dpto. Antioquia, 12 km N Fredonia, road to Medellin; NMNH; ♂; in Ochrotrichia (Metrichia)]. —Muñoz-Quesada 2000: 278 [checklist].

**Distribution.** —Colombia.

***pseudopatagonica*** Bueno-Soria & Holzenthal, 2003: 187 [type locality: Costa Rica, Limón, E.A.R.T.H., forest reserve arroyo, 7.5 km (air) NW Pocora, 10.23°N, 83.56°W, 10 m; UMSP; ♂]. —Bueno-Soria and Santiago-Fragoso 2002: 253 [distribution]. —Armitage et al. 2015a: 7 [checklist]. —Armitage and Cornejo 2015: 195 [checklist]. —Armitage and Harris 2018b: 97 [checklist].

**Distribution.** —Costa Rica, Panama.

***quadrata*** (Flint, 1972a): 14 [type locality: Mexico, Veracruz, Rio Jamapa, north of Coscomatepec; NMNH; ♂; in Ochrotrichia (Metrichia)]. —Bueno-Soria and Flint 1978: 204 [distribution]. —Bueno-Soria 2002: 241 [checklist]. —Bueno-Soria and Holzenthal 2003: 196 [distribution].

**Distribution.** —Costa Rica, Mexico.

***rafaeli*** Santos, Takiya, & Nessimian, 2016: 37 [type locality: Brazil, Ceará, Ubajara, Parque Nacional de Ubajara, Rio das Minas, 03°50'03"S 40°54'18"W, 524 m; DZRJ; ♂].

**Distribution.** —Brazil.

***rawlinsi*** (Flint & Sykora, 1993): 58 [type locality: Dominica, Parish St. Paul, Springfield Estate; CMNH; ♂; in Ochrotrichia (Metrichia)]. —Botosaneanu 2002b: 85 [checklist]. —Botosaneanu and Thomas 2005: 42 [distribution].

**Distribution.** —Dominica, Martinique.

***riva*** (Bueno-Soria, 1983b): 79 [type locality: Mexico, Chiapas, Cascada de Misolha, 20 km SE Palenque; CNIN; ♂; in Ochrotrichia (Metrichia)]. —Bueno-Soria 2002: 241 [checklist]. —Bueno-Soria and Holzenthal 2003: 196 [distribution]. —Bueno-Soria et al. 2005: 75 [distribution]. —Bueno-Soria and Barba-Álvarez 2011: 357 [checklist].

**Distribution.** —Costa Rica, Mexico.

***rona*** (Flint, 1991b): 55 [type locality: Colombia, Dpto. Antioquia, 7 km E. San Jerónimo; NMNH; ♂; in Ochrotrichia (Metrichia)]. —Muñoz-Quesada 2000: 278 [checklist].

**Distribution.** —Colombia.

***sacculifera*** (Flint, 1991b): 55 [type locality: Colombia, Dpto. Antioquia, Quebrada Honda, Marsella, 12 km SW Fredonia; NMNH; ♂; in Ochrotrichia (Metrichia)]. —Muñoz-Quesada 2000: 278 [checklist]. —Armitage et al. 2016: 8 [distribution]. —Armitage and Harris 2018b: 97 [checklist]. —Harris and Armitage 2019: 5, 20 [distribution; ♂].

**Distribution.** —Colombia, Panama.

***savegra*** Bueno-Soria & Holzenthal, 2003: 191 [type locality: Costa Rica, San José, Río Savegra, ca. Sasn Gerardo de Dota, 9.33°N, 83.48°W, 2200 m; CMNH; ♂]. —Armitage et al. 2016: 8 [distribution]. —Armitage and Harris 2018b: 97 [checklist].

**Distribution.** —Costa Rica, Panama.

***sencilla*** Harris & Armitage, 2015: 8 [type locality: Panama, Chiriquí Province, Cuenca 108, Quebrada Grande, Boquete, Valle Escondido, below Sabor Restaurant, 8.77970°N, 82.44016°W, 1122 m; MIUP; ♂]. —Armitage et al. 2015a: 7 [checklist]. —Armitage and Cornejo 2015: 196 [checklist]. —Armitage and Harris 2018b: 98 [checklist]. —Armitage and Harris 2018c: 283 [distribution].

**Distribution.** —Panama.

***separata*** Bueno-Soria & Holzenthal, 2003: 191 [type locality: Costa Rica, Alajuela, Río Agrio, ca. 3.5 km NE Bajos del Toro, 10.243°N, 84.279°W, 1290 m; UMSP; ♂]. —Armitage et al. 2016: 9 [distribution]. —Armitage and Harris 2018b: 98 [checklist]. —Armitage and Harris 2018c: 283 [distribution].

**Distribution.** —Costa Rica, Panama.

***sesquipedalis*** Bueno-Soria & Holzenthal, 2003: 191 [type locality: Costa Rica, San Jose, Río Savegra ca. San Gerardode Dota, 9.33°N, 83.48°W, 200 m; CMNH; ♂]. —Bueno-Soria and Santiago-Fragoso 2002: 253 [distribution]. —Armitage et al. 2015a: 7 [checklist]. —Armitage and Cornejo 2015: 196 [checklist]. —Armitage and Harris 2018b: 98 [checklist].

**Distribution.** —Costa Rica, Panama.

***similis*** (Flint, 1968a): 62 [type locality: Dominica, Boiling Lake; NMNH; ♂; in Ochrotrichia (Metrichia)]. —Flint and Sykora 1993: 50 [checklist]. —Botosaneanu 2002b: 85 [checklist]. —Botosaneanu and Thomas 2005: 55 [checklist].

**Distribution.** —Dominica, Guadeloupe.

***simples*** Santos, Takiya, & Nessimian, 2016: 38 [type locality: Brazil, Paraná, Céu Azul, Parque Nacional do Iguaçu, Rio Azul, 25°09'21"S 53°47'44"W, 510 m; DZRJ; ♂].

**Distribution.** —Brazil.

***sonora*** Bueno-Soria, 2002: 236 [type locality: Mexico, Sonora, Cajón Bonito, 38 miles E de A. P. Waters Falls; CAS; ♂].

**Distribution.** —Mexico.

***spica*** Bueno-Soria & Holzenthal, 2003: 195 [type locality: Costa Rica, Alajuela, Reserva Forestal San Ramón, Río San Lorencito and tribs., 10.216°N, 84.607°W, 980 m; UMSP; ♂]. —Armitage et al. 2016: 9 [distribution]. —Ríos-Touma et al. 2017: 10 [distribution]. —Armitage and Harris 2018b: 98 [checklist]. —Armitage and Harris 2018c: 283 [distribution].

**Distribution.** —Costa Rica, Ecuador, Panama.

***squamigera*** (Flint, 1992a): 385 [type locality: Puerto Rico, El Verde Field Station, Quebrada Prieta; NMNH; ♂; in Ochrotrichia (Metrichia)]. —Botosaneanu 2002b: 85 [checklist]. —Flint and Sykora 2004: 33 [distribution]. —Pérez-Gelabert 2008: 300 [checklist].

**Distribution.** —Dominican Republic, Puerto Rico.

***talhada*** Santos, Takiya, & Nessimian [type locality: Brazil, Alagoas Quebrangulo, Reserva Biológica de Pedra Talhada, Rio Caranguejo, 09°15'26"S 36°25'08"W, 550 m; DZRJ; ♂].

**Distribution.** —Brazil.

***temascalapensis*** Bueno-Soria & Barba-Álvarez, 1999b: 30 [type locality: Mexico, Guerrero, Taxco, Teucisapan, río Temascalapa, 12 km NW Ahuehuepan, Rd. 51, 18°25.26'N, 99°42.5'W, 1052 m; CNIN; ♂]. —Bueno-Soria 2002: 241 [checklist].

**Distribution.** —Mexico.

***tere*** Santos, Takiya, & Nessimian, 2016: 42 [type locality: Brazil, Rio de Janeiro, Teresópolis, Parque Nacional da Serra dos Órgãos, Rio Paquequer, 22°27'25"S 42°59'52"W, 1100 m; DZRJ; ♂].

**Distribution.** —Brazil.

***thirysae*** Jacquemart, 1980a: 303 [type locality: Chile, Arica, Vallee d’Azapa, Quebrada Azapa; IRSNB; ♂]. —Flint 1990: 117 [re-description; ♂]. —Angrisano 1999: 33 [checklist].

**Distribution.** —Chile.

***thomsonae*** Harris & Armitage, 2019: 10 [type locality: Panama, Bocas del Toro Province, Quebrada Rambala, near Rambala Jungle Lodge, 3.74 km SSE Rambala, 8.91627°N and 82.15469°W, 120 m; COZEM; ♂].

**Distribution.** —Panama.

***thurmani*** Harris & Armitage, 2019: 11 [type locality: Panama, Bocas del Toro Province, Quebrada Rambala, near Rambala Jungle Lodge, 3.74 km SSE Rambala, 8.91627°N and 82.15469°W, 120 m; COZEM; ♂]. —Armitage and Harris 2020a: 8 [distribution].

**Distribution.** —Panama.

***trebeki*** Harris & Armitage, 2019: 12 [type locality: Panama, Bocas del Toro Province, Quebrada Rambala, near Rambala Jungle Lodge, 3.74 km SSE Rambala, 8.91627°N and 82.15469°W, 120 m; COZEM; ♂].

**Distribution.** —Panama.

***triangula*** Bueno-Soria & Santiago-Fragoso, 2002: 246 [type locality: Panama, Barro Colorado; NMNH; ♂]. —Armitage et al. 2015a: 7 [checklist]. —Armitage and Cornejo 2015: 196 [checklist]. —Armitage and Harris 2018b: 98 [checklist].

**Distribution.** —Panama.

***trigonella*** (Flint, 1972a): 13 [type locality: Mexico, Veracruz, Fortin de las Flores; NMNH; ♂; in Ochrotrichia (Metrichia)]. —Bueno-Soria and Flint 1978: 204 [distribution]. —Bueno-Soria 2002: 241 [checklist].

**Distribution.** —Honduras, Mexico.

***triquetra*** Bueno-Soria & Holzenthal, 2003: 195 [type locality: Costa Rica, San José, Río Savegra, ca. San Gerardo de Dota, 9.33°N, 83.48°W, 2200 m; CMNH; ♂]. —Bueno-Soria and Santiago-Fragoso 2002: 253 [distribution]. —Armitage et al. 2015a: 7 [checklist]. —Armitage and Cornejo 2015: 196 [checklist]. —Armitage and Harris 2018b: 98 [checklist]. —Harris and Armitage 2019: 5 [distribution].

**Distribution.** —Costa Rica, Panama.

***trisignata*** Mey & Ospina-Torres, 2018: 28 [type locality: Colombia, Bogotá, Chapinero, Quebrada La Vieja; ICN; ♂].

**Distribution.** —Colombia.

***trispinosa*** (Bueno-Soria, 1977): 142 [type locality: Mexico, Veracruz, Eyipantla; CNIN; ♂; in Ochrotrichia (Metrichia)]. —Bueno-Soria 2002: 241 [checklist].

**Distribution.** —Mexico.

***truncata*** Bueno-Soria & Holzenthal, 2003: 195 [type locality: Costa Rica, Alajuela, Río Pizote, ca. 5 km (air) S Brasilia, 10.972°N, 84.345°W, 390 m; UMSP; ♂]. —Bueno-Soria and Santiago-Fragoso 2002: 253 [distribution].

**Distribution.** —Costa Rica.

***ubajara*** Santos, Takiya, & Nessimian, 2016: 43 [type locality: Brazil, Ceará, Ubajara, Parque Nacional de Ubajara, Rio das Minas, 03°49'58"S 40°53'53"W, 420 m; DZRJ; ♂].

**Distribution.** —Brazil.

***vulgaris*** Santos, Takiya, & Nessimian, 2016: 45 [type locality: Brazil, Rio de Janeiro, Itatiaia, Rio Palmital, 22°25'34"S 44°32'52"W, 637 m; DZRJ; ♂].

**Distribution.** —Brazil.

***warema*** (Flint, 1974b): 61 [type locality: Suriname, Litani River, Waremapan Rapids; RMNH; ♂; in Ochrotrichia (Metrichia)].

**Distribution.** —Suriname.

***yalla*** (Flint, 1968b): 50 [type locality: Jamaica, St. Andrew, Chestervale, Yallahs River; NMNH; ♂; in Ochrotrichi (Metrichia)]. —Flint 1968a: 82 [checklist]. —Botosaneanu 2002b: 85 [checklist].

**Distribution.** —Jamaica.

***yavesia*** Bueno-Soria, 2002: 239 [type locality: Mexico, Oaxaca, Santa María de Yavesia (Planta embotelladora de agua), 17°13'36"N 96°25'35"W, 1930 m; CNIN; ♂]. —Razo-González et al. 2020: 5 [distribution].

**Distribution.** —Mexico.

#### Genus *Nothotrichia* Flint, 1967

*Nothotrichia* Flint, 1967a: 56 [type species: *Nothotrichiailliesi* Flint, 1967a, original designation]. —Marshall 1979b: 219 [generic review; considered incertae sedis within Hydroptilidae]. —Harris and Armitage 1997: 123 [re-description; placement]. —Oláh and Johanson 2011: 203 [re-description]. —Parys and Harris 2013: 590 [larva; taxonomic remarks].

*Nothotrichia* contains six species recorded from California, Chile, Costa Rica, and Brazil. Marshall (1979b) was unable to place the genus in a subfamily. Similar to *Caledonotrichia*, Harris and Armitage (1997) added *Nothotrichia* to Ochrotrichiinae based upon phylogenetic assessments made by Kelley (1992) and their own specimen comparison, with the admission that they were still determining synapomorphies. Following a likely mistake in the Trichoptera World Checklist (Morse 2006), Holzenthal et al. (2007b) listed *Nothotrichia* as a member of Orthotrichiinae. Oláh and Johanson (2011) also placed *Nothotrichia* in Ochrotrichiinae, with no discussion or explanation of their analysis. The larva of *N.shasta* was described by Parys and Harris (2013).

***cautinensis*** Flint, 1983: 40 [type locality: Chile, Pcia. Cautín, Río Cautín, Cajón; NMNH; ♂]. —Harris and Armitage 1997: 125 [♂; ♀; re-description]. —Angrisano 1999: 33 [checklist]. —Oláh and Johanson 2011: 213 [distribution].

**Distribution.** —Chile.

***illiesi*** Flint, 1967a: 56 [type locality: Chile, Prov. Cautín, brook on Lago Villarica; NMNH; ♂]. —Flint 1974a: 87 [checklist]. —Harris and Armitage 1997: 124 [♂; ♀; re-description]. —Angrisano 1999: 33 [checklist].

**Distribution.** —Chile.

***munozi*** Holzenthal & Harris, 2002: 106 [type locality: Costa Rica, Guanacaste, Area de Conservación Guanacaste, Parque Nacional Guanacaste, Estación Maritza, Rio Tempisquito, 10.958°N, 85.497°W, 550 m; UMSP; ♂].

**Distribution.** —Costa Rica.

***panama*** Harris & Armitage, 2015: 11 [type locality: Panama, Chiriquí Province, Cuenca 108, Tributary of Quebrada Grande, at waterfall, Boquete, Valle Escondido, 8.78291°N, 82.44579°W, 1253 m; MIUP; ♂]. —Armitage et al. 2015a: 7 [checklist]. —Armitage and Harris 2018b: 98 [checklist]. —Armitage and Harris 2018c: 283 [distribution].

**Distribution.** —Panama.

***shasta*** Harris & Armitage, 1997: 126 [type locality: United States, California, Shasta County, Castle Creek, 0.2 km W Castle Crag State Park on Castle Creek Road; NMNH; ♂]. —Parys and Harris 2013: 589 [larva; distribution]. —Mendez et al. 2019: 118 [checklist].

**Distribution.** —U.S.A.

***tupi*** Holzenthal & Harris, 2002: 109 [type locality: Brazil, Minas Gerais, Parque Estadual Itacolomi, Rio Belchior, 20°25.041'S, 43°25.633'W, 725 m; MZUSP; ♂]. —Paprocki et al. 2004: 11 [checklist]. —Paprocki and França 2014: 49 [checklist].

**Distribution.** —Brazil.

#### Genus *Ochrotrichia* Mosely, 1934

*Polytrichia* Sibley, 1926: 102 [type species: *Ithytrichiaconfusa* Morton, 1905, monotypic; preoccupied].

*Ochrotrichia* Mosely, 1934a: 162 [type species: *Ochrotrichiainsularis* Mosely, 1934, original designation; although synonymized with *Polytrichia* by Mosely (1937b); Ross (1944) recognized *Polytrichia* as preoccupied and resurrected *Ochrotrichia*]. —Ross 1944: 126 [species keys to larvae and adults]. —Denning and Blickle 1972: 141 [review]. —Marshall 1979b: 185 [generic review]. —Blickle 1979: 26 [key to species of America north of Mexico]. —Vinikour 1982: 150 [phoretic association observed]. —Frazer and Harris 1991a: 363 [phylogenetic analysis of *Ochrotrichiashawnee* group]. —Moulton and Stewart 1996: 115 [key to species of the Interior Highlands of North America]. —Bueno-Soria 2009: 60 [revision]. —Oláh and Johanson 2011: 213 [re-description].

The genus *Ochrotrichia* currently contains 226 species, including five fossil species known from Dominican amber. Extant species occur throughout North, Central, and South America. Flint (1972a) attempted to divide the genus into species groups, but as species continued to be added, the group definitions proved too weak to be upheld. *Ochrotrichia* is probably closely related to *Metrichia*, but males of the former often have much more complicated genitalic structures (Marshall 1979b). Larvae have been associated and described for several species (Ross 1944; Roldán-Perez 1988; Wiggins 1996; Keiper and Harris 2002). The pupae of a species from Costa Rica were recorded as being parasitized by a ceraphronid wasp (Luhman et al. 1999).

***affinis*** Bueno-Soria & Holzenthal, 2004: 251 [type locality: Mexico, Tabasco, Municipio de Huimanguillo, Ejido Villa de Guadaloupe, 1^a^ sección Cascada Cerro de Las Flores, 17°21'39"N 93°37'29"W, 540 m; CNIN; ♂]. —Bueno-Soria 2009: 133 [♂].

**Distribution.** —Mexico.

***alargada*** Bueno-Soria & Holzenthal, 2004: 246 [type locality: Mexico, Guerrero, Municipio de Taxco: Teusisapan, Rio Temazcalapa, 12 km. NW Ahuehuepan, Rta 51, 18°25.26'N, 99°42.5'W, 1052 m; CNIN; ♂]. —Bueno-Soria 2009: 112 [♂].

**Distribution.** —Mexico.

***aldama*** (Mosely, 1937b): 185 [type locality: Mexico, Chiapas, Esmeralda; NHMUK; ♂; in *Polytrichia*]. —Blickle and Denning 1977: 289 [checklist]. —Bueno-Soria and Flint 1978: 203 [distribution]. —Wells and Wichard 1989: 46 [in Dominican amber]. —Flint and Pérez-Gelabert 1999: 40 [checklist]. —Botosaneanu 2002b: 86 [checklist]. —Bueno-Soria and Holzenthal 2004: 246 [distribution]. —Bueno-Soria and Holzenthal 2008: 48 [distribution]. —Wichard 2007: 48 [checklist; in amber]. —Eskov et al. 2008: 78 [checklist]. —Pérez-Gelabert 2008: 300 [checklist]. —Bueno-Soria 2009: 110 [♂; distribution]. —Armitage et al. 2015a: 7 [checklist]. —Armitage and Harris 2018b: 98 [checklist]. —Mendez et al. 2019: 128 [checklist].

**Distribution.** —Costa Rica, Dominican Republic (in amber), Mexico, Panama.

***alexanderi*** Denning & Blickle, 1972: 145 [type locality: [United States], Humboldt County, California, Bear River; CAS; ♂; specimen damaged]. —Blickle and Denning 1977: 287 [checklist]. —Blickle 1979: 51, 87 [checklist; ♂]. —Mendez et al. 2019: 118 [checklist].

**Distribution.** —U.S.A.

♰ ***aliceae*** Wichard, 2000: 242 [type locality: Dominican Republic; NMNH; in amber]. —Wichard 2007: 48 [checklist]. —Eskov et al. 2008: 78 [checklist]. —Pérez-Gelabert 2008: 300 [checklist].

**Distribution.** —Dominican amber.

***alsea*** Denning & Blickle, 1972: 143 [type locality: [United States], Tumalo State Park, Deschutes County, Oregon; CAS; ♂]. —Blickle and Denning 1977: 287 [checklist]. —Blickle 1979: 51, 85 [checklist; ♂]. —Ruiter 1999: 165 [distribution]. —Blinn and Ruiter 2013: 291 [biology; distribution]. —Ruiter and Harrris 2015: 328 [♂; distribution]. —Mendez et al. 2019: 118 [checklist].

**Distribution.** —U.S.A.

***amorfa*** Bueno-Soria & Holzenthal, 2004: 252 [type locality: Mexico, Tabasco, Municipio de Huimanguillo, Arroyo las Flores, Villa de Guadalupe, 2^a^ sección, Los Chimalapas km 5 Ruta Malpasito-Carlos A. Madrazo, 17°nn'05"N 93°36'25"W, 540 m; CNIN; ♂]. —Bueno-Soria 2009: 133 [♂].

**Distribution.** —Mexico.

***angularis*** Bueno-Soria, 2009: 131 [type locality: Mexico, Morelos, Huautla, Reserva de la Biosfera de Huautla, 18°20'10"N 98°51'20"W, 900 m; CNIN; ♂].

**Distribution.** —Mexico.

***anisca*** (Ross, 1941a): 58 [type locality: [United States], Illinois, Wolf Lake; INHS; ♂; in *Polytrichia*]. —Ross 1944: 131 [♂; ♀; distribution]. —Unzicker et al. 1970: 172 [distribution]. —Edwards 1973: 506 [distribution]. —Blickle and Denning 1977: 287 [checklist]. —Blickle 1979: 51, 79 [checklist; ♂]. —Hamilton et al. 1983: 18 [distribution]. —Bowles and Mathis 1989: 239 [distribution]. —Frazer and Harris 1991a: 366–367 [♂]. —Mathis and Bowles 1992: 24 [distribution]. —Bowles and Mathis 1992: 32 [distribution]. —Moulton and Stewart 1996: 116 [♂; checklist]. —Moulton and Stewart 1997: 350 [checklist]. —Bowles et al. 2020: 8 [distribution].

**Distribution.** —U.S.A.

***anomala*** Bueno-Soria & Santiago-Fragoso, 1997: 365 [type locality: Panama, Barro Colorado Island, Snyder-Molino Trail, Marker 3; NMNH; ♂]. —Bueno-Soria 2009: 68 [♂]. —Armitage et al. 2015a: 7 [checklist]. —Armitage and Harris 2018b: 98 [checklist]. —Armitage and Harris 2018c: 283 [distribution]. —Harris and Armitage 2019: 5 [distribution].

**Distribution.** —Panama.

***apalachicola*** Harris, Pescador, & Rasmussen, 1998: 224 [type locality: [United States], Florida, Liberty County, Nature Conservancy Apalachicola Bluffs and Ravines Preserve, Beaver Dam Creek; NMNH; ♂]. —Pescador et al. 2004: 133 [checklist]. —Harris et al. 2012: 9 [checklist].

**Distribution.** —U.S.A.

***argentea*** Flint & Blickle in Denning and Blickle 1972: 150 [type locality: United States N[ew] Mex[ico], Near Silver City, Cherry Creek Rec. Area; NMNH; ♂]. —Blickle and Denning 1977: 287 [checklist]. —Blickle 1979: 51, 83 [checklist; ♂]. —Blinn and Ruiter 2005: 69 [distribution; biology]. —Bueno-Soria 2009: 147 [♂]. —Vieira et al. 2009: 257 [distribution]. —Ruiter and Harrris 2015: 3230 [♂; distribution].

**Distribution.** —Mexico, U.S.A.

***arizonica*** Denning & Blickle, 1972: 145 [type locality: [United States], Southwest Research Station, 7 miles west of Portal, Arizona, Chiricahua Mountains, 5400 ft; CAS; ♂]. —Blickle and Denning 1977: 287 [checklist]. —Blickle 1979: 51, 79 [checklist; ♂]. —English and Hamilton 1986: 475 [larva; ♂; distribution]. —Moulton and Stewart 1997: 350 [checklist]. —Blinn and Ruiter 2005: 69 [distribution; biology]. —Mendez et al.,2019: 118 [checklist].

**Distribution.** —U.S.A.

***arranca*** (Mosely, 1937b): 185 [type locality: Mexico, Chiapas, Barranca Honda NHMUK; ♂; in *Polytrichia*]. —Flint 1972a: 7 [re-description; ♂]. —Blickle and Denning 1977: 289 [checklist]. —Bueno-Soria and Flint 1978: 203 [distribution]. —Rojas-Ascensio et al. 2002: 377 [distribution]. —Bueno-Soria et al. 2005: 75 [distribution]. —Bueno-Soria and Holzenthal 2008: 48 [distribution]. —Bueno-Soria 2009: 122 [♂]. —Armitage et al. 2016: 10 [distribution]. —Armitage and Harris 2018b: 98 [checklist]. —Armitage and Harris 2018c: 283 [distribution].

**Distribution.** —Costa Rica, Mexico, Panama.

***arriba*** Bueno-Soria & Santiago-Fragoso, 1997: 361 [type locality: Panama, Chiriqui, Guadalupe Arriba, 8°52'26"N 82°33'13"W; NMNH; ♂]. —Bueno-Soria 2009: 114 [♂]. —Armitage et al. 2015a: 7 [checklist]. —Armitage and Harris 2018b: 98 [checklist].

**Distribution.** —Panama.

***arva*** (Ross, 1941a): 58 [type locality: [United States], Tennessee, Martin Springs; INHS; ♂; in *Polytrichia*]. —Blickle and Denning 1977: 288 [checklist]. —Etnier and Schuster 1979: 18 [distribution]. —Blickle 1979: 51, 87 [checklist; ♂]. —Parker and Voshell 1981: 4 [checklist]. —Huryn and Foote 1983: 790 [distribution]. —Harris et al. 1991: 226 [distribution]. —Mathis and Bowles 1992: 24 [distribution]. —Moulton and Stewart 1996: 117 [♂; distribution]. —Houp 1999: 2 [distribution]. —Armitage et al. 2011: 14 [checklist]. —Houghton et al. 2017: 63 [checklist]. —Bowles et al. 2020: 8 [distribution].

**Distribution.** —U.S.A.

***assita*** Bueno-Soria & Holzenthal, 2004: 251 [type locality: Panama, Chiriqui, Fortuna Dam site near Hornitos, 8°55'N 82°16'W, 1050 m; NMNH; ♂]. —Bueno-Soria and Holzenthal 2008: 48 [distribution]. —Bueno-Soria 2009: 114 [♂]. —Armitage et al. 2015a: 7 [checklist]. —Armitage and Harris 2018b: 98 [checklist].

**Distribution.** —Costa Rica, Panama.

***atezcae*** Bueno-Soria & Santiago-Fragoso, 1981: 384 [type locality: Mexico, Hidalgo, Laguna de Atezca, 3 km de Molango; CNIN; ♂]. —Bueno-Soria 2009: 132 [♂].

**Distribution.** —Mexico.

***attenuata*** Flint, 1972a: 11 [type locality: Guatemala, Huehuetenango, 32 km NW Huehuetenango; NMNH; ♂]. —Blickle and Denning 1977: 289 [checklist]. —Bueno-Soria 2009: 154 [♂].

**Distribution.** —Guatemala.

***avicula*** Bueno-Soria & Holzenthal, 2008: 42 [type locality: Costa Rica, Puntarenas, Río Jaba at rock quarry, 1.4 km (air) W Las Cruces, 8.79°N, 82.97°W, 1150 m; UMSP; ♂].

**Distribution.** —Costa Rica.

***avis*** Bueno-Soria & Holzenthal, 1998: 606 [type locality: Costa Rica, Alajuela, Reserva Forestal San Rámon, Río San Lorencito and tribs, 10°12'96"N, 84°36'42"W; NMNH; ♂]. —Bueno-Soria and Holzenthal 2008: 48 [distribution]. —Bueno-Soria 2009: 115 [♂]. —Armitage et al. 2016: 10 [distribution]. —Armitage and Harris 2018b: 98 [checklist].

**Distribution.** —Costa Rica, Panama.

***ayaya*** Botosaneanu, 1977: 260 [type locality: Cuba, Oriente, Baracoa, Rio Sabanilla; NMNH; ♂; as subspecies of *O.insularis*]. —Botosaneanu 1979: 49 [distribution]. —Flint 1996a: 16 [checklist]. —Botosaneanu in Botosaneanu and Hyslop 1998: 13 [as distinct species]. —Botosaneanu 2002b: 86 [checklist]. —Naranjo López and González Lazo 2005: 149 [checklist].

**Distribution.** —Cuba.

***balra*** Oláh & Johanson, 2011: 214 [type locality: French Guiana, Approuaguekaw, Kaw Mt, 4°33.035'N, 52°11.661'W, 104 m; NHRS; ♂].

**Distribution.** —French Guiana.

***baorucoensis*** Flint & Sykora, 2004: 36 [type locality: Dominican Republic, Barahoma Province, San Rafael, 8.3 km S of Baoruco, 18°01.9'N, 71°8.4'W, 30 m; NMNH; ♂]. —Pérez-Gelabert 2008: 300 [checklist].

**Distribution.** —Dominican Republic.

***bicaudata*** Bueno-Soria & Santiago-Fragoso, 1997: 367 [type locality: Panama, Barro Colorado Island, Snyder-Molino Trail, Marker 3; NMNH; ♂]. —Bueno-Soria 2009: 115 [♂]. —Armitage et al. 2015a: 7 [checklist]. —Armitage and Harris 2018b: 98 [checklist].

**Distribution.** —Panama.

***bickfordae*** Ruiter, 2011: 421 [type locality: USA, California, Fresno County, Little Dry Creek, Marshall Station; CAS; ♂]. —Mendez et al. 2019: 118 [checklist].

**Distribution.** —U.S.A.

***bipartita*** Flint & Bueno-Soria, 1999: 732 [type locality: Peru, Department Cuzco, Province Paucartambo, stream, 50 m E Quitacalzón; MHNJP; ♂; ♀].

**Distribution.** —Peru.

***birdae*** Harris & Armitage, 2019: 15 [type locality: Panama, Bocas del Toro Province, Quebrada Rambala, near Rambala Jungle Lodge, 3.74 km SSE Rambala, 8.91627°N and 82.15469°W, 120 m; COZEM; ♂]. —Armitage and Harris 2020a: 8 [distribution].

**Distribution.** —Panama.

***blanca*** Bueno-Soria & Santiago-Fragoso, 1997: 363 [type locality: Belize, Cayo District, Rio Privassion, Blancaneaux Lodge; NMNH; ♂]. —Bueno-Soria 2009: 129 [♂].

**Distribution.** —Belize.

***bogani*** Ruiter, 2011: 422 [type locality: USA, California, Fresno County, Little Dry Creek, Marshall Station; CAS; ♂]. —Mendez et al. 2019: 118 [checklist].

**Distribution.** —U.S.A.

***boquillas*** Moulton & Harris, 1997: 496 [type locality: United States, Texas, Brewster Co, Glenn Spring, Big Bend National Park; NMNH; ♂]. —Baumgardner and Bowles 2005: 11 [distribution]. —Bowles et al. 2007: 22 [distribution; biology]. —Bueno-Soria et al. 2007: 33 [distribution]. —Armitage et al. 2020: 4 [distribution].

**Distribution.** —Mexico, Panama, U.S.A.

***boydi*** Ruiter & Harris, 2015: 320 [type locality: [United States], California, River County, P. L. Boyd Desert Research Center, 3.5 mi S Palm Desert, Marker #(-)9, beating fan palm *Washingtoniafilifera*) Linden ex André) H. Wendland ex A. de Bary (Arecaceae); EMEC; ♂]. —Mendez et al. 2019: 118 [checklist].

**Distribution.** —U.S.A.

***bractea*** Bueno-Soria & Holzenthal, 2004: 252 [type locality: Mexico, Morelos, Municipio de Huautla, Reserva de la Biosfera de Huautla, 18°20'10"–18°34'20"N 98°51'20"–99°08'15"W, 900 m; CNIN; ♂]. —Bueno-Soria 2009: 130 [♂].

**Distribution.** —Mexico.

***brayi*** Flint, 1968a: 61 [type locality: Dominica, Freshwater Lake; NMNH; ♂]. —Blickle and Denning 1977: 289 [checklist]. —Flint and Sykora 1993: 50 [checklist]. —Botosaneanu 2000: 250 [♀]. —Botosaneanu 2002b: 86 [checklist]. —Botosaneanu and Thomas 2005: 55 [checklist].

**Distribution.** —Dominica, Guadaloupe.

♰ ***brodzinskyi*** Wells & Wichard, 1989: 45 [type locality: Dominican Republic; Collection Wichard; ♂; in amber]. —Flint and Pérez-Gelabert 1999: 40 [checklist]. —Botosaneanu 2002b: 86 [checklist]. —Wichard 2007: 48 [checklist]. —Eskov et al. 2008: 78 [checklist]. —Pérez-Gelabert 2008: 300 [checklist].

**Distribution.** —Dominican amber.

***buccata*** Denning & Blickle, 1972: 147 [type locality: [United States], Burney Falls, Shasta County, California; CAS; ♂; specimen damaged]. —Blickle and Denning 1977: 288 [checklist]. —Blickle, 1979: 51, 87 [checklist; ♂]. —Mendez et al. 2019: 118 [checklist].

**Distribution.** —U.S.A.

***buenoi*** Razo-González, 2018: 29 [type locality: Mexico, Oaxaca, Santa Catarina Lachatao, Las Vigas, 17°10'43"N, 96°26'51"W, 2689 m asl; CNIN; ♂]. —Razo-González et al. 2020: 5 [distribution].

**Distribution.** —Mexico.

***burdicki*** Denning, 1989: 129 [type locality: [United States], California, Fresno County, Dry Creek; CAS; ♂; ♀]. —Mendez et al. 2019: 118 [checklist].

**Distribution.** —U.S.A.

***caatinga*** de Souza, Santos & Takiya, 2014a: 274 [type locality: Brazil, Ceará, Ubajara, Parque Nacional de Ubajara, Rio Cafundó, acima da cachoeira, 3°50'13"S 40°54'19"W, 874 m; CZMA; ♂]. —Paprocki and França 2014: 50 [checklist]. —Cavalcante et al. 2018: 235 [distribution]. —Moreno et al. 2020: 265 [distribution].

**Distribution.** —Brazil.

***cachonera*** Botosaneanu, 1995a: 23 [type locality: Dominican Republic, springs near La Descubierta, S of Sierra de Neiba; ZMUA; ♂]. —Botosaneanu 2002b: 86 [checklist]. —Flint and Pérez-Gelabert 1999: 40 [checklist]. —Flint and Sykora 2004: 36 [distribution]. —Pérez-Gelabert 2008: 300 [checklist].

**Distribution.** —Dominican Republic.

***caimita*** Flint, 1972a: 6 [type locality: Panama, Chiriqui, Rio Caimito, 16 km NW David; NMNH; ♂]. —Blickle and Denning 1977: 289 [checklist]. —Aguila 1992: 538 [distribution]. —Bueno-Soria and Holzenthal 2008: 49 [distribution]. —Bueno-Soria 2009: 134 [♂]. —Armitage et al. 2015a: 7 [checklist]. —Armitage and Harris 2018b: 98 [checklist]. —Harris and Armitage 2019: 5 [distribution; as *caimata*].

**Distribution.** —Costa Rica, Panama.

***calcarata*** Flint & Bueno-Soria, 1999: 733 [type locality: Peru, Department Cuzco, Province Paucartambo, Puente Ssan Pedro at km 152, 44 km (road) W of Pilcopata, 13°03.30'S, 71°32.78'W; MHNJP; ♂].

—Ochrotrichia (O.) n. sp. 3: Flint 1996c: 398. —Flint and Bueno-Soria 1999: 733 [to synonymy].

**Distribution.** —Peru.

***caligula*** Flint, 1968b: 49 [type locality: Jamaica, St. Andrew, Hope River near Newcastle at mile post 16.5; NMNH; ♂]. —Flint 1968a: 82 [checklist]. —Blickle and Denning 1977: 289 [checklist]. —Botosaneanu 2002b: 86 [checklist].

**Distribution.** —Jamaica.

***campanilla*** Flint & Bueno-Soria, 1999: 735 [type locality: Peru, Department Madre de Dios, Province Manu, Pakitza, trail 1, 1^st^ stream; MHNJP; ♂].

—Ochrotrichia (O.) n. sp. 4: Flint 1996c: 398. —Flint and Bueno-Soria 1999: 735 [to synonymy].

**Distribution.** —Peru.

***canicula*** Bueno-Soria, 2009: 148 [type locality: Mexico, Estado de Mexico, Tetesontle, 19°05'37"N 98°36'22"N, 3350 m; CNIN; ♂].

**Distribution.** —Mexico.

***capitana*** Ross, 1944: 275 [type locality: [United States], Texas, McKittrick Creek, McKittrick Canyon (near Frijole); INHS; ♂]. —Edwards 1973: 506 [distribution]. —Blickle and Denning 1977: 288 [checklist]. —Blickle 1979: 51, 87 [checklist; ♂]. —Moulton et al. 1993: 21 [distribution]. —Moulton and Stewart 1997: 350 [checklist]. —Bowles et al. 2007: 22 [distribution; biology].

**Distribution.** —U.S.A.

***caramba*** Botosaneanu, 1977: 262 [type locality: Cuba, Oriente, Gran Piedra, Arroyos de la Idalia; NMNH; ♂]. —Botosaneanu 1979: 48 [distribution]. —Flint 1996a: 16 [checklist]. —Botosaneanu 2002b: 86 [checklist]. —Naranjo López and González Lazo 2005: 149 [checklist].

**Distribution.** —Cuba.

***catarina*** Bueno-Soria & Holzenthal, 2004: 249 [type locality: Mexico, Oaxaca, Santa Catarina La Chatao, 17°15'58"N 96°28'15"W, 2160 m; CNIN; ♂]. —Bueno-Soria 2009: 116 [♂].

**Distribution.** —Mexico.

***cavitectum*** Botosaneanu in Botosaneanu and Hyslop 1998: 13 [type locality: Jamaica, St. Ann, Roaring River W. from Ocho Rios; ZMUA; ♂]. —Botosaneanu 2002b: 86 [checklist].

**Distribution.** —Jamaica.

***cebollati*** Angrisano, 1995a: 509 [type locality: Uruguay, Lavalleja, Río Cebollatí, Picada de Rodriguez; FHCU; ♂; type of subgenus Paratrichia of *Ochrotrichia*].

**Distribution.** —Uruguay.

♰ ***chaulioda*** Wells & Wichard, 1989: 46 [type locality: Dominican Republic; NMNH; ♂; in amber]. —Flint and Pérez-Gelabert 1999: 40 [checklist]. —Botosaneanu 2002b: 86 [checklist]. —Wichard 2007: 48 [checklist]. —Eskov et al. 2008: 78 [checklist]. —Pérez-Gelabert 2008: 300 [checklist].

**Distribution.** —Dominican amber.

***chiapa*** Denning & Blickle, 1972: 147 [type locality: Mexico, Chiapas, 6 miles south of Puebla Nueva; CAS; ♂]. —Blickle and Denning 1977: 289 [checklist]. —Bueno-Soria and Flint 1978: 203 [distribution]. —Bueno-Soria 2009: 148 [♂].

**Distribution.** —Mexico.

***cieneguilla*** Harris in Harris and Moulton 1993: 545 [type locality: Mexico, Nuevo León, Municipio de Santiago, Cola de Caballo, down stream falls, 3 km SW Cieneguilla NMNH; ♂]. —Bueno-Soria 2009: 123 [♂].

**Distribution.** —Mexico.

***citra*** Bueno-Soria & Holzenthal, 2004: 247 [type locality: Mexico, Tabasco, Municipio de Huimanguilla Rta. Malpasito-Carlos A. Madrazo, Ejido Villa de Guadalupe, 1a sección Cascada Cerro de las Flores, 17°21'39"N 93°37'29"W, 540 m; CNIN; ♂]. —Bueno-Soria 2009: 117 [♂].

**Distribution.** —Mexico.

***cochisei*** Ruiter & Harris, 2015: 321 [type locality: [United States], Arizona, Cochise County, Portal; EMEC; ♂].

**Distribution.** —U.S.A.

***compacta*** Bueno-Soria & Holzenthal, 2004: 247 [type locality: Mexico, Tabasco, Municipio de Huimanguillo Rta. Malpasito-Carlos A. Madrazo, Ejido Villa de Guadalupe, 1a sección Cascada Cerro de las Flores, 17°21'39"N 93°37'29"W, 540 m; CNIN; ♂]. —Bueno-Soria 2009: 117 [♂].

**Distribution.** —Mexico.

***concha*** Bueno-Soria & Santiago-Fragoso, 1992: 443 [type locality: Brazil, Amazonas State, AM010, km 246, ca. 20 km W Itacoatiara; NMNH; ♂]. —Angrisano 1999: 34 [checklist]. —Paprocki et al. 2004: 11 [checklist]. —Paprocki and França 2014: 50 [checklist].

**Distribution.** —Brazil.

***conformalis*** Bueno-Soria & Holzenthal, 2008: 45 [type locality: Costa Rica, Alajuela, Reserva Forestal San Ramón Río San Lorencito and tribs., 10.216°N, 84.607°W, 980 m; UMSP; ♂]. —Armitage et al. 2020: 5 [distribution].

**Distribution.** —Costa Rica, Panama.

***confusa*** (Morton, 1905): 69 [type locality: United States, New York, Ithaca; depository not designated; ♂; as *Ithytrichia*]. —Banks 1907a: 50 [catalogue]. —Lloyd 1915: 117 [larva; habitat]. —Betten 1934: 154 [♂; distribution; in *Polytrichia*]. —Ross 1944: 133 [larva; to *Ochrotrichia*]. —Denning and Blickle 1972: 142 [checklist]. —Blickle and Denning 1977: 288 [checklist]. —Etnier and Schuster 1979: 18 [distribution]. —Blickle 1979: 51, 81 [checklist; ♂]. —Vaillant 1984: 407 [larva; biology]. —Sinclair and Marshall 1986: 10 [distribution]. —Morse et al.: 23 [distribution]. —Sinclair 1990: 284 [distribution]. —Harris et al. 1991: 227 [distribution]. —Masteller and Flint 1992: 70 [checklist]. —Moulton and Stewart 1997: 350 [checklist]. —Pescador et al. 2004: 133 [checklist]. —Bueno-Soria et al. 2007: 33 [distribution]. —Bueno-Soria 2009: 124 [♂]. —Armitage et al. 2011: 14 [checklist]. —Harris et al. 2012: 9 [checklist]. —Ruiter and Harrris 2015: 330 [♂; distribution]. —Mendez et al. 2019: 118 [checklist].

**Distribution.** —Canada, Mexico, U.S.A.

***constricta*** de Souza, Santos, & Takiya, 2014a: 278 [type locality: Brazil, Irapiúna, Reserva Ecológica Michelin, Mata da Vila 5, 13°49'22.9"S 39°12'6.5"W, 87 m; DZRJ; ♂]. —Paprocki and França 2014: 50 [checklist].

**Distribution.** —Brazil.

***contorta*** (Ross, 1941a): 60 [type locality: [United States], Missouri, Greet Spring; INHS; ♂; in *Polytrichia*]. —Blickle and Denning 1977: 288 [checklist]. —Blickle 1979: 51, 79 [checklist; ♂]. —Frazer and Harris 1991a: 366–367 [♂]. —Mathis and Bowles 1992: 24 [distribution]. —Moulton and Stewart 1996: 117 [♂; distribution]. —Bowles et al. 2020: 8 [distribution].

**Distribution.** —U.S.A.

***contrerasi*** Harris in Harris and Moulton 1993: 545 [type locality: Mexico, Tamaulipas, Municipio de Gómez Farias, Río Frio at La Poza Azul, 6 km S Gómez Farias; NMNH; ♂]. —Bueno-Soria 2009: 149 [♂].

**Distribution.** —Mexico.

***corneolus*** Bueno-Soria & Santiago-Fragoso, 1997: 365 [type locality: Panama, Barro Colorado Island, Snyder-Molino Trail, Marker 3; NMNH; ♂]. —Bueno-Soria 2009: 135 [♂]. —Armitage et al. 2015a: 7 [checklist]. —Armitage and Harris 2018b: 98 [checklist].

**Distribution.** —Panama.

***crucecita*** Bueno-Soria & Santiago-Fragoso, 1997: 360 [type locality: Panama, Chiriqui, Guadalupe Arriba, 8°52'26"N 82°33'13"W; NMNH; ♂]. —Bueno-Soria 2009: 144 [♂]. —Armitage et al. 2015a: 7 [checklist]. —Armitage and Harris 2018b: 98 [checklist].

**Distribution.** —Panama.

***cruces*** Flint, 1967b: 12 [type locality: Mexico, Las Cruces National Park, La Marquesa; NMNH; ♂]. —Blickle and Denning 1977: 289 [checklist]. —Bueno-Soria and Flint 1978: 203 [distribution]. —Bueno-Soria 2009: 145 [♂].

**Distribution.** —Mexico.

***csiga*** Oláh & Johanson, 2011: 215 [type locality: Peru, San Martin Prov., creek crossing rd. Juan Guerra-Chazuta, 14 km (rd.) E Colombia Bridge, 6°35.594'S, 76°13.172'W; NHRS; ♂].

**Distribution.** —Peru.

***curvata*** Bueno-Soria & Holzenthal, 2004: 250 [type locality: Panama, Chiriqui: Fortuna Dam site near Hornitos, 8°55'N 82°16'W, 1050 m; NMNH; ♂]. —Bueno-Soria 2009: 135 [♂]. —Armitage et al. 2015a: 7 [checklist]. —Armitage and Harris 2018b: 98 [checklist].

**Distribution.** —Panama.

***cuspidatus*** Bueno-Soria & Holzenthal, 2004: 254 [type locality: Mexico, Guerrero, Municipio Taxco, Teusisapan, Río Temascalapa, 12 km. NW Ahuehuepan Rta. 51, 18°25.56'N, 99°42.5'W, 1052 m; CNIN; ♂]. —Bueno-Soria 2009: 149 [♂].

**Distribution.** —Mexico.

***dactylophora*** Flint, 1965: 171 [type locality: United States, Arizona, Coconino County, West Fork, 16 miles southwest of Flagstaff, 6500 ft; NMNH; ♂]. —Denning and Blickle 1972: 142 [checklist]. —Blickle and Denning 1977: 288 [checklist]. —Blickle 1979: 51, 85 [checklist; ♂]. —Moulton et al. 1994: 170 [distribution]. —Moulton and Stewart 1997: 350 [checklist]. —Houghton 2001: 90 [distribution]. —Baumgardner and Bowles 2005: 11 [distribution]. —Blinn and Ruiter 2005: 69 [distribution; biology]. —Blinn and Ruiter 2006: 333 [biology; distribution]. —Bueno-Soria et al. 2007: 33 [distribution]. —Blinn and Ruiter 2009a: 303 [biology]. —Blinn and Ruiter 2009b: 186 [phenology; distribution]. —Bueno-Soria 2009: 125 [♂; distribution].

**Distribution.** —Mexico, U.S.A.

***dardeni*** Harris, 1986a: 613 [type locality: [United States], Alabama, Wilcox County, Chilatchee Creek at Hwy. 5; NMNH; ♂]. —Tarter 1990: 239 [checklist]. —Harris et al. 1991: 228 [distribution].

**Distribution.** —U.S.A.

***delgada*** Bueno-Soria & Holzenthal, 2004: 253 [type locality: Mexico, Tabasco, Municipio de Huimanguillo, km 5 Ruta Malpasito-Carlos A. Madrazo, Arroyo Las Flores Villa de Guadalupe, 2^a^ sección Los Chimalapas, 17°22'05"N 93°36'25"W, 545 m; CNIN; ♂]. —Bueno-Soria 2009: 150 [♂].

**Distribution.** —Mexico.

♰ ***denaia*** Wells & Wichard, 1989: 47 [type locality: Dominican Republic; NMNH; ♂; in amber]. —Flint and Pérez-Gelabert 1999: 40 [checklist]. —Botosaneanu 2002b: 86 [checklist]. —Wichard 2007: 48 [checklist]. —Eskov et al. 2008: 78 [checklist]. —Pérez-Gelabert 2008: 301 [checklist].

**Distribution.** —Dominican amber.

***denningi*** Blickle & Morse, 1957: 50 [type locality: [United States], Plymouth, N.H. [New Hampshire]; INHS; ♂]. —Blickle and Denning 1977: 288 [checklist]. —Blickle 1979: 51, 79 [checklist; ♂]. —Tarter 1990: 239 [checklist]. —Frazer and Harris 1991a: 366–367 [♂]. —Masteller and Flint 1992: 70 [checklist].

**Distribution.** —U.S.A.

***dewalti*** Harris & Armitage, 2019: 15 [type locality: Panama, Bocas del Toro Province, Quebrada Rambala, near Rambala Jungle Lodge, 3.74 km SSE Rambala, 8.91627°N and 82.15469°W, 120 m; COZEM; ♂].

**Distribution.** —Panama.

♰ ***doehleri*** Wichard, 1981: 161 [type locality: Dominican Republic; Collection Wichard; ♂; in amber]. —Wells and Wichard 1989: 48 [♂; re-description]. —Flint and Pérez-Gelabert 1999: 40 [checklist]. —Botosaneanu 2002b: 86 [checklist]. —Wichard 2007: 48 [checklist]. —Pérez-Gelabert 2008: 301 [checklist]. —Eskov et al. 2008: 78 [checklist].

**Distribution.** —Dominican amber.

***dulce*** Bueno-Soria & Holzenthal, 1998: 608 [type locality: Costa Rica, Guanacaste, Río Tizate, 7.2 km N.E. Cañas Dulces, 10°43'98"N 66°[sic, should be 85°]26'94"W; NMNH; ♂]. —Bueno-Soria and Holzenthal 2008: 49 [distribution]. —Bueno-Soria 2009: 140 [♂].

**Distribution.** —Costa Rica.

***ecuatoriana*** Bueno-Soria & Santiago-Fragoso, 1992: 440 [type locality: Ecuador, Pastaza Prov., Puyo; NMNH; ♂]. —Muñoz-Quesada 2000: 278 [checklist]. —Oláh and Johanson 2011: 218 [distribution]. —Ríos-Touma et al. 2017: 10 [checklist].

**Distribution.** —Colombia, Ecuador, Peru.

***eliaga*** (Ross, 1941a): 57 [type locality: [United States], Tennessee, Jasper; INHS; ♂; in *Polytrichia*]. —Ross 1944: 132 [♂; ♀; distribution]. —Blickle and Denning 1977: 288 [checklist]. —Etnier and Schuster 1979: 18 [distribution]. —Blickle 1979: 51, 85 [checklist; ♂]. —Waltz and McCafferty 1983a: 10 [distribution]. —Harris et al. 1991: 229 [distribution]. —Mathis and Bowles 1992: 24 [distribution]. —Bowles and Mathis 1992: 32 [distribution]. —Moulton and Stewart 1996: 117 [♂; distribution]. —Houp 1999: 2 [distribution]. —Bowles et al. 2020: 8 [distribution].

**Distribution.** —U.S.A.

***elongiralla*** Harris, 1986b: 32 [type locality: [United States], Alabama, Tuscaloosa County, unnamed tributary to Wallace Branch, 5 mile southeast Berry (R10W, T17S, S2); NMNH; ♂]. —Harris et al. 1991: 230 [distribution].

**Distribution.** —U.S.A.

***escalantea*** Ruiter & Harris, 2015: 322 [type locality: [United States], Utah, Garfield County, Death Hollow near Boulder Mail Trail, Grand Staircase Escalonte National Monument; CAS; ♂].

**Distribution.** —U.S.A.

***escoba*** Flint, 1972a: 9 [type locality: Guatemala, Izabal, Las Escobas, near Matias de Galvez; NMNH; ♂]. —Blickle and Denning 1977: 289 [checklist]. —Bueno-Soria 2009: 141 [♂]. —Barba-Álvarez et al. 2019: 85 [distribution].

**Distribution.** —Mexico, Guatemala.

***eyipantla*** Bueno-Soria & Santiago-Fragoso, 1998: 363 [type locality: Mexico, Veracruz, Salto de Eyipantla, Eyipantla River; CNIN; ♂]. —Bueno-Soria et al. 2005: 75 [distribution]. —Bueno-Soria 2009: 130 [♂].

**Distribution.** —Mexico.

***felipe*** Ross, 1944: 275 [type locality: [United States], Texas, San Felipe Springs, Del Rio; INHS; ♂]. —Flint 1972a: 9 [♂; distribution]. —Edwards 1973: 506 [distribution]. —Blickle and Denning 1977: 288 [checklist]. —Bueno-Soria and Flint 1978: 203 [distribution]. —Blickle 1979: 51, 87 [checklist; ♂]. —Moulton and Stewart 1997: 350 [checklist]. —Bueno-Soria 2009: 141 [♂]. —Bowles et al. 2007: 22 [distribution; biology].

**Distribution.** —Mexico, U.S.A.

***filiforma*** Flint, 1972a: 9 [type locality: Costa Rica, Cartago, Chitaria; NMNH; ♂]. —Blickle and Denning 1977: 289 [checklist]. —Holzenthal 1988: 62 [distribution]. —Bueno-Soria and Holzenthal 2008: 49 [distribution]. —Bueno-Soria 2009: 142 [♂].

**Distribution.** —Costa Rica.

***fioka*** Oláh & Johanson, 2011: 217 [type locality: Peru, San Martin Prov., La Catarata de Ahuashiyascu, 6°27.544'S, 76°18.192'W; NHRS; ♂].

**Distribution.** —Peru.

***flagellata*** Flint, 1972a: 5 [type locality: Panama Canal Zone, Barro Colorado Island; NMNH; ♂]. —Blickle and Denning 1977: 289 [checklist]. —Aguila 1992: 538 [distribution]. —Bueno-Soria 2009: 110 [♂; distribution]. —Armitage et al. 2015a: 7 [checklist]. —Armitage and Harris 2018b: 98 [checklist]. —Harris and Armitage 2019: 5 [distribution].

**Distribution.** —Panama, Mexico.

***flexura*** Flint & Bueno-Soria, 1999: 372 [type locality: Peru, Department Madre de Dios, Province Manu, Pakitza, trail 2, marker 15, Quebrada Trompetero; MHNJP; ♂].

—Ochrotrichia (O.) n. sp. 8: Flint 1996c: 399. —Flint and Bueno-Soria 1999: 732 [to synonymy].

**Distribution.** —Peru.

***flintiana*** Kumanski, 1987: 18 [type locality: Cuba, Province las Villas, S. foothills of Sierra de Trinidad, Rio Ssan Juan de la Juyua; SOFM; ♂]. —Flint 1996a: 16 [checklist]. —Botosaneanu 2002b: 86 [checklist]. —Naranjo López and González Lazo 2005: 149 [checklist].

**Distribution.** —Cuba.

***footei*** Keiper & Harris, 2002: 292 [type locality: United States, California, Riverside County near Idyl Wild, Fullers Mill Creek; CLEV; ♂; larva; pupa]. —Mendez et al. 2019: 118 [checklist].

**Distribution.** —U.S.A.

***fossi*** Ruiter & Harris, 2015: 324 [type locality: [United States], California, Napa County, Milliken Creek at Atlas Peak road bridge, Circle S Ranch, N38.41649, W122.24987; CAS; ♂]. —Mendez et al. 2019: 118 [checklist].

**Distribution.** —U.S.A.

***glabra*** Bueno-Soria & Santiago-Fragoso, 1997: 364 [type locality: Panama, Chiriqui, Guadalupe Arriba, 8°52'26"N 82°33'13"W; NMNH; ♂]. —Bueno-Soria and Holzenthal 2008: 49 [distribution]. —Bueno-Soria 2009: 110 [♂]. —Armitage et al. 2015a: 7 [checklist]. —Armitage and Harris 2018b: 98 [checklist].

**Distribution.** —Costa Rica, Panama.

***graysoni*** Parker & Voshell, 1980: 369 [type locality: [United States], Virginia, Bath Co., Jackson River, Rt. 603 2 miles S. Rt. 687; NMNH; ♂; ♀]. —Harris et al. 1984: 109 [distribution]. —Tarter 1990: 239 [checklist]. —Harris et al. 1991: 231 [distribution]. —Masteller and Flint 1992: 70 [checklist]. —DeWalt and Heinold 2005: 42 [phenology; distribution].

**Distribution.** —U.S.A.

***gretae*** Bueno-Soria, 2009: 113 [type locality: Mexico, Chihuahua, Mpio. Guachochi, km 114.5 Route 25 Creel-Guachochi, 27°35'05"N 107°32'46"W, 2150 m; CNIN; ♂].

**Distribution.** —Mexico.

***guadalupensis*** Harris & Moulton, 1993: 542 [type locality: United States, Texas, Culberson County, Smith Spring, 2.4 km N Park headquarters, Guadalupe Mountains National Park; NMNH; ♂]. —Moulton and Stewart 1997: 350 [checklist].

**Distribution.** —U.S.A.

***gurneyi*** Flint, 1964: 60 [type locality: Puerto Rico, El Yunque, cabins at La Mina; NMNH; ♂]. —Flint 1968a: 82 [checklist]. —Blickle and Denning 1977: 289 [checklist]. —Blickle 1979: 79 [♂]. —Botosaneanu 2002b: 86 [checklist].

**Distribution.** —Puerto Rico.

***hadria*** Denning & Blickle, 1972: 143 [type locality: [United States], Shasta County, California, Burney Falls; CAS; ♂]. —Blickle and Denning 1977: 288 [checklist]. —Blickle 1979: 51, 87 [checklist; ♂]. —Mendez et al. 2019: 118 [checklist].

**Distribution.** —U.S.A.

***hamatilis*** Flint & Bueno-Soria, 1999: 733 [type locality: Peru, Department Madre de Dios, Province Manu, Pakitza, trail 2, first stream; MHNJP; ♂; ♀].

—Ochrotrichia (O.) n. sp. 6: Flint 1996c: 398. —Flint and Bueno-Soria 1999: 733 [to synonymy].

**Distribution.** —Peru.

***harmas*** Oláh & Johanson, 2011: 218 [type locality: Peru, San Martin Prov., creek crossing rd. Juan Guerra-Chazuta, 14 km (rd.) E Colombia Bridge, 6°35.594'S, 76°13.172'W; NHRS; ♂].

**Distribution.** —Peru.

***hata*** Oláh & Johanson, 2011: 220 [type locality: French Guiana, Approuaguekaw, Kaw Mt, 4°33.072'N, 52°12.462'W, 270 m; NHRS; ♂].

**Distribution.** —French Guiana.

***hondurenia*** Bueno-Soria & Santiago-Fragoso, 1997: 364 [type locality: Belize, Cayo District, Mountain Pine Ridge; NMNH; ♂]. —Bueno-Soria and Holzenthal 2008: 49 [distribution]. —Bueno-Soria, 2009: 111 [♂].

**Distribution.** —Belize, Costa Rica.

***honeyi*** Blickle & Denning, 1977: 292 [type locality: [United States], California, Yosemite National Park, Mariposa County, S. Fork Merced River; ESUW; ♂]. —Blickle 1979: 52, 83 [checklist; ♂]. —Mendez et al. 2019: 118 [checklist].

**Distribution.** —U.S.A.

***igrapiuna*** de Souza, Santos, & Takiya, 2014a: 278 [type locality: Brazil, Igrapiúna, Reserva Ecológica Michelin, Mata de Vila 5, 13°49'22.9"S 39°12'6.5"W, 87 m; DZRJ; ♂]. —Paprocki and França 2014: 50 [checklist].

**Distribution.** —Brazil.

***ildria*** Denning & Blickle, 1972: 145 [type locality: United States, Arizona, Oak Creek Canyon; CAS; ♂]. —Blickle and Denning 1977: 288 [checklist] . —Blickle 1979: 52, 81 [checklist; ♂]. —Moulton et al. 1994: 170 [distribution]. —Houghton 2001: 90 [distribution]. —Blinn and Ruiter 2005: 69 [distribution; biology]. —Blinn and Ruiter 2006: 333 [biology; distribution]. —Bueno-Soria et al. 2007: 33 [distribution]. —Bueno-Soria 2009: 150 [♂]. —Blinn and Ruiter 2009a: 306 [biology]. —Blinn and Ruiter 2009b: 186 [phenology; distribution]. —Razo-González et al. 2020: 5 [distribution].

**Distribution.** —Mexico, U.S.A.

***indefinida*** Bueno-Soria & Holzenthal, 2004: 248 [type locality: Mexico, Tabasco, Municipio de km 5 Ruta Malpasito-Carlos A. Madrazo, Arroyo las Flores Villa de Guadalupe 2^a^ sección Los Chimalapas, 17°22'05"N 93°36'25"W, 545 m; CNIN; ♂]. —Bueno-Soria 2009: 136 [♂].

**Distribution.** —Mexico.

***ingloria*** Botosaneanu 1995: 25 [type locality: Dominican Republic, springs near La Descubierta, S of Sierra de Neiba; ZMAU; ♂]. —Botosaneanu 2002b: 86 [checklist]. —Flint and Pérez-Gelabert 1999: 40 [checklist]. —Flint and Sykora 2004: 37 [distribution]. —Pérez-Gelabert 2008: 301 [checklist].

**Distribution.** —Dominican Republic.

***insularis*** Mosely, 1934a: 163 [type locality: Jamaica, Runnaway Bay; NHMUK; ♂]. —Mosely), 1937b: 185 [to *Polytrichia*]. —Ross 1944: 126 [*Ochrotrichia* resurrected]. —Flint 1968b: 49 [♂; ♀]. —Flint 1968a: 82 [checklist]. —Blickle and Denning 1977: 289 [checklist]. —Botosaneanu and Hyslop 1998: 12 [♂; “enantiomorphic” morphotypes]. —Botosaneanu 2002b: 86 [checklist].

**Distribution.** —Jamaica.

***intermedia*** Flint, 1972a: 10 [type locality: Guatemala, Chimaltenango, Tecpan Guatemala; NMNH; ♂]. —Blickle and Denning 1977: 289 [checklist]. —Bueno-Soria 2009: 142 [♂].

**Distribution.** —Guatemala.

***intortilis*** Flint & Bueno-Soria, 1999: 730 [type locality: Peru, Department Cuzco, Province Paucartambo, Puente San Pedro at km 152, 44 km (road) W of Pilcopata, 13°03.30'S, 71°32/78'W; MHNJP; ♂; ♀].

—Ochrotrichia (O.) n. sp. 1: Flint, 1996c: 398. —Flint and Bueno-Soria 1999: 730 [to synonymy].

**Distribution.** —Peru.

***involuta*** Bueno-Soria & Holzenthal, 2004: 249 [type locality: Mexico, Guerrero, km 7 Route Taxco-Ixcateopan; CNIN; ♂]. —Bueno-Soria 2009: 143 [♂]. —Barba-Álvarez et al. 2019: 85 [distribution].

**Distribution.** —Mexico.

***islenia*** Botosaneanu, 1977: 261 [type locality: Cuba, Isla de Pinos, Santa Fé, Arroyo La Talega; NMNH; ♂]. —Botosaneanu 1979: 49 [distribution]. —Kumanski 1987: 17 [distribution]. —Flint et al. 1999a: 109 [as *O.islena*]. —Naranjo López and González Lazo 2005: 149 [checklist].

**Distribution.** —Cuba.

***ixcateopana*** Bueno-Soria & Santiago-Fragoso, 1997: 360 [type locality: Mexico, Guerrero, km 7 Route Taxco-Ixcateopan; CNIN; ♂]. —Rojas-Ascensio et al. 2002: 377 [distribution]. —Bueno-Soria 2009: 125 [♂].

**Distribution.** —Mexico.

***ixtlahuaca*** Bueno-Soria & Holzenthal, 2004: 253 [type locality: Mexico, Hidalgo, Ixtlahuaco, Ruta 105, Hotel Campestre Conchia, 20°53.04'N, 98°607'W, 1420 m; CNIN; ♂]. —Bueno-Soria 2009: 151 [♂]. —Razo-González 2018: 32 [distribution].

**Distribution.** —Mexico.

***jolandae*** Bueno-Soria & Holzenthal, 2008: 43 [type locality: Costa Rica, Alajuela, Reserva Forestal San Ramón Río San Lorencito and tribs., 10.216°N, 84.607°W, 980 m UMSP; ♂]. —Armitage and Harris 2018a: 9 [distribution]. —Armitage and Harris 2018b: 98 [checklist]. —Armitage and Harris 2018c: 283 [distribution].

— *abrelata* Harris & Armitage, 2015: 11 [type locality: Panama, Chiriquí Province, Cuenca 108, tributary of Quebrada Grande, at waterfall, Boquete, Valle Escondido, 8.78291°N, 82.44579°W, 1253 m; MIUP; ♂]. —Armitage et al. 2015a: 7 [checklist]. —Armitage and Harris 2018a: 10 [to synonymy].

**Distribution.** —Costa Rica, Panama.

***jonssoni*** Oláh & Johanson, 2011: 221 [type locality: French Guiana, Approuaguekaw, Kaw Mt, 4°33.133'N, 52°12.205'W, 263 m; NHRS; ♂].

**Distribution.** —French Guiana.

***ketaga*** Oláh & Johanson, 2011: 222 [type locality: Peru, San Martin Prov., creek crossing rd. Tarapoto-Yurimaguas, ca. 30 km (rd.) NE Tarapoto, 6°24.904'S, 76°18.756'W; NHRS; ♂].

**Distribution.** —Peru.

***ketarca*** Oláh & Johanson, 2011: 224 [type locality: Peru, Chontachaca, Kosnipata-Cusco, humid subtropical forest, 13°01'25"S, 71°28'03"W, 700 m; NHRS; ♂].

**Distribution.** —Peru.

***kettes*** Oláh & Johanson, 2011: 225 [type locality: Peru, San Martin Prov., creek crossing rd. Juan Guerra-Chazuta, 14 km (rd.) E Colombia Bridge, 6°35.594'S, 76°13.172'W; NHRS; ♂].

**Distribution.** —Peru.

***kondratieffi*** Harris & Armitage, 2019: 16 [type locality: Panama, Bocas del Toro Province, tributary of Quebrada Rambala at 2^nd^ footbridge, Rambala Jungle Lodge, 8.91627°N and 82.15469°W, 134 m; COZEM; ♂].

**Distribution.** —Panama.

***labafura*** Oláh & Johanson, 2011: 227 [type locality: French Guiana, Approuaguekaw Kaw Mt, 4°33.035'N, 52°11.661'W, 104 m; NHRS; ♂].

**Distribution.** —French Guiana.

***larimar*** Flint & Sykora, 2004: 38 [type locality: Dominican Republic, Barahona Province, Larimar Mine nr Filipinas; FSCA; ♂; ♀]. —Pérez-Gelabert 2008: 301 [checklist].

**Distribution.** —Dominican Republic.

***legeza*** Oláh & Johanson, 2011: 228 [type locality: Peru, San Martin Prov., La Catarata de Ahuashiyascu, 6°27.544'S, 76°18.192'W; NHRS; ♂].

**Distribution.** —Peru.

***leona*** Bueno-Soria & Holzenthal, 2004: 257 [type locality: Mexico, Distrito Federal, Desierto de Los Leones, Arroyo San Borja, 19°18.140'N, 99°18.648'W, 2650 m; CNIN; ♂]. —Bueno-Soria 2009: 126 [♂].

**Distribution.** —Mexico.

***limeirai*** de Souza, Santos, & Takiya, 2014a: 277 [type locality: Brazil, Ceará, Ubajara, Parque Nacional de Ubajara, 3°50'31.7"S, 40°53'55"W; CZMA; ♂]. —Paprocki and França 2014: 50 [checklist].

**Distribution.** —Brazil.

***limonensis*** Flint, 1981: 29 [type locality: Venezuela, Aragua, Dos Riitos, 6 km N Rancho Grande; NMNH; ♂]. —Flint 1968a: 82 [checklist]. —Botosaneanu 2002b: 86 [checklist].

**Distribution.** —Venezuela.

***lobifera*** Flint, 1968b: 50 [type locality: Jamaica, St. Andrew, Hope River near Newcastle at mile post 16.5; NMNH; ♂]. —Blickle and Denning 1977: 289 [checklist].

**Distribution.** —Jamaica.

***logana*** (Ross, 1941a): 54 [type locality: [United States], Utah, Logan Canyon; INHS; ♂; ♀; in *Polytrichia*]. —Ross 1944: 295 [to *Ochrotrichia*]. —Denning and Blickle 1972: 142 [checklist]. —Flint and Herrmann 1976: 898 [distribution]. —Blickle and Denning 1977: 288, 289 [distribution; ♀] . —Blickle 1979: 52, 83 [checklist; ♂]. —Ruite 1999: 165 [distribution]. —Blinn and Ruiter 2005: 69 [distribution; biology]. —Blinn and Ruiter 2006: 333 [distribution; biology]. —Blinn and Ruiter 2009a: 305 [biology]. —Bueno-Sori 2009: 152 [♂; distribution]. —Vieira et al. 2009: 257 [distribution]. —Kendrick and Huryn 2014: 280 [distribution]. —Mendez et al. 2019: 118 [checklist].

**Distribution.** —Mexico, U.S.A.

***lometa*** (Ross, 1941a): 55 [type locality: [United States], New Mexico, High Rolls; INHS; ♂; in *Polytrichia*]. —Denning and Blickle 1972: 142 [checklist]. —Blickle and Denning 1977: 288 [checklist]. —Resh and Sorg 1978: 396 [distribution]. —Blickle 1979: 52, 83 [checklist; ♂]. —Blinn and Ruiter 2005: 69 [distribution; biology]. —Blinn and Ruiter 2006: 33 [biology; distribution; as *lomenta*]. —Mendez et al. 2019: 118 [checklist].

**Distribution.** —U.S.A.

***longispina*** Bueno-Soria & Holzenthal, 2004: 250 [type locality: Panama, Chiriqui: Fortuna Dam site near Hornitos, 8°55'N, 82°16'W, 1050 m; NMNH; ♂]. —Bueno-Soria and Holzenthal 2008: 50 [distribution]. —Bueno-Soria 2009: 118 [♂]. —Oláh and Johanson 2011: 229 [distribution]. —Armitage et al. 2015a: 7 [checklist]. —Armitage and Harris 2018b: 98 [checklist]. —Harris and Armitage 2019: 5 [distribution].

**Distribution.** —Costa Rica, Panama, Peru.

***lucia*** Denning & Blickle, 1972: 147 [type locality: [United States], Hastings Reservation, Santa Lucia Mountains, Monterey County, California; CAS; ♂; specimen damaged]. —Blickle and Denning 1977: 288, 289 [checklist]. —Blickle 1979: 52, 85 [checklist; ♂]. —Mendez et al. 2019: 118 [checklist].

**Distribution.** —U.S.A.

***lupita*** Bueno-Soria & Santiago-Fragoso, 1997: 368 [type locality: Panama, Chiriqui, Guadalupe Arriba, 8°52'26"N, 82°33'13"W; NMNH; ♂]. —Bueno-Soria 2009: 118 [♂]. —Armitage et al. 2015a: 7 [checklist]. —Armitage and Harris 2018b: 98 [checklist].

**Distribution.** —Jamaica.

***machiguenga*** Flint & Bueno-Soria, 1999: 733 [type locality: Peru, Department Madre de Dios, Province Manu, Pakitza, trail 1, marker 14 (1^st^ stream) MHNJP; ♂].

—Ochrotrichia (O.) n. sp. 7: Flint 1996c: 398 —Flint and Bueno-Soria 1999: 733 [to synonymy].

**Distribution.** —Peru.

***maga*** Oláh & Johanson, 2011: 229 [type locality: Peru, San Martin Prov., La Catarata de Ahuashiyascu, 6°27.544'S, 76°18.192'W; NHRS; ♂].

**Distribution.** —Peru.

***malanae*** Ruiter & Harris, 2015: 324 [type locality: [United States], California, Napa County, Murphy Creek, @ 1010 Shadybrook Lane, 3 mi E Npa, N38.29378, W122.22026; CAS; ♂]. —Mendez et al. 2019: 118 [checklist].

**Distribution.** —U.S.A.

***manuensis*** Flint & Bueno-Soria, 1999: 735 [type locality: Peru, Department Madre de Dios, Province Manu, Pakitza, trail 2, first stream; MHNJP; ♂; ♀]. —de Souza et al. 2014a: 281 [distribution]. —Paprocki and França 2014: 50 [checklist].

—Ochrotrichia (O.) n. sp. 5: Flint 1996c: 398 —Flint and Bueno-Soria 1999: 735 [to synonymy].

**Distribution.** —Brazil, Peru.

***marica*** Flint, 1964: 60 [type locality: Puerto Rico, Maricao Forest, at stone house; NMNH; ♂]. —Flint 1968: 82 [checklist]. —Blickle and Denning 1977: 289 [checklist; as *O.marcia*]. —Botosaneanu 2002b: 86 [checklist].

**Distribution.** —Puerto Rico.

***maya*** Bueno-Soria & Santiago-Fragoso, 1997: 363 [type locality: Mexico, Chiapas, Cascada de Misolja, 20 km S from Palenque; NMNH; ♂]. —Bueno-Soria 2009: 131 [♂].

**Distribution.** —Mexico.

***maycoba*** Bueno-Soria & Santiago-Fragoso, 1997: 363 [type locality: Mexico, Sonora, Maycoba River, west of Maycoba; NMNH; ♂]. —Bueno-Soria 2009: 131 [♂].

**Distribution.** —Mexico.

***membrana*** Bueno-Soria & Holzenthal, 1998: 604 [type locality: Costa Rica, Alajuela, Reserva Forestal San Ramón, Río San Lorencito and tribs, 10°12'96"N, 84°36'42"W; NMNH; ♂]. —Bueno-Soria and Holzenthal 2008: 50 [distribution]. —Bueno-Soria 2009: 119 [♂].

**Distribution.** —Costa Rica.

***mono*** (Ross, 1941a): 55 [type locality: [United States], California, Mono County, Raceway Spring, Hot Creek; INHS; ♂; in *Polytrichia*]. —Blickle and Denning 1977: 288 [checklist]. —Blickle 1979: 52, 87 [checklist; ♂]. —Mendez et al. 2019: 118 [checklist].

**Distribution.** —U.S.A.

***moselyi*** Flint, 1972a: 7 [type locality: Mexico, Veracruz, Rio Tacolapan, route 180, km 551; NMNH; ♂]. —Blickle and Denning 1977: 289 [checklist]. —Bueno-Soria and Flint 1978: 203 [distribution]. —Luhman et al. 1999: 126 [pupae parasitized by Hymenoptera: Ceraphronidae; distribution]. —Bueno-Soria et al. 2005: 75 [distribution]. —Poinar and Anderson 2005: 344 [fossil adult parasitized by Hymenoptera: Braconidae]. —Bueno-Soria et al. 2007: 33 [distribution]. —Bueno-Soria and Holzenthal 2008: 50 [distribution]. —Bueno-Soria 2009: 126 [♂]. —Armitage et al. 2016: 10 [distribution]. —Armitage and Harris 2018b: 98 [checklist]. —Harris and Armitage 2019: 5 [distribution].

**Distribution.** —Costa Rica, Guatemala, Mexico, Panama.

***myersae*** Ruiter & Harris, 2015: 325 [type locality: [United States], California, Mono County, Owen’s Gorge Spring; EMEC; ♂]. —Mendez et al. 2019: 118 [checklist].

**Distribution.** —U.S.A.

***nacora*** Denning & Blickle, 1972: 145 [type locality: [United States], Humboldt County, California, Bear River; CAS; ♂; specimen damaged]. —Blickle and Denning 1977: 288 [checklist]. —Blickle 1979: 52, 85 [checklist; ♂]. —Ruiter and Harrris 2015: 331 [♂; distribution]. —Mendez et al. 2019: 118 [checklist].

**Distribution.** —U.S.A.

***nematomorpha*** Cavalcante, Dumas, & Nessimian, 2018: 230 [type locality: Brazil, Rio de Janeiro, Rio de Janeiro, Parque Nacional da Tijuca, Rio Tijuca, Cascatinha Taunay, 22°57'33.7"S 43°16'40.2"W, 407 m; DZRJ; ♂].

**Distribution.** —Brazil.

***nicaragua*** Bueno-Soria, 2009: 153 [type locality: Nicaragua, Zelaya, Cerro Saslaya, 13°44'N, 85°01'W, 700 m; NMNH; ♂]. —Razo-González 2018: 32 [distribution].

**Distribution.** —Mexico, Nicaragua.

***nimmoi*** Harris & Armitage, 2015: 13 [type locality: [Panama], Chiriquí Province, Cuenca 108, tributary of Quebrada Grande, at waterfall, Boquete, Valle Escondido, 8.78291°N, 82.44579°W, 1253 m; MIUP; ♂]. —Armitage et al. 2015a: 7 [checklist]. —Armitage and Harris 2018b: 98 [checklist]. —Armitage and Harris 2018c: 283 [distribution].

**Distribution.** —Panama.

***oblongata*** Bueno-Soria & Santiago-Fragoso, 1992: 443 [type locality: Trinidad, Arima cascade; NMNH; ♂]. —Botosaneanu and Sakal 1992: 202 [distribution; ecology, as Ochrotrichia (O.)]. —Botosaneanu and Alkins-Koo 1993: 17 [♂; ♀; distribution]. —Flint 1996b: 93 [distribution]. —Botosaneanu 2002b: 86 [checklist].

**Distribution.** —Trinidad, Venezuela.

***obovata*** Flint & Sykora, 2004: 38 [type locality: Dominican Republic, [La Vega Province] 20 km S Constanza; NMNH; ♂]. —Pérez-Gelabert 2008: 301 [checklist]/

**Distribution.** —Dominican Republic

***obtecta*** Flint & Bueno-Soria, 1999: 730 [type locality: Peru, Department Cuzco, Province Paucartambo, Puente San Pedro at km 152, 44 km (road) W of Pilcopata, 13°03.30'S, 71°32.78'W; MHNJP; ♂; ♀].

—Ochrotrichia (O.) n. sp. 2: Flint 1996c: 398 —Flint and Bueno-Soria 1999: 730 [to synonymy].

**Distribution.** —Peru.

***okaloosa*** Harris in Harris and Armitage 1987: 106 [type locality: [United States], Florida, Okaloosa Co., Turkey Creek at Base Road 233, Eglin Air Force Base, 5.0 mile NW Niceville; NMNH; ♂]. —Pescador et al. 2004: 133 [checklist]. —Harris et al. 2012: 9 [checklist].

**Distribution.** —U.S.A.

***okanoganensis*** Flint, 1965: 171 [type locality: [United States], Washington, Okanogan County, Winthrop; NMNH; ♂]. —Blickle and Denning 1977: 288 [checklist; as *O.okanoganesis*]. —Blickle 1979: 52, 83 [checklist; ♂; as *O.okanaganesis*]. —Bueno-Soria et al. 2007: 33 [distribution; as *O.okanogensis*].

**Distribution.** —Mexico, U.S.A.

***oldala*** Oláh & Johanson, 2011: 231 [type locality: French Guiana, Approuaguekaw, Kaw Mt, 4°33.035'N, 52°11.661'W, 104 m; NHRS; ♂].

**Distribution.** —French Guiana.

***oregona*** (Ross, 1938a): 121 [type locality: [United States], Oregon, La Grande, along Grande Ronde River; INHS; ♂; in *Polytrichia*]. —Denning 1947a: 147 [♀; distribution]. —Blickle and Denning 1977: 288 [checklist]. —Blickle 1979: 52, 85 [checklist; ♂].

**Distribution.** —U.S.A.

***ostoroska*** Oláh & Johanson, 2011: 232 [type locality: Peru, San Martin Prov., La Catarata de Ahuashiyascu, 6°27.544'S, 76°18.192'W; NHRS; ♂].

**Distribution.** —Peru.

***pacifica*** Flint, 1972a: 6 [type locality: Panama, Chiriqui, Rio Caimito, 16 km NW David; NMNH; ♂]. —Blickle and Denning 1977: 289 [checklist]. —Bueno-Soria and Flint 1978: 204 [distribution]. —Holzenthal 1988: 62 [distribution]. —Aguila 1992: 538 [distribution]. —Bueno-Soria et al. 2005: 75 [distribution]. —Bueno-Soria and Holzenthal 2008: 50 [checklist]. —Bueno-Soria 2009: 137 [♂]. —Armitage et al. 2015a: 7 [checklist]. —Armitage and Harris 2018b: 98 [checklist]. —Harris and Armitage 2019: 5 [distribution].

**Distribution.** —Costa Rica, Mexico, Panama.

***palitla*** Flint, 1972a: 9 [type locality: Mexico, San Luis Potosi, Palitla; NMNH; ♂]. —Blickle and Denning 1977: 289 [checklist]. —Bueno-Soria and Flint 1978: 204 [distribution]. —Bueno-Soria 2009: 143 [♂].

**Distribution.** —Mexico.

***palmata*** Bueno-Soria & Santiago-Fragoso, 1997: 369 [type locality: Mexico, Estado de Mexico, Temascaltepec; CNIN; ♂]. —Bueno-Soria 2009: 138 [♂].

**Distribution.** —Mexico.

***panamensis*** Flint, 1972a: 10 [type locality: Panama, Chiriqui, Rovira, David; NMNH; ♂]. —Blickle and Denning 1977: 289 [checklist]. —Aguila 1992: 539 [distribution]. —Chamorro-Lacayo et al. 2007: 43 [distribution]. —Bueno-Soria and Holzenthal 2008: 51 [distribution]. —Bueno-Soria 2009: 153 [♂]. —Armitage et al. 2015a: 7 [checklist]. —Armitage and Harris 2018b: 98 [checklist]. —Armitage and Harris 2018c: 283 [distribution].

**Distribution.** —Costa Rica, Nicaragua, Panama.

***paraldama*** Bueno-Soria, 2009: 113 [type locality: Panama, San Blas, Río Cartí Grande, 2 km. Nusagandi; NMNH; ♂]. —Armitage et al. 2015a: 7 [checklist]. —Armitage and Harris 2018b: 98 [checklist]. —Harris and Armitage 2019: 5, 21 [distribution; ♂].

**Distribution.** —Panama.

***patulosa*** (Wasmund & Holzenthal, 2007): 18 [type locality: Brazil, Rio de Janeiro, Parque Nacional da Serra dos Órgãos, Rio Beija-flor, 22°27'04"S, 43°00'04"W, 1125 m; MZUSP; ♂; ♀; in *Rhyacopsyche*]. —Dumas et al. 2009: 367 [distribution]. —Oláh and Johanson 2011: 234 [to *Ochrotrichia*]. —de Souza et al. 2014a: 281 [distribution]. —Paprocki and França 2014: 50 [checklist].

**Distribution.** —Brazil.

***pectinata*** Flint, 1972a: 5 [type locality: Mexico, Veracruz, Rio Jamapa, north of Coscomatepec; NMNH; ♂]. —Blickle and Denning 1977: 289 [checklist]. —Bueno-Soria and Flint 1978: 204 [distribution]. —Bueno-Soria 2009: 111 [♂].

**Distribution.** —Mexico.

***pectinifera*** Flint, 1972a: 7 [type locality: Mexico, Veracruz, Fortin de las Flores; NMNH; ♂]. —Blickle and Denning 1977: 289 [checklist]. —Bueno-Soria and Flint 1978: 204 [distribution]. —Bueno-Soria 2009: 127 [♂].

**Distribution.** —Mexico.

***phenosa*** Ross, 1947: 147 [type locality: [United States], Oregon, Deschutes River, Redmond; INHS; ♂]. —Blickle and Denning 1977: 288 [checklist]. —Blickle 1979: 52, 85 [checklist; ♂]. —Mendez et al. 2019: 128 [checklist].

**Distribution.** —U.S.A.

***poblana*** Bueno-Soria & Santiago-Fragoso, 1997: 370 [type locality: Mexico, Puebla, km 30, route Zacapoaxtla-Zacatlán; CNIN; ♂]. —Bueno-Soria 2009: 154 [♂].

**Distribution.** —Mexico.

***ponta*** Flint, 1968a: 61 [type locality: Dominica, Pont Casse, 0.4 mi E; NMNH; ♂]. —Blickle and Denning 1977: 289 [checklist]. —Flint and Sykora 1993: 58 [distribution]. —Botosaneanu 1994a: 37 [distribution]. —Botosaneanu 2002b: 86 [checklist]. —Botosaneanu and Thomas 2005: 44 [distribution].

**Distribution.** —Dominica, Grenada, Guadeloupe, Martinique, St. Vincent.

***pora*** Angrisano & Sganga, 2009: 62 [type locality: Argentina, Misiones, Parque Provincial Salto Encantado, Salto Acutí; MACN; ♂].

**Distribution.** —Argentina.

***potomus*** Denning, 1947a: 146 [type locality: [United States], Wyoming, Torrington, North Platte River; ESUW; ♂; ♀]. —Denning 1948: 398 [distribution]. —Blickle and Denning 1977: 288 [checklist]. —Blickle 1979: 52, 79 [checklist; ♂]. —Bowles and Mathis 1992: 32 [distribution].

**Distribution.** —U.S.A.

***priapo*** de Souza, Santos, & Takiya, 2014a: 275 [type locality: Brazil, Bahia, Igrapiúna, Reserva Ecológica da Michelin, Mata da Vila 5, 13°49'22.6"S, 39°12'6.5"W, 87 m; DZRJ; ♂].

**Distribution.** —Brazil. —Paprocki and França 2014: 52 [checklist].

***provosti*** Blickle, 1961: 132 [type locality: [United States], Florida, Temple Terrace; INHS; ♂; in *Neotrichia*]. —Blickle and Denning 1977: 288 [checklist]. —Blickle 1979: 52, 79 [checklist; ♂]. —Pescador et al. 2004: 133 [checklist]. —Harris et al. 2012: 9 [checklist].

**Distribution.** —U.S.A.

***pulgara*** Harris & Armitage, 2015: 13 [type locality: [Panama], Chiriquí Province, Cuenca 108, tributary of Quebrada Grande, at waterfall, Boquete, Valle Escondido, 8.78291°N, 82.44579°W, 1253 m; MIUP; ♂]. —Armitage et al. 2015a: 7 [checklist]. —Armitage and Harris 2018b: 98 [checklist]. —Armitage and Harris 2018c: 283 [distribution].

**Distribution.** —Panama.

***puposa*** Oláh & Johanson, 2011: 234 [type locality: Peru, San Martin Prov., La Catarata de Ahuashiyascu, 6°27.544'S, 76°18.192'W; NHRS; ♂].

**Distribution.** —Peru.

***puyana*** Bueno-Soria & Santiago-Fragoso, 1992: 440 [type locality: Ecuador, Pastaza Prov., Puyo; NMNH; ♂]. —Ríos-Touma et al. 2017: 10 [checklist].

**Distribution.** —Ecuador.

***quadrispina*** Denning & Blickle, 1972: 150 [type locality: [United States], Southwest Research Station, Portal, Cochise County, Arizona; CAS; ♂]. —Blickle and Denning 1977: 288 [checklist] . —Blickle 1979: 52, 81 [checklist; ♂]. —Moulton et al. 1994: 170 [distribution]. —Keiper and Walton 2000: 183 [larva]. —Blinn and Ruiter 2005: 69 [distribution; biology]. —Blinn and Ruiter 2006: 333 [biology; distribution]. —Mendez et al. 2019: 118 [checklist].

**Distribution.** —U.S.A.

***quasi*** Bueno-Soria & Holzenthal, 2008: 46 [type locality: Costa Rica, San José, Río Savegra, San Gerardo de Dota, 9.33°N, 83.48°W, 2200 m; CMNH; ♂].

**Distribution.** —Costa Rica.

***quebrada*** Bueno-Soria & Holzenthal, 1998: 607 [type locality: Costa Rica, Guanacaste, P. N. Rincón de la Vieja, Quebrada Zopilote, 10°45'9"N, 83°18'54"W; NMNH; ♂]. —Bueno-Soria and Holzenthal 2008: 51 [distribution]. —Bueno-Soria 2009: 119 [♂].

**Distribution.** —Costa Rica.

***quinealensis*** Bueno-Soria & Holzenthal, 1998: 611 [type locality: Costa Rica, Puntarenas, Río Guineal, ca. 1 km (air) E Finca Helechales, 9°4'56"N, 83°5'52"W; NMNH; ♂]. —Bueno-Soria and Holzenthal 2008: 51 [distribution]. —Bueno-Soria 2009: 138 [♂]. —Armitage et al. 2020: 5 [distribution].

**Distribution.** —Costa Rica, Panama.

***ramona*** Bueno-Soria & Holzenthal, 1998: 610 [type locality: Costa Rica, Alajuela, Reserva Forestal San Ramón, Río San Lorencito and tribs, 10°12'96"N, 84°36'42"W; NMNH; ♂]. —Bueno-Soria and Holzenthal 2008: 52 [distribution]. —Bueno-Soria 2009: 146 [♂]. —Armitage et al. 2016: 11 [distribution]. —Armitage and Harris 2018b: 98 [checklist].

**Distribution.** —Costa Rica, Panama.

***raposa*** Bueno-Soria & Santiago-Fragoso, 1992: 440 [type locality: Colombia, Dept. Valle del Cauca, Río Raposo; NMNH; ♂]. —Muñoz-Quesada 2000: 278 [checklist]. —Ríos-Touma et al. 2017: 10 [checklist].

**Distribution.** —Colombia, Ecuador.

***regina*** Bueno-Soria & Santiago-Fragoso, 1997: 368 [type locality: Panama, Barro Colorado Island, Snyder-Molino Trail, Marker 3; NMNH; ♂]. —Bueno-Soria 2009: 120 [♂]. —Armitage et al. 2015a: 7 [checklist]. —Armitage and Harris 2018b: 98 [checklist].

**Distribution.** —Panama.

***regiomontana*** Bueno-Soria & Holzenthal, 2004: 255 [type locality: Mexico, Nuevo Leon, Municipio Santiago, Potrero Redondo; CNIN; ♂]. —Bueno-Soria 2009: 155 [♂].

**Distribution.** —Mexico.

***riesi*** Ross, 1944: 132 [type locality: [United States], Illinois, Utica, Split Rock Brook; INHS; ♂; ♀]. —Blickle and Denning 1977: 288 [checklist]. —Etnier and Schuster 1979: 18 [distribution]. —Blickle 1979: 52, 81 [checklist; ♂]. —Waltz and McCafferty 1983a: 11 [distribution]. —Harris et al. 1984: 109 [distribution]. —Harris et al. 1991: 232 [distribution]. —Frazer et al. 1991: 20 [distribution]. —Mathis and Bowles 1992: 24 [distribution]. —Moulton and Stewart 1996: 118 [♂; distribution]. —Houp 1999: 2 [distribution]. —Etnier 2010: 486 [distribution]. —Houghton et al. 2017: 63 [checklist]. —Bowles et al. 2020: 8 [distribution].

**Distribution.** —U.S.A.

***robisoni*** Frazer & Harris, 1991a: 364 [type locality: [United States], Arkansas, Perry County, Bear Creek at State Highway 7, two miles south of Hollis; NMNH; ♂]. —Moulton and Stewart 1996: 119 [♂; distribution]. —Etnier 2010: 486 [distribution].

**Distribution.** —U.S.A

***rothi*** Denning & Blickle, 1972: 149 [type locality: [United States], Ariz[ona], Southwest Research Stations, Cochise County, Portal CAS; ♂; ♀]. —Blickle and Denning 1977: 288 [checklist]. —Blickle 1979: 52, 83 [checklist; ♂]. —Moulton and Stewart 1997: 350 [checklist]. —Baumgardner and Bowles 2005: 11 [distribution]. —Blinn and Ruiter 2005: 69 [distribution; biology]. —Bueno-Soria et al. 2007: 33 [distribution]. —Bueno-Soria 2009: 152 [♂]. —Ruiter and Harrris 2015: 329 [♂; distribution]. —Mendez et al. 2019: 118 [checklist].

**Distribution.** —Mexico, U.S.A.

***sagitta*** Cavalcante, Dumas, & Nessimian, 2018: 232 [type locality: Brazil, Rio de Janeiro, Rio de Janeiro, Parque Nacional da Tijuca, Rio Cova da Onça, 22°57'45.2"S, 43°17'36.5"W, 494 m; DZRJ; ♂].

**Distribution.** —Brazil.

***salaris*** Blickle & Denning, 1977: 289 [type locality: [United States], California, Lake County, Butts Creek, Butts Canyon, 0.5 mile S. E. Black Oakes Villa, 825’; CAS; ♂]. —Blickle 1979: 52, 85 [checklist; ♂]. —Mendez et al. 2019: 118 [checklist].

**Distribution.** —U.S.A.

***seiba*** Flint & Sykora, 2004: 38 [type locality: Dominican Republic, El Seibo Province, Pedro Sanchez, small stream; FSCA; ♂; ♀]. —Pérez-Gelabert 2008: 301 [checklist].

**Distribution.** —Dominican Republic.

***serra*** Botosaneanu, 1991: 125 [type locality: Haiti, Département de l’Ouest, Ville Bonheur, Le Saut d’Eau; ZMUA; ♂]. —Flint and Pérez-Gelabert 1999: 41 [checklist]. —Botosaneanu 2002b: 87 [checklist]. —Flint and Sykora 2004: 39 [distribution]. —Pérez-Gelabert 2008: 301 [checklist].

**Distribution.** —Haiti.

***serrana*** Bueno-Soria & Santiago-Fragoso, 1997: 370 [type locality: Mexico, Guerrero, Acahuizotla, 10 km E of Chilpancingo; CNIN; ♂]. —Bueno-Soria 2009: 156 [♂].

**Distribution.** —Mexico.

***shawnee*** (Ross, 1938a): 120 [type locality: [United States], Illinois, Herod; INHS; ♂; in *Polytrichia*]. —Ross 1944: 131 [♂; distribution]. —Morse and Blickle 1953: 72 [distribution]. —Blickle and Denning 1977: 288 [checklist]. —Etnier and Schuster 1979: 18 [distribution]. —Floyd and Schuster 1990: 130, 132 [distribution]. —Harris et al. 1991: 233 [distribution]. —Frazer and Harris 1991a: 366–367 [♂]. —Masteller and Flint 1992: 70 [distribution]. —Moulton and Stewart 1996: 119 [♂; distribution].

**Distribution.** —U.S.A.

***silva*** Bueno-Soria & Holzenthal, 1998: 606 [type locality: Costa Rica, Alajuela, Reserva Forestal San Ramón, Río San Lorencito and tribs, 10°12'96"N, 84°36'42"W; NMNH; ♂]. —Bueno-Soria and Holzenthal 2008: 52 [distribution]. —Bueno-Soria 2009: 121 [♂].

**Distribution.** —Costa Rica.

***spina*** Bueno-Soria & Holzenthal, 2004: 254 [type locality: Mexico, Veracruz, Puente Río Jamapa, Route Coscomatepec-Huatusco, 1320 m; CNIN; ♂]. —Bueno-Soria 2009: 156 [♂].

**Distribution.** —Mexico.

***spinosa*** (Ross, 1938a): 121 [type locality: [United States], Oklahoma, Turner Falls State park, along Honey Creek; INHS; ♂; in *Polytrichia*]. —Ross 1944: 132 [♂; ♀; distribution]. —Etnier 1968: 191 [distribution]. —Blickle and Denning 1977: 288 [checklist]. —Blickle 1979: 52, 85 [checklist; ♂]. —Swegman et al. 1981: 139 [distribution]. —Huryn and Foote 1983: 791 [distribution]. —Steven and Hilsenhoff 1984: 164 [distribution]. —Bowles and Mathis 1989: 239 [distribution]. —Masteller and Flint 1992: 70 [distribution]. —Mathis and Bowles 1992: 24 [distribution]. —Bowles and Mathis 1992: 32 [distribution]. —Moulton and Stewart 1996: 119 [♂; distribution]. —Keiper and Foote 2000: 226 [biology; distribution]. —Houghton et al. 2001: 505 [distribution]. —Armitage et al. 2011: 14 [checklist]]. —Houghton et al. 2011b: 5 [phenology; habitat; distribution]. —Houghton et al. 2011a: 387, 388 [distribution; biology]. —Houghton et al. 2017: 63 [checklist].

**Distribution.** —U.S.A.

***spinosissima*** Flint, 1964: 58 [type locality: Puerto Rico, Toro Negro Forest, near Dona Juana area; NMNH; ♂]. —Flint 1968a: 59 [♂; ♀; distribution]. —Blickle and Denning 1977: 289 [checklist]. —Flint and Sykora 1993: 50 [checklist]. —Botosaneanu 2002b: 87 [checklist]. —Botosaneanu and Thomas 2005: 44 [distribution].

**Distribution.** —Dominica, Martinique, Puerto Rico.

***spinula*** Bueno-Soria & Holzenthal, 2004: 255 [type locality: Mexico, Chiapas, Reserva de la Biosfera El Triunfo; CNIN; ♂]. —Bueno-Soria 2009: 157 [♂].

**Distribution.** —Mexico.

***spinulata*** Denning & Blickle, 1972: 149 [type locality: [United States], Ariz[ona], Portal, Cochise County; CAS; ♂]. —Blickle and Denning 1977: 288 [checklist]. —Blickle 1979: 52, 81 [checklist; ♂]. —Moulton and Stewart 1997: 350 [checklist]. —Baumgardner and Bowles 2005: 11 [distribution]. —Blinn and Ruiter 2005: 69 [biology; distribution]. —Blinn and Ruiter 2006: 33 [biology; distribution]. —Bueno-Soria et al. 2007: 33 [distribution]. —Bueno-Soria 2009: 146 [♂].

**Distribution.** —Mexico, U.S.A.

***spinulosa*** Bueno-Soria, 2009: 124 [type locality: Mexico, Estado de México, Temascaltepec Real de Arriba, 19°02'24"N, 100°02'47"W, 1720 m; CNIN; ♂].

**Distribution.** —Mexico.

***spira*** Thomson & Holzenthal, 2012: 27 [type locality: Venezuela, Monagas, Guachero Cave National Park, 10°10.322'N, 63°33.315'W, 1110 m; UMSP; ♂].

**Distribution.** —Venezuela.

***stylata*** (Ross, 1938a): 120 [type locality: United States, Wyoming, Farson, Little Sandy Creek; INHS; ♂; in *Polytrichia*]. —Denning 1947b: 171 [distribution]. —Denning 1947a: 146 [distribution]. —Flint 1972a: 5 [♂; distribution]. —Denning and Blickle 1972: 143 [checklist]. —Blickle and Denning 1977: 288 [checklist]. —Bueno-Soria and Flint 1978: 204 [distribution]. —Blickle 1979: 52, 87 [checklist; ♂]. —Frazer and Harris 1991a: 366–367 [♂]. —Bowles and Mathis 1992: 32 [distribution]. —Moulton et al. 1994: 170 [distribution]. —Moulton and Stewart 1996: 120 [♂; distribution]. —Moulton and Stewart 1997: 350 [checklist]. —Ruiter 1999: 165 [distribution]. —Houghton 2001: 90 [distribution]. —Blinn and Ruiter 2005: 69 [distribution; biology]. —Blinn and Ruiter 2006: 333 [distribution; biology]. —Bowles et al. 2007: 22 [distribution; biology]. —Bueno-Soria et al. 2007: 33 [distribution]. —Bueno-Soria 2009: 139 [♂]. —Blinn and Ruiter 2009a: 303 [biology]. —Blinn and Ruiter 2009b: 186 [phenology; distribution]. —Razo-González 2018: 32 [distribution]. —Barba-Álvarez et al. 2019: 86 [distribution]. —Mendez et al. 2019: 118 [checklist].

**Distribution.** —Guatemala, Mexico, U.S.A.

***susanae*** Flint & Herrmann, 1976: 894 [type locality: United States, Colorado, Chaffee Co., Trout Creek Spring; NMNH; ♂]. —Blickle 1979: 52, 81 [checklist; ♂].

**Distribution.** —U.S.A.

***tagala*** Flint, 1972a: 8 [type locality: Guatemala, Huehuetenango, 32 km NW Huehuetenango; NMNH; ♂]. —Blickle and Denning 1977: 289 [checklist]. —Maes and Flint 1988: 4 [distribution]. —Maes 1999: 1194 [checklist]. —Bueno-Soria et al. 2005: 75 [checklist]. —Chamorro-Lacayo et al. 2007: 43 [distribution]. —Bueno-Soria and Holzenthal 2008: 52 [distribution]. —Bueno-Soria 2009: 128 [♂; distribution].

**Distribution.** —Costa Rica, Guatemala, Mexico, Nicaragua.

***tarsalis*** (Hagen, 1861): 275 [type locality: Canada (Osten Sacken), St. Lawrence River; MCZ; ♂; in *Hydroptila*]. —Eaton 1873: 148 [comments on general appearance]. —Banks 1907a: 50 [catalogue]. —Betten 1934: 160 [checklist]. —Ross 1938b: 10 [lectotype designated; in *Polytrichia*]. —Ross 1944: 130 [♂; ♀; distribution]. —Etnier 1965: 147 [distribution]. —Unzicker et al. 1970: 172 [distribution]. —Flint 1972a: 6 [♂; distribution]. —Denning and Blickle 1972: 143 [checklist]. —Edwards 1973: 506 [distribution; as *tarsialis*]. —Blickle and Denning 1977: 289 [checklist]. —Bueno-Soria and Flint 1978: 204 [distribution]. —Resh et al. 1978: 383 [distribution]. —Blickle 1979: 53, 79 [checklist; ♂]. —Parker and Voshell 1981: 4 [checklist]. —Harris et al. 1982a: 511 [distribution]. —Huryn and Foote 1983: 791 [distribution]. —Waltz and McCafferty 1983a: 11 [distribution]. —Hamilton et al. 1983: 18 [distribution]. —Harris et al. 1984: 109 [distribution]. —Bowles and Mathis 1989: 239 [distribution]. —Tarter 1990: 239 [checklist]. —Harris et al. 1991: 234 [distribution]. —Frazer et al. 1991: 20 [distribution]. —Frazer and Harris 1991a: 366–367 [♂]. —Masteller and Flint 1992: 70 [checklist]. —Mathis and Bowles 1992: 24 [distribution]. —Bowles and Mathis 1992: 32 [distribution]. —Moulton et al. 1993: 21 [distribution]. —Moulton et al. 1994: 170 [distribution]. —Moulton and Stewart 1996: 120 [♂; distribution]. —Abbott et al. 1997: 44 [distribution]. —Moulton and Stewart 1997: 350 [checklist]. —Houghton and Stewart 1998: 105 [biology; distribution]. —Ruiter 1999: 165 [distribution]. —Houghton et al. 2001: 505 [distribution]. —Pescador et al. 2004: 133 [checklist]. —Baumgardner and Bowles 2005: 11 [distribution]. —Blinn and Ruiter 2005: 69 [distribution; biology]. —Bueno-Soria et al. 2005: 75 [distribution]. —Blinn and Ruiter 2006: 333 [biology; distribution]. —Zeullig et al. 2006: 43 [distribution]. —Bowles et al. 2007: 22 [distribution; biology]. —Blinn and Ruiter 2009a: 304 [biology]. —Blinn and Ruiter 2009b: 186 [phenology; distribution]. —Flint et al. 2009:7 [distribution]. —Bueno-Soria 2009: 139 [♂]. —Flint 2011: 104 [distribution]. —Armitage et al. 2011: 14 [checklist]. —Myers et al. 2011: 108 [distribution]. —Harris et al. 2012: 9 [checklist]. —Blinn and Ruiter 2013: 291 [biology; distribution]. —Houghton et al. 2017: 63 [checklist]. —Barba-Álvarez et al. 2019: 86 [distribution]. —Bowles et al. 2020: 8 [distribution].

**Distribution.** —Canada, Mexico, U.S.A.

***taunay*** Cavalcante, Dumas, & Nessimian, 2018: 232 [type locality: Brazil, Rio de Janeiro, Rio de Janeiro, Parque Nacional da Tijuca, Rio Tijuca, Cascatinha Taunay, 22°57'33.7"S, 43°16'40.2"W, 407 m; DZRJ; ♂].

**Distribution.** —Brazil.

***tenanga*** (Mosely, 1937b): 185 [type locality: Mexico, Chiapas, Saltenango de la Paz; NHMUK; ♂; in *Polytrichia*]. —Ross 1944: 276 [♂; in *Ochrotrichia*]. —Flint 1972a: 8 [♂; distribution]. —Blickle and Denning 1977: 289 [checklist]. —Bueno-Soria and Flint 1978: 204 [distribution]. —Flint 1981: 29 [♂; distribution]. —Holzenthal 1988: 62 [distribution]. —Flint and Reyes 1991: 487 [distribution]. —Aguila 1992: 538 [distribution]. —Bueno-Soria and Holzenthal 2008: 52 [distribution]. —Bueno-Soria 2009: 144 [♂]. —Armitage et al. 2015a: 7 [checklist]. —Armitage and Harris 2018b: 98 [checklist]. —Armitage and Harris 2018c: 283 [distribution]. —Harris and Armitage 2019: 5 [distribution]. —Barba-Álvarez et al. 2019: 86 [distribution].

**Distribution.** —Costa Rica, Guatemala, Honduras, Mexico, Panama, Peru, Venezuela.

***tenuata*** Blickle & Denning, 1977: 293 [type locality: [United States], Oregon, Douglas County, Myrtle Creek; EMEC; ♂; ♀]. —Blickle 1979: 52, 87 [checklist; ♂]. —Ruiter and Harris 2015: 333 [♂; distribution]. —Mendez et al. 2019: 119 [checklist].

**Distribution.** —U.S.A.

***transylvanica*** Harris & Floyd in Floyd et al. 1997: 140 [type locality: [United States], North Carolina, Transylvania County, Corbin Creek, 1.5 mile N Whitewater River bridge at SR 281, ca. 910 m; NMNH; ♂].

**Distribution.** —U.S.A.

***trapoiza*** Ross, 1947: 146 [type locality: [United States], Colorado, Buena Vista, sweeping marsh at Yale Lake; INHS; ♂]. —Blickle and Denning 1977: 288 [checklist]. —Blickle 1979: 53, 81 [checklist; ♂]. —Mendez et al. 2019: 119 [checklist].

**Distribution.** —U.S.A.

***trinitatis*** Flint, 1996b: 93 [type locality: Trinidad, streamlet below Maracas Waterfall, 250 m, 10°44'N, 61°24'W; NMNH; ♂]. —Botosaneanu 1997: 45 [♂; as undescribed species]. —Botosaneanu 2002b: 87 [checklist].

**Distribution.** —Trinidad.

***tuscaloosa*** Harris & Kelley, 1984a: 572 [type locality: [United States], Alabama, Tuscaloosa County, Tyro Creek, 4 miles southeast of Berry; NMNH; ♂; ♀].

**Distribution.** —U.S.A.

***unica*** Bueno-Soria & Santiago-Fragoso, 1992: 443 [type locality: Colombia, Dept. Valle del Cauca, Rio Raposo; NMNH; ♂]. —Muñoz-Quesada 2000: 278 [checklist]. —Armitage et al. 2020: 5 [distribution].

**Distribution.** —Colombia, Panama.

***unicornia*** Bueno-Soria & Holzenthal, 2004: 256 [type locality: Mexico, Oaxaca, Santa Maria de Yavesia (water plant), 17°13'36"N, 96°25'35"W, 1930 m; CNIN; ♂]. —Bueno-Soria 2009: 122 [♂].

**Distribution.** —Mexico.

***unio*** (Ross, 1941a): 56 [type locality: [United States], Illinois, Alto Pass, Union Spring; INHS; ♂; in *Polytrichia*]. —Ross 1944: 129 [♂; larva; distribution]. —Etnier and Schuster 1979: 18 [distribution]. —Blickle 1979: 53, 79 [checklist]; ♂]. —Mathis and Bowles 1992: 24 [distribution]. —Moulton and Stewart 1996: 120 [♂; distribution].

**Distribution.** —U.S.A.

***velascoi*** Bueno-Soria & Santiago-Fragoso, 1997: 371 [type locality: Mexico, Guerrero, route 134, 102 km N.W. Zihuatanejo; CNIN; ♂]. —Bueno-Soria 2009: 157 [♂].

**Distribution.** —Mexico.

***verda*** Flint, 1968c: 153 [type locality: Puerto Rico, el Verde; NMNH; ♂]. —Flint 1968a: 82 [checklist]. —Blickle and Denning 1977: 289 [checklist]. —Botosaneanu 2002b: 87 [checklist]. —Flint and Sykora 2004: 40 [distribution]. —Pérez-Gelabert 2008: 301 [checklist].

**Distribution.** —Dominican Republic, Puerto Rico.

***vertreesi*** Denning & Blickle, 1972: 149 [type locality: [United States], 7 miles west of Roseburg, Douglas County, Oregon, North Umpqua River; CAS; ♂; specimen damaged]. —Blickle and Denning 1977: 288 [checklist]. —Blickle 1979: 53, 87 [checklist; ♂]. —Ruiter and Harrris 2015: 333 [♂; distribution]. —Mendez et al. 2019: 119 [checklist].

**Distribution.** —U.S.A.

***vieja*** Bueno-Soria & Holzenthal, 1998: 610 [type locality: Costa Rica, Guanacaste, P.N. Rincón de la Vieja, Quebrada Provisión, 10°46'14"N, 85°16'86"W; NMNH; ♂]. —Bueno-Soria and Holzenthal 2008: 53 [distribution]. —Bueno-Soria 2009: 147 [♂].

**Distribution.** —Costa Rica.

***villarenia*** Botosaneanu, 1980: 108 [type locality: Cuba, Prov. Las Villas, Cafetal <<Gaviña>>, La Sierrita; ZMUA; ♂]. —Botosaneanu 1979: 48 [distribution]. —Kumanski 1987: 18 [distribution]. —Flint 1996a: 16 [checklist]. —Botosaneanu 2002b: 87 [checklist]. —Naranjo López and González Lazo 2005: 149 [checklist].

**Distribution.** —Cuba.

***weddleae*** Ross, 1944: 274 [type locality: [United States], Oklahoma, Cloudy Creek near Cloudy; INHS; ♂; ♀]. —Unzicker et al. 1970: 172 [distribution; as *weedleae*]. —Blickle and Denning 1977: 288 [checklist]. —Blickle 1979: 53, 79 [checklist; ♂]. —Bowles and Mathis 1989: 239 [distribution; as *weedleae*]. —Bowles and Mathis 1992: 32 [distribution; as *weedleae*]. —Moulton and Stewart 1996: 121 [♂; distribution]. —Etnier 2010: 486 [distribution].

**Distribution.** —U.S.A.

***weoka*** Harris, 1989: 313 [type locality: [United States], Alabama, Elmore County, Fisher Creek on unmarked county road, 3.5 miles southwest Weoka, T20N, R18E, S36; NMNH; ♂]. —Harris et al. 1991: 236 [distribution]. —Roble et al. 2019: 43 [distribution].

**Distribution.** —U.S.A.

***wojcickyi*** Blickle, 1963: 20 [type locality: [United States], Maine, Dennistown; INHS; ♂]. —Blickle and Denning 1977: 288 [checklist]. —Blickle 1979: 53, 83[checklist; ♂]. —Swegman et al. 1981: 139 [distribution]. —Parker and Voshell 1981: 4 [checklist]. —Huryn and Foote 1983: 791 [distribution]. —Light and Adler 1983: 77 [distribution; biology]. —Waltz and McCafferty 1983a: 11 [distribution]. —Usis and Foote 1989: 84 [distribution]. —Masteller and Flint 1992: 70 [checklist]. —Keiper et al. 1998a: 256 [use of algae in case construction]. —Keiper and Foote 2000: 226 [biology; distribution]. —Armitage et al. 2011: 14 [checklist].

**Distribution.** —U.S.A.

***xena*** (Ross, 1938a): 122 [type locality: [United States], Illinois, Herod, along Gibbons Creek; INHS; ♂; in *Polytrichia*]. —Ross 1944: 130 [♂; ♀; distribution]. —Blickle and Denning 1977: 288 [checklist]. —Blickle 1979: 53, 79 [checklist; ♂]. —Waltz and McCafferty 1983a: 11 [distribution]. —Bowles and Mathis 1989: 239 [distribution]. —Moulton and Stewart 1996: 121 [♂; distribution]. —Keiper and Foote 1998: 594 [biology; distribution]. —Keiper 1999: 231 [larva]. —Etnier 2010: 486 [distribution]. —Armitage et al. 2011: 14 [checklist].

**Distribution.** —U.S.A.

***yanayacuana*** Bueno-Soria & Santiago-Fragoso, 1992: 443 [type locality: Ecuador, Tungurahua Prov., Yanayacu; NMNH; ♂]. —Ríos-Touma et al. 2017: 10 [checklist].

**Distribution.** —Ecuador.

***yavapai*** Ruiter & Harris, 2015: 327 [type locality: [United States], Arizona, Yavapai County, Red Tank Draw on FR 618, ephemeral stream near Beaver Creek, Ranger Station N34° 40.7', W111° 43.4'; CAS; ♂].

**Distribution.** —U.S.A.

***yavesia*** Bueno-Soria & Holzenthal, 2004: 256 [type locality: Mexico, Oaxaca, Santa Maria de Yavesia (water plant), 17°13'36"N, 96°25'35"W, 1930 m; CNIN; ♂]. —Bueno-Soria 2009: 158 [♂]. —Razo-González et al. 2020: 5 [distribution].

**Distribution.** —Mexico.

***yepachica*** Harris in Harris and Moulton 1993: 548 [type locality: Mexico, Chihuahua, Río Concheno at Hwy 16, 12 km SW Yepachic; NMNH; ♂]. —Bueno-Soria 2009: 128 [♂].

**Distribution.** —Mexico.

***yetla*** Bueno-Soria, 2009: 129 [type locality: Mexico, Oaxaca, San Mateo Yetla, 17°.75N, 96°.4W, 840 m; CNIN; ♂].

**Distribution.** —Mexico.

***zihuaquia*** Bueno-Soria & Santiago-Fragoso, 1997: 362 [type locality: Mexico, Guerrero, route 134, 102 km N.W. Zihuatanejo; CNIN; ♂]. —Bueno-Soria 2009: 132 [♂].

**Distribution.** —Mexico.

***zioni*** Denning & Blickle, 1972: 143 [type locality: [United States], Zion Canyon National Park, Utah; CAS; ♂]. —Blickle and Denning 1977: 288 [checklist]. —Blickle 1979: 53, 81 [checklist; ♂].

**Distribution.** —U.S.A.

#### Genus *Ragatrichia* Oláh & Johanson, 2011

*Ragatrichia* Oláh & Johanson, 2011: 239 [type species: *Ragatrichiaragada* Oláh & Johanson, 2011, original designation].

The genus *Ragatrichia* includes five species recorded from French Guiana and Argentina. The original description placed the genus near *Metrichia* (Oláh and Johanson 2011). Differences in the form of the male genitalia are used to separate the two genera; the presence of a “harpago” on the inferior appendage of *Ragatrichia* male genitalia is unique within Hydroptilidae (Oláh and Johanson 2011). Larvae have been described for *R.yatay* as *Rhyacopsyche* (Angrisano 2002).

***angrisanae*** Oláh & Johanson, 2011: 240 [type locality: French Guiana, Approuaguekaw, Kaw Mt, 4°33.035'N, 52°11.661'W, 66 m; NHRS; ♂].

**Distribution.** —French Guiana.

***dietzi*** (Flint, 1974b): 63 [type locality: Guyana, Rockstone, Essequibo River; NMNH; ♂; in *Ochrotrichia*]. —Oláh and Johanson 2011: 241 [to *Ragatrichia*; distribution].

**Distribution.** —French Guiana, Guyana, Suriname.

***garuhape*** (Angrisano & Sganga, 2009): 63 [type locality: Argentina, Misiones: Parque Provincial Salto Encantado; MACN; ♂ in *Rhyacopsyche*]. —Oláh and Johanson 2011: 242 [to *Ragatrichia*].

**Distribution.** —Argentina.

***ragada*** Oláh & Johanson, 2011: 242 [type locality: French Guiana, Approuaguekaw, Kaw Mt, 4°33.035'N, 52°11.661'W, 104 m; NHRS; ♂].

**Distribution.** —French Guiana.

***yatay*** (Angrisano, 1989): 157 [type locality: Argentina, Entre Rios, Parque Nacional El Palmar; MACN; ♂; ♀; in *Rhyacopsyche*]. —Angrisano 1999: 35 [checklist]. —Angrisano 2002: 402 [larva; pupa; case; biology; taxonomic remarks]. —Angrisano and Sganga 2007: 31 [♂; larva; distribution]. —Wasmund and Holzenthal 2007: 21 [♂; illustrations after Angrisano 1989]. —Oláh and Johanson 2011: 243 [to *Ragatrichia*].

**Distribution.** —Argentina.

#### Genus *Rhyacopsyche* Müller, 1879

*Rhyacopsyche* Müller, 1879b: 40 [*nomen nudum*]; 1879: 143 [type species: *Rhyacopsychehagenii* Müller, 1879b, monotypic]. —Müller 1880b: 121. —Müller 1880a: 72. —Flint 1971: 516 [definition; revision]. —Marshall 1979b: 187 [generic review]. —Flint 1991b: 59 [key to Antioquian species]. —Angrisano 2002: 403 [taxonomic remarks]. —Wasmund and Holzenthal 2007: 1 [revision; key to species]. —Oláh and Johanson 2011: 203 [re-description].

*Rhyacopsyche* includes 30 species occurring primarily in Central and South America. The genus was originally established for a single Brazilian species that was described solely on the basis of larval cases. The species was subsequently named *hagenii* (Müller 1879b). Descriptions of the adults and larvae were first published by Thienemann (1905). According to Flint’s (1971) description of the genus, the larvae of *Rhyacopsyche* are very similar to those of both *Ochrotrichia* and *Hydroptila*, and the adults are similar to *Metrichia* and can only be separated using features of the male and female genitalia. Larvae have been described for *R.hagenii* (Thienemann, 1905), *R.mexicana* (Flint, 1971), and *R.mutisi* (Mey and Joost 1990); a generic diagnosis of the larva was provided by Wasmund and Holzenthal (2007).

***andina*** Flint, 1991b: 61 [type locality: Colombia, Dpto. Antioquia, Quebrada la Agudelo, 2 km E El Retiro; NMNH; ♂]. —Flint 1996c: 397 [distribution]. —Muñoz-Quesada 2000 [278 [checklist]. —Wasmund and Holzenthal 2007: 7 [♂; distribution].

**Distribution.** —Colombia, Peru, Venezuela.

***angra*** Santos, Jardim, & Nessimian, 2011: 817 [type locality: Brazil, Rio de Janeiro, Angra dos Reis, 23°00'23"S 44°29'15"W, 40 m; DZRJ; ♂]. —Paprocki and França 2014: 54 [checklist].

**Distribution.** —Brazil.

***benwa*** Wasmund & Holzenthal, 2007: 8 [type locality: Peru, Madre de Dios, Manu, Pakitza, 250 m; MHNJP; ♂]. —Ríos-Touma et al. 2017: 11 [checklist].

**Distribution.** —Bolivia, Ecuador, Peru.

***bulbosa*** Wasmund & Holzenthal, 2007: 8 [type locality: Brazil, Rio de Janeiro, Nova Friburgo, municipal water supply, 950 m; MZUSP; ♂]. —Dumas et al. 2009: 366 [distribution]. —Calor 2011: 321 [checklist]. —Dumas and Nessimian 2012: 15 [checklist]. —Paprocki and França 2014: 54 [checklist].

**Distribution.** —Brazil.

***bunkotala*** Oláh & Johanson, 2011: 244 [type locality: Ecuador, Wild Sumaco, near Pacto Sumaco; Collection Oláh; ♂]. —Ríos-Touma et al. 2017: 11 [checklist].

**Distribution.** —Ecuador.

***chichotla*** Bueno-Soria & Hamilton, 1986: 303 [type locality: Mexico, Oaxaca, 7 km NE Huautla de Jimenez; NMNH; ♂]. —Wasmund and Holzenthal 2007: 9 [♂; re-description].

**Distribution.** —Mexico.

***colei*** Wasmund & Holzenthal, 2007: 9 [type locality: Venezuela, Lara, Parque Nacional Dinira, Quebrada Buenos Aires, 09°36'24"N 70°04'11"W, 1850 m; UMSP; ♂].

**Distribution.** —Venezuela.

***colombiana*** Wasmund & Holzenthal, 2007: 10 [type locality: Colombia, Valle Del Cauca, Municipio El Cerrito, Rio Cerrito, 7.1 km E. Hacienda “El Paraiso”, 03°38'59"N 76°09'10"W, 1950 m; UMSP; ♂].

**Distribution.** —Colombia.

***colubrinosa*** Wasmund & Holzenthal, 2007: 11 [type locality: Peru, Cuzco, Paucartambo to Pilcopata Rd., streamlet 50 m E Quiacalzón, 13°01.57'S, 71°29.97'W, 1050 m; MHNJP; ♂; ♀]. —Oláh and Johanson 2011: 244 [checklist]. —Ríos-Touma et al. 2017: 11 [checklist].

**Distribution.** —Ecuador, Peru.

***diacantha*** Santos, Jardim, & Nessimian, 2011: 815 [type locality: Brazil, Pará, PArauapebas (Área de Proteção Ambiental do Igarapé Gelado, Barragem do Gelado, 05°57'56"S 50°13'00"W, 224 m; DZRJ; ♂; ♀]. —Paprocki and França 2014: 54 [checklist].

**Distribution.** —Brazil.

***dikrosa*** Wasmund & Holzenthal, 2007: 11 [type locality: Brazil, São Paulo, Pedregulho, 140 km NE Ribeirão Preto; MZUSP; ♂; ♀]. —Dumas et al. 2009: 366 [distribution]. —Calor 2011: 321 [checklist]. —Dumas and Nessimian 2012: 16 [checklist]. —Paprocki and França 2014: 55 [checklist].

**Distribution.** —Brazil.

***duplicispina*** Flint, 1996b: 91 [type locality: Tobago, Bridge B1/5, 6.5 km N Roxborough; NMNH; ♂]. —Botosaneanu 2002b: 89 [checklist]. —Wasmund and Holzenthal 2007: 12 [♂; re-description].

**Distribution.** —Tobago.

***flinti*** Wasmund & Holzenthal, 2007: 13 [type locality: Venezuela, Guárico, Parque Nacional Guatopo, Queb. Guatopo, 0.5 km N Est. La Colina, 10°0'50"N 66°21'47"W, 600 m; UMSP; ♂; ♀].

**Distribution.** —Venezuela.

***hagenii*** Müller, 1879a: 143 [type locality: Brazil; type depository unknown; larval and pupal cases]. —Thienemann 1905: 287 [larva; ♂]. —Müller 1921: 525 [larva]. —Ulmer 1957: 172, 187 [literature; key to larval genus]. —Angrisano 1995a: 509 [distribution]. —Angrisano 1999: 35 [checklist]. —Paprocki et al. 2004: 12 [checklist]. —Wasmund and Holzenthal 2007: 6 [♂; ♀; distribution]. —Dumas et al. 2009: 366 [distribution]. —Calor 2011: 321 [checklist]. —Dumas and Nessimian 2012: 16 [checklist]. —Paprocki and França 2014: 55 [checklist].

**Distribution.** —Argentina, Brazil, Uruguay.

***hajtoka*** Oláh & Johanson, 2011: 245 [type locality: Ecuador, Alambi; Collection Oláh; ♂]. —Ríos-Touma et al. 2017: 11 [checklist].

**Distribution.** —Ecuador.

***hasta*** Wasmund & Holzenthal, 2007: 13 [type locality: Peru, Cuzco, Paucartambo to Pilcopata rd., streamlet 50 m E Quiacalzón, 13°01.57'S, 71°29.97'W, 1050 m; MHNJP; ♂]. —Oláh and Johanson 2011: 247 [checklist].

**Distribution.** —Peru.

***holzenthali*** Harris & Armitage, 2019: 17 [type locality: Panama, Bocas del Toro Province, Quebrada Rambala, near Rambala Jungle Lodge, 3.74 km SSE Rambala, 8.91627°N and 82.15469°W, 120 m; COZEM; ♂]. —Armitage and Harris 2020a: 8 [distribution].

**Distribution.** —Panama.

***intraspira*** Wasmund & Holzenthal, 2007: 14 [type locality: Peru, Cuzco, Paucartambo to Pilcopata rd., Rio San Pedro at Puente San Pedro, 13°03.30'S, 71°32.78'W, 1445 m; MHNJP; ♂].

**Distribution.** —Peru.

***jimena*** Flint, 1991b: 59 [type locality: Colombia, Dpto. Antioquia, Quebrada la Jimenez, Sopetran; NMNH; ♂]. —Muñoz-Quesada 2000: 278 [checklist]. —Wasmund and Holzenthal 2007: 14 [♂; re-description].

**Distribution.** —Colombia.

***matthiasi*** Flint, 1991: 61 [type locality: Colombia, Dpto, Antioquia, Urrao; NMNH; ♂]. —Muñoz-Quesada 2000: 278 [checklist]. —Wasmund and Holzenthal 2007: 15 [♂; re-description]. —Mey and Ospina-Torres 2018: 30 [distribution].

**Distribution.** —Colombia.

***mexicana*** (Flint, 1967b): 12 [type locality: Mexico, Veracruz, Rio Tacolapan; NMNH; ♂; in *Metrichia*]. —Flint 1971: 519 [♂; ♀; larva; case; distribution; to *Rhyacopsyche*]. —Bueno-Soria and Flint 1978: 205 [distribution]. —Maes 1999: 1195 [checklist]. —Bueno-Soria et al. 2005: 75 [distribution]. —Chamorro-Lacayo et al. 2007: 44 [checklist]. —Wasmund and Holzenthal 2007: 15 [♂; re-description].

**Distribution.** —Costa Rica, Guatemala, Mexico, Nicaragua.

***mutisi*** Mey & Joost, 1990: 134 [type locality: Colombia, Dept, Tolima, Mariquita, Rio Medina; KMUL; ♂; ♀; larva; case]. —Muñoz-Quesada 2000: 278 [checklist]. —Wasmund and Holzenthal 2007: 16 [♂; re-description].

**Distribution.** —Colombia.

***obliqua*** Flint, 1971: 523 [type locality: Mexico, Veracruz, Fortin de las Flores; NMNH; ♂; ♀]. —Bueno-Soria and Flint 1978: 205 [distribution]. —Wasmund and Holzenthal 2007: 16 [♂; re-description]. —Armitage and

2018a: 9 [distribution]. —Armitage and Harris 2018b: 99 [checklist]. —Armitage and Harris 2018c: 284 [distribution].

**Distribution.** —Mexico, Panama.

***peruviana*** Flint, 1975: 568 [type locality: South-Peru, Sivia; ZSZMH; ♂; ♀]. —Wasmund and Holzenthal 2007: 18 [♂; re-description; distribution]. —Ríos-Touma et al. 2017: 11 [checklist].

**Distribution.** —Ecuador, Peru.

***rhamphisa*** Wasmund & Holzenthal, 2007: 19 [type locality: Colombia, Valle, Del Cauca, Municipio El Cerrito, Rio Cerrito, 7.1 kms E. Hacienda “El Paraiso”. 03°38'59"N 076°09'10"W, 1950 m; UMSP; ♂; ♀]. —Oláh and Johanson 2011: 248 [distribution]. —Armitage et al. 2018: 5 [distribution; as *ramphisa*].

**Distribution.** —Colombia, Costa Rica, Panama, Peru.

***shorti*** Thomson & Holzenthal, 2012: 36 [type locality: Venezuela, Bolívar, Gran Sabana, E. Pauji, “Río Curvita”, 4°31.237'N, 61°31.591'W, 869 m; UMSP; ♂]. —Morse 2016 [listed as *Angrisanoia* on the Trichoptera World Checklist].

**Distribution.** —Venezuela.

***tanylobosa*** Wasmund & Holzenthal, 2007: 19 [type locality: Venezuela, Barinas, Parque Nacional Sierra Nevada, Queb. San Juan in Sta. Rosa, 08°27.87'N, 070°50.92'W, 1000 m; UMSP; ♂; ♀]. —Ríos-Touma et al. 2017: 11 [distribution].

**Distribution.** —Ecuador, Peru, Venezuela.

***torulosa*** Flint, 1971: 521 [type locality: Guatemala, Escuintla, Rio Metapa, 10 km SE Escuintla; NMNH; ♂; ♀]. —Holzenthal 1988: 63 [distribution]. —Wasmund and Holzenthal 2007: 20 [♂; re-description]. —Armitage et al. 2018: 5 [distribution]. —Bueno-Soria and Barba-Alvarea 2018: 367 [distribution].

**Distribution.** —Costa Rica, Guatemala, Mexico, Panama.

***totuma*** Thomson & Armitage, 2018: 5 [type locality: Panama, Chiriquí Province, Cuenca 102 (Río Chiriquí Viejo), Quebrada Norte, Mount Totumas Biological Reserve, 8.873613°N, 82.690512°W; COZEM; ♂].

**Distribution.** —Panama.

***turrialbe*** Flint, 19751: 523 [type locality: Costa Rica, Cartago, Citaria; NMNH; ♂; ♀]. —Holzenthal 1988: 63 [distribution]. —Wasmund and Holzenthal 2007: 21 [♂; re-description]. —Armitage et al. 2018: 5 [distribution].

**Distribution.** —Costa Rica, Panama.

### ﻿Subfamily ORTHOTRICHIINAE Nielsen, 1948

Orthotrichiini Nielsen, 1948: 186 [type genus: *Orthotrichia* Eaton, 1873]. —Marshall 1979b: 212 [reviewed as tribe Orthotrichiini].

While currently containing three genera, the subfamily Orthotrichiinae was originally established for *Orthotrichia* and *Ithytrichia* based on several morphological and behavioral affinities of the larvae (Nielsen 1948). However, as noted by Marshall (1979b), the adults and larvae of each of these two genera are very distinct from one another and they may later be found to form distinct groups of their own. The third genus, *Saranganotrichia*, shows closest morphological similarities to *Ithytrichia*, with which it was synonymized by Marshall (1979b). Larval descriptions have been provided for all genera.

#### Genus *Ithytrichia* Eaton, 1873

*Ithytrichia* Eaton, 1873: 139 [type species: *Ithytrichialamellaris* Eaton, 1873, original designation]. —McLachlan 1880: 514 [revision]. —Ross 1944: 123 [revision of North American species]. —Marshall 1979b: 216 [generic review]. —Blickle 1979: 6 [key to species of America north of Mexico]. —Botosaneanu 1992: 109 [key to species in the Levant]. —Moulton and Stewart 1996: 107 [key to species of the Interior Highlands of North America]. —Moulton et al. 1999: 241 [key to North American species]. —Kachalova in Medvedev 1998: 188 [key to the species of the European part of the USSR]. —Rueda Martín 2006: 252 [distribution].

The genus *Ithytrichia* consists of seven species occurring in a primarily Holarctic distribution, but with a single species recorded from northcentral Mexico and another from northwestern Argentina. Similarities shared between the larvae, including features of the mandibles, thoracic sternites, and the fore-coxae, led Nielsen (1948) to conclude that the genera *Ithytrichia* and *Orthotrichia* were closely related. Detailed larval descriptions have been given for *I.lamellaris* (Nielsen 1948) and *I.ferni* (Rueda Martín 2006), and the North American species were reviewed by Wiggins (1996).

***aquila*** González & Malicky, 1988: 66 [type locality: Espagne, Prov. Càdiz, Puente de la Terrona, Rio Guadalete, 360 m; depository not designated; ♂]. —González et al. 1990: 212 [checklist]. —Malicky 2004a: 65 [atlas]. —Malicky 2005b: 545 [checklist]. —González and Menéndez 2011: 119 [distribution].

**Distribution.** —Spain.

***bosniaca*** Murgoci, Botnariuc, & Botosaneanu, 1948: 219 [type locality: [Bosnia]; depository not designated; larva]. —Malicky 1979: 9 [♂; distribution]. —Malicky 1983b: 53 [atlas; ♂]. —Cianficconi et al. 1999: 278 [distribution]. —Cianficconi et al. 2002: 146 [distribution]. —Sipahiler 2003b: 33 [distribution]. —Malicky 2004a: 65 [atlas]. —Malicky 2005b: 545 [checklist]. —Sipahiler 2005: 397 [distribution]. —Malicky 2005a: 65 [distribution]. —Corallini and Cianficconi 2011: 628 [checklist]. —Malicky 2014b: 6 [teratological structures]. —Karaouzas and Malicky 2015: 14 [distribution]. —Oláh 2017: 136 [distribution].

**Distribution.** —Bosnia, Greece, Italy, Turkey.

***clavata*** Morton, 1905: 67 [type locality: [United States], Ithaca, New York; depository not designated; ♂]. —Banks 1907a: 50 [catalogue]. —Tjeder 1930a: 135 [♂; distribution]. —Betten 1934: 156 [♂; distribution]. —Kimmins 1943: 155; 156 [distribution; ♂]. —Ross 1944: 124 [♂; ♀; distribution]. —Denning 1947b: 171 [distribution]. —Ross and Spencer 1952: 46 [distribution]. —Morse and Blickle 1953: 72 [checklist]. —Nybom 1960: 18 [distribution]. —Etnier 1965: 147 [checklist]. —Botosaneanu 1967: 293 [distribution]. —Cloud and Stewart 1974: 806 [biology; distribution]. —Roy and Harper 1975: 1082 [distribution]. —Botosaneanu and Malicky 1978: 340 [checklist]. —Roy and Harper 1979: 152 [checklist]. —Blickle 1979: 50, 57 [checklist; ♂]. —Parker and Voshell 1981: 4 [checklist]. —O’Connor 1982: 548 [distribution]. —Unzicker et al. 1982: 9 [checklist]. —Huryn and Foote 1983: 791 [distribution]. —Malicky 1983b: 53 [atlas; ♂]. —Hamilton et al. 1983: 18 [distribution]. —González and Otero 1983: 118 [distribution]. —Andersen and Wiberg-Larsen 1987: 169 [checklist]. —Malicky and Lounaci 1987: 14, 15, 17 [checklist]. —Spuris 1989: 16 [distribution]. —González et al. 1990: 212 [checklist]. —Masteller and Flint 1992: 70 [checklist]. —Mathis and Bowles 1992: 24 [distribution]. —Bowles and Mathis 1992: 32 [distribution]. —Moulton et al. 1993: 21 [distribution]. —Moulton et al. 1994: 170 [distribution]. —Moulton and Stewart 1996: 107 [♂; larva; distribution]. —Moulton and Stewart 1997: 350 [checklist]. —Houghton and Stewart 1998: 106 [biology; distribution]. —Moulton et al. 1999: 234 [♂; ♀; distribution]. —Huryn and Harris 2000: 193 [distribution]. —Houghton et al. 2001: 504 [distribution]. —Valle 2001: 65 [distribution]. —Gullefors 2002: 138 [distribution]. —Andersen and Kjærandsen 2002: 93 [distribution]. —Mirmoayedi and Malicky 2002: 164 [distribution]. —Malicky 2004a: 65 [atlas]. —Blinn and Ruiter 2005: 69 [distribution; biology]. —Malicky 2005b: 545 [checklist]. —Berlin 2005: 129 [distribution]. —Lubini-Ferlin and Vicentini 2005: 67 [checklist]. —Bowles et al. 2007: 21 [distribution; biology]. —Robert 2007: 83 [checklist]. —Berlin and Thiele 2007: 49 [checklist]. —Gullefors 2008: 64 [checklist]. —Blinn and Ruiter 2009b: 186 [distribution]. —Etnier 2010: 485 [distribution]. —Hinchliffe 2010: 467 [distribution]. —Corallini and Cianficconi 2011: 628 [checklist]. —Armitage et al. 2011: 14 [checklist]. —Myers et al. 2011: 107 [distribution]. —González and Menéndez 2011: 119 [distribution]. —Ivanov 2011: 195 [checklist]. —Harris et al. 2012: 8 [checklist]. —O’Connor and O’Connor 2013: 189 [distribution]. —O’Connor 2013: 64 [distribution]. —Martín et al. 2015: 74 [distribution]. —O’Connor 2015: 28 93 [distribution]. —Gullefors 2016: 155 [checklist]. —Ruiz-García et al. 2016: 4 [distribution]. —Wallace 2016: 21, 23, 55 [conservation status]. —Houghton et al. 2017: 63 [checklist]. —Mendez et al. 2019: 118 [checklist]. —Mabrouki et al. 2020: 13 [distribution].

—*dovporiana* Botosaneanu, 1980: 74 [type locality: Israel, Arugot, dans le dépression de la Mer Morte; ZMUA; ♂; pupa]. —Botosaneanu 1982b: 11 [habitat threat]. —Botosaneanu 1992: 111 [♂; ♀]. —Ruiz-García 1995: 203 [distribution]. —Malicky 2005b: 545 [to synonymy].

**Distribution.** —Canada, England, Finland, Germany, Iran, Ireland, Israel, Italy, Morocco, Portugal, Russia, Spain, Sweden, U.S.A.

***ferni*** Rueda Martín, 2006: 252 [type locality: Argentina, Tucumán Prov., Tafí Viejo, Río Tafí, 26°43'25"S 64°17'26"W, 827 m; IFML; ♂; larva; pupa; case; biology]. —Isa Miranda and Rueda Martín 2014: 199 [distribution].

**Distribution.** —Argentina.

***lamellaris*** Eaton, 1873: 140 [type locality: [England], the Sandy Brook, near Hanging Bridge, Ashbourne, Derbyshire; NHMUK; ♂]. —Klapálek 1897: 6 [larva]. —McLachlan 1880: 515 [revision; ♂; ♀]. —Morton 1888: 171 [larva; case]. —Ris 1897: 431 [distribution]. —Morton 1899b: 281 [distribution]. —Richters 1902: 19 [larva]. —Morton 1904: 325 [distribution]. —Lundblad 1918: 342 [distribution; ecology]. —Martynov 1924: 47 [♂; ♀]. —Tjeder 1930a: 136 [♂; distribution]. —Martynov 1934: 145 [♂; ♀]. —Mosely 1939b: 275 [♂]. —Kimmins 1943: 155 [distribution]. —Nielsen 1948: 114 [larva]. —Kimmins 1957a: 108 [lectotype designation]. —Nybom 1960: 18 [distribution]. —Botosaneanu 1967: 293 [distribution]. —Solem 1970b: 93 [distribution]. —Botosaneanu and Gasith 1971: 98 [distribution]. —Fahy 1972: 202 [distribution]. —Spuris 1972: 28, 30 [checklist]. —Szczęsny 1975: 41 [distribution]. —Botosaneanu and Malicky 1978: 340 [distribution]. —Kumanski 1979: 6 [♂; distribution]. —Malicky 1983b: 53 [atlas; ♂; ♀]. —Kumanski and Malicky 1984: 199 [distribution]. —Kumanski 1985: 152 [♂]. —Andersen and Tysse 1985: 84 [distribution]. —Wiberg-Larsen 1985: 40 [checklist]. —Moubayed and Botosaneanu 1985: 63 [distribution]. —Andersen and Wiberg-Larsen 1987: 169 [checklist]. —Sipahiler and Malicky 1987: 129 [distribution]. —Spuris 1989: 16 [distribution]. —Waringer 1989: 390 [distribution; ecology]. —Usseglio-Polatera and Bournaud 1989: 253 [distribution]. —Andersen et al. 1990: 52 [distribution]. —González et al. 1990: 212 [checklist]. —Botosaneanu 1992: 109 [♂; ♀]. —Andersen et al. 1993b: 3 [distribution]. —Andersen et al. 1993a: 51 [distribution]. —Maier et al. 1995: 148 [distribution]. —Bagge 1995: 94 [distribution; biology]. —Nógradi and Uherkovich 1994: 31 [distribution]. —Uherkovich and Nógradi 1997: 461 [distribution]. —Peissner and Kappus 1998: 162 [distribution]. —Uherkovich and Nógradi 1999: 420 [distribution]. —Malicky 1999c: 96 [distribution]. —Cianficconi et al. 1999: 57 [distribution]. —Uherkovich and Nógradi 2001: 94 [distribution]. —Nógrádi and Uherkovich 2001: 297 [checklist]. —Valle 2001: 64 [distribution]. —Gullefors 2002: 138 [distribution]. —Nógrádi and Uherkovich 2002: 130 [distribution]. —Ujvárosi 2002: 384 [distribution]. —Cibaitė 2003a: 10 [checklist]. —Gullefors 2003: 195 [distribution]. —Arefina 2004: 211 [distribution]. —Malicky 2004a: 65 [atlas]. —Malicky 2005b: 545 [checklist]. —Sipahiler 2005: 397 [distribution]. —Berlin 2005: 127, 129 [distribution]. —Gullefors 2005b: 138 [distribution]. —Hohmann 2005: 106 [checklist]. —Coppa and Tachet 2005: 132 [distribution]. —Malicky 2005a: 66 [distribution]. —Graf et al. 2005: 55 [distribution]. —Lubini-Ferlin and Vicentini 2005: 68 [checklist]. —Beketov 2006: 14 [distribution]. —Voigt et al. 2006: 73 [distribution]. —Chvojka and Komzák 2006: 358 [distribution]. —Robert 2007: 83 [checklist]. —Berlin and Thiele 2007: 49 [checklist]. —Dohet et al. 2008: 46 [distribution; ecology]. —Schrankel et al. 2008: 90 [distribution]. —Chvojka and Komzák 2008: 13 [distribution]. —Szczęsny and Godunko 2008: 15 [checklist]. —Flint and Thomas 2008: 40 [distribution]. —Ujvárosi et al. 2008: 112 [distribution]. —Višinskienė 2009: 27 [checklist]. —Hohmann 2010: 40 [distribution]. —Waringer and Graf 2011: 281 [larval synopsis]. —Ivanov 2011: 195 [checklist]. —González and Menéndez 2011: 119 [distribution]. —Skuja 2011: 425 [distribution; ecology]. —Crofts 2011: 72 [distribution]. —Timm et al. 2011: 408 [distribution]. —Viidalepp et al. 2011: 196 [distribution]. —Komzák and Chvojka 2012: 720 [distribution]. —Andersen and Hagenlund 2012: 136 [distribution]. —O’Connor 2013: 64 [distribution]. —Tempelman et al. 2013: 288 [distribution]. —Drescher 2013: 53 [distribution; biology]. —Corallini et al. 2013: 26 [distribution]. —Tempelman and Sanabria 2013b: 144 [distribution]. —O’Connor and O’Connor 2014: 273 [distribution]. —Martín et al. 2014: 72 [distribution]. —Hohmann et al. 2014: 85 [distribution]. —Martínez et al. 2015: 40 [distribution]. —Stojanović et al. 2015: 52 [larva; case; distribution; ecology]. —O’Connor 2015: 28, 94 [distribution]. —Dia 2015: 51 [distribution]. —Martínez et al. 2016: 52 [distribution]. —O’Connor and O’Connor 2016: 166 [distribution]. —Pan’kov and Krasheninnikov 2016: 333 [distribution]. —Küttner et al. 2016: 179 [distribution]. —D. Smirnova et al. 2016: 401 [distribution]. —van Haaren et al. 2016: 10 [distribution]. —Potikha and Vshivkova 2016: 364 [distribution]. —Gullefors 2016: 155 [checklist]. —O’Connor and O’Connor 2017b: 53 [distribution]. —O’Connor and O’Connor 2018: 82 [distribution]. —Gullefors 2018: 108 [biology; distribution]. —Cerjanec et al. 2020: 13 [distribution]. —Edmonds-Brown 2020: 91 [checklist]. —Kroča and Komzák 2020: 147 [distribution]. —O’Connor 2020: 140 [distribution].

—*brunneicornis* Pictet, 1834: 226 [type locality: [Switzerland]; no holotype designated; in *Hydroptila*]. —Fischer 1961: 104 [considered a junior synonym to either *Ithytrichialamellaris* or *Orthotrichiaangustella*].

**Distribution.** —Austria, Belarus, Bulgaria, Croatia, Czech Republic, Denmark, England, Estonia, Finland, France, Germany, Greece, Hungary, Ireland, Israel, Italy, Kazakhstan, Latvia, Lebanon, Luxembourg, Netherlands, Norway, Poland, Portugal, Romania, Russia, Scotland, Serbia, Spain, Sweden, Switzerland, Turkey, Ukraine.

***mazon*** Ross, 1944: 124 [type locality: [United States], Illinois, Mazon, along Mazon Creek; INHS; ♂]. —Blickle 1979: 50, 57 [checklist; ♂]. —Moulton and Stewart 1996: 107 [♂; distribution]. —Moulton et al. 1999: 236 [♂; ♀; distribution]. —Armitage et al. 2011: 14 [checklist].

**Distribution.** —U.S.A.

***mexicana*** Harris & Contreras-Ramos, 1989: 176 [type locality: Mexico, Tamaulipas, Rio Frio, 6 km S Gomez Farias; NMNH; ♂]. —Moulton et al. 1999: 236 [♂; ♀; distribution]. —Houghton 2001: 90 [distribution]. —Blinn and Ruiter 2005: 69 [distribution; biology]. —Blinn and Ruiter 2006: 332 [biology; distribution]. —Blinn and Ruiter 2009a: 303 [biology]. —Blinn and Ruiter 2009b: 186 [phenology; distribution]. —Vieira et al. 2009: 257 [distribution]. —Razo-González 2018: 32 [distribution]. —Mendez et al. 2019: 128 [checklist].

**Distribution.** —Mexico, U.S.A.

#### Genus *Orthotrichia* Eaton, 1873

*Orthotrichia* Eaton, 1873: 141 [type species: *Hydroptilaangustella* McLachlan, 1865, original designation]. —McLachlan 1880: 518 [revision]. —Mosely 1939: 276 [key to the British species]. —Ross 1944: 139 [revision of North American species; key to Nearctic species]. —Kingsolver and Ross 1961: 28 [revision of North American species]. —Disney 1972: 84 [larvae observed preying on Simuliidae]. —Marshall 1979b: 213 [generic review]. —Blickle 1979: 7 [key to species of America north of Mexico]. —Wells 1979a: 587 [key to males of Australian species]. —Wells 1984: 271 [key to males from New Guinea and New Britain]. —Wells 1985b: 26 [larva; pupa; case]. —Wells 1990b: 395 [key to North Sulawesi species]. —Wells 1991: 491 [key to males of New Guinea]. —Botosaneanu 1992: 99 [key to species in the Levant], —Moulton and Stewart 1996: 122 [key to species of the Interior Highlands of North America]. —Kachalova in Medvedev 1998: 182 [key to the species of the European part of the USSR].

*Clymene* Chambers, 1873: 114 [type species: *Clymeneaegerfasciella* Chambers, 1873, monotypic]. —Flint 1966: 135 [to synonymy].

*Javanotrichia* Ulmer, 1951: 75 [type species: *Javanotrichiamaeandrica* Ulmer, 1951, original designation]. —Marshall 1979b: 213 [to synonymy].

*Orthotrichiella* Ulmer, 1951: 79 [type species: *Orthotrichiellaranauana* Ulmer, 1951, original designation]. —Marshall 1979b: 213 [to synonymy].

*Baliotrichia* Ulmer, 1951: 88 [type species: *Baliotrichialitoralis* Ulmer, 1951, original designation]. —Marshall 1979b: 213 [to synonymy].

*Targatrichia* Neboiss, 1977: 41 [type species: *Targatrichiazonata* Neboiss, 1977, original designation]. —Wells 1979a: 591 [transferred sole species to *Orthotrichia*].

The large, cosmopolitan genus *Orthotrichia* consists of 272 species, including a single fossil species, and is particularly species-rich in Southeast Asia, Australia, and Africa. Marshall (1979b) commented on the characteristic asymmetrical genitalia of the males and divided the genus into four species groups (*angustella*, *litoralis*, *costalis*, and *aegerfasciella*), with the possibility of a fifth (*kokodana*). Wells (1992, 2005) observed one species group of *Orthotrichia* occurring within the pupal cases of various hydropsychid and philopotamid species and concluded that the *Orthotrichia* larvae are preying upon the “host” pupae; the species involved appear to have an early fifth instar larval stage and case distinct from the final stage. Larval descriptions are given for *O.costalis* (Nielsen 1948), *O.angustella* (Jacquemart 1962a), and several others (Ulmer 1957; Wells 1985b, 1992, 2005; Wiggins 1996).

***aberrans*** Wells, 1979a: 621 [type locality: [Australia] Victoria, Mitta Mitta River, 8 km NE. Benambra; NMV; ♂]. —Wells 1985b: 31 [larva, pupa, case]. —Neboiss 1986: 95 [atlas; ♂].

**Distribution.** —Australia.

***acina*** Wells, 2005: 390 [type locality: Australia, N Queensland, 18°57'S 146°10'E, Mt Spec State Forest, Williams Creek above mine, 650 m; NMV; ♂].

**Distribution.** —Australia.

***aculeata*** Wells, 1979a: 603 [type locality: [Australia] Western Australia, Spillway Creek, Ord River Dam; WAM; ♂]. —Neboiss 1986: 87 [atlas; ♂].

**Distribution.** —Australia.

***adornata*** Wells, 1979a: 590 [type locality: [Australia] Victoria, Millgrove, Yarra River; NMV; ♂; ♀]. —Neboiss 1986: 85 [atlas; ♂; ♀]. —Neboiss 2002: 55 [distribution, checklist].

**Distribution.** —Australia.

***advena*** Wells, 1984: 276 [type locality: [Papua] New Guinea, NE., Morobe District, Mt Missim, 1300 m; BPBM; ♂]. —Neboiss 1986: 89 [atlas; ♂]. —Wells 1991: 526 [checklist].

**Distribution.** —Papua New Guinea.

***aegerfasciella*** (Chambers, 1873): 114 [type locality: United States, Kentucky; sex unknown; in *Clymene*]. —Bueno-Soria and Flint 1978: 201 [distribution]. —Resh et al. 1978: 383 [distribution]. —Botosaneanu 1979: 49 [distribution]. —Etnier and Schuster 1979: 18 [checklist]. —Blickle 1979: 53, 59 [checklist; ♂]. —Parker and Voshell 1981: 4 [checklist]. —Harris et al. 1982a: 511 [distribution]. —Huryn and Foote 1983: 791 [distribution]. —Waltz and McCafferty 1983a: 11 [distribution]. —Hamilton et al. 1983: 18 [distribution]. —Harris et al. 1984: 109 [distribution]. —Lake 1984: 220 [distribution]. —Steven and Hilsenhoff 1984: 164 [distribution]. —Bowles and Mathis 1989: 239 [distribution]. —Morse et al.: 23 [distribution]. —Floyd and Schuster 1990: 130, 132 [distribution]. —Botosaneanu 1991: 132 [distribution]. —Harris et al. 1991: 237 [distribution]. —Frazer et al. 1991: 20 [distribution]. —Masteller and Flint 1992: 70 [checklist]. —Mathis and Bowles 1992: 24 [distribution]. —Bowles and Mathis 1992: 32 [distribution]. —Masteller 1993: 134 [distribution]. —Moulton et al. 1993: 21 [distribution]. —Floyd et al. 1993: 91 [phenology; distribution]. —Flint 1996a: 16 [checklist]. —Moulton and Stewart 1996: 122 [♂; distribution]. —Abbott et al. 1997: 44 [distribution]. —Moulton and Stewart 1997: 350 [checklist]. —Maes 1999: 1194 [checklist]. —Ruiter 1999: 166 [distribution]. —Flint and Pérez-Gelabert 1999: 41 [checklist]. —Houghton et al. 2001: 505 [distribution]. —Botosaneanu 2002b: 87 [checklist]. —Pescador et al. 2004: 133 [checklist]. —Flint and Sykora 2004: 40 [distribution]. —Naranjo López and González Lazo 2005: 149 [checklist]. —Zeullig et al. 2006: 43 [distribution]. —Bowles et al. 2007: 22 [distribution; biology]. —Chamorro-Lacayo et al. 2007: 43 [distribution]. —Pérez-Gelabert 2008: 301 [checklist]. —Flint 2011: 104 [checklist]. —Houghton et al. 2011b: 6 [distribution]. —Armitage et al. 2011: 14 [checklist]. —Myers et al. 2011: 108 [distribution]. —Harris et al. 2012: 9 [checklist]. —Wright et al. 2013: 466 [biology; distribution]. —Blinn and Ruiter 2013: 291 [biology; distribution]. —DeWalt et al. 2016: 52 [distribution]. —Denson et al. 2016: 5 [distribution]. —Houghton et al. 2017: 63 [checklist]. —Harris and Rasmussen 2019: 217 [♂; ♀; distribution]. —Bowles et al. 2020: 8 [distribution]. —Houghton and Lardner 2020: 42 [distribution].

—*americana* Banks, 1904b: 116 [type locality: United States, Washington, D. C.; MCZ; ♂]. —Banks 1904a: 216 [distribution]. —Banks 1907a: 50 [catalogue]. —Betten 1934: 151 [♂]. —Ross 1938b: 9 [lectotype desginated]. —Ross 1944: 140 [♂; ♀; distribution]. —Denning 1947a: 18 [distribution]. —Denning 1947b: 172 [distribution]. —Morse and Blickle 1953: 72 [checklist]. —Kingsolver and Ross 1961: 29 [♂; distribution]. —Etnier 1965: 147 [checklist]. —Flint 1966: 135 [to synonymy]. —Unzicker et al. 1970: 172 [distribution; as *Ochrotrichiaamericana*]. —Edwards 1973: 506 [distribution]. —Swegman et al. 1981: 139 [distribution].

—*dorsalis* (Banks, 1904a): 216 [type locality: United States, Washington, D. C.; Collection Banks; ♀; in *Oxyethira*]. —Banks 1907a: 50 [catalogue]. —Betten 1934: 161 [checklist]. —Ross 1938b: 10 [lectotype designated]. —Ross 1944: 140 [as synonym of *americana*]. —Kelley 1984a: 442 [*dorsalis* to *Oxyethira*].

—*brachiata* Morton, 1905: 70 [type locality: [United States], New York, Ithaca; depository not designated; ♂]. —Banks 1907a: 50 [catalogue]. —Betten 1934: 152 [checklist]. —Ross 1938: 9 [as synonym of *americana*].

**Distribution.** —Canada, Cuba, Dominican Republic, Haiti, Mexico, Nicaragua, U.S.A.

***aequatoriana*** Kimmins, 1957c: 15 [type locality: Uganda, Jinja; NHMUK; ♂]. —Johanson 1992: 118 [checklist]. —Wells and Andersen 1995: 145 [checklist]. —Guenda 1996: 245 [distribution].

**Distribution.** —Burkina Faso, Uganda.

***agtuuganonica*** Mey, 1998a: 552 [type locality: [Philippines, Mindanao], northern slope of Mt. Atuuganon range, 1050 m; ZMHB; ♂]. —Wells and Mey 2002: 134 [checklist].

**Distribution.** —Philippines.

***aiema*** Wells, 1991: 514 [type locality: Papua New Guinea, Central Province, Aime River, 9°25'S 147°35'E; ANIC; ♂].

**Distribution.** —Papua New Guinea.

***airterjun*** Malicky, Melnitsky, & Ivanov, 2014a: 833 [type locality: [Indonesia] Papua, Insel Biak, Warsa, Wafsarak Wasserfall, 0°47'39"S, 135°55'31"E, 50 m; ZIN; ♂].

**Distribution.** —Indonesia.

***alata*** Wells, 1990c: 123 [type locality: [Australia] Northern Territory, Kambolgie Creek, 13°32'S 132°23'E; NTM; ♂; case]. —Wells et al. 2019: 33 [detection frequency].

**Distribution.** —Australia.

***albuguttata*** Jacquemart, 1956: 4 [type locality: [Congo], Bukavu (au large); IRSNB; ♂; ♀]. —Jacquemart 1957: 124 [♂; distribution]. —Guenda 1996: 245 [distribution].

**Distribution.** —Burkina Faso, Congo.

***alveata*** Wells, 1979a: 610 [type locality: [Australia] Queensland, Mossman Gorge; ANIC; ♂]. —Neboiss 1986: 91 [atlas; ♂].

**Distribution.** —Australia.

***amgulil*** Oláh & Johanson, 2010a: 39 [type locality: India, Karnataka, Tunga River at Shimoga; Collection Oláh; ♂].

**Distribution.** —India.

***ammanensis*** Malicky, 1996b: 203 [type locality: Jordan, Amman; LNKD; ♂]. —Malicky 2004a: 69 [atlas]. —Malicky 2005b: 545 [checklist]. —Sipahiler 2005: 397 [distribution].

**Distribution.** —Jordan, Turkey.

***amnica*** Wells, 1990c: 119 [type locality: [Australia] Northern Territory, Kambolgie Creek, 13°32'S 132°23'E; NTM; ♂]. —Wells et al. 2019: 33 [detection frequency].

**Distribution.** —Australia.

***andicairnsae*** Wells, 2010a: 51 [type locality: [Australia] North Queensland, Fishery Falls, S of Cairns, 17°11'S 145°52'E; ANIC; ♂].

**Distribution.** —Australia.

***angustella*** (McLachlan, 1865): 95 [type locality: [England]; NHMUK; ♂; in *Hydroptila*]. —Eaton, 1873: 142 [♂; distribution; in *Orthotrichia*]. —McLachlan 1880: 519 [revision; ♂; ♀; in *Orthotrichia*]. —McLachlan 1884: 72 [distribution]. —Morton 1887: 202 [case]. —Morton 1888: 173 [case]. —Morton 1896: 104 [distribution]. —Morton 1904: 326 [distribution]. —Martynov 1924: 50 [♂]. —Ulmer 1929: 255 [morphological notes; comparison with *O.tetensii*]. —Martynov 1934: 123 [♂]. —Mosely 1939b: 277 [♂]. —Kimmins 1943: 155 [distribution]. —Schmid 1952: 652 [distribution]. —Kimmins 1957a: 107 [lectotype designation]. —Jacquemart and Coineau 1962: 49 [♂]. —Jacquemart 1962a: 1 [larva]. —Moretti et al. 1966: 88 [distribution; note on attraction to light]. —Botosaneanu 1967: 293 [distribution]. —Solem 1970b: 93 [distribution]. —Botosaneanu and Malicky 1978: 340 [checklist]. —Moretti and Cianficconi 1981: 200 [checklist]. —González and Otero 1983: 117 [distribution]. —Malicky 1983b: 54, 55 [atlas; ♂; ♀]. —Kumanski and Malicky 1984: 199 [distribution]. —Kumanski 1985: 147 [♂]. —Wiberg-Larsen 1985: 40 [checklist]. —González et al. 1986: 113 [distribution]. —Nógrádi 1986: 135 [distribution; ♂]. —Andersen and Wiberg-Larsen 1987: 169 [checklist]. —Malicky and Lounaci 1987: 15, 17 [checklist]. —Usseglio-Polatera and Bournaud 1989: 253 [distribution]. —Spuris 1989: 17 [checklist]. —González et al. 1990: 212 [checklist]. —Botosaneanu 1993b: 160 [distribution]. —Nógrádi and Uherkovich 1994: 31 [distribution]. —Uherkovich and Nógrádi 1997: 461 [distribution]. —Cianficconi et al. 1999: 277 [distribution]. —Nógrádi and Uherkovich 1998: 338 [distribution]. —Uherkovich and Nógrádi 1999: 420 [distribution]. —Urbanič et al. 2000: 45 [distribution]. —Uherkovich and Nógrádi 2001: 94 [distribution]. —Nógrádi and Uherkovich 2001: 297 [checklist]. —Nógrádi 2001: 85 [distribution]. —Gullefors 2002: 132, 138 [redlisted in Sweden; checklist]. —Cianficconi et al. 2002: 146 [distribution]. —Cibaitė 2003a: 10 [checklist]. —Cibaitė 2003b: 8 [distribution]. —Gullefors 2003: 194 [distribution]. —Bonada et al. 2004: 53 [distribution]. —Malicky 2004a: 68, 69 [atlas]. —Malicky 2005b: 545 [checklist]. —Bonada et al. 2005: 787 [distribution]. —Lubini-Ferlin and Vicentini 2005: 68 [checklist]. —Robert 2007: 83 [checklist]. —Chvojka and Komzák 2008: 13 [distribution]. —Ujvárosi et al. 2008: 112 [checklist]. —Szczęsny and Godunko 2008: 15 [checklist]. —Gullefors 2008: 64 [checklist]. —Višinskienė 2009: 28 [checklist]. —Cianficconi and Corallini 2010: 87 [distribution]. —Corallini and Cianficconi 2011: 628 [checklist]. —González and Menéndez 2011: 119 [distribution]. —O’Connor 2013: 64 [distribution]. Zuyderduyn and Tempelman 2013: 29 [distribution]. —O’Connor 2015: 28, 96 [distribution]. —Martín et al. 2015: 74 [distribution]. —Ruiz-García et al. 2016: 4 [distribution]. —Gullefors 2016: 155 [checklist]. —Wallace 2016: 20, 21, 23, 65 [conservation status]. —Graf et al. 2017: 48 [distribution]. —O’Connor and O’Connor 2017b: 53 [distribution]. —O’Connor and O’Connor 2018: 83 [distribution]. —Komzák and Kroča 2018: 168 [distribution]. —Cerjanec et al. 2020: 13 [distribution]. —Mabrouki et al. 2020: 14 [distribution]. —Smirnova et al. 2020: 68 [distribution].

—*brunneicornis* (Pictet, 1834): 226 [type locality: [Switzerland]; no holotype designated; in *Hydroptila*]. —Fischer 1961: 135 [considered a junior synonym to either *O.angustella* or *Ithytrichialamellaris*].

**Distribution.** —Algeria, Austria, Belgium, Bulgaria, Croatia, Czech Republic, Denmark, England, France, Germany, Hungary, Ireland, Italy, Kazakhstan, Lithuania, Morocco, Netherlands, Norway, Portugal, Romania, Russia, Slovenia, Spain, Sweden, Switzerland, Ukraine.

***annulata*** Wells, 1984: 274 [type locality: [Papua] New Guinea, Mendi, 1497 m; ANIC; ♂]. —Neboiss 1986: 90 [atlas; ♂]. —Wells 1991: 526 [checklist].

**Distribution.** —Papua New Guinea.

***apophysis*** Zhou & Yang in Zhou et al. 2010: 30 [type locality: [China], Jiangxi Province, Jiu Lian Shan National Nature Reserve, Da-Qiu-Tian, 8.2 km northwest of Da-Qiu-Tian, 114°25'50"E, 24°34'15"N, 425 m; NAUJ; ♂]. —Yang et al. 2016: 476 [checklist].

**Distribution.** —China.

***arala*** Oláh & Johanson, 2010a: 40 [type locality: Madagascar, Perinet; MNHN; ♂].

**Distribution.** —Madagascar.

***armata*** Wells, 1979a: 594 [type locality: [Australia] Victoria, Snobs Creek; NMV; ♂]. —Wells 1985b: 29 [case, biology]. —Neboiss 1986: 86 [atlas; ♂; ♀]. —Bovill et al. 2016 [larval predation on Trichoptera eggs].

**Distribution.** —Australia.

***asimetris*** Wells & Malicky, 1997: 189 [type locality: [Indonesia] N Sumatra, 8 km N Sindar Raya; Collection Malicky; ♂]. —Malicky and Chantaramongkol 2007: 1037 [♂; *distribution*]. —Malicky 2007a: 177 [checklist]. —Malicky 2010a: 46 [atlas; ♂]. —Malicky et al. 2014c: 33 [distribution].

**Distribution.** —Cambodia, Indonesia, Malaysia, Thailand.

***atraseta*** Wells, 1979a: 594 [type locality: [Australia] Victoria, Millgrove, Yarra River; NMV; ♂; ♀]. —Wells 1985b: 28 [larva; case]. —Neboiss 1986: 85 [atlas; ♂; ♀].

**Distribution.** —Australia.

***attenuata*** Wells, 1983: 643 [type locality: Australia, New South Wales, Darling R., Burtundy Station, 120 km N. Mildura; NMV; ♂; ♀]. —Neboiss 1986: 93 [atlas; ♂; ♀].

**Distribution.** —Australia.

***avicularis*** Kimmins, 1951: 203 [type locality: India, Behar, Pusa; NHMUK; ♂]. —Oláh and Johanson 2010a: 42 [distribution].

**Distribution.** —India.

***baldufi*** Kingsolver & Ross, 1961: 32 [type locality: [United States], Minnesota, Eaglenest Lake, St, Louis Co.; INHS; ♂; ♀]. —Etnier 1965: 147 [checklist]. —Blickle 1979: 53, 59 [checklist; ♂]. —Roy and Harper 1979: 152 [checklist]. —Roy and Harper 1981: 105 [distribution]. —Harris et al. 1991: 238 [distribution]. —Abbott et al. 1997: 44 [distribution]. —Moulton and Stewart 1997: 350 [checklist]. —Huryn and Harris 2000: 193 [distribution]. —Houghton et al. 2001: 505 [distribution; as *balduffi*]. —Pescador et al. 2004: 133 [checklist]. —Houghton et al. 2011b: 6 [distribution]. —Myers et al. 2011: 108 [distribution]. —Harris et al. 2012: 9 [checklist]. —Wright et al. 2013: 466 [biology; distribution]. —Houghton 2016: 46 [biology]. —Houghton et al. 2017: 63 [checklist; as *balduffi*]. —Harris and Rasmussen 2019: 218 [♂; ♀; distribution].

**Distribution.** —Canada, U.S.A.

***balra*** Oláh, 2012: 49 [type locality: Indonesia, Papua, Raja Empat Archipelago, Batanta Island, Warmon Creek, 1. waterfall; Collection Oláh; ♂]. —Oláh and Kovács 2018: 181 [distribution].

**Distribution.** —Indonesia.

***banisbus*** Wells, 1991: 512 [type locality: Papua New Guinea, Morobe Province, Bulolo, creek behind forestry compound, 7°13'S 146°35'E; ANIC; ♂; larva, case].

**Distribution.** —Papua New Guinea.

***barnardi*** Scott, 1963: 470 [type locality: [South Africa], Great Berg River, Stn. 1; SAMC; ♂; ♀; larva; pupal case]. —Wells and Andersen 1995: 163 [distribution]. —Palmer 1996: 43 [distribution]. —de Moor 2007: 216 [distribution]. —de Moor 2011: 354 [distribution]. —Mey 2011: 345 [checklist].

**Distribution.** —South Africa, Tanzania.

***becca*** Wells & Dostine, 2016: 597 [type locality: [Australia] Northern Territory, Berry Springs; NTM; ♂].

**Distribution.** —Australia.

***bellicosa*** Wells, 1979a: 618 [type locality: [Australia] Western Australia, Mitchell Plateau, Camp Creek; WAM; ♂]. —Neboiss 1986: 94 [atlas; ♂]. —Wells et al. 2019: 33 [detection frequency].

**Distribution.** —Australia.

***bencana*** Oláh, 1989: 291 [type locality: Vietnam, Bac Thai Province, PQuang Chu; HNHM; ♂]. —Armitage et al. 2005: 27 [checklist]. —Zhou et al. 2010: 40 [checklist]. —Malicky 2010a: 52 [atlas; ♂]. —Yang et al. 2016: 476 [checklist].

—*adunca* Yang & Xue, 1992: 29 [type locality: [China] Guilin, Guangxi; NAUJ; ♂]. —Yang et al. 1997b: 93 [checklist]. —Yang et al. 2005: 458 [checklist]. —Malicky and Chantaramongkol 2007: 1041 [♂; distribution, to synonymy].

**Distribution.** —China, Thailand, Vietnam.

***benguelensis*** Marlier, 1965: 69 [type locality: [Angola] District de Benguela, Catumbela, Marco de Canavezes (Cubal da Ganda), Loc. 10656-10; MDLA; ♂]. —Wells and de Moor 2020: 512 [checklist].

**Distribution.** —Angola.

***bensoni*** Wells, 1990c: 125 [type locality: [Australia] NE Queensland, Yuccabine Creek; NMV; ♂].

**Distribution.** —Australia.

***berbaring*** Wells & Malicky, 1997: 188 [type locality: [Indonesia] N Sumatra, Dolok Merangir (Spring), 03°07'N 99°11'E; Collection Malicky; ♂]. —Wells and Huisman 2001: 2012 [distribution]. —Malicky 2007a: 177 [checklist]. —Malicky 2010a: 52 [atlas; ♂].

**Distribution.** —Indonesia.

***bertie*** Wells, 2005: 390 [type locality: Australia, N Queensland, 11°45'S 142°35'E, Bertie Creek, 1 km SE Heathlands HS; NMV; ♂].

**Distribution.** —Australia.

***bilasnating*** Wells, 1991: 416 [type locality: Papua New Guinea, Central Province, Iomari Creek on Bereina-Port Morseby road, 9°04'S 147°06'E; ANIC; ♂].

**Distribution.** —Papua New Guinea.

***biokrotta*** Melnitsky & Malicky, 2008: 25 [type locality: Thailand, Trat Province, Chang island, river Khlong Plu, over the Khlong Plu Waterfall, 12°03'56.74"N 102°18'51.30"E; ZIN; ♂]. —Malicky 2010a: 49 [atlas; ♂].

**Distribution.** —Thailand.

***bipela*** Wells, 1991: 514 [type locality: Papua New Guinea, West Highlands Province, Peregai, 6°09'S 144°11'E; ANIC; ♂].

**Distribution.** —Papua New Guinea.

***bisetula*** Wells & Andersen, 1995: 163 [type locality: Tanzania, Tanga region, West Usambara Mts, Mazumbai, Kaputu Stream, loc. 10, 1420 m a.s.l.; ZMUB; ♂].

**Distribution.** —Tanzania.

***bishopi*** Wells, 1979a: 596 [type locality: [Australia] South Australia, Second Valley, Anacotilla Creek; ANIC; ♂; ♀]. —Wells 1985b: 28 [larva, case]. —Neboiss 1986: 86 [atlas; ♂; ♀].

**Distribution.** —Australia.

***bolyi*** Guenda, 1996: 247 [type locality: [Burkina Faso], à Fon dans la zone des sources du Mouhoun; UOBF; ♂].

**Distribution.** —Burkina Faso.

***bucera*** Yang & Xue, 1992: 29 [type locality: [China], Longsheng, Jinjiang, Guangxi; NAUJ; ♂]. —Yang et al. 1997b: 93 [checklist]. —Yang et al. 2005: 458 [checklist]. —Zhou et al. 2010: 40 [checklist]. —Yang et al. 2016: 476 [checklist].

**Distribution.** —China.

***bullata*** Wells, 1979a: 602 [type locality: [Australia], Queensland, Mossman Gorge; ANIC; ♂]. —Neboiss 1986: 88 [atlas; ♂].

**Distribution.** —Australia.

***bunkosa*** Oláh, 2012: 50 [type locality: Indonesia, Papua, Raja Empat Archipelago, Batanta Island, Site B, small stream, 250 m from mouth, 0°48'47.08"S 130°38'18.91"E; Collection Oláh; ♂].

**Distribution.** —Indonesia.

***butmasensis*** Johanson, Wells, Malm, & Espeland, 2011: 288 [type locality: [Vanuatu], Espiritu Santo, Central Santo, stream crossing track, 3 km N Butmas, 410 m, loc#11, 15°20.856'S, 166°58.347'E; NHRS; ♂].

**Distribution.** —Australia.

***capillata*** Wells, 1979a: 620 [type locality: [Australia] Queensland, Mossman Gorge; ANIC; ♂]. —Wells 1985b: 31 [larva]. —Neboiss 1986: 94 [atlas; ♂]. —Oláh and Johanson 2010a: 43 [distribution].

**Distribution.** —Australia.

***cazaubonae*** Guenda, 1996: 247 [type locality: [Burkina Faso], à Badala, village de la localité de Dédougou, sur le cours moyen du Mouhoun; UOBF; ♂].

**Distribution.** —Burkina Faso.

***cernyi*** Mey, 1990: 3 [type locality: [Philippines], Nord-Luzon, Ifugao, Banaue vic., N Lagawe; ZMHB; ♂]. —Wells and Mey 2002: 134 [checklist].

**Distribution.** —Philippines.

***chitwan*** Malicky & Chantaramongkol, 2007: 1041 [type locality: Nepal, Chitwan NP, Temple Tiger Lodge, 27°32'N 84°04'E, 150 m; Collection Malicky; ♂]. —Oláh and Johanson 2010a: 44 [distribution]. —Mattern 2015: 501 [distribution].

**Distribution.** —India, Nepal.

***cinctigera*** Wells, 1984: 277 [type locality: [Papua] New Guinea, Mendi, 1497 m; ANIC; ♂]. —Neboiss 1986: 90 [atlas; ♂]. —Wells 1991: 526 [checklist].

**Distribution.** —Papua New Guinea.

***conferta*** Wells, 1983: 634 [type locality: Australia, Victoria, Wellington R., 17 km N. of Licola; NMV; ♂; ♀]. —Wells 1985b: 32 [larva, case, pupa, biology]. —Neboiss 1986: 95 [atlas; ♂; ♀]. —Wells 2010a: 52 [♂]. —Oláh and Johanson 2010a: 44 [distribution].

**Distribution.** —Australia.

***constricta*** Wells, 1990c: 127 [type locality: [Australia] Northern Territory, Kakadu National Park, Radon Springs, 12°45'S 132°55'E; NMV; ♂]. —Oláh and Johanson 2010a: 44 [distribution].

**Distribution.** —Australia.

***coreana*** Ito & Park, 2016: 230 [type locality: Korea, Gyeongsangbuk-do, Cheongdo-gun, Unmun-myeon, Sinwon-ri, 35°40'42.6"N, 128°57'29.0"E; NIBR; ♂; ♀]. —Park and Kong 2020: 297 [checklist].

**Distribution.** —Korea.

***cornuta*** Zhou & Yang in Zhou et al. 2010: 31 [type locality: [China], Sichuan Province, Shi-mian County, Li-zi-ping Nature Preserve, Ca-luo-xiang Town, unnamed trib. of Hai-zi-gou stream, 200 m W of 3^rd^-level Hydropower Station, 4.3 km S of G108 from 2600.8 km stone marker, 102°22'08"E, 29°08'27"N, 1384 m; NAUJ; ♂]. —Yang et al. 2016: 476 [checklist].

**Distribution.** —China.

***costalis*** (Curtis, 1834): 218 [type locality: “Britain”; type not designated; in *Hydroptila*]. —Stephens 1836: 153 [distribution]. —McLachlan 1865: 96 [♂]. —Martynov 1924: 53 [♂; in *Oxyethira*]. —Kimmins 1958b: 14 [♀; distribution]. —Neboiss 1963: 594 [lectoholotype designated; to *Orthotrichia*]. —Botosaneanu 1967: 293 [distribution]. —Spuris 1972: 19 [checklist]. —Botosaneanu and Malicky 1978: 340 [checklist]. —Kumanski 1979: 5 [♂; distribution]. —Moretti et al. 1981: 350, 354 [biology; distribution]. —Moretti and Cianficconi 1981: 200 [checklist]. —Andrikovics and Ujhelyi 1983: 6 [distribution]. —Malicky 1983b: 54, 55 [atlas; ♂; ♀]. —Kumanski 1985: 151, 153 [♂]. —Wiberg-Larsen 1985: 40 [checklist]. —Andersen and Wiberg-Larsen 1987: 169 [checklist]. —Malicky and Lounaci 1987: 15 [checklist]. —Sipahiler and Malicky 1987: 129 [distribution]. —Spuris 1989: 17 [checklist]. —Waringer 1989: 390 [distribution; ecology]. —Usseglio-Polatera and Bournaud 1989: 253 [distribution]. —Andersen et al. 1990: 52 [distribution]. —Xue et al. 1992: 353–356 [distribution]. —Botosaneanu 1992: 105 [♂; ♀]. —Andersen et al. 1993b: 3 [distribution]. —Nógrádi and Uherkovich 1994: 31 [distribution]. —Haase 1994: 206 [distribution]. —Dallai and Afzelius 1995: 166 [sperm structure]. —Kahnert 1995: 124 [distribution]. —Chvojka 1996: 131 [distribution]. —Uherkovich and Nógrádi 1997: 461 [distribution]. —Brettfeld 1997: 137 [distribution]. —Uherkovich and Nógrádi 1998: 52 [distribution]. —Nógrádi and Uherkovich 1998: 338 [distribution]. —Uherkovich and Nógrádi 1999: 420 [distribution]. —Cianficconi et al. 1999: 57 [distribution]. —Malicky 1999c: 96 [distribution]. —Wiberg-Larsen and Karsholt 1999: 126 [distribution]. —Morse et al. 2001: 102 [distribution]. —Uherkovich and Nógrádi 2001: 94 [distribution]. —Nógrádi and Uherkovich 2001: 297 [checklist]. —Nógrádi and Uherkovich 2002: 129 [distribution]. —Mirmoayedi and Malicky 2002: 164 [checklist]. —Arefina et al. 2002: 100 [♂; ♀; distribution]. —Gullefors 2002: 138 [checklist]. —Gullefors 2003: 194, 195 [distribution]. —Sipahiler 2003b: 33 [distribution]. —Cibaitė 2003a: 10 [checklist]. —Arefina and Armitage 2003: 17 [distribution]. —Urbanič 2004: 51 [distribution]. —Malicky 2004a: 68, 69 [atlas]. —Graf and Hutter 2004: 147 [distribution]. —Cianficconi et al. 2004: 256, 258 [distribution; biology]. —Berlin 2005: 128, 130 [distribution]. —Gullefors 2005a: 119 [distribution]. —Sipahiler 2005: 397 [distribution]. —Mey 2005b: 119 [distribution]. —Yang et al. 2005: 458 [checklist]. —Malicky 2005a: 66 [distribution]. —Hohmann 2005: 106 [checklist]. —Graf et al. 2005: 55 [distribution]. —Lubini-Ferlin and Vicentini 2005: 68 [checklist]. —Chvojka and Komzák 2006: 358 [distribution]. —Waringer and Graf 2006: 356 [distribution]. —Mey 2006a: 159 [distribution]. —Schiess-Bühler and Rezbanyai-Reser 2006: 73 [distribution]. —Robert 2007: 83 [checklist]. —Gullefors and Johanson 2007: 64 [distribution]. —Berlin and Thiele 2007: 50 [checklist]. —Cianficconi et al. 2007: 569, 575 [distribution]. —Eskov et al. 2008: 78 [checklist; fossil species in amber]. —Waringer and Graf 2008: 142 [distribution]. —Szczęsny and Godunko 2008: 15 [checklist]. —Gullefors 2008: 64 [checklist]. —Chvojka and Komzák 2008: 13 [distribution]. —Ujvárosi et al. 2008: 112 [checklist]. —Schrankel et al. 2008: 90 [checklist]. —Višinskienė 2009: 28 [checklist]. —Zhou et al. 2010: 40 [checklist]. —Ivanov 2011: 195 [checklist]. —Cianficconi et al. 2011: 47 [distribution]. —Viidalepp et al. 2011: 196 [distribution]. —Zuyderduyn and Tempelman 2013: 25 [distribution]. —Ito 2013: 40 [♂; ♀; distribution]. —Tempelman and Sanabria 2013a: 20 [distribution]. —Tempelman and Sanabria 2013b: 144 [distribution]. —Mey 2014: 187 [distribution]. —Malicky 2014b: 17 [teratological structures]. —Hohmann et al. 2014: 85 [distribution]. —O’Connor 2015: 28, 97 [distribution]. —Pan’kov and Krasheninnikov 2016: 333 [distribution]. —Yang et al. 2016: 476 [checklist]. —Tanida and Kuranishi 2016: 71 [checklist]. —Potikha and Vshivkova 2016: 364 [distribution]. —Sipahiler 2016: 15 [checklist]. —Gullefors 2016: 155 [checklist]. —Graf and Leitner 2016: 37 [distribution]. —Wallace 2016: 16, 19, 24 [conservation status]. —Kobayashi et al. 2017: 17 [distribution]. —Park et al. 2018: 103 [♂; ♀; distribution]. —O’Connor and O’Connor 2018: 83 [distribution]. —Kučinić et. al. 2019: 450 [distribution]. —O’Connor 2020: 140 [distribution]. —Park and Kong 2020: 297 [checklist]. —Navara et al. 2020: 46 [distribution].

—*tetensii* Kolbe, 1887: 357 [type locality: [Germany], in der unmittelbaren Nähe von Berlin, in der Nähe des Wellenbades an der oberen Spree; no holotype designated]. —Klapálek 1894: 2 [♂; distribution]. —Klapálek 1897: 9 [larva]. —Morton 1899b: 281 [distribution]. —Ris 1903: 17 [distribution; as *tetensi*]. —Morton 1904: 326 [distribution]. —Martynov 1924: 49 [♂]. —Ulmer 1929: 255 [morphological notes; comparison to *O.angustella*]. —Martynov 1934: 122 [♂]. —Mosely 1939b: 279 [♂]. —Nielsen 1948: 95 [larva]. —Berg 1948: table 14 [distribution]. —Schmid 1959b: 693 [distribution]. —Nybom 1960: 18 [checklist]. —Spuris 1962: 62 [distribution]. —Wang 1963: 58 [larva; distribution]. —Neboiss 1963: 594 [to synonymy]. —Spuris 1964: 13 [distribution]. —Spuris 1972: 19 22 23 [checklist].

**Distribution.** —Austria, Belarus, Bulgaria, China, Croatia, Czech Republic, Denmark, England, Estonia, Finland, France, Germany, Greece, Hungary, Iran, Ireland, Italy, Japan, Korea, Latvia, Luxembourg, Netherlands, Norway, Romania, Russia, Scotland, Slovakia, Slovenia, South Korea, Sudan, Sweden, Switzerland, Turkey, Ukraine.

***cristata*** Morton, 1905: 75 [type locality: United States, Lake Forest, Illinois; depository not designated; ♂]. —Milne 1936: 77 [as junior synonym of *O.americana*]. —Ross 1944: 141 [♂; ♀; distribution]. —Denning 1947a: 19 [distribution]. —Denning 1947b: 173 [distribution]. —Ross and Spencer 1952: 47 [distribution]. —Morse and Blickle 1953: 72 [checklist]. —Kingsolver and Ross 1961: 32 [♂; distribution]. —Etnier 1965: 147 [checklist]. —Flint 1968b: 45 [♂; ♀; distribution]. —Flint 1968a: 82 [distribution]. —Edwards 1973: 506 [distribution]. —Roy and Harper 1975: 1082 [distribution]. —Botosaneanu 1979: 49, 53 [distribution; ♂]. —Roy and Harper 1979: 152 [checklist]. —Blickle 1979: 53 [checklist]. —Parker and Voshell 1981: 4 [checklist]. —Swegman et al. 1981: 139 [distribution]. —Harris et al. 1982a: 511 [distribution]. —Huryn and Foote 1983: 791 [distribution]. —Waltz and McCafferty 1983a: 11 [distribution]. —Hamilton et al. 1983: 19 [distribution]. —Harris et al. 1984: 109 [distribution]. —Lake 1984: 220 [checklist]. —Bowles and Mathis 1989: 239 [distribution]. —Harris et al. 1991: 239 [distribution]. —Floyd 1992: 50 [distribution]. —Masteller and Flint 1992: 70 [checklist]. —Mathis and Bowles 1992: 24 [distribution]. —Bowles and Mathis 1992: 32 [distribution]. —Moulton et al. 1993: 21 [distribution]. —Angrisano 1995a: 509 [distribution]. —Flint 1996a: 16 [checklist]. —Moulton and Stewart 1996: 123 [♂; distribution]. —Abbott et al. 1997: 44 [distribution]. —Moulton and Stewart 1997: 350 [checklist]. —Botosaneanu and Hyslop 1998: 12 [distribution]. —Houghton and Stewart 1998: 105 [biology; distribution]. —Angrisano 1999: 34 [checklist]. —Huryn and Harris 2000: 193 [distribution]. —Houghton et al. 2001: 505 [distribution]. —Botosaneanu 2002b: 87 [checklist]. —Pescador et al. 2004: 133 [checklist]. —Flint and Sykora 2004: 40 [distribution]. —Naranjo López and González Lazo 2005: 149 [checklist]. —Zeullig et al. 2006: 43 [distribution]. —Bowles et al. 2007: 22 [distribution; biology]. —Pérez-Gelabert 2008: 301 [checklist]. —Etnier 2010: 486 [distribution]. —Armitage et al. 2011: 14 [checklist]. —Houghton et al. 2011b: 6 [distribution]. —Myers et al. 2011: 108 [distribution]. —Harris et al. 2012: 9 [checklist]. —Wright et al. 2013: 466 [biology; distribution]. —Blinn and Ruiter 2013: 291 [biology; distribution]. —DeWalt et al. 2016: 52 [distribution]. —Denson et al. 2016: 6 [distribution]. —Houghton 2016: 46 [biology]. —Houghton et al. 2017: 63 [checklist]. —Harris and Rasmussen 2019: 221 [♂; ♀; distribution]. —Mendez et al. 2019: 128 [checklist]. —Bowles et al. 2020: 8 [distribution].

**Distribution.** —Canada, Cuba, Dominican Republic, Jamaica, Uruguay, U.S.A.

***crutwelli*** Wells, 1991: 519 [type locality: Papua New Guinea, Eastern Highlands Province, Mt Gahavasuka Provincial Park, 6°06'S 145°23'E; ANIC; ♂].

**Distribution.** —Papua New Guinea.

***cucullata*** Wells, 1984: 272 [type locality: [Papua] New Guinea, NE., Mt Kaindi, 2100–2350 m; BPBM; ♂]. —Neboiss 1986: 89 [atlas; ♂]. —Wells 1991: 526 [checklist].

**Distribution.** —Papua New Guinea.

***curta*** Kingsolver & Ross, 1961: 33 [type locality: [United States], Florida, Temple Terrace; INHS; ♂]. —Blickle 1979: 53, 59 [checklist; ♂]. —Roy and Harper 1979: 152 [checklist]. —Roy and Harper 1981: 105 [distribution]. —Harris et al. 1982a: 511 [distribution]. —Harris et al. 1991: 240 [distribution]. —Abbott et al. 1997: 44 [distribution]. —Moulton and Stewart 1997: 350 [checklist]. —Houghton et al. 2001: 505 [distribution]. —Pescador et al. 2004: 133 [checklist]. —Etnier 2010: 486 [distribution]. —Harris et al. 2012: 9 [checklist]. —Houghton 2016: 46 [biology]. —Denson et al. 2016: 6 [distribution]. —Houghton et al. 2017: 63 [checklist]. —Harris and Rasmussen 2019: 222 [♂; ♀; distribution].

**Distribution.** —Canada, U.S.A.

***curvata*** (Ulmer, 1951): 77 [type locality: [Indonesia], Java, Buitzenzorg; ZMUH; ♂; in *Javanotrichia*]. —Malicky and Chantaramongkol 2007: 1040 [♂; distribution]. —Malicky 2010a: 50 [atlas; ♂]. —Malicky et al. 2014a: 6 [distribution]. —Malicky et al. 2016: 92 [distribution].

**Distribution.** —Indonesia.

***cuspidigera*** Zhou & Yang in Zhou et al. 2010: 38 [type locality: [China], Jiangxi Province, Jiu Lian Shan National Nature Reserve, Da-Qiu-Tian, 8.2 KM northwest of Da-Qiu-Tian, 114°25'50"E, 24°34'15"N, 425 m; NAUJ; ♂]. —Yang et al. 2016: 476 [checklist].

**Distribution.** —China.

***damasi*** Marlier, 1943: 31 [type locality: [Congo], Ishango; depository not designated; ♂].

**Distribution.** —Congo.

***dampfi*** (Ulmer, 1963): 268 [type locality: [Egypt], Maadi, am Licht; ZMUH; ♂; in *Javanotrichia*]. —Malicky 1983b: 54 [atlas; ♂]. —Malicky 2004a: 68 [atlas]. —Malicky 2005b: 545 [checklist].

**Distribution.** —Egypt.

***dapola*** Guenda, 1996: 245 [type locality: [Burkina Faso], Dapola dans le cours inférieur du Mouhoun; UOBF; ♂].

**Distribution.** —Burkina Faso.

***dentata*** Kingsolver & Ross, 1961: 33 [type locality: [United States], Florida, Temple Terrace; INHS; ♂]. —Blickle 1979: 53, 59 [checklist; ♂]. —Harris et al. 1982a: 511 [distribution]. —Pescador et al. 2004: 133 [checklist]. —Flint et al. 1994: 4 [distribution]. —Harris et al. 2012: 9 [checklist]. —Denson et al. 2016: 6 [distribution]. —Harris and Rasmussen 2019: 225 [♂; ♀; distribution].

**Distribution.** —U.S.A.

***deukalion*** Malicky & Prommi in Malicky et al. 2000: 862 [type locality: [Thailand], Doi Suthep NP, Huai Koo Kao, 550 m; Collection Malicky; ♂]. —Malicky 2010a: 47 [atlas; ♂].

**Distribution.** —Thailand.

***digitata*** Wells, 1984: 281 [type locality: [Papua] New Guinea, Mendi, 1497 m; ANIC; ♂]. —Neboiss, 1986: 91 [atlas; ♂]. —Wells 1991: 526 [checklist].

**Distribution.** —Papua New Guinea.

***dikirilagoda*** Schmid, 1958b: 63 [type locality: [Sri Lanka] Ceylan, Le Vallon, Diyaluma Falls (Uva, 800 ft) 21-II), gros rochers arrosés par les embruns d’une chute, haute et abondante; depository not designated; ♂].

**Distribution.** —Sri Lanka.

***dilgri*** Wells, 1983: 640 [type locality: Australia, New South Wales, Dilgry R., 19 km NW. Rawdon Vale, 151°32'E 31°53'S; NMV; ♂; ♀]. —Neboiss 1986: 86 [atlas; ♂; ♀].

**Distribution.** —Australia.

***discedata*** Zhou & Morse in Zhou et al. 2010: 34 [type locality: [China], Fujian Province, Jiu-qu-xi, 118°01'12"E, 27°27'00"N, 220 m; NAUJ; ♂]. —Yang et al. 2016: 476 [checklist].

**Distribution.** —China.

***disparalis*** Wells, 1984: 276 [type locality: [Papua] New Guinea, NE., Wau, Big Wau Creek, 1300 m; BPBM; ♂]. —Neboiss 1986: 90 [atlas; ♂]. —Wells 1991: 526 [checklist].

**Distribution.** —Papua New Guinea.

***ditenga*** Wells, 1990b: 397 [type locality: [Indonesia] Sulawesi Utara, Motolanga R., Doloduo-Malibagu road; NMV; ♂]. —Wells and Huisman 2001: 212 [distribution].

**Distribution.** —Indonesia.

***divaricata*** Wells, 1983: 639 [type locality: Australia, Queensland, Upper Freshwater Creek.; NMV; ♂]. —Neboiss 1986: 95 [atlas; ♂].

**Distribution.** —Australia.

***echidna*** Malicky, 1999a: 345 [type locality: [Yemen], Provinz Al-Mahwit, 30 km NE Bajil, 5 km NNE Khamis Bani Sa’d, 750 m, 15°1'N, 43°32'E; Collection Malicky; ♂]. —Malicky 2004a: 69 [atlas]. —Malicky 2005b: 545 [checklist].

**Distribution.** —Yemen.

***egena*** Mey, 1998a: 552 [type locality: [Philippines, Mindanao], northern slope of Mt. Atuuganon range, 1050 m; ZMHB; ♂]. —Wells and Mey 2002: 134 [checklist].

**Distribution.** —Philippines.

***eltera*** Oláh, 2012: 50 [type locality: Indonesia, Raja Empat Archipelago, Batanta Island, Warmon Creek, 1. waterfall; ANIC; ♂]. —Oláh 2013: 69 [distribution]. —Oláh and Kovács 2018: 181 [distribution].

**Distribution.** —Indonesia.

***ensiformis*** Wells, 1984: 281 [type locality: [Papua] New Guinea, D.P.I. Urimo Station; ANIC; ♂]. —Neboiss 1986: 94 [atlas; ♂]. —Wells 1991: 526 [checklist]. —Wells and Dostine 2016: 599 [distribution].

**Distribution.** —Australia, Papua New Guinea.

***epupae*** Mey & de Moor, 2019: 142 [type locality: Namibia, Kunene River, Epupa Falls, 17°00.127'S, 13°14.742'E; ZMHB; ♂].

**Distribution.** —Namibia.

***eurhinata*** Wells, 1985a: 102 [type locality: Australia, Northern Territory, Georgetown Billabong, nr Jabiru; NTM; ♂]. —Neboiss 1986: 88 [atlas; ♂]. —Wells et al. 2019: 33 [detection frequency].

**Distribution.** —Australia.

***exigua*** Wells, 1979a: 616 [type locality: [Australia] Western Australia, Fine Spring Creek, on road between Lake Argyle Tourist Village and Duncan Highway; WAM; ♂]. —Neboiss 1986: 92 [atlas; ♂]. —Wells et al. 2019: 33 [detection frequency].

**Distribution.** —Australia.

***extensa*** Martynov, 1935: 117 [type locality: [India], above Kapildhara Fall, Rewah State, C. I..; NZSI; ♂]. —Malicky 2006: 253 [checklist]. —Malicky and Chantaramongkol 2007: 1040 [♂; distribution]. —Mattern 2015: 501 [distribution]. —Malicky 2018: 49 [checklist].

**Distribution.** —India, Nepal.

***feltuna*** Oláh, 2016: 114 [type locality: Indonesia, West Papua, Batanta Island, valley of Kalisamsem River, 00°53'27.54", 130°33'31.62"; Collection Oláh; ♂].

**Distribution.** —Indonesia.

***ferreirae*** Wells & de Moor, 2020: 508 [type locality: Angola, Moxico Province, Cuembo River, Site 6 — Cuembo campsite bridge, -13.5265, 19.27971AGMS; ♂].

**Distribution.** —Angola.

***fimbriata*** Wells, 1991: 519 [type locality: Papua New Guinea, Central Province, Veikabu; ANIC; ♂]. —Wells and Huisman 2001: 212 [distribution].

**Distribution.** —Indonesia, Papua New Guinea.

***flabella*** Wells, 1983: 637 [type locality: Australia, Victoria, McKenzie R., Princes Highway bridge, 25 km W. Cann River; NMV; ♂]. —Neboiss 1986: 96 [atlas; ♂].

—*lapka*Oláh and Johanson 2010a: 47 [type locality: Australia, Tasmania, Ewart Creek,150 m downstream bridge on A10, 41°58.576'S, 145°27.708'E, 221 m; ANIC; ♂]. —Wells 2012: 67 [to synonymy].

**Distribution.** —Australia.

***fonalka*** Oláh & Johanson, 2010a: 44 [type locality: Hong Kong, Sai Kung East Country Park, stream, 1.2 km E Tin Mei Shan Mt., at Luk Wu; NHRS; ♂]. —Malicky 2013: 43 [possibly a junior synonym to *Orthotrichiamomanga*]. —Yang et al. 2016: 476 [checklist].

**Distribution.** —Hong Kong.

***fontinala*** Wells, 1990c: 121 [type locality: [Australia] NE Queensland, Yuccabine Creek; NTM; ♂].

**Distribution.** —Australia.

***fortificata*** Mey, 1998a: 553 [type locality: [Philippines, Mindanao], northern slope of Mt. Atuuganon range, 1050 m; ZMHB; ♂]. —Wells and Mey 2002: 134 [checklist].

**Distribution.** —Philippines.

***foruma*** Oláh, 2016: 115 [type locality: [Indonesia], West Papua, Batanta Island, right side stream of Forum River, 0°52'22.7", 130°27'45.1"; Collection Oláh; ♂]. —Oláh and Kovács 2018: 181 [distribution].

**Distribution.** —Indonesia.

***fosla*** Oláh in Oláh and Kovács 2018: 182 [type locality: Indonesia, West Papua, Batanta Island, valley of Kalijakut River, 00°52'49.1"S, 130°38'04.9"E; Collection Oláh; ♂].

**Distribution.** —Indonesia.

***fragilis*** Wells, 1984: 274 [type locality: [Western New Guinea], Irian Jaya (New Guinea); BPBM; ♂]. —Neboiss 1986: 89 [atlas; ♂]. —Wells 1991: 526 [checklist].

**Distribution.** —Indonesia.

***furcata*** Wells, 1990c: 121 [type locality: [Australia] Northern Territory, South Alligator River above Fisher Creek junction; NTM; ♂].

**Distribution.** —Australia.

***garbunga*** Wells, 1990b: 398 [type locality: [Indonesia] Sulawesi Utara, Dumoga-Bone N.P., Fog 11, NHMUK Plot A; NHMUK; ♂]. —Wells and Huisman 2001: 213 [distribution].

**Distribution.** —Indonesia.

***glebula*** Wells, 1984: 276 [type locality: [Papua] New Guinea, NE., Morobe District, 10 km W. Bulolo, 780 m; BPBM; ♂]. —Neboiss 1986: 90 [atlas; ♂]. —Wells 1991: 526 [checklist].

**Distribution.** —Papua New Guinea.

***gorbek*** Oláh, 2016: 116 [type locality: Indonesia, West Papua, Batanta Island, valley of Kalisamsem River, 00°53'27.54", 130°33'31.62"; Collection Oláh; ♂]. —Oláh and Kovács 2018: 182 [distribution].

**Distribution.** —Indonesia.

***gracilis*** Wells, 1979a: 610 [type locality: [Australia] New South Wales, Coraki; ANIC; ♂; ♀]. —Neboiss 1986: 92 [atlas; ♂; ♀].

**Distribution.** —Australia.

***gressitti*** Wells, 1991: 523 [type locality: Papua New Guinea, New Ireland, SW., ‘Camp Bishop’, 15 km up Kait River; BPBM; ♂]. —Wells 2005: 389 [♂; distribution, parasitoid behavior observed].

**Distribution.** —Australia, Papua New Guinea.

***gudiel*** Malicky & Graf, 2015: 31 [type locality: Ethiopia, Kleiner Waldbach N von Addis Abeba, 9°05'N, 38°43'E, 2800 m; Collection Malicky; ♂].

**Distribution.** —Ethiopia.

***guinkoi*** Guenda, 1996: 243 [type locality: [Burkina Faso], Zindi; UOBF; ♂].

**Distribution.** —Burkina Faso.

***guruluhela*** (Schmid, 1958b): 59 [type locality: [Sri Lanka], Ceylan, Beliul Oya (Sab., 2000 ft) 19-II, ruisseaux torrentueux, dans les buissons; depository not designated; ♂; in *Baliotrichia*].

**Distribution.** —Sri Lanka.

***hajla*** Oláh & Johanson, 2010a: 46 [type locality: Indonesia, Sumba, Lewa, Lainguru National Park, dried river bed, 400 m; Collection Oláh; ♂].

**Distribution.** —Indonesia.

***hanulva*** Oláh, 2016: 117 [type locality: Indonesia, West Papua, Batanta Island, valley of Kalijakut River, 0°52'49.1", 130°38'4.9"; Collection Oláh; ♂].

**Distribution.** —Indonesia.

***hinipitigola*** (Schmid, 1958b): 60 [type locality: [Sri Lanka], Ceylan, Della (Sab., 15oo ft) 18-II, petite rivière affluente de la Kalu Ganga, encaissée et agitée, dans la jungle; depository not designated; ♂; in *Baliotrichia*].

**Distribution.** —Sri Lanka.

***hippomenes*** Malicky, 2004b: 295 [type locality: [Nepal, Bardia National Park], am Rande der nordindischen Ebene im Südwesten von Nepal im Bereich des ersten Hügelkammes des Himalaya (Siwalik Range), unweit des Wehrs des Babai Flusses, über das die Brücke der Ost-West-Haupstraße Nepals (Mahindra Highway), 28°25'N, 81°23'E, 190 m, Budhi Khola; Collection Malicky; ♂]. —Malicky 2006: 253 [checklist]. —Mattern 2015: 501 [distribution].

**Distribution.** —Nepal.

***holaga*** Oláh in Oláh and Kovács 2018: 183 [type locality: Indonesia, West Papua, Batanta Island, Northern cost, Warmon stream, above second waterfall, S00°50'29.47", E130°42'29.16"; Collection Oláh; ♂].

**Distribution.** —Indonesia.

***huaihuat*** Malicky & Chantaramongkol, 2007: 1033 [type locality: Thailand, Huai Huat NP, 16°55'N 104°11'E, 400 m; Collection Malicky; ♂]. —Malicky 2010a: 45 [atlas; ♂]. —Malicky et al. 2014c: 33 [distribution, as *O.huayhuat*].

**Distribution.** —Cambodia, Thailand.

***hydroptiloides*** Wells & Andersen, 1995: 165 [type locality: Tanzania, Morogoro region, Morogoro, Sokoine University of Agriculture, 550 m a.s.l.; ZMUB; ♂].

**Distribution.** —Tanzania.

***ifugao*** Wells & Mey, 2002: 134 [type locality: [Philippines] Luzon, Ifugao Province, Jacmal Bunhian, 24 km E Mayoyao, 800–1000 m; BPBM; ♂].

**Distribution.** —Philippines.

***indah*** Malicky, Melnitsky, & Ivanov, 2014a: 833 [type locality: [Indonesia] Papua, Insel Biak, Wardo, Wapsdori Wasserfall, 1°01'22"S, 135°51'25"E, 10–40 m Seehöhe; ZIN; ♂].

**Distribution.** —Indonesia.

***indica*** Martynov, 1935: 116 [type locality: [India], Inlé Lake, S. Shan States.; NZSI; ♂]. —Schmid 1958b: 58 [distribution]. —Oláh 1989: 288 [distribution]. —Wells and Malicky 1997: 188 [distribution]. —Malicky and Chantaramongkol 2007: 1042 [distribution]. —Malicky 2007a: 177 [checklist]. —Oláh and Johanson 2010a: 46 [distribution]. —Malicky 2010a: 52 [atlas; ♂]. —Malicky et al. 20146 [distribution]. —Wityi et al. 2015: 47 [distribution].

**Distribution.** —India, Indonesia, Laos, Malaysia, Myanmar, Sri Lanka, Thailand, Vietnam.

***inornata*** Wells, 1979a: 605 [type locality: [Australia] Western Australia, Mitchell Plateau; WAM; ♂]. —Neboiss 1986: 88 [atlas; ♂]. —Wells et al. 2019: 33 [detection frequency].

**Distribution.** —Australia.

***instabilis*** Denning, 1948: 397 [type locality: [United States], Florida, Winter Park; ESUW; ♂]. —Blickle and Morse 1957: 48 [distribution; ♀]. —Kingsolver and Ross 1961: 29 [♂; distribution]. —Blickle 1979: 53, 59 [checklist; ♂]. —Bowles and Mathis 1989: 239 [distribution]. —Harris et al. 1991: 241 [distribution]. —Moulton and Stewart 1996: 123 [♂; distribution]. —Moulton and Stewart 1997: 350 [checklist]. —Abbott et al. 1997: 44 [distribution]. —Pescador et al. 2004: 133 [checklist]. —Etnier 2010: 486 [distribution]. —Harris et al. 2012: 9 [checklist] . —Denson et al. 2016: 6 [distribution]. —Harris and Rasmussen 2019: 227 [♂; ♀; distribution].

**Distribution.** —U.S.A.

***iriga*** Wells & Mey, 2002: 132 [type locality: [Philippines] Luzon Is., Camarines Sur Prov., Mt Iriga, 500–600 m; BPBM; ♂].

**Distribution.** —Philippines.

***iriomotensis*** Ito, 2013: 43 [type locality: Japan, Ryûkyû Islands, Iriomote-jima, Aira-gawa, beside Route 217, 24°20'03"N, 123°54'47"E, 3 m above sea level; CBM-ZI; ♂; ♀]. —Tanida and Kuranishi 2016: 71 [checklist].

**Distribution.** —Japan.

***itintikah*** Malicky, 2014c: 43 [type locality: Thailand, Prov. Nan, NP Mae Charim, 260 m, 18°36'N, 100°58'E; Collection Malicky; ♂].

**Distribution.** —Thailand.

***jani*** Wells & Huisman, 1993: 112 [type locality: East Malaysia, Sabah, Long Pa Sia area, Sg. Ritan, 04°24'N 115°42'E, 1160 m; RMNH; ♂]. —Malicky 2010a: 51 [atlas; ♂].

**Distribution.** —Malaysia.

***jembatana*** Wells, 1990b: 398 [type locality: [Indonesia] Sulawesi Utara, Dumoga-Bone N.P., Site 6; NHMUK; ♂].

**Distribution.** —Indonesia.

***jethran*** Malicky, Ivanov, & Melnitsky, 2011: 1492 [type locality: [Indonesia], Lombok, Kembangkuning, 4 km N Kotaraja, 490 m, 8°33'33"S, 116°25'23"E; ZIN; ♂]. —Malicky et al. 2014a: 6 [distribution]. —Malicky et al. 2016: 92 [distribution].

**Distribution.** —Indonesia.

***kabaenica*** Wells & Huisman, 2001: 211 [type locality: Sulawesi Tenggara, P. Kabaena, 4 km S Tankeno, Sungai Lakambula, 300 m; RMNH; ♂].

**Distribution.** —Indonesia.

***kaitica*** Wells, 1991: 521 [type locality: Papua New Guinea, New Ireland, SW., ‘Camp Bishop’, 15 km up Kait River, 4°23'S 152°41'E; BPBM; ♂].

**Distribution.** —Papua New Guinea.

***kalengiensis*** Statzner, 1977: 403 [type locality: Zaire, Kivu Region, Kalengo stream 10 km west of Lake Kivu; ZMHB; ♂; ♀].

**Distribution.** —Congo.

***kalisa*** Oláh, 2016: 118 [type locality: Indonesia, West Papua, Batanta Island, valley of Kalisamsem River, 00°53'27.54", 130°33'31.62"; Collection Oláh; ♂].

**Distribution.** —Indonesia.

***kaonan*** Malicky, Suwannarat, & Laudee, 2018: 1320 [type locality: Thailand, Huai Vat (Nebenbach des Klong Kay) bei Ban Pak Lang, nahe dem Kao Nan Nationalpark, 8°47'N, 99°35'E, 140 m; Collection Malicky; ♂].

**Distribution.** —Thailand.

***kerekded*** Oláh, 2016: 119 [type locality: Indonesia, West Papua, Batanta Island, Kalijakut River, 0°5'52.0", 130°38'8.0"; Collection Oláh; ♂].

**Distribution.** —Indonesia.

***kholoensis*** Wells, 1979a: 612 [type locality: [Australia] Queensland, Brisbane River near Kholo; NMV; ♂]. —Neboiss 1986: 92 [atlas; ♂].

**Distribution.** —Australia.

***kinabalu*** Malicky & Chantaramongkol, 2007: 1043 [type locality: Malaysia, Sabah, Kinabalu NP, Livagu river, 1410 m; Collection Malicky; ♂]. —Malicky 2010a: 49 [atlas; ♂].

**Distribution.** —Malaysia.

***kisbunka*** Oláh, 2012: 50 [type locality: Indonesia, Papua, Raja Empat Archipelago, Batanta Island, Warmon Creek, 2. waterfall, 0°50'23.25"S 130°42'35.18"E; Collection Oláh; ♂].

**Distribution.** —Indonesia.

***kivuensis*** Jacquemart, 1956: 5 [type locality: [Congo], Bukavu, au large; IRSNB; ♂; ♀]. —Jacquemart 1957: 124 [♂; distribution].

**Distribution.** —Congo.

***kokodana*** Kimmins, 1962: 104 [type locality: [Indonesia], Papua, Kokoda, 1200 ft; NHMUK; ♂]. —Neboiss 1986: 89 [atlas; ♂]. —Wells 1991: 526 [checklist].

**Distribution.** —Indonesia.

***krungut*** Wells, 1991: 517 [type locality: Papua New Guinea, Central Province, Laloki River at Rouna Falls, 9°25'S 147°27'E; ANIC; ♂].

**Distribution.** —Papua New Guinea.

***kunenensis*** Mey & de Moor, 2019: 141 [type locality: Namibia, Kunene River, Swartbooisdrif, Kunene River Lodge, 17°20'50"S, 13°52'56"E; ZMHB; ♂].

**Distribution.** —Namibia.

***lalonduwasi*** Wells & Huisman, 2001: 211 [type locality: Sulawesi Tenggara, N slope of Gunung Watuwila, 1100 m, Sungai Lalonduwasi; RMNH; ♂].

**Distribution.** —Indonesia.

***lanna*** Malicky & Chantaramongkol, 2007: 1040 [type locality: Thailand, Chiang Dao WRS, 19°22'N 98°55'E, 500 m; Collection Malicky; ♂]. —Oláh and Johanson 2010a: 49 [distribution]. —Malicky 2010a: 44 [atlas; ♂].

**Distribution.** —Laos, Thailand.

***laposka*** Oláh, 2013: 69 [type locality: Indonesia, Papua, Raja Ampat, Batanta Island, northern coast, Waridor River, S 0.84373°, E 130.52457°, shippable endpoint; Collection Oláh; ♂].

**Distribution.** —Indonesia.

***latiramifera*** Zhou & Yang in Zhou et al. 2010: 32 [type locality: [China], Jiangxi Province, Jiu Lian Shan National Nature Reserve, Unnamed trib. of Xia-Gong-Tang Stream, 114°28'08"E, 24°32'05"N, 630 m; NAUJ; ♂]. —Yang et al. 2016: 476 [checklist].

**Distribution.** —China.

***lebar*** Wells & Huisman, 1993: 112 [type locality: East Malaysia, Sarawak, Bako National Park, Sungai Delima; RNTM; ♂]. —Malicky 2010a: 51 [atlas; ♂].

**Distribution.** —Malaysia.

***lentigo*** Wells, 1984: 274 [type locality: [Papua] New Guinea, NE., Wau, McAdam Park, 1250 m; BPBM; ♂]. —Neboiss 1986: 90 [atlas; ♂]. —Wells 1991: 526 [checklist].

**Distribution.** —Papua New Guinea.

***ligula*** Mey & Freitag, 2019: 209 [type locality: [Philippines], Palawan, Puerto Princesa, Brgy. Cabayugan, spring creek of Cabayugan River, SSW of Martarpi, 10°09'46"N, 118°49'29"E, 80 m a.s.l.; MPMP; ♂; ♀]. —Mey and Freitag 2020: 57 [distribution].

**Distribution.** —Philippines.

***litoralis*** (Ulmer, 1951): 89 [type locality: [Indonesia], Bali, Litoral des Batur-Sees, 1031 m; ZMUH; ♂; in *Baliotrichia*]. —Wells and Malicky 1997: 190 [distribution]. —Malicky and Chantaramongkol 2007: 1033 [♂; distribution]. —Malicky 2007a: 177 [checklist]. —Oláh and Johanson 2010a: 49 [distribution]. —Malicky 2010a: 45 [atlas; ♂]. —Malicky et al. 2014a: 6 [distribution]. —Mattern 2015: 501 [checklist; as *littoralis*]. —Melnitsky et al. 2019: 539 [distribution].

—*kasyi* Chantaramongkol & Malicky, 1986: 516 [type locality: [Sri Lanka], North Central Province, Kantalai, 60 m; MZLU; ♂]. —Malicky and Chantaramongkol 2007: 1033 [to synonymy].

—*veikaba* Wells, 1991: 516 [type locality: Papua New Guinea, Central Province, Veikabu; ANIC; ♂]. —Malicky and Chantaramongkol 2007: 1033 [to synonymy]. —Wells and Dostine 2016: 599 [distribution].

**Distribution.** —Australia, Indonesia, Laos, Nepal, Papua New Guinea, Sri Lanka, Thailand, Vietnam.

***litoris*** Mey, 2006b: 204 [type locality: Indonesia, Sulawesi Selatan, Soroako, Lake Matano; LIPI; ♂].

**Distribution.** —Indonesia.

***litotes*** Wells, 1984: 272 [type locality: [Papua] New Guinea, Mendi, 1497 m; ANIC; ♂]. —Neboiss 1986: 90 [atlas; ♂]. —Wells 1991: 526 [checklist].

**Distribution.** —Papua New Guinea.

***lobophorana*** Mey, 2003b: 434 [type locality: Philippines, Luzon, Quezon province, east of Infanta, Magsaysay; ZMHB, to be transferred to either MPMP or UPLB; ♂].

**Distribution.** —Philippines.

***luonga*** Oláh, 1989: 289 [type locality: Vietnam, Bac Thai Province, Phuluong, River Dongdat; HNHM; ♂]. —Wells and Huisman 2001: 214 [distribution]. —Armitage et al. 2005: 28 [checklist]. —Malicky 2010a: 50 [atlas; ♂].

**Distribution.** —Indonesia, Vietnam.

***luzofortificata*** Mey, 2003b: 434 [type locality: Philippines, Luzon, Quezon province, east of Infanta, Magsaysay; ZMHB, to be transferred to either MPMP or UPLB; ♂].

**Distribution.** —Philippines.

***mackayi*** Wells, 1991: 521 [type locality: Papua New Guinea, West Highlands Province, Baiyer River Sanctuary, Trauna River, 5°30'S 144°10'E; ANIC; ♂; larva; case].

**Distribution.** —Papua New Guinea.

***madagassa*** Oláh & Johanson, 2010a: 49 [type locality: Madagascar, Perinet; MNHN; ♂].

**Distribution.** —Madagascar.

***maeandrica*** (Ulmer, 1951): 76 [type locality: [Indonesia], Java, Buitzenzorg, Bellevue; ZMUH; ♂; in *Javanotrichia*]. —Wells and Malicky 1997: 187 [distribution]. —Malicky and Chantaramongkol 2007: 1038 [♂; distribution]. —Malicky 2007a: 177 [checklist]. —Oláh and Johanson 2010a: 50 [distribution]. —Malicky 2010a: 48 [atlas; ♂]. —Malicky et al. 2014a: 6 [distribution]. —Promwong and Thapanya 2019: 75 [distribution].

**Distribution.** —Indonesia, Laos, Malaysia, Thailand, Vietnam.

***mahisindha*** Oláh & Johanson, 2010a: 51 [type locality: India, Rajasthan, Mahi River, Banswara; Collection Oláh; ♂].

**Distribution.** —India.

***marsyas*** Malicky, 2004b: 295 [type locality: [Nepal, Bardia National Park], am Rande der nordindischen Ebene im Südwesten von Nepal im Bereich des ersten Hügelkammes des Himalaya (Siwalik Range), bei dem Dorf Babai Basar in der Nähe der Straße von Nepalganj nach Birendranagar, ungefähr 30 km flussaufwärts vom Lager 1 (28°21'N, 81°42'E), lag das Ufer des Babai Nadi in wenigen Metern Entfemung, vom “westlicher” Bach, 320 m; Collection Malicky; ♂]. —Malicky 2006: 253 [checklist]. —Mattern 2015: 501 [distribution].

**Distribution.** —Nepal.

***mas*** Malicky & Graf, 2012: 32 [type locality: [Ethiopia], Meribo River; Collection Malicky; ♂].

**Distribution.** —Ethiopia.

***masola*** Oláh & Johanson, 2010a: 52 [type locality: Australia, Queensland, Kondallilla Falls Section, Picnic Creek, dstr waterfall next to track, 26°40.236'S, 152°52.116'E, 169 m; ANIC; ♂].

**Distribution.** —Australia.

***medipitigola*** (Schmid, 1958b): 59 [type locality: [Sri Lanka], Ceylan, Nuwara Eliya (C. P., 6000 ft) 25-II, marais à Carex, aus bords septentrionaux du Gregory Lake; depository not designated; ♂; in *Baliotrichia*].

**Distribution.** —Sri Lanka.

***melitta*** Malicky, 1976: 93 [type locality: Greece, Insel Lesbos, Ayia Paraskevi; Collection Malicky; ♂]. —Botosaneanu and Malicky 1978: 340 [checklist]. —Malicky 1983b: 55 [atlas; ♂]. —Kumanski and Malicky 1984: 199 [distribution]. —Kumanski 1985: 149 [♂]. —Botosaneanu 1992: 103 [♂; ♀]. —Malicky 2004a: 68, 69 [atlas]. —Malicky 2005b: 544 [checklist]. —Sipahiler 2005: 397 [distribution]. —Malicky 2005a: 66 [distribution]. —Malicky 2014b: 17 [teratological structures]. —Dia 2015: 51 [distribution].

**Distribution.** —Bulgaria, Greece, Lebanon, Turkey.

***menarika*** Wells, 1990b: 395 [type locality: [Indonesia] Sulawesi Utara, Dumoga-Bone N.P., Tumpah R. and tributary junction; NMV; ♂; ♀]. —Mey 2006b: 204 [distribution].

**Distribution.** —Indonesia.

***mencenga*** Wells, 1990b: 404 [type locality: [Indonesia] Sulawesi Utara, Dumoga-Bone N.P., Site 6; NHMUK; ♂].

**Distribution.** —Indonesia.

***menjonkok*** Wells & Malicky, 1997: 189 [type locality: [Indonesia] N Sumatra, Huta Padang, 02°45'N 99°14'E; Collection Malicky; ♂]. —Malicky and Chantaramongkol 2007: 1041 [distribution]. —Malicky 2007a: 177 [checklist]. —Malicky 2010a: 52 [atlas; ♂].

**Distribution.** —Indonesia, Thailand.

***meyi*** Wells & de Moor, 2020: 508 [type locality: Angola, Moxico Province, Cuembo River, Site 9 — Cuembo source lake (Salia Kembo), -13.1363, 19.04529; AGMS; ♂].

**Distribution.** —Angola.

***minalwang*** Wells & Mey, 2002: 132 [type locality: [Philippines] Mindanao, Misamis Or., Minalwang, 1050 m; BPBM; ♂].

**Distribution.** —Philippines.

***mlamboi*** Wells & de Moor, 2020: 507 [type locality: Angola, Huila Province, Cubango River, Site 21 — downstream of rapids at ruins of hydropower plant, -14.3384, 16.29331; AGMS; ♂].

**Distribution.** —Angola.

***momanga*** Oláh, 1989: 290 [type locality: Vietnam, Bac Thai Province, Quang Chu; HNHM; ♂]. —Armitage et al. 2005: 28 [checklist]. —Malicky 2010a: 47 [atlas; ♂]. —Malicky 2013: 43 [possibly a senior synonym to *O.fonalka*].

**Distribution.** —Vietnam.

***monga*** Oláh & Johanson, 2010a: 53 [type locality: Vietnam, Lamdong Province, Dalat, Prenn Waterfall; Collection Oláh; ♂].

**Distribution.** —Vietnam.

***morula*** Wells, 1979a: 608 [type locality: [Australia] Queensland, Mossman Gorge; ANIC; ♂; ♀]. —Wells 1985b: 30 [case]. —Neboiss 1986: 91 [atlas; ♂; ♀].

**Distribution.** —Australia.

***moselyi*** Tjeder, 1946: 133 [type locality: [Israel], Palestine, Dagania A., Jordan Valley, 670 ft. below the sea; NHMUK; ♂]. —Botosaneanu and Gasith 1971: 98 [distribution]. —Gasith and Kugler 1973: 57 [distribution; biology]. —Malicky 1983b: 55 [atlas; ♂]. —Moubayed and Botosaneanu 1985: 63 [distribution]. —Botosaneanu 1992: 99 [♂; ♀]. —Malicky 1999a: 344 [distribution]. —Malicky 2004a: 69 [atlas]. —Malicky 2005b: 545 [checklist]. —Dia 2015: 51 [distribution].

**Distribution.** —Israel, Lebanon, Yemen.

***mulehe*** Malicky, 2020: 510 [type locality: Uganda, Lkw Mulehe; collection Malicky; ♂].

**Distribution.** —Uganda.

***muscari*** Wells, 1983: 638 [type locality: Australia, Queensland, Iron Range, Middle Claudie R.; NMV; ♂]. —Neboiss 1986: 95 [atlas; ♂]. —Wells 1990c: 125 [case; distribution]. —Wells 1992: 299 [reported to be a parasitoid of Hydropsychidae pupae]. —Wells 2005: 385 [additional observations of parasitoid behavior]. —Wells et al. 2019: 33 [detection frequency].

**Distribution.** —Australia.

***mussoi*** Guenda, 1996: 247 [type locality: [Burkina Faso], à Zindi près de Gaoua dans le cours inférieur du Mouhoun; UOBF; ♂].

**Distribution.** —Burkina Faso.

***namnao*** Malicky & Chantaramongkol, 2007: 1038 [type locality: Thailand, Nam Nao NP, 16°38'N 101°35'E, 800 m; Collection Malicky; ♂]. —Malicky 2010a: 44 [atlas; ♂].

**Distribution.** —Thailand.

***nehega*** Oláh, 2016: 120 [type locality: Indonesia, West Papua, Batanta Island, Kalijakut River, 0°52'52.0", 130°38'8.0"; Collection Oláh; ♂]. —Oláh and Kovács 2018: 184 [distribution].

**Distribution.** —Indonesia.

***nessos*** Malicky & Chantaramongkol, 2007: 1038 [type locality: [Indonesia] Sumatra, Tinggi Raja, 3°09'N 98°48'E, 300 m; Collection Malicky; ♂]. —Malicky 2007a: 177 [checklist]. —Oláh and Johanson 2010a: 54 [distribution]. —Malicky 2010a: 45 [atlas; ♂].

**Distribution.** —Indonesia.

***newi*** Wells & Huisman, 1993: 111 [type locality: East Malaysia, Sabah, Tenom; NTM; ♂]. —Malicky 2010a: 50 [atlas; ♂].

**Distribution.** —Malaysia.

***nigrovillosa*** Wells & Andersen, 1995: 165 [type locality: Tanzania, Tanga region, West Usambara Mts, Mazumbai, Kaputu Stream, loc. 10, 1420 m a.s.l.; ZMUB; ♂].

**Distribution.** —Tanzania.

***nontaburi*** Malicky & Chantaramongkol, 2007: 1042 [type locality: Thailand Fluss Chaopraya bei Nontaburi; Collection Malicky; ♂]. —Malicky 2010a: 50 [atlas; ♂].

**Distribution.** —Thailand.

***nova*** Marlier, 1978: 295 [type locality: Mali, Pont de Kouoro; MRAC; ♂]. —Guenda 1996: 247 [distribution]. —Wells and de Moor 2020: 512 [checklist].

**Distribution.** —Angola, Burkina Faso, Mali.

***obscura*** Kimmins, 1962: 103 [type locality: [Indonesia], Papua, Kokoda, 1200 ft; NHMUK; ♂]. —Neboiss 1986: 89 [atlas; ♂]. —Wells 1991: 526 [checklist].

**Distribution.** —Indonesia.

***obtecta*** Wells & Dudgeon, 1990: 173 [type locality: Hong Kong, Tai Po Kao Forest stream; NHMUK; ♂; ♀]. —Zhou et al. 2010: 40 [checklist]. —Yang et al. 2016: 476 [checklist].

**Distribution.** —Hong Kong.

***olelo*** Oláh, 2013: 71 [type locality: Indonesia, Batanta Island, northern coast, small stream with Dry mouth, 1000–1500 m above Dry mouth; Collection Oláh; ♂]. —Oláh 2016: 121 [distribution]. —Oláh and Kovács 2018: 184 [distribution].

**Distribution.** —Indonesia.

***ops*** Malicky & Chantaramongkol, 2007: 1039 [type locality: [Indonesia] Sumatra, Aceh, Kruet Selatan NP, 2°59'N 97°23'E, 0 m; Collection Malicky; ♂]. —Malicky 2007a: 177 [checklist]. —Malicky 2010a: 48 [atlas; ♂].

**Distribution.** —Indonesia.

***orbostensis*** Wells, 1979a: 596 [type locality: [Australia] Victoria, Brodribb River, Sardine Creek Track, 39 km N. of Orbost; NMV; ♂]. —Neboiss 1986: 87 [atlas; ♂].

**Distribution.** —Australia.

***orias*** Oláh, 2013: 73 [type locality: Indonesia, Papua, Raja Ampat, Batanta Island, northern coast, Warmon stream, above second waterfall, 0.84152°S, 130.70810°E; Collection Oláh; ♂].

**Distribution.** —Indonesia.

***ostoros*** Oláh & Johanson, 2010a: 56 [type locality: Malaysia, Sabah, Tawau, Maliau Basin, Nepenthes Camp, Camel Trophy Hut, 4°43'59.3"N 116°52'39.7"E, 999 m; NHRS; ♂].

**Distribution.** —Malaysia.

***palikos*** Malicky & Chantaramongkol, 2007: 1039 [type locality: Thailand, Doi Inthanon NP, Mae Klang bei Ban Sob Aeb, 18°32'N 98°36'E, 540 m; Collection Malicky; ♂]. —Oláh and Johanson 2010a: 57 [distribution]. —Malicky 2010a: 44 [atlas; ♂].

**Distribution.** —Laos, Thailand.

***para*** Oláh, 2012: 50 [type locality: Indonesia, Papua, Raja Empat Archipelago, Batanta Island, Site B, small stream, 250 m from mouth, 0°48'47.08"S 130°38'18.91"E; Collection Oláh; ♂]. —Oláh 2016: 121 [distribution]. —Oláh and Kovács 2018: 184 [distribution].

**Distribution.** —Indonesia.

***paranga*** Wells, 1979a: 614 [type locality: [Australia] Western Australia, Ord Dam, at light below dam; WAM; ♂]. —Neboiss 1986: 92 [atlas; ♂]. —Wells 1990c: 123 [case; distribution]. —Wells et al. 2019: 33 [detection frequency].

**Distribution.** —Australia.

***parthenopaios*** Malicky & Chantaramongkol, 2007: 1033 [type locality: Thailand, Pitsanulok Prov., Phu Hin Rongkla NP, Huai Kamunnoi WF, 16°59'N 101°00'E, ; Collection Malicky; ♂]. —Malicky 2010a: 45 [atlas; ♂].

**Distribution.** —Thailand.

***parthenos*** Malicky & Chantaramongkol, 2007: 1038 [type locality: Thailand, Sai Yok NP, 14°26'N 98°51'E, 100 m; Collection Malicky; ♂]. —Malicky 2010a: 48 [atlas; ♂]. —Malicky et al. 2014c: 33 [distribution].

**Distribution.** —Cambodia, Thailand.

***pectinella*** Wells, 1983: 635 [type locality: Australia, Victoria, Warburton, Yarra R.; NMV; ♂]. —Wells 1985b: 32 [larva; case; pupa]. —Neboiss 1986: 96 [atlas; ♂]. —Wells 2010a: 52 [♂].

**Distribution.** —Australia.

***penthesileia*** Malicky & Chantaramongkol, 2007: 1031 [type locality: Thailand, Kao Soi Dao NP, 13°06'N 102°12'E, 300–400 m; Collection Malicky; ♂]. —Malicky 2010a: 43 [atlas; ♂].

**Distribution.** —Thailand.

***persephone*** Malicky, 2008a: 838 [type locality: [Indonesia, Borneo, Kalimantan], im Einzugsbereich der Flüsse Seturan und Rian in einem engen Bereich von ungefähr 8 × 8 km ca. 70 km südlich der Stadt Malinau, 116°29'48"–116°33'29"E, 2°59'29"–3°04'04"N, 100–200 m; MZLS; ♂]. —Malicky 2010a: 47 [atlas; ♂].

**Distribution.** —Indonesia.

***pethericki*** Wells & Dostine, 2016: 597 [type locality: [Australia] Northern Territory, Petherick’s Rainforest Reserve, 13°7.424'S, 130°39.920'E; NTM; ♂].

**Distribution.** —Australia.

***petiti*** Jacquemart, 1962b: 4 [type locality: Congo, Katanga, Sandoa; IRSNBM; ♂]. —Guenda 1996: 247 [♂; distribution]. —de Moor et al. 2000: 112 [distribution]. —Mey and de Moor 2019: 137 [checklist].

**Distribution.** —Burkina Faso, Congo, Namibia.

***polyhymnia*** Malicky, 2008a: 838 [type locality: [Indonesia, Borneo, Kalimantan], im Einzugsbereich der Flüsse Seturan und Rian in einem engen Bereich von ungefähr 8 × 8 km ca. 70 km südlich der Stadt Malinau, 116°29'48"–116°33'29"E, 2°59'29"–3°04'04"N, 100–200 m; MZLS; ♂]. —Malicky 2010a: 44 [atlas; ♂].

**Distribution.** —Indonesia.

***polyxena*** Malicky & Chantaramongkol, 2007: 1031 [type locality: Thailand, ob Ton Nga Chang WF, 6°58'N 100°12'E, 600 m; Collection Malicky; ♂]. —Malicky 2010a: 43 [atlas; ♂].

**Distribution.** —Thailand.

***prenna*** Oláh & Johanson, 2010a: 57 [type locality: Vietnam, Lamdong Province, Dalat, Prenn waterfall; Collection Oláh; ♂].

**Distribution.** —Vietnam.

***prevoti*** Guenda, 1996: 245 [type locality: [Burkina Faso], Zindi; UOBF; ♂].

**Distribution.** —Burkina Faso.

***priapos*** Malicky & Chantaramongkol, 2007: 1032 [type locality: Thailand, Tung Salaeng NP, 16°49'N, 100°57'E, 600 m; ♂]. —Malicky 2010a: 43 [atlas; ♂].

**Distribution.** —Thailand.

***putoei*** Malicky & Chantaramongkol, 2007: 1035 [type locality: Thailand, Putoei NP, headquarters, 14°57'N, 99°28'E, 250 m; Collection Malicky; ♂].

**Distribution.** —Thailand.

***ranauana*** (Ulmer, 1951): 80 [type locality: [Indonesia], Sumatra, Ranau-See, Brandungsufer; ZMUH; ♂; in *Orthotrichiella*]. —Malicky 1998a: 797 [♂; distribution]. —Malicky 2007a: 177 [checklist]. —Malicky 2010a: 51 [atlas; ♂]. —Malicky et al. 2014a: 6 [distribution].

**Distribution.** —Indonesia.

***rentzi*** Wells, 2010a: 51 [type locality: [Australia] North Queensland, 17°05'S 145°35'E, Mt Haig, 22 km NE by N of Atherton; ANIC; ♂].

**Distribution.** —Australia.

***rostrata*** Wells, 1979a: 598 [type locality: [Australia] Western Australia, Spillway Creek, Ord River Dam; WAM; ♂; ♀]. —Neboiss 1986: 86 [atlas; ♂; ♀].

**Distribution.** —Australia.

***runching*** Wells & Huisman, 1993: 111 [type locality: East Malaysia, Sabah, 12 km NNE Ranau, Poring Hot Springs, Sg. Kipogoh,06°03'N 116°42'E. 550 m; RMNH; ♂]. —Malicky 2010a: 51 [atlas; ♂].

**Distribution.** —Malaysia.

***sabazios*** Malicky & Chantaramongkol, 2007: 1035 [type locality: Bhutan, Punakha, Dungkar Rongchhu, 27°39'N 89°46'E, 1370 m; Collection Malicky; ♂].

**Distribution.** —Bhutan.

***sanya*** Mosely, 1948a: 45 [type locality: [Malawi, Lake Malawi], Lake Nyasa, Fort Johnston; NHMUK; ♂]. —Jacquemart 1957: 123 [♂; distribution]. —Mey 2011: 343, 345 [distribution; checklist]. —Mey 2016: 305 [distribution].

**Distribution.** —Congo, Malawai, Namibia.

***savoska*** Oláh, 2012: 51 [type locality: Indonesia, Papua, Raja Empat Archipelago, Batanta Island, Sarinam River, 0°50'04.24"S 130°47'59.22"E; Collection Oláh; ♂]. —Oláh 2016: 121 [distribution].

**Distribution.** —Indonesia.

***scutata*** Wells, 1979a: 600 [type locality: [Australia] Western Australia, Spillway Creek, Ord River Dam; WAM; ♂; ♀]. —Neboiss 1986: 87 [atlas; ♂; ♀]. —Wells 1990c: 125 [case; distribution].

**Distribution.** —Australia.

***scutellata*** Wells & Andersen, 1995: 165 [type locality: Tanzania, Tanga region, West Usambara Mts, Mazumbai, Kaputu Stream, loc. 5, 1650 m a.s.l.; ZMUB; ♂].

**Distribution.** —Tanzania.

***serrata*** Wells, 1990c: 121 [type locality: [Australia] Northern Territory, Kakadu National Park, Radon Springs, 12°45'S 132°55'E; NTM; ♂].

**Distribution.** —Australia.

***shawkah*** Malicky & Chantaramongkol, 2007: 1034 [type locality: United Arab Emirates, Wadi Wurayah; Collection Malicky; ♂].

**Distribution.** —United Arab Emirates.

***shimigaya*** Harris & Davenport, 1999: 29 [type locality: Peru, Loreto, small stream just outside grounds of Explorama Inn; NMNH; ♂].

**Distribution.** —Peru.

***sibuyan*** Malicky & Chantaramongkol, 2007: 1034 [type locality: Philippinen, Sibuyan, Prov. Romblon, Magdiwant; Collection Malicky; ♂]. —Malicky 2009b: 10 [distribution].

**Distribution.** —Philippines.

***sinit*** Wells & Huisman, 1993: 112 [type locality: East Malaysia, Sabah, 12 km NNE Ranau, Poring Hot Springs, Sg. Kipogoh, 06°03'N 116°42'E, 550 m; RMNH; ♂]. —Oláh and Johanson 2010a: 59 [distribution]. —Malicky 2010a: 51 [atlas; ♂].

**Distribution.** —Malaysia.

***sivka*** Oláh, 2016: 121 [type locality: Indonesia, West Papua, Batanta Island, valley of Warai stream, 00°50'51.0", 130°35'14.0"; Collection Oláh; ♂].

**Distribution.** —Indonesia.

***specana*** Wells, 2005: 390 [type locality: Australia, N Queensland, 18°57'S 146°10'E, Mt Spec State Forest, unnamed creek ‘Confusion Creek’, tributary to Paluma Reservoir, 905 m; NMV; ♂].

**Distribution.** —Australia.

***spinicauda*** Kimmins, 1958a: 366 [type locality: [Zimbabwe], S. Rhodesia, Victoria Falls; NHMUK; ♂].

**Distribution.** —Zimbabwe.

***spiralina*** Statzner, 1977: 401 [type locality: Zaire, Kivu Region, Kalengo stream 10 km west of Lake Kivu; ZMHB; ♂; ♀].

**Distribution.** —Congo.

***stipa*** Wells, 1979a: 614 [type locality: [Australia] Western Australia, Mitchell Plateau, Camp Creek; WAM; ♂]. —Wells 1985b: 31 [larva; case]. —Neboiss 1986: 92 [atlas; ♂].

**Distribution.** —Australia.

***straeleni*** Jacquemart, 1956: 1 [type locality: [Congo], Baie de Kabuno, Kirotsche-Shasha, Lac Kivu; IRSNB; ♂; ♀]. —Kimmins 1957c: 15 [♂; distribution]. —Jacquemart 1957: 117 [distribution; ♂; larva]. —Johanson 1992: 118 [checklist]. —Wells and Andersen 1995: 145 [checklist]. —Guenda 1996: 243 [distribution].

**Distribution.** —Burkina Faso, Congo, Uganda.

***styx*** Malicky, 2008a: 839 [type locality: [Indonesia, Borneo, Kalimantan], im Einzugsbereich der Flüsse Seturan und Rian in einem engen Bereich von ungefähr 8 × 8 km ca. 70 km südlich der Stadt Malinau, 116°29'48"–116°33'29"E, 2°59'29"–3°04'04"N, 100–200 m; MZLS; ♂]. —Malicky 2010a: 43 [atlas; ♂].

**Distribution.** —Indonesia.

***submontana*** Mey, 1995: 195 [type locality: [Philippines], Mindoro, Paluan, Calawagan-Fluß; Collection Mey; ♂]. —Wells and Mey 2002: 134 [checklist].

**Distribution.** —Philippines.

***subrhomba*** Zhou & Morse in Zhou et al. 2010: 37 [type locality: [China], Fujian Province, Jiu-qu-xi, 118°01'12"E, 27°27'00"N, 220 m; NAUJ; ♂]. —Yang et al. 2016: 476 [checklist].

**Distribution.** —China.

***suchiara*** Oláh, 1989: 292 [type locality: Vietnam, Bac Thai Province, Phuluong, River Dongdat; HNHM; ♂]. —Armitage et al. 2005: 28 [checklist]. —Oláh and Johanson 2010a: 59 [distribution]. —Malicky 2010a: 52 [atlas; ♂].

**Distribution.** —Laos, Vietnam.

***suteri*** Wells, 1979a: 605 [type locality: [Australia] Western Australia, Mitchell Plateau, Camp Creek; WAM; ♂; ♀]. —Neboiss 1986: 88 [atlas; ♂]. —Wells 1990c: 125 [case; distribution]. —Wells et al. 2019: 33 [detection frequency].

**Distribution.** —Australia.

***tabala*** Oláh, 2016: 122 [type locality: Indonesia, West Papua, Batanta Island, right side stream of Forum River, 0°52'22.7", 130°27'45.1"; Collection Oláh; ♂].

**Distribution.** —Indonesia.

***talea*** Wells, 1984: 278 [type locality: [Papua] New Guinea, Wau, Hospital Creek, 1250 m; BPBM; ♂]. —Neboiss 1986: 90 [atlas; ♂]. —Wells 1991: 526 [checklist].

**Distribution.** —Papua New Guinea.

***taleban*** Malicky & Chantaramongkol, 2007: 1036 [type locality: Thailand, Kao Soi Dao NP, 13°06'N 102°10'E, 400 m; Collection Malicky; ♂]. —Malicky 2010a: 47 [atlas; ♂].

**Distribution.** —Thailand.

***talumalaus*** Malicky & Chantaramongkol, 2007: 1041 [type locality: [Papua New Guinea], Bismarck-Archipel, Insel Mussau, Talumalaus; Collection Malicky; ♂]. —Malicky 2010b: 88 [checklist].

**Distribution.** —Papua New Guinea.

***terpsichore*** Malicky & Chantaramongkol, 2007: 1036 [type locality: Thailand, Nakon Si Thamarat, Mantok Yong NP, Pliew WF, 8°29'N 99°45'E, 110 m; Collection Malicky; ♂]. —Zhou et al. 2010: 34 [distribution]. —Malicky 2010a: 49 [atlas; ♂]. —Yang et al. 2016: 476 [checklist]. —Malicky et al. 2018: 1322 [distribution].

**Distribution.** —China, Thailand.

***thaleia*** Malicky & Chantaramongkol, 2007: 1035 [type locality: Thailand, Nam Cat Tien, 11°26'N 107°26'E, 200 m; Collection Malicky; ♂]. —Malicky 2010a: 46 [atlas; ♂].

**Distribution.** —Thailand.

***thanatos*** Malicky & Chantaramongkol, 2007: 1034 [type locality: Thailand, Doi Inthanon NP, Siribhum WF, 1300 m; Collection Malicky; ♂]. —Malicky 2010a: 49 [atlas; ♂].

**Distribution.** —Thailand.

***thariel*** Malicky & Graf, 2015: 31 [type locality: Sudan, Wadi Halfa; Collection Malicky; ♂]. —Englmaier et al. 2020: 10 [distribution].

**Distribution.** —Ethiopia, Sudan.

***thaumas*** Malicky & Chantaramongkol, 2007: 1037 [type locality: Thailand, Tramot, 7°15'N 100°02'E, 100 m; Collection Malicky; ♂]. —Malicky 2010a: 47 [atlas; ♂].

**Distribution.** —Thailand.

***theia*** Malicky, 2008a: 838 [type locality: [Indonesia, Borneo, Kalimantan], im Einzugsbereich der Flüsse Seturan und Rian in einem engen Bereich von ungefähr 8 × 8 km ca. 70 km südlich der Stadt Malinau, 116°29'48"–116°33'29"E, 2°59'29"–3°04'04"N, 100–200 m; MZLS; ♂]. —Malicky 2010a: 48 [atlas; ♂].

**Distribution.** —Indonesia.

***thersites*** Malicky & Chantaramongkol, 2007: 1039 [type locality: Thailand, Kao Soi Dao NP, 13°06'N 102°10'E, 400 m; Collection Malicky; ♂]. —Malicky 2010a: 48 [atlas; ♂].

**Distribution.** —Thailand.

***thistletoni*** Wells, 19891: 517 [type locality: Papua New Guinea, West Highlands Province, Peregai, 6°09'S 144°11'E; ANIC; ♂; ♀].

**Distribution.** —Papua New Guinea.

***thyone*** Malicky & Chantaramongkol, 2007: 1037 [type locality: Thailand, Prov, Loei, Ban Phangam, Piangtin WF, 17°04'N 101°45'E, 700 m; Collection Malicky; ♂]. —Malicky 2010a: 49 [atlas; ♂].

**Distribution.** —Thailand.

***tinggi*** Wells & Huisman, 2001: 212 [type locality: Sulawesi Tenggara, N slope of Gunung Watuwila, 1100 m, Sungai Lalonduwasi; RMNH; ♂].

**Distribution.** —Indonesia.

***tobfona*** Oláh, 2012: 51 [type locality: Indonesia, Papua, Raja Empat Archipelago, Batanta Island, Warmon Creek, 1. waterfall; Collection Oláh; ♂].

**Distribution.** —Indonesia.

***tombak*** Wells & Malicky, 1997: 187 [type locality: [Indonesia] N Sumatra, “Holzweg 2”, 10 km NE Prapat, 1050 m asl, 2°44'N 98°57'E; Collection Malicky; ♂]. —Malicky 2007a: 177 [checklist]. —Malicky 2010a: 46 [atlas; ♂].

**Distribution.** —Indonesia.

***tomentosa*** Wells, 1990c: 121 [type locality: [Australia] Northern Territory, Kakadu National Park, Radon Springs, 12°45'S 132°55'E; NTM; ♂]. —Wells et al. 2019: 33 [detection frequency].

**Distribution.** —Australia.

***tonjolana*** Wells, 1990b: 404 [type locality: [Indonesia] Sulawesi Utara, Dumoga-Bone N.P., Fog 19, NHMUK Plot A; NHMUK; ♂]. —Wells and Huisman 2001: 214 [distribution]. —Malicky et al. 2010: 163 [distribution].

**Distribution.** —Indonesia.

***tonsai*** Malicky, Melnitsky, & Ivanov, 2019: 427 [type locality: Thailand, Phuket, Ton Sai waterfall, 08°01.694'N, 98°21.944'E, height 137 m; ZIN; ♂].

**Distribution.** —Thailand.

***tortuosa*** Wells, 1979a: 612 [type locality: [Australia] Victoria, Genoa River near Wangarabell; NMV; ♂; ♀]. —Wells 1985b: 30 [larva, case]. —Neboiss 1986: 94 [atlas; ♂; ♀]. —Oláh and Johanson 2010a: 59 [distribution].

**Distribution.** —Australia.

***tragetti*** Mosely, 1930b: 247 [type locality: England, Hampshire, lake at Aubridge Danes, Romsey, but also lists France, Ain, La Dombes, St. Paul-de-Varax; NHMUK; ♂]. —Martynov 1934: 124 [♂]. —Mosely 1939b: 279 [♂]. —Schmid 1947: 531 [distribution]. —Nybom 1960: 18 [checklist]. —Spuris 1962: 62 [distribution]. —Botosaneanu 1967: 293 [distribution]. —Botosaneanu and Malicky 1978: 340 [checklist]. —Moretti and Cianficconi 1981: 200 [checklist]. —Malicky 1983b: 54, 55 [atlas; ♂; ♀]. —Kumanski and Malicky 1984: 199 [distribution]. —Kumanski 1985: 150 [♂]. —Nógrádi 1985: 129 [distribution; ♂; ♀]. —Reusch 1986: 139 [distribution]. —Nógrádi 1986: 139 [distribution]. —Andersen and Wiberg-Larsen 1987: 169 [checklist]. —Botosaneanu and Levanidova 1988: 174 [distribution]. —Oláh 1989: 288 [distribution]. —Spuris 1989: 17 [checklist]. —Xue et al. 1992: 353–356 [distribution]. —Nógrádi and Uherkovich 1994: 31 [distribution]. —Chvojka 1996: 131 [distribution]. —Uherkovich and Nógrádi 1997: 461 [distribution]. —Yang et al. 1997b: 93 [checklist]. —Graf et al. 1998: 206 [distribution]. —Uherkovich and Nógrádi 1998: 52 [distribution]. —Nógrádi and Uherkovich 1998: 338 [distribution]. —Malicky 1999c: 96 [distribution]. —Malicky 1999f: 31 [distribution]. —Uherkovich and Nógrádi 1999: 420 [distribution]. —Morse et al. 2001: 102 [distribution]. —Valle 2001: 64 [distribution]. —Uherkovich and Nógrádi 2001: 94 [distribution]. —Nógrádi and Uherkovich 2001: 297 [checklist]. —Arefina et al. 2002: 99 [distribution]. —Nógrádi and Uherkovich 2002: 130 [distribution]. —Gullefors 2002: 138 [checklist]. —Cianficconi et al. 2002: 146 [distribution]. —Gullefors 2003: 195 [distribution]. —Serafin 2003: 319 [distribution]. —Urbanič 2004: 51 [distribution]. —Malicky 2004a: 68, 69 [atlas]. —Lukas 2004: 685 [distribution]. —Weinzierl et al. 2005: 48 [distribution]. —Malicky 2005a: 66 [distribution]. —Hohmann 2005: 106 [checklist]. —Malicky 2005b: 545 [checklist]. —Sipahiler 2005: 397 [distribution]. —Yang et al. 2005: 458 [checklist]. —Komzák and Chvojka 2005: 65 [distribution]. —Wiggers et al. 2006: 54 [distribution]. —Gullefors 2006: 137 [distribution]. —Voigt et al. 2006: 73 [distribution]. —Waringer and Graf 2006: 356 [distribution]. —Robert 2007: 83 [checklist]. —Berlin and Thiele 2007: 48, 50 [distribution; checklist]. —Szczęsny and Godunko 2008: 15 [checklist]. —Gullefors 2008: 64 [checklist]. —Waringer and Graf 2008: 142 [distribution]. —Ujvárosi et al. 2008: 112 [checklist]. —Chvojka and Komzák 2008: 13 [distribution]. —Nozaki 2010: 22 [distribution]. —Zhou et al. 2010: 40 [checklist]. —Malicky 2010a: 52 [atlas; ♂]. —Cianficconi et al. 2011: 47 [distribution]. —Ivanov 2011: 195 [checklist]. —Ito 2013: 39 [♂; ♀; distribution]. —Lock and Zwaenepoel 2014: 232 [distribution]. —Mey 2014: 184 187 [distribution]. —Yang et al. 2016: 476 [checklist]. —Tanida and Kuranishi 2016: 71 [checklist]. —Vshivkova et al. 2016: 79 [distribution]. —Vrućina et al. 2016: 113 [distribution]. —Potikha and Vshivkova 2016: 364 [distribution]. —Sipahiler 2016: 15 [checklist]. —Gullefors 2016: 155 [checklist]. —Küttner et al. 2016: 179 [distribution]. —Graf and Leitner 2016: 37 [distribution]. —Wallace 2016: 21, 23, 66 [conservation status]. —Kobayashi et al. 2017: 17 [distribution]. —Park et al. 2018: 104 [♂; ♀; distribution]. —Park and Kong 2020: 298 [checklist].

**Distribution.** —Austria, Belgium, Bulgaria, China, Croatia, Czech Republic, England, Finland, France, Germany, Greece, Hungary, Italy, Japan, Korea, Latvia, Netherlands, Poland, Slovenia, Romania, Russia, Slovakia, Sweden, Switzerland, Turkey, Ukraine, Vietnam.

***triacantha*** Mey, 2003b: 434 [type locality: Philippines, Luzon, Laguna, Pangil; ZMHB, to be transferred to either MPMP or UPLB; ♂].

**Distribution.** —Philippines.

***trilineata*** Jacquemart, 1963a: 412 [type locality: [South Africa], Cape Prov., Upintgon, Orange River; IRSNB; ♂]. —Palmer 1996: 43 [distribution]. —Mey 2011: 345 [checklist].

**Distribution.** —South Africa.

***trispinata*** Wells, 2005: 390 [type locality: Australia, N Queensland, 18°57'S 146°10'E, Mt Spec State Forest, Camp Creek, 760 m; NMV; ♂].

**Distribution.** —Australia.

***triton*** Malicky, 2008a: 838 [type locality: [Indonesia, Borneo, Kalimantan], im Einzugsbereich der Flüsse Seturan und Rian in einem engen Bereich von ungefähr 8 × 8 km ca. 70 km südlich der Stadt Malinau, 116°29'48"–116°33'29"E, 2°59'29"–3°04'04"N, 100–200 m; MZLS; ♂]. —Malicky 2010a: 43 [atlas; ♂].

**Distribution.** —Indonesia.

***tronoca*** Oláh & Johanson, 2010a: 59 [type locality: Vietnam, Lamdong Province, Baoloc, Duchma stream; Collection Oláh; ♂].

**Distribution.** —Vietnam.

***tumoris*** Wells, 1984: 277 [type locality: [Papua] New Guinea, Mendi, 1497 m; ANIC; ♂]. —Neboiss 1986: 89 [atlas; ♂]. —Wells 1991: 526 [checklist].

**Distribution.** —Papua New Guinea.

***tunjakkana*** Wells, 1990b: 400 [type locality: [Indonesia] Sulawesi Utara, Dumoga-Bone N.P., Site 6; NHMUK; ♂; ♀; larva; pupa; case]. —Wells and Huisman 2001: 214 [distribution].

**Distribution.** —Indonesia.

***turrita*** Wells, 1979a: 600 [type locality: [Australia] Western Australia, Four Mile Creek, 20 km NE. Lake Argyle Tourist Village; WAM; ♂]. —Wells 1985b: 29 [larva; case]. —Neboiss 1986: 87 [atlas; ♂]. —Wells et al. 2019: 33 [detection frequency].

**Distribution.** —Australia.

***tyche*** Malicky & Chantaramongkol, 2007: 1036 [type locality: Thailand, Tinggi Raja, 3°09'N 98°48'E, 300 m; Collection Malicky; ♂]. —Malicky 2007a: 177 [checklist; distribution]. —Malicky 2010a: 47 [atlas; ♂].

**Distribution.** —Indonesia, Thailand.

***tyleri*** Wells, 1979a: 618 [type locality: [Australia] Western Australia, Mitchell Plateau, Camp Creek; WAM; ♂]. —Neboiss 1986: 94 [atlas; ♂]. —Wells 1990c: 123 [case; distribution]. —Wells et al. 2019: 33 [detection frequency].

**Distribution.** —Australia.

***typhoeus*** Malicky & Chantaramongkol, 2007: 1036 [type locality: Thailand, Nam Mae Sa beim Sirikit Botanischen Garten, 12 km W Mae Rim, 18°54'N 98°52'E, 700 m; Collection Malicky; ♂]. —Malicky 2010a: 45 [atlas; ♂].

**Distribution.** —Thailand.

***tyro*** Malicky & Chantaramongkol, 2007: 1032 [type locality: Thailand, Tung Salaeng NP, 16°49'N 100°57'E, 600 m; Collection Malicky; ♂]. —Malicky 2010a: 43 [atlas; ♂].

**Distribution.** —Thailand.

***udawarama*** (Schmid, 1958b): 61 [type locality: [Sri Lanka], Ceylan, Lindula (C. P., 4100 ft) 3-III, belle rivière assez calme, à fond dallé, dans les plantations de thé; depository not designated; ♂; in *Baliotrichia*]. —Xue and Yang 1991: 22 [distribution]. —Yang et al. 1997b: 93 [checklist]. —Yang et al. 2005: 458 [checklist; distribution]. —Malicky and Chantaramongkol 2007: 1040 [♂; distribution]. —Zhou et al. 2010: 40 [checklist]. —Yang et al. 2016: 476 [checklist].

**Distribution.** —China, Sri Lanka.

♰ ***umbra*** Melnitsky & Ivanov, 2016: 283 [type locality: [Ukraine], Rovno Amber, Bartonian, Eocene; IZSK; ♂; ♀].

**Distribution.** —Rovno amber.

***urania*** Malicky & Chantaramongkol, 2007: 1032 [type locality: Thailand, Prov. Lampang, Chaeson NP, 18°46'N 99°28'E, 500 m; Collection Malicky; ♂]. —Malicky 2010a: 50 [atlas; ♂].

**Distribution.** —Thailand.

***urimica*** Wells, 1984: 279 [type locality: [Papua] New Guinea, D.P.I Urimo Station; BPBM; ♂; ♀]. —Neboiss 1986: 93 [atlas; ♂; ♀]. —Wells 1991: 526 [checklist].

**Distribution.** —Papua New Guinea.

***vadalis*** Mey & de Moor, 2019: 142 [type locality: Namibia, Kunene River, Epupa Falls, 17°00'24"S, 13°14'52"E; ZMHB; ♂].

**Distribution.** —Namibia.

***vakrata*** Oláh & Johanson, 2010a: 61 [type locality: Indonesia, Sumatra, Way Titias, Bukit Barisan Selatan NP, 950 m; Collection Oláh; ♂].

**Distribution.** —Indonesia.

***velata*** Wells, 1983: 641 [type locality: Australia, Queensland, Upper Ross R., below weir; NMV; ♂; ♀]. —Wells 1985b: 30 [larva]. —Neboiss 1986: 88 [atlas; ♂; ♀]. —Wells 1990c: 125 [case; distribution]. —Wells et al. 2019: 33 [detection frequency].

**Distribution.** —Australia .

***verbekei*** Jacquemart, 1957: 122 [type locality: [Congo], Lac Édouard, Mosenda; IRSNB; ♂]. —Guenda, 1996: 243 [distribution].

**Distribution.** —Burkina Faso, Congo.

***vertumnus*** Malicky & Chantaramongkol, 2007: 1032 [type locality: Vietnam, Man Cat Tien NP, 11°26'N 107°26'E, 200 m; Collection Malicky; ♂]. —Malicky 2010a: 50 [atlas; ♂].

**Distribution.** —Vietnam.

***waridora*** Oláh, 2013: 74 [type locality: Indonesia, Papua, Raja Ampat, Batanta Island, northern coast, Waridor river, 0.86492°S, 130.52206°E; Collection Oláh; ♂].

**Distribution.** —Indonesia.

***warmona*** Oláh, 2012: 51 [type locality: Indonesia, Papua, Raja Empat Archipelago, Batanta Island, Warmon Creek, 2. waterfall, 0°50'23.25"S 130°42'35.18"E; Collection Oláh; ♂]. —Oláh and Kovács 2018: 184 [distribution].

**Distribution.** —Indonesia.

***wellsae*** Xue & Yang, 1990: 128 [type locality: [China] Longsheng, Guangxi; NAUJ; ♂]. —Yang et al. 1997b: 93 [checklist]. —Yang et al. 2005: 458 [checklist]. —Malicky and Chantaramongkol 2007: 1037 [♂; distribution]. —Zhou et al. 2010: 40 [checklist]. —Malicky 2010a: 46 [atlas; ♂]. —Yang et al. 2016: 476 [checklist]. —Melnitsky et al. 2019: 539 [distribution].

**Distribution.** —China, Malaysia, Thailand.

***yabbaca*** Wells, 1983: 642 [type locality: Australia, Queensland, Yabba Creek, 10 km W. Imbil; NMV; ♂; ♀]. —Neboiss 1986: 93 [atlas; ♂; ♀].

**Distribution.** —Australia.

***yaowachon*** Malicky & Chantaramongkol, 2007: 1039 [type locality: Thailand, Kao Yai NP, Yaowachon campsite bei Kong Kaeo WF, 680 m; Collection Malicky; ♂]. —Malicky 2010a: 44 [atlas; ♂].

**Distribution.** —Thailand.

***zonata*** (Neboiss, 1977): 41 [type locality: [Australia] Tasmania, St. Patricks River, Targa; NMV; ♂; ♀; in *Targatrichia*]. —Wells 1979a: 591 [♂; ♀; distribution; to *Orthotrichia*]. —Neboiss 1986: 85 [atlas; ♂; ♀]. —Neboiss 2002: 54 [checklist].

—*capa* Oláh & Johanson, 2010a: 42 [type locality: Australia, Tasmania, Wilds Rivers NP, Collingswood River, 100 m upstream bridge on A10, 42°09.718'S, 145°55.602'E, 337 m; ANIC; ♂]. —Wells 2012: 67 [to synonymy].

**Distribution.** —Australia.

#### Genus *Saranganotrichia* Ulmer, 1951

*Saranganotrichia* Ulmer, 1951: 58, 83 [type species: *Saranganotrichiadecussata* Ulmer, 1951, original designation]. —Marshall 1979: 216 [synonymized with *Ithytrichia* Eaton, 1873]. —Malicky 2009a: 16 [resurrected from synonymy].

*Huayptila* Malicky & Chantaramongkol, 2007: 1025 [type species: *Huayptilakaosoidao* Malicky & Chantaramongkol, 2007, original designation]. —Malicky 2009a: 16 [to synonymy].

*Saranganotrichia* consists of four species recorded from Thailand and Indonesia. The genus was established by Ulmer (1951) based largely on features of the wings, which are no longer considered reliable characters in Hydroptilidae. Based on similarities in the larvae and the cases, the genus was synonymized with *Ithytrichia* by Marshall (1979b), who also expressed doubts about the quality of Ulmer’s original preparations of larval *Saranganotrichia*. The genus was later resurrected by Malicky (2009a), based on a re-examination of the larval material. The larval stage of *S.decussata* was described by Ulmer (1957).

***chiangdao*** (Malicky & Chantaramongkol, 2007): 1026 [type locality: Thailand, Mae Ping beim Elephant Camp 12 km S von Chiang Dao, 19°16'N 98°58'E, 360 m; Collection Malicky; ♂; in *Huayptila*]. —Malicky 2010a: 39 [atlas; ♂].

**Distribution.** —Thailand.

***decussata*** Ulmer, 1951: 84 [type locality: [Indonesia], Java, Sarangan, 1450 m; ZMUH; ♂; in *Javanotrichia*]. —Malicky 2009a: 16 [♂]. —Malicky 2010a: 39 [atlas; ♂]. —Malicky et al. 2014a: 6 [distribution].

**Distribution.** —Indonesia.

***kaosoidao*** (Malicky & Chantaramongkol, 2007): 1026 [type locality: Thailand, Kao Soi Dao NP, 13°06'N 102°10'E, 400 m; Collection Malicky; ♂; in *Huayptila*]. —Malicky 2010a: 39 [atlas; ♂].

**Distribution.** —Thailand.

***oldalra*** Oláh, 2012: 49 [type locality: Indonesia, Papua, Raja Empat Archipelago, Batanta Island, Site A, 0°50'04.03"S 130°42'54.14: E; Collection Oláh; ♂]. —Oláh and Kovács 2018: 179 [distribution].

**Distribution.** —Indonesia.

### ﻿Subfamily STACTOBIINAE Botosaneanu, 1956

Stactobiinae Botosaneanu, 1956: 382 [type genus: *Stactobia* McLachlan, 1880]. —Marshall 1979b: 163 [reviewed as tribe Stactobiini]. —Wells 1990a: 817, 820 [phylogeny; key to males and fully mature, cased larvae of genera in New Guinea]. —Bowles et al. 1999: 43 [larval morphology; systematics]. —Harri et al. 2002b: 58 [key to New World genera]. —Malicky and Chantaramongkol 2007: 1042 [discussion of taxonomic limits].

The subfamily Stactobiinae currently consists of 12 described genera with a Holarctic and Oriental distribution, although generic diversity may be greatest in Southeast Asia (Wells 1990a). The subfamily was originally established for the genus *Stactobia* and other closely related genera, which at the time were not named (Botosaneanu 1956). Most likely, Botosaneanu intended to include the genera *Stacobiella*, *Plethus*, *Plethotrichia*, and *Lamonganotrichia* (Marshall 1979b). Ulmer (1957) and Schmid (1959a) provided additional comments regarding the relationships between these and other genera (*Chrysotrichia*, *Macrostactobia*, *Madioxyethira*, *Parastactobia*, *Pseudoxyethira*). Flint (1970) subsequently placed *Plethus* and *Lamonganotrichia* in Stactobiinae, based on morphological features of the larvae and their cases.

Subsequent works have re-evaluated the status and composition of Stactobiinae. Marshall (1979b) concluded that incorrect interpretations of the spur formula, presence of ocelli, and features of the wing venation had led to errors in original generic diagnoses. The grouping that she presented was instead based on features of the male and female genitalia, head and thoracic structures, and amended ocellar counts and spur formulae. Wells (1990a) also re-evaluated Stactobiinae and remarked on the difficulty of maintaining the group when she had to modify and expand Marshall’s (1979b) description of the subfamily in order to account for variations in the spur formula and wing venation. In this work, Wells provided a re-description of the subfamily, a modification based on Marshall’s work, that included features of the adult, pupa, and mature larva. Bowles et al. (1999) agreed that uniting Stactobiinae was problematic and stated that several of the New World genera in particular shared many similar features with members of Leucotrichiinae. Based on a suite of larval characters they considered to be derived for Leucotrichiinae, Bowles and coauthors transferred several genera to Leucotrichiinae. These authors stated that they could not find any uniquely derived larval characters to unite Stactobiinae. Malicky and Chantaramongkol (2007) briefly commented on the taxonomic limits of Stactobiinae and agreed that the subfamily is difficult and that generic limits are often ambiguous. They did not offer any characters that could be used to diagnose or unite the subfamily. Larval descriptions are available for all genera except *Maetalaiptila*, *Orinocotrichia*, and *Tizatetrichia*.

#### Genus *Bredinia* Flint, 1968

*Bredinia* Flint, 1968b: 50 [type species: *Brediniadominicensis* Flint, 1968b, original designation]. —Marshall 1979b: 170 [generic review]. —Angrisano 2002: 398 [♀, larva]. —Harris et al. 2002a: 14 [revision].

Seventeen species currently represent the genus *Bredinia*, which occurs in the Lesser Antilles and is restricted in distribution to the Neotropical faunal region. Flint (1968b) considered *Bredinia* to have affinities with several different genera placed outside of Stactobiinae (*Alisotrichia*, *Mayatrichia*, *Neotrichia*), but stated that similarities in the thoracic nota and the male genitalia made the genus most similar to *Stactobiella* (1968).

***alza*** Harris, Holzenthal, & Flint, 2002: 35 [type locality: Paraguay, Concepción, Concepción; NMNH; ♂]. —Angrisano and Sganga 2007: 28 [♂; larva; distribution].

**Distribution.** —Argentina, Paraguay.

***appendiculata*** Flint & Sykora, 1993: 56 [type locality: Grenada, Parish St. Andrews, Balthazar Estate; FSCA; ♂]. —Flint and Sykora,1993: 49 [checklist]. —Botosaneanu 2002b: 82 [checklist]. —Harris et al. 2002a: 22 [♂; ♀; re-description; distribution].

**Distribution.** —Grenada, Peru, Venezuela.

***costaricensis*** (Flint, 1967b): 13 [type locality: Costa Rica, La Lola near Martina; NMNH; ♂; in *Neotrichia*]. —Holzenthal 1988: 62 [distribution]. —Flint et al. 1999b: 76 [to *Bredinia*]. —Harris et al. 2002a: 24 [♂; ♀; re-description; distribution]. —Armitage et al. 2015a: 6 [checklist]. —Armitage and Harris 2018b: 97 [checklist].

**Distribution.** —Costa Rica, Panama.

***davenporti*** Harris, Holzenthal, & Flint, 2002: 24 [type locality: Peru, Loreto, Río Sucusari at Explornapo Camp; NMNH; ♂].

**Distribution.** —Peru.

***dominicensis*** Flint, 1968a: 51 [type locality: Dominica, Hodges River mouth, swamp forest; NMNH; ♂; ♀]. —Flint and Sykora 1993: 49 [checklist]. —Flint 1996b: 90 [distribution]. —Botosaneanu 2002b: 82 [checklist]. —Harris et al. 2002a: 15 [♂; ♀; re-description; distribution]. —Botosaneanu and Thomas 2005: 38 [distribution]. —Armitage et al. 2015a: 6 [checklist]. —Ríos-Touma et al. 2017: 9 [checklist]. —Armitage and Harris 2018b: 97 [checklist].

**Distribution.** —Costa Rica, Dominica, Ecuador, Martinique, Panama, Trinidad.

***dudosa*** Bueno-Soria & Barba-Álvarez, 2018: 363 [type locality: Mexico, Chiapas, Reserva de la Biósfera Montes azules, Est. Biol. Chajul, Arroyo José, 16°06'50.0"N, 90°56'03.3"W, 150 m asl; CNIN; ♂].

**Distribution.** —Mexico.

***emarginata*** Harris, Holzenthal, & Flint, 2002: 37 [type locality: Costa Rica, Alajuela, Río Pizote, ca 5 km N Dos Ríos, 10.948°N, 85.291°W; NMNH; ♂]. —Armitage et al. 2016: 6 [distribution]. —Armitage and Harris 2018b: 97 [checklist].

**Distribution.** —Costa Rica, Panama.

***espinosa*** Harris, Holzenthal, & Flint, 2002: 20 [type locality: Ecuador, Los Ríos, Quevedo (56 km N), Río Palenque Biological Station; NMNH; ♂; ♀]. —Paprocki et al. 2004: 11 [checklist]. —Nogueira and Cabette 2011: 351 [distribution]. —Oláh and Johanson 2011: 248 [distribution]. —Paprocki and França 2014: 42 [checklist]. —Ríos-Touma et al. 2017: 9 [checklist].

**Distribution.** —Brazil, Ecuador, French Guiana, Venezuela.

***guanacasteca*** Harris, Holzenthal, & Flint, 2002: 17 [type locality: Costa Rica, Guanacaste, Río Tempisquito, ca 3 km S route 1, 10.790°N, 85.552°W, 75 m; NMNH; ♂].

**Distribution.** —Costa Rica.

***manabiensis*** Harris, Holzenthal, & Flint, 2002: 27 [type locality: Ecuador, Manabi, 29 km W Santo Domingo, Rancho Ronald; NMNH; ♂]. —Ríos-Touma et al. 2017: 9 [checklist].

**Distribution.** —Ecuador.

***mexicana*** Harris, Holzenthal, & Flint, 2002: 35 [type locality: Mexico, Tamaulipas, Río Frio at La Poza Azul near Gómez Farias; NMNH; ♂].

**Distribution.** —Mexico.

***pilcopata*** Harris, Holzenthal, & Flint, 2002: 32 [type locality: Peru, Cuzco, Pilcopata, 600 m; NMNH; ♂]. —Oláh and Johanson 2011: 248 [distribution].

**Distribution.** —Peru.

***selva*** Harris, Holzenthal, & Flint, 2002: 19 [type locality: Costa Rica, Heredia, Estación Biológica La Selva; NMNH; ♂].

**Distribution.** —Costa Rica.

***spangleri*** Harris, Holzenthal, & Flint, 2002: 34 [type locality: Ecuador, Pastaza, Puyo (16 km W); NMNH; ♂]. —Ríos-Touma et al. 2017: 9 [checklist].

**Distribution.** —Ecuador.

***sucrensis*** Harris, Holzenthal, & Flint, 2002: 37 [type locality: Venezuela, Sucre, Parque Nacional Peninsula de Paria, Uquire, Río La Viuda, 10°42.830'N, 61°57.661'W, 15 m; NMNH; ♂; ♀]. —Armitage et al. 2018: 5 [distribution].

**Distribution.** —Panama, Venezuela.

***venezuelensis*** Harris, Holzenthal, & Flint, 2002: 29 [type locality: Venezuela, Zulia, Perija El Tucuco, Mission El Tucuco, Río El Tucuco, 11 km from church; NMNH; ♂; ♀]. —Oláh and Johanson 2011: 248 [distribution]. —Ríos-Touma et al. 2017: 9 [checklist].

**Distribution.** —Ecuador, Peru, Venezuela.

***zulia*** Harris, Holzenthal, & Flint, 2002: 39 [type locality: Venezuela, Zulia, El Tucuco, Sierra de Perija; NMNH; ♂].

**Distribution.** —Venezuela.

#### Genus *Catoxyethira* Ulmer, 1912

*Catoxyethira* Ulmer, 1912b: 82 [type species: *Catoxyethirafasciata* Ulmer, 1912b, monotypic]. —Marshall 1979b: 171 [generic review]. —Gibon 1985: 154 [key to Côte d’Ivoire species]. —Gibon 1993: 200 [key to species groups]. —Wells and Andersen 1996: 87 [key to Tanzanian species].

*Sperotrichia* Marlier, 1978: 294 [type species: *Sperotrichiamali* Marlier, 1978, original designation]. —Marshall 1979b: 171 [to synonymy].

*Parastactobia* Schmid, 1958b: 48 [type species: *Parastactobiatalakalahena* Schmid, 1958b, original designation]. —Marshall 1979b: 173 [generic review]. —Malicky and Chantaramongkol 2007: 1053 [re-description]. —Oláh and Johanson 2010a: 62 [to synonymy].

The genus *Catoxyethira* consists of 68 species occurring mostly in Africa. A single species, *C.prima*, has been recorded from the Philippines and Southeast Asia (Mey 2003b). The larva and case of an unidentified species, later placed in *Catoxyethira*, were described by Ulmer (1912b). Several structural similarities occurring in the spur formula and male genitalia are present in adults of *Catoxyethira* and *Stactobiella*, as noted by Morse (1974). Marshall (1979b) also concluded that the genus belongs in the *Stactobiella* group of Stactobiinae, based on features of the adult head and thorax.

***abongae*** Gibon, 1993: 201 [type locality: [Cameroon], sur la Ngoué (bassin dy Nyong) à Pouma; MNHN; ♂].

**Distribution.** —Cameroon.

***ajsae*** Oláh & Johanson, 2010a: 62 [type locality: Indonesia, Sumatra, Way Titias, Bukit Barisan Selatan NP, 950 m; Collection Oláh; ♂].

**Distribution.** —Indonesia.

***apicospinosa*** Wells & Andersen, 1995: 151 [type locality: Tanzania, Tanga region, West Usambara Mts, Dule, Bumbuli River, 1220 m a.s.l.; ZMUB; ♂].

**Distribution.** —Tanzania.

***badyi*** Gibon, 1991: 129 [type locality: [Guinea], au piège lumineux sur le Konkouré à Bady; MNHN; ♂].

**Distribution.** —Guinea.

***bilongae*** Gibon, 1993: 202 [type locality: [Cameroon], sur le Méfou (bassin dy Nyong) à l’intersection avec la route Yaoundé/Douala; MNHN; ♂].

**Distribution.** —Cameroon.

***bombolensis*** Wells & Andersen, 1995: 149 [type locality: Tanzania, Tanga region, East Usambara Mts, Bombole, 830 m a.s.l; ZMUB; ♂].

**Distribution.** —Tanzania.

***botosaneanui*** Guenda, 1997: 221 [type locality: [Burkian Faso], zone des sources du Mouhoun à Fon; UOBF; ♂].

**Distribution.** —Burkina Faso.

***catichae*** Gibon & Ranaivoharindriaka, 1995: 113 [type locality: [Madagascar], sur la Mananara (bassin du Mandrare) à Betanimena près d’Amboasary atsimo; MNHN; ♂].

**Distribution.** —Madagascar.

***cavallyi*** Gibon, 1985: 153 [type locality: Côte-D’ivoire, affluent du Cavally à Wa; MNHN; ♂]. —Gibon, 1991: 128 [♂]. —Kjærandsen and Andersen 1997: 244 [distribution].

**Distribution.** — Côte d’Ivoire, Ghana.

***ciliata*** Wells & Andersen, 1995: 155 [type locality: Tanzania, Tanga region, West Usambara Mts, Mazumbai, Kaputu Stream, loc. 3, 1720 m a.s.l.; ZMUB; ♂].

**Distribution.** —Tanzania.

***crenulata*** Wells & Andersen, 1995: 153 [type locality: Tanzania, Morogoro region, Morogoro, Sokoine University of Agriculture, 550 m a.s.l.; ZMUB; ♂].

**Distribution.** —Tanzania.

***crinita*** Wells & Andersen, 1995: 154 [type locality: Tanzania, Tanga region, West Usambara Mts, Mazumbai, Kaputu Stream, loc. 5, 1650 m a.s.l.; ZMUB; ♂]. —Wells and Andersen 1996: 87 [distribution].

**Distribution.** —Tanzania.

***darrieti*** Gibon, 1993: 204 [type locality: [Cameroon], sur l’Ombe River à l’intersection avec la route Douala/Limbe; MNHN; ♂].

**Distribution.** —Cameroon.

***decampei*** Gibon in Gibon and Ranaivoharindriaka 1995: 109 [type locality: [Madagascar], sur la Namorona, à 11 km en aval de Ranomafana, province de Fianarantsoa; MNHN; ♂].

**Distribution.** —Madagascar.

***disymetrica disymetrica*** Gibon, 1991: 127 [type locality: [Guinea], au piège lumineux sur le Niandan (bassin du Niger) à Bambaya (région de Kissidougou); MNHN; ♂].

**Distribution.** —Guinea.

***disymetrica yaoundeensis*** Gibon, 1993: 202 [type locality: [Cameroon], sur l’Assamba (bassin de la Sanaga) à Nkomeyo (région de Yaoundé); MNHN; ♂].

**Distribution.** —Cameroon.

***djenebae*** Guenda, 1997: 221 [type locality: [Burkian Faso], zone des sources du Mouhoun à Fon; UOBF; ♂].

**Distribution.** —Burkina Faso.

***duatali*** Wells & Malicky, 1997: 179 [type locality: [Indonesia] East Java, Meru Betiri, stream in savannah; ANIC; ♂; in *Chrysotrichia*]. —Malicky and Chantaramongkol 2007: 1054 [to *Parastactobia*]. —Oláh and Johanson 2010a: 62 [to *Catoxyethira*]. —Malicky 2010a: 59 [atlas; ♂]. —Malicky et al. 2014a: 6 [distribution; as *Parastactobia*]. —Malicky et al. 2016: 92 [distribution; as *Parastactobia*].

**Distribution.** —Indonesia.

***elongata*** Wells & Andersen, 1995: 153 [type locality: Tanzania, Morogoro region, Kimboza, Ruvu River, 150 m a.s.l.; ZMUB; ♂].

**Distribution.** —Tanzania.

***elouardi*** Gibon, 1987b: 115 [type locality: Guinea, small affluent of the Milo, near Konsankoro on the track to Beyla; MNHN; ♂].

**Distribution.** —Guinea.

***fasciata*** Ulmer, 1912b: 82 [type locality: [Democratic Republic of Congo], Kongo, Kinchassa; IRSNB; ♂].

**Distribution.** —Democratic Republic of Congo.

***fonensis*** Guenda, 1997: 219 [type locality: [Burkina Faso], zone des sources du Mouhoun près du petit village de Fon, à 500 m d’altitude sur l’axe Bobo-Dioulasso-Orodata; UOBF; ♂].

**Distribution.** —Burkina Faso.

***fonkouae*** Gibon, 1993: 205 [type locality: [Cameroon], sur l’Afamba à Oboua (région de Yaoundé; MNHN; ♂].

**Distribution.** —Cameroon.

***formosae*** (Iwata, 1928): 343 [type locality: [China, Taiwan], Urai, Formosa; depository not designated; larva; in *Hydroptila*]. —Yang et al. 2016: 475 [checklist; in *Catoxyethira*].

**Distribution.** —China.

***foumbani*** Gibon, 1993: 203 [type locality: [Cameroon], sur le Manem (affluent de Mbam, bassin de la Sanaga) à Foumban; MNHN; ♂].

**Distribution.** —Cameroon.

***gariepensis*** Mey, 2011: 349 [type locality: Namibia, Noordoewer, Orange River at Felix Unite; ZMHB; ♂].

**Distribution.** —Namibia.

***giboni*** Wells & Andersen, 1996: 87 [type locality: Tanzania, Uluguru Mts, Morogoro River, 600 m; ZMUC; ♂].

**Distribution.** —Tanzania.

***gimouae*** Gibon, 1993: 205 [type locality: [Cameroon], sur la Ngoué (bassin dy Nyong) à Pouma; MNHN; ♂].

**Distribution.** —Cameroon.

***giudicellii*** Guenda, 1997: 223 [type locality: [Burkian Faso], zone des sources du Mouhoun, près du village de Kourinion; UOBF; ♂].

**Distribution.** —Burkina Faso.

***graboensis*** Gibon, 1985: 154 [type locality: Côte-D’ivoire, affluent du Cavally à quelques kilomètres au nord de Grabo; MNHN; ♂]. —Gibon 1987b: 118 [distribution]. —Gibon et al. 1994: 109 [distribution].

**Distribution.** —Burkina Faso, Côte d’Ivoire, Guinea.

***gura*** Malicky, 2020: 510 [type locality: [Kenya], Kenia, Gura River, power plant, 0°30'S, 36°53'E; collection Malicky; ♂].

**Distribution.** —Kenya.

***hougardi*** Gibon, 1985: 153 [type locality: Côte-D’ivoire, affluent du Cavally à Wa; MNHN; ♂]. —Gibon 1987b: 118 [distribution]. —Gibon 1993: 205 [distribution].

**Distribution.** —Cameroon, Côte d’Ivoire, Guinea.

***iloui*** Gibon, 1993: 203 [type locality: [Cameroon], sur le Mayo Ilou (bassin de la Bénoué) à Finyolé (région de Poli); MNHN; ♂].

**Distribution.** —Cameroon.

***improcera*** Statzner, 1977: 398 [type locality: Zaire, Kivu Region, Kalengo stream 10 km west of Lake Kivu; ZMHB; ♂; ♀].

**Distribution.** —Congo.

***incompta*** Wells & Andersen, 1995: 149 [type locality: Tanzania, Tanga region, West Usambara Mts, Mazumbai, Kaputu Stream, loc. 4, 1680 m a.s.l; ZMUB; ♂].

**Distribution.** —Tanzania.

***khakaeng*** (Malicky & Chantaramongkol, 2007): 1054 [type locality: Thailand, Huai Kha Kaeng WS, headquarters, 15°36'N 99°19'E, 300 m; ♂; in *Parastactobia*]. —Oláh and Johanson 2010a: 64 [distribution]. —Malicky 2010a: 59 [atlas; ♂]. —Malicky et al. 2014c: 34 [checklist].

**Distribution.** —Cambodia, Laos, Thailand.

***kourinioni*** Guenda, 1997: 223 [type locality: [Burkian Faso], zone des sources du Mouhoun, près du village de Kourinion; UOBF; ♂].

**Distribution.** —Burkina Faso.

***kumiskucinga*** (Wells, 1990b): 373 [type locality: [Indonesia] Sulawesi Utara, Motolanga R., Doloduo-Malibagu road; NMV; ♂; in *Parastactobia*].

**Distribution.** —Indonesia.

***kunenica*** Mey & de Moor, 2019: 141 [type locality: Namibia, Kunene River, Swartbooisdrif, Kunene River Lodge, 17°20'50"S, 13°52'56"E; ZMHB; ♂]. —Wells and de Moor 2020: 497 [distribution].

**Distribution.** —Angola, Namibia.

***lanceolata*** Wells & Andersen, 1995: 151 [type locality: Tanzania, Tanga region, East Usambara Mts, Mlesa, 800 m a.s.l.; ZMUB; ♂].

**Distribution.** —Tanzania.

***laurenceae*** Gibon, 1993: 201 [type locality: [Cameroon], sur l’Ombe River au niveau de la route Douala/Limbe; MNHN; ♂].

**Distribution.** —Cameroon.

***lelouma*** Gibon, 1991: 127 [type locality: [Guinea], au piège lumineux sur un petit affluent du Tominé (bassin du Rio Corubal) dans la région de Télimélé; MNHN; ♂]. —Kjærandsen and Andersen 1997: 244 [distribution].

**Distribution.** —Ghana, Guinea.

***leynarti*** Gibon, 1987b: 117 [type locality: Guinea, on the Niger, upstream of the Kissidougou-Faranah road; MNHN; ♂]. —Gibon 1993: 203 [distribution]. —Kjærandsen and Andersen 1997: 244 [distribution].

**Distribution.** —Cameroon, Ghana, Guinea.

***lohoueae*** Gibon, 1993: 204 [type locality: [Cameroon], sur la Ngoué (bassin du Nyong) à Pouma; MNHN; ♂].

**Distribution.** —Cameroon.

***mali*** (Marlier, 1978): 295 [type locality: Mali, pont de Kouoro; MRAC; ♂; in *Sperotrichia*]. —Gibon, 1985: 151 [distribution]. —Gibon 1987b: 118 [distribution]. —Gibon 1993: 203 [distribution]. —Gion and Ranaivoharindriaka 1995: 113 [♂; distribution]. —Kjærandsen and Andersen 1997: 244 [distribution].

**Distribution.** —Cameroon, Côte d’Ivoire, Ghana, Guinea, Madagascar, Mali.

***margemiring*** (Wells & Malicky, 1997): 178 [type locality: N Sumatra, Huta Padang, 02°45'N 99°14'E; Collection Malicky; ♂; in *Chrysotrichia*]. —Malicky and Chantaramongkol 2007: 1054 [to *Parastactobia*]. —Malicky 2007a: 177 [checklist; in *Parastactobia*]. —Oláh and Johanson 2010a: 62 [to *Catoxyethira*]. —Malicky 2010a: 59 [atlas; ♂].

**Distribution.** —Indonesia.

***mouensis*** Gibon, 1993: 206 [type locality: [Cameroon], sur la Mou à Nkounden (région de Foumban); MNHN; ♂].

**Distribution.** —Cameroon.

***namoronae*** Gibon, 1995 in Gibon and Ranaivoharindriaka 1995: 111 [type locality: [Madagascar], sur la Namorona, à 11 km en aval de Ranomafana, province de Fianarantsoa; MNHN; ♂].

**Distribution.** —Madagascar.

***nzoi*** Gibon, 1985: 154 [type locality: Côte-D’ivoire, sur le Nzo au niveau de la piste Man/Danané; MNHN; ♂]. —Gibon 1987b: 118 [distribution]. —Gibon 1993: 202 [distribution]. —Kjærandsen and Andersen 1997: 244 [distribution].

**Distribution.** —Cameroon, Côte d’Ivoire, Ghana, Guinea.

***ocellata*** Statzner, 1977: 396 [type locality: [Congo], Zaire, Kivu Region, Kalengo stream 10 km west of Lake Kivu; ZMHB; ♂; ♀]. —Wells and Andersen 1995: 151 [distribution].

**Distribution.** —Congo, Tanzania.

***ombeensis*** Gibon, 1993: 205 [type locality: [Cameroon], sur l’Ombe River à l’intersection avec la route Douala/Limbe; MNHN; ♂].

**Distribution.** —Cameroon.

***pinheyi*** Kimmins, 1958a: 365 [type locality: [Zimbabwe], S. Rhodesia, Victoria Falls; NMZ; ♂]. —Gibon 1985: 153 [distribution]. —Johanson 1992: 118 [checklist]. —Gibon 1993: 204 [distribution]. —Wells and Andersen 1995: 145 [checklist]. —Guenda 1997: 219 [distribution]. —Kjærandsen and Andersen 1997: 244 [distribution].

**Distribution.** —Burkina Faso, Cameroon, Côte d’Ivoire, Ghana, Zimbabwe.

***pougoueae*** Gibon, 1993: 205 [type locality: [Cameroon], sur un petit affluent forestier de la Sanaga situé entre Sakbayémé et Song-Loulou; MNHN; ♂].

**Distribution.** —Cameroon.

***prima*** Mey, 2003b: 428 [type locality: Philippines, Luzon, Quezon province, east of Infanta, Magsaysay; ZMHB, to be transferred to either MPMP or UPLB; ♂].

**Distribution.** —Philippines.

***razanamiadanae*** Gibon in Gibon and Ranaivoharindriaka 1995: 107 [type locality: [Madagascar], sur la Namorona, à 11 km en aval de Ranomafana, province de Fianarantsoa; MNHN; ♂].

**Distribution.** —Madagascar.

***robisoni*** Gibon & Ranaivoharindriaka, 1995: 111 [type locality: [Madagascar], sur la Namorona, à 11 km en aval de Ranomafana, province de Fianarantsoa; MNHN; ♂].

**Distribution.** —Madagascar.

***ruvuensis*** Wells & Andersen, 1995: 151 [type locality: Tanzania, Morogoro Region, Kimboza, Ruvu River, 150 m a.s.l.; ZMUB; ♂].

**Distribution.** —Tanzania.

***spinifera*** Gibon, 1985: 154 [type locality: Côte-D’ivoire, affluent du Cavally à Wa; MNHN; ♂]. —Gibon 1987b: 118 [distribution]. —Kjærandsen and Andersen 1997: 244 [distribution].

**Distribution.** — Côte d’Ivoire, Ghana, Guinea.

***stolzei*** Wells & Andersen, 1996: 87 [type locality: Tanzania, Uzungwa Mts, Mwanihana Forest, Sanje River, 300–400 m; ZMUC; ♂].

**Distribution.** —Tanzania.

***taengdoa*** (Malicky & Chantaramongkol, 2007): 1054 [type locality: Thailand, Mae Ping beim Royal Ping Resort, 9 km N Mae Taeng, 19°12'N 98°58'E, 350 m; ♂; in *Parastactobia*]. —Malicky 2010a: 59 [atlas; ♂].

**Distribution.** —Thailand.

***taiensis*** Gibon, 1985: 151 [type locality: Côte-D’ivoire, Cavally riv. à Taï; MNHN; ♂]. —Gibon 1987b: 118 [distribution]. —Gibon 1993: 202 [distribution].

**Distribution.** —Cameroon, Côte d’Ivoire, Guinea.

***talakalahena*** (Schmid, 1958b): 48 [type locality: [Sri Lanka], Ceylan, Gurudeniya (C. P., 1500 ft) 15-I, Talatu Oya, petite rivière aux eaux claires et agitées, dans forêt clairsemée; depository not designated; ♂; in *Parastactobia*]. —Malicky and Chantaramongkol 2007: 1099 [♂].

**Distribution.** —Sri Lanka.

***tonyeae*** Gibon, 1993: 201 [type locality: [Cameroon], sur le Ouem (bassin de la Sanaga) à Song-Loulou; MNHN; ♂].

**Distribution.** —Cameroon.

***vanandeli*** Guenda, 1997: 222 [type locality: [Burkian Faso], zone des sources du Mouhoun, à Kourinion; UOBF; ♂].

**Distribution.** —Burkina Faso.

***vedonga*** Oláh, 1989: 266 [type locality: Vietnam, Cucphuong, 400 m a.s.l.; HNHM; ♂]. —Armitage et al. 2005: 28 [checklist]. —Malicky 2010a: 56 [atlas; ♂].

**Distribution.** —Vietnam.

***veruta veruta*** Morse, 1974: 335 [type locality: [Zimbabwe], Kariba, Southern Rhodesia, 636 m; FSCA; ♂]. —Gibon 1993: 202 [distribution]. —Kjærandsen and Andersen 1997: 244 [distribution]. —de Moor et al. 2000: 112 [distribution]. —Mey and de Moor 2019: 137, 139 [checklist; distribution].

**Distribution.** —Cameroon, Ghana, Zimbabwe.

***veruta septentrionalis*** Gibon, 1985: 151 [type locality: Côte-D’ivoire, Cavally riv. à Taï; MNHN; ♂]. —Gibon 1987b: 118 [distribution]. —Kjæerandsen and Andersen 1997: 244 [distribution].

**Distribution.** — Côte d’Ivoire, Ghana, Guinea.

***wouafondayoae*** Gibon, 1993: 203 [type locality: [Cameroon], sur le Noun (bassin de la Sanaga) à quelques kilomètres en amont de Bafia; MNHN; ♂].

**Distribution.** —Cameroon.

#### Genus *Chrysotrichia* Schmid, 1958

*Chrysotrichia* Schmid, 1958b: 54 [type species: *Chrysotrichiahatnagola* Schmid, 1958b, original designation]. —Marshall 1979b: 170 [generic review]. —Wells 1990b: 367 [larva; key to North Sulawesi species]. —Wells 1990a: 835 [key to species of New Guinea].

*Chrysotrichia* currently consists of 70 species, occurring in south and Southeast Asia. Schmid (1958b) concluded that the genus is most closely related to *Plethus*, due to similarities between the male genitalia, while Marshall (1979b) stated that it is also very similar to the *ulmeri* group of *Stactobiella*, based on features of the adult head and thorax and the male genitalia. The final instar larva of *C.berduri* was described by Wells (1990b).

***angkup*** Wells & Huisman, 1993: 95 [type locality: West Malaysia, Kepong, Forest Research Institute of Malaya, on falls; NTM; ♂]. —Malicky 2010a: 68 [atlas; ♂].

**Distribution.** —Malaysia.

***aningalan*** Wells & Mey, 2002: 123 [type locality: [Philippines] Panay, San Reminigio, Aningalan; ZMHB; ♂]. —Malicky 2013: 42 [possible senior synonym to *C.atugan*].

**Distribution.** —Philippines.

***aranuwa*** Schmid, 1958b: 57 [type locality: [Sri Lanka], Ceylan, Ambagaswewa (N. C. P., 400 ft) 22-III, petite rivière assez agitée, à dalles rocheuses et bancs de sable, dans la forêt; depository not designated; ♂].

**Distribution.** —Sri Lanka.

***arapela*** Wells, 1990a: 837 [type locality: Papua New Guinea, Central Province, stream in Kanosia Rubber Plantation, on Port Moresby-Bereina road; ANIC; ♂; ♀]. —Wells 1991: 526 [checklist].

**Distribution.** —Papua New Guinea.

***armiger*** Mey, 2003b: 430 [type locality: Philippines, Leyte, Baybay, Mt. Panasugan; ZMHB, to be transferred to either MPMP or UPLB; ♂].

**Distribution.** —Philippines.

***atugan*** Wells & Mey, 2002: 121 [type locality: [Philippines] Mindano, Atugan River, Bukidnon, 1800 m; BPBM; ♂]. —Malicky 2013: 42 [possible junior synonym to *C.aningalan*].

**Distribution.** —Philippines.

***australis*** Wells, 1990c: 108 [type locality: NE Queensland, Yuccabine Creek; NMV; ♂].

**Distribution.** —Australia.

***bachma*** Murray-Stoker & Morse in Murray-Stoker et al. 2020: 100 [type locality: [Vietnam], Bach Mã National Park, Thùra Thiên Huê Province, tributary to Pheasant Falls (tributary to Truoi River), 16.2287°N, 107.8486°E, 159 m; VNMN; ♂].

**Distribution.** —Vietnam.

***badhami*** Schmid, 1960: 91 [type locality: [Pakistan] Penjab, Hassan Abdal; CNC; ♂]. —Schmid 1958c: 220 [as new species, *nomen nudum*; distribution]. —Lonsdale 2020: 32 [holotype depository].

**Distribution.** —Pakistan.

***barbalis*** Mey, 2003b: 430 [type locality: Philippines, Luzon, Quezon province, east of Infanta, Magsaysay, small stream to the Agos River; ZMHB, to be transferred to either MPMP or UPLB; ♂]. —Mey and Freitag 2020: 57 [distribution].

**Distribution.** —Philippines.

***barisan*** Oláh & Johanson, 2010: 64 [type locality: Indonesia, Sumatra, Way Titias, Bukit Barisan Selatan NP, 950 m; Collection Oláh; ♂].

**Distribution.** —Indonesia.

***berduri*** Wells, 1990b: 367 [type locality: [Indonesia] Sulawesi Utara, Dumoga-Bone N.P., Tumpah R., 1 km above Toraut R. junction; NMV; ♂; ♀; larva; case; pupa]. —Malicky et al. 2011: 162 [distribution].

**Distribution.** —Indonesia.

***bintik*** Wells & Huisman, 1993: 94 [type locality: East Malaysia, Sabah, Long Pa Sia, confluence Sg. Pa Sia - St. Matang, 04°24'N 115°43'E, 1000 m; RMNH; ♂]. —Malicky 2010a: 67 [atlas; ♂].

**Distribution.** —Malaysia.

***choliona*** Oláh, 1989: 264 [type locality: Vietnam, Son La Province, Moc Chau; HNHM; ♂]. —Armitage et al. 2005: 28 [checklist]. —Malicky 2010a: 68 [atlas; ♂].

**Distribution.** —Vietnam.

***coodei*** Wells & Huisman, 1993: [type locality: Brunei, Sg. Temburong, 140 m; RMNH; ♂]. —Malicky 2010a: 67 [atlas; ♂].

**Distribution.** —Brunei.

***distorta*** Wells & Mey, 2002: 123 [type locality: [Philippines] Panay, Culasi, San Vicente, 400 m; ZMHB; ♂].

**Distribution.** —Philippines.

***dotalugola*** Schmid, 1958b: 57 [type locality: [Sri Lanka], Ceylan, Kitulgala (Sab., 750 ft) 2-III, Kelani Ganga, belle rivière coulant dans une vallée étroite et boisée, à la sortie des montagnes; depository not designated; ♂].

**Distribution.** —Sri Lanka.

***echna*** Oláh & Johanson, 2010: 66 [type locality: Vietnam, Lamdong Province, Baoloc, River Da Nga; Collection Oláh; ♂].

**Distribution.** —Vietnam.

***elongata*** Wells & Malicky, 1997: 181 [type locality: [Indonesia] Sumatra, Huta Padang; Collection Malicky; ♂]. —Malicky 2007a: 177 [checklist]. —Malicky 2010a: 65 [atlas; ♂].

**Distribution.** —Indonesia.

***gajah*** Wells & Huisman, 1993: 96 [type locality: East Malaysia, Sabah, Tenom, Tenom Agricultural Research Station; NTM; ♂]. —Malicky 2010a: 65 [atlas; ♂].

**Distribution.** —Malaysia.

***ganjil*** Wells & Huisman, 1993: 97 [type locality: West Malaysia, Genting Highlands, Gombak, tributary of Sg. Gombak above University of Malaya field station; NTM; ♂; case]. —Malicky and Chantaramongkol 2007: 1049 [♂; distribution]. —Malicky 2010a: 70 [atlas; ♂]. —Melnitsky et al. 2019: 539 [distribution].

**Distribution.** —Malaysia, Thailand.

***hacha*** Oláh & Johanson, 2010: 67 [type locality: Vietnam, Lamdong Province, Baoloc, River Da Nga; Collection Oláh; ♂].

**Distribution.** —Vietnam.

***hailana*** Oláh & Johanson, 2010: 69 [type locality: Vietnam, Lamdong Province, Baoloc, Loch Chau stream; Collection Oláh; ♂].

**Distribution.** —Vietnam.

***hapitigola*** Schmid, 1958b: 57 [type locality: [Sri Lanka], Ceylan, Carney (Sab., 900 ft) 1-II, Balawane Oya, rivière de taille moyenne, torrentueuse, coulant sur de gros rochers moussus, à la sortie des montagnes; depository not designated; ♂].

**Distribution.** —Sri Lanka.

***hatnagola*** Schmid, 1958b: 56 [type locality: [Sri Lanka], Ceylan, Konakalagala (C. P., 1700 ft) 17-I, Ping Oya, même aspect qu’à Ambatenna; depository not designated; ♂].

**Distribution.** —Sri Lanka.

***hermani*** Wells & Huisman, 1993: 98 [type locality: East Malaysia, Sabah, Long Pa Sia, confluence Sg. Ritan, Sg. Rurun, 1040 m; RMNH; ♂]. —Malicky 2010a: 65 [atlas; ♂].

**Distribution.** —Malaysia.

***horgos*** Oláh, 2013: 75 [type locality: Indonesia, Papua, Raja Ampat, Batanta Island, northern coast, Warmon stream, below first waterfall, 0.83570°S, 130.71400°E; Collection Oláh; ♂].

**Distribution.** —Indonesia.

***hutapadangensis*** Wells & Malicky, 1997: 180 [type locality: [Indonesia] N Sumatra, Huta Padang; Collection Malicky; ♂]. —Malicky and Chantaramongkol 2007: 1051 [distribution]. —Malicky 2007a: 177 [checklist]. —Malicky 2010a: 65 [atlas; ♂]. —Oláh and Johanson 2010: 69 [distribution].

**Distribution.** —Indonesia, Laos, Thailand.

***iomara*** Wells, 1990a: 838 [type locality: Papua New Guinea, Central Province, Iomari Creek on Port Moresby-Bereina road; ANIC; ♂; ♀; case]. —Wells 1991: 526 [checklist].

**Distribution.** —Papua New Guinea.

***laoana*** Oláh & Johanson, 2010: 70 [type locality: Laos, Vientiane Province, Phamom stream, 125 m upstream Phahom Village, 363 m; NHRS; ♂].

**Distribution.** —Laos.

***likliklang*** Wells, 1990a: 839 [type locality: Papua New Guinea, Central Province, creek in Kanosia Rubber Plantation on Port Moresby-Bereina road; ANIC; ♂; ♀]. —Wells 1991: 526 [checklist].

**Distribution.** —Papua New Guinea.

***limacabanga*** Wells, 1990b: 371 [type locality: [Indonesia] Sulawesi Utara, Dumoga Ketjil, rice paddy; NMV; ♂; ♀]. —Malicky et al. 2011: 162 [distribution].

**Distribution.** —Indonesia.

***lironga*** Oláh & Johanson, 2010: 72 [type locality: Laos, Luang Namtha Province, Tong Om Village, 552 m; NHRS; ♂].

**Distribution.** —Laos.

***maratya*** Wells & Malicky, 1997: 180 [type locality: [Indonesia] N Sumatra, Bukit Maratya, Sungai Bahapal, 03°00'N 99°14'E, 200 m asl; Collection Malicky; ♂]. —Malicky 2007a: 177 [checklist]. —Malicky 2010a: 66 [atlas; ♂].

**Distribution.** —Indonesia.

***matakail*** Wells & Huisman, 1993: 99 [type locality: West Malaysia, Kepong, Forest Research Institute of Malaya; NTM; ♂]. —Malicky 2010a: 68 [atlas; ♂].

**Distribution.** —Malaysia.

***menara*** Wells & Huisman, 1993: 97 [type locality: East Malaysia, Sarawak, Lambir National Park; NTM; ♂]. —Malicky 2010a: 66 [atlas; ♂].

**Distribution.** —Malaysia.

***minutula*** Mey & Freitag, 2019: 211 [type locality: [Philippines], Palawan, Puerto Princesa, Brgy. Cabayugan, spring creek of Cabayugan River, SSW of Martarpi, 10°09'46"N, 118°49'29"E, 80 m a.s.l.; MPMP; ♂; ♀]. —Mey and Freitag 2020: 57 [distribution].

**Distribution.** —Philippines.

***monga*** Oláh, 1989: 265. [type locality: Vietnam, Cucphuong, 400 m a.s.l.; HNHM; ♂]. —Wells & Huisman, 1993: 95 [♂]. —Malicky and Chantaramongkol 2007: 1051 [distribution]. —Armitage et al. 2005: 28 [checklist]. —Malicky 2010a: 68 [atlas; ♂].

**Distribution.** —Malaysia, Thailand, Vietnam.

***pallu*** Malicky & Prommi in Malicky 2009a: 16 [type locality: Thailand, Prov. Songkla, Hat Yai, 7°00'N, 100°30'E; MBBJ; ♂]. —Prommi and Permkam 2010: 295 [distribution]. —Malicky 2010a: 66 [atlas; ♂].

**Distribution.** —Thailand.

***panayana*** Wells & Mey, 2002: 123 [type locality: [Philippines] Panay, San Reminigio, Aningalan; ZMHB; ♂].

**Distribution.** —Philippines.

***paruparu*** Wells & Huisman, 1993: 96 [type locality: East Malaysia, Sarawak, Lambir National Park; NTM; ♂]. —Malicky 2010a: 68 [atlas; ♂].

**Distribution.** —Malaysia.

***phaiaka*** Malicky & Chantaramongkol, 2007: 1051 [type locality: Thailand, Tung Yaw, 19°08'N 98°39'E, 1200 m; Collection Malicky; ♂]. —Malicky 2010a: 67 [atlas; ♂].

**Distribution.** —Thailand.

***piring*** Wells, 1993: 355 [type locality: [Indonesia], Bali, Bali Barat, Sg. Bandangung, N of Medewi; NTM; ♂]. —Malicky 2010a: 66 [atlas; ♂]. —Malicky et al. 2014a: 6 [distribution].

**Distribution.** —Indonesia.

***pisau*** Wells & Huisman, 1993: 98 [type locality: West Malaysia, Cameron Highlands, “40 mile” betweenn Tanah Rata and Tapah, below falls; NTM; ♂]. —Malicky 2010a: 68 [atlas; ♂].

**Distribution.** —Malaysia.

***poecilostola*** Mey, 1998a: 549 [type locality: Locality 1, northern slope of Mt. Agtuuganon range, 1050 m; ZMHB or institute in the Philippines; ♂]. —Malicky et al. 2011: 162 [distribution].

**Distribution.** —Indonesia, Philippines.

***pornsawan*** Chantaramongkol & Malicky, 1986: 518 [type locality: [Sri Lanka], Sabaragamuwa Province, Kitulgala, 21 mi N von Ratnapura, 60–150 m; MZLA; ♂].

**Distribution.** —Sri Lanka.

***pulmonaria*** (Xue & Yang, 1990): 124 [type locality: [China] Bawangling (320 m), Hainan; NAUJ; ♂; in *Stactobiella*]. —Yang et al. 1997b: 93 [checklist]. —Yang et al. 2005: 458 [checklist; in *Stactobiella*]. —Malicky and Chantaramongkol 2007: 1051 [♂; distribution, to *Chrysotrichia*]. —Oláh and Johanson 2010a: 73 [distribution]. —Malicky 2010a: 68 [atlas; ♂]. —Yang et al. 2016: 475 [checklist]. —Malicky et al. 2018: 1321–1324 [distribution].

—*tanduk* Wells & Huisman, 1993: 97 [type locality: West Malaysia, Genting Highlands, Gombak, tributary Sg. Gombak above University of Malaya field station; NTM; ♂]. —Malicky and Chantaramongkol 2007: 1051 [to synonymy].

**Distribution.** —China, Laos, Malaysia, Thailand.

***quirinus*** Malicky & Chantaramongkol, 2007: 1049 [type locality: Thailand, Kao Kitchakut NP, 12°50'N 102°07'E, 100 m; Collection Malicky; ♂]. —Malicky 2010a: 70 [atlas; ♂]. —Malicky et al. 2018: 1323 [distribution].

**Distribution.** —Thailand.

***serrula*** Oláh & Johanson, 2010: 74 [type locality: Vietnam, Lamdong Province, Baoloc, loc. Chau stream; Collection Oláh; ♂].

**Distribution.** —Vietnam.

***simplex*** Wells & Mey, 2002: 123 [type locality: [Philippines] Palawan, Cayasan, Babuyan River, ZMHB; ♂].

**Distribution.** —Philippines.

***sinuosa*** Mey, 2003b: 431 [type locality: Philippines, Leyte, Baybay, Mt. Panasugan; ZMHB, to be transferred to either UPLB or MPMP; ♂].

**Distribution.** —Philippines.

***siriya*** Chantaramongkol & Malicky, 1986: 518 [type locality: [Sri Lanka], Sabaragamuwa Province, 5 mi NNW von Balangoda, 725 m; MZLA; ♂].

**Distribution.** —Sri Lanka.

***skamandros*** Malicky & Chantaramongkol, 2007: 1049 [type locality: Thailand, Doi Suthep NP, Montatan WF, 18°49'N 98°55'E; Collection Malicky; ♂]. —Malicky 2010a: 70 [atlas; ♂].

**Distribution.** —Thailand.

***sparta*** Malicky & Chantaramongkol, 2007: 1050 [type locality: Thailand, Chattrakan, 17°18'N 100°41'E; Collection Malicky; ♂]. —Malicky 2010a: 69 [atlas; ♂].

**Distribution.** —Thailand.

***sukamade*** Wells & Malicky, 1997: 179 [type locality: [Indonesia] East Java, Meru Betiri, stream in savannah; ANIC; ♂]. —Malicky 2007a: 177 [checklist]. —Malicky 2010a: 66 [atlas; ♂]. —Malicky et al. 2014a: 6 [distribution].

**Distribution.** —Indonesia.

***syrinx*** Malicky, 2008a: 839 [type locality: [Indonesia, Borneo, Kalimantan], im Einzugsbereich der Flüsse Seturan und Rian in einem engen Bereich von ungefähr 8 × 8 km ca. 70 km südlich der Stadt Malinau, 116°29'48"–116°33'29"E, 2°59'29"–3°04'04"N, 100–200 m; MZLS; ♂]. —Malicky 2010a: 67 [atlas; ♂].

**Distribution.** —Indonesia.

***tabonensis*** Mey, 1998a: 549 [type locality: [Philippines] Mindanao, Surigao del Sur, Mangagoy, waterfall of Tabon River; ZMHB or institute in the Philippines; ♂]. —Wells and Mey 2002: 134 [checklist].

**Distribution.** —Philippines.

***tajam*** Wells & Huisman, 1993: 95 [type locality: East Malaysia, Sabah, Tenom, stream behind Hotel Tenom; NTM; ♂]. —Malicky 2010a: 68 [atlas; ♂].

**Distribution.** —Malaysia.

***talthybios*** Malicky & Chantaramongkol, 2007: 1050 [type locality: Thailand, Pong Düat; Collection Malicky; ♂]. —Malicky 2010a: 69 [atlas; ♂].

**Distribution.** —Thailand.

***terpisaduri*** Wells, 1993: 355 [type locality: [Indonesia], Bali, Bali Barat, Sg. Bandangung, N of Medewi; NTM; ♂]. —Malicky 2010a: 69 [atlas; ♂]. —Malicky et al. 2014a: 6 [distribution].

**Distribution.** —Indonesia.

***thira*** Oláh & Johanson, 2010: 74 [type locality: Vietnam, Lamdong Province, Baoloc, Baco stream; Collection Oláh; ♂].

**Distribution.** —Vietnam.

***tigacabanga*** Wells, 1990b: 370 [type locality: [Indonesia] Sulawesi Utara, Dumoga-Bone N.P., Tumpah R. tributary above first fall; NMV; ♂]. —Malicky et al. 2011: 162 [distribution].

**Distribution.** —Indonesia.

***trifida*** Mey, 1998a: 547 [type locality: [Philippines] Locality 1, northern slope of Mt. Agtuuganon range, 1050 m; ZMHB or institute in the Philippines; ♂]. —Wells and Mey 2002: 134 [checklist].

**Distribution.** —Philippines.

***trisula*** Wells, 1993: 354 [type locality: [Indonesia], Bali, Bali Barat, Sg. Bandangung, N. of Medewi; NTM; ♂]. —Malicky 2010a: 65 [atlas; ♂]. —Malicky et al. 2014a: 6 [distribution].

**Distribution.** —Indonesia.

***tydeus*** Malicky & Chantaramongkol, 2007: 1050 [type locality: Thailand, Taleban, 6°43'N 100°10'E; Collection Malicky; ♂]. —Oláh and Johanson 2010a: 76 [distribution]. —Malicky 2010a: 69 [atlas; ♂].

**Distribution.** —Laos, Thailand.

***vagot*** Oláh, 2016: 123 [type locality: Indonesia, West Papua, Batanta Island, valley of Kalisamsem River, 00°53'27.54", 130°33'31.62"; Collection Oláh; ♂].

**Distribution.** —Indonesia.

***vaskos*** Oláh, 2016: 124 [type locality: Indonesia, West Papua, Batanta Island, valley of Kalisamsem River, 00°53'27.54", 130°33'31.62"; Collection Oláh; ♂]. —Oláh and Kovács 2018: 184 [distribution].

**Distribution.** —Indonesia.

***volcanus*** Malicky & Chantaramongkol, 2007: 1050 [type locality: Thailand, Prov. Lampang, Chaeson NP, 18°46'N 99°28'E, 500 m; Collection Malicky; ♂]. —Melnitsky and Malicky 2008: 25 [distribution]. —Malicky 2010a: 69 [atlas; ♂]. —Malicky et al. 2018: 1323 [distribution].

**Distribution.** —Thailand.

***watuwila*** Wells & Huisman, 2001: 207 [type locality: [Indonesia] Sulawesi Tenggara, N slope of Gunung Watuwila, 250 m, Sungai Mokowu; RMNH; ♂].

**Distribution.** —Indonesia.

***zoroastres*** Malicky & Chantaramongkol, 2007: 1049 [type locality: Thailand, Namtok Pasua, 19°24'N 97°56'E, 300 m; Collection Malicky; ♂]. —Malicky 2010a: 70 [atlas; ♂].

**Distribution.** —Thailand.

#### Genus *Flintiella* Angrisano, 1995

*Flintiella* Angrisano, 1995a: 502 [type species: *Flintiellaandreae* Angrisano, 1995a, original designation]. —Harris et al. 2002b: 65 [revision; key to males].

The genus *Flintiella* is represented by 17 species occurring in the Neotropical faunal region. Angrisano (1995a) established the genus based on the lack of ocelli and a tarsal formula (0, 2, 3) that is unique within the tribe to the Americas; she described the female, larva, and case of *F.andreae*. Members of *Flintiella* are similar in appearance to those of *Stactobiella*, differing mainly in genitalic features and the lack of ocelli (Flint et al. 1999a).

***alajuela*** Harris, Flint, & Holzenthal, 2002a: 66 [type locality: Costa Rica, Alajuela, Rio Pizote, ca. 5 km N Dos Rios, 10.948°N, 85.291°W, 40 m; NMNH; ♂].

**Distribution.** —Costa Rica.

***andreae*** Angrisano, 1995a: 503 [type locality: Uruguay, Artigas, Ao. de la Invernada; FHCU; ♂; ♀; larva; case]. —Angrisano 1999: 32 [checklist]. —Harris et al. 2002b: 75 [♂; ♀; re-description]. —Angrisano and Sganga 2007: 28 [♂; ♀; larva; pupa; distribution]. —de Souza et al. 2013: 585 [distribution]. —Paprocki and França 2014: 44 [checklist].

**Distribution.** —Argentina, Brazil, Uruguay.

***astilla*** Harris, Flint, & Holzenthal, 2002a: 69 [type locality: Venezuela, Amazonas, Rio Cataniapo, 10 km S Puerto Ayacucho; NMNH; ♂; ♀]. —Calor 2011: 321 [checklist]. —Nogueira and Cabette 2011: 351 [distribution]. —Paprocki and França 2014: 44 [checklist]. —Ríos-Touma et al. 2017: 9 [checklist].

**Distribution.** —Brazil, Costa Rica, Ecuador, Paraguay, Peru, Venezuela.

***boraceia*** Harris, Flint, & Holzenthal, 2002a: 69 [type locality: Brazil, São Paulo, Estaction Biologica Boracéia; MZUSP; ♂]. —Paprocki et al. 2004: 11 [checklist]. —Calor 2011: 321 [checklist]. —Paprocki and França 2014: 44 [checklist].

**Distribution.** —Brazil.

***carajas*** Santos, Jardim, & Nessimian, 2011: 803 [type locality: Brazil, Pará, Parauapebas (Floresta Nacional de Carajás, small stream, 06°04'57"S 50°08'05"W, 642 m; DZRJ; ♂; ♀]. —Paprocki and França 2014: 44 [checklist].

**Distribution.** —Brazil.

***harma*** Oláh & Johanson, 2011: 248 [type locality: French Guiana, Approuaguekaw, Kaw Mt, 4°32.833'N, 52°11.452'W, 77 m; NHRS; ♂].

**Distribution.** —French Guiana.

***harrisi*** de Souza, Santos, & Takiya, 2016b: 341 [type locality: Brazil, Piauí, Piracuruca, Parque Nacional de Sete Cidades, Riacho Piedade, 04°06'34"S 41°4'39"W, 169 m; CZMA; ♂]. —Moreno et al. 2020: 265 [distribution].

**Distribution.** —Brazil.

***heredia*** Harris, Flint, & Holzenthal, 2002a: 77 [type locality: Costa Rica, Heredia, Rio Bijagual on road to Magsasay, 10.408°N, 84.076°W, 140 m; NMNH; ♂; ♀]. —Armitage et al. 2016: 7 [distribution]. —Ríos-Touma et al. 2017: 9 [checklist]. —Armitage and Harris 2018b: 97 [checklist]. —Harris and Armitage 2019: 4 [distribution].

**Distribution.** —Costa Rica, Ecuador, Panama, Peru.

***leloga*** Oláh & Johanson, 2011: 250 [type locality: French Guiana, Approuaguekaw, Kaw Mt, 4°33.035'N, 52°11.661'W, 104 m; NHRS; ♂].

**Distribution.** —French Guiana.

***manauara*** Santos & Nessimian, 2009b: 65 [type locality: Brazil, Amazonas, Manaus, tributary to Rio Branquinho, 02°31'24.6"S 60°20'05.3"W; INPA; ♂; ♀]. —Paprocki and França 2014: 45 [checklist].

**Distribution.** —Brazil.

***pallida*** de Souza, Santos, & Takiya, 2016b: 341 [type locality: Brazil, Maranhão, Carolina, Parque Nacional da Chapada das Mesas, Riacho Cancela, 07°06'43.4"S 47°17'16.6"W, 186 m; CZMA; ♂].

**Distribution.** —Brazil.

***panamensis*** Harris, Flint, & Holzenthal, 2002a: 79 [type locality: Panama, Panama, Barro Colorado Island, Snyder-Molino trail; NMNH; ♂]. —Armitage et al. 2015a: 6 [checklist]. —Armitage and Harris 2018b: 97 [checklist].

**Distribution.** —Panama.

***pizotensis*** Harris, Flint, & Holzenthal, 2002a: 73 [type locality: Costa Rica, Limon, Rio Telire and small tributaries SE Suretka, 9.554°N, 82.892°W, 48 m; NMNH; ♂; ♀]. —Dumas et al. 2010: 8 [distribution]. —Paprocki and França 2014: 45 [checklist]. —Armitage et al. 2015a: 6 [checklist]. —Ríos-Touma et al. 2017: 9 [checklist]. —Armitage and Harris 2018b: 97 [checklist].

**Distribution.** —Brazil, Colombia, Costa Rica, Ecuador, Mexico, Nicaragua, Panama, Peru.

***serrana*** Gama Neto, Ribeiro, & Passos, 2020: 285 [type locality: Brazil, Roraima, Amajari municipality, Serra do Tepequém, unnamed small-order stream, 03°48'17.06"N, 61°44'49.8"W, 600 m a.s.l.; MPEG; ♂].

**Distribution.** —Brazil.

***tamaulipasa*** Harris, Flint, & Holzenthal, 2002a: 79 [type locality: Mexico, Tamaulipas, Rio Frio at La Poza Azul, near Gomez Farias; NMNH; ♂; ♀]. —Ríos-Touma et al. 2017: 9 [distribution]. —Barba-Álvarez et al. 2019: 85 [distribution].

**Distribution.** —Ecuador, Mexico.

***triaena*** Gama Neto, Ribeiro, & Passos, 2020: 286 [type locality: Brazil, Pará, São Geraldo do Araguaia Municipality, Serra das Andorinhas, Santa Cruz stream, 06°13'31.1"S, 48°26'28.1"W, 124 m a.s.l. INPA; ♂].

**Distribution.** —Brazil.

***yanamona*** Harris, Flint, & Holzenthal, 2002a: 79 [type locality: Peru, Loreto, small stream near Explorama Lodge; NMNH; ♂].

**Distribution.** —Peru.

#### Genus *Maetalaiptila* Malicky & Chantaramongkol, 2007

*Maetalaiptila* Malicky & Chantaramongkol, 2007: 1055 [type species: *Maetalaiptilapyramus* Malicky & Chantaramongkol, 2007, original designation].

*Maetalaiptila* contains a single species occurring in Thailand. Malicky and Chantaramongkol (2007) established the genus based on features of the male genitalia and placed it in Stactobiinae because of the presence of the transverse suture on the mesoscutellum. The female and larva are unknown.

***pyramus*** Malicky & Chantaramongkol, 2007: 1055 [type locality: Thailand, Mae Talai (S Chiang Dao), 19°16'N 98°57'E, 400 m; Collection Malicky; ♂]. —Malicky 2010a: 56 [atlas; ♂].

**Distribution.** —Thailand.

#### Genus *Niuginitrichia* Wells, 1990

*Niuginitrichia* Wells, 1990a: 820 [type species: *Niuginitrichiabukamak* Wells, 1990a, original designation]. —Wells 1990a: 822 [key to males]. —Wells and Huisman 2001: 208 [new records].

The genus *Niuginitrichia* consists of 24 species occurring in Indonesia and New Guinea. Wells (1990a) noted that there are many similarities between Niuginitrichia and Plethus, but that the former can be clearly separated by the absence of ocelli and differences in the male genitalia. Female, larva, pupa, and case were described by Wells (1990a).

***arakain*** Wells, 1990a: 825 [type locality: Papua New Guinea, soak at Kapao on Bulolo-Aseki road, 7°15'S 146°20'E; ANIC; ♂]. —Well 1991: 526 [checklist].

**Distribution.** —Papua New Guinea.

***bogos*** Oláh, 2016: 126 [type locality: Indonesia, West Papua, Batanta Island, valley of Warai stream, 00°50'51.0", 130°35'14.0"; Collection Oláh; ♂].

**Distribution.** —Indonesia.

***bomberi*** Wells, 1990a: 833 [type locality: [Western] New Guinea, Irian Jaya, Fak Fak, Bomberi, 2°55'S 132°17'E; BPBM; ♂]. —Wells 1991: 526 [checklist; as *bombieri*].

**Distribution.** —Indonesia.

***brukimnamel*** Wells, 1990a: 828 [type locality: Papua New Guinea, Central Province, Aieme River, 9°25'S 147°35'E; ANIC; ♂]. —Wells 1991: 526 [checklist].

**Distribution.** —Papua New Guinea.

***bukamak*** Wells, 1990a: 823 [type locality: Papua New Guinea, Central Province, Laloki River, Rouna Falls, 9°25'S 147°27'E; ANIC; ♂; ♀; larva; pupa; case]. —Wells 1991: 526 [checklist].

**Distribution.** —Papua New Guinea.

***eiloga*** Wells, 1990a: 825 [type locality: Papua New Guinea, Central Province, Eilogo Creek, nr Sogeri, 9°27'S 147°27'E; ANIC; ♂]. —Wells 1991: 526 [checklist].

**Distribution.** —Papua New Guinea.

***haromsog*** Oláh, 2016: 126 [type locality: Indonesia, West Papua, Batanta Island, valley of Kaliselatan River, 00°53'42.0", 130°35'49.1"; Collection Oláh; ♂].

**Distribution.** —Indonesia.

***harmas*** Oláh in Oláh and Kovács 2018: 184 [type locality: Indonesia, West Papua, Batanta Island, valley of Waibin river, S00°50'01.9", E130°45'24.8"; Collection Oláh; ♂].

**Distribution.** —Indonesia.

***homora*** Oláh in Oláh and Kovács 2018: 185 [type locality: Indonesia, West Papua, Batanta Island, valley of Tanjung Lampu, 00°53'43.0"S, 130°36'38.5"E; Collection Oláh; ♂].

**Distribution.** —Indonesia.

***huzva*** Oláh, 2013: 76 [type locality: Indonesia, Papua, Raja Ampat, Batanta Island, northern coast, Warder River, S .084374°, E 130.52457°, shipable endpoint; Collection Oláh; ♂]. —Oláh and Kovács 2018: 186 [distribution].

**Distribution.** —Indonesia.

***ismayi*** Wells, 1990a: 823 [type locality: Papua New Guinea, Central Province, Tapini, 8°16'S 146°55'E; ANIC; ♂]. —Wells 1991: 526 [checklist].

**Distribution.** —Papua New Guinea.

***ives*** Oláh, 2016: 127 [type locality: Indonesia, West Papua, Batanta Island, valley of Kalisamsem River, 00°53'27.54", 130°33'31.62"; Collection Oláh; ♂].

**Distribution.** —Indonesia.

***kesken*** Oláh, 2016: 128 [type locality: Indonesia, West Papua, Batanta Island, valley of Kaliselatan River, 00°53'42.0", 130°35'49.1"; Collection Oláh; ♂]. —Oláh and Kovács 2018: 186 [distribution].

**Distribution.** —Indonesia.

***kover*** Oláh, 2016: 129 [type locality: Indonesia, West Papua, Batanta Island, valley of Kalisamsem River, 00°53'27.54", 130°33'31.62"; Collection Oláh; ♂].

**Distribution.** —Indonesia.

***kurukut*** Wells, 1990a: 828 [type locality: Papua New Guinea, Central Province, Laloki River, Rouna Falls, on soak, 9°25'S 147°27'E; ANIC; ♂]. —Wells 1991: 526 [checklist].

**Distribution.** —Papua New Guinea.

***namelbanis*** Wells, 1990a: 828 [type locality: Papua New Guinea, East Highlands Province, Ukarumpa, Ram Creek, 6°17'S 145°50'E; ANIC; ♂]. —Wells 1991: 526 [checklist].

**Distribution.** —Papua New Guinea.

***negsog*** Oláh, 2016: 130 [type locality: Indonesia, West Papua, Batanta Island, valley of Kalijakut River, 0°52'52.0", 130°38'8.0"; Collection Oláh; ♂].

**Distribution.** —Indonesia.

***peregai*** Wells, 1990a: 830 [type locality: Papua New Guinea, East Highlands Province, Peregai, 6°09'S 144°11'E; ANIC; ♂]. —Wells 1991: 526 [checklist].

**Distribution.** —Papua New Guinea.

***rouna*** Wells, 1990a: 832 [type locality: Papua New Guinea, Central Province, Laloki River, Rouna Falls, on soak, 9°25'S 147°27'E; ANIC; ♂; ♀]. —Wells 1991: 526 [checklist].

**Distribution.** —Papua New Guinea.

***sapimarere*** Wells, 1990a: 830 [type locality: Papua New Guinea, Central Province, Aieme River, 9·25'S 147°35'E; ANIC; ♂]. —Wells 1991: 526 [checklist].

**Distribution.** —Papua New Guinea.

***sulawesica*** Wells & Huisman, 2001: 208 [type locality: Sulawesi Tenggara, N slope of Gunung Watuwila, 250 m, Sungai Mokowu; RMNH; ♂].

**Distribution.** —Indonesia.

***ukarumpa*** Wells, 1990a: 833 [type locality: [Papua New Guinea], East Highlands Province, Ukarumpa, Ram Creek, 6°16'S 145°50'E; ANIC; ♂]. —Wells 1991: 526 [checklist].

**Distribution.** —Papua New Guinea.

***umboina*** Wells, 1990a: 832 [type locality: Papua New Guinea, Umboi Island, ca. 8 km WNW. Lab Lab; BPBM; ♂; case]. —Wells 1991: 526 [checklist].

**Distribution.** —Papua New Guinea.

***vagva*** Oláh in Oláh and Kovács 2018: 186 [type locality: Indonesia, West Papua, Batanta Island, valley of Kalijakut River, 00°52'49.1"S, 130°38'04.9"E; Collection Oláh; ♂].

**Distribution.** —Indonesia.

#### Genus *Pseudoxyethira* Schmid, 1958

*Scelotrichia* Ulmer, 1951: 73 [type species: *Scelotrichiasaranganica* Ulmer, 1951, original designation]. —Marshall 1979b: 174 [generic review]. —Wells 1990a: 840 [key to New Guinea species]. —Koçak and Kemal 2012: 4 [preoccupied in Hemiptera by Reuter 1890: 291, replaced with *Orientalitrichia*].

*Pseudoxyethira* Schmid, 1958b: 44 [type species: *Pseudoxyethiraasgiriskanda* Schmid, 1958b, original designation]. —Marshall 1979b: 174 [generic review]. —Wells 1990b: 373 [to synonymy with *Scelotrichia*]. —Zhou et al. 2016: 214 [re-established as valid name]. —Ito 2017b: 194 [revision of Japanese species].

*Madioxyethira* Schmid, 1960: 89 [type species: *Madioxyethiramilinda* Schmid, 1960, original designation]. —Marshall 1979b: 173 [generic review]. —Wells 1990b: 373 [to synonymy with *Scelotrichia*].

*Orientalitrichia* Koçak & Kemal, 2012: 4 [type species: *Scelotrichiasaranganica* Ulmer, 1951, replacement name]. —Zhou et al. 2016: 214 [inappropriate replacement name].

The genus *Pseudoxyethira* is represented by 64 species occurring mainly in Southeast Asia. A single species, *P.glandulosa*, has been recorded from Tanzania (Wells and Andersen 1995). Marshall (1979b) stated that the genus belongs in Stactobiinae due to the postoccipital lobes of the adult head, which are very similar to those of *Madioxyethira*, and the presence of the transverse suture on the adult thorax. Wells described the general form of the larvae, pupa, and case of the genus (1990a).

***akaiah*** (Malicky, 2012): 1267 [type locality: China, Setschuan, Qingyin Pavilion, Jingshui, Emei Shan, 180 km SW Chengdu, 800–1200 m; Collection Malicky; ♂; in *Scelotrichia*]. —Yang et al. 2016: 477 [checklist].

**Distribution.** —China.

***alata*** (Wells & Mey, 2002): 116 [type locality: [Philippines] Palawan, Puerto Princesa, Cayasan, Balsahan; ZMHB; ♂; in *Scelotrichia*].

**Distribution.** —Philippines.

***asgiriskanda*** Schmid, 1958b: 45 [type locality: [Sri Lanka], Ceylan, Diyanilla (C. P., 4800 ft) 1-III, petit torrent pierreux, dans les plantations de thé; depository not designated; ♂].

**Distribution.** —Sri Lanka.

***batanta*** (Oláh, 2016): 131 [type locality: Indonesia, West Papua, Batanta Island, valley of Kalisamsem River, 00°53'27.54", 130°33'31.62"; Collection Oláh; ♂; as *Scelotrichia*].

**Distribution.** —Indonesia.

***bercabanghalus*** (Wells & Malicky, 1997): 177 [type locality: [Indonesia] N Sumatra, Huta Padang, 02°45'N 99°14'E; Collection Malicky; ♂; in *Scelotrichia*]. —Malicky 2007a: 177 [checklist]. —Malicky 2010a: 64 [atlas; ♂].

**Distribution.** —Indonesia.

***bilah*** (Wells & Huisman, 1993): 106 [type locality: East Malaysia, Sabah, Long Pa Sia area, Sg. Ritan, 04°24'N 115°42'E, 1160 m; RMNH; ♂; in *Scelotrichia*]. —Malicky 2010a: 63 [atlas; ♂].

**Distribution.** —Malaysia.

***bispinosa*** (Wells & Mey, 2002): 116 [type locality: [Philippines] Panay, San Reminigio, Aningalan; BPBM; ♂; in *Scelotrichia*].

**Distribution.** —Philippines.

***buluhalus*** (Wells & Huisman, 1993): 107 [type locality: East Malaysia, Sabah, 60 km W Lahad Datu, DVFC [=Danum Valley Field Centre] nr bridge, 04°58'N 117°48'E, 150 m; RMNH; ♂; in *Scelotrichia*]. —Wells and Malicky 1997: 176 [distribution]. —Malicky 2007a: 177 [checklist]. —Malicky 2010a: 60 [atlas; ♂].

**Distribution.** —Indonesia, Malaysia.

***cavernosa*** (Mey, 1996): 50 [type locality: Nord Vietnam, nordwestlich von Sa Pa, Fan Si Pan Gebirgsmassiv, Westseite, 1400–1600 m; ZMHB; ♂; in *Scelotrichia*]. —Armitage et al. 2005: 28 [checklist]. —Mey 2005a: 281 [distribution]. —Malicky 2010a: 64 [atlas; ♂].

**Distribution.** —Vietnam.

***cayasana*** (Wells & Mey, 2002): 118 [type locality: [Philippines] Palawan, Cayasan, Babuyan River; ZMHB; ♂; in *Scelotrichia*].

**Distribution.** —Philippines.

***ceesi*** (Wells & Huisman, 1993): 108 [type locality: East Malaysia, Sabah, 8.5 km S Long Pa Sia, Sg. Malabit, 04°21'N 115° 41'E, 1180 m; RMNH; ♂; in *Scelotrichia*]. —Malicky 2010a: 61 [atlas; ♂].

**Distribution.** —Malaysia.

***dasar*** (Wells & Huisman, 1993): 107 [type locality: West Malaysia, Templer’s Park; NTM; ♂; in *Scelotrichia*]. —Malicky 2010a: 61 [atlas; ♂].

**Distribution.** —Malaysia.

***digitata*** (Wells & Mey, 2002): 114 [type locality: [Philippines] Mindanao, Bukidnon, 1480 m, Mt Katanglad; ZMHB; ♂; in *Scelotrichia*].

**Distribution.** —Philippines.

***dolichocera*** (Mey, 1998a): 551 [type locality: [Philippines, Mindanao], northern slope of Mt. Atuuganon range, 1050 m; ZMHB; ♂; in *Scelotrichia*]. —Wells and Mey 2002: 134 [checklist].

**Distribution.** —Philippines.

***egba*** Oláh in Oláh and Kovács 2018: 187 [type locality: Indonesia, West Papua, Batanta Island, valley of Warai stream, 00°50'59.3"S, 130°35'18.0"E; Collection Oláh; ♂].

**Distribution.** —Indonesia.

***funatsuki*** Ito, 2017b: 197 [type locality: [Japan], Ryukyu Islands, Iriomote-jima, Nishi-funatsuki-gawa, Nishi0funatsuki-bashi, 24°18'10"N, 123°51'34"E, 10 m; CBM-ZI; ♂].

**Distribution.** —Japan.

***gerigi*** (Wells & Huisman, 1993): 109 [type locality: East Malaysia, Sabah, 8.5 km S Long Pa Sia, Sg. Malabit, 04°21'N 115°41'E, 1180 m; RMNH; ♂; in *Scelotrichia*]. —Malicky 2010a: 61 [atlas; ♂].

**Distribution.** —Malaysia.

***glandulosa*** (Wells & Andersen, 1995): 148 [type locality: Tanzania, Tanga region, West Usambara Mts, Mazumbai, Kaputu Stream, loc. 5, 1650 m a.s.l; ZMUB; ♂; in *Scelotrichia*].

**Distribution.** —Tanzania.

***insularis*** (Mey, 1995): 193 [type locality: [Philippines], Mindoro, Paluan, Calawagan-Fluß; Collection Mey; ♂; in *Scelotrichia*]. —Wells and Mey 2002: 116 [♂; distribution].

**Distribution.** —Philippines.

***ishiharai*** (Utsunomiya, 1994): 345 [type locality: [Japan], Ôto, Cape Ashizuri-misaki, Kôchi Pref.; depository not designated; ♂; ♀; larva; in *Scelotrichia*]. —Ohkawa and Ito 2002: 450 [♂; ♀; larva; distribution]. —Satake and Kuranishi 2007: 282 [distribution]. —Tanida and Kuranishi 2016: 73 [checklist]. —Ito 2017b: 195 [♂; distribution].

**Distribution.** —Japan.

***jari*** (Wells & Huisman, 1993): 109 [type locality: West Malaysia, Genting Highlands, tributary Sg. Gombak; NTM; ♂; in *Scelotrichia*]. —Wells and Mey 2002: 118 [distribution]. —Oláh and Johanson 2010a: 79 [distribution]. —Malicky 2010a: 61 [atlas; ♂]. —Malicky et al. 2014a: 6 [distribution]. —Malicky et al. 2016: 92 [distribution].

**Distribution.** —Borneo, Indonesia, Malaysia, Philippines.

***kait*** (Wells & Huisman, 1993): 105 [type locality: East Malaysia, Sabah, Kundassang, Mesilau East River; NTM; ♂; in *Scelotrichia*]. —Malicky 2010a: 64 [atlas; ♂].

**Distribution.** —Malaysia.

***kakatu*** (Wells, 1990a): 840 [type locality: Papua New Guinea, West Highlands Province, Peregai, 6°09'S 144°11'E; ANIC; ♂; ♀; larva; pupa; case; in *Scelotrichia*]. —Wells 1991: 526 [checklist].

**Distribution.** —Papua New Guinea.

***kenyella*** (Mey, 1992): 260 [type locality: [Kenya], Meru-Nationalpark; ZMHB; ♂; in *Madioxyethira*]. —Wells and Andersen 1995: 145 [checklist].

**Distribution.** —Kenya.

***kipas*** (Wells & Huisman, 1993): 108 [type locality: East Malaysia, Sabah, Tenom; NTM; ♂; in *Scelotrichia*]. —Malicky 2010a: 60 [atlas; ♂]. —Malicky 2013: 43 [possible junior synonym of *P.thingana*].

**Distribution.** —Malaysia.

***kurta*** (Oláh, 2016): 132 [type locality: Indonesia, West Papua, Batanta Island, valley of Warai stream, 00°50'51.0", 130°35'14.0"; Collection Oláh; ♂; as *Scelotrichia*]. —Oláh and Kovács 2018: 188 [distribution].

**Distribution.** —Indonesia.

***ladik*** (Oláh & Johanson, 2010a): 79 [type locality: India, Tamil Nadu, Doddabetta, Nilgiri Hills, 1000 m; HNHM; ♂; in *Scelotrichia*].

**Distribution.** —India.

***laitimtok*** (Wells, 1990a): 845 [type locality: Papua New Guinea, Central Province, Rouna Falls, 9·25'S 147°27'E; ANIC; ♂; ♀; in *Scelotrichia*]. —Wells 1991: 526 [checklist].

**Distribution.** —Papua New Guinea.

***lampai*** (Wells & Huisman, 1993): 108 [type locality: West Malaysia, Selangor district, Templer’s Park, 20 km NW Kuala Lumpur; NTM; ♂; in *Scelotrichia*]. —Wells and Mey 2002: 118 [distribution]. —Malicky 2010a: 61 [atlas; ♂].

—*hexalocha* (Mey, 1998a): 551 [type locality: [Philippines, Mindanao], northern slope of the Mt. Atuuganon range, 1050 m; ZMHB; ♂; in *Scelotrichia*]. —Wells and Mey 2002: 118 [to synonymy].

**Distribution.** —Malaysia, Philippines.

***levis*** (Wells & Dudgeon, 1990): 163 [type locality: Hong Kong, Tai Po Kao Forest stream; NHMUK; ♂; in *Scelotrichia*]. —Yang et al. 2016: 477 [checklist].

**Distribution.** —Hong Kong.

***licini*** (Wells, 1990b): 376 [type locality: [Indonesia] Sulawesi Utara, Dumoga-Bone N.P., Tumpah R. tributary first fall; NMV; ♂; ♀; larva; case; in *Scelotrichia*]. —Malicky et al. 2010: 163 [distribution].

**Distribution.** —Indonesia.

***litai*** (Malicky & Chantaramongkol, 2007): 1052 [type locality: Bhutan, Tsirang, Rongchhu, 26°59'N 90°09'E, 1700 m; ♂; in *Scelotrichia*]. —Malicky 2010a: 63 [atlas; ♂].

**Distribution.** —Bhutan.

***mador*** (Malicky, 2012): 1267 [type locality: China, Jiangxi, Hinggang Shan, Xiangzhou vill., 374 m, 26°35'N, 114°16'E; Collection Malicky; ♂; in *Scelotrichia*]. —Yang et al. 2016: 477 [checklist].

**Distribution.** —China.

***malayana*** (Oláh & Johanson, 2010a): 80 [type locality: Malaysia, Perak, Temengor Lake; NHMUK; ♂; in *Scelotrichia*].

**Distribution.** —Malaysia.

***marshalli*** (Statzner, 1977): 399 [type locality: Zaire, Kivu Region, Kalengo stream 10 km west of Lake Kivu; ZMHB; ♂; ♀; in *Madioxyethira*].

**Distribution.** —Congo.

***melanella*** (Mey, 1998a): 551 [type locality: [Philippines, Mindanao], nr Caatjaan, valley of the Simulau river; ZMHB; ♂; in *Scelotrichia*]. —Wells and Mey 2002: 134 [checklist].

**Distribution.** —Philippines.

***melanoptera*** (Mey, 1998a): 547 [type locality: [Philippines, Mindanao], nr Caatjaan, valley of the Simulau river; ZMHB; ♂; in *Scelotrichia*]. —Wells and Mey 2002: 134 [checklist].

**Distribution.** —Philippines.

***milinda*** (Schmid, 1960): 90 [type locality: [Pakistan, Karakoram, Shinghai Gah; CNC; ♂; in *Madioxyethira*]. —Schmid 1958c: 220 [as new species, *nomen nudum*]. —Malicky and Chantaramongkol 2007: 1052 [distribution]. —Malicky 2013: 43 [possible senior synonym of *P.nepalensis*]. —Mattern 2015: 502 [distribution]. —Malicky 2018: 49 [checklist]. —Lonsdale 2020: 37 [holotype depository].

**Distribution.** —Nepal, Pakistan.

***milka*** (Malicky, Ivanov, & Melnitsky, 2011): 1494 [type locality: [Indonesia], Lombok, Kembangkuning, 525 m; ZIN; ♂; in *Scelotrichia*]. —Malicky et al. 2014a: 6 [distribution]. —Malicky et al. 2016: 92 [distribution].

**Distribution.** —Indonesia.

***mindanaoensis*** (Wells & Mey, 2002): 116 [type locality: [Philippines] Mindanao, Bukidnon, 1480 m, Mt Katanglad; BPBM; ♂; in *Scelotrichia*].

**Distribution.** —Philippines.

***miselia*** (Mey, 1998a): 551 [type locality: [Philippines, Mindanao], northern slope of Mt. Atuuganon range, 1050 m; ZMHB; ♂; in *Scelotrichia*]. —Wells and Mey 2002: 134 [checklist].

**Distribution.** —Philippines.

***nana*** (Mey, 1996): 50 [type locality: Nord Vietnam, nordwestlich von Sa Pa, Fan Si Pan Gebirgsmassiv, Westseite, 1400–1600 m; ZMHB; ♂; in *Scelotrichia*]. —Armitage et al. 2005: 28 [checklist]. —Mey 2005a: 281 [distribution]. —Malicky 2010a: 60 [atlas; ♂]. —Malicky 2013: 43 [possible junior synonym of *P.thingana*].

**Distribution.** —Vietnam.

***nepalensis*** (Kimmins, 1964): 46 [type locality: [Nepal], Taplejung Distr., Sangu, c. 6,200 ft., mixed vegetation by stream in gully; NHMUK; ♂; in *Madioxyethira*]. —Malicky 2006: 253 [checklist]. —Malicky 2013: 43 [possible junior synonym of *P.milinda*]. —Mattern 2015: 502 [distribution].

**Distribution.** —Nepal.

***nikolayi*** (Malicky, Ivanov, & Melnitsky, 2011): 1493 [type locality: [Indonesia], Lombok, Senaru, 440–590 m; ZIN; ♂; in *Scelotrichia*]. —Malicky et al. 2014a: 6 [distribution]. —Malicky et al. 2016: 92 [distribution].

**Distribution.** —Indonesia.

***paku*** (Wells & Huisman, 1993): 110 [type locality: East Malaysia, Sabah, 2 km SW Long Pa Sia, confluence Sg. Ritan, Sg. Rurun, 04°21'N 115°42'E; RMNH; ♂; in *Scelotrichia*]. —Malicky 2010a: 62 [atlas; ♂].

—*tuskes* (Oláh & Johanson, 2010a): 82 [type locality: Malaysia, Sabah, Tawau, Maliau Basin Nepenthes Camp, Camel Trophy Hut, 4°43'59.3"N 116°52'39.7"E, 999 m; ♂; in *Scelotrichia*]. —Malicky 2013: 43 [to synonymy].

**Distribution.** —Malaysia.

***pucat*** (Wells & Huisman, 1993): 105 [type locality: East Malaysia, Sabah, Kundassang, Mesilau East River; NTM; ♂; in *Scelotrichia*]. —Malicky 2010a: 62 [atlas; ♂].

**Distribution.** —Malaysia.

***rienki*** (Wells & Huisman, 1993): 105 [type locality: West Malaysia, Templer’s Park, 20 km NW of Kuala Lumpur; NTM; ♂; in *Scelotrichia*]. —Malicky 2010a: 64 [atlas; ♂].

**Distribution.** —Malaysia.

***rincorama*** (Oláh, 1989): 269 [type locality: Vietnam, Tamdao, 1300 m a.s.l.; HNHM; ♂; in *Scelotrichia*]. —Armitage et al. 2005: 28 [checklist]. —Malicky 2010a: 63 [atlas; ♂].

**Distribution.** —Vietnam.

***rumput*** (Wells & Huisman, 1993): 106 [type locality: East Malaysia, Sarawak, Lambir National Park; NTM; ♂; in *Scelotrichia*]. —Malicky 2010a: 62 [atlas; ♂].

**Distribution.** —Malaysia.

***saranganica*** (Ulmer, 1951): 74 [type locality: [Indonesia], Java, Sarangan, Wasserfall des Kali Pagergede; ZMUH; ♂; in *Scelotrichia*]. —Wells and Malicky 1997: 176 [distribution]. —Malicky 2007a: 177 [checklist]. —Malicky 2010a: 61 [atlas; ♂]. —Malicky et al. 2014a: 6 [distribution]. —Malicky et al. 2016: 92 [distribution].

**Distribution.** —Indonesia.

***schmidi*** (Mey, 1981): 58 [type locality: [China], Tschatkalski Chrebet, bei Kumyschkan (Nord-westlicher Tienshan); NMPG; ♂; in *Madioxyethira*]. —Spuris 1989: 16 [checklist]. —Malicky, 1983b: 66 [atlas; ♂]. —Malicky 2004a: 74 [atlas]. —Malicky 2005b: 549 [checklist]. —Küçükbasmaci and Canbulat 2020: 114 [distribution].

**Distribution.** —China, Kyrgyzstan, Russia.

***simplex*** (Wells & Malicky, 1997): 177 [type locality: [Indonesia] N Sumatra, Huta Padang, 02°45'N 99°14'E; Collection Malicky; ♂; in *Scelotrichia*]. —Malicky 2007a: 177 [checklist]. —Malicky 2010a: 64 [atlas; ♂].

**Distribution.** —Indonesia.

***supsup*** (Wells, 1990a): 845 [type locality: Papua New Guinea, Central Province, Eilogo Creek, 9°27'S 147°27'E; ANIC; ♂; in *Scelotrichia*]. —Wells 1991: 526 [checklist].

**Distribution.** —Papua New Guinea.

***tatius*** (Malicky & Chantaramongkol, 2007): 1052 [type locality: Thailand, Mae Talai, 19°16'N 98°57'E, 400 m; ♂; in *Scelotrichia*]. —Malicky 2010a: 62 [atlas; ♂].

**Distribution.** —Thailand.

***telegonos*** (Malicky & Chantaramongkol, 2007): 1052 [type locality: Thailand, Doi Inthanon NP, Mae Klang bei 960 m, 18°32'N, 98°34'E; ♂; in *Scelotrichia*]. —Malicky 2010a: 63 [atlas; ♂].

**Distribution.** —Thailand.

***tellus*** (Malicky & Chantaramongkol, 2007): 1052 [type locality: Thailand, Doi Inthanon NP, Bang Khun Klang, 98°32'E 18°32'N, 1200 m; ♂; in *Scelotrichia*]. —Malicky 2010a: 60 [atlas; ♂].

**Distribution.** —Thailand.

***temenos*** (Malicky & Chantaramongkol, 2007): 1053 [type locality: Thailand, Doi Inthanon NP, Bang Khun Klang, 98°32'E 18°32'N, 1200 m; ♂; in *Scelotrichia*]. —Malicky 2010a: 63 [atlas; ♂].

**Distribution.** —Thailand.

***thingana*** (Oláh, 1989): 267 [type locality: Vietnam, Tamdao, tributary from high mountain of Tamdao, 200 m a.s.l.; HNHM; ♂; in *Scelotrichia*]. —Armitage et al. 2005: 28 [checklist]. —Malicky 2010a: 60 [atlas; ♂]. —Malicky 2013: 43 [possible senior synonym to *P.kipas* and *P.nana*]. —Zhou et al. 2016: 214 [♂; distribution]. —Ito 2017b: 197 [♂; distribution].

**Distribution.** —China, Vietnam.

***thunama*** (Oláh, 1989): 270 [type locality: Vietnam, Cucphuong, 400 m a.s.l.; HNHM; ♂; in *Scelotrichia*]. —Armitage et al. 2005: 28 [checklist]. —Malicky 2010a: 64 [atlas; ♂].

**Distribution.** —Vietnam.

***toira*** (Oláh, 1989): 268 [type locality: Vietnam, Tamdao, 1300 m a.s.l.; HNHM; ♂; in *Scelotrichia*]. —Armitage et al. 2005: 28 [checklist]. —Malicky 2010a: 62 [atlas; ♂].

**Distribution.** —Vietnam.

***trifurcata*** (Jacquemart, 1962b): 4 [type locality: Congo, Katanga, Sandoa; IRSNB; ♂; in *Hydroptila*]. —Marshall 1979 [to *Madioxyethira*].

**Distribution.** —Congo.

***vekonul*** (Oláh, 2016): 133 [type locality: Indonesia, West Papua, Batanta Island, valley of Warai stream, 00°50'51.0", 130°35'14.0"; Collection Oláh; ♂; as *Scelotrichia*]. —Oláh and Kovács 2018: 188 [distribution].

**Distribution.** —Indonesia.

***warabai*** (Wells, 1990a): 842 [type locality: Papua New Guinea, West Highland Province, Ukarumpa, Ba’i River, in gorge N. of village, 6°17'S 145°50'E; ANIC; ♂; ♀; in *Scelotrichia*]. —Wells 1991: 526 [checklist].

**Distribution.** —Papua New Guinea.

***willcairnsi*** (Cairns & Wells, 2008): 2612 [type locality: [Australia], North Queensland, Fishery Falls, south of Cairns, 17°11'S, 145°52'E; ANIC; ♂; ♀; larva; in *Scelotrichia*].

**Distribution.** —Australia.

#### Genus *Orinocotrichia* Harris, Flint, & Holzenthal, 2002

*Orinocotrichia* Harris, Flint, & Holzenthal, 2002c: 50 [type species: *Orinocotrichiacalcariga* Harris, Flint, & Holzenthal, 2002c, original designation].

*Orinocotrichia* is represented by three species, occurring in northeastern South America. The larva is unknown. Based on similarities in the adult head and the male and female genitalia, the genus is most closely related to *Flintiella* (Harris et al. 2002c).

***angelus*** de Souza, Santos, & Takiya, 2016b: 338 [type locality: Brazil, Maranhão, Carolina, Parque Nacional da Chapada das Mesas, Riacho Cancela, 07°06'43.4"S 47°17'16.6"W, 186 m; CZMA; ♂].

**Distribution.** —Brazil.

***calcariga*** Harris, Flint, & Holzenthal, 2002b: 51 [type locality: Venezuela, T. F. Amazonas, Río Cataniapo, 10 km S Puerto Ayacucho; NMNH; ♂; ♀].

**Distribution.** —Venezuela.

***tagola*** Oláh & Johanson, 2011: 251 [type locality: French Guiana, Approuaguekaw, Kaw Mt, 4°32.833'N, 52°11.452'W, 77 m; NHRS; ♂].

**Distribution.** —French Guiana.

#### Genus *Plethus* Hagen, 1887

*Plethus* Hagen, 1887: 643 [type species: *Hydroptilacursitans* Hagen, 1887, monotypic]. —Marshall 1979b: 168 [generic review]. —Malicky 2013: 43 [possible junior synonym to *Stactobia*]. —Ito and Saito 2016: 467 [generic review].

*Plethotrichia* Ulmer, 1951: 65 [type species: *Plethotrichiabaliana*Ulmer 1951, original designation]. —Marshall 1979b: 168 [to synonymy].

Twenty-seven species are currently included in the genus *Plethus*, occurring in south and Southeast Asia. Ulmer (1957) provided larval descriptions of both *P.acutus* and *P.cruciatus*. Marshall (1979b) considered *Plethus* to be most closely related to *Stactobia* and stated that it can be distinguished from *Stactobia* by its overall smaller size, less specialized genitalia, and larvae.

***acutus*** Ulmer, 1951: 64 [type locality: [Indonesia], Java, Badequelle am See Bedali; ZMUH; ♂]. —Malicky 2013: 43 [possible junior synonym to *Plethuscruciatus*].

**Distribution.** —Indonesia.

***amogawarsa*** Schmid, 1958b: 52 [type locality: [Sri Lanka], Ceylan, Nuwara Eliya (C. P.) 26-II, cours supérieur de la Nanu Oya, petite rivière rapide, sur lit caillouteux; depository not designated; ♂].

**Distribution.** —Sri Lanka.

***baliana*** (Ulmer, 1951): 66 [type locality: [Indonesia], Bali, Quelle unterhalb Tamantanda nahe Baturiti; ZMUH; ♂; in *Plethotrichia*]. —Wells 1993: 353 [distribution]. —Malicky 2010a: 57 [atlas; ♂]. —Malicky et al. 2014a: 6 [distribution].

**Distribution.** —Indonesia.

***banchaia*** Oláh, 1989: 261 [type locality: Vietnam, Tamdao, 1300 m a.s.l.; HNHM; ♂]. —Armitage et al. 2005: 28 [checklist]. —Malicky and Chantaramongkol 2007: 1047 [♂; distribution]. —Malicky 2010a: 57 [atlas; ♂].

**Distribution.** —Thailand, Vietnam.

***berbulu*** Wells, 1993: 353 [type locality: [Indonesia], Bali, Bali Barat, Sg. Pancoseming, N of Batuagung, near Negara; NTM; ♂]. —Malicky and Chantaramongol 2007: 1048 [distribution]. —Malicky 2010a: 58 [atlas; ♂]. —Malicky et al. 2014a: 6 [distribution]. —Malicky et al. 2016: 92 [distribution].

**Distribution.** —Indonesia.

***bishopi*** Wells & Huisman, 1993: 100 [type locality: West Malaysia, Genting Highlands, Gombak, tributary of Sg. Gombak above University of Malaya field station; NTM; ♂; case]. —Malicky 2010a: 57 [atlas; ♂].

**Distribution.** —Malaysia.

***bodikatuwa*** Schmid, 1958b: 53 [type locality: [Sri Lanka], Ceylan, Kandy (C. P., 2000 ft) 14-I, petite rivière encaissée et raide, dans la jungle, avec ruissellements latéraux; depository not designated; ♂].

**Distribution.** —Sri Lanka.

***cilamegha*** Schmid, 1958b: 53 [type locality: [Sri Lanka], Ceylan, Kitulgala (Sab., 750 ft) 2-III, Kelani Ganga, belle rivière coulant dans une vallée étroite et boisée, à la sortie des montagnes; depository not designated; ♂].

**Distribution.** —Sri Lanka.

***cruciatus*** Ulmer, 1951: 62 [type locality: [Indonesia], Sumatra, Bach beim Hause des Konsuls Schild unweit Padang; ZMUH; ♂; ♀]. —Wells and Malicky 1997: 176 [distribution]. —Malicky 2007a: 177 [checklist]. —Malicky 2010a: 58 [atlas; ♂]. —Malicky 2013: 43 [possible senior synonym to *Plethusacutus*]. —Malicky et al. 2014a: 6 [distribution]. —Malicky et al. 2016: 92 [distribution].

**Distribution.** —Indonesia.

***cursitans*** (Hagen, 1859): 209 [type locality: [Sri Lanka] Ceylon, Rambodde; depository not designated; ♂; in *Hydroptila*]. —Eaton 1873: 148 [comments on general appearance]. —Hagen 1887: 645 [♂, ♀]. —Schmid 1958b: 51 [♂; distribution].

**Distribution.** —Sri Lanka.

***hinchuna*** Oláh & Johanson, 2010a: 76 [type locality: Vietnam, Lamdong Province, Baoloc, Duchma stream; Collection Oláh; ♂].

**Distribution.** —Vietnam.

***kala*** Schmid, 1960: 92 [type locality: [Pakistan] Himalaya, Balakot; CNC; ♂]. —Schmid 1958c: 220 [as new species, *nomen nudum*; distribution]. —Lonsdale 2020: 36 [holotype depository].

**Distribution.** —Pakistan.

***roreta*** Oláh, 1989: 262 [type locality: Vietnam, Tamdao, 1300 m a.s.l.; HNHM; ♂]. —Armitage et al. 2005: 28 [checklist]. —Malicky 2010a: 58 [atlas; ♂].

**Distribution.** —Vietnam.

***sarkos*** Oláh & Johanson, 2010: 78 [type locality: India, Karnataka, Shimoga District, Jog Falls; HNHM; ♂].

**Distribution.** —India.

***scaevola*** Malicky & Chantaramongkol, 2007: 1047 [type locality: Thailand, Boripat WF, 6°59'N 100°09'E, 200 m; Collection Malicky; ♂]. —Malicky 2010a: 57 [atlas; ♂]. —Melnitsky et al. 2019: 539 [distribution].

**Distribution.** —Malaysia, Thailand.

***segitiga*** Wells & Huisman, 1993: 101 [type locality: West Malaysia, Kepong, Forest Research Institute of Malaya; NTM; ♂; case]. —Malicky 2010a: 57 [atlas; ♂].

**Distribution.** —Malaysia.

***sigiama*** Oláh, 1989: 260 [type locality: Vietnam, Hoahbinh, Ha Son Binh Province, singled at a small spring waterfall; HNHM; ♂]. —Armitage et al. 2005: 28 [checklist]. —Malicky 2010a: 58 [atlas; ♂].

**Distribution.** —Vietnam.

***tarquinius*** Malicky & Chantaramongkol, 2007: 1048 [type locality: Thailand, Doi Suthep NP, Montatan WF, 18°49'N 98°55'E; Collection Malicky; ♂]. —Malicky 2010a: 58 [atlas; ♂].

**Distribution.** —Thailand.

***tartaros*** Malicky & Chantaramongkol, 2007: 1048 [type locality: Thailand, Doi Suthep NP, 18°49'N 98°55'E, 1000 m; Collection Malicky; ♂]. —Malicky 2010a: 58 [atlas; ♂]. —Melnitsky et al. 2019: 539 [distribution].

**Distribution.** —Malaysia, Thailand.

***teiresias*** Malicky & Chantaramongkol, 2007: 1048 [type locality: Thailand, Chattrakan, 17°18'N 100°41'E; Collection Malicky; ♂]. —Malicky 2010a: 57 [atlas; ♂].

**Distribution.** —Thailand.

***toana*** Oláh, 1989: 263 [type locality: Vietnam, Tamdao, 1300 m a.s.l.; HNHM; ♂]. —Armitage et al. 2005: 28 [checklist]. —Malicky 2010a: 58 [atlas; ♂].

**Distribution.** —Vietnam.

***tullius*** Malicky & Chantaramongkol, 2007: 1046 [type locality: Thailand, Doi Inthanon NP, Bang Khun Klang, 98°32'E, 18°32'N, 1200 m; Collection Malicky; ♂]. —Malicky 2010a: 57 [atlas; ♂].

**Distribution.** —Thailand.

***udawasadenna*** Schmid, 1958b: 54 [type locality: [Sri Lanka], Ceylan, Kandy (C. P., 2000 ft) 14-I, petite rivière encaissée et raide, dans la jungle, avec ruissellements latéraux; depository not designated; ♂].

**Distribution.** —Sri Lanka.

***ukalegon*** Malicky & Chantaramongkol, 2007: 1047 [type locality: Taiwan, Taipei co., N Shihpei, 24°54'N 121°46'E, 435 m; Collection Malicky; ♂]. —Malicky 2014a: 1623 [checklist]. —Yang et al. 2016: 477 [checklist]. —Ito and Saito 2016: 468 [distribution; ♂; ♀; larva].

**Distribution.** —Japan, Taiwan.

***ulixes*** Malicky & Chantaramongkol, 2007: 1047 [type locality: Thailand, Doi Suthep NP, Montatan WF, 18°49'N 98°55'E; Collection Malicky; ♂]. —Malicky 2010a: 58 [atlas; ♂]. —Malicky et al. 2018: 1323 [distribution].

**Distribution.** —Thailand.

***uranos*** Malicky & Chantaramongkol, 2007: 1048 [type locality: Thailand, Tham Than Lod NP, 14°46'N 99°20'E, 500 m; Collection Malicky; ♂]. —Malicky 2010a: 57 [atlas; ♂].

**Distribution.** —Thailand.

***vajrabodhi*** Schmid, 1958b: 53 [type locality: [Sri Lanka], Ceylan, Kitulgala (Sab., 750 ft) 2-III, Kelani Ganga, belle rivière coulant dans une vallée étroite et boisée, à la sortie des montagnes; depository not designated; ♂]. —Oláh and Johanson 2010: 79 [distribution].

**Distribution.** —India, Sri Lanka.

#### Genus *Stactobia* McLachlan, 1880

*Stactobia* McLachlan, 1880: 505, 517 [type species: *Hydroptilafuscicornis* Schneider, 1845, subsequent designation by Mosely 1933: 162]. —Schmid 1959a: 1 [generic review]. —Kumanski 1979: 4 [key to species of Bulgaria]. —Marshall 1979b: 165 [generic review]. —Botosaneanu 1992: 41 [key to species in the Levant]. —Tobias 1999: 49 [distributional records; larval abundance]. —Malicky and Chantaramongkol 2007: 1042 [diagnostic characters of adults]. —Malicky 2013: 43 [possible senior synonym to *Plethus*]. —Lodovici and Valle 2013: 161 [review of Italian species]. —Ito 2017c: 201 [review of Japanese species].

*Afritrichia* Mosely, 1939d: 35 [type species: *Afritrichiaaurea* Mosely, 1939d, original designation]. —Schmid 1959a: 56 [to synonymy].

*Aratrichia* Mosely, 1948b: 76 [type species: *Aratrichiafahjia* Mosely, 1948b, original designation]. —Schmid 1959a: 51 [to synonymy].

*Lamonganotrichia* Ulmer, 1951: 68 [type species: *Lamonganotrichiacrassa* Ulmer, 1951, original designation]. —Marshall 1979b: 165 [to synonymy].

The genus *Stactobia* presently consists of 164 species and occurs in Southeast Asia, Africa, and in a general Palaearctic distribution. While *Stactobia* is one of the more successful hydroptilid genera in terms of species and abundance of individuals, it does not exhibit the broad geographical range of genera such as *Hydroptila* or *Oxyethira* (Marshall 1979b). Vaillant (1956) contributed most to knowledge of the biology of the genus, while Danecker (1961) gave a detailed life history. Marshall (1979b) hypothesized that its complete absence from the Neotropical faunal region may be attributable to two potential factors: the typically slow rate of dispersal of montane-stream dwellers compared with the faster rate of dispersal of lowland vegetation dwellers and competition with the members of the highly successful subfamily Leucotrichiinae. The six species groups (*furcata*, *martynovi*, *nielseni*, *vaillanti*, *bolzei*, and *japonica*) outlined by Marshall (1979b) followed those of Schmid (1959a) and Jacquemart (1973). The madicolous larvae live in thin sheets of water situated close to running-water habitats and can be found on rock surfaces near streams and (the sometimes nearly vertical) faces of waterfalls (Hynes 1970; Marshall 1979b). Larval adaptations, required by the habitat, include dorso-ventral flattening, heavily sclerotized and fused tergites, and short, robust legs used for clinging (Marshall 1979b).

***aihel*** Malicky, 2012: 1267 [type locality: China, Setschuan, Qingyin Pavilion, Jingshui, Emei Shan, 180 km SW Chengdu, 800–1200 m; Collection Malicky; ♂]. —Yang et al. 2016: 477 [checklist].

**Distribution.** —China.

***alaplica*** Sipahiler, 2012b: 1052 [type locality: Turkey, Zonguldak, Alapli, Gümeli, Bölüklü Yaylasi direction, 40°04'N 31°39'E, 690 m; HUAT; ♂; ♀].

**Distribution.** —Turkey.

***algira*** Vaillant, 1951: 17 [type locality: [Algeria]; depository not designated; ♂]. —Schmid 1959a: 32 [♂]. —Malicky 1983b: 65 [atlas; ♂]. —Malicky 2004a: 77 [atlas]. —Malicky 2005b: 548 [checklist].

**Distribution.** —Algeria.

***alpina*** Bertuetti, Lodovici, & Valle, 2004: 25 [type locality: [Italy], Piemonte, Provincia di Cuneo, Garessio m 650, affl. fiume Tanaro c/o Trappa; MBCG; ♂]. —Schmid 1959a: 32 [♂]. —Malicky 2004a: 77 [atlas]. —Malicky 2005b: 548 [checklist]. —Cianficconi et al. 2007: 67 [proposed as Italian endemic]. —Lodovici and Valle 2013: 164 [distribution]. —Le Guellec et al. 2013: 35 [distribution].

**Distribution.** —France, Italy.

***aoualina*** Botosaneanu & Dia in Dia and Botosaneanu 1983: 127 [type locality: [Lebanon], Station 6, Nabaa Aazibi, source alimentant l’affluent Nahr Aaray (torrent Jezzîne) du Nahr el Aouali, il s’agit plutôt d’un complexe de deux sources, réocrène et limnocrène, Massif de Niha, 900 m; ZMUA; ♂]. —Botosaneanu 1992: 45 [♂, ♀]. —Malicky 2004a: 74 [atlas]. —Malicky 2005b: 548 [checklist]. —Dia 2015: 51 [distribution].

**Distribution.** —Lebanon.

***atra*** (Hagen, 1864a): 825 [type locality: locality not given; depository not designated; description not provided; in *Hydroptila*]. —Hagen 1865a: 218 [morphological description; sex unknown]. —Hagen 1865b: 77 [as new species; [Portugal] Madeira; NHMUK; description; sex unknown abdomen missing]. —Eaton 1873: 142 [revision; to *Orthotrichia*]. —McLachlan 1880: 520 [revision]. —McLachlan 1884: 71 [distribution; to *Stactobia*]. —Morton 1893: 78 [distribution; ♂]. —Nybom 1948: 6 [♂; distribution]. —Schmid 1952: 655 [distribution]. —Schmid 1959a: 31 [♂]. —Nybom 1963: 114 [distribution]. —Nybom 1965: 90 [distribution]. —Botosaneanu 1967: 293 [distribution]. —Botosaneanu and Malicky 1978: 340 [checklist; possible senior synonym of *S.nybomi*]. —Malicky 1983b: 65 [atlas; ♂]. —Malicky 2004a: 77 [atlas]. —Malicky 2005b: 548 [checklist]. —Hughes 2006: 29 [biology].

**Distribution.** —Portugal, Spain.

***aurea*** (Mosely, 1939d): 35 [type locality: [Uganda], Ruwenzori, Namwamba Valley, 6500 ft.; NHMUK; ♂]. —Kimmins 1959: 56 [checklist]. —Schmid 1959b: 6 [species review]. —Johanson 1992: 118 [checklist]. —Wells and Andersen 1995: 145 [checklist]. —Mey 2007: 228 [wing venation].

**Distribution.** —Uganda.

***bademli*** Sipahiler, 2003b: 31 [type locality: [Turkey], Beysehir, Yenisarbademli, direction to Aksu, 5. km, 1350 m, 37°50'N, 31°19'E; depository not designated; ♂]. —Malicky 2004a: 79 [atlas]. —Malicky 2005b: 548 [checklist]. —Sipahiler 2005: 397 [distribution].

**Distribution.** —Turkey.

***balin*** Schmid, 1983: 244 [type locality: [India], Bengale occidental, Shepi; CNC; ♂]. —Lonsdale 2020: 32 [holotype depository].

**Distribution.** —India.

***ballur*** Schmid, 1983: 244 [type locality: [India], Assam, United Jaintia and Khasi Hills, Borghat; CNC; ♂]. —Lonsdale 2020: 32 [holotype depository].

**Distribution.** —India.

***banra*** Oláh, 1989: 259 [type locality: Vietnam, Ha Son Binh Province, Hoa Binh; HNHM; ♂]. —Armitage et al. 2005: 28 [checklist]. —Malicky 2010a: 54 [atlas; ♂].

**Distribution.** —Vietnam.

***beatensis*** Mosely, 1934b: 441 [type locality: France, Haut-Garonne, St.-Béat; NHMUK; ♂]. —Schmid 1952: 653 [distribution]. —Schmid 1959a: 16 [♂]. —Botosaneanu 1967: 293 [distribution]. —Botosaneanu and Malicky 1978: 340 [checklist]. —Moretti and Cianficconi 1981: 200 [checklist]. —Malicky 1983b: 64 [atlas; ♂]. —Cianficconi et al. 1999: 277 [distribution]. —Valle 2001: 64 [distribution]. —Malicky 2004a: 77 [atlas]. —Malicky 2005b: 548 [checklist]. —González and Menéndez 2011: 119 [distribution]. —Le Guellec 2011: 27 [distribution]. —Corallini and Cianficconi 2011: 628 [checklist]. —Lodovici and Valle 2013: 164 [♀; distribution]. —Martín et al. 2015: 74 [distribution].

**Distribution.** —France, Italy, Spain.

***beor*** Schmid, 1983: 280 [type locality: [India], Assam, NEFA, Kameng Frontier Division, Dirang Dzong; CNC; ♂]. —Lonsdale 2020: 32 [holotype depository].

**Distribution.** —India.

***beren*** Schmid, 1983: 272 [type locality: [India], Assam, NEFA, Kameng Frontier Division, Gigaon; CNC; ♂]. —Lonsdale 2020: 33 [holotype depository].

**Distribution.** —India.

***bersisik*** Wells, 1993: 353 [type locality: [Indonesia] Bali, Bali Barat, Sg. Pancoseming, N of Batuagung, near Negara; NTM; ♂]. —Wells and Malicky 1997: 175 [distribution]. —Malicky 2010a: 55 [atlas; ♂]. —Malicky et al. 2014a: 6 [distribution]. —Malicky et al. 2016: 92: [distribution].

**Distribution.** —Indonesia.

***betiri*** Wells & Malicky, 1997: 175 [type locality: [Indonesia] East Java, Meru Betiri; ANIC; ♂]. —Malicky 2010a: 53 [atlas; ♂]. —Malicky et al. 2014a: 6 [distribution].

**Distribution.** —Indonesia.

***bienda*** Oláh, 1989: 255 [type locality: Vietnam, Cucphuong, 400 m a.s.l.; HNHM; ♂]. —Armitage et al. 2005: 28 [checklist]. —Malicky 2010a: 58 [atlas; ♂].

**Distribution.** —Vietnam.

***bifur*** Schmid, 1983: 268 [type locality: [India], Assam, United Jaintia and Khasi Hills, Umlangshor; CNC; ♂]. —Lonsdale 2020: 33 [holotype depository].

**Distribution.** —India.

***bofur*** Schmid, 1983: 269 [type locality: [India], Assam, Manipour, Lithan; CNC; ♂]. —Lonsdale 2020: 33 [holotype depository].

**Distribution.** —India.

***bolzei*** Jacquemart, 1965: 8 [type locality: [Turkey] 5 km avant Gümüsane, St. 149; IRSNB; ♂; larva]. —Malicky 2005b: 548 [checklist].

**Distribution.** —Turkey.

***botvaz*** Oláh & Johanson, 2010a: 83 [type locality: Brunei, Belait district, 1.5 km on path to Bukit Teraja, small stream, 5 km N Kg. Teraja, 4°19'15"N 114°26'24"E; NHRS; ♂].

**Distribution.** —Brunei.

***calin*** Schmid, 1983: 276 [type locality: [India], Assam, NEFA, Kameng Frontier Division, Chug; CNC; ♂]. —Malicky and Chantaramongkol 2007: 1046 [distribution]. —Mattern 2015: 502 [distribution]. —Lonsdale 2020: 33 [holotype depository].

**Distribution.** —India, Nepal.

***campire*** Ito, 2017c: 225 [type locality: [Japan], Ryukyu Islands, Iriomote-jima, Urauchi-gawa, Kampire-no-taki, 24°21'17"N, 123°48'28"E, 83 m; CBM-ZI; ♂; larva].

**Distribution.** —Japan.

***caspersi*** Ulmer, 1950: 296 [type locality: [Bulgaria], Stüßwassergerinnsel am Steilufer dre Warnaer Bucht (Schwarzes Meer); ZMUH; ♂; larva]. —Malicky 1983b: 62 [atlas; ♂]. —Kumanski 1985: 110 [♂]. —Botosaneanu 1956: 366 [larva]. —Schmid 1959a: 20 [♂]. —Botosaneanu 1967: 293 [distribution]. —Malicky 1974: 122 [checklist]. —Botosaneanu and Malicky 1978: 340 [checklist]. —Kumanski 1979: 4 [♂; distribution]. —Moretti and Cianficconi 1981: 200 [checklist]. —Kumanski 1985: 110 [♂]. —Botosaneanu 1992: 42 [♂; ♀]. —Dallai and Afzelius 1995: 166 [sperm structure]. —Sipahiler 1998: 11 [distribution]. —Cianficconi et al. 1999: 57 [distribution]. —Valle 2001: 64 [distribution]. —Cianficconi et al. 2004: 329 [distribution]. —Malicky 2004a: 75 [atlas]. —Malicky 2005b: 548 [checklist]. —Sipahiler 2005: 397 [distribution]. —Cianficconi et al. 2005: 96 [habitat; distribution]. —Malicky 2005a: 68 [distribution]. —Cianficconi et al. 2007: 569, 575 [distribution]. —Ujvárosi et al. 2008: 113 [checklist]. —Ivanov 2011: 196 [checklist]. —Waringer and Graf 2011: 280 [larval synopsis]. —Lodovici and Valle 2013: 165 [♀; distribution]. —Dia 2015: 51 [distribution].

—*eretziana* Botosaneanu & Gasith, 1971: 96 [type locality: [Israel], en Avdat; TAU; ♂]. —Botosaneanu and Malicky 1978: 340 [to synonymy].

**Distribution.** —Bulgaria, Greece, Israel, Italy, Lebanon, Romania, Turkey.

***cataphanes*** Mey, 1998a: 551 [type locality: [Philippines, Mindanao], northern slope of Mt. Atuuganon range, 1050 m; ZMHB; ♂]. —Wells and Mey 2002: 134 [checklist].

**Distribution.** —Philippines.

***cermikensis*** Sipahiler, 1998: 9 [type locality: Turkey, Artvin, Savsat, Çermik Mahallesi, direction Lekoban yaylasi, 1800 m, 42°07'N, 41°35'E; depository not designated; ♂]. —Malicky 2004a: 79 [atlas]. —Malicky 2005b: 548 [checklist]. —Sipahiler 2005: 397 [distribution]. —Sipahiler 2008: 99 [checklist].

**Distribution.** —Turkey.

***chichibu*** Ito, 2017c: 219 [type locality: [Japan], Honshu, Saitama, Chichibu-shi, Otaki, Tochimoto, small stream, 35°56'N, 138°51'E, 670 m; CBM-ZI; ♂; larva].

**Distribution.** —Japan.

***cianficconiae*** Lodovici & Valle, 2013: 167 [type locality: [Italy], Sardegna, Nuoro, hygropetric road Gavoi-Ovodda; CMOR; ♂].

**Distribution.** —Italy.

***crassa*** (Ulmer, 1951): 69 [type locality: [Indonesia], Java, starke Rheokrene am See Lamongan; ZMUH; ♂; in *Lamonganotrichia*]. —Malicky 2010a: 55 [atlas; ♂]. —Malicky et al. 2014a: 6 [distribution].

**Distribution.** —Indonesia.

***culasi*** Wells & Mey, 2002: 119 [type locality: [Philippines] Panay, Culasi, San Vicente, 400 m; ZMHB; ♂].

**Distribution.** —Philippines.

***dain*** Schmid, 1983: 280 [type locality: [India], Assam, United Jaintia and Khasi Hills, Mawkap; CNC; ♂]. —Lonsdale 2020: 33 [holotype depository].

**Distribution.** —India.

***darvazica*** Ivanov, 1992: 231 [type locality: [Tajikistan], Pamir, Darvaz, brook above kishlak Kalai-Khumb along the river Pyandzh; ZIN; ♂].

**Distribution.** —Tajikistan.

***distinguenda*** Botosaneanu & Nozaki, 1996: 61 [type locality: [Japan], Honshu, Gifu, Otohime-buchi, Shimono, Fukuoka-cho; CBM-ZI; ♂]. —Tanida and Kuranishi 2016: 73 [checklist]. —Ito 2017c: 217 [♂; distribution]. —Ito and Shimura 2019: 35 [larva; distribution].

**Distribution.** —Japan.

***doehleri*** Schmid, 1959a: 46 [type locality: Pakistan septentrional, Katzarah Tso; CNC; ♂]. —Schmid, 1958c: 220 [as new species, *nomen nudum*; distribution]. —Lonsdale 2020: 33 [holotype depository].

**Distribution.** —Pakistan.

***dori*** Schmid, 1983: 272 [type locality: [India], Uttar Pradesh, Rishikesh; CNC; ♂]. —Lonsdale 2020: 33 [holotype depository].

**Distribution.** —India.

***durin*** Schmid, 1983: 252 [type locality: [India], Bengale occidental, Shepi; CNC; ♂]. —Lonsdale 2020: 34 [holotype depository].

**Distribution.** —India.

***dwalin*** Schmid, 1983: 246 [type locality: [India], Pauri Garhwal, Hanuman Chatti; CNC; ♂]. —Lonsdale 2020: 34 [holotype depository].

**Distribution.** —India.

***dwalur*** Schmid, 1983: 244 [type locality: [India], Bengale occidental, Dilpa; CNC; ♂]. —Lonsdale 2020: 34 [holotype depository].

**Distribution.** —India.

***eatoniella*** McLachlan, 1880: 517 [type locality: France, Switzerland; NHMUK; ♂]. —Klapálek 1900a: 73 [♂; larva]. —Klapálek 1900b: 3 [♂; larva; distribution]. —Thienemann 1904b: 261 [larva]. —Kimmins 1949: 232 [holotype selected]. —Schmid 1952: 653 [distribution]. —Schmid 1959a: 33 [♂]. —Botosaneanu 1967: 293 [distribution]. —Botosaneanu and Malicky 1978: 340 [checklist]. —Moretti and Cianficconi 1981: 200 [checklist]. —Malicky 1983b: 64 [atlas; ♂]. —Cianficconi et al. 1999: 57 [distribution]. —Urbanič 2004: 51 [distribution]. —Malicky 2004a: 76 [atlas]. —Malicky 2005b: 548 [checklist]. —Graf et al. 2005: 55 [distribution]. —Lubini-Ferlin and Vicentini 2005: 68 [checklist]. —Robert 2007: 82 [distribution]. —González and Mendéndez 2011: 119 [distribution]. —Waringer and Graf 2011: 280 [larval synopsis]. —Lodovici and Valle 2013: 167 [♀; distribution].

—*oredonensis* Mosely, 1934b: 443 [type locality: France, Haut-Garonne, St.-Béat; NHMUK; ♂]. —Kimmins 1949: 232 [to synonymy]. —Vaillant 1951: 16 [♂; wings].

**Distribution.** —Austria, France, Germany, Italy, Slovenia, Spain, Switzerland.

***ericae*** Malicky, 1981b: 337 [type locality: [Italy], Sardinien, Bach südwestlich von Gairo; Collection Malicky; ♂]. —Malicky 1983b: 65 [atlas; ♂]. —Malicky 2004a: 77 [atlas]. —Malicky 2005b: 548 [checklist]. —Cianficconi et al. 2007: 67 [proposed as Italian endemic]. —Lodovici and Valle 2013: 169 [distribution].

**Distribution.** —Italy.

***extensor*** Wells & Mey, 2002: 121 [type locality: [Philippines] Panay, San Renminigio, Aningalan; ZMHB; ♂].

**Distribution.** —Philippines.

***fahija*** (Mosely, 1948b): 76 [type locality: [Yemen], Western Aden Protectorate, Jebel Jihaf, Wadi Leje, beside waterfalls; NHMUK; ♂; in *Aratrichia*]. —Schmid 1959a: 51 [species review]. —Botosaneanu 1973: 66 [taxonomic note]. —Malicky 1983b: 66 [atlas; ♂]. —Botosaneanu 1992: 42 [♂, comparison with *S.pacatoria*]. —Malicky 2004a: 74 [atlas]. —Malicky 2005b: 548 [checklist].

**Distribution.** —Yemen.

***fethiyensis*** Sipahiler, 1989: 132 [type locality: Turkey, Mugla, Fethiye, 10 km to Köycegiz, 29°02'N, 36°45'E; depository not designated; ♂]. —Malicky 2004a: 79 [atlas]. —Malicky 2005b: 548 [checklist]. —Sipahiler 2005: 397 [distribution].

**Distribution.** —Turkey.

***filacea*** Mey, 2003b: 428 [type locality: Philippines, Luzon, Quezon province, east of Infanta, Magsaysay; ZMHB, to be transferred to either MPMP or UPLB; ♂].

**Distribution.** —Philippines.

***fischeri*** Schmid, 1958b: 49 [type locality: [Sri Lanka], Ceylan, Nuwara Eliya (C. P.) 26-II, cours supérieur de la Nanu Oya, petite rivière rapide, sur lit caillouteux, 6000 ft; depository not designated; ♂]. —Schmid 1959a: 44 [as new species, ♂].

**Distribution.** —Sri Lanka.

***forcipata*** Zhou, Yang, & Morse, 2013: 278 [type locality: China, Sichuan Province, Du-jiang-yan City, Guan County, 6 km W of Guan County Seat, Bai-sha River, 103.37°E, 31.00°N, 780 m; NAUJ; ♂]. —Yang et al. 2016: 477 [checklist].

**Distribution.** —China.

***forsslundi*** Schmid, 1959a: 40 [type locality: [Iran septentrional, Waliabad; CNC; ♂]. —Schmid 1959b: 693 [distribution]. —Malicky 1983b: 63 [atlas; ♂]. —Mirmoayedi and Malicky 2002: 164 [checklist]. —Malicky 2004a: 78 [atlas]. —Malicky 2005b: 548 [checklist]. —Sipahiler 2012b: 1054 [distribution]. —Lonsdale 2020: 34 [holotype depository].

**Distribution.** —Iran, Turkey.

***freyi*** Nybom, 1948: 8 [type locality: [Spain], Grand Canary; MZHF; ♂]. —Schmid 1959a: 27 [♂]. —Malicky 1983b: 63 [atlas; ♂]. —Malicky 2004a: 78 [atlas]. —Malicky 2005b: 548 [checklist].

**Distribution.** —Spain.

***froki*** Schmid, 1983: 276 [type locality: [India], Assam, NEFA, Kameng Frontier Division, Lifakpo; CNC; ♂]. —Lonsdale 2020: 34 [holotype depository].

**Distribution.** —India.

***furcata*** Mosely, 1930a: 180 [type locality: [France], Corsica, Corte; NHMUK; ♂]. —Mosely 1932: 176 [♂; distribution]. —Schmid 1959a: 13 [♂]. —Schmid 1952: 653 [distribution]. —Botosaneanu 1967: 293 [distribution]. —Botosaneanu and Malicky 1978: 340 [checklist]. —Moretti and Cianficconi 1981: 200 [checklist]. —Malicky 1983b: 64 [atlas; ♂]. —Malicky 2004a: 76 [atlas]. —Malicky 2005b: 548 [checklist]. —Lubini-Ferlin and Vicentini 2005: 68 [checklist]. —González and Menéndez 2011: 119 [distribution]. —Waringer and Graf 2011: 280 [larval synopsis]. —Lodovici and Valle 2013: 169 [♀; distribution].

**Distribution.** —France, Italy, Portugal, Spain.

***fuscicornis*** (Schneider, 1845): 346 [type locality: [Italy], Sicily, Messina; depository not designated; ♂; in *Hydroptila*]. —Eaton 1873: 137 [♂; distribution; as *Phrixocoma*]. —McLachlan 1880: 517 [revision; ♂; to *Stactobia*]. —McLachlan 1884: 72 [distribution]. —Ris 1897: 418 [♂; distribution]. —Ris 1903: 17 [distribution]. —Kimmins 1949: 229 [♂; revision; distribution]. —Schmid 1959a: 26 [♂]. —Botosaneanu 1967: 293 [distribution]. —Botosaneanu and Malicky 1978: 340 [checklist]. —Moretti and Cianficconi 1981: 200 [checklist]. —Malicky 1983b: 62, 65 [atlas; ♂]. —Cianficconi et al. 1999: 277 [distribution]. —Malicky 2005b: 548 [checklist]. —Corallini and Cianficconi 2011: 628 [checklist]. —Lodovici and Valle 2013: 171 [♂; ♀; distribution]. —Valle and Lodovici 2018: 147 [distribution].

—*obscura* (Kolenati, 1848): 106 [type locality: [Italy], Sicily, Messina; probably NHMW; probably ♂; in *Hydroptila*]. —Hagen 1864a: 825 [to synonymy].

**Distribution.** —France, Germany, Italy, Portugal, Switzerland.

***germani*** Malicky, Ivanov, & Melnitsky, 2011: 1493 [type locality: [Indonesia], Lombok, Kembangkuning, 2 km N Kotaraja, 490 m, 8°33'33"S, 116°25'22"E; ZIN; ♂; in *Scelotrichia*]. —Malicky et al. 2014a: 6 [distribution]. —Malicky et al. 2016: 92 [distribution].

**Distribution.** —Indonesia.

***gerutu*** Wells & Huisman, 1993: 102 [type locality: East Malaysia, Sabah, Sapong Falls, 10 km S of Tenom; NTM; ♂]. —Malicky 2010a: 54 [atlas; ♂].

**Distribution.** —Malaysia.

***gimli*** Schmid, 1983: 248 [type locality: [India], Pauri Garhwal, Akhrotkoti; CNC; ♂]. —Lonsdale 2020: 35 [holotype depository].

**Distribution.** —India.

***gloin*** Schmid, 1983: 278 [type locality: [India], Teri Garhwal, Pau Kal; CNC; ♂]. —Lonsdale 2020: 35 [holotype depository].

**Distribution.** —India.

***gomerina*** Botosaneanu, 1981b: 188 [type locality: [Spain] Canary Islands, Gomera, Bosque de El Cedro; ZMUA; ♂]. —Malicky 1983b: 63 [atlas; ♂]. —Malicky 2004a: 78 [atlas]. —Malicky 2005b: 548 [checklist].

**Distribution.** —Spain.

***gozmanyi*** Mey, 2007: 225 [type locality: Ethiopia, Simien Mts., Jinbar River, 38°05'E, 13°15'N, ca. 3400 m; ZMHB; ♂].

**Distribution.** —Ethiopia.

***grolin*** Schmid, 1983: 278 [type locality: [India], Assam, United Jaintia and Khasi Hills, Pynter; CNC; ♂]. —Lonsdale 2020: 35 [holotype depository].

**Distribution.** —India.

***gunma*** Ito, 2017c: 216 [type locality: [Japan], Honshu, Gunma, Minakami-shi, Okutone, Hidarimata-zawa, 36°48'N, 139·00'E; CBM-ZI; ♂].

**Distribution.** —Japan.

***gwili*** Schmid, 1983: 248 [type locality: [India], Assam, NEFA, Kameng Frontier Division, Nyukmadong; CNC; ♂]. —Lonsdale 2020: 35 [holotype depository].

**Distribution.** —India.

***hattorii*** Botosaneanu & Nozaki, 1996: 56 [type locality: [Japan], Honshu, Shizuoka, Tokusa, Oi-gawa, ca. 1200 m, Akaishi Mts., Shiauoka-shi; CBM-ZI; ♂]. —Tanida and Kuranishi 2016: 73 [checklist]. —Ito 2017c: 215 [♂; distribution].

**Distribution.** —Japan.

***huor*** Schmid, 1983: 266 [type locality: [India], Assam, NEFA, Kameng Frontier Division, Lungdur; CNC; ♂]. —Lonsdale 2020: 35 [holotype depository].

**Distribution.** —India.

***hurin*** Schmid, 1983: 264 [type locality: [India], Pauri Garhwal, Dhur; CNC; ♂]. —Malicky and Chantaramongkol 2007: 1046 [distribution]. —Lonsdale 2020: 36 [holotype depository].

**Distribution.** —India.

***inexpectata*** Botosaneanu & Nozaki, 1996: 55 [type locality: [Japan], Honshu, Kanagawa, Harutake-sawa, 480 m, Minoge, Hadano-shi; CBM-ZI; ♂]. —Tanida and Kuranishi 2016: 73 [checklist]. —Ito 2017c: 213 [♂; larva; distribution].

**Distribution.** —Japan.

***intermedia*** González & Terra, 1981: 203 [type locality: [Spain], Ferreirós de Abaixo (Lugo), Serra do Caurel, Río de Ferreirós, alt. 600 m; USCM; ♂]. —Malicky 1983b: 65 [atlas; ♂]. —Malicky 2004a: 77 [atlas]. —Malicky 2005b: 548 [checklist]. —González and Menéndez 2011: 119 [distribution]. —Martín et al. 2014: 72 [distribution].

**Distribution.** —Portugal, Spain.

***jacquemarti*** Malicky, 1977: 67 [type locality: [Greece], Kreta, Samaria-Schlucht; Collection Malicky; ♂]. —Botosaneanu and Malicky 1978: 340 [checklist]. —Malicky 1983b: 62 [atlas; ♂]. —Kumanski 1985: 106 [♂]. —Malicky 2004a: 75 [atlas]. —Malicky 2005b: 548 [checklist; treated as distinct species]. —Sipahiler 2005: 397 [distribution]. —Malicky 2005a: 69 [distribution]. —Sipahiler 2007: 38 [distribution].

**Distribution.** —Greece, Turkey.

***japonica*** Iwata, 1930: 63 [type locality: [Japan]; no holotype designated; larva]. —Botosaneanu 1990a: 47 [larval case comparison with *S.makartschenkoi*]. —Ito et al. 1993: 142 [checklist]. —Botosaneanu and Nozaki 1996: 54 [Neotype: [Japan], Honshu, Gifu, Otohime-Buchi, Shimono, Fukuokacho; CBM-ZI; ♂; distribution] —Tanida and Kuranishi 2016: 73 [checklist]. —Ito 2017c: 208 [Lectotype: [Japan], Honshu, Gifu, Fukuoka-mura (present address Nakatsugawa-shi, boundary of Shimono and Fukuoka), Otohime-taki Fall, 35°34'35"N, 137°27'53"E, 375 m; KUM; ♂; larva; distribution].

**Distribution.** —Japan.

***kanagawa*** Ito, 2017c: 208 [type locality: [Japan], Honshu, Kanagawa, Yugawara-machi, Makuyama, Niizaki-gawa, 35°10'02"N, 139°5'18"E, 200 m; CBM-ZI; ♂; larva].

**Distribution.** —Japan.

***kaputensis*** Wells & Andersen, 1995: 147 [type locality: Tanzania, Tanga region, West Usambara Mts, Mazumbai, Kaputu Stream, loc. 9, 1450 m a.s.l; ZMUB; ♂].

**Distribution.** —Tanzania.

***keluk*** Wells, 1993: 354 [type locality: [Indonesia], Bali, Tributary of Yeh Balian, nr Batungsel on Antosari - Pengastulan Road; NTM; ♂]. —Wells and Malicky 1997: 175 [distribution]. —Malicky 2010a: 55 [atlas; ♂]. —Malicky et al. 2014a: 6 [distribution].

**Distribution.** —Indonesia.

***kimminsi*** Schmid, 1959a: 15 [type locality: Iran septentrional, Meyur; CNC; ♂]. —Schmid 1959b: 692 [distribution]. —Malicky 1983b: 64 [atlas; ♂]. —Mirmoayedi and Malicky 2002: 164 [checklist]. —Malicky 2004a: 76 [atlas]. —Malicky 2005b: 548 [checklist]. —Sipahiler 2005: 397 [checklist; distribution]. —Lonsdale 2020: 36 [holotype depository].

**Distribution.** —Iran, Turkey.

***kiziroglui*** Sipahiler, 2012b: 1053 [type locality: Turkey, Izmir, Salihli, Birgi direction, 21 km Bozdag; HUAT; ♂].

**Distribution.** —Turkey.

***klapaleki*** Schmid, 1959a: 37 [type locality: Pakistan septentrional, Chhantir Gah; CNC; ♂]. —Schmid 1958c: 220 [as new species, *nomen nudum*; distribution]. —Schmid 1983: 250 [distribution]. —Lonsdale 2020: 36 [holotype depository].

**Distribution.** —India, Pakistan.

***klongpod*** Malicky, Suwannarat, & Laudee, 2018: 1320 [type locality: Thailand, Klong Pod an der Grenze des Kao Nan Nationalparks, 8°48'N, 99°34'E; Collection Malicky; ♂].

**Distribution.** —Thailand.

***kudung*** Wells & Huisman, 1993: 102 [type locality: West Malaysia, Cameron Highlands, “40 mile” falls, between Tanah Rata and Tapah; NTM; ♂]. —Malicky 2010a: 54 [atlas; ♂].

**Distribution.** —Malaysia.

***kyria*** Malicky, 2004b: 296 [type locality: [Nepal, Bardia National Park], am Rande der nordindischen Ebene im Südwesten von Nepal im Bereich des ersten Hügelkammes des Himalaya (Siwalik Range), bei dem Dorf Babai Basar in der Nähe der Straße von Nepalganj nach Birendranagar, ungefähr 30 km flussaufwärts vom Lager 1 (28°21'N, 81°42'E), von hygropetrischen Stellen entlang der Straße bei Babai Bazar; Collection Malicky; ♂]. —Malicky 2006: 253 [checklist]. —Mattern 2015: 502 [distribution].

**Distribution.** —Nepal.

***lavitra*** Oláh & Johanson, 2010a: 85 [type locality: Malaysia, Sabah, Tawau, Maliau Basin, Tributary to Maliau River, 4°44'32.1"N 116°58'14.4"E, 220 m; NHRS; ♂;].

**Distribution.** —Malaysia.

***lekoban*** Sipahiler, 1998: 9 [type locality: Turkey, Artvin, Savsat, Çermik Mahallesi, direction Lekoban yaylasi, 1800 m, 42°07'N, 41°35'E; depository not designated; ♂]. —Malicky 2004a: 79 [atlas]. —Malicky 2005b: 548 [checklist]. —Sipahiler 2005: 397 [distribution]. —Sipahiler, 2008: 99 [checklist].

**Distribution.** —Turkey.

***leptoclada*** Zhou, Yang, & Morse, 2013: 282 [type locality: China, Jiangxi Province, Wu-yuan County, 75 km N of Wu-yuan County Seat, Qing-hua River, 17.51°E, 29.15°N, 250 m; NAUJ; ♂]. —Yang et al. 2016: 477 [checklist].

**Distribution.** —China.

***livadia*** Malicky, 1984: 98 [type locality: Greece, Insel Serifos, 5 km westlich der Stadt Serifos; Collection Malicky; ♂]. —Malicky 2004a: 76 [atlas]. —Malicky 2005b: 548 [checklist]. —Malicky 2005a: 69 [distribution].

**Distribution.** —Greece.

***loki*** Schmid, 1983: 281 [type locality: [India], Teri Garhwal, Pau Kal; CNC; ♂]. —Lonsdale 2020: 36 [holotype depository].

**Distribution.** —India.

***loni*** Schmid, 1983: 278 [type locality: [India], Assam, United Jaintia and Khasi Hills, Mawpran; CNC; ♂]. —Lonsdale 2020: 36 [holotype depository].

**Distribution.** —India.

***maculata*** Vaillant, 1951: 17 [type locality: [Algeria]; depository not designated; ♂]. —Schmid, 1959a: 24 [♂]. —Moretti and Cianficconi 1981: 200 [checklist]. —Lodovici and Valle 2013: 172 [♂; ♀; distribution].

**Distribution.** —Algeria, Italy.

***makartschenkoi*** Botosaneanu & Levanidova, 1988: 169 [type locality: [Russia], Kunashir Island (southernmost of the Kuril Islands), Tyurino River at Sernovodsk; IBSS-RAS; ♂; larva]. —Botosaneanu 1990a: 47 [larval case comparison with Japanese species]. —Botosaneanu and Bozaki 1996: 55 [distribution]. —Chuluunbat and Morse 2007: 54 [distribution]. —Ivanov 2011: 196 [checklist]. —Tanida and Kuranishi 2016: 73 [checklist]. —Chuluunbat et al. 2016: 102 [distribution]. —Potikha and Vshivkova 2016: 364 [distribution]. —Ito 2017c: 203 [♂; ♀; larva; distribution].

**Distribution.** —Japan, Mongolia, Russia.

***malacantosa*** Schmid, 1952: 653 [type locality: Spain; CNC; ♂]. —Schmid 1959a: 23 [♂]. —Botosaneanu 1967: 293 [distribution]. —Botosaneanu and Malicky 1978: 340 [checklist]. —Malicky 1983b: 64 [atlas; ♂]. —Malicky 2004a: 76 [atlas]. —Malicky 2005b: 548 [checklist]. —González and Menéndez 2011: 119 [distribution]. —Lonsdale 2020: 36 [holotype depository].

**Distribution.** —Spain.

***malickyi*** Mey, 1981: 56 [type locality: [China], Tschatkalski Chrebet, bei Kumyschkan (Nord-westlicher Tienshan); NMPG; ♂]. —Malicky 1983b: 62 [atlas; ♂]. —Spuris 1989: 18 [checklist]. —Malicky 2004a: 75 [atlas]. —Malicky 2005b: 548 [checklist]. —Küçükbasmaci and Canbulat 2020: 114 [distribution].

**Distribution.** —China, Kyrgyzstan, Russia.

***manicata*** Wells & Mey, 2002: 119 [type locality: [Philippines] Negros, Patag NR, 750 m; ZMHB; ♂].

**Distribution.** —Philippines.

***mangyanica*** Mey, 1995: 193 [type locality: [Philippines], Mindoro, Calamintao, Bach zum Paghbahan-Fluß; Collection Mey; ♂]. —Wells and Mey 2002: 134 [checklist].

**Distribution.** —Philippines.

***margalitana*** Botosaneanu, 1974: 168 [type locality: [Israel], Nahal Arugot, ruisseau près de En Gedi au bord de la Mer Morte; TAU; ♂]. —Malicky 1983b: 63 [atlas; ♂]. —Botosaneanu 1992: 44 [♂]. —Malicky 2004a: 78 [atlas]. —Malicky 2005b: 548 [checklist].

**Distribution.** —Isra

***marlieri*** Schmid, 1959a: 43 [type locality: Iran septentrional, Polur; CNC; ♂]. —Schmid 1959b: 692 [distribution]. —Malicky 1983b: 63 [atlas; ♂]. —Sipahiler and Malicky 1987: 129 [distribution]. —Mirmoayedi and Malicky 2002: 164 [distribution]. —Malicky 2004a: 78 [atlas]. —Malicky 2005b: 548 [checklist]. —Sipahiler 2005: 398 [distribution]. —Chvojka 2006: 253 [distribution]. —Lonsdale 2020: 37 [holotype depository].

**Distribution.** —Iran, Turkey.

***martynovi*** Schmid, 1959a: 36 [type locality: Pakistan septentrional, Lulu Sar; CNC; ♂]. —Schmid 1958c: 220 [as new species, *nomen nudum*]. —Malicky and Chantaramongkol 2007: 1046 [distribution]. —Mattern 2015: 502 [distribution]. —Malicky 2018: 49 [checklist]. —Lonsdale 2020: 37 [holotype depository].

**Distribution.** —India, Nepal, Pakistan.

***mayeri*** Schmid, 1959a: 41 [type locality: Iran nord-oriental, Bavaman; CNC; ♂]. —Schmid 1959b: 693 [distribution]. —Malicky 1983b: 63 [atlas; ♂]. —Mirmoayedi and Malicky 2002: 164 [checklist]. —Malicky 2004a: 78 [atlas]. —Malicky 2005b: 548 [checklist]. —Lonsdale 2020: 37 [holotype depository].

—*decosteri* Jacquemart, 1965: 6 [type locality: [Turkey] 25 km avant Giresum, St. 158; IRSNB; ♂]. —Schmid 1983: 282 [to synonymy].

**Distribution.** —Iran, Turkey.

***mclachlani*** Kimmins, 1949: 232 [type locality: France, Cantal, Le Lioran; NHMUK; ♂]. —Schmid 1959a: 17 [♂]. —Schmid 1952: 652 [distribution; as *maclachlani*]. —Botosaneanu 1967: 293 [distribution; as *maclachlani*]. —Spuris 1972: 19, 27 [checklist; as *maclachlani*]. —Botosaneanu and Malicky 1978: 340 [checklist; as *maclachlani*]. —Kumanski 1979: 4 [♂; distribution]. —Malicky 1983b: 64 [atlas; ♂; as *maclachlani*]. —Kumanski 1985: 108 [♂]. —Malicky 2004a: 76 [atlas]. —Malicky 2005b: 548 [checklist; as *maclachlani*]. —Sipahiler 2005: 398 [distribution; as *maclachlani*]. —Malicky 2005a: 69 [distribution; as *maclachlani*]. —Robert 2007: 82 [checklist]. —Ujvárosi et al. 2008: 113 [checklist; as *maclachlani*]. —Martínez Menéndez and González 2010: 341 [distribution; as *maclachlani*]. —González and Menéndez 2011: 119 [distribution]. —Waringer and Graf 2011: 280 [larval synopsis]. —Coppa 2013: 123–132 [distribution; as *maclachlani*]. —Chvojka et al. 2016: 44 [distribution].

—*botosaneanui* Schmid, 1959a: 19 [type locality: [Macedonia], Yougoslavie (Macédoine) Perister; CNC; ♂]. —Botosaneanu 1967: 293 [as synonym]. —Botosaneanu and Malicky 1978: 340 [to synonymy]. —Lonsdale 2020: 33 [holotype depository].

—*delamarei* Coineau & Jacquemart, 1961: 540 [type locality: [France], Pyrénées-Orientales; depository not designated; ♂; ♀; larva]. —Jacquemart and Coineau 1962: 3 [checklist]. —Botosaneanu 1967: 293 [as synonym]. —Botosaneanu and Malicky 1978: 340 [to synonymy].

**Distribution.** —Bulgaria, Czech Republic, France, Germany, Greece, Macedonia, Portugal, Romania, Spain, Turkey.

***mindorica*** Mey, 1995: 193 [type locality: [Philippines], Mindoro, Paluan, Calawagan-Fluß; Collection Mey; ♂]. —Wells and Mey 2002: 134 [checklist].

**Distribution.** —Philippines.

***miresa*** Mey, 1998a: 552 [type locality: [Philippines, Mindanao], northern slope of Mt. Atuuganon range, 1050 m, hygropetric site; ZMHB; ♂]. —Wells and Mey 2002: 134 [checklist].

**Distribution.** —Philippines.

***monnioti*** Jacquemart, 1963d: 1 [type locality: Cyprus, Bains d’Aphrodite sur one paroi suintante; IRSNB; larva]. —Jacquemart 1973: 3 [♂; ♀; comment on larva]. —Malicky 2005b: 548 [checklist; note on ♂ description in Jacquemart 1973].

**Distribution.** —Cyprus, Greece.

***morettii*** Schmid, 1959a: 49 [type locality: Pakistan septentrional, Kawai; CNC; ♂]. —Schmid 1958c: 220 [as new species, *nomen nudum*; distribution]. —Schmid 1983: 281 [distribution]. —Lonsdale 2020: 37 [holotype depository].

**Distribution.** —India, Pakistan.

***moselyi*** Kimmins, 1949: 232 [type locality: France, Isère, Bourg d’Oisans; NHMUK; ♂]. —Schmid 1959a: 22 [♂]. —Botosaneanu 1967: 293 [distribution]. —Botosaneanu and Malicky 1978: 340 [checklist]. —Moretti and Cianficconi 1981: 200 [checklist]. —Malicky 1983b: 65 [atlas; ♂]. —Cianficconi et al. 1999: 57 [distribution]. —Cianficconi et al. 1999: 277 [distribution]. —Urbanič 2004: 51 [distribution]. —Malicky 2004a: 77 [atlas]. —Malicky 2005b: 548 [checklist]. —Cianficconi et al. 2005: 96 [habitat; distribution]. —Graf et al. 2005: 55 [distribution]. —Lubini-Ferlin and Vicentini 2005: 68 [checklist]. —Robert 2007: 82 [checklist]. —Cianficconi et al. 2007: 569, 575 [distribution]. —Waringer and Graf 2011: 280 [larval synopsis]. —Lodovici and Valle 2013: 173 [♀; distribution]. —Corallini and Cianficconi 2011: 628 [checklist].

**Distribution.** —Austria, France, Germany, Italy, Slovenia.

***naili*** Schmid, 1983: 270 [type locality: [India], Bengale occidental, Dilpa; CNC; ♂]. —Lonsdale 2020: 37 [holotype depository].

**Distribution.** —India.

***nalin*** Schmid, 1983: 280 [type locality: [India], Assam, NEFA, Kameng Frontier Division, Lungdur; CNC; ♂]. —Malicky and Chantaramongkol 2007: 1046 [distribution]. —Malicky 2010a: 55 [atlas; ♂]. —Lonsdale 2020: 37 [holotype depository].

**Distribution.** —India, Thailand.

***nielseni*** Schmid, 1959a: 50 [type locality: Iran, Zanus (Ost. 2); CNC; ♂]. —Schmid 1958c: 220 [as new species, *nomen nudum*]. —Schmid 1983: 270, 283 [checklist]. —Malicky 1983b: 62 [atlas; ♂]. —Mirmoayedi and Malicky 2002: 164 [checklist]. —Malicky 2004a: 75 [atlas]. —Malicky 2005b: 548 [checklist]. —Lonsdale 2020: 38 [holotype depository].

**Distribution.** —Iran.

***nishimotoi*** Botosaneanu & Nozaki, 1996: 58 [type locality: [Japan], Honshu, Aichi, Shimada-gawa, a tributary of Kansa-gawa, Horai-cho; CBM-ZI; ♂]. —Tanida and Kuranishi 2016: 73 [checklist]. —Ito 2017c: 216 [♂; distribution]. —Park et al. 2018: 108 [♂; distribution]. —Park and Kong 2020: 298 [checklist].

**Distribution.** —Japan, Korea.

***noldi*** Schmid, 1983: 256 [type locality: [India], Kumaon, Loharket; CNC; ♂]. —Lonsdale 2020: 38 [holotype depository].

**Distribution.** —India.

***nori*** Schmid, 1983: 274 [type locality: [India], Pauri Garhwal, Pau Kal; CNC; ♂]. —Malicky 2006: 253 [checklist; distribution]. —Mattern 2015: 502 [distribution]. —Lonsdale 2020: 38 [holotype depository].

**Distribution.** —India, Nepal.

***nybomi*** Schmid, 1959a: 29 [type locality: [Portugal], Madère, Ribera Brava; MZHF; ♂]. —Botosaneanu 1967: 293 [distribution]. —Botosaneanu and Malicky 1978: 340 [possible junior synonym of *S.atra*, checklist]. —Malicky 1983b: 65 [atlas; ♂]. —Malicky 2004a: 77 [atlas]. —Malicky 2005b: 548 [checklist]. —Hughes 2006: 29 [biology].

**Distribution.** —Portugal.

***oin*** Schmid, 1983: 256 [type locality: [India], Pauri Garhwal, Rudraprayag; CNC; ♂]. —Lonsdale 2020: 38 [holotype depository].

**Distribution.** —India.

***olgae*** Martynov, 1927: 177 [type locality: [Uzbekistan?], Turkestan, River Tchimganka, near the Sanatory; depository not designated; ♂]. —Martynov 1934: 156 [♂]. —Mey 1978b: 28 [distribution]. —Mey 1981: 57 [♂; distribution]. —Schmid 1983: 252 [♂]. —Malicky 1983b: 62 [atlas; ♂]. —Spuris 1989: 18 [checklist]. —Malicky 2004a: 75 [atlas]. —Malicky 2005b: 548 [checklist].

**Distribution.** —Russia, Tajikistan, Uzbekistan (?).

***ori*** Schmid, 1983: 254 [type locality: [India] Sikkim, Rhenok; CNC; ♂]. —Lonsdale 2020: 38 [holotype depository].

**Distribution.** —India.

***pacatoria*** Dia & Botosaneanu, 1980: 369 [type locality: [Lebanon], dans un habitat madicole sur le trajet de ruisseau Ouadi Ras el Mâ, près du village Haret Jandal Ech Chouf (Liban, Chouf, bassin du Nahr Aouali: 33°38'N, 35°36'E), à une altitude de 800–900 m, dans une zone calcaire; ZMUA; ♂; larva; case]. —Malicky 1983b: 66 [atlas; ♂]. —Botosaneanu 1992: 47 [♂, ♀]. —Malicky 2004a: 74 [atlas]. —Malicky 2005b: 548 [checklist; suggestion that it should be compared to *S.fahija*]. —Dia 2015: 51 [distribution].

**Distribution.** —Lebanon.

***parva*** Wells & Dudgeon, 1990: 164 [type locality: Hong Kong, Tai Po Kao Forest stream; NHMUK; ♂]. —Yang et al. 2016: 477 [checklist].

**Distribution.** —Hong Kong.

***phix*** Malicky & Chantaramongkol, 2007: 1045 [type locality: Bhutan, Trongsa, Telegangchhu Bridge, 27°29'N 90°31'E, 2100 m; Collection Malicky; ♂].

**Distribution.** —Bhutan.

***plethoides*** Wells & Mey, 2002: 119 [type locality: [Philippines] Panay, Culasi, San Vicente, 400 m; ZMHB; ♂].

**Distribution.** —Philippines.

***polybos*** Malicky & Chantaramongkol, 2007: 1045 [type locality: Bhutan, Chananachhu bei Gasekha Zam, 27°25'N 89°14'E, 2900 m; Collection Malicky; ♂].

**Distribution.** —Bhutan.

***princesa*** Wells & Mey, 2002: 119 [type locality: [Philippines] Palawan, Puerto Princesa, Cayasan, Balsahan; ZMHB; ♂].

**Distribution.** —Philippines.

***pyrrhos*** Malicky & Chantaramongkol, 2007: 1044 [type locality: Thailand, Doi Inthanon NP, Siribhum WF, 18°32'N 98°31'E, 1300 m; Collection Malicky; ♂]. —Malicky 2010a: 55 [atlas; ♂].

**Distribution.** —Thailand.

***python*** Malicky & Chantaramongkol, 2007: 1043 [type locality: Thailand, Doi Inthanon NP, Bang Khun Klang, 98°32'E 18°32'N, 1200 m; Collection Malicky; ♂]. —Malicky 2010a: 55 [atlas; ♂]. —Mattern 2015: 502 [distribution].

**Distribution.** —Nepal, Thailand.

***quadrispina*** Kimmins, 1951: 200 [type locality: [Myanmar], N.E. Burma, Kambaiti, 6300 ft.; NHRS; ♂]. —Schmid 1959a: 39 [♂]. —Wityi et al. 2015: 47 [checklist].

**Distribution.** —Myanmar.

***quezonensis*** Mey, 2003b: 430 [type locality: Philippines, Luzon, Quezon province, east of Infanta, Magsaysay; ZMHB, to be transferred to either MPMP or UPLB; ♂].

**Distribution.** —Philippines.

***radovanovici*** Schmid, 1959a: 53 [type locality: Pakistan, Hindou-Kouch, Khoghozi; CNC; ♂]. —Schmid 1958c: 220 [as new species, *nomen nudum*]. —Schmid 1983: 274 [distribution]. —Malicky 2013: 43 [possible senior synonym to *S.schnorri*]. —Lonsdale 2020: 39 [holotype depository].

**Distribution.** —India, Pakistan.

***rahang*** Wells & Huisman, 1993: 103 [type locality: West Malaysia, Genting Highlands, stream 1.5 km below University of Malaya field station, at fall; NTM; ♂; larva; case]. —Malicky 2010a: 54 [atlas; ♂].

**Distribution.** —Malaysia.

***regularis*** Mey, 1996: 50 [type locality: Nord Vietnam, Okuiho, 14 km nördlich Sa Pa, am Fuße des Fan Si Pan Bergmasiv, 1100 m; ZMHB; ♂]. —Armitage et al. 2005: 28 [checklist]. —Mey 2005a: 281 [distribution]. —Malicky 2010a: 53 [atlas; ♂].

**Distribution.** —Vietnam.

***reticulata*** Wells & Mey, 2002: 121 [type locality: [Philippines] Panay,San Reminigio, Aningalan; ZMHB; ♂].

**Distribution.** —Philippines.

***rhombica*** Zhou, Yang, & Morse, 2013: 281 [type locality: China, Sichuan Province, Zhao-jue County, Jie-fang Village, Jie-fang-gou stream, S307 at 553.0 km, 102.33°E, 27.52°N, 2925 m; NAUJ; ♂]. —Yang et al. 2016: 477 [checklist].

**Distribution.** —China.

***risiana*** Schmid, 1959a: 52 [type locality: Pakistan, Bélouchistan, Central Zarghun; CNC; ♂]. —Lonsdale 2020: 39 [holotype depository].

**Distribution.** —Pakistan.

***ruthiel*** Malicky & Graf, 2015: 31 [type locality: Ethiopia, Kleiner Waldbach N von Addis Abeba, 9°05'N, 38°43'E, 2800 m ; Collection Malicky; ♂].

**Distribution.** —Ethiopia.

***salmakis*** Malicky & Chantaramongkol, 2007: 1044 [type locality: China, Zhejiang prov., Gutien shan, 26°21'N 119°26'E, 450 m; Collection Malicky; ♂]. —Yang et al. 2016: 477 [checklist].

**Distribution.** —China.

***schmidi*** Kimmins, 1964: 47 [type locality: [Nepal], Taplejung Distr., Dobhan, ca. 3,500 ft a.s.l., shady places on shrubby slope above R. Tamur; NHMUK; ♂]. —Schmid 1983: 252 [♂; distribution]. —Malicky 2006: 253 [checklist]. —Mattern 2015: 502 [distribution]. —Malicky 2018: 49 [checklist].

**Distribution.** —India, Nepal.

***schnorri*** Malicky, 2004b: 296 [type locality: [Nepal, Bardia National Park], am Rande der nordindischen Ebene im Südwesten von Nepal im Bereich des ersten Hügelkammes des Himalaya (Siwalik Range), bei dem Dorf Babai Basar in der Nähe der Straße von Nepalganj nach Birendranagar, ungefähr 30 km flussaufwärts vom Lager 1 (28°21'N, 81°42'E), lag das Ufer des Babai Nadi in wenigen Metern Entfemung, vom “westlicher” Bach, 320 m; Collection Malicky; ♂]. —Malicky 2006: 253 [checklist]. —Malicky 2012: 43 [possible junior synonym of *S.radovanovici*]. —Mattern 2015: 502 [distribution].

**Distribution.** —Nepal.

***seki*** Sipahiler, 2000: 26 [type locality: Turkey, Fethiye, Gelemis (Patara), Seki Çayi, 100 m; depository not designated; ♂]. —Malicky 2004a: 79 [atlas]. —Malicky 2005b: 548 [checklist]. —Sipahiler 2005: 398 [distribution].

**Distribution.** —Turkey.

***semele*** Malicky & Chantaramongkol, 2007: 1044 [type locality: Taiwan, Hsinchu co., S Chienshih, Euro, 24°39'N 121°10'E; Collection Malicky; ♂]. —Malicky 2014a: 1623 [checklist]. —Yang et al. 2016: 477 [checklist]. —Ito 2017c: 230 [♂; larva; distribution].

**Distribution.** —Taiwan.

***shahdara*** Ivanov, 1992: 233 [type locality: [Tajikistan], West Pamir, Khorog, brook opposite the Botanical Garden; ZIN; ♂].

**Distribution.** —Tajikistan.

***smoli*** Schmid, 1983: 270 [type locality: [India], Assam, United Jaintia and Khasi Hills, Lakadong; CNC; ♂]. —Lonsdale 2020: 39 [holotype depository].

**Distribution.** —India.

***snori*** Schmid, 1983: 260 [type locality: [India], Assam, NEFA, Kameng Frontier Division, Nyukmadong; CNC; ♂]. —Malicky and Chantaramongkol 2007: 1046 [distribution]. —Lonsdale 2020: 39 [holotype depository].

**Distribution.** —India, Nepal.

***snufi*** Schmid, 1983: 262 [type locality: [India] Sikkim, Karponang; CNC; ♂]. —Lonsdale 2020: 39 [holotype depository].

**Distribution.** —India.

***spicifera*** Zhou, Yang, & Morse, 2013: 283 [type locality: China, Sichuan Province, Wen-chuan County, 13 km S of Wen-chuan County Seat, Ban-qiao-gou stream (trib. of Min-Jiang), 103.34°E, 31.28°N, 1313 m; NAUJ; ♂]. —Yang et al. 2016: 477 [checklist].

**Distribution.** —China.

***storai*** Nybom, 1948: 7 [type locality: [Spain], Canary Islands; MZHF; ♂]. —Schmid 1959a: 29 [♂]. —Botosaneanu 1981b: 186 [distribution]. —Malicky 1983b: 63 [atlas; ♂]. —Botosaneanu 1993b: 160 [distribution]. —Malicky 2004a: 78 [atlas]. —Malicky 2005b: 548 [checklist].

**Distribution.** —Spain.

***sujangsanica*** Kumanski, 1990: 46 [type locality: Korea, Province Hwanghe namdo (Southern Hwanghe), Sujang Mt. (a small mountain near Hedzu), below and above waterfall; SOFM; ♂; ♀]. —Arefina et al. 2002: 103 [distribution]. —Ivanov 2011: 196 [checklist]. —Potikha and Vshivkova 2016: 364 [distribution]. —Park and Kong 2020: 298 [checklist].

**Distribution.** —Korea, Russia.

***takuk*** Wells & Huisman, 1993: 103 [type locality: East Malaysia, Sarawak, Bako National Park, Sg. Delima; NTM; ♂; larva; case]. —Malicky 2010a: 54 [atlas; ♂].

**Distribution.** —Malaysia.

***telamon*** Malicky & Chantaramongkol, 2007: 1043 [type locality: Nepal, Mahadev Khola, 27°53'N 85°39'E, 1300 m; Collection Malicky; ♂]. —Mattern 2015: 502 [distribution]. —Malicky 2018: 49 [checklist].

**Distribution.** —Nepal.

***telchinos*** Malicky & Chantaramongkol, 2007: 1045 [type locality: Nepal, Ganesh Himal, Sheplu, 2100 m; Collection Malicky; ♂]. —Mattern 2015: 502 [distribution].

**Distribution.** —Nepal.

***teldi*** Schmid, 1983: 258 [type locality: [India], Kumaon, Loharket; CNC; ♂]. —Lonsdale 2020: 40 [holotype depository].

**Distribution.** —India.

***telemachos*** Malicky & Chantaramongkol, 2007: 1044 [type locality: Thailand, Doi Inthanon NP, Bang Khun Klang, 98°32'E 18°32'N, 1200 m; Collection Malicky; ♂]. —Malicky 2010a: 53 [atlas; ♂].

**Distribution.** —Thailand.

***telephos*** Malicky & Chantaramongkol, 2007: 1045 [type locality: Thailand, Ban Yang Bong, 19°21'N 98°54'E, 500 m; Collection Malicky; ♂]. —Malicky 2010a: 53 [atlas; ♂].

**Distribution.** —Thailand.

***tenes*** Malicky & Chantaramongkol, 2007: 1043 [type locality: Thailand, Doi Inthanon NP, Bang Khun Klang, 98°32'E 18°32'N, 1200 m; Collection Malicky; ♂]. —Malicky 2010a: 53 [atlas; ♂].

**Distribution.** —Thailand.

***terminus*** Malicky & Chantaramongkol, 2007: 1043 [type locality: Thailand, Chaeson NP, 18°46'N 99°28'E, 500 m; Collection Malicky; ♂]. —Oláh and Johanson 2010a: 85 [distribution]. —Malicky 2010a: 53 [atlas; ♂].

**Distribution.** —Laos, Thailand.

***thacla*** Oláh, 1989: 256 [type locality: Vietnam, Bac Thai Province, Quang Chu village; HNHM; ♂]. —Armitage et al. 2005: 28 [checklist]. —Malicky 2010a: 54 [atlas; ♂].

**Distribution.** —Vietnam.

***thorin*** Schmid, 1983: 2 [type locality: [India], Pauri Garhwal, Jungal Chatti; CNC; ♂]. —Lonsdale 2020: 40 [holotype depository].

**Distribution.** —India.

***thrain*** Schmid, 1983: 250 [type locality: [India] Sikkim, Karponang; CNC; ♂]. —Lonsdale 2020: 40 [holotype depository].

**Distribution.** —India.

***throhir*** Schmid, 1983: 264 [type locality: [India], Assam, Manipour, Sirohi Kashong; CNC; ♂]. —Lonsdale 2020: 40 [holotype depository].

**Distribution.** —India.

***throli*** Schmid, 1983: 258 [type locality: [India] Sikkim, Rangli; CNC; ♂]. —Malicky and Chantaramongkkol 2007: 1046 [distribution]. —Mattern 2015: 502 [distribution]. —Lonsdale 2020: 40 [holotype depository].

**Distribution.** —India, Nepal.

***thror*** Schmid, 1983: 262 [type locality: [India], Assam, NEFA, Kameng Frontier Division, Chug; CNC; ♂]. —Lonsdale 2020: 40 [holotype depository].

**Distribution.** —India.

***tjederi*** Schmid, 1959a: 43 [type locality: Pakistan septentrional, Dunga Nar; CNC; ♂]. —Schmid 1958c: 220 [as new species, *nomen nudum*; distribution]. —Schmid 1983: 252 [distribution]. —Lonsdale 2020: 40 [holotype depository].

**Distribution.** —India, Pakistan.

***tonyi*** Wells & Huisman, 1993: 102 [type locality: East Malaysia, Sabah, Tenom, stream behind Hotel Tenom; NTM; ♂]. —Malicky 2010a: 54 [atlas; ♂].

**Distribution.** —Malaysia.

***trungcha*** Oláh, 1989: 258 [type locality: Vietnam, Bac Thai Province, Quang Chu village; HNHM; ♂]. —Armitage et al. 2005: 28 [checklist]. —Malicky 2010a: 54 [atlas; ♂].

**Distribution.** —Vietnam.

***tuor*** Schmid, 1983: 268 [type locality: [India], Assam, Manipour, Sihai Khulen; CNC; ♂]. —Lonsdale 2020: 41 [holotype depository].

**Distribution.** —India.

***turanica*** Ivanov, 1992: 229 [type locality: [Tajikistan], Varzob valley, 23 km from Dushanbe, hygropetrical fauna, near waterfall; ZIN; ♂].

**Distribution.** —Tajikistan.

***ulmeriana*** Schmid, 1959a: 47 [type locality: Pakistan septentrional, Kaghan; CNC; ♂]. —Schmid 1958c: 220 [as new species, *nomen nudum*; distribution]. —Lonsdale 2020: 41 [holotype depository].

**Distribution.** —Pakistan.

***urania*** Malicky, 1976: 93 [type locality: Cyprus, Ayios Nikolaos, 870 m; Collection Malicky; ♂]. —Malicky 1983b: 63 [atlas; ♂]. —Malicky 2004a: 78 [atlas]. —Malicky 2005b: 548 [checklist].

**Distribution.** —Cyprus.

***urauchi*** Ito, 2017c: 225 [type locality: [Japan], Ryukyu Islands, Iriomote-jima, Urauchi-gawa, Kampire-no-taki, 24°21'N, 123°48'E, 90 m; CBM-ZI; ♂; larva].

**Distribution.** —Japan.

***vaillanti*** Schmid, 1959a: 55 [type locality: Guinée française, sous la roue hydro-électrique de l’Institut Pasteur de Kindia; CNC; ♂]. —Schmid 1983: 282 [checklist]. —Gibon 1985: 249 [distribution]. —Lonsdale 2020: 41 [holotype depository].

**Distribution.** — Côte d’Ivoire, French Guinea.

***wimmeri*** Malicky, 1988b: 63 [type locality: [Turkey], Ost-Türkei, Sumela-Tal 50 km südlich Trabzon; Collection Malicky; ♂]. —Malicky 2004a: 79 [atlas]. —Malicky 2005b: 548 [checklist]. —Sipahiler 2005: 398 [distribution]. —Sipahiler 2008: 99 [checklist].

**Distribution.** —Turkey.

***yenicensis*** Sipahiler, 2012b: 1054 [type locality: Turkey, Karabük, Yenice, Karakaya, 41°13'N 32°28'E, 958 m; HUAT; ♂; ♀].

**Distribution.** —Turkey.

***yona*** Ito, 2017c: 222 [type locality: [Japan], Ryukyu Islands, Okinawa-jima, Kunigami-son, Aha, Fun-gawa, Tanaga-gumui, 26°43'30"N, 128°17'12"E, 78 m; CBM-ZI; ♂; larva].

**Distribution.** —Japan.

***zarva*** Oláh, 2012: 51 [type locality: Indonesia, Papua, Raja Empat Archipelago, Batanta Island, Warmon Creek, 2. waterfall, 0°50'23.25"S 130°42'35.18"E; Collection Oláh; ♂]. —Oláh and Kovács 2018: 188 [distribution].

**Distribution.** —Indonesia.

#### Genus *Stactobiella* Martynov, 1924

*Stactobiella* Martynov, 1924: 57 [type species: *Stactobiaulmeri* Siltala, 1908, monotypic]. —Ross 1948: 202 [species key to males]. —Marshall 1979b: 169 [generic review]. —Blickle 1979: 8 [key to species of America north of Mexico]. —Moulton and Stewart 1996: 130 [key to species of the Interior Highlands of North America]. —Kachalova in Medvedev 1998: 191 [key to the species of the European part of the USSR].

*Tascobia* Ross, 1944: 124 [type species: *Stactobiapalmata* Ross, 1944, original designation]. —Ross 1948: 202 [to synonymy].

The genus *Stactobiella* is presently represented by 22 species, exhibiting a Holarctic distribution. Discussion of the larva of *S.palmata* was provided by Ross (1944) and Wiggins (1996). Marshall (1979b) concluded, based on the unspecialized larvae and basic hydroptilid form, that *Stactobiella* is a primitive member of Stactobiinae and, based on adult features, that it is most closely related to *Plethus* and *Stactobia*. The three species groups recognized by Marshall (*biramosa*, *brustia*, and *ulmeri*), based on features of the male genitalia, followed those of Ross (1948).

***aichi*** Ito, 2020: 565 [type locality: Japan, Honshu, Aichi, Shinshiro-shi, Toyooka, Ôtsutani-gawa, near river mouth, 34.990556N, 137.624722E, 129 m above sea level; CBM-ZI; ♂].

**Distribution.** —Japan.

***alasignata*** Botosaneanu, 1993a: 184 [type locality: [Russia], Primorie, Ussuri River in its upper reach, near village Stepanovka, short distance from River Arsenievka mouth; ZIN; ♂; ♀]. —Botosaneanu 1993c: 247 [addenda]. —Arefina et al. 2002: 104 [distribution]. —Ivanov 2011: 196 [checklist]. —Chuluunbat et al. 2016: 102[distribution].

**Distribution.** —Mongolia, Russia.

***amami*** Ito, 2020: 567 [type locality: Japan, Ryukyu, Amami-o-shima, Uken-son, Kawauchi-gawa, middle reach, 28.2667N, 129.3500E, 90 m above sea level; CBM-ZI; ♂].

**Distribution.** —Japan.

***biramosa*** Martynov, 1929: 297 [type locality: [Russia], River Bija, near the farm of Smolnikov; depository not designated; ♂]. —Martynov 1934: 158 [♂]. —Spuris 1989: 18 [checklist]. —Arefina et al. 2002: 104 [♂; ♀; distribution]. —Arefina and Armitage 2003: 17 [distribution]. —Ivanov 2011: 196 [checklist]. —Zhou et al. 2016: 214 [distribution]. —Chuluunbat et al. 2016: 102[distribution]. —Potikha and Vshivkova 2016: 364 [distribution]. —Zasypkina 2016: 486 [distribution]. —Ito 2020: 562 [♂; ♀; distribution].

**Distribution.** —China, Mongolia, Russia.

***brustia*** (Ross, 1938a): 115 [type locality: [United States], Wyoming, Parco, along North Platte River; INHS; ♂; in *Stactobia*]. —Ross 1944: 124 [in *Tascobia*]. —Denning 1947a: 145 [distribution]. —Blickle 1979: 55, 61 [checklist; ♂]. —Blinn and Ruiter 2005: 69 [distribution; biology].

**Distribution.** —U.S.A.

***cahaba*** Harris, 1985b: 620 [type locality: [United States], Alabama, Bibb County, Schultz Creek, 4 miles north of Centreville; NMNH; ♂; ♀]. —Harris et al. 1991: 264 [distribution].

**Distribution.** —U.S.A.

***celtikci*** Çakin, 1983: 236 [type locality: [Turkey], Ankara, Kızılcahamam, Çeltikçi; HUAT; ♂]. —Sipahiler and Malicky 1987: 122, 143 [distribution]. —Malicky 1983b: 61 [atlas; ♂; ♀]. —Malicky 2004a: 73 [atlas]. —Sipahiler 2005: 398 [distribution]. —Malicky 2005b: 548 [checklist].

**Distribution.** —Turkey.

***danra*** Oláh & Johanson, 2010a: 87 [type locality: Vietnam, Lamdong Province, Baoloc, Duchma stream; Collection Oláh; ♂].

**Distribution.** —Vietnam.

***delira*** (Ross, 1938a): 115 [type locality: [United States], Wisconsin, Spooner, along Namakagon River; INHS; ♂; in *Stactobia*]. —Ross 1944: 124 [in *Tascobia*]. —Denning 1947a: 145 [distribution]. —Morse and Blickle 1953: 72 [distribution]. —Etnier 1965: 148 [distribution]. —Etnier and Schuster 1979: 18 [distribution]. —Blickle 1979: 55, 61 [checklist; ♂]. —Swegman and Ferrington 1980: 288 [distribution]. —Parker and Voshell 1981: 4 [distribution]. —Huryn and Foote 1983: 791 [distribution]. —Waltz and McCafferty 1983a: 12 [distribution]. —Hamilton et al. 1983: 19 [distribution]. —Bowles and Mathis 1989: 240 [distribution]. —Morse et al. 1989: 25 [distribution]. —Tarter 1990: 239 [distribution]. —Floyd and Schuster 1990: 130, 132 [distribution]. —Frazer et al. 1991: 20 [distribution]. —Harris et al. 1991: 265 [distribution]. —Masteller and Flint 1992: 70 [distribution]. —Mathis and Bowles 1992: 24 [distribution]. —Bowles and Mathis 1992: 32 [distribution]. —Floyd and Morse 1993: 177 [distribution]. —Moulton and Stewart 1996: 131 [♂; distribution]. —Wiggins and Parker 1997: 794 [distribution]. —Floyd et al. 1997: 136 [distribution]. USA —Huryn and Harris 2000: 193 [distribution]. —Houghton et al. 2001: 505 [distribution]. —Harris et al. 2002c: 59 [♂]. —DeWalt and Heinold 2005: 42 [phenology; distribution]. —Zack et al. 2006: 134 [phenology; distribution]. —Etnier 2010: 486 [distribution]. —Armitage et al. 2011: 15 [distribution]. —Myers et al. 2011: 110 [distribution]. —Blinn and Ruiter 2013: 291 [biology; distribution]. —Blinn and Ruiter 2013: 279 [biology; distribution]. —Houghton et al. 2017: 63 [checklist]. —Mendez et al. 2019: 119 [checklist]. —Bowles et al. 2020: 8 [distribution].

**Distribution.** —Canada, U.S.A.

***eatoni*** Gibon, 2019: 178 [type locality: Madagascar, bassin de la Betsiboka, tributaire vers Ambalambongo, 16°48'00"S - 47°00'30"E, 48 m; CBGP; ♂].

**Distribution.** —Madagascar.

***kamoro*** Gibon, 2019: 179 [type locality: Madagascar, bassin de la Betsiboka, Kamoro à Ambohimanatrika, 16°28'55"S - 47°10'06"E, 40 m; CBGP; ♂].

**Distribution.** —Madagascar.

***kumejima*** Ito, 2020: 567 [type locality: Japan, Ryukyu, Kume-jima, Kumejima-cho, Shirase-gawa, middle reach, 28.351667N, 129.766944E, 32 m above sea level; CBM-ZI; ♂; ♀].

**Distribution.** —Japan.

***lanceolata*** Gibon, 2019: 180 [type locality: Madagascar, bassin de la Namorona, Namorona vers Ambahona, 21°35'20"S - 48°07'21"E, 20 m; CBGP; ♂].

**Distribution.** —Madagascar.

***marshallae*** Gibon, 2019: 180 [type locality: Madagascar, bassin du Mangoro, Sahamarirana vers Antsily, 19°00'57"S - 48°07'18"E, 860 m; CBGP; ♂].

**Distribution.** —Madagascar.

***martynovi*** Blickle & Denning, 1977: 298 [type locality: [United States], Tennessee, Greenbriar Cove, entrance Great Smokey Mountain; FSCA; ♂]. —Etnier and Schuster 1979: 18 [distribution]. —Blickle 1979: 55, 61 [checklist; ♂]. —Parker and Voshell 1981: 4 [checklist]. —Harris et al. 1984: 109 [distribution]. —Morse et al. 1989: 25 [distribution]. —Harris et al. 1991: 267 [distribution]. —Masteller and Flint 1992: 70 [checklist]. —Houp 1999: 2 [distribution]. —Floyd et al. 1997: 136 [distribution]. USA —DeWalt and Heinold 2005: 42 [phenology; distribution]. —Myers et al. 2011: 110 [distribution]. —Armitage et al. 2011: 15 [distribution].

—*solzhenitsyni* Sykora & Weaver, 1978: 2 [type locality: [United States], Pennsylvania, Whiteoak Run near Holland House, Powdermill Nature Reserve, Westmoreland County; CMNH; ♂]. —Weaver 1990: 360 [to synonymy].

**Distribution.** —U.S.A.

***mutica*** Zhou, Yang, & Morse, 2016: 211 [type locality: China, Si-chuan Province, Ma-bian County, Tian-xing village, Zhong-shan-gou stream, 4.9 km W of bridge in Ma-bian, 28.8492°N, 103.5091°E, 597 m; NAUJ; ♂].

**Distribution.** —China.

***nikulinae*** Arefina, 2004: 210 [type locality: Russia, Primorye, Ilistaya River at Lyalichi Villave, Khanka Lake basin; IBSS-RAS; ♂]. —Ivanov 2011: 196 [checklist]. —Potikha and Vshivkova 2016: 364 [distribution].

**Distribution.** —Russia.

***palmata*** (Ross, 1938a): 116 [type locality: [United States], Wisconsin, Merrill, along Wisconsin River; INHS; ♂; in *Stactobia*]. —Ross 1944: 125 [♂; ♀; distribution; in *Tascobia*]. —Morse and Blickle 1953 [72 [distribution]. —Etnier 1968: 191 [distribution]. —Etnier and Schuster 1979: 18 [distribution]. —Blickle 1979: 55, 61 [checklist; ♂]. —Parker and Voshell 1981: 4 [distribution]. —Huryn and Foote 1983: 791 [distribution]. —Hamilton et al. 1983: 19 [distribution]. —Steven and Hilsenhoff 1984: 164 [distribution]. —Floyd and Schuster 1990: 130, 132 [distribution]. —Frazer et al. 1991: 20 [distribution]. —Harris et al. 1991: 267 [distribution]. —Masteller and Flint 1992: 70 [distribution]. —Bowles and Mathis 1992: 32 [distribution]. —Moulton and Stewart 1996: 1131 [♂; distribution]. —Houghton et al. 2001: 505 [distribution]. —Biondi 2010: 61 [distribution; as *palmate*]. —Etnier 2010: 486 [distribution]. —Armitage et al. 2011: 15 [distribution]. —Myers et al. 2011: 110 [distribution]. —Houghton et al. 2017: 63 [checklist]. —Bowles et al. 2020: 8 [distribution].

**Distribution.** —U.S.A.

***parallelica*** Zhou, Yang, & Morse, 2016: 211 [type locality: China, Jiang-xi Province, Jiu-Lian-shan Mt. National Nature Reserve, at the confluence of Huang-niu-shi & Da-shui-keng Streams, 1.2 km SE of Dun-tou Village, 24.31°N, 114.25°E, 546 m; NAUJ; ♂].

**Distribution.** —China.

***risi*** (Felber, 1908): 720 [type locality: [Switzerland] Rheinufer in der Stadt Basel; possibly deposited in MZHF; ♂; in *Microptila*]. —Ulmer 1929: 253 [to *Stactobiella*]. —Botosaneanu 1967: 293 [distribution]. —Botosaneanu and Malicky 1978: 340 [checklist]. —Kumanski and Malicky 1984: 199 [distribution]. —Kumanski 1985: 110 [♂]. —Andersen and Wiberg-Larsen 1987: 169 [checklist]. —Usseglio-Polatera and Bournaud 1989: 253 [distribution]. —Nógrádi 1994: 271 [distribution ♂ ♀]. —Uherkovich and Nógrádi 1997: 461 [distribution]. —Graf et al. 1998: 207 [distribution]. —Uherkovich and Nógrádi 1998: 52 [distribution]. —Malicky 1999f: 32 [distribution]. —Graf and Waringer 2002: 420 [larva; distribution]. —Ujvárosi 2002: 384 [distribution]. —Gullefors 2002: 138 [checklist]. —Malicky 2004a: 73 [atlas]. —Malicky 2005b: 548 [checklist]. —Malicky 2005a: 69 [distribution]. —Lubini-Ferlin and Vicentini 2005: 68 [checklist]. —Gullefors 2006: 137 [distribution]. —Mey 2006a: 159 [distribution]. —Gullefors 2008: 64 [distribution]. —Ujvárosi et al. 2008: 113 [checklist]. —Višinskienė 2009: 28 [checklist]. —González and Menéndez 2011: 119 [distribution]. —Waringer and Graf 2011: 281 [larval synopsis]. —Gullefors 2016: 156 [checklist].

—*ulmeri* (Siltala, 1908): 14 [type locality: [Finland] Keminjoki, Tervola; depository not designated; ♂; in *Stactobia*]. —Martynov 1924: 58 [♂; ♀; to *Stactobiella*]. —Martynov 1934: 157 [♂]. —Racięcka 1936: 98 [distribution]. —Nybom 1960: 20 [distribution]. —Botosaneanu 1967: 293 [distribution]. —Botosaneanu and Malicky 1978: 340 [to synonymy]. —Kumanski 1979: 4 [♂; distribution]. —Spuris 1989: 18 [as senior synonym of *S.risi*]. — Cibaitė 2003a: 10 [distribution]. —Robert 2007: 82 [checklist]. —Ivanov 2011: 196 [checklist].

**Distribution.** —Austria, Bulgaria, Finland, France, Germany, Greece, Italy, Hungary, Lithuania, Macedonia, Portugal, Romania, Spain, Sweden, Switzerland.

***siribhum*** Malicky & Chantaramongkol, 2007: 1054 [type locality: Thailand, Doi Inthanon NP, Namtok Siribhum, 18°32'N 98°31'E, 1300 m; ♂]. —Malicky 2010a: 56 [atlas; ♂].

**Distribution.** —Thailand.

***tshistjakovi*** (Arefina & Morse) in Arefina et al. 2002: 103 [type locality: [Russia] Southern Primorye Territory, Khasansky Region, Amba River; IBSS-RAS; ♂; in *Stactobia*]. —Arefina and Armitage 2003: 17 [distribution]. —Arefina 2004: 209 [to *Stactobiella*]. —Ivanov 2011: 548 [checklist]. —Tanida and Kuranishi 2016: 73 [distribution]. —Potikha and Vshivkova 2016: 364 [distribution]. —Ito 2020: 562 [♂; ♀; distribution].

**Distribution.** —Japan, Russia.

#### Genus *Tizatetrichia* Harris, Flint, & Holzenthal, 2002

*Tizatetrichia* Harris, Flint, & Holzenthal, 2002b: 55 [type species: *Tizatetrichiacostaricensis* Harris, Flint, & Holzenthal, 2002b, original designation].

*Tizatetrichia* contains two species occurring in Central America. The female and larva are unknown. Based on similarities occurring in the male genitalia, the genus is most closely related to *Bredinia* (Harris et al., 2002b).

***costaricensis*** Harris, Flint, & Holzenthal, 2002b: 58 [type locality: Costa Rica, Guanacaste, Río Tizate, 7.2 km NE Cañas Dulces, 10.773°N, 85.449°W, 275 m; NMNH; ♂].

**Distribution.** —Costa Rica.

***panamensis*** Harris & Armitage, 2019: 18 [type locality: Panama, Bocas del Toro Province, Quebrada Rambala, near Rambala Jungle Lodge, 3.74 km SSE Rambala, 8.91543°N and 82.15527°W, 120 m; COZEM; ♂]. —Armitage and Harris 2020a: 8 [distribution].

**Distribution.** —Panama.

### ﻿HYDROPTILIDAE incertae sedis

#### Genus *Burminoptila* Botosaneanu, 1981 ♰

*Burminoptila* Botosaneanu, 1981a: 75 [type species: *Burminoptilabemeneha* ♰ Botosaneanu, 1981a, original designation].

The genus *Burminoptila* is represented by a single fossil species known from Burmese amber. No further information regarding diagnostic features or placement of the genus within Hydroptilidae was provided.

♰ ***bemeneha*** Botosaneanu, 1981a: 77 [type locality: [Myanmar] Burma; NHMUK; ♂; in amber]. —Eskov et al. 2008: 78 [checklist]. —Ivanov and Melnitsky 2017: 131 [checklist].

**Distribution.** —Burmese amber.

#### Genus *Dicaminus* Müller, 1879

*Dicaminus* Müller, 1879b: 39 [type species: no included species, but *Dicaminus* replaced *Diaulus* and thus received its type species, *ladislavii*]. —Ulmer 1957: 172 [references]. —Marshall 1979b: 220 [generic review].

*Diaulus*Müller 1879a: 142 [type species: *Diaulusladislavii* Müller, 1879a, monotypic]. —Ulmer 1957: 173 [to synonymy].

*Dicaminus* consists of a single species occurring in South America. Müller (1879b) described several atypical larval cases with small dorsal chimneys under the generic name *Dicaminus*. The material was from Brazil, but neither a larval description nor a specific epithet was provided. He then subsequently made reference to these same cases under the name *Diaulusladislavii* (Müller 1879a). *Dialus* was later synonymized with *Dicaminus* (Ulmer 1957). A number of cases with dorsal chimneys have been found in material from Argentina, Bolivia, Ecuador, Panama, and Venezuela (Botosaneanu and Flint 1982). Some of these contain male metamorphotypes of *Metrichia* spp., which suggests that *Dicaminus* may prove to be either synonymous with or closely related to *Metrichia* (Flint et al. 1999b). Neither the adult nor the larval stage has been described.

***ladislavii*** Müller, 1879a: 142 [type locality: South Brazil; type depository unknown; case]. —Muller 1880: 118 [case; figures, type locality: [Brazil], Santa Catarina, Ribeirão dos Bugres, tributary of Itajahy]. —Ulmer 1957: 172 [complete references]. —Angrisano 1999: 32 [checklist]. —Paprocki et al. 2004: 11 [checklist]. —Paprocki and França 2014: 44 [checklist].

**Distribution.** —Brazil.

#### Genus *Electrotrichia* Ulmer, 1912 ♰

*Electrotrichia* Ulmer, 1912a: 42 [type species: *Electrotrichiasubtilis* ♰ Ulmer, 1912a, monotypic]. —Marshall 1979b: 222 [generic review].

The genus *Electrotrichia* is represented by a single fossil species known from Baltic amber. Marshall (1979b) stated that the genus may share similarities with members of the Hydroptilinae in the wing shape and spur formula.

♰ ***subtilis*** Ulmer, 1912a: 43 [type locality: [Baltic region]; holotype missing, originally deposited in “Klebs collection” (x 64); ♂; in amber]. —Eskov et al. 2008: 78 [checklist]. —Wichard 2013: 50 [species review].

**Distribution.** —Baltic amber.

#### Genus *Macrostactobia* Schmid, 1958

*Macrostactobia* Schmid, 1958b: 46 [type species: *Macrostactobiaelawalikanda* Schmid, 1958b, original designation]. —Marshall 1979b: 217 [generic review]. —Wells and Huisman 1992: 94 [larva].

*Macrostactobia* consists of two species recorded from Sri Lanka and West Malaysia. Schmid (1958b) stated that, due to its relatively larger size and complete wing venation, the genus is somewhat primitive. He placed it in a branch of Stactobiinae that also included the genera *Parastactobia*, *Plethus*, and *Chrysotrichia*. Marshall (1979b) noted that the male genitalia are unique and that the antennae are typical of Stactobiinae, but declined to place the genus, leaving it incertae sedis. The final instar larva of *M.runcing* was described by Wells and Huisman (1992).

***elawalikanda*** Schmid, 1958b: 47 [type locality: [Sri Lanka], Ceylan, Horton Plains (C. P., 7000 ft) 7-8-III, partie supérieure de la Beliul Oya, ruisseau calme, assez profond, à fond boueux, formant des <<pools>>; depository not designated; ♂].

**Distribution.** —Sri Lanka.

***runcing*** Wells & Huisman, 1992: 94 [type locality: West Malaysia, Cameron Highlands, falls at “40 mile”, on road between Tapah and Tanah Rata; NTM; ♂; ♀; larva; pupa]. —Malicky 2010a: 38 [atlas; ♂].

**Distribution.** —Malaysia.

#### Genus *Novajerseya* Botosaneanu, Johnson, & Dillon, 1998 ♰

*Novajerseya* Botosaneanu, Johnson, & Dillon, 1998: 225 [type species: *Novajerseyaglesumica* ♰ Botosaneanu, Johnson, & Dillon, 1998, original designation].

The genus *Novajerseya* is represented by a single fossil species known from Upper Cretaceous amber found in New Jersey. No further information regarding diagnostic features or placement of the genus within Hydroptilidae was provided.

♰ ***glesumica*** Botosaneanu, Johnson, & Dillon, 1998: 225 [type locality: United States, New Jersey; ANSP; ♂; in amber]. —Wichard and Lüer 2003: 132 [checklist]. —Eskov et al. 2008: 78 [checklist]. —Sukatsheva and Vassilenko 2016: 411 [wing venation]. —Ivanov and Melnitsky 2017: 131 [checklist].

**Distribution.** —New Jersey amber.

#### Genus *Orphninotrichia* Mosely, 1934

*Orphninotrichia* Mosely, 1934a: 138 [type species: *Orphninotrichiamaculata* Mosely, 1934a, original designation]. —Marshall 1979b: 220 [generic review]. —Wells 1980: 628 [revision; key to males]. —Wells 1985b: 19 [larva; pupa; case]. —Wells 1997: 1–28 [checklist; key to larvae]. —Wells 1999: 221 [new species; zoogeography; new records]. Wells 2002a: 224 [key to males]. —Wells 2010a: 48 [re-description].

The genus *Orphninotrichia* consists of 20 species occurring in Australia. The genus was established on the basis of unique wing venation and unique male genitalia (Mosely 1934a). Marshall (1979b) left the genus as incertae sedis, but did comment on possible affinities with Hydroptilinae and noted ways in which *Orphninotrichia* differs from the genera *Hydroptila* and *Oxyethira*. Wells (1987) also considered the genus to belong to Hydroptilinae. Holzenthal et al. (2007b), based on the World Trichoptera Checklist, treated the genus as incertae sedis. The larvae of *O.maculata* was described by Wells (1985b).

***acta*** Neboiss, 1977: 40 [type locality: [Australia] Tasmania, Ulverstone, 4 km NW waterfalls; NMV; ♂; ♀]. —Wells 1980: 632 [♂, ♀; distribution]. —Neboiss 1986: 66 [atlas; ♂; ♀]. —Neboiss 2002: 52 [checklist].

**Distribution.** —Australia.

***alata*** Wells, 2010a: 50 [type locality: [Australia] Queensland, Crystal Cascades, Tributary of Crystal Creek, 16.96508S 145.67603E ± 2 m, 69 m asl; QM; ♂].

**Distribution.** —Australia.

***barbarae*** Wells, 2010a: 48 [type locality: [Australia] Queensland, Kuranda (Top of the Range), 16°48'S 145°38'E (335 m), 19 Butler Drive; ANIC; ♂].

**Distribution.** —Australia.

***benambrica*** Wells, 1983: 646 [type locality: Australia, Victoria, Benambra Creek, 25 km NE. Benambra at granite falls; NMV; ♂; ♀]. —Wells 1985b: 21 [pupa]. —Neboiss 1986: 65 [atlas; ♂; ♀].

**Distribution.** —Australia.

***bilobata*** Wells, 2002a: 222 [type locality: [Australia] New South Wales, Chichester State Forest, Dundungra Falls; ANIC; ♂].

**Distribution.** —Australia.

***claviculata*** Wells, 2002a: 222 [type locality: [Australia] New South Wales, Chichester State Forest, Dundungra Falls; ANIC; ♂].

**Distribution.** —Australia.

***desleyae*** Wells, 2010a: 49 [type locality: [Australia] Queensland, Crystal Cascades, Tributary of Crystal Creek, 16.96508S 145.67603E ± 2 m, 69 m asl; QM; ♂].

**Distribution.** —Australia.

***dundungra*** Wells, 2002a: 222 [type locality: [Australia] New South Wales, Chichester State Forest, Dundungra Falls; ANIC; ♂].

**Distribution.** —Australia.

***gilva*** Wells, 1999: 226 [type locality: [Australia, Lord Howe Island] Erskine Creek, Erskine Valley; ANIC; ♂; ♀].

**Distribution.** —Australia.

***justini*** Wells, 1983: 645 [type locality: Australia, Victoria, Stevenson Falls, Upper Gellibrand R.; NMV; ♂; ♀]. —Neboiss, 1986: 67 [atlas; ♂; ♀].

**Distribution.** —Australia.

***maculata*** Mosely, 1934a: 139 [type locality: [Australia] Hornsby, New South Wales; Collection Tillyard (transferred to NHMUK according to Wells 1980: 630); ♂]. —Mosely and Kimmins 1953: 511 [♂]. —Wells 1980: 630 [♂; ♀; distribution]. —Wells 1985b: 21 [larva; pupa; case]. —Neboiss 1986: 65 [atlas; ♂; ♀]. —Neboiss 2002: 52 [checklist].

**Distribution.** —Australia.

***media*** Wells, 1980: 632 [type locality: [Australia] Victoria, Porepunkah; NMV; ♂]. —Neboiss 1986: 66 [atlas; ♂].

**Distribution.** —Australia.

***originis*** Wells, 1990c: 111 [type locality: [Australia] Northern Territory, Kakadu National Park, Radon Springs, 12°45'S 132°55'E; NTM; ♂].

**Distribution.** —Australia.

***papillata*** Wells, 1980: 635 [type locality: [Australia] Victoria, Tawonga; NMV; ♂; ♀]. —Neboiss, 1986: 67 [atlas; ♂; ♀]. —Wells 2002a: 224 [♂; distribution].

**Distribution.** —Australia.

***plumosa*** Wells, 1999: 224 [type locality: [Australia, Lord Howe Island] Erskine Creek, Erskine Valley; ANIC; ♂; ♀; case].

**Distribution.** —Australia.

***regia*** Wells, 1980: 632 [type locality: [Australia] Victoria, Kinglake; NMV; ♂; ♀]. —Neboiss 1986: 65 [atlas; ♂; ♀].

**Distribution.** —Australia.

***rugosa*** Wells, 1999: 226 [type locality: [Australia, Lord Howe Island] Erskine Creek, Erskine Valley; ANIC; ♂; ♀; case].

**Distribution.** —Australia.

***silicis*** Wells, 1980: 635 [type locality: [Australia] North Queensland, Tinaroo Lake Road, Stream at M4; NMV; ♂]. —Neboiss 1986: 66 [atlas; ♂]. —Wells 2010a: 50 [distribution].

**Distribution.** —Australia.

***squamosa*** Wells, 1999: 228 [type locality: [Australia, Lord Howe Island] Erskine Creek, Erskine Valley; ANIC; ♂; ♀; case].

**Distribution.** —Australia.

***subulata*** Wells, 1983: 647 [type locality: Australia, New South Wales, Undercliffe Falls, 12 km E. Liston; NMV; ♂]. —Wells 1985b: 21 [pupa]. —Neboiss 1986: 66 [atlas; ♂].

**Distribution.** —Australia.

### ﻿Family PTILOCOLEPIDAE Martynov, 1913

Palaeagapetinae Ross, 1956: 18 [type genus: *Palaeagapetus* Ulmer, 1912a, as *Paleagapetus*].

Ptilocolepinae Martynov, 1913a: 22 [type genus: *Ptilocolepus* Kolenati, 1848]. —Marshall 1979b: 157 [reviewed as subfamily of Hydroptilidae]. —Ito 1998: 85 [world distribution; biology; recent and fossil taxa]. —Malicky 2001b: 20 [elevated from subfamily of Hydroptilidae]. —Malicky 2005b: 542 [confirmed as distinct from Hydroptilidae]. —Malicky 2008b: 43 [family status discussed with respect to work of Thienemann 1904a].

The family Ptilocolepidae contains two small genera known to have a Holarctic distribution. The adults bear a resemblance to some of the smaller members of the caddisfly family Glossosomatidae, while the larval stage indicates an affinity with Hydroptilidae. Males have highly specialized genitalia that are characteristic of the group (Marshall 1979b). The larvae of both genera can be found in small montane springs on vegetation, stones, or other submerged surfaces and are often found in association with bryophytes (Ito 1998).

#### Genus *Palaeagapetus* Ulmer, 1912

*Palaeagapetus* Ulmer, 1912a: 35 [type species: *Palaeagapetusrotundatus* Ulmer, 1912a, monotypic]. —Marshall 1979b: 160 [generic review]. —Blickle 1979: 8 [key to species of America north of Mexico].

Eleven species of *Palaeagapetus*, including two fossil species, occur in a mostly East Palearctic distribution, with a few distributed across the Nearctic faunal region. Characters that unite the genus can be found in features of the wing venation, spur formula, and the male genitalia (Marshall, 1979b). The larvae of *P.celsus* were described by Flint (1962).

***celsus*** (Ross, 1938a): 111 [type locality: United States, North Carolina, Newfound Gap, along Little Pigeon River; INHS; ♂; in *Paragapetus*]. —Roy and Harper 1975: 1082 [distribution]. —Roy and Harper 1979: 150 [checklist]. —Etnier and Schuster 1979: 18 [checklist]. —Blickle 1979: 55, 61 [checklist; ♂]. —Parker and Voshell 1981: 4 [checklist]. —Tarter 1990: 239 [checklist]. —Masteller and Flint 1992: 70 [checklist]. —Bowles and Mathis 1992: 32 [distribution]. —DeWalt and Heinold 2005: 42 [phenology; distribution]. —Myers et al. 2011: 110 [distribution]. —Ito et al. 2014: 211 [♂; ♀; biology; distribution].

**Distribution.** —Canada, U.S.A.

***finisorientis*** Botosaneanu & Levanidova, 1987: 43 [type locality: U.S.S.R., Vodopadny spring, bassin of Kedrovaya River, “Kedrovaya Pad” Nature Reserve, Vladyvostok district; ZIN; ♂]. —Spuris 1989: 18 [checklist]. —Ito and Vshivkova 1999: 141 [♂; ♀, pupa; larva; egg; biology; distribution]. —Ivanov 2011: 194 [distribution].

**Distribution.** —Russia.

***flexus*** Ito, 1991b: 419 [type locality: Japan, Creek Kumanosawa, Takaoka, Tomakomai; EIHU; ♂]. —Ito et al. 1993: 143 [checklist]. —Ito et al. 1997: 100 [distribution]. —Ito 1998: 85 [distribution; biology]. —Minakawa et al. 2004: 51 [distribution]. —Tanida et al. 2005: 441 [♂]. —Ivanov 2011: 194 [distribution]. —Tanida and Kuranishi 2016: 74 [checklist].

**Distribution.** —Japan, Russia.

***fukuiensis*** Ito, 2010: 1 [type locality: Japan, Fukui Prefecture, Katsuyama-shi, Akausagi-yama, small stream, 36°04'N, 136°39'E, 1,300 m; CBM-ZI; ♂; ♀; larva]. —Tanida and Kuranishi 2016: 74 [checklist].

**Distribution.** —Japan.

♰ ***furcilla*** Botosaneanu, Johnson, & Dillon, 1998: 220 [type locality: United States, New Jersey; ANSP; ♂; in amber]. —Wichard and Lüer 2003: 132 [checklist]. —Eskov et al. 2008: 78 [checklist].

**Distribution.** —New Jersey amber.

***kyushuensis*** Ito & Kuhara in Ito et al. 1997: 101 [type locality: Japan, a brooklet in Hikosan Biological Station of Kyushu University, Hikosan, Soeda-cho, Fukuoka; CBM-ZI; ♂]. —Ito 1998: 85 [distribution; biology]. —Tanida et al. 2005: 441 [♂]. —Tanida and Kuranishi 2016: 74 [checklist].

**Distribution.** —Japan.

***nearcticus*** Banks, 1936: 265 [type locality: United States, Washington, White River, Mt. Ranier; MCZ; ♂]. —Blickle 1979: 55, 61 [checklist; ♂]. —Ito et al. 2014: 202 [♂; ♀; pupa; larva; biology; distribution]. —Mendez et al. 2019: 119 [checklist].

—*guppyi* Schmid, 1951: 1 [type locality: [Canada] Mt. Benson; depository not designated; ♂]. —Blickle 1979: 55, 61 [checklist; ♂]. —Botosaneanu and Levanidova 1987: 43 [treated as a synonym]. —Djernaes and Sperling 2011: 86 [abdominal sternum glands].

**Distribution.** —Canada, U.S.A.

***ovatus*** Ito & Hattori, 1986: 143 [type locality: Japan, Rankoshi, Chitose, Hokkaido; EIHU; ♂; ♀; pupa; larva; egg]. —Ito 1988: 148 [life history]. —Ito et al. 1993: 143 [checklist]. —Ito et al. 1997: 98 [♂; distribution]. —Ito 1997: 177 [oviposition site]. —Ito 1998: 85 [distribution, biology]. —Ito 1988: 148 [life history]. —Tanida et al. 2005: 441 [larva; ♂]. —Tanida and Kuranishi 2016: 74 [checklist].

**Distribution.** —Japan.

***parvus*** Ito, 1991a: 359 [type locality: Japan, small stream near Shindai-goya, Mt. Hyonosen, Hyogo Prefecture; EIHU; ♂; ♀; pupa; larva; egg]. —Ito et al. 1993: 143 [checklist]. —Ito et al. 1997: 99 [♂; distribution]. —Tanida et al. 2005: 441 [♂]. —Mey and Nozaki 2006: 24 [distribution]. —Tanida and Kuranishi 2016: 74 [checklist].

**Distribution.** —Japan.

♰ ***rotundatus*** Ulmer, 1912a: 36 [type locality: [Baltic region]; holotype missing, originally deposited in “Klebs collection” (no. 5690); ♂; in amber]. —Eskov et al. 2008: 78 [checklist]. —Wichard 2013: 53 [species review].

**Distribution.** —Baltic amber.

***shikokuensis*** Utsunomiya & Ito in Ito et al. 1997: 103 [type locality: Japan, Sagawa, Shigenobu-cho, Ehime; CBM-ZI; ♂]. —Ito et al. 2002: 21 [biology]. —Tanida et al. 2005: 441 [♂]. —Tanida and Kuranishi 2016: 74 [checklist].

**Distribution.** —Japan.

#### Genus *Ptilocolepus* Kolenati, 1848

*Ptilocolepus* Kolenati, 1848: 102 [type species: *Ptilocolepusturbidus* Kolenati, 1848, monotypic]. —Fischer 1961: 80 [*P.turbidus* synonymized with *Rhyacophilagranulatus* Pictet, 1834]. —Marshall 1979b: 160 [generic review]. —Kachalova in Medvedev 1998: 191 [key to the species of the European part of the USSR]. —Malicky 2001b: 20 [taxonomic notes].

This genus contains eight species recorded in a Palearctic distribution and, at different times, has been placed in the families Rhyacophilidae, Hydroptilidae, and Glossosomatidae (Pictet 1834; Ulmer 1907; Martynov 1913b; Malicky 1983b). Features used to identify members of *Ptilocolepus* include wing venation and the general structure of the male genitalia (Marshall 1979. The larvae of *P.granulatus* have been described by Thienemann (1904a) and Jacquemart and Coineau (1962).

***atiloma*** Schmid, 1990: 239 [type locality: [India] Inde, Assam, United Jaintia and Khasi Hills, Mawpran; CNC; ♂].

**Distribution.** —India.

***colchicus*** Martynov, 1913a: 26 [type locality: [Georgia]; depository not designated; ♂]. —Martynov 1913b: 10 [♀]. —Schmid 1913: 10 [♀; distribution]. —Martynov 1934: 114 [♂]. —Schmid 1959b: 684 [distribution]. —Botosaneanu 1967: 293 [distribution]. —Botosaneanu and Malicky 1978: 339 [checklist]. —Kumanski 1980: 38 [distribution]. —Sipahiler and Malicky 1987: 112, 135 [distribution]. —Spuris 1989: 18 [checklist]. —Mirmoayedi and Malicky 2002: 164 [distribution]. —Malicky 2004a: 50 [atlas]. —Malicky 2005b: 542 [checklist]. —Sipahiler 2005: 396 [distribution]. —Sipahiler 2007: 38 [distribution]. —Sipahiler 2008: 93 [distribution]. —Sipahiler 2008: 103 [checklist]. —Ivanov 2011: 194 [distribution]. —Sipahiler 2012a: 7 [distribution]. —Sipahiler 2016: 13 [distribution]. —Sipahiler 2017a: 10 [note on morphological malformation]. —Küçükbasmaci and Kiyak 2017: 488 [distribution]. —Oláh et al. 2020: 46 [distribution].

**Distribution.** —Georgia, Iran, Russia, Turkey.

***dilatatus dilatatus*** Martynov, 1913a: 23 [type locality: [Georgia]; depository not designated; ♂]. —Martynov 1934: 113 [♂]. —Botosaneanu 1967: 293 [as synonym of *P.granulatus*]. —Botosaneanu and Malicky 1978: 339 [as junior synonym of *P.granulatus*; checklist]. —Kumanski 1980: 38 [♀; distribution]. —Spuris 1989: 18 [checklist]. —Malicky 2004a: 50 [atlas]. —Sipahiler 2005: 396 [distribution; as *granulatus dilatatus*]. —Malicky 2005b: 542 [checklist; treated as distinct species]. —Sipahiler 2007: 38 [distribution]. —Sipahiler 2008: 93 [distribution]. —Sipahiler 2008: 103 [checklist]. —Ivanov 2011: 194 [distribution]. —Sipahiler 2012a: 7 [distribution]. —Sipahiler 2016: 13 [distribution]. —Oláh et al. 2020: 46 [distribution].

**Distribution.** —Georgia, Russia, Turkey.

***dilatatus minor*** Martynov, 1913b: 9 [type locality: [Georgia], Caucase, la province de Batoum et des environs du Novyj Afon; depository not designated; ♂]. —Martynov 1934: 113 [morphological note; distribution].

**Distribution.** —Georgia, Russia.

***extensus*** McLachlan, 1884: 70 [type locality: Portugal, Beira Baixa; depository not designated; ♂]. —Schmid 1952: 649 [distribution]. —Botosaneanu 1967: 293 [distribution]. —Botosaneanu and Malicky 1978: 339 [checklist]. —González et al. 2000: 27 [larva; case; pupa; biology; distribution]. —Malicky 2004a: 50 [atlas]. —Malicky 2005b: 542 [checklist]. —González and Menéndez 2011: 118 [distribution]. —Martín et al. 2014: 67 [distribution]. —Martínez et al. 2015: 40 [distribution]. —Martín et al. 2016: 261 [distribution].

**Distribution.** —Portugal, Spain.

***granulatus*** (Pictet, 1834): 197 [type locality: type locality not given; depository not designated; ♂]. —Mclachlan 1884: 69 [distribution]. —Ris 1897: 431 [distribution; as *grannulatus*]. —Ris 1903: 16 [distribution]. —Thienemann 1904a: 418 [larva]. —Schmid 1952: 649 [distribution]. —Jacquemart and Coineau 1962: 7 [larva]. —Botosaneanu 1967: 293 [distribution]. —Botosaneanu and Malicky 1978: 339 [checklist]. —Moretti and Cianficconi 1981: 200 [checklist]. —Kumanski 1985: 106 [♂]. —Nógrádi 1986: 135 [distribution; ♂; ♀]. —Sipahiler and Malicky 1987: 112, 135 [distribution]. —Spuris 1989: 18 [checklist]. —Wiberg-Larsen et al. 1991: 45 [distribution]. —Kahnert 1995: 124 [distribution]. —Ito 1998: 85 [distribution biology]. —Cianficconi et al. 1999: 57 [distribution]. —Waringer and Graf 2002: 121 [ecology morphology; distribution]. —Malicky 2004a: 50 [atlas]. —Cianficconi et al. 2004: 329 [distribution]. —Cianficconi et al. 2005: 96 [habitat; distribution]. —Malicky 2005b: 542 [checklist]. —Graf et al. 2005: 55 [distribution]. —Lubini-Ferlin and Vicentini 2005: 68 [checklist]. —Robert 2007: 82 [checklist]. —Szczesny and Godunko 2008: 14 [distribution]. —Chvojka and Komzák 2008: 13 [distribution]. —Schrankel et al. 2008: 90 [checklist]. —Chvojka et al. 2009: 82 [distribution]. —Menéndez and González 2010: 341 [distribution]. —Oláh 2010: 91 [distribution]. —González and Menéndez 2011: 118 [distribution]. —Coppa 2013: 123–132 [distribution]. —Mey 2014: 184, 187 [distribution]. —Martín et al. 2016: 261 [distribution]. —Valle and Lodovici 2018: 146 [distribution].

—*funereus* Kolenati, 1859: 203 [type locality: unknown; probably NHMW; probably ♂]. —Fischer 1961: 80 [listed as a synonym].

—*turbidus* Kolenati, 1848: 102 [type locality: [Germany]; probably NHMW; probably ♂]. —Fischer 1961: 80 [listed as a synonym].

**Distribution.** —Austria, Belgium, Czech Republic, England, Denmark, France, Germany, Hungary, Italy, Luxembourg, Spain, Switzerland, Turkey, Ukraine.

***namnao*** Malicky & Chantaramongkol, 1996: 119 [type locality: [Thailand] Nam Nao NP, 800 m; Collection Malicky; ♂].

**Distribution.** —Thailand.

***villosus*** Navás, 1916: 83 [type locality: [Spain] Aragón; depository not designated; ♂; ♀]. —Malicky, 2005b: 542 [checklist].

**Distribution.** —Spain.

### ﻿TRICHOPTERA incertae sedis

#### Genus *Eutonella* Müller, 1921

*Eutonella* Müller, 1921: 531 [type species: *Eutonellapeltopsychoides* Müller, 1921, monotypic]. —Ulmer 1957: 316 [systematic placement]. —Santos et al. 2016a: 460 [systematic placement].

*Eutonella* is a monotypic genus of unplaced systematic position within the order Trichoptera recorded from South America. Only the figure of a pupal mandible is known for the genus, and it could be placed in either Hydroptilidae or Psychomyiidae (Flint et al. 1999b). The mandible lacks teeth or serrations, a state that Müller (1921) concluded was only exhibited by microcaddisflies, placing it in Hydroptilidae (Flint et al. 1999b). Ulmer (1957) associated the mandible with a series of unnamed cases from Müller’s earlier works; the descriptions of the cases led Flint et al. (1999b) to place *Eutonella* in Leucotrichiinae. Based on the 2-4-4 tibial spur formula indicated by Müller (1880a, 1880b), which is inconsistent with that of any other hydroptilid, Santos et al. (2016a) placed the species in Trichoptera incertae sedis.

***peltopsychoides*** Müller, 1921: 531, fig. 184 l [type locality: Brazil; type depository unknown; pupal mandible]. —Ulmer 1957: 316 [bibliography]. —Flint et al. 1999b: 76 [identity; from Psychomyiidae to Hydroptilidae]. —Paprocki et al. 2004: 11 [checklist]. —Paprocki and França 2014: 44 [checklist]. —Santos et al. 2016a: 460 [to Trichoptera incertae sedis].

**Distribution.** —Brazil.

## References

[B1] Abbott JC Stewart KW Moulton SR II (1997) Aquatic insects of the Big Thicket region of East Texas. The Texas Journal of Science 49: 35–50.

[B2] Adachi M (1958) [Notes & Exhibitions]: *Oxyethiramaya*. Proceedings of the Hawaiian Entomological Society 16: 328.

[B3] Agassiz JLR (1846) Nomenclatur zoologicus. Index Universalis 32. 10.1017/S0261340900000734

[B4] Aguila Y (1992) Systematic catalogue of the caddisflies of Panama (Trichoptera). In: Quintero D Aiello A (Eds) Insects of Panama and Mesoamerica: Selected Studies.Oxford University Press, Oxford, 532–548.

[B5] Andersen T (1974) Caddis flies (Trichoptera) from the outer part of Sogn and Fjordane. Norsk Entomologisk Tidsskrift 21(1): 25–29.

[B6] Andersen T (1978) Influence of temperature on the sex ratio of Trichoptera in light-trap catches. Norwegian Journal of Entomology 25(2): 149–151.

[B7] Andersen T Hagenlund LK (2012) Caddisflies (Trichoptera) from Finnmark, northern Norway. Norwegian Journal of Entomology 59: 133–154.

[B8] Andersen T Kjaerandsen J (2002) First record of the microcaddisfly *Ithytrichiaclavata* Morton from Norway (Trichoptera: Hydroptilidae). Norwegian Journal of Entomology 49: 93–94.

[B9] Andersen T Klausen FE (1994) Light trap catches of caddis flies (Trichoptera) from a regulated and acidified southwest Norwegian river. Fauna Norvegica, Series B 41(1): 13–18.

[B10] Andersen T Tysse A (1985) The adult Trichoptera community in two western Norwegian rivers. Notulae Entomologicae 65: 81–91.

[B11] Andersen T Wiberg-Larsen P (1987) Revised check-list of NW European Trichoptera. Entomologica Scandinavica 18: 165–184.

[B12] Andersen T Hansen LO Johanson KA Solhøy T Søli GEE (1990) Faunistical records of caddis flies (Trichoptera) from Aust-Agder and Vest-Agder, south Norway. Fauna Norvegica Series B 37(1): 23–32.

[B13] Andersen T Ligaard S Søli GEE (1990) Faunistical records of caddis flies (Trichoptera) from Telemark, SE Norway. Fauna Norvegica Series B 37(2): 49–56.

[B14] Andersen T Hansen LO Johanson KA Sagvolden BA (1993a) Faunistical records of caddis flies (Trichoptera) from Buskerud, south Norway. Fauna Norvegica Series B 40: 49–57.

[B15] Andersen T Johanson KA Kobro S Ligaard S (1993b) Faunistical records of caddis flies (Trichoptera) from Ostfold and Akershus, SE Norway. Fauna Norvegica Series B 40: 1–12.

[B16] Andrikovics S Ujhelyi S (1983) Trichoptera of the Hungarian part of Lake Ferto (a faunistical and ecological treatise). Folia Entomologica Hungarica 44(2): 5–8.

[B17] Angrisano EB (1984 [1985]) Nuevas especies de Hydroptilidae Argentinos (Trichoptera). Revista de la Sociedad Entomológica Argentina 43: 1–5.

[B18] Angrisano EB (1989) *Rhyacopsycheyatay*, una nueva especie de Hydroptilidae de la Argentina (Trichoptera). Revista de la Sociedad Entomológica Argentina 46: 157–159.

[B19] Angrisano EB (1995a) Contribución al conocimiento de los Trichoptera del Uruguay. II. Familia Hydroptilidae. Revista Brasileira de Entomologia 39: 501–516.

[B20] Angrisano EB (1995b) Contribución para el conocimiento de las *Oxyethira* neotropicales (Trichoptera, Hydroptilidae). Physis (Buenos Aires). Seccion B 50: 27–35.

[B21] Angrisano EB (1999) Orden Trichoptera: Lista preliminar de especies de la Argentina y países limitrofes. Parte 1. Suborden Spicipalpia. Physis (Buenos Aires). Seccion B 57: 25–37.

[B22] Angrisano EB (2002) Contribution to the knowledge on Trichoptera of El Palmar National Park (Argentina). Description of the immature stages of *Bredinia* sp. and *Rhyacopsycheyatay* (Hydroptilidae). Nova Supplementa Entomologica (Proceedings of the 10^th^ International Symposium on Trichoptera) 15: 395–406.

[B23] Angrisano EB Burgos GN (2002) Contribución para el conocimiento do los Leucotrichiini (Trichoptera: Hydroptilidae). Tres especies nuevas de la Argentina. Revista de la Sociedad Entomológica Argentina 61: 103–109.

[B24] Angrisano EB Sganga JV (2005) Contribution to the knowledge of the genus *Metrichia* Ross from Argentina (Trichoptera: Hydroptilidae: Ochrotrichiini). Aquatic Insects 27(2): 113–123. 10.1080/01650420500062782

[B25] Angrisano EB Sganga JV (2007) Guia para la identificacion de los tricopteros (Insecta) del Parque Nacional El Palmar (Provincia Entre Rios, Republica Argentina). Natura Neotropicalis 38(38): 1–55. 10.14409/natura.v1i38.3858

[B26] Angrisano EB Sganga JV (2009) New species of Hydroptilidae (Trichoptera) from Salto Encantado Provincial Park (Misiones province, Argentina). Zootaxa 2162(1): 57–68. 10.11646/zootaxa.2162.1.5

[B27] Angrisano EB Sganga JV (2010) Preimaginal stages of *Acostatrichiasimulans* Mosely 1939, a Neotropical microcaddisfly (Trichoptera: Hydroptilidae: Leucotrichiinae). Zootaxa 2480(1): 54–60. 10.11646/zootaxa.2480.1.5

[B28] Arefina TI (2002) *Hydroptilaitoi* Kobayashi, 1977, a newly recorded caddisfly (Trichoptera: Hydroptilidae) from Russia. Far Eastern Entomologist = Dal’nevostochnyi Entomolog 112: 8.

[B29] Arefina TI (2004) A new species of the genus *Stactobiella* Martynov with reassignment of *Stactobiellatshistjakovi* (Arefina et Morse, 2002) and new records of micro-caddisflies (Trichoptera: Hydroptilidae) from the Russian Far East. Evraziatskii Entomologicheskii Zhurnal 3: 209–211.

[B30] Arefina TI Armitage BJ (2003) New findings of micro-caddisflies (Trichoptera: Hydroptilidae) from the Russian Far East. Braueria 30: 15–18.

[B31] Arefina TI Vshivkova TS Morse JC (2002) New and interesting Hydroptilidae (Insecta: Trichoptera) from the Russian Far East. Nova Supplementa Entomologica (Proceedings of the 10^th^ International Symposium on Trichoptera) 15: 96–106.

[B32] Armitage BJ Cornejo A (2015) Orden Trichoptera (Insecta) en Panamá: Listas de especies y su distribución por cuencas y unidades administrativas. Puente Biológico 7: 175–199.

[B33] Armitage BJ Harris SC (2018a) The Trichoptera of Panama V. Descriptions of new species, new country records, and a synonymy. Insecta Mundi 0604: 1–11.

[B34] Armitage BJ Harris SC (2018b) The Trichoptera of Panama VIII. The Hydroptilidae of Panama: Current status, biodiversity comparisons, projections, and needs. Aquatic Insects 39(2–3): 95–115. 10.1080/01650424.2018.1438629

[B35] Armitage BJ Harris SC (2018c) The Trichoptera of Panama IX. Preliminary comparison of caddisfly assemblages for two proximate watersheds in western Panama. Aquatic Insects 39(2–3): 275–295. 10.1080/01650424.2018.1481217

[B36] Armitage BJ Harris SC (2020a) The Trichoptera of Panama XIV. New species of microcaddisflies (Trichoptera: Hydroptilidae) from Omar Torrijos Herrera National Park. Insecta Mundi 0763: 1–19.

[B37] Armitage BJ Harris SC (2020b) Erratum to Armitage and Harris (2020): The Trichoptera of Panama XIV. New species of microcaddisflies (Trichoptera: Hydroptilidae) from Omar Torrijos Herrera National Park. Insecta Mundi 0764: 1–3.

[B38] Armitage BJ Mey W Arefina TI Schefter PW (2005) The caddisfly fauna (Insecta: Trichoptera) of Vietnam. In: Tanida K Rossiter A (Eds) Proceedings of the 11th International Symposium on Trichoptera.Tokai University Press, Kanagawa, 25–37.

[B39] Armitage BJ Harris SC Schuster GA Usis JD MacLean DB Foote BA Bolton MJ Garono RJ (2011) Atlas of Ohio Aquatic Insects, Volume I. Trichoptera. Ohio Biological Survey Miscellaneous Contribution 13(i–v): 1–92.

[B40] Armitage BJ Harris SC Arefina-Armitage TI Cornejo A (2015a) The Trichoptera of Panama. III. Updated species list for caddisflies (Insecta: Trichoptera) in the Republic of Panama. Insecta Mundi 0442: 1–16.

[B41] Armitage BJ Harris SC Holzenthal RW (2015b) The Trichoptera of Panama I. New records for caddisflies (Insecta: Trichoptera) from the Republic of Panama. Insecta Mundi 0435: 1–10.

[B42] Armitage BJ Harris SC Blahnik RJ Thomson RE (2016) The Trichoptera of Panama IV. New records for caddisflies (Insecta: Trichoptera) from the Republic of Panama. Insecta Mundi 0511: 1–13.

[B43] Armitage BJ Blahnik RJ Harris SC Cornejo A Arefina-Armitage TI (2018) The Trichoptera of Panama VII. Additional new country records for caddisflies from the Republic of Panama. Insecta Mundi 0614: 1–7.

[B44] Armitage BJ Harris SC Blahnik RJ Thomson RE Ríos González TA Aguirre Y (2020) The Trichoptera of Panama XIII. Further new country records for caddisflies (Insecta: Trichoptera) from the Republic of Panama. Insecta Mundi 0744: 1–8.

[B45] Arnold M Zoltán C Csaba D (2005) Spatial and temporal distribution of the *Tricholeiochitonfagesii* (Guinard, 1879) (Trichoptera: Hydroptilidae) in a lowland marsh. Acta Biolologica Debrrecina. Supplementum Oecologica Hungarica 13: 141–145.

[B46] Back RC (1983) Larva and pupa of *Oxyethiraleonensis* (Trichoptera: Hydroptilidae). The Florida Entomologist 66(4): 389–392. 10.2307/3494011

[B47] Bagge P (1982) Caddis flies (Trichoptera) and water bugs (Heteroptera, Corixidae) of small water bodies caught by light trapping in southeastern Finland. Notulae Entomologicae 62: 73–81.

[B48] Bagge P (1995) Emergence and upstream flight of lotic mayflies and caddisflies (Ephemeroptera and Trichoptera) in a lake outlet, central Finland. Entomologica Fennica 6(2–3): 91–97. 10.33338/ef.83844

[B49] Banks N (1904a) A list of neuropteroid insects, exclusive of Odonata, from the vicinity of Washington, D. C. Proceedings of the Entomological Society of Washington 6: 201–217.

[B50] Banks N (1904b) Two new species of Hydroptilidae. Entomological News 15: 116.

[B51] Banks N (1907a) A catalogue of the neuropteroid insects (except Odonata) of the United States. Transactions of the American Entomological Society 33: 1–53. 10.5962/bhl.title.32399

[B52] Banks N (1907b) New Trichoptera and Psocidae. Journal of the New York Entomological Society 15: 162–166.

[B53] Banks N (1911) Description of new species of North American neuropteroid insects. Transactions of the American Entomological Society 37: 335–360. [plates 311–313]

[B54] Banks N (1936) Four new Trichoptera from the United States. Arbeiten über Morphologische und Taxonomische Entomologie 3: 265–268.

[B55] Barba-Álvarez R Bueno-Soria J Ramírez-Martínez C (2019) Trichoptera of the Biosphere Reserve Montes Azules, Chiapas, Mexico. Zoosymposia 14(1): 81–86. 10.11646/zoosymposia.14.1.11

[B56] Barnard KH (1934) South African caddis-flies (Trichoptera). Transactions of the Royal Society of South Africa 21(4): 291–394. 10.1080/00359193409518885

[B57] Barnard PC (1971) The larva of *Agrayleasexmaculata* Curtis (Trichoptera, Hydroptilidae). Entomologist’s Gazette 22: 253–257.

[B58] Barndt D (2014) Contribution to the fauna of arthropods of the sphagnum-dominated bogs Kellsee and Himmelreichsee (Germany; federal state of Brandenburg). (Coleoptera, Heteroptera, Auchenorrhyncha, Hymenoptera part., Odonata, Diptera part., Araneae, Opiliones, Pseudoscorpiones, Diplopoda, Chilopoda etc.). Märkische Entomologische Nachrichten 16(2): 93–137.

[B59] Baryshev IA (2008) Diurnal dynamics of emergence of caddis flies *Agapetusochripes* Curt. and *Hydroptilatineoides* Dalm. in the Far North (Indera River, Kola Peninsula, Russia). Russian Journal of Ecology 39(5): 379–381. 10.1134/S1067413608050123

[B60] Baumgardner DE Bowles DE (2005) Preliminary survey of the mayflies (Ephemeroptera) and caddisflies (Trichoptera) of Big Bend Ranch State Park and Big Bend National Park. Journal of Insect Science 5(1): 1–13. 10.1093/jis/5.1.28 17119610 PMC1615235

[B61] Bayly IAE (1990) Abundance and drift of the larval micro-caddis, *Oxyethiraalbiceps* (McLachlan), in the Waikato River near Lake Taupo. New Zealand Entomologist 13(1): 52–55. 10.1080/00779962.1990.9722592

[B62] Beardsley JW (1960) [Notes & Exhibitions]: *Oxyethiramaya*. Proceedings of the Hawaiian Entomological Society 17: 181.

[B63] Beardsley JW (1971) *Hydroptilaarctia* Ross. Notes and exhibitions. Proceedings of the Hawaiian Entomological Society 21: 15–16.

[B64] Beketov MA (2006) Caddisflies (Trichoptera) of south-western Siberia: New zoogeographical records, aquatic habitat preferences and flight periods. Braueria 33: 13–16.

[B65] Berg K (1948) Biological studies in the River Susan. Folia Limnologica Scandinavica 4: 1–318.

[B66] Berlin A (2005) Zur Köcherfliegenfauna naturnaher Fliessgewässer-Abschnitte in Mecklenburg-Vorpommern - faunistische und typologische Aspekte. Lauterbornia 54: 123–134.

[B67] Berlin A Thiele V (2007) Zur Effizienz unterschiedlicher Erfassungsmethoden von Trichoptera in ausgewählten Fliessgewässertypen Mecklenburg-Vorpommerns. Lauterbornia 61: 43–56.

[B68] Bertuetti E Lodovici O Valle M (2004) Nuovi dati sui Tricotteri italiani. Braueria 31: 25–26.

[B69] Betten C (1934) The caddisflies or Trichoptera of New York State. Bulletin - New York State Museum 292: 1–576. 10.5962/bhl.title.132984

[B70] Bicchierai MC Moretti G (1994) Esame comparativo al microscopio elettronico a scansione dei palpi mascellari e labiali di tricotteri della fauna italiana. Atti del Congresso Nazionale Italiano di Entomologia 17: 107–113.

[B71] Biondi MJ (2010) Records of Trichoptera from South Carolina, USA. Entomological News 121(1): 59–62. 10.3157/021.121.0111

[B72] Blahnik RJ Paprocki H Holzenthal RW (2004) New distribution and species records of Trichoptera from southern and southeastern Brazil. Biota Neotropica 4(1): 1–6. 10.1590/S1676-06032004000100009

[B73] Blickle RL (1961) New species of Hydroptilidae (Trichoptera). Bulletin of the Brooklyn Entomological Society 56: 131–134.

[B74] Blickle RL (1963) New species of Hydroptilidae (Trichoptera). Bulletin of the Brooklyn Entomological Society 58: 17–22.

[B75] Blickle RL (1966) A new Hydroptilidae (Trichoptera). Entomological News 77: 185–187.

[B76] Blickle RL (1969) A new species of Hydroptilidae (Trichoptera). Entomological News 80: 79–81.

[B77] Blickle RL (1979) Hydroptilidae (Trichoptera) of America north of Mexico. New Hampshire Agricultural Experiment Station Bulletin 509: 1–97.

[B78] Blickle RL (1980) A new *Oxyethira* (Hydroptilidae, Trichoptera) of the *aeola* group; with a key to separate the five males of the group. The Pan-Pacific Entomologist 56: 101–104.

[B79] Blickle RL Denning DG (1977) New species and a new genus of Hydroptilidae (Trichoptera). Journal of the Kansas Entomological Society 50: 287–300.

[B80] Blickle RL Morse WJ (1954) New species of Hydroptilidae (Trichoptera). Bulletin of the Brooklyn Entomological Society 49: 121–127.

[B81] Blickle RL Morse WJ (1957) New Hydroptilidae (Trichoptera) from New Hampshire. Bulletin of the Brooklyn Entomological Society 52: 48–50.

[B82] Blinn DW Ruiter DE (2005) Caddisfly (Trichoptera) community structure and distribution in Arizona, USA: effects of selected environmental determinants. In: Tanida K Rossiter A (Eds) Proceedings of the 11th International Symposium on Trichoptera.Tokai University Press, Kanagawa, 63–71.

[B83] Blinn DW Ruiter DE (2006) Tolerance values of stream caddisflies (Trichoptera) in the lower Colorado River Basin, USA. The Southwestern Naturalist 51(3): 326–337. 10.1894/0038-4909(2006)51[326:TVOSCT]2.0.CO;2

[B84] Blinn DW Ruiter DE (2009a) Caddisfly (Trichoptera) assemblages along major river drainages in Arizona. Western North American Naturalist 69(3): 299–308. 10.3398/064.069.0303

[B85] Blinn DW Ruiter DE (2009b) Phenology and distribution of caddisflies (Trichoptera) in Oak Creek, a high-desert perennial stream in Arizona. The Southwestern Naturalist 54(2): 182–194. 10.1894/JC-25.1

[B86] Blinn DW Ruiter DE (2013) Tolerance values and effects of selected environmental determinants on caddisfly (Trichoptera) distribution in northwest and north central Washington, USA. Western North American Naturalist 73(3): 270–294. 10.3398/064.073.0302

[B87] Bochert R (2007) Der aktuelle Status von *Hydroptiladampfi* Ulmer, 1929 (Trichoptera, Hydroptilidae) in Europa mit einer Beschreibung der Genitalmorphologie der Weibchen. Lauterbornia 61: 119–126.

[B88] Bonada N Zamora-Muñoz C Rieradevall M Prat N (2004) Trichoptera (Insecta) collected in Mediterranean river basins of the Iberian Peninsula: Taxonomic remarks and notes on ecology. Graellsia 60(1): 41–69. 10.3989/graellsia.2004.v60.i1.192

[B89] Bonada N Zamora-Muñoz C Rieradevall M Prat N (2005) Ecological and historical filters constraining spatial caddisfly distribution in Mediterranean rivers. Freshwater Biology 50(5): 781–797. 10.1111/j.1365-2427.2005.01357.x 32390672 PMC7201900

[B90] Botosaneanu L (1956) Recherches sur les Trichoptères de Bulgarie recueillis par MM. le Prof. A. Balkanov et B. Rusev (Trichoptera). Beiträge zur Entomologie 6: 354–402.

[B91] Botosaneanu L (1960) Trichoptères recueillis à la lumiere dans la region des Lacs Masuriens de Pologne. Polskie Pismo Entomologiczne 30: 145–151.

[B92] Botosaneanu L (1967) Trichoptera. Limnofauna Europaea. Eine Zusammenstellung aller die europäischen Binnengewässer bewohnenden mehrzelligen Tierarten mit Angaben über ihre Verbreitung und Ökologie. J. Illies. Stuttgart, Gustav Fischer: 285–309.

[B93] Botosaneanu L (1970) Trichoptères de la République Démocratique-Populaire de la Corée. Annales Zoologici 27(15): 275–359.

[B94] Botosaneanu L (1973) Au carrefour des régions orientale, éthiopienne et paléarctique. Essai de reconstitution de l’histoire de quelques lignées ‘cool adapted’ de Trichoptères. Fragmenta Entomologica 9(2): 61–80. 10.5962/bhl.part.75942

[B95] Botosaneanu L (1974) Quatres nouvelles espèces palastiniennes de trichoptères (lnsecta. Trichoptera). Israel Journal of Entomology 9: 159–174.

[B96] Botosaneanu L (1977) Trichoptères (imagos) de Cuba, capturés par moi-même en 1973 (Insecta, Trichoptera). Fragmenta Entomologica 13: 231–284.

[B97] Botosaneanu L (1979) The caddis-flies (Trichoptera) of Cuba and of Isla de Pinos: A synthesis. Studies on the Fauna of Curacao and Other Caribbean Islands 99: 33–62.

[B98] Botosaneanu L (1980) Six nouvelles espèces ou sous-espèces de Trichoptères d’Europe Méridionale. Bulletin Zoologisch Museum. Universiteit van Amsterdam 7(17): 165–179.

[B99] Botosaneanu L (1980) Trichoptères adultes de Cuba collectés par les zoologistes cubain (Trichoptera). Mitteilungen Münchener Entomologischen Gesellschaft 69: 91–116.

[B100] Botosaneanu L (1981a) On a false and a genuine caddis-fly from Burmese Amber (Insecta: Trichoptera, Homoptera). Bulletin Zoologisch Museum. Universiteit van Amsterdam 8: 73–78.

[B101] Botosaneanu L (1981b) On some Trichoptera collected by Mrs. Drs. A.C.Ellis and Dr. W.N. Ellis on Gomera (Canary Islands). Entomologische Berichten 41: 186–190.

[B102] Botosaneanu L (1982a) Étude de quelques Trichoptères ouest-Paléarctiques intéressants appartenant au British Museum (Natural History). Bulletin Zoologisch Museum. Universiteit van Amsterdam 8(22): 177–188.

[B103] Botosaneanu L (1982b) Ordo Trichoptera et Homo insapiens. In: Moretti GP (Ed.) Proceedings of the 3rd International Symposium on Trichoptera.Dr W. Junk, The Hague, 11–19. 10.1007/978-94-009-8641-1_3

[B104] Botosaneanu L (1983) *Hydroptilabajgirana* sp. n. d’Iran et *Cyrnusmaroccanus* sp. n. du Maroc (Trichoptera). Entomologische Berichten 43(9): 139–143.

[B105] Botosaneanu L (1984) Variabilité géographique d’une espèce maghrebino-levantine de *Hydroptila* Dalman (Trichoptera). Entomologische Berichten 44(9): 136–139.

[B106] Botosaneanu L (1988) Trichoptères de la Martinique. Annales de la Société Entomologique de France (N.S.) 24: 215–228.

[B107] Botosaneanu L (1989) Seconde contribution à l’étude des trichoptères de la Martinique. Annales de la Société Entomologique de France (N.S.) 25: 95–104.

[B108] Botosaneanu L (1990a) About Far-eastern *Stactobia* McLachlan (Trichoptera: Hydroptilidae): a correction. Aquatic Insects 12(1): 47–48. 10.1080/01650429009361387

[B109] Botosaneanu L (1990b) Results of a trichopterological (Insecta: Trichoptera) travel to the Lesser Antilles in 1989. Bulletin de l’Institut Royal des Sciences Naturelles de Belgique. Entomologie 60: 39–48.

[B110] Botosaneanu L (1991) Amsterdam expedition to the West Indian Islands, report 71. Trichoptères d’Haïti. Bulletin de l’Institut Royal des Sciences Naturelles de Belgique. Entomologie 61: 113–134.

[B111] Botosaneanu L (1992) Fauna Palaestina. Insecta 6: Trichoptera of the Levant: imagines. The Israel Academy of Sciences and Humanities, Jerusalem, 294 pp.

[B112] Botosaneanu L (1993a) Two new microcaddisfly species from Siberia (Trichoptera: Hydroptilidae). Entomologische Zeitschrift 103(10): 184–188.

[B113] Botosaneanu L (1993b) Additions to the Trichoptera of the Canary Islands. Entomologist’s Gazette 44: 160–162.

[B114] Botosaneanu L (1993c) Addenda et corrigenda to the paper by L. Botosaneanu “Two new microcaddisfly species from Siberia” (Entomologische Zeitschrift, 103 (10): 184–188; 1993). Entomologische Zeitschrift 103(13): 247.

[B115] Botosaneanu L (1994a) Les Trichoptères de la Guadeloupe. Annales de la Société Entomologique de France (N.S.) 30: 33–54.

[B116] Botosaneanu L (1994b) A study of the larvae of caddisflies (Trichoptera) from Cuba. Tropical Zoology 7(2): 451–475. 10.1080/03946975.1994.10539267

[B117] Botosaneanu L (1995a) Caddis flies (Trichoptera) from the Dominican Republic (West Indies). I. the Hydroptilidae. Bulletin de l’Institut Royal des Sciences Naturelles de Belgique. Entomologie 65: 21–33.

[B118] Botosaneanu L (1995b) Caddis Flies (Trichoptera) from Turonian (Upper Cretaceous) amber of New Jersey. American Museum Novitates 3140: 1–7.

[B119] Botosaneanu L (1997) Possible sympatric speciation in Hydroptilidae. In: Holzenthal RW Flint Jr OS (Eds) Proceedings of the 8th International Symposium on Trichoptera.Ohio Biological Survey, Columbus, 43–48.

[B120] Botosaneanu L (2000) Étude d’une faunule madicole de Guadeloupe: Compléments à la connaissance des Trichoptères de l’Ile. Annales de Limnologie 36(4): 249–259. 10.1051/limn/2000023

[B121] Botosaneanu L (2002a) A classical case of insular radiation: the *Hydroptila* species of La Réunion. Nova Supplementa Entomologica (Proceedings of the 10^th^ International Symposium on Trichoptera) 15: 323–330.

[B122] Botosaneanu L (2002b) An annotated checklist of caddisflies from the Caribbean islands, with distribution and bibliography (Insecta, Trichoptera). Bulletin de la Société Entomologique de France 107(1): 79–108. 10.3406/bsef.2002.16821

[B123] Botosaneanu L (2003) Notes sur quelques Hydroptilidae de îles Canaries (Trichopt.). Bulletin de la Société Entomologique de France 108(1): 107–108. 10.3406/bsef.2003.16934

[B124] Botosaneanu L (2005) Interesting Trichoptera from the Netherlands in the collection of the Zoological Museum Amsterdam. Entomologische Berichten 65(1): 17–20.

[B125] Botosaneanu L Alkins-Koo M (1993) The caddis flies (Insecta: Trichoptera) of Trinidad and Tobago, West Indies. Bulletin de l’Institut Royal des Sciences Naturelles de Belgique. Entomologie 63: 5–45.

[B126] Botosaneanu L Bolland HR (1997) A mite (Acari: Erythraeidae) as unusual parasite on an adult caddisfly (Trichoptera: Hydroptilidae) from the Dominican Republic (West Indies). Studies on the Natural History of the Caribbean Region 73: 71–76.

[B127] Botosaneanu L Dumont B (1987) Notes sur quelques espèces d’*Hydroptila* du groupe *unicata* (Trichoptera: Hydroptilidae). Annales de Limnologie 23(2): 115–120. 10.1051/limn/1987007

[B128] Botosaneanu L Flint Jr OS (1982) On some Trichoptera from northern Venezuela and Ecuador (Insecta). Beaufortia 32: 13–26.

[B129] Botosaneanu L Gasith A (1971) Contributions taxonomiques et écologiques à la connaissance des Trichoptères (Insecta) d’Isra. Israel Journal of Zoology 20: 89–129.

[B130] Botosaneanu L Giudicelli J (1981) Observations morphologiques, éthologiques et écologiques sur *Hydroptilahirra* Mosely (Trichoptera: Hydroptilidae). In: Moretti GP (Ed.) Proceedings of the 3rd International Symposium on Trichoptera.Dr. W. Junk, The Hague, 21–29. 10.1007/978-94-009-8641-1_4

[B131] Botosaneanu L Giudicelli J (2004) Contributions to the knowledge of the fauna of caddisflies (Insecta: Trichoptera) from south-east France, with description of new taxa. Annales de Limnologie 40(1): 15–32. 10.1051/limn/2004002

[B132] Botosaneanu L Hyslop EJ (1998) A systematic and biogeographic study of the caddisfly fauna of Jamaica (Insecta: Trichoptera). Bulletin de l’Institut Royal des Sciences Naturelles de Belgique. Entomologie 68: 5–28.

[B133] Botosaneanu L Levanidova IM (1987) The remarkable genus *Palaeagapetus* Ulmer, 1912 (Hydroptilidae). In: Bournaud M Tachet H (Eds) Proceedings of the 5th International Symposium on Trichoptera.Dr. W. Junk, Dordrecht, The Netherlands, 43–46. 10.1007/978-94-009-4043-7_7

[B134] Botosaneanu L Levanidova IM (1988) TrichopteraHydroptilidae (Insecta) from Soviet Union Far-eastern Territories. Bulletin Zoölogisch Museum. Universiteit van Amsterdam 11(21): 169–176.

[B135] Botosaneanu L Malicky H (1978) Trichoptera. In: Illies J (Ed.) Limnofauna Europaea.Eine Zusammenstellung aller die europäischen Binnengewasser bewohnenden mehrzelligen Tierarten mit Angaben über ihre Verbreitung und Ökologie. Gustav Fischer Verlag & Swets & Zeitlinger B.V., Stuttgart & Amsterdam, 333–359.

[B136] Botosaneanu L Nozaki T (1996) Contributions to the knowledge of the genus *Stactobia* McLachlan, 1880 from Japan (Trichoptera: Hydroptilidae). Bulletin Zoölogisch Museum. Universiteit van Amsterdam 15(8): 55–63.

[B137] Botosaneanu L Sakal D (1992) Ecological observations on the caddisflies (Insecta: Trichoptera) from Trinidad and Tobago (W. Indies). Revue d’Hydrobiologie Tropicale 25: 197–207.

[B138] Botosaneanu L Sykora J (1963) Nouvelle contribution à la connaissance des Trichoptères de Bulgarie. Acta Fauna Entomologica Musei Nationalis Pragae 9: 121–142.

[B139] Botosaneanu L Sykora JL (1973) Sur quelques Trichoptères (Insecta: Trichoptera) de Cuba. In: Résultats des expéditions biospéologiques Cubano-Roumaines à Cuba. Editura Academiei Republicii Socialiste Romania, Bucharest, 379–407.

[B140] Botosaneanu L Thomas A (2005) Nouvelles contributions à la connaissance des Trichoptères de Martinique, avec description de deux espèces nouvelles (Trichoptera). Ephemera 6(2004): 33–58.

[B141] Botosaneanu L Viloria AL (2002) The caddisflies (Insecta, Trichoptera) of Isla de Margarita (Venezuela) - with description of two new species. Mitteilungen aus dem Museum für Naturkunde in Berlin Deutsche Entomologische Zeitschrift 49: 105–111. 10.1002/mmnd.20020490108

[B142] Botosaneanu L Johnson RO Dillon PR (1998) New caddisflies (Insecta: Trichoptera) from Upper Cretaceous amber of New Jersey, U.S.A. Polskie Pismo Entomologiczne 67: 219–231.

[B143] Bovill WD Downs BJ Lancaster J (2016) Caddisfly egg mass morphology mediates egg predation: potential costs to individuals and populations. Freshwater Biology (2015) 60(2): 360–372. 10.1111/fwb.12497

[B144] Bowles DE Mathis ML (1989) Caddisflies (Insecta: Trichoptera) of mountainous regions in Arkansas, with new state records for the order. Journal of the Kansas Entomological Society 62(2): 234–244.

[B145] Bowles DE Mathis ML (1992) A preliminary checklist of the caddisflies (Insecta: Trichoptera) of Oklahoma. Insecta Mundi 6: 29–35.

[B146] Bowles DE Harris SC Bueno-Soria J (1999) An assessment of New World Stactobiini (Trichoptera: Hydroptilidae: Hydroptilinae) larvae with new larval descriptions of *Alisotrichia*, *Mejicanotrichia*, and *Scelobotrichia*. In: Malicky H Chantaramongkol P (Eds) Proceedings of the 9th International Symposium on Trichoptera.Faculty of Science, Chiang Mai University, Chiang Mai, Thailand, 43–52.

[B147] Bowles DE Tiemann SG Easley GW (2007) Caddisfly (Insecta: Trichoptera) assemblages of large springs and spring-runs in central Texas, U.S.A. In: Bueno-Soria J Barba-Álvarez R Armitage BJ (Eds) Proceedings of the 12th International Symposium on Trichoptera.The Caddis Press, Columbus, Ohio, 15–29.

[B148] Bowles DE Cheri C Usrey FD Williams JM (2020) Caddisflies (Trichoptera) of the Buffalo National River, Arkansas. Insecta Mundi 0770: 1–17.

[B149] Brand C Miserendino ML (2011a) Characterizing Trichoptera trophic structure in rivers under contrasting land use in Patagonia, Argentina. Zoosymposia 5(1): 29–40. 10.11646/zoosymposia.5.1.3

[B150] Brand C Miserendino ML (2011b) Life history strategies and production of caddisflies in a perennial headwater stream in Patagonia. Hydrobiologia 673(1): 137–151. 10.1007/s10750-011-0768-3

[B151] Brand C Miserendino ML (2014) Biological traits and community patterns of Trichoptera at two Patagonian headwater streams affected by volcanic ash deposition. Zoological Studies (Taipei, Taiwan) 53(1): 72–85. 10.1186/s40555-014-0072-9

[B152] Brand C Miserendino ML Epele LB (2012) Spatial and temporal pattern of caddisfly distribution at a mesohabitat scale in two Patagonian mountain streams subjected to pastoral use. International Review of Hydrobiology 97(2): 83–99. 10.1002/iroh.201111368

[B153] Brettfeld R (1996) Wiederfunde verschollener Köcherfliegen (Insecta, Trichoptera) in Thüringen. Lauterbornia 25: 127–131.

[B154] Brettfeld R (1997) Erstnachweis der Köcherfliege *Orthotrichiacostalis* (Curtis, 1834) (Insecta: Trichoptera) für Thüringen. Thüringer Faunistische Abhandlungen 4: 137–138.

[B155] Brock V (1987) *Tricholeiochitonfagesii* (Guinard 1879) (Trichoptera: Hydroptilidae) in Hamburg wiedergefunden. Drosera 87: 85–88.

[B156] Brophy JT O’Connor JP (2020) A new site for *Tricholeiochitonfagesii* (Guinard, 1879) (Trichoptera: Hydroptilidae) in Ireland. Entomologist’s Record and Journal of Variation 132: 244–248.

[B157] Buczyńska E Buczyński P Zawal A Stępień E (2016) Environmental factors affecting micro-distribution of larval caddisflies (Trichoptera) in a small lowland reservoir under different types of watershed usage. Fundamental and Applied Limnology 188(2): 157–170. 10.1127/fal/2016/0833

[B158] Bueno-Soria J (1977) Una especie nueva de *Ochrotrichia* Mosely (Insecta: Trichoptera: Hydroptilidae). Anales del Instituto de Biologia, Universidad Nacional Autonoma de Mexico, Serie Zoologia 48: 141–144.

[B159] Bueno-Soria J (1983a) Five new species of caddisflies (Trichoptera) from Mexico. Proceedings of the Entomological Society of Washington 85: 450–455.

[B160] Bueno-Soria J (1983b) Three new species of Ochrotrichia (Metrichia) from Chiapas, Mexico (Trichoptera: Hydroptilidae). Proceedings of the Biological Society of Washington 96: 79–83.

[B161] Bueno-Soria J (1984) [1985]) Estudios en insectos acuaticos II. revision para México y Centroamerica del genero *Hydroptila* Dalman, 1819 (Trichoptera: Hydroptilidae). Folia Entomologica Mexicana 59: 79–138.

[B162] Bueno-Soria J (1999) Studies in aquatic insects XV: new species of *Neotrichia* and first record of *Oxyethirahilosa* (Trichoptera: Hydroptilidae) from Mexico. Entomological News 110: 113–117.

[B163] Bueno-Soria J (2002) The genus *Metrichia* Ross (Trichoptera: Hydroptilidae) from Mexico. Transactions of the American Entomological Society 128(2–3): 223–243.

[B164] Bueno-Soria J (2009) A review of the genus *Ochrotrichia* Mosely (Trichoptera: Hydroptilidae) from Mexico and Central America. Transactions of the American Entomological Society 135(1 & 2): 59–160. 10.3157/061.135.0202

[B165] Bueno-Soria J (2010) Some new Trichoptera (Glossosomatidae, Hydroptilidae, Hydropsychidae and Polycentropodidae) from Mexico. Proceedings of the Entomological Society of Washington 112(1): 22–31. 10.4289/0013-8797-112.1.22

[B166] Bueno-Soria J Barba-Alvarez R (1999a) Studies in aquatic insects XVI: two new species of the microcaddisfly genus *Mejicanotrichia* (Trichoptera: Hydroptilidae) from Mexico, with a key to the species in the genus. Entomological News 110: 118–122.

[B167] Bueno-Soria J Barba-Álvarez R (1999b) Studies in aquatic insects, XVII: new species of *Metrichia* (Trichoptera: Hydroptilidae) from Mexico. Anales del Instituto de Biologia, Universidad Nacional Autonoma de Mexico, Serie Zoologia 70(1): 29–33.

[B168] Bueno-Soria J Barba-Álvarez R (2011) Trichoptera de Chiapas. In: F. Álvarez (Ed.) Chiapas: estudios sobre su diversidad biológica. Universidad Nacional Autónoma de México, Mexico City, 345–362.

[B169] Bueno-Soria J Barba-Álvarez R (2018) New species and a new record of caddisflies (Trichoptera: Hydroptilidae) from Chiapas, México. Entomological News 127(4): 361–368. 10.3157/021.127.0408

[B170] Bueno-Soria J Flint Jr OS (1978) Catálogo sistemático de los tricopteros de México (Insecta: Trichoptera), con algunos registros de Norte, Centro y Sudamérica. Anales del Instituto de Biologia, Universidad Nacional Autonoma de Mexico, Serie Zoologia 49: 189–218.

[B171] Bueno-Soria J Hamilton SW (1986) Estudios en insectos acuaticos VI: cinco especies nuevas de trichopteros de México: (Trichoptera: Polycentropodidae; Hydroptilidae; Hydropsychidae). Anales del Instituto de Biologia, Universidad Nacional Autonoma de Mexico, Serie Zoologia 57: 299–310.

[B172] Bueno-Soria J Harris SC (1993) Estudios en insectos acuáticos de México. IX. cuatro especies nuevas del género *Alisotrichia* (Trichoptera: Hydroptilidae). Anales del Instituto de Biologia, Universidad Nacional Autonoma de Mexico, Serie Zoologia 64: 49–60.

[B173] Bueno-Soria J Holzenthal RW (1998) Studies in aquatic insects XIV: descriptions of eight new species of *Ochrotrichia* Mosely (Trichoptera: Hydroptilidae) from Costa Rica. Proceedings of the Biological Society of Washington 111(3): 604–612.

[B174] Bueno-Soria J Holzenthal RW (2003) New species and records of the microcaddisfly genus *Metrichia* Ross from Costa Rica (Trichoptera: Hydroptilidae). Studies on Neotropical Fauna and Environment 38(3): 173–197. 10.1076/snfe.38.3.173.28164

[B175] Bueno-Soria J Holzenthal RW (2004) New species of the genus *Ochrotrichia* Mosely (Trichoptera: Hydroptilidae) from Mexico and Panama. Transactions of the American Entomological Society 130: 245–269.

[B176] Bueno-Soria J Holzenthal RW (2008) The genus *Ochrotrichia* Mosely (Trichoptera: Hydroptilidae) in Costa Rica, with the description of four new species. Zootaxa 1763(1): 41–54. 10.11646/zootaxa.1763.1.3

[B177] Bueno-Soria J Santiago-Fragoso S (1981) Una nueva especie del genero *Ochrotrichia* Mosely (Trichoptera: Hydroptilidae) del Edo. de Hidalgo, Mexico. Anales del Instituto de Biologia, Universidad Nacional Autonoma de Mexico, Serie Zoologia 51: 383–388.

[B178] Bueno-Soria J Santiago-Fragoso S (1992) Studies in aquatic insects, XI: seven new species of the genus Ochrotrichia (Ochrotrichia) from South America (Trichoptera: Hydroptilidae). Proceedings of the Entomological Society of Washington 94: 439–446.

[B179] Bueno-Soria J Santiago-Fragoso S (1996) Estudios en insectos acuáticos. XIII. especie nueva del género *Hydroptila* (Insecta: Trichoptera: Hydroptilidae), de Veracruz, México. Anales del Instituto de Biologia, Universidad Nacional Autonoma de Mexico, Serie Zoologia 67: 343–347.

[B180] Bueno-Soria J Santiago-Fragoso S (1997) Studies in aquatic insects XII: descriptions of nineteen new species of the genus *Ochrotrichia* Mosely (Trichoptera: Hydroptilidae) from Mexico and Central America. Proceedings of the Entomological Society of Washington 99: 359–373.

[B181] Bueno-Soria J Santiago-Fragoso S (2002) Description of five new species of the genus *Metrichia* Ross (Trichoptera: Hydroptilidae) from Panama. Transactions of the American Entomological Society 128: 245–254.

[B182] Bueno-Soria J Santiago-Fragoso S Barba-Álvarez R (2001) Studies in aquatic insects, XVIII: New species and new record of caddisflies (Trichoptera) from Mexico. Entomological News 112: 145–158.

[B183] Bueno-Soria J Morrone JJ Barba-Álvarez R (2005) Trichoptera of Arroyo Las Flores, Tabasco, Mexico, and their biogeographic affinities. In: Tanida K Rossiter A (Eds) Proceedings of the 11th International Symposium on Trichoptera.Tokai University Press, Kanagawa, 73–76.

[B184] Bueno-Soria J Morrone JJ Barba-Álvarez R (2007) Trichoptera of the Sierra Tarahumara, Chihuahua, Mexico. In: Bueno-Soria J Barba-Álvarez R Armitage BJ (Eds) Proceedings of the 12th International Symposium on Trichoptera.The Caddis Press, Columbus, Ohio, 31–35.

[B185] Bunlue P Chantaramongkol P Thapanya D Malicky H (2012) The biodiversity of Trichoptera assemblage in Doi Suthep-Pui and Doi Inthanon National Parks, Chiang Mai, Thailand. Braueria 39: 7–21.

[B186] Cairns A Wells A (2008) Contrasting modes of handling moss for feeding and case-building by the caddisfly *Scelotrichiawillcairnsi* (Insecta: Trichoptera). Journal of Natural History 42(41–42): 2609–2615. 10.1080/00222930802354308

[B187] Çakin F (1983) Some new species and records of Trichoptera in Turkey. Aquatic Insects 5(4): 233–249. 10.1080/01650428309361150

[B188] Çakin F Malicky H (1983) Neue Köcherfliegen (Trichoptera) aus der Turkei und von der Balkanhalbins Entomologische Zeitschrift 93(18): 267–270.

[B189] Calor AR (2011) Checklist of Trichoptera (Insecta) from São Paulo State, Brazil. Biota Neotropica 11(suppl 1): 317–328. 10.1590/S1676-06032011000500028

[B190] Cavalcante BM Dumas LL Nessimian JL (2018) New species and new geographical record of *Ochrotrichia* Mosely 1934 (Trichoptera: Hydroptilidae) from Rio de Janeiro state, Brazil. Zootaxa 4462(2): 229–236. 10.11646/zootaxa.4462.2.4 30314043

[B191] Cerjanec D Kučinić M Vilenica M Ćukušić A Ćuk R Ibrahimi H Vučković I Žalac S Ruk D (2020) Ecological and faunistic features of caddisflies (Insecta: Trichoptera) in different types of habitats in the Dinaric karst area (Central Croatia). Ecologica Montenegrina 36: 6–39. 10.37828/em.2020.36.2

[B192] Chalkley A (2014) Freshwater invertebrate recorder’s annual report. Suffolk Natural History 50: 7–16.

[B193] Chalkley A (2015) Oulton Marshes - an aquatic invertebrate survey of the turf ponds and dykes. Suffolk Natural History 51: 33–54.

[B194] Chambers VT (1873) Micro-Lepidoptera. Canadian Entomologist 5(6): 110–115, 124–128. 10.4039/Ent5110-6

[B195] Chamorro-Lacayo ML Maes J-M Holzenthal RW Blahnik RJ (2007) Updated checklist of the Trichoptera of Nicaragua. In: Bueno-Soria J Barba-Álvarez R Armitage BJ (Eds) Proceedings of the 12th International Symposium on Trichoptera.The Caddis Press, Columbus, Ohio, 37–50.

[B196] Chantaramongkol P Malicky H (1986) [1987]) Beschreibung von neuen Köcherfliegen (Trichoptera, Insecta) aus Sri Lanka. Annalen des Naturhistorischen Museums in Wien. Serie B, Fur Botanik und Zoologie 88/89: 511–534.

[B197] Chuluunbat S Morse JC (2007) Caddisflies (Insecta: Trichoptera) of Selenge River Basin, Mongolia. In: Bueno-Soria J Barba-Álvarez R Armitage BJ (Eds) Proceedings of the 12th International Symposium on Trichoptera.The Caddis Press, Columbus, Ohio, 51–57.

[B198] Chuluunbat S Morse JC Boldbaatar S (2016) Caddisflies of Mongolia: Distribution and diversity. In: Vshivkova TS Morse JC (Eds) Proceedings of the 14th International Symposium on Trichoptera.Zoosymposia 10: 96–116. 10.11646/zoosymposia.10.1.10

[B199] Chvojka P (1996) New faunistic records of Trichoptera (Insecta) from the Czech Republic. Časopis Národního Muzea Řada Přírodovědná 165: 131–132.

[B200] Chvojka P (1997) Contribution to the knowledge of the caddisfly fauna (Trichoptera, Insecta) of Albania. Časopis Národního Muzea Řada Přírodovědná 166: 27–38.

[B201] Chvojka P (2006) Contribution to the knowledge of the caddisfly fauna (Trichoptera) of Iran: Description of new species and new distributional data. Acta Entomologica Musei Nationalis Pragae 46: 245–255.

[B202] Chvojka P Komzák P (2006) Chrostici (Trichoptera) CHKO Kokorinsko. Bohemia Centralis 27: 355–363.

[B203] Chvojka P Komzák P (2008) The history and present state of Trichoptera research in the Czech Republic. Ferrantia 55: 11–21.

[B204] Chvojka P Komzák P Spacek J (2009) New faunistic records of Trichoptera (Insecta) from the Czech Republic, III. Acta Musei Moraviae Scientiae Biologicae (Brno) 94: 81–85.

[B205] Chvojka P Špaček J Komzák P Lukáš j (2016) New faunistic records of Trichoptera from the Czech Republic and Slovakia. Nové faunistické nálezy chrostíku (Trichoptera) z České republiky a Slovenska. Klapalekiana 52: 43–46.

[B206] Cianficconi F Corallini C (2010) Trichopteran fauna in a region of central-southern Italy: Molise. Denisia 29: 81–104.

[B207] Cianficconi F Moretti GP (1987 [1989]) Tricotteri del Friuli-Venezia Giulia. Biogeographia 13: 663–689. 10.21426/B613110284

[B208] Cianficconi F Moretti G Valle M (1993) I tricotteri del Museo di Bergamo (II nota) segnalalzioni nuove per la fauna italiana. Rivista del Museo Civico di Scienze Naturali “Enrico Caffi” Bergamo 16: 255–286.

[B209] Cianficconi F Corallini C Moretti GP (1999a) Trichoptera and their symbionts in the eastern Italian Alps. In: Malicky H Chantaramongkol P (Eds) Proceedings of the 9th International Symposium on Trichoptera.Faculty of Science, Chiang Mai University, Chiang Mai, Thailand, 55–63.

[B210] Cianficconi F de Pietro R Gerecke R Moretti GP (1999b) Catalogo dei Tricotteri della Sicilia. Memorie della Societa Entomologica Italiana 77: 259–309.

[B211] Cianficconi F Corallini C Tucciarelli F (2002) Informazioni sui Tricotteri italiani di ambienti costieri salmastri. Biogeographia 23: 139–155. 10.21426/B6110036

[B212] Cianficconi F Corallini C Tucciarelli F (2004a) Littoral Trichoptera of volcanic lakes Vico and Bolsena (Central Italy). Annales de Limnologie 40(3): 252–259. 10.1051/limn/2004021

[B213] Cianficconi F Mazzerioli S La Porta G (2004b) Tricotterofauna di tre affluenti dell’alto corso del Fiume Tevere (Trichoptera). Fragmenta Entomologica 36(2): 319–358.

[B214] Cianficconi F Todini B Pedrotti CC (2005) Italian caddisflies living on mosses: a preliminary note. In: Tanida K Rossiter A (Eds) Proceedings of the 11th International Symposium on Trichoptera.Tokai University Press, Kanagawa, 91–99.

[B215] Cianficconi F Corallini C Tucciarelli F (2007a) Trichoptera endemic to the Italian fauna. In: Bueno-Soria J Barb-Álvarez R Armitage BJ (Eds) Proceedings of the 12th International Symposium on Trichoptera.The Caddis Press, Columbus, Ohio, 65–74.

[B216] Cianficconi F Tucciarelli F Todini B (2007b) Aggiornamento della tricotterofauna della regione Umbria. Biogeographia 28: 561–586. 10.21426/B6110045

[B217] Cianficconi F Corallini C La Porta G Todini B (2011) Trichopteran fauna in a region of Central Italy: Lazio. Zoosymposia 5(1): 41–62. 10.11646/zoosymposia.5.1.4

[B218] Cianficconi F Corallini C Tucciarelli F Bicchierai MC (2016) The genus *Hydroptila* Dalman 1819 in Italy: Ecology and morphology. In: Vshivkova TS Morse JC (Eds) Proceedings of the 14th International Symposium on Trichoptera.Zoosymposia 10: 117–147. 10.11646/zoosymposia.10.1.11

[B219] Cibaitė G (2003a) Checklist of Lithuanian caddisflies (Insecta, Trichoptera). Braueria 30: 7–14.

[B220] Cibaitė G (2003b) New caddis fly (Trichoptera) species recorded in Lithuania in 1991–2002. New and Rare for Lithuania Insect Species Records and Descriptions 15: 6–10.

[B221] Ciubuc C (2009) Order Trichoptera (Insecta) from the Apuseni Nature Park (Transylvania, Romania). Transylvanian Review of Systematical and Ecological Research 7: 97–124.

[B222] Cloud Jr JJ Stewart KW (1974) Seasonal fluctuations and periodicity in the drift of caddisfly larvae (Trichoptera) in the Brazos River, Texas. Annals of the Entomological Society of America 67(5): 805–811. 10.1093/aesa/67.5.805

[B223] Cockerell TAD (1920) Eocene insects from the Rocky Mountains. Proceedings of the United States National Museum 57(2313): 233–260. 10.5479/si.00963801.57-2313.233

[B224] Coineau Y Jacquemart S (1961) Un Trichoptère Hydroptilide nouveau des Pyrénees-Orientales *Stactobiadelamarien*. sp. Vie et Milieu 12: 537–563.

[B225] Collier KJ Smith BJ (1998) Dispersal of adult caddisflies (Trichoptera) into forests alongside three New Zealand streams. Hydrobiologia 361(1997–1998(1998)): 53–65. 10.1023/A:1003133208818

[B226] Cooter J (1987) *Hydroptilalotensis* Mosely (Trichop., Hydroptilidae) in Hereford. Entomologist’s Monthly Magazine 123: 148.

[B227] Coppa G (2001) *Hydroptilalotensis* Mosely, 1930, une citation nouvelle pour la faune de Belgique (Trichoptera, Hydroptilidae). Ephemera 3(2): 94.

[B228] Coppa G (2010) Addition a la faune des Trichopteres de France: *Hydroptilaivisa* Malicky, 1972 (Trichoptera: Hydroptilidae). Ephemera 11(1): 23–25.

[B229] Coppa G (2013) Inventaire diagnostic des Trichoptères de la Réserve naturelle nationale de la Vallée de Chaudefour (Puy-de-Dôme, France) (Trichoptera). Ephemera 13(2): 97–128.

[B230] Coppa G Gonzalez M (2007) Additions à la faune des Trichopteres de France: *Allotrichiagalaica* Gonzalez & Malicky, 1980 (Trichoptera, Hydroptilidae). Ephemera 7(2): 95–100.

[B231] Coppa G Jolivet S (2008) Redécouverte de *Tricholeiochitonfagesii* (Guinard, 1879) en France (Trichoptera, Hydroptilidae). Ephemera 9: 91–93.

[B232] Coppa G Malicky H (2005) Description d’une nouvelle espèce européenne du genre *Hydroptila* (Trichoptera, Hydroptilidae). Braueria 32: 19.

[B233] Coppa G Tachet H (2004) Complements et corrections à la faune des Trichopteres de France: 3. Description des genitalia femelles d‘*Hydroptilaacuta* Mosely, 1930 et d’*H.cognata* Mosely, 1930 (Trichoptera, Hydroptilidae). Ephemera 4(2): 123–129.

[B234] Coppa G Tachet H (2005) La femelle d’*Hydroptilaphaon* Malicky, 1976 (Trichoptera: Hydroptilidae). Ephemera 6(2): 125–133.

[B235] Corallini C (2007) The goblet cells in Trichoptera. Proceedings of the 12^th^ International Symposium on Trichoptera. J. Bueno-Soria, R. Barba-Álvarez and B. J. Armitage. The Caddis Press, Columbus, Ohio, 75–81.

[B236] Corallini C Bicchierai MC (2016) Trichoptera larvae and gregarines: Host-parasite relationships. Zoosymposia 10: 148–164. 10.11646/zoosymposia.10.1.12

[B237] Corallini C Cianficconi F (2011) I Tricotteri endemici presenti in Sicilia. BIogeographia 30: 627–636. 10.21426/B630110572

[B238] Corallini C Bicchierai MC Cianficconi F Tucciarelli F (2013a) The genus *Hydroptila* Dalman, 1819 in Italy. Braueria 40: 35–40.

[B239] Corallini C Cianficconi F La Porta G Gramegna C (2013b) The trichopteran fauna of the Campania region in southern Italy. Braueria 40: 24–34.

[B240] Cowley DR (1978) Studies on the larvae of New Zealand Trichoptera. New Zealand Journal of Zoology 5(4): 639–750. 10.1080/03014223.1978.10423816

[B241] Crofts SM (2011) Caddisfly Recorder’s Report 2009. The Sorby Record: A Journal of Natural History for the Sheffield Area (Sheffield, Peak National Park, South Yorkshire, North Derbyshire and North Nottinghamshire) 47: 72.

[B242] Curtis J (1831) British entomology; being illustrations and descriptions of the genera of insects found in Great Britain and Ireland: coloured figures from nature of the most rare and beautiful species, and in many instances of the plants upon which they are found. Vol. VIII. London, printed for the author, 1823–1840 [i.e.,1829–1840], plate 346. 10.5962/t.171559

[B243] Curtis J (1834) Description of some hitherto nondescript British species of mayflies of anglers. The London and Edinburgh Philosophical Magazine and Journal of Science 3(4): 120–125, 212–218. 10.1080/14786443408648276

[B244] Czachorowski S (1995) Two caddis flies (Trichoptera, Hydroptilidae) species new to the Polish fauna. Przeglad Zoologiczny 39(3–4): 279–281.

[B245] Czachorowski S Prishchepchik O (1998) Further data on Belarussian Trichoptera. Braueria 25: 11.

[B246] Dallai R Afzelius BA (1995) Sperm structure of Trichoptera. 2. The aflagellated spermatozoa of *Hydroptila*, *Orthotrichia* and *Stactobia* (Hydroptilidae). International Journal of Insect Morphology & Embryology 24(2): 161–170. 10.1016/0020-7322(95)93341-9

[B247] Dalman JW (1819) Några nya insecta-genera beskrifna. Kongliga Vetenskaps-Akademiens Handlingar 40: 117–127.

[B248] Dambri BM Karaouzas I Samraoui B Samraoui F (2020) Contribution to the knowledge of the caddisfly fauna of Algeria: An updated checklist of Algerian Trichoptera with new records from the Aures region. Zootaxa 4786(2): 221–232. 10.11646/zootaxa.4786.2.4 33056484

[B249] Danecker E (1961) Studien zur hygropetrischen fauna. Biologie und Ökologie von *Stactobia* und *Tinodes* (Insecta, Trichoptera). Internationale Revue der Gesamten Hydrobiologie 46(2): 214–254. 10.1002/iroh.19610460206

[B250] de Jalón DG González M (1985) Description of *Hydroptilatagus* sp. n. (Trichoptera: Hydroptilidae) from Spain. Aquatic Insects 7(2): 73–75. 10.1080/01650428509361204

[B251] de Moor FC (2007) Regional biogeographical differences in Trichoptera diversity in South Africa: Observed patterns and processes. In: Bueno-Soria J Barba-Álvarez R Armitage BJ (Eds) Proceedings of the 12th International Symposium on Trichoptera.The Caddis Press, Columbus, Ohio, 211–218.

[B252] de Moor FC (2011) A survey of Trichoptera from the tributaries of the Doring and mainstream Olifants Rivers, Cedarberg, South Africa with implications for conservation. Zoosymposia 5(1): 350–359. 10.11646/zoosymposia.5.1.27

[B253] de Moor FC Bellingan TA (2019) Evaluation of the conservation requirements of Trichoptera from the Tsitsikamma mountain streams in South Africa. Zoosymposia 14(1): 151–164. 10.11646/zoosymposia.14.1.17

[B254] de Moor FC Barber-James HM Harrison AD Lugo-Ortiz CR (2000) The macroinvertebrates of the Cunene River from the Ruacana Falls to the river mouth and assessment of the conservation status of the river. African Journal of Aquatic Science 25(1): 105–122. 10.2989/160859100780177857

[B255] de Souza WRM Santos APM (2017) Taxonomic study of the genus *Oxyethira* Eaton 1873 (Trichoptera: Hydroptilidae) from Northeast Brazil: Eleven new species and distributional records. Zootaxa 4236(3): 484–506. 10.11646/zootaxa.4236.3.4 28264314

[B256] de Souza WRM Santos APM Lima LRC Pinheiro U (2013) A new species and new records of microcaddisflies (Trichoptera: Hydroptilidae) from northeastern Brazil. Zootaxa 3700: 583–587. 10.11646/zootaxa.3700.4.6 26106745

[B257] de Souza WRM Santos APM Takiya DM (2014a) First records of *Ochrotrichia* Mosely, 1934 (Trichoptera: Hydroptilidae) in Northeastern Brazil: Five new species and two new geographical records. Zootaxa 3852(2): 273–282. 10.11646/zootaxa.3852.2.6 25284397

[B258] de Souza WRM Santos APM Takiya DM (2014b) Three new species of *Hydroptila* (Trichoptera: Hydroptilidae) from Northeastern Brazil. Zoologia 31(6): 639–643. 10.1590/S1984-46702014000600010

[B259] de Souza WRM Santos APM Takiya DM (2016a) Description of a new species of *Betrichia* Mosely 1939 from Brazil and redescription of the type-species (Trichoptera: Hydroptilidae: Leucotrichiinae). Zootaxa 4061: 291–295. 10.11646/zootaxa.4061.3.9 27395503

[B260] de Souza WRM Santos APM Takiya DM (2016b) Three new species of Stactobiinae (Trichoptera: Hydroptilidae) with the first record of *Orinocotrichia* Harris, Flint & Holzenthal from Brazil. Zootaxa 4078(1): 337–343. 10.11646/zootaxa.4078.1.28 27395984

[B261] del Guercio G (1907) Intorno ad alcune nuove divisioni del Gen. *Aphis* Linné. Redia (Firenze) 4: 190–192.

[B262] Denning DG (1947a) Hydroptilidae (Trichoptera) from the southern United States. Canadian Entomologist 79(1): 12–20. 10.4039/Ent7912-1

[B263] Denning DG (1947b) New species and records of North American Hydroptilidae (Trichoptera). Psyche 54(3): 170–177. 10.1155/1947/35703

[B264] Denning DG (1948) New species of Trichoptera. Annals of the Entomological Society of America 41(3): 397–401. 10.1093/aesa/41.3.397

[B265] Denning DG (1989) Eight new species of Trichoptera. The Pan-Pacific Entomologist 65(2): 123–131.

[B266] Denning DG Blickle RL (1971) A new Trichoptera from the Hawaiian Islands. The Pan-Pacific Entomologist 47: 164.

[B267] Denning DG Blickle RL (1972) A review of the genus *Ochrotrichia* (Trichoptera: Hydroptilidae). Annals of the Entomological Society of America 65(1): 141–151. 10.1093/aesa/65.1.141

[B268] Denson DR Rasmussen AK Harris SC (2016) Caddisflies (Insecta: Trichoptera) of the Chipola River basin in Florida and southeast Alabama, USA: a faunistic survey. Check List 12(4): 1–19. 10.15560/12.4.1936

[B269] DeWalt RE Heinold BD (2005) Summer emerging Ephemeroptera, Plecoptera, and Trichoptera of Abrams Creek, Great Smoky Mountains National Park. Proceedings of the Entomological Society of Washington 107: 34–48.

[B270] DeWalt RE South EJ Robertson DR Marburger JE Smith WW Brinson V (2016) Mayflies, stoneflies, and caddisflies of streams and marshes of Indiana Dunes National Lakeshore, USA. ZooKeys 556: 43–63. 10.3897/zookeys.556.6725 PMC4740871 26877693

[B271] Dewey SL (1986) Effects of the herbicide atrazine on aquatic insect community structure and emergence. Ecology 67: 148–162. 10.2307/1938513

[B272] Dia A (2015) Diversité, répartition et biogéographie des Trichoptères des rivières du Liban (Trichoptera). Bulletin de la Société d’Histoire Naturelle de Toulouse 151: 47–57.

[B273] Dia A Botosaneanu L (1980) Une *Stactobia* nouvelle du Liban (Trichoptera, Hydroptilidae) ses stades aquatiques et leurs constructions. Contributions to Zoology 50(2): 369–374. 10.1163/26660644-05002007

[B274] Dia A Botosaneanu L (1982) Un cas de gynandromorphisme chez un trichoptère hydroptilide du Liban (Trichoptera: Hydroptilidae). Entomologische Berichten 42(9): 140–141.

[B275] Dia A Botosaneanu L (1983) Six espèces nouvelles de Trichoptères du Liban. Bulletin Zoologisch Museum. Universiteit van Amsterdam 9(14): 125–135.

[B276] Disney RHL (1972) Larval Hydroptilidae (Trichoptera) that prey upon Simuliidae (Diptera) in Cameroon. Entomologist’s Monthly Magazine 108: 84–85.

[B277] Djernaes M (2011) Structure and phylogenetic significance of the sternum V glands in Trichoptera. Zootaxa 2884(1): 1–60. 10.11646/zootaxa.2884.1.1

[B278] Djernaes M Sperling FAH (2011) Evolutionary riddles and phylogenetic twiddles: the ground plan and early diversification of the sternum V gland in Amphiesmenoptera (Trichoptera plus Lepidoptera). Zoosymposia 5: 83–100. 10.11646/zoosymposia.5.1.7

[B279] Dohet A Ferréol M Cauchie H-M Hoffmann L (2008) Caddisfly assemblages characterizing different ecological areas in Luxembourg: From geographical distributions to bioindication. Ferrantia 55: 33–56.

[B280] Dorn A Klima F Weinzierl A (1993) *Oxyethiratristella* Klapálek, 1895 (Trichoptera) - eine neue Köcherfliegenart für Deutschland. Entomologische Nachrichten und Berichte 37(4): 258–259.

[B281] Drescher D (2013) Zum Vorkommen der Köcherfliege *Ithytrichialamellaris* Eaton, 1873 (Trichoptera: Hydroptilidae) im Fließgewässersystem der Leine in Südniedersachsen. Braunschweiger Naturkundliche Schriften 12: 53–60.

[B282] Duke MJ (1994) New records of *Beraeodesminutus* (L.) and *Allotrichiapallicornis* (Eaton) (Trichoptera) in Ireland. Irish Biogeographical Society Bulletin 17(1): 7–8.

[B283] Dumas LL Nessimian JL (2012) Faunistic catalog of the caddisflies (Insecta: Trichoptera) of Parque Nacional do Itatiaia and its surroundings in southeastern Brazil. Journal of Insect Science 12(25): 1–38. 10.1673/031.012.2501 PMC3471775 22958122

[B284] Dumas LL Jardim GA Santos APM Nessimian JL (2009) Tricópteros (Insecta: Trichoptera) do estado do Rio de Janeiro: List de expécies e novos registros. Arquivos do Museu Nacional, Rio de Janeiro 67: 355–376.

[B285] Dumas LL Santos APM Jardim GA Ferreira Jr N Nessimian JL (2010) Insecta, Trichoptera: New records from Brazil and other distributional notes. Check List 6(1): 7–9. 10.15560/6.1.007

[B286] Dzhurtubaev YM Dzhurtubaev MM Zamorov VV (2017) Macrozoobenthos of Cahul Lake (Danube basin, Odessa region, Ukraine). Ukrainian Journal of Ecology 7(3): 56–63. 10.15421/2017_49

[B287] Eaton AE (1873) On the Hydroptilidae, a family of the Trichoptera. The Transactions of the Entomological Society of London 2(2): 125–151. 10.1111/j.1365-2311.1873.tb00639.x

[B288] Edmonds-Brown R (2020) Trichoptera of Hertfordshire. Life History Tables – a valuable ecological tool. Transactions of the Hertfordshire Natural History Society 52(1): 90–97.

[B289] Edwards SW (1973) Texas caddisflies. The Texas Journal of Science 24: 491–516.

[B290] Edwards SW Arnold CR (1961) The caddis flies of the San Marcos river. The Texas Journal of Science 13: 398–415.

[B291] Enderlein G (1929) Entomologica Canaria 2. Zoologischer Anzeiger 84(9/10): 221–234.

[B292] English WR Hamilton SW (1986) The larvae of *Ochrotrichiaarizonica* (Trichoptera: Hydroptilidae) with notes on distribution and geographic variation. Journal of the Kansas Entomological Society 59(3): 474–479.

[B293] Englmaier GK Hayes DS Meulenbroek P Terefe Y Lakew A Tesfaye G Waidbacher H Malicky H Wubie A Leitner P Graf W (2020) Longitudinal river zonation in the tropics: examples of fish and caddisflies from the endorheic Awash River, Ethiopia. Hydrobiologia 847: 4063–4090. 10.1007/s10750-020-04400-0

[B294] Eskov KY Wells A Ivanov VD Kulicka R Sukacheva I (2008) Fossil Hydroptilidae (Trichoptera), their probable biology and paleogeography. Prace Muzeum Ziemi 49: 77–86. 10.1002/mmnd.20020490109

[B295] Etnier DA (1965) An annotated list of the Trichoptera of Minnesota, with description of a new species. Entomological News 76: 141–152.

[B296] Etnier DA (1968) Range extensions of Trichoptera into Minnesota, with descriptions of two new species. Entomological News 79: 188–192.

[B297] Etnier DA (2010) New Trichoptera records from Arkansas and Missouri. Proceedings of the Entomological Society of Washington 112(4): 483–489. 10.4289/0013-8797.112.4.483

[B298] Etnier DA Baxter JJT (1999) Reillustrations of *Hydroptilalloganae*, with a new junior synonym, *Hydroptilamorsei* (Trichoptera: Hydroptilidae). Entomological News 110: 147–150.

[B299] Etnier DA Schuster GA (1979) An annotated list of Trichoptera (caddisflies) of Tennessee. Journal. Tennessee Academy of Science 54: 15–22.

[B300] Etnier DA Way JD (1973) New southeastern Trichoptera. Journal of the Kansas Entomological Society 46(3): 422–430.

[B301] Evenhuis NL (2021) The insect and spider collections of the world website. http://hbs.bishopmuseum/org/codens/ [accessed March 1, 2022]

[B302] Evenhuis NL Arakaki KT Imada CT (2020) Terrestrial Arthropod Survey of Hālona Valley, Joint Base Pearl Harbor-Hickam, Naval Magazine Lualualei Annex, July 2019-September 2019. Contribution No. 2020-008 to the Hawaii Biological Survey, 37 pp.

[B303] Fahy E (1971) The larva of *Hydroptilaforcipata* (Eaton) (Trich., Hydroptilidae). Entomologist’s Monthly Magazine 107: 145–148.

[B304] Fahy E (1972) Some records of Trichoptera from Ireland. The Irish Naturalist’. Journal 17(6): 199–203.

[B305] Felber J (1908) *Microptilarisi* n. sp. eine neue Hydroptilide aus der Umgebung von Bas Zoologischer Anzeiger 32: 720–722.

[B306] Fischer FCJ (1961) Philopotamidae, Hydroptilidae, Stenopsychidae. Trichopterorum Catalogus 2. Nederlandsche Entomologische Vereeniging, Amsterdam, [iv +] 190.

[B307] Fischer FCJ (1971) Supplement to Vol. I and II. Trichopterorum Catalogus 12. Nederlandsche Entomologische Vereeniging, Amsterdam, [vii +] 311.

[B308] Flint Jr OS (1962) The immature stages of *Palaeagapetuscelsus* Ross (Trichoptera: Hydroptilidae). Bulletin of the Brooklyn Entomological Society 42: 40–44.

[B309] Flint Jr OS (1964) The caddisflies (Trichoptera) of Puerto Rico. University of Puerto Rico, Agricultural Experiment Station, Technical Paper 40: 1–80.

[B310] Flint Jr OS (1965) New species of Trichoptera from the United States. Proceedings of the Entomological Society of Washington 67: 168–176.

[B311] Flint Jr OS (1966) On the identity of *Clymeneaegerfasciella* Chambers. Proceedings of the Entomological Society of Washington 68: 135.

[B312] Flint Jr OS (1967a) Studies of Neotropical Caddiflies II, Trichoptera collected by Prof. J. Illies in the Chilean subregion. Beiträge zur Neotropischen Fauna 5: 45–68. 10.1080/01650526709360395

[B313] Flint Jr OS (1967b) Studies of Neotropical caddis flies, IV: New species from Mexico and Central America. Proceedings of the United States National Museum 123: 1–24. 10.5479/si.00963801.123-3619.1

[B314] Flint Jr OS (1968a) Bredin-Archbold-Smithsonian Biological Survey of Dominica, 9. The Trichoptera (Caddisflies) of the Lesser Antilles. Proceedings of the United States National Museum 125(3665): 1–86. 10.5479/si.00963801.125-3665.1

[B315] Flint Jr OS (1968b) The Caddisflies of Jamaica. Bulletin of the Institute of Jamaica, Science Series 19: 1–68.

[B316] Flint Jr OS (1968c) New species of Trichoptera from the Antilles. The Florida Entomologist 51(3): 151–153. 10.2307/3493548

[B317] Flint Jr OS (1970) Studies of Neotropical caddisflies, X: *Leucotrichia* and related genera from North and Central America (Trichoptera: Hydroptilidae). Smithsonian Contributions to Zoology 60(60): 1–64. 10.5479/si.00810282.60

[B318] Flint Jr OS (1971) Studies of Neotropical caddis flies, XI: The genus *Rhyacopsyche* in Central America (Hydroptilidae). Proceedings of the Biological Society of Washington 83: 515–526.

[B319] Flint Jr OS (1972a) Studies of Neotropical caddisflies, XIII: the genus *Ochrotrichia* for Mexico and Central America (Trichoptera: Hydroptilidae). Smithsonian Contributions to Zoology 118(118): 1–28. 10.5479/si.00810282.118

[B320] Flint Jr OS (1972b) Studies of Neotropical caddisflies, XIV: On a collection from northern Argentina. Proceedings of the Biological Society of Washington 85: 223–248.

[B321] Flint Jr OS (1974a) Checklist of the Trichoptera, or Caddisflies, of Chile. Revista Chilena de Entomologia 8: 83–93.

[B322] Flint Jr OS (1974b) The Trichoptera of Surinam. Studies of Neotropical caddisflies, XV. Studies on the Fauna of Suriname and other Guyanas 14: 1–151[pls 151–154].

[B323] Flint Jr OS (1975) Studies of Neotropical caddisflies, XX: Trichoptera collected by the Hamburg South-Peruvian Expedition. Entomologische Mitteilungen aus dem Zoologischen Museum Hamburg 4: 565–573.

[B324] Flint Jr OS (1980a) The results of the Catherwood Foundation Bolivian-Peruvian Altiplano Expedition. Part I. Aquatic insects except Diptera. VI. Trichoptera. Proceedings. Academy of Natural Sciences of Philadelphia 132: 213–217.

[B325] Flint Jr OS (1980b) Studies on Neotropical caddisflies, XXVI: New species from Argentina (Trichoptera). Revista de la Sociedad Entomológica Argentina 39: 137–142.

[B326] Flint Jr OS (1981) Studies of Neotropical caddisflies, XXVIII: The Trichoptera of the Río Limón Basin, Venezuela. Smithsonian Contributions to Zoology 330: 1–61. 10.5479/si.00810282.330

[B327] Flint Jr OS (1982a) Studies of Neotropical caddisflies, XXXI: Five new species from Argentina (Trichoptera). Entomological News 93: 43–47.

[B328] Flint Jr OS (1982b) Trichoptera of the area Platense. Biologia Acuatica 2: 1–70.

[B329] Flint Jr OS (1983) Studies of Neotropical caddisflies, XXXIII: New species from austral South America (Trichoptera). Smithsonian Contributions to Zoology 377(377): 1–100. 10.5479/si.00810282.377

[B330] Flint Jr OS (1990) Studies of Neotropical caddisflies, XLIII: Trichoptera collected in Chile by S. Jacquemart from 1975 to 1977. Bulletin de l’Institut Royal des Sciences Naturelles de Belgique Entomologie 60: 115–121.

[B331] Flint Jr OS (1991a) Studies of Neotropical caddisflies, XLIV: On a collection from Ilha de Maraca, Brazil. Acta Amazonica 21(0): 63–83. 10.1590/1809-43921991211083

[B332] Flint Jr OS (1991b) Studies of Neotropical caddisflies, XLV: The taxonomy, phenology, and faunistics of the Trichoptera of Antioquia, Colombia. Smithsonian Contributions to Zoology 520(520): 1–113. 10.5479/si.00810282.520

[B333] Flint Jr OS (1992a) New species of caddisflies from Puerto Rico (Trichoptera). Proceedings of the Entomological Society of Washington 94: 379–389.

[B334] Flint Jr OS (1992b) Studies of Neotropical caddisflies, XXXVIII: a review of the classification and biology of the Neotropical microcaddisflies, with the description of a new genus (Trichoptera: Hydroptilidae: Leucotrichiini). In: Quintero D Aiello A (Eds) Insects of Panama and Mesoamerica: Selected Studies.Oxford University Press, Oxford, 525–531.

[B335] Flint Jr OS (1996a) Checklist of the Trichoptera, caddisflies, of Cuba. Cocuyo 5: 15–20.

[B336] Flint Jr OS (1996b) Studies of Neotropical caddisflies LV: Trichoptera of Trinidad and Tobago. Transactions of the American Entomological Society 122: 67–113.

[B337] Flint Jr OS (1996c) The Trichoptera collected on the expeditions to Parque Manu, Madre de Dios, Peru. In: Wilson DE Sandoval A (Eds) Manu, the biodiversity of southeastern Peru.Smithsonian Institution, Washington, D. C., 369–430.

[B338] Flint Jr OS (2011) Trichoptera from the Great Falls and Turkey Run units of the George Washington Memorial Parkway, Fairfax Co., Virginia, USA. Zoosymposia 5(1): 101–107. 10.11646/zoosymposia.5.1.8

[B339] Flint Jr OS (2014) Caddisfly species new to, or rarely recorded from, the state of Virginia (Insecta: Trichoptera). Banisteria 43: 89–92.

[B340] Flint Jr OS Bueno-Soria J (1998) Studies of Neotropical caddisflies LVI: descriptions of five new species of the genus *Metrichia* Ross (Trichoptera: Hydroptilidae) from Pakitza, Peru, with a checklist and bibliography of the described species of the genus. Proceedings of the Entomological Society of Washington 100: 489–496.

[B341] Flint Jr OS Bueno-Soria J (1999) Studies of Neotropical caddisflies LVIII: new species of the genus *Ochrotrichia* Mosely (Trichoptera: Hydroptilidae) from Peru. Proceedings of the Entomological Society of Washington 101: 729–736.

[B342] Flint Jr OS Harris SC (1991[1992]) Studies of Neotropical caddisflies, XLII: *Taraxitrichiaamazonensis*, a new genus and species of microcaddisfly from Venezuela (Trichoptera: Hydroptilidae). In: Tomaszewski C (Ed.) Proceedings of the 6th International Symposium on Trichoptera.Adam Mickiewicz University Press, Poznan, Poland, 411–414.

[B343] Flint Jr OS Herrmann SJ (1976) The description of, and environmental characterization for, a new species of *Ochrotrichia* from Colorado (Trichoptera: Hydroptilidae). Annals of the Entomological Society of America 69(5): 894–898. 10.1093/aesa/69.5.894

[B344] Flint Jr OS Pérez-Gelabert DE (1999) Checklist of the Caddisflies (Trichoptera) of Hispaniola. Novitates Caribaea 1999: 33–46.

[B345] Flint Jr OS Reyes L (1991) Studies of Neotropical caddisflies, XLVI: The Trichoptera of the Río Moche Basin, Department of La Libertad, Peru. Proceedings of the Biological Society of Washington 104: 474–492.

[B346] Flint Jr OS Sykora JL (1993) New species and records of caddisflies (Insecta: Trichoptera) from the Lesser Antilles, with special reference to Grenada. Annals of the Carnegie Museum 62(1): 47–62. 10.5962/p.215118

[B347] Flint Jr OS Sykora JL (2004) Caddisflies of Hispaniola, with special reference to the Dominican Republic (Insecta: Trichoptera). Annals of the Carnegie Museum 73(1): 1–60. 10.5962/p.215150

[B348] Flint Jr OS Thomas A (2008) Long term evolution of caddisfly community in the river Arize at the Mas d’Azil cave (Prepyrenees, SW France). 1. preliminary results: 1963–1989 period (Trichoptera). Ephemera 10(1): 35–41. 10.5962/p.215150

[B349] Flint Jr OS Harris SC Botosaneanu L (1994) Studies of Neotropical caddisflies, L: the description of *Cerasmatrichia*, new genus, a relative of *Alisotrichia*, with the descriptions of new and old species and the larva (Trichoptera: Hydroptilidae). Proceedings of the Biological Society of Washington 107: 360–382.

[B350] Flint Jr OS Holzenthal RW Harris SC (1999a) Catalog of the Neotropical Caddisflies (Trichoptera). Columbus, Ohio: Special Publication, Ohio Biological Survey.

[B351] Flint Jr OS Holzenthal RW Harris SC (1999b) Nomenclatural and systematic changes in the Neotropical caddisflies. Insecta Mundi 13: 73–84.

[B352] Flint Jr OS Englund RA Kumashiro B (2003) A reassessment and new state records of Trichoptera occurring in Hawai’i with discussion on origins and potential ecological impacts. Bishop Museum, Occasional Papers 73: 31–40.

[B353] Flint Jr OS Hoffman RL Parker CR (2009) An annotated list of the caddisflies (Trichoptera) of Virginia: Part III. Emendations and biogeography. Banisteria 34: 3–16.

[B354] Floyd MA (1992) New microcaddisfly (Trichoptera: Hydroptilidae) records for Kentucky. Transactions of the Kentucky Academy of Science 53(1–2): 50.

[B355] Floyd MA Morse JC (1993) Caddisflies (Trichoptera) of Wildcat Creek, Pickens County, South Carolina. Entomological News 104: 171–179.

[B356] Floyd MA Schuster GA (1990) The caddisflies (Insecta: Trichoptera) of the Buck creek system, Pulaski County, Kentucky. Transactions of the Kentucky Academy of Science 51: 3–4.

[B357] Floyd MA Morse JC McArthur JV (1993) Aquatic insects of Upper Three Runs Creek, Savannah River Site, South Carolina. Part IV: Caddisflies (Trichoptera) of the lower reaches. Journal of Entomological Science 28(1): 85–95. 10.18474/0749-8004-28.1.85

[B358] Floyd MA Morse JC Harris SC (1997) Aquatic insects of Lake Jocassee catchment, North and South Carolina. Part II: Caddisflies (Trichoptera) of six additional drainages with a description of a new species. Journal of the Elisha Mitchell Scientific Society 113(3): 133–142.

[B359] Forsslund KH (1955) On the type of the genus *Hydroptila* Dalman (Trichoptera). Entomologisk Tidskrift 76: 125–126.

[B360] Frazer KS Harris SC (1991a) Cladistic analysis of the *Ochrotrichiashawnee* Group (Trichoptera: Hydroptilidae) and description of a new member from the Interior Highlands of northwestern Arkansas. Journal of the Kansas Entomological Society 64: 363–371.

[B361] Frazer KS Harris SC (1991b) New caddisflies (Trichoptera) from the Little River Drainage in northeastern Alabama. Bulletin of the American Museum of Natural History 11: 5–9.

[B362] Frazer KS Harris SC Ward GM (1991) Survey of the Trichoptera in the Little River Drainage of northeastern Alabama. Bulletin of the American Museum of Natural History 11: 17–22.

[B363] Gama Neto JL Passos MAB (2019) The genus *Neotrichia* Morton 1905 (Insecta: Trichoptera: Hydroptilidae) in Roraima state, Brazil: New records and descriptions of seven new species. Zootaxa 4695(6): 516–528. 10.11646/zootaxa.4695.6.2 31719323

[B364] Gama Neto JL Passos MAB (2020) Additional six new species of *Neotrichia* Morton 1905 (Insecta: Trichoptera: Hydroptilidae) from Roraima state, Brazil. Zootaxa 4881(1): 179–188. 10.11646/zootaxa.4881.1.11 33311136

[B365] Gama Neto JL Ribeiro JMF Passos MAB (2019) Two new species of Hydroptilidae (Insecta: Trichoptera) from the Serra dos Carajás, Pará state, northern Brazil. Zootaxa 4695(4): 385–390. 10.11646/zootaxa.4695.4.6 31719344

[B366] Gama Neto JL Ribeiro JMF Passos MAB (2020) Two new species of *Flintiella* Angrisano 1995 (Trichoptera: Hydroptilidae: Stactobiini) from northern Brazil. Zootaxa 4890(2): 283–288. 10.11646/zootaxa.4890.2.9 33311239

[B367] Gasith A Kugler J (1973) Bionomics of the Trichoptera of Lake Tiberias (Kinneret). Israel Journal of Entomology 8: 55–67.

[B368] GBIF The Global Biodiversity Informatino Facility (2022) GBIF Registry of Scientific Collections. https://www.gbif.org/grscicoll/collection/search [accessed 1 March 2022]

[B369] Gibon F-M (1985) Recherches sur les trichoptères d’Afrique occidentale. II. Stactobiini (Hydroptilidae) de Côte-D’Ivoire. Revue Française d’Entomologie 7: 149–155. [Nouvelle Serie]

[B370] Gibon F-M (1987a) Recherches sur les trichoptères d’Afrique occidental. 8. Hydroptilini (Hydroptilidae). Revue d’Hydrobiologie Tropicale 20: 121–130.

[B371] Gibon F-M (1987b) Studies on West African Trichoptera. 7. Two new *Catoxyethira* from Guinea (Hydroptilidae). Aquatic Insects 9(2): 115–118. 10.1080/01650428709361281

[B372] Gibon F-M (1991) Trichoptères d’Afrique Occidentale (XIII): Trois nouvelles *Catoxyethira* de Guinée. Revue Française d’Entomologie 13(3): 125–130. [Hydroptilidae] [Nouvelle Série]

[B373] Gibon F-M (1993) Trichoptères du Cameroun. Un nouvel exemple de la richesse des *Catoxyethira* (Hydroptilidae). Revue d’Hydrobiologie Tropicale 26(3): 199–211.

[B374] Gibon F-M (2019) Le genre *Stactobiella* à Madagascar (Trichoptera, Hydroptilidae). Bulletin de la Société Entomologique de France 124(2): 177–182. 10.32475/bsef_2080

[B375] Gibon F-M Ranaivoharindriaka F (1995) Présence du genre *Catoxyethira* à Madagascar et description de premières espèces. Revue Française d’Entomologie 17(3): 107–114. [Trichoptera, Hydroptilidae] [Nouvelle Serie]

[B376] Gibon F-M Guenda W Coulibaly B (1994) Observations sur la zonation des cours d’eau de la savane ouest-africaine: Trichoptères du Sud-Ouest du Burkina Faso. Annales de Limnologie 30(2): 101–121. 10.1051/limn/1994007

[B377] Giudicelli J Vaillant F (1967) La larve et la nymphe d’*Allotrichiapallicornis* Eaton (Trichoptera). Travaux du Laboratoire d’Hydrobiologie et de Pisciculture de l’Université de Grenoble 57–58(1965): 29–36.

[B378] Givens DR (2014) An annotated list of caddisflies (Trichoptera) collected in Lassen Volcanic National Park, California, USA during 2011–2013. Entomological News 124(3): 153–175. 10.3157/021.124.0301

[B379] Glapska G (1986) Caddisflies (Trichoptera) of the rivers in the loess margins of the Holy Cross Mountains (Świętokryzyskie Mountains). Fragmenta Faunistica (Warsaw) 30(2): 25–33. 10.3161/00159301FF1986.30.2.025

[B380] Gombeer SC Knapen D Bervoets L (2011a) The influence of different spatial-scale variables on caddisfly assemblages in Flemish lowland streams. Ecological Entomology 36(3): 355–368. 10.1111/j.1365-2311.2011.01280.x

[B381] Gombeer SC Knapen D Bervoets L (2011b) Trichoptera in Flanders (Belgium): An ecological and phylogenetic characterization of the order. Zoosymposia 5(1): 108–114. 10.11646/zoosymposia.5.1.9

[B382] González MA Cobo F (1994) Description of *Hydroptilaandalusiaca* sp.n. (Trichoptera, Hydroptilidae) from Spain. Aquatic Insects 16(4): 253–255. 10.1080/01650429409361562

[B383] González MA Terra LSW (1981) Una nueva especie del genero *Stactobia* en la peninsula Ibérica (Trichoptera, Hydroptilidae). Nouvelle Revue d’Entomologie 11(2): 203–206.

[B384] González MA Terra LSW (1982) Una nueva especie del genero *Oxyethira*, *O.iglesiasi*, en la peninsula Ibérica (Trichoptera: Hydroptilidae). Nouvelle Revue d’Entomologie 12(3): 299–302.

[B385] González MA Malicky H (1980) Eine neue *Allotrichia* (Trichoptera: Hydroptilidae) von der Iberischen Halbins Entomologische Zeitschrift 90(19): 214–216.

[B386] González MA Malicky H (1988) Description de quatre nouvelles espèces de trichoptères de l’Espagne et du Maroc (Trichoptera). Mitteilungen der Entomologischen Gesellschaft Basel 38: 66–71.

[B387] González M Martínez Menéndez J (2008) Observaciones sobre los Trichopteros de la Peninsula Ibérica. X: Tricópteros de Aragón (NE de España) (Insecta: Trichoptera). Boletin de la SEA 43: 187–192.

[B388] González MA Martínez Menéndez J (2011) Checklist of the caddisflies of the Iberian Peninsula and Balearic Islands (Trichoptera). Zoosymposia 5(1): 115–135. 10.11646/zoosymposia.5.1.10

[B389] González MA Otero JC (1983) Observaciones sobre los Tricopteros de la Península Ibérica. 4. Tricópteros de Caceres (oeste de España). Descripción de *Cyrnusmonserrati* n. sp. (Trichoptera: Polycentropodidae). Nouvelle Revue d’Entomologie 13(1): 117–124.

[B390] González MA Valiela J González T (1986) Observationes sobre los Trichopteros de la Peninsula Iberica. VII: Sierra Segundera (Noroeste de España). Trabajos Compostelanos de Biología 13: 109–118.

[B391] González MA Cobo F Iglesias JC (1990) Observaciones sobre los tricopteros de la Peninsula Iberica. 9: Provincias de Cadíz y Huelva, Suroeste de España. (Insecta: Trichoptera). Boletin de la Asociacion Espanola de Entomologia 14: 211–218.

[B392] González MA Vieira-Lanero R Cobo F (2000) The immature stages of *Ptilocolepusextensus* McLachlan, 1884 (Trichoptera: Hydroptilidae: Ptilocolepinae) with notes on biology. Aquatic Insects 22(1): 27–38. 10.1076/0165-0424(200001)22:1;1-Z;FT027

[B393] González MA Martínez J Ruíz A (2013) Two new species of caddisflies (Trichoptera: Hydroptilidae, Psychomyiidae) from central and south Spain. Zootaxa 3664(3): 397–400. 10.11646/zootaxa.3664.3.10 26266311

[B394] Graf W Hutter G (2004) Köcherfliegen aus Vorarlberg II- Beitrag zur Kenntnis der Trichopteren des Alten Rheins - ein Vergleich zweier ökomorphologisch unterschiedlicher Standorte. Vorarlberger Naturschau Forschen und Entdecken 14: 143–152.

[B395] Graf W Leitner P (2016) Biodiversität im Stadtgebiet von Klagenfurt: Das Natura 2000 - Gebiet Lendspitz-Maiernigg - Ergebnisse des GEO-Tags der Artenvielfalt 2015. Von *Hydroptiladampfi* und *Caenisrobusta*: Eintagsfliegen und Köcherfliegen. Carinthia II 206(126): 37.

[B396] Graf W Waringer J (2002) The larva of *Stactobiellarisi* (Felber, 1908) (Trichoptera: Hydroptilidae). Nova Supplementa Entomologica (Proceedings of the 10^th^ International Symposium on Trichoptera) 15: 420–424.

[B397] Graf W Schmidt-Kloiber A Moritz C (1998) Köcherfliegenfunde aus Österreich. Lauterbornia 34: 205–213.

[B398] Graf W Waringer J Zika-Römer J (2004) The larva of *Microptilaminutissima* Ris, 1897 (Trichoptera: Hydroptilidae). Aquatic Insects 26(1): 31–38. 10.1076/aqin.26.1.31.35374

[B399] Graf W Hutter G Schmidt-Kloiber A (2005) Ein Beitrag zur Kenntnis der Köcherfliegen (Trichoptera) Vorarlbergs. Lauterbornia 54: 53–61.

[B400] Graf W Heckes U Hess M Zweidick O Malicky H (2017) Neue Nachweise von Köcherfliegen (Insecta: Trichoptera) aus Österreich. Braueria 44: 48–49.

[B401] Guahyba RR (1991) Estágios imaturos de *Anchitrichiaduplifurcata* Flint, 1983 (Trichoptera, Hydroptilidae). Revista Brasileira de Entomologia 35(1): 121–125.

[B402] Guenda W (1996) Contribution à l’étude des Hydroptilidae (Insecta: Trichoptera) de l’Afrique de l’Ouest: le genre *Orthotrichia* Eaton de la rivière Mouhoun (Burkina Faso). Annales de Limnologie 32(4): 241–249. 10.1051/limn/1996023

[B403] Guenda W (1997) Nouvelles especes du genre *Catoxyethira* Ulmer du Burkina Faso (Trichoptera, Hydroptilidae). Bulletin de la Société Entomologique de France 102(3): 217–224. 10.3406/bsef.1997.17334

[B404] Guinard E (1878 [1879]) Metamorphose d’un genre nouveau de Phryganide (*LeiochitonFagesii*). Académie des Sciences et Lettres de Montpellier. Mémoires de la Section des Sciences 9: 139–144.

[B405] Gullefors B (1989) *Oxyethirafalcata* Morton, 1893 (Trichoptera, Hydroptilidae), a caddis fly species new to Sweden. Entomologisk Tidskrift 110(3): 119–120.

[B406] Gullefors B (2001) *Oxyethiraklingstedti* (Trichoptera, Hydroptilidae), en for Sverige ny nattslanda. Entomologisk Tidskrift 122: 188.

[B407] Gullefors B (2002) Sveriges nattslandor (Trichoptera), en provinskatalog med nyare fynduppgifter. Entomologisk Tidskrift 123: 131–147.

[B408] Gullefors B (2003) Nya svenska provinsfynd av nattslandor (Trichoptera). Entomologisk Tidskrift 124(3): 193–199.

[B409] Gullefors B (2005a) Nya provinsfynd av nattslandor (Trichoptera) i Sverige 2004. Entomologisk Tidskrift 126(3): 117–120.

[B410] Gullefors B (2005b) Trichoptera from the brackish water of the Gulf of Bothnia. Proceedings of the 11^th^ International Symposium on Trichoptera. K. Tanida and A. Rossiter. Kanagawa, Tokai University Press, 137–147.

[B411] Gullefors B (2006) *Hydroptilalotensis* Mosely, 1920, en ny nattslanda (Trichoptera) for Sverige och nya provinsfynd av nattslandor 2003–2005. Entomologisk Tidskrift 127(3): 135–141.

[B412] Gullefors B (2008) Limes norrlandicus - a natural biogeographical border for caddisflies (Trichoptera) in Sweden. Ferrantia 55: 61–65.

[B413] Gullefors B (2016) Sveriges nattsländor (Trichoptera), utbredning, vanlighetsgrad, habitat och flygtider. The Swedish caddisflies (Trichoptera), distribution, frequency, habitat and flight times. Entomologisk Tidskrift 136(4): 137–146.

[B414] Gullefors B (2018) Är svärmande nattsländor (Trichoptera) starkare flygare än icke-svärmande? Entomologisk Tidskrift 139(2): 99–110. [Are swarming caddisflies (Trichoptera) stronger flyers than non-swarming?]

[B415] Gullefors B Johanson KA (2007) Gotlands nattslandor (Trichoptera). Entomologisk Tidskrift 128: 61–70.

[B416] Haase P (1994) Neue Vorkommen von *Hydroptilasparsa* Curtis, 1834, *Orthotrichiacostalis* (Curtis, 1834) (Trichoptera, Hydroptilidae) und *Caenisbeskidensis* Sowa, 1973 (Ephemeroptera, Caenidae) im niedersachsischen Hugel- und Bergland. Entomologische Nachrichten und Berichte 38(3): 206.

[B417] Hagen HA (1855) Versuch, die Phryganiden Pictet’s zu bestimmen. Stettiner Entomologische Zeitung 16: 204–210.

[B418] Hagen HA (1859) Synopsis der Neuroptera Ceylons (Pars II). Verhandlungen der Kaiserlich-Königlichen Zoologischen-Botanischen Gesellschaft in Wien 9: 199–212.

[B419] Hagen HA (1861) Synopsis of the Neuroptera of North America with a list of the South American species. Smithsonian Institution Miscellaneous Collections 4(1): 1–347. 10.5962/bhl.title.60275

[B420] Hagen HA (1864a) Phryganidarum synopsis synonymica. Verhandlungen der Kaiserlich-Königlichen Zoologischen-Botanischen Gesellschaft in Wien 14: 799–890.

[B421] Hagen HA (1864b) Ueber Phryganiden-Gehäuse. Stettiner Entomologische Zeitung 25: 113–144, 221–262.

[B422] Hagen HA (1865a) Beiträge zur Kenntnis der Phryganiden. Stettiner Entomologische Zeitung 26: 205–214, 217–233.

[B423] Hagen HA (1865b) Neuroptera of Maderia. Entomologist’s Monthly Magazine 2: 8–11, 25–28, 59–62, 75–81.

[B424] Hagen HA (1887) Ueber *Plethuscursitans*. Verhandlungen der Zoologisch-Botanischen Gesellschaft in Wein 37: 643–645.

[B425] Hamilton SW Holzenthal RW (1986) Two new species of caddisflies from Georgia (Trichoptera: Polycentropodidae, Hydroptilidae). Proceedings of the Entomological Society of Washington 88(1): 163–166.

[B426] Hamilton RW Buttner JK Brunetti RG (1975) Lethal levels of sodium chloride and potassium chloride for an oligochaete, a chironomid midge, and a caddisfly of Lake Michigan. Environmental Entomology 4(6): 1003–1006. 10.1093/ee/4.6.1003

[B427] Hamilton SW Schuster GA DuBois MB (1983) Checklist of the Trichoptera of Kansas. Transactions of the Kansas Academy of Science 86(1): 10–23. 10.2307/3628419

[B428] Hanna HM (1961) The larvae of *Hydroptilasparsa* Curtis (Trichoptera: Hydroptilidae). Entomologist’s Gazette 12: 69–75.

[B429] Hansen LJ Gíslason GM (2020) Trichoptera in the Faroe Islands. Zoosymposia 18(1): 127–134. 10.11646/zoosymposia.18.1.16

[B430] Harper PP (1973) *Hydroptilaeramosa* a new caddis fly from Southern Ontario (Trichoptera, Hydroptilidae). Canadian Journal of Zoology 51(3): 393–394. 10.1139/z73-055

[B431] Harper PP (1976) *Oxyethirabarnstoni* n. sp. un nouveau trichoptire de Radissonie, Quebec (Hydroptilides). Annales de la Société Entomologique de Quebec 21(1): 35–38.

[B432] Harper PP (1989) Zoological relationships of aquatic insects (Ephemeroptera, Plecoptera and Trichoptera) from the eastern James Bay drainage. Canadian Field Naturalist 103: 535–546.

[B433] Harper PP (1990) Associations of aquatic insects (Ephemeroptera, Plecoptera, and Trichoptera) in a network of subarctic lakes and streams in Quebec. Hydrobiologia 199(1): 43–64. 10.1007/BF00007833

[B434] Harper PP Turcotte P (1985) New Ecuadorian Trichoptera. Aquatic Insects 7(3): 133–140. 10.1080/01650428509361212

[B435] Harris SC (1985a) New Hydroptilidae (Trichoptera) from Alabama. Journal of the Kansas Entomological Society 58: 248–253.

[B436] Harris SC (1985b) New microcaddisflies (Trichoptera: Hydroptilidae) from Alabama. Proceedings of the Entomological Society of Washington 87(3): 606–621.

[B437] Harris SC (1986a) Hydroptilidae (Trichoptera) of Alabama with descriptions of three new species. Journal of the Kansas Entomological Society 59(4): 609–619.

[B438] Harris SC (1986b) New species of caddisflies (Trichoptera) from Alabama. Proceedings of the Entomological Society of Washington 88(1): 30–41.

[B439] Harris SC (1989) New Trichoptera from Alabama. Journal of the New York Entomological Society 97(3): 309–316.

[B440] Harris SC (1990) New species of *Neotrichia* (Trichoptera: Hydroptilidae) from Central and South America. Journal of the New York Entomological Society 98: 246–260.

[B441] Harris SC (1991) New caddisflies (Trichoptera) from Alabama and Florida. Bulletin of the American Museum of Natural History 11: 11–16.

[B442] Harris SC (1994) Proposed replacement name for *Hydroptilasetigera* (Trichoptera: Hydroptilidae). Entomological News 105(5): 284.

[B443] Harris SC (2002) New species of microcaddisflies (Trichoptera: Hydroptilidae) from northern Florida. Annals of the Carnegie Museum 71(1): 47–57. 10.5962/p.215805

[B444] Harris SC Armitage BJ (1987) New Hydroptilidae (Trichoptera) from Florida. Entomological News 98(3): 106–110.

[B445] Harris SC Armitage BJ (1997) New member of the Chilean genus *Nothotrichia* from North America (Trichoptera: Hydroptilidae). In: Holzenthal RW Flint Jr OS (Eds) Proceedings of the 8th International Symposium on Trichoptera.Ohio Biological Survey, Columbus, 123–128.

[B446] Harris SC Armitage BJ (2015) The Trichoptera of Panama. II. Ten new species of microcaddisflies (Trichoptera: Hydroptilidae). Insecta Mundi 0437: 1–17.

[B447] Harris SC Armitage BJ (2019) The Trichoptera of Panama X. The Quebrada Rambala drainage, with description of 19 new species of microcaddisflies (Trichoptera: Hydroptilidae). Insecta Mundi 0707: 1–54.

[B448] Harris SC Bueno-Soria J (1993) *Scelobotrichia*, a new genus of microcaddisflies from Mexico (Trichoptera: Hydroptilidae). Folia Entomologica Mexicana 87: 73–83.

[B449] Harris SC Contreras-Ramos A (1989) *Ithytrichiamexicana* (Trichoptera: Hydroptilidae), a new species of caddisfly from Mexico. Entomological News 100: 176–178.

[B450] Harris SC Davenport LJ (1992) New species of microcaddisflies from the Amazon region, with especial reference to northeastern Peru (Trichoptera: Hydroptilidae). Proceedings of the Entomological Society of Washington 94: 454–470.

[B451] Harris SC Davenport LJ (1999) New species of Hydroptilidae (Trichoptera) from the Amazon region of northeastern Peru. Proceedings of the Entomological Society of Washington 101: 26–38.

[B452] Harris SC Etnier DE (1994) A new synonym in *Hydroptila* (Trichoptera: Hydroptilidae). Entomological News 105: 262.

[B453] Harris SC Flint Jr OS (1992) Studies of Neotropical caddisflies, XLVII; *Kumanskiella*, a new genus from Cuba and Puerto Rico. Journal of the New York Entomological Society 100: 581–593.

[B454] Harris SC Flint Jr OS (1993) Studies of Neotropical caddisflies, XLVIII; the larva of *Celaenotrichiaedwardsi* Mosely, with an assessment of the genus (Trichoptera: Hydroptilidae). In: Otto C (Ed.) Proceedings of the 7th International Symposium on Trichoptera.Backhuys Publishers, Leiden, The Netherlands, 101–106.

[B455] Harris SC Flint Jr OS (2002) New *Alisotrichia* (Trichoptera: Hydroptilidae) from Central and South America and the Greater Antilles. Proceedings of the Entomological Society of Washington 104: 195–210.

[B456] Harris SC Flint Jr OS (2016) New species of microcaddisflies (Trichoptera: Hydroptilidae) from the western United States, Canda, Mexico, and Belize. Insecta Mundi 0499: 1–22.

[B457] Harris SC Holzenthal RW (1990) Hydroptilidae (Trichoptera) from Costa Rica: The genus *Mayatrichia* Mosely. Journal of the New York Entomological Society 98: 453–460.

[B458] Harris SC Holzenthal RW (1993) Phylogeny of the species groups of *Alisotrichia*, *sensu lato*, with the description of a new species from Costa Rica (Trichoptera: Hydroptilidae). In: Otto C (Ed.) Proceedings of the 7th International Symposium on Trichoptera.Backhuys Publishers, Leiden, The Netherlands, 155–160.

[B459] Harris SC Holzenthal RW (1994) Hydroptilidae (Trichoptera) of Costa Rica and the Neotropics: Systematics of the genus *Byrsopteryx* Flint (Stactobiini). Journal of the New York Entomological Society 102: 154–192.

[B460] Harris SC Holzenthal RW (1997) *Mejicanotrichia*, a new genus of microcaddisflies from Mexico and Guatemala (Trichoptera: Hydroptilidae). In: Holzenthal RW Flint Jr OS (Eds) Proceedings of the 8th International Symposium on Trichoptera.Ohio Biological Survey, Columbus, 123–128.

[B461] Harris SC Holzenthal RW (1999) Hydroptilidae (Trichoptera) of Costa Rica: The genus *Hydroptila* Dalman. Studies on Neotropical Fauna and Environment 34(1): 16–51. 10.1076/snfe.34.1.16.8916

[B462] Harris SC Huryn AD (2000) New and rare microcaddisflies (Trichoptera: Hydroptilidae) from the eastern United States. Entomological News 111: 77–83.

[B463] Harris SC Kelley RW (1984) New species of Hydroptilidae (Trichoptera) from Alabama. Proceedings of the Entomological Society of Washington 86(3): 572–577.

[B464] Harris SC Keth AC (2002) Two new microcaddisflies (Trichoptera: Hydroptilidae) from Alabama and Florida. Entomological News 113: 73–79.

[B465] Harris SC Moulton II SR (1993) New species of Ochrotrichia (Ochrotrichia) from the southwestern United States and northern Mexico. Journal of the New York Entomological Society 101(4): 542–549.

[B466] Harris SC Rasmussen AK (2010) The *Neotrichiacaxima* Group (Trichoptera: Hydroptilidae) in the southeastern United States. Zootaxa 2608: 25–44. 10.11646/zootaxa.2608.1.2

[B467] Harris SC Rasmussen AK (2019) Review of the *Orthotrichia* (Trichoptera: Hydroptilidae) of Florida, with descriptions of previously unknown females of three species. Zoosymposia 14(1): 215–230. 10.11646/zoosymposia.14.1.24

[B468] Harris SC Sykora JL (1996) New species of microcaddisflies from the eastern United States (Insecta: Trichoptera: Hydroptilidae). Annals of the Carnegie Museum 65(1): 17–25. 10.5962/p.215132

[B469] Harris SC Tiemann SG (1993) New species on *Neotrichia* from Texas and Panama, with a preliminary review of the *N.canixa* group (Trichoptera: Hydroptilidae). Proceedings of the Entomological Society of Washington 95: 286–292.

[B470] Harris SC Lago PK Holzenthal RW (1982a) An annotated checklist of the caddisflies (Trichoptera) of Mississippi and Southeastern Louisiana. Part II: Rhyacophiloidea. Proceedings of the Entomological Society of Washington 84: 509–512.

[B471] Harris SC Lago PK Scheiring JF (1982b) An annotated list of Trichoptera of several streams on Eglin Air Force Base, Florida. Entomological News 93: 79–84.

[B472] Harris SC Lago PK O’Neil PE (1984) Trichoptera of the Cahaba River system in Alabama. Entomological News 95: 103–112.

[B473] Harris SC O’Neil PE Lago PK (1991) Caddisflies of Alabama. Geological Survey of Alabama Bulletin 142: 1–442.

[B474] Harris SC Kondratieff BC Stark BP (1996) New records of Ephemeroptera, Plecoptera and Trichoptera from Alabama. Entomological News 107: 237–242.

[B475] Harris SC Pescador ML Rasmussen AK (1998) Two new species of microcaddisflies (Trichoptera: Hydroptilidae) from Northern Florida. The Florida Entomologist 81(2): 221–224. 10.2307/3496090

[B476] Harris SC Holzenthal RW Flint Jr OS (2002) Review of the Neotropical genus *Bredinia* (Trichoptera: Hydroptilidae: Stactobiini). Annals of the Carnegie Museum 71(1): 13–45. 10.5962/p.215804

[B477] Harris SC Flint Jr OS Holzenthal RW (2002a) Review of the Neotropical genus *Flintiella* (Trichoptera: Hydroptilidae: Stactobiini). Journal of the New York Entomological Society 110(1): 65–90. 10.1664/0028-7199(2002)110[0065:ROTNGF]2.0.CO;2

[B478] Harris SC Flint Jr OS Holzenthal RW (2002b) Two new genera of Hydroptilidae from the neotropics (Trichoptera: Hydroptilidae: Stactobiini). Journal of the New York Entomological Society 110: 49–64. 10.1664/0028-7199(2002)110[0049:TNGOHF]2.0.CO;2

[B479] Harris SC Rasmussen AK Denson DR (2012) An annotated list of the caddisflies (Trichoptera) of Florida: Part I. The family Hydroptilidae, with descriptions of five new species. Insecta Mundi 273: 1–32.

[B480] Hart DD (1985a) Causes and consequences of territoriality in a grazing stream insect. Ecology 66(2): 404–414. 10.2307/1940390

[B481] Hart DD (1985b) Grazing insects mediate algal interactions in a stream benthic community. Oikos 44(1): 40–46. 10.2307/3544041

[B482] Hart DD (1992) Community organization in streams: the importance of species interactions, physical factors, and chance. Oecologia 91: 220–228. 10.1007/BF00317787 28313460

[B483] Hart DD Robinson CT (1990) Resource limitation in a stream community: Phosphorous enrichment effects on periphyton and grazers. Ecology 71(4): 1494–1502. 10.2307/1938286

[B484] Hart DD Kohler SL Carlton RG (1991) Harvesting of benthic algae by territorial grazers: The potential for prudent predation. Oikos 60(3): 329–335. 10.2307/3545075

[B485] Henriksen KL (1937) XXXVIII. Planipennia and Trichoptera. In: Jensen AS Lundbeck W Mortensen T Späck R (Eds) The Zoology of the Faroes.Andr. Fred. Høst & Son, Copenhagen, 1–11.

[B486] Hickin NE (1967) Caddis larvae: larvae of the British Trichoptera. Hutchinson, London, 476 pp.

[B487] Higler LWG (1974) *Oxyethirafischeri* n. sp. A new *Oxyethira* species from Madeira (Trichoptera: Hydroptilidae). Entomologische Berichten 34: 62–63.

[B488] Hiilivirta P (1982) *Tricholeiochitonfagesii* (Guinard) (Hydroptilidae) new for Finland. Notulae Entomologicae 62: 154.

[B489] Hinchliffe RP (2010) First record of *Ithytrichia* (Trichoptera: Hydroptilidae) in Alberta, Canada. Entomological News 121(5): 466–468. 10.3157/021.121.0508

[B490] Hirabayashi K Kimura G Inoue E (2011) Adult caddisflies (Trichoptera) attracted to artificial lights in the middle reaches of the Shinano River from 2005 to 2007. Zoosymposia 5(1): 143–146. 10.11646/zoosymposia.5.1.12

[B491] Hohmann M (1998) Bemerkenswerte Köcherfliegen-Fange (Insecta, Trichoptera) im Tiefland Sachsen-Anhalts. Lauterbornia 34: 73.

[B492] Hohmann M (1999) Bemerkenswerte Köcherfliegen-Fänge (Insecta, Trichoptera) im Tiefland Sachsen-Anhalts. Lauterbornia 36: 33–40.

[B493] Hohmann M (2005) Die Köcherfliegen-Fauna (Trichoptera) der Dübener Heide, Sachsen-Anhalt. Lauterbornia 54: 103–114.

[B494] Hohmann M (2010) Ein Beitrag zur Kenntnis der Eintags-, Stein- und Köcherfliegen (Insecta: Ephemeroptera, Plecoptera, Trichoptera) im Nationalpark Harz, Sachsen-Anhalt. Entomologische Mitteilungen Sachsen-Anhalt Sonderheft 2: 34–54.

[B495] Hohmann M Brauns M Jährling M Kleinsteuber W Tappenbeck L (2006) Neu - und Wiederfunde von Köcherfliegen (Insecta, Trichoptera) in Sachsen-Anhalt seit 1994. Abhandlungen und Berichte für Naturkunde 29: 105–124.

[B496] Hohmann M Kleinsteuber W Spitzenberg D (2014) Information about aquatic insects (Ephemeroptera, Plecoptera, Heteroptera, Coleoptera, Trichoptera) of nature reserve ‘Okertal’ near Wulperode (district Harz/Saxony-Anhalt). Abhandlungen und Berichte aus dem Museum Heineanum 10: 71–91.

[B497] Holmes PR Boyce DC Reed DK Wallace ID (1992) *Oxyethiramirabilis* Morton (Trichopt., Hydroptilidae) found in Wales. Entomologist’s Monthly Magazine 128(1540–1543): 202.

[B498] Holzenthal RW (1988) [1989]) Catalogo systematico de los Trichopteros de Costa Rica (Insecta: Trichoptera). Brenesia 29: 51–82.

[B499] Holzenthal RW Calor AR (2017) Catalogo of the Neotropical Trichoptera (Caddisflies). ZooKeys 654: 1–566. 10.3897/zookeys.654.9516 PMC5345355 28331396

[B500] Holzenthal RW Harris SC (1991) The larva of *Byrsopteryxmirifica* Flint, with an assessment of the phylogenetic placement of the genus within the Leuchotrichiini (Trichoptera: Hydroptilidae). In: Tomaszewski C (Ed.) Proceedings of the 6th International Symposium on Trichoptera.Adam Mickiewicz University Press, Poznan, Poland, 403–407.

[B501] Holzenthal RW Harris SC (1992) Hydroptilidae (Trichoptera) of Costa Rica: The genus *Oxyethira* Eaton. Journal of the New York Entomological Society 100: 155–177.

[B502] Holzenthal RW Harris SC (1999) The genus *Costatrichia* Mosely in Costa Rica, with a review of the Neotropical species (Trichoptera: Hydroptilidae). Proceedings of the Entomological Society of Washington 101: 540–568.

[B503] Holzenthal RW Harris SC (2002) New species of *Nothotrichia* Flint (Trichoptera: Hydroptilidae) from Brazil and Costa Rica. Proceedings of the Entomological Society of Washington 104: 106–110.

[B504] Holzenthal RW Kelley RW (1983) New micro-caddisflies from the southeastern United States (Trichoptera: Hydroptilidae). The Florida Entomologist 66(4): 464–472. 10.2307/3494017

[B505] Holzenthal RW Blahnik RJ Kjer KM Prather AP (2007a) An update on the phylogeny of caddisflies (Trichoptera). In: Bueno-Soria J Barba-Alvarez R Armitage B (Eds) Proceedings of the 12th International Symposium on Trichoptera.The Caddis Press, Columbus, Ohio, 143–153.

[B506] Holzenthal RW Blahnik RJ Prather AL Kjer KM (2007b) Order Trichoptera Kirby, 1813 (Insecta), caddisflies. Zootaxa 1668(1): 639–698. 10.11646/zootaxa.1668.1.29

[B507] Houghton DC (2001) Caddisfly (Trichoptera) records from the Apache National Forest, eastern Arizona. Entomological News 112: 85–93.

[B508] Houghton DC (2016) The caddisflies (Trichoptera) of an undisturbed lower Michigan habitat. Great Lakes Entomologist 49(1–2): 41–54.

[B509] Houghton DC (2020) New state species records and noteworthy re-captures of Michigan (USA) Trichoptera. Great Lakes Entomologist 53(1–2): 47–52.

[B510] Houghton DC Holzenthal RW (2003) Updated conservation status of protected Minnesota caddisflies. Great Lakes Entomologist 36: 35–40.

[B511] Houghton DC Holzenthal RW (2010) Historical and contemporary biological diversity of Minnesota caddisflies: A case study of landscape-level species loss and trophic composition shift. Journal of the North American Benthological Society 29(2): 480–495. 10.1899/09-029.1

[B512] Houghton DC Lardner R (2020) Ash-free dry mass values for northcentral USA caddisflies (Insecta, Trichoptera). ZooKeys 951: 37–46. 10.3897/zookeys.951.49790 32774104 PMC7390801

[B513] Houghton DC Stewart KW (1998) Seasonal flight distribution of six microcaddisflies (Trichoptera: Hydroptilidae, Glossosomatidae) in the Brazos River, Texas, with notes on larval biology and site records. Entomological News 109: 103–109.

[B514] Houghton DC Holzenthal RW Monson MP MacLean DB (2001) Updated checklist of the Minnesota caddisflies (Tricoptera [Trichoptera]) with geographic affinities. Transactions of the American Entomological Society 127: 495–512.

[B515] Houghton DC Berry EA Gilchrist A Thompson J Nussbaum MA (2011a) Biological changes along the continuum of an agricultural stream: Influence of a small terrestrial preserve and use of adult caddisflies in biomonitoring. Journal of Freshwater Ecology 26(3): 381–397. 10.1080/02705060.2011.563513

[B516] Houghton DC Brandin CM Brakel KA (2011b) Analysis of the caddisflies (Trichoptera) of the Manistee River watershed, Michigan. Great Lakes Entomologist 44: 1–15.

[B517] Houghton DC Brandin CM Reynolds L Elzinga LL (2013) Discontinuity in the Insect Assemblages of a Northern Lower Michigan Stream. Great Lakes Entomologist 46(1–2): 31–41.

[B518] Houghton DC DeWalt RE Pytel AJ Brand CM Rogers SE Ruiter DE Bright E Hudson PL Armitage BJ (2017) Updated checklist of the Michigan (USA) caddisflies, with regional and habitat affinities. ZooKeys 730: 57–74. 10.3897/zookeys.730.21776 PMC5799788 29416396

[B519] Houp RE (1999) New caddisfly (Trichoptera) records from Kentucky with implications for water quality. Journal of the Kentucky Academy of Science 60: 1–3.

[B520] Houp RE Houp KH Harris SC (1998) Two new species of microcaddisflies (Trichoptera: Hydroptilidae) from Kentucky. Entomological News 109: 99–102.

[B521] Hsu L-P Chen C-S (2002) A new species of *Ugandatrichia* (Trichoptera: Hydroptilidae) from Taiwan. The Pan-Pacific Entomologist 78: 74–79.

[B522] Huang X-y Zhang J-h Wang W (2005) Studies on Trichoptera in Xinjiang. Shihezi Daxue Xuebao. Ziran Kexue Ban 23(4): 468–472.

[B523] Hudson GV (1886) On the metamorphosis of the caddis fly. Transactions of the New Zealand Institute 18: 213–214.

[B524] Hughes SJ (2006) Temporal and spatial distribution patterns of larval Trichoptera in Madeiran streams. Hydrobiologia 553(1): 27–41. 10.1007/s10750-005-0627-1

[B525] Hunt AS (2017) List of Rhode Island caddisflies (Trichoptera) with new records from Block Island. Entomological News 127(2): 107–111. 10.3157/021.127.0204

[B526] Huryn AD (1983) A description of the female of *Hydroptilajackmanni* Blickle (Trichoptera: Hydroptilidae), with biological notes. Entomological News 94(3): 93–94.

[B527] Huryn AD (1985) A new species of *Hydroptila* (Trichoptera: Hydroptilidae) from North Carolina. Proceedings of the Entomological Society of Washington 87(2): 444–447.

[B528] Huryn AD Foote BA (1983) An annotated list of the caddisflies (Trichoptera) of Ohio. Proceedings of the Entomological Society of Washington 85: 783–796.

[B529] Huryn AD Harris SC (2000) High species richness of caddisflies (Trichoptera) from a riparian wetland in Maine. Northeastern Naturalist 7(3): 189–204. 10.1656/1092-6194(2000)007[0189:HSROCT]2.0.CO;2

[B530] Hynes HBN (1970) The ecology of running waters. Liverpool, 555 pp.

[B531] Ibrahimi H Kučinić M Gashi A Grapci-Kotori L (2012) The caddisfly fauna (Insecta, Trichoptera) of the rivers of the Black Sea basin in Kosovo with distributional data for some rare species. ZooKeys 182: 71–85. 10.3897/zookeys.182.2485 PMC3337045 22539915

[B532] Ibrahimi H Jahiji E Bilalli A (2017) New records for the caddisfly (Insecta: Trichoptera) fauna of Serbia. Entomological News 127(3): 185–191. 10.3157/021.127.0302

[B533] Isa Miranda ÁV Rueda Martín PA (2014) El Orden Trichoptera en Tucumán, Argentina: nuevo registro de Leucotrichia lerma (Angrisano y Burgos, 2002) (Trichoptera: Hydroptilidae), descripción de sus estados inmaduros, lista de especies y claves de identificación ilustradas. Acta Zoológica Lilloana 58: 194–223.

[B534] Ito T (1988) Life histories of *Paleagapetusovatus* and *Eubasilissaregina* (Trichoptera) in a spring stream, with special reference to the predator-prey relationship. Kontyû, Tokyo 56: 148–160.

[B535] Ito T (1991a) Description of a new species of *Palaeagapetus* from central Japan, with notes on bionomics (Trichoptera, Hydroptilidae). Japanese Journal of Entomology 59: 357–366.

[B536] Ito T (1991b) Morphology and bionomics of *Palaeagapetusflexus* n. sp. from northern Japan (Trichoptera: Hydroptilidae). In: Tomaszewski C (Ed.) Proceedings of the 6th International Symposium on Trichoptera.Adam Mickiewicz University Press, Poznan, Poland, 419–426.

[B537] Ito T (1997) Oviposition preference and behavior of hatched larvae of an oligophagous caddisfly, *Palaegapetusovatus* (Hydroptilidae: Ptilocolepinae). Holzenthal RW, Flint Jr OS (Eds) Proceedings of the 8^th^ International Symposium on Trichoptera. Ohio Biological Survey, Columbus, 177–181.

[B538] Ito T (1998) The biology of the primitive, distinctly crenophilic caddisflies, Ptilocolepinae (Trichoptera, Hydroptilidae). A review. In: Botosaneanu L (Ed.) Studies in Crenobiology - The Biology of Springs and Springbrooks.Backhuys Publishers, Leiden, The Netherlands, 85–94.

[B539] Ito T (2010) A new species of the genus *Palaeagapetus* Ulmer (Trichoptera, Hydroptilidae) from Japan. Limnology 11(1): 1–3. 10.1007/s10201-009-0276-6

[B540] Ito T (2013) The genus *Orthotrichia* Eaton (Trichoptera, Hydroptilidae) in Japan. In: Tojo K Tanida K Nozaki T (Eds) Proceedings of the 1st Symposium of the Benthological Society of Asia. Japan, Scientific Research Society of Inland Water Biology. Biology of Inland Waters Supplement No.2: 39–47.

[B541] Ito T (2015) The Genus *Hydroptila* Dalman (Trichoptera: Hydroptilidae) in the Ryukyu Islands, Southwestern Japan. Entomological Research Bulletin. The Entomological Society of Korea 31(1): 7–17.

[B542] Ito T (2017a) The genus *Microptila* Ris (Trichoptera, Hydroptilidae) in Japan. Zootaxa 4232(1): 104–112. 10.11646/zootaxa.4232.1.7 28264382

[B543] Ito T (2017b) The genus *Pseudoxyethira* Schmid (Trichoptera, Hydroptilidae) in Japan. Zootaxa 4319(1): 194–200. 10.11646/zootaxa.4319.1.12

[B544] Ito T (2017c) The genus *Stactobia* McLachlan (Trichoptera, Hydroptilidae) in Japan. Zootaxa 4350(2): 201–233. 10.11646/zootaxa.4350.2.1 29245550

[B545] Ito T (2020) The genus *Stactobiella* Martynov (Trichoptera, Hydroptilidae) in Japan. Zootaxa 4748(3): 561–571. 10.11646/zootaxa.4748.3.9 32230068

[B546] Ito T Hattori T (1986) Descriptions of a new species of *Palaeagapetus* (Trichoptera: Hydroptilidae) from northern Japan, with notes on bionomics. Kontyû, Tokyo 54: 143–151.

[B547] Ito T Kawamura H (1980) Morphology and biology of the immature stages of *Hydroptilaitoi* Kobayashi (Trichoptera, Hydroptilidae). Aquatic Insects 2(2): 113–122. 10.1080/01650428009361015

[B548] Ito T Kawamura H (1984) Morphology and ecology of immature stages of *Oxyethiraactua* Kobayashi (Trichoptera, Hydroptilidae). Japanese Journal of Limnology 45(4): 313–317. 10.3739/rikusui.45.313

[B549] Ito T Nagasaka Y (2014) Caddisfly (Trichoptera) fauna of Koshunai, Bibai-shi, Hokkaido, northern Japan. Biology of Inland Waters 29: 5–16.

[B550] Ito T Ohkawa A (2012) The genus *Ugandatrichia* Mosely (Trichoptera, Hydroptilidae) in Japan. Zootaxa 3394(1): 48–58. 10.11646/zootaxa.3394.1.5

[B551] Ito T Oláh J (2017) The genus *Oxyethira* Eaton (Trichoptera, Hydroptilidae) in Japan. Opuscula Zoologica Budapest 48(1): 3–25. 10.18348/opzool.2017.1.3

[B552] Ito T Park SJ (2016) A new species of the Genus *Orthotrichia* (Trichoptera, Hydroptilidae) from Korea. Animal Systematics, Evolution and Diversity 32(3): 230–233. 10.5635/ASED.2016.32.3.017

[B553] Ito T Saito R (2016) First record of *Plethus* Hagen (Trichoptera, Hydroptilidae) from Japan, with description of a species. Zootaxa 4154(4): 466–476. 10.11646/zootaxa.4154.4.6 27615852

[B554] Ito T Shimura N (2019) Notes on six microcaddisfly species (Trichoptera: Hydroptilidae) recorded for Japan, one a newly described species. Zootaxa 4629(1): 026–038. 10.11646/zootaxa.4629.1.2 31712531

[B555] Ito T Vshivkova T (1999) *Palaeagapetusfinisorientis*: description of all stages and biological observations (Trichoptera, Hydroptilidae, Ptilocolepinae). Malicky H, Chantaramongkol P (Eds) Proceedings of the 9^th^ International Symposium on Trichoptera. Faculty of Science, Chiang Mai University, Chiang Mai, Thailand, 141–148.

[B556] Ito T Tanida K Nozaki T (1993) Checklist of Trichoptera in Japan. I. Hydroptilidae and Lepidostomatidae. Japanese Journal of Limnology 54(2): 141–150. 10.3739/rikusui.54.141

[B557] Ito T Utsunomiya Y Kuhara N (1997) Morphological and geographical notes on the genus *Palaeagapetus* in the Asian Far East, with descriptions of two new species (Trichoptera: Hydroptilidae). Japanese Journal of Entomology 65: 97–107.

[B558] Ito T Yamamoto E Doi M Ohkawa A (2002) The family Lepidostomatidae and the genus *Palaeagapetus* of the family Hydroptilidae in Shikoku, western Japan (Trichoptera). Hyogo Freshwater Biology 54: 21–40.

[B559] Ito T Ohkawa A Hattori T (2011) The genus *Hydroptila* Dalman (Trichoptera, Hydroptilidae) in Japan. Zootaxa 2801(1): 1–26. 10.11646/zootaxa.2801.1.1 33756561

[B560] Ito T Wisseman RW Morse JC Colbo MH Weaver JS III (2014) The genus *Palaeagapetus* Ulmer (Trichoptera, Hydroptilidae, Ptilocolepinae) in North America. Zootaxa 3794(2): 201–221. 10.11646/zootaxa.3794.2.1 24870319

[B561] Ito T Nishimoto H Nishimoto F (2018) First record of the tropical-subtropical genus *Ugandatrichia* Mosely (Trichoptera, Hydroptilidae) from a temperate zone, with description of a new species. Zootaxa 4370(5): 492–500. 10.11646/zootaxa.4370.5.2 29689820

[B562] Ivanov VD (1992) New species of Glossosomatidae and Hydroptilidae (Trichoptera) from Pamir, Hissar and Tienshan Mountains. Aquatic Insects 14(4): 223–241. 10.1080/01650429209361488

[B563] Ivanov VD (2011) Caddisflies of Russia: Fauna and biodiversity. Zoosymposia 5(1): 171–209. 10.11646/zoosymposia.5.1.15

[B564] Ivanov VD Melnitsky SI (2007) New data of the Trichoptera of Siberia. Braueria 34: 31–35.

[B565] Ivanov VD Melnitsky SI (2017) New caddisflies species (Insecta: Trichoptera) from the Cretaceous Taymyr amber. Cretaceous Research 77: 124–132. 10.1016/j.cretres.2017.04.009

[B566] Iwata M (1928) Five new species of Trichopterous larvae from Formosa. Annotationes Zoologicae Japonenses 11(4): 341–351.

[B567] Iwata M (1930) Trichopterous larvae from Japan. V. Dobutsugaku Zasshi 42: 59–66. 10.5026/jgeography.42.59

[B568] Jacquemart S (1956) Trois *Orthotrichia* nouveaux du Lac Kivu (Trichoptera, Hydroptilidae). Bulletin de l’Institut Royal des Sciences Naturelles de Belgique 32(9): 1–6.

[B569] Jacquemart S (1957) Trichoptera des lacs Kivu et Édouard. Resultats Scientifique Explorations Hydrobiologique des Lacs Kivu, Édouard, et Albert, 1952–1954 3(2): 67–129.

[B570] Jacquemart S (1958) À propos d’*Agrayleapallidula* MacLachlan (Trichoptera, Hydroptilidae). Bulletin de l’Institut Royal des Sciences Naturelles de Belgique 34(14): 1–4.

[B571] Jacquemart S (1960) A propos de deux Trichopteres nouveaux pour la faune belge et d’une espèce rare. Bulletin de l’Institut Royal des Sciences Naturelles de Belgique 36(13): 1–5.

[B572] Jacquemart S (1962a) La larve d’*Orthotrichiaangustella* MacLachlan (Trichoptère, Hydroptilidae). Bulletin de l’Institut Royal des Sciences Naturalles de Belgique 38(12): 1–10.

[B573] Jacquemart S (1962b) Trois Trichoptères nouveaux d’Afrique centrale. Bulletin de l’Institut Royal des Sciences Naturalles de Belgique. Entomologie 38(34): 1–11.

[B574] Jacquemart S (1963a) Chapter IV. Trichoptera. In: Hanström B Brinck P Rudebeck G (Eds) South African Animal Life: Results of the Lund University Expedition in 1950–1951.Swedish Natural Science Research Council, Stockholm, 337–415.

[B575] Jacquemart S (1963b) Deux Trichoptères nouveaux d’Argentine. In: Delamare C Rapaport E (Eds) Biologie de l’Amerique Australe, vol.2. Centre National de la Recherche Scientifique, Paris, 339–342.

[B576] Jacquemart S (1963c) Trichoptères nouveaux des Iles Maurice et de La Réunion. Bulletin de l’Institut Royal des Sciences Naturelles de Belgique 39(39): 1–7.

[B577] Jacquemart S (1963d) Un Trichoptère nouveau de Chypre: *Stactobiamonnioti* sp. n. (Hydroptilidae). Bulletin de l’Institut Royal des Sciences Naturalles de Belgique 39(13): 1–9.

[B578] Jacquemart S (1965) Resultats de l’expédition Belge an Moyen-Orient (Première note). Sept Trichoptères nouveaux de Turquie et d’Iran. Bulletin de l’Institut Royal des Sciences Naturelles de Belgique 41: 1–19.

[B579] Jacquemart S (1973) Description de deux trichoptères hydroptilides nouveaux et de l’imago de *Stactobiamonnioti* Jacquemart (Ile de Rhodes). Bulletin de l’Institut Royal des Sciences Naturelles de Belgique. Entomologie 49(4): 1–16.

[B580] Jacquemart S (1980a) Un trichoptère hydroptilide nouveau du nord du Chili: *Metrichiathirysae* sp. n. Brenesia 17: 303–318.

[B581] Jacquemart S (1980b) Un trichoptère nouveau de l’Air: *Hydroptilaairensis* sp. n. (Hydroptilidae). Bulletin de l’Institut Royal des Sciences Naturelles de Belgique. Entomologie 52(13): 1–5.

[B582] Jacquemart S Coineau Y (1962) Missions S. Jacquemart dans les Pyrénées Orientales (2^me^ note). Les Hydropilides des Alberes. Bulletin de l’Institut Royal des Sciences Naturalles de Belgique 38(24): 1–81.

[B583] Jacquemin G Coppa G (2015) *Oxyethiradistinctella* McLachlan, 1880 en Lorraine: Une espèce nouvelle pour la faune de France. Ephemera 15(2): 107–113. [Trichoptera, Hydroptilidae]

[B584] Jacquemin G Coppa G Le Guellec G (2019) *Oxyethirasimplex* Ris, 1897: Nouvelles observations en France. Ephemera 20(2): 99–105. [Trichoptera, Hydroptilidae]

[B585] James ABW Suren AM (2009) The response of invertebrates to a gradient of flow reduction - an instream channel study in a New Zealand lowland river. Freshwater Biology 54(11): 2225–2242. 10.1111/j.1365-2427.2009.02254.x

[B586] Johanson KA (1992) A catalog of the caddis flies of East Africa (Insecta, Trichoptera). Steenstrupia (Copenhagen) 18(7): 113–141.

[B587] Johanson KA Mary N (2009) Description of three new caddisfly species from Mayotte Island, Comoros Archipelago (Insecta: Trichoptera). Zootaxa 2089(1): 1–9. 10.11646/zootaxa.2089.1.1

[B588] Johanson KA Wells A (2019) New Caledonia’s Trichoptera - present status of knowledge. Zoosymposia 14: 087–102. 10.11646/zoosymposia.14.1.12

[B589] Johanson KA Wells A Malm T Espeland M (2011) The Trichoptera of Vanuatu. Deutsche Entomologische Zeitschrift 58(2): 279–320. 10.1002/mmnd.201100031

[B590] Joy MK Death RG (2000) Stream invertebrate communities of Campbell Island. Hydrobiologia 439(1/3): 115–124. 10.1023/A:1004103815444

[B591] Kachalova OL Muhametšina S (1979) Eine neue Art der Köcherfliegen der Gattung *Hydroptila* Dalman (Trichoptera, Hydroptilidae) aus dem Wolgadelta. Latvijas Entomologs 21: 82–85.

[B592] Kahnert M (1995) Beiträg zur Köcherfliegenfauna in Quell-Biotopen am Mindelsee. Lauterbornia 22: 121–129.

[B593] Karaouzas I Malicky H (2015) New faunistic records of Trichoptera in Greece. Braueria 42: 13–20.

[B594] Karaouzas I Malicky H (2016) New Trichoptera records in islands of the Aegean. Braueria 43: 18.

[B595] Keiper JB (1999) Morphology of final instar *Ochrotrichiaxena* (Trichoptera: Hydroptilidae). Entomological News 110: 231–235.

[B596] Keiper JB Bartolotta RJ (2003) Taxonomic and ecological notes on *Leucotrichiapictipes* (Trichoptera: Hydroptilidae), a microcaddisfly newly recorded from Ohio, U.S.A. Entomological News 114: 255–259.

[B597] Keiper JB Foote BA (1998) Biological notes on *Ochrotrichiaxena* (Ross) (Trichoptera: Hydroptilidae), a species newly recorded from Ohio. Proceedings of the Entomological Society of Washington 100: 594–595.

[B598] Keiper JB Foote BA (1999) Biology and immature stages of two species of Hydroptilidae Dalman (Trichoptera: Hydroptilidae) which consume *Cladophora* (Chlorophyta). Proceedings of the Entomological Society of Washington 101(3): 514–521.

[B599] Keiper JB Foote BA (2000) Biology and larval feeding habits of coexisting Hydroptilidae (Trichoptera) from a small woodland stream in northeastern Ohio. Annals of the Entomological Society of America 93(2): 225–234. 10.1603/0013-8746(2000)093[0225:BALFHO]2.0.CO;2

[B600] Keiper JB Harris SC (2002) Biology and immature stages of *Ochrotrichiafootei* (Trichoptera: Hydroptilidae), a new microcaddisfly from a torrential mountain stream. Proceedings of the Entomological Society of Washington 104(2): 291–299.

[B601] Keiper JB Walton WE (1999) Biology and morphology of *Oxyethiraarizona* Ross (Trichoptera: Hydroptilidae). The Pan-Pacific Entomologist 75: 212–200.

[B602] Keiper JB Walton WE (2000) Biology and immature stages of *Ochrotrichiaquadrispina* Denning and Blickle (Trichoptera: Hydroptilidae), a spring-inhabiting scraper. Proceedings of the Entomological Society of Washington 102(1): 183–187.

[B603] Keiper JB Casamatta DA Foote BA (1998a) Incorporation of B*atrachospermum gelatinosum* (Rhodophyta) into cases of *Ochrotrichiawojcickyi* (Trichoptera: Hydroptilidae). Entomological News 109(4): 256.

[B604] Keiper JB Casamatta DA Foote BA (1998b) Use of algal monocultures by larvae of *Hydroptilawaubesiana* and *Oxyethirapallida* (Trichoptera: Hydroptilidae). Hydrobiologia 380(1/3): 87–91. 10.1023/A:1003468432655

[B605] Kelley RW (1981) New species of *Oxyethira* (Trichoptera: Hydroptilidae) from the southeastern United States. Journal of the Georgia Entomological Society 16(3): 368–375.

[B606] Kelley RW (1983) New Neotropical species of *Oxyethira* (Trichoptera: Hydroptilidae). Proceedings of the Entomological Society of Washington 85: 41–54.

[B607] Kelley RW (1984a) Phylogeny, morphology and classification of the micro-caddisfly genus *Oxyethira* Eaton (Trichoptera: Hydroptilidae). Transactions of the American Entomological Society 110: 435–463.

[B608] Kelley RW (1984b) The *falcata*-species complex of the genus *Oxyethira* (Trichoptera: Hydroptilidae). In: Morse JC (Ed.) Proceedings of the 4th International Symposium on Trichoptera.Dr. W. Junk, The Hague, 185–190.

[B609] Kelley RW (1985) Revision of the micro-caddisfly genus *Oxyethira* (Trichoptera: Hydroptilidae). Part II: subgenus Oxyethira. Transactions of the American Entomological Society 111: 223–253.

[B610] Kelley RW (1986) Revision of the micro-caddisfly genus *Oxyethira* (Trichoptera: Hydroptilidae) Part III: subgenus Holarctotrichia. Proceedings of the Entomological Society of Washington 88(4): 777–785.

[B611] Kelley RW (1989) New species of micro-caddisflies (Trichoptera: Hydroptilidae) from New Caledonia, Vanuatu and Fiji. Proceedings of the Entomological Society of Washington 91: 190–202.

[B612] Kelley RW (1992) Phylogenetic relationships of micro-caddisfly genera (Hydroptilidae). Bulletin of the North American Benthological Society 9(145). [Abstract]

[B613] Kelley RW Harris SC (1983) New Hydroptilidae (Trichoptera) from Alabama and South Carolina. Entomological News 94: 181–186.

[B614] Kelley RW Morse JC (1982) A key to the females of the genus *Oxyethira* (Trichoptera: Hydroptilidae) from the southern United States. Proceedings of the Entomological Society of Washington 84(2): 256–269.

[B615] Kelsey LP (1969) A revision of the Scenopinidae (Diptera) of the World. Bulletin - United States National Museum 277: 1–336. 10.5962/bhl.title.16405

[B616] Kendrick MR Huryn AD (2014) The Plecoptera And Trichoptera of the Arctic North Slope of Alaska. Western North American Naturalist 74(3): 275–285. 10.3398/064.074.0303

[B617] Keth AC (2003) Five new species of *Neotrichia* (Trichoptera: Hydroptilidae: Neotrichiini) from southern Mexico and northern Belize. Entomological News 114: 164–178.

[B618] Keth AC Harris SC Armitage BJ (2015) The genus *Neotrichia* Morton (Trichoptera: Hydroptilidae) in North America, Mexico, and the Caribbean Islands. The Caddis Press, Columbus, Ohio, [v +] 147 pp.

[B619] Kimmins DE (1943) A list of the Trichoptera (caddis flies) of the Lake District with distributional and seasonal data. Journal of the Society for British Entomology 2: 136–157.

[B620] Kimmins DE (1949) The identity of *Stactobiafuscicornis* (Schneider) (Trichoptera, Hydroptilidae). Entomologist, London 82: 229–233.

[B621] Kimmins DE (1950) The type-species of certain genera in the Trichoptera. Entomologist 83: 58–60.

[B622] Kimmins DE (1951) Indian caddisflies IV. New genera and species of the family Hydroptilidae. Annals & Magazine of Natural History 12(39): 193–213. 10.1080/00222935108654144

[B623] Kimmins DE (1957a) Lectotypes of Trichoptera from the McLachlan collection now in the British Museum (Natural History). Bulletin of the British Museum (Natural History). Entomology 6(4): 91–126. 10.5962/bhl.part.17102

[B624] Kimmins DE (1957b) Neuroptera and Trichoptera collected by Mr. J.D. Bradley on Guadalcanal Island, 1953–54. Bulletin of the British Museum (Natural History). Entomology 5(7): 287–308. 10.5962/bhl.part.1511

[B625] Kimmins DE (1957c) New and little-known species of African Trichoptera. Bulletin of the British Museum (Natural History). Entomology 6(1): 1–37. 10.5962/bhl.part.17102

[B626] Kimmins DE (1958a) On some Trichoptera from S. Rhodesia and Portuguese East Africa. Bulletin of the British Museum (Natural History). Entomology 7: 359–368.

[B627] Kimmins DE (1958b) The British species of the genus *Oxyethira* (Trichoptera: Hydroptilidae). Entomologist’s Gazette 9: 7–17.

[B628] Kimmins DE (1959) .Trichoptera. Ruwenzori Expedition 1952 (British Museum, Natural History) 11(9): 47–61.

[B629] Kimmins DE (1961) A species of *Hydroptila* (Trichoptera) new to Britain. Entomologist’s Gazette 12: 32–35.

[B630] Kimmins DE (1962) Miss L.E. Cheesman’s expeditions to New Guinea. Trichoptera. Bulletin of the British Museum (Natural History). Entomology 11(4): 97–187.

[B631] Kimmins DE (1964) On the Trichoptera of Nepal. Bulletin of the British Museum (Natural History). Entomology 15(2): 33–55. 10.5962/bhl.part.20534

[B632] Kimmins DE (1966) A revised check-list of the British Trichoptera. Entomologist’s Gazette 17: 111–120.

[B633] Kimura G Inoue E Hirabayashi K (2008) Seasonal abundance of adult caddisfly (Trichoptera) in the middle reaches of the Shinano River in central Japan. In: Robinson WH Bajomi D (Eds) Proceedings of the Sixth International Conference on Urban Pests.OOK-Press Kft., Hungary, 259–266.

[B634] Kimura G Murai Y Tanikawa T (2016) Distribution and abundance of adult caddisflies (Trichoptera) in the vicinity of food and pharmaceutical factories. Zoosymposia 10(1): 243–247. 10.11646/zoosymposia.10.1.22

[B635] King JJFX (1886) A contribution towards a neuropterous fauna of Ireland. Transactions of the Natural History Society of Glasgow 2: 259–292.

[B636] Kingsolver JM Ross HH (1961) New species of Nearctic *Orthotrichia* (Hydroptilidae, Trichoptera). Illinois State Academy of Science Transactions 54: 28–33.

[B637] Kiss O (2012) Trichoptera collected by light trapping from the Hungarian section of the River Tisza. Braueria 39: 25–31.

[B638] Kiss O Szentkirályi F Schmera D (2006) Tegzesek (Trichoptera) szezonális rajzás-aktivitásának jellemzése eltérő élőhelyeken történő fénycsapdás monitorozás alapján. Acta Biologica Debrecina Supplementum Oecologica Hungarica 14: 139–149.

[B639] Kjærandsen J (1997) *Wlitrichiaintropertica* new genus, new species, and *Cyclopsiellaanderseni* new genus, new species, two new monobasic genera of microcaddisflies from Ghana (Trichoptera: Hydroptilidae: Hydroptilini). In: Holzenthal RW Flint Jr OS (Eds) Proceedings of the 8th International Symposium on Trichoptera.Ohio Biological Survey, Columbus, 227–237.

[B640] Kjærandsen J (2004) A revision of the Afrotropical genus *Dhatrichia* (Trichoptera, Hydroptilidae). Zoologica Scripta 33(2): 131–185. 10.1111/j.1463-6409.2004.00143.x

[B641] Kjærandsen J Andersen T (1997) Preliminary check-list of the caddisflies (Trichoptera) of Ghana, West-Africa. In: Holzenthal RW Flint Jr OS (Eds) Proceedings of the 8th International Symposium on Trichoptera.Ohio Biological Survey, Columbus, 239–247.

[B642] Kjærandsen J Andersen T (2002) A review of *Jabitrichia* Wells, 1990 (Trichoptera: Hydroptilidae), with the description of a new Afrotropical species. Nova Supplementa Entomologica (Proceedings of the 10^th^ International Symposium on Trichoptera) 15: 133–144.

[B643] Kjærandsen J Ito T (2009) First records of *Microptila* Ris (Trichoptera: Hydroptilidae) from Japan, with description of a new species. Entomological Science 12(2): 177–181. 10.1111/j.1479-8298.2009.00320.x

[B644] Klapálek F (1890) Die metamorphosestadien der *Oxyethiracostalis*, Curt. (*Lagenopsyche* Fr. Müller). Sitzungberichte der Königlich Böhmischen Gesellschaft der Wissenschaften in Prag 1890: 204–208.

[B645] Klapálek F (1891) I. Dodatky ku Seznamu Českých Trichopter za Rok 1890. Sitzungberichte der Königlich Böhmischen Gesellschaft der Wissenschaften in Prag 1891: 176–196. [Contribution to the knowledge of Bohemian Trichoptera for the year 1890]

[B646] Klapálek F (1893) Untersuchungen über die Fauna der Ggewässer Böhmens. I. Metamorphose der Trichopteren (2^nd^ series, continued from 1888). Archiv für die Naturwissenschaftliche Landesdurchforschung von Böhmen 8(6): 1–142.

[B647] Klapálek F (1894) Beiträge zur Kenntnis der böhmischen Hydroptiliden. Sitzungberichte der Königlich Böhmischen Gesellschaft der Wissenschaften in Prag 43: 1–10.

[B648] Klapálek F (1895) *Oxyethiratristella* n. sp. The Entomologist’s Monthly Magazine (series 2) 6: 168.

[B649] Klapálek F (1897) Příspěvek ku znalosti vývoje Českých Hydroptilid. Věstník Královské České Společnosti Náuk 10: 16 pp.

[B650] Klapálek F (1900a) Beiträge zur Kenntnis der Neuropteren von Krain und Kärnthen. Bulletin International de l’Academie des Sciences de Böhmen 6: 72–78.

[B651] Klapálek F (1900b) Příspěvek ku znalosti Neuropteroid z Krajiny und Korutan. Rozpravy České Akademie věd a umění, Praze 9(14): 12 pp.

[B652] Klapálek F (1902) O morfologii kroužků a přívěsků pohlavních u Trichopter. I. Rhyacophilidae, Philipotamidae, et Hydroptilidae. Rozpravy České Akademie věd a umění, Praze 9: 39 pp.

[B653] Kloet GS Hincks WD (1944) Nomenclatorial notes on two generic names in the Trichoptera. Entomologist, London 77: 97.

[B654] Kobayashi M (1974) On two new species of Hydroptilidae from Japan (Insecta: Trichoptera). Kanagawa Kenritsu Hakubutsukan Kenkyu Hokoku, Shizen Kagaku 7: 67–70.

[B655] Kobayashi M (1977) The list and new species of the caddisflies from Hokkaido, Japan (Trichoptera, Insecta). Kanagawa Kenritsu Hakubutsukan Kenkyu Hokoku, Shizen Kagaku 10: 1–14.

[B656] Kobayashi S Nozaki T Takemon Y (2017) Caddisfly community in the Seta-Uji River, the outlet of Lake Biwa. Japanese Journal of Ecology 67: 13–29.

[B657] Koçak AO Kemal M (2012) A generic nomenclatural correction among micro-caddisflies in the Oriental region. (Hydroptilidae, Trichoptera). Centre for Entomological Studies Miscellaneous Papers 158: 4–5.

[B658] Kolbe HJ (1887) Ueber eine neue von Herrn H. Tetens bei Berlin aufgefundene Art der Phyrganiden. Entomologishe Nachrichten 13: 356–359.

[B659] Kolenati FA (1848) Genera et species Trichopterorum, Pars prior. Acta Regiae Bohemoslovenicae Societatis Scientiarum, Prague 6: 1–108. 10.5962/bhl.title.130921

[B660] Kolenati FA (1859) Genera et species Trichopterorum, Pars Altera. Nouveaux Mémoires de la Société Impériale des Naturalistes de Moscou 11: 141–296.

[B661] Komzák P Chvojka P (2005) New faunistic records of Trichoptera (Insecta) from the Czech Republic, II. Časopis Národního Muzea Řada Přírodovědná 174(1–4): 65–66.

[B662] Komzák P Chvojka P (2012) Caddis flies (Trichoptera) of the Bílé Karpaty Protected Landscape Area and Biosphere Reserve (Czech Republic). Acta Musei Moraviae. Scientiae Biologicae 96: 697–761.

[B663] Komzák P Kroča J (2011) New faunistic records of Trichoptera (Insecta) from the Czech Republic, IV. Acta Musei Moraviae. Scientiae Biologicae 96(1): 189–192.

[B664] Komzák P Kroča J (2018) New faunistic records of Hydroptilidae (Insecta, Trichoptera) from the Czech Republic. Acta Musei Silesiae. Scientiae Naturales 67(2): 165–173. 10.2478/cszma-2018-0011

[B665] Kristensen NP (1997) Early evolution of the Lepidoptera + Trichoptera lineage: phylogeny and the ecological scenario. In: Grandcolas P (Ed.) The Origin of Biodiversity in Insects: Phylogenetic Tests of Evolutionary Scenarios.Mémoires du Muséum national d’histoire naturelle, Éditions du Muséum, Paris, 253–271.

[B666] Kroča J Komzák P (2020) Trichoptera (Insecta) of the Javorníky Mts. (Czech Republic). Acta Musei Silesiae. Scientiae Naturales 69(2): 141–159. 10.2478/cszma-2020-0010

[B667] Krušnik C (1991) A contribution to the knowledge of the caddis-fly fauna (Insecta, Trichoptera) from the southwestern edge of the Karst. Bioloski Vestnik 39(3): 11–20.

[B668] Kučinić M Vučković I Kutnjak H Jelaska LS Marguš D (2011) Diversity, distribution, ecology and biogeography of caddisflies (Insecta: Trichoptera) in the Krka River (National Park ‘Krka’, Croatia). Zoosymposia 5(1): 255–268. 10.11646/zoosymposia.5.1.19

[B669] Kučinić M Ćukušić A Žalac S Delić A Cerjanec D Podnar M Ćuk R Vučković I Previšić A Vuković M Koštroman SS Bukvić V Šalinović A Plantak M (2020) Springs: DNA barcoding of caddisflies (Insecta: Trichoptera) in Croatia with notes on taxonomy and conservation biology. Natura Croatica 29(1): 73–98. 10.20302/NC.2020.29.8

[B670] Küçükbasmaci I Canbulat S (2020) A list of the caddisflies (Insecta: Trichoptera) from Kyrgyzstan, with a new record (*Triaenodesreuteri* McLachlan 1880). Zootaxa 4896(1): 113–122. 10.11646/zootaxa.4896.1.6 33756876

[B671] Küçükbasmaci I Kiyak S (2017) A study on the caddisfly fauna (Insecta: Trichoptera) of Kastamonu and a new species record for Turkey. Munis Entomology & Zoology 12(2): 486–499.

[B672] Kumanski KP (1972) Eine neue *Hydroptila*-Art aus Bulgarien (Trichoptera: Hydroptilidae). Comptes rendus de l’Académie bulgare des Sciences: sciences mathématiques et naturelles 25(9): 1261–1263.

[B673] Kumanski KP (1974) Description de *Hydroptilaangulifera*, une nouvelle espèce du Rhodope bulgare (Trichoptera, Hydroptilidae). Reichenbachia 15(10): 71–75.

[B674] Kumanski KP (1979) The family Hydroptilidae (Trichoptera) in Bulgaria. Acta Zoologica Bulgarica 13: 3–20.

[B675] Kumanski KP (1980) A contribution to the knowledge of Trichoptera (Insecta) of the Caucasus. Acta Zoologica Bulgarica 14: 32–48.

[B676] Kumanski KP (1983) Notes on the group of *Sparsa* of genus *Hydroptila* Dalm., with description of a new species (Trichoptera, Hydroptilidae). Reichenbachia 21(2): 15–18.

[B677] Kumanski KP (1985) Trichoptera, Annulipalpia. Fauna na Bulgariya 15: 1–244.

[B678] Kumanski KP (1987) On caddisflies (Trichoptera) of Cuba. Acta Zoologica Bulgarica 34: 3–35.

[B679] Kumanski KP (1990) Studies on the fauna of Trichoptera (Insecta) of Korea. 1. Superfamily Rhyacophiloidea. Historia Naturalis Bulgarica 2: 36–60.

[B680] Kumanski KP (1993) Addition to Volume 15 (Trichoptera, Annulipalpia) and Volume 19 (Trichoptera, Integripalpia) of the series “Fauna of Bulgaria”. Historia Naturalis Bulgarica 4: 39–46.

[B681] Kumanski KP Malicky H (1984) On the fauna and the zoogeographical significance of Trichoptera from the Strandzka Mts. (Bulgaria). In: Morse JC (Ed.) Proceedings of the 4th International Symposium on Trichoptera.Dr. W. Junk, The Hague, 197–201.

[B682] Küttner R Plesky B Voigt H (2016) Interessante und neue Nachweise von Wasserinsekten in Sachsen (Ephemeroptera, Plecoptera, Trichoptera, Megaloptera). Entomologische Nachrichten und Berichte 60(3/4): 177–184.

[B683] Labat F Auzeric E Courte M Fernandez N Gaillard D Grac C Lambert J Meyer A Moreau A Poujardieu B Tarozzi N (2019) Nouvelles localités de *Tricholeiochitonfagesii* (Guinard, 1879) en France. Ephemera 20(2): 107–112. [Trichoptera, Hydroptilidae]

[B684] Lake RW (1984) Distribution of caddisflies (Trichoptera) in Delaware. Entomological News 95: 215–224.

[B685] Larned ST Kilroy C (2014) Effects of *Didymospheniageminata* removal on river macroinvertebrate communities. Journal of Freshwater Ecology 29(3): 345–362. 10.1080/02705060.2014.898595

[B686] Laudee P (2004) Life history and larval morphology of the giant microcaddisfly, *Ugandatrichiakerdmuang* Malicky & Chantaramongkol 1991 (Hydroptilidae: Trichoptera). Braueria 31: 21–24.

[B687] Laudee P (2008) Larval morphology and diagnosis of the giant microcaddisfly species, *Ugandatrichia* spp. (Hydroptilidae: Trichoptera) in Thailand. Zootaxa 1825(1): 29–39. 10.11646/zootaxa.1825.1.3

[B688] Laudee P Mesuk K (2019) Biodiversity of Trichoptera from waterfalls on islands in the Thai Gulf and the Andaman Sea, Thailand. Zoosymposia 14(1): 108–112. 10.11646/zoosymposia.14.1.14

[B689] Laudee P Prommi TO (2011) Biodiversity and distribution of Trichoptera species along the Tapee River, Surat Thani Province, southern Thailand. Zoosymposia 5(1): 279–287. 10.11646/zoosymposia.5.1.21

[B690] Lauterborn R (1934) Der Rhein. Naturgeschichte eines deutschen Stromes. Berichte der Naturforschenden Gesellschaft zu Freiburg i. Br. 33: 1–325.

[B691] Le Guellec G (2011) Rediscovery of *Stactobiabeatensis* Mosely, 1934 in France (Trichoptera, Hydroptilidae). Ephemera 12(1): 27–29.

[B692] Le Guellec G Niel A Cagan O Coppa G (2013) Additions à la faune des Trichoptères de France: *Stactobiaalpina* Bertuetti, Lodovici & Valle, 2004 et *Tinodesluscinia* Ris, 1903. Ephemera 14(1): 35–38. [Trichoptera, Hydroptilidae & Psychomyiidae]

[B693] Le Guellec G Guidi T Coppa G (2020) *Rhyacophilaarcangelina* Navás, 1932 et *Hydroptilaruffoi* Moretti, 1981 deux espèces nouvelles pour la faune de France. Ephemera 21(2): 139–140. [Trichoptera, Rhyacophilidae & Hydroptilidae]

[B694] Leader JP (1968) Hairs of the Hydroptilidae (Trichoptera). Tane. Journal of the Auckland University Field Club 16: 121–129.

[B695] Leader JP (1972) The New Zealand Hydroptilidae (Trichoptera). Journal of Entomology 41: 191–200. 10.1111/j.1365-3113.1972.tb00047.x [Series B]

[B696] Lepneva SG (1932) Zum Studium der Trichopterenlarven in den Wasserbecken der Systeme des Dnipro und des Sud-Bugs. Zh. bio-zool. Tsyklu Kyyiv 3: 71–115.

[B697] Lepneva SG (1953) Caddisflies: Trichoptera. In: Fauna of the USSR. Vol. 4. The Forest Zone. Doklady Akademii Nauk SSSR, Moscow, 404–324. [in Russian]

[B698] Lepneva SG (1964) [Larvae and pupae of the suborder Annulipalpia. Trichoptera. II (1)]. Zoologicheskogo Instituta Akademii Nauk SSSR (N.S.) 88: 1–562.

[B699] Lepneva SG (1970) Fauna of the USSR, Trichoptera II(1). Larvae and pupae of the Annulipalpia. Zoological Institute of the Academy of Science of the USSR, New Series 88: 1–638.

[B700] Lewis DJ Fairchild WL (1984) A phoretic association between a caddisfly and a copepod fish parasite. Canadian Journal of Zoology 62(1): 134–135. 10.1139/z84-021

[B701] Light RW Adler PH (1983) Predicting the colonization cycle of aquatic invertebrates. Freshwater Invertebrate Biology 2(2): 74–87. 10.2307/1467112

[B702] Lillehammer A (1978) The Trichoptera of Øvre Heimdalsvatn. Holarctic Ecology 1(2–3): 255–260. 10.1111/j.1600-0587.1978.tb00958.x

[B703] Lloyd JT (1915) Notes on *Ithytrichiaconfusa* Morton. Canadian Entomologist 47(4): 117–121. 10.4039/Ent47117-4

[B704] Lock K (2014) *Oxyethirafalcata* Morton, 1893 new to Belgium (Trichoptera: Hydroptilidae). Bulletin de la Société Royale Belge d’Entomologie 150(3): 199–200.

[B705] Lock K Goethals PLM (2012) Updated checklist of the Belgian caddisflies (Trichoptera). Bulletin de la Société Royale Belge d’Entomologie/Bulletin van de Koninklijke Belgische Vereniging voor Entomologie 148(1): 27–33.

[B706] Lock K van Butsel J (2017) *Hydroptilaangulata* Mosely, 1922, *Hydroptilasimulans* Mosely, 1920 and *Tinodesmaculicornis* (Picter, 1834) confirmed for Belgium (Trichoptera: Hydroptilidae, Psychomyiidae). Bulletin de la Société royale belge d’Entomologie/Bulletin van de Koninklijke Belgische Vereniging voor Entomologie 153: 32–35.

[B707] Lock K van Butsel J (2018) *Hydroptilalotensis* Mosely 1930 and *Tinodesmaculicornis* (Picter 1834): two caddisflies new to the Grand Duchy of Luxembourg (Trichoptera: Hydroptilidae & Psychomyiidae). Entomologie Faunistique -. Entomologie Faunistique 71: 1–6.

[B708] Lock K Zwaenepoel A (2014) *Orthotrichiatragetti* Mosely, 1930 new to the Belgian fauna (Trichoptera: Hydroptilidae). Bulletin de la Société Royale Belge d’Entomologie/Bulletin van de Koninklijke Belgische Vereniging voor Entomologie 150(3): 232–234.

[B709] Lock K Tempelman D Sanabria M (2013) Three new caddisflies for the Belgian fauna: *Holocentropusinsignis* Martynov, 1924; *Hydroptilatineoides* Dalman, 1819 and *Oxyethirasimplex* Ris, 1897 (Trichoptera). Bulletin de la Société Royale Belge d’Entomologie/Bulletin van de Koninklijke Belgische Vereniging voor Entomologie 149: 22–26.

[B710] Lodovici O Valle M (2013) The genus *Stactobia* McLachlan, 1880 (Trichoptera, Hydroptilidae) in Italy. La Rivista del Museo Civico di Scienze Naturali “Enrico Caffi” di Bergamo 26: 161–181.

[B711] Lonsdale O (2020) Name-bearing type specimens of Trichoptera (Insecta) in the Canadian National Collection of Insects, Arachnids & Nematodes (CNC), with a biography of Fernand Schmid. Opuscula Zoolologica (Budapest) 51(S1): 03–141. 10.18348/opzool.2020.S1.3

[B712] López del Castillo P López CN Triana JLF Lazo DG Quintana AT Ozoria JP (2004) Insectos acuáticos del Parque Nacional “La Bayamesa”, Cuba. Boletin de la SEA 35: 225–231.

[B713] Lubini-Ferlin V Vicentini H (2005) Der aktuelle Kenntnisstand der Köcherfliegenfauna (Insecta: Trichoptera) der Schweiz. Lauterbornia 54: 63–78.

[B714] Luhman JC Holzenthal RW Kjaerandsen JK (1999) New host record of a ceraphronid (Hymenoptera) in Trichoptera pupae. Journal of Hymenoptera Research 8: 126.

[B715] Lukáš J (2004) Invasive and newly-recorded caddisflies (Trichoptera) from Slovakia. Biologia 59(5): 685–686.

[B716] Lukáš J Chvojka P (2011) New faunistic records of Trichoptera from Slovakia. Klapalekiana 47(1–2): 115–117.

[B717] Lundblad [Lundblatt] O (1918) *Ithytrichialamellaris* Eaton. Entomologisk Tidskrift 39: 342–343.

[B718] Mabrouki Y Taybi AF Alami ME Wiggers R Berrahou A (2020) New data on fauna of caddisflies (Insecta: Trichoptera) from northeastern Morocco with notes on chorology. Aquatic Insects 41(4): 356–390. 10.1080/01650424.2020.1797817

[B719] Macdonald WW (1950) The larvae of *Mystacidesazurea* L., *Cyrnusflavidus* McLachlan and *Oxyethirasimplex* Ris (Trichoptera). Proceedings of the Royal Entomological Society of London 25(1–3): 19–28. 10.1111/j.1365-3032.1950.tb00080.x

[B720] Maes J-M (1999) Orden Trichoptera. In: Maes J-M (Ed.) Insectos de Nicaragua.Catálogo de los Insectos y Artropodos Terrestres de Nicaragua Vol. III, Managua, Nicaragua, 1184–1199.

[B721] Maes J-M Flint Jr OS (1988) Catalogo de los Trichoptera de Nicaragua. Revista Nicaraguense Entomologica 2: 1–11.

[B722] Maier K-J Kampwerth U Peissner T Speidel E (1995) Beiträg zur Kenntnis der Köcherfliegenfauna Baden-Wurttembergs (Insecta: Trichoptera). Lauterbornia 22: 143–156.

[B723] Malicky H (1972) Weitere neue Arten und Fundorte von westpaläarktischen Köcherfliegen (Trichoptera), vor allem aus dem östlichen Mediterrangebiet. Mitteilungen der Entomologischen Gesellschaft Basel 22(2/3): 25–68.

[B724] Malicky H (1974) Die Köcherfliegen (Trichoptera) Griechenlands. Übersicht und Neubeschreibungen. Annales Musei Goulandris 2: 105–135.

[B725] Malicky H (1975) Fünfzehn neue mediterrane Köcherfliegen. Mitteilungen der Entomologischen Gesellschaft Basel 25(3): 81–96.

[B726] Malicky H (1976) Beschreibung von 22 neuen westpaläarktischen Köcherfliegen (Trichoptera). Zeitschrift der Arbeitsgemeinschaft Österreichischer Entomologen 27: 89–104.

[B727] Malicky H (1977) Weitere neue und wenig bekannte mediterrane Köcherfliegen (Trichoptera). Nachrichtenblatt der Bayerischen Entomologen 26: 65–77.

[B728] Malicky H (1979) Notes on some caddisflies (Trichoptera) from Europe and Iran. Aquatic Insects 1(1): 3–16. 10.1080/01650427909360974

[B729] Malicky H (1980a) Beschreibungen von neuen mediterranen Köcherfliegen und Bemerkungen zu bekannten (Trichoptera). Zeitschrift der Arbeitsgemeinschaft Österreichischer Entomologen 32(1–2): 1–17.

[B730] Malicky H (1980b) Vier neue Köcherfliegen von der Insel Guadeloupe (Kleine Antillen, Mittelamerika) (Trichoptera). Zeitschrift für Entomologie 1: 219–225.

[B731] Malicky H (1981a) Neues über mediterrane, vorderasiatische und europäische Köcherfliegen (Trichoptera). Entomofauna. Zeitschrift fur Entomologie 2(16): 175–188.

[B732] Malicky H (1981b) Weiteres Neues über Köcherfliegen aus dem Mittelmeergebiet (Trichoptera). Entomofauna 2(27): 335–355.

[B733] Malicky H (1982) [1983a]) Köcherfliegen (Trichoptera) von den Kapverdischen Inseln. Zeitschrift der Arbeitsgemeinschaft Oesterreichischer Entomologen 34(3–4): 106–110.

[B734] Malicky H (1983b) Atlas of European Trichoptera. Series Entomologica 24. Dordrecht: Dr W Junk, 387 pp. 10.1007/978-94-017-5164-3_1

[B735] Malicky H (1983c) Trichoptères des petites Antilles (Trichoptera). Annalen des Naturhistorischen Museums in Wien 85: 263–271. 10.1007/978-94-017-5164-3_1

[B736] Malicky H (1984) Fünf neue griechische Köcherfliegen (Trichoptera). Mitteilungen der Entomologischen Gesellschaft Basel 34(3): 96–102.

[B737] Malicky H (1987) *Hydroptilajuba*, bona species. Trichoptera Newsletter 14: 30.

[B738] Malicky H (1988a) A comment on figuring three-dimensional structures. Trichoptera Newsletter 15: 21–24.

[B739] Malicky H (1988b) Eine neue *Stactobia* (Trichoptera: Hydroptilidae) aus der Ost-Türkei. Entomologische Zeitschrift 98: 63–64.

[B740] Malicky H (1992a) Köcherfliegen (Insecta: Trichoptera) von den Seychellen, Komoren und Maskarenen. Annalen des Naturhistorischen Museums in Wien. Serie B, Fur Botanik und Zoologie 93: 143–160.

[B741] Malicky H (1992b) Vier neue griechische Köcherfliegen (Trichoptera). Entomologische Zeitschrift mit Insektenborse 102(3): 40–45.

[B742] Malicky H (1993) Three new caddisflies from Mahé Island, Seychelles. Braueria 20: 19–21.

[B743] Malicky H (1996a) Beschreibung und Vergreitung von *Hydroptilabrissaga* n. sp. einer neuen europäischen Hydroptilidae (Trichoptera). Entomologische Berichte (Luzern) 36: 101–104.

[B744] Malicky H (1996b) Zwei neue Köcherfliegen aus Jordanien (Trichoptera: Hydroptilidae). Entomologische Zeitschrift 106(5): 203–205.

[B745] Malicky H (1997) Die mediterranen, vorderasiatischen und europäischen Arten der *Hydroptilasparsa*-Gruppe (Trichoptera, Hydroptilidae). Entomologische Berichte (Luzern) 38: 137–153.

[B746] Malicky H (1998a) Köcherfliegen (Trichoptera) von Java und Sumatra, mit Revision einiger Ulmer - Typen aus dem Hamburger Museum. Linzer Biologische Beitrage 30: 795–814.

[B747] Malicky H (1998b) Über einige *Hydroptila*- Arten aus der *occulta*-Gruppe (Trichoptera, Hydroptilidae). Stapfia 55: 395–397.

[B748] Malicky H (1999a) Eine Köcherfliegen-Ausbeute aus dem Jemen (Trichoptera). Esperiana 7: 343–348.

[B749] Malicky H (1999b) Einige Köcherfliegen von der Insel Sokotra (Insecta, Trichoptera). Entomologische Zeitschrift 109(12): 492–495.

[B750] Malicky H (1999c) Köcherfliegen (Trichoptera) vom Marchfeldkanal (Niederösterreich). Zeitschrift der Arbeitsgemeinschaft Österreichischer Entomologen 51: 89–98.

[B751] Malicky H (1999d) Neue Köcherfliegen aus Europa, Asien und von den Seychellen. Braueria 26: 44–48.

[B752] Malicky H (1999e) The net-spinning larvae of the Giant Microcaddisfly, *Ugandatrichia* spp. (Trichoptera, Hydroptilidae). In: Malicky H Chantaramongkol P (Eds) Proceedings of the 9th International Symposium on Trichoptera.Faculty of Science, Chiang Mai University, Chiang Mai, Thailand, 199–204.

[B753] Malicky H (1999f) Eine aktualisierte Liste der österreichischen Köcherfliegen (Trichoptera). Braueria 26: 31–40.

[B754] Malicky H (2001a) Construction behavior for new pupal cases by case-making caddis larvae: reply to Wiggins. (Trichoptera: Integripalpia). Braueria 28: 9.

[B755] Malicky H (2001b) Notes on the taxonomy of Rhadicoleptus, Ptilocolepus and Pseudoneureclipsis. Braueria 28: 19–20.

[B756] Malicky H (2002) Einige Köcherfliegen (Trichoptera) aus Frankreich und Italien. Entomofauna 23(1): 1–12.

[B757] Malicky H (2004a) Atlas of European Trichoptera (2^nd^ edn.). Dordrecht, Netherlands: Springer. 10.1007/978-1-4020-3026-0_1

[B758] Malicky H (2004b) Neue Köcherfliegen (Trichoptera) aus dem Bardia Nationalpark, Nepal. Denisia 13: 291–300. 10.1007/978-1-4020-3026-0_1

[B759] Malicky H (2005a) Die Köcherfliegen Griechenlands. Denisia 17: 1–240. 10.1007/978-1-4020-3026-0_1

[B760] Malicky H (2005b) Ein kommentiertes Verzeichnis der Köcherfliegen (Trichoptera) Europas und des Mediterrangebietes. Linzer Biologische Beitrage 37(1): 533–596. 10.1007/978-1-4020-3026-0_1

[B761] Malicky H (2006) Caddisflies from Bardia National Park, Nepal, with a preliminary survey of Nepalese species (Insecta, Trichoptera). Entomofauna 27: 241–263.

[B762] Malicky H (2007a) A survey of the Trichoptera of Sumatra. In: Bueno-Soria J Barba-Álvarez R Armitage BJ (Eds) Proceedings of the 12th International Symposium on Trichoptera.The Caddis Press, Columbus, Ohio, 175–179.

[B763] Malicky H (2007b) Nachträge und Korrekturen zum Atlas der europäischen Köcherfliegen und zum Verzeichnis der Köcherfliegen Europas (2). Braueria 34: 51–52.

[B764] Malicky H (2008a) Köcherfliegen (Insecta, Trichoptera) aus der Umgebung von Malinau (Kalimantan, Borneo, Indonesien). Linzer Biologische Beitrage 40(1): 833–879.

[B765] Malicky H (2008b) On the migrations of *Ptilocolepus* through the Trichoptera system. Braueria 35: 43–44.

[B766] Malicky H (2009a) Beiträge Kenntnis asiatischer Trichopteren. Braueria 36: 11–58. [Contribution on the knowledge of Asian Trichoptera]

[B767] Malicky H (2009b) Caddisflies from the Island of Sibuyan (Philippines). Entomologica Austriaca : Zeitschrift der Osterreichischen Entomologischen Gesellschaft 16: 9–18.

[B768] Malicky H (2010a) Atlas of Southeast Asian Trichoptera. Chiangmai University, Chiangmai.

[B769] Malicky H (2010b) Köcherfliegen (Trichoptera) von der Noona Dan Expedition 1961–1962 zu den Philippinen, dem Bismarck-Archipel und den Salomon-Inseln. Zeitschrift der Arbeitsgemeinschaft Österreichischer Entomologen 62: 87–95.

[B770] Malicky H (2012) Neue asiatische Köcherfliegen aus neuen Ausbeuten (Insecta, Trichoptera). Linzer Biologische Beitrage 44(2): 1263–1310.

[B771] Malicky H (2013) Synonyms and possible synonyms of Asiatic Trichoptera. Braueria 40: 41–54.

[B772] Malicky H (2014a) Köcherfliegen (Trichoptera) von Taiwan, mit Neubeschreibungen. Linzer Biologische Beitrage 46: 1607–1646.

[B773] Malicky H (2014b) Mißgebildete Köcherfliegen (Trichoptera). Braueria 41: 5–31.

[B774] Malicky H (2014c) Neue Beiträge zur Kenntnis asiatischer und mediterraner Köcherfliegen (Trichoptera). Braueria 41: 43–50.

[B775] Malicky H (2015) Trichopteren von Nosy Bé (Madagaskar): Beschreibungen von neuen Arten und Kommentare zu bekannten. Braueria 42: 41–49.

[B776] Malicky H (2016a) *Hydroptilavectis* Curtis 1834 und *Hydroptilacorsicana* Mosely 1930. Braueria 43: 39.

[B777] Malicky H (2016b) Zur Unterscheidung von *Hydroptilabrissaga* Malicky 1996 und *H.tacheti* Coppa & Malicky 2005. Braueria 43: 22.

[B778] Malicky H (2018) Die Köcherfliegen einiger Gewässer in Nepal: Faunistik und Phänologie, mit Diskussion der Erfassungsmethodik (Trichoptera). Entomologische Zeitschrift Schwanfeld 128(1): 47–60.

[B779] Malicky H (2020) Beiträge zur Kenntnis afrikanischer Köcherfliegen (Insecta, Trichoptera). Linzer Biologische Beitrage 52(1): 509–536.

[B780] Malicky H Chantaramongkol P (1991) Elf neue Köcherfliegen (Trichoptera) aus Thailand und angrenzenden Ländern. Entomologische Zeitschrift mit Insektenborse 101: 80–89.

[B781] Malicky H Chantaramongkol P (1996) Neue Köcherfliegen aus Thailand (Trichoptera). Entomologische Berichte (Luzern) 36: 119–128.

[B782] Malicky H Chantaramongkol P (2007) Beiträge zur Kenntnis asiatischer Hydroptilidae (Trichoptera). Linzer Biologische Beitrage 39: 1009–1099.

[B783] Malicky H González MA (1981) *Hydroptilavilaverde* n. sp. eine neue Köcherfliege (Trichoptera: Hydroptilidae) von der Iberischen Halbins Entomologische Zeitschrift 51(13): 151–152.

[B784] Malicky H Graf W (2012) A small collection of Trichoptera from Ethiopia. Braueria 39: 32–38.

[B785] Malicky H Graf W (2015) Einige neue afrikanische Köcherfliegen (Trichoptera). Braueria 42: 31–35.

[B786] Malicky H Lounaci A (1987) Beitrag zur Taxonomie und Faunistik der Köcherfliegen von Tunesien, Algerien und Marokko (Trichoptera). Opuscula Zoologica Fluminensia 14: 1–20.

[B787] Malicky H Moretti GP (1987) Die *Hydroptilauncinata* Morton 1893 - Verwandtschaft mit Beschreibung einer neuen Art aus Sardinien (Trichoptera: Hydroptilidae). Entomologische Zeitschrift 97(14): 193–196.

[B788] Malicky H Chantaramongkol P Chaibu P Prommi T-O Silalom S Sompong S Thani I (2000) Neue Köcherfliegen aus Thailand (Insecta, Trichoptera) (Arbeit über thailändische Köcherfliegen Nr. 30). Linzer Biologische Beitrage 32(2): 861–874.

[B789] Malicky H O’Connor JP Ashe P Dowling C (2010) Further records of caddisflies (Trichoptera) from Sulawesi, Indonesia, including seven new species. Entomologist’s Monthly Magazine 146: 155–168.

[B790] Malicky H Ivanov VD Melnitsky SI (2011) Beschreibungen von 27 neuen Köcherfliegen - Arten (Insecta, Trichoptera) von Lombok, Bali und Java (Indonesien), mit Kommentaren zu bekannten. Linzer Biologische Beitrage 43(2): 1491–1511.

[B791] Malicky H Ivanov VD Melnitsky SI (2014a) Caddisflies (Trichoptera) from Lombok, Bali and Java (Indonesia), with a discussion of Wallace’s Line. Deutsche Entomologische Zeitschrift 61(1): 3–14. 10.3897/dez.61.7046

[B792] Malicky H Melnitsky S Ivanov V (2014b) Köcherfliegen von den Inseln Ambon (Papua) und Biak (Molukken), mit Beschreibungen von 14 neuen Arten (Trichoptera). Linzer Biologische Beitrage 46: 829–843.

[B793] Malicky H Melnitsky S Ivanov V (2014c) Köcherfliegen von Kambodscha, mit der Beschreibung einer neuenOecetis- Art (Trichoptera). Braueria 41: 33–34.

[B794] Malicky H Melnitsky S Ivanov V (2016) New data on caddisflies (Insecta: Trichoptera) from Lombok (Indonesia) with descriptions of two new species. Zootaxa 4066: 88–94. 10.11646/zootaxa.4066.1.10 27395536

[B795] Malicky H Suwannarat N Laudee P (2018) Köcherfliegen (Trichoptera) aus dem Süden Thailands, mit der Beschreibung von vier neuen Arten. Linzer Biologische Beitrage 50(2): 1319–1328.

[B796] Malicky H Melnitsky SI Ivanov VD (2019) Fauna of caddisflies (Insecta: Trichoptera) of the Phuket Island, Thailand. Russian Entomological Journal 28(4): 425–432. 10.15298/rusentj.28.4.11

[B797] Malicky H Melnitsky SI Ivanov VD (2020) Neue Köcherfliegen (Insecta, Trichoptera) von Papua. Linzer Biologische Beitrage 52(1): 537–552.

[B798] Mangeaud A (1996) Trichopterans in a river of the Gran Chaco, Argentina. Studies on Neotropical Fauna and Environment 31(3–4): 152–155. 10.1076/snfe.31.3.152.13343

[B799] Manuel KL Bohart RM (1993) First report of a twisted-wing insect (Strepsiptera) larva in a caddisfly (Trichoptera). Entomological News 104: 139.

[B800] Manzo V Romero F Rueda Martín P Molineri C Nieto C Rodriguez J Dominguez E (2014) Insectos acuáticos del Parque Provincial Urugua-í, Argentina. Revista de la Sociedad Entomológica Argentina 73: 155–170.

[B801] Marlier G (1943) Trichoptera. Exploration du Parc National Albert. Mission H. Damas (1935–1936) 11: 1–34.

[B802] Marlier G (1965) Les Trichoptères du Musée de Dundo. Publicações Culturais da Compahnia de Diamantes Angola, Lisboa 72: 13–80.

[B803] Marlier G (1978) Sur une collection des Trichoptères de l’Afrique occidentale. Revue de Zoologie Africaine 92(2): 283–302.

[B804] Marlier G Marlier M (1982) Les Trichoptères de l’Ile de la Réunion. Bulletin de l’Institut Royal des Sciences Naturalles de Belgique 54(13): 1–48[, pls 41-11].

[B805] Marlier G Vaillant F (1967) Un *Allotrichia* nouveau du Congo (Trichoptera). Travaux de la Laboratoire Hydrobiologique (Grenoble) 57–58: 25–28.

[B806] Marshall JE (1977) *Hydroptilamartini* sp. n. and *Hydroptilavalesiaca* Schmid (Trichoptera: Hydroptilidae) new to the British Isles. Entomologist’s Gazette 28: 115–122.

[B807] Marshall JE (1979a) A description of the female of *Hydroptilatigurina* Ris (Trichoptera: Hydroptilidae). Entomologist’s Gazette 30(3): 213–214.

[B808] Marshall JE (1979b) A review of the genera of the Hydroptilidae (Trichoptera). Bulletin of the British Museum (Natural History). Entomology 39: 135–239.

[B809] Marshall JS Larson DJ (1982) The adult caddisflies (Insecta: Trichoptera) of insular Newfoundland. Memorial University of Newfoundland Occasional Papers in Biology 6: 1–85.

[B810] Martín L Martínez J González MA (2014) Observaciones sobre los tricópteros (Insecta: Trichoptera) de las montañas orientales de Galicia (Sierra de Ancares, Courel e Invernadeiro). Asociación española de Entomología 38: 67–90.

[B811] Martín L Martínez J González MA (2015) Tricópteros (Insecta: Trichoptera) de la provincia de Albacete (sudeste de España). SABUCO Revista de Estudios Albacetenses 11: 65–97.

[B812] Martín L Martínez J González R González MA (2016) Tricópteros (Insecta, Trichoptera) de la Montaña Palentina (Parque Natural de las Fuentes Carrionas y Fuente Cobre) y de la sierra de La Cabrera (León). Caddisflies (Insecta, Trichoptera) from Montaña Palentina (Parque Natural de las Fuentes Carrionas y Fuente Cobre) and Sierra de la Cabrera (León). Boletin de la Asociacion Espanola de Entomologia 40(3–4): 251–268.

[B813] Martínez J Martín L González MA (2015) Tricópteros (Insecta: Trichoptera) de la serra do Xistral (Galicia, NO de España). Nova Acta Científica Compostelana (Bioloxía) 22: 33–47.

[B814] Martínez J Martín L González MA (2016) Nuevos datos sobre los tricópteros (Inseca, Trichoptera) de Asturias (N. España). Boletin de la Asociacion Espanola de Entomologia 40(1–2): 43–66.

[B815] Martínez Menéndez J González MA (2010) Observaciones sobre los Tricópteros de la Península Ibérica. XI: Tricópteros de Cataluña (NE de España) (Insecta: Trichoptera). Boletin de la Asociacion Espanola de Entomologia 33: 337–353.

[B816] Martynov AV (1910) Les Trichoptères de la Sibèrie et des régions adjacentes. II. La sous f. des Brachycentrinae, les fam. des Molannidae, Leptoceridae, Hydropsychidae, Philopotamidae, Polycentropidae, Psychomyidae, Rhacophilidae et des Hydroptilidae. Annuaire du Musée Zoologique de l’Académie Impériale des Sciences de Saint Pétersbourg 15: 351–429.

[B817] Martynov AV (1913a) Contribution to the knowledge of the Trichopterous fauna of the Caucasus. Travaux Laboratoire Zoologie Université Warsaw 1913: 1–111. [In Russian]

[B818] Martynov AV (1913b) Contributions à la faune des Trichoptères du Caucase. II. Trichoptères de la province de batoum et des environs du Novyj Afon. Horae Societatis Entomologicae Rossicae 40: 30 pp.

[B819] Martynov AV (1924) Rucheiniki (Trichoptera) [caddisflies (Trichoptera)] [in Russian]. In: Bogdanova-Kat’kova (Ed.) Prakticheskaya entomologiya, Vol. 5. Leningrad, [iv +] 384 pp.

[B820] Martynov AV (1927) Contributions to the aquatic entomofauna of Turkestan. I. TrichopteraAnnulipalpia. Annuaire du Musée Zoologique de l’Académie Impériale des Sciences de Saint Pétersbourg 28: 162–193.

[B821] Martynov AV (1929) On a collection of Trichoptera from the River Bija and from the vicinities of the Lake Teletzkoje. Konowia, Vienna 8: 293–311.

[B822] Martynov AV (1933) On an interesting collection of Trichoptera from Japan. Annotationes Zoologicae Japonenses 14: 139–156.

[B823] Martynov AV (1934) Tableaux analytiques de la faune de l’U.R.S.S. TrichopteraAnnulipalpia. I. Opred. Faune SSSR 13: 343.

[B824] Martynov AV (1935) On a collection of Trichoptera from the Indian Museum. Part I. Annulipalpia. Records of the Indian Museum 37(2): 93–209. 10.26515/rzsi/v37/i2/1935/162993

[B825] Martynov AV (1936) On a collection of Trichoptera from the Indian Museum. Part II. Integripalpia. Records of the Indian Museum 38(3): 239–306. 10.26515/rzsi/v38/i3/1936/162320

[B826] Masteller EC (1993) The Trichoptera (caddisflies) of Presque Isle State Park and Lake Erie, Erie County, Pennsylvania. Journal of the Pennsylvania Academy of Science 67(3): 132–136.

[B827] Masteller EC Flint Jr OS (1992) The Trichoptera (Caddisflies) of Pennsylvania: An annotated checklist. Journal of the Pennsylvania Academy of Science 66: 68–78.

[B828] Mathis ML Bowles DE (1989) A new microcaddisfly genus (Trichoptera: Hydroptilidae) from the interior highlands of Arkansas, U.S.A. Journal of the New York Entomological Society 97: 187–191.

[B829] Mathis ML Bowles DE (1990) Three new species of microcaddisflies (Trichoptera: Hydroptilidae) from the Ozark Mountains, U.S.A. Proceedings of the Entomological Society of Washington 92: 86–92.

[B830] Mathis ML Bowles DE (1992) A preliminary survey of the Trichoptera of the Ozark mountains, Missouri, U.S.A. Entomological News 103(1): 19–29.

[B831] Matsumura S (1931) 6000 illustrated insects of Japan-empire. Tokyo, Japan, The Tōkō Shoin.

[B832] Mattern D (2015) The fauna of caddisflies of Nepal (Insecta: Trichoptera). Verein der Freunde & Förderer des Naturkundemuseums Erfurt e. 5: 487–521.

[B833] McAuliffe JR (1982) Behavior and life history of *Leucotrichiapictipes* (Banks) (Trichoptera: Hydroptilidae) with special emphasis on case reoccupancy. Canadian Journal of Zoology 60(7): 1557–1561. 10.1139/z82-204

[B834] McAuliffe JR (1984) Competition for space, disturbance, and the structure of a benthic stream community. Ecology 65(3): 894–908. 10.2307/1938063

[B835] McIntosh MD Benbow ME Burky AJ (2002) Effects of stream diversion on riffle macroinvertebrate communities in a Maui, Hawaii, stream. River Research and Applications 18(6): 569–581. 10.1002/rra.694

[B836] McIntosh MD Benbow ME Burky AJ (2003) Effect of water removal on introduced caddisflies from a tropical mountain stream. Annales de Limnologie 39(4): 297–306. 10.1051/limn/2003024

[B837] McLachlan R (1862) Characters of new species of exotic Trichoptera; also of one new species inhabiting Britain. Transactions of the Entomological Society of London, 3^rd^ Series 1: 301–311. 10.1111/j.1365-2311.1862.tb00608.x

[B838] McLachlan R (1865) Trichoptera Britannica; a monograph of the British species of caddis-flies. Transactions of the Entomological Society of London, Series 3 5: 1–184.

[B839] McLachlan R (1875) Neuroptera. In: Fedtschenko AP (Ed.) Reise in Turkestan von Alexis Fedtschenko, auf Veranlassung des General-Gouverneurs von Turkestan, General von Kaufmann., herausgegeben von der Gesellschaft der Freunde der Naturwissenschaften in Moskau. Zoogeographicheskia Izsledovania, Tipografiya M. Strasvuleyicha, St. Petersburg, 2(5), 1–60, 64 pls. [in German]

[B840] McLachlan R (1880) A monographic revision and synopsis of the Trichoptera of the European fauna, Part 9. John van Voorst London, 501–523 with supplement, xiii–lxxxiv, pls 52–59.

[B841] McLachlan R (1884) A monographic revision and synopsis of the Trichoptera of the European fauna. First additional supplement (with seven plates). London, John van Voorst, 76 pp.[, 7 pls]

[B842] Medvedev GS [Ed.] (1998) Keys to the insects of the European part of the USSR. Volume IV. Part VI: Megaloptera, Raphidioptera, Neuroptera, Mecoptera and Trichoptera. Enfield, Science Publishers, Inc. i–xvii, 302 pp.

[B843] Melnitsky SI Ivanov VD (2016) New species of caddisflies (Insecta, Trichoptera) from the Rovno Amber. Zoosymposia 10(1): 278–291. 10.11646/zoosymposia.10.1.26

[B844] Melnitsky SI Ivanov VD (2017) Contribution to the caddis fauna (Trichoptera) of the Vologda Region, Russia. Braueria 44: 19.

[B845] Melnitsky S Malicky H (2008) Trichoptera from Chang island, southeastern Thailand, with the description of three new species. Braueria 35: 25–27.

[B846] Melnitsky SI Ivanov VD Malicky H (2017) A small collection of caddisflies (Trichoptera) from northwestern Turkey). Braueria 44: 6.

[B847] Melnitsky SI Ivanov VD Malicky H (2019) Fauna of Caddisflies (Trichoptera) of Langkawi Island, Malaysia. Entomological Review 99(4): 534–543. 10.1134/S0013873819040158

[B848] Mendez PK Myers MJ Damerow JE Lew C Resh VH (2019) Species occurrence and distribution of Trichoptera (caddisflies) in California. Zoosymposia 14(1): 113–133. 10.11646/zoosymposia.14.1.15

[B849] Mey W (1978a) Köcherfliegen aus dem Ural (UdSSR). Entomologische Nachrichten 22(7,8): 122–125.

[B850] Mey W (1978b) Köcherfliegen aus Mittelasien. Entomologisches Nachrichtenblatt (Vienna, Austria) 22(2): 27–28.

[B851] Mey W (1981) Die von R. Jung und A Müller in Mittelasien gesammelten Köcherfliegen. Deutsche Entomologische Zeitschrift für Natur Forschung 28(1–3): 55–66. 10.1002/mmnd.19810280107

[B852] Mey W (1990) Neue Köcherfliegen von den Philippinen (Trichoptera). Opuscula Zoologica Fluminensia 57: 1–19.

[B853] Mey W (1991) Wenig bekannte Köcherfliegen in Deutschland (Insecta, Trichoptera). Entomologische Nachrichten und Berichte 35(4): 270–273.

[B854] Mey W (1992) Beschreibung von vier neuen Köcherfliegen aus Ostafrika (Insecta, Trichoptera). Mitteilungen aus dem Zoologischen Museum in Berlin 68(2): 259–265. 10.1002/mmnz.19920680206

[B855] Mey W (1993) Beschreibung von vier neuen Köcherfliegen aus Nord-China (Trichoptera, Annulipalpia). Deutsche Entomologische Zeitschrift für Natur Forschung 40(2): 333–340. 10.1002/mmnd.19930400214

[B856] Mey W (1995) Beitrag zur Kenntnis der Köcherfliegenfauna der Philippinen, I. (Trichoptera). Deutsche Entomologische Zeitschrift für Natur Forschung 42(1): 191–209. 10.1002/mmnd.19950420116

[B857] Mey W (1996) Die Köcherfliegenfauna des Fan Si Pan-Massivs in Nord-Vietnam. 1. Deschreibung neuer und endemischer arten aus den Unterordunugen Spicipalpia und Annulipalpia (Trichoptera). Beiträge zur Entomologie 46(1): 39–65.

[B858] Mey W (1998a) Contribution to the knowledge of the caddisflies of the Philippines 2. The species of the Mt. Agtuuganon Range on Mindanao (Insecta: Trichoptera). Nachrichten des Entomologischen Vereins Apollo (Supplementum 17): 537–576.

[B859] Mey W (1998b) Contribution to the knowledge of the caddisfly fauna of the Philippines, III (Insecta: Trichoptera). Entomofauna 19(1): 1–32.

[B860] Mey W (2003a) *Agrayleataymyrensis* n. sp. - eine neue arktische Köcherfliege aus Sibirien (Trichoptera, Hydroptilidae). Entomologische Nachrichten und Berichte 47(1): 39–40.

[B861] Mey W (2003b) Contribution to the knowledge of the caddisfly fauna of the Philippines, V (Insecta, Trichoptera). Insecta Koreana 20: 425–452.

[B862] Mey W (2005a) The Fan Si Pan Massif in north Vietnam - towards a reference locality for Trichoptera in SE Asia. In: Tanida K Rossiter A (Eds) Proceedings of the 11th International Symposium on Trichoptera.Tokai University Press, Kanagawa, 273–284.

[B863] Mey W (2005b) Über die Dynamik der Köcherfliegenfauna eines stehenden Gewässers bei Potsdam, Teil 2 (Insecta: Trichoptera). Lauterbornia 54: 115–122.

[B864] Mey W (2006a) Ein Blick zurück: Köcherfliegen am Rhein bei St. Goarshausen im Jahre 1890 (Insecta, Trichoptera). Lauterbornia 56: 155–167.

[B865] Mey W (2006b) Notes on the caddisfly fauna of Lake Matano in Central Sulawesi - (Insecta, Trichoptera). Beiträge zur Entomologie 56(1): 199–212. 10.21248/contrib.entomol.56.1.199-212

[B866] Mey W (2007) A new species of the genus *Stactobia* McLachlan from Ethiopia (Trichoptera: Hydroptillidae). Acta Zoologica Academiae Scientiarum Hungaricae 53(Supplement 1): 225–229.

[B867] Mey W (2011) Observations on the caddisfly fauna (Insecta, Trichoptera) of the lower Orange and Fish Rivers in southern Africa with the description of a new species. Zoosymposia 5(1): 338–349. 10.11646/zoosymposia.5.1.26

[B868] Mey W (2014) Die Köcherfliegenfauna des NSG Zarth bei Treuenbrietzen - ein Refugium für seltene Arten (Insecta, Trichoptera). Märkische Entomologische Nachrichten 16(2): 175–192.

[B869] Mey W (2016) A case study on the Trichoptera fauna of springs in the escarpment mountains of southern Africa (Insecta, Trichoptera). Zoosymposia 10(1): 301–311. 10.11646/zoosymposia.10.1.28

[B870] Mey W de Moor FC (2019) The Trichoptera (Insecta) of the lower Kunene River in Namibia and Angola. Zoosymposia 14(1): 134–150. 10.11646/zoosymposia.14.1.16

[B871] Mey W Freitag H (2019) new species of caddisflies (Insecta: Trichoptera) from emergence traps at streams in central Palawan, Philippines. Aquatic Insects 40(3): 207–235. 10.1080/01650424.2019.1617423

[B872] Mey W Freitag H (2020) Diversity of Trichoptera emergence and their longitudinal distribution along streams in central Palawan, the Philippines. Zoosymposia 18: 053–062. 10.11646/zoosymposia.18.1.9

[B873] Mey W Joost W (1990) *Rhyacopsychemutisi* n. sp. - A new microcaddisfly with an unusual larva from Colombia (Trichoptera, Hydroptilidae). Studies on Neotropical Fauna and Environment 25(3): 133–138. 10.1080/01650529009360813

[B874] Mey W Nozaki T (2006) The caddisflies from the “All-continent expert tour” in central Japan 2003. Braueria 33: 23–25.

[B875] Mey W Ospina-Torres R (2018) Contribution to the Trichoptera fauna of the river La Vieja, Bogotá, Colombia (Insecta: Trichoptera). Zootaxa 4505(1): 023–040. 10.11646/zootaxa.4504.1.2 30486034

[B876] Milne LJ (1934) Studies in North American Trichoptera, 1. Privately printed, Cambridge, Massachusetts, 19 pp.

[B877] Milne LJ (1936) Studies in North American Trichoptera, 3. Privately printed, Cambridge, Massachusetts, 74 pp. [+ 2 plates]

[B878] Minakawa N Arefina TI Ito T Nozaki T Kuhara N Nishimoto H Uenishi M Teslenko VA Bennett DJ Gara RI Kurowski KL Oberg PBH (2004) Caddisflies (Trichoptera) of the Kuril Archipelago. Bulletin of the Hokkaido University Museum 1: 49–80.

[B879] Mirmoayedi A Malicky H (2002) An updated check-list of caddisflies (Insecta, Trichoptera) from Iran, with new records. Zoology in the Middle East 26(1): 163–168. 10.1080/09397140.2002.10637932

[B880] Miserendino ML Brand C (2007) Trichoptera assemblages and environmental features in a large arid Patagonian river. Fundamental and Applied Limnology 169(4): 307–318. 10.1127/1863-9135/2007/0169-0307

[B881] Mogensen B (1971) Vårfluen *Oxyethirafrici* (Klapálek 1890), ny for Danmark (Trichoptera). Flora and Fauna 77: 13–14.

[B882] Monson MP Holzenthal RW (1993) A new species and new records of *Oxyethira* (Trichoptera: Hydroptilidae) from Minnesota. Journal of the North American Benthological Society 12(4): 438–443. 10.2307/1467625

[B883] Moreno LAS Desidério GR de Souza WRM Lima LRC (2020) Updated checklist of caddisflies (Insecta: Trichoptera) from the state of Piauí, Northeast Brazil, including a new species and new geographical records. Zootaxa 4838(2): 257–272. 10.11646/zootaxa.4838.2.6 33056825

[B884] Moretti GP (1981) New Trichoptera species and subspecies found in Italy. In: Moretti GP (Ed.) Proceedings of the 3rd International Symposium on Trichoptera.Dr. W. Junk, The Hague, 165–192. 10.1007/978-94-009-8641-1_22

[B885] Moretti GP Bicchierai MC (1979) Struttura androconiale di *Hydroptilaaegyptia* Ulm. (Trichoptera). Rivista di Idrobiologia 18(2): 173–195.

[B886] Moretti GP Cianficconi F (1963) Sulle formazioni androconiali di alcune specie di Tricotteri. Atti della Accademia Nazionale Italiana di Entomologia Rendiconti 11: 199–202.

[B887] Moretti GP Cianficconi F (1981) First list of Italian Trichoptera. In: Moretti GP (Ed.) Proceedings of the 3rd International Symposium on Trichoptera.Dr. W. Junk, The Hague, 199–211. 10.1007/978-94-009-8641-1_24

[B888] Moretti GP Corallini-Sorcetti C (1978) Biologia e morfologia di *Hydroptilasparsa* Curt. reperita in un biotopo reico de acque salmastre (InsectaTrichoptera). Bolletino di Zoologia, Supplement 45: 36–36. 10.1080/11250007809440224

[B889] Moretti GP Viganò A Taticchi MI (1966) Effetto attrattivo deila ‘luce nera’ nei confronti di *Ecnomustenellus* Ramb. (Trichoptera-Psychomyidae). Atti della Accademia Nazionale Italiana di Entomologia Rendiconti 13(2): 88–89.

[B890] Moretti GP Tucciarelli F Cruccolini E (1978) La larva di *Hydroptilaaegyptia* Ulm. Rivista di Idrobiologia 17(1): 27–84.

[B891] Moretti GP Cianficconi F Tucciarelli F (1981a) Ripartizione dei tricotteri nel sistema idrico del Lago di Piediluco e nella Cascata delle Marmore (Umbria-Terni). Studi Trentini di Scienze Naturali Acta Biologica 58: 315–373.

[B892] Moretti GP Tucciarelli F Cianficconi F (1981b) Composizione e consistenza del popolamento tricotterologico nel ‘ecosistema fluviale del medio Po (Caorso-Piacenza). Rivista di Idrobiologia 20(1): 231–244.

[B893] Moretti GP Cianficconi F Corallini C (1996) Caddisflies in Italian springs. Crunoecia 5: 295–298.

[B894] Morris R (2016) First and second caddis records for Leicestershire and Rutland. British Journal of Entomology and Natural History 29: 246–247.

[B895] Morse JC (1974) New caddisflies (Trichoptera) from southern Africa. Journal of the Kansas Entomological Society 47(3): 328–344.

[B896] Morse JC (1997) Checklist of World Trichoptera. In: Holzenthal RW Flint Jr OS (Eds) Proceedings of the 8th International Symposium on Trichoptera.Ohio Biological Survey, Columbus, 339–342.

[B897] Morse JC [Ed.] (2006) Trichoptera World Checklist. [Available from] http://entweb.sites.clemson.edu/database/trichopt [accessed 1 March 2022]

[B898] Morse JC Hamilton SW Hoffman KM (1989) Aquatic insects of Lake Jocassee catchment in North and South Carolina, with description of four new species of caddisflies (Trichoptera). Journal of the Elisha Mitchell Scientific Society 105(1): 14–33.

[B899] Morse WJ Blickle RL (1953) A checklist of the Trichoptera (caddiflies) of New Hampshire. Entomological News 14: 68–73.

[B900] Morse JC Tanida K Vshivkova TS (2001) The caddisfly fauna of four great Asian lakes: Baikal, Hovsgol, Khanka, and Biwa. In: Bae YJ (Ed.) The 21st Century and Aquatic Entomology in East Asia: Proceedings of the 1st Symposium of the Aquatic Entomological Societies of East Asia.Korean Society of Aquatic Entomology, Korea, 97–116.

[B901] Morse JC Rozhkova NA Prather AL Vshivkova TS Harris SC (2006) Trichoptera of Mongolia, with emphasis on the Hövsgöl drainage fauna. In: Goulden CE Sitnikova T Gelhaus J Boldgiv B (Eds) The geology, biodiversity and ecology of Lake Hövsgöl (Mongolia).Backhuys, Leiden, 305–332.

[B902] Morton KJ (1886) On the case, etc. of *Agrayleamultipunctata*, Curt. Entomologist’s Monthly Magazine 22: 269–272.

[B903] Morton KJ (1887) On the cases of *Oxyethiracostalis* Curt. Entomologist’s Monthly Magazine 23: 201–202.

[B904] Morton KJ (1888) The larva and case of *Ithytrichialamellaris*, Eaton, with references to other species of Hydroptilidae. Entomologist’s Monthly Magazine 24: 171–175.

[B905] Morton KJ (1893) Notes on Hydroptilidae belonging to the European fauna, with descriptions of new species. Transactions of the Royal Entomological Society of London 1893: 75–82[, pls 75–76]. 10.1111/j.1365-2311.1893.tb02054.x

[B906] Morton KJ (1896) Hydroptilidae collected in Algeria by the Rev. A. E. Eaton. Entomologist’s Monthly Magazine 32: 102–104.

[B907] Morton KJ (1898) Two new Hydroptilidae from Scotland and Algeria respectively. Entomologist’s Monthly Magazine 34: 107–109.

[B908] Morton KJ (1899a) Entomological notes from Glen Lochay and Loch Tay including record of an *Oxyethira* new to Britain. Entomologist’s Monthly Magazine 35: 53–55.

[B909] Morton KJ (1899b) Neuroptera and Trichoptera observed in Wigtownshire during July 1899, including two species of Hydroptilidae new to the British list. Entomologist’s Monthly Magazine 35: 278–281.

[B910] Morton KJ (1902) A new Indian micro-Trichopteron. The Entomologist’s Monthly Magazine (series 2) 13: 283.

[B911] Morton KJ (1904) Further notes on the Hydroptilidae belonging to the European fauna, with descriptions of new species. The Transactions of the Entomological Society of London 1904(2): 323–328. 10.1111/j.1365-2311.1904.tb02748.x

[B912] Morton KJ (1905) North American Hydroptilidae. Bulletin of the New York State Museum 86: 63–75[, plates 13–15].

[B913] Mosely ME (1919a) Scent-organs in the genus *Hydroptila* (Trichoptera). Transactions of the Royal Entomological Society of London 1919(3–4): 393–397[, pls 318–319]. 10.1111/j.1365-2311.1920.tb00011.x

[B914] Mosely ME (1919b [1920]) A new *Hydroptila*. Transactions of the Entomological Society of London for the year 1919: 391–392. 10.1111/j.1365-2311.1920.tb00010.x

[B915] Mosely ME (1922) Two new British species of *Hydroptila*. Transactions of the Entomological Society of London for the year 1922: 178–180. 10.1111/j.1365-2311.1922.tb02829.x

[B916] Mosely ME (1923) Scent-organs in the genus *Hydroptila* (Trichoptera). Transactions of the Royal Entomological Society of London 1923(3–4): 291–294[, pls 214–215].

[B917] Mosely ME (1924) New Zealand Hydroptilidae (order Trichoptera). Transactions of the New Zealand Institute 55: 670–673.

[B918] Mosely ME (1930a) Corsican Trichoptera. Eos-Revista Española Entomologia 6: 147–184.

[B919] Mosely ME (1930b) New European Trichoptera and Plecoptera. The Transactions of the Entomological Society of London 78(2): 237–253. 10.1111/j.1365-2311.1930.tb00386.x

[B920] Mosely ME (1932) Corsican Trichoptera and Neuroptera (s. l.). Eos (Washington, D. C.) 8: 165–184.

[B921] Mosely ME (1933) The genus *Stactobia*, McLach. (Trichoptera). Stylops 2: 162–165. 10.1111/j.1365-3113.1993.tb00994.x

[B922] Mosely ME (1934a) New exotic Hydroptilidae. Transactions of the Royal Entomological Society of London 82: 137–163. 10.1111/j.1365-2311.1934.tb00031.x

[B923] Mosely ME (1934b) New Trichoptera in the French Pyrenees. Annals and Magazine of Natural History 10(13): 433–444. 10.1080/00222933408654835

[B924] Mosely ME (1937a) A new Corsican *Hydroptila* species (Trichoptera). Proceedings of the Royal Entomological Society of London 6: 121–122. 10.1111/j.1365-3113.1937.tb00308.x

[B925] Mosely ME (1937b) Mexican Hydroptilidae (Trichoptera). Transactions of the Royal Entomological Society of London 86(10): 151–189. 10.1111/j.1365-2311.1937.tb00242.x

[B926] Mosely ME (1939a) The Brazilian Hydroptilidae (Trichoptera). Novitates Zoologicae 41: 217–239.

[B927] Mosely ME (1939b) The British caddis flies (Trichoptera). A collector’s handbook. With an introduction by N.D.Riley. Geo. Routledge & Sons, Ltd, London, [xiii +] 320 pp.

[B928] Mosely ME (1939c) Trichoptera collected by J. Omer Cooper, Esq., in Egypt. Annals & Magazine of Natural History 11(3): 43–48. 10.1080/03745481.1939.9723573

[B929] Mosely ME (1939d) Trichoptera. Ruwenzori Expedition 1934–35. British Museum 3: 1–40. [Natural History]

[B930] Mosely ME (1948a) Trichoptera collected by Miss R. H. Lowe at Lake Nyasa. Annals & Magazine of Natural History 12(1): 31–47. 10.1080/00222934808653886

[B931] Mosely ME (1948b) Trichoptera. Expedition to South-West Arabia 1937–8. British Museum 1(9): 67–86. [Natural History]

[B932] Mosely ME Kimmins DE (1953) The Trichoptera (Caddis-Flies) of Australia and New Zealand. London, British Museum (Natural History), 550 pp. 10.5962/bhl.title.118696

[B933] Moubayed Z Botosaneanu L (1985) Recherches sur les Trichoptères du Liban et principalement des bassins supérieurs de l’Oronte et du Litani (Insecta: Trichoptera). Bulletin Zöologisch Museum. Universiteit van Amsterdam 10(11): 61–76.

[B934] Moulton II SR Harris SC (1997) New species of southwestern Nearctic microcaddisflies (Trichoptera: Hydroptilidae). Proceedings of the Entomological Society of Washington 99(3): 494–501.

[B935] Moulton SR II Harris SC (1999) Redescriptions of the *Oxyethiraaeola* Group species in North America (Trichoptera: Hydroptilidae): clarification of a taxonomic enigma. Journal of the North American Benthological Society 18(4): 545–552. 10.2307/1468386

[B936] Moulton II SR Stewart KW (1996) Caddisflies (Trichoptera) of the Interior Highlands of North America. Memoirs of the American Entomological Institute 56: 1–313.

[B937] Moulton II SR Stewart KW (1997) A preliminary checklist of Texas caddisflies (Trichoptera). In: Holzenthal RW Flint Jr OS (Eds) Proceedings of the 8th International Symposium on Trichoptera.Ohio Biological Survey, Columbus, Ohio, 349–353.

[B938] Moulton II SR Petr D Stewart KW (1993) Caddisflies (Insecta: Trichoptera) of the Brazos River Drainage in North-Central Texas. The Southwestern Naturalist 38(1): 19–23. 10.2307/3671639

[B939] Moulton II SR Stewart KW Young KL (1994) New records, distribution and taxonomic status of some northern Arizona caddisflies (Trichoptera). Entomological News 105(3): 164–174.

[B940] Moulton II SR Harris SC Slusark JP (1999) The microcaddisfly genus *Ithytrichia* Eaton (Trichoptera: Hydroptilidae) in North America. Proceedings of the Entomological Society of Washington 101: 233–241.

[B941] Müller F (1879a) Notes on the cases of some South Brazilian Trichoptera. Transactions of the Royal Entomological Society of London 4: 131–144.

[B942] Müller F (1879b) Über Phryganiden (letters to his brother). Zoologischer Anzeiger 2: 38–40, 180–182, 283–284, 404–407.

[B943] Müller F (1880a [1881]) Über die von den Trichopterenlarven der Provinz Santa Catharina verfertigten Gehäuse. Zeitschrift für Wissenschaftliche Zoologie 35: 47–87[, pls 44–45].

[B944] Müller F (1880b [1878]) Sobre as casas construidas pelas larvas de insectos Trichopteros da Provincia de Santa Catharina. Archivos do Museu Nacional, Rio de Janeiro 3 (1878) : 99–134, 209–214[, pls 138–111].

[B945] Müller F (1887) Eine deutsche *Lagenopsyche.* Entomologisches Nachrichtenblatt (Vienna, Austria) 13: 337–340.

[B946] Müller F (1921) Briefe und noch nicht veröffentliche Abhandlungen aus dem Nachlass 1854–1897. In: Möller A (Ed.) Fritz Müller: Werke, Briefe und Leben, 2.G. Fischer, Jena, 383–642.

[B947] Muñoz-Quesada F (2000) Especies del orden Trichoptera (Insecta) en Colombia. Biota Colombiana 1: 267–288. [Colombian species of the order Trichoptera (Insecta)]

[B948] Murgoci A Botnariuc N Botosaneanu L (1948) Sur la présence en Yougoslavie d’une espèce nouvelle de Trichoptère, *Ithytrichiabosniaca* n. sp. Annales Scientifiques de l’Université de Jassy 2: 1–22.

[B949] Murray-Stoker KM Morse JC Genco MS Pham HT (2020) New and variable caddisfly species (Insecta: Trichoptera) from Bach Mã National Park in Vietnam. Zoosymposia 18: 093–102. 10.11646/zoosymposia.18.1.12

[B950] Muzón J Spinelli GR Pessacq P von Elllenrieder N Estevez AL Marino PI Goodwyn PJP Anrisano EB Díaz F Fernández LA Mazzucconi S Rossi G Salomón OD (2005) Insectos acuáticos de la Meseta del Somuncura, Patagonia, Argentina. Inventario preliminar. Revista de la Sociedad Entomológica Argentina 64(3–4): 64.

[B951] Myers LW Kondratieff BC Mihuc TB Ruiter DE (2011) The Mayflies (Ephemeroptera), Stoneflies (Plecoptera), and Caddisflies (Trichoptera) of the Adirondack Park (New York State). Transactions of the American Entomological Society 137: 63–140. 10.3157/061.137.0118

[B952] Naranjo López C González Lazo DD (2005) Situación actual del estudio del orden Trichoptera en Cuba. Boletin de la SEA 36: 147–152.

[B953] Navara T Lukáš J Cíbik J Chvojka P (2020) Contribution to the knowledge of the caddisfly fauna (Trichoptera) of the Váh River (the Danube Basin, Slovakia). Biodiversity and Environment 12(1): 42–51.

[B954] Navás L (1916) Trichópteros de Aragon. Revista de la Academia de Ciencias Exactas, Físicas. Químicas y Naturales de Zaragoza 1916: 73–85.

[B955] Navás L (1917a) Tricópteros nuevos de Espana (quarta serie). Broteria 15: 63–68.

[B956] Navás L (1917b) Tricópteros nuevos de Espana (tercera serie). Broteria 15: 16–28.

[B957] Neboiss A (1963) The Trichoptera types of species described by J. Curtis. Beiträge zur Entomologie 13(5–6): 582–635.

[B958] Neboiss A (1977) A taxonomic and zoogeographic study of Tasmanian caddis flies (Insecta: Trichoptera). Memoirs of the National Museum of Victoria 38: 1–208[, pls 201–203]. 10.24199/j.mmv.1977.38.01

[B959] Neboiss A (1986) Atlas of Trichoptera of the SW Pacific-Australian Region. Series Entomologica 38. Dr. W. Junk, Hingham, Massachusetts, 286 pp. 10.1007/978-94-009-4814-3

[B960] Neboiss A (2002) New genera and species, and new records, of Tasmanian Trichoptera (Insecta). Papers and Proceedings of the Royal Society of Tasmania 136: 43–82. 10.26749/rstpp.136.43

[B961] Nelson JM Panter AJ (1984) *Hydroptilavalesiaca* Schmid (Trichoptera: Hydroptilidae) from Whitlaw Mosses, near Selkirk, southern Scotland. Entomologist’s Gazette 35(1): 39–40.

[B962] Neu PJ (2010) Identification of the female species of the *Hydroptila*-group occurring in Germany. Lauterbornia 71: 147–155.

[B963] Newell RL Ruiter D Strenge D (2001) Adult caddisfly (Trichoptera) phenology in two cold-desert endorheic spring-streams in Washington State. The Pan-Pacific Entomologist 77: 190–195.

[B964] Nielsen A (1948) Postembryonic development and biology of the Hydroptilidae. A contribution to the phylogeny of the caddis flies and to the question of the origin of the case-building instinct. Biologiske Skrifter 5: 1–200.

[B965] Nielsen A (1951) *Hydroptilaocculta* Eaton, new. to the Danish fauna. With descriptions of the specific characters. Entomologiske Meddelelser 26: 122–129.

[B966] Nimmo AP (1996) Bibliographia Trichopterorum: A World Bibliography of Trichoptera, Vol. 1, 1961–1970. Pensoft Publishers, Sofia-Moscow-St. Petersburg, [viii +] 597 pp.

[B967] Nógrádi S (1985) Further caddisfly species new to the Hungarian fauna (Trichoptera). Folia Entomologica Hungarica 46: 129–135.

[B968] Nógrádi S (1986) New data to the caddisfly fauna of Hungary (Trichoptera). Folia Entomologica Hungarica 47(1–2): 135–140.

[B969] Nógrádi S (1994) New data to the caddisfly (Trichoptera) fauna of Hungary, III. Folia Entomologica Hungarica 55: 271–280.

[B970] Nógrádi S (2001) Further data to the caddisflies (Trichoptera) of Hungary. Folia Historico Naturalia Musei Matraensis 25: 83–90.

[B971] Nógrádi S Uherkovich A (1994) The Trichoptera fauna of the Lake Balaton and its catchment area (Hungary). A Janus Pannonius Múzeum Évkönyve 38: 27–45.

[B972] Nógrádi S Uherkovich Á (1998) Újabb eredmények a Duna-Dráva Nemzeti Park Dráva menti területei tegzes (Trichoptera) faunájának kutatásában. Dunántúli Dolgozatok (A). Természettudományi Sorozat 9: 331–358.

[B973] Nógrádi S Uherkovich Á (2001) Somogy megye tegzeseinek (Trichoptera) jegyzéke. Natura Somogyiensis 1: 295–301. 10.24394/NatSom.2001.1.295

[B974] Nógrádi S Uherkovich Á (2002) On the caddisflies (Trichoptera) from the catchment area of the rivers Mura and Kerka, southwest Hungary. Somogyi Muzeumok Kozlemenyei 15 Termeszettudomany 2002: 129–144.

[B975] Nogueira DS Cabette HSR (2011) Novos registros e notas sobre distribução geográfica de Trichoptera Kirby, 1813 (Insecta) do Estado de Mato Grosso, Brasil. Biota Neotropica 11(2): 347–355. 10.1590/S1676-06032011000200033

[B976] Nowinszky L Kiss O Szentkirályi F Puskás J (2011) Tegzes (Trichoptera) fajok fénycsapdás fogásának változása eltéró holdfázisokban. Changes of the light-trap catch of caddisflies (Trichoptera) species in different moon phases. e-Acta Naturalia Pannonica 2(3): 227–242.

[B977] Nozaki T (2010) Caddisflies of Tsukanoiri-ike pond, Nagoya, central Japan (a preliminary report). Nature of Irrigation Pond 49: 2.

[B978] Nozaki T Tanida K (2007) The caddisfly fauna of a huge spring-fed stream, the Kakida River, in central Japan. In: Bueno-Soria J Barba-Álvarez R Armitage BJ (Eds) Proceedings of the 12th International Symposium on Trichoptera.The Caddis Press, Columbus, Ohio, 243–255.

[B979] Nozaki T Togashi S Sato T (2016) The caddisfly fauna of a small spring brook in the Jimoto-yusui, Niigata, central Japan. Zoosymposia 10(1): 323–330. 10.11646/zoosymposia.10.1.30

[B980] Nozaki T Ito T Tojo K (2019) Caddisflies collected using a Malaise trap at a spring-fed brook of Shimauchi-yusui in the Matsumoto Basin, central Japan: Fauna and phenology. Zoosymposia 14(1): 165–176. 10.11646/zoosymposia.14.1.18

[B981] Nybom O (1948) Iter entomologicum et botanicum ad Insulas Madeiram et Azores anno 1938 a Richard Frey, Ragnar Stora et Carl Cedercreutz facturn. No 14. The Trichoptera of the Atlantic Islands. Commentationes Biologicae, Helsinki 8(14): 1–19.

[B982] Nybom O (1954) Entomological results of the Finnish Expedition to the Canary Islands 1947–51. No. 9. Some additions to the Trichopterous fauna of the Canary islands. Commentationes Biologicae, Helsingfors 14: 1–3.

[B983] Nybom O (1960) List of Finnish Trichoptera. Fauna Fennica 6: 1–56.

[B984] Nybom O (1963) Further notes on the Trichopterous fauna of Madeira. Notulae Entomologicae 43: 114–117.

[B985] Nybom O (1965) Trichoptera from Madeira and the Azores. Boletim do Museu Municipal do Funchal 19: 88–94.

[B986] Nybom O (1983) *Oxyethiraklingstedti* sp. n. (Trichoptera, Hydroptilidae) from Finland. Notulae Entomologicae 63: 65–66.

[B987] O’Connor JP (2015) A catalogue and atlas of the caddisflies (Trichoptera) of Ireland. Occasional Papers of the Irish Biogeographical Society 11: [viii +] 646 pp.

[B988] O’Connor JP (1978) *Hydroptilatigurina* Ris new to Ireland with notes on *Apataniawallengreni* McLachlan and *Limnephilusbinotatus* Curtis (Insecta: Trichoptera). The Irish Naturalists’ Journal 19(6): 191–192.

[B989] O’Connor JP (1982) *Ithytrichiaclavata* (Trichoptera: Hydroptilidae) a caddisfly new to Ireland. The Irish Naturalists’ Journal 20: 548–549.

[B990] O’Connor JP (2013) The caddisflies (Trichoptera) of Co. Kerry, Ireland. Part 1: Records of species taken at light. Irish Biogeographical Society Bulletin 37: 58–82.

[B991] O’Connor JP (2019a) *Hydroptilavectis* Curtis, 1834, new to Ireland and the first Irish adult of *Tricholeiochitonfagesii* (Guinard, 1879) (Trichoptera: Hydroptilidae). Entomologist’s Monthly Magazine 155(3): 163–167. 10.31184/M00138908.1553.3987

[B992] O’Connor JP (2019b) *Oxyethiramirabilis* Morton (Trichoptera: Hydroptilidae), a caddisfly new to Ireland. British Journal of Entomology and Natural History 32: 231–234.

[B993] O’Connor JP (2020) Some further distribution records of caddisflies (Trichoptera) from Ireland. Bulletin - Irish Biogeographical Society 44: 138–166.

[B994] O’Connor JP Ashe P (1992) *Jabitrichiawellsae* sp. n. (Trichoptera, Hydroptilidae) from Tasek Bera, Malaysia. Aquatic Insects 14(4): 255–257. 10.1080/01650429209361491

[B995] O’Connor JP Bond KGM (2009) *Hydroptilasparsa* Curtis (Trichoptera: Hydroptilidae) new to Co. Tyrone. The Irish Naturalists’ Journal 29(2): 131.

[B996] O’Connor JP Bond KGM (2014) *Hydroptilaangulata* and *Oxyethiraflavicornis* (Trichoptera: Hydroptilidae), caddisflies new to Northern Ireland. British Journal of Entomology and Natural History 27(1): 24–25.

[B997] O’Connor JP Bond KGM (2018) *Hydroptilasimulans* Mosely, 1920 (Trich: Hydroptilidae), a caddisfly new to Northern Ireland. Entomologist’s Record and Journal of Variation 130: 193–194.

[B998] O’Connor JP O’Connor MA (1980) *Hydroptilaocculta* (Eaton) (Trichoptera: Hydroptilidae), a caddisfly new to Ireland with notes on *Hydroptilamartini* Marshall. Entomologist’s Record and Journal of Variation 92(7–8): 167–168.

[B999] O’Connor JP O’Connor M (2013) *Hydroptilavalesiaca* Schmid (Trich.: Hydroptilidae) new to Ireland with records of two other notable species. Entomologist’s Record and Journal of Variation 125: 189–193.

[B1000] O’Connor JP O’Connor MA (2014) Further records of caddisflies (Trichoptera) from Co. Fermanagh including seven species new to Northern Ireland. Irish Biogeographical Society Bulletin 38: 272–279.

[B1001] O’Connor JP O’Connor MA (2016) Some new distributional records for Irish caddisflies (Trichoptera) including a species list for Lough Neagh. Bulletin - Irish Biogeographical Society 40: 164–183.

[B1002] O’Connor JP O’Connor MA (2017a) *Agrayleasexmaculata* Curtis and *Leptocerustineiformis* Curtis: Caddisflies (Trichoptera) new to Northern Ireland. British Journal of Entomology and Natural History 30: 243–245.

[B1003] O’Connor JP O’Connor MA (2017b) Further distributional and flight-period records for Irish caddisflies (Trichoptera). Bulletin - Irish Biogeographical Society 41: 51–89.

[B1004] O’Connor JP O’Connor MA (2018) Records of Irish caddisflies (Trichoptera) including a county list. Bulletin - Irish Biogeographical Society 42: 75–154.

[B1005] O’Connor JP O’Connor MA (2019) *Hydroptilalotensis* Mosely, 1930, new to Ireland and a second Irish record of *Hydroptilavectis* Curtis, 1834 (Trichoptera, Hydroptilidae). Entomologist’s Monthly Magazine 155(4): 229–234. 10.31184/M00138908.1554.4006

[B1006] O’Connor JP O’Hanrahan BM (1988) *Agrayleasexmaculata* new to Ireland with notes on *Tricholeiochitonfagesii* and *Phacopteryxbrevipennis* (Trichoptera). The Irish Naturalists’ Journal 22: 478–480.

[B1007] O’Connor JP O’Connor MA McNaughton C (2018) *Holocentropusdubius* (Rambur, 1842) new to Northern Ireland with other caddisfly (Trichoptera) records from the region. Bulletin - Irish Biogeographical Society 42: 22–33.

[B1008] Obr S (1975) K poznani chrostiku (Trichoptera) Ceskoslovenska 3. Novi a malo znami chrostici severni Moravy. Casopis slersk. Mus. Opava 24(2): 127–136.

[B1009] Ohkawa A Ito T (2002) Redescription of *Scelotrichiaishiharai* Utsunomiya (Trichoptera, Hydroptilidae) with special reference to the biology of the immature stages. Nova Supplementa Entomologica (Proceedings of the 10^th^ International Symposium on Trichoptera) 15(2002): 449–458.

[B1010] Oláh J (1989) Thirty-five new hydroptilid species from Vietnam (Trichoptera, Hydroptilidae). Acta Zoologica Hungarica 35: 255–293.

[B1011] Oláh J (1994) Three new Trichoptera from the Kopet-Dagh and Karakoram Mountains. Folia Entomologica Hungarica 55: 281–286.

[B1012] Oláh J (2010) New species and new records of Palaearctic Trichoptera in the material of the Hungarian Natural History Museum. Annales Historico-Naturales Musei Nationalis Hungarici 102: 65–117.

[B1013] Oláh J (2012) New species and records of Trichoptera from Batanta and Waigeo Islands (Indonesia, Raja Empat Archipelago, Papua (Irian Jaya)). Braueria 39: 39–57.

[B1014] Oláh J (2013) On the Trichoptera of Batanta Island (Indonesia, West Papua, Raja Ampat Archipelago). Folia Entomologica Hungarica 74: 21–78. 10.17112/FoliaEntHung.2014.75.91

[B1015] Oláh J (2016) On the Trichoptera of Batanta Island (Indonesia, West Papua, Raja Ampat Archipeago) V. Folia Historico-Naturalia Musei Matraensis 40: 95–135.

[B1016] Oláh J (2017) Trichoptera endemic in the Carpathian Basin and the adjacent areas. Folia Entomologica Hungarica 78: 111–255. 10.17112/FoliaEntHung.2017.78.111

[B1017] Oláh J Beshkov S (2016) New records of Trichoptera in the Balkan Peninsula and Romania, with description of new *Rhyacophila* sibling species by speciation traits. Folia Entomologica Hungarica 77: 87–104. 10.17112/FoliaEntHung.2016.77.87

[B1018] Oláh J Flint Jr OS (2012) Description of new species in the Leucotrichiini tribe (Trichoptera: Hydroptilidae). Annales Historico-Naturales Musei Nationalis Hungarici 104: 131–213.

[B1019] Oláh J Ito T (2013) Synopsis of the *Oxyethiraflavicornis* species group with new Japanese *Oxyethira* species (Trichoptera, Hydroptilidae). Opuscula Zoologica 44(1): 23–46.

[B1020] Oláh J Johanson KA (2008) Reasoning an appendicular and functional caddisfly genital terminology. Braueria 35: 29–40.

[B1021] Oláh J Johanson KA (2010a) Description of 46 new Old World Hydroptilidae (Trichoptera). Folia Entomologica Hungarica 71: 1–91.

[B1022] Oláh J Johanson KA (2010b) Fifteen new Trichoptera (Insecta) species from Sumatra, Indonesia. Zootaxa 2618(1): 1–35. 10.11646/zootaxa.2618.1.1

[B1023] Oláh J Johanson KA (2011) New Neotropical Hydroptilidae (Trichoptera). Annales Historico-Naturales Musei Nationalis Hungarici 103: 117–255.

[B1024] Oláh J Kovács T (2018) On the Trichoptera of Batanta Island (Indonesia, Papua, Raja Ampat Archipelago) VI. Folia Historica-Naturalia Musei Matraensis 42: 163–195.

[B1025] Oláh J Vinçon G Kerimova I Kovács T Manko P (2020) On the Trichoptera of the Caucasus with western and eastern relatives. Opuscula Zoologica (Budapest) 51(S3): 03-174. 10.18348/opzool.2020.S3.3

[B1026] Özdikmen H (2007) A nomenclatural act: Replacement names for two homonymous caddisfly generic names (Trichoptera). Munis Entomology & Zoology 2: 443–444.

[B1027] Özdikmen H (2008) A nomenclatural act for caddis flies (Trichoptera). Munis Entomology & Zoology 3: 614–616.

[B1028] Palmer RW (1996) Invertebrates in the Orange River with emphasis on conservation and management. Southern African Journal of Aquatic Sciences 22(1–2): 3–51. 10.1080/10183469.1996.9631371

[B1029] Pan’kov NN Krasheninnikov AB (2016) Current state of knowledge of a faunal inventory of Trichoptera (Hexapoda, Trichoptera) from the Ural Mountains and neighboring regions. In: VshivkovaMorse JC (Eds)Proceedings of the 14th International Symposium on Trichoptera.Zoosymposia 10: 331–339. 10.11646/zoosymposia.10.1.31

[B1030] Paprocki H França D (2014) Brazilian Trichoptera Checklist II. Biodiversity Data Journal 2: e1557[, 1–109]. 10.3897/BDJ.2.e1557 PMC4206778 25349524

[B1031] Paprocki H Holzenthal RW Blahnik RJ (2004) Checklist of the Trichoptera (Insecta) of Brazil I. Biota Neotropica 4: 1–22. 10.1590/S1676-06032004000100008

[B1032] Park SJ Kong D (2020) A checklist of Trichoptera (Insecta) of the Korean Peninsula. Journal of Species Research 9(3): 288–323.

[B1033] Park SJ Ito T Nozaki T Kong D (2018) Six new records of Hydroptilidae (Trichoptera) from Korea. Animal Systematics, Evolution and Diversity 34(2): 101–109.

[B1034] Parker CR Voshell Jr JR (1979) A new species of *Hydroptila* from Virginia (Trichoptera: Hydroptilidae). Proceedings of the Entomological Society of Washington 81(1): 43–45.

[B1035] Parker CR Voshell Jr JR (1980) *Ochrotrichiagraysoni*, a new species of caddisfly from Virginia (Trichoptera: Hydroptilidae). Annals of the Entomological Society of America 73(4): 369–371. 10.1093/aesa/73.4.369

[B1036] Parker CR Voshell Jr JR (1981) A preliminary checklist of the caddisflies (Trichoptera) of Virginia. Journal of the Georgia Entomological Society 16: 1–7.

[B1037] Parys KA Harris SC (2013) Larva of *Nothotrichiashasta* Harris & Armitage (Trichoptera: Hydroptilidae) from California, USA, with its phylogenetic and taxonomic implications. Zootaxa 3620: 589–595. 10.11646/zootaxa.3620.4.8 26120728

[B1038] Peissner T Kappus B (1998) Zur Köcherfliegenfauna (Insecta, Trichoptera) der Jagst (Baden-Württemberg). Lauterbornia 34: 159–168.

[B1039] Peissner T Maier K-J Kappus B (1998) Erstnachweis von *Hydroptilalotensis* (Trichoptera) in Deutschland. Lauterbornia 34: 169–173.

[B1040] Pérez-Gelabert DE (2008) Arthropods of Hispaniola (Dominican Republic and Haiti): A checklist and bibliography. Zootaxa 1831(1): 1–530. 10.11646/zootaxa.1831.1.1 32230049

[B1041] Pes AMO Hamada N (2003) The occurrence of *Taraxitrichia* Flint & Harris, 1992 (Trichoptera: Hydroptilidae) in Brazil, with description of the final larval stage. Zootaxa 328(1): 1–7. 10.11646/zootaxa.328.1.1

[B1042] Pes AMO Hamada N (2004) *Ceratotrichia* Flint, 1992 (Trichoptera: Hydroptilidae) larval and pupal description and new genus records for Brazil. Entomotrópica 19: 31–37.

[B1043] Pescador ML Rasmussen AK Harris SC (2004) Identification manual for the caddisfly (Trichoptera) larvae of Florida. Revised Edition 2004. Tallahassee: State of Florida, Department of Environmental Protection, Division of Water Resource Management, 136 pp.

[B1044] Pictet FJ (1834) Recherches pour servir à l’histoire et l’anatomie des Phryganides. A. Cherbuliez, Geneva, 306 pp. 10.5962/bhl.title.8547

[B1045] Poinar Jr G Anderson NH (2005) Hymenoptera parasites of Trichoptera: the first fossil record. In: Tanida K Rossiter A (Eds) Proceedings of the 11th International Symposium on Trichoptera.Tokai University Press, Kanagawa, 343–346.

[B1046] Posada-García JA Roldán-Pérez G (2003) Clave ilustrada y diversidad de las larvas de Trichoptera en el nor-occidente de Colombia. Caldasia 25(1): 169–192.

[B1047] Potikha E Vshivkova T (2016) The caddisfly faunas (Insecta, Trichoptera) of Protected Natural Areas in southern Far East Russia. Zoosymposia 10(1): 357–383. 10.11646/zoosymposia.10.1.33

[B1048] Previšić A Mihaljević Z Kerovec M (2007) Caddisfly (Insecta: Trichoptera) fauna of altered and man-made habitats in the Drava River, NW Croatia. Natura Croatica 16(3): 181–187.

[B1049] Previšić A Dvorski P Cetinić K Ivković M (2013) New records for the Croatian caddisfly (Trichoptera, Insecta) fauna from the Plitvice Lakes National Park. Entomologia Croatica 17(1–4): 7–12.

[B1050] Prommi T-O Permkam S (2010) The caddisfly (Insecta, Trichoptera) of Ko Hong Hill nature preserve, southern Thailand. Denisia 29: 295–302.

[B1051] Promwong W Thapanya D (2019) Monthly diversity and abundance of caddisflies in upstream and downstream areas of the Mae Ngat Somboonchol Dam, Chiang Mai Province, Thailand. Zoosymposia 14: 068–080. 10.11646/zoosymposia.14.1.10

[B1052] Quinn JM Williamson RB Smith RK Vickers ML (1992) Effects of riparian grazing and channelisation on streams in Southland, New Zealand. 2. Benthic invertebrates. New Zealand Journal of Marine and Freshwater Research 26(2): 259–273. 10.1080/00288330.1992.9516520

[B1053] Quinn JM Boothroyd IKG Smith BJ (2004) Riparian buffers mitigate effects of pine plantation logging on New Zealand streams. 2. Invertebrate communities. Forest Ecology and Management 191(1–3): 129–146. 10.1016/j.foreco.2003.11.013

[B1054] Racięcka M (1936) Neue und seltenere Trichopterenarten der Umgegend von Wilno. Travaux de la Société des Sciences et des Lettres de Wilno 11: 97–102.

[B1055] Racięcka M (1937) Nowy gatunek chróścika z rodziny Hydroptilidae. Eine neue Trichopterenart aus der Familie Hydroptilidae. Annales Musei Zoologici Polonici 11: 477–480.

[B1056] Razo-González M (2018) Caddisflies (Insecta: Trichoptera) from Santa Catarina Lachatao, Oaxaca, México: New species, new geographical records, and checklist. Zootaxa 4388(1): 022–040. 10.11646/zootaxa.4388.1.2 29690462

[B1057] Razo-González M Castaño-Meneses G Novelo-Gutiérrez R Márquez J (2020) Preliminary evaluation of the nocturnal flight of caddisflies (Insecta: Trichoptera) in a temperate forest in Oaxaca, Mexico. Aquatic Insects 41(4): 1–17. 10.1080/01650424.2020.1797818

[B1058] Resh VH Houp RE (1986) Life history of the caddisfly *Dibusaangata* and its association with the red alga *Lemaneaaustralis*. Journal of the North American Benthological Society 5: 28–40. 10.2307/1467745

[B1059] Resh VH Sorg KL (1978) Midsummer flight activity of caddisfly adults from a northern California stream. Environmental Entomology 7(3): 396–398. 10.1093/ee/7.3.396

[B1060] Resh VH White DS White SJ (1978) Lake Texoma caddisflies (Insecta: Trichoptera): 1. species present and faunal changes since impoundment. The Southwestern Naturalist 23(3): 381–388. 10.2307/3670245

[B1061] Reusch H (1986) Erstnachweis von *Orthotrichiatragetti* Mosely 1930 in Deutschland (Trichoptera: Hydroptilidae). Entomologische Zeitschrift 96(10): 139–141.

[B1062] Reuter (1890) [Title unknown]. Revue d’Entomologie Français 9: 291.

[B1063] Ribeiro JMF Magalhaes C Rafael JA Henriques AL (2009) Catalogue of type specimens of the Collection of Invertebrates of Instituto Nacional de Pesquisas da Amazonia, Manaus, Brazil. III. Hexapoda: Isoptera, Mantodea, Mecoptera, Orthoptera, Plecoptera, Trichoptera and Zoraptera. Revista Brasileira de Entomologia 53(1): 32–35. 10.1590/S0085-56262009000100008

[B1064] Richters F (1902) Beiträge zur Kenntnis der Fauna der Umgebung von Frankfurt a M. VIII. Die Larve von *Ithytrichialamellaris* Eat. Bericht der Senckenbergischen Naturforschenden Gesellschaft Frankfurt am Main 1902: 19–21.

[B1065] Rinne A Wiberg-Larsen P (2017) Trichoptera larvae of Finland: a key to the caddis larvae of Finland and nearby countries. Trificon, Tampere, 151 pp.

[B1066] Ríos-Touma B Holzenthal RW Huisman J Thomson R Ràzuri-Gonzales E (2017) Diversity and distribution of the Caddisflies (Insecta: Trichoptera) of Ecuador. PeerJ 5: e2851. 10.7717/peerj.2851 PMC5237369 28097062

[B1067] Ris F (1894) Vier Schweizerische Hydroptiliden. Mitteilungen der Schweizerische Entomologische Gesellschaft 9: 131–134.

[B1068] Ris F (1895) Neue Phryganiden der Schweizerischen Fauna. Mitteilungen der Schweizerische Entomologische Gesellschaft 9: 227.

[B1069] Ris F (1897) Neuropterologischer Sammelbericht 1894–96. Mitteilungen der Schweizerische Entomologische Gesellschaft 9: 415–442.

[B1070] Ris F (1903) Trichopteren des Kantons Tessin und angrenzender Gebiete. Mitteilungen der Schweizerische Entomologische Gesellschaft 11: 5–18.

[B1071] Robert B (2007) Systematisches Verzeichnis der Köcherfliegen (Insecta: Trichoptera) Deutschlands. Fortschreibung 08/2007. Lauterbornia 61: 79–99.

[B1072] Robinson J (2009) The Trichoptera of St Kilda, including a new family record based on *Oxythira* [*Oxyethira*] *falcata* Morton, 1893, and reflections on the dispersal distances of U.K. caddis. Entomologist’s Gazette 60(2): 117–125.

[B1073] Roble SM Flint Jr OS Harris SC (2019) New Virginia Records of Trichoptera and Neuroptera (Insects). Banisteria 52: 42–45.

[B1074] Rocha IC Dumas LL de Souza WRM (2018) Two new species and updated checklist of *Oxyethira* Eaton, 1873 (Trichoptera, Hydroptilidae) from Brazil. Anais da Academia Brasileira de Ciências 90(1): 147–154. 10.1590/0001-3765201720170252 29044322

[B1075] Rojas-Ascencio A Bueno-Soria J Gaviño-Rojas R (2002) Trichoptera from Arroyo Colorado, Municipality of Temascaltepec, State of Mexico, Mexico. Nova Supplementa Entomologica (Proceedings of the 10^th^ International Symposium on Trichoptera) 15: 375–378.

[B1076] Rojas-Camousseight F Tachet H (1988 [1990]) Les femelles d’*Hydroptila* du groupe *sparsa* (Trichoptera, Hydroptilidae). Rivista di Idrobiologia 27(2–3): 309–316.

[B1077] Roldán-Perez G (1988) Guia para el Estudio de los Macroinvertebrados Acuáticos del Departamento de Antioquia. Antioquia, Colombia: Universidad de Antioquia, 217 pp.

[B1078] Ross HH (1938a) Descriptions of Nearctic caddis flies (Trichoptera) with special reference to the Illinois species. Bulletin - Illinois Natural History Survey 21(1–8): 101–183. 10.21900/j.inhs.v21.261

[B1079] Ross HH (1938b) Lectotypes of North American caddisflies in the Museum of Comparative Zoology. Psyche (Cambridge, Massachusetts) 45(1): 1–61. 10.1155/1938/25928

[B1080] Ross HH (1939) New species of Trichoptera from the Appalachian region. Proceedings of the Entomological Society of Washington 41: 63–72.

[B1081] Ross HH (1941a) Descriptions and records of North American Trichoptera. Transactions of the American Entomological Society 67: 35–126.

[B1082] Ross HH (1941b) New species of Trichoptera from Canada and northern United States. Canadian Entomologist 73(1): 15–19. 10.4039/Ent7315-1

[B1083] Ross HH (1944) The caddisflies, or Trichoptera, of Illinois. Bulletin - Illinois Natural History Survey 23(1–5): 1–326. 10.21900/j.inhs.v23.199

[B1084] Ross HH (1947) Descriptions and records of North American Trichoptera, with synoptic notes. Transactions of the American Entomological Society 73: 125–168.

[B1085] Ross HH (1948) Notes and descriptions of Nearctic Hydroptilidae (Trichoptera). Journal of the Washington Academy of Sciences 38: 201–206.

[B1086] Ross HH (1956) Evolution and Classification of the Mountain Caddisflies. University of Illinois Press, Urbana, [vii +] 213 pp.

[B1087] Ross HH (1967) The evolution and past dispersal of the Trichoptera. Annual Review of Entomology 12(1): 169–206. 10.1146/annurev.en.12.010167.001125

[B1088] Ross LR Murkin HC (1993) The effect of above-normal flooding of a northern prairie marsh on *Agrayleamultipunctata* Curtis (Trichoptera: Hydroptilidae). Journal of Freshwater Ecology 8(1): 27–35. 10.1080/02705060.1993.9664721

[B1089] Ross HH Spencer GJ (1952) A preliminary list of the Trichoptera of British Columbia. Proceedings of the Entomological Society of British Columbia 48: 43–51.

[B1090] Roy D Harper PP (1975) Nouvelles mentions de trichoptères du Québec et description de *Limnephilusnimmoi* sp. nov. (Limnephilidae). Canadian Journal of Zoology 55: 1080–1088. 10.1139/z75-125

[B1091] Roy D Harper PP (1979) Liste préliminaire des trichoptères (insectes) de Québec. Annales de la Société Entomologique de Québec 24: 148–172.

[B1092] Roy D Harper PP (1980) *Oxyethiraroberti* n. sp., Trichoptére nouveau du sud du Québec (Hydroptilidés). Naturaliste Canadien 107(2): 117–119.

[B1093] Roy D Harper PP (1981) An analysis of an adult Trichoptera community in the Laurentian highlands of Quebec. Holarctic Ecology 4(2): 102–115. 10.1111/j.1600-0587.1981.tb00986.x

[B1094] Rueda Martín PA (2006) New record of the genus *Ithytrichia* (Trichoptera: Hydroptilidae) for South America, with descriptions of male, larva and pupa of a new species from northwestern Argentina. Aquatic Insects 28(4): 251–256. 10.1080/01650420601072276

[B1095] Rueda Martín PA (2011) New Hydroptilidae and new records from north-western Argentina and Bolivia (Trichoptera: Hydroptilidae). Aquatic Insects 33(1): 1–11. 10.1080/01650424.2011.577279

[B1096] Ruiter DE (1990) A new species of *Neotrichia* (Trichoptera: Hydroptilidae) from Colorado with additions and corrections to the distributions and records of Colorado Trichoptera. Entomological News 101: 88–92.

[B1097] Ruiter DE (1999) A new species and new synonym in the genus *Psychoronia* (Limnephilidae), with significant records for caddisflies (Trichoptera) from western North America. The Great Basin Naturalist 59: 160–168. 10.5962/bhl.part.15820

[B1098] Ruiter DE (2007) Two new species of *Neotrichia* from Arizona, U.S.A. (Trichoptera: Hydroptilidae). In: Bueno-Soria J Barba-Álvarez R Armitage BJ (Eds) Proceedings of the 12th International Symposium on Trichoptera.The Caddis Press, Columbus, Ohio, 275–277.

[B1099] Ruiter DE (2011) Two new species of *Ochrotrichia* (Trichoptera: Hydroptilidae) from the southwestern United States. Zoosymposia 5(1): 420–424. 10.11646/zoosymposia.5.1.35

[B1100] Ruiter DE Harris SC (2015) New *Ochrotrichia* Mosely, 1934 (Trichoptera: Hydroptilidae) from Western North America. The Pan-Pacific Entomologist 91(4): 318–336. 10.3956/2015-91.4.318

[B1101] Ruiter DE Boyle EE Zhou X (2013) DNA barcoding facilitates associations and diagnoses for Trichoptera larvae of the Churchill (Manitoba, Canada) area. BMC Ecology 13(1): 1–39. 10.1186/1472-6785-13-5 23425021 PMC3691766

[B1102] Ruiz-García A (1995) Primera cita de *Ithytrichiadovporiana* Botosaneanu, 1980 (Trichoptera: Hydroptilidae) en la Peninsula Iberica. Boletin de la Asociacion Espanola de Entomologia 19: 203.

[B1103] Ruiz-García A Herrera Grao AF Ferreras-Romero M (2006) Distribution of Trichoptera communities in the Hozgarganta catchment (Los Alcornocales Natural Park, SW Spain). International Review of Hydrobiology 91(1): 71–85. 10.1002/iroh.200510822 32336939 PMC7165828

[B1104] Ruiz-García A Sáinz-Bariáin M Zamora-Muñoz C (2016) Contribución al conocimiento de los tricópteros (Insecta: Trichoptera) de Andalucía. Graellsia 72(2): 1–24. 10.3989/graellsia.2016.v72.162

[B1105] Sáinz-Bariáin M Zamora-Muñoz C Soler JJ Bonada N Sáinz-Cantero CE Alba-Tercedor J (2016) Changes in Mediterranean high mountain Trichoptera communities after a 20-year period. Aquatic Sciences 78(4): 669–682. 10.1007/s00027-015-0457-9

[B1106] Salokannel J Mattila K (2018) Suomen vesiperhoset. Trichoptera of Finland. Hyönteistarvike Tibiale Oy, Helsinki, 448 pp.

[B1107] Salokannel J Wahlberg N Vesterinen EJ Martinez J González M (2012) A taxonomic study of the caddisfly *Oxyethirafalcata* Morton, 1893 (Trichoptera: Hydroptilidae) using genital morphology and DNA barcoding. Entomologica Fennica 23(4): 199–205. 10.33338/ef.7386

[B1108] Sanabria MJ Tempelman D (2016) Venrode: Een mooi ven met een grote populatie van de schietmot *Tricholeiochitonfagesii*. De Digitale Kokerjuffer 12(19): 14–17.

[B1109] Santos APM (2011) Four new species of the microcaddisfly genus *Alisotrichia* Flint (Trichoptera: Hydroptilidae) from southeastern Brazil. Zootaxa 29(1): 59–68. 10.11646/zootaxa.3112.1.4

[B1110] Santos APM (2020) A review of the Neotropical microcaddisfly genus *Acostatrichia* Mosey, 1939 with description of a new species from Brazil (Trichoptera: Hydroptilidae: Leucotrichiinae). Zootaxa 4755(2): 201–230. 10.11646/zootaxa.4755.2.1 32230179

[B1111] Santos APM Nessimian JL (2009a) New species and records of *Neotrichia* (Trichoptera: Hydroptilidae) from central Amazonia, Brazil. Zoologia 26(4): 758–768. 10.1590/S1984-46702009000400022

[B1112] Santos APM Nessimian JL (2009b) A new species of microcaddisfly genus *Flintiella* Angrisano (Trichoptera: Hydroptilidae) from Amazonas State, Brazil. Zootaxa 2004: 65–68. 10.11646/zootaxa.2004.1.6

[B1113] Santos APM Nessimian JL (2010a) Description of a new species of *Byrsopteryx* (Trichoptera: Hydroptilidae) from Rio de Janeiro State, Brazil, including its immature stages. Zootaxa 2668(1): 44–54. 10.11646/zootaxa.2668.1.4

[B1114] Santos APM Nessimian JL (2010b) The occurrence of the microcaddisfly *Costatrichia* (Trichoptera: Hydroptilidae: Hydroptilinae) in Brazil with description of two new species. Zoologia 27(5): 837–843. 10.1590/S1984-46702010000500022

[B1115] Santos APM Henriques-Oliveira AL Nessimian JL (2009) New species and records of *Oxyethira* Eaton (Trichoptera: Hydroptilidae) from Amazonas State, Brazil. Zootaxa 2169(1): 35–44. 10.11646/zootaxa.2169.1.3

[B1116] Santos APM Jardim GA Nessimian JL (2011) Three new species of microcaddisflies (Trichoptera: Hydroptilidae) from Brazil. Zoologia 28: 812–818. 10.1590/S1984-46702011000600015

[B1117] Santos APM Nessimian JL Takiya DM (2016a) Revised classification and evolution of leucotrichiine microcaddisflies (Trichoptera: Hydroptilidae) based on morphological and molecular data. Systematic Entomology 41(2): 458–480. 10.1111/syen.12168

[B1118] Santos APM Takiya DM Nessimian JL (2013) Two new *Costatrichia* (Trichoptera: Hydroptilidae: Leucotrichiinae). Zoologia 30(4): 447–450. 10.1590/S1984-46702013000400012

[B1119] Santos APM Takiya DM Nessimian Jorge JL (2016b) Integrative taxonomy of *Metrichia* Ross (Trichoptera: Hydroptilidae: Ochrotrichiinae) microcaddisflies from Brazil: descriptions of twenty new species. PeerJ 4: 1–54. 10.7717/peerj.2009 PMC4860326 27169001

[B1120] Satake K Kuranishi RB (2007) Further studies on caddisflies (Insecta: Trichoptera) collected from the Bonin Islands and Izu Archipelago, Japan. In: Bueno-Soria J Barba-Álvarez R Armitage BJ (Eds) Proceedings of the 12th International Symposium on Trichoptera.The Caddis Press, Columbus, Ohio, 279–284.

[B1121] Sattler W Sykora JL (1977) Über eine, besonders durch ihr Bauinstinkt, merkwürdige neotropische Köcherfliege - *Leucotrichiabrasiliana* n. sp. Amazoniana 6: 137–255.

[B1122] Scheibler EE Debandi GO (2008) Spatial and temporal patterns in the aquatic insect community of a high altitude Andean stream (Mendoza, Argentina). Aquatic Insects 30(2): 145–161. 10.1080/01650420701880974

[B1123] Schiess-Bühler H Rezbanyai-Reser L (2006) Zur Insektenfauna vom Hanenriet bei Giswil, 470m, Kanton Obwalden. V. Trichoptera (Kücherfliegen). Entomologische Berichte (Luzern) 56: 65–82.

[B1124] Schmid F (1947) Sur quelques Trichoptères suisses nouveaux ou peu connus. Mitteilungen der Schweizerische Entomologische Gesellschaft 20: 519–535.

[B1125] Schmid F (1951) Quelques nouveaus trichoptères néartiques. Bulletin de l’Institut Royal des Sciences Naturelles de Belgique 27: 1–16.

[B1126] Schmid F (1952) Contribution à l’étude des Trichoptères d’Espagne. Pirineos 26: 627–695.

[B1127] Schmid F (1958a) Contribution à l’étude des Trichoptères néotropicaux III. Mitteilungen aus dem Zoologischen Museum in Berlin 34(1): 183–217. 10.1002/mmnz.19580340110

[B1128] Schmid F (1958b) Trichoptères de Ceylan. Archiv für Hydrobiologie 54: 1–173.

[B1129] Schmid F (1958c) Trichoptères du Pakistan. Tijdschrift voor Entomologie 101: 181–221.

[B1130] Schmid F (1959a) Le genre *Stactobia* Mch. Miscelánea Zoológica, Barcelona 1: 1–56.

[B1131] Schmid F (1959b) Trichoptères d’Iran (Trichoptera). Beiträge zur Entomologie 9: 199–219; 199(193–194): 376–412; 199(195–196): 682–698; 199(197–198): 760–799.

[B1132] Schmid F (1960) Trichopteres du Pakistan. III (Hydroptilidae, Philopotamidae, Polycentropodidae). Tijdschrift voor Entomologie 103: 83–109.

[B1133] Schmid F (1970) 210. Trichoptera III. Ergebnisse der zoologischen Forschungen von Dr. Z. Kaszab in der Mongolei. Reichenbachia 13(9): 114–124.

[B1134] Schmid F (1983) Encore quelques *Stactobia* McLachlan (Trichoptera, Hydroptilidae). Naturaliste Canadien 110: 239–283.

[B1135] Schmid F (1990 [1991]) Quelques nouveaux trichoptères indiens (Trichoptera). Naturaliste Canadien 117: 239–251.

[B1136] Schmidt-Brücken R (1996) Erstnachweis von *Hydroptilatigurina* (Trichoptera) fur Deutschland. Lauterbornia 26: 85.

[B1137] Schneider WG (1845) Verzeichnis der von Herrn Oberlehrer Zeller in Jahre 1844 in Sicilien und Italien gesammelten Neuropteren mit Beschreibung einiger neuen Arten. Stettiner Entomologische Zeitung 6: 338, 346.

[B1138] Schrankel I Neu P Dohet A Schoos F (2008) Checklist of the Trichoptera of the Grand Duchy of Luxembourg - first revision. Ferrantia 55: 89–92.

[B1139] Scott KMF (1963) Some new African caddisflies (Trichoptera) from the Western Cape Province, IV. Some Hydroptilidae. Annals of the South African Museum 46(19): 469–478.

[B1140] Scott KMF (1976) The larval and pupal stages of *Ugandatrichia* Mosely (Trichoptera: Hydroptilidae) from Rhodesia, with the description of a new species. Annals of the Cape Provincial Museums 11: 117–127. [Natural History]

[B1141] Scudder GGE (1971) Comparative morphology of insect genitalia. Annual Review of Entomology 16(1): 379–406. 10.1146/annurev.en.16.010171.002115

[B1142] Sekhi S Haouchine S Lounaci-Daoudi D Moutaouakil MEAE Lounaci A (2016) Contribution à la connaissance des Trichoptères de Grande-Kabylie (Algérie). Ephemera 17(1): 51–69. [Trichoptera]

[B1143] Šemnički P Previšić A Ivković M Čmrlec K Mihaljević Z (2011) Emergence of caddisflies (Trichoptera, Insecta) at Tufa barriers in Plitvice Lakes National Park. Entomologia Croatica 15(1–4): 145–161.

[B1144] Serafin E (2003) *Orthotrichiatragetti* Mosely, 1930 (Trichoptera: Hydroptilidae) - a microcaddisfly species new for the fauna of Poland. Polskie Pismo Entomologiczne 72(4): 319–321.

[B1145] Sheath RG Müller KM Larson DJ Cole KM (1995) Incorporation of freshwater Rhodophyta into the cases of caddisflies (Trichoptera) from North America. Journal of Phycology 31(6): 889–896. 10.1111/j.0022-3646.1995.00889.x

[B1146] Sibley CK (1926) A preliminary biological survey of the Lloyd-Cornell Reservation: Studies on Trichoptera. Bulletin of the Lloyd Library 27: 102–108; 185–247, 186–221, 236–247.

[B1147] Šidagytė E Višinskienė G Arbačiauskas K (2016) *Tricholeiochitonfagesii* (Guinard, 1879) - a new caddisfly (Trichoptera, Hydroptilidae) species to Lithuanian fauna. New and Rare for Lithuania Insect Species Records and Descriptions 28: 79–82.

[B1148] Siltala (Silfvenius) AJ (1908) Zur Trichopteren-Fauna der Nördlichen Fenno-Skandia. Acta Societatis pro Fauna et Flora Fennica 31: 1–19.

[B1149] Sinclair BJ (1990) The madicolous fauna of southern Ontario, with emphasis on the Niagara Escarpment. In: Allen GM Eagles PFJ Price SD (Eds) Conserving Carolinian Canada.Conservation biology in the deciduous forest region. University of Waterloo Press, Waterloo, Ontario, 281–288.

[B1150] Sinclair BJ Marshall SA (1986 [1987]) The madicolous fauna in southern Ontario. Proceedings of the Entomological Society of Ontario 117: 9–14.

[B1151] Sipahiler F (1989) Seven new species and a new subspecies of Trichoptera from South Western Anatolia. Aquatic Insects 11(3): 129–140. 10.1080/01650428909361360

[B1152] Sipahiler F (1994) Three new species of Trichoptera from southern Anatolia. Braueria 21: 12–14.

[B1153] Sipahiler F (1996) New species and subspecies of Trichoptera from Turkey (Glossosomatidae; Hydroptilidae; Limnephilidae). Braueria 23: 29–31.

[B1154] Sipahiler F (1997) New species of caddisflies from Turkey (Trichoptera: Rhyacophilidae, Hydroptilidae, Beraeidae). Braueria 24: 15–17.

[B1155] Sipahiler F (1998) New species of Hydroptilidae, Hydropsychidae and Beraeidae, and new records of Trichoptera from Turkey. Braueria 25: 9–11.

[B1156] Sipahiler F (2000) New species and subspecies of Trichoptera from Turkey (Hydroptilidae, Philopotamidae, Phryganeidae, Lepidostomatidae). Braueria 27: 26–28.

[B1157] Sipahiler F (2003a) A new species of the *occulta*-Group of the genus *Hydroptila* Dalman, 1819 from Turkey (Trichoptera, Hydroptilidae). Aquatic Insects 25(1): 19–22. 10.1076/aqin.25.1.19.14024

[B1158] Sipahiler F (2003b) The Trichoptera fauna of the Lakes District in southern Turkey, with the description of a new species (Hydroptilidae). Braueria 30: 31–34.

[B1159] Sipahiler F (2005) A checklist of the caddisflies of Turkey (Trichoptera). In: Tanida K Rossiter A (Eds) Proceedings of the 11th International Symposium on Trichoptera.Kanagawa, Tokai University Press, 393–405.

[B1160] Sipahiler F (2007) The Trichoptera fauna of north-western Turkey with the descriptions of a new species and of some previously unknown females (Philopotamidae, Sericostomatidae). Braueria 34: 36–42.

[B1161] Sipahiler F (2008) Zoogeographical characteristics of the Trichoptera fauna of Turkey. Ferrantia 55: 93–109.

[B1162] Sipahiler F (2012a) Five new species of Trichoptera with the faunistic list of Sinop and Ordu provinces in Turkey (Glossosomatidae, Philopotamidae, Hydropsychidae, Sericostomatidae). Munis Entomology & Zoology 7(1): 1–17.

[B1163] Sipahiler F (2012b) Four new species of Hydroptilidae from Turkey (Trichoptera). Munis Entomology & Zoology 7(2): 1051–1057.

[B1164] Sipahiler F (2016) Faunistic studies on the Trichoptera fauna of northwestern Turkey and Thrace. Braueria 43: 11–16.

[B1165] Sipahiler F (2017a) Malformation of the male genitalia of *Ptilocolepuscolchicus* Martynov, 1923 found in Turkey (Trichoptera, Ptilocolepidae). Braueria 44: 10.

[B1166] Sipahiler F (2017b) A new species of *Apatania* Kolenati and the faunistic list for Köprüçay River and the surrounding area from southern Turkey (Trichoptera, Apataniidae). Braueria 44: 11–16.

[B1167] Sipahiler F (2018) Three new species of caddisflies (Trichoptera: Hydroptilidae, Leptoceridae) from Turkey and faunistic list for the Seyhan and Ceyhan rivers. Nova Acta Científica Compostelana (Bioloxía) 25: 37–43.

[B1168] Sipahiler F Malicky H (1987) Die Köcherfliegen der Türkei (Trichoptera). Part 1. Entomofauna 8(7): 77–168.

[B1169] Skuja A (2011) Microhabitat preference of caddisfly (Trichoptera) communities in a medium-sized lowland stream in Latvia. Zoosymposia 5: 425–433. 10.11646/zoosymposia.5.1.36

[B1170] Smirnova D Kushnikova L Evseeva A Grishaeva O Kraynyuk V Pilin D Sklyarova O Epova J Baymukanova Z Timirkhanov S (2016) The Trichoptera of Kazakhstan: a review. In: Vshivkova TV Morse JC (Eds) Proceedings of the 14th International Symposium on Trichoptera.Zoosymposia 10: 398–408. 10.11646/zoosymposia.10.1.36

[B1171] Smirnova D Ivanov VD Melnitsky SI Sklyarova O (2020) Trichoptera of Saur and Tarbagatay Mountains (Kazakhstan). Zoosymposia 18: 063–071. 10.11646/zoosymposia.18.1.10

[B1172] Smith BJ (2008) Two new species of caddisflies (Trichoptera) from New Zealand. Aquatic Insects 30(1): 43–50. 10.1080/01650420701687155

[B1173] Solem JO (1970a) Trichoptera from South Varanger, North Norway. Rhizocrinus, Occasional Papers of the Zoological Museum, University of Oslo 1(4): 1–8.

[B1174] Solem JO (1970b) Trichoptera new to Norway. Norsk Entomologisk Tidsskrift 17: 93–95.

[B1175] Solem JO (1972) The larvae of *Agrayleacognatella* McLachlan (Trichoptera, Hydroptilidae). Norsk Entomologisk Tidsskrift 19: 77–79.

[B1176] Solem JO Gullefors B (1996) Trichoptera, caddisflies. Aquatic insects of North Europe. A Taxonomic handbook. Volume 1. Ephemeroptera - Plecoptera - Heteroptera - Neuroptera - Megaloptera - Coleoptera - Trichoptera - Lepidoptera. A. Nilsson. Apollo Books, Stenstrup, 223–255.

[B1177] Spandl H (1923) Beobachtungen an Larven von *Phryganeagrandis* L. und *Oxyethiracostalis* Curt. Zeitschrift für Wissenschaftliche Insektenbiologie 18: 357–358.

[B1178] Spinelli G Corallini C (2002) Morphology of first pair of legs of Italian Trichoptera larvae: a comparative SEM study. Nova Supplementa Entomologica (Proceedings of the 10^th^ International Symposium on Trichoptera) 15: 29–36.

[B1179] Spuris Z (1962) Contribution on the fauna of caddis-flies of lakes of the Latvian SSR. Latvijas Entomologs 6: 55–75.

[B1180] Spuris Z (1964) Die Köcherfiiegen der Seen der Nordlettland. Latvijas Entomologs 8: 3–24.

[B1181] Spuris Z (1972) Materialen für die Fauna der Köcherfliegen Estlands]. Uchenye Zapiski Tartuskogo Gosudarstvennogo Universiteta 6: 18–39.

[B1182] Spuris Z (1989) Synopsis of the fauna of the Trichoptera of the USSR. Latvijas Entomologs (Supplement 4): 1–84.

[B1183] Stanić-Koštroman S Previšić A Planinić A Kucinić M Škobić D Dedić A Durbešić P (2015) Environmental determinants of contrasting caddisfly (Insecta, Trichoptera) biodiversity in the Neretva and Bosna river basins (Bosnia and Herzegovina) under temperate and mediterranean climates. International Review of Hydrobiology 100(2): 79–95. 10.1002/iroh.201301631

[B1184] Statzner B (1977) Taxonomische Studien an der Hydroptilidae-Imagines aus dem zentralafrikanishen Bergbach Kalengo. Deutsche Entomologische Zeitschrift für Natur Forschung 25: 393–405. 10.1002/mmnd.4800240413

[B1185] Stephens JF (1836–1937) Illustrations of British Entomology; or a Synopsis of Indigenous Insects: Containing their Generic and Specific Distinctions; with an Account of their Metamorphoses, Times of Appearance, Localities, Food, and Economy, as far as Practicable. Mandibulata. Vol. VI. Baldwin and Cradock, London, 240 pp.

[B1186] Steven JC Hilsenhoff WL (1984) The caddisflies (Trichoptera) of Otter Creek, Wisconsin. Transactions of the Wisconsin Academy of Sciences, Arts, and Letters 72: 157–172.

[B1187] Stewart TW Miner JG Lowe RL (1998) Macroinvertebrate communities on hard substrates in Western Lake Erie: Structure effects of *Dreissena*. Journal of Great Lakes Research 24(4): 868–879. 10.1016/S0380-1330(98)70868-8

[B1188] Stojanović K Živić I Žnidaršić TK Živić M Žunić M Simić V Marković Z (2015) *Ithytrichia* Eaton, 1873 (Hydroptilidae: Trichoptera): A genus new for the entomofauna of Serbia. Entomological News 125(1): 52–62. 10.3157/021.125.0111

[B1189] Stroot P (1989) The variability of larval coloration patterns of *Agrayleamultipunctata* in a population from Belgium (Trichoptera: Hydroptilidae). Entomologische Berichten 49(10): 157–160.

[B1190] Sukatsheva ID Vassilenko DV (2016) Mesozoic Trichoptera with reduced venation. In: Vshivkova TS Morse JC (Eds) Proceedings of the 14th International Symposium on Trichoptera.Zoosymposia 10: 409–412. 10.11646/zoosymposia.10.1.37

[B1191] Svensson BW Tjeder B (1975) *Oxyethiraboreella* n. sp. from northern Sweden (Trichoptera: Hydroptilidae). Entomologica Scandinavica 6(2): 131–133. 10.1163/187631275X00217

[B1192] Sweeney P (2006) New records of three rare Irish caddis larvae (Trichoptera). The Irish Naturalists’ Journal 28: 300–301.

[B1193] Swegman BG Ferrington Jr LC (1980) New records of western Trichoptera with notes on their biology. The Great Basin Naturalist 40: 287–291.

[B1194] Swegman BG Walker W Sykora JL (1981) The adult Trichoptera of Linesville Creek, Crawford County, Pennsylvania with notes on their flight activity. Transactions of the American Entomological Society 107: 125–147.

[B1195] Sykora JL (1967) Trichoptera collected by prof. J. Illies in New Guinea and New Caledonia. Pacific Insects 9(4): 585–595.

[B1196] Sykora JL Harris SC (1994) Five new species of *Hydroptila* from eastern United States (Insecta: Trichoptera: Hydroptilidae). Annals of the Carnegie Museum 63(1): 67–75. 10.5962/p.215811

[B1197] Sykora JL Weaver JS III (1978) Three new species of Trichoptera from western Pennsylvania. Annals of the Carnegie Museum 47: 1–12. 10.5962/p.215818

[B1198] Szczęsny B (1975) Caddis-flies (Trichoptera) of the River Raba. Acta Hydrobiologica (Kraków) 17: 35–51.

[B1199] Szczesny B Godunko R (2008) Checklist of Ukrainian Trichoptera. Braueria 35: 11–20.

[B1200] Tanida K Kuranishi R (2016) Order Trichoptera. Catalogue of the Insects of Japan. Volume 5: Neuropterida, Mecoptera, Siphonaptera, Trichoptera and Strepsiptera. Entomological Society of Japan 5: 62–138.

[B1201] Tanida K Nozaki T Ito T Hattori T (2005) Trichoptera. In: Kawai T Tanida T (Eds) Aquatic insects of Japan: manual with keys and illustrations.Tokai University Press, Hadano, Kanagawa, 393–572.

[B1202] Tarter DC (1990) A checklist of the caddisflies (Trichoptera) from West Virginia. Entomological News 101: 236–245.

[B1203] Tempelman D Sanabria M (2013a) Schietmotten aan de Beekloop en de Maaij bij Bergeijk (Brabant), met nieuwe vondsten van *Oxyethirafalcata*. De Digitale Kokerjuffer 9(15): 18–21.

[B1204] Tempelman D Sanabria MJ (2013b) Trichoptera of Bra.s.l.av Lakes in 2013 with *Hydroptilapulchricornis* Pictet, 1834 (Hydroptilidae) and *Ceracleariparia* (Albarda, 1874) (Leptoceridae) as species new for the Belarus fauna. Lauterbornia 75: 143–146.

[B1205] Tempelman D Sanabria MJ Kruijt D (2013) Schietmotten in de Meinweg. Natuurhistorisch Maandblad 102(10): 286–291.

[B1206] Thani I Chantaramongkol P (1999) Life history of *Ugandatrichiamaliwan* (Trichoptera: Hydroptilidae) in Mae Klang stream, Doi Inthanon range, northern Thailand. In: Malicky H Chantaramongkol P (Eds) Proceedings of the 9th International Symposium on Trichoptera.Faculty of Science, Chiang Mai University, Chiang Mai, Thailand, 411–413.

[B1207] Thienemann A (1904a) *Ptilocolepusgranulatus* Pict., eine Uebergangsform von den Rhyacophiliden zu den Hydroptiliden. Allgemeine Zeitschrift für Entomologie 9: 418–424, 437–441.

[B1208] Thienemann A (1904b) Zur Trichopteren-Fauna von Tirol. Allgemeine Zeitschrift für Entomologie 9: 209–215, 257–262.

[B1209] Thienemann A (1905) Trichopterenstudien I–III (II. *Rhyacopsychehageni* Fr. Müller). Zeitschrift für Wissenschaftliche Insektenbiologie 1: 285–291.

[B1210] Thomas JA Frandsen PB Prendini E Zhou X Holzenthal RW (2020) A multigene phylogeny and timeline for Trichoptera (Insecta). Systematic Entomology 45(3): 670–686. 10.1111/syen.12422

[B1211] Thomson RE (2012) Descriptions of new species of Leucotrichiinae (Trichoptera: Hydroptilidae) from Brazil. Psyche 2012: 1–7. 10.1155/2012/916718

[B1212] Thomson RE (2019) A revision of the Neotropical caddisfly genus *Ascotrichia* Flint, 1983 (Trichoptera, Hydroptilidae). PeerJ 7: 1–22. 10.7717/peerj.7560 PMC6707345 31497406

[B1213] Thomson RE Armitage BJ (2018) The Trichoptera of Panama. VI. Seven new species of microcaddisflies (Insecta: Trichoptera: Hydroptilidae) from Mount Totumas Cloud Forest and Biological Reserve. Insecta Mundi 0613: 1–15.

[B1214] Thomson RE Holzenthal RW (2012) New species and records of Hydroptilidae (Trichoptera) from Venezuela. ZooKeys 185: 19–39. 10.3897/zookeys.185.2909 PMC3345792 22577311

[B1215] Thomson RE Holzenthal RW (2015) A revision of the Neotropical caddisfly genus *Leucotrichia* Mosely, 1934 (Hydroptilidae, Leucotrichiinae). ZooKeys 499: 1–100. 10.3897/zookeys.499.8360 PMC4410161 25931968

[B1216] Timm H Käiro K Möls T Virro T (2011) An index to assess hydromorphological quality of Estonian surface waters based on macroinvertebrate taxonomic composition. Limnologica 41(4): 398–410. 10.1016/j.limno.2011.09.006

[B1217] Tjeder B (1930a) *Ithytrichialamellaris* Eat. and *clavata* Morton in Dalecarlia. Entomologisk Tidskrift 51: 134–138.

[B1218] Tjeder B (1930b) Notes on some Swedish Trichoptera. Entomologisk Tidskrift 51: 198–205.

[B1219] Tjeder B (1940) Plecoptera, Mecoptera, Neuroptera and Trichoptera collected by Mr. Hans Lohmander in the island of Bornholm in the Baltic. Göteborgs Vetenskaps Samhälles Handlingar 1(5): 1–26.

[B1220] Tjeder B (1941) New Trichoptera from Sweden. Opuscula Entomologica 6(1): 7–11.

[B1221] Tjeder B (1946) Trichoptera from the River Jordan, Palestine. Opuscula Entomologica 11: 132–136.

[B1222] Tobias W (1970) Die Trichopteren der Lule Lappmark (Schweden). 4. Taxonomie, Verbreitung und Ökologie einiger *Oxyethira*-Arten (Hydroptilidae). Entomologische Zeitschrift 80(22): 225–231.

[B1223] Tobias W (1999) Köcherfliegen-Neufunde vom unteren Main in Hessen (Trichoptera). Entomologische Zeitschrift 109: 49–55.

[B1224] Tobias W Saarela E Salokannel J (2009) Description of the females of *Oxyethiraklingstedti* Nybom, 1983 and *Oxyethiratamperensis* Malicky, 1999 (Trichoptera: Hydroptilidae). Entomologische Zeitschrift 119: 25–26.

[B1225] Towns DR (1981) Life histories of benthic invertebrates in a Kauri forest stream in northern New Zealand. Australian Journal of Marine and Freshwater Research 32(2): 191–211. 10.1071/MF9810191

[B1226] Turunen H (1999) Taxa new to Finland, new provincial records and deletions from the fauna of Finland. Entomologica Fennica 10: 1–5.

[B1227] Uherkovich A Nógrádi S (1997) Studies on caddisfly (Trichoptera) communities of larger rivers in Hungary. In: Holzenthal RW Flint Jr OS (Eds) Proceedings of the 8th International Symposium on Trichoptera.Ohio Biological Survey, Columbus, 459–465.

[B1228] Uherkovich A Nógrádi S (1998) The caddisfly (Trichoptera) fauna of the Szatmár-Bereg Plain, Northeast Hungary. A. Janus Pannonius Múzeum Évkönyve 41–42(1996–1997): 49–62.

[B1229] Uherkovich A Nógrádi S (1999) The survey of caddisflies (Trichoptera) of the Hungarian catchment area of the River Dráva. In: Malicky H Chantaramongkol P (Eds) Proceedings of the 9th International Symposium on Trichoptera.Faculty of Science, Chiang Mai University, Chiang Mai, Thailand, 415–423.

[B1230] Uherkovich A Nógrádi S (2001) The Trichoptera of the Szigetkoz, upper Hungarian Danube Region (northwest Hungary), I. A compendium of faunistical research. Folia Historico Naturalia Musei Matraensis 25: 91–110.

[B1231] Ujvárosi L (2002) The present stage of knowledge on the Trichoptera of the Central Group of the Eastern Carpathians in Romania. Nova Supplementa Entomologica (Proceedings of the 10^th^ International Symposium on Trichoptera) 15: 379–394.

[B1232] Ujvárosi L Robert SC Neu P Robert B (2008) First revision of the Romanian caddisflies (Insecta: Trichoptera). Part 1: systematic checklist (updated 12/2005). Ferrantia 55: 110–124.

[B1233] Uljanin W (1869) Verzeichnis der Netz-und Geradflügler der Gouvernements des Moskauer Lehrbezirks. Gesellschaft der Freunde der Naturwissenschaften 6(2): 219 pp.

[B1234] Ulmer G (1905) Über die geographische Verbreitung der Trichopteren. Zeitschrift für Wissenschaftliche Insektenbiologie 1: 16–32, 68–80, 119–126.

[B1235] Ulmer G (1906) Neuer Beitrag zur Kenntnis aussereuropäischer Trichopteren. Notes from the Leyden Museum 28: 1–116.

[B1236] Ulmer G (1907) Trichoptera. In: Wytsman P (Ed.) Genera Insectorum 60: 1–259.

[B1237] Ulmer G (1912a) Die Trichopteren des Baltischen Bernsteins. Beiträge zur Naturkunde Preussens, 10. Schriften der Physikalisch-Ökonomischen Gesellschaft zu Königsberg, Leipzig, 380 pp.

[B1238] Ulmer G (1912b) Trichopteren von Äquatorial-Afrika. Deutsche Zentralafrika-Expedition 4: 81–125.

[B1239] Ulmer G (1925) Fauna Færøensis. Trichoptera. Entomologiske Meddelelser 14: 431–440.

[B1240] Ulmer G (1929) Über einige deutsche Hydroptiliden. Zoologischer Anzeiger 80: 253–266.

[B1241] Ulmer G (1932) Aquatic insects of China. Article III. Neue chinesische Trichopteren, nebst übersicht über die bisher aus China, bekannten arten. Peking Natural History Bulletin 7(1): 39–70.

[B1242] Ulmer G (1950) Eine neue *Stactobia*-Art und ihre Larve aus Bulgarien, nebst Bemerkungen uber die anderen europäischen Arten der Gattung (Trichopt.). Archiv für Hydrobiologie 44: 294–300.

[B1243] Ulmer G (1951) Köcherfliegen (Trichopteren) von den Sunda-Inseln. Teil I. Archiv für Hydrobiologie (Supplement 19): 1–528.

[B1244] Ulmer G (1957) Köcherfliegen (Trichopteren) von den Sunda-Inseln. Teil III. Larven und Puppen der Annulipalpia. Archiv für Hydrobiologie (Supplement 23): 109–470.

[B1245] Ulmer G (1963) Trichopteren (Köcherfliegen) aus Ägypten. Archiv für Hydrobiologie 59(2): 257–271.

[B1246] Unzicker JD Aggus L Warren LO (1970) A preliminary list of the Arkansas Trichoptera. Journal of the Georgia Entomological Society 5: 167–174.

[B1247] Unzicker JD Resh VH Morse JC (1982) Trichoptera. In: Brigham AR, Brigham WV, Gnilka A (Eds) Aquatic insects and oligochaetes of North and South Carolina. Midwest Aquatic Enterprises, Mahomet, Illinois, 9.1–9.138.

[B1248] Urbanič G (2004) New records of the family Hydroptilidae for the caddisfly (Insecta: Trichoptera) fauna of Slovenia. Natura Sloveniae 6(2): 49–52.

[B1249] Urbanič G Krušnik C Toman MJ (2000) New records for the caddisfly fauna of Slovenia (Insecta: Trichoptera). Acta Entomologica Slovenica (Ljubljana) 8(1): 43–48.

[B1250] Usis JD Foote BA (1989) New records of caddisflies (Trichoptera) from Ohio, with particular reference to Stillfork Swamp, Carroll County. Entomological News 100: 83–85.

[B1251] Usseglio-Polatera P Bournaud M (1989) Trichoptera and Ephemeroptera as indicators of environmental changes of the Rhône River at Lyon over the last twenty-five years. Regulated Rivers 4(3): 249–262. 10.1002/rrr.3450040304

[B1252] Utsunomiya Y (1994) Occurrence of the genus *Scelotrichia* in Japan with the description of a new species (Trichoptera: Hydroptilidae). Transactions of the Shikoku Entomological Society 20(3–4): 345–348.

[B1253] Vaillant F (1951) Contribution à l’etude des Trichoptères du genre *Stactobia* MacLachlan. Bulletin de la Société Zoologique de France 76: 13–17.

[B1254] Vaillant F (1956) Recherches sur la fauna madicole de France, de Corse et d’Afrique du Nord. Mémoires du Muséum National d’Histoire Naturelle de Paris (A) 11: 1–258.

[B1255] Vaillant F (1984) The hydroptilid larvae living on dripping rocks. In: Morse JC (Ed.) Proceedings of the 4th International Symposium on Trichoptera.Dr. W. Junk, The Hague, 407–412.

[B1256] Valladolid M Martínez-Bastida JJ Arauzo M (2011) The Trichoptera fauna of the Oja River (La Rioja, Spain). Zoosymposia 5(1): 497–507. 10.11646/zoosymposia.5.1.42

[B1257] Vallania EA Medina AI Sosa M (1998) Estructura de la comunidad de Trichoptera en un arroyo regulado de la provincia de San Luis, Argentina. Revista de la Sociedad Entomológica Argentina 57: 7–11.

[B1258] Valle M (2001) Contributo alla conoscenza die tricotteri italiani (Insecta, Trichoptera). Rivista del Museo Civico di Scienze Naturali “Enrico Caffi” Bergamo 20: 59–86.

[B1259] Valle M Lodovici O (2018) I Tricotteri di Calabria (Insecta, Trichoptera). Rivista del Museo Civico di Scienze Naturali “Enrico Caffi” Bergamo 31: 139–186.

[B1260] van Haaren T Tempelman D van Mil J (2016) Eerste vondst van larven van *Ithytrichialamellaris* in Nederland sinds begin 20 eeuw. De Digitale Kokerjuffer 12(19): 10–11.

[B1261] Varga I Andrikovics S Hufnagel L (1998) New data on the macrofauna of Lake Ferto, Hungary. Opuscula Zoologica 31: 143–148.

[B1262] Vásquez-Ramos JM Osorio-Ramírez DP Caro-Caro CI (2020) First record of the larvae *Byrsopteryx* Flint, 1981 (Trichoptera: Hydroptilidae). Revista de la Academia Colombiana de Ciencias Exactas, Físicas y Naturales 44(171): 482–492. 10.18257/raccefyn.1025

[B1263] Vieira NKM Kondratieff BC Ruiter DE Durfee RS (2009) The aquatic insects of the Valles Caldera National Preserve, Sandoval County, New Mexico, excluding Diptera, with notes on new state records. Journal of the Kansas Entomological Society 82(3): 250–262. 10.2317/JKES00812.31.1

[B1264] Viidalepp J Timm H Salokannel J (2011) Estonian Caddisflies (Insecta: Trichoptera). An annotated checklist. Entomologica Fennica 21(4): 193–201. 10.33338/ef.84531

[B1265] Vineyard RN (1982) An annotated checklist of the caddisflies (Trichoptera) of SE Alaska. Journal of the Entomological Society of British Columbia 79: 71–75.

[B1266] Vinikour WS (1982) Phoresis between the snail *Oxytrema* (= *Elima*) *carinifera* and aquatic insects, especially *Rheotanytarsus* (Diptera: Chironomidae). Entomological News 93(5): 143–151.

[B1267] Višinskienė G (2009) The updated checklist of Lithuanian caddisflies (Insecta: Trichoptera) with notes on species rarity. Acta Zoologica Lituanica 19(1): 25–40. 10.2478/v10043-009-0005-8

[B1268] Voigt H Küttner R Plesky B Heise S Beilharz M (2006) Beitrag zur Köcherfliegenfauna Sachsens (Trichoptera). Lauterbornia 58: 71–77.

[B1269] Vrućina I Previšić A Merdić E (2016) First record of *Oecetisfurva* (Rambur, 1842) and *Orthotrichiatragetti* (Mosely, 1930) (Insecta, Trichoptera) for the Croatian fauna. Natura Croatica 25(1): 109–118. 10.20302/NC.2016.25.7

[B1270] Vshivkova TS Flint Jr OS Ito T Ivanov VD Holzenthal RW Melnitsky SI Mey W Nozaki T Oh MW Drozdov K Tojo K Saito R Tori T (2016) The List of Caddisflies (Insecta, Trichoptera) collected in South Primorye during the symposium and post-symposium excursions of the XIV International Symposium on Trichoptera (5 and 8–13 July 2012). In: Vshivkova TS Morse JC (Eds) Proceedings of the 14th International Symposium on Trichoptera.Zoosymposia 10: 64–84. 10.11646/zoosymposia.10.1.7

[B1271] Wallace ID (2016) A review of the status of the caddis flies (Trichoptera) of Great Britain – Species Status No. 27. Natural England Commissioned Reports, Number 191: 127 pp. www.gov.uk/government/organisations/natural-england

[B1272] Wallace ID Wallace B O’Connor JP O’Connor MA (1983) *Phacopteryxbrevipennis* new to Ireland with notes on *Oxyethirasimplex* and *Beraeodesminutus* (Insecta: Trichoptera). The Irish Naturalists’ Journal 21(4): 168–169.

[B1273] Wallace ID Wallace B Philipson GN (2003) Keys to the case-bearing caddis larvae of Britain and Ireland. Scientific Publication - Freshwater Biological Association 61: 1–259.

[B1274] Waltz RD McCafferty WP (1983a) The caddisflies of Indiana (Insecta: Trichoptera). Purdue University Agricultural Experiment Research Station Bulletin 978: 1–25.

[B1275] Waltz RD McCafferty WP (1983b) New caddisfly records for New Mexico (Insecta: Trichoptera). The Southwestern Naturalist 28(3): 353–356. 10.2307/3670797

[B1276] Waltz RD McCafferty WP (1983c) New caddisfly records for New Mexico (Insecta: Trichoptera). The Southwestern Naturalist 28(4): 413–415. 10.2307/3670820

[B1277] Wang Y-K Kennedy JH (2004) Life history of *Mayatrichiaponta* Ross (Trichoptera: Hydroptilidae) in Honey Creek, Oklahoma. Proceedings of the Entomological Society of Washington 106: 523–530.

[B1278] Ward JB Henderson IM (2004) Eleven new species of micro-caddis (Trichoptera: Hydroptilidae) from New Zealand. Records of the Canterbury Museum 18: 9–22.

[B1279] Waringer JA (1989) The abundance and temporal distribution of caddisflies (Insecta: Trichoptera) caught by light traps on Austrian Danube from 1986 to 1987. Freshwater Biology 21(3): 387–399. 10.1111/j.1365-2427.1989.tb01371.x

[B1280] Waringer J Graf W (1997) Atlas der Österreichischen Köcherfliegenlarven, unter Einschluss der angrenzenden Gebeite [Atlas of Austrian caddisfly larvae, with inclusion of adjacent areas.]. Facultas Universitätsverlag, Vienna, 286 pp.

[B1281] Waringer J Graf W (2002) Ecology, morphology and distribution of *Ptilocolepusgranulatus* (Pictet 1834) (Insecta: Trichoptera) in Austria. Lauterbornia 43: 121–129.

[B1282] Waringer J Graf W (2006) Light-trapping of Trichoptera at the march, a lowland river in Eastern Austria. Archiv für Hydrobiologie. Supplement 158(3): 351–372. 10.1127/lr/16/2006/351

[B1283] Waringer J Graf W (2008) Light-trapping of Trichoptera at the March, an eight-order Austrian lowland river - Proceedings of the 1^st^ Conference on Faunistics and Zoogeography of European Trichoptera. Ferrantia 55: 141–142.

[B1284] Waringer J Graf W (2011) Atlas of Central European Trichoptera larvae. Erik Mauch Verlag, Germany, 468 pp.

[B1285] Wasmund AM Holzenthal RW (2007) A revision of the Neotropical caddisfly genus *Rhyacopsyche*, with the description of 13 new species (Trichoptera: Hydroptilidae). Zootaxa 1634(1): 1–59. 10.11646/zootaxa.1634.1.1

[B1286] Watts E (1976) The pupation of *Agrayleamultipunctata* Curtis (Trichoptera, Hydroptilidae). Entomologist’s Monthly Magazine 111: 15–22.

[B1287] Weaver III JS (1984) The evolution and classification of Trichoptera. Part I: the groundplan of Trichoptera. In: Morse JC (Ed.) Proceedings of the 4th International Symposium on Trichoptera.Dr. W. Junk, The Hague, 413–419.

[B1288] Weaver III JS (1990) A new synonym in Hydroptilidae (Trichoptera). Proceedings of the Entomological Society of Washington 92: 360.

[B1289] Weaver III JS Morse JC (1986) Evolution of feeding and case-making behavior in Trichoptera. Journal of the North American Benthological Society 5(2): 150–158. 10.2307/1467869

[B1290] Weinzierl A (1997) *Oxyethirafalcata* Morton, 1893 - neu für Bayern (Trichoptera, Hydroptilidae). Nachrichtenblatt der Bayerischen Entomologen 46(3–4): 80–81.

[B1291] Weinzierl A Dorn A (1995) Neue und wiedergefundene Köcherfliegen (Trichoptera) für Bayern. Lauterbornia 20: 43–48.

[B1292] Weinzierl A Hess M Heckes U (2005) Neunachweise and Wiederfunde von Köcherfliegen in Bayern. Lauterbornia 54: 45–52.

[B1293] Wells A (1978) A review of the Australian species of *Hydroptila* Dalman (Trichoptera: Hydroptilidae) with descriptions of new species. Australian Journal of Zoology 26(4): 745–762. 10.1071/ZO9780745

[B1294] Wells A (1979a) The Australian species of *Orthotrichia* Eaton (Trichoptera: Hydroptilidae). Australian Journal of Zoology 27(4): 585–622. 10.1071/ZO9790585

[B1295] Wells A (1979b) A review of the Australian genera *Xuthotrichia* Mosely and *Hellyethira* Neboiss (Trichoptera: Hydroptilidae), with descriptions of new species. Australian Journal of Zoology 27: 311–329. 10.1071/ZO9790311

[B1296] Wells A (1980) A review of the Australian genera *Orphninotrichia* Mosely and *Maydenoptila* Neboiss (Trichoptera: Hydroptilidae), with descriptions of new species. Australian Journal of Zoology 28(4): 627–645. 10.1071/ZO9800627

[B1297] Wells A (1981) The genera *Oxyethira* Eaton, *Gnathotrichia* Ulmer and *Stenoxyethira* Kimmins (Trichoptera: Hydroptilidae) in Australia. Australian Journal of Zoology 29(1): 103–118. 10.1071/ZO9810103

[B1298] Wells A (1982) *Tricholeiochiton* Kloet & Hincks and new genera in the Australian Hydroptilidae (Trichoptera). Australian Journal of Zoology 30(2): 251–270. 10.1071/ZO9820251

[B1299] Wells A (1983) New species in the Australian Hydroptilidae (Trichoptera), with observations on relationships and distributions. Australian Journal of Zoology 31(4): 629–649. 10.1071/ZO9830629

[B1300] Wells A (1984) *Hydroptila* Dalman and *Orthotrichia* Eaton (Trichoptera: Hydroptilidae) from the islands of New Guinea and New Britain, with observation on relationships. Australian Journal of Zoology 32(2): 261–282. 10.1071/ZO9840261

[B1301] Wells A (1985a) Four new species of Hydroptilidae (Trichoptera) from the Alligator River region, Northern Territory. Transactions of the Royal Society of South Australia 109: 97–102.

[B1302] Wells A (1985b) Larvae and pupae of Australian Hydroptilidae (Trichoptera), with observations on general biology and relationships. Australian Journal of Zoology. Supplementary Series 113: 1–69. 10.1071/AJZS113

[B1303] Wells A (1987) On the biogeography of the *Oxyethira* group, tribe Hydroptilini (Hydroptilinae, Hydroptilidae, Trichoptera). In: Bournaud M Tachet H (Eds) Proceedings of the 5th International Symposium on Trichoptera.Dr. W. Junk, Dordrecht, The Netherlands, 133–138. 10.1007/978-94-009-4043-7_23

[B1304] Wells A (1990a) The hydroptilid tribe Stactobiini (Trichoptera: Hydroptilidae) in New Guinea. Invertebrate Taxonomy 3(6): 817–849. 10.1071/IT9890817

[B1305] Wells A (1990b) The micro-caddisflies (Trichoptera: Hydroptilidae) of North Sulawesi. Invertebrate Taxonomy 3(4): 363–406. 10.1071/IT9890363

[B1306] Wells A (1990c) New species and a new genus of micro-caddisfly from northern Australia, including the first Australian record of the tribe Stactobiini (Trichoptera: Hydroptilidae). Transactions of the Royal Society of South Australia 114: 107–128.

[B1307] Wells A (1991) The hydroptilid tribes Hydroptilini and Orthotrichini in New Guinea (Trichoptera: Hydroptilidae: Hydroptilinae). Invertebrate Taxonomy 5(3): 487–526. 10.1071/IT9910487

[B1308] Wells A (1992) The first parasitic Trichoptera. Ecological Entomology 17(3): 299–302. 10.1111/j.1365-2311.1992.tb01061.x

[B1309] Wells A (1993) Micro-caddisflies (Trichoptera: Hydroptilidae) from Bali, Indonesia. Zoologische Mededelingen (Leiden) 67(1–26): 351–359.

[B1310] Wells A (1995) New Caledonian Hydroptilidae (Trichoptera), with new records, descriptions of larvae and a new species. Aquatic Insects 17(4): 223–239. 10.1080/01650429509361591

[B1311] Wells A (1997) A preliminary guide to the identification of larval Hydroptilidae (Insecta: Trichoptera). Co-operative Research Centre for Freshwater Ecology Identification Guide 13: 1–28.

[B1312] Wells A (1998) Two new species of Hydroptilidae (Trichoptera) from Tasmania’s World Heritage Area. Australian Entomologist 25: 81–84.

[B1313] Wells A (1999) The micro-caddisflies of Lord Howe Island (Hydroptilidae: Trichoptera: Insecta). Aquatic Insects 21(3): 221–230. 10.1076/aqin.21.3.221.4516

[B1314] Wells A (2002a) Three new species of *Orphninotrichia* Mosely (Trichoptera: Hydroptilidae) from Barrington Tops, New South Wales, a distribution extended, and remarks on generic placement. Australian Journal of Entomology 41(3): 221–225. 10.1046/j.1440-6055.2002.00295.x

[B1315] Wells A (2002b) Two new species of *Oxyethira* Eaton (Hydroptilidae: Trichoptera: Insecta) for Tasmania. Papers and Proceedings of the Royal Society of Tasmania 136: 39–41. 10.26749/rstpp.136.39

[B1316] Wells A (2005) Parasitism by hydroptilid caddisflies (Trichoptera) and seven new species of Hydroptilidae from northern Queensland. Australian Journal of Entomology 44(4): 385–391. 10.1111/j.1440-6055.2005.00492.x

[B1317] Wells A (2010a) Five new species and new records of Hydroptilidae (Trichoptera) from the Wet Tropics of northeastern Queensland. Zootaxa 2641(1): 47–54. 10.11646/zootaxa.2641.1.5

[B1318] Wells A (2010b) Thirty years of hydroptilid studies - 1979 to 2009. Denisia 29: 437–443.

[B1319] Wells A (2012) New synonyms in the Australian micro-caddisfly fauna (Trichoptera: Hydroptilidae). Zootaxa 3177(1): 66–68. 10.11646/zootaxa.3177.1.7

[B1320] Wells A (2020) Curious Caddis Couture: Form and function among cases of Australian Hydroptilidae. Zoosymposia 18: 024–033. 10.11646/zoosymposia.18.1.6

[B1321] Wells A Andersen T (1995) Tanzanian micro-caddisflies (Trichoptera: Hydroptilidae). Tijdschrift voor Entomologie 138: 143–167.

[B1322] Wells A Andersen T (1996) Two new *Catoxyethira* species from Tanzania (Trichoptera, Hydroptilidae) and a revised key to Tanzanian hydroptilids. Tijdschrift voor Entomologie 139: 85–89.

[B1323] Wells A de Moor FC (2020) Hydroptilidae (Trichoptera) of Angola, a new genus, seven new species, and five new records. Zootaxa 4868(4): 495–514. 10.11646/zootaxa.4868.4.2 33311379

[B1324] Wells A Dostine P (2016) New and newly recorded micro-caddisfly species (Insecta: Trichoptera: Hydroptilidae) from Australia’s north, including islands of Torres Strait. Zootaxa 4127: 591–600. 10.11646/zootaxa.4127.3.11 27395643

[B1325] Wells A Dudgeon D (1990) Hydroptilidae (Insecta: Trichoptera) from Hong Kong. Aquatic Insects 12(3): 161–175. 10.1080/01650429009361400

[B1326] Wells A Huisman J (1992) Micro-caddisflies in the tribe Hydroptilini (Trichoptera: Hydroptilidae: Hydroptilinae) from Malaysia and Brunei. Zoologische Mededelingen (Leiden) 66: 91–126.

[B1327] Wells A Huisman J (1993) Malaysian and Bruneian micro-caddisflies in the tribes Stactobiini and Orthotrichiini. Zoologische Mededelingen (Leiden) 67: 91–125.

[B1328] Wells A Huisman J (2001) New hydroptilid caddisfly species from southern Sulawesi (Insecta: Trichoptera: Hydroptilidae). Zoologische Mededelingen (Leiden) 75: 207–216.

[B1329] Wells A Johanson KA (2012) Review of the New Caledonian species of *Paroxyethira* Mosely, 1924 (Trichoptera: Hydroptilidae). Zootaxa 3478(1): 330–344. 10.11646/zootaxa.3478.1.31

[B1330] Wells A Johanson KA (2014) Review of the New Caledonian species of *Acritoptila* Wells, 1982 (Trichoptera, Insecta), with descriptions of 3 new species. ZooKeys 397: 1–23. 10.3897/zookeys.397.7059 PMC3978229 24715795

[B1331] Wells A Johanson KA (2015) Review of New Caledonian species of *Oxyethira* Eaton, with description of 17 new species, and new records for *Hydroptila* Dalman and *Hellyethira* Neboiss (Trichoptera, Hydroptilidae). ZooKeys 530: 37–90. 10.3897/zookeys.530.6047 PMC4668906 26692799

[B1332] Wells A Kjer KM (2016) Norfolk Island’s caddisfly is a New Zealander (Trichoptera: Hydroptilidae). Australian Entomologist 43(2): 49–54.

[B1333] Wells A Malicky H (1997) The micro-caddisflies of Sumatra and Java (Trichoptera: Hydroptilidae). Linzer Biologische Beitrage 29: 173–202.

[B1334] Wells A Mey W (2002) Microcaddisflies of the Philippines (Trichoptera, Hydroptilidae). Mitteilungen aus dem Museum für Naturkunde in Berlin Deutsche Entomologische Zeitschrift 49: 113–136. 10.1002/mmnd.20020490109

[B1335] Wells A Wichard W (1989) Caddisflies of Dominican amber VI. Hydroptilidae (Trichoptera). Studies on Neotropical Fauna and Environment 24(1): 41–51. 10.1080/01650528909360774

[B1336] Wells A Yule CM (2008) The caddisflies (Trichoptera) from a tropical peat swamp in Selangor, Peninsular Malaysia, including two new species. Aquatic Insects 30(1): 69–76. 10.1080/01650420701687163

[B1337] Wells A Johanson KA Mary-Sasal N (2013) The New Caledonian genus *Caledonotrichia* Sykora (Trichoptera, Insecta) reviewed, with descriptions of 6 new species. ZooKeys 287: 59–89. 10.3897/zookeys.287.4615 PMC3677356 23794848

[B1338] Wells A Johanson KA Dostine P (2019) Why are so many species based on a single specimen? Zoosymposia 14: 032–038. 10.11646/zoosymposia.14.1.5

[B1339] Westwood JO (1838) Description of a new genus of exotic bees. The Transactions of the Entomological Society of London 2(2): 112–113. 10.1111/j.1365-2311.1836.tb00305.x

[B1340] Westwood JO (1838–1840) Synopsis of the Genera of British Insects. [Trichoptera, pages 49–51, published June 1839. Usually bound with Vol. 2,] An Introduction to the Modern Classification of Insects Founded on Natural Habits and Corresponding Organisation of the Different Families. Longman, Orme, Brown, Green, and Longmans, London, 158 pp. 10.5962/bhl.title.12455

[B1341] White TR Fox RC (1979) Chironomid (Diptera) larvae and hydroptilid (Trichoptera) pupae attached to a macromiid nymph (Anisoptera). Notulae Odonatologicae 1: 76–77.

[B1342] Wiberg-Larsen P (1981) *Tricholeiochitonfagesii* (Guinard) and *Triaenodessimulans* Tjeder (Trichoptera) new to Denmark. Entomologiske Meddelelser 49: 28–30.

[B1343] Wiberg-Larsen P (1985) Revision of the Danish Hydroptilidae (Trichoptera). Entomologiske Meddelelser 53(1): 39–45.

[B1344] Wiberg-Larsen P Czachorowski S (2002) *Oxyethiratristella* Klapálek, 1895 (Trichoptera: Hydroptilidae) a caddis-fly species new to the fauna of Poland. Polskie Pismo Entomologiczne 71(2): 151–153.

[B1345] Wiberg-Larsen P Holm P (1999) Varfluen *Hydroptilamartini* Marshall, 1977 - ny for Danmark og NV-Europa (Trichoptera: Hydroptilidae). Entomologiske Meddelelser 67(4): 117–121.

[B1346] Wiberg-Larsen P Karsholt O (1999) The traffic of adult Trichoptera above the city of Copenhagen (Denmark). Entomologiske Meddelelser 67(4): 123–136.

[B1347] Wiberg-Larsen P Iversen TM Thorup J (1991) First Danish record of *Ptilocolepusgranulatus* (Pictet) (Trichoptera, Hydroptilidae). Entomologiske Meddelelser 59: 5–50.

[B1348] Wichard W (1976) Morphologische Komponenten bei der Osmoregulation von Trichopterenlarven. In: Malicky H (Ed.) Proceedings of the 1st International Symposium on Trichoptera.Dr. W. Junk, The Hague, 171–177. 10.1007/978-94-010-1579-0_25

[B1349] Wichard W (1981) Köcherfliegen des Dominikanischen Bernsteins I. *Ochrotrichiadoehler* sp. nov. Mitteilungen Münchener Entomologischen Gesellschaft 71: 161–162.

[B1350] Wichard W (2000) Köcherfliegen des Dominikanischen Bernsteins XI. *Ochrotrichiaaliceae* n. sp., eine neue fossile Hydroptilidae (Insecta, Trichoptera). Mitteilungen aus dem Geologisch-Paläontologischen Institut der Universität Hamburg 84: 241–246. [Caddisflies of Dominican amber. XI. *Ochrotrichiaaliceae* n. sp., a new fossil hydroptilid specimen (Insecta, Trichoptera)]

[B1351] Wichard W (2007) Overview and descriptions of caddisflies (Insecta, Trichoptera) in Dominican amber (Miocene). Stuttgarter Beitrage zur Naturkunde. Serie B, Geologie und Palaontologie 366: 1–51.

[B1352] Wichard W (2013) Overview and descriptions of Trichoptera in Baltic Amber: Spicipalpia and Integripalpia. Remagen-Oberwinter, Museum für Naturkunde Berlin, Verlag Kessel, 230 pp.

[B1353] Wichard W Bölling A-C (2000) Recent knowledge of caddis flies (Trichoptera) from Cretaceous amber of New Jersey. In: Grimaldi D (Ed.) Studies on fossils in amber with particular reference to the Cretaceous of New Jersey.Leiden, Backhuys Publishers, 345–354.

[B1354] Wichard W Lüer C (2003) *Phylocentropusswolenskyi* n. sp., eine Köcherfliege aus dem New Jersey Bernstein (Trichoptera, Dipseudopsidae). Mitteilungen aus dem Geologisch-Paläontologischen Institut der Universität Hamburg 87: 131–139.

[B1355] Wiggers R van den Hoek T-H van Maanen B Higler B van Kleef H (2006) Some rare and new caddisflies recorded for the Netherlands (Trichoptera). Nederlandse Faunistische Mededelingen 25: 53–68.

[B1356] Wiggins GB (1977) Larvae of the North American caddisfly genera (Trichoptera). University of Toronto Press, Canada, 401 pp.

[B1357] Wiggins GB (1996) Larvae of the North American Caddisfly Genera (Trichoptera) (2^nd^ edn.). University of Toronto Press, Canada, 472 pp. 10.3138/9781442623606

[B1358] Wiggins GB Parker CR (1997) Caddisflies (Trichoptera) of the Yukon, with analysis of the Beringian and Holarctic species of North America. In: Danks HV Downes JA (Eds) , Insects of the Yukon.Biological Survey of Canada (Terrestrial Arthropods), Ottawa, 787–866.

[B1359] Wiggins GB Wichard W (1989) Phylogeny of pupation in Trichoptera, with proposals on the origin and higher classification of the order. Journal of the North American Benthological Society 8(3): 260–276. 10.2307/1467330

[B1360] Williams NE Williams DD (1979) Distribution and feeding records of the caddisflies (Trichoptera) of the Matamek River region, Quebec. Canadian Journal of Zoology 57(12): 2402–2412. 10.1139/z79-312

[B1361] Winterbourn MJ (1998) Insect faunas of acidic coal mine drainages in Westland, New Zealand. New Zealand Entomologist 21(1): 65–72. 10.1080/00779962.1998.9722038

[B1362] Winterbourn MJ Crowe ALM (2001) Flight activity of insects along a mountain stream: Is directional flight adaptive? Freshwater Biology 46(11): 1479–1489. 10.1046/j.1365-2427.2001.00766.x

[B1363] Wise KAJ (1964) Insects of Campbell Island. Trichoptera. Pacific Insects Monographs 7: 253–254.

[B1364] Wise KAJ (1972) Trichoptera of the Auckland Islands. Records of the Auckland Institute and Museum 9: 253–267.

[B1365] Wise KAJ (1978) Further records of subantarctic Trichoptera. Records of the Auckland Institute and Museum 15: 111–113.

[B1366] Wise KAJ (1998) Two new species of *Oxyethira* (Trichoptera: Hydroptilidae) in New Zealand. New Zealand Entomologist 21(1): 17–23. 10.1080/00779962.1998.9722036

[B1367] Wityi H Nozaki T Fujino T (2015) A List of Myanmar Caddisflies (Trichoptera) including Recently Collected Data. Entomological Research Bulletin: The Entomological Society of Korea 31: 41–55.

[B1368] Wolf B Angersbach R (2014) Köcherfliegenfänge (Trichoptera) von Bodrog und Thieß bei Tokaj, Ungarn. Braueria 41: 32–33.

[B1369] Wolf B Angersbach R Flügel H-J (2012) Plecoptera and Trichoptera in the Tagliamento flood plains and in some tributaries in Friuli Venezia Giulia (Italy). Gortania, Botanica. Zoologia 34: 73–77.

[B1370] Wright DR Pytel AJ Houghton DC (2013) Nocturnal flight periodicity of the caddisflies (Insecta: Trichoptera) in a large Michigan river. Journal of Freshwater Ecology 28: 463–476. 10.1080/02705060.2013.780187

[B1371] Wrubleski DA Ross LCM (1989) Diel periodicities of adult emergence of Chironomidae and Trichoptera from the Delta Marsh, Manitoba, Canada. Journal of Freshwater Ecology 5(2): 163–169. 10.1080/02705060.1989.9665831

[B1372] Xue Y Wang H (1995) A new species of the genus *Hydroptila* (Trichoptera: Hydroptilidae) from China. Entomotaxonomia 17(3): 208–210.

[B1373] Xue Y Yang L-F (1990) Seven new species of Hydroptilidae from China (Insecta: Trichoptera). Acta Agricultura Universitatis Henanensis 24: 124–131.

[B1374] Xue Y Yang L-F (1991) Acta Agricultura Universitatis Henanensis 25: 19–23. [Six new records of Hydroptilidae from China (Insecta: Trichoptera)]

[B1375] Xue Y Luo M Guo X (1992) Hydroptilidae (Insecta: Trichoptera) from Henan Province. Acta Agricultura Universitatis Henanensis 26: 353–356, 365.

[B1376] Yang L Xue Y (1992) Six new species of Hydroptilidae (Insecta: Trichoptera) from China. Entomotaxonomia 14: 26–34.

[B1377] Yang L Xue Y (1994) Six new species of Hydroptila (Trichoptera: Hydroptilidae) from China. Braueria 21: 9–11.

[B1378] Yang L Wang B Leng K (1997a) Seven new species of Trichoptera (Insecta: Mecopteroidea) from Funiu Mountain. Entomotaxonomia 19(4): 279–288.

[B1379] Yang L-F Kelley RW Morse JC (1997b) Six new species of *Oxyethira* from southern China. Aquatic Insects 19(2): 91–105. 10.1080/01650429709361641

[B1380] Yang L-F Sun C-H Wang B-X Morse JC (2005) Present status of Chinese Trichoptera, with an annotated checklist. In: Tanida K Rossiter A (Eds) Proceedings of the 11th International Symposium on Trichoptera.Tokai University Press, Kanagawa, 441–460.

[B1381] Yang L-F Sun C-H Morse JC (2016) An amended checklist of the caddisflies of China (Insecta, Trichoptera). In: Vshivkova TS Morse JC (Eds) Proceedings of the 14th International Symposium on Trichoptera.Zoosymposia 10: 451–479. 10.11646/zoosymposia.10.1.42

[B1382] Zack RS Ruiter DE Strenge DL Landolt PJ (2006) Adult caddisfly (Trichoptera) phenology at the Hanford Reach National Monument, Washington State. Proceedings of the Entomological Society of Washington 108: 131–138.

[B1383] Zasypkina IA (2016) Current knowledge on caddisflies (Trichoptera) in northern Far East Russia. In: Vshivkova TS Morse JC (Eds) Proceedings of the 14th International Symposium on Trichoptera.Zoosymposia 10: 480–492. 10.11646/zoosymposia.10.1.43

[B1384] Zasypkina IA Ryabukhin AS (2001) Amphibiotic Insects of the Northeast of Asia. Pensoft & Backhuys Publishers BV, Sofia, Bulgaria / Leiden, The Netherlands, 183 pp.

[B1385] Zhou L Sun C-H Yang L-F (2009a) Four new species of *Hydroptila* (Trichoptera, Hydroptilidae) from China. Dong Wu Fen Lei Xue Bao 34(4): 905–911.

[B1386] Zhou L Sun C-H Yang L-F (2009b) A study of geunus [genus] *Hydroptila* with two new species and two new record species from China (Trichoptera, Hydroptilidae). Dong Wu Fen Lei Xue Bao 34(2): 353–359.

[B1387] Zhou L Yang L-F Morse JC (2010) Six new species and 1 new species record of *Orthotrichia* (Trichoptera: Hydroptilidae) from China. Zootaxa 2560: 29–41. 10.11646/zootaxa.2560.1.3 27394538

[B1388] Zhou L Yang L-F Morse JC (2013) New Species of *Stactobia* McLachlan (Trichoptera: Hydroptilidae) from China. Journal of the Kansas Entomological Society 86(3): 277–286. 10.2317/JKES130401.1

[B1389] Zhou L Yang L-F Morse JC (2016) New species of microcaddisflies from China (Trichoptera: Hydroptilidae). Zootaxa 4097(2): 203–219. 10.11646/zootaxa.4097.2.3 27394538

[B1390] Zimmerman EC (1957) Insects of Hawaii (Vol. 6). Honolulu, Hawaii: University of Hawaii Press, [xii +] 209 pp.

[B1391] Zuellig RE Kondratieff BC Schmidt JP Durfee RS Ruiter DE Prather IE (2006) An annotated list of aquatic insects of Fort Sill, Oklahoma, excluding Diptera with notes on several new State records. Journal of the Kansas Entomological Society 79(1): 34–54. 10.2317/505.03.1

[B1392] Zuyderduyn C (2016) *Molannaalbicans* en *Oxyethirasagittifera* nieuw voor Friesland. De Digitale Kokerjuffer 12(19): 7–9.

[B1393] Zuyderduyn C Tempelman D (2013) Schietmotten van het Vechtplassengebied. De Digitale Kokerjuffer 9(15): 22–30.

